# Transcriptional Pathology Evolves over Time in Rat Hippocampus after Lateral Fluid Percussion Traumatic Brain Injury

**DOI:** 10.1089/neur.2021.0021

**Published:** 2021-11-23

**Authors:** Rinaldo Catta-Preta, Iva Zdilar, Bradley Jenner, Emily T. Doisy, Kayleen Tercovich, Alex S. Nord, Gene G. Gurkoff

**Affiliations:** ^1^ Department of Neurobiology, Physiology, and Behavior, University of California Davis, Davis, California, USA.; ^2^ Department of Neurological Surgery, University of California Davis, Davis, California, USA.; ^3^ Center for Neuroscience, University of California Davis, Davis, California, USA.

**Keywords:** differential expression, longitudinal, neurodegeneration, rat, TBI

## Abstract

Traumatic brain injury (TBI) causes acute and lasting impacts on the brain, driving pathology along anatomical, cellular, and behavioral dimensions. Rodent models offer an opportunity to study the temporal progression of disease from injury to recovery. Transcriptomic and epigenomic analysis were applied to evaluate gene expression in ipsilateral hippocampus at 1 and 14 days after sham (*n* = 2 and 4, respectively per time point) and moderate lateral fluid percussion injury (*n* = 4 per time point). This enabled the identification of dynamic changes and differential gene expression (differentially expressed genes; DEGs) modules linked to underlying epigenetic response. We observed acute signatures associated with cell death, astrocytosis, and neurotransmission that largely recovered by 2 weeks. Inflammation and immune signatures segregated into upregulated modules with distinct expression trajectories and functions. Whereas most down-regulated genes recovered by 14 days, two modules with delayed and persistent changes were associated with cholesterol metabolism, amyloid beta clearance, and neurodegeneration. Differential expression was paralleled by changes in histone H3 lysine residue 4 trimethylation at the promoters of DEGs at 1 day post-TBI, with the strongest changes observed for inflammation and immune response genes. These results demonstrate how integrated genomics analysis in the pre-clinical setting has the potential to identify stage-specific biomarkers for injury and/or recovery. Though limited in scope here, our general strategy has the potential to capture pathological signatures over time and evaluate treatment efficacy at the systems level.

## Introduction

The U.S. Centers for Disease Control and Prevention reported roughly 3 million traumatic brain injury (TBI) incident-related in- and outpatient emergency room visits in the United States in 2014.^1^ Among military service personnel, almost 20,000 soldiers experienced a TBI in 2019, mostly mild to moderate.^2^ Whereas severe TBI can have long-lasting effects resulting in a chronic disease state,^3^ most persons who experience a mild, and many with a moderate, severity of TBI do recover. However, it has also become clear that a percentage of mild and moderate TBIs are associated with increased risk for late-onset neurodegenerative diseases such as Parkinson's and Alzheimer's diseases.^4–7^

Measurement of the perturbations in gene expression in bulk tissue and single cells in rodent models of TBI has provided a systems-level perspective of the impacts of TBI.^8–12^ In the initial period after TBI, transcriptional studies have identified signatures of neuroinflammation, and cell death, that correlate with long-term outcomes such as cognitive dysfunction,^8^ with elevated transcription of inflammatory mediators observed for up to 1 year.^13^ Parallel to transcriptomic changes, initial studies of the epigenetic impacts of TBI^8,14,15^ found that 7 days post-injury, DNA methylation was impacted at hundreds of sites across the genome. Other epigenetic signatures, for example changes to histone proteins, have also been reported after TBI,^14,16^ though have not been explored in depth at genome-wide resolution. Despite the application of genomic approaches to dissect the impacts of TBI, few studies have paired full transcriptome RNA-sequencing (RNA-seq) and functional assays to link gene-regulatory control and transcriptional outcomes at the systems level.

Changes in gene expression post-TBI are dynamic and responsive to the state of recovery post-injury. For example, it is well described that cell death peaks acutely post-injury, and that there are immediate changes in neurotransmission that reduce plasticity,^17,18^ but that cognitive function recovers over a period of weeks. Pairing longitudinal transcriptional and epigenetic changes has the potential to illuminate mechanisms underlying long-term molecular pathology and the underlying epigenetic responses associated with both acute and long-lasting pathology. Toward these goals, we interrogated acute (1-day) and subchronic (14-day) transcriptional changes paired with genome-wide histone H3 lysine residue 4 trimethylation (H3K4me3) profiling in ipsilateral hippocampus in rats exposed to lateral fluid percussion (LFP) injury. Our results map molecular signatures associated with distinct expression trajectories post-TBI and find evidence of parallel alterations in histone H3K4me3 at relevant gene promoters.

## Methods

### Test animals

**FIG. 1.**
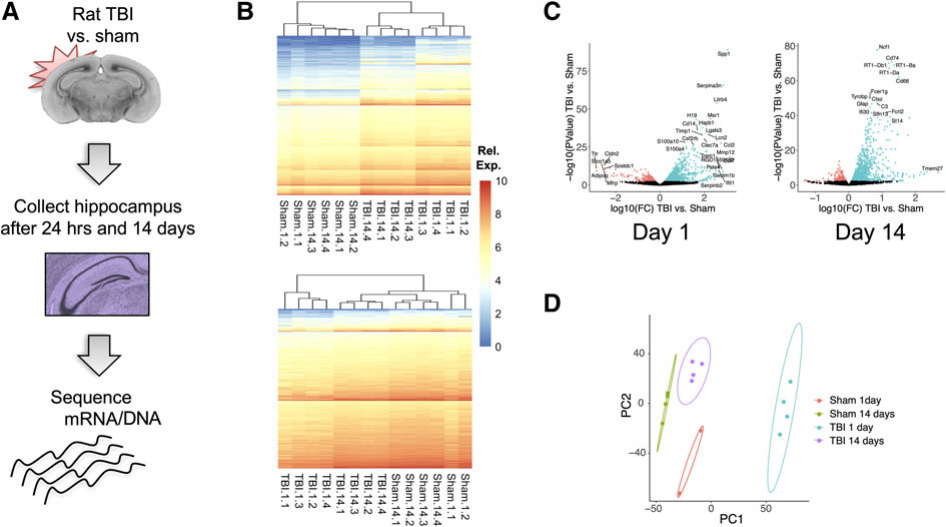
Differential expression of genes subjected to TBI. (**A**) Summary of experimental design. (**B**) Clustered heatmap of reads from upregulated (upper panel, *n* = 6870) and downregulated genes (lower panel, *n* = 10,302) at both time points (1 and 14 days after TBI) for genes with unadjusted *p* value <0.05. (**C**) Volcano plots of the differentially expressed genes at each of the time points. Each dot represents a gene; those colored in red were downregulated, whereas the ones in green were upregulated, TBI versus sham control (significance level is 99%, *α* = 0.05). Differential gene expression presented in log_10_ scale, with example genes labeled. (**D**) Principal component analysis plot showing the first two components. Ellipses represent cluster confidence intervals at the 95% confidence level (when there were only two data points, an ellipse was manually drawn to enclose both points). mRNA, messenger RNA; TBI, traumatic brain injury.

Adult male Sprague-Dawley rats (300–375 g; Envigo, Livermore, CA) were randomly assigned to sham control (*n* = 6) and LFP injury (*n* = 8) groups (Fig. 1A). These groups were further randomly separated for acute assessment 1 day post-injury (*n* = 2 sham, *n* = 4 LFP) and chronic assessment on day 14 post-injury (*n* = 2 sham, *n* = 4 LFP). Rats were housed in a campus vivarium with regulated temperature (22°C) and humidity (40–60%) and on a 12-h light/dark cycle. Rats had free access to food and water throughout the experiment. All procedures adhered to the National Institutes of Health guidelines and were approved by the University of California Davis Institutional Animal Care and Use Committee.

### Lateral fluid percussion injury rat model

Rats were randomly assigned to receive either LFP or sham injury.^19^ Sham animals received identical surgical procedures as TBI, including duration of anesthesia, except that the fluid percussion injury was not administered. Anesthesia was induced using 4% isoflurane (in air). Animals were then intubated, shaved, transferred to a stereotaxic frame, and mechanically ventilated to maintain a surgical plane of anesthesia using 1.5–3.0% isoflurane (in 2 NO_2_/1 O_2_) for the remainder of the surgery. After sterile preparation, a subcutaneous injection of 0.25% bupivacaine (0.1 mL) was delivered above the dorsal surface of the skull. A midline scalp incision was made, and the skin was retracted to expose the dorsal cranial surface. A circular 4.8-mm diameter parasagittal craniectomy was made over the right hemisphere midway between bregma and lambda and 3 mm lateral to midline using a trephine. Two stainless steel screws (0–80 degrees) were secured in the skull, anterior and posterior to the craniectomy. A custom three-dimensional printed injury hub, designed based on the properties of a traditional Leur Lock, was placed in the craniectomy and cemented to the skull with a combination of super glue gel and dental acrylic. The injury hub was then filled with sterile saline.

The fluid percussion device was calibrated to produce an injury of 2.12 ± 0.02 atm of pressure. In our hands, this injury typically results in a moderate injury with persistent spatial learning deficits for at least 2 weeks post-injury.^20,21^ The animal was removed from anesthesia, attached to the device, and the injury hammer was released upon return of the toe pinch reflex.^22^ Immediately post-injury, animals were observed for the recovery of the toe pinch withdrawal and then were returned to 2.5% isoflurane. Finally, the injury hub was removed, and the wound was sutured closed (4.0 braided silk suture), and the animal was placed in a heated cage and observed until becoming sternal.

### Hippocampal tissue collection

Rats were placed in an anesthesia chamber with continuous flow of 4% isoflurane with air as the carrier for 4 min and then, while still anesthetized, rapidly decapitated. The brain was exposed and rolled out of the skull and placed on filter paper atop a glass dish sitting on ice. The cortex was hemisected rostral to caudal, then rolled back (medial to lateral), exposing the underlying hippocampus. A curved, plastic spatula was used to roll the hippocampus out of the cortex and onto the filter paper. The same spatula was used to completely separate the hippocampus from the cortex and place it into a sterile microcentrifuge tube. Ipsilateral (right) and contralateral (left) hippocampus were collected separately.

### RNA-sequencing experiments

Fresh hippocampus samples from injury ipsilateral tissue were prepared as previously described.^23^ Total RNA was isolated from samples using Ambion RNAqueous (catalog no.: AM1912; ThermoFisherScientific, Waltham, MA) and assayed using an Agilent BioAnalyzer instrument (Agilent Technologies, Santa Clara, CA). A TruSeq Stranded mRNA kit (Illumina P/N 20020594; Illumina, San Diego, CA) was used to prepare the stranded mRNA libraries that were sequenced on the Illumina HiSeq platform using a single-end 50-bp strategy. Libraries were pooled at 6–12 samples per lane; each library was quantified and pooled before submission for sequencing. All samples were sequenced at the UC Davis DNA Technologies Core.

### ChIP-seq

Chromating immunoprecipitation sequencing (ChIP-seq) experiments were perfromed following established protocols.^24^ Frozen hippocampus tissue samples were individually crosslinked with a 1% formaldehyde buffer solution for 10 min, then washed and isolated. Resuspended crosslinked pellets were treated with protease inhibitor and sheared by sonication. Samples were then washed off of unbound DNA and free proteins and incubated with Histone H3K4me3 antibody (monoclonal antibody; catalog no.: 61979; Active Motif, Carlsbad, CA) and magnetic beads (Dynabeads^®^; ThermoFisherScientific) for 2 h at 4°C. After reaction, beads were magnetically separated, washed and de-crosslinked. Resulting DNA was then purified, size selected, and libraries were prepared using the Ovation Ultralow System V2 preparation kit (NuGEN P/N 0344; NuGEN Technologies, Inc., San Carlos, CA). Input control libraries were prepared from DNA before antibody pulldown. Libraries were quantified and pooled before submission for sequencing. The UC Davis DNA Technologies Core sequenced the libraries as 50-bp single-end reads on an Illumina HiSeq 4000 instrument.

### Quantification and statistical analysis

#### Differential gene expression analysis

We determined differential gene expression (DGX) between the TBI and sham control samples by individually aligning the FASTQ reads from the RNA-seq experiment to the rat genome (rn5) using STAR version 2.4.2a,^25^ after quality-control evaluation using FASTQC version 0.11.8^26^ and counting reads with featureCounts version 1.5.0,^27^ with UCSC gene annotations. We then analyzed samples using a custom R script running the limma-voom model.^28^ For gene expression trajectories across 1 and 14 days, we established sham at day 14 as the baseline, because they had recovered from acute sham injury, and normalized TBI gene expression (reads per kilobase of transcript, per million mapped reads; RPKM), using the DGX determined by the limma-voom method. Differentially expressed genes (DEGs) with similar trajectories were assigned into clusters. We conducted Gene Ontology (GO) analysis using the R clusterProfiler package,^29^ using *p*-value and *q*-value cutoffs of 0.05, for the genes in each of the expression pattern clusters defined at day 1 and day 14.

#### Chromatin immunoprecipitation sequencing data analysis

We quality checked and trimmed adapter sequences from the reads of the FASTQ files from ChIP and input control samples using FASTQC and Trim Galore! Version 0.5.0,^30^ respectively. Filtered reads were aligned to the rn5 genome (UCSC gene annotation) with bwa version 0.7.16a,^31^ with duplicate reads removed with Samtools version 1.8.^32^ We generated read coverage genome-wide estimates and peak calls for sham and TBI samples using the pileup outputs from MACS2 version 2.1.2.^33^ Using custom R scripts, we intersected coverages (pileups) from H3K4me3 in TBI samples and respective sham controls and filtered those annotated to gene promoters. For each individual set (TBI and sham), we ranked the coverages and used the ranks to compare against one another (to compensate for variations in sensitivity among data sets). We assigned up- or downregulated genes associated with each TBI-sham pair at each time point by association with the differential expression (DE) data.

We used the MACS2 bdgdiff function to compare normalized H3K4me3 enrichment, focusing analysis on gene promoters. Log likelihood of differential H3K4me3 between TBI and sham samples was estimated for all genes, and the top 100 up- and down-regulated genes ranked by differential promoter H3K4me3 were selected and tested for enriched GO terms and Reactome and PANTHER pathways using Enrichr (https://amp.pharm.mssm.edu).^34^ To determine whether differences in H3K4me3 change were greater than expected by chance, average log likelihood for differential TBI versus sham H3K4me3 for DEGs for each gene expression trajectory module was compared against genes that were not DE by linear regression, and ANOVA with Tukey's *post hoc* analysis were used to generate statistically significant results.

#### Data and Code Availability

The genomic data generated in this study and presented in this publication have been deposited in the NCBI database and are accessible through GEO Series accession number GSE173975 (https://www.ncbi.nlm.nih.gov/geo/query/acc.cgi?acc=GSE173975) and can be visualized in UCSC track hubs whose information is provided on the Nord Lab GitHub page (https://nordneurogenomicslab.github.io/publications/).

## Results

### Gene expression perturbations after traumatic brain injury are both transient and persistent

Adult male Sprague-Dawley rats were randomly assigned to sham control (*n* = 6) and LFP (*n* = 8) groups (Fig. 1A). These groups were separated for acute assessment 1 day post-injury (*n* = 2 sham, *n* = 4 LFP) and subchronic assessment on day 14 post-injury (*n* = 4 sham, *n* = 4 LFP). RNA-seq was performed on bulk ipsilateral hippocampal tissue. We determined differential gene expression using the multiple-testing–corrected *p* value <0.05 between TBI and sham control at each time point. Full DGX results are reported in Supplementary Tables S1–S3. Overall, 1351 up- and 1013 downregulated DEGs were identified 1 day post-TBI, and 439 up- and 41 downregulated DEGs at 14 days, relative to time-point–matched sham controls.

Sham and TBI samples hierarchically clustered by group and time point, indicating overall DEG signatures that robustly discriminate TBI from sham as well as acute from subchronic time points (Fig. 1B). A volcano plot demonstrates the magnitude of change for all tested DEGs, with the top DEGs (lowest *p* value and/or highest absolute expression fold change) labeled (Fig. 1C). In addition, a principal component analysis (PCA), using full transcriptomic data, similarly captured a well-defined separation of condition and time point (Fig. 1D), with the 1-day TBI signature driving the largest proportion of variance in PCA space (i.e., PC1).

**FIG. 2.**
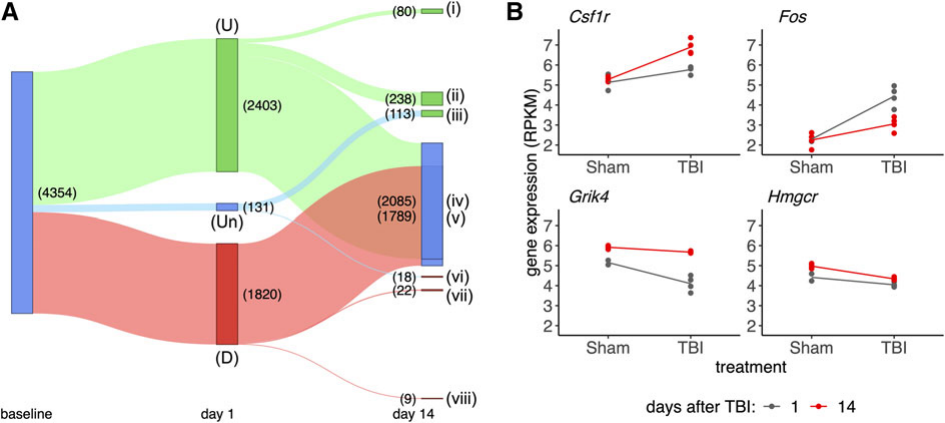
Gene expression trajectories over time after TBI. (**A**) Sankey plot showing the relative gene expression trajectory across the period 1–14 days after TBI (limma-voom model with *p* < = 0.05, expression normalized/corrected for batch effect and sham effect over time). Gene trajectory modules are labeled as follows: (i) denotes persistently upregulated genes; (ii) means acutely upregulated genes showing partial recovery at day 14; (iii) denotes genes with delayed upregulation; (iv) and (v) denote acutely up- and downregulated genes, respectively, showing full recovery at day 14; (vi) represents genes with delayed downregulation; (vii) depicts acutely downregulated genes showing partial recovery; and (viii) denotes persistently downregulated genes. (**B**) Expression level comparisons at both time points, TBI and sham control, for selected genes (*Csf1r*, *Fos*, *Grik4*, and *Hmgcr*) showing different trajectories. Gene expression is given in log RPKM (reads per kilobase of transcript, per million mapped reads). *Csf1r*, colony-stimulating factor 1 receptor; *Fos*, Fos proto-oncogene, AP-1 transcription factor subunit; *Grik4*, glutamate ionotropic receptor kainate type subunit 4; *Hmgcr*, 3-hydroxy-3-methylglutaryl-CoA reductase; TBI, traumatic brain injury.

Genes that were differentially expressed at adjusted *p* value <0.05 at either time point (4354 total genes) were assigned to a coexpression module based on expression changes across time points (Fig. 2A). A relatively small subset of genes was increased early after injury and then further increased in expression at 14 days (80 genes, module i). A larger subset had a larger increase on day 1 as compared to day 14, but remained significantly elevated at the later time point (238 genes, module ii). The largest module of genes (*n* = 2085) was significantly upregulated at 1 day, but was not statistically different from sham at the 14-day time point (module iv). This cluster was followed in size by the module of genes that showed the opposite effect of downregulated at day 1 but statistically similar by 14 days (1789 genes, module v).

There were 22 acutely downregulated genes that demonstrated only partial recovery (module vii) by 2 weeks whereas only nine acutely downregulated genes were even more significantly perturbed at 14 days (module viii). In addition, there were two additional subsets of genes that were not acutely changed on day 1, with DE only on day 14. Of these genes, 113 had delayed upregulation (module iii) and 18 were downregulated only at day 14 (module vi). Overall, this analysis identified clear differences in expression and recovery among genes sensitive to TBI in the ipsilateral hippocampus, which may reflect both the trajectory of recovery and onset of long-term pathology.

### Differentially expressed gene expression trajectory modules are associated with distinct traumatic brain injury pathology

Critically, many of the observed DEGs are related to known changes in protein expression post-injury, the longitudinal design allowed for analyses of the trajectory for such genes over time. For example, Figure 2B shows expression differences for four example genes from four different modules with known TBI associations: colony-stimulating factor 1 receptor (*Csf1r*; neuroinflammation, module iii); Fos proto-oncogene, AP-1 transcription factor subunit (*Fos*; persistent activation, module iv); glutamate ionotropic receptor kainate type subunit 4 (*Grik4*; synaptic signaling, module v); and 3-hydroxy-3-methylglutaryl-CoA reductase (*Hmgcr*; cholesterol synthesis, module vii).

**FIG. 3.**
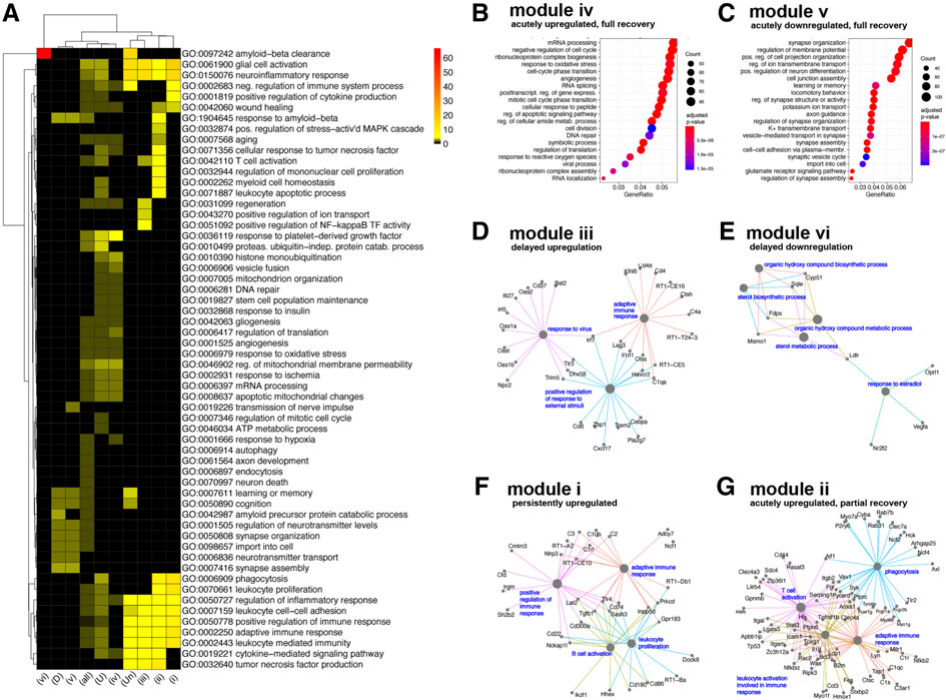
Gene Ontology (GO) analysis stratified according to expression trajectories. (**A**) Heatmap depicting brain-specific or general biological term enrichment for clusters identified as in Figure 2A (adjusted *p* value < = 0.05, 10 top enriched terms for each gene group defined). (**B**) Dot plot showing significance of top 20 GO categories for modules showing acutely upregulated genes showing full recovery at day 14 (*p* < = 0.05, *q* < = 0.05). (**C**) Same as (B) for acutely downregulated genes. (**D–G**) Network plots depicting genes annotated to up to the five most significant brain-related GO terms for the module with (D) delayed upregulation of gene expression in response to TBI, (E) delayed downregulation of gene expression, (F) persistent, increasing perturbation in gene expression, and (G) acutely upregulated genes showing a partial recovery at two weeks. ATP, adenosine triphosphate; MAPK, mitogen-activated protein kinase; mRNA, messenger RNA; NF, nuclear factor; TBI, traumatic brain injury.

Moving from known individual markers to a systems-level approach to identify biological pathways and processes associated, each of the DE trajectories was evaluated by GO analysis performed on module DEG sets (*p*-value and *q*-value cutoffs of 0.05; full results in Supplementary Table S4; Fig. 3A). As expected, the modules harboring DEGs noted above were associated with expected biological functions (i.e., neuroinflammation, synaptic signaling, and cholesterol synthesis, respectively). In addition to these representative genes and pathways, we report the set of enriched GO terms for each trajectory module and show representative DEGs in Figure 3B–G.

Most of the GO terms that were enriched acutely (Fig. 3B, module iv) were associated with biological processes similar to previously described physiological progression of TBI recovery.^35^ As examples, *Capn2* and *Bax*, both involved in regulation of apoptotic cell death, were expressed acutely, but returned to baseline by day 14. Similarly, well-characterized inflammatory markers, such as *Il1b* and *Tnf*, were induced acutely and also returned to baseline by day 14. Other module iv upregulated GO terms included cellular homeostasis and immune/angiogenic response (ribonucleoprotein complex biosynthesis, angiogenesis, DNA repair, cell division, etc.), immediate response to environmental stimulus (response to oxidative stress, cellular response to peptide, etc.), and apoptosis (Fig. 3B, module iv). Module v, representing genes that were acutely downregulated, was enriched for GO terms associated with synapse function and organization, potentially capturing changes in neuronal state and neurotransmission in response to both the acute excitotoxic period and activation of surrounding astro- and microglia (Fig. 3C, module v).

Of genes that are differentially expressed at 14 days, many were also differentially expressed acutely. These include genes in module ii that were upregulated at both time points, which were enriched for GO terms associated with immediate and adaptive immune response and phagocytosis (Fig. 3G). As a representative gene, *Il18* is a cytokine of the IL-1 family involved in neuroinflammation.^36^ Another example, *Tyrobp*, has previously been identified as chronically upregulated post-TBI.^37^ In contrast to the relatively large number of acutely upregulated genes showing partial recovery, only 22 acutely downregulated genes (module vii) showed continued downregulation at day 14, and these genes did not show statistically significant enrichment of specific GO terms.

Although there were a relatively small number of DEGs (80) in module i, genes upregulated acutely and with higher levels at day 14, these were strongly associated with specific GO terms, such as glial cell activation. Likewise, very few genes (nine) exhibited increased downregulation at day 14; however, these genes were not related to brain-specific GO terms. The final modules represent genes that had a pattern of delayed transcriptional perturbation, with DEGs significantly altered only at the 2-week time point. GO terms associated with delayed upregulation (module iii, 113 DEGs) are associated with innate and adaptive immune activation and inflammation (Fig. 3D). As an example, *Nrlp3* has been shown to be a driver of inflammation and immune response post-TBI.^38^ A very limited number of DEGs (18) exhibited a delayed downregulation (module vi) and were associated with sterol biosynthesis and metabolism (Fig. 3E), suggesting a possible change in cell-membrane–related cholesterol homeostasis.^39^

Interestingly, we detected a limited number of gene changes within modules that were not associated with GO terms or brain-specific GO terms. For example, some of the genes represented in module vii include *Cacna1i*, *Cd300lf*, *Cntnap4*, *Cpne7*, *Dhcr24*, *Fam65b*, *Ftl1*, *Hmgcr*, *Lgr4*, *Mlxipl*, *Nrros*, *P2ry12*, *P2ry13*, *Padi2*, *Pnck*, *Ptprcap*, *Ptprn*, *Rgs10*, *Spint1*, *Srebf2*, *T2*, and *Trpc7*. Although each gene has been associated with one or more GO terms, the module as a whole did not show enrichment for one particular ontology. Of note, *Hmgcr* is associated with cholesterol homeostasis, which has been described in module vi. Likewise, module viii includes genes such as *Adra2a*, *Ccdc33*, *Dpp10*, *Fdft1*, *Hmcn1*, *Klhl14*, *Slc38a4*, *Tacr3*, and *Tfrc*, which do not show significant enrichment for any term as a whole, even though each gene is a part of other ontologies and are associated with neurodegeneration.

### Parallel histone H3 lysine residue 4 trimethylation epigenetic changes at differentially expressed gene promoters 1 day after traumatic brain injury

To explore the link between transcriptional changes and TBI-induced acute epigenetic effects, we conducted ChIP-seq with antibodies targeting H3K4me3, a histone H3 post-translational modification found to be a signature for active transcription history that is characteristically found at gene promoters.^40,41^ H3K4me3 is generally associated with transcriptional activation, and, as expected, overall gene expression levels were correlated with H3K4me3 at the promoter.

**FIG. 4.**
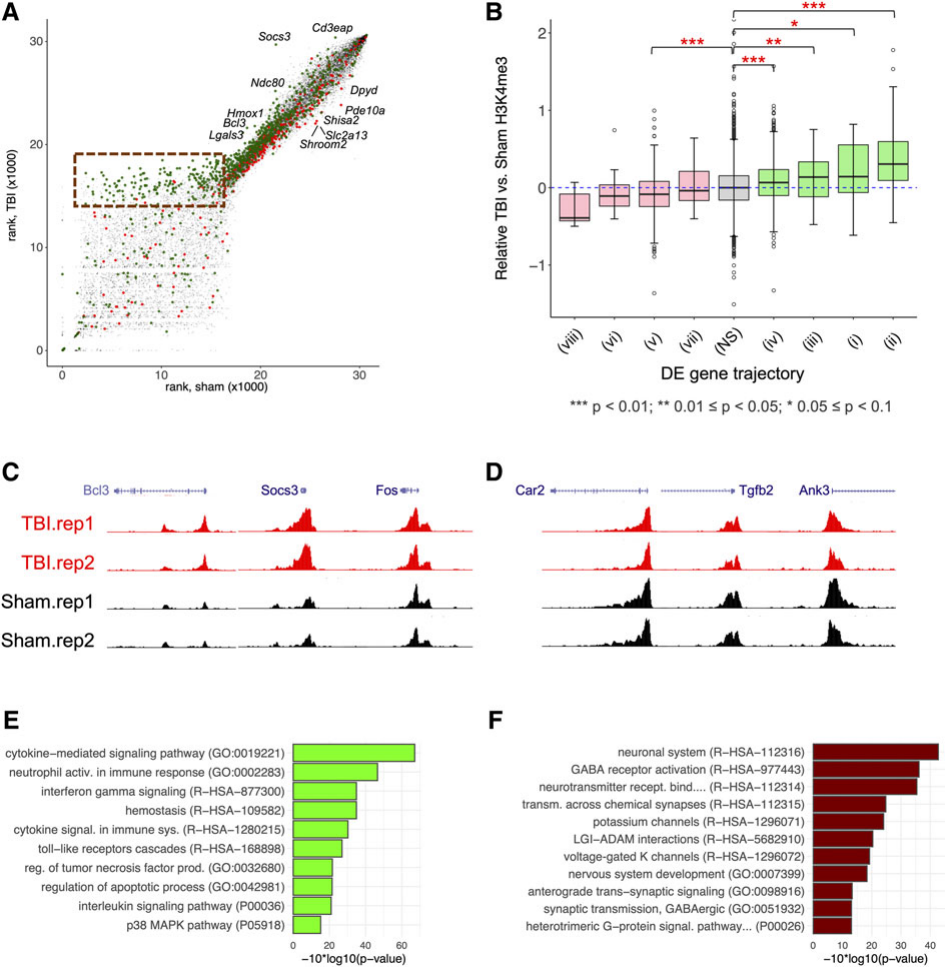
H3K4me3 at promoters of genes differentially expressed after TBI. (**A**) Coverage rank plot for TBI versus sham for H3K4me3 at day 1. *y*-axis is for the TBI sample, whereas the *x*-axis is for the sham control. Each dot is a gene promoter locus present at either of the two conditions. Green-colored dots are associated with promoters of upregulated genes, whereas red-colored ones are for downregulated genes. Region highlighted with dashed red box denotes genes with distinctive H3K4me3 enrichment in TBI at day 1. Dots labeled with name of genes were manually annotated for example genes that showed more prominent differences between TBI and its sham control. (**B**) Box plot showing the distribution of relative likelihood ratios for each of the DE gene trajectories defined in Figure 2A compared to genes that were not differentially expressed (NS). Green- and pink-filled boxes denote up- and downregulated associated genes, respectively; red stars above the plot indicate the statistical significance of Tukey means comparison between groups and the NS group (*p* values shown below the plot). (**C**) Example of a representation of H3K4me3 genomic enrichment coverage region for sham and TBI at day 1 for upregulated loci (*Bcl3*, *Socs3,* and *Fos*). (**D**) Same as (C) for downregulated genes (*Car2*, *Tgfb2*, and *Ank3*). (**E**) Bar plot depicting the most significant function annotation terms (GO biological process, Reactome, and PANTHER pathways) for the upregulated genes showing H3K4me3 differential enrichment. (**F**) Same as (E) for downregulated genes. *Ank3*, ankyrin 3; *Bcl3*, BCL3 transcription coactivator; *Car2*, carbonic anhydrase 2; DE, differential expression; *Fos*, Fos proto-oncogene, AP-1 transcription factor subunit; H3K4me, histone H3 lysine residue 4 tri-methylation; *Socs3*, suppressor of cytokine signaling 3; TBI, traumatic brain injury; *Tgfb2*, transforming growth factor beta 2.

Specifically, in line with DEG changes post-TBI, upregulated genes had generally higher H3K4me3 enrichment in TBI versus sham samples, and the inverse was true for downregulated genes (Fig. 4A). Changes in H3K4me3 in TBI samples were generally subtle, and many DEGs did not exhibit changes at the level of sensitivity of these experiments. However, a small set of upregulated DEGs (*n* = 223) showed strong enrichment of H3K4me3 in TBI samples when compared with sham controls, as indicated in the dashed box in Figure 4A. For these genes, the level of promoter H3K4me3 in sham controls was low or undetectable, suggesting transition from a silent to active transcriptional state post-TBI.

Genes exhibiting this pattern were highly enriched in GO terms associated with leucocyte migration and activation of immune response and phagocytosis (Supplementary Table S5). Although most DEGs exhibited subtle epigenetic impacts, we found significant increases in H3K4me3 for all but one cluster of acutely upregulated expression trajectory modules (*p* < 0.05) and a significant decreases H3K4m3 for acutely downregulated modules (Fig. 4B). Figure 4C,D depicts ChIP-seq coverage genomic representation of H3K4me3 changes for TBI and sham controls at day 1 around up- and downregulated DEGs, respectively. Genes shown are *Bcl3*, *Scocs3*, and *Fos* for upregulated and *Car2*, *Tgfb2*, and *Ank3* for downregulated.

Gene set enrichment analysis was conducted on the set of 100 up- and downregulated genes with the strongest acute change in H3K4me3 (Fig. 4E,F). As noted above, genes with the largest increases in H3K4me3 were associated with inflammation/immune response and were acutely upregulated genes showing partial recovery at day 14. Overall, genes with strong epigenetic changes at day 1 were enriched with GO terms and biological pathways akin to the full set of up- and downregulated DEGs, reflecting the same general pathophysiological processes associated with transcriptional and epigenetic response to TBI. These findings indicate that epigenetic changes in H3K4me3 both parallel and can precede transcriptional changes, given that all trajectory modules showed some evidence for epigenetic changes in the same direction as transcriptional changes.

## Discussion

TBI activates a cascade of events that can lead to acute secondary effects as well as chronic pathology. In the LFP model, cell death peaks in the first 24 h, with negative effects significantly reduced by 1 week post-injury,^42^ and cognitive performance is most significantly diminished in the first weeks post-injury.^43^ Similarly, the acute inflammatory response and persistent neuroinflammation is well characterized,^44^ which is associated with ongoing neurodegeneration.^45,46^ Our results corroborate previous transcriptome studies and recapitulate known physiological pathology that occurs in the injury and recovery.^47–49^ For example, we detected a peak in excitotoxicity acutely post-injury, with DEGs and GO terms associated with cell death mostly absent at the delayed time point. Although downregulation of neurotransmission and synaptic plasticity genes were also obvious acutely, these genes were included in modules that largely resolved by 14 days.

Our results capture neuroinflammation responses that separate into three DEG modules, including those that: 1) peak acutely; 2) maintain similar elevation in the transition from acute to 2 weeks; and 3) are higher at 14 days post-injury as compared to acutely post-injury. Finally, we identified epigenetic substrates of DEG evident at 1 day post-injury. TBI-induced genes exhibited promoter H3K4me3 changes, indicating epigenetic activation as a response to TBI and suggesting that such changes may underlie long-term transcriptional dysregulation.

Many studies have used transcriptomics approaches to understand the molecular and cellular changes in the brain post-TBI, though far fewer have examined genome-wide epigenetic changes. Our study corroborates and expands on this literature. First, the dominant transcriptomic signatures we describe correspond to effects that are well established, including induction of immunity and inflammation, cell death, and downregulation of neuronal genes.^8–12^ This demonstrates the validity of our study, and our systems-level analysis provides further evidence and increased granularity of cellular and molecular outcomes associated with TBI. Second, our 1- and 14-day time points capture the trajectory of recovery during the transition from subacute to chronic, leveraging RNA-seq and systems-level analysis, an improvement in sensitivity compared to previous candidate and microarray time-course studies. Finally, few studies have examined both epigenetic and transcriptomic changes in parallel in the same TBI model.^8,50,51^ These studies have focused on DNA methylation, which has distinct biological relevance compared to H3K4me3, a promoter-associated epigenetic signature that correlates with transcriptional activation.

Transcriptomics studies have consistently identified major DE signatures across various TBI models,^8,47,50,52–58^ and the limited number featuring time-course designs reveal dynamic changes across periods from the hours post-injury to months and even years later.^13,59–62^ Our results support a previously proposed mechanistic model where surviving neurons activate a transcriptomic signature of cellular reprogramming, development, and regeneration in the aftermath of injury,^53^ with relevant terms enriched here in upregulated modules ii and iv. Our findings are also in line with a study that used NanoString to profile candidate genes, finding differences in trajectories of sets of immune and inflammation genes, as well as decreased and shorter persistence of downregulation signatures post-TBI.^60^

A recent microarray study included the same two time points as used here and found similar time-dependent inflammation signatures.^13,59^ Our results are generally aligned with findings from this study, though our methods are anchored on DE changes rather than covariance and are thus by transcriptional pathology. Further, by integrating expression and H3K4me3 ChIP-seq in our comparison, we identified underlying epigenetic activation and repression of differentially expressed loci, as describe below.

As noted, the signatures identified here differ substantially between the genes that are induced (i.e., upregulated) post-injury and those that are downregulated. TBI induces waves of primary (modules ii and iv) and secondary (module iii) induced signatures capturing distinct inflammation and immune responses. In contrast, downregulation was dominated by early changes at day 1, with most genes returning to baseline by day 14 and only a small, but relevant, set of new DEGs emerging at this later time period. Many of the lasting downregulated DEGs are involved in processes relevant to recovery and long-term neurodegeneration post-TBI.

We identified a delayed-onset DEG module comprised of genes related to cholesterol metabolism and amyloid-beta clearance. This signature is of particular interest, given that formation of plaques in the rat model of LFP has not been observed. In addition, a small set of loci associated primarily with sterol metabolism and maintenance were significantly decreased only at the delayed 14-day time point. TBI and cholesterol metabolism have been suggested as causative factors in Alzheimer's and other neurogenerative diseases.^63–66^

There was also a small number of genes (*n* = 9) that were downregulated early, but were even further diminished at the 2-week time point, including *Adra2a*, *Ccdc33*, *Dpp10*, *Fdft1*, *Hmcn1*, *Klhl14*, *Slc38a4*, *Tacr3*, and *Tfrc*. Each of these genes have been reported as downregulated in models of chronic neurodegenerative disease such as Alzheimer's and Parkinson's diseases.^66–77^ These lasting transcriptomic changes may be drivers of functional and cognitive impairment post-TBI. A primary finding from our module-based approach is the identification of distinct, time-dependent sequence of neuroimmune and inflammation DEG modules that are induced post-TBI.

Studies that looked at later time points than this effort using similar rodent TBI models report that persistent immune and inflammation signatures that overlap with modules identified here last up to 2 years post injury,^60^ and that this is, at least in part, attributable to microglial transition to a persistent altered inflammatory state.^61,78^ Some of these long-term immune and inflammation transcriptional signatures can also be modulated by drug treatment.^60,62^ The genes and modules identified by our approach capture molecular mechanisms and represent new potential biomarkers for stage and biological pathway-specific components of recovery and lasting pathology post-TBI. Further, future studies evaluating the intersection between neurodegeneration, neuroinflammation, and neurotransmission over time may provide better insight into why behavior and, in particular, cognition shows some recovery over time, but perhaps not quite to an equivalent performance as an age-matched control.

Although transcriptomics has been widely adopted, far fewer studies have examined genome-wide epigenetic changes post-TBI. Among these, one relevant recent study paired RNA-seq with genome-wide DNA methylation analysis at a single time point, 7 days after fluid percussive injury, and used similar systems-level approaches to examine gene networks and tie epigenetic changes to expression.^8^ In addition to the increased information from inclusion of two RNA-seq time points, our study revealed novel insights versus this previous study through our interrogation of H3K4me3, with revealed global epigenetic changes at gene promoters paralleling expression. Most strikingly, the strongest H3K4me3 increases occurred at promoters of upregulated immune and inflammation loci, representing an epigenetic mechanism that may underlie persistent pathological immune and inflammation post-TBI. In contrast, the differential methylation signatures in the previous analysis were not described as having distinct enrichment for specific transcriptional signatures that we describe.

These differences are likely attributable to specifics of DNA methylation versus post-transcriptional histone modification (here H3K4me3) with regard to epigenetic responsiveness to TBI. Expanded studies of histone post-translational modification changes genomewide, of which ours is among the first, will be particularly relevant in understanding molecular and genomic changes that may be responsive to histone deacetylase inhibitors, which are a promising emerging therapeutic tool in TBI.^16,79,80^

## Conclusion

Given that TBI can result in progressive inflammation and neurodegeneration and lasting behavioral disorders, intersecting transcriptomics and epigenetic changes as the brain transitions from an acute response to recovery and ultimately into the chronic state can provide insight into long-term genomic impacts. Our paired RNA-seq and H3K4me3 ChIP-seq data provide novel insights on the trajectory of transcriptomic signatures and identify epigenetic underpinnings of lasting inflammation and immune expression dysregulation. Moving beyond mRNA, single-time-point transcriptomic studies have also examined long intervening/intergenic non-coding RNA,^81^ microRNA,^82^ and circular RNA^83^ and applied methods to profile exosome RNA,^84^ epitranscriptomic modifications,^85^ and refine readout to the single-cell level.^12^ Future studies are needed to similarly examine the variety of epigenetic modifications associated with gene regulation, to integrate emerging multi-dimensional omics and single-cell data over time toward understanding pathological genomic and transcriptomic changes post-TBI at the level of specific cell types and circuits and evaluate additional chronic time points to determine whether 2 weeks is a time point representative of ongoing dynamic changes or a more stable transcriptomic phase. This initial investigation reveals novel insights into dynamic genomic processes associated with recovery post-TBI. Though limited in scope, our data demonstrate the power of systems-level epigenetic and transcriptomic analyses to understand mechanistic changes in the days to weeks after TBI.

## Supplementary Material

Supplemental data

## Abbreviations Used

ANOVA: analysis of variance
bp: base pair
ChIP: chromatin immunoprecipitation
ChIP-seq: ChIP with sequencing
*Csf1r*: colony-stimulating factor 1 receptor
DE: differentially expressed
DEGs: differentially expressed genes
DGX: differential gene expression
*Fos*: Fos proto-oncogene, AP-1 transcription factor subunit
GO: Gene Ontology
*Grik4*: glutamate ionotropic receptor kainate type subunit 4
H3K4me3: histone H3 lysine residue 4 tri-methylation
*Hmgcr*: 3-hydroxy-3-methylglutaryl-CoA reductase
LFP: lateral fluid percussion
mRNA: messenger RNA
PCA: principal component analysis
RNA-seq: RNA-sequencing
RPKM: reads per kilobase of transcript, per million mapped reads
TBI: traumatic brain injury
