# Supplementary material for: Transcriptional Pathology Evolves over Time in Rat Hippocampus after Lateral Fluid Percussion Traumatic Brain Injury
Source: Neurotrauma Rep. 2021 Nov 23;2(1):512–25. doi: 10.1089/neur.2021.0021 (PMC8667199; doi:10.1089/neur.2021.0021)
Supplement: Supplemental data [file Supp_Table1-5.pdf]

Gene	logFC	AveExpr	t	P.Value	adj.P.Val	B	Sham.1.1	TBI.1.1	TBI.1.2	Sham.1.2	TBI.1.3	TBI.1.4	Sham.14.1	TBI.14.1	Sham.14.2	TBI.14.2	TBI.14.3	TBI.14.4	Sham.14.3	Sham.14.4
S100a10	3.663934	5.086356	20.80223	3.22E-12	3.87E-08	18.18157	4.460789	7.853917	7.957507	4.228565	7.91582	8.273867	4.177031	4.032662	3.577725	3.820684	3.916315	3.843548	3.772036	3.454224
Hspb1	5.978009	5.489637	18.97711	1.17E-11	7.03E-08	16.74535	3.569521	8.989659	9.100563	2.475838	8.946214	8.8006	4.074361	5.265641	3.648551	4.797268	4.930302	5.576895	3.143247	3.61702
Clic1	2.980681	5.373107	17.25745	4.40E-11	1.76E-07	15.78784	4.819083	7.332891	7.551024	4.509141	7.697217	7.969298	3.957761	4.876565	4.023757	4.894014	4.784073	5.119046	3.811576	3.932632
Lsp1	4.016047	4.311866	16.29631	9.74E-11	2.93E-07	14.84223	3.388015	7.068934	6.85369	2.389316	6.938409	6.597528	2.518182	3.749088	3.100223	4.10172	3.953901	3.936334	2.964355	2.932182
Tmbim1	3.20576	6.177155	15.59607	1.78E-10	3.66E-07	14.44964	5.134966	8.284009	8.255485	4.713732	7.917459	8.032532	5.172864	5.751372	5.13958	5.603168	5.366343	6.053848	5.469249	5.61423
Timp1	6.600683	3.84049	15.56904	1.83E-10	3.66E-07	13.76606	2.342161	7.804119	8.164394	0.531921	7.832831	7.935823	1.544223	3.645768	1.393785	2.783118	2.912	3.662244	1.465469	2.050068
Serpina3n	9.395691	1.163608	14.11387	7.00E-10	1.20E-06	10.61579	-0.760787	8.038933	8.573794	-1.388744	7.603583	7.90799	-2.558588	0.301855	-1.536754	-0.311823	-0.411343	-0.434228	-2.232431	-2.653844
Lcn2	7.477657	3.384477	13.36772	1.46E-09	2.19E-06	12.01734	2.32672	9.309475	8.274466	0.621404	8.879516	8.934859	0.118118	2.68033	0.091681	2.454393	3.366837	1.593679	-1.047095	0.499386
Tubb6	5.354449	3.88828	13.21849	1.70E-09	2.27E-06	12.06714	2.927466	6.905792	7.254448	1.273392	7.752757	7.600731	2.433388	3.201289	1.743567	3.249928	2.792614	3.52291	1.859646	2.142093
H19	5.414467	2.320972	12.6645	3.02E-09	3.63E-06	11.22646	0.955837	6.627018	6.37774	1.097434	6.546912	6.007026	-0.529223	1.239467	0.317129	0.799637	1.456531	2.63155	-0.040977	-0.204094
Msn	2.826589	5.539247	12.37157	4.13E-09	4.29E-06	11.35266	4.96336	7.302066	7.767731	4.552446	7.307748	7.930826	4.443022	5.217552	4.534483	4.738473	4.632967	5.357694	4.416965	4.429189
S100a4	3.994297	4.571418	12.33781	4.28E-09	4.29E-06	11.281	3.127854	6.424217	6.508552	2.184345	6.739621	6.761145	2.8014	5.577792	2.846732	5.131403	4.827749	5.690774	2.363894	3.133933
Tagln2	2.804173	6.273968	12.17813	5.10E-09	4.71E-06	11.13535	6.072769	8.301352	8.436269	5.078135	8.309355	8.437818	5.397647	5.491027	5.395739	5.711719	5.38366	5.782499	4.806206	5.258947
Cebpd	2.300227	5.016104	12.04268	5.91E-09	5.08E-06	10.99994	4.29613	6.517434	6.830278	4.270665	6.609099	6.356803	4.113042	4.961999	4.316905	4.833203	4.72823	5.08342	3.817137	3.551406
Flnc	3.254764	5.527639	11.88222	7.07E-09	5.66E-06	10.82377	4.432216	7.264765	7.921111	3.980586	7.3272	7.284285	4.348928	5.629	4.081214	5.102246	5.075905	5.928941	4.383621	4.673894
Hck	2.886093	2.949575	11.80797	7.68E-09	5.77E-06	10.61921	2.127257	4.528825	4.839859	1.548999	4.866025	4.472821	1.862293	3.301815	1.549949	3.214035	2.977336	3.277021	1.347449	1.659607
S1pr3	3.87344	5.332262	11.64989	9.18E-09	6.49E-06	10.57103	3.608352	7.046003	7.90707	3.268089	7.075409	7.15268	4.129307	5.742456	4.01306	5.192625	5.082283	6.116878	4.130242	4.24638
Stat3	2.129605	6.700065	11.49387	1.10E-08	7.32E-06	10.35969	6.11158	8.018967	8.562255	5.808165	7.909518	7.862034	5.730014	6.538597	5.915612	6.382664	6.358399	6.735236	5.933365	5.952165
Gfap	3.086784	11.87529	11.43255	1.18E-08	7.35E-06	10.31776	10.60808	13.61985	12.76622	9.900098	13.66999	13.31926	10.49915	12.66397	10.49452	12.77598	12.06002	12.77121	10.30069	10.80564
Trim47	3.056795	4.16217	11.3986	1.22E-08	7.35E-06	10.27765	3.136736	6.037533	6.04958	2.41457	5.593651	5.517933	3.713543	4.026799	3.495578	3.896239	3.678847	3.995035	3.098279	3.728093
Tmem100	1.923324	5.173492	11.30014	1.37E-08	7.84E-06	10.14303	4.97331	6.846174	6.986708	5.186565	6.869507	7.308819	4.150713	4.294162	3.918782	4.445302	4.168924	4.588013	4.314529	4.432837
Cd14	5.582742	1.795636	11.24651	1.46E-08	7.97E-06	9.329863	0.176305	5.42163	5.335399	-0.502853	5.244216	5.059383	0.202532	1.658838	-1.536754	1.064403	2.014226	1.97042	0.434663	-0.297732
Ehd2	2.18312	4.159327	11.15742	1.62E-08	8.46E-06	10.011	3.522857	5.454283	5.412825	3.035565	5.52075	5.379636	3.537848	3.897831	3.907275	3.867646	3.639021	4.018805	3.278395	3.858608
Tyrobp	2.375201	5.291039	10.95431	2.06E-08	1.03E-05	9.754603	4.495729	6.387314	6.600475	3.810268	6.856462	6.201252	3.756062	5.933183	4.101553	5.907019	5.88226	6.160272	3.844626	4.19144
Ptpn6	2.153314	4.789902	10.77172	2.56E-08	1.23E-05	9.549118	3.881004	5.888303	5.652234	3.468286	6.111796	5.599745	3.742028	5.499519	3.634661	5.3205	5.30222	5.744511	3.515771	3.769079
Clec4a1	4.249031	2.477979	10.66216	2.93E-08	1.35E-05	9.18582	1.541696	4.63158	5.408707	0.22441	5.111109	4.934612	0.68572	2.738882	0.628769	2.681644	2.592682	2.880186	0.182287	0.970221
Pdlim4	2.366896	6.091894	10.60301	3.15E-08	1.40E-05	9.299479	5.871991	7.804119	7.675917	4.708637	7.645071	7.454469	5.038651	6.049074	5.102217	6.035967	5.826363	5.996342	4.992221	5.114213
Slc39a1	1.57301	6.433184	10.56371	3.30E-08	1.42E-05	9.242865	5.855945	7.166544	7.159619	5.48363	7.423408	7.206771	5.914952	6.488286	5.901181	6.387707	6.330987	6.678164	5.911436	6.17548
Ifi30	2.466519	4.892169	10.47556	3.68E-08	1.48E-05	9.190479	3.973144	6.027289	5.960495	3.1898	6.214937	5.888325	3.261094	5.893462	3.555781	5.606633	5.512044	6.076676	3.502026	3.901371
Alox5ap	2.174112	2.8749	10.47007	3.71E-08	1.48E-05	9.19134	2.555898	4.649501	4.955402	2.511387	4.635794	4.515605	1.297885	2.555575	1.717448	2.184529	2.616315	2.516041	1.724133	2.087586
Plin2	2.749341	4.321465	10.39964	4.04E-08	1.57E-05	9.104037	3.453461	5.773142	6.347942	3.1898	6.133587	5.96133	3.436917	4.481568	3.038267	3.890566	3.70112	4.625653	3.195422	3.372991
Tagln	3.653578	6.19746	10.33962	4.36E-08	1.63E-05	8.984981	5.566568	8.508243	7.814392	3.906865	8.637087	8.497554	5.417651	5.669074	5.495173	5.770048	5.221695	5.725858	4.987296	5.578442
Sbno2	2.353791	6.297447	10.31961	4.47E-08	1.63E-05	8.949004	5.057571	7.661641	7.747223	5.015553	7.254698	6.885424	6.085815	6.3425	6.001999	6.359234	6.031446	6.269325	5.507587	5.967339
Fabp5	1.635038	6.373372	10.28213	4.68E-08	1.65E-05	8.887302	6.032885	7.422907	7.343427	5.44432	7.564359	7.144525	5.790926	6.261188	5.95247	6.229289	6.170637	6.332336	5.75073	5.807897
Nek6	1.413054	6.290337	10.22668	5.02E-08	1.65E-05	8.816645	5.878625	7.056675	7.112294	5.492552	7.27146	6.939845	5.630681	6.422825	5.7739	6.335418	6.407481	6.5322	5.697451	5.53497
Cd44	3.594498	5.207556	10.21861	5.07E-08	1.65E-05	8.846978	4.026686	7.442741	8.43171	4.399576	7.467717	7.898053	3.231264	5.418994	2.882434	4.716194	4.387261	5.657847	3.515771	3.503633
Hmox1	3.676974	4.426435	10.21844	5.07E-08	1.65E-05	8.887127	3.205888	6.174829	7.332962	2.613062	6.637889	6.08106	2.688847	4.937208	2.894141	4.549787	4.530966	4.854238	2.771602	2.814049
Fgr	3.419483	2.987706	10.16306	5.44E-08	1.72E-05	8.78236	2.01613	4.71337	5.35122	1.571628	5.472498	5.127168	1.258992	2.709903	1.29127	2.934159	3.03984	3.644018	1.316372	1.684306
Flna	2.59268	7.139738	10.1102	5.81E-08	1.79E-05	8.670516	6.252057	8.428751	8.165004	5.158812	8.270117	8.293464	7.221857	6.897828	7.070835	6.983775	6.736824	7.049833	6.422655	7.018065
Pdpn	2.757542	6.590064	10.06683	6.14E-08	1.80E-05	8.613364	6.541762	8.191983	8.853598	5.368806	9.005861	8.78378	4.912754	6.261188	5.134654	6.136463	6.102197	6.53385	5.145962	5.311421
Lgals3	6.582918	3.849046	10.05923	6.20E-08	1.80E-05	8.510484	1.669604	7.131374	7.207813	0.041976	7.698806	7.225792	0.952703	5.167806	0.736738	4.250879	4.056804	5.49113	1.316372	1.32462
Ncf2	2.819812	2.385844	10.04865	6.29E-08	1.80E-05	8.548555	1.212615	3.822768	3.89502	1.097434	4.102065	3.884005	1.372662	2.875639	1.393785	2.423294	2.494078	3.145002	0.921344	1.158276
Gldn	3.316057	4.418274	9.980062	6.86E-08	1.92E-05	8.577952	3.035892	5.970204	7.076834	3.515737	6.206022	7.112365	3.616879	3.614836	3.648551	3.48645	3.474203	3.686193	3.719553	3.791988
Gmfg	2.054473	2.744672	9.908889	7.52E-08	2.05E-05	8.49846	2.63325	4.271601	4.566831	2.108996	4.230707	4.495797	1.63815	2.862532	1.29127	2.831299	2.468337	2.726993	1.218908	1.386135
Sdc1	5.452641	1.559661	9.847274	8.14E-08	2.17E-05	7.98998	0.785575	5.003124	5.746607	-0.81875	5.305596	4.928293	-0.399109	0.923327	-0.632503	0.280604	1.319276	1.375373	-0.512812	-0.297732
Lyve1	2.185223	2.271802	9.80969	8.55E-08	2.23E-05	8.367052	2.246933	4.472889	4.305558	2.029493	4.113552	4.304105	1.000645	1.155984	1.255413	1.253084	1.230182	2.046399	1.465469	1.355706
Spp1	10.11298	4.758084	9.766931	9.04E-08	2.25E-05	7.951931	0.307952	10.24801	9.718905	-0.093872	10.22257	10.19133	1.258992	6.185581	-0.080923	5.322609	5.685448	7.522684	0.375586	0.321874
Ifitm3	3.743074	3.985969	9.764863	9.06E-08	2.25E-05	8.313456	2.927466	5.85908	6.027371	1.353927	5.866839	5.493884	2.893159	4.584936	2.707706	3.978769	3.827988	4.637984	2.701169	3.097617
Capg	3.918065	4.350575	9.756982	9.16E-08	2.25E-05	8.30618	3.395435	6.676655	6.635548	2.045748	6.842148	6.192489	2.644268	5.039747	3.130231	4.643844	3.802462	4.856878	2.562371	2.570827
Fn1	2.620235	6.937656	9.68942	1.00E-07	2.40E-05	8.114986	6.709648	8.746825	9.304366	5.827097	8.57231	8.92537	6.381221	5.875632	6.707663	5.83352	5.357605	6.207579	6.142728	6.552757
Casp4	3.050107	3.162325	9.671074	1.02E-07	2.40E-05	8.167371	1.80948	4.916971	4.924971	1.526009	4.418385	4.452412	2.078759	3.784265	1.867508	3.58107	3.165302	3.827486	1.770732	2.387449
Itga5	3.379646	3.321005	9.660504	1.04E-07	2.40E-05	8.178766	2.595092	5.382875	5.839269	1.548999	5.335334	5.018319	2.415815	3.070611	2.107651	2.998871	2.627988	3.46871	1.859646	2.446395
LOC691141	3.371291	1.690674	9.645253	1.06E-07	2.40E-05	7.871518	0.485316	3.874479	3.776458	0.166125	3.378569	3.390443	0.851758	1.564863	0.17081	1.652245	1.864385	2.136071	0.375586	0.65743
Tnfrsf1a	2.337409	5.177126	9.606415	1.12E-07	2.48E-05	8.040445	4.878536	6.593465	7.01	3.888957	6.740238	6.463363	3.863678	5.24811	3.83015	5.107147	5.101249	5.669908	4.038003	4.102607
Ripk3	3.843672	1.925372	9.542816	1.21E-07	2.65E-05	7.619781	0.369528	3.643613	3.426105	-0.502853	3.900209	3.563537	0.743216	2.603631	0.787836	2.543858	2.049375	2.726993	0.546019	1.193114
Myl12a	1.920048	5.497183	9.492531	1.30E-07	2.75E-05	7.870087	5.254031	6.554366	6.645497	4.405886	7.008791	6.742055	5.083863	4.971187	4.972951	5.171725	5.136409	5.265326	4.731605	5.056258
Maff	4.244743	1.872076	9.477953	1.32E-07	2.75E-05	7.768531	0.739674	5.10405	5.086634	0.485009	4.715852	4.206507	0.903114	0.705741	0.97585	1.21727	0.676952	1.219993	0.745852	0.119416
Myo1f	2.587428	4.304368	9.476484	1.33E-07	2.75E-05	7.938604	3.214303	5.099711	5.332504	2.451642	5.780598	5.332553	3.280644	5.154424	2.962454	5.094864	4.820137	5.453251	3.079891	3.310901
Ier5l	1.518213	4.050266	9.453874	1.37E-07	2.78E-05	7.877738	3.543041	4.953495	5.34405	3.665254	5.111109	5.057453	3.356296	3.798099	3.563133	3.962637	3.627438	4.014082	3.445698	3.365375
Spi1	2.029959	4.022898	9.441459	1.39E-07	2.78E-05	7.8815	3.522857	5.134062	5.04656	2.787519	5.346735	5.104925	2.717819	4.860173	2.55384	4.324262	4.549498	4.735888	2.816716	2.942457
Ptpn7	2.61318	1.704466	9.430225	1.41E-07	2.78E-05	7.648244	0.369528	2.953644	3.042552	0.577356	3.188895	2.898483	0.563361	2.14364	0.736738	2.108365	1.978199	2.502617	0.649393	0.753927
Fxyd5	2.051239	5.857112	9.399746	1.47E-07	2.85E-05	7.727976	5.711829	7.395292	7.319865	5.143788	7.707045	7.467966	4.455977	5.625125	4.768722	5.655958	5.543201	6.004699	4.178567	5.056258
Gpnmb	4.241738	6.446878	9.380039	1.51E-07	2.88E-05	7.735822	4.899562	7.997554	8.676691	4.062753	9.432911	8.726352	3.797364	7.741688	4.055381	7.014096	7.123435	8.711929	3.969738	4.088797
Plp2	3.794276	1.447453	9.357028	1.55E-07	2.92E-05	7.40845	0.369528	3.240489	3.555895	-0.502853	3.746377	3.756629	0.202532	1.239467	0.97585	0.799637	1.199219	1.593679	0.649393	0.257494
Steap4	3.922988	1.137391	9.344217	1.58E-07	2.92E-05	7.411213	-0.046857	3.655486	4.275724	-0.02435	3.568609	3.563537	0.202532	0.972957	-0.175642	0.134646	0.263333	0.514819	-0.040977	0.119416
Fam38a	2.390571	4.867963	9.321304	1.63E-07	2.97E-05	7.701972	3.848935	5.977317	6.024683	3.114742	5.69653	5.690919	4.380979	5.268128	4.076084	5.15289	4.845355	5.698154	4.056929	4.384671
Csf2rb	5.077299	3.421508	9.281974	1.72E-07	3.09E-05	7.643384	1.853246	6.225489	6.825652	0.705659	6.134527	5.90344	0.563361	4.229271	1.255413	3.463704	3.525031	4.706916	1.377871	1.473753
Emp3	3.729597	2.745275	9.259867	1.77E-07	3.12E-05	7.608977	2.127257	4.670131	4.936458	0.386344	5.062547	4.847983	1.372662	2.68033	1.426399	2.529327	2.455292	3.376046	1.150071	1.779078
Cyba	2.478902	4.079096	9.252558	1.79E-07	3.12E-05	7.638941	3.247483	5.269631	5.255111	2.475838	5.642047	5.067073	3.060831	4.624294	3.006262	4.531675	4.380377	5.037614	2.58862	3.041369
Tnfrsf12a	3.324667	3.257908	9.219359	1.87E-07	3.22E-05	7.606496	2.342161	5.35766	6.395188	2.337441	5.416407	5.391936	1.726334	2.364261	2.127623	2.923085	2.729	2.203999	2.301782	2.228598
Hcls1	2.309977	3.794141	9.198414	1.93E-07	3.25E-05	7.566369	3.100876	4.984419	4.938364	2.613062	5.517871	5.129006	2.717819	4.332691	2.568526	3.92982	3.990531	4.526331	2.253378	2.660694
Bcl3	4.877348	2.277674	9.191613	1.95E-07	3.25E-05	7.289895	0.369528	5.080024	5.132299	-0.166914	4.930226	4.277834	1.047044	3.137429	0.837186	2.499819	2.388236	2.502617	-0.123798	0.606649
Myc	2.033769	4.032733	9.075391	2.28E-07	3.75E-05	7.35959	3.410162	5.307749	6.085277	3.756964	5.685027	5.381179	3.137884	3.645768	2.995434	3.448339	3.792123	3.895831	3.079891	2.952659
Arpc1b	2.797179	5.033872	9.011168	2.49E-07	4.05E-05	7.258753	4.523084	6.492828	6.478091	2.960388	6.87289	6.147857	4.091065	5.235456	4.002283	5.213226	5.305858	5.408965	3.658907	4.147707
Ybx3	1.372541	5.969203	8.978162	2.61E-07	4.14E-05	7.134511	5.469722	6.93592	6.738111	5.801	7.350445	7.008854	5.363679	5.756695	5.393679	5.655958	5.68405	5.741657	5.347714	5.348595
Fermt3	2.805036	4.038531	8.975137	2.62E-07	4.14E-05	7.274911	2.977763	5.331995	5.349789	1.910273	5.322193	4.804937	3.221182	4.710941	3.23521	4.556968	4.192699	4.926391	2.913344	3.212449
Psmb9	2.063679	3.055452	8.961221	2.67E-07	4.17E-05	7.260526	2.63325	4.286948	4.172648	1.760745	4.094356	4.317063	1.782265	3.40486	2.04602	3.352542	3.228006	3.529544	2.022549	2.372328
Ppp1r18	1.830892	4.949041	8.950291	2.71E-07	4.18E-05	7.150473	4.618111	6.044322	6.038962	3.701615	6.059303	5.741059	4.27775	5.05423	4.15118	5.190317	4.949035	5.318009	4.070962	4.129836
Rac2	2.753386	3.90787	8.908822	2.87E-07	4.37E-05	7.177972	3.205888	5.053348	5.435939	2.045748	5.73794	5.095555	2.613764	4.710941	2.583064	4.268484	4.582868	4.789383	2.220182	2.516844
Hapln1	-1.829173	4.308956	-8.898129	2.91E-07	4.38E-05	7.076299	5.231406	3.263946	3.301354	5.530587	3.488528	4.181925	4.362751	4.388622	4.321248	4.233056	4.49636	4.619447	4.549825	4.443728
Nes	3.68575	3.004006	8.867779	3.04E-07	4.51E-05	7.086865	1.853246	5.039823	4.254466	0.386344	4.820023	4.736655	3.169687	2.619301	2.508863	2.795315	2.568656	2.568523	2.333172	2.685374
Sox7	3.202463	2.628599	8.81838	3.26E-07	4.78E-05	7.065842	2.542593	4.755351	5.803125	1.548999	5.128199	5.063233	1.782265	1.356279	1.959523	1.704918	1.482482	1.375373	1.150071	1.529341
Clec4a3	3.59992	2.003549	8.772327	3.48E-07	5.03E-05	6.942632	1.212615	4.064569	4.897808	0.705659	4.545387	4.413727	-0.399109	2.38274	0.007959	2.256873	1.557647	2.568523	-0.123798	0.65743
Rhoc	1.93716	5.665996	8.711621	3.79E-07	5.42E-05	6.770953	5.488925	7.185605	6.827195	4.473536	6.906847	6.703716	5.067074	5.300076	5.050846	5.553767	5.368084	5.417931	4.792156	5.223575
Arhgap30	2.384197	3.324765	8.684228	3.94E-07	5.57E-05	6.874136	2.682606	4.332034	4.981512	2.108996	4.750601	4.900582	2.100922	3.517851	2.223535	3.335938	3.331578	3.895831	1.983515	2.59708
Tpm4	1.886762	5.589247	8.625686	4.28E-07	5.89E-05	6.641715	5.426916	6.76356	6.704354	4.800109	7.152142	7.348409	5.044381	5.275563	5.016447	5.049759	4.908931	5.355829	4.615127	4.825924
Npl	1.376568	4.069348	8.621037	4.31E-07	5.89E-05	6.716929	3.782575	4.814794	5.168468	3.50989	5.163626	4.889781	3.454237	4.208703	3.432752	3.984107	3.93523	4.069761	3.270309	3.388105
Cd74	2.538284	6.89513	8.620538	4.31E-07	5.89E-05	6.636939	5.516408	7.318028	7.628312	4.654018	7.86971	7.637353	4.722163	8.860466	4.13647	9.046928	9.227651	9.40135	5.330318	5.208755
Litaf	1.456049	6.101186	8.580411	4.57E-07	6.17E-05	6.557529	5.994313	7.127117	7.462093	5.6695	7.417242	7.136324	5.038651	5.81058	5.308757	5.635752	5.887125	6.158133	5.343866	5.452898
Psmb10	1.536848	4.493499	8.554528	4.74E-07	6.33E-05	6.604162	4.132599	5.581085	5.505713	3.578533	5.251166	5.170651	3.999651	4.60868	3.996864	4.429738	4.489977	4.229042	3.949625	4.060775
C5ar1	3.773898	2.468017	8.538596	4.85E-07	6.39E-05	6.654294	1.371547	4.24827	5.195808	1.000693	5.282035	4.85244	0.851758	2.665313	0.736738	2.088679	2.442128	2.880186	1.218908	0.190106
Cnn3	1.467393	8.274769	8.531947	4.89E-07	6.39E-05	6.498818	8.384076	9.449569	9.538144	7.658388	9.519743	9.454741	7.413901	7.921493	7.459073	7.917743	7.885216	8.243857	7.452922	7.553632
Tgfbi	3.094617	3.84726	8.512809	5.03E-07	6.50E-05	6.617805	2.742017	5.333844	6.560204	2.57996	5.544991	5.488167	3.231264	2.835955	3.225976	3.195748	3.302737	2.941167	3.286436	3.73402
Serping1	3.466453	5.912543	8.49344	5.17E-07	6.61E-05	6.483173	5.268246	8.047688	7.563734	3.419222	7.761606	7.727466	3.654827	7.278825	3.540964	6.555753	6.412551	7.365173	3.772036	4.458122
Fcgr2b	3.00686	4.32052	8.485097	5.24E-07	6.62E-05	6.568001	3.100876	5.840998	6.163932	2.57996	5.674724	5.598419	2.186319	5.209811	2.223535	5.131403	5.037033	6.128942	2.860463	2.879681
Mmp19	2.692358	2.621298	8.477332	5.29E-07	6.63E-05	6.581791	1.514695	3.943895	4.000932	1.327578	4.05518	4.281144	1.576217	2.964197	1.819202	2.221154	2.940364	3.261164	1.1849	1.931282
Cd63	2.266501	6.740298	8.463546	5.40E-07	6.69E-05	6.393192	6.242832	7.82956	7.729724	4.886126	8.033623	7.684676	5.995536	6.810945	5.985677	6.961623	6.815497	7.180452	5.890488	6.335095
Fgl2	3.086311	3.325257	8.453701	5.48E-07	6.72E-05	6.548366	2.144973	5.438908	5.934953	2.523044	4.8728	5.38426	1.836107	3.159031	1.636128	2.770817	2.792614	3.732929	2.285827	2.261799
Gadd45a	2.257261	3.685084	8.388564	6.02E-07	7.28E-05	6.410971	3.31946	5.101882	5.725795	2.943136	4.930226	5.70331	2.613764	3.645768	2.446625	3.12019	3.639021	3.587916	2.62712	2.341603
Myd88	1.654771	3.862093	8.381572	6.08E-07	7.28E-05	6.383341	3.582581	5.057828	5.393507	3.247155	5.050645	4.709886	3.094359	3.770297	2.917273	3.759747	3.66758	4.014082	2.964355	2.962789
Klf6	1.604747	5.559296	8.377044	6.12E-07	7.28E-05	6.263533	5.466203	6.872481	7.529695	5.537788	6.798928	7.224073	4.764433	4.886311	4.530738	4.650573	5.010524	4.980656	4.974911	4.642728
Ncf4	5.001885	2.237256	8.348397	6.38E-07	7.45E-05	6.154486	0.031429	5.148837	4.546985	-0.502853	4.735811	4.040642	1.091998	2.665313	1.549949	2.454393	2.544222	2.489067	0.111637	1.048408
Fcgr3a	3.64947	3.028245	8.341964	6.44E-07	7.45E-05	6.394433	1.619793	5.414661	4.581538	1.158502	5.370867	4.594888	1.697535	3.000548	1.60797	3.258764	3.149191	3.931333	1.521045	1.779078
Mcm6	2.550449	3.229625	8.33108	6.55E-07	7.45E-05	6.36952	2.742017	4.899604	4.340564	2.093442	5.264968	5.240331	2.534559	2.781287	2.47808	2.572488	2.873292	2.941167	2.333172	2.325991
Lcp1	1.834905	6.100423	8.330689	6.55E-07	7.45E-05	6.190901	5.45204	7.298284	7.352388	5.953093	7.788431	7.716199	4.781687	5.880517	4.846099	5.664295	6.044571	6.492005	5.05004	5.114213
Kank4	-1.729464	3.451316	-8.328521	6.57E-07	7.45E-05	6.334051	3.912376	2.279532	2.434653	4.331596	2.586747	2.349037	3.769961	3.923301	3.980484	3.310669	3.944596	3.637891	4.121279	3.901371
Psme1	1.288022	5.818137	8.3054	6.80E-07	7.63E-05	6.155118	5.780028	6.910754	6.685757	5.295932	6.826528	6.861852	5.138455	5.708064	5.112276	5.829078	5.660089	5.740228	4.737483	5.198074
Mvp	2.105564	5.460128	8.290152	6.95E-07	7.73E-05	6.164802	5.235546	6.914464	6.649574	4.022255	6.806025	6.492213	4.571588	5.689629	4.405458	5.510034	5.38366	5.728743	4.266558	4.809178
Anpep	3.313785	3.862866	8.235112	7.54E-07	8.31E-05	6.202221	3.388015	5.361289	6.352951	2.310787	6.528437	6.234928	2.880403	2.91426	2.984524	3.258764	2.782205	3.794816	2.363894	3.079111
Ctsz	2.354047	5.745017	8.224367	7.66E-07	8.36E-05	6.078773	4.938178	6.634536	6.433524	3.829173	7.179725	6.618616	4.455977	6.782522	4.417676	6.570021	6.497725	7.078391	4.216936	4.817576
Ncf1	1.970674	4.212451	8.202856	7.90E-07	8.48E-05	6.174437	2.875353	4.499482	4.408127	2.350586	4.875052	4.446528	3.180134	5.855927	3.140097	5.644206	5.71858	6.144152	2.838755	3.11589
Rgs1	3.212526	1.805303	8.202378	7.91E-07	8.48E-05	6.162243	0.995438	4.240408	4.432669	0.74601	3.76592	3.632543	0.903114	1.830204	-0.080923	1.804823	1.844511	2.300247	-0.123798	-0.297732
Ret	2.575302	4.58766	8.187577	8.08E-07	8.60E-05	6.063465	3.748215	6.240347	7.089752	3.791112	6.074077	5.947855	3.479833	3.972932	3.606474	4.031278	3.827988	4.689249	3.989574	3.820121
Gadd45g	2.196947	4.205584	8.152727	8.51E-07	8.97E-05	6.010072	4.087502	5.797457	6.630252	3.504019	5.726763	5.737448	3.383673	4.009066	2.984524	3.709073	3.925804	3.843548	3.013624	2.635584
Nfkbiz	1.945194	2.260974	8.12169	8.91E-07	9.31E-05	6.08397	1.741237	3.953545	4.139901	2.13961	3.775594	3.622139	1.297885	2.186344	1.01926	1.898256	2.197736	2.2368	0.598632	1.260365
Vat1	1.361366	6.199808	8.073209	9.58E-07	9.92E-05	5.804509	5.771499	6.756687	6.852175	5.213795	7.006739	6.77823	5.448228	6.598126	5.546148	6.599881	6.402392	6.820209	5.320563	5.706042
Rin3	1.762921	3.758841	8.066097	9.68E-07	9.94E-05	5.929516	3.730722	4.859106	4.766671	2.697792	5.150675	4.998346	2.787802	3.749088	2.810125	3.790537	3.744661	3.890687	2.84965	2.932182
Stk40	1.35743	6.530759	8.052783	9.87E-07	0.000101	5.763575	6.270333	7.532393	7.639776	5.763408	7.193321	7.121627	6.132288	6.39351	6.092423	6.524956	6.330987	6.495398	5.954966	6.003364
Mcm2	2.11511	3.581729	8.028452	1.02E-06	0.000103	5.9027	3.064101	5.112689	4.346317	2.523044	5.13948	4.953408	3.01488	3.321106	2.962454	3.186517	3.265855	3.619352	2.783013	3.002614
Fcgr1a	3.2735	2.682043	8.000605	1.07E-06	0.000107	5.897486	1.787089	4.298352	4.503885	0.43652	4.457973	3.901317	1.754571	2.875639	1.663747	2.998871	2.592682	3.56872	1.347449	1.708589
Fgfrl1	1.581249	5.2022	7.966828	1.12E-06	0.000112	5.6733	5.043471	6.381964	5.994779	4.277563	6.406884	6.136023	4.39898	4.934079	4.596704	5.162339	5.010524	5.323745	4.488413	4.722402
Sptssb	-2.001692	2.639035	-7.943488	1.16E-06	0.000114	5.783681	3.627383	2.493084	1.554575	4.281	2.28197	1.708392	2.688847	2.619301	2.55384	2.956054	2.639567	2.390473	2.62712	2.802815
Rnh1	1.488126	5.526203	7.934692	1.18E-06	0.000114	5.628693	4.95334	6.261865	6.197736	4.196165	5.939636	5.801089	5.495004	5.689629	5.42831	5.64589	5.576715	5.519733	5.334202	5.363965
Rras	1.827606	4.464896	7.934395	1.18E-06	0.000114	5.670043	4.373312	6.066725	5.47059	3.468286	5.723019	5.650538	4.051782	4.026799	3.985965	4.12123	3.972332	4.105722	3.536146	4.037
Zfp36	2.304069	4.910158	7.933687	1.18E-06	0.000114	5.638144	4.602167	6.729564	7.267392	4.098255	6.303692	6.267836	3.857182	4.696241	4.342766	4.560545	4.231474	4.701051	3.36447	3.786295

Clec5a	2.829184	1.999584	7.920569	1.20E-06	0.000115	5.783949	0.955837	3.914553	4.311452	1.353927	4.074901	3.485336	-0.067151	2.227821	0.317129	2.529327	2.100543	2.910998	0.111637	0.383504
Fcer1g	2.155823	6.136258	7.915907	1.21E-06	0.000115	5.567602	5.841078	7.402796	7.493538	4.906296	8.108489	7.075188	4.34429	6.808384	4.797058	6.453641	6.535402	6.895326	4.637302	4.639574
Emp1	3.391539	3.609166	7.871256	1.30E-06	0.000122	5.651142	3.776905	5.890816	7.040481	2.061822	5.983075	6.088636	2.814871	2.227821	2.127623	2.469694	2.506778	3.009199	2.060555	2.673087
Lyz2	3.194257	6.205028	7.861758	1.32E-06	0.000122	5.496805	5.507875	7.684999	8.619197	4.677679	8.663377	8.147625	4.583442	6.18821	4.628589	5.457277	5.88226	7.265372	4.737483	4.858845
Cp	2.675975	4.952248	7.858665	1.32E-06	0.000122	5.563408	4.319721	6.288796	6.342196	3.197095	6.216714	6.765879	3.669732	5.837624	3.456633	4.902496	4.920843	5.819511	3.665088	3.998126
Apobec1	2.508274	2.595372	7.84477	1.35E-06	0.000124	5.681721	1.741237	4.042133	3.8018	1.478901	4.28928	4.196023	1.576217	3.534475	1.255413	2.966878	2.782205	3.30822	0.375586	1.355706
Prkcdbp	1.943208	4.519038	7.815952	1.41E-06	0.000128	5.484896	4.284188	5.703367	5.392117	3.600707	6.125099	6.25021	3.957761	3.972932	3.985965	4.349296	3.995046	4.069761	3.695599	3.96323
Nol3	1.139881	5.107151	7.806535	1.43E-06	0.000129	5.418666	4.889087	6.168628	5.788409	4.738944	6.114657	5.732621	4.682297	4.876565	4.673435	4.815347	4.686975	4.843629	4.722744	4.814782
Slc6a11	-1.348341	4.9179	-7.801774	1.44E-06	0.000129	5.426112	5.566568	3.972652	4.251403	5.220971	3.882413	4.067729	5.211572	4.889545	5.395739	4.930413	5.071637	5.177238	5.77665	5.493644
Odc1	1.07713	6.409141	7.789726	1.47E-06	0.00013	5.352856	6.36029	7.149854	7.356669	6.147318	7.305663	7.50807	5.998491	5.852616	5.942639	6.030818	6.26617	6.028312	5.965021	5.835724
Cpne8	1.161201	4.770194	7.786294	1.48E-06	0.00013	5.407352	4.586045	5.459372	5.738974	4.324952	5.630099	5.607677	4.56363	4.548571	4.355524	4.361652	4.638748	4.644111	4.254311	4.129836
Baz1a	1.565687	3.434229	7.742364	1.58E-06	0.000139	5.468893	3.082606	4.339413	4.645925	2.602113	4.136256	4.407682	2.955303	3.501034	3.059216	3.385186	3.196995	3.454835	2.736815	2.733504
Psmb8	2.524773	2.373579	7.727541	1.62E-06	0.000141	5.507099	1.594228	4.24827	3.468922	1.065902	3.701411	3.830786	1.335758	2.709903	0.449965	2.819403	3.074378	2.838051	1.11438	1.386135
Ehd4	1.195441	5.413638	7.717253	1.64E-06	0.000142	5.266657	5.204201	6.287843	6.406934	4.952291	6.395166	5.987909	4.757474	5.22269	4.818719	5.128996	5.512044	5.465412	4.898279	4.792236
Msr1	7.382354	0.58939	7.698843	1.69E-06	0.000145	4.948584	-1.417928	5.637547	6.342196	-1.223791	5.323842	5.688428	-3.260591	0.377517	-1.813089	-0.806258	0.263333	2.064785	-2.232431	-2.228037
Icam1	2.226536	3.772434	7.680548	1.74E-06	0.000148	5.392325	3.180343	4.989118	4.751577	1.892408	4.6725	4.446528	2.841442	4.477275	2.882434	4.237532	3.990531	4.342647	3.098279	3.151754
Samsn1	3.828267	0.620853	7.676853	1.75E-06	0.000148	5.144927	-1.052261	2.411416	2.962413	-0.243853	3.300313	3.353184	-1.009457	0.645799	-0.773548	0.475109	0.676952	1.008331	-0.755361	-0.748265
Ptprc	2.523867	4.439581	7.669886	1.77E-06	0.000148	5.313777	3.334978	5.257997	5.858518	3.175097	5.937483	5.998002	2.703406	5.364251	2.707706	5.06243	4.822678	5.92643	2.736815	3.372991
B3gat2	-1.725413	4.894569	-7.660372	1.79E-06	0.000149	5.226421	5.360227	3.375827	3.336461	5.242285	3.464807	4.146066	5.271424	5.380434	5.42831	5.402519	5.255952	5.502977	5.997222	5.425691
Tgm2	1.970028	4.810499	7.645574	1.83E-06	0.000151	5.205855	4.414065	6.203417	6.198532	3.800721	6.133587	5.713147	4.390007	4.134313	4.355524	4.567674	3.760656	4.805872	4.306643	4.626888
Capn2	0.916235	7.36981	7.642012	1.84E-06	0.000151	5.120573	7.087198	8.047969	7.982194	7.277224	8.151612	8.209305	7.093715	7.028264	6.99268	7.101414	6.931901	7.168813	6.988669	7.126241
Pycard	2.483054	2.54739	7.640561	1.85E-06	0.000151	5.37735	1.644914	3.563961	3.370733	0.932396	3.780406	4.135132	1.576217	3.252427	1.819202	2.819403	2.958968	3.237047	1.493524	1.415936
S100a6	2.970239	3.182076	7.609509	1.94E-06	0.000156	5.269081	3.417469	5.45088	6.035406	1.910273	5.010247	5.804544	1.607516	2.976416	1.794428	2.795315	2.100543	2.772447	1.252133	1.889408
Hk3	5.743235	0.104392	7.609071	1.94E-06	0.000156	4.552584	-2.235785	3.184227	3.661186	-1.388744	3.882413	3.114281	-2.558588	-0.048075	-1.536754	0.348395	-0.318799	0.28321	-1.413173	-0.397871
Fam167a	3.711403	1.369795	7.605644	1.95E-06	0.000156	5.181824	-0.046857	2.904741	4.011832	-0.243853	3.359399	3.402651	0.202532	1.198329	0.736738	0.893396	0.805871	1.345607	0.879431	0.554014
Adamts1	2.709753	4.245518	7.588782	2.00E-06	0.000159	5.163972	4.055074	6.089878	7.008186	2.951788	5.827854	5.786023	3.318966	3.458111	3.197914	3.747244	3.981461	3.721386	2.984265	3.417866
Cd38	1.634093	4.123385	7.57427	2.05E-06	0.000162	5.125568	4.059751	5.40062	5.202167	3.41297	5.510649	5.295387	3.094359	4.112335	3.31576	3.432808	3.817832	4.183498	3.502026	3.489688
Cdt1	3.138968	1.54284	7.547546	2.13E-06	0.000168	5.216558	0.915118	3.532115	3.359399	0.531921	4.039207	4.227248	0.498056	1.198329	0.17081	0.699359	0.764165	1.252437	0.037347	1.122575
Ltbr	1.716824	5.630616	7.523836	2.21E-06	0.000172	4.970823	5.367791	6.650197	6.47678	4.228565	6.464835	6.406148	4.92212	5.724457	4.905145	5.8929	5.770848	6.067588	4.806206	5.18082
Ly86	2.538027	3.107	7.522127	2.22E-06	0.000172	5.190968	2.072763	4.379337	3.914512	1.548999	4.863759	4.090548	1.962529	3.818604	1.769222	3.649853	3.518775	4.056042	2.003164	2.087586
Fam129b	1.99606	6.18212	7.517357	2.24E-06	0.000172	4.940965	5.897038	7.257967	7.576944	4.700959	7.246896	7.052123	5.87853	5.698876	5.896824	6.013956	5.645806	6.096917	5.524682	6.086786
RT1-Da	3.515645	4.468184	7.49632	2.31E-06	0.000177	5.127806	3.035892	5.033013	5.762828	1.327578	5.850914	5.829628	2.361772	6.072029	1.769222	6.592911	6.421802	6.883722	2.913344	2.858134
Kcnn4	4.93213	1.827292	7.490947	2.33E-06	0.000177	4.999603	0.692264	3.934181	4.673644	-1.223791	4.641095	4.446528	-0.529223	2.753156	-0.504027	2.866406	2.729	3.843548	-1.047095	-0.505482
Vav1	2.371417	3.109521	7.480912	2.37E-06	0.000179	5.120816	2.529164	3.972652	4.242177	1.405223	4.64901	4.247696	1.888013	3.910622	1.60797	3.861885	3.656224	4.17506	1.493524	2.142093
Smagp	2.483351	1.058126	7.470757	2.41E-06	0.000181	5.064151	0.955837	2.713546	2.735741	-0.166914	2.81965	2.791131	-0.399109	0.76329	-0.386063	0.938085	1.199219	1.11804	0.434663	0.257494
Sp140	2.218767	3.080184	7.446734	2.50E-06	0.000186	5.079137	2.179767	4.03306	3.73759	1.300739	3.965054	3.901317	2.582602	4.009066	2.024877	3.796617	3.408041	4.028205	1.983515	2.402413
Arhgdib	1.911729	5.155109	7.437291	2.53E-06	0.000187	4.845026	4.927981	6.39353	6.488533	4.170451	6.94646	5.960298	3.945565	5.331332	4.351284	5.131403	5.012752	5.499602	4.018826	4.046557
Ece1	1.398002	5.908829	7.435833	2.54E-06	0.000187	4.809955	5.495845	6.548807	6.915941	5.054231	6.594826	6.610086	5.662696	5.697031	5.557236	5.519255	5.426066	6.086832	5.730244	5.852167
A3galt2	2.696074	3.161162	7.432629	2.55E-06	0.000187	5.043554	2.730329	4.432057	4.564365	1.245516	4.695613	4.755816	2.501617	2.939445	2.364852	2.572488	2.568656	3.600573	2.933966	2.570827
Carhsp1	1.513552	6.485652	7.428979	2.57E-06	0.000187	4.784741	6.052965	7.424208	7.232872	5.539224	7.518288	7.046298	5.891303	6.387803	5.859989	6.400738	6.344316	6.857597	5.898379	6.364048
Lmna	1.082807	6.065762	7.413553	2.63E-06	0.00019	4.761077	5.865327	6.544822	6.884163	5.529143	6.854181	6.824339	5.699474	5.761998	5.840457	5.827594	5.724028	6.020091	5.654608	5.914821
C3ar1	2.71107	2.617762	7.405461	2.67E-06	0.000192	5.011369	2.10932	3.953545	4.751577	1.478901	4.565043	4.529589	1.047044	2.80888	0.571595	2.40749	2.782205	3.542721	1.465469	1.009844
Clec12a	5.265745	1.137565	7.384528	2.75E-06	0.000197	4.534098	-0.518419	3.828023	3.118472	-2.040259	3.864394	3.536092	-1.73438	2.709903	-1.304935	2.758411	2.288794	3.277021	-1.218554	-0.621773
Pdlim7	1.059275	5.96987	7.361565	2.86E-06	0.000203	4.674047	5.895731	6.949847	6.752805	5.741122	6.976128	6.824906	5.694017	5.460906	5.72885	5.759192	5.457481	5.432163	5.312711	5.619042
Tuba1c	2.573698	2.776661	7.312405	3.09E-06	0.000218	4.878828	2.035255	3.724743	4.015447	0.932396	4.424547	3.775529	2.380012	2.121805	2.694377	2.499819	2.930971	2.300247	2.614401	2.697558
LOC100909675	2.584486	2.049631	7.305184	3.12E-06	0.000219	4.870509	1.459129	3.790827	4.217279	1.033666	3.654998	3.426763	1.091998	1.596873	1.393785	1.73055	1.959843	2.284645	0.313987	1.227132
Rasd2	-1.324968	6.592577	-7.297685	3.16E-06	0.000221	4.56513	7.04359	6.062272	5.567351	6.946476	5.835967	5.230091	7.03089	6.779911	6.883865	7.078516	6.958148	6.854298	7.092063	6.950948
Tspo	4.246758	2.580817	7.288353	3.21E-06	0.000223	4.816209	1.669604	4.936565	4.671355	-0.705589	4.720868	3.875271	1.63815	2.650138	1.579251	2.843096	2.455292	2.89053	1.574559	1.80183
Aspg	3.440328	3.240786	7.265565	3.33E-06	0.00023	4.809568	1.996747	4.585778	4.040502	0.280432	4.638447	4.639415	1.836107	4.866752	1.360416	4.546183	4.327666	4.983074	1.252133	2.325991
Pstpip1	2.926243	3.142419	7.259637	3.36E-06	0.000231	4.755171	2.742017	5.489533	5.450701	1.679737	4.748146	4.654809	2.324585	2.939445	2.47808	2.945148	2.568656	2.726993	1.150071	2.341603
Lgmn	1.643422	7.878759	7.251998	3.40E-06	0.000232	4.494988	7.143915	8.358081	8.663215	6.87777	9.065359	8.531479	6.97048	8.389028	7.086863	8.219604	7.91738	8.8457	6.956275	7.28505
Cxcl1	5.497775	-0.774833	7.241843	3.45E-06	0.000234	4.358708	-1.052261	3.719097	4.28174	-1.575022	3.470774	3.901317	-2.558588	-0.150641	-1.536754	-2.618976	-2.697527	-1.660823	-3.260591	-2.228037
Plce1	1.516617	4.143621	7.240797	3.46E-06	0.000234	4.598322	3.953171	5.3793	5.2148	3.387687	4.822358	5.263945	3.691803	3.991112	3.392049	3.734632	3.897149	3.931333	3.97969	3.468514
P2ry6	1.983025	3.496533	7.210167	3.63E-06	0.000244	4.66224	2.853968	4.397125	4.082466	2.045748	4.779733	4.336284	2.186319	4.171987	2.493553	4.056832	4.026255	4.506292	2.614401	2.570827
Il17ra	1.356669	5.669533	7.205016	3.66E-06	0.000245	4.450635	5.000326	6.19429	6.599873	4.775953	6.285188	5.879617	5.146469	5.959582	5.257563	5.905614	5.677044	6.089079	5.161347	5.474301
Rps27l	2.068207	2.476614	7.186626	3.77E-06	0.000251	4.682053	2.035255	3.49955	3.398685	1.097434	3.882413	3.53054	2.398025	2.24812	1.794428	2.484835	2.388236	2.064785	2.236876	1.931282
Pamr1	-1.774153	3.246865	-7.179268	3.82E-06	0.000252	4.587747	4.128153	2.439156	1.593793	3.622545	2.295481	2.18908	3.554005	3.653399	3.525993	3.643163	3.537463	4.056042	3.719553	3.874793
Kif18a	2.608456	0.202409	7.171235	3.87E-06	0.000254	4.522418	-0.410969	1.972946	2.356997	-0.166914	2.198134	2.257897	0.028455	-0.380636	-1.304935	0.134646	-0.231835	-0.070959	-0.755361	-1.040339
Anxa2	3.269442	4.41002	7.164295	3.91E-06	0.000255	4.500203	4.506048	6.104028	6.80439	2.124384	6.724717	6.421978	3.038038	4.318363	2.973531	3.973412	4.124292	4.901034	2.508394	3.334499
Ncald	-1.072859	8.708556	-7.134174	4.11E-06	0.000267	4.326813	9.07356	8.243285	8.207698	9.654852	8.389106	8.316029	8.744568	8.612702	8.788645	8.698488	8.810978	8.742231	8.936487	8.705026
Acsl6	-0.938119	7.544348	-7.12667	4.16E-06	0.000268	4.288269	8.191329	7.213482	7.209786	8.018184	6.92217	7.324946	7.710423	7.324946	7.707773	7.490308	7.383538	7.572522	7.748632	7.811496
Mthfd2	1.729452	2.656025	7.102622	4.32E-06	0.000278	4.528513	1.957182	3.486315	4.106393	2.029493	3.551819	3.637716	2.206902	2.364261	2.147322	2.745897	2.442128	2.475388	2.393975	1.910497
Rassf4	2.207588	4.352123	7.09573	4.37E-06	0.000279	4.395221	3.529616	4.941423	5.96611	3.019194	5.159937	5.753028	3.328389	4.889545	3.216682	4.613168	4.717344	5.121244	3.689548	3.079111
Vasp	1.219765	5.179978	7.089883	4.41E-06	0.00028	4.277063	4.878536	5.855224	5.96611	4.331596	5.92667	5.512311	4.781687	5.162468	4.812563	5.292808	5.142525	5.410762	4.646703	4.845219
Tax1bp3	1.848118	3.839632	7.081307	4.47E-06	0.000283	4.410871	3.481621	5.028455	4.878087	2.363612	4.553844	4.472821	3.428178	3.838823	3.307029	4.14048	3.853071	4.04219	3.186856	3.302949
Ccnf	1.435155	2.661363	7.075546	4.51E-06	0.000284	4.485373	2.054131	3.317238	3.365078	2.013053	3.378569	3.732651	2.550752	2.268138	2.539002	2.14695	2.388236	2.475388	2.760099	2.530531
Itgb2	2.464096	5.248687	7.065243	4.59E-06	0.000287	4.313018	3.948134	5.78805	6.362918	3.211576	6.146692	5.776673	4.171806	6.403725	3.889839	6.254907	5.946597	7.23981	4.013992	4.384671
Ftsj3	0.901269	5.94595	7.052008	4.69E-06	0.000292	4.170055	5.519806	6.474514	6.58109	5.696777	6.58798	6.394733	5.802806	5.83091	5.795909	5.875772	5.615344	5.793565	5.53653	5.763674
C1s	2.170369	4.033752	7.046426	4.73E-06	0.000293	4.352054	3.395435	5.030736	4.985204	2.427034	4.919359	5.245424	2.955303	4.584936	3.130231	4.4607	3.939921	4.997502	3.013624	3.503633
Lcp2	1.497359	4.330628	7.01227	5.00E-06	0.000308	4.211894	3.827152	5.048854	5.076287	3.742078	5.546404	5.426725	3.445603	4.76477	3.408468	4.369831	4.444493	5.072104	3.195422	3.35002
Hnrnpf	0.886156	5.360535	6.997525	5.12E-06	0.000313	4.09422	5.165666	5.851358	6.112538	5.240521	6.217601	6.170346	4.855242	5.186334	4.887683	5.084962	5.274637	5.290932	4.863765	4.885716
Mmp9	2.235412	2.531939	6.995639	5.14E-06	0.000313	4.357807	1.669604	4.099756	4.48836	2.169587	3.823012	4.149692	2.380012	0.818632	2.381582	2.048482	1.430105	2.100868	1.837926	2.387449
Tp53	0.990426	5.843836	6.956967	5.47E-06	0.000332	4.012793	5.568211	6.276355	6.648992	5.527697	6.659602	6.56123	5.331236	5.947219	5.438339	5.730896	5.778723	5.750202	5.128176	5.49539
Tacc3	1.850558	3.075052	6.940568	5.62E-06	0.00034	4.263024	2.196854	4.028502	3.463639	1.874319	4.169655	3.798811	2.501617	3.653399	2.524011	3.463704	3.220315	3.536147	2.168911	2.660694
Fkbp10	1.642501	4.439273	6.936137	5.66E-06	0.00034	4.086379	4.300089	5.637547	5.336845	3.218762	5.33206	5.195423	3.790562	4.411301	3.689436	4.502215	4.33124	4.659314	3.713602	4.111741
Lyn	1.577583	4.856956	6.91978	5.82E-06	0.000348	4.015281	4.692281	5.796117	5.901155	3.829173	5.847478	5.736243	3.999651	4.924651	3.842287	4.815347	5.071637	5.607003	3.949625	4.046557
Enpp3	2.72869	1.992546	6.906945	5.94E-06	0.000353	4.242165	1.895723	3.678943	4.579097	0.932396	4.241217	3.761378	1.576217	1.429209	0.317129	1.28803	1.167576	1.284167	0.921344	1.355706
Slc17a8	-2.41235	2.589664	-6.892741	6.08E-06	0.00036	4.195828	3.474632	0.369671	1.220561	3.425447	1.173177	1.496914	2.644268	3.449372	3.09008	2.966878	3.695584	2.8487	3.689548	3.069767
Rab7b	1.520448	4.010685	6.880303	6.21E-06	0.000366	4.064317	3.197423	4.610386	4.598509	3.002635	4.774918	4.446528	3.241276	4.58094	3.100223	4.441427	4.376922	4.908688	3.169571	3.808933
Trib1	2.213446	2.524823	6.874469	6.27E-06	0.000367	4.125908	2.144973	4.339413	5.140599	2.511387	4.151195	4.472821	1.754571	1.830204	0.736738	1.597576	1.959843	2.100868	1.747621	1.227132
Il4r	1.791961	5.492387	6.869379	6.32E-06	0.000369	3.899354	4.598957	6.712616	7.076834	4.833262	6.219374	6.021942	5.041519	5.214976	5.450283	5.282014	4.990317	5.359557	4.836642	5.293472
Ung	1.770805	2.175172	6.846837	6.56E-06	0.000381	4.131685	2.144973	3.631641	3.131857	1.379803	3.696327	3.519371	1.335758	1.830204	1.255413	2.108365	2.149958	2.170435	1.11438	1.386135
LOC100910973	1.59847	4.868317	6.839838	6.63E-06	0.000383	3.867037	4.873231	5.985571	6.316784	4.018141	6.167966	5.641552	3.837517	4.971187	3.907275	4.803319	4.918469	5.350218	3.671242	3.757487
Itgal	3.569885	3.545924	6.819938	6.86E-06	0.000394	4.091529	1.916502	4.585778	4.947854	1.21709	5.660437	5.114234	2.247205	4.438054	1.959523	3.962637	4.099353	5.312249	2.220182	2.17732
Glis2	1.570718	4.910641	6.813374	6.93E-06	0.000397	3.83905	4.388265	6.098602	6.120952	4.054744	5.643368	5.270622	4.764433	4.532109	4.526983	4.772804	4.632967	4.744468	4.43154	4.823147
RT1-Db1	3.210258	3.912035	6.801697	7.07E-06	0.000402	4.049163	2.730329	4.667202	5.253581	0.823477	4.925889	4.71479	2.033384	5.427919	1.326257	6.047485	6.042391	6.306496	2.348615	2.341603
Bak1	1.079882	4.696696	6.796962	7.12E-06	0.000404	3.794871	4.65258	5.777223	5.487608	4.328278	5.686309	5.308423	4.123906	4.511265	4.250151	4.398098	4.670138	4.502925	3.964736	4.156561
Gjd2	-2.085302	2.998974	-6.780471	7.32E-06	0.000413	3.970606	3.163058	1.833582	1.613009	4.210655	1.664612	1.519512	3.148563	3.492551	3.563133	3.28495	3.555912	3.405197	4.009141	3.769079
Mt1a	2.752047	2.499292	6.771009	7.44E-06	0.000418	3.984055	2.091157	4.616473	5.385148	1.856	3.939464	4.809529	1.000645	1.856886	1.579251	1.73055	1.697158	2.136071	1.347449	1.32462
Il18	1.397056	3.580393	6.750309	7.70E-06	0.00043	3.865118	3.154337	4.309667	4.514142	2.748327	4.237722	4.247696	2.880403	4.061624	3.006262	3.935342	3.537463	4.04219	2.562371	3.031778
Padi2	-1.521925	6.505692	-6.738271	7.85E-06	0.000435	3.636024	6.7565	5.419891	4.65754	6.788223	5.672137	5.278924	6.587582	7.32912	6.500222	7.359345	7.278214	7.764365	6.914731	6.79455
Chdh	1.693432	2.781177	6.738218	7.85E-06	0.000435	3.889835	2.694686	3.97739	4.245259	2.283631	3.873432	4.518413	1.809437	2.724465	1.393785	2.529327	2.288794	2.859272	1.902128	2.105984
Psd4	1.951955	2.650869	6.7224	8.06E-06	0.000444	3.928313	2.179767	3.953545	3.575619	1.300739	3.607042	3.450478	1.913281	3.40486	1.663747	2.758411	2.949696	3.153617	1.465469	2.030937
Tnf	2.908838	0.187974	6.705654	8.29E-06	0.000454	3.768075	-0.129637	1.972946	2.379614	-1.223791	2.014259	1.668504	-0.067151	-0.380636	-0.632503	-0.311823	-0.072212	-0.153565	-0.628995	-0.116166
Eva1b	1.837257	3.498858	6.702836	8.33E-06	0.000454	3.817495	3.171726	4.515857	4.39431	2.029493	4.380852	4.291029	2.629097	3.660989	2.905753	3.940842	3.586147	4.149447	2.437952	3.050898
Aprt	1.330253	4.679805	6.700471	8.36E-06	0.000454	3.666354	4.488808	5.40062	5.306181	3.567317	5.513542	5.132675	4.455977	4.757708	4.236421	4.706539	4.621334	4.73014	4.318455	4.346493
Fn3k	-1.005302	4.953969	-6.698543	8.39E-06	0.000454	3.588288	5.752845	4.782675	4.680492	5.815293	4.921539	4.74387	4.915882	4.568886	4.905145	4.747917	5.198397	4.856878	4.789329	4.728352
RGD1561778	6.063123	-2.435515	6.684174	8.59E-06	0.000463	2.575876	-2.659044	0.973336	1.951721	-3.260591	1.912714	1.378286	-3.260591	-2.550306	-3.260591	-2.618976	-2.697527	-1.925331	-2.232431	-3.260591
Anxa1	3.626042	2.943101	6.658041	8.98E-06	0.00048	3.842185	2.162475	4.32833	4.866124	0.43652	4.923716	5.154498	1.962529	3.798099	1.579251	2.819403	2.544222	3.895831	1.700256	1.355706
Hspb8	1.745126	5.089911	6.656851	8.99E-06	0.00048	3.54332	5.083068	6.279236	6.850152	4.155549	5.968389	6.296743	4.447353	4.67015	3.996864	4.830243	4.949035	5.151664	4.161181	4.472374
Serpine1	6.741417	1.572049	6.647809	9.13E-06	0.000486	3.796915	0.176305	5.751179	7.814392	-0.411255	6.035346	6.292649	0.202532	-0.150641	-0.504027	-0.420466	-0.411343	-0.541385	-0.305233	-0.116166
Dok3	2.034927	2.169493	6.638854	9.27E-06	0.000491	3.823541	0.785575	2.736085	2.877559	1.033666	3.106978	2.889836	1.962529	2.24812	1.426399	2.484835	2.66245	2.89053	1.521045	2.142093
Card9	1.416023	3.45415	6.631018	9.39E-06	0.000495	3.711769	2.810225	3.919485	3.789185	2.213416	4.031153	3.866483	3.01488	4.15058	2.962454	4.210463	3.872828	4.208518	2.535634	2.932182
Vim	3.361729	7.880758	6.614208	9.66E-06	0.000507	3.434503	7.305253	9.349311	9.354197	5.162543	9.842655	9.810919	6.51928	9.090766	6.364798	7.759442	7.609364	9.189132	6.424478	6.559448
Tsc22d3	-0.916235	5.460629	-6.606908	9.78E-06	0.000511	3.412745	6.149375	5.212508	5.312072	6.32348	5.434839	5.326158	5.5803	4.927801	5.41415	5.140992	5.313107	5.198209	5.75073	5.401689
Cyth4	1.716961	5.173438	6.599448	9.90E-06	0.000515	3.483565	4.59574	5.80147	5.601424	3.595195	6.124153	5.636392	4.579502	5.89185	4.453722	5.741966	5.570679	6.090202	4.080242	4.716427
Clec4a2	5.372217	-2.477144	6.567939	1.04E-05	0.000539	2.836927	-2.659044	1.607774	2.456093	-2.344749	2.198134	1.839907	-3.260591	-1.196868	-3.260591	-3.260591	-2.697527	-3.260591	-3.260591	-3.260591
Vwf	1.842703	6.821266	6.567598	1.05E-05	0.000539	3.339848	6.531696	8.755643	7.578167	5.894839	7.926171	7.942632	6.708889	6.215528	6.609	6.381653	5.905225	6.265356	6.087425	6.711375
Clcf1	5.944669	2.618804	6.559359	1.06E-05	0.000541	3.571076	0.428583	4.970229	5.536232	-1.788967	4.698159	4.487224	2.979429	3.301815	2.00342	2.681644	2.481265	2.619163	1.001683	2.030937
Lix1	-1.181026	6.054367	-6.558387	1.06E-05	0.000541	3.323678	6.92747	5.694763	5.27937	6.309244	5.513542	5.273948	6.369857	5.850958	6.249911	6.248268	6.183549	6.091323	6.508512	6.28551
Dusp15	-1.25412	5.68659	-6.557837	1.06E-05	0.000541	3.335649	6.090021	5.138299	4.579097	6.294015	5.178286	4.883261	5.77033	6.000542	5.955267	6.063962	5.62557	6.066448	5.772362	6.227874
Casp1	2.124453	2.737082	6.549736	1.08E-05	0.000544	3.636409	2.179767	3.939046	3.484656	1.405223	4.424547	3.653128	2.12275	2.822481	2.147322	2.90068	2.782205	2.999674	1.700256	2.030937
RGD1565374	6.593984	-1.493449	6.5497	1.08E-05	0.000544	3.081811	-2.235785	3.53854	4.31439	-2.040259	4.368121	3.136474	-3.260591	-3.260591	-2.604521	-2.176425	-2.697527	-2.668207	-2.656794	-2.653844
Sdc4	1.112536	6.32255	6.546046	1.08E-05	0.000545	3.304357	6.000408	6.831203	7.122387	5.533472	6.616519	6.934074	5.97168	6.433944	5.595383	6.508381	6.442607	6.660895	5.921797	5.96356
Fzd9	-1.304275	4.031686	-6.536532	1.10E-05	0.000551	3.424214	4.614937	3.27168	2.795835	4.331596	3.563034	3.099357	4.629911	4.032662	4.268256	4.437541	4.216089	4.308338	4.357143	4.623699
Fhl3	2.670786	1.778677	6.513298	1.15E-05	0.000569	3.613183	0.995538	3.01022	3.234684	-0.166914	3.099296	2.526873	1.21902	2.307359	1.061401	2.39151	1.740817	2.268872	0.598632	1.193114
Brinp2	-0.779521	7.249714	-6.509697	1.15E-05	0.000569	3.230558	7.181956	6.304905	6.595049	7.248984	6.443755	6.400832	7.92904	7.63974	7.695281	7.554056	7.445869	7.572522	7.743895	7.751404
Gcnt2	1.237818	3.60624	6.508447	1.15E-05	0.000569	3.415319	3.474632	4.435504	4.787963	3.145235	4.430682	4.467019	3.060831	3.159031	3.367065	3.352542	3.288097	3.70975	2.994118	2.952659
Rbms1	1.001495	3.773574	6.507808	1.16E-05	0.000569	3.35503	4.007445	4.820078	4.992859	3.737082	4.6725	4.967859	3.200804	3.440579	3.120298	3.302147	3.082885	3.46871	2.974345	3.169358
Cmklr1	2.360366	3.729908	6.492867	1.19E-05	0.000582	3.446373	3.32724	4.738705	4.742881	2.061822	5.120628	5.423733	2.12275	4.213873	2.204856	4.332655	4.460902	4.877826	3.003904	1.755962
Mcl1	0.938298	5.131416	6.48927	1.19E-05	0.000583	3.232715	5.017259	5.890816	6.196144	5.095807	5.863442	6.022931	4.559635	4.866752	4.372361	4.687033	4.884809	5.058407	4.757867	4.61409
Tgfb1	1.830561	4.898391	6.473895	1.22E-05	0.000596	3.29821	4.201945	5.337533	5.418297	3.309066	5.927755	5.559419	3.889373	5.798587	3.866259	5.418377	5.703489	6.308426	3.725479	4.178459
Ntm	-0.987781	6.168275	-6.466001	1.24E-05	0.000601	3.163264	6.494176	5.492845	5.601424	6.432581	5.245957	5.564861	6.144342	6.407114	6.399094	6.348928	6.41508	6.575318	6.637481	6.619913
Gpr183	2.091067	1.784169	6.44734	1.28E-05	0.000618	3.512576	1.212615	2.904741	3.131857	0.621404	2.910256	2.809587	-1.009457	3.180315	-0.27702	3.223093	3.031075	3.594258	0.182287	0.257494
Cnnm2	-0.772309	5.362434	-6.443633	1.29E-05	0.00062	3.147441	5.627699	4.670131	4.915329	5.68515	4.993765	4.957551	5.528095	5.539186	5.512366	5.437958	5.459115	5.578496	5.599199	5.609402
Tgif1	1.820464	2.364032	6.435615	1.31E-05	0.000626	3.47532	1.541696	3.176008	3.404211	1.300739	3.397487	2.82781	1.511505	2.926907	1.579251	3.00938	2.568656	3.237047	1.493524	1.473753

Lap3	1.082722	6.98843	6.432726	1.31E-05	0.000626	3.099032	6.686396	7.894245	7.417363	6.571681	7.949059	7.579378	6.413678	7.155287	6.429581	7.028375	6.698588	7.206042	6.339116	6.482322
Cd68	3.83123	4.219117	6.430711	1.32E-05	0.000626	3.411679	2.387517	4.916971	5.823889	1.526009	6.335105	5.848717	1.782265	6.671357	1.937064	6.029528	5.989079	7.236772	0.879431	1.931282
Sphk1	3.681852	0.817145	6.427333	1.33E-05	0.000627	3.421506	0.592497	3.33211	3.500221	-1.223791	2.927714	2.791131	0.282279	1.112357	-0.175642	0.20947	0.535373	-0.153565	-1.047095	0.119416
Ecel1	3.423887	2.283913	6.410036	1.37E-05	0.000644	3.449297	2.372556	4.175923	4.143209	-0.502853	4.059146	4.355253	1.63815	1.356279	1.60797	2.529327	1.533025	1.568131	1.493524	2.142093
Tlr1	3.187387	0.791462	6.39639	1.40E-05	0.000656	3.347414	-0.410969	3.019437	2.508341	-0.600663	2.575715	2.04074	0.357848	1.067371	0.007959	-0.116374	0.764165	1.045835	-0.211665	0.190106
Acap1	3.983792	-0.315048	6.389149	1.42E-05	0.000662	2.821556	-1.908947	1.747452	1.061799	-2.344749	1.390206	0.915838	-0.169545	0.301855	-0.929891	0.134646	-0.149817	-0.657145	-0.512812	-0.621773
S100a11	4.022641	0.463567	6.385917	1.42E-05	0.000663	3.289879	-0.518419	2.36878	2.837276	-1.075778	3.680967	3.099357	-1.009457	0.705741	-0.632503	-0.311823	-0.149817	0.28321	-0.040977	-1.040339
Hbegf	2.52126	3.593475	6.375877	1.45E-05	0.000672	3.223349	2.718545	4.610386	6.169622	3.019194	5.181928	5.541588	2.582602	3.047633	3.059216	3.456042	2.66245	3.037402	2.639729	2.745289
Cd300le	4.982889	1.206402	6.368704	1.47E-05	0.000676	3.362902	0.105685	3.713429	4.854061	-0.243853	5.413312	5.106791	-1.21312	0.301855	-0.929891	0.280604	0.764165	1.186803	-0.305233	-0.748265
Fam105a	1.751454	3.705731	6.367748	1.47E-05	0.000676	3.256818	2.263247	3.929299	4.576652	2.77782	4.234219	4.313834	3.445603	4.411301	3.244385	4.268484	3.867914	4.426789	2.838755	3.417866
Pate4	6.138532	-3.169149	6.364708	1.48E-05	0.000677	2.204282	-3.260591	0.934883	0.049949	-3.260591	1.230571	1.402804	-2.088409	-3.260591	-3.260591	-3.260591	-2.697527	-3.260591	-3.260591	-3.260591
Bag3	1.279216	5.16889	6.360489	1.49E-05	0.000677	3.02049	4.810783	5.70765	6.29017	4.738944	6.14296	6.058089	4.84543	5.167806	4.12658	5.011064	4.845355	5.296777	4.680656	4.689229
Cd53	1.738021	4.822347	6.359922	1.49E-05	0.000677	3.07387	4.092076	5.238395	6.012977	3.950685	6.023216	5.722916	3.624549	5.331332	3.416608	5.190317	5.238925	6.017734	3.707626	4.012827
Mafb	2.262964	3.539618	6.352451	1.51E-05	0.000683	3.227813	2.488111	4.216563	5.286867	2.499634	4.829341	4.597545	2.67414	3.94833	2.430637	3.409193	3.901965	4.526331	2.494578	2.417223
Arl11	2.597634	2.946448	6.349362	1.52E-05	0.000685	3.297002	2.542593	3.9244	4.459457	1.000693	4.794082	3.96856	1.697535	3.825375	1.458292	3.48645	3.695584	4.153748	1.252133	1.29285
Lilrb4	8.375313	1.330993	6.342431	1.53E-05	0.00069	2.99109	-1.417928	6.143555	6.271396	-2.040259	6.602999	5.656923	-1.21312	2.901501	-1.813089	1.104165	1.319276	3.361247	-3.260591	-1.040339
Ube2l6	1.822918	2.594726	6.335148	1.55E-05	0.000696	3.250892	2.529164	4.129856	3.647273	1.593908	3.873432	3.727807	1.17791	2.68033	1.769222	2.945148	2.401899	2.980434	1.252133	1.931282
Cldn10	-1.5815	5.309146	-6.330836	1.56E-05	0.000699	2.957476	6.172233	4.938996	3.684081	5.72859	4.513947	4.401612	5.528095	5.557602	5.52183	5.852614	5.402472	5.864809	5.528077	5.678646
Sh3bp2	1.403612	4.530208	6.319294	1.60E-05	0.00071	3.000356	4.373312	5.010077	5.684382	3.781437	5.513542	5.642839	3.830902	4.7399	3.627666	4.613168	4.678581	4.997502	3.302384	3.704139
Il2rg	2.416709	2.039163	6.284011	1.70E-05	0.000752	3.217761	1.401336	3.667262	3.489863	1.379803	4.105904	3.830786	1.443753	2.345542	1.142163	1.780488	0.631286	1.691577	0.598632	1.529341
Hmha1	1.377122	4.844916	6.277997	1.71E-05	0.000757	2.953596	4.105711	5.146735	5.292838	3.538889	5.399302	4.891948	4.156015	5.845972	4.070936	5.737232	5.4978	5.980693	3.817137	4.410806
Myo1e	1.126363	5.404395	6.276193	1.72E-05	0.000757	2.862125	5.137181	5.779937	6.154947	4.783243	6.034339	6.352089	4.798736	5.589771	4.707008	5.34974	5.373295	5.810006	4.877138	4.953324
Zyx	0.965636	6.775493	6.256565	1.78E-05	0.000778	2.787368	6.675771	7.560686	7.857074	6.448715	7.505266	7.186614	6.351488	6.584221	6.398067	6.442051	6.657889	6.526407	6.307856	6.36977
Parp3	1.677643	3.734018	6.255677	1.78E-05	0.000778	2.941447	4.243653	4.934131	5.132299	2.853641	5.308931	5.368786	2.774074	3.282262	2.973531	3.335938	3.609886	3.277021	2.701169	2.622863
Gsap	2.57972	1.349896	6.253545	1.79E-05	0.000778	3.188837	0.785575	2.466372	2.498042	-0.243853	3.060255	2.907078	0.798508	1.747064	0.17081	1.481601	1.199219	2.082939	0.598632	0.119416
Map6d1	-1.271096	5.402757	-6.237665	1.84E-05	0.000797	2.786898	5.744154	4.275453	4.440757	5.99241	4.843207	4.836779	5.60183	5.800307	5.6132	5.312036	5.665763	5.800438	6.114777	5.638132
Itgam	2.824757	5.704161	6.230998	1.86E-05	0.000803	2.833253	4.865238	6.922471	7.15389	3.41297	6.9189	6.738447	5.105949	6.325884	3.682701	5.986294	5.921916	7.223536	4.529643	5.114213
Fes	2.595012	3.698085	6.226095	1.87E-05	0.000807	3.060007	2.311111	4.909553	4.802683	1.720809	4.651639	3.926902	2.644268	4.792678	2.973531	4.794232	4.204441	4.838295	2.097585	3.270693
Cd3eap	0.94554	4.088686	6.224218	1.88E-05	0.000807	2.838254	4.251852	4.879497	5.211652	3.911307	4.868287	5.103056	3.30948	4.067347	3.298245	3.772142	4.030659	3.900956	3.387081	3.35002
Slc25a24	1.412737	2.940415	6.214098	1.91E-05	0.000818	2.997066	2.620642	3.582736	4.033387	2.401998	4.047215	3.935331	2.880403	2.268138	2.734001	2.572488	2.303426	2.980434	2.522078	2.503027
Mcm3	3.515903	2.648049	6.200976	1.96E-05	0.000834	3.077008	1.853246	5.088807	3.793402	0.785264	5.180108	5.012356	2.078759	2.364261	1.520039	2.514649	1.883989	2.8487	1.252133	1.260365
Tnfsf9	2.308747	0.876369	6.199075	1.96E-05	0.000834	3.070325	-0.310971	2.397344	1.792283	-0.02435	2.198134	1.839907	0.118118	0.449407	0.449965	1.14286	0.885831	1.045835	0.745852	0.499386
Camkk2	-1.217009	5.484782	-6.188599	2.00E-05	0.000846	2.698876	5.801132	4.57644	4.770955	5.89596	4.516833	4.667512	5.701288	5.877262	5.759573	5.537522	6.025942	5.875263	6.315735	5.504087
Ripk1	1.206816	4.841445	6.184419	2.02E-05	0.000847	2.753135	4.391979	5.624015	5.569811	3.997398	5.124418	5.250499	4.778253	4.600808	4.781385	4.979343	4.609607	4.97094	4.442376	4.716427
Slc39a12	1.954274	2.173656	6.183161	2.02E-05	0.000847	3.044135	1.916502	3.382986	3.746318	1.033666	3.036313	3.295428	1.888013	2.24812	1.489496	1.652245	2.133674	1.568131	1.747621	1.684306
Dlgap5	2.96752	1.156164	6.181962	2.02E-05	0.000847	3.022936	-0.129637	2.21681	2.053692	-0.705589	2.847434	2.458346	0.498056	1.628188	0.736738	1.419918	0.721218	1.805289	0.546019	0.970221
Hspa2	1.179333	5.542326	6.17904	2.03E-05	0.000849	2.675394	5.180775	5.994946	6.71662	5.317849	6.334287	6.663062	4.812232	5.551489	5.045607	5.192625	5.240827	5.565643	5.006894	5.005709
Dscam	-0.873821	5.309317	-6.172241	2.06E-05	0.000856	2.663088	5.615043	4.948678	5.086634	5.979775	4.815341	4.854663	5.312364	5.350235	5.2845	5.264574	5.322117	5.460213	5.600812	5.47607
Gabrd	-1.581029	4.72051	-6.169438	2.07E-05	0.000856	2.756489	4.630741	3.176008	3.642605	5.126698	3.365818	3.030221	5.41323	5.354922	5.444323	5.756075	4.994832	5.533	5.00202	5.69393
Gpr61	-0.812965	4.273561	-6.168529	2.07E-05	0.000856	2.758428	4.358202	3.735969	3.693138	4.488478	3.482634	3.55809	4.633716	4.540363	4.697018	4.626882	4.543347	4.440934	4.595845	4.521176
Trem1	6.662972	-2.503974	6.161473	2.10E-05	0.000863	1.991747	-3.260591	1.248835	1.809043	-3.260591	1.643636	0.809818	-3.260591	-2.076466	-3.260591	-3.260591	-1.978322	-0.541385	-3.260591	-2.653844
Ch25h	2.490005	2.284632	6.154467	2.12E-05	0.000868	3.00525	1.80948	4.014741	3.370733	0.74601	3.568609	3.862069	1.576217	3.047633	1.426399	2.655119	2.166061	3.028062	0.598632	0.554014
Mfap3l	-1.217428	6.089917	-6.152801	2.13E-05	0.000868	2.603211	7.267337	5.814767	5.747694	6.634827	5.638075	5.739856	5.802806	6.057725	5.778645	6.052575	6.064036	6.498783	6.345864	5.839853
Mob3a	1.3531	4.450443	6.152687	2.13E-05	0.000868	2.73495	4.235407	5.230479	5.147205	3.355447	5.328778	4.80033	4.005537	4.379449	4.06577	4.456866	4.366508	4.76429	4.134703	4.111741
Cox6a2	2.527728	0.330659	6.146392	2.15E-05	0.000874	3.017068	1.071521	3.142653	2.954902	-0.243853	2.927714	2.28453	-0.672244	-0.992527	-0.386063	-0.806258	-0.318799	-1.243945	-0.512812	-0.748265
Grik4	-1.229643	6.958963	-6.142486	2.17E-05	0.000875	2.584466	6.834315	5.204449	5.932087	7.140591	6.20781	5.682182	7.763926	7.476198	7.691512	7.391335	7.396392	7.387712	7.713653	7.618747
Plk5	-2.187013	5.155971	-6.141963	2.17E-05	0.000875	2.756909	4.563167	2.493084	2.508341	5.060244	2.987204	2.592292	6.894799	6.574206	6.783581	6.857868	6.003714	6.326634	5.8105	6.822634
Cyp26b1	-1.890068	4.256855	-6.121177	2.25E-05	0.000904	2.642018	4.891713	3.929299	2.932131	5.824744	3.152236	3.980827	4.297077	3.997121	4.170562	4.4607	4.33124	4.616334	4.817348	4.287263
Osmr	3.770191	2.150448	6.115344	2.27E-05	0.000909	2.954963	0.995438	4.931692	5.379549	0.823477	3.832311	4.313834	1.000645	1.934092	1.255413	1.322149	0.721218	2.153355	1.040235	0.929479
FAM187A	5.247602	1.304551	6.114364	2.28E-05	0.000909	2.924281	-0.760787	4.856537	5.500563	-0.166914	3.8553	4.307355	-0.831021	2.007376	-1.536754	1.180545	1.199219	2.118577	-0.893866	-1.040339
Bcl2a1	2.723343	2.054108	6.111503	2.29E-05	0.000911	2.932304	1.459129	3.439014	3.642605	1.033666	4.402865	4.164107	1.000645	2.345542	0.317129	1.704918	1.996325	2.880186	-0.123798	1.048408
Sipa1	1.368396	5.743195	6.104115	2.32E-05	0.000919	2.568798	5.06457	6.195307	5.96891	4.348075	6.222914	5.868657	5.611511	6.169707	5.436339	6.406713	5.960512	6.537146	5.066477	5.580088
Phkb	-0.70438	5.805726	-6.099574	2.34E-05	0.000924	2.520276	5.99309	5.445761	5.255111	6.075905	5.364471	5.265617	6.187702	5.93475	6.101288	5.695542	5.867564	5.885643	6.174487	6.062262
Igsf6	3.746599	1.55712	6.093457	2.36E-05	0.000927	2.911123	-0.310971	3.317238	3.855228	-0.02435	3.990197	2.665044	1.335758	1.356279	1.326257	1.419918	1.456531	1.11804	0.649393	0.383504
Parp9	2.107014	4.358648	6.092213	2.37E-05	0.000927	2.695986	3.664706	5.605773	5.288362	2.62393	5.020453	4.957551	3.914618	4.886311	3.440756	4.924873	4.414477	5.421502	3.125428	3.825682
Timd2	5.103251	-1.405413	6.091861	2.37E-05	0.000927	1.84522	-3.260591	-0.103798	-0.007587	-3.260591	-0.107758	-0.1418	-3.260591	1.658838	-2.155202	0.799637	1.507975	1.738143	-2.656794	-1.899685
Mapkapk3	1.398348	4.541688	6.084799	2.40E-05	0.000936	2.573247	4.64948	5.445761	5.662646	3.737082	5.726763	5.45191	4.068749	3.852146	3.836231	4.464524	4.411103	4.619447	3.789115	3.942904
Rab32	3.768952	1.478537	6.076948	2.43E-05	0.000943	2.883219	0.485316	3.1924	3.910635	-0.81875	3.245679	3.301961	0.282279	2.287882	-0.175642	1.704918	2.274011	3.292705	-0.755361	-0.505482
LOC303566	4.721814	-0.838319	6.076554	2.43E-05	0.000943	2.062797	-2.659044	1.047298	0.52603	-2.731185	1.72577	0.915838	-1.450335	-0.654381	-0.929891	-1.136114	-0.318799	-0.24119	-1.047095	-0.748265
Sp110	1.483428	3.911068	6.072725	2.45E-05	0.000947	2.649793	3.730722	4.616473	4.766671	2.862845	4.735811	4.883261	3.094359	4.360928	2.894141	4.281549	4.017407	4.832942	2.805569	2.992761
Tmem151b	-1.011419	5.202145	-6.04867	2.56E-05	0.000984	2.465703	5.70438	4.528825	4.254466	5.158812	4.285899	4.616013	5.761661	5.280499	5.589995	5.408486	5.28389	5.594403	5.752183	5.656972
Pla1a	2.485859	2.858162	6.044701	2.57E-05	0.000988	2.761877	2.460078	4.598135	4.78373	1.273392	3.900209	3.875271	3.116285	2.471744	2.364852	2.423294	2.014226	2.419337	2.11575	2.460763
Wdhd1	1.662954	3.263995	6.022477	2.68E-05	0.001024	2.639244	2.682606	4.771807	3.967729	2.602113	4.082714	4.377073	3.337752	2.964197	2.639794	3.214035	2.707156	2.838051	2.601568	3.088394
Osbp2	-0.819102	7.070922	-6.020773	2.68E-05	0.001024	2.362384	7.150508	6.338435	6.489833	7.540383	6.548324	6.727569	7.404465	7.32912	7.251199	7.22716	7.205093	7.227622	7.294002	7.27136
Tmem106a	2.581802	1.120969	6.017296	2.70E-05	0.001027	2.796899	0.785575	2.439156	2.654608	-0.502853	2.496038	2.734303	0.563361	1.356279	-0.504027	1.253084	1.03357	2.064785	-0.123798	0.383504
Phactr3	-0.80229	7.619083	-6.008735	2.74E-05	0.001039	2.351271	8.228415	7.414634	7.083307	8.123641	7.484404	7.513006	7.745139	7.485311	7.606896	7.673263	7.513113	7.520188	7.66244	7.621547
Socs3	5.839102	1.495296	6.003498	2.77E-05	0.001043	2.706245	-0.410969	5.186149	5.485003	-1.223791	4.587184	3.835297	-0.831021	2.077116	-0.504027	1.21727	0.998029	1.950783	0.111637	-0.297732
Fos	1.971885	3.781749	6.003095	2.77E-05	0.001043	2.547341	3.136736	5.17794	5.536232	3.002635	4.970791	4.422747	3.105363	3.878428	2.95129	3.696121	3.250834	4.105722	3.32598	2.516844
Ahcyl2	-1.149476	7.164538	-5.9965	2.80E-05	0.001052	2.321455	7.492908	5.982039	6.753887	7.798075	6.55536	6.689462	7.434228	7.299641	7.449187	7.411284	7.614873	7.385429	7.280982	7.167886
PVR	2.306467	2.453291	5.991701	2.83E-05	0.001058	2.695416	2.01613	3.838476	4.4568	1.245516	3.926497	3.288866	1.913281	2.054242	1.520039	2.256873	1.978199	2.619163	2.041677	1.529341
Tbxas1	2.584448	2.788719	5.985658	2.86E-05	0.001066	2.719978	1.787089	3.52566	3.599901	0.621404	4.143745	3.563537	2.100922	3.749088	1.636128	3.327565	3.149191	4.204378	1.943396	1.991897
Cldn1	-2.731404	2.352717	-5.973099	2.92E-05	0.001086	2.628948	4.017097	0.24948	1.269823	3.504019	1.877213	0.809818	2.033384	2.099634	2.707706	2.966878	3.755344	2.568523	2.269694	3.220915
Aif1	1.340401	4.877326	5.966797	2.95E-05	0.001095	2.374095	4.3544	5.591938	5.219509	3.761892	5.570221	5.163494	4.489964	5.648222	4.146293	5.478234	5.200353	5.595984	3.794763	4.327019
Ano6	1.068325	5.995703	5.951167	3.04E-05	0.001119	2.253667	5.369676	6.36127	6.949398	5.695489	6.365048	6.729994	6.011712	5.880517	5.974693	5.779288	5.576715	5.796318	5.691408	5.783814
Gprc5b	-1.005724	7.548717	-5.94963	3.04E-05	0.001119	2.239015	8.096921	6.998435	6.882184	7.732748	6.89691	6.864613	7.573487	7.753274	7.595737	7.851754	7.517434	8.081789	7.841266	8.004364
Des	1.984168	3.710505	5.949609	3.04E-05	0.001119	2.491758	3.334978	5.066748	4.581538	2.169587	4.743225	4.401612	3.496648	3.542716	3.400282	3.957219	3.387587	3.732929	2.639729	3.629792
Birc3	2.697108	1.089525	5.946174	3.06E-05	0.001121	2.677874	0.243629	3.151065	2.778919	-0.093872	2.436306	2.336365	0.202532	2.007376	0.245823	0.799637	1.290185	1.11804	-0.628995	0.321874
Lurap1	-1.018332	3.018963	-5.944991	3.07E-05	0.001121	2.453993	3.859704	2.570375	2.916749	3.633342	2.507694	2.907078	2.880403	3.126505	2.928701	2.911926	2.853541	3.153617	3.107385	3.106782
Tln1	1.073951	7.078236	5.942925	3.08E-05	0.001122	2.223936	6.692433	7.700841	7.452124	6.123585	7.439851	7.326148	6.881257	7.304495	6.770144	7.128781	7.070951	7.591668	6.671649	6.953491
Sec23b	1.014758	5.148502	5.934422	3.13E-05	0.001135	2.255737	5.152589	5.888303	6.012977	4.541025	5.862307	5.647977	4.96806	4.696241	4.887683	4.971302	4.974403	4.933912	4.652936	4.935431
Arhgap17	0.897789	5.111831	5.916188	3.23E-05	0.001169	2.233222	4.904771	5.398856	5.659183	4.494412	5.693981	5.605038	4.828927	5.245588	4.707008	5.372305	5.210093	5.344586	4.43154	4.716427
Ifi47	2.164511	2.712866	5.905206	3.29E-05	0.001189	2.527004	2.357438	3.97739	4.19832	1.245516	3.956574	3.485336	1.408645	3.413873	1.690848	3.223093	2.958968	3.926315	1.040235	1.415936
Lrrc2	1.129774	2.584136	5.89467	3.36E-05	0.001206	2.418974	2.853968	3.613496	3.623781	2.475838	3.952316	3.909896	2.206902	1.958936	2.024877	1.678822	1.959843	2.118577	1.881043	2.211707
Klhl26	-0.888337	6.111466	-5.893816	3.36E-05	0.001206	2.141419	6.435999	5.10405	5.572268	6.270002	5.626095	5.549845	6.321129	6.455927	6.3669	6.320727	6.512756	6.281167	6.512802	6.25492
Tppp	-1.125027	5.900507	-5.886496	3.41E-05	0.001218	2.130832	6.295338	5.188194	5.506998	6.513701	5.014338	5.408679	5.967162	6.133337	5.969169	5.794558	6.055417	6.191982	6.573205	6.022862
Dynlt1	0.86727	4.142486	5.872852	3.49E-05	0.001244	2.184481	4.428604	5.010077	5.083193	4.232121	5.242473	5.429711	3.36548	3.492551	3.548392	3.796617	3.644778	3.805789	3.508915	3.503633
Slfn13	2.004918	4.848552	5.866877	3.53E-05	0.001254	2.302023	3.045357	5.060063	4.718692	3.091442	5.416407	5.070904	4.150713	6.825372	3.613573	6.80456	5.948925	6.820209	3.294432	4.098018
Plek	1.608335	4.167235	5.859716	3.57E-05	0.001266	2.240863	3.901994	4.892097	4.678213	3.075697	5.481392	5.238629	3.038038	4.949657	2.984524	4.524366	4.618412	5.1581	2.954296	2.952659
Mcm7	0.980924	4.755529	5.85739	3.59E-05	0.001268	2.150846	4.643261	5.527177	5.098612	4.102146	5.410211	5.34526	4.502506	4.761243	4.355524	4.763521	4.49954	4.695162	4.398536	4.534822
Dok2	1.997185	1.65421	5.854494	3.61E-05	0.00127	2.519979	0.915118	2.232748	3.013923	0.705659	2.838232	2.863579	1.809437	1.067371	1.326257	1.180545	1.629088	1.715048	1.150071	1.260365
Mcm5	4.302384	1.461068	5.841505	3.69E-05	0.001293	2.506606	0.643243	4.466163	3.359399	-0.243853	4.733331	4.978093	-0.067151	0.377517	-0.27702	0.646479	0.202178	1.345607	0.434663	0.799858
Scamp2	1.313886	5.172414	5.841219	3.69E-05	0.001293	2.132763	4.582799	5.78805	5.522324	3.815017	5.595018	5.088014	4.946803	5.452183	4.893527	5.555561	5.427736	5.852956	4.879797	5.060981
Ephb2	-1.224783	4.160197	-5.837515	3.72E-05	0.001297	2.195482	4.137033	2.747223	3.063656	4.560953	3.416161	3.301961	4.39898	4.806432	4.600281	4.609719	4.404331	4.888187	4.605518	4.803553
Clec4d	5.730338	-3.301559	5.836316	3.72E-05	0.001297	1.574313	-3.260591	-0.186163	1.514261	-3.260591	0.422095	0.117189	-3.260591	-3.260591	-3.260591	-3.260591	-2.697527	-3.260591	-3.260591	-2.228037
Kcnj9	-0.765166	6.375147	-5.83356	3.74E-05	0.0013	2.028037	6.257157	5.58264	5.671838	6.560411	5.546404	5.777845	6.565836	6.809239	6.776483	6.873771	6.607926	6.734519	6.725872	6.782447
Cdc42ep1	0.976603	5.762661	5.823551	3.81E-05	0.001319	2.023694	5.684832	6.320836	6.464931	5.224545	6.589352	6.330684	5.249269	5.630934	5.187937	5.573377	5.505731	5.894664	5.415271	5.634968
RGD1559482	7.000137	-2.732068	5.817937	3.85E-05	0.001329	1.593591	-3.260591	1.725091	1.631973	-3.260591	2.692656	0.846031	-3.260591	-3.260591	-3.260591	-3.260591	-2.697527	-1.437379	-3.260591	-2.653844
St8sia5	-0.939431	4.311577	-5.809382	3.91E-05	0.001346	2.112213	4.485336	3.302211	3.642605	4.470529	3.384903	3.817169	4.481542	4.778791	4.659784	4.549787	4.606661	4.653252	4.82012	4.795073
Fbl	0.958254	5.021257	5.805064	3.94E-05	0.001352	2.010462	5.048187	5.662767	5.916704	4.969434	5.984119	6.291829	4.455977	4.576933	4.632088	4.606261	4.842853	4.562362	4.533027	4.266966
Exo5	1.315408	2.842323	5.800951	3.97E-05	0.001358	2.275867	2.620642	3.49955	3.871277	2.255954	3.96082	3.57437	2.01015	2.695192	2.568526	2.758411	3.108108	3.018661	1.963595	2.124152
Nrm	2.202946	2.36413	5.798131	3.99E-05	0.00136	2.386467	1.895723	3.411272	3.077557	0.74601	4.018988	3.321383	2.450751	2.186344	1.937064	2.256873	1.978199	2.118577	1.724133	2.325991
Tnip2	1.501519	3.859414	5.797114	3.99E-05	0.00136	2.178706	3.188908	5.051103	4.705324	3.002635	4.335792	4.261168	3.537848	3.910622	3.563133	4.11072	3.706635	4.114573	3.013624	3.655003
Col4a1	1.199558	7.614954	5.790183	4.04E-05	0.001373	1.944707	6.801179	7.974932	7.652601	6.411567	7.943706	7.64476	8.293794	7.556221	8.03568	7.804263	7.232591	7.670317	7.541673	8.054664
Dse	1.472251	3.784519	5.784045	4.09E-05	0.001382	2.149414	3.713016	4.343088	4.634216	2.57996	4.457973	4.881081	3.328389	3.756192	3.271566	3.802672	3.949256	4.083352	3.003904	3.302949
Lfng	1.469105	5.157011	5.780643	4.11E-05	0.001382	1.986837	5.163495	6.313361	6.350091	4.066741	5.880351	5.725349	4.822272	4.692543	4.933786	4.588849	4.768337	5.065272	4.989761	4.885716
Usp53	1.129587	6.086494	5.780452	4.12E-05	0.001382	1.930526	5.957189	6.908895	7.110605	5.920408	6.76473	7.485649	5.439557	5.954958	5.330461	5.902798	5.445986	6.101377	5.522982	5.390475
RT1-Ba	3.249536	3.997519	5.779052	4.13E-05	0.001382	2.281519	2.787845	4.625556	5.473221	1.033666	5.510649	4.677595	2.144252	5.5474	1.360416	6.477491	6.005952	6.765057	2.022549	1.755962
Pdlim1	1.772141	3.012032	5.778911	4.13E-05	0.001382	2.192513	3.395435	4.664267	4.74506	2.029493	4.272298	4.06389	2.01015	2.505864	2.568526	2.681644	2.568656	2.568523	2.079189	2.245294
Rai14	1.680982	3.698188	5.773449	4.17E-05	0.001391	2.05451	4.47136	5.202427	5.652234	2.977436	5.266683	5.337331	2.518182	2.634802	2.785194	3.090803	3.401255	3.170693	2.62712	2.791492
Casp7	1.501172	2.926076	5.769912	4.19E-05	0.001396	2.202522	3.026365	4.244344	3.95649	2.061822	4.147475	3.698398	2.48486	2.738882	2.166756	2.558244	2.66245	3.110019	2.060555	2.278116
Mmp12	8.328184	-1.609074	5.767999	4.21E-05	0.001397	1.5527	-3.260591	2.607522	3.359399	-3.260591	3.409963	2.654873	-3.260591	-0.992527	-3.260591	-0.665882	-2.293564	1.643459	-3.260591	-3.260591
Twf1	0.782318	4.592859	5.766108	4.22E-05	0.001397	1.979306	4.3544	5.352198	5.160327	4.555288	5.276936	5.167077	4.390007	4.351577	4.303799	4.320048	4.065415	4.468815	4.262487	4.338735
Slc1a5	2.314835	2.72664	5.765185	4.23E-05	0.001397	2.268156	2.620642	4.142566	4.742881	1.128291	3.731543	3.826262	1.809437	2.695192	1.717448	2.469694	2.696109	3.118845	1.79348	1.971974
LOC308990	2.379552	1.514132	5.761407	4.26E-05	0.0014	2.370391	0.873216	2.36878	2.607481	-0.411255	2.810268	2.131561	0.498056	1.883083	0.884903	1.828755	1.762161	2.300247	1.001683	1.32462
Id1	1.370196	4.575554	5.760244	4.27E-05	0.0014	1.993751	4.573017	5.461064	5.368284	3.617117	5.714243	5.236926	4.42995	4.078726	4.299404	4.609719	4.227643	4.233112	3.913738	4.365708
C1qc	1.746694	6.313888	5.759369	4.28E-05	0.0014	1.907519	5.838358	7.429836	7.434004	4.956595	7.0646	6.613373	5.012583	7.055875	5.401902	6.685923	6.439298	7.358202	5.465713	5.661644
Rsu1	0.89238	5.064418	5.757678	4.29E-05	0.0014	1.915164	5.176474	5.820051	5.910893	5.175528	6.316174	6.219483	4.096591	4.905609	4.421726	4.7573	4.615483	4.811327	4.357143	4.369521
Ehbp1l1	1.027626	5.970717	5.75463	4.31E-05	0.001404	1.904052	5.56163	6.487026	6.151665	5.001038	6.319484	6.255268	5.834566	6.295728	5.684033	6.431345	6.010418	6.414379	5.435298	5.734407
Pacsin1	-0.833344	7.781805	-5.751814	4.33E-05	0.001408	1.883865	8.235925	7.392635	7.534427	8.36521	7.256858	7.685925	7.823452	7.837743	7.901467	7.606911	7.756202	7.780794	8.076809	7.698205
Tcirg1	1.363235	5.579786	5.740287	4.42E-05	0.00143	1.920618	4.810783	6.070055	5.895281	4.214255	5.894848	5.60107	5.246787	6.379776	5.299983	6.280078	5.845237	6.389067	4.725704	5.500615
Angpt2	2.607489	2.003872	5.739902	4.43E-05	0.00143	2.275799	2.072763	3.859159	4.521788	0.664146	3.440689	3.713177	1.511505	0.972957	1.819202	1.023513	1.347792	1.082389	0.649393	1.889408
Fblim1	3.661744	-0.459707	5.737871	4.44E-05	0.001432	2.162434	-1.052261	1.425638	2.47722	-1.788967	1.877213	1.688586	-0.529223	-1.196868	-1.536754	-1.33445	-0.318799	-0.24119	-1.904936	-1.040339
Shmt2	0.916962	5.598047	5.734845	4.47E-05	0.001436	1.870739	5.573126	6.132979	6.176096	4.872521	6.039366	6.181017	5.384156	5.443407	5.397796	5.618694	5.376758	5.467141	5.282878	5.460068
Rasal3	1.50436	3.272008	5.727053	4.53E-05	0.001447	2.161784	2.460078	3.7962	3.74196	2.061822	3.922149	3.502454	2.854546	4.073048	2.241976	3.814705	3.84309	4.304475	2.363894	3.012401
Btbd17	-2.16642	3.988106	-5.726073	4.54E-05	0.001447	2.084924	4.805223	2.943995	1.723223	3.527359	2.109124	1.353343	4.848708	4.729108	4.983948	5.06998	4.967529	5.056112	4.680656	5.204492
Dab2	1.482311	5.719207	5.725892	4.54E-05	0.001447	1.835392	6.277378	6.960654	7.449454	5.153196	6.9486	7.403363	4.798736	4.860173	4.922398	5.094864	5.478587	4.928903	4.757867	5.070381

Mgp	1.932238	4.718213	5.723065	4.56E-05	0.001449	1.920476	4.883821	6.179975	5.54876	3.315784	6.335105	5.938452	4.510807	4.123366	4.116621	4.505931	4.49954	4.40891	3.646466	4.111741
Cd276	0.804061	5.422417	5.722498	4.57E-05	0.001449	1.845283	5.384666	6.178948	5.973563	5.197519	5.933168	6.28525	5.092185	5.285418	5.061268	5.318389	5.142525	5.290932	4.763639	5.044382
Ifitm1	3.866899	2.120242	5.717656	4.61E-05	0.001455	2.269155	2.263247	4.721865	4.698594	-0.411255	5.020453	4.071557	-0.067151	2.488905	0.449965	1.253084	1.82436	3.269114	0.037347	0.753927
Shc1	1.074758	5.251205	5.717072	4.61E-05	0.001455	1.879804	4.785591	5.793433	5.441325	4.253272	5.69653	5.413212	4.994938	5.512163	5.021793	5.472549	5.329285	5.841005	4.839377	5.165551
Cth	1.123875	3.738447	5.714325	4.64E-05	0.001459	1.960819	3.963192	4.442374	4.835775	3.36843	4.803569	4.9923	3.569982	3.000548	3.289407	3.293574	2.930971	3.300483	3.32598	3.35002
Tmem117	1.122167	4.283175	5.70895	4.68E-05	0.001469	1.914083	3.912376	5.148837	4.96291	4.054744	5.224928	5.086123	3.479833	4.095628	3.709451	3.924277	3.792123	4.387158	4.075609	4.195741
Slc3a2	1.104675	8.047475	5.702944	4.73E-05	0.001481	1.791637	7.835361	8.496327	8.805469	7.14908	8.551346	8.54221	7.734103	8.002516	7.790747	8.042903	7.704769	8.143096	7.709921	8.162975
Ostf1	1.038293	3.74693	5.701517	4.75E-05	0.001481	1.976104	3.683012	4.496185	4.182328	3.067759	4.548211	4.361521	3.299932	3.777297	3.375441	3.588098	3.656224	3.906064	3.333761	3.302949
Mboat1	2.272896	1.824804	5.699367	4.76E-05	0.001483	2.25473	0.739674	2.33964	2.992072	0.664146	3.546179	2.743931	1.372662	1.49863	1.663747	1.678822	1.456531	1.989793	1.724133	1.634478
Tubb2b	1.116864	7.144848	5.696191	4.79E-05	0.001485	1.773369	7.247998	8.305126	7.673345	7.015673	8.459436	8.549778	6.577738	6.310282	6.797673	6.432321	6.482535	6.617184	6.798655	6.772403
Iqgap3	2.338067	2.078234	5.694646	4.80E-05	0.001485	2.238951	1.108109	2.570375	2.939761	0.577356	3.617838	3.295428	1.726334	1.934092	1.981639	2.423294	1.959843	2.268872	1.150071	1.971974
Arc	-1.854382	6.699896	-5.694273	4.81E-05	0.001485	1.77163	7.261766	4.996138	5.312072	7.550742	6.12699	5.76962	7.6973	6.421709	6.835353	7.383277	6.779316	7.394996	7.071853	7.215793
Ctsb	1.041405	10.26161	5.691949	4.83E-05	0.001488	1.833085	9.968194	10.66251	10.83016	9.636858	11.13121	10.75929	9.499842	10.48651	9.756965	10.33367	10.22404	10.72459	9.681833	9.968149
Rgs6	-0.759672	4.526917	-5.690446	4.84E-05	0.001488	1.82587	4.97826	4.472889	4.369101	5.236986	4.36492	4.209984	4.595201	4.616508	4.504248	4.328465	4.473897	4.519683	4.383621	4.392186
Plau	2.775684	2.208007	5.670564	5.02E-05	0.001538	2.195662	0.592497	3.317238	3.289459	1.033666	3.832311	3.737479	0.903114	2.724465	1.180909	2.831299	3.013384	4.037544	0.037347	0.887553
Stom	2.23595	3.336354	5.667729	5.04E-05	0.00154	2.058233	2.799078	4.393585	4.331892	1.502648	4.654263	3.939526	3.169687	3.599118	2.917273	3.050666	3.031075	4.069761	2.452318	2.982839
Hmga1	0.921865	5.271317	5.667183	5.05E-05	0.00154	1.732015	5.487189	6.230459	6.770027	5.59137	6.22468	6.615341	4.599099	4.729108	4.473365	4.556968	4.612548	4.665351	4.82012	4.468825
Il1rn	4.694721	0.132135	5.659773	5.12E-05	0.001557	2.103581	-0.760787	2.411416	3.863274	-1.575022	3.617838	2.872384	-1.21312	-0.81357	-0.929891	-0.420466	0.263333	0.618025	-1.904936	-1.632384
Stt3b	1.026883	6.047798	5.643123	5.27E-05	0.0016	1.686885	5.290305	6.506004	6.593236	5.795003	6.35943	6.827738	5.87853	5.779538	5.905526	5.667616	5.752302	6.155991	5.979972	6.202449
Lpxn	1.22045	3.328092	5.641789	5.29E-05	0.0016	1.948931	3.05476	3.719097	3.967729	2.427034	4.213017	3.830786	3.200804	3.311493	3.038267	3.385186	3.525031	3.52291	2.725031	2.83626
Chi3l1	1.861242	7.693977	5.639392	5.31E-05	0.001603	1.671788	7.081459	9.326748	7.715626	6.558288	9.18497	8.472798	6.873231	7.957382	6.930798	8.031348	7.61524	7.985553	6.768834	7.222133
Parvg	1.608491	3.056834	5.628974	5.41E-05	0.00163	1.964228	2.63325	3.713429	3.834912	2.154676	4.484758	3.862069	2.05625	3.272386	2.204856	3.100666	3.091342	3.744381	2.575555	2.278116
Calml4	-2.910848	2.946857	-5.60814	5.62E-05	0.001684	1.870553	5.19147	0.679782	2.162035	4.256768	2.460496	1.910004	2.518182	2.822481	3.069578	2.945148	4.755092	2.691938	3.003904	3.060363
Cacna1i	-0.821946	6.923838	-5.607253	5.63E-05	0.001684	1.603752	6.613507	6.061157	6.014784	6.957795	6.113704	5.67087	7.72655	7.302676	7.651981	7.016052	7.169093	7.095817	7.774231	7.780332
Anxa4	0.948592	4.579789	5.606927	5.63E-05	0.001684	1.658648	4.899562	5.411164	5.603828	4.638025	6.025245	5.805694	3.699086	4.161323	3.924502	3.962637	4.052479	4.300602	3.954679	3.751656
Kcnc1	-0.909515	6.885687	-5.600719	5.70E-05	0.001699	1.590144	6.794179	6.022713	6.092997	7.353476	6.481185	6.061943	7.232562	7.117858	7.306453	7.021902	7.01148	7.048679	7.421778	7.446714
Pabpc1	0.895089	8.148487	5.598139	5.72E-05	0.001702	1.605874	7.870062	8.603257	8.90176	8.145725	8.911949	9.195738	7.551023	8.098224	7.528319	7.9392	7.917677	8.292935	7.528174	7.600614
Ssc5d	1.544358	6.176752	5.596952	5.74E-05	0.001702	1.605442	5.362121	7.350802	6.374928	5.45653	7.047234	7.036048	5.478169	6.847992	5.444323	6.416616	6.100103	6.726602	4.982355	5.875144
Plekha4	2.393476	2.530086	5.592039	5.79E-05	0.00171	2.012641	1.717752	3.914553	4.106393	1.033666	2.901447	3.914167	1.697535	3.024282	1.255413	3.12019	2.604547	3.261164	1.574559	1.634478
Ptbp1	1.271381	5.791153	5.59156	5.79E-05	0.00171	1.60962	5.550041	6.462737	6.528929	4.635342	6.434625	5.988921	5.721099	5.722645	5.577343	5.875772	5.768213	5.871352	5.217096	5.752039
Rpl3	0.801406	8.77982	5.587753	5.83E-05	0.001718	1.594893	8.580971	9.320003	9.040954	8.187301	9.321656	9.060211	8.60688	8.730782	8.603616	8.937862	8.855255	8.754671	8.408963	8.512002
Atf3	4.174116	1.254831	5.583518	5.88E-05	0.001727	2.059418	-0.518419	3.055728	4.475294	-0.166914	3.726565	3.444586	0.851758	2.030999	-0.27702	0.646479	1.03357	1.910689	-0.893866	-0.621773
Rhbdf2	1.494261	3.129451	5.580821	5.91E-05	0.001731	1.912099	2.555898	3.61957	3.750662	1.740915	3.721569	3.314938	2.703406	3.690957	2.568526	3.978769	3.493474	3.921279	2.220182	2.733504
Abcc3	2.014518	2.784222	5.573513	5.99E-05	0.001749	1.980294	1.212615	3.42521	2.992072	1.593908	3.534831	3.658228	2.144252	3.638097	2.127623	3.784431	3.189137	3.811244	1.521045	2.622863
Lrrc42	0.729971	5.065005	5.572609	6.00E-05	0.001749	1.594116	4.902169	5.656872	5.453368	4.72387	5.691429	5.360987	4.971071	4.952752	4.902249	4.973987	4.847853	4.872617	4.800602	4.847954
Rnf152	-1.352894	1.454408	-5.566166	6.07E-05	0.001761	1.936701	2.706665	1.58312	1.269823	2.546081	1.018831	1.353343	0.798508	1.393205	1.360416	1.388059	1.347792	1.008331	1.859646	1.32462
Gngt2	3.458219	0.586933	5.566089	6.07E-05	0.001761	2.006087	-0.760787	2.21681	2.804219	-0.600663	3.409963	1.960428	-1.009457	1.198329	-1.10526	1.28803	1.719153	1.082389	-1.413173	-0.886923
Arid5a	1.439242	4.246691	5.545614	6.30E-05	0.001824	1.606667	4.128153	5.469496	6.024683	3.847833	5.26325	4.90488	3.609168	3.904241	3.555781	3.973412	3.73929	4.304475	3.495104	3.326676
Gdf10	-2.545186	3.525709	-5.544335	6.31E-05	0.001824	1.793474	3.695089	0.24948	2.067688	4.577819	2.335271	1.766229	3.837517	4.337436	4.71363	4.285878	3.882605	4.502925	4.376105	4.976008
Plcb3	1.168243	5.245135	5.540512	6.36E-05	0.001832	1.531962	5.123842	6.065613	5.611014	4.446243	6.04037	6.060017	4.937596	5.199424	4.840059	5.26018	4.820137	5.350218	4.634155	5.086685
Frmd8	0.979483	4.806612	5.538947	6.38E-05	0.001833	1.56578	4.506048	5.252144	5.158693	4.174153	5.685027	5.154498	4.468816	4.836907	4.325577	4.788142	4.789281	5.103568	4.579577	4.528015
Adarb1	-0.696879	7.887944	-5.532904	6.45E-05	0.001849	1.472055	8.003204	7.414197	7.380743	8.059046	7.193321	7.349592	8.348368	7.976898	8.03535	8.085428	8.117989	8.117729	8.298749	8.057916
Slain1	-1.024724	5.734695	-5.529137	6.49E-05	0.001858	1.470808	6.085441	5.101882	5.058875	6.478209	5.564652	5.313282	5.617289	5.903095	5.575527	5.765405	5.762929	6.086832	6.081661	5.921329
Cyr61	4.150213	2.832309	5.527648	6.51E-05	0.001858	1.932711	0.739674	4.538474	5.526131	1.033666	5.146953	4.704965	3.647317	1.198329	4.044916	1.942797	1.783193	1.989793	1.700256	2.011549
Adam15	0.884545	7.063124	5.525865	6.53E-05	0.00186	1.451984	6.621438	7.186629	7.287341	6.226101	7.561217	7.193218	7.134575	7.038498	7.083672	7.109373	6.943625	7.279215	6.915379	7.315508
Pcna	0.99982	5.323524	5.521649	6.58E-05	0.00187	1.476438	5.123842	5.985571	5.787352	5.206584	6.426973	6.457523	4.767901	4.97728	4.840059	5.013676	4.955998	5.300661	4.890388	4.836982
Rsph10b	-1.686585	6.15865	-5.519802	6.60E-05	0.001872	1.46413	6.19686	3.967899	5.145557	6.677179	4.677668	5.184859	7.214887	6.945999	6.984489	6.946664	6.580404	6.666925	6.267804	6.793131
Adrb3	-3.613366	-0.560799	-5.517245	6.64E-05	0.001876	1.841248	1.245833	-2.680539	-2.432145	0.485009	-0.954037	-2.369898	0.68572	-0.048075	-0.632503	0.475109	0.071497	0.567345	0.434663	1.122575
Arhgap20	-1.104953	6.219488	-5.515714	6.65E-05	0.001877	1.435418	6.330601	5.798796	5.759599	7.207593	5.338601	5.768441	6.444337	6.473276	6.424544	6.258215	6.358399	6.345555	6.393172	6.193874
Lamc1	0.807211	6.597956	5.509926	6.72E-05	0.001892	1.422062	6.438695	7.099678	7.285845	6.278624	7.043236	7.23222	6.554839	6.242338	6.343609	6.413652	6.068326	6.599953	6.363075	6.423939
Atic	0.75062	4.850402	5.50337	6.81E-05	0.001907	1.483488	4.569741	5.134062	5.453368	4.552446	5.285425	5.357855	4.732848	4.771798	4.628589	4.509637	4.778847	4.885603	4.689778	4.610873
Ttll12	0.762657	4.770983	5.50282	6.81E-05	0.001907	1.46736	4.894334	5.52556	5.543762	4.613698	5.674724	5.303548	4.34429	4.308732	4.526983	4.406073	4.502714	4.390807	4.314529	4.503936
Nsmce2	0.968521	3.573651	5.500822	6.84E-05	0.001907	1.609358	3.516065	4.466163	4.153089	3.197095	4.548211	4.098075	3.200804	3.201289	3.216682	3.28395	3.493474	3.405197	3.116435	3.270693
Fez1	-0.777381	8.230684	-5.500769	6.84E-05	0.001907	1.421446	8.367194	7.46526	7.649987	8.646571	7.882636	7.918922	8.348947	8.446429	8.485011	8.413811	8.336937	8.351907	8.451375	8.470004
Stxbp2	1.759379	3.425375	5.492677	6.94E-05	0.001926	1.698827	3.035892	4.294561	4.493553	1.945353	4.191499	3.839794	2.955303	4.244507	2.772565	3.669864	3.644778	3.738666	2.236876	3.060363
Apobec3b	2.464078	0.3003	5.492657	6.94E-05	0.001926	1.864363	-0.634519	1.480065	1.143363	-0.941548	1.600747	1.785004	-0.279766	1.393205	-1.304935	0.938085	0.676952	1.152831	-0.893866	-0.297732
Cav2	1.280543	4.269382	5.491396	6.96E-05	0.001926	1.478242	4.499177	5.294522	5.303227	4.062753	5.732983	5.871954	3.850657	3.012464	3.641623	3.595091	3.20481	4.018805	3.91892	3.858608
Trip13	2.239782	0.300236	5.488301	7.00E-05	0.001933	1.872095	-0.518419	1.480065	1.061799	-0.705589	1.96438	1.450626	-0.067151	-0.048075	0.317129	0.134646	0.071497	0.460309	-0.123798	0.119416
Pole	1.803841	3.276495	5.48458	7.04E-05	0.001939	1.726427	2.279379	4.573314	3.590238	2.061822	3.909025	3.784887	3.577905	3.311493	3.392049	3.385186	2.86345	3.397965	3.042398	2.879681
Pfkm	-0.625081	9.146799	-5.483122	7.06E-05	0.001939	1.415347	9.455373	8.771584	8.701869	9.431828	8.960994	8.83957	9.304488	9.170703	9.332606	9.152923	9.20591	9.101963	9.367408	9.260784
Tspan2	-1.211374	7.172346	-5.482857	7.07E-05	0.001939	1.371645	7.330149	6.786687	6.035406	7.687493	6.357821	6.015001	7.390475	7.541904	7.263662	7.564783	7.277751	7.910663	7.587543	7.675671
Myo1g	2.398692	1.500339	5.472528	7.20E-05	0.001971	1.86493	0.830061	2.884706	3.171281	0.22441	3.068148	2.230763	0.118118	2.400986	0.449965	2.202958	1.719153	2.170435	-0.512812	0.799858
Ptpn2	0.824022	4.25578	5.471315	7.22E-05	0.001971	1.463268	3.968177	4.88707	5.118919	4.270665	4.86149	4.909165	4.218166	3.929599	3.805565	4.005262	4.012962	3.778199	3.999391	3.901371
Lgi1	-1.432468	6.78994	-5.451969	7.48E-05	0.002038	1.317387	6.50193	4.830589	5.705797	7.377756	5.516429	5.964422	7.282504	7.413547	7.625011	7.257782	7.260513	7.308923	7.575699	7.456157
Sprr1a	6.41259	-2.200577	5.450223	7.50E-05	0.002039	1.231751	-1.908947	2.248512	2.645305	-3.260591	1.72577	3.172479	-3.260591	-2.550306	-2.155202	-3.260591	-1.978322	-2.668207	-3.260591	-3.260591
Tnfaip8l2	1.164778	3.52596	5.449253	7.52E-05	0.002039	1.56169	3.303772	4.155166	3.902849	2.656049	4.421469	4.017012	3.038038	4.123366	2.597457	3.913127	3.944596	4.105722	2.601568	2.733504
Tecpr1	-0.60765	7.824174	-5.447924	7.54E-05	0.00204	1.307922	7.730358	7.110501	7.062053	7.777539	7.259014	7.154035	8.364776	8.130361	8.172351	7.999086	8.124699	8.111663	8.327772	8.221628
Fosl1	2.333573	3.000013	5.442002	7.62E-05	0.002057	1.615221	2.213741	3.899656	5.559943	2.499634	4.677668	4.526803	2.266943	2.419003	1.981639	2.454393	2.442128	2.542521	2.783013	1.971974
Pik3r5	2.042855	1.948525	5.437104	7.69E-05	0.002068	1.770713	1.459129	2.854122	3.177748	0.621404	3.060255	2.940957	0.57848	2.80888	0.97585	2.221154	1.959843	3.009199	1.28461	1.048408
LOC690349	0.86625	3.652502	5.437016	7.69E-05	0.002068	1.466177	3.689063	4.435504	4.284739	3.40669	4.60357	4.297582	3.159163	3.330656	3.271566	3.302147	3.592118	3.245131	3.409342	3.237699
Sspn	-1.070021	4.343901	-5.429915	7.79E-05	0.00209	1.380962	4.983193	3.948728	3.520715	4.616421	3.628553	3.848745	4.376444	4.446862	4.189688	4.613168	4.424552	4.926391	4.683703	4.689229
Tjp2	1.021324	6.407934	5.428695	7.81E-05	0.00209	1.270108	6.309154	7.167581	7.720341	6.403706	7.141427	7.483495	5.900809	6.031614	5.63952	5.91961	5.741596	6.368863	5.949913	5.953435
Cntn1	-1.290495	8.419445	-5.422101	7.90E-05	0.00211	1.283552	8.843025	7.206941	7.918941	9.227906	7.608666	8.247034	8.482088	8.639123	8.806371	8.212231	8.538056	8.472576	8.924461	8.749775
Pdcl3	0.668102	4.479611	5.417592	7.97E-05	0.002122	1.329474	4.630741	5.075612	5.274852	4.357872	5.163626	5.108656	4.113042	4.279444	4.06577	4.201325	4.160911	4.136468	4.130242	4.088797
Mical2	-0.823568	8.409016	-5.415668	8.00E-05	0.002122	1.26464	8.644235	7.516205	7.734119	8.674933	8.097203	7.999001	8.753996	8.623911	8.813902	8.454757	8.601412	8.479231	8.722214	8.615959
Pkn3	1.484385	3.088473	5.414511	8.01E-05	0.002122	1.564166	2.718545	4.4112	3.609501	2.184345	3.827669	3.830786	2.787802	2.91426	3.09008	3.050666	2.468337	2.89053	2.601568	3.050898
Skap2	1.215208	4.285158	5.414317	8.02E-05	0.002122	1.367683	4.045673	4.788078	4.930726	3.861672	5.488762	5.432691	3.231264	4.604749	3.440756	4.394093	4.160911	4.775496	3.508915	3.417866
Tex15	-1.99099	2.569478	-5.411983	8.05E-05	0.002127	1.574552	3.881004	1.082904	1.921212	3.315784	1.578815	1.839907	2.787802	2.030999	2.928701	2.572488	3.807603	2.568523	3.03287	2.932182
Gmnn	1.644891	2.191198	5.40822	8.11E-05	0.002132	1.600079	2.035255	3.613496	3.131857	2.13961	3.994346	4.153309	1.938115	1.429209	1.663747	1.451089	1.430105	1.11804	1.377871	1.608903
Ifitm2	2.128132	3.438494	5.406474	8.13E-05	0.002132	1.528411	3.695089	4.567042	4.380359	1.379803	4.827017	4.568038	2.659281	3.40486	2.74697	3.456042	3.656224	3.686193	2.423441	2.868948
Mllr1	3.391083	0.444504	5.406021	8.14E-05	0.002132	1.721845	-0.518419	1.854335	2.861581	-1.223791	2.661692	1.708392	-0.831021	1.155984	-1.10526	1.28803	1.03357	1.618784	-1.904936	-0.505482
Il1b	6.135383	-0.59802	5.405849	8.14E-05	0.002132	1.227072	-2.659044	2.100024	3.196978	-2.731185	2.781752	1.519512	-1.21312	-0.150641	-1.536754	-0.665882	-0.731011	-0.070959	-1.904936	-1.899685
Anks1a	0.956278	6.109235	5.400746	8.22E-05	0.002145	1.231789	5.7427	6.906413	6.638481	5.388058	6.29783	6.23151	6.073259	6.050519	5.909857	6.213469	6.005952	6.187794	5.92695	5.979863
Elfn1	-1.40432	5.291567	-5.40031	8.23E-05	0.002145	1.26356	5.169999	4.339413	3.93756	6.2812	4.622454	4.440619	5.710327	5.225253	5.699127	5.533887	5.465635	5.591235	6.034699	6.076327
Ctnna2	-0.745147	6.981694	-5.39854	8.25E-05	0.002147	1.210391	7.31995	6.412895	6.681781	7.501873	6.684205	6.883792	6.991393	7.02973	7.024984	6.979773	7.100053	6.939584	7.233943	6.972416
Rgs10	-1.036128	6.118648	-5.396497	8.29E-05	0.00215	1.212728	6.339284	5.196344	5.532452	6.890201	5.597747	5.987909	5.961117	6.606012	6.093693	6.72231	6.459854	6.543715	5.842211	5.912209
Dsn1	1.619219	1.934112	5.395037	8.31E-05	0.002152	1.701033	0.915118	2.713546	2.238266	1.128291	2.856578	2.665044	2.266943	1.49863	1.959523	1.898256	1.864385	1.715048	1.60059	2.194616
Pknox2	-0.804853	5.421178	-5.384858	8.47E-05	0.002188	1.209255	5.67572	5.019295	5.05361	6.060988	4.919359	5.272286	5.513711	5.473892	5.575527	5.693914	5.472126	5.474036	5.235902	5.493644
Lgals1	1.830637	4.372597	5.38371	8.48E-05	0.002188	1.285861	4.958358	5.337533	6.210408	3.295536	6.126045	6.013011	3.40164	3.966821	3.341641	3.913127	3.760656	4.257294	3.203938	3.524301
Tfec	4.637658	-1.750164	5.379552	8.55E-05	0.002199	1.162846	-3.260591	0.632508	1.004744	-1.788967	1.173177	1.221719	-2.088409	-0.81357	-2.604521	-1.564464	-0.510232	-0.541385	-3.260591	-3.260591
Ppap2b	-0.966163	8.398765	-5.378158	8.57E-05	0.002199	1.20122	9.300222	8.142221	7.88402	8.664116	8.067241	7.979012	8.308748	8.290014	8.313353	8.456678	8.292245	8.662233	8.577149	8.650252
Tubb4a	-0.993253	10.5979	-5.377466	8.58E-05	0.002199	1.270088	11.1347	10.12871	10.28753	11.40373	10.44242	10.24264	10.13423	10.82101	10.5289	10.42745	10.66621	10.8637	10.76857	10.5219
Sla	2.016675	2.229673	5.37483	8.62E-05	0.002205	1.582569	1.936985	3.224637	4.204668	1.502648	3.89578	3.408717	0.903114	1.908813	0.837186	1.852296	2.401899	2.827322	1.60059	1.122575
Gal	2.478799	1.987118	5.36852	8.73E-05	0.002225	1.634544	1.310059	2.690649	3.98258	0.932396	3.998482	3.426763	1.000645	1.658838	1.142163	2.027955	1.456531	2.268872	1.077784	1.32462
Syk	1.111746	4.248706	5.367756	8.74E-05	0.002225	1.322118	3.529616	4.386479	4.559421	3.706736	5.074352	4.887611	3.742028	4.718236	3.620637	4.606261	4.512193	4.923876	3.488149	3.814538
Sp100	1.803106	2.039392	5.35788	8.90E-05	0.002255	1.610385	1.568201	2.943995	3.091325	0.932396	3.020129	2.932562	1.408645	2.38274	1.426399	2.543858	1.533025	2.715403	1.040235	1.445134
Gnb4	1.409957	3.333141	5.356955	8.91E-05	0.002255	1.412016	3.154337	4.175923	4.483148	2.41457	3.864394	4.124114	3.116285	3.104407	2.984524	2.831299	3.022257	3.692118	2.994118	2.868948
Mocos	2.038965	1.670332	5.355693	8.94E-05	0.002255	1.653452	0.915118	3.091129	2.588191	0.386344	2.692656	2.146157	1.63815	1.564863	1.520039	1.986005	1.260496	1.826994	0.962073	1.355706
Mpp2	-0.655783	6.540915	-5.35543	8.94E-05	0.002255	1.131119	6.50193	6.001938	6.038074	6.704312	5.9092	5.844248	6.901128	6.888159	6.981063	6.570909	6.742875	6.659383	6.978159	6.869156
Pak1ip1	0.945032	4.560762	5.354801	8.95E-05	0.002255	1.209628	4.206171	5.127683	5.674127	4.706082	5.383574	5.42523	4.150713	4.193082	4.277224	4.361652	4.324082	4.051439	3.969738	4.070176
RGD1306730	0.688619	5.017644	5.35146	9.01E-05	0.002259	1.174902	4.862563	5.438908	5.633536	4.883868	5.709204	5.457773	4.637512	4.980316	4.534483	4.830243	4.879936	4.908688	4.850267	4.689229
Dhrs3	-1.158639	4.369159	-5.351139	9.01E-05	0.002259	1.230138	4.840987	3.696289	3.353699	4.897366	4.05518	3.780216	4.123906	4.308732	4.461611	4.647212	5.032648	4.76429	4.582846	4.704402
Rcc1	1.03376	3.118068	5.349788	9.03E-05	0.002259	1.451	2.706665	3.595119	3.376367	2.255954	3.391209	3.622139	3.159163	3.282262	2.834633	3.00938	3.132898	3.179156	3.098279	3.195366
Tal1	1.186598	3.240239	5.349236	9.04E-05	0.002259	1.453208	2.658139	3.894655	3.453014	2.198954	3.633881	3.414757	3.049479	3.727562	2.894141	3.649853	3.474203	3.848862	2.639729	3.002614
P4hb	0.869054	7.9878	5.34568	9.10E-05	0.002269	1.121998	7.717465	8.197577	8.483854	7.516162	8.673923	8.592371	7.484091	7.9643	7.656284	7.993803	7.819608	8.239059	7.610143	7.886917
Slc18a3	2.977688	0.108213	5.33925	9.21E-05	0.002292	1.626158	-0.899176	2.570375	2.597868	-0.411255	1.685287	1.821837	-2.088409	0.04768	-0.632503	0.280604	-0.149817	-0.24119	-0.305233	-0.748265
Zc3h12d	3.189077	0.10645	5.337027	9.25E-05	0.002294	1.590926	-1.22354	2.184396	2.456093	-0.81875	1.533923	1.627481	0.028455	0.301855	-0.504027	-0.961779	0.432628	0.151783	-0.628995	-1.212031
Dmd	-0.692667	5.152927	-5.336651	9.26E-05	0.002294	1.136655	5.448478	4.646529	4.666764	5.476153	4.815341	4.955481	5.469678	5.278033	5.360307	4.99529	5.168735	5.225023	5.415271	5.2651
Impdh2	0.733882	6.321105	5.335054	9.28E-05	0.002296	1.093666	6.017339	6.915699	6.788632	6.257843	6.836383	6.945071	6.286495	6.076293	6.040696	6.240483	6.004833	6.119079	5.829885	6.156849
Eif4a1	0.613828	7.467023	5.330662	9.36E-05	0.00231	1.084026	7.212732	7.697614	7.967573	7.14201	7.816817	7.684051	7.412795	7.375416	7.408442	7.429005	7.458999	7.35073	7.284499	7.306587
Slc7a7	1.88412	2.540552	5.32716	9.42E-05	0.002319	1.544753	1.764345	3.037697	3.070623	0.896996	3.686105	2.82781	1.782265	3.301815	1.914249	3.258764	3.324422	3.637891	1.28461	2.105984
Irf9	1.025691	5.215195	5.326236	9.44E-05	0.002319	1.139127	4.745509	5.675942	5.910893	4.549599	5.490232	5.594433	5.162364	5.35726	4.827903	5.559142	5.08016	5.642628	4.55649	4.904232
Lrrfip2	0.737248	5.615744	5.320679	9.54E-05	0.002338	1.083734	5.425104	6.2433	6.302059	5.556344	6.103179	6.262822	5.44173	5.35258	5.497094	5.568055	5.246517	5.145199	5.174405	5.33501
Ermn	-1.656184	6.236902	-5.318757	9.57E-05	0.002342	1.068253	6.690926	5.871858	4.652905	7.262086	5.739176	5.055522	6.33171	6.527221	6.39395	6.380641	5.993598	6.709176	6.992361	6.739636
Rplp0	1.150891	8.593763	5.317015	9.60E-05	0.002345	1.080397	8.524355	9.44765	9.086132	7.58173	9.297939	8.987986	8.397041	8.402957	8.276651	8.69605	8.493288	8.615254	8.07042	8.439497
Ccnd1	1.342812	5.726681	5.30698	9.78E-05	0.002384	1.063626	5.643365	6.283067	6.332809	4.965167	7.088081	6.854649	5.345229	4.921495	5.370819	5.504473	5.215906	5.647211	5.573143	5.460068
Arhgap9	1.781189	3.301111	5.305738	9.80E-05	0.002385	1.420501	2.32672	3.42521	3.81015	1.679737	4.070978	3.653128	3.169687	4.229271	2.858731	4.196735	3.760656	4.365074	2.079189	2.780079
Lrrc4	-1.049738	7.108373	-5.301643	9.88E-05	0.002398	1.027827	7.246973	5.8744	6.47678	7.690081	6.551142	6.768829	7.340704	7.369637	7.435232	7.288308	7.229242	7.417525	7.342048	7.498554
Plcl1	-0.873956	5.549528	-5.300553	9.90E-05	0.002398	1.037608	5.995534	5.40062	5.25205	6.47296	5.223162	5.577032	5.54436	5.507961	5.399851	5.229048	5.381938	5.521398	5.797899	5.423859
Pth1r	1.438173	3.817994	5.296375	9.98E-05	0.002398	1.272943	3.303772	4.63158	4.28174	2.613062	4.325951	4.257812	3.669732	3.698353	3.641623	4.390078	3.549789	4.078836	3.254	3.874793
Slc6a1	-0.732753	9.024358	-5.296346	9.98E-05	0.002398	1.056411	9.324111	8.476594	8.596448	9.234458	8.649635	8.466633	9.198635	8.957079	9.25387	8.985713	8.981612	9.114979	9.452836	9.651533
Recql4	1.632293	2.371059	5.295925	9.98E-05	0.002398	1.522657	1.212615	2.833367	2.568639	0.823477	2.575715	2.446602	3.116285	2.268138	2.539002	2.783118	2.568656	2.703718	2.348615	2.745289
Ubr7	0.708819	5.865742	5.295906	9.98E-05	0.002398	1.041882	5.365903	6.333825	6.010262	5.380068	6.098369	5.883978	5.821279	6.094625	5.82219	5.967554	6.039116	6.030652	5.613652	5.686309
Adcy7	2.147947	2.900507	5.294852	0.0001	0.002398	1.493977	1.741237	3.576505	3.495051	0.705659	3.181638	2.957603	2.247205	3.960684	2.347926	3.478908	3.454671	4.220868	2.562371	2.942457
Plekhg2	1.440429	4.998229	5.294188	0.0001	0.002398	1.109116	4.774252	5.994946	5.693438	3.696477	5.56186	5.373446	4.714996	5.113519	4.818719	5.249138	5.073772	4.956243	4.116777	4.89103
Tsku	1.921167	2.539513	5.288442	0.000101	0.002414	1.410893	2.501925	3.459476	4.065129	1.158502	3.403739	3.775529	1.297885	2.650138	1.255413	2.359011	3.013384	2.960934	2.022549	1.951771

Nfkb2	1.313134	3.401958	5.287573	0.000101	0.002414	1.297633	3.016775	3.958345	4.079015	2.363612	4.227186	3.637716	2.717819	4.026799	2.995434	3.58107	3.461211	3.843548	2.771602	3.106782
RGD1562084	-0.834935	3.78446	-5.287335	0.000101	0.002414	1.197116	4.012279	3.037697	3.453014	3.967847	3.075998	3.045873	4.023054	4.015001	4.165741	4.173561	3.906764	4.037544	3.97969	4.208569
Timeless	1.767013	2.560859	5.284791	0.000102	0.002421	1.390135	2.460078	3.806886	3.084457	1.430203	3.891338	3.901317	2.100922	2.16515	1.981639	2.239124	2.166061	2.668085	1.881043	2.372328
Fyb	1.74232	2.842487	5.282325	0.000102	0.002427	1.335952	2.43149	3.713429	4.126591	2.154676	3.721569	4.458273	1.511505	3.763261	1.142163	2.998871	2.921517	3.794816	1.651284	1.684306
Ppp1r14b	1.630615	4.415242	5.279729	0.000103	0.00243	1.233694	3.595524	4.958296	4.539472	2.389316	4.600852	4.251076	4.682297	4.662608	4.628589	5.150519	4.695321	4.816761	4.191471	4.737232
Abcd2	-0.748715	5.272176	-5.279559	0.000103	0.00243	1.016256	5.599065	5.184101	4.847991	5.791393	4.810643	4.963744	5.347548	5.045558	5.299983	5.294957	5.296745	5.437464	5.479806	5.452898
Atf4	0.783733	7.920551	5.271199	0.000105	0.002463	0.98893	8.14173	8.744569	8.993628	7.936634	8.772172	8.78582	7.310709	7.421389	7.375415	7.445545	7.79004	7.428659	7.562516	7.185835
Myo1c	1.208647	5.125019	5.268595	0.000105	0.002471	1.030136	4.875886	6.270577	5.81976	4.361123	5.719264	5.469427	5.064257	4.899205	4.939446	4.922095	4.650242	5.196126	4.573018	5.037209
Il10rb	1.226327	3.828755	5.26568	0.000106	0.002479	1.168558	3.639931	4.236461	4.501309	2.994284	4.866025	4.472821	3.190506	4.166665	2.95129	4.14048	3.882605	4.492777	2.933966	3.25429
Mapt	-0.694508	9.78671	-5.262681	0.000106	0.002488	1.017127	9.879452	9.362273	9.375452	10.24921	9.208707	9.527448	9.974127	9.884719	9.960405	9.847518	9.822763	9.882039	10.03344	10.00821
Rab13	1.18744	4.693519	5.261696	0.000106	0.002488	1.040636	4.759952	5.857796	5.241285	3.915736	5.563257	5.387335	3.969854	4.572916	4.060584	5.005825	4.650242	4.978233	3.794763	4.017694
Nr4a1	-1.026533	5.743875	-5.259127	0.000107	0.002495	0.959745	6.202158	5.328292	5.133963	6.389448	5.310595	5.316512	6.290117	5.340814	5.764364	6.012651	5.835201	5.995144	5.863867	5.661644
Gpr84	1.25638	3.912927	5.255731	0.000108	0.002506	1.176574	3.334978	4.509329	4.113157	2.908002	4.643738	4.192511	3.094359	4.60868	3.059216	4.51702	4.464162	5.044579	2.954296	3.454224
St3gal4	1.091627	6.406033	5.253718	0.000108	0.002508	0.941735	6.198981	7.553565	7.129076	5.733616	6.924346	6.613373	6.107871	6.190834	6.055107	6.341669	6.234069	6.497937	5.989854	6.133497
RGD1309676	-0.788149	6.870951	-5.25315	0.000108	0.002508	0.933267	7.161974	6.172765	6.27743	7.009989	6.49076	6.252741	6.992875	6.900235	7.160203	7.308128	6.865658	7.116895	7.158189	7.339518
Tbc1d9	-0.953869	6.534386	-5.252269	0.000108	0.002508	0.932284	7.212732	6.082202	6.467572	7.066348	5.936405	6.257791	6.566832	6.437263	6.501179	6.437194	6.974241	6.301659	6.708023	6.5494
Crip1	1.858416	2.849772	5.248804	0.000109	0.002519	1.349988	2.460078	3.339488	3.789185	1.033666	3.76592	3.221545	2.967416	3.059168	2.928701	2.745897	2.415434	2.761217	2.549064	3.106782
Ptprf	0.997564	7.09149	5.242488	0.00011	0.002544	0.915757	6.712622	7.803785	7.806589	6.369407	7.223232	7.316098	6.989167	6.891389	6.98312	6.79168	6.879763	7.238798	7.026966	7.260108
Ampd3	0.889039	6.755772	5.234126	0.000112	0.002579	0.899078	6.82129	7.520675	7.685309	6.332618	7.343145	7.310844	6.368716	6.642892	6.287014	6.473701	6.287488	6.808664	6.276913	6.436715
Gstt3	-0.992929	5.915414	-5.232256	0.000112	0.002579	0.900425	6.698444	5.94261	5.423748	6.465427	5.763686	5.240331	5.658967	5.919009	5.935576	5.83056	5.866333	6.195115	5.790851	6.112039
Nr1h3	1.524786	2.635478	5.232133	0.000112	0.002579	1.266942	2.569081	3.411272	3.365078	1.740915	4.031153	3.751866	1.962529	2.709903	2.107651	2.945148	2.346454	2.315682	1.724133	2.194616
Triobp	0.826542	4.491879	5.229258	0.000113	0.002586	0.993713	4.47136	5.02617	5.181399	4.00988	5.128199	4.889781	4.258161	4.402272	4.245589	4.441427	4.298745	4.575244	3.959716	4.070176
Dagla	-0.7801	8.682028	-5.228642	0.000113	0.002586	0.907167	8.523085	7.507224	7.903904	8.599344	7.688922	8.02786	9.116877	9.329758	9.18674	9.22964	9.010744	9.197097	9.014196	9.217283
C1qb	1.660957	6.854454	5.227187	0.000114	0.002588	0.895879	6.295338	7.998137	7.623287	5.349294	7.516847	6.768239	5.715723	7.7608	6.008745	7.508943	7.212865	8.061905	5.970022	6.188488
Ifit1	6.539161	-3.005543	5.22446	0.000114	0.002596	0.827277	-3.260591	0.973336	2.148929	-3.260591	1.840817	0.056669	-2.558588	-3.260591	-2.604521	-2.618976	-3.260591	-3.260591	-3.260591	-3.260591
Hlx	2.049388	1.860519	5.223633	0.000114	0.002596	1.392195	1.430522	2.452828	3.021133	0.485009	3.339971	2.872384	1.297885	2.227821	0.884903	2.291729	2.468337	2.516041	0.037347	1.260365
Kcnc2	-1.254152	5.502516	-5.221309	0.000115	0.002602	0.908812	5.485452	4.364946	4.608118	6.306717	4.808289	4.80033	5.816264	5.56975	5.761172	5.453434	5.668592	5.737365	6.529836	6.163453
Arhgap22	1.026862	3.412682	5.21927	0.000115	0.002602	1.13652	3.163058	4.240408	3.871277	2.758225	4.035185	3.756629	2.905804	3.292072	2.858731	3.649853	3.661913	3.680242	2.923692	3.133933
Mgst1	1.671682	3.049386	5.2192	0.000115	0.002602	1.230707	2.555898	3.61957	3.679531	1.874319	4.325951	3.775529	2.266943	3.466799	2.430637	3.740952	3.181236	3.78376	1.626159	2.584013
Pabpc4	0.599667	5.463732	5.218126	0.000115	0.002603	0.903099	5.254031	5.779937	5.743341	5.134319	5.78659	5.857613	5.463276	5.338449	5.366624	5.483897	5.282044	5.425065	5.25856	5.354378
Pygb	-0.626918	8.101846	-5.215815	0.000116	0.002609	0.878106	8.431315	7.639394	7.835252	8.226598	7.733653	7.604699	8.300393	8.127967	8.19086	8.165132	8.186442	8.23754	8.442857	8.309589
Il11	3.822041	0.377518	5.212076	0.000117	0.002623	1.383641	-0.518419	1.425638	2.932131	-1.575022	2.712936	2.743931	0.028455	-0.511025	0.385075	-0.311823	0.202178	0.151783	0.037347	-0.748265
Fam81a	-0.78403	7.186429	-5.206449	0.000118	0.002645	0.852759	7.792096	7.112117	7.312905	8.229714	7.012886	7.467603	7.095785	6.758843	7.034271	6.919063	6.929545	6.767164	7.134442	7.054638
Hpse	1.331014	2.582103	5.201856	0.000119	0.002663	1.221589	2.246933	3.61957	3.381979	1.962578	3.523394	3.143674	2.286414	1.958936	2.185932	2.438927	2.228732	2.761217	2.003164	2.685374
Tk1	2.067009	3.121712	5.198928	0.00012	0.002673	1.145887	2.987615	4.744275	3.656563	1.996424	5.032606	4.675081	2.12275	2.650138	2.127623	2.807409	2.949696	3.074167	2.562371	2.530531
Slfn2	1.636349	3.816271	5.193748	0.000121	0.002694	1.072271	3.388015	4.551241	4.990724	2.57996	4.545387	4.261168	3.374605	4.58892	2.846732	4.66726	4.160911	4.862143	2.301782	2.446395
Csf3r	2.15884	3.524665	5.191086	0.000121	0.002694	1.137085	3.109925	4.037603	4.944066	1.780306	4.997903	4.192511	2.266943	4.24955	2.347926	3.940842	3.817832	4.703986	2.494578	2.635584
Btk	1.941978	1.748883	5.19106	0.000121	0.002694	1.297562	1.669604	2.904741	2.962413	0.932396	3.644478	3.22842	-0.169545	1.958936	0.571595	1.28803	1.82436	2.187314	0.745852	1.32462
Gar1	0.87116	3.160461	5.190848	0.000121	0.002694	1.04991	3.073383	4.064569	4.207831	3.348912	4.128728	3.926902	2.361772	2.795149	2.834633	2.911926	2.568656	2.805622	2.783013	2.622863
Fhod1	1.697709	4.724705	5.185235	0.000123	0.002717	0.969809	4.239536	5.633051	4.942168	2.968937	5.56186	4.949252	4.567614	5.102412	4.241012	5.394523	5.176705	5.407164	3.707626	4.323092
Mybbp1a	0.753189	6.83805	5.183389	0.000123	0.002722	0.80178	6.426071	7.337044	7.362713	6.416264	7.037217	6.958572	6.878854	6.923298	6.825452	6.781753	6.607189	6.709906	6.614511	6.867809
Eef1b2	0.734788	6.519985	5.177358	0.000125	0.002748	0.789562	6.57808	7.275384	7.046236	6.549765	7.372126	7.497793	6.234815	6.049074	6.079664	6.265905	6.017092	6.016554	6.00577	6.309524
Nhlh1	-2.72825	2.979075	-5.172553	0.000126	0.002767	1.10746	3.664706	-0.273517	1.269823	4.773515	1.96438	3.121686	3.445603	3.221962	3.525993	3.368956	3.712129	3.323571	3.107385	3.814538
Acpl2	-1.046028	4.739443	-5.171111	0.000126	0.00277	0.88755	5.014852	3.1924	3.883197	4.635342	3.818339	4.178379	5.089416	5.154424	5.149382	5.396526	5.27278	5.257355	5.132643	5.244487
Abhd3	-0.785618	7.158448	-5.165862	0.000127	0.002792	0.770664	7.825095	6.779245	7.012263	7.665309	6.871763	7.178649	7.070738	6.995618	7.053322	7.07914	6.863808	7.317573	7.189613	7.327318
Spry1	1.422226	2.404884	5.163157	0.000128	0.002801	1.099834	2.295333	4.129856	4.23289	2.546081	3.649748	3.334188	1.962529	1.429209	1.663747	2.048482	1.719153	1.618784	1.724133	1.659607
Atcay	-0.722768	7.850007	-5.161676	0.000128	0.002801	0.76601	7.883317	6.921856	7.380343	8.006553	7.200072	7.388861	8.069827	8.20722	8.102483	8.081698	8.198479	8.152528	8.272171	8.041879
Dkk3	-1.104361	9.001419	-5.161305	0.000128	0.002801	0.792119	9.038001	7.456803	8.178973	9.292761	8.269262	8.343006	9.2004	9.448133	9.507724	9.28583	9.357012	9.427671	9.661593	9.55617
Pmf1	2.073813	1.004132	5.158473	0.000129	0.002808	1.308828	0.830061	2.747223	1.874203	0.166125	2.995506	2.410784	0.282279	0.645799	0.628769	0.750369	0.535373	0.34469	0.491415	0.257494
St14	3.180533	2.002602	5.158054	0.000129	0.002808	1.325508	0.785575	3.27168	2.778919	-0.81875	3.196117	2.753495	-0.067151	4.106787	0.17081	3.88487	3.461211	4.718575	0.249639	0.321874
Mtbp	2.462876	0.992386	5.154453	0.00013	0.002822	1.313258	0.692264	2.914655	2.067688	-0.243853	2.630048	2.743931	0.563361	0.871929	0.317129	0.134646	0.998029	0.804622	-0.628995	1.009844
Rnf217	0.800436	3.465738	5.148209	0.000132	0.00285	0.976574	3.205888	3.894655	4.201497	3.145235	3.864394	3.901317	3.318966	3.292072	3.464507	3.293574	3.331578	3.269114	3.286436	3.195366
Slfn3	4.65238	3.12885	5.14497	0.000132	0.00286	1.295402	0.031429	4.472889	5.211652	0.166125	4.493577	4.392459	2.380012	4.782275	1.549949	4.581825	3.66758	5.677395	1.001683	1.80183
MGC114464	-1.772038	3.265999	-5.144337	0.000133	0.00286	0.919654	4.247758	1.532513	2.885483	4.84492	3.293596	3.365711	3.038038	2.964197	3.262563	3.186517	3.50618	3.046681	3.627601	3.106782
Cd86	2.293664	1.419739	5.141682	0.000133	0.00287	1.291318	0.692264	2.279532	2.498042	-0.243853	2.732935	2.101918	-0.399109	2.505864	-0.386063	2.375352	2.213317	2.980434	0.791767	0.554014
Clec7a	6.67747	1.472344	5.139832	0.000134	0.002874	1.222502	-0.899176	4.042133	5.03414	-2.040259	5.190992	4.793392	-1.21312	3.749088	-1.536754	2.614395	3.03984	4.680333	-1.413173	-2.653844
Atp2b2	-0.932094	9.065236	-5.136945	0.000134	0.002885	0.746207	8.825622	7.969889	8.276539	9.474106	8.071174	8.547546	9.646079	9.464762	9.573816	9.374494	9.300235	9.375002	9.454844	9.562429
Snrpa1	0.783152	4.686138	5.135693	0.000135	0.002887	0.794824	4.432216	5.167613	5.289856	4.48252	5.465045	5.032137	4.729295	4.498613	4.433808	4.546183	4.49636	4.245254	4.442376	4.407101
Dnajb13	-0.959642	4.688841	-5.12395	0.000138	0.002946	0.789355	4.791228	3.678943	3.679531	4.91074	3.952316	4.271191	5.249269	4.853564	5.132184	5.133806	5.255952	5.023584	4.772253	5.005709
RGD1305347	-2.170926	0.001066	-5.12039	0.000139	0.00296	1.260288	1.278303	-0.273517	-0.761158	0.705659	-1.235601	-1.612049	0.357848	0.04768	0.091681	0.699359	0.961591	0.666985	0.249639	0.706485
Kpna2	1.313253	1.29799	5.118089	0.000139	0.002968	1.165101	1.341131	2.411416	2.588191	1.405223	2.742832	2.889836	0.625837	0.923327	0.736738	0.799637	0.138317	1.008331	0.491415	0.799858
Cdhr1	-1.440799	1.384211	-5.116393	0.00014	0.002972	1.177768	2.372556	0.895377	0.945338	1.874319	0.210178	0.809818	1.913281	1.564863	1.393785	1.597576	1.803924	1.568131	1.815874	1.260365
Tril	-1.333359	7.422502	-5.114446	0.00014	0.002978	0.673402	8.125647	6.544822	5.815619	7.536794	6.879634	6.762922	7.954367	7.581938	7.565857	7.611229	7.617072	8.059042	7.918317	7.952248
Slc2a9	1.58134	2.975985	5.11317	0.000141	0.00298	1.125657	1.568201	2.904741	3.164784	1.571628	3.378569	3.038068	3.577905	3.104407	3.35864	3.784431	3.20481	3.136336	2.84965	3.246018
Fchsd1	1.051316	3.844029	5.112093	0.000141	0.002981	0.863567	3.595524	4.763603	4.569292	3.275	4.339058	4.234096	3.616879	3.818604	3.620637	3.856048	3.324422	3.794816	3.36447	3.757487
Fads6	-0.870917	4.564819	-5.104264	0.000143	0.00302	0.768876	4.748409	3.99151	3.370733	4.526619	3.952316	3.794184	4.887476	4.82682	5.016447	4.827276	4.840346	5.058407	5.011753	5.127783
Arhgef40	1.080386	6.166498	5.100289	0.000144	0.003037	0.664431	5.771499	6.836428	6.425395	5.038072	6.460344	6.192489	6.194159	6.35776	6.086058	6.434273	6.170637	6.39089	5.756531	6.239378
Ctsc	2.211521	3.93488	5.096345	0.000145	0.003054	0.841116	4.3544	4.914502	5.255111	2.169587	5.840583	5.616876	2.247205	4.234368	2.41447	4.046664	4.348982	4.783844	2.151407	2.858134
Emc8	0.790433	4.255847	5.087713	0.000148	0.003099	0.765346	3.983028	4.735912	4.600917	3.752019	4.803569	4.467019	4.267989	4.095628	4.070936	4.320048	4.404331	4.196062	3.97969	3.988241
Cmtm7	1.570626	3.392955	5.083732	0.000149	0.003117	0.939496	2.917193	3.806886	3.81015	2.029493	4.675086	3.737479	2.717819	4.101219	2.694377	3.973412	4.061116	4.200226	2.379013	2.570827
Kif20b	1.613717	1.485785	5.076464	0.000151	0.003155	1.135358	0.485316	2.200694	2.2133	1.273392	2.575715	2.889836	1.372662	1.239467	1.142163	1.481601	1.03357	1.186803	1.1849	1.122575
Chst15	-1.227057	4.12712	-5.074349	0.000151	0.003162	0.784196	4.040949	2.519311	2.954902	4.433945	2.838232	3.747086	4.510807	4.655026	4.65291	4.581825	4.582868	4.840964	4.808999	4.719417
Pecam1	1.149995	5.070533	5.070872	0.000152	0.003177	0.643037	5.080769	6.18408	6.032733	4.405886	5.856622	5.462154	4.842144	4.655026	4.830952	4.818339	4.755092	4.997502	4.306643	4.809178
Hhip	-1.600742	2.623299	-5.068023	0.000153	0.003189	0.913983	3.230989	1.725091	1.966737	3.696477	1.48759	2.374054	2.814871	2.80888	2.680923	2.06872	2.274011	2.999674	3.627601	3.25429
Eng	1.365858	6.216545	5.065567	0.000154	0.003194	0.59616	5.919085	6.909515	6.582919	4.926188	7.133925	6.490784	6.208264	6.148265	6.196547	6.317559	6.031446	6.51308	5.539897	6.136857
Sele	2.921961	0.371949	5.06529	0.000154	0.003194	1.164642	-0.518419	1.833582	2.412888	0.041976	3.391209	2.623922	-0.279766	-0.048075	-0.504027	-0.961779	-0.731011	-0.070959	-0.628995	0.257494
Cd97	1.881766	4.574316	5.062596	0.000155	0.003205	0.76325	4.105711	5.587296	5.379549	2.656049	5.114924	4.804937	4.855242	4.411301	4.778229	4.712967	3.93523	4.73014	4.156802	4.888375
Ccl3	4.783898	-0.05494	5.060279	0.000155	0.003213	0.984848	-1.052261	1.506527	3.013923	-2.731185	2.530726	1.563674	-2.088409	1.532126	-1.536754	0.981432	1.375754	1.461155	-1.904936	-1.899685
Msantd3	0.647221	3.970162	5.058011	0.000156	0.003222	0.70297	3.973144	4.588877	4.650582	3.902409	4.573386	4.509973	3.609168	3.653399	3.761508	3.814705	3.84309	3.631738	3.614885	3.558103
Nos3	1.85296	4.880199	5.052657	0.000158	0.003249	0.662762	4.474867	6.351267	6.30944	3.617117	5.579915	5.277267	5.170246	4.536242	4.942268	4.712982	3.838074	4.308338	4.262487	5.003257
Rtn4r	-0.987433	6.533182	-5.050294	0.000158	0.003258	0.555108	6.438695	4.869338	5.518508	6.361313	5.655207	5.597091	7.077041	7.049383	7.32062	7.156821	6.852037	7.200857	7.014313	7.373613
Fgd2	1.38994	3.396383	5.046822	0.000159	0.003274	0.906721	2.706665	3.833259	3.376367	1.799605	3.86892	3.365711	2.566765	4.860173	2.47808	4.66726	4.489977	4.775496	2.236876	2.70964
RGD1306058	1.155701	3.357027	5.044684	0.00016	0.003281	0.822681	3.016775	4.091039	3.933744	2.62393	4.113552	3.703341	3.137884	3.492551	3.110295	3.440594	3.013384	3.228918	2.748504	3.503633
Fas	3.053638	1.135296	5.043446	0.00016	0.003284	1.126408	0.873216	2.452828	2.588191	-1.388744	2.575715	2.570814	0.202532	1.934092	-0.080923	1.253084	0.631286	2.153355	0.313987	0.844371
Vars	0.664213	6.600919	5.040673	0.000161	0.003295	0.528678	6.197921	6.933484	6.992681	6.182661	6.754372	6.735434	6.648099	6.454836	6.675802	6.683464	6.425987	6.560818	6.423567	6.760112
Rasgrp4	2.461892	1.653383	5.038248	0.000162	0.003305	1.058022	1.619793	2.864389	3.975174	0.105385	3.181638	2.781814	1.478026	1.318383	1.180909	1.451089	0.846404	0.760186	1.001683	1.193114
Taldo1	0.639488	6.921476	5.033538	0.000163	0.003325	0.512073	6.728131	7.125517	7.301845	6.391039	7.305663	7.060334	6.79807	7.071622	6.813172	6.961623	6.874873	6.982525	6.701273	6.798091
Aen	1.039961	3.348097	5.033264	0.000164	0.003325	0.76654	3.136736	3.904639	4.546985	3.002635	4.002607	3.914167	3.026505	3.081965	3.140097	3.241039	3.235656	3.204252	2.816716	2.780079
Lrg1	3.371733	2.334774	5.031417	0.000164	0.003331	0.945417	2.620642	5.481218	5.416931	0.621404	4.981279	3.698398	0.743216	2.121805	0.931093	1.481601	2.149958	1.375373	0.249639	1.32462
Kars	0.539946	6.840585	5.030072	0.000165	0.003334	0.50468	6.698444	7.129247	7.242159	6.598099	7.253833	7.126236	6.682493	6.68817	6.68088	6.756243	6.838285	6.742393	6.59926	6.746249
Atp2b3	-1.595091	8.249559	-5.026949	0.000165	0.003348	0.521654	8.567281	6.300186	7.534742	9.104824	7.343958	7.785724	8.552633	8.685912	8.806565	8.176251	8.720001	8.395684	8.879654	8.646525
Rap1b	0.902173	4.419037	5.024865	0.000166	0.003354	0.620171	4.012279	4.774532	4.884032	4.098255	5.020453	5.139986	4.272878	4.455617	4.106593	4.046664	4.376922	4.383501	4.495367	3.874793
Stk10	1.039283	4.694468	5.024218	0.000166	0.003354	0.596567	4.460789	5.051103	5.603828	4.030446	5.463549	4.969911	4.301868	4.937208	4.15605	4.772804	4.588853	5.099115	4.116777	4.233886
Cytip	4.253876	-0.008949	5.017902	0.000168	0.003389	0.829837	-2.235785	0.24948	1.65069	-2.344749	1.912714	1.275818	-1.21312	1.628188	-0.632503	1.023513	1.605665	1.805289	-0.893866	-1.212031
Vamp3	0.690215	5.688762	5.013049	0.00017	0.003414	0.491037	5.633986	6.268645	6.058368	5.488098	6.498813	6.173021	5.499182	5.347885	5.399851	5.628953	5.510468	5.492828	5.288894	5.384835
Ddx54	0.79047	5.939778	5.005032	0.000173	0.003461	0.472528	5.823308	6.467796	6.666917	5.432006	6.468568	6.055191	5.695838	5.974888	5.6308	5.901388	5.856444	5.959969	5.640262	5.609402
Stk32b	-2.227742	0.446989	-4.994791	0.000176	0.003523	1.039777	0.785575	-1.271663	-0.575682	1.63746	0.027932	-1.427823	1.091998	0.972957	1.393785	0.847278	1.068256	0.28321	1.651284	1.29285
Eif4ebp1	1.594604	3.534181	4.993004	0.000176	0.003529	0.692076	3.652372	4.796145	4.645925	2.297273	4.664713	4.005051	2.880403	3.330656	2.694377	3.636356	3.331578	3.698019	2.760099	3.237699
Acoxl	6.218987	-0.977446	4.990191	0.000177	0.003542	0.521223	-2.659044	2.801663	2.187895	-3.260591	1.113405	0.881356	-2.558588	0.377517	-2.604521	0.981432	0.764165	0.760186	-2.656794	-2.653844
Ak2	1.069814	3.67283	4.986327	0.000179	0.003562	0.63633	3.589067	4.755351	4.377553	3.083591	4.472915	3.96856	3.496648	3.458111	3.35864	3.417108	3.366837	3.38339	3.262178	3.558103
Acsm5	-3.64032	1.027646	-4.983352	0.00018	0.003577	1.016746	2.875353	-1.463779	-1.367151	1.571628	-0.613814	-1.26448	2.534559	2.724465	1.60797	2.06872	2.995472	2.300247	0.111637	1.846283
Clec10a	2.202448	1.461645	4.981167	0.000181	0.003585	0.93493	1.717752	2.702143	3.049621	0.386344	3.482634	3.402651	0.498056	-0.26106	0.683763	0.893396	1.430105	1.433124	0.921344	0.844371
Cd180	1.459253	4.165011	4.980154	0.000181	0.003586	0.594502	3.810597	4.71337	4.620039	3.182467	5.271819	5.136335	2.760215	4.943446	2.995434	4.673881	4.470659	5.440987	3.262178	3.133933
Mal	-1.667917	7.860904	-4.969401	0.000185	0.003655	0.401173	8.51757	7.422472	6.67494	8.303178	6.987098	5.893742	7.860461	8.219159	7.976687	8.237872	8.114622	8.797386	8.611959	8.443798
Pnp	0.785274	7.022338	4.959632	0.000188	0.003717	0.369785	7.203251	7.856491	7.734667	6.577982	7.572012	7.536741	6.688909	6.705707	6.616087	6.849119	6.787145	6.830319	6.571561	6.795259
Lpar6	1.665992	3.097785	4.955854	0.000189	0.003738	0.702003	3.00712	4.138342	3.728808	1.837445	4.505252	3.817169	2.144252	3.19084	2.00342	3.602051	3.228006	3.668268	1.943396	2.768575
RT1-CE15	1.185323	2.955856	4.954623	0.00019	0.003738	0.705003	2.799078	3.532115	3.89109	2.124384	3.339971	3.683465	2.343298	3.221962	2.430637	3.463704	2.930971	3.361247	2.317563	2.159814
C1r	1.656232	5.487742	4.953827	0.00019	0.003738	0.408844	5.19785	6.292603	6.862248	4.458437	6.118463	6.618616	3.927077	5.83091	4.768722	5.692284	5.132316	6.269325	4.602301	5.098219
Rrm1	0.803735	5.003139	4.953282	0.00019	0.003738	0.420203	4.796842	5.3793	5.210076	4.661948	5.854341	5.677165	4.848708	4.853564	4.762349	4.760414	4.842853	4.781067	4.82012	4.845219
Slc24a6	1.404715	3.745786	4.950347	0.000191	0.003752	0.650217	3.205888	4.21255	4.263615	2.255954	4.209453	3.718071	3.504983	4.193082	3.383769	4.164187	3.858035	4.496167	3.294432	3.808933
Dpf3	-1.28608	2.610636	-4.949486	0.000192	0.003752	0.675519	3.016775	1.79116	2.379614	3.752019	1.78444	2.515676	2.787802	2.939445	2.785194	2.558244	2.684976	2.2368	2.902922	2.685374
Adck4	1.140543	6.268412	4.946541	0.000193	0.003761	0.355404	5.875975	7.423341	6.740844	5.567645	6.655678	6.618616	6.362996	5.958043	6.228108	6.36436	5.92665	5.970977	5.772362	6.313488
Rpl18	1.178763	6.659847	4.946433	0.000193	0.003761	0.348762	6.550097	7.463572	7.034258	5.564828	7.560167	6.868469	6.385741	6.658143	6.456478	6.88879	6.662152	6.566474	6.197056	6.398042
Itgb7	4.402268	-0.411699	4.945248	0.000193	0.003761	0.89307	-0.899176	1.747452	3.426105	-2.040259	2.411703	2.349037	-1.21312	0.04768	-1.536754	-1.838237	-1.500575	-1.073407	-1.904936	-0.397871
Cttnbp2nl	0.9485	5.985663	4.944838	0.000193	0.003761	0.345716	5.88127	6.796103	7.23791	6.11687	6.703337	7.053575	5.000844	5.847636	5.22317	5.535706	5.660089	6.186745	5.435298	5.147901
Osbpl9	0.693877	6.420397	4.943896	0.000194	0.003762	0.340651	6.212697	6.965431	6.752263	6.235874	6.942709	7.009851	6.401452	6.246128	6.18468	6.377603	6.175617	6.228116	5.894439	6.277416
RGD1559896	-0.916717	7.551153	-4.941896	0.000195	0.00377	0.340143	8.311166	7.115344	6.825652	7.508485	7.136274	6.90433	7.570512	7.691151	7.546051	7.94023	7.737129	7.897902	7.784558	7.756144
Hace1	-0.688975	5.837395	-4.938661	0.000196	0.003788	0.34946	5.858632	5.064523	5.355505	5.94228	5.074352	5.349996	6.328191	6.252422	6.243062	5.986294	6.134267	5.90618	6.171234	6.085628
Lpl	-1.465017	6.085396	-4.936042	0.000197	0.0038	0.338498	6.266292	4.496185	4.72312	6.876635	5.446997	5.762532	6.352643	6.212949	6.506909	6.061439	6.550814	6.315161	6.937249	6.713627
Irf1	1.394	4.012293	4.933413	0.000198	0.003813	0.525852	3.601952	4.859106	5.32815	3.099251	4.513947	4.192511	3.347054	4.166665	3.472337	4.126066	4.13251	4.496167	3.508915	3.432519
Klf4	1.185828	3.204033	4.932453	0.000198	0.003814	0.587736	3.118917	4.240408	4.275724	2.77782	3.986037	3.96856	3.601415	2.488905	2.894141	2.770817	2.031907	2.921125	2.994118	2.972849
Rpl11	0.841655	6.975736	4.924823	0.000201	0.003864	0.3028	7.24338	7.920006	7.546034	6.661443	7.997138	7.707922	6.561847	6.581224	6.489651	6.802295	6.689565	6.50805	6.350666	6.6143
Pa2g4	0.702473	6.957544	4.92204	0.000202	0.003878	0.298248	7.022181	7.583393	7.862855	7.290073	7.988066	8.000508	6.238569	6.595158	6.556557	6.439139	6.52919	6.47148	6.42083	6.421186
Aldh1l2	2.092656	1.479709	4.919346	0.000203	0.003889	0.875635	-0.310971	2.33964	2.508341	1.273392	2.373993	2.924117	0.952703	1.658838	1.180909	1.180545	1.03357	1.989793	1.001683	1.260365
Irf8	1.517808	3.766593	4.918894	0.000203	0.003889	0.584365	2.987615	3.838476	4.31439	2.511387	4.377679	4.422747	2.955303	5.077105	2.330799	4.630291	4.709125	5.521398	2.480628	2.721621
Kcns3	-1.144331	3.454153	-4.914695	0.000205	0.00391	0.510516	3.838085	2.151237	2.718102	4.082584	3.346477	3.061358	3.742028	3.413873	3.774234	3.552611	3.352837	3.668268	3.822677	3.988241

Ada	1.677946	1.463305	4.91439	0.000205	0.00391	0.800762	1.693879	2.758277	2.663852	0.705659	2.987204	2.845805	0.429655	1.279465	0.091681	1.652245	1.167576	1.152831	0.182287	1.556352
Mob1a	0.748018	4.741028	4.906338	0.000208	0.003964	0.333005	4.683216	5.263826	5.408707	4.693241	5.575768	5.486734	4.301868	4.666384	3.985965	4.390078	4.464162	4.827568	4.322371	4.365708
Spdl1	1.469209	1.761689	4.905161	0.000209	0.003964	0.801851	1.278303	2.747223	2.122353	0.860705	2.542105	2.602913	1.335758	1.464337	1.843557	1.920699	1.605665	2.064785	1.347449	1.415936
Kcnj11	-0.74895	5.097628	-4.904751	0.000209	0.003964	0.325468	5.187202	4.771807	4.308508	5.244047	4.405982	4.410708	5.415442	5.119041	5.334762	5.557352	5.27278	5.419718	5.460392	5.507551
Akap12	1.389666	6.165671	4.891781	0.000214	0.004057	0.242059	5.959694	7.623992	8.352642	6.538322	7.069523	7.515467	5.388667	5.057109	5.462129	5.445717	4.932657	5.621048	5.587858	5.790939
Crem	0.783482	3.819102	4.890103	0.000215	0.004064	0.406258	3.695089	4.532048	4.337679	3.701615	4.656883	4.395516	3.790562	3.301815	3.432752	3.663225	3.639021	3.346294	3.621257	3.468514
Tnfaip1	0.559787	6.354734	4.888842	0.000215	0.004065	0.233103	6.383796	6.875657	6.85873	6.11687	6.730324	6.771773	6.105133	6.144209	6.071954	6.118373	6.264302	6.225054	6.192784	6.126755
Ripk2	0.636352	4.743221	4.88675	0.000216	0.004065	0.308578	4.559869	5.218524	5.157058	4.53528	5.161783	5.19191	4.641297	4.803005	4.51566	4.782026	4.561722	4.656286	4.258405	4.421864
St18	-1.445268	4.600491	-4.886744	0.000216	0.004065	0.349749	4.481855	2.874583	3.776458	5.4149	4.176973	3.193712	5.146469	5.204627	5.304377	4.913728	4.72823	4.864769	5.225485	5.178649
Phlpp1	-0.652059	7.248828	-4.886718	0.000216	0.004065	0.227648	7.417472	6.835124	6.735922	7.615132	6.986057	6.899492	7.378051	7.389191	7.258577	7.209609	7.308905	7.563661	7.542932	7.354023
Trip6	1.492221	4.542381	4.881634	0.000218	0.004098	0.433032	3.589067	5.307749	4.583975	2.985885	4.627805	4.537915	4.861747	4.995405	4.614504	5.124169	4.594813	5.030616	4.028447	4.786544
Sec14l4	-2.087938	1.48617	-4.8784	0.00022	0.004103	0.755096	2.63325	-0.572649	1.705429	1.996424	-0.038319	-0.072565	2.266943	2.121805	2.066858	1.852296	2.181985	1.848377	1.651284	1.910497
Cd8a	4.132796	-1.310261	4.878209	0.00022	0.004103	0.726543	-1.642648	1.369077	1.169557	-2.040259	2.762423	2.00915	-2.558588	-1.435005	-3.260591	-0.537962	-0.991788	-0.920914	-3.260591	-1.899685
Apobr	1.04839	3.27678	4.877904	0.00022	0.004103	0.486187	3.035892	3.785434	4.254466	2.862845	4.169655	3.713177	2.841442	3.395791	2.759824	2.988286	3.394437	3.594258	2.614401	2.635584
LOC681325	5.716878	-2.277597	4.87751	0.00022	0.004103	0.355724	-2.659044	0.118359	2.175023	-3.260591	1.173177	1.327961	-2.558588	-2.076466	-3.260591	-2.618976	-1.310338	-1.925331	-3.260591	-2.653844
Cst7	5.366441	-2.669115	4.876255	0.000221	0.004103	0.424939	-1.908947	1.117652	2.298852	-3.260591	1.390206	0.846031	-3.260591	-1.720374	-2.604521	-3.260591	-3.260591	-2.668207	-3.260591	-3.260591
Map4k1	1.331832	3.183883	4.876139	0.000221	0.004103	0.538475	2.8647	4.155166	3.8018	2.213416	4.035185	3.402651	2.534559	3.94833	2.223535	3.48645	3.434871	3.906064	2.285827	2.474989
Spock2	-1.392256	7.915509	-4.873906	0.000222	0.004114	0.215413	8.158725	6.656857	7.476263	8.653051	6.399083	7.520728	8.082065	8.214008	8.495616	7.834128	8.1154	8.148224	8.656069	8.414342
Gjb6	-1.185298	5.18652	-4.869524	0.000223	0.004142	0.227196	6.218984	5.101882	4.156367	5.573263	4.899589	4.717236	5.331236	4.677653	5.351842	5.181051	4.944375	5.292883	5.563249	5.647583
Ciita	2.875602	0.432339	4.866978	0.000224	0.004156	0.807374	-0.410969	1.010791	1.966737	-1.223791	1.997824	2.297664	-0.831021	0.871929	-0.632503	1.064403	1.533025	1.97042	-1.047095	-0.886923
Limd1	1.094999	3.753033	4.861199	0.000227	0.004196	0.374417	3.891537	4.643552	4.720907	3.059778	4.656883	4.164107	3.36548	3.221962	3.367065	3.51623	3.525031	3.750073	3.394539	3.388105
Mefv	2.65321	0.820185	4.857573	0.000229	0.004219	0.79716	-0.046857	2.21681	2.322392	-1.075778	1.96438	1.107027	-0.067151	1.983359	0.091681	1.451089	1.533025	1.848377	-0.040977	-0.621773
Dhcr24	-1.02016	5.70866	-4.855519	0.000229	0.004224	0.191397	5.930623	4.390036	5.086634	6.046925	5.264968	5.121639	6.169466	5.722645	6.258994	5.422315	5.696576	5.785273	6.680829	6.377363
Unc93b1	1.330457	4.710187	4.855271	0.00023	0.004224	0.328207	4.078311	4.699101	4.996223	3.315784	5.323842	4.996333	3.51327	6.138783	3.675936	5.637447	5.640054	6.346495	3.627601	4.027379
Fgf12	-0.878811	4.742582	-4.854096	0.00023	0.004227	0.213947	5.35833	4.283127	4.648255	5.553504	4.735811	4.641992	4.510807	4.596856	4.62508	4.390078	4.847853	4.542821	5.123695	4.597931
Shmt1	1.320321	2.281376	4.852776	0.000231	0.004227	0.627414	1.693879	2.943995	3.151703	1.658753	3.036313	2.753495	2.415815	2.454377	2.313467	1.920699	2.481265	2.046399	1.724133	1.684306
Ppp1r15a	1.024255	5.238618	4.852525	0.000231	0.004227	0.197813	5.239674	6.018122	6.7759	4.996864	5.908101	5.848717	4.739927	4.870031	4.614504	5.013676	5.041404	5.049203	4.677602	4.591416
Apbb1ip	0.834166	4.828808	4.850029	0.000232	0.004241	0.224353	4.785591	5.36852	5.449365	4.53528	5.700344	5.438632	4.102096	5.196815	3.947156	4.879766	4.791878	5.374373	3.91892	4.174106
Cxcl16	2.457069	4.191148	4.837642	0.000237	0.004332	0.341841	4.210384	4.607333	6.012977	2.297273	6.109886	5.858722	2.943086	4.60868	2.446625	4.2553	4.80736	5.275228	2.771602	2.59708
Irak4	0.886907	2.971523	4.837435	0.000238	0.004332	0.477256	2.810225	3.34683	3.324853	2.269859	3.352952	3.59047	2.659281	3.232189	2.625819	3.080873	3.116419	3.212521	2.549064	2.635584
Ddc	-1.925799	2.828384	-4.833639	0.000239	0.004357	0.535281	3.529616	1.506527	0.20995	2.87199	1.895073	1.708392	3.727855	3.301815	3.606474	3.753509	2.802949	3.454835	3.508915	4.041787
Ahnak	1.391311	6.100427	4.830168	0.000241	0.004379	0.145515	5.488925	7.06226	6.564529	5.046174	6.20781	6.780571	6.460498	6.207776	6.011435	6.180138	5.655819	6.252381	5.368694	6.143552
Plxdc1	-0.953141	5.011722	-4.823972	0.000244	0.004425	0.188327	4.796842	3.854016	4.207831	5.341085	4.513947	3.905613	5.328891	5.35258	5.648188	5.24692	5.393952	5.518066	5.44431	5.666301
Serinc5	-1.004729	7.384046	-4.818338	0.000246	0.004466	0.095059	7.437378	6.686092	6.118433	7.712527	6.819531	6.658615	7.583359	7.882662	7.49939	7.621969	7.505618	8.055886	7.823416	7.982125
Frem3	-2.921554	2.605535	-4.814673	0.000248	0.004491	0.521933	3.365524	-1.685466	1.841991	4.109897	1.338932	1.976852	3.241276	3.169712	3.307029	2.854798	3.380703	3.083214	3.913738	3.220915
Egr4	-1.266777	3.878373	-4.813536	0.000249	0.004494	0.308386	4.235407	2.100024	3.070623	3.915736	3.060255	2.949304	4.714996	3.942114	4.429792	4.464524	4.570823	4.331301	4.509176	4.129836
Cyth3	-0.730404	5.666672	-4.794015	0.000258	0.004659	0.066733	5.791322	4.909553	5.382351	6.074915	5.158089	5.357855	5.814589	5.988526	5.82983	5.741966	5.874931	5.782499	5.978732	5.680182
Serpinb8	4.803085	-1.766668	4.791899	0.000259	0.004671	0.468737	-1.908947	0.679782	1.966737	-2.731185	1.510943	2.469996	-2.088409	-2.550306	-2.155202	-2.618976	-1.500575	-1.660823	-2.656794	-3.260591
Nxt1	0.940239	2.738564	4.789968	0.00026	0.004681	0.344019	2.832262	3.52566	3.495051	2.748327	3.917788	3.94371	2.380012	2.14364	2.185932	2.14695	2.556491	2.346065	2.301782	2.068949
Spry3	-0.799424	2.433288	-4.787802	0.000261	0.004694	0.33102	3.342675	2.383133	2.528721	3.41297	2.619344	2.809587	2.286414	2.326577	2.260183	1.964563	2.166061	1.910689	2.285827	2.068949
Nrep	-0.912463	3.062715	-4.785671	0.000262	0.004706	0.300219	3.753999	3.117121	2.445413	3.425447	2.530726	2.675144	3.148563	3.211662	3.159628	3.090803	3.280721	3.195935	3.178239	2.858134
Gmip	1.019203	4.740494	4.780941	0.000265	0.004734	0.138271	4.10118	5.364909	5.191021	4.125276	4.923716	5.043877	4.53949	5.033913	4.60385	4.862481	4.543347	5.110222	4.11226	4.872343
Trpm3	-1.443202	5.490275	-4.7806	0.000265	0.004734	0.04153	6.744929	4.848802	5.355505	5.97129	4.424547	5.020301	5.465413	5.405502	5.475827	5.416404	6.361024	5.239258	5.75073	5.422024
Slc11a1	2.346783	2.923744	4.780292	0.000265	0.004734	0.457633	1.594228	3.97739	5.072821	1.910273	3.649748	3.57437	2.534559	2.695192	2.508863	2.438927	3.189137	3.33876	2.285827	2.402413
Aldh1a1	-1.257187	4.943713	-4.778973	0.000266	0.004737	0.079615	5.375315	3.60127	4.598509	5.598275	4.006719	4.68261	5.599886	4.958923	5.20918	5.463023	5.11997	5.179349	4.913935	4.960925
Efemp2	1.354439	5.430376	4.778075	0.000266	0.004737	0.086986	4.920286	6.403245	5.810426	4.185201	5.824363	5.547098	5.615366	5.528853	5.458191	5.780822	5.314913	5.346466	4.772253	5.55687
RGD1310110	-1.237125	4.912729	-4.777685	0.000266	0.004737	0.091653	5.325689	3.24835	4.082466	5.270223	4.5954	4.287741	5.172864	4.911984	5.271094	5.09979	5.109599	5.27917	5.694433	5.488394
Sec61a1	0.66464	6.809301	4.775452	0.000268	0.00475	0.011949	6.339284	6.817398	6.92702	6.055981	6.956063	6.743855	6.832419	6.946775	6.88313	7.066615	6.877931	7.193043	6.675481	7.029556
Ggn	1.466106	1.113987	4.769513	0.000271	0.004797	0.552648	0.785575	2.607522	2.820842	1.273392	2.062471	2.386402	0.625837	0.645799	0.449965	1.180545	0.484915	0.460309	0.249639	0.383504
Fancd2	0.998269	3.189213	4.766418	0.000272	0.004806	0.291542	2.787845	4.171795	3.505372	2.853641	3.741449	3.870884	3.159163	2.901501	3.027678	3.129854	3.065821	2.838051	2.713149	3.060363
Rit2	-1.06353	6.007383	-4.76616	0.000272	0.004806	-0.006243	6.483771	5.298313	5.68211	7.165444	6.021185	6.042569	5.88812	5.724457	5.982939	5.743541	6.149533	5.859553	6.221376	5.865727
Tmprss6	-1.761942	0.391539	-4.766055	0.000272	0.004806	0.630899	1.430522	-0.813242	-0.411358	0.531921	-0.831496	-0.646023	1.443753	0.449407	0.97585	1.14286	0.676952	0.666985	1.1849	1.085968
Dscaml1	-0.838568	7.30381	-4.764958	0.000273	0.004806	-0.01103	7.233581	6.151961	6.756049	7.668265	6.709658	6.828304	7.737644	7.650742	7.689834	7.538721	7.410401	7.552304	7.639474	7.697067
Akna	0.853879	5.075076	4.764104	0.000274	0.004806	0.047459	5.108122	5.778581	5.580833	4.440107	5.678596	5.440113	4.743453	5.170467	4.504248	5.021485	5.168735	5.321836	4.391098	4.751911
Gprc5a	2.464713	-0.207902	4.762705	0.000274	0.004806	0.629265	-0.217457	1.397635	2.067688	-0.941548	1.877213	1.450626	-0.067151	-2.076466	-0.773548	-0.806258	-0.231835	-0.541385	-1.218554	-0.505482
Prr5	1.210145	3.718552	4.762222	0.000275	0.004806	0.151122	4.167689	5.060063	5.128966	3.197095	4.829341	4.449473	3.562015	2.888628	3.006262	2.977621	2.977336	3.212521	3.212403	3.524301
Cstb	0.869493	5.973226	4.761786	0.000275	0.004806	-0.013947	6.29831	6.570917	7.270421	5.983999	7.105923	7.090835	5.54638	5.499519	5.464094	5.418377	5.280196	5.580094	5.132643	5.410968
Mcm10	3.093361	0.712407	4.761744	0.000275	0.004806	0.627552	-0.518419	2.411416	1.554575	-0.502853	2.865664	2.965855	1.000645	0.301855	-0.504027	0.534523	-0.231835	0.618025	0.249639	0.442608
Gpr65	5.166951	-0.947602	4.757122	0.000277	0.004837	0.251199	-1.908947	1.047298	0.604933	-3.260591	1.312595	2.04074	-2.558588	0.58326	-2.604521	-0.116374	-0.149817	1.045835	-1.218554	-2.228037
Esrrg	-1.415575	3.011241	-4.756881	0.000277	0.004837	0.181452	3.94308	2.279532	2.877559	4.580611	2.692656	3.563537	2.266943	3.159031	2.834633	2.694725	3.065821	2.581351	3.310292	2.516844
Tmem218	-0.691849	4.053462	-4.751032	0.00028	0.004885	0.124546	4.421353	3.557647	3.500221	4.042646	3.523394	3.57437	4.238302	4.198308	4.106593	4.509637	4.33124	4.405307	4.246089	4.19144
Cass4	2.65797	-0.106405	4.747367	0.000282	0.004913	0.564998	-0.518419	1.184734	1.220561	-2.040259	1.143601	0.527154	-1.009457	0.301855	-0.773548	0.134646	0.321999	0.930264	-0.628995	-0.204094
RGD1565785	2.537968	0.203276	4.734301	0.00029	0.005026	0.568759	-0.310971	1.151583	1.340699	-1.575022	1.96438	0.881356	-0.672244	0.972957	-0.773548	1.064403	1.260496	1.542121	-0.755361	-1.406952
Anxa3	0.968002	6.866249	4.734016	0.00029	0.005026	-0.069748	6.669664	7.536813	7.012263	6.001814	7.372525	7.282221	6.360702	7.375992	6.082225	7.343858	7.193356	7.76155	5.965021	6.184165
Pde5a	-0.919705	3.249787	-4.729443	0.000292	0.005063	0.18216	3.488576	2.383133	2.954902	3.819751	2.60856	3.006419	3.790562	3.358932	3.563133	3.12019	3.116419	3.269114	3.743115	3.447025
Ttpa	-1.343772	1.389216	-4.726041	0.000294	0.005086	0.411564	2.63325	1.310208	0.883381	2.061822	0.562819	1.378286	1.408645	1.020936	1.393785	1.322149	1.403186	1.433124	2.003164	1.260365
Flrt1	-1.033172	4.731666	-4.725459	0.000295	0.005086	-0.023957	4.970829	4.361326	4.293698	5.712135	4.070978	4.543439	4.633716	4.411301	5.021793	4.564114	4.561722	4.978233	5.198041	4.983491
Ephx4	-0.686506	4.375267	-4.724873	0.000295	0.005086	-0.010281	4.983193	4.244344	4.385956	5.00935	4.098216	4.512792	4.068749	4.084383	4.321248	4.246444	4.473897	4.368779	4.290742	4.242228
Ifngr1	0.855914	6.423288	4.722076	0.000297	0.005107	-0.083185	5.977107	6.535215	6.858227	5.471649	6.55536	6.356803	5.950476	7.055155	6.131274	6.952803	6.728037	7.271321	5.966272	6.135738
Batf3	3.931003	-0.73865	4.720967	0.000297	0.005107	0.169127	-1.908947	-0.273517	0.158563	-3.260591	0.027932	0.336685	-1.73438	1.393205	-1.10526	1.451089	1.068256	1.461155	-0.893866	-2.228037
Olr1	3.249397	2.58104	4.720555	0.000297	0.005107	0.46314	0.592497	3.08236	4.092769	1.033666	4.306064	4.521215	0.68572	3.683523	0.736738	3.51623	3.42821	4.880423	0.791767	1.227132
Zfhx2	-0.80656	6.639849	-4.718718	0.000299	0.005118	-0.102551	6.553417	6.140391	6.046049	7.314596	6.09064	6.236634	6.870009	6.890582	6.958242	6.680179	6.618204	6.634212	7.066609	6.874532
Glipr1	1.107854	2.863499	4.717473	0.000299	0.005123	0.193236	2.595092	3.570246	4.036949	3.051752	4.223657	3.879645	2.361772	2.539194	1.937064	2.325762	2.912	2.960934	2.003164	1.931282
Efcab1	-1.775902	2.377395	-4.715025	0.000301	0.005136	0.231511	4.026686	0.812964	1.669168	3.211576	2.424057	2.422822	2.186319	2.709903	2.066858	2.06872	2.388236	2.555581	3.070609	2.011549
Esyt1	1.147104	5.235573	4.714102	0.000301	0.005136	-0.034948	4.716181	6.02042	5.573494	4.348075	5.710466	5.390404	5.393164	5.237996	5.239322	5.489538	4.92558	5.440987	4.582846	5.273263
Psme2	1.287075	2.704896	4.713985	0.000301	0.005136	0.272411	2.776524	3.595119	3.447672	1.740915	3.434596	3.541623	2.613764	2.436799	2.364852	2.438927	2.243983	2.680061	1.963595	2.847239
Rps5	1.078447	6.765709	4.707342	0.000305	0.005168	-0.120985	6.680334	7.670078	7.121548	5.774423	7.460614	6.955986	6.669575	6.635206	6.538022	6.912779	6.679784	6.656356	6.356406	6.623908
Runx3	5.469395	-2.363638	4.706344	0.000306	0.005168	0.18207	-1.908947	0.533043	2.135702	-3.260591	2.046579	1.302125	-3.260591	-3.260591	-3.260591	-2.176425	-1.978322	-1.660823	-2.656794	-3.260591
Kazn	-0.515107	6.094542	-4.706248	0.000306	0.005168	-0.125621	6.552588	6.053325	6.104923	6.677831	6.1532	6.091467	5.821279	6.081959	5.820657	6.004794	6.102197	5.97827	6.02512	5.879161
Slamf8	2.018582	1.854798	4.706117	0.000306	0.005168	0.405652	1.48718	3.134193	3.728808	0.860705	2.856578	2.781814	0.798508	1.775313	0.628769	1.569444	2.014226	2.931181	0.836265	1.085968
Rfc4	0.895286	3.300523	4.706046	0.000306	0.005168	0.148895	3.09177	3.939046	3.619037	2.916866	3.969275	4.044542	3.479833	2.875639	3.307029	3.204921	2.90242	2.715403	3.051863	3.25429
Tmod3	0.949417	4.791164	4.70574	0.000306	0.005168	-0.0734	5.017259	5.326436	5.71583	4.685481	5.965222	6.167666	4.263083	4.229271	4.36817	4.361652	4.021838	4.304475	4.20426	4.5074
Lrtm2	-1.159541	5.235872	-4.704751	0.000307	0.005168	-0.051123	4.870572	3.774588	4.501309	5.626918	3.832311	4.251076	5.560444	5.791689	5.967785	5.912629	5.441032	5.913807	5.851726	6.056361
Trip10	1.04337	4.564401	4.703846	0.000307	0.005168	-0.033531	4.59574	5.771779	5.20058	4.026356	5.198203	5.214594	4.311404	4.166665	4.222559	4.571225	4.316888	4.265266	3.73726	4.373323
Zfp57	-0.687286	4.514192	-4.7035	0.000307	0.005168	-0.035562	4.64948	4.279295	4.123244	4.971562	3.969275	4.153309	4.531353	4.612599	4.693673	4.429738	4.606661	4.698109	4.858381	4.692276
Eif3d	0.56033	6.499319	4.703494	0.000307	0.005168	-0.132027	6.540089	6.829894	7.15921	6.390244	7.052715	7.058406	6.35495	6.233457	6.303568	6.280078	6.231202	6.190936	6.193853	6.189567
Gucy1b3	-0.711682	6.357603	-4.701222	0.000309	0.005183	-0.135416	6.453878	5.773142	6.113381	6.967959	6.032323	6.08011	6.51516	6.36823	6.420502	6.314384	6.510393	6.425092	6.64688	6.40456
Ccl2	8.676118	0.863211	4.699596	0.00031	0.005184	0.430151	-1.908947	5.459372	7.497421	-2.040259	6.231725	5.431202	-0.831021	1.564863	-1.304935	-0.806258	-0.510232	1.345607	-1.904936	-2.653844
Glra1	3.648468	-2.081	4.699205	0.00031	0.005184	0.416472	-1.417928	0.854759	1.317458	-2.344749	0.952152	1.785004	-1.73438	-3.260591	-3.260591	-2.176425	-2.697527	-3.260591	-2.656794	-1.212031
Cyp4f4	-1.809506	2.68058	-4.698984	0.00031	0.005184	0.2779	3.543041	2.619695	0.883381	2.708042	1.113405	0.881356	3.392685	3.024282	3.424703	3.440594	2.986432	3.220743	2.974345	3.642453
Fli1	0.985875	3.477813	4.6948	0.000313	0.005219	0.057971	3.536344	4.150978	4.477916	3.342347	4.384017	4.634248	2.550752	3.884925	2.772565	3.080873	3.116419	3.56872	2.575555	2.768575
Ahcyl1	-0.621279	8.735402	-4.689988	0.000316	0.00526	-0.113504	9.309089	8.766119	8.744832	9.43617	8.620073	8.872723	8.727975	8.268126	8.533068	8.432722	8.545192	8.534075	8.818585	8.690622
Plaur	5.072278	0.025298	4.689238	0.000316	0.005261	0.456727	-1.642648	3.563961	3.545931	-1.575022	3.174343	2.05628	-3.260591	0.58326	-1.536754	-0.027758	0.138317	1.691577	-1.638202	-0.886923
Ryr2	-1.279791	8.941913	-4.687448	0.000317	0.005272	-0.138161	8.228155	7.349889	7.677629	9.539239	7.463235	7.908793	10.0369	9.698233	9.886325	9.406154	9.461419	9.264762	9.724257	9.545889
Crispld2	1.545062	3.971181	4.682878	0.00032	0.005311	0.075473	3.683012	4.664267	5.107108	2.499634	4.565043	4.044542	3.488265	3.960684	3.669138	4.205901	4.168924	4.433879	3.3415	3.874793
Xdh	1.07321	4.133314	4.681968	0.000321	0.005313	-0.074499	4.15463	5.372123	5.676412	4.4153	5.181928	5.200677	3.863678	2.951874	4.029076	3.595091	3.196995	3.70975	3.125428	3.496677
Pik3ap1	1.936271	2.477912	4.679372	0.000322	0.005331	0.280954	2.542593	3.091129	3.693138	0.860705	3.76592	3.637716	1.697535	2.80888	1.458292	2.454393	2.346454	3.412394	1.815874	1.445134
Dpyd	-1.399588	3.502932	-4.678082	0.000323	0.005331	0.037472	3.963192	2.046927	2.812554	4.412169	2.874693	3.426763	3.769961	3.42283	3.866259	3.186517	3.906764	3.35379	4.424271	3.728093
Ank1	-0.715803	5.361667	-4.678026	0.000323	0.005331	-0.16272	5.796936	5.515823	5.170091	6.126454	5.038644	5.277267	5.417651	4.90241	5.38748	5.266765	5.078034	5.227065	5.543256	5.354378
Axl	1.323841	6.018463	4.677134	0.000324	0.005331	-0.152025	5.764353	6.558324	6.670934	4.664582	6.3786	6.500046	5.390917	6.645764	5.15669	6.331236	6.229288	7.212241	5.227574	5.555197
Ccl6	4.225348	-0.39275	4.676716	0.000324	0.005331	0.411989	-1.052261	1.339943	2.607481	-2.344749	2.619344	1.378286	-2.088409	-0.511025	-0.504027	-1.33445	-0.149817	-0.334482	-1.413173	-0.621773
Tyw1	0.819766	4.391701	4.673695	0.000326	0.00535	-0.05966	4.082914	4.864231	4.773092	3.997398	5.006144	4.781755	4.051782	4.511265	4.106593	4.520698	3.930525	4.509652	4.089463	4.33484
Prtg	-1.813981	0.186667	-4.673428	0.000326	0.00535	0.460339	1.401336	-0.950476	-0.575682	0.74601	0.027932	-0.984601	1.047044	0.517885	0.571595	0.348395	0.202178	-0.334482	1.11438	1.048408
Npr3	-3.164399	0.364403	-4.66983	0.000328	0.00538	0.415716	2.658139	-0.572649	0.353965	2.213416	-2.73597	1.249022	0.68572	0.705741	0.007959	0.475109	1.068256	0.714338	0.546019	-0.116166
Omg	-1.027875	7.758608	-4.667371	0.00033	0.005398	-0.185907	8.473116	7.08932	7.336941	8.536748	7.886547	7.597747	7.334872	7.696239	7.657143	7.596494	7.546556	7.935853	8.016755	7.923684
Csrp3	4.979236	-3.554922	4.664239	0.000332	0.005424	0.019754	-3.260591	-0.572649	-0.974047	-3.260591	1.338932	-0.457681	-3.260591	-2.550306	-3.260591	-2.618976	-3.260591	-3.260591	-3.260591	-3.260591
Iqgap1	0.778769	5.940555	4.66258	0.000333	0.00543	-0.202903	5.991867	6.579511	6.661159	5.639022	6.461094	6.664964	5.973182	5.667191	5.911298	5.466841	5.28389	5.689294	5.44431	5.760774
Pola2	1.308391	2.524666	4.662263	0.000333	0.00543	0.181928	2.607924	3.769134	3.104962	1.780306	3.670635	3.359461	2.266943	1.830204	1.794428	2.454393	2.317912	2.330954	2.041677	2.310209
Ccdc85a	-1.56542	3.242344	-4.660373	0.000334	0.005443	0.089969	3.064101	1.480065	2.10888	4.324952	2.411703	2.634313	3.601415	3.741948	3.947156	3.734632	3.838074	3.536147	3.772036	3.395603
Galntl6	-1.321392	2.973497	-4.65944	0.000335	0.005445	0.122785	2.987615	1.632013	2.08155	3.732068	2.124346	2.410784	3.072094	3.301815	3.383769	2.866406	3.338699	3.475597	3.743115	3.704139
Csk	0.632981	5.712152	4.657865	0.000336	0.005454	-0.205236	5.71776	6.159277	6.09812	5.521898	6.317002	6.42871	5.406572	5.449994	5.304377	5.687384	5.537023	5.799066	5.314678	5.258947
Sstr2	-1.386175	4.0388	-4.655994	0.000337	0.005464	-0.052767	4.158996	3.125682	2.528721	4.849557	3.280067	3.648009	3.850657	4.327931	4.461611	4.429738	4.136602	4.789383	4.409621	4.655275
Hn1l	0.990392	3.980335	4.655041	0.000338	0.005464	-0.047909	3.92781	4.616473	4.629506	3.342347	4.895159	4.294309	3.850657	3.784265	3.424703	3.721909	3.776475	4.046822	3.569478	3.948012
Cdc42bpg	-0.840333	4.260759	-4.654198	0.000338	0.005464	-0.089086	4.47136	3.69053	3.814306	4.503267	3.488528	3.606391	4.633716	4.468651	4.492744	4.653926	4.168924	4.772703	4.282726	4.689229
Ppargc1a	-1.104448	4.212263	-4.654188	0.000338	0.005464	-0.142915	4.810783	3.833259	4.380359	5.451963	3.886882	4.017012	4.118484	4.095628	4.116621	3.552611	4.316888	3.931333	4.631001	3.917086
Slc14a1	1.276969	4.549247	4.651154	0.00034	0.005489	-0.107581	4.406741	5.202427	5.521053	3.600707	4.970791	5.34526	3.824257	4.624294	3.682701	4.505931	4.444493	5.333256	4.258405	4.041787
Sept5	-0.584109	10.0674	-4.646782	0.000343	0.005528	-0.157185	10.66861	9.872549	10.16028	10.56245	9.988336	10.11066	9.972531	9.955481	10.01972	10.04663	9.89896	9.909002	9.901911	9.878017
Clasp2	-0.547595	8.518128	-4.645179	0.000344	0.005531	-0.223982	8.436944	8.119095	7.961495	8.767887	8.045416	8.087685	8.870455	8.677985	8.75794	8.653762	8.663343	8.630956	8.819105	8.766137
Dram1	2.253167	1.67584	4.645129	0.000344	0.005531	0.365477	1.178614	2.064844	2.568639	-0.166914	2.936364	2.974059	1.297885	1.279465	1.426399	1.540753	1.740817	2.433555	0.962073	1.80183
Cd36	6.824178	-1.754735	4.643713	0.000345	0.005539	0.041826	-2.659044	1.047298	2.804219	-3.260591	2.651221	2.634313	-3.260591	-3.260591	-1.10526	-2.618976	-2.293564	-1.243945	-2.232431	-1.899685
S100a9	5.089565	0.104708	4.641144	0.000347	0.005559	0.411241	-0.760787	3.899656	4.511585	-1.223791	4.430682	2.504391	-1.450335	-0.654381	-2.155202	-0.116374	-0.616402	-0.920914	-2.232431	-0.748265
Pla2g4a	1.439705	3.023104	4.640184	0.000348	0.00556	0.039382	2.63325	3.769134	3.95649	2.951788	4.380852	4.798021	2.305626	2.539194	2.55384	2.572488	2.494078	3.245131	1.881043	2.460763
Atf1	0.62831	4.796299	4.639613	0.000348	0.00556	-0.178055	4.640141	5.240367	5.242827	4.520817	5.242473	5.097433	4.861747	4.568886	4.485023	4.680472	4.627162	4.840964	4.539769	4.6173
Spred3	0.853247	3.687153	4.638965	0.000348	0.00556	-0.07493	3.683012	4.361326	4.868125	3.567317	4.451953	4.188991	3.356296	3.321106	3.456633	3.158464	3.250834	3.245131	3.576053	3.262515
Gmds	0.598384	4.22141	4.636777	0.00035	0.005566	-0.152748	4.335237	4.817439	4.862114	4.199801	4.936707	4.830014	3.882992	3.94833	3.83015	3.832569	4.116027	3.838214	3.999391	3.757487
Rpl4	0.651296	8.32119	4.636659	0.00035	0.005566	-0.238755	8.334515	9.06726	8.673405	8.182025	8.913456	8.980751	8.044678	8.012563	8.022741	8.327473	8.078655	8.029802	7.833398	8.000989

Cd300a	1.792953	2.465764	4.635885	0.000351	0.005566	0.242696	1.831529	2.943995	3.633224	1.000693	2.640673	3.340548	0.903114	3.475434	1.29127	3.463704	3.414796	4.037544	1.252133	1.659607
Kif22	2.844193	0.973122	4.63532	0.000351	0.005566	0.400424	0.369528	2.082541	1.430088	-1.223791	2.762423	2.592292	0.563361	0.923327	1.061401	0.799637	1.102128	1.315214	0.745852	0.119416
Dpep2	4.583462	0.174681	4.63496	0.000351	0.005566	0.242743	-0.410969	1.607774	1.775325	-3.260591	1.803478	0.915838	-0.672244	1.198329	-0.773548	0.847278	0.764165	1.568131	0.182287	0.190106
Ntf3	-1.760699	3.960714	-4.634328	0.000352	0.005566	-0.061118	4.073693	1.725091	2.984714	4.91074	2.79132	3.420773	4.980068	4.49437	4.538218	4.850841	4.208334	4.536248	4.042758	4.022545
Pgbd5	-1.164266	6.897976	-4.631198	0.000354	0.005593	-0.273292	7.246973	5.538441	6.560823	7.625646	6.254387	6.728782	6.95915	7.161321	7.059183	6.939126	7.260044	7.057312	7.285001	6.90965
C1qbp	0.599273	6.438804	4.625194	0.000358	0.005651	-0.284975	6.328664	6.758752	6.950815	6.261328	7.133925	6.731204	6.130942	6.288396	6.32535	6.271373	6.357522	6.270316	6.113648	6.239378
Jak2	0.737208	5.615727	4.622307	0.00036	0.005675	-0.259548	4.997891	5.800134	6.154947	5.511695	5.934248	6.087691	5.759921	5.567732	5.416182	5.560929	5.478587	5.53465	5.435298	5.412816
Rassf1	0.71896	4.029385	4.619817	0.000362	0.005695	-0.126905	3.805036	4.279295	4.641253	3.701615	4.581681	4.352109	4.017238	3.904241	3.767885	4.14048	3.958531	3.906064	3.713602	3.739923
Uhrf1	1.326043	3.765081	4.616381	0.000364	0.005724	-0.012732	3.09177	4.535265	3.776458	2.602113	4.299374	4.032808	4.118484	3.763261	4.241012	3.92982	3.776475	3.269114	3.556237	3.842239
Rbm3	1.417843	7.18107	4.61588	0.000364	0.005724	-0.299486	6.934491	8.262093	8.975415	6.714516	7.607651	8.128686	6.995096	6.740154	6.929376	7.13719	6.546208	6.556764	6.151559	6.867809
Camkk1	-0.8251	8.440656	-4.610296	0.000368	0.005779	-0.297464	8.452144	7.205428	8.040575	8.543739	7.723693	7.728072	8.900381	8.799661	8.888589	8.712423	8.866231	8.698009	8.936008	8.679148
Lpcat1	0.638745	5.768277	4.602956	0.000374	0.005852	-0.298545	5.317901	5.701936	5.914769	5.24932	6.025245	6.038663	5.67752	5.947219	5.672184	5.962155	5.658667	5.898513	5.828509	5.892471
Tmem119	-1.89794	3.918368	-4.602482	0.000374	0.005852	-0.089597	4.813555	2.724859	2.039558	3.838533	3.060255	1.960428	4.253222	4.889545	4.409542	4.320048	4.298745	5.428618	4.066299	4.896325
Podxl	0.841796	6.514661	4.601194	0.000375	0.005856	-0.332782	6.641858	7.270567	7.270043	6.263935	7.140023	7.493518	6.369857	5.999045	6.532415	6.143636	5.722668	6.0958	5.897067	6.383033
C1ql3	-1.993859	6.154351	-4.600798	0.000375	0.005856	-0.311477	5.88391	4.08227	4.880071	7.663335	4.418385	5.725349	6.332881	6.854627	7.015636	6.706251	6.423477	6.886309	6.573205	6.744782
Kif18b	2.945872	1.021502	4.598128	0.000377	0.005879	0.31395	0.176305	3.019437	1.775325	-0.243853	3.434596	3.045873	0.282279	0.76329	0.245823	0.591587	0.535373	1.618784	0.249639	-0.204094
Eif3b	0.46593	6.892714	4.596727	0.000378	0.005888	-0.344006	6.678054	7.104017	7.208207	6.704952	7.184271	7.132664	6.807366	6.822836	6.743697	6.905061	6.877931	6.913192	6.722912	6.706108
Pecr	-0.747405	4.360666	-4.592205	0.000382	0.005931	-0.237836	4.785591	3.859159	3.702138	4.569411	4.230707	3.939526	4.725734	4.490115	4.504248	4.509637	4.444493	4.390807	4.302685	4.673894
Utp14a	0.876239	5.067888	4.591325	0.000382	0.005931	-0.291068	4.664912	5.880736	5.558705	4.835601	5.758817	5.314898	4.946803	5.077105	4.908035	5.138601	4.884809	4.744468	4.40224	4.883051
Nr2c2ap	0.667851	4.864881	4.590952	0.000383	0.005931	-0.282586	4.835542	5.426835	5.376741	4.512069	5.372461	5.170651	4.781687	4.696241	4.663209	4.800297	4.840346	4.451452	4.615127	4.620503
Ifi27l2b	2.036114	0.644551	4.584148	0.000388	0.005996	0.31138	-0.310971	1.702379	1.451597	-0.81875	1.143601	1.014597	0.68572	0.818632	0.007959	1.355481	1.319276	1.738143	0.182287	-0.116166
Btbd3	-1.170862	6.459813	-4.583927	0.000388	0.005996	-0.364746	6.638736	5.007763	5.964241	7.104675	5.585425	6.235782	6.945436	6.660038	6.844433	6.369468	6.668525	6.815466	6.927642	6.689429
Nadk2	-0.672328	5.909776	-4.583024	0.000388	0.005996	-0.366913	6.623019	5.762202	5.845376	6.501967	5.921234	6.032783	5.924304	5.425693	5.602537	5.887213	5.613877	5.776934	5.951178	5.895119
Gpr37	-1.155701	7.144776	-4.582706	0.000389	0.005996	-0.367884	7.626597	6.332901	6.121791	7.622263	6.993848	6.430202	7.259289	7.330906	7.17767	7.128781	6.885245	7.725891	7.756975	7.646508
Grn	1.163821	7.351763	4.580255	0.000391	0.006017	-0.372134	6.658914	7.272977	7.523361	6.129317	7.955723	7.474487	6.572791	8.290625	6.784368	8.143828	7.852318	8.830838	6.437174	7.008928
Ak4	-1.097696	4.008419	-4.578442	0.000392	0.006031	-0.266429	4.938178	3.445867	3.688616	4.79531	4.299374	3.642872	3.669732	3.811801	3.930199	3.478908	4.164923	4.060629	4.467348	3.825682
Krt2	-1.949756	3.204392	-4.577056	0.000393	0.006039	-0.115405	4.078311	0.812964	2.787402	4.357872	3.181638	2.244393	2.991343	2.951874	3.729193	3.508843	4.180861	3.461789	3.294432	3.496677
Folr2	4.57996	0.505276	4.575762	0.000394	0.006047	0.278377	0.428583	2.383133	2.597868	-2.731185	3.546179	2.469996	0.118118	-0.26106	-1.10526	0.348395	1.102128	1.738143	-0.755361	-0.505482
Nat8l	-0.702187	8.395895	-4.57341	0.000396	0.006065	-0.370783	8.447467	7.405435	7.874099	8.453146	7.829643	7.888047	8.680014	8.599925	8.622872	8.523729	8.725784	8.813784	8.935209	8.748312
Npm1	0.802294	5.489816	4.572898	0.000396	0.006065	-0.366112	5.159142	5.972579	6.222188	5.707033	6.495158	6.261985	5.122294	5.371209	5.144489	5.121749	5.276493	5.229104	5.05004	4.760648
Selv	-1.238233	1.864284	-4.570994	0.000398	0.00608	0.048861	2.957854	1.874793	1.758166	2.499634	1.600747	0.809818	2.266943	1.71825	2.364852	1.625169	1.482482	1.805289	1.902128	1.889408
Npepl1	0.983374	4.473416	4.569862	0.000399	0.006086	-0.271931	4.428604	5.344885	5.08147	3.737082	5.093814	4.692588	4.028845	4.49437	3.974983	4.673881	4.460902	4.733017	3.719553	4.238063
Spock3	-1.352834	6.235819	-4.567871	0.0004	0.006101	-0.395056	6.412421	5.060063	5.389334	7.456238	5.725516	6.148763	6.165528	6.271141	6.52019	6.232656	6.004833	6.367012	6.814729	6.755748
Cadm2	-1.191286	5.474026	-4.564317	0.000403	0.006136	-0.341662	5.237611	3.661386	4.559421	5.63231	3.926497	4.807235	5.979177	6.276092	6.230419	5.734068	6.121937	6.078939	6.364974	6.070483
Dusp4	-1.758268	3.046513	-4.563678	0.000403	0.006136	-0.119913	3.431974	0.934883	2.538805	4.467515	2.361201	2.949304	3.159163	3.211662	3.408468	3.060806	3.50618	3.237047	3.569478	3.041369
Sec14l2	-0.739873	6.79104	-4.562967	0.000404	0.006137	-0.410519	6.99252	6.446598	6.235424	6.9153	6.375422	5.803393	6.980974	6.915387	7.23633	6.885941	7.118272	6.880482	7.197629	7.105817
S100a3	2.271383	2.130879	4.558086	0.000408	0.006188	0.195381	0.955837	3.673115	2.322392	0.166125	2.732935	2.3616	1.135593	3.377479	1.819202	3.267546	2.930971	3.220743	1.077784	1.260365
Bcl7c	0.836451	3.691079	4.554884	0.00041	0.006218	-0.193916	3.467609	4.354059	4.000932	3.16769	4.223657	4.009049	3.29032	3.458111	3.367065	4.031278	3.797302	3.744381	3.356854	3.531125
Dmtn	-0.634262	8.633623	-4.549108	0.000415	0.006281	-0.401061	8.923017	8.306303	8.355675	9.222219	8.58526	8.502526	8.719271	8.584043	8.785309	8.626715	8.593806	8.357744	8.545254	8.767582
Negr1	-1.34114	5.248198	-4.547326	0.000416	0.006295	-0.398743	5.480228	3.86939	4.994392	6.244702	4.43374	4.767664	5.14913	5.382731	5.741865	5.06243	5.17869	5.387214	6.090872	5.737361
Fam163b	-1.187585	5.793829	-4.54457	0.000419	0.006321	-0.426618	5.982044	4.544872	5.541256	6.541908	4.730847	5.465068	5.54638	6.182948	6.060311	6.08771	6.294829	6.347434	6.09202	5.726997
Tnfrsf1b	2.039595	2.120022	4.542744	0.00042	0.006332	0.117071	1.48718	3.099845	3.436929	0.531921	3.106978	2.244393	1.091998	2.436799	1.717448	2.572488	2.627988	3.25317	1.150071	1.355706
Csf2ra	1.530931	4.578216	4.542378	0.00042	0.006332	-0.268628	3.896775	5.273489	5.153781	3.218762	5.346735	4.501484	4.591292	4.647404	4.585917	5.013676	4.630068	5.127817	3.576053	4.604416
Slc26a2	0.963943	2.867316	4.536803	0.000425	0.006393	-0.128048	2.832262	3.812199	3.515619	2.487785	3.359399	3.756629	2.703406	2.650138	2.611707	2.668442	2.428842	2.960934	2.269694	2.310209
Rhbdf1	1.175239	4.968251	4.534746	0.000427	0.006411	-0.356138	4.664912	5.42163	5.219509	3.716922	5.592283	5.159902	5.154438	5.07142	4.910919	5.277674	4.857801	5.237233	4.345646	4.978507
Entpd5	-0.911717	3.777797	-4.531229	0.00043	0.006443	-0.301355	4.096635	3.224637	3.387569	4.479531	3.372207	3.552622	3.585784	3.991112	3.729193	3.508843	4.017407	3.854157	4.274664	3.932632
Cdc45	1.593498	1.814848	4.530907	0.00043	0.006443	0.067078	1.278303	2.884706	2.10888	1.245516	3.099296	3.255598	1.544223	1.49863	1.520039	1.322149	1.507975	1.715048	1.521045	1.386135
Pold1	1.287748	3.42245	4.524753	0.000435	0.006513	-0.145313	2.718545	4.051149	3.409716	2.401998	4.209453	3.683465	3.727855	3.583227	3.424703	3.552611	3.004456	3.419555	3.178239	3.704139
Fhdc1	-0.824678	4.91841	-4.523502	0.000436	0.006521	-0.40093	4.692281	4.279295	4.102999	5.278844	4.272298	4.032808	5.384156	5.400976	5.347591	5.463023	5.065211	5.348344	4.999576	5.246561
Adcy8	-0.795821	4.186061	-4.522317	0.000437	0.006521	-0.330935	4.331374	3.702025	3.453014	4.314927	3.252623	3.732651	4.36733	4.424739	4.680213	4.732143	4.387261	4.689249	4.341792	4.287263
Prom1	-1.070779	4.734565	-4.521933	0.000438	0.006521	-0.399017	5.41236	4.339413	3.728808	4.706082	3.97768	3.931123	5.513711	4.785751	5.330461	4.949639	4.356018	4.980656	4.974911	5.362053
Tnfaip2	1.478848	2.733137	4.521371	0.000438	0.006521	-0.074069	1.9771	3.551306	3.719973	2.13961	3.529124	3.288866	1.697535	3.756192	2.223535	2.39151	2.873292	3.368666	1.922909	2.087586
Rhoj	1.419877	3.033917	4.520976	0.000438	0.006521	-0.196006	3.163058	4.502772	4.648255	2.463791	4.098216	3.57437	2.247205	2.603631	2.313467	2.438927	2.374443	2.794648	2.881849	2.584013
Rpl14	0.730302	6.70966	4.519323	0.00044	0.006527	-0.496074	6.710392	7.498187	7.065545	6.654172	7.457237	7.624706	6.412571	6.246128	6.20597	6.729478	6.39899	6.41079	6.219277	6.31744
Clstn3	-0.618639	8.411615	-4.519234	0.00044	0.006527	-0.486719	7.909141	7.255043	7.583656	8.188674	7.572359	7.310034	9.207599	8.800521	9.197705	8.849602	8.686902	8.838204	9.052715	9.315788
Larp6	-0.656658	5.243446	-4.515465	0.000443	0.006567	-0.466892	5.511294	4.75259	5.023408	5.660292	4.733331	5.207652	5.135774	5.427919	5.282274	5.266765	5.441032	5.401751	5.391239	5.215125
Ky	-1.68768	0.624127	-4.511774	0.000446	0.006606	0.140526	1.033981	-0.103798	-0.33572	1.63746	0.422095	-0.862697	1.135593	1.198329	1.393785	0.534523	1.167576	0.514819	1.150071	1.009844
Prdx1	0.923141	4.235016	4.507545	0.00045	0.006653	-0.375736	4.193457	4.951089	4.662159	3.665254	5.244216	4.509973	3.876582	4.106787	3.930199	4.116377	4.235294	4.092341	3.760537	4.032198
Hopx	1.038565	4.92453	4.504603	0.000453	0.006683	-0.471888	5.069216	6.089878	6.311647	4.654018	5.618052	5.555324	4.35355	4.411301	4.091419	4.930413	4.653101	4.948838	3.913738	4.399663
Tlr8	4.39618	-0.598406	4.503288	0.000454	0.006692	0.154071	-0.634519	1.453108	2.753168	-2.040259	3.060255	3.242073	-3.260591	0.04768	-1.536754	0.134646	-0.510232	1.045835	-2.656794	-3.260591
Clmn	-1.010667	5.837237	-4.500802	0.000456	0.006717	-0.521935	6.177623	5.419891	5.460016	6.637511	5.022485	5.692163	5.842809	6.047627	5.720655	5.637447	5.790457	6.064165	6.446174	5.790939
Wnk2	-0.553284	7.68753	-4.498746	0.000458	0.006731	-0.528639	7.959131	7.608424	7.442421	7.976926	7.220136	7.38771	7.82095	7.707278	7.750662	7.627523	7.80136	7.685991	7.892869	7.751769
Grm3	-1.067406	5.853625	-4.498447	0.000458	0.006731	-0.52758	6.468902	5.596564	4.917262	6.487164	5.692706	5.45631	5.773784	5.653939	5.882203	5.706885	5.55395	6.216857	6.489482	6.083308
Dock8	1.299621	4.36461	4.497222	0.000459	0.006736	-0.341134	3.608352	4.390036	4.691832	3.329127	5.089943	4.841271	3.504983	5.282961	3.324439	4.984678	4.894507	5.899794	3.701625	3.648741
Stk32c	-0.633894	6.3071	-4.496795	0.00046	0.006736	-0.538123	6.416986	5.752562	6.077517	6.696612	5.719264	6.143317	6.353797	6.475429	6.435601	6.52404	6.48895	6.374401	6.477241	6.383975
Mif4gd	1.311159	3.672228	4.49471	0.000462	0.006751	-0.233625	3.230989	4.393585	4.12993	2.255954	4.05518	3.519371	3.691803	3.858761	3.480126	4.056832	3.858035	4.009344	3.229186	3.774841
Wdr78	-0.991412	4.001461	-4.494362	0.000462	0.006751	-0.429876	4.950824	3.61957	3.530855	4.619139	3.785203	4.240912	3.863678	3.929599	3.935874	3.566911	4.32049	3.721386	4.262487	3.774841
Ecm1	1.514718	4.107905	4.493832	0.000462	0.006751	-0.34177	3.896775	5.355842	4.608118	2.908002	4.7917	4.814106	4.145391	3.559057	4.002283	4.332655	3.678847	3.732929	3.333761	4.450943
Stk39	-1.054285	6.579649	-4.493053	0.000463	0.006751	-0.547265	7.515217	5.94503	6.23232	7.412251	6.629912	6.832258	6.419201	6.307866	6.455491	6.227039	6.970808	6.284113	6.582215	6.318427
Nuak2	1.21958	2.850605	4.492223	0.000464	0.006751	-0.190849	3.045357	3.707738	3.614277	1.892408	3.736505	3.53054	2.629097	2.822481	1.843557	2.854798	2.833517	3.10114	2.003164	2.530531
Rbl1	1.654416	1.966898	4.491712	0.000464	0.006751	-0.040788	1.541696	2.914655	2.434653	1.405223	3.428477	3.637716	1.782265	1.318383	1.717448	1.540753	1.533025	1.738143	1.724133	1.260365
Grem1	-1.08416	2.671912	-4.491367	0.000465	0.006751	-0.166237	3.109925	1.506527	1.874203	2.943136	2.09374	2.31068	3.01488	2.849305	2.568526	2.90068	2.921517	3.092205	3.318158	3.169358
Lst1	3.037614	-0.274811	4.490143	0.000466	0.006759	0.140773	-0.217457	1.812527	1.966737	-1.788967	2.030509	1.07687	-0.399109	0.377517	-0.632503	-1.33445	-0.318799	-0.24119	-1.904936	-1.632384
Prdx5	0.682597	6.775473	4.487625	0.000468	0.006774	-0.558869	6.95158	7.431995	7.833461	6.825182	7.589256	7.430447	6.344538	6.185581	6.488686	6.176646	6.606451	6.224032	6.476362	6.307538
Amigo1	-1.149845	4.841083	-4.487574	0.000468	0.006774	-0.477737	4.995452	3.375827	4.402616	5.453487	4.173318	4.323498	4.851979	5.385024	5.050846	4.850841	5.136409	5.185663	5.493764	5.156753
Mt2A	4.088253	1.811491	4.487161	0.000468	0.006774	0.106285	0.369528	4.414697	4.650582	-0.502853	3.864394	2.570814	0.798508	2.287882	1.142163	2.06872	1.533025	2.375822	0.182287	0.321874
Ank3	-0.766703	8.689644	-4.486051	0.000469	0.006778	-0.535152	8.422262	8.022982	8.12436	9.182225	7.928883	8.056152	9.181675	9.046902	9.007372	8.834906	8.765629	8.85893	9.179829	9.04696
Zhx2	-0.620404	4.846715	-4.485573	0.00047	0.006778	-0.511784	5.350716	4.841025	4.617663	5.337788	4.755497	4.695072	4.394501	5.028055	4.666626	4.868265	4.763054	5.081164	4.81457	4.695317
Ppp4r1	0.810229	5.430863	4.484637	0.000471	0.006783	-0.512006	5.069216	5.953471	5.635887	4.731426	5.816184	5.422235	5.615366	5.456551	5.420236	5.571605	5.376758	5.521398	5.040563	5.438452
Slpi	6.252034	-0.734559	4.481444	0.000474	0.006817	0.085005	-2.235785	3.33211	5.203752	-1.388744	3.990197	3.275651	-1.73438	-0.992527	-1.813089	-2.618976	-0.855515	-1.925331	-3.260591	-2.653844
Prkab1	0.928096	5.119395	4.480565	0.000475	0.006821	-0.500725	4.878536	5.905805	5.644084	4.364367	5.473985	5.14726	4.943741	5.240531	4.827903	5.445717	5.188577	5.206513	4.526252	4.925106
Acsl3	-0.882302	8.342461	-4.479684	0.000475	0.006824	-0.545734	8.716552	7.649076	8.044561	9.094973	7.918278	8.481277	8.414013	8.098573	8.461353	8.242334	8.090041	8.378792	8.493879	8.715736
Cxcr2	6.135799	-2.529271	4.478882	0.000476	0.006827	-0.186265	-2.235785	1.480065	3.111733	-3.260591	2.09374	1.107027	-3.260591	-3.260591	-3.260591	-3.260591	-2.293564	-2.668207	-3.260591	-2.228037
Chaf1b	1.495709	2.026663	4.477444	0.000477	0.006834	-0.10966	2.01613	3.037697	2.673036	1.502648	3.617838	3.568964	1.000645	1.80302	1.489496	1.322149	1.94125	1.97042	1.60059	1.260365
Wnk4	-1.073037	5.681901	-4.476915	0.000478	0.006834	-0.548809	5.621385	4.204491	5.118919	6.172519	4.698159	5.260595	6.274358	5.979449	6.39498	5.916821	5.855203	5.613262	6.095456	6.375469
Nme6	1.351489	2.7088	4.476473	0.000478	0.006834	-0.158431	2.474162	3.287027	3.177748	1.658753	3.900209	3.158148	2.48486	2.634802	2.127623	3.100666	2.761158	2.619163	2.133689	2.660694
Fam53b	-0.55832	5.696529	-4.475954	0.000479	0.006834	-0.558556	5.841078	5.277336	5.148852	5.746104	5.254629	5.265617	5.88493	5.834271	5.743484	5.789994	6.001472	6.066448	5.897067	6.03131
Ttyh3	-0.565878	8.46441	-4.472818	0.000482	0.006868	-0.567243	8.593324	7.878502	8.072811	8.471876	7.994552	7.925019	8.579518	8.668838	8.704299	8.697269	8.509352	8.856129	8.729052	8.825805
Sorl1	-1.108847	8.955356	-4.471918	0.000483	0.006871	-0.560786	8.684921	7.214987	7.749124	9.267969	8.131978	8.37143	9.731576	9.423801	9.642012	9.275664	9.348658	9.403609	9.54369	9.589345
Drap1	0.584299	7.303943	4.470944	0.000484	0.006876	-0.584808	7.650438	7.958852	8.124987	7.391335	8.302053	8.037678	6.720566	6.945999	6.831553	6.841787	7.054826	6.835013	6.851777	6.718866
Pip5k1b	-1.561092	4.853327	-4.468325	0.000486	0.006904	-0.522992	5.805316	2.812308	3.98258	5.301019	4.539721	4.573448	5.114144	5.232912	4.899348	5.074991	5.677044	4.928903	5.078103	4.990935
Nfil3	0.715095	3.569297	4.46762	0.000487	0.006905	-0.388936	3.357948	4.279295	4.214137	3.622545	4.223657	4.116722	3.116285	3.330656	3.079866	3.14899	3.543639	3.587916	3.387081	3.097617
Osbpl1a	-0.526444	7.579939	-4.466038	0.000488	0.006907	-0.589538	8.069063	7.754956	7.576638	8.244093	7.647378	7.538817	7.448343	7.405662	7.491705	7.230532	7.454909	7.35727	7.522653	7.385429
Tuba4a	-0.924631	8.731301	-4.465234	0.000489	0.006907	-0.565055	9.231795	7.769711	8.547349	9.235455	8.516797	8.411251	8.719943	8.865607	8.969661	8.610629	9.051269	8.679114	8.988704	8.644756
Procr	2.514324	1.45429	4.465093	0.000489	0.006907	0.023679	1.669604	2.758277	2.744481	-0.600663	3.00376	3.045873	0.903114	0.705741	1.061401	1.540753	1.674823	2.027776	-0.512812	1.122575
Abi3	1.153205	3.751417	4.465035	0.000489	0.006907	-0.320008	3.365524	3.806886	3.789185	2.523044	4.542557	4.135132	3.190506	4.624294	2.984524	4.472141	4.383823	4.578447	3.03287	3.220915
Shisa2	-1.457095	1.246612	-4.45917	0.000495	0.006979	-0.045106	2.263247	0.769921	0.52603	1.780306	0.562819	0.571087	0.429655	1.279465	1.690848	1.597576	1.803924	1.715048	1.700256	1.473753
Stk38l	0.736589	5.120766	4.456272	0.000498	0.007003	-0.554649	4.742602	5.709075	5.614594	4.708637	5.176461	5.343677	5.415442	5.039747	5.175999	4.960511	4.842853	4.953779	5.05004	5.003257
Fmnl1	-0.683329	8.027335	-4.456216	0.000498	0.007003	-0.606719	8.137311	7.45553	7.547283	8.469619	7.913905	7.561113	8.236447	8.236408	8.203558	8.262791	8.016736	8.198525	7.954092	8.195556
Rab31	0.568997	7.128077	4.454375	0.000499	0.007015	-0.624906	7.205363	7.642007	7.601805	6.845636	7.561217	7.571965	6.647158	7.067345	6.691821	7.193572	7.124981	7.392724	6.660865	6.598144
Tlr2	1.628919	3.007652	4.454153	0.0005	0.007015	-0.178493	2.342161	3.08236	3.647273	1.430203	3.89578	3.207696	2.247205	3.791198	2.347926	3.478908	3.324422	4.166573	2.771602	2.59708
Mfsd6	-0.943719	7.445856	-4.452192	0.000502	0.007034	-0.626317	7.338354	6.396186	6.643162	8.112592	6.994365	7.087057	7.652047	7.669678	7.743804	7.499656	7.590844	7.654092	7.949352	7.920439
Cabp1	-0.620378	6.86958	-4.450647	0.000503	0.007047	-0.634458	7.303775	6.533608	6.33136	6.858921	6.642522	6.340237	7.13256	6.957602	7.132672	6.976429	7.054285	6.9062	7.012497	7.005257
Adam11	-1.00181	8.613424	-4.447742	0.000506	0.007076	-0.624632	8.299102	7.965723	7.106797	8.377905	7.292668	6.976549	9.594778	9.146913	9.591675	9.338352	8.922263	9.153472	9.202581	9.624268
Slc2a13	-1.59221	7.155528	-4.447258	0.000507	0.007076	-0.634733	6.977767	4.984419	6.020192	8.177191	6.432334	6.487922	7.601448	7.405097	7.761487	7.466194	7.368395	7.577332	7.87798	8.053479
LOC100911177	1.09872	5.157887	4.4468	0.000507	0.007076	-0.592404	5.108122	6.728159	6.085277	4.783243	5.624757	5.725349	4.989008	4.643578	4.673435	4.971302	4.768337	4.689249	4.686744	4.780829
Plcd1	1.140762	5.529139	4.444144	0.00051	0.007105	-0.602075	5.466203	6.611101	6.245463	4.569411	6.047374	5.692163	5.372815	5.333708	5.306568	5.589225	5.196438	5.470592	5.033414	5.50928
Acot11	-1.034877	2.239976	-4.443063	0.000511	0.007111	-0.244806	3.230989	2.184396	1.613009	2.57996	2.014259	1.72793	2.078759	2.077116	2.364852	2.184529	2.259075	2.268872	2.601568	2.516844
Kctd4	-1.91957	6.320009	-4.44182	0.000512	0.00712	-0.641799	6.834315	4.283127	5.807301	8.098782	5.739176	6.323478	6.053507	6.582224	6.618735	6.358207	6.645739	6.323774	6.696	6.139092
Was	1.794509	2.391389	4.440414	0.000513	0.007132	-0.16445	2.162475	3.790827	3.028308	1.065902	3.8553	2.781814	1.607516	3.126505	1.29127	2.854798	2.792614	3.204252	0.962073	1.32462
Orai1	1.183071	4.07919	4.43847	0.000515	0.007144	-0.497901	4.247758	4.869338	5.365454	3.309066	4.949583	4.554424	3.40164	3.550909	3.963917	3.709073	3.939921	4.023513	3.508915	3.814538
Tmem202	1.637166	2.620789	4.438383	0.000515	0.007144	-0.219303	1.874641	4.256089	3.330668	1.996424	3.280067	3.420773	2.286414	2.58779	2.295923	3.080873	2.288794	2.715403	1.28461	2.278116
Rcbtb2	0.664967	4.070233	4.436033	0.000518	0.007161	-0.506827	4.096635	4.735912	4.61051	3.69132	4.451953	4.401612	3.824257	3.94833	3.722643	4.031278	4.056804	4.078836	3.754753	3.673625
Kntc1	1.31479	3.053339	4.435096	0.000519	0.007161	-0.252451	2.230432	3.655486	2.924461	2.061822	3.67581	3.55809	3.662299	2.964197	2.905753	3.267546	2.853541	2.970717	2.954296	3.262515
Nfatc2	-1.044884	2.915552	-4.43498	0.000519	0.007161	-0.353182	3.516065	1.812527	2.578448	3.41297	2.672087	2.592292	2.880403	3.093229	2.810125	3.195748	3.331578	2.869767	3.40196	2.868948
Aida	0.499287	5.97932	4.434824	0.000519	0.007161	-0.662352	6.334466	6.80812	6.605881	6.215363	6.859876	6.820363	5.501266	5.537125	5.393679	5.578679	5.51519	5.528039	5.569853	5.46898
Afg3l1	0.834534	4.966416	4.434234	0.00052	0.007161	-0.593163	4.902169	5.744246	5.488909	4.383678	5.504845	5.145445	4.732848	4.662608	4.639062	4.902496	4.939699	5.138705	4.576302	4.820364
Arhgap39	-0.633378	7.244276	-4.433561	0.00052	0.007162	-0.66787	7.136739	6.437172	6.823076	7.463441	6.719089	6.686344	7.630783	7.663551	7.5899	7.475225	7.473629	7.362853	7.576929	7.392004
Nckap1l	0.933012	5.187323	4.432013	0.000522	0.007168	-0.578376	4.683216	5.330145	5.120598	4.140492	5.638075	5.253872	4.438678	6.283487	4.359752	6.052575	5.879821	6.457633	4.310591	4.722402
Fggs	1.157018	4.679168	4.431995	0.000522	0.007168	-0.520155	3.953171	5.361289	5.123951	3.752019	5.040651	4.490087	4.983054	4.70361	4.846099	5.011064	4.373459	4.662336	4.334055	4.938001
Ddx56	0.776728	3.727264	4.430459	0.000523	0.007181	-0.451062	3.495497	4.346754	4.185784	3.315784	4.312723	3.862069	3.776861	3.583227	3.440756	3.424979	3.66758	3.703897	3.569478	3.61702
Pcdha12	-1.643804	1.201368	-4.429385	0.000525	0.007188	-0.057306	1.514695	-0.687936	-0.007587	1.996424	0.985877	0.481842	1.862293	1.80302	1.663747	1.780488	1.135223	1.593679	1.859646	1.659607
Adamts7	3.084129	2.088799	4.428095	0.000526	0.007195	-0.118926	1.310059	4.350411	5.096907	0.74601	3.686105	3.02233	2.206902	1.239467	1.663747	1.064403	0.805871	1.152831	1.150071	2.245294
Prkcd	1.209388	4.920989	4.427756	0.000526	0.007195	-0.574341	4.762823	5.847482	5.44535	3.727037	5.258083	5.195423	4.248266	5.844307	3.854323	5.650933	5.417684	5.888226	3.589113	4.225496
Gstm7	-0.752565	8.141176	-4.420111	0.000534	0.007295	-0.666947	8.710987	7.787355	7.975714	8.834895	8.256581	8.062418	7.847001	7.844008	8.31825	8.124623	8.260256	7.901742	8.088353	7.969935
Mast3	-0.802364	9.436834	-4.419517	0.000535	0.007296	-0.647557	9.195993	8.280659	8.646264	9.732923	8.827133	8.890712	9.895773	9.834315	9.942282	9.788586	9.62207	9.745086	9.730625	9.985721

Lyl1	1.636835	2.584298	4.415993	0.000539	0.007338	-0.239811	2.372556	3.339488	3.560852	1.245516	3.917788	2.734303	2.01015	3.024282	1.891068	2.866406	3.013384	3.245131	1.60059	1.659607
Crlf1	-1.115358	6.010841	-4.414833	0.00054	0.007346	-0.679227	6.123917	4.167656	4.940267	6.034761	5.348357	5.382721	6.819947	6.232184	6.820092	6.387707	6.087476	6.342733	6.530682	6.962353
Nop58	0.655575	6.170788	4.414276	0.00054	0.007346	-0.700117	5.834269	6.800788	6.766277	6.267405	6.585232	6.676322	6.109239	5.96112	6.040696	6.055113	5.822558	5.835662	5.715431	5.943239
C3	1.10921	6.504357	4.412423	0.000542	0.007365	-0.672446	5.331503	6.264775	5.839269	4.973688	6.481924	6.444296	5.738875	8.602637	5.163961	8.15777	7.93452	8.731302	5.623207	5.796614
Gbp2	3.083425	4.157839	4.408099	0.000547	0.007419	-0.454191	3.230989	5.099711	7.521456	2.602113	5.305596	5.937404	3.127125	4.768288	2.667343	3.989425	3.690026	5.257355	2.317563	2.825198
Zbtb7b	0.781836	4.557673	4.407374	0.000548	0.007421	-0.646077	4.777095	5.512563	5.53119	4.514991	5.517871	5.130842	3.96382	3.979018	3.780555	4.159476	4.082483	4.465359	4.337929	4.125333
Sectm1b	6.946769	-1.97017	4.406733	0.000548	0.007422	-0.309337	-3.260591	1.58312	3.265371	-3.260591	0.845972	0.846031	-2.558588	-2.076466	-2.604521	-2.176425	-1.978322	-0.78301	-3.260591	-1.899685
Akr1b8	2.795469	-0.603026	4.404337	0.000551	0.007436	-0.025924	-1.22354	0.04804	1.340699	-1.575022	1.664612	1.249022	-1.450335	-0.654381	-0.504027	-0.961779	-0.411343	-0.78301	-1.218554	-1.212031
Srebf2	-0.593416	8.169837	-4.404188	0.000551	0.007436	-0.713354	8.022201	7.343022	7.540399	8.262683	7.499805	7.812851	8.690329	8.238946	8.706375	8.323267	8.21066	8.338983	8.632015	8.761973
B2m	0.928025	9.623356	4.404087	0.000551	0.007436	-0.654765	9.943651	10.28747	10.6494	9.230686	10.61002	10.53065	8.560402	9.629656	8.686737	9.63821	9.377037	9.913526	8.619737	9.05201
B4galt1	2.00693	2.253526	4.399878	0.000556	0.007489	-0.200032	2.569081	3.24835	3.289459	0.105385	3.167012	3.172479	1.408645	2.471744	1.867508	2.308845	2.213317	2.941167	1.28461	1.910497
Astn2	-0.744739	5.297001	-4.399263	0.000557	0.00749	-0.667525	5.005184	4.63158	4.346317	5.365572	4.158607	4.657358	5.785805	5.706232	5.632548	5.748254	5.640054	5.923914	5.65771	5.943239
Plcg2	1.291936	2.932909	4.38623	0.000571	0.00767	-0.369474	2.63325	3.354134	3.222225	1.92792	3.904623	3.683465	2.73209	3.340143	2.107651	3.276274	2.921517	3.52291	2.060555	2.59708
Ift80	-0.604673	4.667115	-4.386028	0.000571	0.00767	-0.676373	4.85451	4.21255	4.287732	4.982159	4.3745	4.389395	4.977075	4.628172	4.697018	4.630291	4.812484	4.752996	5.066477	4.737232
Ttll9	-1.737249	4.012632	-4.384065	0.000573	0.007691	-0.504884	4.251852	3.758163	2.08155	3.972106	2.542105	1.327961	5.211572	4.655026	4.833994	4.778959	4.451079	4.677349	4.573018	5.213005
S100a8	5.000639	-1.561103	4.381023	0.000577	0.007729	-0.077642	-1.642648	2.736085	3.906747	-1.788967	2.944963	1.785004	-2.088409	-3.260591	-3.260591	-2.618976	-1.719763	-2.668207	-3.260591	-1.406952
LOC24906	5.825813	-2.116897	4.378982	0.000579	0.007751	-0.354144	-3.260591	1.425638	2.274922	-2.731185	0.770579	0.846031	-3.260591	-2.550306	-3.260591	-1.564464	-0.510232	0.714338	-3.260591	-3.260591
Svil	0.98056	5.284107	4.375458	0.000583	0.007797	-0.721142	4.915133	6.023859	5.860529	4.605497	5.313918	5.745858	5.419857	5.162468	5.239322	5.480124	4.92558	5.288978	4.760756	5.277328
Sh3bp4	-0.636048	6.103113	-4.371586	0.000588	0.007848	-0.782873	6.496765	5.52556	5.619353	6.154831	5.932087	5.68093	6.102389	6.298164	6.247632	6.147209	6.434321	6.327586	6.347787	6.15243
Cdh13	-0.942136	7.186846	-4.37066	0.000589	0.007853	-0.791487	7.227358	5.97968	6.910612	7.660699	6.587294	6.52539	7.323725	7.512312	7.498432	7.517251	7.245909	7.570915	7.333343	7.734504
Tspan33	-0.922368	5.405982	-4.360622	0.000601	0.008001	-0.745251	5.268246	3.747109	4.464755	5.302711	4.820023	4.401612	5.873711	5.774298	6.049883	6.013956	6.027045	5.858236	5.924376	6.20138
Spats2l	-0.741602	4.738396	-4.359873	0.000601	0.008004	-0.73794	5.055231	3.879549	4.371924	5.126698	4.534033	4.600198	4.788531	4.905609	4.790809	4.741628	5.008293	4.901034	4.939655	4.754829
Lingo1	-1.017306	7.97586	-4.356957	0.000605	0.008041	-0.809647	8.163351	6.396186	7.324608	8.128419	7.506719	7.292509	8.173463	8.265328	8.558119	8.228064	8.265871	8.310616	8.502318	8.553441
Adcy4	1.744959	3.992607	4.352516	0.00061	0.008103	-0.467998	2.730329	4.496185	3.814306	2.077719	4.329239	3.862069	3.876582	5.165139	3.563133	5.229048	4.530966	5.37253	2.902922	4.070176
Tmem132d	-0.893429	3.343175	-4.351009	0.000612	0.008118	-0.558166	3.388015	2.466372	3.049621	3.866255	2.619344	2.82781	3.684484	3.559057	3.577725	3.276274	3.499841	3.562264	3.999391	3.591131
Ccpg1	-0.700305	6.584843	-4.347877	0.000616	0.008159	-0.838907	6.870706	6.189194	6.047816	7.166838	6.352176	6.686968	6.598333	6.59119	6.677497	6.6427	6.434321	6.609377	6.564966	6.772403
Bmp4	-0.829471	4.194822	-4.343852	0.000621	0.008215	-0.709045	4.765689	3.780021	3.688616	4.304833	3.935155	3.420773	4.05746	4.455617	4.116621	4.643844	4.32049	4.859513	4.125768	4.342619
Itgb1	0.896229	7.25708	4.342332	0.000623	0.008229	-0.846095	7.284423	7.63115	7.988646	6.735336	7.931589	8.075107	6.971232	6.886541	6.833074	6.985772	6.726681	7.471493	6.908883	7.179877
Zfp385b	-0.833217	5.466824	-4.341881	0.000623	0.008229	-0.824756	5.714798	4.591969	5.110492	6.112053	5.44245	5.174216	5.624957	5.452183	5.538709	5.420347	5.699345	5.387214	5.744907	5.558541
Tcte1	-0.925056	5.270786	-4.340337	0.000625	0.008245	-0.803836	5.637119	4.820078	4.179109	5.4149	4.748146	4.68261	5.967162	5.199424	5.523715	5.752952	5.170732	5.390862	5.467482	5.880498
Pmp2	2.507141	0.003818	4.33948	0.000626	0.008246	-0.125897	-0.634519	1.151583	1.089503	-1.223791	1.981199	1.249022	-0.279766	0.58326	-1.304935	0.348395	-0.616402	0.889587	-0.893866	-0.397871
Napsa	1.95055	0.554042	4.339186	0.000626	0.008246	-0.165167	0.307952	1.894965	2.322392	-0.166914	2.295481	1.165512	0.282279	0.301855	0.512061	-0.116374	-0.411343	0.34469	0.037347	0.190106
Ccr5	1.184335	4.006771	4.337203	0.000629	0.008269	-0.690208	3.963192	4.687583	5.145557	3.462244	4.693063	4.986228	2.73209	4.259584	3.059216	3.814705	3.972332	4.921355	3.220819	3.286911
Csf1	0.981404	5.408697	4.334939	0.000632	0.008297	-0.818505	4.89695	5.633051	6.231543	5.130514	6.085789	6.02688	4.622269	5.575785	4.646003	5.408486	5.240827	5.999929	5.116948	5.145679
Anp32b	0.695757	7.305657	4.333724	0.000633	0.008308	-0.859614	7.293387	8.091501	7.742323	7.506284	8.242482	8.300413	6.801457	7.055875	6.796114	7.190112	6.848923	7.082344	6.547508	6.791001
Zc3h12a	1.581295	2.614954	4.329725	0.000638	0.008358	-0.409864	2.501925	3.643613	3.590238	1.158502	3.458815	2.714852	2.078759	3.000548	1.937064	3.177227	2.883067	3.179156	1.54805	2.030937
Bnip2	0.616724	4.594535	4.329551	0.000639	0.008358	-0.775454	4.539921	5.037557	5.048326	4.263733	5.030587	4.934612	4.693279	4.264575	4.51566	4.386051	4.192699	4.552625	4.364758	4.56506
Bsn	-0.957976	9.414353	-4.328228	0.00064	0.008368	-0.832094	9.038594	7.945918	8.542502	9.819743	8.618058	8.77324	10.04604	9.778057	9.985246	9.621717	9.703625	9.735345	10.18697	10.00852
Dnajc27	-0.828949	7.324274	-4.327861	0.000641	0.008368	-0.872758	7.793851	6.641268	6.863251	7.85424	7.231602	7.245413	7.349987	7.201562	7.344283	7.135992	7.544632	7.226602	7.794812	7.322409
Tmem43	0.767735	5.563926	4.326631	0.000642	0.008379	-0.841273	5.137181	5.551208	6.044281	5.158812	5.985161	6.072488	5.170246	5.5474	5.268848	5.578679	5.455844	5.845	5.455051	5.658531
Fstl3	4.695398	-1.442647	4.325961	0.000643	0.008381	-0.432521	-2.235785	0.934883	1.593793	-3.260591	-0.424712	0.231108	-1.21312	-0.81357	-1.536754	-1.838237	-1.310338	-1.437379	-1.413173	-1.632384
Tc2n	-1.752528	1.368394	-4.324954	0.000644	0.008389	-0.384339	3.035892	1.047298	1.514261	2.41457	0.88224	0.527154	1.297885	0.517885	1.426399	1.419918	2.568656	0.567345	1.316372	1.29285
Tmem38a	-0.708219	5.973678	-4.322718	0.000647	0.008417	-0.856427	5.666549	4.846214	5.216372	5.765863	5.271819	4.697551	6.369857	6.446076	6.636268	6.502814	6.36626	6.496245	6.632759	6.745516
Palb2	1.10558	2.457954	4.321329	0.000649	0.008426	-0.436447	1.9771	3.411272	3.271431	1.945353	2.651221	2.881136	2.266943	2.665313	2.241976	2.558244	2.519368	2.252925	1.922909	2.142093
Car12	-1.366816	5.343937	-4.321097	0.000649	0.008426	-0.825464	5.66348	3.224637	4.983359	5.439714	4.342316	4.116722	6.118774	5.512163	6.421514	5.627248	5.913595	5.287022	5.988622	6.22472
Prickle1	-1.034639	6.96096	-4.316048	0.000656	0.008501	-0.90029	7.056519	5.932887	6.213559	7.811506	6.511893	6.934074	7.086096	7.291717	7.22826	7.061575	7.20704	7.025399	7.16747	6.938811
Kirrel3	-0.77897	6.744154	-4.314226	0.000658	0.008522	-0.903527	6.636389	5.55755	6.213559	6.859496	6.250056	5.853172	7.136588	6.959142	7.303713	7.192419	6.975955	7.05559	7.048993	7.391536
Cmtm3	1.535463	2.713144	4.312424	0.00066	0.008543	-0.404901	1.957182	3.208609	2.977318	1.245516	3.568609	2.634313	1.726334	3.777297	1.959523	3.508843	3.345785	4.046822	1.983515	2.325991
Gnb2l1	0.880688	7.221838	4.310179	0.000663	0.008572	-0.911761	7.019182	7.688975	7.277213	6.138819	7.650338	7.21155	7.243188	7.282521	7.167456	7.470954	7.326474	7.362853	7.022159	7.254965
Junb	0.784442	5.792233	4.307289	0.000667	0.008611	-0.896838	5.658863	6.169663	6.592631	5.321191	6.388878	5.931096	5.578326	5.537125	5.519942	5.713327	5.677044	5.985526	5.65616	5.390475
Mnda	4.898413	-1.945209	4.306773	0.000668	0.008611	-0.272779	-1.642648	0.583632	2.322392	-2.731185	2.348294	2.398644	-3.260591	-1.196868	-3.260591	-0.961779	-2.697527	-2.668207	-3.260591	-2.653844
LOC100910558	-0.95242	4.709424	-4.301431	0.000675	0.008693	-0.857549	5.231406	4.32833	3.719973	5.215592	4.831661	4.244308	4.909618	4.600808	4.881816	5.087444	4.709125	4.680333	4.692807	4.861554
Col13a1	1.701335	1.787535	4.300767	0.000676	0.008696	-0.402607	1.874641	2.924502	2.508341	0.966949	3.217567	3.632543	1.17791	1.393205	0.512061	1.828755	1.605665	1.869448	0.491415	1.556352
Nras	0.724609	4.26264	4.299618	0.000677	0.008704	-0.805607	4.251852	4.704825	5.003522	3.963576	4.951718	4.631657	3.951676	4.213873	3.811751	3.890566	3.972332	4.229042	4.221137	3.96323
Fkrp	-0.704456	6.185862	-4.29917	0.000678	0.008704	-0.930212	6.461851	5.391775	5.939717	6.517348	5.810314	5.994982	6.071857	6.281026	6.378406	6.200917	6.362771	6.486049	6.422655	6.304554
Ezr	1.022192	7.641288	4.297911	0.00068	0.008717	-0.920208	8.336925	8.642935	8.908337	7.187588	8.595878	9.004476	6.848064	7.079432	6.777273	7.191843	7.469174	7.227112	6.744232	6.973669
Ttc7a	0.98509	5.388147	4.295912	0.000682	0.008733	-0.867346	4.940716	5.693323	5.407332	4.263733	5.74903	5.460695	5.660833	5.443407	5.551703	5.748254	5.426066	5.674405	4.890388	5.563542
Mgll	-0.867093	6.116693	-4.295862	0.000682	0.008733	-0.936486	6.871371	5.721836	5.705797	6.266538	5.468031	5.912006	6.223496	6.241073	5.997936	5.975615	6.153577	6.403585	6.422853	6.091411
Cntnap4	-0.625913	4.49156	-4.293658	0.000685	0.008762	-0.820559	4.512886	4.019342	3.767911	4.563778	3.909025	3.980827	5.125	4.342165	4.930947	4.382013	4.493172	4.350161	5.195908	5.38295
P2ry14	1.495633	1.917233	4.287383	0.000694	0.008858	-0.397399	1.310059	3.382986	2.538805	1.188094	2.530726	2.458346	2.100922	1.747064	1.663747	2.184529	1.430105	1.869448	1.493524	1.386135
Cyp4f18	3.175157	-1.004891	4.28658	0.000695	0.008858	-0.229906	-1.052261	1.812527	2.262807	-1.788967	1.143601	0.435061	-1.21312	-1.720374	-1.536754	-1.564464	-2.293564	-1.437379	-1.413173	-1.632384
LOC100151767	0.532649	5.300877	4.28655	0.000695	0.008858	-0.915097	5.105862	5.686107	5.752034	5.060244	5.479914	5.538825	5.286006	5.165139	5.192685	5.226798	5.192513	5.346466	5.047677	5.172115
Slc34a1	2.009811	0.307017	4.283688	0.000699	0.008889	-0.228365	-0.518419	1.679303	1.386086	-0.81875	0.917619	0.809818	0.68572	0.449407	-0.080923	0.280604	0.764165	-0.24119	0.037347	0.321874
Nup43	0.922511	2.748771	4.283423	0.000699	0.008889	-0.615724	2.542593	3.625618	3.4152	2.666599	3.741449	3.321383	2.12275	2.121805	2.364852	2.325762	2.639567	2.461578	2.860463	2.516844
Col11a2	-1.694618	8.887567	-4.2828	0.0007	0.008889	-0.949954	8.814248	7.99872	6.561441	8.409259	6.797148	6.319459	10.46632	9.694771	10.20038	10.23407	9.23566	9.953928	9.457517	10.28881
Rps3	0.787877	7.285102	4.28176	0.000702	0.008889	-0.966781	7.438276	8.105635	7.930954	6.824594	7.957317	7.681861	7.151265	6.889775	7.012283	7.285068	6.888888	6.870065	6.827873	7.138023
Fut7	2.012926	1.193815	4.281636	0.000702	0.008889	-0.249219	-0.634519	1.972946	1.951721	0.105385	1.082564	1.627481	0.357848	2.80888	0.385075	1.986005	2.388236	2.619163	0.182287	0.753927
Tmem88	1.539178	2.334163	4.281589	0.000702	0.008889	-0.482821	2.263247	3.544937	3.447672	1.158502	3.293596	2.504391	1.668148	2.121805	2.024877	2.291729	2.494078	2.170435	1.963595	2.068949
F2rl1	1.491536	-0.712528	4.278587	0.000706	0.008923	-0.254507	-0.410969	0.895377	1.061799	0.041976	1.43972	1.519512	-1.73438	-0.81357	-1.536754	-1.136114	-1.142289	-1.437379	-1.904936	-1.632384
Stat1	0.839713	5.801622	4.278553	0.000706	0.008923	-0.957913	5.538358	6.092064	6.650737	5.605147	6.300345	6.597528	5.283586	5.865813	5.144489	5.692284	5.674232	6.380836	5.250362	5.176474
Tbc1d21	-3.459794	-0.70579	-4.277263	0.000708	0.008937	-0.242072	0.485316	-3.260591	-2.787702	0.932396	-0.831496	-0.750296	0.118118	0.301855	0.091681	-0.311823	1.319276	0.007173	0.037347	-0.505482
Midn	0.692048	6.009589	4.274856	0.000711	0.00897	-0.980066	6.105938	6.627771	7.14855	6.180822	6.574189	6.98976	5.312364	5.697031	5.471927	5.551971	5.579724	5.762926	5.666979	5.490147
Nudt6	0.901313	3.152571	4.27356	0.000713	0.008984	-0.688561	3.09177	4.051149	4.051108	2.768056	3.761059	3.408717	2.979429	2.849305	2.667343	3.100666	3.048553	2.910998	2.838755	2.791492
Hpd	-1.464163	0.762678	-4.272942	0.000714	0.008985	-0.355935	1.957182	0.679782	0.52603	1.405223	0.027932	-0.1418	1.335758	0.04768	0.787836	1.064403	0.676952	0.930264	0.921344	1.445134
Fstl5	-1.30934	5.280144	-4.271737	0.000716	0.008997	-0.956988	5.538358	4.240408	4.498728	6.502703	4.664713	5.44897	5.211572	5.268128	5.291156	5.092395	5.152662	5.357694	5.86521	5.832966
Tmsb10	1.060025	6.624781	4.270632	0.000717	0.009007	-0.989442	6.94401	7.900807	7.189128	5.919306	7.631485	7.229652	5.900809	6.473276	6.170309	6.5964	6.717833	6.479211	5.730244	5.881833
Isy1	0.618264	4.068222	4.27014	0.000718	0.009007	-0.844314	4.036211	4.535265	4.48836	3.888957	4.589928	4.6901	3.734959	4.318363	3.416608	3.95178	3.999546	4.087853	3.621257	3.692011
Stbd1	1.618508	2.0573	4.268974	0.00072	0.009014	-0.462809	1.853246	2.736085	2.456093	1.000693	3.563034	3.22842	1.297885	2.16515	1.843557	1.678822	1.783193	2.100868	1.574559	1.931282
Asf1b	5.38174	-0.645174	4.2687	0.00072	0.009014	-0.418635	-1.642648	1.934482	0.442561	-3.260591	2.411703	1.839907	-1.73438	-0.380636	-0.929891	-0.961779	0.001431	-0.541385	-0.305233	-1.406952
Prr18	-1.21092	3.33146	-4.267468	0.000722	0.009023	-0.630121	3.334978	2.452828	1.775325	3.099251	2.348294	1.563674	4.123906	3.690957	4.245589	4.135691	3.525031	4.285003	4.009141	4.250521
Dysf	1.564327	2.182866	4.267148	0.000722	0.009023	-0.429734	1.541696	2.769246	3.091325	0.860705	2.448452	2.515676	2.703406	1.908813	2.583064	2.558244	1.978199	2.046399	1.700256	2.228598
Mapkapk2	0.603625	5.872576	4.26613	0.000724	0.009032	-0.98354	5.806708	6.100775	6.28344	5.425809	6.281798	6.199504	5.390917	5.919009	5.570064	5.927943	6.121937	6.213771	5.387506	5.61423
Pld4	1.22376	6.10178	4.264637	0.000726	0.009049	-0.965861	5.714798	6.419873	6.098972	4.514991	6.591407	6.195124	5.027123	7.247659	5.257563	7.108151	6.834512	7.629986	5.191633	5.620643
Icam2	1.211858	3.052907	4.262446	0.000729	0.009079	-0.630008	2.937667	3.879549	3.453014	1.856	3.617838	3.34688	3.116285	2.822481	3.289407	2.977621	2.568656	2.827322	2.794335	3.454224
Slc41a3	1.212862	2.48545	4.25732	0.000736	0.009157	-0.512996	1.936985	3.240489	2.578448	1.379803	2.856578	2.714852	2.100922	2.695192	2.430637	2.600561	2.428842	2.656009	2.601568	2.868948
Muc1	1.142804	3.252911	4.257057	0.000737	0.009157	-0.759926	3.303772	4.414697	4.634216	2.943136	3.741449	4.20302	2.361772	3.180315	2.74697	3.249928	3.352837	2.880186	1.943396	2.768575
Ppm1k	-0.768115	4.594181	-4.255435	0.000739	0.009177	-0.94418	4.955851	3.884602	4.549481	5.201152	4.299374	4.495797	4.571588	4.596856	4.670035	4.345154	4.570823	4.709839	4.926852	4.607648
Arhgap25	0.594879	5.00421	4.254789	0.00074	0.009179	-0.967605	4.922855	5.565437	5.335399	4.888381	5.693981	5.405649	4.263083	5.385024	4.37654	5.176396	5.150641	5.465412	4.294734	4.187126
Kcnj4	-1.082379	5.731635	-4.251034	0.000746	0.009238	-0.982933	5.45204	3.822768	4.988886	5.791393	4.614391	4.704965	6.492286	6.242338	6.677497	6.413652	6.107418	6.415274	6.039464	6.51711
Ankrd34c	-1.438742	0.758133	-4.248588	0.000749	0.009274	-0.44066	1.401336	0.854759	0.20995	2.451642	0.649545	0.61372	0.118118	1.020936	0.571595	0.646479	1.319276	0.567345	0.836265	0.321874
Bco2	1.233317	2.23855	4.244532	0.000755	0.009333	-0.568398	2.144973	3.354134	3.253175	1.526009	2.901447	2.634313	1.697535	2.753156	1.717448	2.600561	2.317912	2.252925	1.077784	1.473753
Eprs	0.69038	7.427338	4.244305	0.000756	0.009333	-1.041252	7.01738	7.760121	7.879564	7.441725	7.779144	8.259255	7.169075	7.338026	7.278254	7.253921	6.992416	7.262388	7.258165	7.302605
Nipal4	-1.065924	4.054302	-4.242987	0.000758	0.009348	-0.853054	4.231267	3.418258	2.626517	4.203428	3.644478	3.006419	4.233294	4.604749	4.31255	4.472141	4.204441	4.859513	4.345646	4.704402
Rpl27	0.601557	4.052304	4.242382	0.000759	0.00935	-0.902334	4.17633	4.607333	4.509023	3.800721	4.578921	4.631657	3.797364	3.770297	3.655446	3.867646	4.021838	3.778199	3.743115	3.890799
Car2	-1.027202	7.732348	-4.240927	0.000761	0.009364	-1.043276	8.485771	7.418559	6.601678	7.629358	7.325966	6.784075	7.902866	7.83858	7.864009	8.004677	8.118248	8.148224	8.075069	8.063811
Ctsl1	0.713486	7.382438	4.240561	0.000761	0.009364	-1.050874	7.256682	7.705488	7.861349	6.796651	7.684436	7.709765	6.95231	7.440259	7.097662	7.505236	7.305275	7.890834	6.943619	7.214204
Fxyd7	-0.894479	6.640907	-4.239783	0.000763	0.009369	-1.054454	7.310169	6.675198	6.430819	6.957795	6.087731	5.770798	6.341051	6.847992	6.719227	6.85058	6.920081	6.74025	6.820278	6.51711
Nudt3	-0.467048	7.725191	-4.238922	0.000764	0.009373	-1.046455	7.998643	7.441455	7.456785	7.938812	7.493591	7.61654	7.734546	7.764328	7.78014	7.888154	7.747198	7.800127	7.838876	7.660968
Etv5	-0.834846	6.372963	-4.238541	0.000764	0.009373	-1.05668	6.591063	5.853937	6.001174	7.33582	6.27158	6.389375	6.456206	6.290844	6.337191	6.212333	6.442607	6.397251	6.517933	6.143552
Aldh5a1	-0.58572	7.490958	-4.237391	0.000766	0.009385	-1.050927	7.8442	7.50927	7.160435	8.020756	7.291826	7.4226	7.442931	7.301463	7.351731	7.353685	7.429704	7.471924	7.731433	7.550277
Adcy3	-0.617994	6.756361	-4.236192	0.000768	0.009389	-1.058708	6.50021	5.691883	5.933043	6.672607	6.29699	5.953053	7.436954	7.043588	7.306453	7.050169	6.938947	7.082908	7.277456	7.420767
Sh3d21	1.440028	2.727153	4.236124	0.000768	0.009389	-0.596735	2.474162	3.512664	3.62851	1.273392	2.901447	2.982217	2.415815	3.292072	2.330799	3.223093	2.833517	3.390696	2.060555	2.124152
Nup93	0.78063	4.389342	4.235069	0.00077	0.009398	-0.8959	3.948134	4.57956	4.856079	3.63871	4.424547	4.389395	4.380979	4.895992	4.421726	4.361652	4.467414	4.529644	4.314529	4.319155
Magee2	-1.322555	2.393278	-4.234639	0.00077	0.009398	-0.624889	2.730329	0.583632	1.631973	3.035565	2.308867	1.766229	2.717819	2.753156	2.772565	2.308845	2.707156	2.555581	2.944167	3.031778
Synm	-0.846092	6.742757	-4.231869	0.000775	0.00944	-1.070509	6.745655	6.139334	6.173672	7.47961	6.32031	6.43318	6.872426	7.003849	6.696007	6.639321	6.701353	7.179925	6.976917	7.052268
Cacnb1	-0.520052	7.972877	-4.229831	0.000778	0.009443	-1.060485	8.191862	7.518239	7.806849	8.167011	7.755814	7.559751	8.080323	8.047368	8.239277	8.110354	8.038766	7.917002	8.036257	8.157749
Tmem178a	-0.882322	6.815517	-4.229075	0.000779	0.009443	-1.075497	7.106541	6.057805	6.266853	7.58868	6.519829	7.014334	7.009081	6.717584	6.839143	6.792441	6.690261	6.84834	7.004598	6.976172
Mrc1	2.019917	2.339549	4.228938	0.000779	0.009443	-0.643418	2.342161	3.411272	4.689571	1.615848	3.359399	4.291029	1.297885	1.49863	1.180909	1.875459	1.482482	2.738492	1.28461	2.068949
Slc37a2	1.177956	2.365752	4.228836	0.000779	0.009443	-0.551921	1.80948	2.248512	2.770386	1.327578	3.052318	2.734303	1.862293	2.76729	2.066858	2.668442	2.940364	3.065063	1.902128	2.228598
Ptma	0.665748	6.121156	4.228799	0.000779	0.009443	-1.06943	6.172233	6.531999	6.64433	5.785355	6.970875	6.422728	5.816264	5.86417	5.82983	5.982299	5.960512	6.076676	5.949913	5.71206
Cbx6	-0.51453	7.679202	-4.228651	0.00078	0.009443	-1.068815	7.98303	7.332891	7.478229	7.801968	7.266323	7.438252	7.889001	7.61553	7.747842	7.691731	7.702701	7.752363	8.029985	7.786761
Cxcl13	5.815405	-0.934365	4.224541	0.000786	0.009511	-0.451139	-1.052261	1.558038	2.298852	-3.260591	3.563034	2.086865	-1.450335	-0.511025	-3.260591	-0.806258	-1.142289	-0.541385	-1.218554	-2.653844
Ier3	1.279462	3.068867	4.223281	0.000788	0.009525	-0.783196	3.311637	3.53854	4.204668	2.363612	3.952316	4.600198	2.550752	2.888628	2.127623	3.139454	2.853541	2.931181	2.151407	2.55752
Elf1	0.767437	3.681521	4.220102	0.000793	0.009568	-0.909303	3.881004	4.259983	4.546985	3.480296	4.380852	4.543439	3.038038	3.534475	3.253503	3.463704	3.165302	3.854157	3.134365	3.133933
Cx3cl1	-0.900101	9.335019	-4.220016	0.000793	0.009568	-1.04813	9.314603	7.950443	8.522317	9.527892	8.823057	8.792939	9.329047	9.795511	9.748224	9.889206	9.693853	9.845869	9.711063	9.755622
B3galt5	-0.751226	5.315004	-4.216359	0.000799	0.009628	-1.038925	5.045831	4.313419	4.74506	5.496992	4.559454	4.469923	5.415442	5.722645	5.682347	5.622122	5.725387	5.804547	5.957486	5.892471
Lypd1	-1.352999	4.473889	-4.213396	0.000803	0.009671	-1.024643	4.674093	3.822768	3.665794	5.995551	4.51711	4.14243	4.171806	4.061624	4.953501	4.560545	4.26176	4.285003	4.662235	5.09131
St6galnac3	-1.068497	4.540276	-4.213069	0.000804	0.009671	-1.025873	4.878536	3.649562	3.838998	5.501419	4.39974	4.618632	4.267989	4.576933	4.417676	4.542569	4.518478	4.822175	4.882452	4.719417
Kcnj12	-0.874049	2.803668	-4.212134	0.000805	0.009679	-0.706147	2.843156	1.725091	1.841991	2.916866	2.424057	2.086865	3.190506	3.081965	3.159628	3.019813	3.124682	3.323571	3.32598	3.432519
Slc25a25	-0.582187	5.915922	-4.211663	0.000806	0.009679	-1.094285	6.017339	5.106215	5.628824	5.99241	5.506298	5.441593	6.179914	6.073452	6.045953	6.142443	6.204165	6.158133	6.2617	6.091411
Pcmtd2	-0.609888	6.602815	-4.205666	0.000816	0.009782	-1.124014	7.022181	6.272505	6.547776	7.217492	6.437675	6.78174	6.823283	6.36823	6.615203	6.454602	6.450024	6.284113	6.507652	6.673325
Gpr132	2.40913	-0.811693	4.20515	0.000817	0.009782	-0.366428	-0.760787	1.279848	1.386086	-1.388744	1.463853	0.284862	-1.450335	-0.81357	-1.813089	-1.33445	-0.616402	0.007173	-1.904936	-2.228037
Prkg1	-2.312659	2.167559	-4.204832	0.000817	0.009782	-0.734529	2.947796	-0.103798	1.669168	4.526619	1.285769	2.982217	2.01015	2.555575	1.891068	1.920699	2.374443	2.064785	2.736815	1.931282
Chrm3	-1.184736	6.35939	-4.202004	0.000822	0.009827	-1.118035	6.300288	4.525594	5.469272	6.745945	5.307264	6.035726	6.827443	6.93743	6.762404	6.707058	6.812303	6.891468	7.094928	6.770962
Nlrp3	1.387503	3.009313	4.197508	0.000829	0.009905	-0.736884	2.488111	3.397198	3.47943	1.996424	3.808949	3.713177	2.343298	3.727562	1.891068	3.602051	3.116419	4.376159	2.151407	2.261799
Camk4	-1.185739	4.708556	-4.196407	0.000831	0.009913	-1.073372	5.31007	4.317161	4.210987	5.5248	3.761059	4.659903	4.606864	5.235456	4.646003	4.502215	5.037033	4.594355	5.343866	4.233886

Pip5k1c	-0.466717	9.676689	-4.196079	0.000832	0.009913	-1.073119	9.968738	9.413754	9.578798	9.976875	9.525039	9.509039	9.764926	9.603306	9.722497	9.635669	9.63695	9.575439	9.847582	9.716961
RGD1309821	-1.119716	4.142625	-4.194434	0.000834	0.009931	-1.040126	5.521503	3.894655	3.871277	4.440107	3.736505	3.918424	4.248266	3.575215	3.930199	3.64312	4.026255	4.277141	4.652936	4.35421
Atp11a	-0.687849	7.146327	-4.194169	0.000835	0.009931	-1.14638	7.54513	6.895817	6.492429	7.036666	6.621891	6.40842	7.320777	7.335063	7.234029	7.336574	7.440914	7.572923	7.323608	7.49594
Pinx1	0.627256	3.721367	4.191857	0.000839	0.009967	-0.990872	3.742407	4.382912	4.57175	4.030446	4.614391	4.490087	3.383673	3.252427	3.367065	3.566911	3.288097	3.315916	3.186856	3.031778
Cast	0.488846	6.123209	4.19047	0.000841	0.009985	-1.146824	5.999191	6.418132	6.512395	6.058988	6.466329	6.673176	6.07466	5.796866	5.922774	5.898564	5.861397	6.031821	5.999669	6.033714
Ddx39a	1.198402	4.610621	4.189256	0.000843	0.009998	-0.983651	3.84352	5.33569	4.747236	3.556013	5.028566	4.458273	5.030014	4.532109	4.858103	4.987339	4.661644	4.603816	4.107729	4.866959
Gpr68	-1.025693	3.076465	-4.188417	0.000844	0.009998	-0.784443	3.100876	2.028784	2.508341	3.160244	1.96438	1.943815	2.979429	3.567159	3.584966	3.545408	3.661913	3.890687	3.794763	3.551406
Veph1	-1.306376	1.14545	-4.188338	0.000844	0.009998	-0.573166	2.32672	0.310827	0.307539	1.679737	1.113405	1.165512	1.576217	1.532126	1.180909	0.893396	0.998029	1.252437	1.218908	1.227132
Mmp17	-0.958729	4.628285	-4.187176	0.000846	0.010011	-1.001412	4.399379	2.914655	3.307264	4.48252	4.154906	3.552622	5.256692	5.220124	5.253025	5.089921	5.144558	5.263337	5.509306	5.325228
Mrto4	0.530801	4.758327	4.18564	0.000849	0.010032	-1.098447	4.832812	5.42163	5.397668	4.802503	5.161783	5.407165	4.531353	4.415795	4.461611	4.509637	4.638748	4.426789	4.349488	4.319155
Cst6	-1.30572	5.489216	-4.183509	0.000853	0.010064	-1.112654	5.851906	3.33211	4.608118	5.492552	4.5954	4.876712	5.636383	5.860878	6.225794	6.051304	5.902825	6.064165	6.381021	6.013146
Syt13	-0.835894	5.716755	-4.182503	0.000854	0.010067	-1.134707	5.586154	4.646529	5.107108	5.880183	4.822358	5.012356	5.86078	6.142854	6.147259	5.759192	5.989079	6.132215	6.593601	6.38774
Tubb3	-0.773472	8.237547	-4.182369	0.000855	0.010067	-1.145215	8.617518	7.438022	8.105615	8.71185	8.045416	7.981049	8.239885	8.147348	8.492731	8.078271	8.512307	8.143096	8.511779	8.306112
Slc10a3	0.841272	3.997223	4.178851	0.00086	0.010128	-0.976193	3.759759	4.673054	4.454139	3.361953	4.457973	3.984893	4.05746	3.798099	4.007682	3.95178	3.848089	4.014082	3.689548	4.003043
Ggta1p	1.247443	2.74212	4.17597	0.000865	0.010169	-0.86863	3.230989	3.61957	3.902849	2.350586	4.078813	4.40465	1.443753	2.471744	1.794428	2.40749	2.506778	3.38339	1.54805	1.529341
March3	1.329885	2.715223	4.175808	0.000866	0.010169	-0.729212	1.936985	2.790938	3.8018	1.996424	2.95351	3.502454	2.67414	2.76729	2.127623	2.572488	2.792614	2.960934	2.66462	2.721621
Rela	0.701936	5.672106	4.172364	0.000872	0.010229	-1.169634	5.841078	6.289749	6.47678	5.282277	6.282646	5.985882	5.219191	5.478195	5.129711	5.566277	5.544742	5.771347	5.198041	5.375386
Dab1	-0.574569	5.539764	-4.17084	0.000874	0.01025	-1.180253	5.950908	5.544839	5.740067	6.299956	5.385155	5.537442	5.309988	5.445606	5.323984	5.382447	5.588712	5.365131	5.476296	5.240329
Oscar	2.835232	0.734706	4.169134	0.000877	0.010275	-0.456785	0.369528	2.411416	2.761803	-1.075778	2.640673	1.327961	0.028455	1.112357	0.091681	-0.311823	-0.149817	0.84773	0.546019	0.887553
Serpinh1	1.295074	6.538624	4.165607	0.000883	0.010337	-1.195675	6.578895	6.966622	6.726579	5.438176	7.866036	7.634444	6.206988	6.254932	6.00335	6.56112	6.349613	6.596798	6.033505	6.341903
Tjp3	-0.937163	4.399974	-4.162615	0.000889	0.010389	-1.026984	4.432216	3.091129	3.859257	4.098255	3.252623	3.061358	4.974076	4.840254	5.099692	5.03183	5.29125	4.761475	4.81457	5.07972
Evc2	1.096652	2.583109	4.161046	0.000891	0.01041	-0.734386	2.10932	2.982209	2.812554	1.63746	3.020129	2.957603	2.550752	3.012464	2.00342	3.080873	2.519368	3.153617	2.041677	2.55752
Rasd1	-1.123691	5.288893	-4.160618	0.000892	0.01041	-1.1681	5.630846	3.752647	4.734132	5.782933	4.822358	4.998346	5.526049	5.698876	5.80524	5.307785	5.778723	5.218879	5.692921	5.338905
Ppapdc1b	0.823873	4.680894	4.158619	0.000896	0.010441	-1.104318	4.485336	5.210498	4.977811	3.997398	5.322193	4.717236	4.855242	4.303891	4.793937	4.677181	4.489977	4.485971	4.463807	4.814782
Arhgap18	1.210238	3.792274	4.157922	0.000897	0.010445	-1.02701	4.180631	5.267699	4.544485	2.951788	4.730847	4.455345	3.706333	3.126505	3.613573	3.368956	3.013384	3.300483	3.270309	3.685908
Nol12	0.935092	3.255203	4.156339	0.0009	0.010468	-0.926003	3.171726	3.49955	4.191945	2.738361	4.109733	3.653128	2.893159	3.024282	2.95129	3.249928	3.324422	3.018661	2.994118	2.921834
G6pd	0.702486	7.350181	4.154776	0.000903	0.01049	-1.222283	7.227358	8.04374	7.578778	6.975934	7.983899	7.60536	7.184724	7.356842	7.288269	7.33918	7.112051	7.10584	6.874625	7.235773
Pdzd2	-0.761505	5.369159	-4.154328	0.000903	0.01049	-1.200786	5.77577	4.938996	5.615785	6.004935	4.85009	5.103056	5.647719	5.225253	5.544292	4.946908	5.257831	5.009416	5.740523	5.546803
Tead3	1.130013	3.041917	4.152971	0.000906	0.010504	-0.882445	2.821286	4.167656	3.993619	2.41457	3.273255	3.491064	3.083269	2.91426	2.539002	2.934159	2.968181	2.816513	2.522078	2.847239
Apc2	-0.498333	8.157683	-4.152664	0.000906	0.010504	-1.218077	8.023997	7.490336	7.475607	8.063044	7.470698	7.744652	8.556397	8.376692	8.526031	8.374721	8.286272	8.501335	8.638508	8.685087
Naca	0.48456	7.850459	4.15144	0.000909	0.01052	-1.212187	8.03354	8.520062	8.421358	8.085085	8.588694	8.64342	7.41556	7.521202	7.508463	7.609935	7.511538	7.401792	7.319695	7.333187
Syt2	-1.065101	2.856489	-4.150266	0.000911	0.010525	-0.89533	3.109925	2.279532	2.345554	3.572936	2.702832	1.875381	3.094359	2.795149	3.130231	2.572488	2.580719	3.292705	3.278395	3.591131
Tgfb2	-0.882324	4.86007	-4.150207	0.000911	0.010525	-1.181893	5.785686	4.298352	4.845962	5.169977	4.439837	4.767664	4.547582	4.789219	4.585917	4.738473	5.162729	4.983074	4.893023	5.088999
Cds1	-0.790615	5.557537	-4.149507	0.000912	0.010528	-1.204447	5.480228	4.619507	5.039476	6.073925	5.034621	5.257237	5.692193	5.583794	5.832874	5.625481	5.749633	5.940188	5.91533	5.995984
Kalrn	-0.821979	8.616398	-4.148796	0.000913	0.010528	-1.207187	8.70652	7.720755	8.271068	9.032878	7.742312	8.458432	8.922716	8.959774	8.82216	8.716037	8.840751	8.738299	8.991781	8.710297
Hcn2	-0.665101	7.046144	-4.148618	0.000914	0.010528	-1.237796	7.085479	6.415516	6.225313	6.784595	6.450564	5.991955	7.51846	7.321358	7.407422	7.568785	7.227805	7.653333	7.433592	7.57484
Epha2	1.342548	1.866185	4.145698	0.000919	0.010574	-0.669266	1.669604	2.790938	2.877559	0.860705	2.386673	2.160607	1.913281	1.49863	2.024877	2.007132	1.557647	1.760874	1.436856	1.634478
Ascl1	-0.689859	2.456227	-4.145482	0.00092	0.010574	-0.905136	3.045357	2.747223	2.466695	3.322471	2.484288	2.3616	2.144252	2.364261	2.024877	2.499819	2.580719	2.529342	2.11575	1.991897
Gpc3	-2.37008	3.610934	-4.144397	0.000921	0.010587	-0.930349	4.069061	-0.366503	2.379614	4.452353	2.226624	3.268997	3.975863	4.576933	4.453722	4.696819	3.930525	4.594355	3.817137	4.748987
Slc16a2	-1.592298	4.310637	-4.143028	0.000924	0.010602	-1.152021	5.586154	2.354284	3.926082	5.136218	4.173318	4.53237	4.074361	4.438054	4.530738	3.973412	4.63586	4.153748	4.467348	4.458122
Aifm3	-1.191619	6.045243	-4.142705	0.000925	0.010602	-1.233445	6.727396	5.976134	4.850017	6.072934	5.133851	4.907024	6.851336	5.78128	6.523963	6.259316	6.517471	6.003508	6.381959	6.674096
Eef1g	0.535575	7.684914	4.138655	0.000932	0.010673	-1.247439	7.78187	8.18432	7.959854	7.458517	8.200907	8.278222	7.383711	7.510213	7.370709	7.677387	7.501658	7.575731	7.283997	7.429468
Egr1	-1.440444	6.064252	-4.138371	0.000933	0.010673	-1.254103	6.893156	4.779965	6.305016	7.605226	5.823197	6.308957	5.77033	6.049074	6.02081	5.71011	6.06511	5.966095	6.189572	5.438452
RGD1311458	1.017836	2.553044	4.136417	0.000936	0.010697	-0.844672	1.895723	3.091129	3.259286	2.568755	3.188895	3.468012	2.501617	2.68033	2.066858	2.239124	2.592682	2.772447	1.747621	1.951771
Slc4a10	-0.953517	7.388178	-4.136293	0.000936	0.010697	-1.25852	7.40236	6.327345	7.061616	7.971086	6.417736	7.121627	7.698209	7.742583	7.814429	7.293691	7.49729	7.321879	7.92316	7.851575
Alyref	0.681249	5.094373	4.135069	0.000939	0.010713	-1.213485	4.915133	5.533624	5.474535	5.036039	6.004828	5.614253	4.725734	5.048455	4.956296	4.827276	5.130266	4.864769	4.662235	4.575
Slc31a2	0.877991	3.136134	4.134615	0.00094	0.010713	-0.91146	2.957854	3.563961	3.420663	2.297273	3.601614	3.34688	2.918339	2.939445	2.905753	3.409193	3.31723	3.461789	2.860463	3.088394
Rhobtb2	-0.551946	5.739703	-4.133189	0.000942	0.010733	-1.256991	6.005266	5.519076	5.733497	6.299109	5.564652	5.586428	5.61921	5.819086	5.570064	5.560929	5.746959	5.781109	5.878569	5.701512
Snx20	1.530072	1.614696	4.13233	0.000944	0.010741	-0.602123	0.995438	2.279532	1.951721	0.166125	2.496038	1.450626	1.297885	2.14364	1.29127	2.256873	2.066633	2.330954	1.252133	1.193114
Tmem14a	-1.060349	2.357786	-4.131237	0.000946	0.010749	-0.834361	2.853968	1.184734	1.631973	2.728325	1.803478	2.323579	2.629097	2.454377	2.493553	2.423294	2.718119	2.461578	3.03287	2.59708
Cxcr4	2.171064	2.535418	4.13102	0.000946	0.010749	-0.680736	1.594228	2.425352	3.209656	0.485009	3.726565	3.106838	2.266943	3.070611	1.959523	2.807409	2.259075	3.179156	2.58862	3.142871
Ephb1	-0.900181	6.074213	-4.128987	0.00095	0.010782	-1.266642	6.426071	5.741464	4.839859	6.372633	5.6394	5.79647	6.021912	6.402594	6.123835	6.347893	6.244533	6.707714	6.20344	6.196023
Pitpnm2	-0.734708	9.197773	-4.124908	0.000958	0.01086	-1.244605	9.066605	8.496533	8.557923	9.317971	8.297234	8.475871	9.748238	9.637852	9.741151	9.776139	9.365208	9.460805	9.143078	9.687102
Ltb	2.354275	0.505724	4.124175	0.000959	0.010866	-0.563016	0.428583	2.36878	2.718102	-0.502853	2.553396	1.107027	-0.067151	0.04768	0.17081	0.055728	-0.231835	0.007173	-0.305233	0.045082
Mbd2	0.456608	5.999952	4.123156	0.000961	0.010877	-1.283348	6.139466	6.432004	6.653639	6.073925	6.628579	6.53582	5.647719	5.803739	5.546148	5.799108	5.907622	5.785273	5.581336	5.490147
Eif2ak2	0.778853	5.308048	4.118839	0.00097	0.010961	-1.250839	5.19147	5.686107	6.101525	4.881605	5.604547	5.847601	5.055772	5.285418	4.787674	5.437958	5.132316	5.764333	4.683703	4.89368
Lat2	1.636003	2.976029	4.113822	0.000979	0.011061	-0.857635	2.144973	3.486315	3.431527	1.760745	4.094356	3.22842	1.862293	4.49437	1.867508	3.62956	3.786926	4.588013	1.316372	2.228598
Thsd7a	-1.405557	4.025965	-4.111322	0.000984	0.011102	-1.119181	3.759759	3.055728	3.447672	5.086016	2.682409	2.965855	4.263083	4.643578	4.762349	3.896239	4.095154	4.353904	4.994677	4.468825
Furin	0.606327	6.573562	4.110778	0.000985	0.011102	-1.307968	6.225243	6.713326	6.865256	5.92371	6.599599	6.53928	6.544769	6.596148	6.663029	6.656138	6.51433	6.929587	6.530682	6.745516
Ckmt1b	-0.913646	8.031101	-4.110544	0.000986	0.011102	-1.292819	8.243654	7.266702	7.410858	8.900605	8.146263	7.803098	8.117828	8.069281	8.291112	7.970463	8.147181	7.901742	8.071584	8.101263
Rpl10	0.783929	5.603123	4.108464	0.00099	0.011137	-1.261266	5.19147	5.781293	5.603828	4.563778	5.837122	5.394994	5.900809	5.684052	5.700795	6.050032	5.812363	5.912539	5.526381	5.517894
Slc4a4	-1.024816	5.713625	-4.105308	0.000996	0.01119	-1.313605	6.642638	5.230479	5.861534	6.280342	4.970791	5.673392	5.712128	5.499519	5.661948	5.174062	5.62557	5.755871	6.301919	5.631797
Adi1	-0.793735	4.518345	-4.105156	0.000996	0.01119	-1.261526	5.392103	4.184143	4.172648	5.184732	4.677668	4.942996	4.362751	4.161323	4.308181	4.602796	4.414477	4.342647	4.24196	4.338735
MGC105649	4.883664	-0.994815	4.101929	0.001003	0.011252	-0.604449	-3.260591	0.812964	2.726949	-0.411255	2.198134	2.559953	-2.558588	-0.992527	-2.155202	-1.136114	-1.310338	-0.541385	-1.904936	-2.228037
Syngr1	-1.231237	6.222491	-4.101062	0.001005	0.011261	-1.326853	6.702936	4.822713	6.134314	6.91751	5.612665	5.728989	6.19287	6.228357	6.40422	5.927943	6.541586	6.515588	7.043073	6.365003
Car14	-2.142524	3.57722	-4.098508	0.00101	0.011298	-1.082478	5.582908	2.758277	2.250588	3.348912	2.702832	1.585258	3.337752	3.891392	3.416608	4.620041	4.896921	4.335093	3.903318	3.62342
Synpr	-1.705281	5.628205	-4.098487	0.00101	0.011298	-1.276763	5.298244	2.801663	4.472666	6.380664	4.090486	5.119791	6.407022	5.93475	6.534286	6.343747	6.100103	6.223009	6.491223	6.644508
Smco4	-1.171584	1.927965	-4.095306	0.001016	0.011359	-0.795602	2.091157	0.533043	1.408255	2.310787	0.952152	1.302125	2.450751	2.603631	2.364852	2.558244	2.360517	2.064785	2.522078	1.931282
Robo1	-1.026476	5.857954	-4.094548	0.001018	0.011365	-1.327608	6.044731	4.798824	5.301747	6.389448	4.995836	5.66074	5.918076	6.311488	6.111352	5.603168	6.129142	6.013007	6.642972	6.119982
Gabra4	-1.220362	5.80379	-4.09401	0.001019	0.011367	-1.323033	5.723667	4.256089	5.307656	6.660783	4.743225	5.564861	5.961117	6.201283	6.269144	5.945834	6.230245	6.098034	6.2617	6.061084
Clec4a	2.189172	-0.060712	4.092566	0.001022	0.011379	-0.567934	-0.760787	0.533043	0.945338	-1.575022	0.606834	0.949514	0.028455	0.645799	-1.10526	1.21727	0.805871	1.315214	-1.638202	-0.748265
Nthl1	1.238017	2.88857	4.092563	0.001022	0.011379	-0.963904	2.742017	4.163505	3.104962	2.013053	3.721569	3.414757	2.67414	3.081965	2.347926	3.080873	2.86345	2.738492	2.301782	2.417223
Necab1	-1.554892	4.786541	-4.089261	0.001028	0.011433	-1.31784	5.441326	3.996186	5.028784	6.744701	4.703236	4.413727	4.085519	4.870031	4.815644	3.896239	4.935008	4.365074	5.250362	4.528015
Arhgef25	-0.542411	7.870173	-4.089251	0.001028	0.011433	-1.352904	7.757244	7.027849	7.075103	7.571591	7.357709	7.030159	8.435429	8.296424	8.388992	8.307653	8.330254	8.11883	8.176713	8.315777
Jtb	0.501167	5.420662	4.088476	0.00103	0.01144	-1.315799	5.172161	5.522322	5.62528	5.128607	5.604547	5.846484	5.31947	5.328951	5.368723	5.533887	5.486624	5.465412	5.260602	5.263052
Tlr6	1.143387	1.434699	4.085643	0.001036	0.011494	-0.763748	1.212615	2.117298	2.31067	1.097434	2.424057	2.203108	0.563361	1.532126	0.512061	1.569444	1.068256	1.930875	1.040235	1.122575
Reln	-0.780607	6.51633	-4.08486	0.001038	0.011502	-1.360784	6.764401	6.10186	5.945414	6.376654	5.475469	5.641552	7.075642	6.723033	6.763777	6.597271	6.771443	6.518926	7.16747	7.324375
Rnaseh2b	0.764229	3.597675	4.084025	0.001039	0.01151	-1.164856	3.875708	4.317161	4.075556	3.268089	4.578921	4.313834	2.979429	3.458111	3.333065	3.28495	3.359854	3.376046	3.212403	3.069767
Rpl15	0.870029	6.305866	4.083403	0.001041	0.011514	-1.351819	5.899649	6.783985	6.432172	5.365572	6.778025	6.001017	6.403683	6.209071	6.432594	6.594657	6.517471	6.375322	6.195989	6.313488
RGD1563349	-0.778342	6.180685	-4.082797	0.001042	0.011517	-1.366271	6.461851	6.045451	5.768195	6.831642	5.609964	6.058089	6.33171	5.829227	6.217664	5.907019	5.8956	6.39089	6.609712	6.594891
Slc8a2	-1.344159	8.694575	-4.081137	0.001045	0.011545	-1.346945	8.4718	6.63828	7.646203	9.244945	7.471814	8.298781	9.21031	9.395075	9.504267	9.239158	9.022676	9.12213	9.15287	9.310468
Ezh1	-0.527744	6.989789	-4.080258	0.001047	0.011554	-1.375998	7.043	6.508461	6.721054	7.350616	6.810737	6.635525	7.002473	7.163326	7.087501	7.008868	7.156554	7.015981	7.212476	7.153032
Apol9a	1.822575	2.095236	4.073691	0.001061	0.011696	-0.809092	1.514695	3.151065	3.376367	0.860705	2.348294	2.924117	1.607516	2.926907	1.360416	2.707689	1.903329	3.065063	0.698429	1.32462
Lmnb1	0.92802	3.795565	4.071461	0.001066	0.011733	-1.173899	3.417469	4.766343	3.851187	3.450082	4.361713	4.490087	3.727855	3.591194	3.716062	3.808701	3.512491	3.656194	3.333761	3.571405
Cdh9	-1.258339	5.811959	-4.071202	0.001066	0.011733	-1.359954	5.312032	4.220564	4.703085	6.694681	4.76524	5.298657	6.304513	6.312693	6.540818	6.21574	5.965121	6.112466	6.384771	6.570256
Dnajc22	2.208064	-0.114835	4.070277	0.001068	0.011743	-0.608836	-0.310971	0.895377	0.715742	-1.575022	1.600747	0.881356	-0.831021	0.449407	-1.536754	0.20947	0.071497	0.714338	-0.755361	-0.116166
Cxcl12	-1.137501	7.433347	-4.067912	0.001073	0.011785	-1.395533	7.548461	5.751179	6.51814	7.835663	7.220579	6.725141	7.734103	7.728203	8.058944	7.786807	7.614873	7.970091	7.667836	7.917187
Gng7	-0.89211	7.497707	-4.067603	0.001074	0.011785	-1.395872	7.994069	6.554366	6.675511	7.497078	7.087595	7.102576	7.944825	7.600313	7.61355	8.010901	7.367525	8.011554	7.648471	7.868849
Gabarapl1	-0.641015	8.78993	-4.065728	0.001078	0.011818	-1.359403	9.187336	8.440593	8.732942	9.393741	8.613683	8.811802	8.817066	8.660342	8.817749	8.621801	8.95449	8.554142	8.918653	8.538278
Relt	1.065038	3.142482	4.06433	0.001081	0.01184	-1.019202	2.501925	3.613496	3.759312	2.310787	3.391209	3.053636	3.211029	3.542716	2.720913	3.440594	3.173291	3.721386	2.652228	3.088394
Cnnm1	-0.731668	7.311645	-4.062845	0.001084	0.011865	-1.409579	7.138951	6.28211	6.972365	7.619211	6.529152	6.802621	7.758708	7.669208	7.757888	7.481843	7.449163	7.589685	7.578976	7.743713
Pcsk2	-1.213963	7.77717	-4.061804	0.001086	0.011877	-1.404361	7.760839	5.790744	7.245626	8.18936	7.013908	6.990266	8.101088	8.246848	8.505905	7.987173	8.123669	8.065048	8.414935	8.453485
Mad2l2	1.014308	3.653049	4.061397	0.001087	0.011877	-1.128251	3.42474	4.302134	3.910635	2.613062	4.230707	3.606391	3.554005	3.698353	3.525993	3.796617	3.525031	3.854157	3.4385	3.791988
Ppp2r1b	1.043219	4.807502	4.060455	0.001089	0.011883	-1.306996	4.369549	5.883263	5.354078	4.14804	5.226692	4.734242	4.656342	4.632039	4.670035	4.885482	4.624251	4.978233	4.334055	4.866959
Ninj2	-1.11803	3.582622	-4.060268	0.00109	0.011883	-1.180471	4.350588	3.309744	2.487668	3.654695	3.339971	2.458346	3.445603	3.784265	3.91304	3.832569	3.70112	4.023513	4.107729	3.890799
Klk6	1.552443	6.031787	4.055391	0.0011	0.011976	-1.393643	5.386529	7.156135	5.980053	5.134319	7.61373	6.485772	4.791941	6.513661	5.454242	6.365383	5.752302	6.669931	5.497232	5.670943
Cdk5r2	-0.820969	7.363535	-4.055179	0.001101	0.011976	-1.42308	7.662404	6.175859	6.778562	7.478114	6.811325	7.233502	7.462321	7.667796	7.664851	7.657481	7.676945	7.663548	7.572415	7.594519
Foxm1	0.92243	3.235172	4.054764	0.001102	0.011976	-1.088305	3.082606	3.889638	3.365078	2.363612	3.789984	3.468012	3.148563	3.115498	3.110295	3.040455	3.288097	3.454835	3.134365	3.212449
Cdh10	-0.566359	5.864197	-4.05452	0.001102	0.011976	-1.407419	5.832904	5.159298	5.227324	6.034761	5.445483	5.641552	6.20187	6.064895	6.270267	5.990279	5.950088	6.074409	6.113648	6.119982
Daam1	-0.542519	6.91033	-4.052995	0.001106	0.011991	-1.430952	6.795581	6.520683	6.495021	7.278944	6.445271	6.519096	7.269721	7.074467	7.040211	7.027083	6.915326	7.091901	7.139444	7.145269
Gjc2	-1.604011	5.498332	-4.052615	0.001106	0.011991	-1.35962	5.629274	4.256089	2.861581	5.624215	5.040651	4.028875	5.799422	6.329461	5.944048	6.347893	5.832682	6.701118	6.215071	6.416587
Selplg	-1.403743	5.46832	-4.052525	0.001107	0.011991	-1.391442	6.29236	4.077865	4.866124	5.676041	4.977093	4.377073	5.286006	6.186896	5.534975	5.472549	5.560056	6.651042	5.45148	6.187409
Diras1	-0.901037	6.595133	-4.051246	0.001109	0.012011	-1.432477	7.165233	5.85908	6.46955	7.578242	6.618536	6.931969	6.329365	6.461371	6.501179	6.378616	6.576644	6.350247	6.651557	6.477031
Neurod2	-1.079817	6.465542	-4.048256	0.001116	0.012061	-1.432005	6.490716	4.876964	5.696819	6.801446	5.425653	6.254426	6.625343	6.98583	6.909326	6.821804	7.129607	6.878535	7.035937	6.605437
Dok1	1.648238	1.853395	4.047183	0.001119	0.012061	-0.82357	1.874641	2.747223	2.390791	0.105385	2.496038	2.537984	1.17791	2.68033	1.360416	2.291729	1.903329	2.668085	0.836265	1.386135
Ppp1r1b	-0.863764	6.834178	-4.04691	0.001119	0.012061	-1.441323	7.727053	6.850699	6.656536	7.019794	6.395166	6.145135	6.575761	6.804963	6.635396	7.245056	7.324682	7.037668	6.625646	6.649221
Magee1	-0.909467	6.23713	-4.046625	0.00112	0.012061	-1.43378	6.248988	4.899604	5.626462	6.687576	5.844035	5.857613	6.650919	6.376322	6.883865	6.171976	6.432658	6.155991	6.857186	6.649221
Dusp9	-0.873282	2.123841	-4.046492	0.00112	0.012061	-0.972414	2.63325	1.679303	1.613009	2.43939	1.877213	1.541762	2.361772	1.934092	2.822431	1.755735	2.259075	1.930875	2.771602	2.489076
Phf5a	0.801556	3.896239	4.046199	0.001121	0.012061	-1.247003	3.695089	4.535265	4.229782	3.515737	4.745687	4.101824	3.616879	3.878428	3.83015	3.709073	3.949256	3.686193	3.766298	3.395603
Thy1	-0.762223	9.958484	-4.046028	0.001121	0.012061	-1.371163	10.17789	8.914918	9.419434	10.12921	9.740319	9.498768	9.942738	10.20894	10.24534	10.18964	10.11907	10.25816	10.3301	10.24589
Madd	-0.574278	7.764338	-4.044576	0.001124	0.012085	-1.443427	7.374471	7.00483	7.258267	7.880249	6.988659	6.958055	8.354435	8.089813	8.266835	7.943316	8.062885	8.009479	8.302471	8.214757
Rab11fip4	-0.774757	6.904958	-4.043824	0.001126	0.012093	-1.447997	7.331117	6.738662	6.806478	7.351843	6.031314	6.692572	7.100606	6.903438	6.912927	6.743709	6.975955	6.934594	7.284499	6.875203
Bst1	2.605398	0.076069	4.042897	0.001128	0.012098	-0.681384	0.176305	1.151583	2.626517	-0.941548	2.496038	1.803538	-0.831021	-0.048075	-0.175642	-0.806258	-0.231835	0.151783	-0.405292	-1.899685
Ercc6l	3.194092	-0.592781	4.042719	0.001129	0.012098	-0.669336	-1.908947	1.894965	-0.067513	-1.575022	1.285769	1.378286	-1.21312	-0.26106	-0.929891	-0.961779	-0.616402	-0.434228	-1.413173	-0.621773
Atp2b4	-1.260205	5.469775	-4.041078	0.001132	0.012127	-1.430881	5.72955	5.142523	5.618164	6.779137	4.352047	4.870133	5.722886	5.326567	5.781799	4.744776	5.278346	5.355829	6.122658	5.790939
Epsti1	1.081292	3.21573	4.039444	0.001136	0.012155	-1.128023	2.799078	3.696289	3.330668	2.687469	4.070978	4.17126	2.286414	4.166665	1.981639	4.046664	3.760656	4.372473	1.881043	1.971974
Fam129a	1.492301	1.502348	4.031923	0.001153	0.012328	-0.877062	1.143792	2.324846	2.924461	1.158502	2.335271	2.809587	1.091998	1.68885	0.787836	0.938085	1.135223	2.330954	0.546019	0.442608
Wdr46	0.897417	6.132549	4.031379	0.001154	0.012329	-1.452975	5.621385	6.83708	6.666342	5.464109	6.261288	5.988921	6.411463	6.153655	6.185871	6.321781	5.970862	5.990343	5.785187	6.220504
Fam222a	-0.92762	2.669754	-4.030998	0.001155	0.012329	-1.163029	3.562947	2.679063	2.61703	3.361953	2.448452	2.422822	2.501617	2.007376	2.493553	2.558244	2.288794	3.18757	2.66462	2.83626
Ninj1	0.948726	4.410141	4.030073	0.001157	0.012341	-1.333151	4.439412	4.866787	4.928811	3.50989	5.283731	4.53237	3.908348	4.388622	4.13647	4.528025	4.534071	4.523011	3.98464	4.25465
Scnn1a	1.575877	0.936534	4.0282	0.001162	0.012376	-0.773177	0.643243	2.279532	2.025285	0.041976	1.705671	1.378286	0.202532	1.49863	0.091681	1.322149	1.319276	1.869448	-0.628995	0.321874
Syt10	-1.940668	0.858245	-4.027344	0.001164	0.012382	-0.83268	1.341131	0.24948	0.679743	2.534609	-0.954037	0.481842	1.258992	0.871929	1.663747	0.981432	1.199219	0.969826	1.493524	0.257494
Eef1d	0.567661	6.192245	4.027106	0.001164	0.012382	-1.473387	6.031695	6.71758	6.308704	5.846944	6.666117	6.332281	6.040665	6.118252	6.118855	6.234897	6.175617	6.163475	5.875907	6.082147
Elf4	2.913114	0.966501	4.025063	0.001169	0.012421	-0.771513	0.643243	2.167912	4.008208	-0.411255	2.308867	2.965855	-0.279766	1.112357	-1.304935	1.023513	0.764165	1.760874	0.249639	-0.297732
Rps24	0.779608	7.058366	4.021539	0.001177	0.012498	-1.484888	7.576884	8.478684	7.765049	7.252927	8.356243	8.172124	6.454055	6.419473	6.33182	6.854229	6.368871	6.436609	6.150458	6.213097
Agpat2	3.260957	-0.233616	4.020639	0.001179	0.012509	-0.696194	0.785575	1.184734	1.7408	-2.731185	2.014259	1.541762	-1.73438	-0.150641	-1.536754	0.055728	0.321999	-0.434228	-0.628995	-0.748265

E2f7	1.887501	-0.523647	4.018219	0.001185	0.012551	-0.727017	-0.310971	1.632013	0.642822	-0.502853	1.533923	1.72793	-1.73438	-0.81357	-1.10526	-0.806258	-1.142289	-0.070959	-1.904936	-1.632384
Cntn4	-1.143137	4.460388	-4.01808	0.001185	0.012551	-1.388987	4.227114	3.696289	3.570714	5.645703	3.836938	4.146066	4.714996	4.477275	4.746291	4.461334	4.467414	4.619447	4.962418	4.943127
Perp	-2.057194	3.512286	-4.01759	0.001187	0.012553	-1.225311	3.549707	0.632508	2.423812	5.025832	3.159643	2.743931	3.200804	3.985077	4.321248	4.201325	3.911548	4.140807	3.944552	4.129836
Slc43a3	1.192715	3.122023	4.017062	0.001188	0.012555	-1.127687	2.765113	3.817493	3.661186	2.169587	3.585206	3.479584	2.73209	3.638097	2.524011	3.417108	2.802949	3.99982	2.575555	2.733504
Agt	-1.019684	7.235001	-4.016604	0.001189	0.012555	-1.502139	7.608753	6.464425	6.472841	6.861793	6.269869	5.663279	7.58778	7.773985	7.711086	7.856127	7.317491	7.945497	7.714026	8.055255
Opalin	-1.229571	7.546576	-4.014474	0.001194	0.012598	-1.502216	7.776907	7.230449	6.172053	7.721725	6.912338	5.77315	7.828026	8.007734	7.84189	8.00599	7.793283	8.366107	7.988076	8.244386
Epb41l3	-0.931441	7.422753	-4.013812	0.001196	0.012603	-1.502826	7.609152	6.307729	6.800729	8.071504	7.405218	7.118855	7.582867	7.537272	7.720568	7.336574	7.581869	7.434857	7.828932	7.592074
Ugt8	-1.502315	6.617843	-4.008504	0.001208	0.012727	-1.514472	6.973435	5.477878	4.74506	7.435955	6.563759	6.034746	6.703467	6.875166	6.788294	6.572681	6.621857	7.295363	7.285502	7.295608
Ucn2	5.448062	-2.263636	4.007248	0.001211	0.012747	-0.91418	-3.260591	1.369077	1.809043	-2.731185	1.143601	-0.371994	-2.088409	-3.260591	-3.260591	-1.564464	-2.293564	-2.668207	-2.656794	-1.212031
Cd2	5.512344	-2.029455	4.00583	0.001215	0.012773	-0.87286	-1.908947	0.480615	2.645305	-3.260591	1.685287	1.327961	-2.558588	-2.076466	-2.155202	-3.260591	-1.500575	-2.668207	-3.260591	-1.632384
Inppl1	0.679266	6.484854	4.003113	0.001221	0.012823	-1.526243	6.462734	7.141963	6.702112	5.886966	6.85304	6.711711	6.311657	6.508411	6.133744	6.573567	6.385299	6.663159	6.12378	6.347713
Phlda2	-2.132242	0.283582	-4.002586	0.001223	0.012823	-0.793773	1.033981	-1.685466	-0.33572	1.615848	0.091273	-0.646023	-0.169545	0.76329	1.061401	-0.027758	1.230182	0.218993	1.28461	1.122575
Fam83f	2.563077	0.588671	4.002538	0.001223	0.012823	-0.729122	-0.410969	2.134368	0.818644	-1.788967	1.018831	0.809818	0.563361	1.239467	0.385075	1.51148	1.199219	1.252437	-0.123798	1.009844
Mpi	0.527679	5.717701	3.999081	0.001231	0.012901	-1.514508	5.379063	5.967826	5.834161	5.629617	6.25179	6.079159	5.599886	5.715373	5.66878	5.608362	5.582726	5.602291	5.612053	5.546803
Ppp1r13b	-0.464106	7.332331	-3.998277	0.001233	0.01291	-1.539191	7.316045	6.910135	7.109759	7.533916	6.877951	6.945593	7.478282	7.560792	7.523145	7.448927	7.494901	7.397265	7.593227	7.472645
Mblac2	-0.996402	2.892774	-3.996059	0.001239	0.012957	-1.204851	2.896425	1.972946	2.726949	3.766803	2.295481	2.410784	3.026505	2.964197	3.225976	2.707689	3.091342	2.999674	3.349197	3.286911
Ccdc80	-0.878275	4.687232	-3.993762	0.001245	0.013005	-1.461836	5.352623	3.864284	4.000932	4.756333	4.332519	4.487224	4.90017	4.934079	4.589521	5.104699	4.72823	5.302599	4.481425	4.850685
Notch1	0.777894	7.028835	3.99256	0.001248	0.013025	-1.550095	6.824729	7.757369	7.484437	6.404494	7.247765	7.074235	6.936991	6.986585	7.042185	7.030956	6.726681	7.188858	6.67395	7.036767
Pwwp2b	-0.845423	4.968544	-3.992005	0.001249	0.013028	-1.476582	5.019661	3.822768	4.377553	5.306089	4.611694	4.452412	5.127701	5.403241	5.069035	5.104699	5.524588	5.265326	5.544933	4.983491
Nrp1	-1.32573	6.276801	-3.990916	0.001252	0.013045	-1.544275	6.126148	4.664267	5.396283	7.27162	5.372461	6.051319	6.507921	6.84716	6.762181	6.187098	6.566064	6.609377	6.976296	6.560282
Clcn4	-0.921698	7.045563	-3.990429	0.001253	0.013047	-1.555239	6.964733	5.919409	6.269883	7.568782	6.322787	6.865164	7.403351	7.275121	7.423163	6.87449	7.286524	7.450235	7.616934	7.410164
Camk2g	-0.707294	6.600551	-3.989379	0.001256	0.013063	-1.5584	6.930665	6.266712	6.821528	7.188046	6.180763	6.138763	6.640244	6.632313	6.515461	6.446891	6.645021	6.599165	6.854484	6.561949
Evi2b	1.612559	3.375609	3.985798	0.001265	0.013144	-1.29498	3.247483	3.769134	4.519244	2.806722	5.116828	5.041927	2.165439	3.858761	1.520039	3.545408	3.480655	4.597515	1.902128	1.889408
Pfn1	0.758976	7.065836	3.985268	0.001266	0.013144	-1.563826	6.986391	7.418559	7.610496	7.002201	7.808889	8.175908	6.95231	6.932735	6.685098	6.788633	6.858244	6.818855	6.562485	6.333144
Snx18	0.520667	5.369868	3.984986	0.001267	0.013144	-1.535386	5.304169	5.706224	5.886424	5.399171	5.964165	5.930042	5.000844	5.394161	4.733316	5.204106	5.322117	5.523062	4.982355	4.866959
Hhex	1.00649	2.614079	3.982042	0.001274	0.01321	-1.136217	2.658139	3.028596	3.028308	1.679737	3.259533	3.221545	2.165439	2.926907	1.937064	3.14899	2.729	3.237047	1.859646	1.991897
Lactb	0.726886	4.353809	3.98042	0.001278	0.013242	-1.471212	4.040949	4.894604	4.911454	4.651364	5.159937	5.351571	3.975863	4.044317	3.895674	4.150009	3.930525	4.224961	3.760537	4.041787
Themis2	1.839009	1.135267	3.979477	0.001281	0.013244	-0.849477	0.955837	1.934482	2.33402	-0.600663	1.643636	1.585258	0.625837	1.958936	0.931093	1.322149	1.903329	1.869448	0.313987	-0.03329
Dos	-0.499955	8.270996	-3.979474	0.001281	0.013244	-1.557463	8.381743	7.705131	8.073678	8.463204	7.982334	7.931616	8.491031	8.209162	8.514463	8.360939	8.19235	8.355645	8.50512	8.632711
Rgs7bp	-1.400776	4.818922	-3.978364	0.001283	0.013262	-1.523138	5.241734	3.684748	4.519244	6.162305	4.086605	4.907024	4.652595	4.998404	4.812563	4.345154	5.182653	4.816761	5.594349	4.521176
Ascc2	0.698019	4.818059	3.976235	0.001289	0.013307	-1.485919	4.633881	5.412913	5.137285	4.196165	5.070428	4.807235	4.874668	4.968131	4.739818	4.891176	4.797057	4.783844	4.579577	4.6173
RGD1311892	1.026758	2.596951	3.974745	0.001293	0.013335	-1.148447	2.279379	3.324693	3.138503	2.013053	3.293596	2.872384	2.433388	2.207232	2.381582	2.745897	2.066633	2.941167	2.601568	2.325991
Pcsk5	-0.774565	5.071391	-3.972369	0.001299	0.01338	-1.523518	4.745509	4.393585	4.419086	5.682553	4.502342	4.48148	5.521948	5.263149	5.6132	5.150519	5.168735	5.162375	5.340009	5.604558
Snx9	0.561104	4.467508	3.972226	0.001299	0.01338	-1.500778	4.536569	4.955898	5.216372	4.488478	4.964461	5.141808	3.951676	4.279444	3.91304	4.528025	4.144751	4.523011	3.91892	4.056051
Lingo2	-1.151671	3.469338	-3.970991	0.001303	0.013402	-1.352561	3.562947	2.397344	3.530855	4.405886	2.672087	2.743931	3.221182	3.784265	3.540964	3.901891	3.493474	4.032882	3.713602	3.722142
Rpl18a	0.79449	8.441567	3.968824	0.001308	0.013437	-1.563469	8.889253	9.53851	9.197433	8.122684	9.469503	9.001093	7.878214	8.053869	7.950296	8.275635	8.197745	7.99607	7.680858	7.935628
Ifrd1	0.706713	5.899071	3.968807	0.001308	0.013437	-1.583052	5.78286	6.055567	6.629073	5.549235	6.451319	6.343407	6.098263	5.593743	5.74833	5.660966	5.825096	5.463681	5.863867	5.548486
Kif23	1.279095	1.556288	3.967899	0.001311	0.01345	-1.017185	1.310059	2.667383	1.936547	1.245516	2.79132	2.772436	1.000645	0.923327	1.326257	1.481601	0.885831	1.542121	1.316372	1.158276
Uqcr10	-0.868053	2.847073	-3.966945	0.001313	0.013464	-1.249275	3.171726	2.643736	1.951721	3.218762	2.496038	2.323579	3.504983	2.488905	3.298245	3.129854	2.843564	2.761217	3.195422	3.060363
Mtm1	-0.690445	4.9835	-3.965827	0.001316	0.013479	-1.540038	5.163495	4.159341	4.438066	5.280562	4.840905	4.687608	5.135774	5.159792	5.243904	4.997931	5.289414	5.058407	5.366799	4.998341
Hecw1	-1.24902	4.443459	-3.965245	0.001318	0.013479	-1.509507	4.661839	3.859159	4.349185	5.624215	3.26641	4.124114	4.743453	4.718236	4.818719	3.978769	4.518478	4.114573	5.143751	4.365708
Ccdc102a	1.009724	3.558328	3.965137	0.001318	0.013479	-1.337257	3.799454	4.019342	4.179109	2.451642	4.202298	3.972661	3.159163	3.698353	3.253503	3.747244	3.760656	3.644018	2.954296	3.11589
Tlr4	1.691426	1.201393	3.962221	0.001326	0.013546	-0.951855	0.915118	2.028784	2.607481	0.531921	2.198134	2.537984	-0.279766	1.856886	-1.304935	1.920699	1.375754	2.346065	0.375586	0.65743
Mms22l	2.655083	0.074894	3.961666	0.001327	0.01355	-0.829848	-0.760787	1.914859	0.883381	-0.325128	2.72297	2.481552	-1.009457	0.04768	-0.773548	-0.311823	-0.616402	0.151783	-0.755361	-0.748265
Eya2	1.941972	1.515498	3.96024	0.001331	0.013577	-1.049362	2.162475	2.724859	3.525794	0.43652	2.60856	3.668376	0.563361	1.747064	0.091681	0.699359	0.846404	1.715048	0.745852	0.383504
Bub1	1.787532	1.707738	3.958874	0.001335	0.013603	-1.016043	1.033981	2.854122	1.921212	1.128291	3.333437	3.295428	1.408645	1.239467	1.102347	1.388059	1.167576	1.738143	1.218908	1.608903
Pdilt	-3.393979	-0.628459	-3.958068	0.001337	0.013613	-0.82047	0.031429	-3.260591	-2.787702	0.105385	-0.424712	-2.747572	1.754571	0.923327	0.512061	0.280604	0.961591	0.460309	0.037347	0.753927
Spg21	0.44568	5.492004	3.955467	0.001344	0.013672	-1.592871	5.362121	5.693323	5.859524	5.292531	5.763686	5.768441	5.185883	5.567732	5.319649	5.474446	5.645806	5.619495	5.233825	5.136759
Nop14	0.585964	5.568126	3.954894	0.001345	0.013677	-1.600305	5.333436	6.107273	6.081833	5.521898	6.043376	5.8251	5.526049	5.543299	5.481658	5.537522	5.354096	5.210647	5.145962	5.275297
Sema5a	-1.6607	8.032115	-3.952886	0.001351	0.01372	-1.615711	7.904273	5.506021	7.253683	8.998191	6.841573	7.547089	8.410967	8.609028	8.642959	8.511768	8.327348	8.68135	8.569756	8.653578
Camk1g	-0.736912	4.858794	-3.951355	0.001355	0.013751	-1.575088	5.569851	4.554415	4.620039	5.070211	4.899589	4.251076	4.915882	4.696241	5.024458	4.865376	4.79964	4.454942	5.198041	5.158958
Snurf	-1.851978	1.763514	-3.949772	0.001359	0.013778	-1.242628	2.843156	0.769921	1.890044	4.094353	0.845972	3.084277	2.05625	1.71825	1.636128	1.419918	1.068256	0.760186	1.626159	1.415936
Lsm2	0.716967	3.470452	3.949287	0.00136	0.013778	-1.423335	3.614724	4.24827	4.082466	3.400384	4.513947	4.028875	3.083259	3.301815	3.059216	3.177227	3.434871	2.910998	3.042398	2.83626
Vcl	0.578396	6.33966	3.949086	0.001361	0.013778	-1.633984	6.231475	6.441464	6.592631	5.988211	6.842148	6.871217	6.176005	6.090416	6.148482	6.168464	6.176611	6.37348	6.338149	6.33607
Tuba8	-1.602158	6.074553	-3.94805	0.001364	0.013795	-1.632727	6.759378	4.086661	5.771406	7.342004	5.943932	5.96133	5.97168	6.330651	6.418477	5.812672	6.391305	5.860869	6.454227	5.96608
Clic4	0.993417	4.862992	3.947526	0.001365	0.013798	-1.569147	4.97826	5.354021	5.8992	4.301452	5.647325	5.581066	4.403445	4.736311	4.401362	4.542569	3.958531	5.025932	4.698844	4.610873
Lhx6	-0.673766	4.28506	-3.94596	0.00137	0.01383	-1.527808	4.559869	4.244344	3.926082	4.748906	3.956574	3.835297	4.371894	4.259584	4.413615	4.224061	4.269233	3.859433	4.822887	4.581589
Actl6a	0.694973	4.061688	3.943551	0.001376	0.013885	-1.434626	3.646165	4.445797	4.02265	3.374877	4.12116	4.213454	4.267989	3.997121	4.303799	4.164187	4.03505	4.078836	3.91892	4.369521
Lig1	0.905481	3.992448	3.942927	0.001378	0.013891	-1.464657	3.652372	4.777251	4.079015	3.665254	4.866025	4.546193	3.969854	4.12885	3.854323	3.867646	3.414796	3.674268	3.748946	3.751656
Mdfic	1.773425	2.795275	3.937026	0.001394	0.014044	-1.321671	3.759759	3.904639	4.275724	1.430203	4.173318	4.781755	1.754571	2.121805	1.489496	2.529327	3.196995	2.816513	1.436856	1.755962
Mpp3	-0.803758	7.134253	-3.933748	0.001403	0.014115	-1.66964	7.284922	5.953471	6.620197	7.390142	6.876266	6.683845	7.682679	7.378872	7.476698	7.243387	7.39383	7.059606	7.522653	7.324866
Cdca7	1.661485	2.364883	3.933702	0.001404	0.014115	-1.153683	2.091157	3.125682	2.548818	1.158502	3.726565	3.568964	2.078759	2.287882	2.147322	2.655119	2.455292	2.931181	1.150071	1.529341
Thbs4	2.420096	-0.313625	3.931568	0.00141	0.014163	-0.862234	-0.217457	0.973336	1.574317	-1.575022	1.338932	1.136566	-0.831021	0.222004	-0.504027	0.134646	-1.142289	0.567345	-1.638202	-1.899685
Olfm3	-1.184816	3.660404	-3.927091	0.001422	0.014274	-1.483047	3.992844	2.383133	3.013923	4.563778	3.196117	3.794184	3.699086	3.884925	3.31576	3.608978	3.892318	3.744381	4.098625	4.19144
Ypel3	-0.509444	7.644463	-3.926844	0.001423	0.014274	-1.672293	7.951286	7.503123	7.397414	8.154183	7.612044	7.658507	7.483036	7.466485	7.737725	7.682732	7.686047	7.536747	7.519672	7.641392
Htr1a	-2.057646	3.930092	-3.922626	0.001435	0.014378	-1.464071	3.978094	0.725555	2.673036	5.256321	3.210452	3.616909	4.017238	4.596856	4.534483	4.086912	4.407721	4.489378	4.916528	4.673894
Ptx3	2.262719	1.973912	3.922386	0.001436	0.014378	-1.12594	1.245833	3.055728	4.544485	1.273392	2.672087	3.568964	0.563361	2.345542	1.520039	1.828755	1.430105	2.170435	0.649393	1.260365
Sat1	0.71525	7.240293	3.92148	0.001438	0.014393	-1.693599	6.783614	7.979367	7.484437	6.85489	7.242543	7.426716	7.471387	7.131581	7.276577	7.291002	7.029745	7.200337	6.97194	7.230542
Kcna6	-0.916727	6.50612	-3.919917	0.001443	0.014426	-1.694922	6.663531	6.005422	5.582052	6.894134	5.829016	6.040617	6.524414	6.875981	6.345742	6.671107	6.466371	7.173056	7.226126	6.806553
Dleu7	-1.093132	1.832743	-3.918767	0.001446	0.014447	-1.218032	2.542593	0.812964	1.65069	2.666599	2.124346	1.496914	1.938115	1.239467	2.260183	1.920699	1.762161	1.345607	2.133689	2.228598
Dixdc1	-0.590138	6.521736	-3.918005	0.001448	0.014457	-1.70093	6.535899	6.176889	5.893317	6.988602	6.253522	6.370076	6.740115	6.548861	6.601879	6.563796	6.478511	6.57932	6.77813	6.812867
Upp1	1.860296	2.901747	3.91662	0.001453	0.014485	-1.355534	2.896425	5.171753	4.928811	2.169587	3.775594	3.585123	2.433388	2.488905	2.39812	2.256873	2.303426	1.930875	1.837926	2.697558
Cdk4	0.566885	6.344929	3.915067	0.001457	0.014518	-1.705806	6.344085	6.758064	6.713285	6.383864	7.067556	7.182637	5.979177	6.043277	5.946861	6.306949	6.142429	6.203436	5.783768	5.993516
Slco2a1	1.96827	2.255225	3.914127	0.00146	0.014531	-1.165017	2.682606	2.864389	3.679531	0.22441	3.114621	3.491064	1.754571	1.596873	2.260183	1.73055	2.014226	1.667719	2.860463	2.030937
Vrk2	1.539312	0.725065	3.913511	0.001462	0.014531	-0.989127	0.915118	1.991799	1.792283	-0.243853	1.705671	1.627481	0.028455	0.972957	0.571595	0.591587	-0.231835	0.84773	0.182287	0.442608
Ntrk1	-2.196303	2.951733	-3.912987	0.001463	0.014531	-1.186273	3.724844	0.185409	0.715742	2.213416	0.808768	1.402804	3.933266	4.692543	4.002283	4.010503	3.366837	4.584831	3.725479	4.338735
Tlr7	1.353179	3.493405	3.912916	0.001463	0.014531	-1.434019	2.967843	4.509329	4.860105	3.067759	3.973484	4.105563	1.888013	3.878428	2.39812	3.728285	3.555912	4.701051	2.827778	2.610029
Htr2b	4.811231	-1.912771	3.91245	0.001465	0.014531	-1.002001	-1.642648	0.769921	1.061799	-3.260591	1.685287	0.734546	-2.558588	-1.196868	-2.604521	-3.260591	-1.500575	-0.334482	-1.904936	-3.260591
Ctnna1	0.744536	6.673953	3.911782	0.001467	0.014531	-1.712839	6.879992	7.535609	7.117349	6.165098	7.342738	7.063702	6.291322	6.535503	6.247632	6.453641	6.360149	6.77974	6.241162	6.437623
Fat4	-1.765833	5.211279	-3.911399	0.001468	0.014531	-1.618656	4.611755	2.991607	4.526863	6.343342	3.129785	4.174824	6.049239	6.209071	6.313411	5.587473	5.71448	5.765739	6.03231	5.568526
Il17rc	1.333717	2.263477	3.911343	0.001468	0.014531	-1.148437	1.9771	3.125682	2.498042	0.823477	2.81965	2.297664	2.266943	2.186344	2.04602	2.600561	2.616315	2.375822	2.003164	2.402413
Wwtr1	0.903429	3.43188	3.910881	0.001469	0.014532	-1.504419	3.621067	4.042133	4.856079	3.381296	4.132497	4.512792	3.094359	2.835955	2.734001	2.934159	2.968181	3.33876	3.042398	2.70964
Mest	-0.853292	3.401006	-3.909881	0.001472	0.01455	-1.49162	3.912376	2.758277	3.077557	3.980586	3.476716	3.069038	3.038038	3.201289	3.729193	3.223093	3.712129	3.30822	3.695599	3.584586
Cpm	-1.176681	5.50097	-3.908703	0.001476	0.014572	-1.666472	5.550041	4.309667	3.797607	5.666875	4.625132	5.026231	5.703101	6.098823	5.61497	5.974275	5.793051	6.315161	6.597646	5.982355
Lmtk2	-0.534858	7.8186	-3.907029	0.001481	0.014609	-1.718033	7.646556	7.172754	7.438724	7.88675	6.978748	7.337321	8.257565	8.046643	8.175652	7.910753	7.969061	8.10668	8.267608	8.27291
Plod3	0.758659	5.878773	3.904766	0.001487	0.014653	-1.697682	5.599065	6.208464	6.04958	5.066232	6.070152	6.016987	5.758179	6.031614	5.840457	6.068994	5.758953	6.124567	5.495499	6.24146
Gnaz	-0.81179	5.836783	-3.904724	0.001488	0.014653	-1.71989	6.19686	5.080024	5.584488	6.640858	5.735463	6.024907	5.787514	5.89668	5.86446	5.655958	5.960512	5.733061	5.92695	5.655412
Zdhhc8	-0.485647	6.70559	-3.903584	0.001491	0.014674	-1.73144	6.851951	6.264775	6.591421	6.885129	6.35943	6.316236	6.849701	6.915387	6.864655	6.933619	6.682586	6.889536	6.710266	6.778868
Paqr4	-0.704564	6.531265	-3.902294	0.001495	0.01469	-1.732549	6.871371	5.85651	5.822858	6.765706	6.507546	6.27117	6.442169	6.690026	6.500222	6.639321	6.730747	6.964278	6.666267	6.727062
Ddah1	0.896988	5.91523	3.902243	0.001495	0.01469	-1.729114	6.48985	7.184069	6.984405	5.85273	6.985015	7.110041	4.828927	4.915162	5.094627	5.34974	5.840228	5.591235	5.486802	5.130032
B3gat1	-0.581029	8.475559	-3.901385	0.001498	0.014703	-1.71824	8.415208	7.436303	8.061927	8.199384	7.686361	7.728982	8.951969	8.927687	8.98394	8.821924	8.787425	8.813954	8.967294	8.880266
Acvr2a	-0.820927	4.937	-3.9002	0.001501	0.014716	-1.668962	4.902169	4.538474	4.770955	5.577462	3.922149	4.467019	5.419857	4.949657	5.275577	4.910928	4.992577	4.832942	5.208658	5.403549
Gstcd	0.870939	2.219071	3.900131	0.001501	0.014716	-1.237683	1.916502	3.000943	2.508341	1.799605	2.651221	2.72461	2.165439	1.958936	2.185932	2.359011	2.197736	2.064785	1.770732	2.105984
C1rl	1.541132	1.196346	3.897651	0.001509	0.014768	-1.050893	0.643243	2.064844	2.286937	0.485009	1.803478	2.025031	-0.529223	2.207232	0.787836	1.780488	1.456531	2.136071	-0.405292	0.844371
Lhfpl2	0.884031	5.455587	3.897164	0.00151	0.014768	-1.69799	5.375315	5.998447	5.995694	4.711187	6.09451	5.590436	4.674929	5.702559	4.589521	5.65763	5.729457	6.306496	5.011753	4.978507
Rpa2	0.564059	3.83316	3.897147	0.00151	0.014768	-1.551146	3.838085	4.275453	4.172648	3.54462	4.36492	4.178379	3.537848	3.653399	3.722643	3.844356	3.644778	3.822092	3.508915	3.667444
Rbpj	0.835823	5.007943	3.894995	0.001517	0.01482	-1.713187	5.062241	5.690441	6.446303	5.421144	5.807959	6.366185	4.248266	4.65122	4.384862	4.2553	4.44779	4.677349	4.549825	4.156561
Pcmtd1	-0.77092	7.241141	-3.894469	0.001519	0.014823	-1.74364	7.468492	6.727457	6.844574	8.129849	7.097752	7.438252	7.282504	7.285593	7.083672	7.153272	7.353531	7.127867	7.218792	7.174983
Rfc3	1.1198	3.039776	3.894084	0.00152	0.014823	-1.376779	3.09177	3.996186	3.431527	1.92792	3.628553	3.334188	2.918339	2.849305	3.100223	3.167876	3.004456	2.910998	2.549064	2.847239
Rnf182	-1.289642	3.618991	-3.892362	0.001525	0.01486	-1.523839	3.917539	2.01041	3.035447	4.37727	2.892584	3.479584	3.585784	4.020912	4.081214	3.129854	3.882605	3.980582	4.383621	4.032198
Cdkn2aipnl	0.466872	5.257034	3.892032	0.001526	0.01486	-1.724113	5.344979	5.846187	5.835184	5.435094	5.826691	5.91946	4.848708	4.866752	4.821787	4.902496	5.117902	4.864769	5.080417	4.930278
Smarca2	-0.681667	7.733777	-3.891205	0.001528	0.014873	-1.744932	7.772284	7.21097	7.266255	8.355055	7.321433	7.724733	7.883418	7.844841	7.923056	7.639834	7.893098	7.80731	7.939187	7.698962
Cacng3	-0.984255	5.701516	-3.890766	0.00153	0.014874	-1.736958	5.913928	4.554415	5.265774	6.415482	5.240728	5.651818	5.31947	6.054847	6.047264	5.915425	5.906424	5.894664	5.977491	5.69545
Mgat3	-1.163081	6.600345	-3.887426	0.00154	0.014951	-1.761181	6.849927	5.046602	6.263817	7.314596	5.953551	6.403113	6.728595	6.840491	6.805444	6.349962	6.895543	6.849004	7.233423	6.888549
Lgi2	-0.960539	3.061293	-3.887381	0.00154	0.014951	-1.488037	3.271877	2.667383	3.12518	4.117607	2.519256	2.695134	3.337752	2.795149	2.905753	2.889346	2.823399	3.037402	3.621257	3.246018
Jakmip1	-0.686946	6.891839	-3.886714	0.001542	0.014953	-1.763625	7.338354	6.381964	6.761442	7.606939	6.980317	7.01781	6.719671	6.958372	6.884598	6.768668	6.917111	6.620295	6.920555	6.62311
Rtkn2	-1.361975	1.252769	-3.886503	0.001543	0.014953	-1.157514	1.568201	0.533043	0.851376	2.427034	0.266146	1.136566	1.17791	1.628188	0.884903	1.180545	1.375754	1.643459	1.983515	1.582867
Stmn1	-0.688804	6.421698	-3.88579	0.001545	0.014963	-1.767275	7.065268	6.634536	6.055737	7.046796	6.727212	6.057124	6.294932	6.140141	6.274752	6.373541	6.303044	6.30069	6.27893	6.36977
Mtmr7	-0.592207	5.735347	-3.883841	0.001551	0.015009	-1.754438	5.801132	5.55755	5.38794	6.264804	5.403987	5.426725	6.09964	5.733485	5.862971	5.84823	5.852718	5.53465	5.806312	5.744718
Dcc	-1.422084	4.713833	-3.882697	0.001555	0.015032	-1.666437	4.502616	3.24835	4.054626	5.809355	3.691225	3.956189	4.802122	5.350235	5.282274	4.402091	5.322117	5.03994	5.65616	4.945683
Abcd3	-0.489139	6.888518	-3.881358	0.001559	0.015049	-1.776908	6.87602	6.595778	6.376335	7.151898	6.464835	6.664331	7.263594	6.917765	7.055278	7.0154	6.869351	6.976469	7.078814	7.146936
Rai1	-0.563858	6.824221	-3.881332	0.001559	0.015049	-1.775769	6.678815	6.273469	6.594445	7.002201	6.134527	6.105539	7.090257	7.175301	7.158383	6.991746	7.054285	7.013025	7.17236	7.109241
Kcnt2	-0.871288	5.204593	-3.880448	0.001562	0.015063	-1.702721	4.794038	4.216563	4.462108	5.589986	4.01491	4.613389	5.998491	5.663417	6.026139	5.544765	5.307674	5.162375	5.77665	5.74178
Adcy2	-0.529052	7.641924	-3.879813	0.001564	0.01507	-1.774847	7.560063	7.01926	7.072936	7.748351	7.099198	7.308819	7.815097	7.75283	8.021407	7.955595	7.705802	7.857937	7.900092	8.177728
Pank1	-1.029345	3.947692	-3.877629	0.001571	0.015124	-1.629377	4.231267	2.934281	3.458336	4.835601	3.607042	4.028875	4.129307	3.910622	3.774234	3.715505	4.334806	3.941318	4.44597	4.027379
Xylb	0.9566	5.226751	3.876616	0.001574	0.015143	-1.721201	4.810783	6.371204	5.442668	4.711187	5.536481	5.522136	5.061434	5.204627	4.989416	5.493286	5.194477	5.287022	4.456698	5.136759
Jph4	-0.681763	7.987604	-3.876133	0.001575	0.015146	-1.776226	8.231786	6.890803	7.393947	7.807043	7.509258	7.565532	8.384673	8.35609	8.302687	8.326422	8.207989	8.384521	8.266847	8.205455
Spata20	2.250854	-0.032845	3.875737	0.001577	0.015146	-0.966235	-1.22354	1.833582	0.715742	-0.502853	1.556544	0.915838	-1.21312	0.301855	-0.929891	-0.210791	-0.411343	0.403657	0.182287	-0.03329
Acbd5	-0.752329	4.18025	-3.874619	0.00158	0.015168	-1.708768	4.791228	4.142566	4.14651	5.347656	4.27571	4.729403	3.857182	4.020912	3.935874	3.740952	3.621611	3.946285	4.20426	3.853172
Arhgap11a	1.217309	3.10147	3.870714	0.001593	0.015269	-1.447416	2.542593	3.934181	3.098159	2.697792	4.209453	4.127796	2.967416	3.012464	2.667343	3.019813	2.750518	3.376046	2.562371	2.648193
Srgap3	-0.953279	7.071966	-3.87051	0.001593	0.015269	-1.794966	7.290902	6.404125	6.948926	7.839747	6.29531	6.795694	7.189257	7.348052	7.169866	6.73186	7.232591	7.057312	7.742435	6.973669
Prkd3	0.662589	4.585202	3.868814	0.001599	0.015298	-1.688976	4.464322	4.894604	5.037699	4.132904	4.859217	5.020301	4.330288	4.58892	4.208563	4.751052	4.33124	4.980656	4.23782	4.421864
Olfm1	-0.982008	10.50659	-3.868766	0.001599	0.015298	-1.709629	10.54463	9.047262	9.87044	10.94969	10.04324	10.08709	10.42615	10.89549	11.00631	10.8112	10.78505	10.8779	10.8802	10.87781
C4b	2.159577	1.327879	3.866564	0.001606	0.015354	-1.072485	0.307952	2.33964	1.408255	0.041976	2.682409	2.634313	1.443753	1.71825	1.061401	1.540753	0.764165	2.046399	0.434663	0.887553
Fam203a	0.617739	4.487423	3.864435	0.001613	0.015388	-1.677543	4.123692	4.570182	4.785848	4.132904	5.062547	4.551685	4.43432	4.596856	4.372361	4.602796	4.655954	4.376159	4.28674	4.342619
Plcxd2	-1.173703	7.74885	-3.864223	0.001614	0.015388	-1.792078	8.132046	7.372994	7.493538	8.868838	6.909595	7.523525	7.557048	7.986747	7.713978	7.302264	8.146928	7.641133	8.436999	7.405993

Rreb1	-1.220925	6.601438	-3.864185	0.001614	0.015388	-1.79861	5.989418	5.030736	5.792629	7.171012	5.29218	5.318124	7.645465	7.276974	7.727128	7.288847	6.963916	6.935219	6.739847	7.268811
Eif2c1	-0.659526	6.31131	-3.863858	0.001615	0.015388	-1.807573	6.325755	5.969015	6.004816	6.870944	5.672137	6.114845	6.451901	6.553966	6.518301	6.192872	6.479317	6.371635	6.579763	6.273352
Cdc42bpa	-0.609135	7.606871	-3.861468	0.001623	0.01545	-1.806115	7.822001	7.579503	7.297769	8.148079	7.136743	7.485649	7.786751	7.550102	7.601104	7.485141	7.466739	7.657503	7.945866	7.541432
Pla2g2c	-1.119115	1.788928	-3.860296	0.001626	0.015474	-1.255841	2.402324	0.895377	1.340699	1.962578	0.649545	1.402804	1.668148	2.054242	2.241976	2.484835	1.996325	2.502617	1.79348	2.142093
Chn2	-1.042547	4.665215	-3.859787	0.001628	0.015478	-1.744253	5.406864	4.479583	3.520715	5.140008	4.545387	4.425741	4.228269	4.961999	4.526983	4.640468	4.597784	5.222978	4.962418	4.719417
Mtus2	-0.645321	3.635727	-3.859049	0.00163	0.015488	-1.625156	3.827152	3.445867	3.453014	4.281	3.326873	3.456346	3.727855	3.910622	3.761508	3.559779	3.537463	3.390696	3.772036	3.578011
Ubl3	-0.615953	7.327489	-3.857284	0.001636	0.015531	-1.820501	7.600752	6.737964	7.092321	7.709661	7.080784	7.247107	7.514853	7.337434	7.277695	7.177936	7.482899	7.36934	7.683524	7.282524
Il1r2	6.63977	-1.956359	3.854312	0.001646	0.015612	-1.13766	-2.659044	2.151237	3.719973	-3.260591	1.415175	1.165512	-2.088409	-2.550306	-3.260591	-2.176425	-1.310338	-1.925331	-2.656794	-3.260591
Plekhb1	-0.937919	8.945913	-3.851363	0.001656	0.015692	-1.785978	9.572718	9.111974	8.342604	9.484205	8.589893	8.310168	9.037766	8.837414	9.114459	9.239298	8.485288	9.096807	8.728498	9.295015
Ctdsp1	0.757458	6.075088	3.847267	0.001669	0.015808	-1.836992	6.38193	6.856497	6.673796	5.563417	6.868942	6.507131	5.617289	5.708064	5.485533	6.004794	5.987948	6.105823	5.509306	5.805084
Pde1b	-0.788611	6.374839	-3.84692	0.001671	0.015808	-1.842974	6.558384	5.735883	6.590209	6.966359	5.721768	5.845367	6.310469	6.67511	6.485787	6.573567	6.536176	6.481779	6.399674	6.3868
Nsun2	0.456228	6.049392	3.845397	0.001676	0.015844	-1.839537	5.824683	6.276355	6.546527	6.083798	6.455086	6.364625	5.970175	5.914254	5.892453	5.829078	5.912402	5.812728	5.947379	5.885832
P2rx4	0.752218	5.095073	3.841574	0.001689	0.015948	-1.782339	4.618111	5.430295	5.563652	4.738944	5.26153	5.463612	4.674929	5.387314	5.071615	5.128996	4.857801	5.433932	4.573018	5.174296
Nbn	0.506867	5.263713	3.841354	0.001689	0.015948	-1.822693	5.241734	5.574846	5.889383	5.388058	5.946075	5.874148	5.138455	4.983347	4.936619	4.924873	5.050107	4.870006	5.009325	4.906858
RGD1307443	-0.699193	5.850131	-3.840359	0.001693	0.015963	-1.833794	5.55998	5.15303	5.014403	6.202733	5.070428	5.508079	6.491238	6.181629	6.44359	5.812672	5.888339	5.917605	6.314752	6.372622
Rpl24	0.611645	6.122044	3.839739	0.001695	0.015963	-1.847019	5.967183	6.565421	6.192157	5.611986	6.494426	6.344989	6.120131	5.903095	6.007398	6.410682	6.120905	6.104713	5.882553	6.005816
Tpm3	0.702701	7.523751	3.839724	0.001695	0.015963	-1.846454	7.448567	7.911985	8.493445	7.909134	8.542344	8.579993	6.886051	7.17861	7.025649	6.971063	7.222521	7.162424	7.079392	6.930447
Irs1	-0.923663	2.839884	-3.838039	0.001701	0.016005	-1.586681	3.522857	2.425352	3.196978	3.847833	2.712936	2.714852	2.550752	2.988532	2.493553	2.469694	2.792614	2.794648	3.229186	2.245294
Pes1	0.4413	6.291587	3.837021	0.001704	0.016025	-1.861634	6.236648	6.510914	6.75713	6.235874	6.754983	6.685095	5.96867	6.293288	6.123835	6.225913	6.219677	6.110255	6.017894	5.962298
Svop	-0.756146	7.084889	-3.835627	0.001709	0.016051	-1.867136	6.988846	5.507659	6.390324	6.752769	6.253522	6.30327	7.511237	7.5709	7.65155	7.599105	7.520177	7.682647	7.773874	7.695929
Lrrk1	1.041673	3.69969	3.835429	0.00171	0.016051	-1.583813	3.736577	4.171795	3.989949	2.350586	4.027109	3.99299	3.328389	4.239446	3.341641	3.978769	3.976904	4.327499	3.061266	3.403062
Mturn	-1.010924	5.423106	-3.834363	0.001713	0.016073	-1.853676	6.03882	4.817439	5.35122	6.497542	4.914989	5.9395	5.511644	5.065713	5.202134	4.924873	5.233205	5.227065	5.885203	5.352453
Acacb	-1.220133	3.658871	-3.832482	0.00172	0.016121	-1.529437	3.652372	2.924502	1.705429	3.1898	2.226624	2.071653	4.595201	4.540363	4.461611	4.441427	4.056804	4.862143	4.080242	4.584872
Gfra2	-1.004166	3.463349	-3.828812	0.001733	0.016228	-1.669556	4.012279	3.34683	3.151703	4.207046	3.557438	2.434761	3.616879	3.395791	3.416608	3.683052	3.048553	3.594258	3.254	3.917086
Nuf2	2.178813	1.523085	3.826354	0.001741	0.016283	-1.257541	0.995438	2.934281	1.966737	0.860705	3.691225	3.703341	1.000645	0.76329	0.683763	1.180545	0.631286	1.284167	1.11438	1.158276
Grid2	-0.841261	3.187531	-3.826087	0.001742	0.016283	-1.59681	3.350332	2.397344	2.845423	3.781437	2.79132	2.898483	3.038038	3.340143	3.178898	3.310669	3.091342	3.600573	3.614885	3.56477
Sppl2b	1.12527	6.566883	3.825601	0.001744	0.016283	-1.869061	5.749954	7.303953	6.770562	5.380068	6.759865	5.91414	6.974987	6.629414	6.774901	6.969045	6.591625	6.776955	6.346826	7.01198
Gtf3a	0.653517	4.242164	3.825539	0.001744	0.016283	-1.757461	4.350588	4.528825	4.792184	3.924553	5.048652	4.748661	3.945565	4.050109	3.947156	4.178226	4.140682	4.316033	3.640205	3.864023
Uaca	0.899166	4.926599	3.825188	0.001745	0.016283	-1.818417	4.965854	5.971392	5.419662	4.4153	5.427188	5.51653	4.983054	4.556731	4.739818	4.800297	4.313278	4.677349	4.229503	5.0106
Plk3	0.687112	3.496232	3.822847	0.001753	0.016347	-1.654242	3.334978	3.934181	4.257522	3.41297	4.255114	3.775529	3.318966	3.40486	3.207329	3.385186	3.099749	3.38339	3.03287	3.286911
Mb21d2	-0.630702	4.62519	-3.822179	0.001756	0.016349	-1.822302	4.870572	4.279295	4.483148	5.346016	4.5954	4.568038	4.571588	4.451246	4.504248	4.441427	4.644506	4.641051	4.916528	4.503936
Mrps18b	0.600418	5.170043	3.822013	0.001756	0.016349	-1.847657	5.178626	5.604242	5.722481	4.941472	5.927755	5.373446	4.989008	5.138201	4.961869	5.026667	4.974403	4.803137	4.797792	4.985976
Spata2L	-0.505653	6.505275	-3.820839	0.001761	0.016364	-1.895918	6.619063	5.885785	6.194551	6.649526	6.356211	6.078208	6.579712	6.702033	6.675802	6.718312	6.77997	6.538791	6.695245	6.618311
Cep55	2.764309	-0.117878	3.820802	0.001761	0.016364	-1.074928	-0.634519	2.01041	0.715742	-0.941548	2.212449	2.336365	-1.009457	-0.654381	-0.929891	-1.136114	0.001431	0.514819	-0.305233	-1.632384
Ros1	-3.251695	0.416081	-3.819683	0.001765	0.016388	-1.095903	1.212615	-0.366503	-0.130036	1.700419	-3.260591	-0.862697	1.511505	1.564863	1.819202	1.28803	0.071497	1.568131	1.1849	1.708589
Dusp19	-0.88193	2.830823	-3.818003	0.001771	0.016431	-1.512057	3.118917	2.411416	1.723223	2.916866	2.28197	2.217002	3.148563	2.862532	3.244385	3.12019	2.782205	3.037402	3.270309	3.73402
Eln	1.054607	4.412752	3.817268	0.001773	0.016443	-1.791891	4.62444	5.216521	5.846391	3.82447	4.974995	5.000356	4.34429	3.591194	4.316905	4.150009	3.827988	3.931333	3.783444	4.425531
Atad2	1.105209	3.681462	3.814439	0.001783	0.016524	-1.658196	3.00712	4.46953	3.595078	3.425447	4.487704	4.781755	3.585784	3.668539	3.383769	3.448339	3.387587	3.71558	3.409342	3.302949
Cacna2d3	-0.611583	7.761481	-3.813364	0.001787	0.016547	-1.90923	7.488145	6.68247	6.996802	7.802267	7.289296	7.164827	8.406803	8.13854	8.13059	8.006318	7.973928	8.104738	8.254603	8.229254
Bok	-1.019455	5.79822	-3.811788	0.001793	0.016586	-1.888792	6.268314	4.196386	4.942168	5.736122	5.437888	5.332553	6.399218	6.10022	6.075814	6.166118	6.197325	6.02362	6.37632	5.954705
Igsf3	-0.992145	4.67038	-3.810375	0.001798	0.016621	-1.808786	4.33909	3.661386	4.000932	5.425809	3.832311	4.105563	4.822272	5.042656	5.154258	4.919311	4.653101	5.22093	4.949817	5.325228
Fcn1	1.517049	1.55006	3.80966	0.001801	0.016632	-1.247869	1.033981	2.200694	2.548818	0.485009	2.062471	2.025031	0.282279	2.555575	1.061401	2.469694	1.959843	2.761217	-0.211665	1.122575
Adk	-0.524389	5.698566	-3.808318	0.001805	0.016664	-1.909315	6.207437	5.62854	5.43459	5.947696	5.700344	5.453378	5.671979	5.520532	5.690761	5.632356	5.526148	5.803178	5.925664	5.66785
Pak7	-1.059157	5.118238	-3.804198	0.001821	0.01678	-1.899064	5.587774	4.171795	4.9741	6.244702	4.852378	5.420735	4.785113	5.237996	5.037713	4.741628	5.069498	5.243299	5.413437	4.922513
Slc50a1	0.707974	5.137401	3.804111	0.001821	0.01678	-1.880086	5.214725	5.851358	5.499273	4.751386	5.80678	5.589101	4.893837	4.9924	4.970188	5.133806	4.781463	4.9879	4.438773	5.058621
C1ql2	-2.560602	5.710331	-3.802913	0.001825	0.016808	-1.862959	5.806708	1.279848	4.472666	7.062858	4.424547	5.179547	5.883333	6.596148	6.743697	6.701398	6.297573	6.657113	6.25044	6.663266
Slit2	-1.208592	6.085834	-3.801825	0.001829	0.016827	-1.915219	5.917798	4.03306	5.261214	6.654172	5.546404	5.44897	6.666792	6.276092	6.827743	6.151959	6.308495	6.126756	7.076497	6.93367
Kitlg	-0.982657	4.852524	-3.801582	0.00183	0.016827	-1.884547	5.139392	3.874479	4.913392	5.948777	4.810643	4.639415	4.739927	4.70361	5.144489	4.879766	4.752428	4.519683	4.874473	5.051519
Phyhd1	1.156993	4.995255	3.800659	0.001834	0.016845	-1.816968	4.406741	6.052203	5.05361	3.857074	5.318889	4.707427	5.133088	5.194202	4.91667	5.335194	4.974403	5.392683	4.652936	4.990935
Hmgcr	-0.549532	6.683461	-3.799682	0.001837	0.016865	-1.939603	6.524946	6.110512	6.137635	6.965291	6.126045	6.411444	7.363801	6.524103	7.201421	6.581512	6.495337	6.698912	7.114255	7.329277
Kcnk1	-0.663071	5.86	-3.795798	0.001852	0.016985	-1.933382	6.05648	5.12555	5.512125	6.173444	5.318889	5.846484	5.998491	5.78302	5.980196	6.140054	6.109501	5.945159	5.89969	6.178743
Dpp6	-0.934948	8.313733	-3.795388	0.001853	0.016986	-1.928453	8.208017	6.989082	7.749667	8.871975	7.568886	8.110892	8.469633	8.735058	8.649233	8.513384	8.471992	8.648395	8.767439	8.64397
Stat6	0.697919	4.711903	3.793045	0.001862	0.017053	-1.832472	4.602167	4.92435	4.992559	3.82447	4.8478	4.81867	4.626095	4.9924	4.485023	4.916522	4.960622	5.235205	4.330171	4.472374
Rhoq	0.635397	4.229457	3.791919	0.001866	0.017079	-1.782946	3.881004	4.47624	4.377553	3.752019	4.598129	4.339463	4.35355	3.891392	4.096495	4.290193	4.457635	4.136468	4.258405	4.388433
Kcnn1	-0.788127	3.998261	-3.790769	0.00187	0.017105	-1.758018	4.002595	3.822768	3.12518	4.16674	3.482634	2.845805	4.43432	4.134313	4.363967	4.0968	4.302392	4.187698	4.47088	4.645875
Polr1b	0.54812	4.218428	3.790052	0.001873	0.017117	-1.811096	3.88628	4.763603	4.519244	4.140492	4.430682	4.551685	4.197745	4.090016	4.14139	4.046664	3.853071	3.99982	4.357143	4.16536
Lrrc32	1.495556	2.700452	3.789145	0.001877	0.017131	-1.514846	2.670424	3.894655	3.945162	1.454757	3.036313	3.143674	2.450751	1.958936	2.47808	2.90068	2.66245	2.691938	2.060555	2.721621
Gng12	0.643223	4.098161	3.788902	0.001877	0.017131	-1.814962	4.158996	4.755351	4.451472	3.857074	4.796459	4.581525	3.734959	4.061624	3.91304	3.64312	3.797302	4.187698	3.766298	3.763295
Xrcc5	0.640544	5.021595	3.788449	0.001879	0.017133	-1.904109	5.002757	5.797457	5.137285	4.773515	5.669545	5.508079	4.828927	4.846924	4.656351	4.865376	4.755092	4.778284	4.763639	4.968486
Fen1	0.589687	4.182068	3.787161	0.001884	0.017165	-1.825683	4.119218	4.820078	4.43537	4.030446	4.772505	4.629061	3.981847	4.073048	3.930199	3.957219	4.180861	3.778199	4.103184	3.825682
Ltbp2	1.318236	3.069184	3.786424	0.001887	0.017177	-1.492655	2.595092	3.159427	3.560852	1.300739	2.936364	3.150929	3.727855	3.047633	3.456633	3.28495	3.099749	2.726993	3.203938	3.927469
Plp1	-1.25971	11.32808	-3.78464	0.001894	0.017226	-1.853592	11.60148	10.74348	9.70309	11.48125	10.81879	9.84822	11.4649	11.86437	11.55299	11.63717	11.33881	12.21081	12.09463	12.23386
Dtnb	-0.464902	6.212796	-3.783062	0.0019	0.017256	-1.966998	6.365967	5.744246	5.867547	6.254351	5.998646	5.770798	6.456206	6.338956	6.507862	6.412663	6.430993	6.224032	6.372548	6.25595
Dhrs9	4.77572	-0.465071	3.783022	0.0019	0.017256	-1.154601	-1.908947	0.632508	1.7408	-2.731185	1.230571	2.932562	-1.450335	0.04768	-1.813089	0.134646	0.721218	1.715048	-2.656794	-1.040339
Slc32a1	-0.800502	6.028024	-3.78263	0.001901	0.017257	-1.95083	5.741245	4.678883	5.145557	6.159507	5.178286	5.594433	6.64904	6.115493	6.610775	6.371506	6.092751	6.370712	6.685397	7.025937
Ndc1	0.732367	3.248304	3.782102	0.001903	0.017258	-1.682127	3.05476	3.563961	3.843073	3.043681	3.799498	3.879645	3.072094	3.070611	2.858731	2.600561	3.048553	3.277021	3.245776	3.286911
LOC499781	1.23923	2.647885	3.781823	0.001904	0.017258	-1.492825	1.916502	3.339488	3.759312	1.892408	2.800826	2.581593	2.787802	2.738882	2.41447	2.655119	2.531849	2.680061	2.689088	2.544088
Cox4i2	1.37238	1.636665	3.780981	0.001908	0.017274	-1.341874	1.430522	2.833367	2.298852	0.621404	2.399242	1.875381	1.047044	1.68885	1.794428	1.780488	1.290185	1.542121	1.11438	1.73247
Elovl4	-0.768892	5.005998	-3.78038	0.00191	0.017274	-1.911043	5.119368	3.9244	4.74506	5.341085	4.534033	4.623856	4.96806	5.030987	5.440337	5.16704	5.170752	5.162375	5.378131	5.529868
Wscd2	-1.251352	4.802193	-3.779571	0.001913	0.017274	-1.850864	4.865238	2.324846	3.89502	4.861085	3.981865	4.164107	5.41323	5.421231	5.473878	5.107147	5.465635	5.281137	5.666979	5.38295
Slc3a1	-0.539443	5.592893	-3.77943	0.001914	0.017274	-1.929831	5.180775	4.613433	4.868125	5.52335	4.824689	4.951331	6.334051	5.742456	6.183488	5.854072	6.034738	5.918869	6.17557	6.131254
Gpt2	-0.664653	5.000822	-3.779146	0.001915	0.017274	-1.948532	5.660403	5.005446	4.709794	5.723547	4.951718	5.454845	4.743453	4.76477	4.765539	4.633691	4.904139	4.928903	5.078103	4.737232
Cfd	2.152226	0.315748	3.779128	0.001915	0.017274	-1.148978	0.176305	0.973336	1.451597	-1.575022	1.05105	1.302125	-1.450335	1.429209	-0.632503	1.322149	0.961591	1.97042	-0.893866	0.045082
Plscr3	0.531009	4.60978	3.778219	0.001918	0.017293	-1.890416	4.692281	5.196344	4.998051	4.331596	5.042655	4.915569	4.362751	4.49437	4.401362	4.626882	4.437876	4.451452	4.200009	4.450943
Il1a	-1.920268	0.001846	-3.777474	0.001921	0.017306	-1.165601	0.995438	-1.685466	-1.705055	0.280432	-0.257548	-0.646023	0.798508	0.645799	0.317129	1.21727	0.885831	1.433124	-0.211665	-0.03329
Prkx	0.595914	3.935436	3.776772	0.001924	0.017317	-1.814621	3.92781	4.138342	4.496143	3.742078	4.581681	4.467019	3.462819	3.838823	3.689436	3.709073	3.882605	3.906064	3.800389	3.558103
Bpifb4	1.963604	0.284066	3.775316	0.001929	0.017355	-1.155692	-0.760787	1.874793	1.089503	-0.705589	0.845972	0.61372	-0.672244	1.067371	-0.386063	1.21727	0.321999	1.11804	0.111637	-0.297732
S1pr2	0.839573	3.272461	3.773338	0.001937	0.017411	-1.710899	3.180343	4.064569	3.975174	3.002635	3.943761	3.708268	2.433388	3.137429	2.667343	3.177227	3.022257	3.848862	2.860463	2.962789
Stx11	1.444316	0.742423	3.771109	0.001946	0.01746	-1.261731	0.105685	1.480065	1.775325	0.577356	1.803478	1.875381	0.357848	-0.380636	1.061401	0.750369	0.631286	0.28321	0.037347	1.048408
Col6a2	1.438849	6.135133	3.770926	0.001947	0.01746	-1.961632	5.95468	6.810777	6.554625	4.370833	6.541253	6.436895	6.236068	5.615391	6.386717	6.130458	6.046746	5.718619	5.854432	7.259595
Rrp9	0.645294	5.254451	3.770796	0.001947	0.01746	-1.937814	5.019661	5.68321	5.601424	4.706082	5.463549	5.268955	5.505426	5.048455	5.479717	5.27332	5.126156	5.085673	4.982355	5.360138
Trim37	-0.850818	7.274453	-3.77046	0.001948	0.01746	-1.991401	7.707812	6.740056	6.919804	8.196885	7.197825	7.54537	7.207884	7.168661	7.146809	6.865118	7.36883	7.152788	7.590388	7.044538
Dip2c	-0.700096	6.158144	-3.769196	0.001953	0.017492	-1.994678	6.277378	5.753943	5.987435	6.556871	5.343487	5.786023	6.389122	6.431727	6.324268	5.988952	6.410018	6.169858	6.621679	6.196023
Arl6	-0.731577	3.84302	-3.768313	0.001957	0.017507	-1.832005	4.087502	3.167741	3.604709	4.443178	3.488528	3.879645	3.945565	3.770297	4.165741	3.588098	4.039427	3.755742	4.152409	3.825682
Cryab	0.837655	5.783068	3.768011	0.001958	0.017507	-1.995572	5.991867	6.82596	7.235587	5.831792	6.761691	6.169453	5.061434	5.068569	5.180786	5.233536	5.452566	5.519733	5.266712	5.394223
Susd4	-0.583373	6.815794	-3.765664	0.001967	0.017577	-2.009627	7.079733	6.335671	6.681781	6.802044	6.008935	6.403872	6.991393	6.979773	7.112144	6.896595	6.935429	6.98976	7.101209	7.117199
Rps2	1.254706	5.70361	3.764613	0.001972	0.017597	-1.907559	4.70428	6.256029	5.362618	3.866255	5.062547	5.431202	6.387996	6.439471	6.062907	6.025651	5.555479	5.760108	6.468433	6.503296
Eif5	0.532461	6.401211	3.764251	0.001973	0.017597	-2.009513	6.123917	6.80679	6.824107	6.507846	6.727212	7.036538	6.341051	5.911074	6.212998	6.244936	6.232158	6.136567	6.238055	6.292556
Mcm4	0.678849	4.16856	3.763993	0.001974	0.017597	-1.852709	3.608352	4.302134	4.586407	4.199801	4.833978	4.631657	4.223227	3.838823	4.086326	3.901891	4.086719	3.921279	4.080242	4.147707
Bhlhe23	-2.574672	0.929918	-3.761973	0.001982	0.017656	-1.272968	1.401336	-2.680539	0.851376	2.184345	-0.613814	0.284862	1.754571	1.067371	1.690848	1.804823	1.903329	1.618784	2.151407	1.32462
Cecr2	-0.994544	4.423032	-3.761286	0.001985	0.017657	-1.888771	4.119218	3.833259	4.015447	5.307775	3.313654	3.775529	4.785113	4.803005	4.893527	4.592349	4.390692	4.662336	4.737483	4.772214
LOC64038	0.61713	4.394562	3.761204	0.001985	0.017657	-1.907333	4.658759	4.946264	4.79429	3.993213	5.09188	4.896271	3.96382	4.294162	3.991425	4.441427	4.380377	4.327499	3.828196	3.993192
Mgst2	2.467779	-0.158102	3.760076	0.00199	0.017684	-1.203238	0.031429	2.232748	2.47722	-1.075778	0.731352	1.585258	-0.399109	-1.196868	-0.773548	-1.136114	-0.731011	0.007173	-1.047095	-0.748265
Scn8a	-0.995274	7.945355	-3.757989	0.001998	0.017746	-2.016035	7.58907	6.747009	7.48248	8.419626	6.631911	7.170193	8.511859	8.611723	8.561335	7.952535	8.349771	8.249393	8.748464	8.216875
Stk17b	0.793872	3.237115	3.75669	0.002003	0.017779	-1.793537	3.639931	4.184143	4.53192	3.152759	3.922149	4.044542	2.760215	2.505864	2.524011	2.831299	2.81321	3.037402	2.994118	2.55752
Rpl5	0.437621	7.395423	3.756236	0.002005	0.017782	-2.017406	7.702214	8.042045	8.063237	7.646113	8.167543	8.174574	6.839851	7.201562	6.911487	7.071011	7.03633	6.987955	6.724393	6.977422
Vwc2	-0.98173	1.701614	-3.755193	0.002009	0.017806	-1.454635	2.127257	1.010791	1.061799	2.013053	1.364797	1.014597	2.01015	1.318383	1.819202	1.942797	2.083688	2.118577	2.301782	2.142093
Scrg1	-0.750774	4.446908	-3.75467	0.002011	0.01781	-1.90779	4.859884	4.129856	3.834912	4.440107	4.12116	3.524966	4.682297	4.384043	4.765539	4.751052	4.363019	4.851593	4.719778	4.904232
Grm1	-1.444627	6.314783	-3.753846	0.002015	0.017828	-2.019786	6.063484	4.175923	5.521053	7.346111	5.228454	6.09241	6.791271	6.704789	6.868369	6.629987	6.56758	6.651802	6.925712	6.867135
Usp2	-0.620278	5.423654	-3.753373	0.002017	0.017828	-2.003376	5.88391	4.965467	5.390726	5.573263	4.895159	5.168865	5.44173	5.619293	5.514264	5.582203	5.857683	5.440987	5.42623	5.208755
Brca1	1.515322	1.306023	3.753118	0.002018	0.017828	-1.392638	0.915118	2.294795	1.792283	1.033666	2.640673	3.129052	0.851758	0.517885	1.061401	0.981432	0.535373	1.375373	0.921344	0.929479
Dear	-0.628796	3.2729	-3.752351	0.002021	0.017834	-1.8432	3.875708	3.713429	3.463639	4.267203	3.245679	3.408717	3.299932	3.126505	3.130231	2.843096	2.696109	2.749899	3.079891	3.088394
Prkaa2	-1.379917	3.050191	-3.752172	0.002022	0.017834	-1.81316	3.963192	2.082541	3.295418	4.538155	2.79132	3.295428	2.703406	3.104407	3.027678	2.127786	3.387587	2.375822	3.772036	2.446395
Igtp	1.550082	3.219672	3.751867	0.002023	0.017834	-1.642715	2.832262	4.03306	4.514142	1.856	3.534831	3.308464	2.717819	3.591194	2.568526	3.51623	3.338699	4.353904	2.379013	2.721621
Aff3	-0.859434	5.554979	-3.749257	0.002033	0.017909	-2.016757	5.719239	4.864231	5.181399	6.179902	5.002029	5.316512	5.611511	5.827542	5.709103	5.235776	5.889551	5.552674	6.244261	5.470756
Farsa	0.575325	5.497904	3.748798	0.002035	0.017909	-1.993823	5.218914	5.584194	5.631182	4.867958	5.900385	5.342094	5.630681	5.482485	5.677274	5.589225	5.518329	5.405362	5.553287	5.604558
Nsun7	-0.810925	2.373071	-3.748683	0.002036	0.017909	-1.587617	2.682606	1.702379	2.162035	2.634716	1.664612	1.875381	2.854546	2.454377	2.95129	2.342482	2.604547	1.950783	2.760099	2.900911
Gpr17	-1.174478	4.055771	-3.747224	0.002042	0.017949	-1.915274	4.97826	3.914553	3.495051	4.616421	3.568609	3.547133	3.593621	4.342165	3.577725	3.844356	4.004032	4.975806	4.731605	3.692011
Tenm2	-0.826618	7.377584	-3.743367	0.002058	0.018076	-2.052714	6.987005	6.309609	6.907697	7.924598	6.553253	6.739049	7.810901	7.742136	8.000574	7.340741	7.467551	7.442786	8.068381	8.002217
Ier2	1.210595	3.758123	3.741036	0.002067	0.018147	-1.80958	3.467609	4.515857	4.709794	2.994284	4.7007	3.76611	3.804135	3.201289	4.018418	3.784431	3.53126	3.848862	3.333761	3.060363
Camta2	-0.487992	9.777729	-3.739314	0.002075	0.018197	-1.994213	9.8981	9.245494	9.281185	9.901634	9.512439	9.60956	10.09698	9.86226	10.0253	9.928048	9.802223	9.823689	9.940958	9.962165
Dus4l	0.869865	2.412975	3.73879	0.002077	0.018203	-1.596824	2.054131	3.099845	2.700244	2.093442	2.987204	2.974059	2.534559	1.934092	2.47808	2.39151	2.481265	2.36102	2.041677	1.951771
Trib2	-0.811838	5.226237	-3.736786	0.002085	0.018262	-2.045469	5.605478	5.097537	4.893885	6.406069	5.163626	5.638975	5.261619	4.796129	5.024458	5.067468	5.094955	4.898474	5.25856	5.003257
Evi2a	-1.068789	6.047647	-3.735846	0.002089	0.018262	-2.056935	6.416986	5.169685	4.766671	6.484931	6.025245	5.581066	5.821279	6.384369	5.893911	6.339588	6.180579	6.86745	6.413506	6.347713
Rpl41	0.867498	5.742674	3.735809	0.002089	0.018262	-2.034046	5.616631	6.363082	5.836206	4.861085	6.449054	5.750642	5.684875	5.670955	5.684033	6.007418	5.891974	5.54123	5.493764	5.576794
Mtmr11	1.251861	4.650183	3.735525	0.00209	0.018262	-1.951795	4.481855	5.97968	5.338289	3.82447	5.190992	5.06899	4.739927	4.490115	4.549366	4.938684	3.925804	4.338875	3.695599	4.607648
Spock1	-0.805214	7.086292	-3.73537	0.002091	0.018262	-2.070239	6.935763	5.78805	6.693111	7.263824	6.357016	6.33706	7.395527	7.49171	7.470847	7.099571	7.386977	7.417078	7.906302	7.677978
Isg15	1.632449	2.373882	3.733465	0.002099	0.018319	-1.577797	2.460078	2.963229	3.688616	1.128291	3.159643	3.585123	1.726334	2.68033	1.180909	2.866406	2.228732	3.692118	0.921344	1.32462
Kcnj8	1.320206	2.507847	3.732979	0.002101	0.018324	-1.532575	1.764345	3.479652	2.869592	1.405223	2.640673	2.549011	2.967416	2.665313	2.583064	3.070874	2.374443	2.691938	2.097585	2.245294
Mthfd1	0.50611	4.925779	3.73222	0.002104	0.01833	-1.998378	4.748409	5.163462	4.981512	4.616421	5.431783	5.170651	4.931426	4.943446	4.781385	4.922095	4.852835	4.851593	4.78366	4.834225
Rps18	1.936648	1.772613	3.732107	0.002105	0.01833	-1.325064	0.692264	2.570375	2.09528	-0.325128	2.046579	1.378286	2.380012	1.747064	2.364852	2.745897	1.978199	1.691577	1.747621	2.261799
Anxa7	0.565365	6.240554	3.730575	0.002111	0.018361	-2.075702	6.148277	6.546417	6.866257	6.200013	6.739003	6.804347	5.953525	6.064895	6.097495	5.815669	6.231202	5.940188	6.215071	5.766569
Shroom2	-0.907243	6.598094	-3.730553	0.002112	0.018361	-2.079522	6.933854	5.675942	6.289424	7.349389	6.479707	6.490069	6.513096	6.686311	6.630156	6.249376	6.891311	6.584904	7.094355	6.522257
Atp1a3	-0.757708	12.16244	-3.729542	0.002116	0.018374	-1.935834	11.95062	10.9444	11.41728	12.37609	11.61753	11.64295	12.68007	12.44023	12.83923	12.44856	12.18452	12.38788	12.48233	12.86284
Htra1	0.758676	8.030733	3.729479	0.002116	0.018374	-2.062693	8.013788	8.936823	8.839008	7.808829	8.215639	8.688835	7.923221	7.271407	7.907617	7.788334	7.343389	7.801498	7.804643	8.093504
Arhgdia	0.657888	8.80201	3.72882	0.002119	0.018385	-2.044764	8.968811	9.298158	9.236775	8.297887	9.532457	9.105494	8.256023	8.662235	8.50805	8.678872	8.845289	8.756616	8.57674	8.508332

Rab15	-0.636155	7.918632	-3.728306	0.002121	0.018391	-2.06735	8.452811	7.296389	7.697456	8.183633	7.679292	8.063139	7.994136	7.870438	7.970825	7.869168	7.950006	7.80731	8.133349	7.899497
Ugdh	0.474623	4.880543	3.726885	0.002127	0.01843	-2.015849	4.69529	5.232463	5.238194	4.837937	5.288806	5.207652	4.874668	4.548571	4.830952	4.859579	4.678581	4.769904	4.602301	4.716427
Alcam	-0.887223	6.955314	-3.724507	0.002137	0.018504	-2.091694	7.03886	5.753943	6.396575	7.452433	6.394382	6.885424	7.097853	7.021646	7.49363	6.828496	6.890706	7.024225	7.471555	7.638235
Atp2a2	-0.678222	10.15861	-3.723854	0.00214	0.018504	-2.018969	9.869663	9.052884	9.536022	10.41925	9.533348	9.740749	10.65186	10.38683	10.61238	10.28976	10.34832	10.41927	10.63965	10.7221
Nkiras1	-0.768123	6.416079	-3.723823	0.00214	0.018504	-2.090224	6.573181	5.5207	6.093852	6.769381	5.967334	6.028851	6.526463	6.632313	6.588432	6.38972	6.917111	6.439254	7.036533	6.361179
Stat5a	1.000108	2.667024	3.722298	0.002147	0.018548	-1.665962	2.694686	3.432128	3.240874	1.799605	3.122223	3.061358	2.078759	3.024282	2.00342	2.923085	2.428842	3.195935	2.348615	2.245294
Slitrk6	-1.443898	1.70675	-3.721926	0.002149	0.018548	-1.569199	2.091157	1.184734	0.785152	2.994284	1.78444	0.915838	1.836107	1.80302	2.04602	0.893396	0.846404	1.715048	2.805569	2.733504
Cenpn	0.764814	2.253318	3.721388	0.002151	0.018555	-1.602432	2.035255	2.690649	2.456093	1.856	2.962008	2.685174	2.01015	2.38274	2.024877	2.108365	2.259075	2.187314	2.133689	2.087586
Erbb4	-1.531062	3.40671	-3.72004	0.002157	0.018592	-1.860071	3.759759	2.904741	3.158259	4.656666	1.997824	2.734303	3.231264	3.929599	3.669138	2.586593	3.518775	3.502822	4.640443	3.571405
Mme	-1.199366	1.502004	-3.719637	0.002159	0.018594	-1.471323	1.644914	1.339943	0.484899	2.045748	0.320024	0.734546	2.05625	2.307359	2.260183	1.652245	1.581856	1.760874	1.651284	1.80183
Lzts3	-0.607343	8.025317	-3.718874	0.002162	0.018609	-2.096078	7.695842	7.276345	7.411545	8.031509	7.159549	7.176428	8.640131	8.486761	8.641224	8.611922	8.220813	8.284135	8.179144	8.545669
Wdr1	0.589995	8.498444	3.717622	0.002167	0.01864	-2.071827	8.48425	8.742483	9.340662	8.545169	9.10042	9.242123	8.159011	8.234183	8.165122	8.05785	8.341597	8.186244	8.341102	8.042476
Ppan	0.662619	5.695763	3.717342	0.002169	0.01864	-2.084802	5.600671	6.487026	6.28344	5.485121	6.1532	5.893742	5.679362	5.400976	5.685718	5.698792	5.32032	5.251348	5.25856	5.573492
Baiap2	-0.646127	7.245945	-3.716564	0.002172	0.018656	-2.10709	7.280922	6.441464	6.859736	7.618192	6.765944	7.14498	7.393845	7.598832	7.497952	7.36447	7.462263	7.267357	7.485592	7.272379
Jakmip3	-0.719342	5.688666	-3.714154	0.002182	0.018734	-2.077529	5.455594	5.449176	4.770955	5.899318	4.974995	4.675081	6.19158	5.999045	6.123835	6.081498	5.78395	6.080069	5.983686	6.205652
Siglec10	2.20136	-0.079713	3.71289	0.002188	0.018768	-1.249956	-0.899176	0.533043	1.363571	-1.388744	0.649545	0.695385	-0.169545	0.923327	-0.27702	0.534523	-0.231835	-0.070959	-1.218554	0.321874
Klhl14	-1.059867	2.4435	-3.71173	0.002193	0.018795	-1.721858	2.917193	1.58312	1.792283	3.261145	2.919011	1.910004	3.094359	1.958936	3.280514	2.184529	1.430105	1.930875	3.098279	3.169358
Ift46	-0.446718	5.556246	-3.711463	0.002194	0.018795	-2.100452	5.967183	5.39532	5.323783	5.87678	5.618052	5.566219	5.428646	5.454368	5.523715	5.49889	5.665763	5.497911	5.522982	5.481365
Emilin1	0.894235	3.897506	3.709279	0.002204	0.018852	-1.861832	3.627383	4.368558	3.906747	2.806722	3.926497	4.164107	3.844102	4.208703	3.709451	4.361652	4.030659	4.390807	3.372046	3.958175
Pls1	-0.862712	3.347658	-3.709269	0.002204	0.018852	-1.829451	3.582581	2.854122	2.456093	3.550328	2.892584	2.675144	3.504983	3.653399	3.81791	3.62956	3.082885	3.975732	3.866246	3.489688
Plac8	2.596773	-0.428596	3.708491	0.002208	0.018868	-1.276044	-0.634519	1.894965	1.451597	-1.075778	1.173177	1.688586	0.282279	-1.435005	-1.536754	-0.806258	0.378374	-2.668207	-0.893866	-0.748265
Rpsa	1.104003	7.221906	3.706605	0.002216	0.018909	-2.124176	7.027563	7.94047	7.290701	5.760949	7.751225	6.992792	7.291566	7.233729	7.245499	7.659984	7.36142	7.289512	6.988053	7.284545
Ccdc81	1.211658	2.369416	3.70652	0.002216	0.018909	-1.718376	2.706665	3.769134	3.265371	1.92792	3.203302	3.770827	2.078759	2.505864	1.690848	2.007132	1.068256	2.027776	2.022549	1.473753
Unc5c	-1.067144	6.009095	-3.706358	0.002217	0.018909	-2.115428	5.726612	5.097537	5.110492	7.109516	5.425653	5.782524	6.37555	5.942556	6.357417	5.888637	6.053254	6.20136	6.696755	6.385859
RGD1309079	-0.795553	5.015018	-3.704823	0.002224	0.018943	-2.082923	5.19997	4.171795	4.59126	5.802197	4.951718	5.114234	5.003787	5.016268	5.076761	5.054841	5.034842	5.147358	4.994677	5.100515
RGD1565641	0.545548	3.79016	3.704761	0.002224	0.018943	-1.982399	4.105711	4.499482	4.464755	3.924553	4.824689	4.434686	3.383673	3.169712	3.333065	3.293574	3.408041	3.30822	3.562873	3.468514
Ccp110	-0.608507	6.541727	-3.70385	0.002228	0.018964	-2.132119	6.464499	6.115893	5.82492	6.938881	6.245713	6.192489	6.785295	6.598126	6.675802	6.743709	6.460671	6.844355	6.76668	6.944573
Oxnad1	0.562053	4.418038	3.701509	0.002239	0.01903	-2.040794	4.45014	4.921894	5.145557	4.476537	4.979188	5.043877	4.223227	3.929599	4.231815	4.285878	4.338363	4.00459	3.822677	4.074854
Rapgef5	-0.749183	7.559262	-3.701421	0.002239	0.01903	-2.133059	7.536766	6.620975	7.13532	8.047993	6.913434	7.50099	7.725658	7.810275	7.676761	7.916348	7.779939	7.847687	7.816838	7.509394
Tap1	1.514436	3.888599	3.700842	0.002242	0.019039	-1.837401	3.474632	4.379337	5.003522	2.283631	4.05518	3.952041	3.347054	4.402272	3.216682	4.349296	4.124292	5.173007	3.061266	3.739923
Sat2	-0.562191	5.441314	-3.700431	0.002244	0.019042	-2.120997	6.049442	5.647985	5.158693	5.814108	5.295546	5.387335	5.519893	5.321786	5.332613	5.526589	5.388815	5.319923	5.105631	5.346662
Rnf128	-1.193987	4.084016	-3.699822	0.002246	0.019051	-1.885738	3.753999	1.632013	2.761803	4.144271	3.500244	3.114281	4.835551	4.768288	5.008391	4.475935	4.480351	4.637984	5.05004	5.138994
Kifc1	2.192093	1.398521	3.698773	0.002251	0.019078	-1.421995	0.485316	2.383133	1.451597	0.280432	3.293596	2.949304	1.258992	1.532126	1.180909	0.981432	1.135223	1.568131	0.598632	1.158276
Tmem130	-0.865229	9.840384	-3.697988	0.002255	0.019095	-2.084471	9.641417	8.319423	8.907729	9.992748	9.316177	9.266752	10.17389	10.2711	10.43866	10.35507	10.10869	10.10662	10.29696	10.57208
Rbbp9	-0.479796	4.263574	-3.695621	0.002266	0.019166	-2.059505	4.857199	4.379337	4.223544	4.835601	4.279114	4.594888	3.96382	4.117861	3.974983	4.041554	4.188764	4.083352	4.134703	4.098018
Mycl	-0.878312	3.023199	-3.695456	0.002266	0.019166	-1.799233	3.197423	2.232748	2.548818	3.315784	2.361201	2.398644	2.746221	3.784265	2.785194	3.267546	3.512491	3.906064	3.502026	2.972849
Sell	3.969609	-1.398943	3.694849	0.002269	0.019176	-1.27903	-1.642648	1.894965	3.387569	-1.223791	2.575715	0.949514	-2.558588	-2.550306	-2.155202	-2.176425	-1.310338	-2.668207	-3.260591	-1.632384
Slc2a1	1.105814	7.747408	3.692795	0.002279	0.019242	-2.146178	7.810946	9.038363	8.721645	6.839822	8.122297	7.842282	7.591699	7.308729	7.634639	7.564337	7.129094	7.631529	7.478152	7.757963
Pde8b	-0.495308	5.093851	-3.690015	0.002291	0.019337	-2.115979	5.566568	5.060063	4.841896	5.277123	4.779733	5.026231	5.224248	4.998404	5.142036	5.042103	5.026046	5.204441	4.997129	5.174296
Gcom1	0.994651	1.351637	3.688549	0.002298	0.019369	-1.523442	1.071521	2.309899	1.905712	1.128291	2.124346	1.976852	1.000645	1.532126	0.628769	1.28803	0.998029	1.186803	0.962073	1.445134
Diras2	-1.512886	6.458904	-3.688498	0.002298	0.019369	-2.159472	6.912033	4.522355	5.805215	7.507018	5.964165	6.475695	6.495427	6.982805	6.615203	6.238251	6.980515	6.478354	7.19603	6.272334
Sipa1l1	-0.649178	7.923803	-3.685646	0.002312	0.019458	-2.156002	8.17687	7.171721	7.47495	8.247391	7.692756	7.915199	7.997093	8.130703	8.007327	7.844436	8.10029	8.038258	8.226668	7.91621
Mei1	-1.764637	5.547006	-3.685531	0.002312	0.019458	-2.100036	5.434139	2.914655	5.569811	6.140712	3.174343	4.329906	6.598333	6.122382	6.1811	6.164943	6.115732	5.938943	6.335245	6.690191
Rwdd1	0.522756	4.96417	3.68457	0.002317	0.019482	-2.126956	4.968344	5.352198	5.691179	5.252825	5.778194	5.711921	4.678618	4.477275	4.666626	4.673881	4.655954	4.565594	4.529643	4.548339
Pdgfc	1.099101	2.358774	3.684035	0.002319	0.019489	-1.73121	2.776524	3.159427	2.954902	1.571628	3.231692	3.563537	1.754571	2.227821	1.549949	2.027955	2.442128	2.761217	1.700256	1.634478
Pclo	-1.210236	8.198442	-3.683202	0.002323	0.019497	-2.156202	7.89285	7.293543	7.395681	9.196515	6.828853	7.804249	8.598872	8.780843	8.67215	8.013187	8.519767	8.316862	9.082603	8.388271
Unc13a	-0.610671	8.534912	-3.683135	0.002323	0.019497	-2.155638	8.22243	7.960946	7.864359	8.623574	7.452722	7.966216	9.214445	8.927097	9.112894	8.79977	8.685157	8.743658	8.905335	9.014761
Sfxn5	-0.763097	6.601317	-3.68096	0.002334	0.019569	-2.179707	7.632888	6.730968	6.721608	7.019794	6.351367	6.455327	6.439997	6.072029	6.253324	6.350995	6.359274	6.726602	6.773132	6.547718
Sp7	-1.732848	0.260662	-3.680447	0.002336	0.019576	-1.3715	1.108109	-0.186163	-0.761158	0.531921	-0.424712	-1.612049	0.202532	0.76329	0.787836	0.799637	0.484915	1.345607	0.879431	1.32462
Phka1	-0.59656	4.378083	-3.679941	0.002338	0.019582	-2.072173	4.67714	3.859159	4.126591	4.743933	4.248182	4.220367	4.531353	4.388622	4.449761	4.046664	4.363019	4.526331	4.777968	4.410806
Hcrtr2	-2.069338	0.759649	-3.678191	0.002347	0.019638	-1.48817	1.212615	-0.687936	-0.491181	2.523044	-0.613814	1.563674	1.408645	1.020936	0.787836	0.475109	0.484915	1.404538	1.837926	0.844371
Chst7	-1.060061	3.481635	-3.67724	0.002351	0.019648	-1.938148	3.481621	2.532247	2.548818	4.344794	2.847434	3.57437	3.554005	3.698353	3.722643	3.377094	3.673224	3.738666	4.13915	3.661237
Slc12a6	-0.64346	6.985754	-3.677235	0.002351	0.019648	-2.188166	6.937671	6.139334	6.684054	7.419676	6.487821	6.827172	7.295175	7.025326	7.261969	6.89447	7.036877	7.093021	7.321653	7.38919
Nmb	-3.248858	1.937301	-3.6764	0.002355	0.019668	-1.470757	2.742017	-3.260591	0.442561	2.297273	-0.038319	1.627481	3.060831	3.081965	3.307029	3.424979	2.883067	3.212521	3.73726	3.012401
Ssr4	0.818611	5.145791	3.675691	0.002359	0.019682	-2.112832	4.995452	5.452583	5.427823	4.256768	5.83943	5.016334	5.000844	4.983347	5.07419	5.235776	5.248409	5.216826	5.033414	5.305462
Tox3	-0.629977	3.802441	-3.674807	0.002363	0.019704	-2.020587	4.167689	3.208609	3.89894	4.291262	3.716556	3.552622	3.889373	3.606999	3.980484	3.844356	3.656224	3.838214	3.748946	3.948012
Pde10a	-1.017943	5.190505	-3.673117	0.002371	0.019757	-2.142235	5.112631	4.208526	5.062374	5.977659	4.173318	4.66498	5.486611	5.599679	5.450283	5.094864	5.600609	5.285063	5.816065	5.18082
Lpar4	-1.033424	1.552281	-3.672428	0.002374	0.019771	-1.69649	2.372556	1.248835	0.975347	2.666599	1.643636	2.217002	1.17791	1.564863	1.142163	1.355481	1.199219	1.618784	1.574559	1.529341
Aig1	-0.754062	6.020658	-3.671242	0.00238	0.019796	-2.181029	5.913928	4.769077	5.187821	6.218951	5.557661	5.731411	6.239818	6.334216	6.424544	6.392733	6.237883	6.353055	6.534063	6.420268
Nme2	0.684891	6.841048	3.671105	0.00238	0.019796	-2.198916	6.977767	7.557921	7.218046	6.299956	7.428016	7.08327	6.782727	6.571188	6.715108	6.859321	6.706179	6.49455	6.609712	6.484961
Pla2g2d	3.832342	-1.107327	3.669725	0.002387	0.019838	-1.319329	-1.908947	0.895377	1.116684	-1.575022	2.399242	2.336365	-1.73438	-0.992527	-1.10526	-0.961779	-2.697527	-0.657145	-3.260591	-1.632384
Mtss1	-0.510702	6.30002	-3.669286	0.002389	0.019842	-2.19885	6.271342	5.775864	5.896262	6.63684	6.058313	6.046464	6.369857	6.394648	6.454503	6.352027	6.253981	6.469756	6.607307	6.633452
Grid1	-0.638579	5.534922	-3.668584	0.002393	0.019856	-2.174101	5.613453	4.707679	5.415563	5.851575	5.062547	5.18132	5.645836	5.78302	5.732115	5.408486	5.697961	5.772746	5.839481	5.812105
Inpp5d	0.784045	5.294228	3.66663	0.002402	0.01992	-2.15656	5.32763	5.966635	5.798935	4.597249	5.842885	5.34684	4.795342	5.715373	4.549366	5.708498	5.460748	5.975843	4.491894	4.584872
Dnase2	1.118638	5.234356	3.665345	0.002408	0.019959	-2.141657	5.414188	5.910767	5.373927	3.942027	6.151343	5.674651	4.316148	5.839298	4.19443	5.901388	5.719944	6.133304	4.182881	4.575
Ints4	0.469451	5.170423	3.664397	0.002413	0.019983	-2.153977	4.875886	5.530404	5.375335	4.971562	5.462053	5.209391	4.871449	5.214976	4.953501	5.305655	5.246517	5.200289	5.082728	5.130032
Lphn1	-0.54645	8.94283	-3.661908	0.002425	0.02006	-2.186503	8.788942	8.20416	8.557768	8.866266	7.949059	8.417092	9.386141	9.319003	9.364251	9.221895	9.142217	9.291232	9.36729	9.327718
Chst5	-1.131907	1.389969	-3.661394	0.002428	0.02006	-1.581474	1.936985	0.854759	0.484899	1.679737	0.649545	0.881356	1.511505	1.429209	1.520039	1.852296	1.375754	2.419337	1.724133	1.779078
Zc3hav1	1.069423	3.036224	3.661301	0.002428	0.02006	-1.832205	2.906846	3.418258	3.818451	2.045748	3.259533	3.524966	3.083269	3.292072	2.55384	2.998871	3.03984	3.721386	2.689088	2.357047
Psmb4	0.66482	6.12247	3.661123	0.002429	0.02006	-2.202967	5.988191	6.524735	6.3938	5.332829	6.348129	6.016987	6.257191	5.922171	6.232726	6.155512	6.289327	6.009452	6.163614	6.102907
Ptpla	0.832959	2.176127	3.660072	0.002434	0.020078	-1.660905	1.764345	2.595246	2.250588	1.430203	2.530726	2.271274	2.450751	2.099634	2.41447	2.239124	2.133674	2.300247	2.060555	2.278116
Rbm24	-0.946717	3.682043	-3.660009	0.002434	0.020078	-2.015962	3.742407	2.747223	2.932131	4.523721	3.551819	3.57437	4.123906	3.825375	3.901486	3.808701	3.981461	3.738666	3.515771	3.710165
Dynll2	-0.696596	8.825159	-3.658979	0.002439	0.020106	-2.177554	9.146694	8.293538	8.534966	9.52979	8.807817	8.92788	8.717479	8.691945	8.871295	8.651038	8.972914	8.711382	9.066497	8.632513
Lamp5	-0.942086	5.810903	-3.657784	0.002445	0.020141	-2.210393	6.085441	4.733113	5.757442	6.405282	5.586799	5.121639	5.521948	5.756695	6.083504	5.987624	5.879821	5.950112	6.395033	6.117717
Tyw5	0.773465	3.003324	3.657251	0.002448	0.020149	-1.88564	2.810225	3.86939	3.575619	2.748327	3.529124	3.22842	3.01488	2.862532	2.772565	2.854798	2.892776	2.433555	2.652228	3.002614
Trim32	-0.718465	6.331922	-3.656309	0.002453	0.020173	-2.228202	6.672721	5.56071	6.085277	6.891688	6.199749	6.40842	6.27801	6.263683	6.465333	6.146019	6.574383	6.272295	6.560828	6.287527
Irak3	0.991208	3.025238	3.655258	0.002458	0.020193	-1.885027	2.885927	3.934181	3.719973	2.534609	3.691225	3.408717	2.550752	3.159031	2.260183	3.241039	2.673757	3.323571	2.041677	3.133933
Vsnl1	-1.036459	8.519612	-3.654911	0.00246	0.020193	-2.181865	9.062824	7.903921	8.642909	10.04851	8.740726	8.78232	8.483143	8.199422	8.348348	7.996117	8.485088	8.032723	8.539798	8.013226
Tacc2	-0.508152	7.419952	-3.654798	0.00246	0.020193	-2.228018	7.550125	6.900813	7.206627	7.82599	7.32432	7.28758	7.441846	7.587425	7.49939	7.491714	7.326026	7.489468	7.435853	7.521438
Tnfrsf9	2.208042	0.354968	3.654415	0.002462	0.020195	-1.413736	0.105685	1.725091	2.607481	-0.81875	1.20216	1.193889	-0.067151	0.76329	-0.080923	1.064403	0.071497	0.007173	-1.047095	-0.297732
Dap	0.801984	4.605092	3.653144	0.002468	0.020221	-2.15733	5.087656	5.303982	5.230438	4.1518	5.709204	5.388871	3.776861	4.379449	4.121609	4.414005	4.778847	4.552625	3.754753	3.890799
Sh2b2	1.068462	3.196589	3.653095	0.002469	0.020221	-1.867403	2.896425	3.780021	3.855228	2.255954	3.827669	3.014396	2.343298	3.858761	2.00342	4.020928	3.892318	4.277141	2.236876	2.685374
Tpmt	0.678503	3.083013	3.652587	0.002471	0.020228	-1.996218	3.529616	4.073446	3.875261	3.233029	4.136256	4.120423	2.688847	2.14364	2.493553	2.308845	2.968181	2.581351	2.736815	2.474989
Nudt12	-0.632109	4.586513	-3.652073	0.002474	0.020235	-2.179754	4.958358	4.528825	4.551972	5.633655	4.680246	4.919823	4.34429	4.337436	4.534483	4.2553	4.32049	4.179285	4.611932	4.421864
Chn1	-1.006102	10.7995	-3.651374	0.002477	0.02025	-2.113406	11.41205	9.998424	10.64591	11.96574	10.98511	11.10193	10.50967	10.83989	10.77534	10.53642	10.8847	10.46897	10.76075	10.30905
Smad7	-0.645503	5.009622	-3.650391	0.002482	0.020277	-2.185126	5.137181	4.40418	4.459457	5.521898	4.886257	5.000356	4.874668	5.181064	5.05867	5.054841	5.126156	5.253353	5.191633	5.034811
Slc16a3	1.654848	1.914867	3.649415	0.002487	0.020303	-1.59471	1.916502	2.279532	2.932131	0.041976	2.619344	2.230763	1.372662	2.054242	1.29127	2.375352	2.544222	1.950783	1.465469	2.211707
Cdc6	2.382233	0.451313	3.648888	0.00249	0.020305	-1.483636	0.176305	2.631765	1.245402	0.041976	2.79132	3.045873	-0.672244	-0.26106	0.317129	0.475109	-0.991788	-0.657145	-0.512812	0.190106
Trpc7	-1.257539	1.400235	-3.648691	0.002491	0.020305	-1.56814	1.401336	0.24948	0.398943	1.856	1.113405	-0.006501	2.186319	1.318383	2.107651	1.355481	1.864385	1.219993	2.614401	2.622863
Mid1ip1	0.593923	6.570034	3.647629	0.002496	0.020335	-2.245286	6.676532	7.107802	6.817392	6.07887	6.999018	6.955468	6.236068	6.452651	6.24649	6.624016	6.354891	6.763651	6.363075	6.321382
Prr13	0.656224	5.887767	3.645125	0.002509	0.02041	-2.246293	6.101408	6.286889	6.839994	6.029663	6.842724	6.912891	5.032899	5.625125	5.420236	5.370269	5.795641	5.648735	5.40053	5.150119
Chchd10	-0.626406	7.772683	-3.645122	0.002509	0.02041	-2.233416	8.503691	7.518645	7.679624	8.260073	8.132919	7.695881	7.611132	7.552145	7.803607	7.53736	7.859763	7.410354	7.671678	7.587989
Rab9a	0.50909	4.689075	3.643921	0.002515	0.020446	-2.190094	4.840987	5.375716	5.342612	4.856485	5.58405	5.127168	4.292269	4.213873	4.245589	4.373903	4.53717	4.281077	4.372333	4.266966
Map3k6	2.97688	1.836139	3.643459	0.002517	0.020451	-1.636451	1.401336	4.741493	4.924971	0.577356	2.781752	3.114281	1.091998	1.067371	1.061401	1.51148	0.202178	1.186803	1.28461	1.355706
Slco3a1	-0.777081	4.920109	-3.641413	0.002527	0.02052	-2.168817	4.938178	3.752647	4.096187	5.021729	4.705768	4.254448	5.114144	5.3896	5.102217	5.107147	5.162729	5.389039	5.584601	5.319327
Tdrd5	-1.407071	1.737146	-3.641104	0.002529	0.02052	-1.657977	1.371547	0.480615	0.484899	2.890108	0.952152	1.302125	2.361772	2.268138	2.166756	1.964563	2.259075	2.2368	2.253378	1.868007
Sec14l5	-0.867245	3.16084	-3.640779	0.002531	0.02052	-1.843738	3.035892	2.200694	1.792283	2.768056	2.373993	1.839907	3.830902	3.966821	4.091419	3.530893	3.380703	3.83286	3.959716	3.858608
Asah2	-0.899615	2.855965	-3.639915	0.002535	0.020539	-1.918752	3.295864	2.843782	2.548818	3.322471	2.014259	2.323579	3.190506	2.888628	3.079866	2.184529	2.506778	3.009199	3.556237	3.468514
Nhej1	1.605291	0.932371	3.639662	0.002536	0.020539	-1.457662	0.485316	1.310208	1.089503	-0.705589	1.705671	1.378286	0.357848	1.80302	1.102347	1.597576	1.199219	1.315214	0.249639	1.085968
Fanca	1.01815	3.643591	3.63839	0.002543	0.020575	-1.967944	3.073383	4.661326	3.8018	2.890108	4.023054	3.519371	3.639767	3.720315	3.448717	3.968034	3.678847	3.936334	3.195422	3.584586
Slit3	-1.012361	6.266944	-3.638117	0.002544	0.020575	-2.247133	5.802528	4.324616	5.430534	6.609763	5.475469	5.529113	7.067928	6.723033	7.125862	6.574452	6.585651	6.586495	6.981877	6.944573
Ccser1	-1.25743	2.000102	-3.637575	0.002547	0.020584	-1.798944	2.730329	1.425638	2.025285	2.899083	1.364797	1.473956	1.607516	2.539194	2.107651	1.451089	2.616315	1.593679	2.760099	1.824228
Gnal	-0.640427	5.57536	-3.636231	0.002554	0.020626	-2.237109	5.426916	4.721865	5.295814	5.956321	5.172806	5.016334	5.61921	5.883764	5.902631	5.682467	5.776103	5.820864	5.949913	5.864377
Txn1	0.939277	5.299266	3.634113	0.002565	0.020701	-2.201548	5.1936	5.770415	5.763903	4.136703	5.803238	5.02228	5.478169	5.258153	5.473878	5.271138	5.341744	5.090167	5.306793	5.321296
Thoc6	0.697115	4.315703	3.6333	0.002569	0.020721	-2.110643	4.158996	4.499482	4.729737	3.584109	4.718362	4.264517	4.412334	4.481568	4.316905	4.328465	4.404331	4.204378	4.042758	4.35421
Dnph1	1.101852	2.460668	3.632185	0.002575	0.020754	-1.828371	2.620642	3.655486	3.118472	1.658753	3.300313	2.772436	2.033384	2.307359	2.204856	2.239124	2.259075	2.643831	1.675978	2.261799
Tnc	1.273808	4.409239	3.630946	0.002581	0.020792	-2.063101	3.529616	4.184143	5.014403	3.322471	4.794082	4.734242	4.028845	5.465247	3.947156	4.491011	4.284065	5.288978	4.187182	4.538213
Sh3tc1	1.41273	3.138361	3.63039	0.002584	0.020801	-1.857243	2.843156	4.117033	3.295418	1.593908	3.930833	3.038068	2.717819	3.852146	2.870631	3.58107	3.196995	3.827486	2.480628	2.791492
Pctp	1.221313	0.40121	3.629327	0.00259	0.020832	-1.532219	0.592497	1.702379	1.363571	0.166125	1.364797	1.747207	-0.279766	0.377517	-0.773548	0.20947	-0.072212	0.889587	0.037347	-0.397871
Sox8	-0.606021	5.086429	-3.627165	0.002601	0.020909	-2.252323	5.658863	5.023882	4.944066	5.584433	5.244216	4.856883	4.696922	5.048455	4.739818	5.024078	4.882375	5.412558	5.137097	5.003257
Prmt8	-0.99079	5.953827	-3.625399	0.002611	0.02097	-2.281631	6.344085	4.588877	5.621726	6.609079	5.682458	6.033765	5.977681	6.107182	6.049883	6.025651	6.228329	5.959969	6.26068	5.891146
Itgbl1	-1.98492	4.538553	-3.624208	0.002617	0.021001	-2.168905	5.080769	1.339943	3.376367	5.690329	4.402865	4.401612	5.368254	5.025118	4.989416	4.464524	4.540262	3.931333	5.505866	5.528164
Asl	0.77183	4.029269	3.623789	0.002619	0.021001	-2.04118	3.556342	4.236461	4.099597	3.019194	4.165982	3.678453	4.34429	4.198308	4.227195	4.494755	4.107714	4.166573	3.989574	4.225496
Zdhhc2	-0.653928	5.138516	-3.623205	0.002622	0.021001	-2.260447	5.362121	4.96785	4.742881	5.924809	4.886257	5.38426	5.103206	5.077105	5.040349	4.902496	5.109599	5.147358	5.237977	5.098219
Tmem120a	0.702623	5.627321	3.623082	0.002623	0.021001	-2.247517	5.174319	5.96425	5.558705	4.87707	5.695256	5.684684	5.918076	5.777793	5.730483	5.959447	5.480198	5.740228	5.312711	5.941959
Chek1	1.018049	2.555414	3.623014	0.002623	0.021001	-1.882845	2.357438	3.382986	2.744481	2.324175	3.67581	3.62735	2.688847	1.934092	2.295923	2.274406	1.996325	2.100868	2.097585	2.55752
Jph1	-1.38392	3.967026	-3.622014	0.002628	0.021029	-2.122772	4.29216	1.953842	3.772191	4.890633	3.152236	3.879645	3.933266	4.60868	4.263751	4.298786	4.570823	4.056042	4.379868	3.604134
Acan	-1.001072	3.317756	-3.621167	0.002633	0.021051	-1.931355	2.906846	1.679303	2.787402	3.54462	2.16907	2.28453	4.320877	3.897831	4.231815	3.58107	3.461211	3.61312	3.876936	4.283226
Mfi2	-1.501693	2.518931	-3.62054	0.002636	0.021058	-1.825664	2.753611	-0.103798	1.7408	3.075697	1.78444	2.244393	3.159163	3.301815	2.759824	2.694725	2.912	3.118845	3.286436	2.900911
Cuedc2	0.893649	3.523364	3.620333	0.002637	0.021058	-2.059474	3.84352	4.332034	3.838998	2.825673	4.528323	4.120423	3.060831	3.311493	3.169295	3.530893	3.537463	3.346294	2.974345	3.050898
Map3k8	1.147951	2.039981	3.618512	0.002647	0.021122	-1.725164	1.212615	2.914655	2.787402	1.658753	2.702832	1.910004	1.372662	2.454377	1.690848	2.438927	2.401899	2.691938	1.316372	1.415936
Slc12a5	-0.479512	9.672636	-3.616664	0.002657	0.021183	-2.257552	9.312522	8.932712	8.983743	9.538252	9.0175	8.84643	10.29133	9.95007	10.30075	10.07739	9.879789	10.01732	9.972293	10.2989
Ece2	-0.676192	5.514694	-3.616431	0.002658	0.021183	-2.238145	4.975787	4.418186	4.59126	5.317849	4.442875	4.443576	6.328191	5.940998	6.336118	6.140054	5.641494	6.045771	5.998446	6.625503
Tox	-0.877863	3.706643	-3.614785	0.002667	0.021223	-2.222968	4.519693	4.625556	3.918379	5.530587	4.109733	4.020978	3.280644	2.875639	2.973531	3.310669	3.324422	3.269114	3.003904	3.262515
Fetub	5.497007	-3.198202	3.614674	0.002668	0.02123	-1.525271	-3.260591	0.118359	1.472789	-3.260591	1.018831	-1.427823	-3.260591	-3.260591	-3.260591	-0.311823	-2.697527	-3.260591	-3.260591	-3.260591
Cdh20	-0.755479	4.293168	-3.614063	0.002671	0.021231	-2.209375	4.621279	4.625556	4.029817	5.013489	3.913413	3.74229	4.192594	4.038501	4.18493	4.349296	4.250477	4.523011	4.254311	4.44734

Sall2	-0.651556	5.836929	-3.613999	0.002671	0.021231	-2.30122	6.099138	5.082224	5.423748	6.198196	5.50339	5.974681	5.694017	6.093224	5.733744	5.968901	6.088533	6.117979	5.916626	5.850804
Scn4b	-0.830212	5.188475	-3.61244	0.00268	0.021279	-2.243305	5.221003	4.397125	4.860105	5.153196	3.736505	4.422747	5.471805	5.672833	5.456218	5.364141	5.758953	5.903629	5.662352	5.606174
Srgn	0.88499	4.578515	3.61223	0.002681	0.021279	-2.214284	4.686244	5.344885	5.274852	4.001571	4.960226	5.285532	4.306644	4.155962	4.429792	4.630291	3.944596	4.814046	3.91892	4.414502
Rps6ka2	-0.485719	6.182096	-3.610915	0.002688	0.021321	-2.312928	6.290372	5.588845	6.07232	6.237644	5.697802	5.750642	6.298532	6.403725	6.442594	6.311202	6.480122	6.29875	6.389444	6.309524
Psme4	0.783125	7.568139	3.609871	0.002694	0.021352	-2.321587	7.131194	7.921543	7.675917	6.514431	7.469953	7.350774	7.591699	8.165138	7.867725	7.672023	7.777648	8.096943	7.300468	7.427183
Krt81	-1.647049	0.484212	-3.608772	0.0027	0.021369	-1.601861	0.995438	-1.10215	-0.195392	2.061822	0.371962	0.881356	0.68572	1.067371	0.736738	0.20947	0.676952	0.889587	0.491415	0.257494
Cln5	0.747198	2.399077	3.608771	0.0027	0.021369	-1.883054	2.263247	2.97275	2.885483	2.241914	2.962008	3.143674	1.544223	2.419003	1.717448	2.308845	2.468337	2.805622	2.151407	2.011549
Lrrtm3	-0.829958	4.599534	-3.608502	0.002701	0.021369	-2.234116	4.566458	3.696289	4.242177	5.4227	4.162299	4.570746	4.84543	4.424739	4.821787	4.722594	4.486775	4.706916	4.890388	4.901601
Mpp6	-0.599629	5.698303	-3.607527	0.002706	0.021397	-2.320657	6.143878	5.63605	5.588134	6.575185	5.7649	6.056158	5.628776	5.394161	5.508563	5.229048	5.576715	5.325652	5.844936	5.53497
Sertad1	0.736716	2.992367	3.605128	0.00272	0.021463	-1.996848	2.787845	3.361401	3.847136	2.87199	3.711525	3.301961	2.613764	2.505864	2.772565	2.807409	2.949696	2.960934	2.701169	2.900911
P2rx1	1.969941	0.954211	3.605087	0.00272	0.021463	-1.515597	0.485316	2.151237	1.758166	-0.941548	1.578815	0.881356	0.903114	1.564863	0.837186	0.750369	1.375754	1.515634	0.249639	1.193114
P2ry12	-0.893384	4.370196	-3.605019	0.00272	0.021463	-2.195805	4.889087	3.551306	3.359399	4.334907	3.780406	4.178379	3.783728	5.51426	3.963917	5.224545	4.842853	5.475754	4.13915	4.229697
Tnfrsf10b	1.48986	0.284452	3.603701	0.002727	0.021506	-1.503501	0.105685	1.558038	0.945338	-0.705589	0.845972	0.982423	0.118118	0.222004	0.17081	0.799637	-0.072212	0.28321	-0.305233	0.383504
Calcb	2.08089	-0.158194	3.603344	0.002729	0.021508	-1.45757	-1.22354	0.533043	1.089503	-0.705589	1.705671	0.435061	0.563361	-0.26106	-0.632503	0.280604	-0.616402	-0.657145	-0.305233	-0.505482
Zdhhc12	0.807184	3.916521	3.601135	0.002742	0.02159	-2.099511	3.633671	4.259983	4.102999	3.083591	4.424547	3.817169	3.577905	4.161323	3.952765	4.241995	3.78171	4.114573	3.748946	4.037
Filip1	-1.042895	4.586717	-3.600743	0.002744	0.021593	-2.259903	4.827336	3.459476	4.662159	5.606517	3.841551	4.709886	4.182238	4.995405	4.481148	4.913728	4.85532	5.078905	4.391098	4.279179
Myof	1.077372	4.54586	3.598758	0.002755	0.021616	-2.205233	4.361994	5.620991	4.872118	3.633342	4.881785	4.878898	4.652595	4.17729	4.60385	4.821324	3.930525	4.465359	4.052221	4.760648
Tmcc3	-0.698026	6.237134	-3.598514	0.002756	0.021616	-2.342105	6.313077	5.573282	5.954858	6.857195	5.874737	6.14695	6.034921	6.420591	6.115109	6.270281	6.335443	6.645708	6.544158	6.25492
Batf	3.189397	-1.607343	3.598364	0.002757	0.021616	-1.449556	-1.052261	-0.465897	0.604933	-2.731185	1.43972	0.915838	-2.088409	-0.81357	-2.604521	-0.806258	-0.991788	-1.660823	-3.260591	-2.228037
Sept8	-0.450466	8.618743	-3.598292	0.002757	0.021616	-2.310887	8.86624	8.534801	8.2195	8.974771	8.583368	8.53772	8.663355	8.650367	8.614272	8.554552	8.510732	8.648776	8.671501	8.636474
Ptger4	2.994165	-1.018133	3.597904	0.00276	0.021616	-1.447749	-2.235785	0.24948	1.116684	-1.575022	-0.424712	1.648138	-1.450335	-1.720374	-0.929891	-0.665882	-1.142289	-0.657145	-1.413173	-1.406952
F2rl2	2.068571	-0.433371	3.597782	0.00276	0.021616	-1.493502	-0.410969	0.369671	2.053692	-0.81875	1.173177	1.473956	-0.529223	-1.435005	-0.773548	-0.961779	-0.616402	-0.434228	-1.047095	-1.632384
Cdk2	0.888605	3.316479	3.597658	0.002761	0.021616	-2.057629	3.303772	4.364946	3.656563	2.806722	4.018988	3.703341	3.137884	3.377479	2.707706	3.508843	3.141068	3.118845	2.58862	3.160583
Sv2a	-0.502934	8.715887	-3.597619	0.002761	0.021616	-2.325812	8.312637	7.765609	8.001007	8.636209	8.087285	8.03032	9.092542	8.987394	9.206534	9.109319	8.980474	9.111253	9.267872	9.438033
Kcnq5	-1.427293	6.775374	-3.596605	0.002767	0.021627	-2.346036	6.822666	4.719039	6.279687	7.780881	6.443755	6.037684	7.112245	7.229269	7.275459	6.439139	7.193356	6.860231	7.608941	7.069949
Clec4e	4.317466	-2.773116	3.596551	0.002767	0.021627	-1.488187	-3.260591	1.082904	1.966737	-1.388744	1.312595	-0.457681	-3.260591	-3.260591	-3.260591	-2.618976	-2.293564	-2.668207	-3.260591	-3.260591
Pqlc3	1.168826	2.621278	3.596402	0.002768	0.021627	-1.863724	2.460078	2.97275	3.070623	1.430203	3.601614	2.623922	2.100922	2.862532	2.127623	2.819403	3.196995	3.212521	2.060555	2.431883
Ankrd55	-1.164901	3.315525	-3.594723	0.002777	0.021686	-2.010952	3.32724	1.184734	2.225837	3.572936	3.044338	2.685174	3.727855	3.865347	3.799353	4.10172	4.103539	3.772618	3.589113	3.610591
Lhx2	-0.48255	6.816901	-3.59391	0.002782	0.021708	-2.352613	7.665092	6.874387	6.999999	7.471736	7.142362	7.329749	6.606102	6.450463	6.508814	6.53953	6.535402	6.498783	6.380082	6.448478
Rbm46	-1.517105	0.039652	-3.590557	0.002801	0.02183	-1.609448	1.108109	-0.813242	0.158563	1.454757	0.845972	-0.750296	0.118118	0.222004	-0.27702	-0.537962	0.432628	-0.070959	0.491415	-0.204094
Rps8	1.322922	2.751569	3.5905	0.002801	0.02183	-1.788115	1.80948	3.117121	2.578448	1.097434	2.936364	2.336365	3.471351	3.012464	2.905753	3.409193	3.258364	3.220743	2.437952	3.195366
Sigirr	1.259574	1.958474	3.588742	0.002811	0.021893	-1.776925	1.916502	2.812308	2.238266	0.860705	2.874693	2.481552	2.144252	1.628188	1.937064	2.40749	1.652137	1.404538	1.60059	1.889408
Cad	0.859392	5.810547	3.585104	0.002832	0.02203	-2.32621	5.012441	6.342113	6.051342	5.062243	5.652584	5.541588	6.302124	5.822474	6.121347	6.188254	5.637168	5.93395	5.592729	6.116583
Hpx	2.210354	-0.128323	3.585001	0.002832	0.02203	-1.486351	-1.052261	1.010791	0.818644	-1.075778	0.266146	1.708392	-0.279766	0.04768	-0.773548	0.534523	-0.991788	0.081289	0.111637	-0.297732
Nod1	0.861906	3.902792	3.584577	0.002835	0.02203	-2.1644	3.912376	4.820078	4.278735	3.175097	4.251652	4.213454	3.454237	3.972932	3.432752	3.962637	3.765948	4.323687	3.36447	3.820121
Eif3g	0.468465	6.694094	3.584378	0.002836	0.02203	-2.374655	6.750726	7.062818	7.185522	6.504174	7.239051	6.894097	6.483878	6.59119	6.583017	6.620593	6.616008	6.457633	6.387577	6.356384
RT1-A2	1.047763	5.943314	3.58351	0.002841	0.022054	-2.352712	6.018541	6.332901	6.76735	4.91074	6.071135	6.837889	4.7114	6.254932	4.666626	6.736611	6.672053	6.909382	5.170065	5.176474
Sstr3	-0.895523	5.271676	-3.580878	0.002856	0.022143	-2.313241	5.278315	3.52566	4.629506	5.317849	4.705768	4.71234	5.759921	5.63672	6.062907	5.398527	5.600609	5.472315	5.948647	5.800855
Mall	1.614944	1.743753	3.580874	0.002856	0.022143	-1.772435	2.01613	3.42521	2.673036	0.43652	2.411703	2.570814	0.028455	2.287882	0.736738	2.165862	1.652137	2.581351	1.11438	0.887553
Unc13d	1.379479	3.216289	3.579475	0.002864	0.022181	-1.92629	2.342161	3.934181	3.158259	1.760745	3.770765	2.791131	3.36548	3.942114	2.822431	4.005262	3.684447	4.028205	2.393975	3.220915
Pgp	-0.491486	5.756042	-3.579402	0.002865	0.022181	-2.368114	6.078543	5.41815	5.385148	5.885838	5.556259	5.603716	5.768601	5.672833	5.80524	5.74039	5.955889	5.730184	6.132728	5.880498
Polr2d	0.627931	3.489424	3.577511	0.002876	0.022251	-2.171219	3.633671	4.155166	4.072089	3.450082	4.387176	4.040642	3.445603	3.104407	3.100223	3.310669	3.380703	2.910998	2.964355	3.041369
rnf141	-0.694322	4.552577	-3.577211	0.002877	0.022251	-2.278959	4.774252	3.958345	4.302603	4.736442	3.579695	4.392459	4.693279	4.7399	4.69032	4.712982	4.647377	4.872617	5.028628	4.676974
Lrrc7	-0.831143	5.786853	-3.576442	0.002882	0.022271	-2.365946	5.541706	4.835816	5.282373	6.369407	4.8478	5.536057	6.172085	6.259939	6.260125	5.834998	5.973152	5.876565	6.272872	5.983599
Sh3glb1	0.816078	5.895691	3.575605	0.002887	0.022294	-2.383471	5.705873	6.273469	6.527664	6.120711	6.969822	7.148609	5.305224	5.601653	5.262088	5.394523	5.392242	5.93395	5.591107	5.340848
Fgf9	-1.044322	3.100541	-3.574487	0.002893	0.022318	-2.147759	3.582581	2.200694	2.901201	4.291262	2.95351	3.541623	3.383673	2.781287	3.350165	2.628098	3.265855	2.643831	3.229186	2.847239
Gprasp1	-0.771043	8.817887	-3.574441	0.002894	0.022318	-2.354827	8.599762	8.199859	8.271824	9.691876	8.452103	8.559352	9.095477	8.83846	9.138342	8.834352	8.696288	8.70352	9.126097	9.246731
Mpped2	-1.102363	5.005264	-3.573198	0.002901	0.02236	-2.344662	5.264199	3.643613	4.850017	5.95417	4.445908	5.065155	4.943741	5.465247	5.099692	5.000567	5.464008	5.053812	5.202297	4.673894
Gucy1a3	-1.060137	3.424816	-3.571778	0.002909	0.022396	-2.130809	3.92781	3.49955	2.845423	3.686144	1.912714	2.800388	3.987806	3.798099	3.613573	3.456042	3.352837	3.668268	4.042758	3.517445
Cdon	-0.685522	4.601766	-3.571379	0.002911	0.022396	-2.260899	4.239536	3.7962	4.029817	4.746422	3.464807	3.964448	5.138455	5.13548	5.253025	4.894014	4.79964	4.980656	4.887747	5.165551
Tor1b	0.56206	4.854454	3.570992	0.002914	0.022396	-2.283008	4.399379	5.044346	4.872118	4.14804	4.786925	4.623856	5.069886	4.97728	4.849109	5.150519	4.896921	5.251348	4.987296	4.960925
Sh3gl3	-0.481941	6.047721	-3.570868	0.002914	0.022396	-2.396951	6.293354	5.851358	5.74443	6.561118	6.204233	5.985882	6.060593	5.849298	6.153361	5.984964	5.934897	5.873961	6.071227	6.123372
Rilpl1	-0.638933	6.181426	-3.570818	0.002915	0.022396	-2.399964	6.481158	5.573282	6.227653	6.906426	6.14296	6.274495	6.014634	6.193454	6.231573	6.071503	6.094855	6.167734	6.097743	6.084468
Rasgrp3	-1.0167	4.901627	-3.569963	0.00292	0.022412	-2.322012	5.313991	4.010125	3.420663	5.082081	4.886257	4.440619	5.006725	5.394161	4.778229	5.453434	5.13845	5.758697	5.006894	4.990935
Kcnh5	-1.773472	0.394914	-3.569819	0.002921	0.022412	-1.666076	0.830061	0.895377	-0.007587	2.154676	-0.718553	-0.646023	0.357848	1.318383	0.317129	0.134646	0.263333	0.218993	1.1849	0.554014
Prepl	-0.775669	7.853568	-3.569136	0.002925	0.022415	-2.390369	7.918828	7.101306	7.620915	8.608777	7.44857	7.778423	7.943293	8.01996	7.980124	7.608208	7.999147	7.753072	8.304452	7.871876
Dpagt1	0.669486	6.226495	3.569003	0.002925	0.022415	-2.389136	5.820555	6.621732	6.348659	5.530587	6.409993	5.991955	6.27801	6.395786	6.408308	6.507455	6.145478	6.357722	5.92952	6.446674
Pstpip2	0.735607	2.224913	3.568808	0.002927	0.022415	-2.019287	2.445855	3.33211	3.404211	2.666599	2.95351	3.462191	1.726334	1.155984	2.024877	1.540753	1.375754	1.805289	1.943396	1.684306
Bace1	-0.542652	6.248389	-3.567783	0.002933	0.022447	-2.397027	6.150471	5.494499	5.849432	6.211766	5.668248	5.540207	6.466912	6.70754	6.517355	6.303751	6.515901	6.644945	6.849743	6.578514
RGD1563441	-0.897581	4.018891	-3.566645	0.002939	0.022485	-2.333219	4.813555	4.125594	4.331892	5.567645	3.89578	4.841271	3.616879	3.668539	3.742206	3.319142	3.765948	3.509549	3.949625	3.220915
Vac14	0.546196	5.483937	3.565899	0.002944	0.022504	-2.383798	5.476734	5.771779	6.057492	5.372033	6.162446	5.881799	4.874668	5.465247	5.13958	5.215497	5.486624	5.435699	5.380011	5.09131
Adamts19	-1.631471	1.038269	-3.564712	0.002951	0.022544	-1.643438	0.830061	-0.687936	-0.33572	1.593908	-0.18071	-0.006501	2.415815	1.747064	2.024877	1.898256	1.430105	1.082389	1.316372	2.402413
Gal3st3	-0.948995	7.014668	-3.561945	0.002968	0.022656	-2.416961	7.072808	5.057828	6.033624	6.564648	6.484138	5.894822	7.641218	7.517025	7.799333	7.552709	7.49171	7.535924	7.752265	7.822316
Adcyap1r1	-0.604143	8.201023	-3.560447	0.002977	0.02271	-2.405738	8.322401	8.032687	7.712567	8.288312	7.68058	7.377308	8.474416	8.253139	8.319064	8.402394	8.272626	8.599577	8.399726	8.685087
Ly6c	4.034837	-3.013804	3.560091	0.002979	0.022712	-1.571219	-1.642648	-0.572649	1.472789	-3.260591	0.691028	-0.371994	-3.260591	-3.260591	-3.260591	-3.260591	-2.293564	-3.260591	-2.656794	-2.653844
Kcnma1	-1.078646	6.244835	-3.55938	0.002983	0.02273	-2.416897	6.166823	4.974974	5.858518	7.080717	5.308931	6.029835	6.664934	6.586216	6.647551	5.8453	6.445083	6.351184	7.025766	6.464608
Gabra1	-0.888385	8.014473	-3.557997	0.002991	0.02277	-2.406035	8.030267	7.298757	7.626245	9.093385	7.622132	8.135789	7.960819	8.052787	8.027731	7.941946	7.909331	8.071313	8.13056	8.307851
Trim65	0.992163	3.409317	3.5578	0.002992	0.02277	-2.068692	2.917193	4.000847	3.945162	2.389316	3.384903	3.172479	3.231264	3.606999	3.525993	3.802672	3.380703	3.575147	3.278395	3.673625
Rnf180	-0.547306	4.677526	-3.557574	0.002994	0.02277	-2.381003	5.371558	4.804167	4.648255	5.462596	4.921539	5.116089	4.510807	4.438054	4.290573	4.285878	4.477127	4.212646	4.713828	4.295302
Fgf2	1.246603	1.671579	3.555943	0.003004	0.02283	-1.854003	1.669604	2.643736	2.984714	1.245516	2.014259	2.998397	0.851758	1.318383	1.29127	1.322149	1.102128	1.643459	1.837926	1.009844
Abcb1b	-1.662717	3.47251	-3.55541	0.003007	0.02283	-2.091416	3.00712	0.480615	2.578448	4.427757	2.507694	2.685174	4.085519	4.224157	4.429792	3.968034	3.872828	4.149447	4.080242	4.330935
Ndrg4	-0.49202	10.62832	-3.555339	0.003007	0.02283	-2.338382	10.53164	9.949459	10.24591	10.75103	10.31373	10.08833	10.95934	10.77819	10.99824	10.70596	10.92876	10.67028	10.96769	10.90889
Magi2	-0.781116	6.65986	-3.554172	0.003014	0.022869	-2.435674	6.802575	6.11804	6.377037	7.452814	6.098369	6.791638	6.625343	6.904237	6.868369	6.400738	6.728715	6.528065	6.942983	6.615104
Gabbr1	-0.485011	10.54872	-3.553436	0.003019	0.022889	-2.350451	10.45132	9.785916	9.796792	10.36218	10.07657	10.02983	10.92161	10.79068	10.91795	10.90203	10.69076	10.99109	10.89749	11.06901
Cd200r1	2.507931	-0.381784	3.55217	0.003027	0.022933	-1.536524	-1.908947	0.426209	1.245402	-0.600663	1.415175	0.881356	-1.73438	-0.048075	-1.813089	-0.665882	0.484915	1.219993	-1.218554	-0.397871
Rap2a	-1.294481	0.73543	-3.550319	0.003038	0.023005	-1.800705	1.693879	-0.273517	0.715742	2.108996	0.952152	1.193889	-0.067151	0.871929	0.97585	0.534523	0.846404	0.34469	1.077784	0.321874
Rpl28	0.831029	5.661874	3.549112	0.003045	0.023046	-2.413636	5.72955	6.492001	6.075786	4.890633	6.326904	5.641552	5.521948	5.589771	5.481658	5.687384	5.500978	5.508024	5.252416	5.599697
Marveld2	-0.916244	1.917146	-3.547907	0.003053	0.023088	-1.960317	2.387517	1.082904	2.10888	2.758225	1.578815	1.875381	1.754571	1.856886	2.241976	1.920699	1.996325	2.100868	1.651284	1.951771
Slc27a1	-0.717209	6.986318	-3.546715	0.00306	0.023129	-2.450739	7.373532	6.405005	6.251607	6.628096	6.487821	5.993973	7.363801	7.034121	7.310827	7.328728	7.284681	7.457646	7.248422	7.653561
Itgb1bp1	0.646883	4.877137	3.545915	0.003065	0.023152	-2.336478	4.589284	4.996138	4.811028	3.980586	5.056608	4.823218	5.154438	4.971187	5.035072	5.164691	5.09285	4.755828	4.803407	5.100515
Slc44a2	0.652569	7.099047	3.545264	0.003069	0.023168	-2.452164	6.618271	7.4307	7.082015	6.371021	7.156776	6.91449	7.184724	7.38862	7.107752	7.444093	7.101101	7.586508	6.727349	7.28505
Tmem114	-1.884258	0.616424	-3.543081	0.003083	0.023256	-1.610757	0.785575	-0.813242	-0.974047	0.621404	-2.049163	-0.006501	0.903114	1.596873	1.579251	1.625169	1.375754	1.667719	2.003164	1.951771
Gpr114	2.990646	-0.486556	3.542342	0.003087	0.023265	-1.548789	-1.22354	0.185409	1.061799	-2.731185	-0.107758	0.231108	-0.672244	0.137473	-0.504027	1.064403	0.764165	1.515634	-1.413173	-1.899685
Ablim3	-0.889259	4.884893	-3.542256	0.003088	0.023265	-2.401807	4.96336	4.267739	4.905621	5.89147	4.162299	4.827752	4.912754	5.022174	5.159118	5.011064	4.763054	4.898474	4.665321	4.993408
Atp1a2	-0.751937	10.90818	-3.540546	0.003099	0.023331	-2.351712	11.46061	10.79013	10.2263	10.90222	10.36198	10.34162	11.322	10.70154	11.04743	11.00404	10.79455	11.23308	11.05967	11.47021
Hsd3b7	0.730955	3.8967	3.53762	0.003117	0.023455	-2.218956	3.439172	4.456016	3.978882	3.211576	3.990197	3.789543	3.857182	4.067347	4.029076	4.182876	3.972332	3.911153	3.646466	4.129836
Foxs1	1.528542	1.241158	3.536223	0.003126	0.023497	-1.707931	0.873216	1.725091	1.921212	-0.243853	2.046579	1.275818	1.21902	0.923327	0.97585	1.755735	1.456531	1.667719	0.791767	1.708589
Ankrd34a	-0.766615	6.414537	-3.535434	0.003131	0.023497	-2.47335	6.860689	5.724656	6.345072	7.235318	6.499542	6.554406	6.134975	6.551926	6.365849	6.243824	6.64789	6.219936	6.395033	6.043293
Chst1	-0.695371	8.359614	-3.535074	0.003133	0.023497	-2.456842	8.262797	7.04092	7.694359	8.395452	7.912535	7.891841	8.577544	8.755587	8.784916	8.707995	8.715905	8.747753	8.844516	8.707664
Rgs16	1.076041	2.41558	3.534832	0.003135	0.023497	-2.076989	2.230432	3.108509	4.096187	2.634716	3.601614	3.165332	1.478026	2.030999	1.360416	1.828755	2.181985	2.300247	2.133689	1.991897
Aoah	2.940041	-1.293884	3.53483	0.003135	0.023497	-1.560856	-0.899176	1.506527	1.758166	-2.040259	1.05105	0.056669	-1.450335	-0.992527	-3.260591	-1.136114	-0.149817	0.151783	-3.260591	-2.653844
Gpx1	1.082961	5.737299	3.534574	0.003136	0.023497	-2.451134	6.275369	6.458507	6.568841	4.646043	6.747015	6.344989	5.092185	5.587782	4.959085	5.737232	6.210001	5.757285	4.916528	5.05389
Calb2	-0.80202	3.308823	-3.53416	0.003139	0.023497	-2.195663	3.271877	3.142653	2.812554	3.980586	2.762423	2.695134	3.593621	3.466799	3.271566	3.545408	3.387587	3.065063	3.689548	3.803307
Pik3c2b	-0.815269	5.573983	-3.533971	0.00314	0.023497	-2.428915	5.216821	5.337533	4.340564	5.850418	4.693063	4.562608	6.095506	6.057725	6.198908	5.918216	5.645806	5.890805	6.056021	6.208847
Tmem198b	0.856616	3.343633	3.533963	0.00314	0.023497	-2.127716	3.154337	3.817493	3.609501	2.337441	3.731543	3.158148	3.392685	3.301815	3.307029	3.559779	3.421519	3.509549	3.186856	3.482664
Nuak1	-0.93885	4.793839	-3.532655	0.003148	0.023544	-2.418286	5.024455	3.790827	4.716472	5.810545	4.511054	4.889781	4.39898	4.974236	4.830952	4.657271	4.875046	5.090167	4.839377	4.763548
Mmp14	1.72043	4.685776	3.531591	0.003155	0.02358	-2.368524	5.350716	5.265764	6.521957	2.994284	5.739176	5.849832	3.471351	4.498613	3.367065	4.336834	5.006058	5.229104	4.009141	4.037
Pou2f2	0.617568	2.877275	3.529479	0.003169	0.023666	-2.09262	2.607924	3.224637	3.177748	2.511387	3.00376	3.268997	2.534559	3.19084	2.597457	3.090803	2.912	3.25317	2.452318	2.673087
Ccr1	3.988272	-1.582821	3.528112	0.003178	0.023717	-1.57338	-0.634519	1.558038	3.560852	-2.344749	1.556544	1.193889	-3.260591	-2.550306	-3.260591	-2.176425	-2.293564	-1.660823	-1.904936	-1.899685
Pxn	0.86407	7.039956	3.527555	0.003181	0.023729	-2.48668	6.45121	7.804785	7.550713	6.478958	6.990736	6.966818	7.301169	6.968348	7.223628	7.443124	6.802035	6.766462	6.777417	7.046326
Cdadc1	-0.452857	4.567632	-3.527147	0.003184	0.023734	-2.401738	4.710243	4.486247	4.323168	5.029924	4.380852	4.504319	4.547582	4.50706	4.635579	4.528025	4.50904	4.575244	4.734547	4.541596
Agpat3	-0.392388	8.740329	-3.526667	0.003187	0.023742	-2.457789	8.830762	8.454252	8.374799	8.796686	8.385952	8.470444	8.873073	8.745136	8.82694	8.923696	8.83368	8.912414	8.943496	8.997003
Slc25a45	1.523677	1.160303	3.525582	0.003194	0.02378	-1.670478	0.592497	1.702379	1.033553	-0.81875	1.338932	1.07687	1.047044	2.121805	0.683763	1.898256	2.317912	1.910689	0.879431	1.29285
Pip4k2c	-0.530553	6.147546	-3.525025	0.003197	0.023785	-2.486947	6.493311	5.870585	5.873535	6.173444	5.848624	5.619493	6.120131	6.371704	6.311229	6.207777	6.492945	6.162408	6.455119	6.087944
Fabp3	-0.55883	4.36589	-3.5247	0.0032	0.023785	-2.423427	5.20842	4.582672	4.655224	5.0052	4.478849	4.472821	4.123906	3.871902	4.508062	4.201325	4.176893	3.698019	4.156802	4.060775
Nrxn2	-0.492202	8.972742	-3.524548	0.003201	0.023785	-2.455817	9.011084	8.292588	8.694277	9.071844	8.542167	8.672385	9.142145	9.09655	9.250173	9.130383	8.930535	9.259284	9.194592	9.333585
Cyp4f1	-1.512705	2.736776	-3.523136	0.00321	0.023838	-2.028373	3.25566	2.36878	1.220561	2.634716	2.308867	0.056669	3.36548	3.395791	3.23521	3.48645	3.108108	3.323571	3.372046	3.517445
Lynx1	-0.437402	8.786061	-3.521512	0.00322	0.023902	-2.46496	9.033995	8.417901	8.506027	8.824922	8.621918	8.426839	8.978959	8.641283	8.9449	8.823785	8.718125	8.84653	9.150807	9.072474
Rhoa	0.512786	7.408317	3.520618	0.003226	0.023931	-2.494708	7.612738	7.922771	7.927121	7.436725	8.068963	8.23236	6.880457	7.375416	7.028972	7.001649	7.070416	7.271321	7.0955	6.801622
Kcnk3	-0.990823	1.820449	-3.51997	0.00323	0.023947	-2.002331	2.196854	1.279848	1.574317	2.853641	2.139408	1.275818	1.782265	1.198329	2.127623	1.540753	1.674823	2.00891	2.151407	2.142093
Snai1	1.45371	2.212601	3.519386	0.003234	0.023961	-1.967635	1.957182	3.588941	3.580509	1.327578	2.519256	2.537984	2.01015	1.958936	1.914249	2.221154	1.94125	2.064785	1.347449	2.372328
Sgsm2	-0.533179	7.956729	-3.518801	0.003238	0.023965	-2.491208	8.226856	7.369844	7.700267	8.206632	7.871402	7.796463	8.186476	7.830189	8.174752	8.10424	7.717117	7.836027	8.078257	8.302128
Slamf9	6.092776	-0.283562	3.518482	0.00324	0.023965	-1.625874	-1.642648	0.812964	2.175023	-3.260591	3.326873	2.845805	-1.73438	0.871929	-2.155202	0.20947	0.321999	2.330954	-1.413173	-1.899685
Itk	2.227203	-0.098825	3.518258	0.003241	0.023965	-1.620059	0.485316	1.248835	1.472789	-2.040259	0.470544	1.193889	0.028455	0.04768	-0.27702	-0.116374	-0.072212	-0.657145	-0.512812	-0.621773
Snx13	-0.740156	5.649015	-3.51786	0.003244	0.023965	-2.495844	5.900953	5.173818	5.621726	6.566761	5.393031	5.786023	5.517835	5.623184	5.487466	5.251354	5.57822	5.641098	5.973761	5.602939
Sort1	-0.716705	10.11171	-3.51777	0.003245	0.023965	-2.434155	9.96835	8.915844	9.48397	10.31461	9.544354	9.757779	10.30623	10.48745	10.36519	10.26626	10.41537	10.59948	10.66261	10.47799
Enpp6	-1.284852	2.540159	-3.516612	0.003252	0.024007	-1.99643	2.515609	1.914859	0.307539	2.645422	2.254562	1.014597	2.613764	3.660989	2.870631	3.440594	3.467722	3.60686	2.736815	2.868948
RGD1306739	-0.604167	3.920722	-3.516085	0.003256	0.024011	-2.385324	4.748409	4.150978	3.772191	4.390058	4.070978	3.879645	3.454237	4.026799	3.682701	3.994723	3.50618	3.843548	3.783444	3.692011
Cct4	0.541167	6.946115	3.515907	0.003257	0.024011	-2.51237	6.584586	6.859708	7.186324	6.814549	7.481821	7.436397	6.896384	6.806675	6.891915	6.829978	6.837029	6.757307	6.953752	6.922034
Hivep2	-0.725529	8.173769	-3.515184	0.003262	0.024032	-2.494403	8.14173	7.330116	8.029444	8.726577	7.466225	8.00777	8.503304	8.42402	8.374961	8.161014	8.186935	8.253907	8.494096	8.338324
Isg20	1.397947	1.249729	3.513049	0.003276	0.024086	-1.781463	0.369528	2.248512	2.200654	0.664146	1.840817	1.221719	1.297885	1.564863	0.97585	1.419918	0.764165	1.515634	0.879431	1.227132
Anxa5	0.619308	6.676334	3.512576	0.003279	0.024086	-2.518557	7.010752	7.290215	7.151838	6.652846	7.60494	7.75451	6.136317	6.383222	6.265768	6.393736	6.056497	6.509728	6.103443	6.171118
Clstn1	-0.580161	10.50969	-3.512212	0.003281	0.024086	-2.438574	10.12154	9.309944	9.837504	10.45506	9.824057	9.863068	10.99542	10.92184	11.0753	10.90968	10.79637	10.93586	10.93474	11.15639
Aox1	-1.005278	4.855933	-3.512202	0.003281	0.024086	-2.37912	4.835542	4.40418	3.336461	4.433945	3.440689	3.408717	5.662696	5.742456	5.299983	5.809668	5.285734	5.715713	4.992221	5.683248
Kcnip4	-1.227228	4.547972	-3.512094	0.003282	0.024086	-2.44491	4.915133	3.24835	4.179109	5.866522	4.424547	4.795709	4.33497	4.757708	4.639062	4.145252	4.81504	4.233112	4.982355	4.407101
RT1-T24-1	2.251619	-0.322787	3.511407	0.003287	0.024086	-1.601734	-2.235785	-0.572649	-0.33572	-1.788967	-0.18071	0.386712	0.429655	0.76329	0.245823	0.699359	0.631286	1.186803	-0.628995	-0.621773

Fancb	0.999424	1.983813	3.511393	0.003287	0.024086	-1.934088	1.568201	2.532247	2.262807	1.353927	2.564599	2.398644	2.433388	1.628188	1.690848	2.256873	1.844511	1.826994	1.574559	2.245294
Gpr165	-1.067144	1.535295	-3.511343	0.003287	0.024086	-1.964974	1.936985	0.895377	1.472789	2.613062	1.113405	1.473956	1.544223	0.818632	1.769222	1.355481	0.961591	1.375373	2.333172	2.402413
Pprc1	0.49814	5.808031	3.511339	0.003287	0.024086	-2.503367	5.546713	5.96425	6.452317	5.752308	6.124153	6.049379	5.588166	5.812285	5.648188	5.690653	5.590204	5.790806	5.715431	5.615836
Klhdc1	-0.751989	2.768672	-3.509595	0.003299	0.024146	-2.142756	2.765113	2.425352	2.010869	3.335752	2.268331	2.613455	3.328389	2.888628	3.100223	2.819403	2.673757	2.568523	3.042398	3.160583
Ccdc103	5.736476	1.563267	3.50949	0.003299	0.024146	-1.635536	-1.052261	5.214516	5.693438	-1.223791	3.333437	3.045873	0.118118	1.658838	0.17081	1.388059	2.100543	1.989793	0.434663	0.119416
Gnai2	0.557338	8.857371	3.509218	0.003301	0.024146	-2.483625	9.058741	9.388299	9.170124	8.490922	9.524143	9.251224	8.315576	8.856339	8.421713	8.808071	8.734078	8.964139	8.516914	8.506386
Fgd3	0.710096	3.715872	3.507205	0.003315	0.024229	-2.263876	3.474632	4.175923	3.8018	3.002635	3.917788	3.857641	3.241276	4.15058	3.169295	4.026112	3.877725	4.357637	3.495104	3.597647
Fam214b	0.526763	5.868995	3.503252	0.003341	0.024409	-2.514206	5.668082	6.298294	6.130151	5.372033	5.95568	5.794155	5.985148	5.933183	5.695787	5.967554	5.848982	5.913807	5.755083	5.875144
Usp31	-0.758539	5.874893	-3.502277	0.003348	0.024442	-2.526578	6.017339	5.082224	5.573494	6.431808	5.366073	5.838643	6.111969	5.967255	5.939818	5.606633	5.969716	5.890805	6.547508	5.932969
Rpl7	0.678292	7.054654	3.500062	0.003363	0.024527	-2.543525	6.910091	7.614139	7.032921	6.491621	7.653948	7.208076	7.081924	7.082261	6.96866	7.248941	7.000297	6.930214	6.612114	6.942015
Stard4	-0.734673	3.18752	-3.499959	0.003364	0.024527	-2.297086	3.765497	3.08236	3.12518	3.829173	2.901447	3.165332	3.479833	2.901501	3.416608	2.375352	3.189137	2.656009	3.828196	3.088394
Robo2	-0.879465	7.236542	-3.49931	0.003368	0.024545	-2.5429	7.14996	6.315233	6.866257	8.066036	6.496621	7.228366	7.345933	7.549591	7.456114	7.167417	7.261918	7.329978	7.637904	7.451218
Phf19	0.995584	2.28001	3.495902	0.003391	0.024699	-2.019865	2.10932	2.595246	2.548818	1.405223	3.210452	2.515676	2.247205	1.856886	2.462438	2.499819	2.166061	2.203999	2.236876	2.194616
Cenpt	2.389347	1.172314	3.495227	0.003396	0.024712	-1.738101	0.485316	2.134368	0.945338	-0.81875	3.00376	2.28453	1.000645	1.658838	1.218642	1.180545	0.885831	1.315214	0.836265	1.122575
Kcng4	-1.181479	4.564359	-3.495047	0.003397	0.024712	-2.442619	5.276307	4.064569	2.908996	4.680285	4.608991	3.673423	5.083863	4.58094	4.618038	4.850841	4.804791	5.285063	4.615127	4.925106
Zmynd8	-0.50938	7.684329	-3.494191	0.003403	0.02473	-2.549985	7.602356	7.487433	7.224697	7.940986	7.289296	7.043864	8.088665	7.858522	8.063487	7.825977	7.76647	7.675561	7.821342	7.899826
Bax	0.612843	4.820465	3.494093	0.003404	0.02473	-2.457528	4.751303	5.159298	5.039476	4.1518	5.095746	4.92618	4.778253	4.820056	4.887683	5.079985	4.72823	4.811327	4.484924	4.828697
Srsf2	0.501779	8.053062	3.492963	0.003412	0.024769	-2.54416	7.711904	8.331268	8.308385	7.913015	8.419117	8.195118	8.206417	7.882662	8.087903	8.098099	7.812272	7.868441	7.76311	8.151119
Cckbr	-0.712017	3.791374	-3.492642	0.003414	0.024769	-2.411902	4.674093	4.037603	3.706618	4.228565	3.446756	3.775529	3.496648	3.42283	3.472337	3.734632	3.621611	3.489274	4.009141	4.079517
Dpp10	-0.665859	4.50429	-3.492404	0.003415	0.024769	-2.474469	4.824591	4.313419	4.385956	5.160679	4.306064	4.320284	4.551611	4.279444	4.530738	3.913127	4.065415	4.179285	5.096513	5.204492
Birc2	0.613955	5.062269	3.492115	0.003417	0.024769	-2.500255	4.813555	5.263826	5.822858	4.965167	5.235481	5.68093	4.788531	4.980316	4.670035	5.003199	4.944375	4.906142	5.087338	4.757741
Ncam1	-0.598164	8.331948	-3.490305	0.00343	0.024834	-2.543267	8.197709	7.739352	7.99667	8.691493	7.708939	7.937135	8.377314	8.590019	8.669598	8.38131	8.239243	8.636344	8.780663	8.706534
Slc17a9	1.278528	1.885468	3.490231	0.00343	0.024834	-1.906201	1.310059	2.801663	2.262807	0.74601	2.436306	1.585258	1.544223	2.364261	1.29127	2.359011	2.274011	2.619163	1.436856	1.824228
Nicn1	-0.417668	6.607344	-3.488968	0.003439	0.024882	-2.567687	6.885931	6.401484	6.321172	6.906426	6.592776	6.600181	6.74628	6.544764	6.71263	6.533172	6.639985	6.468031	6.55834	6.607859
Cpd	-0.881804	6.820677	-3.48856	0.003442	0.024888	-2.567002	6.751449	5.622504	6.347942	7.32254	6.161524	6.485772	7.026554	7.236907	7.106494	6.575336	6.97138	7.271321	7.525204	7.099518
Rufy1	0.473928	5.837565	3.486844	0.003454	0.024959	-2.552341	5.672669	6.238375	5.842326	5.618792	6.29699	6.099927	5.731791	5.832592	5.618503	5.907019	5.703489	5.913807	5.589483	5.687836
Nr4a3	-2.07973	5.71521	-3.486333	0.003458	0.02497	-2.547266	6.157034	3.08236	4.821391	7.613088	5.237232	6.034746	5.905539	6.043277	5.690761	5.052302	6.040209	5.846329	6.768116	5.760774
Slc26a7	-1.867807	-0.313237	-3.485418	0.003464	0.025001	-1.762807	1.48718	-1.10215	-1.223881	0.705659	-0.33871	0.117189	-0.169545	-0.81357	-0.773548	0.055728	0.321999	-1.243945	-0.211665	0.970221
Cdip1	-0.76361	8.157124	-3.484663	0.003469	0.025006	-2.554234	8.469825	7.10564	7.924239	8.536209	8.039156	7.895356	8.120542	8.275871	8.281952	8.186416	8.534378	8.192789	8.499726	8.143346
MGC94207	0.633805	5.059514	3.484507	0.00347	0.025006	-2.511577	5.09451	5.741464	5.421025	4.572219	5.456049	5.230091	4.567614	4.97728	4.659784	4.976667	5.054439	5.140873	5.035801	4.953324
Fibin	-1.240868	2.391968	-3.484438	0.003471	0.025006	-2.180292	2.906846	0.895377	2.47722	3.533136	2.60856	1.943815	2.415815	2.268138	3.006262	1.780488	1.844511	1.848377	3.178239	3.11589
Mars	0.429032	7.343294	3.483021	0.003481	0.025063	-2.57855	6.988232	7.482859	7.49839	6.951877	7.315643	7.298647	7.611614	7.31656	7.546978	7.428516	7.232113	7.249888	7.31479	7.579785
C2	1.202872	4.828157	3.480073	0.003502	0.025197	-2.368727	3.968177	4.835816	4.59126	2.951788	4.728358	4.389395	4.390007	6.506306	4.111616	6.218007	5.736214	6.854958	3.828196	4.555051
Tep1	0.91048	5.318119	3.479548	0.003505	0.025207	-2.49274	4.960861	5.774504	5.050089	3.950685	5.273527	5.316512	5.241808	5.94877	5.154258	5.865686	5.590204	6.232189	4.937104	5.200217
Jmjd6	0.55472	4.428062	3.479298	0.003507	0.025207	-2.458881	4.280186	4.785379	4.766671	4.129095	5.024515	4.446528	4.510807	4.24955	4.263751	4.357545	4.334806	4.342647	4.216936	4.358053
Numb	-0.432775	4.768676	-3.477061	0.003523	0.025306	-2.522711	5.080769	4.479583	4.636566	5.119037	4.733331	4.81867	4.818933	4.81326	4.71363	4.66726	4.757751	4.838295	4.659142	4.683115
Mybl2	2.058109	1.768864	3.476651	0.003526	0.025312	-1.980223	1.341131	3.570246	2.010869	1.000693	3.926497	3.334188	1.297885	0.705741	1.520039	2.088679	1.375754	1.461155	0.879431	0.799858
LOC500956	0.783654	6.760774	3.475361	0.003535	0.025363	-2.59232	6.459198	7.768345	7.460436	6.59465	7.108796	6.901644	6.80568	6.647676	6.671557	6.73186	6.358399	6.377163	6.149356	6.631072
Phf21b	-0.73477	3.173028	-3.470907	0.003567	0.025577	-2.350777	3.582581	3.046741	3.301354	3.959292	2.586747	3.242073	2.841442	3.292072	3.341641	3.258764	3.065821	3.083214	2.794335	3.203933
Svip	-0.417558	5.078051	-3.468283	0.003586	0.025698	-2.579786	5.681801	5.184101	5.206917	5.686446	5.349976	5.32776	4.815587	4.616508	4.821787	4.797268	4.79964	4.956243	4.999576	4.896325
Galnt18	-0.704132	6.127687	-3.467751	0.00359	0.02571	-2.582057	5.847855	4.63158	4.93264	5.823566	5.421037	5.536057	6.694385	6.757959	6.611662	6.863671	6.553112	6.932093	6.574027	6.634245
Utp20	0.561143	5.548172	3.467242	0.003594	0.025721	-2.558268	4.907368	5.779937	5.597812	5.115191	5.351594	5.567575	6.016092	5.449994	5.823721	5.506329	5.575209	5.600717	5.486802	5.930391
Bcl11a	-0.87543	6.277966	-3.466601	0.003598	0.025739	-2.611203	6.696193	5.587296	6.158221	7.390142	6.336739	6.586199	5.944361	6.384369	6.201266	5.976955	6.373213	6.060734	6.349707	5.867076
Enc1	-1.396761	9.655004	-3.466021	0.003603	0.025754	-2.545471	9.752936	7.538016	8.934947	10.52412	8.966548	9.534053	9.878937	10.11812	9.872614	9.814847	10.23273	9.989626	10.3318	9.683087
Tex30	0.703112	2.3245	3.464872	0.003611	0.025786	-2.143792	1.916502	2.914655	2.700244	2.350586	2.910256	2.845805	2.613764	1.983359	2.295923	2.108365	1.996325	1.950783	2.151407	2.124152
Kcnc3	-0.906303	8.696218	-3.464618	0.003613	0.025786	-2.577344	8.668746	7.626256	8.254911	9.591817	8.362875	8.644866	8.581244	8.985506	8.910369	8.710613	8.739973	8.853322	8.897318	8.923212
Osgin1	1.63513	1.515384	3.464487	0.003614	0.025786	-1.886462	1.48718	1.679303	2.778919	-0.325128	1.997824	1.839907	1.938115	1.429209	1.489496	1.419918	1.533025	1.691577	1.316372	1.556352
Sptbn2	-0.542849	10.57318	-3.464242	0.003616	0.025786	-2.524794	10.7136	10.01479	10.04623	10.4047	9.72776	10.28313	11.10173	10.77327	10.85134	10.76568	10.6428	10.8171	10.92294	10.96055
Coro1b	0.39661	6.910099	3.461704	0.003634	0.025903	-2.621873	6.755779	7.172754	6.917391	6.577283	7.142362	7.017314	6.77068	7.02753	6.817021	7.000717	6.992981	7.080087	6.680829	6.800211
Fabp4	2.39612	0.121084	3.45929	0.003652	0.025999	-1.786738	0.031429	2.702143	1.65069	-0.705589	2.651221	0.772673	-1.21312	0.705741	-0.632503	0.348395	0.071497	0.34469	-1.638202	-1.406952
Stxbp6	-1.192704	4.820044	-3.458749	0.003656	0.025999	-2.591498	5.421475	4.03306	5.030571	6.44259	4.342316	5.538825	4.267989	4.876565	4.723506	4.520698	4.865217	4.568818	4.734547	4.174106
Pbld1	-1.145551	2.562841	-3.458722	0.003656	0.025999	-2.193083	3.197423	2.232748	1.534559	2.728325	2.361201	1.249022	2.854546	2.571773	2.41447	2.694725	2.81321	3.195935	2.871195	3.454224
Inmt	3.453972	-1.306246	3.458704	0.003656	0.025999	-1.695698	0.031429	0.632508	1.687412	-2.731185	1.765147	1.627481	-2.558588	-1.435005	-3.260591	-1.136114	0.321999	-2.668207	-2.232431	-1.632384
Enpp2	-2.641061	7.856764	-3.456659	0.003671	0.026091	-2.594165	11.16842	6.946227	6.675511	7.660039	7.019516	6.513478	7.032332	7.509687	7.282714	9.03106	10.08335	7.6396	7.641435	7.800242
Spata9	-1.107082	1.359945	-3.455767	0.003678	0.026123	-2.030014	1.717752	0.480615	0.679743	2.389316	1.364797	1.450626	1.258992	1.68885	1.426399	1.388059	1.533025	1.252437	1.922909	1.122575
Arhgap5	-1.019387	5.363651	-3.455438	0.00368	0.026125	-2.618594	6.05648	4.871884	5.547512	6.587728	5.058591	5.725349	5.219191	4.998404	4.849109	4.696819	5.311298	5.202367	5.991085	5.015475
Mpdz	-0.806939	7.636438	-3.453699	0.003694	0.026193	-2.631046	7.358899	7.08329	6.869257	8.35036	6.887461	7.341687	8.373052	7.856457	8.153601	7.755156	7.509173	7.539215	7.843652	7.997297
RGD1309492	-0.438372	6.762928	-3.453559	0.003695	0.026193	-2.639234	6.884613	6.432867	6.722714	7.113859	6.514062	6.574106	6.774994	6.779911	6.897015	6.744496	6.906966	6.617184	6.934693	6.798091
Nup54	0.535481	4.469673	3.452804	0.0037	0.026218	-2.495034	4.045673	4.625556	4.673644	4.094353	4.659497	4.458273	4.8055	4.388622	4.500423	4.670575	4.483567	4.365074	4.314529	4.561731
Pcsk1	-1.550818	2.180028	-3.452149	0.003705	0.026224	-2.070566	1.764345	-0.103798	1.195284	3.137672	1.143601	1.563674	2.361772	2.951874	3.178898	2.572488	2.696109	2.404977	2.954296	3.151754
Galnt4	1.2164	1.942059	3.452106	0.003705	0.026224	-2.150441	2.488111	2.790938	3.215954	1.405223	2.847434	3.563537	1.607516	1.429209	1.01926	1.322149	1.996325	2.2368	0.546019	1.193114
Bub3	0.456707	5.099998	3.451609	0.003709	0.026235	-2.583296	5.110378	5.311505	5.44535	4.783243	5.549226	5.288824	4.931426	5.045558	4.870008	5.114467	5.109599	5.021232	5.047677	4.817576
Acvrl1	1.02005	4.733889	3.451177	0.003712	0.026242	-2.517597	4.59574	5.532015	4.821391	3.686144	5.408657	4.827752	5.044381	4.424739	5.119774	4.794232	4.417843	4.506292	4.274664	4.883051
Vps13b	-0.492707	6.312365	-3.448396	0.003733	0.026375	-2.64045	6.093446	6.052203	5.798935	6.482694	5.656516	5.683433	6.797221	6.530333	6.724974	6.338547	6.510393	6.40539	6.684637	6.635037
Cpsf4	0.675165	3.846685	3.4463	0.003749	0.026471	-2.449365	3.646165	4.604273	4.519244	3.611667	4.05518	4.028875	3.742028	3.798099	3.88398	3.808701	3.56201	3.674268	3.445698	3.584586
Nptx1	-1.473589	9.992434	-3.443092	0.003774	0.026622	-2.583675	9.911343	7.615279	9.521874	10.85979	9.049873	9.470685	10.06642	10.53901	10.62182	10.18841	10.32912	10.46108	10.74081	10.52051
Adcy1	-1.548721	6.849255	-3.44292	0.003775	0.026622	-2.653938	7.12897	5.677398	6.715509	7.436725	4.478849	6.057124	7.281291	7.493306	7.272098	6.900128	7.197279	7.724807	7.853495	6.690191
Cap2	-1.212093	7.029534	-3.442139	0.003781	0.026648	-2.655988	7.464529	5.3793	6.684622	7.943157	6.748244	7.147249	6.959908	7.398869	7.123999	6.993732	7.348249	7.077259	7.378708	6.778868
Klhl4	-0.940701	2.614189	-3.440918	0.00379	0.026664	-2.333083	3.154337	1.972946	2.456093	3.696477	2.856578	2.705027	2.361772	2.634802	2.166756	2.007132	2.374443	2.502617	3.02328	2.952659
Sh3gl2	-1.069186	7.266504	-3.440847	0.00379	0.026664	-2.657614	7.626991	6.097515	6.839994	8.000311	6.931935	7.10538	7.141943	7.620405	7.500348	6.912779	7.791338	7.138755	7.961958	7.07229
RGD1305537	1.942335	0.040657	3.440844	0.003791	0.026664	-1.757866	0.105685	1.310208	0.25957	-1.788967	0.845972	0.982423	-0.067151	0.222004	0.628769	0.055728	-0.072212	-0.24119	0.182287	-0.116166
Cyp3a9	-1.451675	2.057727	-3.440692	0.003792	0.026664	-2.029449	2.295333	1.480065	0.20995	1.799605	0.15195	0.809818	3.105363	2.849305	2.822431	2.956054	2.729	2.960934	1.983515	3.169358
Jak3	1.1148	5.068044	3.440067	0.003797	0.026674	-2.585283	4.365777	6.60116	5.271833	4.763722	5.581294	5.297023	4.685967	5.079939	4.752736	5.345599	4.967529	5.340819	4.11226	4.836982
Dlx1	-0.577867	4.756366	-3.439917	0.003798	0.026674	-2.588299	4.904771	4.196386	4.266652	5.197519	4.715852	4.726978	5.024227	4.647404	4.855111	4.766622	4.672958	4.536248	5.110168	5.02759
Oasl2	1.525952	3.188637	3.43628	0.003826	0.026843	-2.226294	2.515609	3.479652	3.910635	1.720809	3.365818	3.632543	3.137884	3.811801	2.583064	3.765957	3.302737	4.877826	2.333172	2.402413
Usp46	-0.560733	6.876918	-3.436223	0.003826	0.026843	-2.672878	7.142814	6.486195	6.680074	7.506284	6.839268	7.048242	6.983213	6.673235	6.771731	6.643543	6.885853	6.663159	7.094928	6.871847
RT1-CE10	1.187412	4.455204	3.435843	0.003829	0.026848	-2.476867	4.29216	4.24827	5.010785	2.890108	5.249432	4.434686	4.05746	5.074265	3.70281	5.181051	5.067356	5.506344	3.707626	4.032198
Prickle3	1.012144	2.932648	3.433526	0.003847	0.026958	-2.274782	2.474162	3.655486	3.215954	2.376521	3.770765	3.091837	2.979429	2.634802	2.858731	3.060806	2.921517	2.859272	2.133689	3.237699
Fcho2	-0.543207	5.010868	-3.432543	0.003855	0.026996	-2.649431	5.487189	5.028455	5.211652	5.907124	4.968684	5.414719	4.848708	4.873301	4.64946	4.640468	4.606661	4.864769	4.911337	4.789392
Tmpo	0.679525	5.289246	3.432242	0.003857	0.026997	-2.630635	4.849116	5.585746	5.219509	5.192053	5.985161	6.020953	5.14913	5.202027	5.000289	5.294957	5.122035	5.310324	5.114691	5.044382
Rpl10a	1.234254	2.001066	3.430037	0.003875	0.027093	-2.062002	1.278303	2.439156	2.445413	1.21709	2.672087	2.257897	2.144252	2.007376	2.568526	2.274406	1.557647	1.760874	1.316372	2.489076
Olfml2a	0.924472	4.813198	3.429677	0.003877	0.027093	-2.599329	5.010026	5.783999	5.708032	4.034524	5.109198	5.127168	4.637512	4.351577	4.815644	4.703306	4.235294	4.594355	4.394821	4.938001
Gnb5	-0.675505	4.795367	-3.42963	0.003878	0.027093	-2.587989	4.674093	3.785434	4.31439	5.066232	4.368121	4.310598	5.047237	5.243062	5.154258	4.882627	5.265325	4.990306	5.286892	4.806368
Lims1	0.991619	3.457322	3.428668	0.003885	0.02713	-2.465686	3.838085	4.302134	5.093491	3.075697	4.147475	4.131469	2.760215	3.036005	2.894141	2.831299	2.912	3.447848	3.186856	2.900911
Gpr4	1.139632	3.063997	3.427669	0.003893	0.027148	-2.338843	3.42474	4.000847	4.123244	1.962578	3.680967	3.334188	3.200804	2.724465	2.894141	2.934159	2.428842	2.680061	2.676906	3.031778
Pcdh19	-1.645296	5.255604	-3.427536	0.003894	0.027148	-2.617348	4.808006	2.679063	4.385956	6.549765	4.169655	4.863523	5.679562	5.872367	6.158224	5.257979	5.5737	5.628793	6.151559	5.856248
Fjx1	-0.677863	7.244692	-3.427471	0.003895	0.027148	-2.687987	7.523282	6.531193	6.795482	7.587987	6.784028	7.401083	7.273385	7.321956	7.242067	7.343858	7.264725	7.724084	7.215111	7.428098
Pcdhga9	-0.872703	1.980349	-3.426859	0.0039	0.027165	-2.194542	2.144973	1.369077	1.631973	2.890108	1.803478	1.892796	2.033384	2.099634	2.147322	1.964563	1.605665	2.203999	2.003164	2.341603
Lrrk2	-0.67952	5.376379	-3.426598	0.003902	0.027165	-2.6493	5.329568	5.391775	4.600917	5.764636	4.669909	4.850213	5.870489	5.539186	5.762769	5.463023	5.530819	5.456736	5.455051	5.623839
Tacr3	-1.216787	1.518064	-3.425653	0.003909	0.027201	-2.09712	1.787089	0.310827	0.851376	2.511387	1.705671	1.046069	2.01015	0.972957	2.55384	1.180545	1.260496	0.760186	2.11575	2.802815
Tmem132e	-0.657353	4.516517	-3.425084	0.003914	0.027216	-2.541334	4.193457	3.678943	3.945162	4.569411	3.741449	3.552622	5.196213	4.796129	5.154258	4.85376	4.549498	4.835621	4.808999	5.431174
Syde1	0.925933	3.2096	3.424739	0.003916	0.02722	-2.382635	3.549707	3.607396	4.182328	2.337441	3.823012	3.683465	3.094359	2.888628	2.694377	3.060806	3.091342	3.25317	2.913344	2.932182
Gzmm	-0.821543	3.12716	-3.422673	0.003933	0.027309	-2.281237	2.8647	1.812527	2.356997	2.960388	2.046579	2.160607	3.554005	3.683523	3.941526	4.020928	3.537463	3.805789	3.36447	3.880148
Itfg1	-0.667858	7.979417	-3.422556	0.003934	0.027309	-2.688386	7.977787	6.900813	7.501938	8.199838	7.581005	7.702994	8.176727	7.956996	8.301589	8.008611	8.142865	8.198265	8.486699	8.582269
Snx8	0.72899	4.26379	3.419675	0.003957	0.027452	-2.548283	4.369549	4.897106	4.519244	3.498125	4.725866	4.446528	4.330288	4.166665	3.91304	4.345154	4.107714	4.179285	4.056929	4.221283
Zfp593	0.790636	3.499059	3.418021	0.00397	0.027528	-2.42579	3.357948	3.713429	4.354903	2.908002	3.939464	3.59047	3.299932	3.567159	3.555781	3.401235	3.525031	3.600573	3.302384	3.012401
Large	-0.854805	7.759175	-3.415388	0.003991	0.027654	-2.710146	7.711904	6.001938	7.061616	7.724567	7.20947	7.182194	8.275641	8.17478	8.300765	8.100251	8.082637	8.212249	8.270905	8.327829
Pkib	-1.036823	2.041607	-3.415203	0.003993	0.027654	-2.274054	2.357438	1.58312	1.966737	3.40669	1.85913	2.101918	1.962529	1.68885	1.891068	1.569444	2.428842	2.027776	2.423441	1.708589
Folh1	-0.729479	4.594778	-3.414505	0.003998	0.027677	-2.649904	5.473232	4.874426	4.586407	5.019674	3.998482	4.613389	4.785113	4.145178	4.523219	4.290193	4.012962	4.575244	4.792156	4.704402
Fnbp1	-0.619377	6.667026	-3.413857	0.004004	0.027697	-2.71835	7.10541	6.822673	6.56761	7.30281	6.431569	6.519096	6.413678	6.763258	6.464352	6.463228	6.217747	6.854298	6.823735	6.604628
Kctd8	-1.199481	1.44387	-3.413479	0.004007	0.027703	-2.162277	1.764345	1.248835	0.642822	2.925676	1.43972	1.541762	1.258992	0.645799	1.636128	1.355481	1.652137	1.461155	1.770732	1.473753
LOC361016	-0.943461	4.895242	-3.412393	0.004015	0.027748	-2.690718	6.004053	5.232463	4.346317	5.808165	5.669545	4.634248	4.292269	4.616508	4.659784	4.31582	4.530966	4.733017	4.836642	4.909479
Plcd3	0.912272	4.998932	3.411545	0.004022	0.027779	-2.615027	4.602167	5.741464	5.15542	4.034524	5.223162	4.774727	5.100459	5.235456	4.83703	5.551971	4.92558	5.231141	4.51603	5.10738
Smad6	1.484137	1.227667	3.408761	0.004045	0.02792	-1.96206	1.212615	1.655851	1.687412	-0.411255	1.997824	1.72793	1.576217	0.923327	1.489496	1.678822	1.403186	1.008331	0.649393	1.32462
Cybb	3.311817	0.619124	3.408087	0.004051	0.027942	-1.876053	0.307952	1.184734	2.947351	-1.223791	2.944963	3.321383	0.028455	0.645799	-1.10526	-0.210791	0.263333	2.346065	-0.628995	-0.621773
Tecr	-0.517506	8.170585	-3.407628	0.004054	0.027952	-2.710691	8.357966	7.667882	7.743686	8.379709	8.094549	7.900208	8.16888	8.111095	8.522264	8.283503	8.058309	8.184143	8.312841	8.608719
Arf2	0.586167	4.750507	3.40209	0.0041	0.028241	-2.646705	4.579546	5.119134	5.389334	4.427757	5.044657	4.786421	4.551611	4.725493	4.60385	4.643844	4.949035	4.724369	4.713828	4.307278
Igfbpl1	-2.174273	1.604597	-3.401862	0.004102	0.028241	-2.129939	3.31946	-0.273517	0.158563	1.892408	0.808768	1.136566	1.443753	1.775313	1.819202	2.572488	3.695584	1.315214	1.493524	1.971974
Mum1l1	-1.298438	1.746139	-3.401552	0.004104	0.028241	-2.214833	2.091157	0.480615	0.975347	3.027403	1.997824	1.766229	2.247205	1.532126	2.04602	1.597576	1.903329	0.84773	2.437952	2.011549
Slc15a3	0.919317	4.132383	3.401418	0.004106	0.028241	-2.472175	3.522857	3.967899	3.926082	2.844379	4.430682	4.001042	3.999651	4.65122	4.13647	4.949639	4.502714	5.035286	3.602057	4.380898
Myo7a	1.499014	2.784651	3.400528	0.004113	0.028275	-2.19819	2.43149	3.1924	2.829082	0.823477	3.365818	2.845805	2.206902	3.923301	2.024877	3.204921	3.181236	4.087853	2.736815	2.402413
Pold2	0.408832	4.764523	3.399921	0.004118	0.028294	-2.663478	4.722094	5.146735	4.921122	4.624561	5.214297	5.043877	4.595201	4.628172	4.635579	4.683756	4.655954	4.594355	4.634155	4.661508
Rragd	-1.351299	4.394242	-3.395904	0.004152	0.028506	-2.625615	4.492273	2.383133	4.217279	5.419585	3.534831	4.220367	4.468816	4.816662	4.670035	4.406073	5.113756	4.718575	4.924278	4.221283
Zfp597	-0.954127	2.884529	-3.395656	0.004154	0.028506	-2.411983	3.163058	2.324846	2.558762	3.578533	2.198134	2.644629	2.814871	3.340143	3.140097	2.454393	3.132898	2.910998	3.671242	2.673087
Dcun1d2	-0.744699	3.912408	-3.394259	0.004165	0.02857	-2.59319	4.036211	3.390109	3.614277	4.659309	3.464807	3.97675	3.691803	3.966821	4.002283	4.005262	4.160911	3.631738	4.262487	4.017694
Fzd4	-0.539342	4.588872	-3.393955	0.004168	0.028572	-2.67559	5.26014	4.658378	4.432669	4.700959	4.436792	4.234096	4.587372	4.402272	4.62508	4.5783	4.534071	4.447955	4.686744	4.72538
Hyal2	0.8149	4.390379	3.393563	0.004171	0.028578	-2.589523	4.114729	4.926801	4.951633	3.561676	4.587184	4.094316	4.320877	4.318363	4.393136	4.369831	4.341911	4.66836	4.318455	4.575
Vwa1	1.029314	5.361654	3.393104	0.004175	0.028586	-2.705226	5.249943	6.200381	5.27032	4.575022	6.561664	5.708237	5.499182	5.010338	5.321818	5.279845	4.894507	5.058407	4.937104	5.536666
Slc17a7	-0.780754	10.531	-3.392873	0.004177	0.028586	-2.679829	10.21807	8.801613	9.777495	10.37982	9.784031	9.720775	10.93507	11.0633	11.24521	10.95795	10.99571	11.10859	11.23404	11.21355
Gpsm3	1.060546	3.324039	3.392308	0.004182	0.028596	-2.439651	3.467609	3.557647	4.123244	2.283631	4.255114	3.62735	2.814871	3.501034	3.100223	3.52358	3.324422	3.794816	2.974345	2.357047
Accs	0.948601	2.646696	3.392133	0.004183	0.028596	-2.293779	2.501925	3.263946	3.4152	1.740915	2.619344	2.836835	2.905804	2.58779	2.694377	2.655119	2.580719	2.568523	2.466543	2.474989
Zfp36l1	0.651845	6.330325	3.390956	0.004193	0.028648	-2.761763	6.652735	7.038655	7.336579	6.170667	6.864983	7.009851	5.586203	6.303023	5.519942	6.063962	5.99247	6.662405	5.710957	5.732928
Atxn1	-0.855517	5.001708	-3.390451	0.004198	0.028661	-2.708175	5.154777	4.619507	4.669061	5.70831	3.935155	5.101184	5.356788	5.307349	5.343328	4.922095	4.860277	4.862143	5.254467	4.981001
Shisa5	0.611432	6.329134	3.390126	0.0042	0.028663	-2.761955	6.342166	6.536821	6.897938	6.155767	7.070506	6.932496	5.57239	6.131972	5.749941	6.271373	6.279184	6.560008	5.998446	6.129006
RGD1562618	-0.63728	7.260874	-3.389666	0.004204	0.028674	-2.763914	7.524129	6.392644	6.805435	7.574395	7.237302	7.213284	7.295775	7.32016	7.357028	7.429984	7.4272	7.313255	7.406642	7.365523
Kank2	0.775461	5.38948	3.388344	0.004216	0.028734	-2.717709	5.390247	5.650954	5.582052	4.591724	5.683743	6.11206	5.256692	5.387314	5.144489	5.734068	5.17869	5.562412	4.852977	5.363965

Pomgnt2	-0.646764	5.228852	-3.387769	0.00422	0.028737	-2.703381	5.099062	4.010125	4.764524	5.321191	4.8728	4.592225	5.61921	5.3896	5.765958	5.535706	5.381938	5.533	5.569853	5.793779
Cacna1h	-0.676453	8.308993	-3.387711	0.004221	0.028737	-2.764207	7.598744	6.988495	7.262076	7.98195	6.991774	7.211983	9.295657	8.944889	9.391996	9.205851	8.643353	8.894859	8.663999	9.256415
Nrros	0.641831	4.051276	3.387449	0.004223	0.028737	-2.498365	3.388015	3.96313	3.918379	3.067759	4.05518	3.502454	4.005537	4.655026	3.974983	4.738473	4.64163	4.908688	3.892823	4.107181
Rab6b	-0.640934	10.25625	-3.386913	0.004228	0.028752	-2.671672	10.71016	9.8617	10.32201	10.92206	10.16824	10.35198	10.18333	10.0972	10.09591	9.954716	10.33709	10.05604	10.56384	9.96453
Unc5a	-0.882241	7.935464	-3.385344	0.004241	0.028826	-2.765208	7.75364	6.406762	7.447113	8.320192	7.454228	7.310439	8.288372	8.294595	8.529554	8.238989	7.952619	8.227375	8.219337	8.660402
Mdfi	1.803217	0.093856	3.384848	0.004245	0.028826	-1.872671	-0.046857	1.397635	0.715742	-1.388744	1.05105	0.435061	-0.831021	0.137473	-0.504027	0.893396	0.961591	0.666985	-0.755361	0.257494
Tmem150a	0.901309	5.22707	3.384807	0.004246	0.028826	-2.688318	4.816322	5.87567	5.312072	4.214255	5.536481	4.915569	5.349864	5.567732	5.206835	5.583962	5.221695	5.496219	4.698844	5.427521
Nt5c	1.058838	2.026376	3.384188	0.004251	0.028846	-2.220696	1.936985	3.27168	2.761803	1.353927	2.484288	2.203108	1.862293	1.68885	1.819202	1.898256	1.94125	2.2368	1.626159	1.684306
Mul1	-0.501687	5.193228	-3.383116	0.00426	0.028892	-2.735361	5.308106	4.749824	5.08147	5.611986	4.936707	5.06899	4.991976	5.250627	5.171196	5.155258	5.357605	5.374373	5.328373	5.362053
Ilk	0.574717	7.316249	3.38205	0.00427	0.028938	-2.779392	7.240808	7.788366	7.381744	6.929604	7.872811	7.593093	7.386533	7.2552	7.187812	7.294766	7.046695	7.168813	6.911485	7.37977
Cd163	2.731611	0.476797	3.381718	0.004272	0.028941	-1.968143	0.307952	2.33964	3.510504	-0.502853	1.312595	2.743931	-0.529223	-0.81357	-0.773548	-0.665882	-0.616402	1.219993	-0.040977	0.706485
Rasgrf2	-2.110584	6.158469	-3.38089	0.00428	0.028974	-2.762776	6.562509	2.874583	5.706915	7.541817	5.271819	5.842009	6.345699	6.908229	6.483852	5.875772	6.821228	6.455892	7.399245	6.163453
Tnfrsf26	2.673921	0.784679	3.380198	0.004286	0.028998	-1.931575	0.485316	2.724859	2.753168	-1.223791	2.240661	0.695385	0.429655	1.067371	0.317129	1.104165	1.068256	1.219993	-0.405292	-0.297732
Psma6	0.524639	5.710921	3.378228	0.004303	0.029097	-2.76873	5.538358	5.83579	6.162303	5.658972	6.340818	6.152383	5.715723	5.295206	5.497094	5.416404	5.730811	5.374373	5.689893	5.575144
Dtx3l	1.640555	1.840328	3.377593	0.004308	0.029118	-2.155669	1.341131	2.943995	3.277465	0.932396	2.254562	2.422822	1.408645	2.419003	1.218642	1.540753	1.290185	2.970717	1.150071	1.085968
Hmgcl	0.831456	3.908671	3.375626	0.004325	0.029218	-2.54415	3.730722	4.479583	3.93756	2.908002	4.377679	3.74229	3.804135	3.916976	3.941526	4.219542	3.949256	4.114573	3.811576	3.896095
Apbb1	-0.516703	8.540127	-3.374603	0.004334	0.029249	-2.772306	8.728535	7.814415	8.061053	8.350155	8.302471	7.920515	8.769213	8.863964	8.785898	8.676401	8.887176	8.72734	8.94286	8.736182
Mzt2b	0.589899	3.959018	3.374538	0.004335	0.029249	-2.593325	3.621067	3.958345	4.072089	3.68095	4.487704	4.431711	3.830902	4.193082	3.889839	3.978769	4.012962	3.895831	3.67737	3.797658
Kcnq3	-1.439331	6.173858	-3.373301	0.004346	0.029291	-2.78521	6.047088	4.902098	5.799984	7.320036	4.393472	5.870856	6.421404	6.794649	6.519246	5.794558	6.687475	6.347434	7.172902	6.388679
Tnfsf12	0.569141	4.885615	3.37303	0.004348	0.029291	-2.761165	5.348806	5.630045	5.487608	5.119037	6.107015	5.976724	3.889373	4.612599	4.055381	4.657271	4.524735	4.747316	4.075609	4.225496
Tbl3	0.640972	4.846492	3.372609	0.004352	0.029291	-2.682081	4.460789	5.136182	5.030571	4.07864	4.906209	4.543439	5.133088	4.918332	5.035072	5.082476	4.840346	4.872617	4.716806	5.152334
Zfp608	-0.506185	5.282565	-3.372596	0.004352	0.029291	-2.760723	5.35833	4.738705	5.025202	5.700631	5.194602	5.139986	5.415442	5.407759	5.468016	5.238011	5.427736	5.187762	5.415271	5.279355
Tspan12	0.61751	5.313668	3.372226	0.004355	0.029291	-2.772763	5.622966	5.630045	6.218272	5.204776	6.306197	5.949937	5.103206	4.721869	4.867041	4.741628	4.812484	4.978233	5.130412	5.145679
Hnmt	-0.976008	2.537024	-3.372177	0.004356	0.029291	-2.389857	2.957854	2.843782	1.874203	3.059778	2.28197	1.302125	2.787802	2.14364	2.882434	2.641672	2.481265	2.516041	2.994118	3.041369
Tmem59l	-0.657629	8.653357	-3.363059	0.004437	0.029819	-2.799618	8.49249	7.325943	7.761286	8.353424	8.230442	7.749739	9.072879	9.078774	9.326949	9.251245	8.982749	9.097784	9.041771	9.385931
LOC100365773	1.552503	0.505486	3.36258	0.004441	0.029832	-1.983599	0.539902	1.953842	0.715742	-0.705589	1.510943	1.302125	0.357848	0.517885	0.317129	0.280604	0.535373	0.804622	0.249639	-0.116166
Lppr5	-0.957642	5.897032	-3.36074	0.004458	0.029926	-2.813046	6.139466	4.595055	5.500563	6.722754	5.728009	6.058089	5.86078	5.939438	6.177512	5.811171	5.940759	5.92643	5.993542	6.192799
Slco1c1	-1.115683	7.00041	-3.359926	0.004465	0.029959	-2.823261	7.720791	6.679565	5.317939	7.221522	6.73343	6.704949	7.545983	6.743733	7.608673	7.245056	6.894939	6.953218	7.031757	7.617946
Scrt2	-0.880629	2.438467	-3.359002	0.004473	0.029998	-2.371193	2.682606	1.532513	2.039558	2.816229	2.496038	1.450626	2.613764	2.650138	2.74697	2.499819	2.792614	2.268872	2.933966	2.921834
Nalcn	-0.928778	7.196651	-3.358163	0.004481	0.030031	-2.828386	6.849927	5.71476	6.604681	7.822751	6.470058	6.832258	7.691378	7.507058	7.777772	7.069756	7.369265	7.319967	7.831338	7.904104
Acsl1	-0.375981	6.416963	-3.357905	0.004483	0.030031	-2.82729	6.518165	6.273469	6.090428	6.551901	6.024231	6.251054	6.627254	6.436157	6.52019	6.491614	6.382718	6.502159	6.574848	6.611888
Sbds	0.527529	6.931028	3.357424	0.004488	0.030035	-2.830381	6.787849	7.657219	7.124481	7.021336	7.389555	7.552921	6.65467	6.709372	6.704343	6.858594	6.530745	6.754478	6.491223	6.810765
Nkiras2	0.609652	3.143641	3.357185	0.00449	0.030035	-2.495076	3.073383	3.53854	3.381979	2.697792	3.649748	3.365711	3.094359	3.047633	2.772565	3.019813	3.149191	3.009199	3.160849	3.229331
Dnaja4	-1.084633	5.566315	-3.357016	0.004491	0.030035	-2.798575	5.507875	4.064569	4.673644	6.346626	5.095746	5.534671	5.789221	6.047627	5.802136	5.495156	6.115732	5.709884	6.12378	5.658531
Bcl10	0.501679	4.581406	3.356285	0.004498	0.030063	-2.720676	4.373312	4.972603	4.988886	4.331596	4.587184	4.8591	4.555628	4.481568	4.277224	4.653926	4.686975	4.781067	4.349488	4.307278
Brinp3	-0.719751	4.002303	-3.355539	0.004505	0.030091	-2.682314	4.184919	3.943895	3.761292	4.654018	3.680967	3.462191	4.074361	3.95452	3.985965	4.111508	3.467722	4.296718	4.221137	4.327019
Syt6	-0.951257	1.971705	-3.353471	0.004524	0.030201	-2.276177	2.035255	1.310208	1.705429	2.427034	1.230571	0.982423	1.913281	2.436799	2.260183	2.202958	2.468337	2.252925	2.794335	2.011549
LOC619574	0.563237	4.880634	3.350998	0.004546	0.030335	-2.724319	4.488808	4.982063	4.852041	4.066741	4.854661	4.647132	4.641297	5.290321	4.833994	5.262379	5.075905	5.475754	4.926852	4.985976
Nsfl1c	0.425981	7.373775	3.349845	0.004557	0.030389	-2.838091	7.478133	8.032403	8.114913	7.593871	7.86745	7.832162	6.940836	7.097025	7.150474	7.134193	7.165593	6.913192	6.949328	6.973043
Cntn6	-1.279405	2.837284	-3.349389	0.004561	0.030395	-2.517727	2.906846	3.224637	2.010869	4.235668	2.030509	2.174914	2.905804	2.287882	3.216682	3.019813	2.274011	3.170693	3.107385	3.395603
Cdk1	3.420676	0.47609	3.349199	0.004563	0.030395	-1.960023	-0.518419	2.607522	0.945338	-0.941548	3.623205	2.949304	-1.009457	0.449407	0.245823	0.699359	-0.149817	0.714338	-0.628995	-0.748265
Trh	3.900815	2.335557	3.347626	0.004577	0.030475	-2.172581	0.830061	4.499482	5.142254	0.577356	2.60856	5.843129	1.17791	1.112357	1.914249	2.558244	2.100543	1.805289	1.077784	1.910497
Angptl2	1.982233	1.724343	3.347335	0.00458	0.030476	-2.331636	3.431974	3.055728	3.855228	0.041976	3.434596	3.866483	0.282279	1.020936	-0.080923	1.104165	1.533025	1.515634	0.791767	0.970221
Drc1	-0.649009	5.403115	-3.346865	0.004585	0.030488	-2.798667	5.492389	4.979704	4.817254	5.277123	4.805931	4.348958	6.054927	5.724457	5.862971	5.752952	5.696576	5.366984	5.511022	5.99228
Mcee	-0.560815	4.260138	-3.344639	0.004605	0.030609	-2.744338	4.816322	4.138342	3.776458	4.560953	4.282511	4.326706	4.32559	4.095628	4.263751	4.154751	4.345451	4.319865	4.310591	4.007943
Faim2	-0.753852	8.532188	-3.342636	0.004624	0.030716	-2.831906	8.455698	7.534805	8.043012	9.06563	8.285221	8.160488	8.385519	8.70875	8.896613	8.805623	8.588028	8.813954	8.680491	9.031251
R3hdm1	-0.449992	7.948803	-3.340456	0.004644	0.030823	-2.845866	8.167691	7.73096	8.026312	8.485905	7.742928	8.00627	8.081368	7.842757	7.971171	7.865196	7.827855	7.790147	7.856874	7.894213
Tpx2	1.489478	2.734301	3.340374	0.004645	0.030823	-2.476321	2.569081	3.63764	2.939761	2.297273	4.740757	4.326706	2.206902	2.650138	1.794428	2.256873	2.303426	2.726993	1.902128	2.194616
Arfgap3	0.410015	4.783033	3.339552	0.004653	0.030857	-2.788547	4.824591	5.123415	5.186218	4.619139	5.12631	5.07663	4.648839	4.782275	4.511866	4.535316	4.770972	4.7671	4.536402	4.510857
Lrfn1	-0.449776	5.525404	-3.339002	0.004658	0.030875	-2.808067	5.241734	4.667202	5.08147	5.197519	4.662107	4.659903	6.096885	5.81058	6.096229	5.859891	5.823827	5.920132	6.185278	6.090256
Gna15	0.934502	3.29833	3.336994	0.004677	0.030971	-2.468684	2.765113	3.309744	3.056656	2.154676	3.761059	3.365711	2.398025	4.544473	2.55384	4.246444	4.095154	4.761475	2.614401	2.733504
Itgb3bp	0.635834	2.479094	3.336918	0.004678	0.030971	-2.46811	2.670424	3.037697	3.098159	2.376521	3.403739	3.038068	2.186319	2.207232	1.794428	2.202958	2.374443	2.268872	2.269694	2.068949
Msh5	2.780178	0.27568	3.33625	0.004684	0.030979	-1.91338	-0.518419	1.747452	1.220561	-2.344749	0.691028	-0.006501	0.743216	1.356279	0.17081	1.355481	0.321999	0.889587	-0.405292	0.499386
Cdh5	0.932793	4.369023	3.335881	0.004688	0.030979	-2.769553	4.889087	5.507659	5.437287	3.805502	5.097676	5.004367	4.07995	3.865347	4.421726	4.015725	3.258364	4.056042	3.731382	4.079517
Gabbr2	-0.691021	9.224125	-3.33572	0.004689	0.030979	-2.825982	9.213284	8.062812	9.021311	9.512624	8.736863	8.874231	9.430297	9.468146	9.472165	9.37157	9.350201	9.47021	9.554239	9.602566
Cnp	-0.836523	9.059784	-3.335707	0.004689	0.030979	-2.83288	9.399157	8.697611	7.926881	9.000669	8.749649	8.077489	8.886692	9.495335	9.094461	9.389657	9.349099	9.833775	9.470809	9.468903
Tbc1d2b	0.504627	5.174248	3.335133	0.004695	0.030982	-2.806371	4.846411	5.455981	5.504427	4.761263	5.216075	5.049711	5.141132	5.412264	4.83703	5.436012	5.350577	5.600717	4.957391	4.914707
Atrn	-0.657078	8.892059	-3.335116	0.004695	0.030982	-2.827688	8.767836	7.954654	8.477807	9.302306	8.340035	8.737992	9.033991	9.085135	9.108033	8.908863	9.037386	9.147824	9.317885	9.272539
Gja5	1.755615	0.315548	3.334496	0.004701	0.030988	-2.065791	0.592497	0.934883	1.430088	-0.411255	2.046579	2.481552	-0.399109	0.301855	0.449965	-0.537962	-0.616402	0.007173	-0.211665	-0.204094
Hipk4	-0.945519	5.874842	-3.334478	0.004701	0.030988	-2.837694	5.288313	3.972652	4.949745	6.193644	5.130085	5.123484	6.57675	6.316303	6.760583	6.174313	6.184537	6.473202	6.544158	6.592446
Nup205	0.434034	5.143021	3.333561	0.00471	0.031028	-2.804297	4.640141	5.116989	5.187821	4.874798	5.201795	5.263945	5.461136	5.255649	5.264345	5.140992	5.050107	5.053812	5.167891	5.367782
Gja4	1.133507	2.099287	3.331331	0.004731	0.031151	-2.321987	2.402324	2.679063	2.626517	0.860705	2.901447	2.581593	2.398025	1.464337	1.981639	2.239124	1.844511	1.950783	1.837926	2.011549
Nubp2	0.625295	5.017176	3.330055	0.004743	0.031215	-2.821107	5.119368	5.506021	5.569811	4.552446	5.721768	5.018319	4.812232	4.895992	4.703686	4.971302	4.944375	4.856878	4.777968	4.839733
Cyth1	-0.45969	5.474461	-3.329448	0.004749	0.031217	-2.858091	5.611862	5.146735	5.449365	5.790187	5.231972	5.138162	5.517835	5.642482	5.375002	5.541148	5.416002	5.731623	5.654608	5.431174
Rps27	0.679749	3.658417	3.328973	0.004754	0.031217	-2.598709	2.997401	3.958345	3.436929	3.254167	3.965054	3.897009	3.957761	3.720315	3.555781	3.962637	3.609886	3.587916	3.695599	3.745801
Setd7	-1.076109	5.024837	-3.328952	0.004754	0.031217	-2.847488	5.382801	4.407694	4.792184	6.188163	4.339058	5.3068	4.877881	5.220124	4.875924	4.491011	5.190547	5.046893	5.691408	4.588148
RT1-M1-4	2.987399	0.416648	3.328934	0.004754	0.031217	-1.914316	-0.760787	1.082904	0.307539	-2.731185	0.649545	0.435061	2.078759	-0.048075	0.787836	1.180545	0.924208	1.315214	0.879431	1.824228
Samd5	-0.954287	3.406973	-3.325128	0.004791	0.031441	-2.687476	3.793849	3.159427	3.370733	4.503267	2.81965	3.479584	3.40164	3.292072	3.110295	2.831299	3.61576	3.18757	4.143583	3.142871
Dpysl4	0.442167	6.084949	3.323426	0.004807	0.031532	-2.890711	5.874648	6.564634	6.573753	6.100429	6.286034	6.295926	5.868875	5.958043	6.01546	6.098824	5.861397	5.930195	5.813285	5.971108
Cd93	1.202869	3.6721	3.323007	0.004811	0.031542	-2.679287	3.311637	3.673115	5.186218	3.567317	4.633136	5.020301	3.328389	3.221962	3.648551	2.934159	2.673757	3.555779	3.286436	3.503633
Vgll4	-0.466056	4.439698	-3.322058	0.00482	0.03157	-2.793586	4.69529	3.967899	4.156367	4.682885	4.325951	4.437656	4.371894	4.616508	4.227195	4.585342	4.64163	4.677349	4.484924	4.358053
Rbpms	1.220424	1.782936	3.322034	0.004821	0.03157	-2.306198	2.091157	2.631765	2.663852	0.785264	3.028244	2.05628	1.047044	1.80302	1.663747	1.828755	1.507975	1.760874	1.001683	1.582867
Tonsl	0.73936	2.93179	3.320175	0.004839	0.031671	-2.553199	2.718545	3.982112	3.138503	2.816229	3.422332	3.524966	3.060831	2.436799	2.858731	2.469694	2.415434	2.738492	2.805569	2.868948
Ttc3	-0.60944	9.68979	-3.319639	0.004844	0.031688	-2.841359	9.447252	8.90485	9.256575	10.19622	9.225236	9.455291	10.00567	9.783392	9.970116	9.737649	9.808167	9.778628	10.05919	10.03081
Ckap2	3.115215	-0.229118	3.317205	0.004868	0.031827	-1.954669	-0.310971	1.874793	-0.007587	-2.344749	2.198134	1.426914	-0.529223	0.301855	-0.929891	-0.027758	-0.411343	0.514819	-1.218554	-1.212031
Aass	-0.963263	3.123014	-3.316541	0.004875	0.031853	-2.671852	4.369549	3.339488	2.250588	3.533136	3.075998	3.334188	3.392685	2.488905	2.870631	2.923085	3.082885	2.838051	3.125428	3.286911
Rps11	0.486121	7.486521	3.316194	0.004878	0.031858	-2.905736	7.593512	8.078055	7.658104	7.397285	8.096238	8.09099	7.24754	7.320759	7.249492	7.455666	7.322439	7.281674	6.975053	7.053453
Nap1l5	-0.612954	6.800753	-3.313379	0.004906	0.032022	-2.917856	7.117239	6.850699	6.772166	7.783306	6.937332	6.785241	6.847245	6.277327	6.701847	6.444957	6.673462	6.228116	7.120453	6.685611
Tagln3	-0.674391	8.426051	-3.312289	0.004917	0.032067	-2.881699	9.014401	8.023555	8.35621	9.166633	8.691115	8.595698	8.090743	8.33133	8.546575	8.182358	8.518983	8.080095	8.377785	7.993905
Samd4a	0.631719	3.412943	3.31215	0.004918	0.032067	-2.668233	3.31946	3.889638	3.855228	3.36843	4.023054	4.124114	3.419386	3.024282	3.09008	3.293574	2.771719	3.482452	2.984265	3.286911
Eif2s2	0.566032	6.09053	3.310889	0.004931	0.032126	-2.920423	5.979578	6.501077	6.931332	6.478209	6.645823	7.103511	5.681202	5.674709	5.678967	5.71814	5.814919	5.683357	5.809105	5.591559
Irgm	0.987833	3.593043	3.310489	0.004935	0.032126	-2.659571	3.556342	4.134105	4.544485	2.862845	3.89578	4.105563	3.148563	3.838823	2.882434	3.856048	3.73929	4.709839	2.549064	2.622863
Cdca8	1.44926	1.206023	3.31033	0.004936	0.032126	-2.203535	0.539902	1.874793	1.534559	0.860705	2.936364	2.146157	0.282279	1.279465	1.01926	0.750369	1.03357	1.515634	1.040235	0.799858
Tsc22d1	-0.501834	8.433689	-3.310167	0.004938	0.032126	-2.897218	8.705215	7.878819	8.32341	8.646571	8.179434	8.320661	8.515215	8.459817	8.490323	8.316407	8.69334	8.427112	8.696609	8.423311
Il20rb	2.567537	-1.340858	3.30856	0.004954	0.032214	-1.973739	-1.052261	0.934883	1.195284	-1.575022	1.600747	0.175273	-3.260591	-1.196868	-1.304935	-1.33445	-2.697527	-0.920914	-2.232431	-1.406952
Gfra1	0.655159	5.670559	3.306196	0.004978	0.03235	-2.890451	4.950824	5.571717	5.716941	5.334484	6.138281	5.770798	5.439557	5.629	5.789655	5.46111	5.770848	5.843669	6.027521	5.974866
Grm7	-1.136888	5.468537	-3.304682	0.004993	0.032432	-2.882465	5.216821	3.317238	4.79429	6.019411	4.698159	5.08423	5.891303	5.940998	6.204796	5.693914	5.730811	5.733061	6.0966	6.180915
Reep6	-0.538809	5.436159	-3.303067	0.005009	0.032521	-2.923379	6.105938	5.165539	5.400436	5.936843	5.709204	5.650538	5.015502	5.157111	5.24619	5.474446	5.244623	5.281137	5.322519	5.432996
Flot1	0.430003	7.653061	3.302291	0.005017	0.032554	-2.931657	7.570753	7.949238	7.875343	7.648776	8.25334	8.079153	7.418321	7.38232	7.546514	7.638566	7.48651	7.432204	7.360733	7.508962
Stmn3	-0.635505	8.72432	-3.301991	0.00502	0.032556	-2.897259	9.128024	8.09232	8.508113	9.334811	8.862596	8.923253	8.680474	8.47685	8.858467	8.637976	8.608426	8.459174	8.811986	8.761792
Scn1b	-0.51364	9.061479	-3.301098	0.005029	0.032598	-2.910905	9.088086	8.062812	8.39124	8.563631	8.50975	8.296943	9.462617	9.405985	9.534385	9.587168	9.447385	9.561851	9.515961	9.436103
Fbxo5	1.368959	0.719349	3.298466	0.005056	0.032748	-2.1477	0.428583	1.607774	1.033553	0.041976	2.226624	1.327961	0.851758	0.517885	0.385075	0.646479	0.263333	1.045835	0.434663	0.257494
Gpr155	-0.827368	6.993704	-3.298283	0.005058	0.032748	-2.949011	6.870706	5.59964	6.155766	7.287085	6.33183	6.91662	7.671615	7.266439	7.355441	7.268212	7.130633	7.251895	7.411705	7.407848
Rcl1	0.483401	4.15643	3.297533	0.005066	0.032754	-2.823707	4.167689	4.432057	4.819324	4.294667	4.710819	4.883261	3.585784	3.904241	3.848317	4.031278	4.004032	4.092341	3.743115	3.763295
Nlgn3	-0.851014	7.421325	-3.297446	0.005066	0.032754	-2.944555	7.481187	6.335671	7.24601	8.199611	6.997985	7.374211	7.344773	7.694391	7.615761	7.27149	7.434697	7.485209	7.791284	7.635863
Gfod1	-1.213403	4.885582	-3.297397	0.005067	0.032754	-2.879572	5.210525	3.151065	4.59126	5.6695	4.421469	4.700027	4.986034	5.243062	4.881816	4.546183	5.136409	5.143038	5.86521	4.912095
Nod2	1.465655	-0.248778	3.296564	0.005076	0.032783	-2.080322	0.031429	1.184734	1.430088	-0.600663	1.082564	0.571087	-0.529223	-1.196868	0.245823	-0.806258	-1.500575	-0.24119	-0.755361	-0.297732
Arl4d	-0.64563	3.280911	-3.296265	0.005079	0.032783	-2.689468	3.832629	3.117121	2.770386	3.533136	2.901447	3.37813	3.29032	3.201289	3.350165	3.417108	3.50618	2.980434	3.515771	3.302949
Lars	0.376404	6.726137	3.296157	0.00508	0.032783	-2.953406	6.436899	6.983794	6.953644	6.737839	6.898571	7.019793	6.769816	6.628447	6.586629	6.609411	6.587147	6.610942	6.659318	6.698551
Ric3	-0.464484	5.990644	-3.293263	0.00511	0.032937	-2.943938	5.770073	5.428566	5.686652	6.25085	5.465045	5.607677	6.166842	6.149614	6.292553	6.042377	6.131194	6.182542	6.392241	6.328255
Sec61g	0.668483	3.028266	3.293058	0.005112	0.032937	-2.66846	3.402817	4.175923	3.510504	2.844379	3.726565	3.722947	2.613764	2.709903	2.47808	2.819403	2.651054	2.783591	2.535634	2.622863
Papss2	0.748937	4.579306	3.293049	0.005112	0.032937	-2.878885	4.849116	5.763574	5.408707	4.390058	4.812994	5.466522	4.451671	3.966821	3.952765	4.14048	4.03505	4.296718	4.330171	4.315206
Apc	-0.496835	9.237641	-3.29211	0.005121	0.032967	-2.90834	9.483605	9.008153	9.153199	9.400768	8.565182	9.058765	9.515525	9.222758	9.408543	9.102126	9.143616	9.251656	9.555071	9.460661
RGD1305733	-0.53869	7.331948	-3.292069	0.005122	0.032967	-2.954874	7.779745	7.104017	7.345223	7.822161	7.360925	7.240745	6.957633	7.167329	7.398716	7.207329	7.476457	7.106949	7.546284	7.1436
Add2	-0.922464	6.648522	-3.291511	0.005128	0.032978	-2.9613	6.739837	5.482885	6.340756	7.152837	5.797314	6.47063	6.882057	7.017957	7.072124	6.511157	6.876708	6.910654	7.393673	6.447576
Top2a	2.778024	2.204435	3.291297	0.00513	0.032978	-2.398239	1.764345	3.914553	2.498042	1.033666	5.131969	4.9923	1.047044	1.356279	1.326257	1.780488	1.319276	2.252925	1.28461	1.608903
Hebp2	-1.068014	1.848239	-3.291108	0.005132	0.032978	-2.458611	2.529164	1.082904	1.809043	2.666599	2.373993	0.915838	1.607516	1.658838	2.295923	1.652245	2.166061	1.568131	1.902128	2.105984
Slc16a1	0.837831	6.001175	3.289736	0.005146	0.033046	-2.953779	5.545046	6.249189	6.830791	6.015289	6.291944	7.103979	5.623044	5.742456	5.6308	5.583962	5.417684	6.190936	5.804913	6.011927
Mgat4c	-1.121308	2.779726	-3.289464	0.005149	0.033046	-2.645461	2.843156	2.082541	2.36835	4.277563	2.399242	3.045873	3.105363	2.030999	2.810125	2.558244	2.468337	2.619163	3.372046	3.178079
Chtf18	1.079096	2.664852	3.289304	0.005151	0.033046	-2.471021	2.144973	3.159427	2.673036	1.780306	3.601614	2.675144	3.105363	2.488905	2.759824	2.966878	2.317912	2.594065	2.408783	2.890335
Serpinb1a	1.681038	2.103162	3.288792	0.005156	0.033062	-2.441106	2.706665	3.159427	4.139901	0.74601	3.152236	2.762997	0.028455	2.724465	0.628769	2.375352	3.220315	2.715403	0.375586	1.227132
Necap2	0.618891	5.894324	3.288323	0.005161	0.033066	-2.947224	5.88259	6.338435	5.980978	5.22989	6.38177	5.980801	5.730014	5.993044	5.623786	6.082743	5.863867	6.197199	5.446106	5.816302
Stc1	-0.92089	2.225358	-3.288003	0.005164	0.033066	-2.497623	2.810225	1.117652	2.47722	2.613062	1.643636	1.857753	2.398025	2.38274	2.347926	2.007132	2.568656	2.2368	2.614401	2.431883
Gnao1	-0.751044	8.689246	-3.287943	0.005165	0.033066	-2.927386	8.913519	8.017242	8.49085	9.494604	8.474549	8.826602	8.474946	8.774297	8.684632	8.373196	8.885809	8.607829	8.997609	8.63766
Pim1	0.999345	1.267307	3.287621	0.005168	0.03307	-2.353473	1.278303	2.397344	2.837276	1.454757	1.912714	2.217002	0.743216	1.067371	0.628769	1.180545	0.484915	1.219993	0.649393	0.383504
Mrpl45	0.432759	5.241139	3.286881	0.005176	0.033102	-2.932718	5.243791	5.677398	5.746607	5.251074	5.746573	5.547098	4.795342	4.934079	5.142036	5.140992	5.176705	5.00228	5.112432	4.901601
Lta4h	0.428281	5.512491	3.285949	0.005186	0.033137	-2.939329	5.401346	5.551208	5.667249	5.258066	5.977847	5.826233	5.365968	5.45873	5.357796	5.44765	5.504148	5.544509	5.33614	5.472529
H3f3b	0.490738	7.242881	3.28584	0.005187	0.033137	-2.959019	7.704829	8.063091	8.496681	7.957726	8.186476	8.543934	6.562845	6.409369	6.598305	6.540437	6.657177	6.617963	6.611314	6.461932
Yae1d1	0.488309	4.494837	3.285473	0.005191	0.033144	-2.900781	4.689266	4.977341	5.236646	4.812037	5.349976	5.385799	3.999651	4.009066	3.88398	3.940842	4.086719	4.208518	4.28674	4.134325
Psmc4	0.403383	6.582232	3.284496	0.005201	0.033191	-2.975703	6.538414	6.913229	6.879211	6.384663	7.017989	6.647116	6.523389	6.428395	6.565736	6.49909	6.455767	6.367012	6.403375	6.544349
Layn	0.853521	2.832724	3.284228	0.005204	0.033192	-2.566364	2.8647	3.256169	3.520715	2.029493	3.245679	3.038068	3.060831	2.505864	2.462438	2.454393	2.873292	2.999674	2.736815	2.83626
Mcam	0.879836	6.499636	3.283857	0.005208	0.033199	-2.968104	5.817796	7.134559	6.484626	5.927005	7.206791	6.182788	6.518251	6.735668	6.342541	6.544958	6.154586	6.910018	6.322593	6.730771
Klhl8	-0.415228	5.636607	-3.282462	0.005222	0.033262	-2.956495	5.781445	5.228494	5.30913	5.760949	5.490232	5.396521	5.86078	5.698876	5.912737	5.585718	5.802096	5.647211	5.862523	5.607789
Dnmt1	0.398832	6.094973	3.282401	0.005223	0.033262	-2.97231	5.898344	6.466111	6.292407	6.058988	6.321962	6.430947	5.967162	6.062031	6.06161	5.968901	5.764251	6.057295	5.949913	6.052809
Paics	0.711543	2.656769	3.281846	0.005229	0.033274	-2.554742	2.658139	3.176008	2.916749	2.124384	3.052318	3.193712	2.206902	2.454377	2.772565	2.39151	2.568656	2.827322	2.601568	2.503027
Ska1	2.605983	0.066229	3.281699	0.00523	0.033274	-2.066257	-0.410969	1.655851	0.25957	-1.223791	2.411703	2.131561	-0.067151	0.04768	-0.175642	-0.311823	-0.149817	0.514819	-1.047095	-0.886923

Melk	4.844636	-0.749776	3.280738	0.005241	0.033321	-2.002245	-1.052261	1.506527	-0.195392	-3.260591	2.661692	1.993092	-1.21312	-0.380636	-1.813089	-0.537962	-0.731011	-0.78301	-1.218554	-0.886923
RT1-Db2	4.049712	-1.022515	3.280459	0.005244	0.033322	-2.043276	-2.235785	-0.813242	0.158563	-3.260591	0.691028	-0.1418	-2.558588	1.155984	-3.260591	0.893396	1.740817	1.691577	-0.755361	-1.040339
Ppp1r16b	-0.802905	6.267282	-3.27984	0.00525	0.033346	-2.982286	6.627752	6.165518	6.257725	6.710061	5.494631	5.551217	6.294932	6.42617	6.159437	6.194024	6.030347	6.706982	6.81125	6.333144
Hagh	-0.422002	5.488239	-3.277072	0.00528	0.033509	-2.965205	5.666549	5.097537	5.38794	5.801	5.459054	5.30192	5.634485	5.347885	5.600752	5.386484	5.602089	5.329459	5.663896	5.591559
Cacna2d1	-1.330595	6.487392	-3.27691	0.005281	0.033509	-2.985222	6.208491	4.670131	5.927297	7.584862	5.264968	6.383228	6.812411	7.013516	7.091955	6.291963	6.816772	6.675923	7.254583	6.848133
Nnmt	1.591125	0.223196	3.275738	0.005294	0.033551	-2.099417	0.176305	0.369671	1.220561	-1.075778	1.390206	0.881356	0.202532	-0.511025	0.571595	0.055728	0.202178	0.081289	0.111637	0.887553
Zfp217	1.002888	2.455254	3.275524	0.005296	0.033551	-2.501956	2.372556	2.631765	3.307264	1.63746	2.640673	3.255598	2.05625	2.76729	1.819202	2.572488	2.66245	3.204252	1.626159	2.124152
Cnr2	1.413277	1.847946	3.2755	0.005296	0.033551	-2.299485	1.430522	2.780133	2.597868	0.33436	1.78444	1.747207	0.743216	3.180315	0.736738	2.923085	2.930971	3.440827	1.1849	0.606649
Tmem132b	-1.349369	5.02836	-3.274686	0.005305	0.033589	-2.933472	5.080769	3.08236	4.884032	6.165098	4.176973	4.907024	5.108686	5.307349	5.489397	4.680472	5.140489	5.449758	5.725816	5.25483
Cacna1c	-1.048614	6.256913	-3.274172	0.005311	0.033606	-2.978131	5.696893	4.386479	5.745519	6.782778	4.989615	5.623411	7.079135	6.78513	6.99336	6.577103	6.615275	6.563245	6.980019	6.803737
Pter	-1.184705	5.554736	-3.273893	0.005314	0.033607	-2.978217	5.646478	4.449211	4.97038	6.982282	5.394602	5.705775	5.528095	5.207221	5.41415	5.937604	5.27092	5.760108	5.507587	6.027695
Fndc5	-0.551155	7.235729	-3.273323	0.00532	0.033628	-2.998517	7.574026	6.507642	6.734826	7.212101	6.981885	7.146795	7.360934	7.381171	7.193153	7.429984	7.437187	7.496258	7.53156	7.323392
Mllt4	-0.53	6.859803	-3.270768	0.005347	0.033785	-3.005237	6.766549	6.569349	6.64841	7.448236	6.465583	6.625794	7.11769	6.969877	7.067608	6.698966	6.805894	6.685609	7.196563	6.984897
Hrasls5	1.958649	-0.758866	3.269945	0.005356	0.033794	-2.04245	-0.634519	-0.273517	0.353965	-1.788967	0.517419	1.249022	-0.672244	-0.992527	-0.504027	-1.33445	-0.991788	-0.541385	-1.413173	-0.748265
Tmeff1	-1.066865	6.250338	-3.269764	0.005358	0.033794	-2.996984	6.011315	4.921894	5.202167	7.144842	5.611315	6.311387	6.2658	6.523062	6.564821	6.466092	6.529968	6.788755	6.706526	6.479679
Mxi1	-0.405364	6.693705	-3.269612	0.00536	0.033794	-3.007868	7.239778	6.735869	6.662312	6.893012	6.695078	6.553038	6.526463	6.660985	6.360585	6.800027	6.78193	6.752352	6.478996	6.586726
Psmd8	0.452123	6.239592	3.269589	0.00536	0.033794	-2.998964	6.144979	6.314297	6.537126	5.886966	6.683563	6.331483	6.161579	6.115493	6.289232	6.061439	6.330987	6.134393	6.253519	6.13013
Cplx2	-1.314054	8.174808	-3.267576	0.005382	0.033914	-2.969655	8.95444	7.065045	8.394193	9.65692	7.565405	8.945198	8.041458	7.97233	7.96702	7.548212	7.976784	8.055886	8.508346	7.802006
Sema4d	-0.648153	5.696482	-3.263608	0.005425	0.03417	-2.978955	5.582908	5.184101	4.718692	5.601028	5.154387	4.739064	5.68304	6.321101	5.956663	6.128049	5.912402	6.499627	6.253519	6.049248
Luzp1	-0.796221	6.736314	-3.263078	0.005431	0.034188	-3.019398	6.891845	6.10511	6.415168	7.607966	6.395951	6.895178	6.683411	6.907432	6.594723	6.445925	6.977666	6.732364	7.138334	6.532496
Itpripl2	1.458026	0.831798	3.262057	0.005442	0.03422	-2.219796	0.592497	0.973336	2.274922	-0.093872	1.643636	1.496914	0.429655	1.020936	0.736738	0.413143	0.584127	1.345607	0.836265	0.321874
Bcas3	-0.449729	6.499966	-3.261866	0.005444	0.03422	-3.021803	6.691679	6.139334	6.28269	6.856043	6.429273	6.446509	6.430183	6.673235	6.430586	6.332283	6.686777	6.500472	6.638267	6.479679
Axin2	-0.488549	5.192938	-3.261187	0.005452	0.03422	-2.986348	5.638683	5.298313	4.97038	5.499945	4.843207	5.219778	5.229287	5.127283	5.25075	5.169384	5.142525	5.175124	4.890388	5.289453
Zrsr1	-0.805231	5.50069	-3.261128	0.005453	0.03422	-3.010691	5.827428	5.005446	5.481086	6.732201	5.431783	5.979783	5.211572	5.535061	5.345461	5.183373	5.429405	5.196126	5.456834	5.229881
Def6	1.075105	0.40443	3.261067	0.005453	0.03422	-2.194703	-0.217457	0.895377	1.033553	0.485009	1.312595	1.426914	0.028455	0.449407	0.007959	0.699359	0.676952	0.804622	-0.305233	-0.397871
Bmp3	-1.21659	3.084223	-3.261064	0.005453	0.03422	-2.767376	3.37306	2.232748	3.619037	4.610969	2.399242	2.872384	3.094359	2.939445	2.973531	2.529327	2.651054	3.237047	3.766298	3.079111
Tifa	0.664007	3.22641	3.259908	0.005466	0.034282	-2.711748	3.171726	3.551306	3.797607	2.835056	3.563034	3.68846	3.049479	3.458111	2.568526	3.393233	3.359854	3.721386	2.892424	2.294252
Ccng2	-0.574816	5.096501	-3.258449	0.005482	0.034366	-2.980927	5.490658	4.382912	4.827573	5.383269	5.068462	5.156301	5.081078	5.107976	4.983948	5.06998	5.250298	5.27917	5.098798	5.217242
Mvb12a	0.609762	5.011467	3.257763	0.00549	0.034384	-2.951503	4.970829	5.437189	5.262736	4.37727	5.397737	5.008367	5.081078	4.833553	4.964647	5.251354	4.988055	5.081164	4.683703	4.872343
Agmo	-1.520447	0.996156	-3.257558	0.005492	0.034384	-2.264353	1.401336	0.369671	-0.974047	1.353927	0.606834	-0.072565	1.297885	1.564863	0.97585	1.51148	0.961591	1.97042	2.079189	1.868007
Plbd1	2.862776	-0.357348	3.257416	0.005494	0.034384	-2.114612	0.643243	0.934883	2.025285	-2.040259	2.484288	1.648138	-1.73438	0.58326	-2.604521	-0.210791	-0.231835	1.950783	-2.656794	-1.899685
Ppap2a	0.638708	5.592921	3.256938	0.005499	0.034399	-2.997606	5.204201	5.474531	5.87453	5.397589	6.175292	6.23151	5.209024	5.493155	5.42831	5.551971	5.512044	5.608571	5.422586	5.750578
Fn3krp	-0.733814	5.07406	-3.256133	0.005508	0.034437	-2.985775	5.426916	4.220564	4.829628	5.589986	4.757939	5.278924	5.027123	5.132753	5.127233	4.815347	5.311298	5.044579	5.471014	5.051519
Prg4	2.253365	2.71752	3.251371	0.005561	0.03475	-2.795113	4.846411	4.208526	5.870544	1.526009	4.985453	6.256109	1.63815	1.830204	1.360416	0.750369	0.805871	0.889587	1.770732	1.73247
Entpd6	-0.478294	6.755299	-3.251146	0.005564	0.03475	-3.042193	6.638736	6.010053	6.147553	6.745323	6.554658	6.144226	6.996575	6.867805	7.198474	7.033533	6.91473	6.874632	7.162018	7.301108
Frmpd4	-1.30855	5.799254	-3.249266	0.005585	0.034858	-3.027953	5.945863	4.49288	5.543762	6.898616	4.822358	5.581066	5.841165	6.377474	5.86446	5.199524	6.397285	5.807279	6.750785	5.698484
Cyp2d4	-0.764428	4.589648	-3.249001	0.005588	0.034858	-2.913051	5.031616	3.909605	3.830814	4.155549	3.736505	3.812601	5.052933	4.803005	4.875924	4.885482	4.650242	5.316091	4.944745	5.321296
Rab3c	-1.159902	3.864572	-3.248772	0.00559	0.034858	-2.923582	4.474867	3.418258	3.933744	5.268492	3.574163	3.939526	3.496648	3.749088	3.570447	2.934159	4.099353	3.594258	4.855682	3.310901
Nop2	0.585349	4.342035	3.248581	0.005593	0.034858	-2.93727	4.123692	4.861671	5.032356	4.575022	5.214297	4.647132	4.096591	4.166665	4.121609	3.973412	4.216089	3.794816	3.974722	4.070176
Epha7	-1.523239	6.998517	-3.247599	0.005604	0.034902	-3.045149	6.70816	4.71337	6.022889	7.821276	5.111109	7.096482	7.394967	7.856871	7.691093	7.586878	7.417985	7.669942	7.477713	7.427183
Rab40c	-0.527937	3.413423	-3.247447	0.005605	0.034902	-2.778588	3.676936	3.184227	3.151703	3.50989	2.910256	3.02233	3.270902	3.484018	3.722643	3.559779	3.53126	3.61312	3.614885	3.685908
Scamp5	-0.782334	7.227783	-3.247194	0.005608	0.034902	-3.050633	7.064105	6.081102	6.850152	7.654087	6.531294	6.839574	7.119048	7.589415	7.661858	7.293691	7.480889	7.531386	7.908257	7.595333
St6gal1	-0.598844	4.892932	-3.246612	0.005615	0.034925	-3.00052	5.674195	4.655425	4.566831	5.105531	5.066493	4.86573	4.822272	4.515458	4.800172	4.850841	5.012752	4.872617	4.757867	4.988458
Itgb3	1.582407	0.184034	3.245448	0.005628	0.034989	-2.156715	-0.760787	0.185409	1.143363	-0.166914	1.312595	1.378286	-0.169545	0.137473	-0.504027	0.20947	0.676952	1.11804	-0.305233	-0.204094
Rims2	-0.586033	6.647735	-3.243165	0.005654	0.035133	-3.06011	6.965356	6.866747	6.562677	7.13443	6.063258	6.366185	6.905857	6.317504	6.770144	6.450752	6.430993	6.560008	7.015523	6.674867
Rhoh	1.242451	0.457942	3.242026	0.005667	0.035195	-2.195909	-0.046857	1.010791	0.914692	-0.243853	1.390206	0.772673	0.282279	1.279465	-0.27702	0.591587	0.805871	1.404538	-0.305233	0.045082
Psmg1	0.498215	4.895074	3.240327	0.005687	0.035298	-2.984028	4.768549	5.220523	5.021611	4.577819	5.463549	4.971961	4.693279	4.792678	4.887683	4.800297	4.942039	4.706916	4.942203	4.795073
Kbtbd11	-0.971029	5.815378	-3.238998	0.005702	0.035357	-3.051442	5.907455	4.760857	5.722481	6.651519	4.859217	5.878525	5.868875	6.146914	6.052497	5.649254	6.179588	6.051545	6.090872	5.625434
Fam64a	3.265571	-0.904243	3.238992	0.005702	0.035357	-2.082074	-1.22354	0.895377	0.049949	-2.040259	2.575715	1.496914	-2.558588	-1.196868	-0.27702	-0.961779	-0.731011	-1.073407	-2.232431	-1.406952
Mfsd4	-0.579008	4.564438	-3.236238	0.005734	0.035536	-2.914877	4.231267	3.649562	4.061636	4.424653	3.359399	3.922669	4.962019	5.091219	5.190313	4.876899	4.894507	4.980656	5.180889	5.150119
Cdc42ep4	-0.650825	6.34234	-3.23585	0.005738	0.035546	-3.071434	7.110492	6.29924	5.922492	6.244702	6.076036	5.811428	6.44542	6.295728	6.299172	6.553062	6.403241	6.65787	6.393172	6.300566
Mettl7a	-0.829142	5.966339	-3.234573	0.005753	0.035603	-3.051406	6.248988	5.581085	4.620039	5.660292	5.501934	4.823218	6.043529	6.394648	6.164279	6.629987	6.333662	6.729486	6.315735	6.51022
Myt1l	-0.919609	6.898653	-3.2343	0.005756	0.035603	-3.075876	6.979002	6.168628	6.654219	7.735256	6.116562	6.805497	6.946967	7.31956	7.057883	6.523123	7.347808	6.809346	7.410785	6.72036
Rfx3	-1.418948	4.235069	-3.234283	0.005757	0.035603	-2.906793	3.788223	2.519311	3.89894	5.542091	2.81965	3.756629	5.105949	4.761243	4.983948	4.298786	4.670138	4.241218	4.539769	4.465266
Sik3	-0.467079	7.977082	-3.233969	0.00576	0.035608	-3.057741	8.082628	7.774483	7.916768	8.643069	7.892671	7.993715	7.803741	8.009222	7.916613	7.875285	7.887039	7.950449	8.007968	7.931765
Ddo	-0.630249	3.281223	-3.233297	0.005768	0.03561	-2.736491	3.334978	2.279532	2.654608	3.182467	2.672087	2.889836	3.624549	3.741948	3.662308	3.728285	3.848089	3.475597	3.665088	3.35002
Cpne4	-1.826069	7.528759	-3.233226	0.005769	0.03561	-3.066118	8.000165	4.522355	6.528929	8.412593	7.165999	7.293329	8.020537	8.068568	7.771042	7.518171	8.200924	7.841369	8.555895	7.514998
Cmtm4	-1.068919	4.030496	-3.233178	0.005769	0.03561	-2.936396	4.243653	3.200527	4.242177	5.111335	3.144791	3.839794	4.0114	4.12885	4.081214	3.456042	4.03505	4.158035	4.839377	4.037
Nfe2	3.379407	-2.203414	3.232677	0.005775	0.035627	-2.085981	-2.659044	-0.950476	1.340699	-2.344749	0.952152	-0.862697	-2.558588	-2.076466	-2.604521	-1.564464	-1.978322	-2.249472	-2.656794	-2.653844
Tmem86a	0.732334	3.927811	3.232065	0.005782	0.035643	-2.849639	3.891537	4.10842	4.096187	3.027403	4.377679	4.090548	3.137884	4.685117	3.495578	4.456866	4.302392	4.735888	3.143247	3.551406
Celsr2	-0.793659	8.692131	-3.231952	0.005784	0.035643	-3.056098	8.297122	7.234907	8.097537	9.023932	7.944511	8.188523	9.202003	9.177996	9.277526	8.711016	9.00516	9.030975	9.353961	9.148915
Ppt1	-0.563541	6.455485	-3.230829	0.005797	0.035694	-3.077661	6.469781	5.645011	5.801032	6.349085	6.026258	5.910938	6.264573	6.828745	6.662174	6.913478	6.709616	7.132232	6.827873	6.854948
Camk2b	-0.955099	10.81631	-3.230731	0.005798	0.035694	-2.978043	10.88185	9.180984	10.44267	11.23848	10.16566	10.64358	11.04793	11.30144	11.07043	11.08067	11.23347	11.08127	11.16897	10.89196
Kif3c	-0.670676	7.443347	-3.230481	0.005801	0.035694	-3.077298	7.667776	6.723232	7.185522	8.048497	7.273168	7.567566	7.433683	7.50284	7.404355	7.270398	7.556897	7.417078	7.835797	7.329277
Creld2	0.805751	4.376215	3.228429	0.005825	0.035824	-2.912958	3.992844	4.428602	4.089343	3.606198	5.128199	4.741469	4.527267	4.433629	4.37654	4.653926	4.302392	4.36136	4.195746	4.5074
Chst14	0.997286	1.862262	3.226819	0.005844	0.035913	-2.469304	1.717752	2.167912	2.08155	1.000693	2.883666	2.131561	1.544223	2.307359	1.549949	2.184529	1.864385	2.220493	1.28461	1.582867
Kcnab1	-0.98367	6.574296	-3.226702	0.005845	0.035913	-3.090565	6.745655	5.108376	6.060994	7.250299	6.308697	6.573431	6.908215	6.842161	6.749348	6.417603	6.96794	6.528893	6.989285	6.607052
Tle4	-0.58595	5.78587	-3.226074	0.005853	0.03594	-3.076846	5.889175	5.216521	5.448028	6.191819	5.349976	5.804544	5.754688	5.953414	5.741865	5.711719	6.050004	5.928941	6.218227	5.77234
Ephx1	-0.984846	4.819381	-3.224621	0.00587	0.036019	-2.989019	5.630846	3.354134	4.102999	4.267203	4.262012	4.056182	4.934514	5.22781	5.190313	5.418377	5.373295	5.183562	5.137097	5.396093
Klf12	-0.964044	2.837307	-3.224488	0.005872	0.036019	-2.822215	3.569521	2.822876	3.077557	3.959292	2.28197	3.061358	2.324585	3.159031	2.917273	1.986005	2.616315	2.404977	3.40196	2.372328
Fam181b	0.800204	4.217664	3.223867	0.005879	0.036027	-2.934707	4.243653	4.977341	4.397084	3.538889	4.757939	4.586885	4.145391	3.923301	3.689436	4.130887	3.776475	4.346409	4.495367	4.125333
Mypop	-0.417457	5.429648	-3.22383	0.00588	0.036027	-3.072314	5.781445	5.106215	5.386545	5.721019	5.446997	5.390404	5.395408	5.188961	5.495173	5.4065	5.493021	5.259352	5.533155	5.447498
Rab3ip	-0.680336	4.932664	-3.223615	0.005882	0.036027	-3.051373	5.152589	4.449211	4.703085	5.836471	4.843207	5.275609	4.871449	4.899205	4.710323	4.815347	5.075905	4.933912	4.916528	4.626888
Zfp251	-0.786239	4.800458	-3.222128	0.0059	0.036099	-3.035638	5.080769	3.884602	4.696344	5.504362	4.380852	5.049711	4.838851	4.927801	4.614504	4.824303	5.00382	4.761475	5.089637	4.607648
Asphd2	-0.723154	6.695599	-3.222128	0.0059	0.036099	-3.094357	6.552588	5.005446	5.832112	6.372633	6.032323	6.077257	7.252499	7.191115	7.342147	7.312377	7.060759	7.209145	7.19122	7.324375
Nradd	1.216261	0.711453	3.219975	0.005926	0.036233	-2.223224	0.243629	0.725555	0.750864	-0.705589	1.173177	0.846031	0.563361	1.239467	0.317129	1.569444	1.260496	1.667719	0.546019	0.799858
Dnal1	-0.853297	3.910712	-3.219737	0.005928	0.036233	-2.985724	4.563167	3.375827	4.123244	4.947973	3.596165	4.498643	3.647317	3.741948	3.669138	3.344264	3.858035	3.600573	4.294734	3.597647
Arl3	-0.471183	7.371106	-3.219534	0.005931	0.036233	-3.095443	7.937056	7.392635	7.285845	8.034566	7.714607	7.664539	7.173657	6.974453	7.319535	7.179682	7.250636	6.996959	7.224558	7.056413
Rpl29	0.853	6.694098	3.219051	0.005937	0.03625	-3.104404	7.468931	7.953452	7.620915	6.409999	8.297653	7.291689	5.896064	6.103009	6.011435	6.341669	6.203189	6.184645	5.863867	6.087944
Cnr1	-1.054783	8.090094	-3.215554	0.005979	0.036488	-3.09602	7.939914	6.558324	7.373317	8.967324	7.871684	7.785142	8.365348	8.162801	8.234681	8.078895	8.183231	8.208636	8.869654	8.668742
Pi4ka	-0.503183	8.495674	-3.215155	0.005983	0.036499	-3.086676	8.471581	8.007142	8.384616	8.886008	8.008976	8.300209	8.673327	8.707376	8.638181	8.43808	8.498463	8.518319	8.838376	8.573206
Trim59	-0.934691	3.342763	-3.214241	0.005995	0.036538	-2.844341	3.652372	2.631765	2.225837	3.861672	3.422332	3.091837	3.529702	3.024282	3.289407	3.463704	3.196995	3.822092	3.850062	3.901371
Mapre1	0.435864	6.489113	3.214129	0.005996	0.036538	-3.117304	6.615096	6.997269	6.894507	6.734709	7.256858	7.293329	6.024813	6.244866	5.945455	6.138858	6.175617	6.383585	6.142728	6.018012
Them6	-0.624655	5.408544	-3.21225	0.006019	0.036658	-3.065776	5.448478	4.167656	4.764524	5.26676	5.109198	4.870133	5.846094	5.595724	5.800582	5.692284	5.679851	5.894664	5.739059	5.8845
Metrnl	1.02942	1.719819	3.211049	0.006033	0.036729	-2.503772	1.717752	1.79116	2.498042	1.033666	2.564599	2.537984	1.258992	1.830204	1.458292	1.253084	1.533025	2.100868	1.54805	1.445134
Slc15a2	-0.649328	6.397537	-3.21037	0.006042	0.036761	-3.119508	6.770121	6.354002	5.569811	6.24734	5.766114	5.756599	6.516191	6.654346	6.330744	6.760914	6.680485	7.030085	6.565792	6.582626
Pak1	-0.621298	7.552753	-3.209613	0.006051	0.036787	-3.108723	7.886611	7.535609	7.730274	8.730981	7.686361	7.789506	7.040955	7.434734	7.23518	7.180263	7.49171	7.385429	7.448897	7.17062
Ntng2	-0.440241	5.528467	-3.209511	0.006052	0.036787	-3.060959	5.150398	4.710528	4.712023	5.138114	4.725866	4.672562	6.049239	6.072029	6.068084	6.140054	5.876155	6.165606	5.778076	6.177657
Camk2n1	-0.630268	8.304781	-3.209093	0.006057	0.036793	-3.090409	8.931336	8.505581	8.44802	9.041896	7.780045	8.690858	8.211837	7.943445	8.215855	8.034567	7.9938	8.061619	8.362653	8.050514
Srpk2	-0.558509	7.575668	-3.208935	0.006059	0.036793	-3.116454	7.771927	7.143018	7.455454	8.25942	7.453852	7.774612	7.47753	7.589415	7.535344	7.406819	7.592707	7.461987	7.72553	7.419848
Pygl	1.104081	2.554583	3.208183	0.006068	0.03683	-2.598693	2.091157	2.758277	3.426105	1.526009	2.828971	2.458346	2.361772	3.19084	2.166756	2.758411	2.639567	3.516245	2.097585	2.228598
Ctsk	0.970231	4.128749	3.207132	0.006081	0.03689	-2.854273	3.84352	3.631641	4.354903	2.475838	4.039207	4.294309	4.287446	4.511265	4.277224	4.475935	4.383823	4.465359	4.208498	4.652149
Gimap9	1.441132	0.275789	3.206622	0.006088	0.036909	-2.213479	-0.310971	0.679782	0.353965	-0.81875	1.082564	0.915838	0.357848	0.301855	1.102347	0.055728	0.071497	0.460309	0.037347	0.929479
Ikzf1	1.030607	2.559723	3.204	0.00612	0.037087	-2.71282	2.832262	2.843782	3.431527	1.874319	3.339971	3.722947	1.297885	2.988532	1.717448	2.543858	2.651054	3.397965	1.859646	1.634478
Polh	0.630896	3.086043	3.202773	0.006135	0.03716	-2.771832	2.8647	3.42521	3.463639	2.591079	3.280067	3.214637	3.241276	3.126505	3.31576	3.030171	3.074378	2.980434	2.676906	3.106782
Rpl22	0.712216	4.215748	3.202492	0.006139	0.037162	-2.978597	3.987945	5.028455	4.266652	3.843191	4.424547	4.795709	4.248266	4.17729	4.180157	4.425821	4.148808	4.083352	3.817137	3.67978
Cd22	2.067964	2.967739	3.201553	0.00615	0.037214	-2.456254	0.428583	3.432128	2.778919	1.273392	2.702832	2.705027	1.809437	5.151733	1.636128	4.712982	4.992577	6.235236	1.79348	2.228598
Stac2	0.599268	6.034475	3.200542	0.006163	0.037271	-3.142233	6.416074	7.377481	6.759287	6.363746	7.033694	6.782908	5.259158	5.333708	5.218522	5.824622	5.530819	5.917605	5.206541	5.484884
Sptbn1	-0.524954	10.37423	-3.199311	0.006178	0.037346	-3.05301	10.38129	10.06336	10.04197	10.77751	9.795963	10.31022	10.65514	10.48464	10.6355	10.23372	10.12133	10.42617	10.65908	10.65452
Clgn	-0.977156	3.885028	-3.197989	0.006195	0.037427	-2.883468	3.31946	2.184396	2.607481	4.314927	3.452798	3.193712	4.791941	4.323155	4.62508	4.369831	4.116027	4.153748	4.463807	4.601177
Ptpn21	0.580198	3.332711	3.197322	0.006203	0.037458	-2.884608	3.536344	3.774588	3.926082	3.051752	3.696327	4.028875	3.180134	3.126505	2.74697	3.110461	3.302737	3.33876	2.881849	3.11589
RGD1304982	0.833131	3.003094	3.196642	0.006212	0.037485	-2.775753	3.082606	3.74155	3.277465	2.198954	3.352952	3.432728	2.967416	2.665313	2.653634	2.911926	3.280721	3.212521	2.494578	2.972849
Dnm1	-0.674917	10.78107	-3.196467	0.006214	0.037485	-3.044088	10.8432	9.820767	10.45878	11.2127	10.67405	10.46117	10.93492	11.0048	11.11296	10.71461	11.00032	10.69645	11.13632	10.86487
Hexim1	-0.455964	6.416906	-3.194967	0.006233	0.03758	-3.15579	6.735458	6.15719	6.387537	6.979111	6.390452	6.670654	6.238569	6.279794	6.357417	6.36436	6.502489	6.339905	6.30983	6.142438
Ptch1	-0.452151	4.991714	-3.194104	0.006243	0.037627	-3.113084	5.494118	5.171753	5.127296	5.400752	4.713338	4.971961	4.842144	4.747049	5.066451	4.696819	4.720073	4.880423	5.128176	4.973505
Khdrbs2	-1.051217	3.024707	-3.191029	0.006282	0.037842	-2.921105	3.453461	2.100024	3.006676	4.541025	3.252623	3.462191	2.644268	2.951874	3.059216	3.060806	3.116419	2.447635	2.805569	2.648193
Siva1	0.712164	2.788838	3.189338	0.006304	0.037953	-2.801094	2.742017	3.544937	3.070623	2.697792	3.751288	3.365711	2.550752	2.24812	2.680923	2.469694	2.761158	2.691938	2.186205	2.516844
Nudt22	0.796924	3.506555	3.187862	0.006323	0.03801	-2.819152	3.263791	3.838476	3.525794	2.310787	3.716556	3.158148	3.797364	3.542716	3.767885	3.759747	3.499841	3.56872	3.683472	3.803307
Nmnat2	-1.04237	5.379622	-3.187851	0.006323	0.03801	-3.140219	5.492389	4.350411	5.508281	6.484931	4.723369	5.190151	5.157085	5.772547	5.589995	5.029251	5.547818	5.399942	5.906227	5.202356
Sdha	-0.450493	8.207775	-3.187843	0.006323	0.03801	-3.138614	8.687757	8.010902	8.353891	8.795786	8.294293	8.508892	8.043248	7.97233	8.104059	7.828204	8.115918	7.959383	8.180763	8.058802
Sgtb	-0.735256	5.402085	-3.187269	0.00633	0.038023	-3.152233	5.899649	4.948678	5.536232	6.246461	5.26325	5.597091	5.164996	5.321786	5.22317	4.815347	5.452566	5.042261	5.878569	5.277328
Pros1	0.843181	4.286758	3.187181	0.006331	0.038023	-3.030537	4.502616	4.649501	4.600917	3.617117	4.912799	5.371895	3.577905	4.379449	3.577725	4.464524	4.295089	4.890765	3.40196	3.858608
Asb1	-0.867216	4.244582	-3.186505	0.00634	0.038056	-3.029459	4.55325	3.142653	3.879235	4.608236	3.746377	4.056182	4.451671	4.446862	4.263751	3.844356	4.627162	4.49955	5.073464	4.319155
Tfpi2	2.639842	-0.139189	3.186041	0.006346	0.038073	-2.244688	-0.518419	0.480615	2.761803	-1.075778	1.643636	1.519512	-0.279766	0.58326	-0.773548	-0.311823	-0.855515	0.34469	-2.232431	-0.886923
Gusb	0.799283	3.727342	3.185574	0.006352	0.03809	-2.88238	3.279917	3.914553	3.855228	2.925676	4.109733	3.678453	3.454237	4.303891	3.140097	4.178226	3.999546	4.870006	3.116435	3.482664
Bcan	-0.603713	8.970983	-3.1853	0.006356	0.038092	-3.137746	9.27681	8.644239	8.148637	8.67738	8.41931	8.285657	9.345964	9.254147	9.184358	9.265563	9.000958	9.508282	9.138921	9.446856
Alg5	0.44989	4.528144	3.184751	0.006363	0.038097	-3.043994	4.15463	4.604273	4.67593	4.256768	4.698159	4.641992	4.535428	4.632039	4.57868	4.585342	4.421201	4.562362	4.326276	4.789392
Erbb2	1.67366	0.865949	3.184546	0.006365	0.038097	-2.351084	0.539902	2.184396	2.010869	-0.325128	0.808768	1.72793	0.743216	0.871929	0.245823	0.413143	0.998029	1.667719	0.249639	0.929479
RGD1564053	-0.644184	3.806517	-3.18449	0.006366	0.038097	-3.022064	4.105711	3.588941	3.98258	4.667211	3.639189	3.780216	3.749062	3.784265	3.716062	3.424979	3.882605	3.405197	4.121279	3.558103
Cbfa2t3	-0.582802	5.192474	-3.184111	0.006371	0.038107	-3.134035	5.509586	4.54806	4.720907	5.5248	5.12631	5.340508	5.167624	5.159792	5.216192	5.224545	5.099154	5.275228	5.467482	5.35822
Ccna2	3.035015	0.802833	3.182434	0.006392	0.038218	-2.368941	-0.760787	2.943995	1.430088	0.823477	4.006719	3.703341	-0.067151	-0.150641	-0.504027	0.475109	0.001431	0.666985	0.037347	-0.116166
Homez	-0.638753	1.962094	-3.181878	0.0064	0.038233	-2.758416	2.896425	2.134368	2.31067	2.797152	2.198134	2.203108	1.726334	1.958936	1.717448	1.064403	1.762161	1.461155	2.022549	1.634478
Gria1	-1.331426	8.633621	-3.181515	0.006404	0.038233	-3.156109	8.292907	6.226485	7.6782	9.309055	7.609004	8.362473	8.994396	9.352788	9.300526	8.919001	9.076115	9.171876	9.350849	9.232148
Prdm11	-1.04567	2.486607	-3.181504	0.006404	0.038233	-2.736755	2.832262	2.01041	2.726949	2.985885	1.230571	1.519512	3.200804	2.603631	2.822431	2.325762	2.782205	2.447635	3.169571	2.460763
Gpn2	0.453493	3.939071	3.180793	0.006414	0.038268	-2.991352	3.907194	4.309667	4.245259	3.622545	4.255114	4.03673	3.810873	3.910622	3.924502	3.759747	3.949256	3.794816	3.903318	3.820121
Mvb12b	-0.570193	6.593798	-3.179548	0.00643	0.038332	-3.186839	7.297848	6.353091	6.724924	6.984391	6.444513	6.76233	6.190288	6.599114	6.148482	6.436221	6.531522	6.780435	6.728825	6.347713

Thyn1	-0.515921	5.750223	-3.17948	0.006431	0.038332	-3.175774	6.326725	5.551208	5.563652	6.086747	5.9726	5.675909	5.740641	5.642482	5.61497	5.628953	6.036929	5.468867	5.621619	5.601319
Eif3m	0.421471	6.208265	3.178809	0.006439	0.038365	-3.185487	6.313077	6.593465	6.654219	6.441055	6.901335	7.044352	5.834566	5.807164	5.831353	5.929327	5.877378	5.945159	5.849014	5.916125
Serpine2	-0.520669	7.880378	-3.178479	0.006444	0.038372	-3.170444	8.484902	7.733763	7.630377	8.089257	7.743236	7.963386	7.881019	7.624778	7.872171	7.835605	7.532262	7.909392	7.819265	8.212636
Pttg1	3.12032	-0.38414	3.177883	0.006451	0.038399	-2.173481	-2.659044	-0.103798	-0.33572	-1.223791	1.877213	1.302125	-0.831021	-0.048075	-0.175642	-0.027758	0.001431	-0.070959	0.249639	-0.621773
Kcng1	-1.346522	5.961105	-3.177625	0.006455	0.0384	-3.171903	5.904858	3.96313	5.160327	7.07756	5.450021	5.986896	5.775507	6.084783	6.514513	6.077758	6.039116	6.206544	6.54918	6.694757
Pcyt1b	-0.697542	4.396745	-3.177367	0.006458	0.038401	-3.084144	4.716181	3.884602	4.166157	4.906296	4.023054	4.383247	4.35355	4.477275	4.334197	4.173561	4.650242	4.216763	5.082728	4.262873
Tmem25	-0.4158	6.442861	-3.177116	0.006461	0.038401	-3.183962	6.230438	5.941398	6.074054	6.387058	5.911395	5.647977	6.740997	6.682587	6.829268	6.789395	6.581154	6.719366	6.770984	6.91292
Ralyl	-0.592514	5.151459	-3.175462	0.006483	0.038503	-3.16599	5.523197	4.846214	5.197401	6.027618	5.437888	5.255556	5.100459	4.892772	5.192685	4.908123	5.136409	4.659314	5.159159	4.828697
Dhx37	0.825426	5.400255	3.175303	0.006485	0.038503	-3.122673	4.873231	5.972579	5.851456	4.555288	5.33042	4.988255	5.777228	5.541244	5.675579	5.659299	5.210093	5.472315	5.163531	5.571839
Six4	-1.191293	1.508328	-3.175071	0.006488	0.038503	-2.640634	1.895723	0.533043	0.975347	2.934433	1.947363	1.627481	1.607516	1.628188	1.579251	1.28803	1.456531	1.315214	1.983515	0.929479
Prss35	-1.14486	3.596449	-3.17423	0.006499	0.038549	-2.993514	3.658553	2.466372	2.869592	4.91074	3.293596	3.997021	3.356296	3.653399	3.83015	3.493653	3.981461	3.555779	3.91892	3.503633
Rims1	-0.580402	8.474415	-3.173987	0.006502	0.038549	-3.161569	8.953183	8.541215	8.454027	8.902559	7.89794	8.497554	8.639185	8.133433	8.385108	8.208246	8.292015	8.398405	8.859078	8.484359
Lmbrd1	-0.692251	7.196345	-3.172719	0.006519	0.038599	-3.198791	7.198487	6.174829	6.635548	7.590413	6.78283	7.21285	7.570015	7.032659	7.59887	7.128781	7.073621	7.280691	7.579385	7.901144
Ankrd13a	0.430344	6.913421	3.172719	0.006519	0.038599	-3.199825	6.947169	7.377481	7.129494	6.691456	7.227203	7.261423	6.645274	6.956831	6.550097	7.029021	6.772101	7.169875	6.425389	6.618311
Il21r	1.557339	1.393484	3.172613	0.00652	0.038599	-2.433801	0.592497	1.425638	1.493674	0.386344	2.542105	2.481552	0.498056	2.505864	1.01926	2.108365	1.507975	2.346065	0.313987	0.970221
Kat3	-0.713813	3.344085	-3.171359	0.006537	0.038678	-2.986115	3.701089	2.874583	3.240874	4.298064	3.458815	3.601103	3.383673	2.939445	3.59934	2.956054	3.373787	2.63155	3.576053	3.34228
Gabra2	-0.924613	7.456117	-3.170582	0.006547	0.03872	-3.20144	7.004094	5.893325	6.640823	8.036855	6.666117	7.173314	7.845771	7.890351	8.118478	7.598235	7.680102	7.678922	8.088353	8.081061
Nrn1	-1.59299	7.115767	-3.169849	0.006557	0.038758	-3.194708	7.654309	4.861671	6.573753	8.502378	6.965074	7.527363	6.818276	7.138394	7.373325	7.097725	7.246856	7.211725	7.526053	7.137465
Slc35d3	-0.670022	2.299225	-3.167413	0.006589	0.03893	-2.781922	3.127854	2.117298	1.936547	2.546081	2.198134	2.410784	2.415815	1.883083	2.508863	2.165862	2.014226	1.805289	2.652228	2.733504
Nlrp1a	0.907649	2.280431	3.166779	0.006598	0.038961	-2.714607	2.144973	2.452828	3.138503	1.892408	3.129785	2.854719	2.01015	2.227821	2.166756	2.39151	2.332254	2.516041	1.077784	1.931282
Pcdhac2	-1.000508	4.308381	-3.163333	0.006644	0.039213	-3.034413	4.132599	2.383133	3.887149	4.577819	3.333437	3.756629	4.753981	4.747049	5.050846	4.246444	4.733642	4.594355	5.341939	4.872343
Rpl19	0.625537	6.852981	3.162756	0.006652	0.03924	-3.217246	6.743476	7.353538	6.831303	6.028641	7.11833	6.73483	6.968221	6.857933	6.891185	7.083498	6.873647	6.990361	6.676247	6.804442
Egln3	-0.614816	4.126559	-3.162156	0.00666	0.039268	-3.069257	4.350588	3.894655	3.474186	4.43703	4.143745	3.648009	4.134688	4.112335	4.055381	4.332655	4.111876	4.622553	4.398536	4.147707
Slc6a9	-0.696226	6.81472	-3.161341	0.006671	0.039314	-3.221955	6.982701	6.506823	5.866546	6.885693	6.259566	6.325083	6.71429	7.000862	6.963807	7.060312	6.766171	7.365637	7.208778	7.514568
Atf5	0.758368	6.174505	3.158408	0.00671	0.039528	-3.221951	6.065811	6.95466	6.215918	6.075905	6.931935	7.21155	5.658967	6.168376	5.788087	6.188254	5.730811	6.232189	5.302835	5.940678
Gins4	0.766242	2.56137	3.15809	0.006715	0.039534	-2.755555	2.213741	3.099845	2.423812	2.108996	3.238702	2.949304	2.266943	2.738882	2.524011	2.614395	2.494078	2.606669	2.508394	2.341603
Prpf38a	0.410541	4.85769	3.157576	0.006721	0.039555	-3.166924	4.862563	5.140412	5.404577	4.965167	5.421037	5.326158	4.707794	4.624294	4.663209	4.712982	4.675772	4.546096	4.398536	4.61409
Anapc15	0.577173	4.452011	3.157303	0.006725	0.039558	-3.114115	4.579546	5.234443	4.740698	4.04669	4.895159	4.667512	4.292269	4.254576	4.281688	4.433645	4.470659	4.387158	4.080242	4.037
Psat1	-0.579068	7.282839	-3.156564	0.006735	0.039597	-3.229557	7.719683	7.184581	6.646663	7.318365	7.077855	6.854649	7.205329	7.362672	7.203775	7.654973	7.262386	7.821909	7.320185	7.337086
Tmem255a	-0.844613	3.033943	-3.155223	0.006753	0.03967	-2.891527	2.799078	2.47979	2.770386	3.879919	2.348294	2.493017	3.105363	2.862532	3.518449	2.966878	3.181236	3.083214	3.459987	3.728093
Slc24a2	-1.363322	9.376024	-3.155177	0.006754	0.03967	-3.183619	9.105863	7.042617	8.806383	10.12431	8.270331	8.891118	9.75043	10.11017	9.836856	9.319342	9.970975	9.850098	10.37078	9.818073
Astn1	-0.737163	8.720681	-3.154413	0.006765	0.039697	-3.208621	8.300337	7.422907	7.983118	9.105915	8.190549	8.261351	9.362685	9.056466	9.196819	8.711619	8.990541	9.028928	9.172662	9.309724
Kctd7	-0.94223	2.308322	-3.154357	0.006765	0.039697	-2.789157	2.875353	1.082904	2.122353	2.853641	2.268331	2.18908	2.227194	2.268138	2.330799	1.920699	2.592682	2.691938	2.838755	2.387449
Pdyn	-2.328582	5.701112	-3.153853	0.006772	0.03971	-3.166133	5.550041	1.047298	5.197401	6.990702	4.361713	4.986228	6.173393	6.467876	6.498306	6.357179	6.78193	6.452405	6.285965	6.746982
Fdft1	-0.43884	6.522415	-3.153711	0.006774	0.03971	-3.237634	6.929388	6.389093	6.256198	6.730945	6.643183	6.278641	6.831591	5.985507	6.907161	6.130458	6.354891	6.178326	6.926356	6.789579
Pcdhga8	-1.098221	3.769386	-3.153152	0.006782	0.039735	-3.022941	3.707065	2.452828	3.12518	4.667211	2.828971	3.980827	3.920861	4.592894	3.866259	4.07195	3.678847	3.869926	4.152409	3.983274
Glrb	-0.885186	6.147506	-3.152031	0.006797	0.039802	-3.224755	5.870661	4.544872	5.392117	6.600164	5.655207	5.798782	6.377821	6.418354	6.71758	6.211195	6.41508	6.504687	6.831312	6.752102
Szrd1	0.485431	6.502309	3.151839	0.0068	0.039802	-3.240079	6.611915	6.829239	6.778562	6.383065	7.252535	7.06802	6.023363	6.344858	5.882203	6.513004	6.425151	6.555139	6.104581	6.27843
Pcbp3	0.57954	6.458252	3.151595	0.006803	0.039802	-3.240794	6.547601	6.800788	7.546034	6.659463	7.090505	7.29783	5.988123	6.118252	6.128798	6.032107	6.302134	5.767143	6.157051	5.998448
Mospd1	-1.306228	0.447423	-3.151264	0.006808	0.039805	-2.438571	1.717752	-0.572649	0.25957	0.577356	0.091273	-0.291112	1.000645	0.76329	0.628769	0.475109	1.199219	0.007173	0.791767	0.844371
LOC680039	-0.527394	7.512907	-3.15107	0.00681	0.039805	-3.23631	7.522859	7.061703	7.38873	8.004216	7.11928	7.373049	7.569022	7.630105	7.454139	7.428027	7.676593	7.652194	7.865957	7.443552
Cib1	1.010477	4.186893	3.149895	0.006827	0.039881	-3.031694	4.22295	5.02159	4.290718	2.835056	4.667313	4.06389	3.756062	4.485848	3.606474	4.683756	4.50588	4.594355	3.882251	4.093415
Abat	-0.741224	6.369532	-3.148996	0.006839	0.039921	-3.245639	6.955977	6.297347	6.440264	6.755242	5.837122	5.885066	6.297333	6.405985	6.146036	6.079006	6.428492	6.527236	6.99543	6.142438
Tmem156	1.634338	0.626722	3.148797	0.006842	0.039921	-2.297854	-0.899176	0.854759	0.484899	-0.705589	1.390206	0.117189	0.625837	2.16515	0.007959	2.108365	1.959843	2.433555	-0.512812	0.045082
Gnrhr	-2.787181	0.445705	-3.148674	0.006843	0.039921	-2.395892	1.071521	-3.260591	0.158563	2.108996	-0.424712	0.65513	0.498056	1.155984	1.180909	0.413143	1.260496	1.219993	1.859646	1.227132
Hspa12b	1.051975	4.145435	3.148433	0.006847	0.039921	-3.020055	3.92781	4.931692	4.354903	2.934433	4.5954	3.964448	4.641297	3.832115	4.347031	4.535316	3.728487	3.990233	3.882251	4.465266
Itga11	-1.077039	3.200793	-3.147601	0.006858	0.03995	-2.824168	2.569081	2.747223	1.574317	3.437817	1.912714	1.72793	3.654827	3.777297	4.281688	4.178226	3.73929	3.990233	3.508915	3.927469
Trabd	0.487277	5.006449	3.147588	0.006868	0.03995	-3.165912	4.771404	5.230479	5.133963	4.529512	5.290494	4.883261	5.081078	4.937208	5.144489	5.215497	4.988055	4.968501	4.831156	5.13452
Cars	0.37393	6.050562	3.146921	0.006868	0.039985	-3.24224	5.913928	6.310548	6.483321	6.07095	6.315345	6.356018	5.787514	5.936314	5.908415	5.952657	5.896807	5.888226	5.820225	6.091411
Scg5	-0.716286	8.916514	-3.146513	0.006873	0.039998	-3.210474	8.91255	7.887025	8.347273	9.372251	8.66939	8.799735	8.920771	8.93377	9.27291	9.220906	8.918258	9.054453	9.120907	9.404399
Dennd5a	-0.459182	9.203734	-3.145858	0.006882	0.040032	-3.200249	9.551184	9.083006	8.846639	9.255139	9.014695	8.833524	9.376236	9.171035	9.184358	9.337961	9.042301	9.418206	9.311989	9.428699
Ppp1r14c	-0.752507	4.190133	-3.145127	0.006893	0.040071	-3.135154	4.410408	3.404252	3.875261	5.027879	4.169655	4.428729	4.527267	3.991112	4.06577	4.130887	4.152854	3.961084	4.453131	4.152141
Fezf2	-0.567967	5.543895	-3.144644	0.006899	0.040091	-3.242373	5.772924	5.535232	5.303227	6.505644	5.723019	5.737448	5.538282	5.143629	5.395739	5.412451	5.309487	5.39813	5.449691	5.423859
Vamp5	1.632737	0.237936	3.14378	0.006911	0.040142	-2.342848	0.307952	0.895377	1.340699	-1.223791	1.285769	0.435061	0.282279	0.222004	-0.386063	0.646479	0.676952	0.760186	0.313987	-0.748265
Rnd1	0.727415	4.251478	3.143301	0.006918	0.040161	-3.132261	4.506048	4.926801	5.390726	3.897939	4.897375	4.443576	3.863678	3.777297	3.930199	4.041554	4.095154	3.827486	4.13915	3.869418
Gsdmd	0.541399	3.573532	3.142768	0.006925	0.040185	-3.018571	3.707065	4.163505	3.830814	3.197095	3.909025	4.028875	3.003159	3.683523	3.059216	3.64312	3.967746	3.869926	3.070609	3.031778
Rapgef2	-0.658057	7.353985	-3.142324	0.006932	0.040202	-3.256769	7.353195	6.631533	7.206627	7.850775	6.754983	7.180422	7.458034	7.628654	7.449187	7.218128	7.657499	7.416183	7.843992	7.316496
Eps8l2	-1.27761	3.139559	-3.141904	0.006938	0.040216	-2.983737	4.516294	1.725091	3.077557	3.348912	2.630048	3.038068	3.137884	2.753156	3.169295	3.432808	4.144751	2.805622	3.107385	3.262515
Psmd5	0.420865	4.94587	3.140514	0.006957	0.040301	-3.186503	4.785591	5.121276	5.378145	4.693241	5.101527	5.026231	4.946803	4.934079	4.872969	4.856672	5.034842	4.816761	4.967428	4.757741
Sprn	-1.055945	4.169474	-3.14038	0.006959	0.040301	-3.102629	4.210384	2.47979	3.772191	4.872521	3.391209	4.261168	4.182238	4.564846	4.589521	4.173561	4.518478	4.376159	4.754973	4.323092
Micu3	-0.955665	5.420898	-3.13912	0.006977	0.040384	-3.23881	5.488925	4.379337	5.186218	6.467691	4.93239	5.587765	5.692193	5.473892	5.562747	4.938684	5.697961	5.283102	5.83126	5.409117
Trim25	1.198919	2.268153	3.138574	0.006984	0.040398	-2.799904	2.753611	3.294639	3.545931	1.478901	2.692656	3.49677	1.862293	2.099634	1.458292	1.73055	1.697158	2.910998	1.436856	1.659607
Nrbf2	-0.922917	1.328394	-3.13847	0.006986	0.040398	-2.608958	1.764345	0.04804	1.245402	1.818649	1.143601	1.07687	1.443753	1.318383	1.769222	1.451089	1.557647	1.488652	1.651284	1.473753
Syp	-0.901076	9.238907	-3.138163	0.00699	0.0404	-3.222303	9.131639	7.542019	8.750266	9.545146	8.624596	8.840552	9.423306	9.512087	9.854565	9.535503	9.352511	9.532428	9.78067	9.922327
Krcc1	0.510848	5.181915	3.137975	0.006993	0.0404	-3.236437	5.457367	5.659822	5.679833	5.273677	6.184399	5.971611	4.685967	4.747049	4.511866	5.14338	5.008293	5.116845	4.512607	4.639574
Ctrc	1.784663	1.21979	3.137622	0.006998	0.040409	-2.423016	0.105685	2.655608	1.705429	-0.166914	1.664612	0.734546	1.697535	1.532126	1.218642	1.828755	1.102128	1.461155	0.879431	1.415936
Eci3	1.428432	1.666493	3.137107	0.007005	0.040432	-2.641493	2.357438	2.506257	2.250588	0.22441	2.892584	2.82781	1.17791	2.077116	1.426399	1.569444	1.82436	1.11804	0.598632	1.048408
Gfpt1	-0.613758	5.137159	-3.13661	0.007012	0.040453	-3.222081	5.348806	4.456016	5.0952	5.527697	4.693063	5.043877	5.256692	5.307349	5.192685	4.896847	5.227461	5.225023	5.624793	5.070381
Ftl1	0.915636	3.772737	3.135615	0.007026	0.040515	-2.847881	2.706665	3.479652	3.347976	2.045748	3.352952	2.889836	4.390007	4.548571	3.980484	5.036976	4.609607	4.948838	3.822677	3.797658
Gpr158	-1.042805	7.679889	-3.134802	0.007038	0.040562	-3.26903	7.38056	5.735883	7.21018	8.224146	6.879634	7.204158	8.128993	8.149036	8.105948	7.874567	8.002233	8.005321	8.464059	8.162701
Gls	-0.527782	8.569614	-3.133917	0.00705	0.040615	-3.251419	8.3321	8.025271	7.776279	8.805961	8.288603	8.06626	9.168455	8.677283	9.029766	8.737726	8.816249	8.489262	8.886448	8.879263
Rrm2	2.469979	-0.489885	3.133591	0.007055	0.040622	-2.299476	-1.417928	0.118359	0.442561	-0.941548	1.997824	1.72793	-0.067151	-0.81357	-0.773548	-0.537962	-0.855515	0.007173	-1.218554	-1.899685
Epm2aip1	-0.692345	7.428944	-3.13221	0.007075	0.040716	-3.272589	7.529619	6.686815	7.187927	8.198703	7.256426	7.552921	7.602419	7.195695	7.42921	7.190689	7.422181	7.384971	7.852141	7.524861
Bop1	0.464405	5.786029	3.131604	0.007083	0.040717	-3.246828	5.488925	5.671563	5.943517	5.175528	5.99658	5.559419	5.908683	5.89668	5.912737	5.880073	6.023735	5.805913	5.795084	5.974866
Cntn2	-0.679627	7.243689	-3.131579	0.007084	0.040717	-3.281506	7.184099	7.025563	6.27517	7.257732	6.622561	6.246828	7.579418	7.611129	7.512741	7.469528	7.178549	7.897902	7.836824	7.724864
Grm5	-1.04023	6.32442	-3.131494	0.007085	0.040717	-3.270824	6.005266	4.897106	5.635887	7.022876	5.198203	6.157795	6.609005	6.994117	6.780431	6.168464	6.687476	6.654839	7.148847	6.603819
Osm	2.582335	-0.859497	3.130523	0.007099	0.040777	-2.255988	-1.417928	-0.572649	1.613009	-2.344749	-0.18071	-0.072565	-1.009457	0.301855	-1.536754	-0.210791	-0.231835	-0.657145	-1.218554	-1.212031
Crebl2	-0.437947	6.377169	-3.129814	0.007109	0.040816	-3.282771	6.535899	5.941398	6.110004	6.699183	6.274994	6.393205	6.617678	6.206479	6.328589	6.410682	6.370609	6.381753	6.499029	6.529943
Msl1	-0.358739	7.381397	-3.12942	0.007115	0.040829	-3.279982	7.615917	7.181505	7.309596	7.740882	7.37093	7.416969	7.446721	7.350401	7.282714	7.256129	7.240217	7.360065	7.334313	7.442648
Cyp46a1	-0.422012	8.505168	-3.128602	0.007126	0.040877	-3.263428	8.596144	7.976116	8.215184	8.372679	8.104657	7.959259	8.570364	8.722418	8.750715	8.907459	8.620162	8.810553	8.618145	8.852952
Il1rap	-0.621167	5.709108	-3.12754	0.007142	0.040942	-3.253983	5.313991	4.929249	5.323783	6.000772	4.970791	4.932509	6.064827	6.107182	6.316677	6.210057	5.708994	5.922654	5.976249	6.181999
Jph3	-0.530881	8.911967	-3.127079	0.007148	0.040942	-3.252946	9.15259	8.042328	8.536382	8.774455	8.492807	8.671439	9.267765	9.018563	9.039023	9.159572	9.025437	9.215085	9.243297	9.132181
Neto1	-1.103502	7.151603	-3.12683	0.007152	0.040942	-3.286957	7.185705	5.601176	6.686891	8.006293	6.81602	6.856315	7.326667	7.453431	7.41709	6.749206	7.603833	7.093021	8.043392	7.295608
Tshz3	-0.75703	3.336278	-3.126334	0.007159	0.040942	-3.159236	4.167689	4.060109	4.275724	5.042128	3.639189	3.468012	2.598267	2.522625	2.785194	2.807409	3.099749	2.794648	2.760099	2.858134
Ube2c	2.833511	-0.341053	3.126258	0.00716	0.040942	-2.313023	-1.052261	1.082904	-0.007587	-1.388744	2.448452	2.025031	-0.529223	-0.81357	-0.773548	-0.961779	-0.731011	0.28321	-1.218554	-0.748265
Mafk	0.537092	4.311261	3.12607	0.007163	0.040942	-3.135133	4.092076	4.563896	4.87411	3.893455	4.312723	4.336284	4.123906	4.346879	4.325577	4.468337	4.258009	4.482556	4.125768	4.233886
Vcam1	-0.611867	5.827623	-3.125881	0.007166	0.040942	-3.285654	6.345043	5.774504	5.731301	6.466182	5.522187	6.150574	5.509574	5.987017	5.317478	5.745113	5.530819	6.180436	5.548281	5.806491
Mark1	-0.483654	5.817653	-3.125793	0.007167	0.040942	-3.282595	6.014933	5.764945	5.828007	6.38626	5.402427	5.877432	5.953525	5.51426	5.868916	5.765405	5.757625	5.499602	5.976249	5.865727
Gas5	0.848361	5.156354	3.125682	0.007168	0.040942	-3.198561	4.751303	5.64203	5.401818	4.181527	5.473985	4.709886	5.67752	5.232912	5.460161	5.394523	4.932657	4.992709	4.929422	5.454694
Zbtb26	-0.878014	2.413203	-3.124486	0.007186	0.041022	-2.875573	2.595092	1.769471	1.890044	3.329127	2.046579	2.734303	2.659281	2.186344	2.430637	2.308845	2.729	2.082939	2.838755	2.489076
Rcan1	0.467918	5.946848	3.123644	0.007198	0.041056	-3.283704	5.980811	6.108354	6.404868	5.634999	6.229088	6.350514	5.686708	6.060597	5.598964	5.995574	6.105332	5.790806	5.862523	5.472529
Adap2	0.943871	2.970449	3.123314	0.007203	0.041056	-2.801702	2.474162	3.099845	2.588191	1.571628	3.011968	3.045873	2.893159	3.591194	3.178898	3.7783	3.273307	3.859433	2.549064	2.890335
Wdr35l	-0.409733	5.41963	-3.123288	0.007203	0.041056	-3.274217	5.705873	5.315253	5.273343	5.87678	5.346735	5.597091	5.424258	5.347885	5.422258	5.136206	5.432737	5.164508	5.497232	5.371589
Pvalb	-0.644607	6.000382	-3.122967	0.007208	0.041056	-3.298178	7.009544	6.223497	6.044281	6.811582	6.655023	6.144226	6.09964	5.438999	6.148482	5.374339	5.612409	5.423285	5.659259	5.386717
Qdpr	-0.703652	7.552598	-3.122891	0.007209	0.041056	-3.291998	7.862386	6.971973	6.540894	7.76716	7.691799	7.239895	7.598043	7.585432	7.658001	7.755936	7.677296	7.91447	7.825142	7.656684
Ifit3	2.084548	2.314778	3.122263	0.007218	0.041089	-2.698062	1.741237	3.769134	3.99728	0.896996	2.892584	2.705027	1.986536	3.036005	1.743567	2.39151	1.581856	3.859433	1.040235	1.158276
Pcdh9	-0.758473	6.976011	-3.120938	0.007237	0.041168	-3.299647	7.255153	6.177919	6.939442	7.883362	6.905196	7.215881	6.884455	6.894612	6.858693	6.611999	6.781277	6.932719	7.262248	7.074043
Fam185a	-0.708793	4.363889	-3.120837	0.007239	0.041168	-3.19043	4.488808	3.479652	4.044046	5.038072	4.234219	4.46411	4.407896	4.490115	4.481148	4.205901	4.638748	4.208518	4.522853	4.468825
Cacng2	-0.892596	5.433632	-3.120386	0.007245	0.041186	-3.295619	6.151567	5.294522	5.667249	6.849113	5.214297	6.255268	4.949859	5.173124	4.930947	4.763521	5.0566	4.978233	5.766625	5.058621
Fa2h	-0.983764	5.861895	-3.119255	0.007262	0.041261	-3.292506	6.409676	5.713341	4.755906	6.349903	6.10893	5.0341	5.590126	5.97793	5.754765	5.71814	5.778723	6.332336	6.216124	6.355423
Egfl6	0.799289	3.514282	3.117237	0.007292	0.04141	-3.039476	3.788223	4.117033	3.95649	2.677071	4.209453	3.727807	3.337752	3.19084	3.570447	3.493953	2.958968	3.065063	3.515771	3.73402
Ppfia3	-0.365606	8.398254	-3.116611	0.007301	0.041442	-3.281916	8.77087	8.343476	8.286485	8.52683	8.220295	8.286069	8.531115	8.34759	8.43755	8.463621	8.259318	8.271823	8.434288	8.4009
Sf3b4	0.443778	6.291417	3.116167	0.007307	0.041456	-3.311266	6.629326	6.769718	7.172224	6.490137	7.14423	6.926695	5.617289	6.024276	5.948265	5.901388	6.10115	5.898513	5.816065	5.661644
Gpr64	-1.015649	1.809759	-3.115983	0.00731	0.041456	-2.725025	2.054131	0.812964	1.220561	2.324175	1.600747	1.165512	2.186319	1.908813	1.690848	1.73055	1.629088	2.668085	2.480628	2.341603
Kcnd3	-0.778833	6.542005	-3.115494	0.007317	0.041477	-3.310127	6.217938	5.39	6.32773	7.214799	5.987244	6.043544	6.918391	6.524103	6.930798	6.592037	6.497725	6.650281	7.170189	7.141372
Lxn	0.719346	5.890556	3.115089	0.007323	0.041492	-3.31094	6.189409	6.80679	6.462946	6.440286	7.293089	7.572303	5.058606	5.273089	5.056067	5.519255	5.152662	5.423285	5.078103	5.172115
Slc28a2	1.463363	4.90217	3.112935	0.007355	0.041632	-3.123346	3.595524	5.989094	5.427823	3.567317	4.424547	4.333098	5.713926	5.030987	5.368723	5.585718	4.820137	4.980656	4.539769	5.313401
Sulf2	-0.479677	8.703921	-3.112747	0.007358	0.041632	-3.286832	8.630742	8.118559	8.322498	8.917266	8.522373	8.211909	8.834738	8.927884	8.88566	8.69605	8.669004	9.040006	9.074935	9.007134
Rcsd1	0.617385	3.59411	3.11271	0.007358	0.041632	-3.073012	3.589067	3.996186	4.019053	3.225913	3.761059	4.271191	3.083269	3.559057	3.225976	3.994723	3.53126	3.859433	2.954296	3.380568
Fbxl17	-0.722751	5.406955	-3.110925	0.007385	0.041763	-3.299955	5.777191	4.972603	5.364037	6.162305	4.863759	5.786023	5.246787	5.434577	5.360307	4.984678	5.445986	5.390862	5.707966	5.238245
Tpt1	0.511147	8.47511	3.110607	0.00739	0.04177	-3.285201	8.754734	9.260634	8.790119	8.485905	9.315867	9.156738	8.178031	7.978418	8.110346	8.326685	8.237339	8.161098	7.861927	8.038291
Slco4a1	1.063687	2.457254	3.109784	0.007402	0.04182	-2.834273	2.230432	3.696289	3.145118	1.818649	2.874693	2.581593	2.582602	2.471744	2.55384	2.39151	2.014226	2.404977	1.626159	2.310209
Gbp5	1.273888	2.806578	3.10932	0.007409	0.041823	-2.871784	2.43149	3.091129	3.855228	1.910273	3.091571	3.568964	2.48486	3.413873	2.241976	2.614395	2.833517	4.074306	1.700256	2.228598
Cyp20a1	0.544557	4.458426	3.109273	0.00741	0.041823	-3.214544	4.495729	4.774532	4.725329	4.242735	4.870545	5.262271	4.192594	4.101219	4.303799	4.382013	4.180861	4.555878	4.107729	4.295302
Nme4	1.050592	2.006015	3.1073	0.007439	0.04197	-2.727973	1.717752	2.833367	2.298852	1.245516	2.619344	2.28453	2.398025	2.121805	2.066858	2.108365	1.533025	1.515634	1.377871	2.372328
Pde4dip	-0.547443	7.989536	-3.106337	0.007454	0.042032	-3.315385	7.930048	7.625502	7.745591	8.506605	7.423793	7.883699	8.255097	8.196487	8.133062	7.822256	8.031368	7.957846	8.334331	8.014139
Fgfr3	0.56731	6.965985	3.105287	0.007469	0.042102	-3.332807	7.082034	7.742487	7.221571	6.541908	7.136743	7.410182	7.025106	6.529296	6.902097	6.677711	6.496133	6.824935	6.85786	7.088573
Aldh3b1	-0.591378	4.824183	-3.104867	0.007476	0.042115	-3.23925	5.050539	3.953545	4.159638	4.711187	4.439837	4.586885	5.185883	5.173124	5.099692	5.339365	5.017197	4.936411	4.797792	5.145679
Hdac9	-0.912107	4.716776	-3.104663	0.007479	0.042115	-3.267274	5.262171	3.661386	4.438066	5.411768	4.635794	4.942996	4.571588	5.001397	4.560429	4.281549	5.117902	4.677349	5.116948	4.418187
Dpy19l3	-0.690547	7.74928	-3.103363	0.007499	0.042191	-3.331591	7.650825	6.458507	7.026665	7.86258	7.194223	7.586085	7.900893	8.155095	8.017398	7.974826	8.105518	8.179933	8.205092	8.180172
Ccdc86	0.77873	4.194926	3.103152	0.007502	0.042191	-3.194338	4.53321	4.892097	5.071085	3.572936	5.028566	4.254448	3.96382	4.084383	3.742206	4.000002	4.056804	3.995035	3.866246	3.757487
Ppp2r2c	-0.747993	7.480512	-3.102794	0.007507	0.042191	-3.329007	7.931006	6.925538	7.477901	8.013801	6.832912	7.665173	7.468726	7.51807	7.311919	7.170347	7.557661	7.517688	8.011307	7.334163
Grip1	-0.613031	5.126086	-3.10278	0.007507	0.042191	-3.291313	5.026846	4.88455	5.026994	5.908236	4.573386	4.959619	5.478169	4.971187	5.341191	5.03183	5.103341	4.923876	5.310741	5.271227
Ccdc34	-0.615931	3.517174	-3.102312	0.007514	0.042191	-3.127148	4.33909	3.287027	3.319014	3.649386	3.245679	3.652817	3.496648	3.321106	3.416608	3.204921	3.586147	3.46871	3.805994	3.61702
Icam5	-0.789556	9.124549	-3.102158	0.007517	0.042191	-3.312654	8.588476	7.209964	7.941682	8.807004	8.375653	8.109496	10.12107	9.767439	10.14174	9.869136	9.472791	9.630863	9.603142	10.10879
Gria2	-0.728442	9.98357	-3.102144	0.007517	0.042191	-3.266777	9.5606	8.952998	9.286898	10.54853	9.322481	9.731024	10.53094	10.24813	10.52766	10.1193	10.00123	10.03346	10.35689	10.55152

Clstn2	-1.273122	6.899315	-3.101335	0.007529	0.042229	-3.337879	6.589447	4.741493	6.100675	7.698459	5.963107	6.662428	7.253118	7.566361	7.37437	6.888078	7.347808	7.2539	7.91086	7.25651
Id4	-0.669757	5.450466	-3.101143	0.007532	0.042229	-3.327721	6.013728	5.097537	5.543762	6.329302	5.223162	6.13785	5.216656	5.191584	5.197417	5.357987	5.204257	5.381725	5.156968	5.291464
Ptges	2.829145	-0.159284	3.101006	0.007534	0.042229	-2.368518	0.428583	2.100024	2.200654	-2.344749	0.985877	0.386712	-1.450335	0.517885	-0.504027	-0.420466	-0.318799	-0.541385	-0.893866	0.045082
Dact2	-0.819128	4.334888	-3.10065	0.00754	0.042234	-3.255577	5.312032	4.907073	4.4568	4.814411	3.935155	3.698398	3.927077	4.009066	4.241012	4.535316	4.530966	4.183498	4.143583	4.074854
Ppfia2	-1.035983	7.049376	-3.100487	0.007542	0.042234	-3.340715	6.940844	5.445761	6.594445	7.90357	6.442237	7.051638	7.471387	7.438604	7.38944	7.218128	7.299812	7.114691	7.316263	7.077544
Gpd1	1.036926	7.445644	3.098306	0.007575	0.0424	-3.327524	7.743137	9.525731	8.952851	7.793871	8.82786	7.904507	6.217169	6.690026	6.307951	6.704635	6.724644	7.043473	7.18209	6.631866
Psmg4	0.681453	3.282206	3.097514	0.007587	0.042448	-2.986755	3.118917	3.570246	3.330668	2.401998	3.446756	3.340548	3.577905	3.19084	3.350165	3.689602	3.394437	3.18757	3.237505	3.278825
LOC100911069	1.304201	2.955172	3.096479	0.007603	0.042517	-2.822189	1.669604	3.833259	2.726949	1.910273	2.81965	3.030221	3.734959	2.888628	3.271566	3.595091	2.81321	3.092205	2.652228	3.558103
Klhl24	-0.718407	5.966391	-3.096168	0.007608	0.042524	-3.344485	6.120563	5.44747	5.482393	6.804434	5.721768	6.329885	6.073259	5.931615	5.880732	5.706885	5.921916	5.966095	6.258637	5.909593
Zfyve9	-0.670574	6.077478	-3.095241	0.007622	0.042584	-3.347288	6.125033	5.419891	5.851456	6.846216	5.957806	6.032783	6.260887	6.059162	6.10507	5.874336	6.152567	5.972195	6.532374	5.918729
Slc46a3	-0.670105	2.526127	-3.094182	0.007639	0.042647	-2.920777	2.832262	1.747452	2.322392	2.797152	2.28197	2.217002	2.48486	2.724465	2.462438	3.00938	2.506778	2.931181	2.480628	2.847239
Tapbpl	0.975442	3.975897	3.094047	0.007641	0.042647	-3.098279	4.092076	4.196386	3.948948	2.463791	4.158607	4.535145	3.51327	4.540363	3.627666	4.487257	4.366508	4.674359	3.36447	3.803307
Nqo1	0.532814	3.61348	3.09379	0.007645	0.042649	-3.151543	3.983028	4.112733	4.123244	3.36843	4.158607	4.373976	3.231264	3.395791	3.169295	3.258764	3.273307	3.440827	3.48116	3.35002
Egr3	-1.42292	4.929791	-3.092484	0.007665	0.04273	-3.326269	5.862653	3.466233	5.485003	6.495325	4.460974	5.559419	4.145391	5.062851	4.582303	4.281549	5.130266	4.718575	5.366799	4.458122
Tmem97	0.674392	4.159342	3.092306	0.007668	0.04273	-3.157537	3.932918	4.449211	4.019053	3.394049	4.68795	4.156917	4.107579	3.942114	4.231815	4.390078	4.21995	4.136468	4.187182	4.465266
Dph5	0.454745	3.604497	3.092038	0.007672	0.04273	-3.188819	3.821655	4.320894	4.149803	4.001571	4.531181	4.475713	3.105363	2.976416	3.178898	3.214035	3.273307	3.10114	3.262178	3.186749
Alox5	0.896969	1.978059	3.091717	0.007677	0.04273	-2.869478	2.035255	3.294639	3.049621	2.077719	2.856578	2.592292	2.380012	0.301855	2.330799	0.799637	0.484915	1.643459	1.837926	2.489076
Eif6	0.547795	5.735047	3.091704	0.007677	0.04273	-3.332517	5.603877	5.999612	6.100675	5.289121	6.286034	5.578378	5.54638	5.722645	5.794348	5.628953	5.888339	5.665397	5.686859	5.529868
Parp14	1.163296	3.05618	3.09087	0.00769	0.042782	-2.933364	2.8647	3.117121	3.693138	1.780306	3.339971	3.579757	2.760215	3.668539	2.381582	3.232094	3.31723	4.426789	2.236876	2.59708
Nppa	0.958785	2.533001	3.09024	0.0077	0.042808	-2.818982	2.311111	2.991607	2.761803	1.273392	2.72297	2.3616	2.582602	2.555575	3.188437	2.088679	2.556491	2.268872	3.134365	2.952659
Cplx3	1.107228	2.881598	3.090108	0.007702	0.042808	-3.213731	4.788412	5.587296	5.130633	3.114742	4.654263	4.734242	1.443753	1.067371	1.255413	2.274406	1.883989	1.488652	1.724133	1.529341
Ucp2	0.663468	6.096448	3.089743	0.007707	0.042816	-3.343272	6.104807	6.346698	6.228432	5.060244	6.246583	6.131446	5.742404	6.298164	5.880732	6.321781	6.557697	6.509728	6.136069	5.809301
Pex5l	-0.603238	5.78315	-3.08956	0.00771	0.042816	-3.34211	6.049442	5.519076	5.333952	5.765863	4.897375	5.470877	6.004382	6.034539	6.115109	5.706885	5.838973	5.991545	6.442581	5.823268
Spsb1	0.98035	4.053639	3.088793	0.007722	0.042862	-3.193935	3.932918	4.613433	5.59056	3.675737	4.567829	4.304105	3.190506	3.897831	3.375441	3.696121	4.048142	4.292824	3.97969	3.685908
Bmp1	0.758838	6.638856	3.087235	0.007746	0.042944	-3.361579	6.230438	7.338426	6.746293	5.757252	6.537704	6.380915	6.676971	6.938211	6.644955	7.129985	6.504865	6.936468	6.331364	6.806553
Trpv3	1.518007	1.909218	3.087194	0.007747	0.042944	-2.656998	0.105685	3.142653	1.951721	1.548999	2.411703	1.943815	2.05625	2.400986	1.981639	1.755735	1.629088	2.568523	1.770732	1.931282
Tcof1	0.40028	5.361196	3.087156	0.007748	0.042944	-3.334126	5.375315	5.59964	5.850444	5.290827	5.790174	5.684684	5.006725	5.495279	4.855111	5.244698	5.261583	5.363275	5.165713	5.111939
Donson	0.855742	3.577518	3.086877	0.007752	0.042949	-3.014045	2.821286	4.286948	3.468922	2.677071	3.551819	3.129052	4.074361	3.591194	3.935874	3.784431	3.555912	3.619352	3.695599	4.032198
Aqp11	-0.573244	3.281919	-3.086172	0.007763	0.04299	-3.037372	3.402817	2.943995	2.853524	3.182467	2.800826	2.297664	3.632178	3.492551	3.689436	3.566911	3.518775	3.562264	3.48116	3.692011
Cthrc1	1.123389	0.236474	3.084332	0.007792	0.04313	-2.513932	-0.410969	1.047298	1.430088	0.485009	1.018831	0.949514	0.202532	-0.048075	-0.632503	0.055728	0.138317	0.081289	0.111637	0.257494
Sept3	-0.586826	7.965155	-3.083297	0.007808	0.0432	-3.358596	8.342934	7.408946	8.119749	8.303601	7.602225	7.821971	7.932133	8.162801	7.890551	7.782218	8.113325	7.90812	8.294519	7.83547
Arhgap27	0.659633	3.704708	3.082968	0.007813	0.043209	-3.101639	3.522857	4.086661	3.706618	2.934433	3.89578	3.812601	3.496648	4.224157	3.375441	4.159476	3.867914	4.440934	3.079891	3.388105
Txnrd1	0.538447	6.708863	3.082015	0.007828	0.043272	-3.378992	6.567445	6.853923	7.626836	6.843894	7.094375	7.405639	6.57675	6.364749	6.391887	6.42449	6.461486	6.388155	6.507652	6.432165
P2ry13	-0.934562	2.075227	-3.081242	0.007841	0.043319	-2.814519	2.658139	1.151583	1.269823	1.892408	1.463853	1.473956	1.754571	3.115498	1.794428	2.641672	2.739799	3.461789	1.943396	2.105984
Kidins220	-0.60235	9.040313	-3.08083	0.007847	0.043319	-3.33823	8.818222	8.2797	8.615033	9.531053	8.469901	8.91808	9.278592	9.176838	9.290872	9.148175	8.9732	9.246506	9.335188	9.486126
Glul	0.586501	10.78985	3.080789	0.007848	0.043319	-3.254996	11.35762	11.99343	11.74515	10.81704	11.55841	11.40341	10.22129	10.29133	10.02896	10.35995	10.20925	10.59943	10.20914	10.26444
Grik2	-0.56389	5.710584	-3.07728	0.007903	0.043607	-3.35245	5.233477	5.15303	5.125624	5.997642	4.748146	5.204169	6.246047	5.944112	6.285903	5.926557	5.813642	5.969758	6.206621	6.125629
C1qtnf4	-0.603799	6.977867	-3.07676	0.007912	0.043632	-3.382948	6.647307	5.630045	5.797886	6.372633	6.28688	5.9088	7.614024	7.416353	7.966673	7.803131	7.340731	7.568098	7.459604	7.892889
Smim3	0.742701	3.568865	3.076178	0.007921	0.043637	-3.150328	3.896775	4.397125	4.065129	2.934433	4.113552	3.96856	3.337752	3.081965	3.280514	3.709073	3.61576	3.52291	2.923692	3.25429
Tbx1	-2.0396	0.663804	-3.076054	0.007923	0.043637	-2.552144	0.369528	-2.267963	0.642822	2.227735	-0.613814	0.056669	1.000645	1.429209	1.579251	1.322149	1.260496	1.404538	1.077784	1.355706
Zfpm2	-1.096988	4.593473	-3.075679	0.007929	0.043637	-3.305354	4.439412	3.472958	4.340564	5.981889	3.935155	4.722115	4.567614	4.80985	4.596704	4.449167	4.757751	4.747316	5.009325	4.548339
Gad1	-0.641215	7.707657	-3.075652	0.007929	0.043637	-3.382948	7.522859	6.966027	7.172629	8.34154	7.588913	7.430074	7.925939	7.813258	7.894563	7.752812	7.656431	7.616028	8.004925	8.228992
Col5a3	0.72328	7.066256	3.075575	0.007931	0.043637	-3.390701	6.668899	7.712254	7.075536	6.714516	7.257721	7.608986	7.300571	6.961449	7.044156	7.129383	6.363644	7.112483	6.706526	7.283535
Aldh1b1	-0.666226	3.790773	-3.075217	0.007936	0.043648	-3.204426	4.180631	2.884706	3.468922	4.155549	3.799498	3.826262	3.706333	3.923301	3.918782	3.669864	4.152854	3.83286	3.892823	3.774841
Kit	-0.965082	6.921337	-3.073542	0.007963	0.043763	-3.394734	6.483771	5.208484	6.261535	7.5635	6.574189	6.183672	7.275213	7.241344	7.591699	7.041875	7.195809	7.069883	7.587137	7.635467
mrpl24	0.502733	4.986243	3.073453	0.007965	0.043763	-3.318338	4.791228	5.35766	5.039476	4.632654	5.517871	4.940905	5.041519	4.886311	4.919536	5.107147	4.997084	4.983074	4.794977	4.847954
Insig2	-0.873051	3.560087	-3.071092	0.008003	0.043952	-3.140664	3.689063	2.082541	3.240874	3.959292	3.075998	3.365711	3.569982	3.622632	3.854323	3.656554	4.004032	3.727169	4.294734	3.842239
Pcdhga3	-0.983047	2.932155	-3.070449	0.008013	0.043988	-3.038455	2.957854	1.397635	2.466695	3.659984	2.496038	2.965855	2.905804	3.137429	3.480126	2.977621	2.912	3.195935	3.467079	3.25429
Gabra5	-1.135208	7.700691	-3.07023	0.008016	0.043988	-3.390415	7.883976	5.914477	6.948453	8.506788	7.735203	7.639611	7.923221	7.787051	7.889089	7.655391	7.891888	7.883732	8.183995	7.975261
Dlg3	-0.886675	7.555833	-3.069895	0.008022	0.043998	-3.393993	7.590687	6.373902	7.353459	8.419236	7.119755	7.62275	7.570015	7.880632	7.725082	7.401344	7.827539	7.590875	7.80709	7.508097
Tcf15	-0.890245	2.217605	-3.067857	0.008055	0.044159	-2.907829	2.445855	1.082904	1.705429	2.718219	2.361201	1.668504	2.450751	2.724465	2.707706	2.40749	2.568656	2.064785	2.203293	2.294252
Bard1	1.756233	-0.869589	3.066254	0.008081	0.044281	-2.432406	-1.417928	0.769921	-0.195392	-0.502853	0.649545	1.450626	-1.21312	-1.196868	-0.929891	-1.838237	-1.500575	-1.243945	-0.628995	-1.212031
Sept2	0.520735	7.370016	3.065534	0.008093	0.044325	-3.405906	7.604358	7.953151	7.703071	7.155648	7.987546	7.959517	7.218693	6.990356	6.976252	7.194147	6.838913	7.383599	7.013708	7.21102
R3hdm2	-0.373168	8.037792	-3.065243	0.008097	0.044331	-3.384807	8.497455	8.185856	8.114491	8.712327	8.196638	8.426839	7.869768	7.733613	7.792312	7.825233	7.758527	7.799097	7.75045	7.872548
Hps5	0.57899	4.672949	3.064727	0.008106	0.044357	-3.275137	4.082914	5.075612	4.631863	4.170451	4.614391	4.515605	5.052933	4.83019	4.815644	4.499529	4.561722	4.862143	4.58936	4.686175
Il1rapl1	-1.580675	1.199907	-3.064408	0.008111	0.044366	-2.711581	1.071521	-0.186163	1.245402	2.656049	0.210178	0.175273	1.17791	1.775313	2.107651	1.023513	1.375754	1.515634	2.317563	1.122575
Foxo1	-0.741352	4.72226	-3.063925	0.008119	0.04437	-3.356276	5.017259	4.364946	4.947854	5.681253	4.213017	4.911303	4.348928	4.856872	4.62508	4.456866	4.781463	4.703986	4.944745	4.319155
Nrp2	-1.30001	5.965794	-3.063726	0.008122	0.04437	-3.394256	5.851906	3.713429	5.612208	6.885129	5.145088	5.76608	6.30809	6.375169	6.391887	5.862791	6.195365	6.435726	6.710266	6.298567
Lsm7	0.572953	4.493458	3.063519	0.008126	0.04437	-3.314169	4.73969	5.108376	4.998051	4.163019	5.266683	4.6901	4.272878	4.219024	4.231815	4.311581	4.387261	3.990233	4.212723	4.388433
Runx1	1.225172	1.430908	3.063451	0.008127	0.04437	-2.779511	1.514695	1.607774	3.145118	1.065902	2.295481	2.734303	0.851758	1.356279	0.317129	1.451089	1.430105	1.375373	0.921344	0.606649
Psmb2	0.499252	6.199993	3.063067	0.008133	0.044385	-3.413498	6.336395	6.516621	7.057677	6.203639	6.975604	6.523994	5.775507	5.869094	6.073242	5.8929	6.033642	5.818157	5.846296	5.899081
Exo1	2.446146	-0.525311	3.062256	0.008146	0.044437	-2.432481	-1.052261	1.506527	-0.263851	-1.223791	1.840817	1.450626	-0.672244	-0.81357	-1.10526	-0.116374	-0.731011	-1.073407	-0.512812	-1.899685
Kcna1	-1.241982	5.545933	-3.06194	0.008152	0.044445	-3.400913	5.949649	4.69623	5.644084	6.877203	4.598129	5.735037	4.92212	5.832592	5.473878	5.003199	5.719944	5.59282	6.392241	5.242409
Lrrn1	-0.671874	6.846026	-3.061593	0.008157	0.044452	-3.418	6.417897	5.584194	6.120113	7.100791	6.446028	6.200378	7.040239	7.378872	7.319535	7.315907	7.076818	7.3451	7.148296	7.365523
Arhgef2	-0.3638	7.855564	-3.061413	0.00816	0.044452	-3.414578	7.627779	7.365333	7.333686	7.720143	7.273595	7.267694	8.32796	8.244324	8.183425	8.229469	8.005313	8.155479	8.032079	8.218725
Ddx39b	0.531703	8.351468	3.059728	0.008188	0.044577	-3.403457	7.989786	8.919387	8.53921	8.011217	8.406332	8.258205	8.478654	8.362214	8.478708	8.52739	8.218643	8.213794	8.110319	8.411571
Smn1	0.527134	4.731328	3.059573	0.008191	0.044577	-3.353571	4.70428	4.948678	5.354078	4.890633	5.674724	5.314898	4.35355	4.498613	4.481148	4.479719	4.606661	4.296718	4.266558	4.429189
Kif11	2.288134	1.061341	3.058725	0.008204	0.044633	-2.636047	0.105685	2.452828	1.116684	0.485009	3.440689	3.179592	-0.067151	1.112357	0.317129	0.847278	0.378374	1.082389	0.598632	0.706485
Gna14	-0.814705	3.324703	-3.058415	0.00821	0.04464	-3.14914	3.676936	2.264105	3.12518	3.732068	3.224646	2.915623	3.616879	3.534475	3.289407	2.956054	3.786926	3.179156	3.91892	3.489688
Ldhb	-0.477898	8.427714	-3.058001	0.008216	0.044657	-3.401195	8.790174	8.221236	8.0171	8.338038	8.281407	7.829621	8.720837	8.373231	8.659987	8.616655	8.544229	8.429331	8.554855	8.615959
Ppm1h	-0.730429	5.049427	-3.057691	0.008222	0.044665	-3.378037	5.1936	4.305905	4.942168	5.780507	4.7007	5.078533	5.021324	5.13548	4.899348	4.785087	5.219768	5.255355	5.539897	4.883051
Wnt7b	0.633493	4.300886	3.056728	0.008238	0.044732	-3.28704	4.137033	4.532048	5.2148	4.038591	4.519714	4.581525	4.040359	3.985077	4.189688	3.918713	4.128407	4.078836	4.605518	4.323092
Rnf14	-0.585185	8.866211	-3.055598	0.008256	0.044813	-3.388279	8.956636	8.222733	8.51611	9.365185	8.916328	8.645026	9.001224	8.865402	8.94929	8.719041	9.104201	8.772433	9.245361	8.851419
Mlxipl	-0.798861	5.246597	-3.055368	0.00826	0.044814	-3.353439	4.867907	4.117033	4.029817	5.424255	4.614391	4.657358	5.236813	6.478654	5.069035	6.230412	5.804669	6.687093	4.893023	5.394223
Fgfr2	-0.480229	7.441132	-3.054647	0.008272	0.044839	-3.42931	7.794553	7.283058	6.873247	7.458517	7.387585	7.043377	7.389913	7.549591	7.296012	7.60258	7.646422	7.772778	7.498629	7.588807
Tec	1.093788	3.44917	3.054639	0.008272	0.044839	-3.092121	3.488576	4.479583	3.733206	2.093442	3.680967	3.513754	3.356296	3.509467	2.95129	3.838474	3.822919	3.816678	2.676906	3.482664
Exosc2	0.446748	4.697882	3.054412	0.008276	0.044839	-3.371389	4.930537	5.605773	5.405955	4.892881	5.095746	5.326158	4.339638	4.548571	4.131534	4.341	4.49954	4.273193	4.290742	4.152141
Pi16	0.777041	3.765399	3.053739	0.008287	0.04488	-3.175504	3.627383	4.24827	4.065129	2.968937	4.216572	3.703341	3.471351	4.284367	3.23521	4.303064	4.052479	4.689249	2.736815	3.237699
Armcx2	-0.49762	6.676781	-3.05267	0.008305	0.044957	-3.438301	6.906201	6.318971	6.510475	7.287513	6.639215	6.927751	6.644332	6.363586	6.661318	6.514849	6.511969	6.503002	6.923136	6.778151
Bag1	0.452241	5.800916	3.052072	0.008315	0.044971	-3.428021	5.891801	6.273469	6.075786	5.928101	6.564457	6.534434	5.448228	5.449994	5.56641	5.716538	5.544742	5.573689	5.268742	5.405408
Orai3	0.692032	4.145498	3.052068	0.008315	0.044971	-3.245295	4.119218	4.302134	4.627145	3.315784	4.625132	3.99299	3.999651	4.155962	4.081214	4.10172	4.180861	4.346409	4.004274	4.275119
Clmp	-1.559412	6.262931	-3.05178	0.00832	0.044976	-3.423383	6.481158	3.418258	5.261214	6.9928	5.530781	6.460446	6.487036	6.804963	6.65703	6.5277	6.734126	6.741679	7.022159	6.589997
Pcdhga11	-0.929154	3.301011	-3.05089	0.008335	0.045037	-3.161002	3.562947	2.279532	2.735741	3.902409	3.346477	2.881136	3.30948	4.032662	3.416608	3.327565	2.912	3.10114	4.038003	3.537917
Cadm1	-0.551889	6.331459	-3.050524	0.008341	0.04505	-3.436023	6.231475	5.624015	6.084417	6.663419	5.8056	6.068663	6.416994	6.338956	6.762979	6.122009	6.298486	6.485196	6.885253	6.87319
Tmem179b	0.765877	3.233312	3.047086	0.008399	0.045342	-3.090926	3.188908	3.785434	3.370733	2.350586	3.706477	3.200721	2.659281	3.484018	2.995434	3.588098	3.366837	3.698019	2.84965	3.195366
Ndufs4	-0.385674	6.750454	-3.046666	0.008406	0.04536	-3.448662	7.314579	6.810113	7.033812	7.401239	7.066079	6.979609	6.540721	6.348388	6.65703	6.328091	6.59237	6.304563	6.685397	6.459252
Ccl7	8.665297	-0.812591	3.046163	0.008415	0.045386	-2.4036	-3.260591	2.425352	5.807301	-2.731185	3.761059	4.410708	-2.558588	-0.26106	-2.604521	-1.564464	-1.142289	0.760186	-2.656794	-3.260591
Dlgap1	-1.076114	8.262177	-3.045686	0.008423	0.045409	-3.419384	8.618707	6.911373	8.310961	9.245055	7.724318	8.480557	8.215968	8.396151	8.3234	7.903377	8.429058	8.19096	8.8001	8.125984
Celf6	-0.536764	3.74753	-3.044101	0.00845	0.045534	-3.208912	3.713016	3.452688	3.347976	3.805502	3.346477	2.781814	4.129307	3.852146	4.19443	4.041554	3.901965	3.838214	4.103184	4.079517
Raly	0.445437	6.480337	3.043553	0.008459	0.045564	-3.454454	6.765117	6.823988	6.848633	6.262197	7.151213	7.007855	6.069049	6.303023	6.133744	6.35409	6.425151	6.310354	6.254544	6.033714
Cfb	1.082962	4.258574	3.043046	0.008468	0.04559	-3.262629	4.243653	4.747052	4.284379	3.051752	4.68795	5.091789	4.238302	4.840254	3.952765	4.653926	3.892318	4.565594	3.302384	4.156561
Plxna2	-0.556304	7.434096	-3.042819	0.008472	0.04559	-3.456521	6.958484	6.636783	7.049325	7.722673	6.591407	6.85576	7.927103	7.771796	7.996507	7.542796	7.628012	7.623799	7.942689	7.839943
Cald1	0.836201	5.484759	3.041968	0.008486	0.045629	-3.43131	5.850557	5.842297	6.045165	5.001038	6.299507	6.828304	5.305224	4.873301	5.460161	5.171725	4.653101	5.302599	4.982355	5.208755
Akt3	-0.970597	6.139996	-3.041904	0.008487	0.045629	-3.456682	6.703684	5.212508	6.150022	7.221075	5.969443	6.629694	6.057763	6.123756	5.962235	5.537522	6.186512	6.035321	6.530682	5.663198
Prex2	-0.758914	6.648403	-3.041512	0.008494	0.045629	-3.458612	7.562949	6.966622	6.873745	7.157519	6.180763	6.390142	6.795524	5.979449	6.488686	6.465138	6.013759	6.69744	6.969445	6.552757
Pip4k2b	-0.705128	6.036218	-3.040868	0.008505	0.045629	-3.455413	6.402329	5.433746	5.995694	6.693391	5.731741	6.207355	5.829598	6.037457	5.832874	5.765405	6.14852	6.109149	6.553351	5.790939
Snai2	-1.279861	0.364418	-3.040771	0.008507	0.045629	-2.64473	0.739674	-0.572649	-0.761158	1.430203	0.770579	0.231108	0.903114	0.377517	0.385075	0.280604	0.484915	0.567345	1.1849	0.383504
Zcchc12	-1.057541	7.1344	-3.04074	0.008507	0.045629	-3.456953	7.443655	5.39	7.148138	7.805552	6.712179	7.010849	7.088178	7.285593	7.49939	7.399847	7.214801	7.170936	7.270886	7.453914
Nipa1	-0.684474	4.343453	-3.040695	0.008508	0.045629	-3.324992	4.315816	3.7962	3.906747	4.785664	3.654998	4.135132	4.390007	4.60868	4.504248	4.081942	4.417843	4.775496	5.019009	4.496982
Prox1	-1.631122	5.09125	-3.040597	0.00851	0.045629	-3.399746	4.865238	3.019437	4.928811	6.799051	3.557438	5.250499	4.874668	5.751372	5.408039	5.341446	5.450923	5.583286	5.526381	4.978507
Nipsnap1	-0.38375	7.005769	-3.040315	0.008514	0.045629	-3.462744	7.223194	6.72605	6.765204	7.095432	6.901335	6.711098	7.067928	7.091419	7.149253	7.078516	7.268925	6.937091	7.168014	6.90965
Pcgf5	0.665292	2.716341	3.040183	0.008517	0.045629	-3.084002	2.582145	2.943995	3.585381	2.787519	3.540516	3.282273	2.05625	2.522625	2.260183	2.641672	2.346454	2.619163	2.522078	2.584013
Col7a1	1.47004	3.801511	3.039571	0.008527	0.045656	-3.042265	2.742017	4.601207	3.585381	1.874319	3.61245	3.22842	4.722163	3.884925	4.321248	4.703306	3.838074	4.105722	3.5956	4.541596
Cadps	-1.005665	7.11714	-3.039446	0.008529	0.045656	-3.459835	7.223194	5.809463	6.68859	7.990898	6.884112	7.015328	7.24381	7.479421	7.286605	6.649434	7.401927	6.98675	7.843992	7.148601
B4galt5	0.484843	5.707705	3.038207	0.008551	0.045749	-3.442571	5.403188	5.839697	6.308704	5.700631	6.042375	5.958232	5.2443	5.808873	5.243904	5.777752	5.670004	5.980693	5.345791	5.61423
Klhl29	-0.582699	5.857286	-3.037918	0.008556	0.045756	-3.448635	5.850557	5.186149	5.258166	6.167885	5.298904	5.96648	6.279225	6.066325	5.918481	5.998214	5.987948	5.859553	6.117033	6.07516
Nelfe	0.356804	5.608604	3.035267	0.008601	0.04598	-3.455048	5.889175	6.082202	6.112538	5.580254	6.124153	6.038663	5.198785	5.520532	5.232422	5.515573	5.457481	5.374373	5.123695	5.303471
St3gal2	-0.565134	7.113677	-3.034355	0.008617	0.046009	-3.474162	7.157071	6.0031	6.47678	6.957258	6.689332	6.798584	7.677158	7.257705	7.481556	7.460461	7.244014	7.394996	7.357874	7.647686
Mastl	1.898021	-0.554973	3.034322	0.008618	0.046009	-2.504966	-0.634519	1.369077	0.049949	-1.075778	1.113405	1.07687	-0.529223	-2.076466	-1.304935	0.055728	-0.731011	-0.334482	-1.047095	-1.040339
Serpinf2	1.400446	0.767725	3.03429	0.008618	0.046009	-2.576436	-0.217457	1.339943	0.307539	-0.243853	1.72577	1.07687	0.903114	1.067371	0.884903	0.938085	0.885831	1.11804	0.649393	1.29285
Adcy9	-1.46054	4.66617	-3.033775	0.008627	0.046036	-3.369883	4.73969	2.452828	4.369101	5.760949	3.623205	4.649696	4.858498	5.495279	4.959085	4.294496	5.101249	4.958703	5.578065	4.561731
Rnaset2	1.042965	5.506614	3.03283	0.008644	0.046097	-3.39815	5.19997	5.370323	5.221076	4.022255	6.346508	5.608994	4.394501	6.700192	4.413615	6.534082	6.492945	7.212241	4.677602	4.940566
Parvb	0.510433	5.876396	3.03268	0.008646	0.046097	-3.462832	6.061153	6.3676	6.282457	5.510231	6.389665	6.0858	5.592083	5.530925	5.618503	5.560929	5.685448	5.950112	5.86521	5.750578
Rps3a	1.010042	2.507787	3.032249	0.008654	0.046117	-2.846964	1.693879	2.506257	2.286937	1.033666	2.72297	1.839907	2.967416	2.939445	3.027678	3.167876	2.696109	2.9008	2.535634	3.097617
Notch4	0.836514	5.414487	3.030823	0.008679	0.046229	-3.425022	5.117126	6.389093	5.929849	4.746422	5.381992	5.379636	5.730014	5.181064	5.424278	5.562714	5.182653	5.263337	4.964926	5.607789
Pcdhb5	-0.87751	3.302866	-3.029964	0.008694	0.046288	-3.1736	3.380557	2.01041	2.548818	3.732068	2.962008	3.214637	3.957761	3.741948	3.307029	3.493953	3.338699	3.361247	3.379583	3.983274
Lrrc6	-1.265164	0.472504	-3.029506	0.008702	0.04631	-2.735927	1.245833	-0.366503	0.484899	1.740915	0.917619	0.117189	0.282279	1.067371	-0.27702	0.134646	0.764165	-0.070959	1.218908	0.554014
Spef2	-1.350129	1.063146	-3.027977	0.008729	0.046411	-2.722191	1.853246	-0.687936	0.52603	1.065902	0.649545	0.056669	1.862293	1.564863	1.180909	1.180545	2.274011	1.404538	1.747621	1.085968
Anks1b	-0.634187	7.956755	-3.027936	0.008729	0.046411	-3.463175	8.249296	7.473667	8.098176	8.771557	7.705464	8.227006	7.931747	7.939549	7.757087	7.628801	7.935107	7.778359	8.118787	7.786405
Nfix	-0.720072	6.235895	-3.027689	0.008734	0.046411	-3.48618	6.699943	5.701936	6.485929	7.193983	6.109886	6.607451	5.740641	6.274856	6.004701	6.037251	6.103242	6.292913	6.348747	5.722532
RT1-M6-2	-1.734683	1.650945	-3.027373	0.008739	0.046411	-2.919393	2.162475	0.118359	0.851376	3.145235	1.622351	1.302125	0.851758	2.488905	3.480126	1.180545	1.260496	1.345607	1.700256	2.194616
Ttc33	-0.913587	4.512868	-3.027329	0.00874	0.046411	-3.419026	4.865238	3.817493	4.454139	5.785355	4.522589	4.861313	4.35355	4.647404	4.254698	3.861859	4.64163	4.145134	4.850267	4.19144
Slc19a2	-0.515878	4.289673	-3.025158	0.008778	0.046577	-3.342399	4.33909	3.582736	3.789185	4.421542	3.836938	4.247696	4.714996	4.117861	4.632088	4.298786	4.50904	4.338875	4.56643	4.743122
Gphn	-0.398292	6.376408	-3.02512	0.008779	0.046577	-3.488797	6.393092	5.941398	6.316784	6.699183	6.125099	6.209094	6.455131	6.494672	6.451535	6.381653	6.44178	6.306496	6.587919	6.484961
Rfc2	0.783566	4.055662	3.024398	0.008792	0.046607	-3.274902	4.045673	4.693353	4.191945	3.043681	4.454966	3.892687	4.171806	4.061624	4.034376	4.205901	4.196624	4.046822	3.772036	4.065483
Klf13	-0.940106	3.157244	-3.024359	0.008792	0.046607	-3.258298	3.978094	2.324846	3.595078	4.042646	2.781752	3.513754	2.73209	3.252427	2.694377	2.641672	3.310002	3.496064	3.467079	2.55752

Pgam2	-0.805659	2.449816	-3.023788	0.008802	0.04664	-3.032383	2.765113	2.117298	2.61703	2.718219	1.578815	1.450626	2.717819	2.555575	2.667343	2.600561	2.506778	2.794648	2.913344	2.59708
Fam110d	1.189061	2.547487	3.023295	0.008811	0.046664	-3.053519	2.706665	4.309667	3.353699	1.799605	3.245679	2.781814	2.078759	2.207232	2.295923	2.854798	2.133674	2.153355	2.003164	2.030937
Rspo2	-2.230112	4.888994	-3.023057	0.008815	0.046664	-3.370753	5.19997	0.369671	3.99728	6.038827	4.296017	4.734242	5.003787	5.854273	5.648188	4.908123	5.504148	5.346466	6.027521	5.650719
Cdca4	0.585158	3.662694	3.022872	0.008819	0.046664	-3.23767	3.549707	3.904639	3.763618	3.145235	4.292652	3.727807	3.632178	3.622632	3.742206	3.676473	3.822919	3.581545	3.423994	3.517445
Slc1a4	0.708189	4.634746	3.021145	0.008849	0.046806	-3.383658	4.692281	5.216521	5.224203	3.902409	4.866025	4.659903	4.316148	4.411301	4.496589	4.311581	4.627162	4.877826	4.728657	4.620503
S1pr5	-1.032363	6.044459	-3.02018	0.008867	0.046877	-3.486745	6.254099	5.777223	4.366273	6.482694	6.015073	5.226661	6.126897	6.294509	6.183488	6.562013	5.8956	6.690797	6.224518	6.5494
Hscb	0.533568	3.756208	3.018249	0.008901	0.047002	-3.318887	3.963192	4.320894	4.443444	3.627954	4.516833	3.997021	3.713543	3.42283	3.375441	3.545408	3.58015	3.594258	3.286436	3.31881
Rpl21	0.646111	4.082745	3.01819	0.008902	0.047002	-3.251434	3.670834	4.064569	3.784955	3.051752	4.292652	3.830786	4.421169	4.284367	4.338488	4.623466	4.397527	4.281077	4.061622	4.152141
Exd2	-0.489513	6.668182	-3.017963	0.008906	0.047002	-3.505245	6.693938	5.926776	6.334258	6.869804	6.255251	6.652877	6.796373	6.789464	6.864655	6.741347	6.669937	6.858256	6.887898	7.029556
Glcci1	-0.618557	4.748581	-3.017698	0.008911	0.047002	-3.44671	4.968344	4.302134	4.808946	5.633655	4.646377	4.980131	4.696922	4.685117	4.730053	4.421893	4.720073	4.444449	4.959907	4.541596
Fam111a	4.192084	0.005088	3.01766	0.008912	0.047002	-2.53601	-1.22354	2.894758	0.945338	-0.941548	3.8553	3.94371	-1.009457	-0.511025	-1.10526	-1.33445	-0.616402	-0.153565	-1.218554	-1.040339
Atp13a4	-0.969114	3.507818	-3.017538	0.008914	0.047002	-3.209804	4.110227	3.317238	2.10888	3.204354	3.052318	2.336365	4.023054	3.42283	3.786848	3.935342	3.786926	3.880344	3.954679	4.346493
Prkce	-1.265249	8.057613	-3.017309	0.008918	0.047003	-3.480624	8.446575	6.496959	7.976873	9.133745	7.372923	8.256104	7.944059	8.417874	8.022741	7.64279	8.474014	8.169617	8.755722	7.703123
Efna1	0.574177	3.857576	3.016681	0.008929	0.047042	-3.31368	3.601952	4.397125	4.296673	3.857074	4.534033	4.005051	3.436917	3.727562	3.540964	3.861859	3.690026	4.014082	3.278395	3.874793
Trim55	1.598993	1.702323	3.015521	0.00895	0.047131	-2.830496	1.541696	3.184227	1.493674	0.485009	2.927714	2.695134	1.576217	2.030999	1.142163	2.127786	1.844511	2.100868	0.313987	0.929479
Lrfn5	-0.798795	5.992659	-3.014697	0.008965	0.047189	-3.492119	5.799735	4.397125	5.454701	6.348266	5.557661	5.675909	6.342214	6.316303	6.370047	6.223659	6.241687	6.293887	6.371603	6.531645
Adam23	-1.229106	6.39085	-3.013769	0.008982	0.047229	-3.503341	6.255119	4.24827	5.957679	7.053343	5.39146	6.074398	6.536661	7.090716	6.83003	6.259316	6.81932	6.801145	7.348303	6.828878
Zfp238	-1.053994	7.654473	-3.013651	0.008984	0.047229	-3.505461	7.800504	5.960666	7.048443	8.182255	7.138618	7.600731	7.709071	8.296119	7.906172	7.565673	8.325333	7.895336	8.200578	7.541854
Slc22a23	-0.440568	6.866772	-3.013623	0.008984	0.047229	-3.514862	6.898388	6.461892	6.280438	6.89245	6.614499	6.464819	6.943138	7.087903	6.834594	6.981775	6.972525	7.267853	7.194428	7.253934
Sash3	0.839695	2.840905	3.013109	0.008994	0.047239	-3.094208	2.718545	3.24835	3.203331	2.13961	3.736505	2.800388	1.888013	3.660989	1.426399	3.649853	3.53126	3.70975	1.983515	2.325991
Slc12a9	0.830216	5.150929	3.013064	0.008995	0.047239	-3.384074	4.637014	5.292622	5.083193	3.649386	4.985453	4.46411	5.41323	5.719014	5.469972	5.639139	5.269057	5.75304	5.143751	5.642865
Cdh8	-1.013862	5.676697	-3.012868	0.008998	0.047239	-3.486465	5.428725	4.005493	5.233545	6.52968	5.068462	5.538825	5.759921	6.130606	6.094961	5.852614	5.791755	6.015372	6.058371	5.999679
Cd8b	5.074504	-2.187902	3.012517	0.009004	0.047252	-2.465809	-3.260591	-2.267963	0.105278	-3.260591	1.48759	0.386712	-3.260591	-0.654381	-2.604521	-2.176425	-0.411343	0.403657	-2.232431	-2.653844
Ifi44	0.787934	3.98092	3.012211	0.00901	0.04726	-3.314166	3.932918	4.51911	4.39431	3.342347	4.584435	4.146066	3.374605	4.397736	3.23521	4.588849	4.017407	5.042261	2.923692	3.34228
Ppp1r37	-0.383015	7.485308	-3.011453	0.009024	0.047311	-3.516455	7.601554	7.100221	6.993598	7.342826	7.268466	6.996824	7.788459	7.547545	7.729581	7.766429	7.601984	7.634609	7.619721	7.811496
Mfsd3	0.444755	4.395967	3.011246	0.009027	0.047311	-3.329376	3.838085	4.286948	4.226666	3.747057	4.36492	4.060041	4.551611	4.616508	4.790809	4.700066	4.543347	4.610089	4.509176	4.775092
Irak1bp1	-0.583203	3.883605	-3.010545	0.00904	0.047344	-3.362901	4.189194	3.24835	3.960246	4.509141	3.86892	3.972661	3.662299	3.997121	3.81791	3.734632	3.939921	3.71558	4.009141	3.853172
Slc9a7	-1.05931	3.849683	-3.01046	0.009042	0.047344	-3.255159	3.569521	2.117298	3.271431	4.196165	2.732935	3.165332	4.161298	4.552657	4.359752	3.689602	4.352504	4.224961	5.141536	4.489994
Gria3	-0.522642	8.546016	-3.010083	0.009049	0.047358	-3.495157	8.310185	8.060302	7.784512	8.87112	8.021987	8.395683	8.990506	8.664596	8.858093	8.778808	8.574141	8.653339	8.779239	8.906088
Adm	2.012085	1.308506	3.009887	0.009052	0.047358	-2.795506	1.717752	3.091129	3.674966	-0.243853	1.840817	1.857753	0.118118	1.49863	0.512061	1.180545	1.199219	1.252437	0.546019	0.844371
Cdk14	-0.712039	7.754042	-3.009656	0.009056	0.047359	-3.503966	8.109383	7.245256	7.716737	8.586554	7.600865	7.979776	7.780328	7.596854	7.619292	7.311316	7.84671	7.480509	8.231622	7.458843
Dnajc9	0.438513	4.9957	3.006986	0.009105	0.047593	-3.470081	5.002757	5.471176	5.069347	4.965167	5.628766	5.523534	4.722163	4.803005	4.849109	4.821324	4.79964	4.859513	4.704856	4.769331
Zfp219	0.581316	5.546037	3.006741	0.00911	0.047596	-3.476964	5.380933	5.919409	5.558705	4.711187	5.585425	5.423733	5.594038	5.731684	5.412116	5.949932	5.657244	5.852956	5.240048	5.661644
Ttc30b	-1.059468	3.91938	-3.006424	0.009115	0.047606	-3.402992	4.488808	2.690649	3.684081	5.166265	4.051203	4.626461	3.706333	3.845499	3.662308	3.508843	4.172914	3.594258	3.964736	3.820121
Tmprss7	-1.390424	2.712127	-3.005794	0.009127	0.047629	-3.011701	2.957854	2.758277	1.951721	2.499634	0.266146	0.571087	3.428178	3.865347	3.23521	3.377094	3.295436	3.625558	3.070609	3.417866
Yars	0.348337	6.597274	3.005747	0.009128	0.047629	-3.530055	6.71707	6.952857	7.165732	6.700467	7.098716	7.010849	6.231052	6.369389	6.316677	6.32914	6.543128	6.339905	6.398747	6.204585
Gga3	-0.364049	7.065732	-3.004669	0.009148	0.047712	-3.532814	7.026966	6.786012	6.745204	7.323374	6.875142	6.837889	7.213617	7.046488	7.238054	7.205617	7.213349	7.046367	7.083436	7.29059
Rem2	-1.08128	4.364532	-3.004376	0.009153	0.047719	-3.383067	4.827336	2.397344	3.688616	4.48252	3.8553	4.267857	4.8055	4.76477	4.635579	4.542569	4.918469	4.677349	5.145962	4.182799
Mat2b	-0.603288	6.329328	-3.004023	0.00916	0.047732	-3.532884	6.699194	5.843595	6.31898	7.053845	6.263009	6.666863	6.20954	6.081959	6.427568	6.06648	6.484944	5.980693	6.372548	6.161255
Klhdc8b	-0.724198	5.112291	-3.003778	0.009164	0.047735	-3.478715	5.810876	4.979704	4.884032	5.011421	4.496504	4.377073	5.236813	5.425693	5.171196	5.46111	5.774791	5.316091	4.608729	5.065689
Tmem178b	-1.021855	7.093686	-3.003247	0.009174	0.047765	-3.533036	6.860019	5.467813	6.487882	7.792065	6.393597	6.858532	7.362081	7.514409	7.238628	6.953483	7.514685	7.404501	8.026089	7.450768
Sept10	0.689298	3.122355	3.003042	0.009178	0.047765	-3.238429	3.453461	3.758163	3.599901	2.77782	3.789984	3.99299	2.880403	3.081965	2.524011	2.956054	2.544222	3.38339	2.575555	2.584013
Tor2a	0.431586	4.953689	3.000562	0.009224	0.047931	-3.444871	4.774252	4.998471	4.97038	4.328278	5.089943	4.845749	4.983054	5.016268	4.992142	5.176396	5.03922	5.239258	4.895654	5.05389
Ston2	-0.768238	4.238024	-3.000296	0.009229	0.047931	-3.460388	5.216821	4.619507	4.895848	5.025832	3.731543	4.160517	3.908348	3.770297	4.175368	3.588098	4.056804	4.257294	4.221137	3.791988
Nfkb1	0.49181	5.382772	3.000053	0.009233	0.047931	-3.487681	5.245844	5.614923	5.624096	4.814411	5.45153	5.378091	5.408795	5.482485	5.234726	5.528417	5.369823	5.755871	5.047677	5.440265
Vash2	0.859113	2.180779	2.999921	0.009235	0.047931	-2.938758	1.80948	2.690649	2.597868	1.273392	2.046579	2.146157	2.854546	2.207232	2.381582	2.239124	2.031907	2.046399	2.253378	2.310209
Memo1	0.398293	5.053523	2.999777	0.009238	0.047931	-3.480369	4.940716	5.501094	5.292838	4.945809	5.313918	5.260595	5.219191	4.729108	4.961869	4.968612	4.879936	4.797651	4.869129	5.116484
Sirt7	0.504114	5.346665	2.999777	0.009238	0.047931	-3.488445	5.043471	5.543242	5.556225	5.015553	5.796127	5.23522	5.402117	5.314586	5.518052	5.530243	5.17869	5.168764	5.128176	5.461855
Foxn3	-0.749676	4.783959	-2.999748	0.009239	0.047931	-3.473258	5.076159	4.788078	4.588836	5.418025	3.89578	4.74387	4.61073	4.937208	4.703686	4.982013	4.363019	5.181457	5.038184	4.707418
Gatad2a	0.399426	6.567796	2.999411	0.009245	0.047931	-3.539177	6.379126	6.588829	6.942768	6.234102	6.74763	6.542042	6.504808	6.618735	6.433597	6.578868	6.492147	6.733083	6.636695	6.533345
Fam69a	-0.628876	5.600681	-2.999377	0.009246	0.047931	-3.51789	5.728082	4.598135	5.378145	6.029663	5.419496	5.593102	5.666417	5.518445	5.753159	5.639139	5.700728	5.799066	5.832633	5.786668
Dlgap4	-0.340518	8.111662	-2.998832	0.009256	0.047963	-3.518653	8.550139	8.039499	8.247437	8.423519	8.154859	8.147625	7.926327	8.062133	7.949244	7.948445	8.071994	7.980115	8.095807	7.971817
Cnksr3	0.472096	4.5742	2.99846	0.009263	0.047978	-3.463517	4.637014	5.302095	5.170091	4.748906	4.93455	5.257237	4.192594	4.406794	4.060584	4.159476	4.168924	4.625653	4.08486	4.358053
Sytl5	-1.590942	2.991528	-2.997196	0.009286	0.048063	-3.184702	3.09177	0.118359	2.977318	4.192519	2.109124	2.957603	3.684484	3.104407	3.495578	3.070874	3.099749	3.092205	4.028447	3.133933
Nr4a2	-0.652934	5.808509	-2.997151	0.009287	0.048063	-3.542794	6.363131	6.441464	5.71805	6.828709	5.519311	6.105539	5.765135	5.243062	5.483597	5.378399	5.624113	5.607003	5.648382	5.622242
Bin2	0.811803	4.98106	2.995546	0.009317	0.048193	-3.446217	4.762823	5.210498	4.636566	4.018141	5.614014	5.308423	4.53949	5.883764	4.437813	5.773134	5.566136	5.731623	3.828196	4.479448
Ppara	-0.716707	1.547087	-2.995387	0.00932	0.048193	-2.909916	1.996747	0.854759	1.514261	1.679737	1.20216	0.915838	1.888013	1.658838	1.867508	1.780488	1.605665	1.542121	1.902128	1.80183
Kctd1	-0.602794	6.156692	-2.995028	0.009327	0.048207	-3.548144	6.4521	5.596564	6.087855	6.851427	5.991401	6.519797	5.97168	6.33184	6.106329	6.050032	6.30668	6.035321	6.009418	5.90566
Tor4a	0.705279	3.446042	2.994604	0.009335	0.048227	-3.209164	3.214303	3.390109	3.62851	2.523044	3.917788	3.255598	3.392685	3.660989	3.35864	3.559779	3.723055	4.023513	3.36447	3.380568
Hrh3	0.731959	7.229607	2.991685	0.00939	0.04849	-3.555489	7.443207	7.926145	7.560953	6.293164	7.408719	7.498148	7.36036	6.756188	7.134524	7.067244	6.975384	6.950749	7.270379	7.580196
S1pr1	-0.548991	7.169306	-2.991379	0.009396	0.048499	-3.551729	8.057416	7.357632	7.316206	7.460034	7.088566	7.08327	7.127845	6.519935	7.044813	6.805314	6.78193	7.092461	7.318715	7.327318
Plxnb2	0.620281	7.459018	2.990892	0.009405	0.048526	-3.557274	7.436029	7.68753	7.56219	6.495325	7.510706	7.580722	7.288551	7.775297	7.172272	7.691731	7.7178	8.122674	7.021557	7.373138
Gata2	0.755573	3.710645	2.990048	0.009421	0.048587	-3.354332	3.810597	4.946264	4.12993	3.431645	4.282511	4.138786	3.537848	3.282262	3.780555	3.588098	2.968181	3.361247	3.143247	3.673625
LOC100125362	-1.188354	7.7102	-2.988542	0.009449	0.048693	-3.553232	7.504963	5.797457	6.911582	8.676402	7.177446	7.711913	7.64169	8.198445	7.991067	7.957632	8.210418	8.193051	8.160945	7.818486
Jup	0.576743	6.325769	2.988522	0.00945	0.048693	-3.556284	6.380061	6.921856	6.575591	5.6695	6.598237	6.300826	6.249771	6.066325	6.281453	6.262614	6.164639	6.460239	6.298941	6.350609
Lsm11	-1.658836	2.508716	-2.988262	0.009455	0.048693	-3.043421	2.843156	0.310827	1.631973	2.908002	1.997824	0.982423	3.003159	3.232189	3.09008	1.964563	3.621611	3.212521	4.161181	2.530531
Thbd	0.72949	3.945429	2.988116	0.009457	0.048693	-3.388454	3.963192	3.967899	4.511585	3.69132	4.735811	4.953408	4.177031	3.542716	3.696138	3.796617	3.408041	3.587916	3.725479	3.584586
Trim9	-0.51317	8.101849	-2.987421	0.009471	0.04874	-3.548926	8.006841	7.326407	7.879316	8.473379	7.789923	7.911732	8.318534	8.309462	8.398013	8.165132	8.251793	8.053298	8.307172	8.240748
F7	3.015167	-3.015487	2.986802	0.009482	0.04878	-2.489557	-2.659044	-1.271663	1.245402	-2.344749	-0.107758	-0.750296	-2.558588	-2.550306	-3.260591	-3.260591	-3.260591	-3.260591	-2.232431	-2.653844
Npdc1	0.442388	8.347551	2.985864	0.0095	0.048823	-3.550278	8.10656	8.58164	8.399564	7.847012	8.533997	8.161162	8.365062	8.317889	8.439549	8.575478	8.271928	8.409687	8.287025	8.574032
Tle1	-0.629803	5.927948	-2.985806	0.009501	0.048823	-3.551894	5.846502	4.843622	5.588134	6.272594	5.473985	5.804544	6.237319	6.282257	6.187061	6.269188	6.150545	6.14739	5.898379	6.016797
Syt17	-1.061113	6.393516	-2.985725	0.009503	0.048823	-3.563168	6.434199	4.528825	6.082695	7.045786	6.347319	5.739856	6.652795	6.512612	6.94564	6.460359	6.824402	6.310354	6.908883	6.736687
Haus8	0.887337	2.532911	2.98441	0.009528	0.048931	-3.039515	2.072763	3.134193	2.548818	1.679737	2.838232	2.481552	2.841442	3.012464	2.639794	2.795315	2.197736	2.606669	2.220182	2.673087
Lman1l	2.617699	-0.805222	2.983923	0.009537	0.048958	-2.542008	0.307952	1.991799	0.484899	-2.731185	0.470544	0.336685	-0.399109	-2.076466	-0.773548	-1.33445	-1.310338	-0.920914	-0.628995	-1.040339
Slc7a4	-0.636495	5.904459	-2.983097	0.009553	0.049019	-3.536472	5.624546	4.40418	4.847991	5.633655	5.388311	5.321343	6.501688	6.140141	6.673257	6.381653	6.247374	6.281167	6.488611	6.758659
Ppil3	0.645976	3.150575	2.982856	0.009558	0.049021	-3.252128	3.263791	3.702025	3.370733	2.708042	3.97768	3.426763	3.083269	2.603631	3.216682	2.966878	3.031075	2.761217	3.116435	3.060363
E2f1	0.723224	2.890021	2.981895	0.009576	0.049092	-3.222741	2.977763	3.570246	2.962413	2.62393	3.859855	3.683465	2.760215	2.539194	2.524011	2.454393	2.739799	2.516041	2.794335	2.673087
Notum	-1.524215	2.334175	-2.981652	0.009581	0.049092	-3.004566	2.810225	2.064844	-0.33572	2.213416	1.390206	1.193889	3.428178	2.976416	3.495578	2.956054	2.166061	3.028062	2.508394	3.229331
Ptpn12	0.411777	5.775479	2.981507	0.009584	0.049092	-3.56634	5.693887	6.123393	6.460296	6.106253	6.38809	6.278641	5.54436	5.403241	5.456218	5.428201	5.29125	5.446256	5.618437	5.647583
Pcdha7	-1.164452	0.618734	-2.981099	0.009592	0.049092	-2.730295	0.915118	-0.465897	-0.263851	0.860705	-0.718553	0.65513	0.743216	0.923327	1.326257	0.938085	0.805871	0.969826	1.651284	1.473753
Eif5a	0.693487	7.501983	2.981074	0.009592	0.049092	-3.57278	7.703335	8.07419	8.058646	6.802642	8.186929	7.468692	7.149272	7.32016	7.399743	7.283986	7.64355	7.312293	7.428606	7.204632
Coro2a	-0.711196	2.750176	-2.979074	0.009631	0.049269	-3.24668	3.09177	2.702143	2.869592	3.595195	2.702832	2.323579	2.905804	2.419003	2.508863	2.184529	2.627988	2.568523	3.416687	2.825198
Lhfp	0.45604	5.906306	2.978739	0.009637	0.049281	-3.570155	5.886545	6.433729	6.190559	5.782933	6.228207	6.308146	5.57239	5.557602	5.570064	5.911228	5.674232	6.098034	5.632699	5.868424
Pard3	-0.516433	5.345099	-2.977102	0.009669	0.049423	-3.551173	5.399502	4.755351	5.05361	5.85273	5.302254	5.334148	5.335916	5.577792	5.389549	5.229048	5.464008	5.350218	5.479806	5.346662
Slc24a4	-0.605674	4.363076	-2.976632	0.009678	0.049443	-3.440727	4.260004	3.812199	4.106393	4.621853	3.333437	4.101824	4.587372	4.485848	4.765539	4.456866	4.730939	4.398075	4.646703	4.85613
Sept7	-0.683029	7.056006	-2.976287	0.009685	0.049443	-3.578141	7.585019	6.954059	6.960693	8.335561	7.386796	7.801082	6.769816	6.581224	6.823157	6.442051	6.585651	6.682635	6.984968	6.903747
RGD1559747	-0.544106	4.340007	-2.97626	0.009685	0.049443	-3.484483	4.805223	4.532048	3.952724	4.751386	4.511054	3.97675	4.502506	4.015001	4.397255	4.21501	4.309659	3.880344	4.605518	4.384671
Nrxn1	-0.860432	7.685134	-2.975372	0.009703	0.049511	-3.575169	7.823721	6.929211	7.635671	8.626785	6.924346	7.966987	7.756964	7.734962	7.885428	7.139584	7.585241	7.660529	8.074779	7.855658
Slc22a8	-1.420958	4.700958	-2.974123	0.009727	0.049614	-3.499609	6.309154	4.051149	3.196978	4.374055	3.557438	4.872329	4.92833	4.523808	5.213859	5.133806	5.364599	4.616334	4.576302	5.165551
Efnb3	-0.886018	7.699858	-2.973294	0.009743	0.049653	-3.588621	7.327243	5.992608	7.248317	8.117406	7.096788	7.001347	7.807115	8.229086	8.296084	8.189307	7.987301	8.057321	8.069838	8.386861
Kcnj3	-1.373777	5.864867	-2.973108	0.009747	0.049653	-3.568174	5.749954	3.445867	5.248982	6.864659	5.089943	5.912006	5.945892	6.407114	6.437603	5.980965	6.289327	6.224032	6.446174	6.099468
Vhl	0.553895	5.644336	2.973101	0.009747	0.049653	-3.564613	5.765785	6.001938	6.188959	5.074178	5.999679	5.679676	5.513711	5.382731	5.473878	5.615258	5.78395	5.59282	5.600812	5.379173
Rnf19b	0.596097	4.683271	2.971666	0.009775	0.049775	-3.510591	4.70129	4.835816	5.731301	4.514991	5.052636	5.159902	4.330288	4.527965	4.405458	4.307328	4.63586	4.529644	4.484924	4.410806
Sept9	0.46892	5.981263	2.971287	0.009783	0.049792	-3.591823	6.261224	6.501077	6.778562	5.997642	6.482662	6.625794	5.406572	5.695184	5.625543	5.511883	6.044571	5.581691	5.710957	5.540053
Ppm1b	-0.52021	7.085876	-2.970252	0.009803	0.04985	-3.597404	7.245435	6.892058	6.7759	7.741818	6.891915	7.330548	7.175616	7.036311	6.979003	6.919063	7.123435	6.975862	7.243784	6.883225
Samd12	-0.982999	2.548258	-2.97018	0.009804	0.04985	-3.164773	2.753611	2.383133	1.981598	3.145235	1.173177	2.458346	2.717819	2.835955	2.462438	2.529327	3.189137	2.656009	3.262178	2.417223
Yif1a	0.681634	4.128716	2.970078	0.009807	0.04985	-3.444614	4.300089	4.681789	4.514142	3.670505	5.143221	4.281144	3.769961	3.818604	3.793114	3.901891	3.986003	3.931333	3.89808	4.204306
Slitrk2	-0.589982	4.554024	-2.968125	0.009845	0.050001	-3.507881	4.70129	3.719097	4.539472	5.054231	4.43374	4.443576	4.510807	4.65122	4.71693	4.437541	4.615483	4.433879	4.78366	4.783689
RGD1563365	0.807113	2.888318	2.96812	0.009845	0.050001	-3.261539	2.765113	3.828023	4.092769	2.925676	3.633881	3.038068	2.165439	2.926907	2.024877	2.600561	2.843564	2.761217	2.713149	2.341603
Bin3	0.395993	4.960333	2.967943	0.009849	0.050001	-3.517869	4.698293	5.097537	5.165217	4.605497	5.010247	4.909165	4.974076	5.082767	5.056067	5.181051	5.010524	4.846288	4.852977	5.005709
Coq2	-0.50559	6.029184	-2.967711	0.009853	0.050003	-3.5958	6.051792	5.467813	5.669545	6.507846	5.803238	6.158695	6.106502	6.109958	6.001999	6.076509	6.091697	6.038813	6.190643	6.157952
Yaf2	-0.548067	6.331049	-2.966981	0.009868	0.050055	-3.606235	6.805363	5.983217	6.412428	7.082688	6.598919	6.588873	6.306898	5.996048	6.166694	5.907019	6.338111	6.048084	6.414424	6.005816
Eltd1	0.770911	4.096729	2.965738	0.009892	0.050159	-3.447353	4.132599	5.02617	4.90172	3.716922	4.562251	4.257812	4.085519	3.330656	4.18493	3.924277	3.220315	4.00459	3.772036	4.330935
Pnmal1	-0.694439	5.87191	-2.964574	0.009916	0.050255	-3.59821	5.935721	5.169685	5.482393	6.54334	5.436364	6.09241	5.919635	6.047627	6.052497	5.796076	6.068326	5.796318	6.237019	5.656972
Cdc25a	0.516081	3.672215	2.964149	0.009924	0.050277	-3.362597	3.509242	4.155166	3.684081	3.329127	3.917788	3.980827	3.734959	3.858761	3.696138	3.64312	3.467722	3.581545	3.445698	3.531125
Zswim5	-0.464898	4.762438	-2.963763	0.009932	0.050295	-3.514048	4.762823	4.459406	4.440757	4.833262	4.508156	3.939526	5.052933	4.989389	5.15669	4.902496	4.920843	4.923876	4.944745	4.898966
Slitrk1	-1.067492	6.814863	-2.963193	0.009943	0.050331	-3.611418	6.774395	5.184101	6.474812	7.857121	6.373831	6.948719	6.992135	7.028997	7.056581	6.860772	6.735475	6.841692	7.117639	7.17716
Mras	-0.412709	8.500634	-2.960709	0.009993	0.050562	-3.577076	8.956166	8.383959	8.747416	9.112441	8.599286	8.758175	8.187123	8.343469	8.213813	8.217339	8.360306	8.3123	8.491924	8.329543
Ankh	-0.589856	7.334147	-2.959762	0.010012	0.050637	-3.618327	7.367886	6.304905	7.04314	7.56843	7.078832	7.086111	7.388787	7.551634	7.500826	7.359858	7.397245	7.654472	7.707303	7.678747
Arhgef3	-0.802217	6.458028	-2.959394	0.010019	0.050653	-3.619114	6.799782	5.283087	6.193753	6.920818	6.401428	6.349726	6.96218	6.654346	6.39498	6.432321	6.754903	6.26635	6.722912	6.294562
Nudt16	-0.528468	3.733892	-2.956635	0.010075	0.05091	-3.427329	4.105711	3.74155	3.550922	3.980586	3.706477	3.084277	3.699086	3.606999	3.735714	3.808701	3.986003	3.941318	3.754753	3.692011
Ttf2	0.576014	3.324235	2.956451	0.010079	0.05091	-3.285487	3.205888	3.452688	3.404211	2.613062	3.391209	3.616909	3.280644	3.583227	3.518449	3.267546	3.414796	3.440827	3.098279	3.410483
Tmem229b	-0.377915	5.831327	-2.956241	0.010083	0.05091	-3.607622	5.806708	5.286909	5.433239	5.956321	5.652584	5.642839	5.964143	6.057725	5.84348	5.982299	6.114696	6.056147	5.998446	5.871116
Ifih1	0.923606	2.927538	2.95586	0.010091	0.050928	-3.227613	2.810225	3.200527	3.575619	2.241914	3.203302	3.693437	3.190506	2.901501	2.278164	2.956054	2.833517	3.668268	2.562371	2.087586
Enoph1	-0.462485	6.233059	-2.954907	0.01011	0.051004	-3.622809	6.256138	5.721836	5.683247	6.44259	6.321136	5.8251	6.442169	6.295728	6.48482	6.31014	6.40409	6.285093	6.428117	6.383975
Zmym6nb	1.12578	0.788032	2.95362	0.010136	0.051096	-2.735349	-0.046857	0.895377	0.785152	-0.166914	1.43972	0.695385	0.625837	1.356279	0.17081	1.419918	1.347792	1.433124	0.879431	1.158276
Trpc3	-0.695969	3.627987	-2.953473	0.010139	0.051096	-3.351949	3.621067	3.582736	2.423812	3.633342	2.944963	2.889836	4.394501	3.763261	4.18493	3.676473	3.66758	3.936334	3.994491	4.217057
Epn1	-0.378657	8.86431	-2.953379	0.010141	0.051096	-3.578111	9.479138	8.829884	9.008897	9.343467	9.040375	9.256804	8.599845	8.59498	8.590409	8.684416	8.693514	8.649918	8.68715	8.644953
Cntnap5b	-1.407755	2.474976	-2.951856	0.010172	0.051231	-3.098094	1.957182	2.064844	1.493674	3.309066	0.210178	1.450626	2.841442	3.14827	3.432752	2.572488	3.116419	2.594065	3.805994	3.022122
Bdkrb2	1.384326	1.265959	2.950337	0.010203	0.051346	-2.885096	1.108109	1.480065	2.778919	0.280432	1.72577	1.943815	0.498056	1.464337	1.29127	1.064403	0.721218	1.869448	1.001683	1.193114
Grm8	-1.005858	2.102091	-2.950321	0.010203	0.051346	-3.198391	2.357438	2.264105	2.09528	3.504019	1.173177	2.349037	1.938115	1.596873	2.04602	1.852296	1.652137	2.220493	2.317563	2.446395
Tnr	-0.954818	6.589092	-2.949498	0.01022	0.05141	-3.637306	6.393092	5.734484	6.284938	7.32795	5.303926	6.299194	6.786151	7.122676	6.972116	6.269188	6.751572	6.944557	7.406642	6.668691
Tmem50b	-0.607096	7.734095	-2.948856	0.010234	0.051432	-3.639492	7.45657	6.328272	6.846097	7.513242	7.380471	6.957021	8.057838	8.245587	8.073496	8.219604	8.277961	8.338747	8.373074	8.217668
Serpini1	-1.102792	8.06324	-2.948741	0.010236	0.051432	-3.624893	7.823721	6.270577	7.260554	8.944477	7.513961	8.07296	8.497311	7.997279	8.537973	8.274274	7.983898	8.228394	8.64419	8.842531
Cnpy4	0.504012	4.314134	2.948656	0.010238	0.051432	-3.501349	4.377065	4.755351	4.397084	3.893455	4.812994	4.565325	4.150713	4.323155	3.918782	4.556968	4.204441	4.390807	4.066299	4.065483
Pcdha3	-1.63644	1.037072	-2.948202	0.010247	0.051458	-2.871695	1.143792	-1.463779	0.20995	1.996424	0.266146	1.107027	1.297885	2.14364	1.60797	0.938085	1.803924	1.760874	1.837926	0.887553

Picalm	0.514508	7.086019	2.94726	0.010266	0.05153	-3.640261	7.010752	7.587272	7.780534	7.515067	7.817695	7.922371	6.538692	6.884922	6.639749	6.457483	6.697896	6.830319	6.96005	6.573565
RGD1562114	0.722071	3.120466	2.94689	0.010274	0.05153	-3.326915	3.311637	3.967899	3.510504	2.677071	4.035185	3.301961	2.955303	3.242343	2.583064	3.080873	2.986432	3.10114	2.689088	2.431883
Nefh	-0.712552	7.844228	-2.94654	0.010281	0.05153	-3.632624	7.869396	6.848762	7.32388	8.417871	8.052894	7.496725	7.997463	7.833131	8.158463	7.985511	7.793931	7.947046	8.069255	8.03199
Snrpb	0.618426	4.963534	2.946458	0.010283	0.05153	-3.547805	4.857199	5.169685	5.349789	4.106027	5.172806	4.657358	5.012583	4.905609	5.114779	5.136206	5.122035	5.058407	4.984828	4.89368
Epha5	-0.949183	7.139935	-2.946403	0.010284	0.05153	-3.64502	6.988232	5.60271	6.262296	7.534996	6.098369	7.281808	7.534324	7.492774	7.467913	7.222651	7.36883	7.548227	7.937912	7.63071
Pus3	0.543808	4.094705	2.946211	0.010288	0.05153	-3.48293	3.736577	4.570182	4.819324	4.163019	4.469939	4.131469	4.085519	3.910622	3.924502	4.015725	3.867914	3.816678	3.969738	3.937777
Timp4	-0.702718	3.980731	-2.946044	0.010291	0.05153	-3.46178	4.586045	3.96313	3.234684	3.829173	3.761059	3.084277	4.316148	3.878428	4.277224	4.178226	4.056804	3.961084	4.28674	4.421864
Fam219b	0.648051	3.333705	2.945797	0.010297	0.051534	-3.324941	2.977763	4.042133	3.702138	2.994284	3.306999	3.491064	3.083269	3.368235	3.495578	3.344264	3.331578	3.419555	3.372046	2.900911
Hivep3	-0.67278	5.603524	-2.945373	0.010305	0.051557	-3.635775	6.001624	5.56071	5.841308	6.450243	5.088003	5.725349	5.105949	5.837624	5.253025	5.27332	5.524588	5.864809	5.692921	5.263052
Ubtd1	0.765969	3.475568	2.943563	0.010343	0.051723	-3.343176	3.516065	3.702025	4.096187	2.568755	3.935155	3.365711	3.221182	3.386664	3.159628	3.566911	3.661913	3.83286	3.40196	3.388105
Espl1	1.970058	0.778922	2.942745	0.01036	0.051786	-2.813975	0.739674	1.655851	0.914692	-0.502853	3.075998	2.28453	0.429655	0.871929	0.571595	0.348395	0.138317	0.666985	-0.040977	0.799858
Snx6	0.442214	5.267643	2.94202	0.010375	0.051821	-3.614917	5.214725	5.404143	5.703557	5.283991	5.71801	5.934253	5.111417	5.062851	4.908035	4.902496	5.158711	5.13436	5.233825	5.017906
Trim5	0.627745	4.253418	2.94191	0.010377	0.051821	-3.481012	4.331374	4.51911	4.524328	3.400384	4.457973	4.389395	4.063116	4.692543	3.755102	4.760414	4.597784	4.846288	3.459987	3.836741
Plxdc2	-0.557048	5.573447	-2.941792	0.01038	0.051821	-3.632441	5.732483	5.361289	5.150497	6.028641	5.43941	5.356287	5.188472	5.808873	5.319649	5.464933	5.795641	6.238276	5.60564	5.571839
Ptpru	0.58575	6.92062	2.940777	0.010401	0.051903	-3.64713	6.200041	6.779923	6.253904	5.624215	6.60028	6.348149	7.792295	7.212579	7.637689	7.276393	7.185972	7.106395	7.179933	7.705387
Serpinb6	0.549215	4.941365	2.940593	0.010405	0.051903	-3.611682	5.174319	5.36852	5.473221	5.086016	5.910298	5.955127	4.571588	4.038501	4.60741	4.475935	4.670138	4.539538	4.631001	4.731318
LRRTM1	-1.088521	6.599989	-2.939013	0.010438	0.052046	-3.653438	6.463617	4.294561	5.74225	6.930699	6.29699	6.08106	7.211708	6.880052	7.258577	6.969718	6.879763	6.892112	7.20772	7.311055
Zfp180	-0.424873	7.138401	-2.938037	0.010458	0.052126	-3.663046	7.154887	6.709772	6.651318	7.309136	7.024089	6.843497	7.569519	7.117168	7.178867	7.22716	7.118272	7.231187	7.548376	7.265745
Dnajb5	-0.488743	7.824786	-2.937689	0.010466	0.052141	-3.644991	8.208017	7.402796	7.705871	8.363793	8.039156	8.042319	7.639799	7.787484	7.784078	7.748898	7.865321	7.63845	7.727008	7.60102
Stim1	-0.390079	7.034349	-2.937259	0.010475	0.052164	-3.664739	6.889876	6.242317	6.709384	6.893573	6.470058	6.584191	7.169731	7.387476	7.287715	7.420172	7.286524	7.422435	7.313806	7.416166
Got1	-0.592285	8.167182	-2.936917	0.010482	0.052179	-3.642349	8.342454	7.462728	7.903173	8.604154	8.25766	7.901284	8.284443	8.210456	8.353127	8.090077	8.456527	7.911616	8.479265	8.089176
Rxfp3	-1.492537	0.149167	-2.936665	0.010487	0.052183	-2.727266	-0.217457	-1.10215	-0.86368	1.097434	-0.257548	-1.26448	0.563361	0.517885	1.255413	0.348395	0.378374	0.151783	1.747621	1.445134
Nubp1	0.434706	5.239402	2.936456	0.010491	0.052184	-3.597887	5.002757	5.42163	5.283873	4.706082	5.39146	5.047769	5.482396	5.199424	5.395739	5.384467	5.323912	5.259352	5.14817	5.346662
Clec14a	0.751798	3.160348	2.935556	0.01051	0.052229	-3.340705	3.311637	3.801553	3.647273	2.57996	3.86892	3.390443	3.038038	2.619301	3.178898	3.00938	2.580719	3.361247	2.760099	3.278825
Cenpj	0.372553	4.870637	2.935422	0.010513	0.052229	-3.580426	4.70428	5.190235	4.919194	4.588954	5.010247	4.955481	4.750481	5.036833	4.575048	5.194928	5.052274	5.05151	4.539769	4.673894
Adamts9	1.148193	4.265934	2.935405	0.010514	0.052229	-3.474445	3.064101	4.892097	5.596606	4.217846	4.213017	4.512792	4.253222	4.438054	3.930199	4.290193	3.678847	4.653252	3.887547	4.182799
Birc5	1.501272	1.495394	2.934988	0.010522	0.052251	-3.00842	1.514695	2.643736	1.493674	0.785264	3.409963	2.932562	1.047044	1.155984	0.931093	0.981432	1.319276	1.284167	1.252133	0.799858
Sapcd2	1.013038	1.738309	2.933835	0.010547	0.052351	-2.968	1.341131	2.643736	1.687412	0.705659	1.947363	1.766229	2.01015	1.71825	2.00342	2.06872	1.290185	1.691577	1.922909	2.030937
Eif2b2	0.359322	5.352013	2.933533	0.010553	0.052358	-3.63044	5.333436	5.462754	5.699068	5.166265	5.657824	5.605038	5.183288	5.396437	5.310942	5.15289	5.361107	5.231141	5.217096	5.189473
Nap1l3	-0.952692	5.617269	-2.933357	0.010557	0.052358	-3.656249	5.812263	4.554415	5.258166	6.790636	5.533634	6.042569	5.497094	5.629	5.86446	5.27332	5.624113	5.275228	5.963768	5.55687
Map1b	-1.012024	11.00812	-2.933096	0.010562	0.052363	-3.545749	10.97045	9.869207	10.75933	12.20236	10.49762	11.16309	11.12392	11.12299	11.14202	10.56509	11.13507	10.94926	11.57088	11.04321
Enpp5	-0.630416	8.285331	-2.932762	0.01057	0.052377	-3.65431	8.10769	7.149329	7.888955	8.568561	7.872811	7.92078	8.302785	8.523397	8.71527	8.466007	8.402332	8.568306	8.67361	8.840129
Tbr1	-0.744903	4.740929	-2.931833	0.010589	0.052453	-3.627841	5.426916	5.240367	4.777357	5.640361	4.132497	5.032137	4.477313	4.823442	4.413615	4.373903	4.763054	4.677349	4.728657	3.927469
RGD1306772	-0.407265	5.242204	-2.931285	0.010601	0.052489	-3.636563	5.671142	5.224514	5.20058	5.359083	5.137606	4.86573	5.22677	5.138201	5.129711	5.345599	5.282044	5.214769	5.353466	5.283403
Kctd16	-1.27772	2.534646	-2.930808	0.010611	0.052508	-3.226807	2.706665	1.010791	2.10888	3.355447	1.390206	2.549011	2.598267	3.550909	2.973531	2.308845	3.173291	2.502617	3.372046	2.194616
LOC102549726	0.500299	3.824565	2.930695	0.010613	0.052508	-3.536455	3.992844	4.557582	4.850017	4.277563	4.36492	4.770022	3.454237	3.492551	3.424703	3.352542	3.568082	3.300483	2.994118	3.262515
Cntnap1	-0.377025	9.076063	-2.930032	0.010628	0.052556	-3.633373	9.161865	8.897995	8.849553	9.102155	8.760041	8.512418	9.416971	8.986073	9.370289	9.104119	8.932889	9.126649	9.387405	9.459432
Zic4	-1.272934	0.542246	-2.928857	0.010653	0.052622	-2.854088	1.212615	-0.273517	-0.575682	0.966949	-0.613814	1.07687	0.952703	0.923327	0.571595	1.451089	1.375754	0.567345	0.598632	0.554014
Nrgn	-0.839453	11.31612	-2.928821	0.010653	0.052622	-3.527502	11.99371	10.71216	11.20624	12.45496	11.83247	11.78831	11.03175	11.18172	11.26253	10.99762	11.2974	10.92151	11.12677	10.61923
Sugp2	-0.42557	6.767327	-2.928763	0.010655	0.052622	-3.681502	6.826102	6.209471	6.695653	7.074294	6.590037	6.703099	6.874036	6.826216	6.848951	6.764796	6.818046	6.704786	7.098357	6.823329
Cdca7l	1.333488	2.260975	2.928514	0.01066	0.052622	-3.077965	1.594228	2.082541	2.200654	1.327578	3.238702	3.513754	2.550752	2.307359	2.223535	1.804823	2.684976	2.2368	2.186205	2.050068
Col26a1	-1.771213	-0.275141	-2.92838	0.010663	0.052622	-2.731936	1.371547	-0.465897	-0.665442	-0.325128	-1.087961	-2.070862	0.798508	0.04768	0.787836	0.534523	-0.731011	-0.657145	0.375586	0.554014
Fam120b	-0.608723	6.815711	-2.927935	0.010672	0.052648	-3.682591	6.759378	5.821369	6.400036	7.225541	6.574189	6.737845	6.960666	6.967583	6.998792	6.835157	7.068276	6.89018	7.213003	6.982409
Cflar	0.544596	4.130886	2.926868	0.010695	0.052739	-3.560001	4.264063	4.658378	5.145557	4.253272	4.380852	5.004367	3.837517	3.865347	3.742206	3.753509	3.83304	3.650119	3.83917	3.698087
Rab3a	-0.839961	9.54527	-2.926038	0.010713	0.052791	-3.604219	10.05091	8.705484	9.590433	10.5899	9.792096	9.83765	9.300753	9.383651	9.568118	9.117539	9.653374	9.134422	9.693029	9.218605
Fntb	-0.543345	4.85941	-2.925924	0.010716	0.052791	-3.607713	4.930537	4.320894	4.092769	5.149441	4.875052	4.719677	4.977075	5.016268	4.843082	4.908123	5.10543	5.037614	5.105631	5.005709
Casp8	1.133718	1.802928	2.925579	0.010723	0.052791	-3.051243	2.127257	2.264105	2.2133	0.577356	2.308867	2.854719	1.668148	2.436799	1.255413	2.239124	1.783193	2.118577	0.698429	1.193114
Hmgcs1	-0.560632	7.997608	-2.925556	0.010723	0.052791	-3.654418	8.758875	7.797436	8.216559	8.86283	8.386149	8.604643	7.982991	7.023119	8.047195	7.186643	7.381384	7.273299	8.347841	8.104126
Pigv	0.479307	2.887516	2.924368	0.010749	0.052896	-3.274107	2.595092	3.06466	3.042552	2.511387	2.910256	3.084277	2.893159	3.14827	2.707706	2.934159	2.977336	3.018661	2.84965	2.900911
Pdzd8	-0.595618	6.514517	-2.923607	0.010765	0.052955	-3.689878	6.583775	5.684659	6.464931	7.032594	6.274994	6.424226	6.561847	6.513661	6.554714	6.350995	6.492147	6.772767	6.795137	6.714376
Lig4	-0.637148	5.282967	-2.923272	0.010773	0.052969	-3.63047	4.902169	4.902098	4.195136	5.677346	4.578921	4.988255	5.801115	5.526777	5.699127	5.526589	5.513618	5.419718	5.621619	5.652285
Scarb2	-0.704891	7.205344	-2.920846	0.010825	0.053205	-3.690202	7.8019	6.739359	7.055923	7.736508	6.886346	7.578706	6.964448	7.101913	6.911487	7.019955	6.818046	7.659395	7.321653	7.290087
Cilp2	-0.74128	2.715022	-2.920571	0.010831	0.053213	-3.238104	2.718545	2.309899	2.162035	2.758225	1.745593	1.839907	3.428178	2.939445	3.324439	3.377094	2.750518	3.136336	2.805569	2.972849
Inpp1	-0.477823	4.047808	-2.918713	0.010872	0.05339	-3.548141	4.167689	3.854016	3.8018	4.520817	3.986037	3.857641	4.258161	4.112335	4.060584	4.005262	4.265502	3.816678	4.200009	3.858608
Fam19a5	-0.450053	7.35409	-2.918507	0.010876	0.05339	-3.698829	7.546796	6.624	7.139467	7.37936	7.150749	7.141341	7.626014	7.439707	7.405889	7.340221	7.527986	7.491169	7.656248	7.498119
Faf1	0.366011	5.397609	2.918118	0.010885	0.053407	-3.650679	5.057571	5.56701	5.338289	5.136218	5.554855	5.396521	5.482396	5.368893	5.595383	5.478234	5.500978	5.24128	5.431678	5.454694
Lrrc4c	-0.699382	6.126697	-2.917938	0.010889	0.053407	-3.693828	6.092305	4.982063	5.875525	6.739089	5.991401	6.010022	6.350332	6.148265	6.332896	6.108631	6.16564	6.167734	6.386642	6.446674
Mink1	-0.431828	10.21669	-2.917373	0.010901	0.053446	-3.613459	10.24591	9.908342	10.02756	10.55355	9.783207	10.14949	10.47317	10.29667	10.45968	10.28666	10.07571	10.12474	10.21442	10.43588
H2afy2	-0.396926	4.321658	-2.915393	0.010944	0.053637	-3.606981	4.689266	4.332034	4.564365	4.826222	4.165982	4.383247	4.040359	4.279444	4.213244	4.154751	4.168924	4.296718	4.152409	4.315206
Ccdc22	0.552249	4.529678	2.914929	0.010954	0.053665	-3.548681	4.145858	4.771807	4.579097	3.82447	4.581681	4.196023	4.828927	4.632039	4.726784	4.882627	4.49636	4.496167	4.491894	4.831464
Efcc1	-1.184998	0.333587	-2.914381	0.010966	0.053693	-2.788769	0.592497	-0.273517	-0.86368	0.33436	0.027932	-1.26448	1.443753	0.705741	1.393785	0.591587	0.961591	0.514819	1.001683	0.970221
Chst8	-0.601722	4.560207	-2.914217	0.01097	0.053693	-3.548551	4.193457	3.354134	3.711083	4.479531	4.220119	3.663311	4.977075	4.7399	5.119774	5.057375	4.974403	5.035286	5.023827	5.367782
Gatsl2	-0.751822	5.478969	-2.914061	0.010974	0.053693	-3.692205	5.802528	4.936565	5.496689	6.449479	5.141352	5.918397	5.286006	5.217552	5.237026	5.067468	5.39566	5.456736	5.908834	5.427521
Epn2	-0.443101	8.023713	-2.912864	0.011	0.0538	-3.698334	7.98856	7.238365	7.675346	8.147844	7.68058	7.908793	8.347789	8.156438	8.288065	8.139947	8.152748	8.154943	8.245867	8.212901
Nfe2l2	0.673008	5.198844	2.911066	0.01104	0.053972	-3.664349	5.551702	5.494499	5.702436	4.514991	5.872485	5.708237	4.971071	5.170467	4.700356	5.301385	4.992577	5.544509	4.56643	4.737232
Kif13b	-0.567948	5.512855	-2.910181	0.011059	0.054029	-3.675491	5.466203	5.425102	4.880071	5.616073	4.598129	5.014346	5.892891	5.840969	5.832874	5.682467	5.31852	5.836999	5.881226	5.930391
Trex1	0.734554	4.074072	2.910132	0.01106	0.054029	-3.526324	4.137033	4.486247	4.785848	3.329127	4.578921	3.931123	4.07995	3.891392	3.696138	4.264103	4.254248	4.12775	3.860871	3.710165
Mycbp	0.636901	3.272789	2.909922	0.011065	0.05403	-3.433172	3.639931	3.673115	3.814306	2.768056	4.01491	3.751866	2.67414	3.070611	2.984524	3.12019	3.50618	3.083214	2.892424	2.992761
Cacng7	-0.319539	6.497556	-2.909615	0.011072	0.054041	-3.719075	6.950321	6.633786	6.544026	6.887386	6.530581	6.690084	6.361849	6.244866	6.374233	6.357179	6.174622	6.368863	6.387577	6.477914
Amph	-0.792508	5.862925	-2.908485	0.011097	0.054142	-3.711595	6.1228	5.15303	5.747694	6.758944	5.882591	5.80799	5.570406	6.00353	5.871879	5.384467	6.061886	5.54123	6.431746	5.763674
Clu	0.850361	11.20593	2.908277	0.011102	0.054142	-3.604411	11.35442	11.71706	11.46749	10.01472	11.8249	11.14861	10.57898	11.65809	10.71804	11.60559	11.42813	11.71921	10.54814	11.10039
Slc22a4	2.066991	0.371526	2.907579	0.011117	0.054196	-2.71256	-0.217457	0.533043	1.669168	-1.788967	0.470544	0.386712	0.028455	1.279465	0.007959	0.799637	0.321999	1.252437	1.347449	0.606649
P4ha1	-0.701032	6.053423	-2.907081	0.011128	0.054228	-3.714894	6.251035	5.080024	5.514681	6.572382	6.038362	6.206485	6.084426	5.965724	6.260125	6.033395	5.835201	6.075543	6.37632	6.478797
Sepsecs	0.494094	4.348618	2.90624	0.011147	0.054298	-3.574896	4.231267	4.80949	4.483148	3.902409	4.573386	4.361521	4.228269	4.067347	4.473365	4.417954	4.287749	4.496167	4.23782	4.388433
Rprml	-0.682733	5.604694	-2.904663	0.011182	0.054448	-3.683758	5.504448	4.200444	4.798493	5.605147	5.103448	5.373446	6.094126	5.928474	6.082225	6.118373	6.16163	5.88435	5.916626	5.729965
Kif2c	2.598648	0.034689	2.903681	0.011204	0.054533	-2.746136	-0.899176	1.558038	-0.007587	-0.941548	2.472441	2.101918	-0.399109	-0.26106	-0.632503	-0.311823	-0.318799	0.567345	-1.047095	0.442608
Mmd2	-0.499692	7.975022	-2.902937	0.011221	0.054584	-3.71813	8.257972	7.545213	7.292192	7.872296	7.677681	7.750037	7.946356	8.0055	7.939038	8.362989	8.162061	8.465458	8.148592	8.231351
Pde7b	-0.912789	2.976184	-2.90281	0.011224	0.054584	-3.445698	3.145563	3.142653	3.006676	4.242735	2.014259	3.091837	2.566765	2.76729	2.984524	2.966878	3.196995	2.951084	3.051863	2.745289
Ermp1	-0.479331	6.516431	-2.902097	0.01124	0.054622	-3.730038	6.354591	5.833178	6.147553	6.845056	6.188025	6.315429	6.723247	6.548861	6.731514	6.419574	6.581154	6.8073	6.893831	6.858344
Fgf14	-1.367293	2.673227	-2.902058	0.011241	0.054622	-3.442187	3.163058	1.480065	3.049621	4.632654	2.16907	3.456346	2.415815	2.951874	2.364852	1.898256	2.823399	2.082939	3.125428	2.087586
Rabgap1l	-1.144886	3.96588	-2.901671	0.011249	0.054628	-3.623217	4.453699	3.151065	4.179109	5.567645	3.452798	4.672562	3.392685	4.078726	3.860303	3.232094	4.004032	3.536147	4.498832	3.551406
Prss12	-1.090017	4.276212	-2.901421	0.011255	0.054628	-3.607599	4.435818	4.418186	3.131857	5.206584	2.919011	4.578838	4.017238	4.299035	4.380707	4.673881	4.316888	4.744468	4.453131	4.380898
Rpp21	0.950575	3.753198	2.901398	0.011256	0.054628	-3.497	4.021899	4.749824	4.235992	2.797152	4.659497	3.68846	3.419386	3.368235	3.748669	3.896239	3.61576	3.509549	3.48116	3.475607
Kcnv1	-1.427787	4.898022	-2.901124	0.011262	0.054636	-3.623617	4.997891	1.747452	4.191945	5.372033	4.539721	4.440619	5.261619	5.275563	5.743484	5.229048	5.314913	5.251348	5.653054	5.631797
Tas1r3	1.946835	-0.722883	2.900837	0.011268	0.054645	-2.612467	-1.22354	-0.025882	1.089503	-2.040259	-0.424712	-0.750296	-0.169545	-0.654381	-0.175642	-0.420466	-0.616402	-0.920914	-0.405292	-0.621773
Nrsn2	-0.639327	6.960327	-2.899077	0.011308	0.054816	-3.738881	7.119481	6.261865	6.870754	7.272052	6.77622	6.316236	6.578725	7.168661	7.223628	7.300125	6.974241	6.974647	7.260718	7.360263
Erap1	0.48233	5.075767	2.896338	0.01137	0.055075	-3.665164	4.851815	4.965467	5.247446	4.580611	5.245957	5.310045	5.193638	5.178422	5.151822	4.968612	4.935008	5.442746	4.924278	5.111939
RT1-A1	2.243328	4.305538	2.896321	0.011371	0.055075	-3.415842	3.736577	4.397125	4.299641	1.740915	6.854181	4.116722	3.669732	5.057109	3.592171	5.213226	4.79447	5.597563	3.549571	3.769079
Nsf	-0.887195	9.43241	-2.895475	0.01139	0.055147	-3.681282	9.560703	8.141958	9.178714	10.13462	9.250603	9.275181	9.40686	9.716104	9.705365	9.162959	9.805921	9.402254	9.957969	9.356938
Nkain4	-0.922974	4.227288	-2.895222	0.011396	0.055152	-3.555263	4.883821	3.752647	3.431527	3.675737	3.517641	2.705027	4.416758	4.402272	4.787674	4.788142	4.512193	4.848943	4.579577	4.976008
Ccni	-0.369971	7.707444	-2.894854	0.011404	0.055171	-3.735899	8.029969	7.644243	7.421116	7.837706	7.549283	7.641866	7.899313	7.602285	7.625011	7.768364	7.693009	7.835358	7.637904	7.726351
Cntn5	-1.04104	2.378376	-2.894461	0.011413	0.055174	-3.295111	2.41698	2.452828	2.225837	3.36843	1.018831	1.892796	2.398025	2.505864	3.120298	2.694725	2.213372	2.100868	2.783013	2.431883
Pygo1	-0.782169	2.853191	-2.894427	0.011414	0.055174	-3.487867	3.417469	2.667383	3.319014	4.163019	2.828971	3.248852	2.324585	2.724465	2.597457	2.108365	2.883067	2.346065	3.186856	2.357047
Vamp2	-0.613794	10.64183	-2.894117	0.011421	0.055181	-3.621643	11.25132	10.44786	10.90828	11.66815	10.84602	11.18155	10.23216	10.40029	10.44936	10.22663	10.59579	10.10793	10.54065	10.13064
Ip6k2	-0.390149	6.838667	-2.893958	0.011425	0.055181	-3.7497	6.834315	6.531193	6.565762	7.21075	6.629246	6.803772	6.95231	6.959911	7.026314	6.921151	6.907565	6.726602	6.838166	6.848133
Dnm2	0.44671	7.457147	2.893123	0.011444	0.055234	-3.747646	7.346033	7.879135	7.710896	7.002201	7.599164	7.292919	7.402236	7.556729	7.329809	7.594314	7.457773	7.649912	7.217215	7.370763
Bcl2l2	-0.377431	8.028466	-2.893076	0.011445	0.055234	-3.724938	8.355353	8.14011	8.142451	8.675749	7.963414	8.302858	7.978883	7.607205	7.939744	7.75203	7.783205	7.683019	8.076229	8.004364
Scn7a	2.692697	1.927853	2.891326	0.011485	0.055406	-2.960222	-0.310971	2.545069	1.905712	0.932396	3.174343	4.213454	0.743216	3.878428	0.837186	2.681644	2.166061	3.744381	0.491415	0.606649
Casc4	0.486232	8.008835	2.890668	0.0115	0.055457	-3.733254	7.955371	8.250887	8.490525	8.196885	8.60828	8.900162	7.494592	7.799569	7.582684	7.621541	7.785813	7.954769	7.811276	7.677594
Klf2	0.91063	3.749143	2.890149	0.011512	0.055461	-3.523098	3.870393	4.822713	4.583975	3.010938	4.023054	3.892687	4.134688	3.252427	3.907275	3.62956	2.823399	3.502822	3.515771	3.642453
Unc79	-0.544659	6.620675	-2.890115	0.011513	0.055461	-3.750688	6.204272	5.929223	6.139293	6.718324	5.574383	6.027866	7.46125	7.000114	7.256878	6.87449	6.761542	6.76013	6.970069	7.028954
Epm2a	-1.29683	1.343709	-2.89003	0.011515	0.055461	-3.085725	1.764345	0.310827	1.669168	2.154676	0.266146	0.527154	1.335758	1.958936	1.819202	0.799637	1.94125	1.593679	2.348615	1.009844
Il17rb	1.948535	0.399493	2.889602	0.011525	0.055476	-2.775536	-0.760787	-0.366503	1.143363	-0.81875	1.745593	1.402804	0.028455	0.58326	0.736738	0.280604	0.676952	0.714338	1.11438	0.442608
Tdp1	0.647396	3.482525	2.889499	0.011527	0.055476	-3.466432	3.303772	4.112733	3.545931	3.019194	3.935155	3.632543	3.419386	3.534475	3.518449	3.545408	3.421519	3.368666	2.892424	3.648741
Smpd2	-0.649349	6.005801	-2.88881	0.011543	0.055509	-3.730634	5.713314	4.535265	5.336845	5.92151	5.529352	5.258917	6.568821	6.405985	6.803893	6.484099	6.199283	6.400874	6.253519	6.697034
Snrpa	0.711248	4.735993	2.888769	0.011544	0.055509	-3.669323	4.862563	5.267699	5.503141	4.246256	5.615361	4.634248	4.394501	4.446862	4.485023	4.896847	4.50904	4.539538	4.55649	4.407101
Dgkz	-0.495743	10.55779	-2.888595	0.011548	0.055509	-3.668436	10.63854	9.906324	9.831749	10.25682	9.907812	10.16822	11.04851	10.87957	10.89164	11.11247	10.62357	10.91353	10.63808	10.99335
Fbxo2	-0.393198	6.762833	-2.888014	0.011562	0.055527	-3.761125	7.164147	6.60116	6.611266	6.762022	6.581101	6.487206	6.594433	6.818601	6.867627	6.875926	6.76551	7.062468	6.759477	6.743314
Cdk20	0.673669	4.481187	2.887859	0.011565	0.055527	-3.626678	4.243653	5.34305	5.025202	4.066741	4.576156	4.364645	4.36733	4.351577	4.449761	4.623466	4.543347	4.212646	4.20426	4.436477
Nlgn1	-0.851383	6.099825	-2.887714	0.011569	0.055527	-3.753855	6.123917	4.972603	6.012072	6.990178	5.664348	6.165876	6.059178	6.33184	6.341473	5.768502	6.062962	5.992746	6.591169	6.344811
Alb	-1.172686	2.079043	-2.887638	0.01157	0.055527	-3.289983	2.987615	2.874583	2.039558	2.748327	1.664612	0.386712	2.12275	1.71825	2.107651	2.14695	2.317912	2.082939	2.060555	2.261799
Rbfox3	-0.897335	7.352894	-2.886777	0.01159	0.055601	-3.748086	7.825439	6.66569	7.714793	8.652222	7.193772	7.790087	6.894006	7.574421	7.127102	6.921151	7.391692	7.095817	7.370694	6.733732
Lca5	-0.562757	3.088114	-2.886099	0.011606	0.055654	-3.526127	3.95819	3.091129	3.222225	3.701615	3.028244	3.708268	2.659281	2.505864	2.772565	3.050666	2.968181	2.9008	2.944167	2.91141
Gpc1	-0.467009	7.917941	-2.885152	0.011628	0.055735	-3.764662	7.561713	6.593465	7.181103	7.241065	7.134865	6.832258	8.521904	8.494216	8.557199	8.528075	8.386957	8.67538	8.531999	8.618564
Dcaf12	-0.802679	5.29943	-2.884866	0.011635	0.055735	-3.732515	5.435939	4.271601	4.924971	6.12167	5.345112	5.36255	5.361385	5.183701	5.544292	5.052302	5.4978	5.014154	5.849014	5.269187
Sec23a	-0.762995	6.429235	-2.884781	0.011637	0.055735	-3.764965	6.427881	5.601176	6.319711	7.277654	5.966279	6.47063	6.4258	6.576215	6.693497	6.198623	6.632037	6.265356	6.891856	6.281469
Kdelr2	0.372704	5.568873	2.884531	0.011643	0.05574	-3.724198	5.146006	5.608829	5.602627	5.290827	5.695256	5.460695	5.592083	5.627064	5.604319	5.700414	5.444337	5.757285	5.666979	5.800855
Epn3	-1.94082	0.625482	-2.883757	0.011661	0.055792	-3.018036	3.287913	-0.572649	-0.263851	0.531921	0.266146	0.435061	0.498056	0.76329	0.245823	0.938085	1.883989	0.714338	1.077784	0.119416
Zmat3	-0.625353	6.580966	-2.883665	0.011663	0.055792	-3.768806	6.667367	5.924325	6.46427	7.317528	6.55255	6.526088	6.705276	6.478654	6.479002	6.375573	6.705491	6.536323	6.991131	6.426686
Wdr83os	0.530617	5.140133	2.882505	0.01169	0.0559	-3.68945	4.686244	4.965467	5.186218	4.664582	5.690151	4.971961	4.994938	5.212396	5.237026	5.264574	5.419364	5.269295	5.217096	5.227782
Inpp5j	-0.408113	7.162481	-2.881666	0.01171	0.055972	-3.773487	7.014973	6.900813	6.745749	7.196715	6.69125	6.454594	7.572496	7.390333	7.60734	7.446995	7.242591	7.163491	7.355009	7.503767
Scrt1	-0.498832	5.433966	-2.880342	0.011741	0.056098	-3.75407	6.031695	5.645011	5.421025	5.759717	5.340231	5.186625	5.321831	5.207221	5.104739	5.208673	5.293084	5.539588	5.713941	5.338905
Gps2	0.437979	5.919875	2.879721	0.011755	0.056145	-3.765453	5.96967	6.484532	6.316051	5.845784	6.350559	6.228942	5.829598	5.798587	5.762769	5.868575	5.581226	5.687812	5.328373	5.852167
Pawr	1.158201	1.578221	2.879101	0.01177	0.056178	-3.182713	2.10932	2.01041	3.381979	1.000693	2.322129	2.809587	1.135593	1.393205	0.628769	1.355481	0.998029	1.219993	1.11438	1.193114
Gjc1	0.761482	2.364309	2.879033	0.011771	0.056178	-3.253821	1.9771	2.532247	2.753168	1.945353	2.874693	2.665044	2.814871	1.80302	2.313467	2.586593	2.049375	2.63155	2.269694	2.194616
Nip7	0.624202	3.124143	2.878352	0.011787	0.056232	-3.44662	2.917193	3.452688	3.941366	2.977436	3.534831	3.314938	3.251219	2.781287	3.23521	2.911926	3.173291	2.726993	2.760099	2.942457

Nr3c1	-0.42432	6.510653	-2.877779	0.011801	0.056254	-3.780408	6.684126	6.155101	6.369289	7.033103	6.528437	6.685095	6.44542	6.581224	6.338262	6.408699	6.543128	6.554326	6.450653	6.389618
Brinp1	-1.02265	7.847652	-2.877761	0.011801	0.056254	-3.767276	7.903623	6.043193	7.486392	8.480497	7.214368	7.93556	7.917379	8.224931	8.149334	7.947421	8.17927	8.191483	8.088066	8.11325
Hap1	-0.400034	7.334476	-2.877405	0.01181	0.056272	-3.778705	7.35605	6.843581	7.273443	7.577543	7.128274	7.022268	7.51072	7.169326	7.586297	7.289386	7.21238	7.224048	7.653142	7.846112
Tmem140	1.001882	2.054869	2.876776	0.011825	0.056321	-3.103289	1.717752	2.607522	2.08155	0.621404	1.877213	1.927009	1.962529	2.345542	1.690848	2.854798	2.506778	2.816513	2.236876	1.931282
Ttc4	-0.53353	3.558357	-2.876547	0.01183	0.056324	-3.595476	3.992844	3.167741	3.609501	4.263733	3.8553	3.747086	3.488265	3.660989	3.31576	3.186517	3.434871	3.277021	3.549571	3.403062
Ranbp6	-0.720097	5.027492	-2.876275	0.011837	0.056333	-3.721705	4.930537	3.982112	4.703085	5.726071	4.789315	4.961683	5.246787	4.937208	5.190313	4.891176	5.233205	5.004662	5.58623	5.252767
Oscp1	-0.490151	4.558706	-2.876034	0.011842	0.056338	-3.684059	4.883821	3.879549	4.464755	4.72387	4.469939	4.413727	4.543542	4.552657	4.575048	4.616609	4.741723	4.597515	4.885102	4.541596
Gabrb2	-1.191988	3.685031	-2.874777	0.011872	0.056457	-3.49916	3.439172	2.36878	3.47943	4.228565	1.822268	2.915623	3.545949	4.490115	4.189688	3.676473	4.53717	4.281077	4.689778	4.074854
Pcdh7	-0.730716	4.96373	-2.873304	0.011907	0.056601	-3.736161	5.274296	4.232503	5.107108	5.625567	4.499426	5.024257	4.977075	4.892772	5.084445	4.277207	4.930302	4.968501	5.566555	5.084367
Sptb	-0.869589	7.512195	-2.871289	0.011955	0.056755	-3.787124	7.574435	6.323629	7.232872	7.950999	6.458844	7.555998	7.784185	7.894382	7.755082	7.343858	7.742844	7.805945	8.230841	7.526142
Trak1	-0.336672	7.76294	-2.871193	0.011958	0.056755	-3.778207	8.020102	7.778221	7.675632	8.158861	7.67639	7.879337	7.807536	7.586429	7.595737	7.651203	7.659278	7.632684	7.847733	7.719272
Parl	0.405069	4.804064	2.871191	0.011958	0.056755	-3.685824	4.485336	4.938996	5.039476	4.405886	4.805931	4.608127	4.802122	4.930944	4.815644	5.072488	4.872595	4.81947	4.885102	4.831464
Slc30a9	-0.350833	6.820034	-2.87116	0.011958	0.056755	-3.793904	6.908796	6.418132	6.614249	6.960477	6.621221	6.681968	7.090949	6.736566	6.872074	6.734238	6.933078	6.84502	7.120453	6.957296
Sobp	-0.768572	6.659699	-2.870571	0.011972	0.056777	-3.790244	7.331601	6.509279	7.042697	7.941529	6.556763	7.359412	5.962631	6.575211	6.106329	6.096361	6.500902	6.425981	6.760199	6.083308
Lysmd1	-0.395052	4.389325	-2.870571	0.011973	0.056777	-3.681822	4.671039	3.996186	4.352047	4.616421	4.342316	4.291029	4.421169	4.420274	4.372361	4.373903	4.44779	4.300602	4.605518	4.315206
Fgf13	-0.917724	7.875605	-2.87024	0.01198	0.056792	-3.774802	8.400763	6.533608	7.540085	8.523932	8.205387	7.906919	8.102119	7.649789	8.00463	7.601278	7.987584	7.64075	8.217761	7.950976
Cebpa	0.70226	4.403588	2.86932	0.012002	0.056875	-3.616655	3.646165	4.073446	4.305558	3.972106	4.979188	4.6901	3.699086	4.899205	3.924502	4.963216	5.0566	5.417931	4.089463	4.012827
Akr1b1	0.48919	5.208016	2.868734	0.012017	0.056919	-3.725145	5.012441	5.198375	5.322324	4.560953	5.586799	4.969911	5.141132	5.526777	5.089545	5.318389	5.552419	5.414351	5.156968	5.105095
Kcnh1	-1.251838	5.615017	-2.867867	0.012037	0.056973	-3.7568	5.635554	2.982209	5.058875	6.126454	5.062547	5.354716	6.010249	6.175018	5.963625	5.852614	6.011533	5.943917	6.371603	6.102907
Wnt4	-1.403923	4.115475	-2.867867	0.012037	0.056973	-3.531045	3.848935	0.632508	3.458336	4.291262	3.440689	3.02233	4.828927	4.88307	5.008391	4.542569	4.647377	4.797651	5.208658	5.161159
Mst4	-1.453978	0.932483	-2.866919	0.01206	0.057035	-3.037349	1.310059	-1.463779	0.975347	1.818649	1.018831	0.175273	1.782265	1.393205	1.520039	0.981432	0.961591	1.345607	1.1849	1.085968
Kif15	0.806873	2.859915	2.866792	0.012063	0.057035	-3.379687	2.246933	3.9244	3.077557	2.634716	3.252623	2.809587	3.003159	2.795149	2.984524	2.614395	2.415434	2.816513	2.827778	2.858134
Cyp7b1	-0.545444	5.329933	-2.866733	0.012065	0.057035	-3.756735	5.312032	4.649501	4.89192	5.653679	5.163626	5.05165	5.206471	5.869094	5.351842	5.530243	5.285734	5.925172	5.3611	5.407263
Peli3	-0.496383	3.701672	-2.866096	0.01208	0.057071	-3.611899	3.95819	3.404252	3.855228	4.217846	3.665442	3.444586	3.504983	3.645768	3.860303	3.493953	3.786926	3.60686	3.929227	3.571405
St8sia3	-1.14506	5.158729	-2.865949	0.012084	0.057071	-3.756203	5.216821	3.801553	5.088351	6.265671	4.412197	5.065155	4.802122	5.63672	5.293368	4.744776	5.457481	5.22093	6.093166	5.172115
Fam188a	-0.445741	6.15886	-2.865828	0.012087	0.057071	-3.801773	6.486379	5.94261	6.272907	6.866376	6.244843	6.463363	6.232308	6.018379	6.112605	5.881504	5.965121	5.852956	6.011844	5.895119
Pdgfd	-1.073579	0.444085	-2.865548	0.012093	0.057081	-2.956977	1.541696	-0.186163	0.049949	0.33436	0.266146	-0.548781	0.851758	0.705741	0.837186	0.799637	0.535373	0.889587	0.598632	0.753927
Aspm	0.966287	2.459391	2.863885	0.012134	0.057248	-3.293075	1.9771	3.472958	2.200654	1.945353	2.944963	3.129052	2.841442	2.16515	2.39812	2.484835	2.133674	2.475388	2.186205	2.372328
Syn2	-0.996311	8.62783	-2.863296	0.012148	0.057271	-3.767342	8.871404	7.273459	8.537482	9.515636	8.42143	8.560204	8.503044	8.782149	8.900427	8.222994	8.972199	8.443671	9.259344	8.530424
Fermt2	0.438504	7.654648	2.863294	0.012148	0.057271	-3.79809	7.648886	8.217737	8.179981	7.570538	7.796768	7.998247	7.774307	7.286206	7.574522	7.486552	7.116201	7.431318	7.414917	7.676825
Phyhipl	-0.42698	7.08148	-2.86302	0.012155	0.05728	-3.803535	7.494205	7.024991	7.090609	7.833616	7.358513	7.471593	6.718776	6.836307	6.854954	6.846923	6.966792	6.838356	6.999104	6.817758
Casp2	0.676398	4.554368	2.86215	0.012176	0.057339	-3.680783	4.193457	5.3793	5.086634	4.185201	4.55103	4.449473	4.757474	4.446862	4.610962	4.553382	4.404331	4.346409	4.225326	4.639574
Sipa1l3	-1.124075	7.640481	-2.86211	0.012177	0.057339	-3.797948	7.934512	5.790744	7.152659	8.458468	7.407942	7.938183	7.706815	7.984858	7.68563	7.71479	7.90364	7.703698	7.860581	7.733025
Zdhhc6	0.40121	5.423134	2.861101	0.012202	0.057433	-3.755606	5.103599	5.474531	5.368284	4.906296	5.616707	5.158103	5.584238	5.445606	5.430321	5.555561	5.519896	5.523062	5.599199	5.67557
Dio2	-0.754548	2.815544	-2.860172	0.012224	0.057517	-3.562338	3.742407	3.302211	3.359399	3.906865	2.962008	2.695134	2.305626	1.80302	2.241976	2.202958	2.707156	3.18757	2.881849	2.357047
Rps25	0.789333	2.064811	2.85827	0.012271	0.057693	-3.3348	2.144973	3.167741	2.861581	2.154676	3.372207	2.349037	1.888013	1.68885	1.690848	1.104165	1.456531	1.515634	1.922909	1.991897
Prpf3	0.454265	5.914163	2.858258	0.012271	0.057693	-3.804392	5.707365	6.332901	6.119273	5.912674	6.358626	6.249365	6.054927	5.691483	5.802136	5.991604	5.616809	5.633419	5.493764	5.860318
Ubxn4	0.357476	7.049429	2.857559	0.012288	0.05772	-3.818424	7.007124	7.339806	7.493862	7.135854	7.335399	7.547089	6.642444	6.949102	6.823923	6.806821	6.830729	7.005312	6.963188	6.823329
Mdh1	-0.557892	8.982535	-2.857484	0.01229	0.05772	-3.774337	9.206181	8.23836	8.751215	9.182111	9.027402	8.534603	9.240728	8.979068	9.286853	8.824901	9.285225	8.795495	9.341835	9.064706
Galk1	0.71322	4.182871	2.857433	0.012291	0.05772	-3.613122	4.096635	4.628571	4.269682	3.182467	4.600852	3.839794	4.102096	4.244507	4.347031	4.281549	4.359523	4.357637	4.13915	4.20003
Gpr34	-1.057264	3.937953	-2.856049	0.012325	0.057858	-3.64134	4.689266	2.631765	2.735741	4.140492	4.102065	3.94371	3.699086	4.284367	3.207329	4.494755	4.400933	5.087922	3.866246	3.96323
Mtmr10	0.535273	5.361118	2.855454	0.01234	0.057881	-3.771693	5.28032	5.839697	5.755281	4.814411	5.388311	5.329359	5.295647	5.347885	5.029775	5.491413	5.237021	5.66389	5.272795	5.348595
Egfl7	1.179183	4.712609	2.855368	0.012342	0.057881	-3.643065	4.307974	5.514194	5.170091	3.152759	4.936707	3.918424	4.946803	4.905609	5.000289	5.249138	4.711869	4.758654	4.360956	5.109662
Ror2	-0.916628	2.664839	-2.855258	0.012345	0.057881	-3.349257	2.885927	1.248835	1.825611	2.62393	1.745593	2.515676	2.644268	2.976416	3.416608	3.530893	2.823399	3.028062	2.994118	3.326676
Trpc1	-0.440078	5.134577	-2.854474	0.012364	0.05795	-3.746759	4.777095	4.256089	4.620039	5.222759	4.705768	4.66498	5.556439	5.407759	5.436339	5.402519	5.311298	5.461948	5.571499	5.536666
Parp16	0.663667	2.449095	2.854098	0.012374	0.05797	-3.281147	2.054131	2.619695	2.682163	1.720809	2.661692	2.160607	2.613764	2.849305	2.430637	2.694725	2.568656	2.827322	2.236876	2.460763
Stat2	0.471313	6.041071	2.853562	0.012387	0.058004	-3.811708	5.748506	6.466111	6.318248	5.763408	6.236111	5.888325	6.053507	6.025747	5.840457	6.081498	5.923101	6.395437	5.863867	5.99475
Smg9	0.504707	5.237881	2.853421	0.01239	0.058004	-3.762779	4.917711	5.789398	5.380951	4.840268	5.340231	5.028202	5.125	5.331332	5.438339	5.390509	5.294916	5.251348	5.143751	5.100515
Klhl22	-0.365275	6.17345	-2.853149	0.012397	0.058013	-3.821092	6.361238	5.709075	5.994779	6.25085	6.102218	5.955127	6.262117	6.267417	6.288123	6.150773	6.295744	6.074409	6.419003	6.319413
Rnf144b	-0.558788	4.478226	-2.852738	0.012407	0.05802	-3.7254	4.725042	4.54806	4.399853	4.854179	4.018988	3.980827	4.56363	4.552657	4.646003	4.398098	4.558676	4.703986	4.737483	4.079517
Zc2hc1c	-0.885049	1.327593	-2.852699	0.012408	0.05802	-3.223939	2.162475	1.248835	1.534559	2.045748	1.173177	0.982423	1.135593	1.239467	0.385075	1.21727	1.456531	1.252437	2.041677	1.355706
Elavl3	-0.472184	6.460119	-2.852255	0.012419	0.058049	-3.828125	6.544267	6.042063	6.223752	6.835153	6.200647	6.40539	6.389122	6.548861	6.459436	6.287653	6.584154	6.521424	6.937249	6.480561
Itpkc	0.575442	4.332132	2.851793	0.012431	0.05808	-3.671482	4.087502	4.528825	5.019812	3.879919	4.176973	4.469923	4.253222	4.244507	4.359752	4.564114	4.437876	4.261285	4.266558	4.178459
Nsmce1	0.413416	4.489271	2.8515	0.012438	0.058087	-3.74948	4.618111	5.019295	4.924971	4.783243	5.154387	5.36255	3.999651	4.106787	4.236421	4.324262	4.160911	4.16231	3.939462	4.129836
Nefm	-1.41328	8.329578	-2.851343	0.012442	0.058087	-3.801207	8.495083	5.928	7.573269	9.396715	8.476218	8.153782	8.535445	8.527035	8.898975	8.468865	8.483482	8.349331	8.742083	8.591888
Lrp1b	-1.136551	5.650256	-2.850269	0.012469	0.058168	-3.784993	4.830077	4.000847	4.551972	6.582167	4.445908	5.30192	6.518251	6.529296	6.353182	5.812672	5.778723	5.758697	6.372548	6.305549
Polq	1.199938	0.826091	2.85026	0.012469	0.058168	-2.985577	0.428583	2.117298	1.143363	0.041976	1.173177	1.136566	1.443753	1.020936	0.837186	0.938085	0.676952	0.460309	0.313987	0.753927
Tcf20	-0.65841	6.750332	-2.849741	0.012482	0.058187	-3.834785	6.887247	5.990266	6.780157	7.378157	6.410769	6.712937	6.633919	7.014998	6.638879	6.466092	6.9183	6.8876	7.121577	6.678713
Hjurp	1.475337	1.315483	2.849706	0.012483	0.058187	-3.011596	0.307952	1.854335	1.061799	0.33436	2.472441	1.648138	1.511505	2.345542	0.97585	1.942797	1.068256	1.97042	0.649393	0.970221
Fig4	-0.350641	6.23148	-2.848708	0.012508	0.058281	-3.832794	6.510498	6.140391	5.888397	6.44259	6.305362	6.17213	6.249771	6.160365	6.306857	6.163768	6.297573	6.159203	6.241162	6.223667
Ssfa2	0.413668	6.298899	2.848344	0.012517	0.058292	-3.832153	6.345043	6.538425	6.589603	6.001814	6.621891	6.59354	6.399218	6.177666	6.182295	6.166118	5.943097	6.389067	6.040653	6.216276
Exoc6	-0.620019	5.527272	-2.848227	0.01252	0.058292	-3.816044	5.758611	4.859106	5.373927	6.135026	5.283731	5.786023	5.439557	5.657738	5.682347	5.279845	5.697961	5.344586	5.763748	5.354378
Map3k11	0.423615	5.427546	2.846738	0.012557	0.058443	-3.795635	5.360227	5.691883	5.658028	4.994772	5.71801	5.32295	5.130397	5.573776	5.163961	5.544765	5.510468	5.731623	5.334202	5.287439
Lrrn3	-1.100682	6.705904	-2.844928	0.012603	0.058632	-3.84068	6.527481	4.595055	6.073187	7.406362	6.174378	6.608769	6.998789	7.083674	7.140065	7.083498	6.823133	6.995162	7.159284	7.23159
Tox2	-0.494638	4.320771	-2.844316	0.012618	0.058681	-3.76766	4.71914	4.616473	4.603321	5.349294	4.611694	4.358391	3.969854	3.804966	4.023757	3.850214	4.13251	3.931333	4.341792	4.258767
Zfp157	-0.469742	4.277035	-2.843807	0.012631	0.058718	-3.696468	4.123692	3.822768	3.978882	4.605497	3.780406	4.024932	4.718584	4.49437	4.380707	4.373903	4.49636	4.405307	4.484924	4.271049
Abhd1	-0.710732	4.84503	-2.843245	0.012645	0.058749	-3.740048	5.172161	4.496185	3.922236	4.473536	4.371314	3.673423	5.428646	5.004383	5.408039	5.316274	4.990317	5.053812	4.972421	5.607789
Ect2	2.339855	0.278359	2.843043	0.01265	0.058749	-2.906356	-0.217457	1.117652	0.353965	-0.81875	2.732935	2.549011	0.282279	-0.150641	0.17081	-0.537962	0.071497	0.007173	0.037347	-0.204094
Cldn12	-0.39348	5.560884	-2.842965	0.012652	0.058749	-3.816185	5.574761	5.051103	5.376741	5.705755	5.172806	5.38426	5.564437	5.613437	5.634294	5.749822	5.609468	5.907454	5.769496	5.77234
Stau2	-0.606275	7.419931	-2.842754	0.012658	0.058751	-3.833878	7.881006	7.158745	7.5702	8.416114	7.470326	7.968271	7.253118	7.103307	7.2184	6.982442	7.335401	7.005907	7.513263	7.01198
Ntrk3	-0.596192	6.482325	-2.842282	0.012669	0.058771	-3.842923	6.071612	5.904562	6.100675	6.835738	5.530781	5.900214	6.86597	6.856281	7.093225	6.821804	6.440953	6.678164	6.677776	6.99357
Scml4	-0.679394	2.923201	-2.842194	0.012672	0.058771	-3.481857	2.810225	2.780133	2.548818	3.538889	2.062471	2.705027	3.462819	2.634802	3.533498	2.866406	2.986432	2.761217	3.245776	3.203933
Dkc1	0.56433	6.863255	2.840422	0.012717	0.058957	-3.850425	6.478541	7.277786	6.670934	6.318471	6.923802	6.975017	6.869202	6.919348	7.084949	7.344377	6.801391	6.833003	6.540801	7.061725
Hrh2	-1.001692	1.52618	-2.840022	0.012727	0.058981	-3.202412	1.594228	1.655851	0.604933	2.227735	1.20216	0.481842	1.63815	1.856886	1.937064	1.322149	1.740817	1.667719	2.437952	1.582867
Scara3	0.722445	7.321136	2.839462	0.012741	0.059016	-3.852603	7.117799	8.141166	7.633615	6.561825	7.479233	6.987736	6.829933	7.773985	6.841413	7.595186	7.447929	7.98072	6.921201	7.194458
Tmem246	-0.560909	5.772501	-2.839343	0.012744	0.059016	-3.806167	5.488925	4.489567	5.055367	5.384868	5.359656	4.586885	6.279225	6.145562	6.476084	6.149586	6.159621	6.251378	6.405223	6.615906
Fbxo33	-0.966543	4.869003	-2.838485	0.012766	0.059026	-3.782996	4.985653	3.151065	4.541981	5.624215	4.703236	4.924064	4.997894	4.895992	5.032426	4.891176	5.269057	5.074375	5.167891	4.96597
Lhfpl4	-0.755812	4.359614	-2.838417	0.012768	0.059026	-3.729268	4.464322	3.184227	4.296673	4.87707	3.913413	4.234096	4.218166	4.771798	4.334197	4.259708	4.573843	4.647164	4.850267	4.489994
Il16	-1.289668	5.84579	-2.838398	0.012768	0.059026	-3.822004	5.597458	3.134193	5.47059	6.51297	5.018417	5.382721	6.538692	6.361258	6.194181	6.297333	6.362771	6.287052	6.51964	6.20031
Zdhhc17	-0.491753	5.885667	-2.838294	0.012771	0.059026	-3.840851	5.787097	5.238395	5.619353	6.266538	5.500476	5.782524	5.96867	5.926901	6.112605	5.727718	5.906424	6.01419	6.358315	6.217334
Gpam	0.659763	5.925009	2.837913	0.012781	0.059026	-3.846304	6.162481	6.817398	6.598669	5.569051	6.190739	6.483618	5.53625	5.779538	5.259827	5.466841	5.749633	6.106932	5.948647	5.307451
Synpo	-0.963598	6.802859	-2.837886	0.012781	0.059026	-3.854588	7.328212	5.58264	7.105526	7.738384	6.655678	6.928279	6.260887	6.966052	6.677497	6.563796	7.038518	6.803199	7.141662	6.464608
Spryd3	-0.451398	7.770527	-2.83784	0.012782	0.059026	-3.845703	7.932919	7.280185	7.673059	8.068524	7.525114	7.721387	7.717613	7.766528	7.977719	7.707548	8.041496	7.646864	8.024287	7.711408
Shisa6	-1.751542	4.920716	-2.83772	0.012786	0.059026	-3.751427	4.958358	1.506527	4.408127	5.92151	4.078813	4.636834	5.130397	5.942556	5.389549	4.741628	5.544742	5.325652	6.228697	5.161159
Wdr47	-0.663961	7.502828	-2.837333	0.012795	0.059049	-3.852094	7.413366	6.657595	7.121968	8.167939	7.137212	7.58575	7.668372	7.633967	7.739754	7.378725	7.716776	7.404501	7.896485	7.526142
Tns3	0.648047	7.562561	2.835613	0.012839	0.05922	-3.85846	7.481623	8.127378	7.795593	6.761406	7.707992	7.444908	7.554038	7.574421	7.320078	7.79632	7.387406	8.076136	7.283495	7.573601
Ifi35	1.017887	2.20135	2.835387	0.012845	0.05922	-3.329257	2.569081	3.125682	2.597868	1.188094	2.987204	2.685174	1.809437	2.207232	1.914249	2.628098	2.750518	2.100868	0.921344	1.708589
Pias1	-0.594872	5.023558	-2.835304	0.012847	0.05922	-3.825234	5.425104	4.749824	5.407332	5.822387	4.888487	5.067073	4.629911	5.11075	4.970188	4.475935	5.075905	4.97094	5.068809	4.716427
Vamp1	-0.476445	8.650839	-2.834699	0.012863	0.059269	-3.825161	8.801563	8.226967	8.40698	9.136237	8.862454	8.470444	8.801925	8.487828	8.801523	8.526018	8.664584	8.402251	8.899777	8.627149
Lrrc10b	-1.412469	5.335824	-2.834282	0.012874	0.059296	-3.74905	5.502732	1.79116	3.99728	4.783243	4.162299	4.830014	6.267025	6.212949	6.016799	6.31014	6.116768	6.485196	6.121535	6.174391
Napg	-0.680653	7.392483	-2.833428	0.012896	0.059363	-3.858001	7.601955	6.612625	7.203065	8.163291	7.375709	7.614901	7.461785	7.227992	7.361778	7.10754	7.558423	7.153862	7.719975	7.34146
Atp4a	-1.122472	0.750147	-2.83333	0.012898	0.059363	-3.100387	1.874641	-0.273517	0.25957	0.966949	0.027932	1.249022	0.798508	0.705741	1.218642	0.981432	1.102128	0.34469	1.077784	1.158276
Tex261	0.549165	4.805637	2.831613	0.012943	0.059545	-3.760763	4.643261	4.926801	4.909512	4.155549	5.237232	4.687608	4.43432	4.873301	4.680213	4.908123	4.978968	4.97094	5.0524	4.877707
C1qa	0.628583	7.334869	2.831048	0.012957	0.059556	-3.869517	7.176582	7.641634	7.453457	6.507846	7.474786	7.308819	6.480712	8.257839	6.626651	8.106994	7.640311	8.548437	6.613712	6.861055
Fry	-0.531675	7.625549	-2.831007	0.012958	0.059556	-3.86663	7.419293	7.091507	7.295912	7.839456	6.758036	7.243717	8.018355	7.989764	7.897111	7.529624	7.872699	7.790147	8.246897	7.773514
Tmem204	-0.81757	3.206047	-2.830773	0.012964	0.059556	-3.528985	3.689063	2.736085	2.356997	3.051752	2.987204	2.146157	3.830902	3.024282	4.060584	3.588098	3.467722	3.461789	3.349197	3.31881
Kcnq2	-0.779906	7.964061	-2.83068	0.012967	0.059556	-3.861405	7.705575	6.752547	7.740686	8.302122	7.017989	7.38502	8.292891	8.374674	8.378872	7.776462	8.383733	8.253406	8.920269	8.219518
Eif5a2	-0.623432	6.272234	-2.830555	0.01297	0.059556	-3.871658	6.617478	5.949859	6.321172	7.283235	6.385723	6.650959	6.329365	5.735283	6.062907	5.826109	6.196346	5.850309	6.559999	6.063439
Grik3	-1.030848	3.552239	-2.829935	0.012986	0.059582	-3.681503	3.827152	4.073446	3.614277	4.804892	2.386673	3.186669	3.436917	3.449372	3.91304	2.966878	3.441501	3.772618	3.772036	3.229331
Ppm1g	0.324806	6.716785	2.829853	0.012988	0.059582	-3.873224	6.665834	6.860991	7.142363	6.709423	6.981362	7.065142	6.358404	6.641934	6.529603	6.67028	6.67768	6.734519	6.565792	6.446674
Pno1	0.535231	4.55509	2.829765	0.01299	0.059582	-3.780811	4.474867	4.613433	5.178177	4.646043	5.212518	5.365672	4.123906	4.332691	4.316905	4.18751	4.50904	4.200226	4.453131	4.225496
Slc12a7	0.931324	3.867616	2.828794	0.013016	0.059614	-3.620652	4.218773	3.948728	4.385956	2.427034	4.209453	4.254448	3.654827	4.134313	3.225976	4.066928	4.327666	4.296718	3.40196	3.710165
Tgfb1i1	0.456252	5.25827	2.828775	0.013016	0.059614	-3.839051	5.681801	5.810791	5.637061	4.945809	5.840583	5.767261	5.050088	5.059983	4.989416	5.016284	4.994832	4.901034	4.949817	5.01304
Vps26b	-0.551592	7.193037	-2.82868	0.013019	0.059614	-3.871865	7.439623	6.616426	7.047119	7.732434	7.142362	7.331346	6.992135	7.222231	7.119643	6.987102	7.436357	7.157079	7.449793	7.039761
RGD1563986	-0.62383	4.518163	-2.828525	0.013023	0.059614	-3.740607	4.478365	4.12132	4.139901	4.608236	3.346477	4.08677	5.030014	4.789219	5.048229	4.741628	4.363019	4.814046	5.098798	4.661508
Kif21b	-0.493104	6.890551	-2.82819	0.013031	0.059614	-3.877033	7.057689	6.77517	7.019932	7.227324	6.25179	6.551668	6.983959	6.94211	7.093225	6.757022	6.710989	6.901732	7.215637	6.992952
Tcf4	-0.657935	6.974245	-2.828186	0.013031	0.059614	-3.874334	7.07223	6.664956	6.77803	7.897705	6.57834	7.281395	6.945436	7.079432	6.94212	6.656138	7.010923	6.83702	7.197096	6.711375
Antxr1	-0.796498	4.000848	-2.828163	0.013032	0.059614	-3.783776	5.37719	4.57644	3.525794	4.629961	4.165982	4.589557	3.454237	3.501034	3.655446	3.538169	4.095154	4.196062	3.4385	3.372991
Epha3	-1.241177	3.15411	-2.827625	0.013046	0.059655	-3.50526	2.620642	1.310208	2.778919	4.188865	1.803478	2.836835	3.504983	3.791198	3.563133	3.129854	3.543639	3.727169	4.033233	3.537917
Fam212b	-0.646739	7.332252	-2.827001	0.013062	0.059693	-3.873512	7.552201	6.461892	7.124481	7.854528	7.306914	7.333341	7.310709	7.489048	7.401794	7.137789	7.63887	7.234236	7.644566	7.171166
Trappc9	-0.430003	5.842216	-2.826926	0.013064	0.059693	-3.856986	5.908752	5.144631	5.611014	5.790187	5.649957	5.263945	6.147008	5.867454	6.080945	5.960802	6.180579	5.915074	6.195989	6.102907
Zfp639	0.452875	4.123886	2.82656	0.013074	0.059699	-3.726774	3.901994	4.71337	4.627145	4.210655	4.405982	4.310598	4.123906	4.026799	3.848317	3.978769	3.858035	3.727169	4.08486	4.007943
Fam217b	-0.74846	3.698388	-2.826491	0.013076	0.059699	-3.718493	4.335237	3.432128	4.044046	4.473536	3.259533	3.870884	3.410541	3.770297	3.120298	3.241039	3.892318	3.489274	4.221137	3.34228
Dnm1l	-0.589993	6.936943	-2.826019	0.013088	0.059699	-3.879074	7.105976	6.517434	6.848633	7.762558	6.844448	7.162586	7.054505	6.837145	7.085587	6.408699	6.88342	6.595218	7.220367	6.803737
Polr1e	0.603995	4.169252	2.825937	0.01309	0.059699	-3.690178	3.765497	4.482919	4.514142	3.756964	4.352047	4.101824	4.473071	4.327931	4.401362	4.361652	3.953901	3.951235	3.748946	4.266966
Dnm3	-0.740281	5.421673	-2.825924	0.01309	0.059699	-3.861281	5.600671	5.198375	5.528663	6.523165	5.228454	5.338921	4.915882	5.617343	5.117279	5.021485	5.27092	5.412558	5.875907	5.291464
Gria4	-0.413354	6.362432	-2.825382	0.013105	0.059741	-3.878072	6.334466	5.918178	6.288677	6.683686	5.983075	6.194246	6.422504	6.34368	6.462388	6.330188	6.337222	6.364232	6.668576	6.762288
Mcart1l	-0.612055	3.415766	-2.824848	0.013119	0.059782	-3.629362	3.701089	3.073537	2.597868	3.810268	3.639189	3.3278	3.410541	3.449372	3.324439	3.608978	3.499841	3.440827	3.529386	3.558103
Zfhx4	-0.762677	4.082054	-2.822896	0.01317	0.05995	-3.731777	3.997728	3.678943	3.780713	4.899604	3.152236	4.185462	4.316148	4.620407	4.115617	4.246444	4.341911	4.136468	3.892823	3.880148
Cacnb3	-0.363126	7.975905	-2.822822	0.013172	0.05995	-3.872113	8.279686	7.711898	7.668474	7.90078	7.724318	7.80827	8.148742	7.934078	8.07671	8.18844	7.992954	7.957846	8.075069	8.201718
Tom1l2	-0.701742	6.051687	-2.822804	0.013172	0.05995	-3.88266	6.680334	5.573282	6.226094	6.563237	5.850914	6.025894	5.724671	6.184265	5.877787	5.500753	6.131194	5.987936	6.619293	5.802266
Itpr2	0.514174	5.379134	2.822689	0.013175	0.05995	-3.838776	5.274296	5.833178	5.674127	4.937121	5.408657	5.551217	5.211572	5.467413	5.163961	5.251354	5.184631	5.843669	5.33614	5.208755
Oas1k	2.15262	-1.932646	2.822298	0.013186	0.059974	-2.867884	-1.642648	1.339943	1.966737	-0.411255	0.845972	-0.371994	-3.260591	-2.076466	-3.260591	-1.838237	-1.719763	-1.660823	-2.656794	-3.260591
Dnajc6	-0.683152	8.470899	-2.821991	0.013194	0.059974	-3.853237	8.697169	7.880717	8.384791	9.146166	8.078031	8.610082	8.446831	8.533769	8.476276	7.929895	8.605846	8.350971	9.008283	8.448312
Rpl34	0.5723	5.568507	2.82192	0.013196	0.059974	-3.878212	6.262239	6.887661	6.28344	5.563417	6.357821	6.400832	4.977075	4.88307	5.092089	5.309912	4.882375	5.023584	4.906128	5.165551
Prim2	0.607305	3.222534	2.821373	0.01321	0.060017	-3.628486	3.350332	3.702025	3.540923	3.329127	4.213017	4.320284	2.688847	2.795149	2.94004	2.923085	2.940364	2.9008	2.725031	2.921834

Scn2b	-1.379978	6.192556	-2.820096	0.013244	0.060147	-3.882175	6.116079	4.129856	5.400436	7.513607	5.666949	6.521197	6.060593	6.613855	6.384644	5.945834	6.528412	6.51308	6.916675	6.410123
Rcn2	-0.4265	7.173492	-2.819345	0.013264	0.060215	-3.894143	7.130639	6.504363	6.740844	7.22331	6.831754	6.925109	7.289155	7.119925	7.462026	7.409301	7.208498	7.392724	7.469791	7.732655
Chrm1	-1.113898	6.933104	-2.818858	0.013276	0.060251	-3.891755	7.011959	4.525594	6.252373	7.099818	6.45659	6.520497	7.03089	7.542931	7.53207	7.346969	7.365348	7.478796	7.499494	7.416166
Slc35g2	-0.739274	3.390352	-2.818341	0.01329	0.06029	-3.631758	3.460552	2.532247	3.063656	4.050723	3.210452	3.288866	3.410541	3.104407	3.811751	3.552611	2.930971	3.489274	3.576053	4.138799
Tmem88b	-0.690127	5.244547	-2.816697	0.013334	0.06044	-3.871152	5.723667	5.200402	5.160327	5.974478	5.318889	4.967859	4.633716	5.178422	5.127233	4.738473	4.942039	5.662382	5.616844	5.221467
Aurka	0.539749	3.102303	2.816545	0.013338	0.06044	-3.540447	2.875353	3.512664	3.295418	2.797152	3.628553	3.061358	2.582602	3.440579	2.785194	3.530893	3.324422	3.454835	2.827778	2.503027
Eef1a2	-0.505907	10.05258	-2.81653	0.013338	0.06044	-3.802813	10.43257	9.730078	10.07656	10.75293	10.33642	10.20392	9.724121	9.884415	10.00537	9.815595	10.05937	9.82647	10.10509	9.784645
Ppfia4	-0.443523	7.110212	-2.815175	0.013374	0.060581	-3.90241	6.879331	6.949244	6.485929	7.181624	6.497352	6.418225	7.608717	7.3398	7.473288	7.482315	7.206553	7.224559	7.389946	7.418008
Cachd1	-0.460214	5.663191	-2.814695	0.013387	0.060616	-3.884195	5.774348	5.230479	5.394896	6.123585	5.785394	5.548472	5.759921	5.581796	5.803689	5.360041	5.368084	5.687812	5.975005	5.922627
Wipf1	0.421593	6.051812	2.813905	0.013408	0.060689	-3.8993	6.025731	6.625509	6.61246	6.382265	6.604357	6.661793	5.503348	5.803739	5.469972	5.815669	5.685448	6.209646	5.648382	5.701512
RGD1565283	-0.932487	1.104349	-2.812911	0.013435	0.060787	-3.055093	0.692264	-0.273517	-0.067513	1.033666	0.266146	0.056669	2.343298	1.958936	2.204856	1.322149	1.557647	1.315214	1.922909	2.011549
Fads3	0.587984	5.158526	2.812555	0.013445	0.060807	-3.824733	5.045831	5.075612	5.508281	4.281	5.378822	4.990279	4.946803	5.202027	5.211521	5.335194	5.289414	5.259352	5.274818	5.465422
Cdk5r1	-0.907938	5.663716	-2.810791	0.013492	0.060996	-3.883322	5.836997	4.112733	5.43998	6.134076	5.190992	5.540207	5.54638	6.06918	5.759573	5.511883	6.176611	5.962422	6.30588	5.738835
Tspan7	-0.515605	10.41308	-2.810505	0.0135	0.060996	-3.812356	10.61286	9.748903	10.27994	10.69114	10.34538	10.17746	10.19288	10.42278	10.61188	10.59112	10.37162	10.53135	10.45986	10.74713
Dnajb2	-0.389878	7.216277	-2.81043	0.013502	0.060996	-3.909222	7.290405	7.119101	6.726579	7.532114	7.21792	7.019793	7.178876	7.266439	7.152912	7.406819	7.254877	7.365637	7.224558	7.282524
Tatdn2	0.343411	5.584627	2.809428	0.013529	0.061081	-3.884965	5.551702	5.733085	5.956739	5.435094	5.802055	5.847601	5.509574	5.516354	5.489397	5.500753	5.572191	5.399942	5.60564	5.29748
Pfdn1	0.414757	6.340362	2.809358	0.013531	0.061081	-3.911984	6.72519	7.178423	6.74466	6.393423	7.107839	6.861299	5.958085	5.860878	6.00335	6.003481	6.136312	5.987936	6.009418	5.814904
Mgst3	-0.5143	6.179972	-2.808817	0.013545	0.061118	-3.910156	6.591871	5.726064	5.910893	6.528958	6.166128	6.380915	6.602223	5.667191	6.568479	6.055113	5.753634	5.969758	6.151559	6.469057
Epha4	-1.372806	8.568457	-2.808683	0.013549	0.061118	-3.888918	8.281189	5.777223	7.745591	9.227795	7.68604	8.319655	9.060563	9.204045	9.231838	8.787993	9.083287	9.043776	9.350609	9.164497
Adrbk2	-0.530512	5.054729	-2.80789	0.01357	0.061191	-3.865408	5.286319	4.801498	5.22264	5.517534	4.478849	4.982166	5.450387	5.045558	5.081888	4.680472	4.827749	4.733017	5.485056	5.221467
Pgf	1.516711	1.414842	2.806972	0.013595	0.061281	-3.196872	1.278303	3.208609	2.939761	0.485009	1.556544	1.668504	0.028455	1.80302	0.736738	1.597576	1.762161	1.667719	0.698429	1.048408
Sgpp2	-1.068792	4.107651	-2.80659	0.013606	0.061284	-3.758137	3.88628	2.833367	3.772191	5.245807	3.306999	4.098075	3.945565	4.576933	4.481148	3.709073	4.400933	4.447955	4.710843	4.19144
Sh3bp5	-0.661986	6.447307	-2.806565	0.013606	0.061284	-3.916961	6.502789	5.527177	6.488533	7.157519	6.200647	6.453861	6.436733	6.551926	6.597411	6.358207	6.668525	6.345555	6.541641	6.450279
Wbscr17	-1.242451	6.410585	-2.805615	0.013632	0.061378	-3.900967	6.135041	3.576505	5.80417	6.677831	5.383574	5.845367	6.79807	7.237541	7.098927	6.789395	7.042885	7.057312	7.319205	7.008317
Snap91	-0.689666	8.410701	-2.805262	0.013642	0.061398	-3.883434	8.695291	7.763553	8.434582	9.284865	8.354227	8.648394	8.274118	8.283574	8.401606	7.913903	8.476437	8.131695	8.795177	8.297132
Hcfc2	0.517061	3.804824	2.805007	0.013649	0.061406	-3.677791	3.481621	4.382912	3.89502	3.437817	3.89578	3.747086	3.908348	3.638097	3.947156	3.890566	3.733898	3.767014	3.67737	3.978289
Gpr75	-0.561249	3.881638	-2.803948	0.013678	0.061513	-3.767157	4.33909	3.324693	3.855228	4.383678	3.886882	4.113012	3.504983	3.698353	3.930199	3.602051	3.872828	3.895831	4.148003	3.896095
Zfp398	-0.549072	3.964715	-2.803711	0.013684	0.061519	-3.756549	3.907194	3.678943	4.004574	4.632654	3.446756	3.789543	4.063116	3.997121	4.146293	3.796617	4.086719	3.985416	3.97969	4.093415
Sh2d1a	-0.827062	2.343055	-2.802701	0.013712	0.061617	-3.4947	2.706665	1.339943	2.401882	3.160244	2.240661	2.446602	2.450751	1.628188	2.568526	2.40749	1.883989	2.447635	2.522078	2.921834
Slit1	-0.580483	7.938566	-2.802541	0.013716	0.061617	-3.917868	7.512234	6.873752	7.601504	8.186843	7.20947	7.390011	8.516504	8.367443	8.5739	7.900204	8.126243	8.05646	8.437677	8.394599
Unc5d	-1.745088	4.37853	-2.802166	0.013726	0.06164	-3.742489	4.137033	1.184734	4.421813	5.498469	2.399242	4.149692	4.618433	5.321786	5.154258	4.4607	4.655954	4.926391	5.505866	4.988458
Usp29	-0.609801	5.930085	-2.801106	0.013755	0.06174	-3.918823	5.88127	5.56858	5.897242	6.705592	5.361263	5.912006	6.179914	5.711724	6.053802	5.737232	6.057577	5.731623	6.336214	5.913516
Kcnj6	-0.986634	8.313205	-2.800865	0.013762	0.06174	-3.90922	7.875379	6.557533	7.74423	9.07556	7.514683	8.133272	8.770294	8.847635	8.639703	8.571051	8.646405	8.587905	8.877826	8.549035
Galns	0.85018	2.884522	2.800614	0.013769	0.06174	-3.449393	2.230432	2.843782	3.028308	2.093442	2.892584	3.207696	2.380012	3.583227	2.653634	2.966878	3.173291	4.056042	2.84965	2.648193
Gpr63	-1.270751	3.513923	-2.800604	0.013769	0.06174	-3.708143	3.639931	2.134368	3.271431	4.888381	2.322129	4.261168	3.616879	3.734773	3.59934	3.204921	3.592118	3.668268	4.1699	3.246018
Fbxo27	0.623376	3.485769	2.799928	0.013788	0.0618	-3.614592	3.00712	3.730367	3.989949	3.067759	3.446756	3.450478	3.727855	3.368235	3.592171	3.352542	3.776475	3.668268	3.160849	3.604134
Rps7	0.370387	6.989797	2.799737	0.013793	0.061801	-3.924651	7.57933	7.814084	7.769605	7.340769	7.966057	7.772849	6.637714	6.465711	6.529603	6.498158	6.299399	6.329488	6.285965	6.581804
Zfp455	0.778525	2.207626	2.799064	0.013811	0.061845	-3.394036	1.669604	2.619695	2.984714	2.124384	2.847434	2.230763	2.247205	1.80302	2.147322	1.942797	1.978199	2.330954	1.837926	2.489076
Zfp609	-0.470681	6.843212	-2.799002	0.013813	0.061845	-3.933302	6.937671	6.569349	7.032029	7.445941	6.527006	6.754611	6.686163	6.98734	6.762181	6.719912	6.953516	6.824261	6.91797	6.700822
Lbr	0.658569	3.141258	2.798237	0.013834	0.061915	-3.537649	2.694686	3.582736	3.370733	2.534609	3.273255	2.845805	3.127125	3.232189	3.448717	3.080873	3.031075	3.52291	3.079891	3.334499
Psd2	-0.558477	6.872226	-2.798047	0.013839	0.061915	-3.933544	7.028756	6.548011	5.933043	6.540475	6.2673	6.160494	7.410578	7.021646	7.127722	7.509869	6.98506	7.390448	7.069525	7.232637
Nt5dc3	-1.094656	6.310404	-2.797876	0.013844	0.061915	-3.928423	6.435999	4.785379	6.022889	7.056355	5.58405	6.199504	6.452979	6.823682	6.384644	5.927943	6.956991	6.324728	7.283495	6.129006
Fbxo4	0.657163	2.183066	2.796999	0.013868	0.062	-3.415573	2.127257	2.904741	2.568639	1.892408	2.226624	2.924117	1.986536	2.007376	1.937064	1.898256	2.100543	2.100868	2.060555	2.17732
Rpl17	0.533453	3.558473	2.796725	0.013876	0.062011	-3.714833	3.621067	4.298352	3.855228	3.538889	4.031153	4.271191	3.545949	2.901501	3.408468	3.463704	3.345785	2.921125	3.195422	3.558103
MGC109340	-0.843756	5.122613	-2.79653	0.013881	0.062012	-3.918006	5.78286	4.512597	5.244369	6.460888	5.608612	5.739856	4.861747	4.073048	4.872969	4.81235	4.400933	4.698109	5.229661	5.467202
Gpx7	0.628467	2.978308	2.796142	0.013892	0.062037	-3.556618	3.082606	3.24835	3.177748	2.324175	3.346477	3.456346	2.398025	2.988532	2.973531	3.110461	3.173291	2.838051	2.933966	2.847239
Kcnh8	-1.570495	0.781085	-2.794915	0.013926	0.062166	-3.147584	1.278303	0.04804	0.604933	1.658753	-0.424712	-0.291112	1.258992	1.356279	0.884903	0.591587	1.347792	0.34469	2.771602	0.554014
Plat	0.836455	6.487685	2.79106	0.014034	0.062613	-3.938985	6.22316	7.263796	7.166951	5.647035	6.291944	6.352876	6.437822	6.813502	6.35636	6.378616	5.993598	6.876585	6.395962	6.647651
Tuft1	0.634167	2.410693	2.790703	0.014044	0.062613	-3.491331	2.402324	2.713546	2.718102	2.241914	3.106978	3.235263	1.754571	2.24812	1.819202	2.469694	2.627988	2.330954	2.333172	2.050068
Irg1	2.229178	-0.755737	2.79047	0.01405	0.062613	-2.870744	-1.22354	0.480615	1.493674	-1.788967	0.562819	-0.862697	-1.21312	-0.380636	0.007959	-0.961779	-1.310338	-1.073407	-0.512812	-0.886923
Lrrn2	-0.787823	8.472846	-2.790448	0.014051	0.062613	-3.932876	8.23515	6.54562	7.73768	8.331838	7.951728	7.756592	9.099955	9.031613	9.132022	8.912016	8.804563	8.969943	8.894855	9.221508
Cdh11	-0.555995	6.844428	-2.790389	0.014052	0.062613	-3.949859	6.757939	6.018122	6.384045	7.311239	6.672603	6.838451	7.011277	6.933519	7.084949	6.792441	6.774729	6.854298	7.14443	7.258053
Atp2a3	-1.091307	3.207883	-2.789468	0.014078	0.062705	-3.634525	3.334978	3.27168	1.874203	3.879919	2.702832	2.422822	3.529702	2.38274	3.280514	3.753509	3.61576	3.980582	3.318158	3.751656
Dpf1	-0.357881	6.365196	-2.789115	0.014088	0.062714	-3.945651	6.278382	5.932887	6.012977	6.407642	5.974701	6.021942	6.549813	6.528259	6.757382	6.693274	6.380132	6.358654	6.502484	6.733732
Cyp2j3	-0.64679	5.240844	-2.789031	0.014091	0.062714	-3.900775	5.541706	4.953495	4.188746	5.324526	5.154387	4.872329	5.44173	5.199424	5.225489	5.604901	5.238925	5.735932	5.314678	5.619042
Grin1	-0.412656	10.11723	-2.788461	0.014107	0.062745	-3.874145	9.872324	9.612992	9.714116	10.30946	9.678097	9.701834	10.49798	10.33217	10.57019	10.27261	10.08745	10.17869	10.23657	10.57817
Klhl23	-0.650498	5.911341	-2.788407	0.014108	0.062745	-3.947895	6.327695	5.29072	5.867547	6.730316	5.850914	6.502175	5.86078	5.706232	5.74833	5.597955	5.790457	5.774143	6.000891	5.737361
Zdhhc18	0.434605	5.482648	2.786795	0.014154	0.062924	-3.911725	5.195727	5.675942	5.907005	5.164405	5.497557	5.371895	5.365968	5.539186	5.471927	5.506329	5.381938	5.641098	5.385636	5.687836
Cytl1	-1.901471	-0.358624	-2.785005	0.014204	0.063125	-2.98777	1.541696	-1.947514	-1.909221	-0.411255	-0.18071	-0.548781	0.357848	0.04768	0.317129	-0.116374	0.998029	-0.070959	-0.512812	-0.116166
Cacng8	-0.696232	7.594908	-2.783723	0.01424	0.063263	-3.962204	6.837042	5.686107	6.395882	7.378558	6.6524	6.908617	8.391713	8.320288	8.424995	8.315613	8.059657	8.30531	8.291027	8.371734
Syt1	-1.272343	9.066679	-2.783414	0.014249	0.063279	-3.911925	9.000935	6.971973	8.727304	10.1264	8.427387	9.040691	9.045286	9.472062	9.449037	8.795602	9.33069	9.275285	9.813645	9.460661
Slc36a4	-0.857531	4.492083	-2.783221	0.014255	0.06328	-3.868054	4.794038	3.466233	4.59368	5.240521	3.76592	4.781755	4.489964	4.424739	4.393136	4.116377	4.655954	4.584831	5.080417	4.575
Ndrg1	-0.587399	7.158948	-2.782415	0.014278	0.063358	-3.963187	7.341238	7.103475	6.49178	7.535356	7.141894	6.666231	7.10541	7.081554	7.297114	7.301195	6.867506	7.562044	7.240166	7.501597
Nrxn3	-0.84979	6.700649	-2.781815	0.014295	0.063411	-3.963609	6.361238	5.330145	6.315318	7.337885	5.810314	6.53928	7.074943	7.150577	7.0435	6.558439	6.908762	6.883075	7.435853	7.076378
Htra3	0.759931	4.450802	2.779892	0.01435	0.063631	-3.838553	4.779933	4.701966	4.731936	3.561676	5.078266	5.116089	4.253222	3.818604	4.241012	4.453021	4.467414	4.440934	4.200009	4.541596
Synj1	-0.746921	8.71272	-2.778901	0.014378	0.063733	-3.930183	8.770335	7.967807	8.652517	9.490435	8.36127	8.549092	8.683231	8.921578	8.785898	8.233397	9.000257	8.612914	9.308662	8.64456
Acer2	1.380303	4.84805	2.778181	0.014399	0.063801	-3.913646	5.112631	7.407631	6.826681	4.549599	5.295546	5.288824	4.301868	4.203515	4.441807	4.483493	3.771221	4.331301	3.665088	4.258767
Ccdc126	-0.718614	3.33324	-2.777941	0.014405	0.063808	-3.693477	3.42474	2.200694	2.947351	3.902409	3.26641	3.371934	3.280644	3.377479	3.31576	3.48645	3.543639	3.405197	3.582598	3.722142
Rsad1	0.799342	1.50577	2.776633	0.014443	0.063951	-3.224254	0.995438	1.972946	1.792283	0.74601	1.643636	1.165512	1.726334	1.658838	1.60797	1.704918	1.783193	1.593679	1.521045	1.73247
Taf1c	0.766063	5.092345	2.776047	0.01446	0.064002	-3.854799	4.512886	5.512563	4.923048	3.967847	4.964461	4.600198	5.638278	5.275563	5.446313	5.611814	4.985788	5.316091	4.939655	5.647583
Irak2	0.780583	4.165219	2.775363	0.01448	0.06404	-3.753354	3.676936	4.861671	4.559421	3.355447	3.956574	3.784887	4.571588	4.166665	4.222559	4.509637	4.128407	4.101275	4.116777	4.392186
Gpr176	-0.744576	4.979072	-2.775249	0.014483	0.06404	-3.861367	4.523084	3.24835	4.19832	4.984269	4.158607	4.410708	5.684875	5.373521	5.754765	5.690653	5.441032	5.65936	5.145962	5.491897
Pfkfb3	0.820751	5.352019	2.775057	0.014488	0.06404	-3.941949	4.945779	5.860363	6.697619	5.546382	5.855482	5.863146	4.591292	5.312177	4.972951	4.630291	5.278346	5.24128	5.509306	4.664615
Lrrc55	-0.802333	2.777886	-2.774864	0.014494	0.06404	-3.505268	2.474162	1.248835	2.162035	2.951788	2.139408	2.116816	2.534559	3.825375	2.894141	3.360772	3.53126	3.732929	3.278395	2.900911
Ina	-0.681446	8.289571	-2.774823	0.014495	0.06404	-3.945206	8.544309	7.707984	8.076493	9.233571	8.629772	8.40955	8.00299	8.211749	8.295533	7.942631	8.424255	8.095546	8.40689	8.07786
Plk4	0.829078	1.827289	2.774396	0.014508	0.064063	-3.470562	2.072763	2.884706	2.238266	1.9796	3.252623	3.038068	1.443753	1.356279	0.97585	0.847278	1.719153	1.375373	1.436856	1.445134
Bex1	-0.630308	5.509942	-2.774272	0.014511	0.064063	-3.969993	6.2011	5.062295	5.670692	6.370214	5.760036	6.121323	5.349864	5.361925	5.56458	5.183373	5.221695	4.86739	5.381888	5.058621
Tiam1	-0.988027	7.4321	-2.774066	0.014517	0.064066	-3.972824	7.207999	6.251146	7.423158	8.632009	6.57834	7.463967	7.655795	7.725944	7.743804	7.499656	7.543862	7.486914	7.341084	7.505501
Plk1	1.233501	2.327656	2.773677	0.014528	0.064092	-3.433819	1.80948	3.176008	1.921212	1.720809	3.568609	3.340548	2.266943	1.856886	2.166756	2.40749	2.303426	2.419337	1.700256	2.261799
Xrcc4	-0.543407	3.201774	-2.772005	0.014577	0.064281	-3.690081	3.417469	2.914655	2.691232	3.600707	3.106978	3.200721	3.36548	3.180315	3.424703	2.934159	3.42821	3.046681	3.589113	3.097617
Ghsr	-2.123881	1.096237	-2.771834	0.014582	0.064281	-3.193007	1.514695	-2.680539	0.642822	2.077719	0.266146	1.275818	1.258992	0.76329	2.241976	1.875459	1.674823	1.848377	1.963595	2.211707
Ppp4r4	-0.947825	4.55244	-2.77156	0.01459	0.064292	-3.874074	4.403064	2.904741	4.136585	5.402331	4.415294	4.348958	4.940672	4.76477	4.890608	4.386051	4.850346	4.509652	5.114691	4.74018
Atp1b1	-0.937356	11.04189	-2.768757	0.014671	0.064616	-3.878676	10.88082	9.172617	10.27236	11.44724	10.77305	10.69513	11.30934	11.29551	11.58624	11.42042	11.19301	11.35556	11.46742	11.71865
Nat10	0.429231	5.174809	2.768536	0.014678	0.064616	-3.930745	4.89695	5.452583	5.471906	5.003121	5.446997	5.14726	5.43302	4.980316	5.175999	5.208673	5.099154	5.060699	4.882452	5.231976
Gaa	-0.464636	8.034569	-2.768488	0.014679	0.064616	-3.986117	7.878029	7.252114	7.204253	7.476616	7.33499	7.063702	8.561151	8.458729	8.510428	8.631188	8.417124	8.82714	8.175631	8.699547
Atp6v0e2	-0.638393	7.294357	-2.768091	0.014691	0.064643	-3.992117	7.409707	5.985571	6.833353	7.127764	6.968768	6.734227	7.263594	7.620405	7.683523	7.544152	7.777976	7.646864	7.877647	7.658244
Stk24	-0.876325	3.817558	-2.767361	0.014712	0.064708	-3.834824	4.026686	2.747223	3.99728	4.941472	3.446756	4.220367	3.790562	3.749088	3.641623	3.530893	3.827988	3.778199	4.089463	3.774841
Csad	-0.475733	5.329545	-2.767065	0.014721	0.064708	-3.962214	5.948388	5.502738	5.187821	5.384868	5.070428	5.002363	5.214116	5.149037	5.293368	5.384467	5.43606	5.486023	5.227574	5.365875
Peli2	-0.507383	3.228824	-2.767041	0.014722	0.064708	-3.721649	3.536344	3.091129	3.247038	3.696477	2.72297	3.390443	3.148563	3.272386	2.973531	3.223093	3.165302	3.489274	3.229186	3.186749
Rph3a	-0.501578	8.109724	-2.766649	0.014733	0.064734	-3.967433	8.671422	8.354216	8.183198	8.573475	7.804171	8.142402	7.805429	7.990141	7.856923	7.991817	7.763827	8.114148	8.373545	7.917187
Ttc19	-0.613784	6.259689	-2.766391	0.014741	0.064744	-3.989715	6.182992	5.284999	6.07492	6.777921	6.04037	6.063866	6.346858	6.298164	6.490615	6.083986	6.409173	6.306496	6.7717	6.524823
Thsd7b	-0.512187	3.904559	-2.765744	0.01476	0.064791	-3.72811	3.621067	3.125682	3.247038	3.659984	2.978854	3.172479	4.718584	4.384043	4.549366	4.246444	4.345451	3.848862	4.519446	4.361886
Txndc17	0.486319	4.354834	2.765661	0.014762	0.064791	-3.882017	4.406741	4.681789	4.835775	4.314927	5.275232	4.581525	4.123906	3.923301	4.044916	4.210463	4.21995	4.101275	4.187182	4.138799
Hdac8	-0.54706	2.763347	-2.765434	0.014769	0.064796	-3.586928	2.853968	2.582864	2.053692	2.977436	2.361201	2.559953	2.955303	3.252427	2.962454	2.807409	3.057213	2.960934	2.805569	2.733504
Syn1	-0.464905	10.52302	-2.76487	0.014785	0.064846	-3.877775	11.20464	10.54201	10.79069	11.26922	10.84699	10.91119	9.94781	10.3312	10.31041	10.13108	10.35399	10.15669	10.47384	10.05357
Zswim2	-1.058034	1.106051	-2.764615	0.014793	0.064855	-3.29476	1.212615	0.24948	0.715742	2.198954	0.371962	1.473956	1.809437	1.393205	1.549949	0.534523	1.230182	1.082389	1.347449	1.085968
Cdc20	2.100223	1.04278	2.764284	0.014803	0.064874	-3.131474	1.033981	1.854335	0.818644	-0.941548	3.196117	2.131561	1.17791	0.972957	0.931093	0.348395	1.290185	1.219993	0.598632	0.887553
Map3k13	-0.725697	3.515691	-2.762318	0.014861	0.065073	-3.781937	3.417469	3.24835	3.505372	4.566597	3.020129	3.371934	3.593621	3.583227	3.540964	3.352542	3.288097	3.454835	3.999391	3.417866
LOC365985	-0.723733	8.173365	-2.762208	0.014864	0.065073	-3.981612	8.324832	6.99084	8.065201	8.643069	7.887104	8.103431	8.091435	8.331925	8.333108	8.192769	8.60363	8.166694	8.548183	8.150565
Oas1b	1.527868	1.532064	2.762194	0.014864	0.065073	-3.192442	0.955837	1.833582	2.31067	-0.093872	1.578815	1.708392	1.544223	2.121805	0.736738	2.558244	2.288794	3.489274	0.111637	0.970221
Prc1	1.942042	2.794628	2.76147	0.014886	0.065143	-3.441356	2.054131	3.151065	2.200654	1.128291	4.454966	4.164107	2.828218	3.081965	2.313467	2.274406	2.213317	3.16218	3.40196	2.962789
Bdnf	-1.314638	5.180071	-2.761242	0.014893	0.065149	-3.932804	5.089944	2.532247	4.380359	5.925907	4.466957	5.337331	5.211572	5.593743	5.858496	5.650933	5.654393	5.524723	5.694433	5.656972
Spns2	-0.418172	6.748056	-2.760238	0.014922	0.065256	-4.006016	6.587019	6.193272	6.416536	6.784595	6.474519	5.987909	7.136588	6.787732	7.225367	6.984441	6.738171	6.960601	6.965068	7.246179
Ctbs	0.63889	4.436016	2.759396	0.014947	0.065341	-3.843191	4.235407	4.841025	4.501309	3.617117	4.5954	4.281144	4.618433	4.429191	4.639062	4.703306	4.208334	4.7949	4.13915	4.575
Ifrd2	0.893619	4.190509	2.758906	0.014962	0.065382	-3.784366	3.881004	4.841025	4.326082	3.107017	4.703236	3.622139	4.579502	4.234368	4.389005	4.410044	4.254248	4.220868	3.866246	4.323092
Tceb3	0.296257	6.07288	2.757508	0.015004	0.065496	-4.004868	6.036449	6.402365	6.436224	6.096533	6.340818	6.27117	5.900809	6.047627	5.95247	5.881504	5.867564	5.969758	5.92695	5.913516
Nusap1	2.150474	0.421395	2.757376	0.015008	0.065496	-3.113385	0.643243	1.310208	0.785152	-0.941548	3.188895	2.116816	-0.279766	0.301855	0.385075	0.055728	-0.318799	0.760186	-0.305233	-0.397871
Cldn11	-0.833056	7.853414	-2.757106	0.015016	0.065496	-4.00243	7.91915	7.262826	6.68859	8.191872	7.786638	7.146795	7.516915	8.350234	7.676761	8.123111	7.897927	8.692293	8.336753	8.365305
Tspyl4	-1.087394	6.792576	-2.757009	0.015019	0.065496	-4.010481	7.1206	5.35766	6.571913	7.85222	6.566548	7.090363	6.711592	7.026061	6.745314	6.288731	7.138306	6.673679	7.446208	6.522257
Rprm	-0.554155	2.062696	-2.757006	0.015019	0.065496	-3.622996	3.109925	2.758277	2.238266	2.934433	2.240661	2.685174	1.862293	1.279465	1.60797	1.625169	1.740817	1.738143	1.521045	1.931282
Klhl6	0.838185	2.627346	2.7569	0.015022	0.065496	-3.49371	2.035255	2.248512	2.558762	1.92792	3.068148	3.314938	2.033384	3.484018	1.843557	3.186517	3.310002	3.619352	2.133689	2.294252
Ppargc1b	-0.880126	2.825544	-2.756751	0.015026	0.065496	-3.661863	2.832262	2.506257	3.021133	3.857074	1.930143	2.493017	2.644268	3.115498	2.973531	2.707689	3.022257	2.668085	3.394539	2.622863
Clip4	-0.616569	4.461354	-2.756073	0.015047	0.06556	-3.892543	4.331374	3.655486	4.156367	4.956595	4.086605	4.223812	4.618433	4.850248	4.65291	4.259708	4.778847	4.49955	4.979878	4.482972
Cenpi	1.501814	0.869077	2.75563	0.01506	0.065594	-3.212121	0.592497	1.310208	1.089503	0.33436	2.661692	2.581593	0.118118	0.645799	0.512061	0.799637	0.071497	0.889587	0.836265	0.65743
Ust	-0.823175	2.758333	-2.755261	0.015071	0.065619	-3.691378	2.853968	2.354284	2.795835	4.058754	2.77212	2.714852	2.266943	2.58779	2.611707	2.484835	2.415434	3.065063	2.794335	3.079111
Vwa7	1.718403	1.118772	2.754805	0.015085	0.065655	-3.127969	0.307952	3.046741	1.220561	-0.411255	1.173177	0.982423	1.297885	1.155984	1.180909	1.481601	0.998029	1.568131	1.11438	1.355706
Pard6b	-0.790714	2.331823	-2.754362	0.015098	0.065689	-3.509739	2.787845	1.310208	2.053692	2.310787	2.226624	1.402804	2.598267	2.471744	2.680923	2.40749	3.082885	2.619163	2.652228	2.372328
Fbxo9	-0.450327	6.688175	-2.753584	0.015121	0.065762	-4.020686	6.779367	6.15719	6.474155	7.090544	6.515506	6.791638	7.053084	6.508411	6.897015	6.534992	6.746896	6.498783	6.842944	6.759385
Aldh7a1	0.558626	6.157227	2.753438	0.015126	0.065762	-4.01592	6.608727	7.035819	6.430819	5.791393	6.76473	6.794536	5.728235	5.78128	5.71901	6.019165	6.025942	6.003508	5.594349	5.926514
Ispd	-0.6935	2.744025	-2.752566	0.015152	0.065846	-3.690832	3.380557	2.100024	2.558762	3.387687	3.245679	2.854719	2.582602	2.76729	2.667343	2.308845	3.031075	2.502617	2.794335	2.474989
Ctla2a	1.032566	0.766621	2.75243	0.015156	0.065846	-3.226102	1.401336	1.874793	1.493674	-0.166914	1.463853	1.426914	1.17791	0.222004	0.628769	-0.027758	0.263333	0.460309	0.745852	0.753927
Lrch4	0.51306	5.965304	2.751321	0.01519	0.065968	-4.000651	5.564924	6.354913	5.989274	5.526249	5.950352	5.9395	6.19158	6.184265	6.083504	6.221401	5.833942	6.077808	5.53653	6.085628
Pih1d1	0.616233	3.298042	2.751123	0.015196	0.06597	-3.600919	2.542593	3.42521	2.709201	2.43939	3.144791	3.165332	3.742028	3.734773	3.324439	3.62956	3.858035	3.60686	3.529386	3.489688
Adamts18	-2.497404	0.15221	-2.750651	0.01521	0.065983	-3.053675	0.176305	-0.465897	0.20995	1.478901	-0.613814	-3.260591	1.372662	1.020936	1.426399	0.847278	0.138317	1.045835	0.836265	1.227132
Scrn1	-0.516243	6.765256	-2.750584	0.015212	0.065983	-4.025288	7.12004	6.720409	6.928459	7.586251	6.541961	7.154938	6.687079	6.747302	6.65359	6.305884	6.606451	6.423312	6.807762	6.444869
Ptprs	-0.404354	9.813649	-2.750484	0.015215	0.065983	-3.973164	9.281447	9.064619	9.012519	9.446442	8.970633	8.786985	10.68903	10.2129	10.56488	10.3264	10.02777	10.19338	10.22099	10.59506

Calb1	-1.082668	5.839929	-2.749681	0.015239	0.06605	-4.018216	6.151567	4.236461	5.91767	7.065352	6.047374	5.878525	5.540311	5.625125	6.276989	5.746684	5.750968	5.745936	5.952442	5.853529
Gabrg2	-0.94684	6.227078	-2.749612	0.015241	0.06605	-4.015039	5.965937	4.382912	5.652234	6.822236	5.60319	6.132362	6.437822	6.708456	6.776483	6.339588	6.471239	6.474921	6.7303	6.705354
Actb	0.485126	10.65823	2.748893	0.015263	0.06612	-3.929109	10.36833	10.4693	10.78053	10.35201	11.01042	11.12626	10.61269	10.88469	10.69768	10.55285	10.7678	10.74678	10.51917	10.32766
Tceal7	-0.780085	0.294855	-2.748661	0.01527	0.066127	-3.309414	1.371547	0.426209	1.220561	1.679737	0.691028	0.734546	-0.399109	-0.511025	-0.080923	0.348395	-0.149817	0.007173	0.249639	-0.116166
Aplp1	-0.456305	11.0487	-2.748116	0.015287	0.066175	-3.915011	11.24004	10.52264	10.61794	10.95458	10.86147	10.56818	10.93077	11.32365	11.25024	11.34901	11.07973	11.40282	11.14758	11.43389
Mybl1	0.650336	2.419987	2.747138	0.015317	0.06628	-3.623409	2.32672	3.000943	3.145118	2.787519	3.129785	3.55809	2.078759	1.983359	2.147322	2.027955	1.533025	1.989793	2.614401	1.868007
Usp11	-0.504257	7.74824	-2.746761	0.015328	0.066296	-4.021102	7.775486	7.053314	7.686727	8.270268	7.627484	7.707306	7.811742	7.793108	7.977719	7.713184	7.719506	7.544137	7.886601	7.91621
Cdkn1b	-0.449109	5.095949	-2.746652	0.015331	0.066296	-4.011614	5.748506	5.071186	5.403198	5.968096	5.578534	5.582409	4.736391	4.747049	4.549366	4.706539	4.573843	4.948838	4.964926	4.811983
Oxct1	-0.425332	7.785743	-2.745548	0.015365	0.066418	-4.017878	8.254661	7.753231	7.51253	8.21721	7.957052	8.018965	7.77732	7.434181	7.556218	7.49919	7.669198	7.684506	7.924127	7.749211
Asb13	-0.55284	3.909234	-2.744887	0.015385	0.066467	-3.864138	4.137033	3.774588	3.967729	4.33821	3.306999	3.703341	3.876582	4.106787	4.023757	3.602051	4.246696	3.800313	4.125768	3.825682
Fam131b	-0.390881	7.609174	-2.744814	0.015388	0.066467	-4.027909	7.776907	7.509679	7.567432	7.913292	7.172879	7.566888	7.553034	7.525366	7.625889	7.540986	7.651078	7.624961	7.909559	7.598585
Tll1	-1.474078	2.490215	-2.743719	0.015421	0.066582	-3.52922	2.295333	-0.273517	1.825611	3.443962	1.173177	2.932562	2.967416	3.201289	3.324439	2.600561	2.771719	2.783591	2.964355	3.246018
Rps6ka1	0.65481	4.690422	2.743585	0.015425	0.066582	-3.886522	4.239536	5.066748	4.349185	3.795925	4.852378	4.407682	4.591292	5.057109	4.347031	5.126584	5.017197	5.613262	4.582846	4.683115
Dnajc25	0.594659	3.165518	2.743256	0.015435	0.066602	-3.769663	3.230989	3.49955	3.99728	3.361953	3.8553	4.178379	2.380012	3.321106	2.707706	2.681644	2.892776	3.346294	2.452318	2.59708
F13a1	2.283256	0.681555	2.742055	0.015472	0.066734	-3.25501	1.459129	0.934883	2.984714	-0.502853	2.919011	3.479584	-1.21312	0.301855	-0.386063	0.413143	0.846404	0.889587	-1.638202	0.554014
Col14a1	-1.309677	0.164952	-2.741897	0.015477	0.066734	-3.133526	0.830061	0.24948	-0.665442	0.577356	-0.516166	-0.984601	1.091998	0.377517	0.385075	0.413143	0.263333	1.593679	0.037347	0.190106
Eif3i	0.445978	5.121229	2.741226	0.015498	0.06678	-3.983677	5.167834	5.31899	5.515958	4.741441	5.600471	5.143628	4.92212	5.033913	5.050846	5.054841	5.156697	4.81947	5.06414	5.152334
Nr1d2	-0.423567	6.261119	-2.741188	0.015499	0.06678	-4.042879	6.641078	6.110512	6.22921	6.794248	6.179853	6.657979	6.33522	6.021331	6.018137	6.205494	6.197325	6.001123	6.115906	6.168932
Nat1	0.627763	1.920873	2.740248	0.015528	0.066869	-3.564593	2.41698	2.924502	2.626517	2.013053	3.00376	2.762997	1.258992	1.532126	1.180909	2.108365	1.652137	2.136071	0.649393	1.085968
Slc25a14	-0.42171	5.729947	-2.740084	0.015533	0.066869	-4.025036	5.643365	5.393549	5.298783	6.085764	5.386733	5.70331	5.96867	5.565712	5.98841	5.907019	5.74428	5.727301	5.825753	6.010707
Pcdha5	-1.229668	1.145605	-2.739981	0.015536	0.066869	-3.316825	1.212615	-0.186163	0.914692	2.124384	-0.424712	1.688586	1.335758	1.532126	1.520039	1.253084	1.375754	1.375373	1.651284	1.473753
Dstn	0.468943	4.282971	2.738958	0.015568	0.066981	-3.865954	4.069061	4.532048	4.079015	3.633342	4.448934	4.199526	4.287446	4.451246	4.496589	4.453021	4.483567	4.292824	4.266558	4.350357
Otof	-0.854077	5.532196	-2.7387	0.015576	0.066983	-4.013104	5.45204	4.838423	5.025202	6.093604	5.660437	4.17126	6.109239	5.507961	6.228108	5.157622	5.681252	4.913769	6.206621	6.443061
Rab4a	-0.330366	6.148871	-2.738437	0.015584	0.066983	-4.043705	6.342166	6.081102	5.865545	6.396595	6.221145	5.991955	6.149668	5.983995	6.244205	6.086469	6.141411	6.153846	6.285965	6.162354
Dlg1	-0.344411	6.162122	-2.738405	0.015585	0.066983	-4.048121	6.539252	6.494482	6.300579	6.807417	6.176206	6.348149	5.97168	5.795142	5.9925	5.84823	5.827629	5.912539	6.195989	6.082147
Klf9	-0.80907	6.474877	-2.738213	0.015591	0.066984	-4.04864	7.037082	6.095337	6.893033	7.624294	6.19435	6.898953	6.080248	6.35191	6.133744	5.852614	6.535402	6.248365	6.842263	5.879161
Reep1	-0.549383	7.003287	-2.737574	0.015611	0.067045	-4.051378	6.839085	5.93654	6.532718	7.214799	6.717207	6.7221	7.08262	7.158642	7.220145	7.163306	7.252994	7.321879	7.473317	7.42352
Calm3	-0.682791	9.911726	-2.737051	0.015627	0.067071	-3.955002	10.54066	9.376578	9.887924	10.78634	10.1699	10.49279	9.625204	9.824104	9.715401	9.484982	9.882075	9.673044	9.919144	9.387694
Nipsnap3b	0.50751	3.603273	2.736936	0.01563	0.067071	-3.755137	3.109925	3.557647	3.637922	3.233029	3.973484	3.541623	3.537848	3.484018	3.83015	3.669864	3.802462	3.529544	3.964736	3.704139
Tmem229a	-0.579759	6.573627	-2.736846	0.015633	0.067071	-4.052669	7.043	6.514992	6.09556	7.009989	6.438436	6.741454	6.730374	6.038915	6.235029	6.543151	6.370609	6.740964	6.82925	6.715874
Ipo4	0.411041	6.964246	2.736654	0.015639	0.067072	-4.051492	6.60313	7.096415	6.955527	6.413917	6.966131	6.657979	7.376916	6.979773	7.287715	7.110594	6.853902	6.925191	6.972563	7.312541
Necap1	-0.718656	7.554579	-2.736335	0.015649	0.067091	-4.039839	7.915929	6.70049	7.624471	8.361563	7.524039	7.834135	7.475104	7.459973	7.663142	7.185485	7.678349	7.268845	7.788102	7.293101
Prickle2	-0.545572	7.990548	-2.735873	0.015664	0.067114	-4.039661	8.032945	7.104558	7.670195	8.239905	7.558768	8.033268	8.207694	8.214653	8.05406	7.948445	8.260491	8.110004	8.357415	8.081642
Klhl41	0.981086	0.340994	2.735799	0.015666	0.067114	-3.141312	0.031429	0.679782	1.143363	-0.093872	0.470544	1.221719	0.429655	0.301855	0.17081	-0.027758	0.138317	0.618025	0.546019	0.383504
Pcdhga2	-0.79078	4.426191	-2.735327	0.015681	0.067117	-3.884309	4.235407	2.679063	3.793402	4.488478	4.078813	3.693437	4.812232	5.186334	5.102217	4.564114	4.763054	4.594355	5.176569	4.883051
Map3k14	0.47023	4.066396	2.735268	0.015682	0.067117	-3.863784	3.854329	4.03306	4.59368	3.727037	4.209453	4.164107	3.876582	4.166665	3.895674	4.056832	4.128407	4.440934	3.944552	3.932632
Cacna1a	-0.484687	8.877645	-2.73519	0.015685	0.067117	-4.023788	8.457693	8.286874	8.310777	9.042149	7.976055	8.479657	9.477516	9.286096	9.320998	9.171172	8.956227	9.153337	9.183736	9.188301
Gpr18	1.985417	-0.915647	2.735066	0.015689	0.067117	-2.931534	-1.642648	0.04804	-0.575682	-2.040259	-0.831496	0.571087	-2.088409	1.49863	-2.604521	0.646479	0.998029	1.315214	-2.656794	-1.040339
Lrp10	0.596846	6.315375	2.734333	0.015712	0.06718	-4.047125	6.422444	6.575611	6.511115	5.424255	6.705869	6.270337	5.922749	6.390089	6.215333	6.432321	6.49454	6.707714	6.066565	6.296566
Prkag2	-0.428961	6.896729	-2.73423	0.015715	0.06718	-4.058166	6.82816	6.207456	6.657115	7.200804	6.78103	6.695676	7.095785	7.012033	7.103346	6.883087	7.088483	6.917625	7.055477	7.041554
Zfp575	-0.480901	4.595741	-2.734029	0.015721	0.067183	-4.010879	5.290305	4.563896	5.065865	5.576063	4.897375	5.278924	3.920861	4.406794	4.338488	4.353427	4.231474	4.140807	4.1699	4.174106
Slc6a7	-0.58772	6.467438	-2.733711	0.015731	0.067194	-4.052783	6.409676	6.37749	5.421025	6.55758	5.995546	5.803393	7.117011	6.233457	7.168662	6.654465	6.222567	6.408092	6.86995	7.324375
Kcnab2	-0.778131	8.542032	-2.733461	0.015739	0.067194	-4.019288	8.869408	7.506815	8.426798	9.292655	8.387135	8.895715	8.5619	8.515045	8.374178	8.303389	8.508168	8.599971	8.925588	8.42606
Trpc5	-1.298481	4.33522	-2.733395	0.015741	0.067194	-3.878068	4.040949	2.264105	3.952724	5.072196	2.742832	4.028875	4.531353	5.194202	4.913797	4.111508	5.010524	4.814046	5.576426	4.538213
Prr16	-0.580044	2.235994	-2.733237	0.015746	0.067194	-3.717315	3.100876	2.532247	2.795835	3.462244	2.883666	2.634313	1.17791	1.49863	2.04602	1.652245	1.978199	2.2368	1.815874	1.846283
RGD1303130	0.674423	5.524563	2.732895	0.015757	0.067215	-4.004963	5.497569	5.763574	5.611014	4.461469	5.940711	5.258917	5.185883	5.767283	5.317478	5.800621	5.914787	6.101377	5.172237	5.586654
Ltbp4	0.568511	6.829106	2.73238	0.015773	0.06726	-4.057345	6.405088	7.223985	6.753887	6.16044	6.770184	6.653516	7.343612	6.816054	7.296563	6.972406	6.529968	6.748803	6.654666	7.292599
Pim2	-0.504133	5.359075	-2.731392	0.015804	0.067369	-4.034902	5.569851	4.919435	5.371108	5.946615	5.493166	5.233512	5.203913	5.245588	5.469972	5.213226	5.622656	5.044579	5.456834	5.275297
Swsap1	-0.429911	4.404182	-2.730543	0.01583	0.067459	-3.952334	4.614937	3.894655	4.419086	4.716273	4.445908	4.17126	4.376444	4.370218	4.433808	4.494755	4.612548	4.394445	4.505736	4.283226
Cyp4v3	0.604033	4.97751	2.729066	0.015877	0.067633	-4.001151	5.059908	5.551208	5.390726	4.629961	5.407102	5.426725	4.722163	4.792678	4.363967	5.024078	4.875046	5.29872	4.103184	5.09131
Dhrs4	0.571243	3.54101	2.728408	0.015898	0.067697	-3.823224	3.713016	4.10842	3.614277	3.175097	4.355276	3.947882	3.270902	3.340143	3.375441	3.440594	3.366837	3.376046	3.152075	3.475607
Zfp667	-0.684856	4.745663	-2.728204	0.015904	0.067701	-3.990789	4.748409	3.953545	4.287732	5.480644	4.539721	4.953408	4.958988	4.58892	4.942268	4.772804	5.02825	4.482556	5.06414	4.698352
Tm9sf4	-0.39093	5.849656	-2.727117	0.015938	0.067823	-4.045888	5.680283	5.057828	5.437287	5.85273	5.532208	5.472326	6.026261	6.141498	6.143586	6.088949	5.937244	6.105823	6.167973	6.279444
Dot1l	0.717898	6.990625	2.726392	0.015961	0.067897	-4.070006	6.299299	7.649447	7.670768	6.64019	6.666767	6.764697	7.217426	6.982048	7.155346	7.075394	6.650038	7.023638	6.858535	7.227919
Irgq	-0.473846	6.986352	-2.72582	0.01598	0.06795	-4.073775	7.233064	6.380176	6.819461	7.204429	6.797741	6.982662	6.964448	6.969877	6.903546	6.907872	7.129094	7.067035	7.438562	7.02352
Lrrtm4	-0.957326	4.694517	-2.725052	0.016004	0.068021	-3.97428	4.55325	2.934281	4.408127	5.476153	4.169655	4.685111	4.704179	5.082767	5.076761	5.049759	4.730939	5.062987	4.906128	4.950782
Gpaa1	0.574531	6.131156	2.72473	0.016014	0.068021	-4.038697	5.599065	6.049956	5.789465	4.971562	6.039366	5.540207	6.725924	6.254932	6.616087	6.626578	6.228329	6.379	6.254544	6.785304
LOC683077	-0.713718	7.2468	-2.724639	0.016017	0.068021	-4.0708	7.439623	6.272505	6.954115	7.947759	7.332125	7.358629	7.19442	7.122676	7.400769	6.979104	7.451628	7.038249	7.636725	7.337573
Cdca2	2.638832	-0.549111	2.724583	0.016019	0.068021	-3.016805	-1.642648	0.04804	0.049949	-0.941548	2.436306	1.927009	-0.831021	-0.81357	-0.773548	-1.136114	-0.991788	-0.541385	-0.123798	-1.632384
P4ha3	1.601589	1.190254	2.723867	0.016042	0.068073	-3.468089	2.213741	3.719097	3.063656	0.664146	1.78444	3.3278	-0.067151	0.137473	0.512061	0.699359	-0.731011	0.969826	0.375586	0.929479
Fgfbp3	-0.505133	4.185091	-2.72372	0.016046	0.068073	-3.953408	4.421353	3.735969	4.201497	4.7588	4.031153	4.370872	4.05746	4.084383	4.101553	4.429738	3.999546	3.946285	4.357143	4.182799
Flii	0.436233	7.304058	2.72356	0.016052	0.068073	-4.077106	6.980236	7.717927	7.286967	6.907538	7.318955	7.192778	7.515884	7.364416	7.427197	7.438757	7.135241	7.383141	7.119891	7.477953
Oas1a	1.554429	0.945058	2.723486	0.016054	0.068073	-3.174331	-0.217457	1.184734	1.143363	0.041976	1.312595	1.927009	0.952703	1.68885	0.007959	1.678822	1.290185	2.941167	0.037347	0.190106
Pnmal2	-1.009561	7.985537	-2.723089	0.016067	0.068087	-4.06326	7.888584	6.162401	7.630966	8.6989	7.473672	7.870025	7.993025	8.404088	8.232377	8.074523	8.488093	8.201903	8.616551	8.069388
Glra2	-1.450555	2.32634	-2.723028	0.016068	0.068087	-3.516419	2.01613	-0.186163	1.514261	3.182467	1.143601	2.244393	2.206902	3.201289	2.734001	2.90068	2.81321	2.516041	3.614885	3.088394
Myo5b	-0.699826	6.234555	-2.722777	0.016076	0.068097	-4.076197	6.404169	5.366716	6.64608	6.994894	5.927755	6.053257	6.545779	6.168376	6.573038	5.947201	6.102197	5.961196	6.364974	6.249758
Exoc4	-0.561173	5.151211	-2.721885	0.016105	0.068194	-4.020046	5.139392	4.535265	4.709794	5.400752	4.705768	4.891948	5.246787	5.441205	5.20918	4.952365	5.507311	5.460213	5.832633	5.130032
Tmem200c	-0.628858	5.135799	-2.720811	0.016139	0.068315	-4.015493	5.143805	4.509329	4.462108	5.285703	5.107284	4.281144	5.151787	5.571764	5.159118	5.233536	5.144558	5.769947	5.631121	5.497134
Atl1	-0.476549	7.581932	-2.719857	0.01617	0.068421	-4.081395	7.36647	6.609576	7.156348	7.757632	7.29014	7.285109	7.937532	7.783143	7.992429	7.720798	7.620728	7.737756	7.90467	7.993288
Polr3g	0.61365	3.190205	2.717895	0.016233	0.068664	-3.820846	3.016775	3.806886	4.214137	3.595195	3.596165	4.075375	3.190506	2.505864	3.059216	2.783118	2.346454	2.859272	2.689088	3.106782
Peo1	-0.448671	5.643232	-2.717101	0.016259	0.068748	-4.074946	5.858632	5.267699	5.456031	6.204544	5.666949	5.942639	5.67752	5.655839	5.518052	5.637447	5.519896	5.504661	5.604033	5.523038
Aaas	0.478979	5.492015	2.716102	0.016291	0.06886	-4.056189	5.348806	6.177919	5.633536	5.287413	5.761253	5.618185	5.6496	5.366574	5.408039	5.521092	5.238925	5.255355	5.130412	5.526457
Kcns2	-1.360248	2.924621	-2.714932	0.016329	0.068988	-3.644131	2.682606	0.310827	2.626517	3.533136	1.600747	2.434761	2.905804	3.897831	3.472337	2.998871	3.633241	3.631738	4.066299	3.432519
Bcl2l12	1.3454	1.621203	2.714469	0.016344	0.068988	-3.347454	1.108109	2.904741	1.874203	0.485009	2.386673	1.275818	1.408645	2.186344	0.385075	2.048482	2.519368	1.930875	1.493524	1.260365
Hrsp12	-0.33868	5.606714	-2.714407	0.016346	0.068988	-4.076732	5.926787	5.668637	5.339731	5.857342	5.648642	5.563503	5.384156	5.682188	5.347591	5.849693	5.638612	5.873961	5.347714	5.397961
Lrrfip1	0.521441	5.547709	2.714396	0.016346	0.068988	-4.071394	5.306139	5.929223	6.056615	5.784144	6.054345	6.234928	5.64395	5.328951	5.512366	5.434063	5.045762	5.13436	4.924278	5.313401
Myo16	-0.614686	5.558465	-2.714282	0.01635	0.068988	-4.070731	5.672669	4.649501	5.273343	6.074915	5.626095	5.482427	5.626867	5.551489	5.692438	5.251354	5.627025	5.480898	6.070063	5.773779
Lsamp	-0.696048	6.461854	-2.71377	0.016367	0.069034	-4.096147	6.602329	6.029572	6.158221	7.371321	6.328548	6.647758	6.053507	6.514708	6.414418	6.016563	6.435151	6.442773	6.980019	6.489347
Klk11	-1.817774	0.170929	-2.713281	0.016383	0.069077	-3.165391	0.592497	-1.463779	-2.147119	1.000693	-0.18071	0.772673	0.282279	1.112357	0.317129	0.413143	0.885831	0.514819	1.675978	0.554014
Vps41	-0.605367	8.058925	-2.711328	0.016446	0.069314	-4.080114	8.196117	7.306779	7.6983	8.630326	8.128915	8.095932	8.199052	8.08735	8.057968	7.838555	8.253207	7.888901	8.439706	8.009871
Trim21	0.977072	1.847064	2.711201	0.01645	0.069314	-3.484063	2.127257	2.557778	2.700244	0.896996	2.240661	2.217002	1.408645	2.077116	1.867508	2.048482	1.557647	2.502617	1.11438	1.009844
Eif1ad	0.43897	4.975368	2.71026	0.016481	0.069419	-4.025133	4.970829	5.35766	5.273343	4.512069	5.161783	4.907024	5.044381	4.930944	5.032426	5.018886	5.008293	4.895909	4.828405	4.763548
Rgs7	-0.63367	6.869347	-2.709962	0.016491	0.069436	-4.101167	7.329665	6.245266	7.0638	7.718243	7.164159	7.086111	6.594433	6.832111	6.796114	6.535901	6.884637	6.447159	6.935972	6.551079
Vps37d	-0.444642	4.488333	-2.709635	0.016502	0.069457	-3.994061	4.840987	4.393585	4.195136	4.532399	4.415294	3.96856	4.447353	4.620407	4.473365	4.5783	4.825216	4.412503	4.686744	4.517745
Ss18	0.338821	6.72516	2.709076	0.01652	0.069494	-4.104983	6.615096	6.834471	6.778562	6.582167	7.04923	7.086111	6.625343	6.614832	6.630156	6.706251	6.769468	6.738819	6.438076	6.698551
Oprm1	-1.772077	-0.037321	-2.708994	0.016523	0.069494	-3.15242	0.031429	-2.267963	-0.067513	1.300739	-0.718553	-0.548781	-0.169545	0.76329	0.837186	0.134646	0.846404	-0.24119	1.316372	0.321874
Gcnt1	0.962053	2.829102	2.708836	0.016528	0.069494	-3.66971	2.387517	2.582864	3.111733	2.337441	3.482634	4.028875	2.629097	2.795149	2.539002	2.966878	2.639567	3.440827	2.379013	2.516844
Sh2b3	0.597647	5.269865	2.708509	0.016538	0.069515	-4.03955	5.247895	5.5207	5.514681	4.424653	5.556259	5.104925	5.377362	5.425693	5.134654	5.386484	5.38366	5.730184	4.806206	5.206625
Eno1	0.382558	6.827283	2.708298	0.016545	0.069519	-4.106978	7.03352	7.116956	7.14195	6.59741	7.410661	7.120242	6.588563	6.538597	6.750153	6.720712	6.751572	6.651802	6.547508	6.6263
Casp6	0.724367	1.641791	2.707929	0.016557	0.069531	-3.436289	1.541696	1.747452	1.966737	1.065902	2.212449	2.04074	1.21902	1.628188	1.326257	1.898256	1.864385	1.930875	1.1849	1.868007
Fam96a	0.627947	3.362719	2.707862	0.01656	0.069531	-3.807414	3.474632	3.74155	3.530855	2.787519	4.136256	3.55809	3.280644	3.012464	3.110295	3.530893	3.448101	3.46871	3.160849	2.992761
Park2	-0.664223	2.810103	-2.707633	0.016567	0.069538	-3.758368	3.522857	2.248512	2.924461	3.051752	2.597694	2.654873	2.73209	3.126505	2.707706	2.423294	2.986432	2.9008	3.203938	2.489076
Sptan1	-0.32916	10.89354	-2.707018	0.016587	0.069599	-4.000343	10.77671	10.47481	10.65351	10.9703	10.45729	10.59104	11.22336	11.04383	11.21289	10.92155	10.93328	10.93945	11.15915	11.15322
Nkain3	-1.552778	2.56519	-2.706753	0.016596	0.069611	-3.64666	2.620642	-0.572649	1.890044	3.670505	2.575715	2.537984	2.930765	2.901501	2.928701	2.641672	3.013384	2.568523	3.589113	3.012401
Amdhd2	0.655862	4.538219	2.706278	0.016612	0.069653	-3.958827	4.467845	4.934131	4.823454	3.589663	4.531181	4.383247	4.674929	4.873301	4.575048	4.850841	4.594813	4.851593	4.033233	4.421864
Phyh	-0.399968	6.0411	-2.706016	0.01662	0.069664	-4.107951	6.652735	6.244284	5.809385	6.417045	6.221145	6.267836	5.891303	5.720831	5.991138	5.937604	5.773478	5.983111	5.77093	5.918729
Tars	0.507954	5.593322	2.705682	0.016631	0.069686	-4.088846	5.264199	5.667172	6.204878	5.865378	6.058313	6.369298	5.495004	5.371209	5.50284	5.190317	5.296745	5.338932	5.363002	5.352453
Gdpd1	-0.428585	5.343946	-2.702985	0.016721	0.070036	-4.055795	5.055231	4.591969	4.790075	5.372033	4.938861	4.827752	5.722886	5.585789	5.775484	5.608362	5.496209	5.314172	5.682296	6.094869
Elp5	0.402033	4.833675	2.702434	0.016739	0.070068	-4.033656	4.843702	5.250188	4.975957	4.514991	5.176461	4.911303	4.903326	4.643578	4.878873	4.839106	4.781463	4.718575	4.652936	4.636413
Pmepa1	0.472982	5.662587	2.702398	0.01674	0.070068	-4.10433	6.002839	5.925551	6.438245	5.61062	6.2673	6.474972	5.111417	5.173124	5.154258	5.392518	5.637168	5.542871	5.248305	5.329149
Tgm1	1.626299	0.956677	2.701537	0.016769	0.070129	-3.243357	0.785575	1.769471	2.61703	-0.600663	1.05105	0.881356	1.478026	1.356279	1.102347	0.646479	0.631286	0.618025	0.698429	1.260365
Tbc1d10a	0.463326	4.527619	2.701511	0.01677	0.070129	-4.018396	4.710243	4.771807	5.115554	4.277563	5.150675	4.753435	4.263083	4.429191	4.429792	4.19213	4.530966	4.390807	4.337929	4.102607
Il18bp	1.009056	3.949033	2.701276	0.016777	0.070129	-3.85604	3.569521	4.951089	4.369101	2.934433	4.220119	3.456346	4.091065	3.845499	4.018418	4.394093	3.990531	3.880344	3.394539	4.279179
Hipk2	-0.85542	6.042247	-2.701262	0.016778	0.070129	-4.112714	6.411507	5.952268	6.149199	6.654172	4.843207	5.76962	6.039231	6.104401	6.008745	6.35409	5.46726	6.363303	6.537436	5.962298
Galnt7	-0.557382	3.312777	-2.700223	0.016813	0.070249	-3.854127	4.045673	2.843782	3.203331	3.302317	3.036313	3.308464	3.445603	3.211662	3.432752	3.177227	3.196995	3.575147	3.394539	3.365375
Tspan31	0.51708	5.810762	2.699753	0.016828	0.070284	-4.085646	5.630846	5.998447	5.592981	4.932758	6.008935	5.574336	5.809551	5.968785	5.788087	6.194024	5.923101	6.186745	5.792263	5.978616
Ppie	0.523708	4.222662	2.69962	0.016833	0.070284	-3.963659	4.172016	4.798824	4.388746	3.819751	4.475885	4.398567	4.412334	4.161323	4.116621	4.402091	3.953901	3.926315	3.876936	4.299305
Pbk	2.843154	0.100978	2.699448	0.016838	0.070284	-3.201014	0.243629	2.064844	0.604933	-0.941548	3.585206	3.150929	-1.73438	-0.048075	-1.10526	-0.806258	-0.510232	0.081289	-0.211665	-0.886923
Dnajc5	-0.797515	6.171361	-2.698751	0.016862	0.070316	-4.123875	6.71781	5.428566	6.613653	7.143427	6.034339	6.450925	5.721099	6.155	5.851008	5.659299	6.292081	5.952583	6.518787	5.883167
Crlf2	0.741437	3.528155	2.698731	0.016862	0.070316	-3.726648	3.045357	3.563961	3.295418	2.154676	3.434596	2.974059	3.783728	3.727562	4.018418	4.07195	3.656224	4.166573	3.646466	4.003043
Appl2	-0.533499	6.356984	-2.698521	0.016869	0.070316	-4.119797	6.659685	6.306788	5.709149	6.230552	5.939636	5.697128	6.619598	6.468958	6.462388	6.780987	6.247374	6.701118	6.440781	6.753562
Gpc4	-0.944068	5.552261	-2.698446	0.016872	0.070316	-4.076485	5.121607	3.822768	4.96665	6.086747	4.643738	5.197177	5.910253	6.063464	6.481914	6.136463	5.523026	5.897231	5.638993	6.280457
Cntln	-0.453405	3.378534	-2.698244	0.016879	0.070316	-3.89503	3.683012	3.479652	3.042552	3.972106	3.458815	3.579757	3.270902	3.262441	3.149895	3.28495	3.243265	3.220743	3.349197	3.454224
Ppp2r5a	0.375534	5.032725	2.698169	0.016881	0.070316	-4.03762	4.60537	5.121276	5.079744	4.675069	5.111109	4.753435	5.072692	5.170467	5.107255	5.029251	5.078034	5.269295	5.180889	5.252767
Optn	-0.442256	6.823336	-2.697257	0.016912	0.070407	-4.128197	7.098039	6.452565	6.924138	7.39054	6.811325	7.019298	6.79807	6.853799	6.68594	6.605953	6.811663	6.687093	6.882603	6.519686
LOC681383	1.150589	-0.293299	2.697166	0.016915	0.070407	-3.171811	-0.217457	1.397635	0.442561	-0.411255	0.691028	0.571087	-1.450335	0.377517	-0.632503	-0.420466	-0.318799	0.28321	-1.413173	-0.886923
Lmod1	0.610756	4.294231	2.696549	0.016936	0.070469	-4.047129	4.796842	5.36852	4.775226	4.514991	5.176461	5.738653	3.410541	3.910622	3.592171	3.92982	3.58015	4.179285	3.431265	3.803307
Cfl1	0.342296	9.032793	2.695818	0.01696	0.070547	-4.090962	8.843872	8.928743	9.055074	8.598311	9.180995	9.093894	9.03597	9.197862	9.155315	9.063726	9.229927	9.081793	9.086782	8.909857
Postn	0.865909	1.546871	2.695059	0.016986	0.070627	-3.570843	2.035255	2.36878	2.238266	1.548999	2.927714	2.99033	1.091998	0.58326	1.393785	1.14286	1.533025	0.460309	0.649393	1.29285
Acvr1c	-2.521826	0.743564	-2.694894	0.016991	0.070627	-3.251759	1.108109	-3.260591	0.484899	1.9796	-0.18071	0.846031	1.544223	1.429209	1.867508	0.413143	1.740817	1.252437	2.466543	1.445134
Gad2	-1.160856	5.391057	-2.694641	0.017	0.070632	-4.089375	5.20842	3.747109	4.964781	6.406856	4.598129	5.262271	5.428646	5.728075	5.243904	5.092395	5.893184	5.704031	6.590357	5.649152
Nsl1	1.097229	0.700221	2.69444	0.017007	0.070632	-3.329985	0.643243	1.833582	1.089503	0.485009	2.268331	1.353343	0.118118	-0.048075	0.385075	0.799637	1.135223	0.618025	0.182287	-0.03329
Ghrl	-0.977701	0.888291	-2.694334	0.01701	0.070632	-3.390237	1.108109	0.480615	1.143363	1.892408	-0.107758	0.772673	1.335758	1.356279	1.01926	0.938085	1.03357	0.460309	1.040235	0.844371
Stmn2	-0.655129	8.942887	-2.693992	0.017022	0.070656	-4.080402	9.336572	8.129242	8.862908	9.589134	9.199079	9.045444	8.745008	8.880313	8.978623	8.776119	9.033551	8.731122	9.190982	8.705592
Cox11	-0.545235	2.736884	-2.693574	0.017036	0.07069	-3.725235	2.987615	2.411416	2.10888	2.87199	2.424057	2.634313	2.746221	2.738882	3.120298	2.745897	3.057213	2.606669	3.160849	2.942457
Lin7a	-1.248574	0.133245	-2.692771	0.017063	0.07075	-3.316234	0.739674	-0.813242	0.484899	1.700419	-0.107758	0.61372	-0.169545	0.871929	0.091681	-0.027758	0.071497	-0.657145	0.791767	-0.204094

Krt71	1.059372	0.575924	2.692675	0.017066	0.07075	-3.361915	1.310059	1.453108	2.135702	0.166125	1.803478	1.450626	0.625837	-0.992527	1.180909	-0.537962	0.001431	-0.334482	0.111637	0.929479
Tmem231	-0.455287	4.447365	-2.692624	0.017068	0.07075	-3.986339	4.403064	3.544937	4.02265	4.324952	4.039207	4.005051	4.438678	4.729108	4.621564	4.888332	4.773602	4.832942	4.863765	4.850685
Auh	-0.425066	6.004324	-2.691407	0.017109	0.07088	-4.13026	6.080846	5.517451	5.831087	6.413135	6.106057	5.834142	6.087204	5.983995	6.169105	5.952657	6.191437	5.86743	6.057196	5.993516
Paqr9	-0.920877	3.63397	-2.691349	0.017111	0.07088	-3.885867	3.334978	2.21681	3.319014	4.470529	3.060255	3.359461	3.632178	4.308732	3.895674	3.448339	4.061116	3.880344	4.156802	3.869418
Rasa2	-0.553664	3.791337	-2.690296	0.017147	0.070997	-3.918555	3.627383	3.719097	3.489863	4.203428	2.95351	3.359461	4.358158	3.811801	4.268256	3.796617	3.78171	3.686193	4.070962	4.070176
Pmaip1	1.028393	0.867343	2.690153	0.017152	0.070997	-3.384281	1.212615	2.184396	2.148929	0.43652	1.510943	1.353343	1.135593	0.517885	0.931093	-0.027758	-0.149817	0.567345	1.001683	0.257494
Sez6l	-0.913777	8.450607	-2.689997	0.017157	0.070997	-4.12162	8.084064	6.497783	8.1352	8.840584	7.656895	7.90799	8.926406	8.974316	9.169232	8.537863	8.726972	8.761379	9.069558	9.025524
Pam	-0.441595	8.732881	-2.688733	0.0172	0.071123	-4.111494	8.613745	8.134554	8.206315	8.923464	8.270331	8.694121	8.775471	8.99417	9.030098	8.938722	8.752525	8.886808	8.949997	9.093805
Pianp	-0.449931	7.67287	-2.688588	0.017205	0.071123	-4.130129	7.987946	7.427242	7.809455	8.215191	7.709254	7.661685	7.205968	7.432519	7.711499	7.595186	7.583744	7.624186	7.635152	7.828908
Syndig1	-1.023454	4.764707	-2.688581	0.017205	0.071123	-4.041499	5.216821	2.582864	4.14651	4.85187	4.750601	4.48148	5.030014	5.235456	4.961869	5.000567	5.250298	4.995108	5.235902	5.034811
Zmat4	-0.829071	5.182294	-2.688388	0.017212	0.071126	-4.113922	5.071534	4.98677	5.007158	6.600164	5.394602	4.672562	5.009657	4.850248	5.253025	5.301385	5.345284	5.103568	4.916528	5.084367
Ttbk1	-0.708914	5.642455	-2.688026	0.017224	0.071133	-4.130229	6.382863	5.39532	5.652234	5.943365	5.093814	5.669608	5.548398	5.539186	5.481658	5.192625	5.563099	5.875263	6.362124	5.32719
Cacna2d2	-0.625008	4.850274	-2.687987	0.017225	0.071133	-4.080814	5.029233	5.263826	4.427251	5.458049	4.241217	4.594888	5.33825	4.604749	5.102217	4.7573	4.653101	4.401696	4.850267	5.238245
Camta1	-0.681774	7.728145	-2.687186	0.017253	0.071222	-4.126128	7.865394	7.526748	7.667325	9.005222	7.658857	8.148305	7.529226	7.478884	7.548368	7.296376	7.63923	7.395904	7.923805	7.518007
Pmp22	0.443255	6.345473	2.685935	0.017296	0.071334	-4.14481	6.377253	6.530388	6.678934	6.025571	6.79952	6.564629	5.775507	6.484013	5.893911	6.397741	6.293914	6.82763	6.117033	6.090256
Lima1	0.492659	5.376181	2.685677	0.017305	0.071334	-4.105298	5.339219	5.870585	5.422387	4.996864	5.6394	5.702076	5.404346	5.333708	5.220848	5.394523	5.142525	5.627247	4.86645	5.344726
Dmtf1	-0.500503	5.209682	-2.685602	0.017307	0.071334	-4.096857	5.06457	4.585778	4.712023	5.630964	5.014338	5.093673	5.43302	5.430142	5.362416	5.140992	5.576715	5.218879	5.44431	5.271227
Plekho1	0.434172	5.823191	2.685561	0.017308	0.071334	-4.139237	6.120563	6.549602	6.061869	5.698063	6.504641	6.251898	5.417651	5.872367	5.28894	5.940353	5.822558	5.717167	5.103357	5.204492
Arhgap35	-0.609324	7.545406	-2.685528	0.01731	0.071334	-4.14189	7.739861	6.758752	7.372612	8.005515	7.199174	7.725037	7.541432	7.726396	7.455127	7.417215	7.810033	7.563257	7.947768	7.38213
Scamp4	0.580518	5.726404	2.684389	0.017349	0.071471	-4.104005	5.499292	5.71901	5.716941	4.688073	5.936405	5.292109	5.59599	5.901494	5.901181	6.048759	5.954731	6.015372	5.827132	6.104052
Lifr	-0.830459	3.853082	-2.683698	0.017373	0.071544	-3.990221	4.251852	3.24835	4.354903	4.613698	3.238702	3.536092	3.824257	3.972932	4.111616	3.223093	3.848089	3.900956	4.463807	3.468514
Vav3	-0.886981	5.004109	-2.681848	0.017436	0.071782	-4.08495	5.059908	3.61957	4.69409	5.501419	4.051203	5.18309	5.490813	5.302504	5.204486	5.564496	5.641494	5.025932	4.806206	4.96597
Sntg1	-0.670253	2.558605	-2.680047	0.017499	0.072014	-3.702316	2.372556	2.064844	2.262807	3.099251	2.124346	1.910004	2.943086	2.665313	2.797714	2.934159	2.843564	2.330954	3.125428	2.622863
Otog	-1.5762	1.030061	-2.679315	0.017524	0.072094	-3.264721	0.307952	-1.271663	0.56602	1.571628	-1.585779	0.336685	2.165439	1.596873	2.47808	2.127786	1.482482	1.826994	1.859646	2.211707
Jade3	-0.640737	2.306162	-2.677963	0.017571	0.072263	-3.72573	2.595092	1.854335	2.645305	3.059778	1.877213	2.386402	2.380012	2.307359	2.204856	2.06872	1.959843	2.203999	2.549064	2.516844
Scn2a1	-0.915908	8.747918	-2.676711	0.017615	0.072417	-4.124601	8.571586	7.575603	8.460009	9.850141	8.069209	9.065739	9.033271	8.743349	8.970871	8.598064	8.725615	8.728421	9.115134	8.967749
Adcyap1	-1.178064	3.08725	-2.675762	0.017648	0.072518	-3.891942	2.927466	2.47979	2.47722	4.826222	2.386673	3.622139	3.072094	2.650138	3.038267	3.267546	2.627988	3.390696	3.178239	3.475607
Tnfsf10	-0.925412	0.782326	-2.675664	0.017651	0.072518	-3.407515	1.831529	1.010791	0.25957	0.966949	0.422095	0.336685	1.17791	0.972957	0.512061	1.023513	0.535373	1.284167	0.546019	1.009844
Ptprt	-0.998395	4.28659	-2.674987	0.017675	0.072578	-4.03658	4.424983	4.091039	4.360599	5.042128	2.810268	3.713177	4.587372	4.35626	4.227195	3.844356	4.291424	4.440934	5.428048	4.482972
Elavl4	-0.879936	4.738105	-2.674903	0.017678	0.072578	-4.137698	5.233477	4.817439	4.768815	6.443357	4.824689	5.44897	4.063116	4.472969	4.217909	3.88487	4.802218	4.136468	5.057107	4.225496
Dlgap2	-0.72462	5.224726	-2.674487	0.017692	0.072613	-4.120773	5.392103	4.588877	5.235097	5.57186	4.342316	4.854663	5.395408	5.565712	5.408039	4.976667	5.532373	5.22093	6.064229	5.041995
Rasgrp1	-0.943686	8.467845	-2.674038	0.017708	0.072633	-4.141703	8.621082	7.119637	7.949262	9.049725	7.854672	8.645989	8.435974	8.978689	8.557659	8.135157	8.944168	8.648776	9.223933	8.389915
Sst	-0.675723	6.715535	-2.673999	0.01771	0.072633	-4.171957	6.816461	5.773142	6.156585	7.117228	6.352176	6.882158	6.70979	6.467876	6.801564	6.893052	6.695124	6.856278	7.19122	7.320441
Eif4ebp3	0.893664	3.903924	2.673488	0.017727	0.072682	-3.896236	3.357948	4.806831	3.759312	3.043681	4.329239	3.491064	4.05746	4.123366	4.055381	4.361652	3.990531	3.83286	3.495104	4.060775
Mtpap	0.339019	4.802646	2.673218	0.017737	0.072696	-4.082425	4.661839	4.934131	5.016208	4.583397	5.080218	4.807235	4.760958	4.718236	4.793937	4.930413	4.825216	4.680333	4.808999	4.692276
Gpsm1	0.668121	6.12671	2.672766	0.017753	0.072736	-4.162306	6.075082	7.02899	6.769492	5.583041	6.324435	5.856504	6.011712	5.78128	6.057711	6.150773	6.018201	5.901073	6.065397	6.173301
Rptor	-0.323072	6.414875	-2.67156	0.017795	0.072867	-4.171858	6.327695	5.94261	6.204086	6.463916	6.155054	5.989933	6.642444	6.617761	6.550097	6.420559	6.601279	6.63036	6.703526	6.57769
Sft2d2	1.112713	3.687553	2.671372	0.017802	0.072867	-3.901233	4.406741	4.10842	4.337679	1.962578	4.154906	4.297582	3.36548	3.960684	3.04878	3.595091	4.116027	4.145134	2.994118	3.270693
Rars	0.499694	5.47518	2.671265	0.017806	0.072867	-4.143411	5.043471	5.419891	5.986514	5.651025	6.06227	5.927932	5.390917	5.35258	5.351842	5.343524	5.355852	5.281137	5.366799	5.154545
Klhl42	-0.748794	3.751133	-2.671032	0.017814	0.072867	-3.971897	3.896775	2.904741	3.642605	4.324952	3.106978	3.784887	3.706333	4.073048	3.641623	3.545408	4.152854	3.625558	4.456698	3.774841
Mki67	1.863566	3.445347	2.671001	0.017815	0.072867	-3.702224	1.568201	4.010125	2.250588	2.475838	4.852378	4.543439	3.713543	3.804966	3.805565	3.545408	3.173291	3.60686	3.488149	3.571405
Arsb	-0.555575	6.956568	-2.670425	0.017835	0.072925	-4.17954	7.117239	6.213492	6.590209	7.182084	6.79596	6.776472	6.587582	7.365578	6.832314	6.994394	7.086369	7.539626	7.243784	7.079873
Rpl35	0.66068	6.922128	2.668596	0.0179	0.073165	-4.180879	7.200077	8.101301	7.262457	6.528235	7.599164	7.127156	6.703467	6.487219	6.651867	6.933619	6.663571	6.437491	6.438076	6.789579
Gng2	-0.771186	8.28992	-2.667673	0.017933	0.073239	-4.149603	8.781351	7.375688	8.372334	9.071348	8.325497	8.553889	8.188418	8.152069	8.186403	7.915999	8.486491	8.027753	8.696043	7.930798
Adtrp	-0.740755	1.241996	-2.667483	0.017939	0.073239	-3.447027	1.669604	0.973336	0.353965	1.21709	0.691028	0.915838	1.726334	1.239467	1.326257	1.540753	1.533025	1.488652	1.60059	1.80183
Rarres2	0.683218	4.687771	2.667454	0.01794	0.073239	-4.145869	5.66348	5.904562	5.411453	4.804892	5.925585	6.397786	3.81758	4.038501	3.91304	3.826639	3.744661	4.257294	3.777752	4.217057
Opcml	-1.20841	6.563131	-2.667396	0.017943	0.073239	-4.181622	6.644196	4.738705	6.454316	7.596632	6.016093	6.422728	6.241066	7.02753	6.851956	6.025651	6.984493	6.678164	7.483845	6.737425
Ica1	-0.393316	5.794584	-2.667023	0.017956	0.073251	-4.168709	5.898344	5.208484	5.755281	6.021467	5.682458	5.614253	5.880133	5.939438	5.889532	5.966206	5.97886	5.794942	5.846296	5.677109
RGD1308923	0.501469	3.447918	2.666969	0.017958	0.073251	-3.889456	3.453461	3.673115	3.565791	2.916866	3.909025	3.541623	3.454237	3.567159	3.448717	3.51623	3.609886	3.284884	3.220819	3.25429
Scn1a	-0.771386	5.215547	-2.665773	0.018	0.0734	-4.128676	4.785591	5.005446	4.808946	5.974478	4.154906	4.518413	5.717517	5.469576	5.653363	4.896847	5.388815	5.181457	5.971269	5.536666
Zhx3	-0.663359	5.549558	-2.665563	0.018008	0.073405	-4.177102	6.102542	5.449176	5.895281	6.447187	5.414861	5.685933	5.154438	5.467413	5.173599	4.960511	5.645806	5.314172	6.011844	5.005709
Fam110c	1.739885	-1.238494	2.664755	0.018037	0.073498	-3.180473	-0.518419	0.118359	1.705429	-0.941548	0.266146	1.221719	-1.450335	-0.81357	-1.536754	-1.838237	-1.500575	-2.668207	-1.904936	-2.653844
Ppic	1.233318	2.323021	2.664406	0.018049	0.073524	-3.68135	2.515609	2.991607	2.924461	1.430203	2.987204	3.722947	1.135593	2.888628	1.690848	2.127786	1.319276	3.127617	1.983515	2.030937
Magi1	-0.467141	5.00358	-2.664205	0.018056	0.073528	-4.141276	5.108122	5.210498	4.990724	5.664246	4.622454	4.881081	4.983054	5.010338	4.986685	4.885482	4.981204	4.928903	5.091933	4.754829
Calca	1.467268	0.243798	2.664023	0.018063	0.07353	-3.29176	-0.410969	0.725555	1.554575	0.386344	1.947363	1.302125	1.17791	-0.654381	-0.386063	-0.311823	-1.500575	-0.78301	1.347449	0.65743
Tmsb4x	0.506599	9.555001	2.663585	0.018078	0.073546	-4.113029	9.678194	9.974927	10.03681	9.868677	10.5127	10.59408	9.399055	9.10961	9.151814	9.148621	9.268892	9.118421	9.02565	8.884768
Dync1i1	-0.750029	7.312367	-2.663567	0.018079	0.073546	-4.188915	7.448567	5.949859	6.940869	7.700706	7.266752	7.139519	7.477753	7.510213	7.60155	7.196449	7.668845	7.2579	7.809882	7.414783
Etv6	0.552914	2.396831	2.663043	0.018098	0.073598	-3.758173	2.41698	3.019437	3.131857	2.511387	2.962008	2.932562	1.809437	2.400986	1.636128	2.291729	2.049375	2.761217	2.186205	1.755962
Trhr	-1.667825	1.609194	-2.662657	0.018112	0.073625	-3.566343	1.48718	-0.465897	1.493674	3.309066	1.143601	0.982423	1.782265	1.318383	1.891068	1.355481	0.846404	1.738143	2.783013	3.510555
Rpl6	0.804934	2.848997	2.662511	0.018117	0.073625	-3.728542	2.342161	3.294639	3.028308	2.169587	3.458815	2.434761	2.854546	3.126505	2.55384	3.28495	2.968181	2.619163	2.933966	3.041369
Cadm3	-0.733778	8.369593	-2.66228	0.018125	0.073634	-4.173813	8.333549	6.835124	7.955862	8.536748	8.039657	7.982321	8.417053	8.774516	8.832274	8.408611	8.690038	8.652389	8.937605	8.783744
Ndc80	2.076991	0.293013	2.661897	0.018139	0.073665	-3.269232	-0.760787	1.339943	0.398943	0.22441	2.79132	2.458346	-0.169545	0.04768	-0.27702	-0.311823	-0.510232	0.514819	-0.040977	-0.116166
Paqr5	-1.451545	2.399038	-2.66128	0.018161	0.073715	-3.742343	4.078311	2.493084	0.945338	2.184345	1.685287	1.648138	2.8014	2.054242	2.583064	2.911926	3.73929	2.300247	2.003164	2.503027
Herc3	-0.876133	7.192324	-2.661215	0.018163	0.073715	-4.192548	7.293883	5.797457	6.8547	7.979837	6.937871	7.446383	7.380318	7.219021	7.338939	6.954163	7.434697	7.152788	7.678187	7.235773
Satb1	-0.614649	6.252367	-2.66102	0.01817	0.073718	-4.197194	6.674246	6.960055	6.076652	7.447089	6.237862	6.514181	6.056346	5.659633	5.885139	5.926557	5.975438	6.002316	6.150458	5.988566
Uba52	0.538322	8.446025	2.660672	0.018183	0.073744	-4.164036	8.81009	9.13296	8.927651	8.096346	9.270087	8.641167	8.119525	8.084531	8.304882	8.360939	8.084492	8.088544	8.020676	8.307106
Ptpre	-0.73249	5.891149	-2.660506	0.018189	0.073744	-4.172376	5.448478	4.489567	5.456031	6.387058	5.317234	5.472326	6.071857	6.433944	6.39498	6.128049	5.97886	6.399064	6.239092	6.288534
Pde4d	-0.974263	3.961483	-2.659388	0.018229	0.073882	-4.027889	4.227114	2.97275	3.929918	4.659309	3.137307	3.812601	3.920861	4.498613	3.974983	3.214035	4.414477	3.99982	4.906128	3.901371
Mpp7	-0.892457	2.745577	-2.659143	0.018238	0.073893	-3.797601	2.569081	1.558038	2.345554	3.644058	2.014259	3.014396	3.221182	2.964197	2.995434	2.795315	3.099749	2.816513	2.760099	2.890335
Rad51ap1	0.516787	2.960743	2.658483	0.018262	0.073965	-3.848391	2.843156	3.466233	3.164784	2.916866	3.546179	3.420773	2.979429	3.081965	2.772565	2.998871	2.750518	2.529342	2.62712	2.55752
Zfp799	-0.498029	3.85411	-2.658158	0.018274	0.073977	-4.017744	3.973144	3.49955	3.754994	4.304833	3.452798	3.870884	3.876582	4.032662	3.87219	3.656554	3.986003	3.936334	4.221137	3.629792
Cyp27a1	-0.551657	3.021917	-2.658059	0.018277	0.073977	-3.887129	3.664706	3.037697	2.682163	3.16769	2.60856	3.129052	2.880403	3.159031	3.383769	2.998871	2.958968	3.028062	2.892424	2.91141
Arhgef9	-0.980935	8.136734	-2.656559	0.018331	0.074171	-4.175495	8.470045	6.790057	8.238378	9.178545	7.859797	8.488633	8.164612	8.20105	8.097112	7.654555	8.291786	7.920792	8.566664	7.998221
Slc24a3	-0.810618	8.233177	-2.65614	0.018347	0.074208	-4.191873	7.907196	6.442321	7.365194	8.492406	7.766465	7.983591	8.693978	8.545862	8.730451	8.592178	8.511913	8.660912	8.772099	8.805895
Cdc42se1	0.34458	6.728708	2.655416	0.018373	0.074289	-4.207148	6.671193	6.729564	6.987169	6.534008	7.171505	6.898953	6.503768	6.871081	6.528664	6.679357	6.688172	6.84369	6.580581	6.529091
Kcns1	-0.845418	2.24669	-2.654974	0.018389	0.074329	-3.683015	2.917193	1.082904	1.858187	2.108996	1.981199	1.668504	2.467906	2.24812	3.130231	1.875459	2.684976	2.203999	2.639729	2.942457
Tmem144	-0.6639	3.697946	-2.654485	0.018407	0.074376	-4.013048	4.331374	3.466233	3.228468	3.937678	3.300313	3.888353	3.624549	3.630385	3.159628	4.005262	3.448101	4.261285	3.766298	3.847716
RGD1305014	-0.789484	2.814908	-2.652994	0.018461	0.074567	-3.853391	3.118917	1.632013	2.770386	3.468286	2.460496	3.129052	2.688847	3.19084	2.834633	2.600561	3.108108	2.794648	3.042398	2.802815
Sncb	-0.473572	8.997137	-2.652734	0.018471	0.074567	-4.151603	9.558742	9.075569	9.23251	9.656507	9.357221	8.870802	8.68346	8.738425	8.922746	8.724831	9.143997	8.607829	8.857224	8.533189
Calm2	-0.716933	8.034948	-2.652678	0.018473	0.074567	-4.191828	8.391516	6.995518	7.787423	8.484974	8.1537	7.954861	8.295296	7.812832	8.222835	7.921921	8.485689	7.801498	8.287275	7.900156
Prnp	-0.550174	10.47798	-2.652419	0.018482	0.07458	-4.116803	10.5489	9.51457	10.13074	10.65285	10.23938	10.32563	10.25318	10.70739	10.63332	10.71517	10.6813	10.82643	10.82874	10.6352
Oplah	0.694808	6.21511	2.651877	0.018502	0.07461	-4.183316	5.693887	6.633035	5.84436	5.062243	6.025245	5.771975	6.311657	6.866165	6.294763	6.865118	6.613808	7.04521	5.646821	6.360221
Mta2	0.381137	6.617522	2.651752	0.018507	0.07461	-4.21121	6.331568	6.900813	6.808043	6.289756	6.604357	6.453128	6.715188	6.757074	6.584824	6.429978	6.579653	6.720092	6.4006	6.686375
Ntng1	-0.459787	5.603414	-2.651712	0.018508	0.07461	-4.190283	5.613453	5.60271	5.108801	6.004935	5.358047	5.349996	5.501266	5.695184	5.939818	5.464933	5.457481	5.669908	5.724337	5.989805
RGD1307461	-0.537241	2.685356	-2.651265	0.018524	0.074651	-3.840533	3.016775	2.780133	2.709201	3.1898	2.640673	2.18908	2.343298	2.488905	2.870631	2.681644	2.833517	2.581351	2.794335	2.721621
Fcf1	0.468219	4.59384	2.650789	0.018542	0.074696	-4.086212	4.276172	4.747052	4.703085	4.199801	5.054623	4.313834	4.579502	4.540363	4.693673	4.797268	4.621334	4.653252	4.519446	4.680048
Tmem79	1.250698	0.739688	2.65051	0.018552	0.074712	-3.274633	0.307952	1.279848	1.430088	-0.705589	0.470544	0.571087	0.68572	0.517885	1.180909	0.938085	0.805871	1.375373	1.316372	1.193114
RGD1560884	-0.929524	1.343663	-2.650339	0.018558	0.074712	-3.553911	1.895723	1.679303	1.089503	1.856	0.422095	0.772673	2.206902	1.393205	1.549949	1.419918	0.805871	1.515634	1.770732	1.085968
Elac1	-0.740341	3.453354	-2.649886	0.018575	0.074754	-4.005645	3.771212	2.864389	3.855228	4.367604	2.874693	3.708268	3.318966	3.517851	3.432752	3.319142	3.345785	2.859272	3.805994	3.454224
Rybp	-0.611715	6.26164	-2.649479	0.01859	0.074789	-4.214116	6.263253	5.331995	6.122629	6.824594	5.818526	6.453128	6.431277	6.261188	6.479002	6.150773	6.230245	6.31324	6.466665	6.537588
Galnt9	-0.531443	7.406382	-2.64803	0.018643	0.074964	-4.222133	7.262273	5.998447	6.643746	6.955108	7.00417	6.662428	7.870978	7.838998	7.906172	7.907597	7.828488	7.936165	7.861927	8.023244
Wdr7	-0.972234	6.708237	-2.64796	0.018646	0.074964	-4.219681	7.005912	5.720423	6.783341	7.778147	6.291944	6.875056	6.407022	6.922509	6.692659	5.999533	7.019257	6.616406	7.399709	6.419348
Spred2	-0.581602	4.625455	-2.647492	0.018663	0.075008	-4.150018	4.922855	4.256089	4.725329	5.349294	4.516833	4.722115	4.551611	4.833553	4.571407	4.106622	4.594813	4.379835	5.011753	4.279179
Tmem8a	0.62723	5.039457	2.647132	0.018676	0.075037	-4.123689	4.67714	5.355842	5.477159	4.277563	4.836291	4.719677	5.552424	4.930944	5.319649	5.229048	4.972116	4.94388	4.9009	5.409117
Elmo1	-0.376551	6.220555	-2.646274	0.018708	0.07511	-4.225059	6.733996	6.64276	6.452317	6.920267	6.516948	6.193368	5.765135	5.888621	5.927054	5.904206	6.043481	6.051545	6.132728	5.936829
Tob1	-0.613515	5.071096	-2.646171	0.018712	0.07511	-4.19269	5.661942	4.393585	5.148852	5.787773	5.238981	5.649258	4.848708	4.820056	4.80328	4.844986	5.122035	5.025932	4.86645	4.831464
Slc35d2	0.903642	2.575157	2.64613	0.018713	0.07511	-3.654311	1.936985	3.055728	2.250588	1.548999	3.028244	2.217002	2.501617	2.888628	2.759824	2.911926	2.544222	3.212521	2.452318	3.022122
LOC690276	3.190015	0.833811	2.645393	0.018741	0.075194	-3.132424	1.693879	0.426209	0.914692	-3.260591	2.154315	1.275818	0.743216	1.934092	0.628769	1.597576	2.083688	2.36102	1.077784	0.844371
Sp9	-0.8632	1.133303	-2.645077	0.018752	0.075216	-3.451562	0.915118	-0.103798	0.715742	1.700419	0.606834	0.734546	1.408645	1.356279	1.426399	1.253084	1.430105	1.593679	1.837926	1.755962
Tmlhe	0.478598	2.828144	2.644454	0.018776	0.075284	-3.803611	2.445855	2.874583	3.042552	2.62393	3.036313	3.084277	3.116285	2.964197	2.882434	2.572488	2.771719	2.794648	2.748504	2.858134
Aldh16a1	0.832658	5.038302	2.643083	0.018826	0.075449	-4.122563	4.915133	5.619476	5.236646	3.800721	5.271819	4.570746	5.229287	5.159792	5.15669	5.601432	4.990317	5.294831	4.637302	5.102807
Zdhhc15	-0.63264	2.816633	-2.643008	0.018829	0.075449	-3.895836	2.927466	2.679063	2.977318	3.752019	2.448452	2.800388	2.867532	2.539194	2.834633	2.256873	2.415434	2.880186	3.212403	3.069767
Arhgap31	0.595198	4.569668	2.641894	0.018871	0.07559	-4.143474	4.95334	5.292622	5.487608	4.214255	4.740757	5.145445	4.263083	4.308732	4.277224	4.046664	4.152854	4.628746	4.298715	4.233886
Myh10	-0.543472	8.83452	-2.64139	0.01889	0.075616	-4.192533	8.743512	8.232674	8.47124	9.401361	8.370077	9.035678	9.017883	8.961311	8.976732	8.826388	8.884898	8.849682	9.041623	8.873737
Cdca3	2.851774	-0.237792	2.64138	0.01889	0.075616	-3.147718	-1.642648	1.010791	-0.491181	-1.223791	2.564599	1.668504	0.202532	-0.654381	-1.304935	-0.210791	-0.510232	0.460309	-0.305233	-0.621773
Cby1	-0.504362	3.762744	-2.639957	0.018943	0.075766	-4.069971	4.509471	3.418258	3.656563	4.026356	4.051203	3.892687	3.90205	3.517851	3.669138	3.368956	3.717602	3.744381	3.743115	3.578011
Il33	-0.423574	5.289803	-2.63979	0.018949	0.075766	-4.21359	5.916509	5.204449	5.306181	5.705755	5.627431	5.408679	4.89066	5.127283	4.73657	5.303522	5.242726	5.666902	5.028628	4.932857
Ndst4	-1.285164	3.346527	-2.639738	0.018951	0.075766	-3.932923	2.937667	2.769246	1.723223	4.605497	1.912714	3.794184	3.609168	3.798099	3.924502	3.448339	3.461211	4.028205	3.665088	3.357718
Tek	0.523936	5.202751	2.639661	0.018954	0.075766	-4.186664	5.408698	5.779937	5.414195	4.733936	5.566046	5.598419	5.324188	4.886311	5.408039	4.997931	4.564762	5.049203	4.916528	5.234069
Wdr55	0.38278	3.584854	2.639407	0.018964	0.075766	-4.05872	3.907194	4.180039	4.320248	3.884445	4.20588	4.392459	3.094359	3.358932	3.188437	3.327565	3.074378	3.292705	2.827778	3.270693
LOC100361083	-1.199366	3.201523	-2.639214	0.018971	0.075766	-3.910878	3.621067	2.151237	3.365078	3.665254	1.230571	2.99033	3.545949	3.852146	3.448717	2.733273	3.454671	3.516245	4.306643	3.142871
Elovl7	-1.193367	2.594355	-2.639068	0.018976	0.075766	-3.757192	3.759759	2.324846	1.245402	1.996424	1.877213	1.302125	2.717819	2.665313	2.917273	2.628098	3.401255	3.092205	3.186856	3.510555
Dusp3	-0.393151	8.536423	-2.639033	0.018978	0.075766	-4.206989	8.799292	8.652409	8.212431	8.63453	8.165245	8.263026	8.718823	8.402108	8.586582	8.610844	8.419015	8.686553	8.699246	8.664093
Racgap1	1.353169	0.058516	2.638438	0.019	0.07583	-3.33062	0.176305	0.973336	0.353965	-0.243853	1.622351	2.05628	-0.399109	0.04768	-0.632503	-0.537962	0.202178	-0.24119	-0.305233	-0.621773
LOC100174910	-0.84037	3.991542	-2.638199	0.019009	0.075841	-4.064111	4.346765	3.774588	2.626517	4.328278	4.184254	3.502454	4.267989	4.308732	4.646003	4.051757	3.639021	3.405197	4.379868	4.528015
Gale	0.715861	4.048391	2.637525	0.019034	0.075916	-4.018149	3.753999	4.80949	4.175882	3.335752	4.430682	3.611659	4.187425	4.067347	4.091419	4.290193	3.972332	3.956168	3.892823	4.20003
Hsd17b13	-0.630071	1.343655	-2.636356	0.019078	0.076067	-3.571145	1.693879	1.369077	1.004744	1.740915	1.082564	1.014597	1.63815	1.532126	1.769222	1.21727	1.482482	1.008331	1.465469	1.415936
Ptpra	-0.506428	8.42798	-2.635857	0.019097	0.076094	-4.219297	8.431766	7.533197	7.956332	8.647569	8.26862	8.376662	8.519849	8.624154	8.500171	8.635222	8.484887	8.707365	8.565426	8.745197
Gpr19	0.508183	4.005652	2.635649	0.019105	0.076094	-4.062302	3.992844	4.361326	4.549481	3.696477	4.36492	4.098075	3.479833	4.009066	3.675936	3.92982	3.765948	4.110154	4.116777	4.027379
Trim72	2.087552	1.26073	2.635391	0.019115	0.076094	-3.410873	0.539902	3.382986	2.977318	-0.02435	1.415175	1.302125	1.726334	1.067371	0.628769	1.804823	0.378374	1.152831	0.434663	1.634478
Lhx9	-1.449132	2.471136	-2.635353	0.019116	0.076094	-3.783486	2.730329	-0.465897	2.09528	3.486263	2.619344	2.398644	2.828218	3.000548	2.330799	2.39151	3.091342	2.691938	3.070609	2.721621
Zbtb7a	-0.597415	6.494319	-2.635334	0.019117	0.076094	-4.245212	6.623809	5.80147	6.482668	7.16172	6.255251	6.640688	6.386869	6.584221	6.425553	6.231534	6.601279	6.519759	6.878619	6.344811
Prodh	-0.780114	6.077143	-2.63412	0.019163	0.076251	-4.231298	6.518165	6.1351	5.608623	5.810545	5.128199	4.680104	6.527486	6.111343	6.433597	6.370487	6.274549	6.362375	6.389444	6.755748
Nsg2	-0.714656	10.48426	-2.633756	0.019177	0.076281	-4.138293	10.78348	9.540513	10.42077	11.26754	10.59388	10.69299	10.04085	10.48116	10.37397	10.45762	10.52244	10.48916	10.66897	10.44742
B4galnt1	-0.464499	7.605724	-2.633408	0.01919	0.076308	-4.242666	7.657012	6.852635	7.167764	7.8198	7.583416	7.492805	7.59999	7.749719	7.790356	7.8949	7.622552	7.7172	7.710295	7.829946
Hey2	0.501027	3.531218	2.633138	0.0192	0.076324	-4.066103	4.064414	4.4112	4.477916	3.617117	4.01082	4.419747	2.814871	3.059168	3.262563	3.14899	2.949696	3.447848	2.827778	3.069767

Oxr1	-0.512745	8.276241	-2.632364	0.019229	0.076366	-4.215704	8.594735	8.541215	8.392631	9.159881	8.248355	8.265953	8.160331	8.074974	8.107834	7.650364	8.234001	7.940216	8.486699	8.015357
Pdrg1	0.377824	5.431759	2.632202	0.019236	0.076366	-4.230469	5.671142	6.315233	5.995694	5.589986	5.832496	5.88941	5.089416	5.004383	5.206835	5.242473	5.210093	5.046893	4.987296	5.000801
Tbrg4	0.59835	4.948059	2.632175	0.019237	0.076366	-4.153191	4.543265	5.34305	5.53749	4.446243	4.941012	4.537915	4.92212	5.113519	5.086997	5.074991	5.128213	4.822175	4.797792	5.030001
Bspry	-0.40274	5.687188	-2.632077	0.01924	0.076366	-4.23909	5.949649	5.39532	5.71583	6.234102	5.937483	5.708237	5.386413	5.471736	5.649915	5.599695	5.546281	5.642628	5.70497	5.709055
Dxo	0.533307	4.098625	2.632023	0.019242	0.076366	-4.052532	3.865059	4.525594	4.399853	3.595195	4.230707	3.875271	4.187425	4.12885	4.111616	4.464524	4.172914	4.037544	3.772036	4.107181
Pnoc	0.735006	3.607691	2.630393	0.019304	0.076586	-3.976317	3.31946	3.397198	3.679531	3.137672	4.475885	4.247696	3.241276	3.534475	3.525993	3.850214	3.42821	3.931333	3.32598	3.544677
Troap	2.676812	0.954733	2.630074	0.019317	0.076609	-3.329732	-0.634519	2.679063	0.442561	-0.02435	3.494398	2.549011	0.357848	1.279465	0.17081	1.253084	0.321999	1.315214	0.249639	0.929479
Akap5	-1.11953	5.658528	-2.629223	0.019349	0.076679	-4.245281	6.069294	4.528825	5.846391	7.016705	5.290494	6.009024	5.33825	6.098823	5.529356	5.036976	6.048919	5.204441	6.212963	5.022756
Camk1d	-1.089971	7.160901	-2.62891	0.019361	0.076679	-4.251918	7.400978	5.742856	7.173842	8.117887	6.471546	7.281395	7.051663	7.656449	7.132054	6.605087	7.637427	7.264875	7.874317	6.854268
Rarg	0.606205	4.417139	2.628861	0.019363	0.076679	-4.109572	4.3275	5.068969	4.397084	3.805502	4.782135	4.422747	4.39898	4.455617	4.308181	4.5783	4.254248	4.433879	3.989574	4.692276
Vnn1	2.127791	-1.229314	2.628808	0.019365	0.076679	-3.077687	-2.659044	-0.366503	-0.86368	-1.788967	0.210178	-1.26448	-2.088409	0.301855	-2.155202	0.055728	0.535373	1.461155	-2.232431	-1.632384
Sdccag8	-0.391099	5.19528	-2.628764	0.019367	0.076679	-4.20621	5.146006	4.833205	4.71425	5.508766	5.058591	5.156301	5.283586	5.275563	5.122264	5.384467	5.316718	5.292883	5.338076	5.346662
Prrx2	2.671283	-0.473737	2.628615	0.019372	0.076679	-3.161271	0.307952	-0.186163	1.65069	-2.731185	0.562819	1.165512	-0.279766	0.645799	-1.304935	0.280604	-0.318799	0.34469	-2.656794	-0.621773
Reep5	-0.511477	8.877594	-2.62812	0.019391	0.076728	-4.213736	8.892042	8.300644	8.608316	9.462896	8.772927	8.977567	8.66126	8.849297	8.860893	8.952061	8.854324	9.033749	9.000056	9.063676
Scg3	-0.45312	9.172463	-2.627308	0.019422	0.076825	-4.211657	9.433239	8.967655	8.66163	9.09363	8.973262	8.640362	9.171403	9.313445	9.253159	9.497633	9.031904	9.465245	9.366816	9.547888
Exoc6b	-0.453994	6.988242	-2.627149	0.019428	0.076825	-4.261647	6.983317	6.340276	6.736469	7.39054	6.917262	6.936175	7.144612	6.988095	6.962418	6.88879	7.072019	6.968557	7.326048	7.193383
Cic	0.445862	8.282238	2.625767	0.019481	0.076994	-4.238172	8.393139	8.875171	8.942563	8.044711	8.35463	8.491137	8.086931	8.127625	8.033035	8.228627	8.001112	8.274047	7.957873	8.145849
Chpt1	-0.462687	4.671345	-2.625702	0.019484	0.076994	-4.208954	5.457367	4.806831	5.03592	5.17368	4.622454	4.934612	4.412334	4.12885	4.31255	4.320048	4.160911	4.674359	4.649823	4.772214
Camk2a	-0.694146	10.67685	-2.625093	0.019507	0.077061	-4.154935	11.32703	9.671535	10.6875	10.69183	10.33612	10.59199	10.81885	10.72803	10.71495	10.46789	10.63061	10.91031	11.20461	10.69567
E2f4	0.344665	5.935025	2.624854	0.019517	0.077072	-4.255824	5.950908	6.220503	6.497608	6.004935	6.485612	6.084853	5.763399	5.742456	5.672184	5.842364	5.805955	5.718619	5.60885	5.718054
Mknk1	0.367246	5.157287	2.624502	0.01953	0.077077	-4.181912	4.785591	5.167613	4.998051	4.600004	5.062547	5.004367	5.333578	5.478195	5.163961	5.606633	5.32032	5.549414	4.957391	5.219356
Arhgap10	-0.525089	4.054867	-2.624343	0.019536	0.077077	-4.089298	3.901994	3.452688	3.926082	4.500322	3.746377	3.601103	4.32559	4.213873	4.445789	4.353427	4.188764	4.288919	3.892823	4.027379
Sema3e	-0.885355	4.904165	-2.624323	0.019537	0.077077	-4.168412	4.442997	3.361401	4.058135	5.560592	4.151195	4.907024	5.456846	5.157111	5.485533	4.693564	5.202306	4.856878	5.77665	5.607789
Atad3a	0.557633	5.750276	2.623588	0.019565	0.077164	-4.230642	5.371558	6.171732	5.933998	5.193877	5.992438	5.258917	6.008784	5.793417	5.955267	5.966206	5.641494	5.616382	5.662352	5.967339
Col15a1	1.098395	1.058016	2.622915	0.019591	0.077241	-3.500922	1.108109	1.655851	1.116684	0.43652	2.198134	2.336365	0.798508	1.155984	1.142163	1.180545	0.631286	1.152831	0.375586	0.321874
Ubash3a	1.604505	-0.216751	2.621174	0.019659	0.077481	-3.329519	0.307952	2.279532	0.750864	-0.81875	0.562819	1.402804	-0.169545	-0.992527	-0.175642	0.055728	-1.142289	-0.657145	-1.413173	-0.886923
Shank1	-0.468339	10.29925	-2.620935	0.019668	0.077492	-4.172557	10.78692	10.33236	10.06826	10.56461	9.887882	10.5426	10.46625	10.11302	10.34487	10.21199	9.836505	10.2521	10.39417	10.38923
Sept1	0.863376	1.646703	2.61954	0.019722	0.077633	-3.596346	1.401336	2.619695	2.238266	1.097434	2.09374	1.426914	1.408645	1.80302	1.218642	1.964563	1.719153	2.170435	1.252133	1.158276
Nup88	0.342727	5.452179	2.619438	0.019726	0.077633	-4.225843	5.112631	5.551208	5.331054	5.140008	5.647325	5.349996	5.638278	5.526777	5.653363	5.589225	5.421042	5.414351	5.42623	5.565205
Itpka	-0.767957	7.019437	-2.619394	0.019728	0.077633	-4.274392	7.101446	5.148837	6.29687	6.686928	6.717207	6.336264	7.496683	7.556221	7.579062	7.589069	7.540392	7.42555	7.393208	7.418469
Pm20d2	-0.54085	4.76754	-2.61935	0.01973	0.077633	-4.210804	4.835542	4.628571	4.742881	5.585823	4.342316	4.990279	4.61073	4.840254	4.281688	4.850841	4.862749	5.072104	4.698844	4.461699
Sypl2	1.143636	2.668785	2.617471	0.019803	0.077896	-3.920704	2.357438	4.305905	4.716472	3.083591	3.452798	3.006419	1.607516	1.830204	2.04602	2.127786	2.346454	2.594065	1.859646	2.310209
Pcyox1	-0.677972	6.882739	-2.617294	0.01981	0.077898	-4.279664	6.844516	5.721836	6.619603	7.257296	6.336739	6.810088	6.739232	7.196348	7.01965	6.883087	7.005899	7.286088	7.427243	7.224766
Rwdd2a	-0.854001	1.603258	-2.616164	0.019854	0.078046	-3.707921	2.213741	0.480615	2.039558	2.363612	1.895073	1.302125	1.258992	1.429209	1.843557	1.540753	1.605665	1.515634	1.626159	1.868007
Nlgn2	-0.48492	8.356123	-2.615988	0.019861	0.078048	-4.260598	8.189196	7.538416	8.191012	8.606724	7.733343	8.190504	8.617489	8.565963	8.681258	8.37599	8.357679	8.561646	8.602732	8.778736
Tulp4	-0.712229	6.434096	-2.615767	0.01987	0.078056	-4.282376	6.826788	6.054446	6.759826	7.258604	5.847478	6.657979	6.217169	6.449367	6.311229	5.918216	6.373213	6.343674	6.937887	6.140208
Stim2	-1.057485	7.012498	-2.615438	0.019883	0.078073	-4.281213	7.058857	4.790772	6.509193	7.503713	6.625239	6.960122	7.219959	7.361508	7.35597	7.182006	7.438845	7.321401	7.460937	7.40088
Dnase1l1	0.663428	3.973585	2.615328	0.019887	0.078073	-4.042536	3.765497	4.512597	3.978882	3.145235	4.20588	3.737479	4.017238	3.966821	3.985965	4.332655	4.095154	4.342647	3.495104	4.152141
Atg4c	-0.522138	5.528068	-2.614694	0.019912	0.078145	-4.260271	5.481971	4.838423	5.461342	6.196377	5.466539	5.502418	5.570406	5.557602	5.648188	5.392518	5.605045	5.474036	5.615248	5.61744
Klk8	-1.09973	3.510077	-2.613851	0.019945	0.078249	-3.966963	3.549707	1.151583	2.298852	3.727037	3.217567	3.462191	3.529702	4.061624	4.222559	4.145252	3.755344	3.921279	4.274664	3.998126
Arrdc4	0.803203	1.863895	2.613604	0.019955	0.078262	-3.645071	1.310059	2.21681	2.663852	1.571628	1.981199	2.04074	1.862293	1.958936	1.794428	1.986005	1.347792	2.300247	1.943396	1.556352
Id3	0.774243	5.163073	2.613048	0.019977	0.078322	-4.196553	5.150398	5.611879	5.217941	3.920151	5.494631	4.850213	5.064257	5.615391	4.787674	5.915425	5.30222	5.842338	4.611932	4.945683
Rpl39	1.262358	0.205624	2.612413	0.020002	0.078395	-3.278045	-0.634519	0.480615	0.945338	-0.600663	0.320024	0.386712	1.17791	0.76329	0.449965	0.534523	-0.149817	0.28321	0.546019	-0.204094
Bhlhe22	-1.719963	7.226429	-2.612097	0.020014	0.078418	-4.275569	7.67922	4.37216	6.704914	8.821091	6.981885	8.042319	7.30833	7.54908	7.243784	7.235017	7.435527	7.395451	7.453815	6.961722
Cttnbp2	-0.621217	8.406956	-2.611768	0.020027	0.078443	-4.268413	8.032648	8.105094	7.444769	8.852472	7.711776	8.008269	9.038663	8.705772	9.006529	8.722438	8.594549	8.327132	8.532844	8.619365
Sos1	-0.584387	6.464167	-2.610902	0.020061	0.078549	-4.291652	6.538414	6.018122	6.460296	7.320454	6.184399	6.71722	6.212087	6.602074	6.313411	6.234897	6.499315	6.3918	6.686917	6.337044
Elk3	0.48007	5.031781	2.610755	0.020067	0.078549	-4.243042	5.306139	5.144631	5.568581	4.90852	5.768538	5.840887	4.489964	5.08559	4.445789	4.856672	5.019414	5.183562	4.341792	4.534822
Gja1	-0.555587	9.108419	-2.610593	0.020073	0.078549	-4.232596	9.73413	9.078879	8.662927	9.456831	9.106182	9.311955	8.722625	9.213109	8.550511	9.03669	8.977911	9.521238	9.133638	9.014153
Ppm1j	1.224723	2.526323	2.610171	0.02009	0.078589	-3.741928	1.764345	3.859159	2.187895	1.780306	2.927714	3.069038	2.305626	2.436799	2.95129	3.302147	2.346454	2.475388	1.837926	2.417223
Il1r1	1.106599	2.516606	2.60919	0.020129	0.078661	-3.83876	2.8647	4.023929	3.431527	1.63746	2.424057	3.402651	2.165439	2.364261	2.583064	2.342482	1.719153	2.346065	1.963595	2.261799
Uvrag	-0.749307	3.570371	-2.609166	0.02013	0.078661	-4.040896	3.695089	2.607522	3.426105	3.967847	2.672087	3.601103	3.677127	4.12885	3.487872	3.52358	4.069701	3.854157	4.103184	3.310901
Bcorl1	-0.42011	5.226134	-2.609128	0.020132	0.078661	-4.255299	5.419656	4.848802	4.942168	5.617434	5.103448	5.503835	5.22677	5.31699	5.112276	5.157622	5.130266	5.288978	5.27077	5.269187
Tram1l1	-0.774832	5.149894	-2.609043	0.020135	0.078661	-4.2489	5.169999	3.914553	4.847991	5.964894	5.099602	5.303548	5.114144	5.05423	5.332613	5.238011	5.075905	5.216826	5.235902	5.576794
Gpm6a	-0.748395	10.01726	-2.607878	0.020181	0.078816	-4.206147	10.25767	8.962886	9.844063	10.78751	9.961903	10.33252	10.30601	9.67511	10.17998	9.902553	9.624075	9.80783	10.05316	10.54789
Tmem208	0.518007	4.675969	2.607407	0.0202	0.078864	-4.187655	4.569741	4.974974	4.919194	4.225001	5.120628	4.623856	4.595201	4.254576	4.646003	5.021485	4.63586	4.680333	4.463807	4.795073
LOC501110	-0.542033	4.281652	-2.606925	0.020219	0.078913	-4.194394	4.805223	4.749824	4.467397	4.748906	3.981865	3.76611	4.182238	4.145178	4.029076	4.250879	4.107714	4.249279	4.274664	4.266966
Acvr1	-0.45487	5.192773	-2.60504	0.020294	0.079181	-4.233997	4.965854	4.350411	4.738513	5.295932	4.755497	4.861313	5.251748	5.657738	5.675579	5.412451	5.311298	5.381725	5.458614	5.627027
Pcdhb9	-0.607907	3.496155	-2.603525	0.020355	0.079392	-4.031439	3.633671	2.822876	2.885483	3.68095	3.224646	3.282273	4.113042	3.645768	3.367065	3.924277	3.280721	3.56872	3.633916	4.027379
Efhd2	0.390496	7.729559	2.602754	0.020386	0.079457	-4.285907	8.052438	8.364651	8.44014	8.112592	8.275667	8.812231	7.467128	7.154615	7.230571	7.24116	7.39383	7.260893	7.226126	7.189614
Dnajc16	-0.533755	5.460054	-2.602688	0.020389	0.079457	-4.260208	5.268246	4.393585	5.194214	5.639022	4.901799	5.18132	5.588166	5.805453	5.837429	5.497024	5.63283	5.778327	5.979972	5.780954
Syt7	-0.522354	7.397945	-2.602619	0.020391	0.079457	-4.307577	7.213257	6.842933	7.129076	7.251614	6.130763	6.739049	7.84413	7.935643	7.817506	7.807278	7.646063	7.811059	7.776731	7.636259
Ahdc1	-0.370432	6.667482	-2.602282	0.020405	0.079468	-4.308424	6.659685	6.252124	6.576815	7.06585	6.524856	6.615997	6.583652	6.737464	6.746122	6.654847	6.78454	6.706982	6.825116	6.672554
Ehd3	-0.59401	6.707061	-2.602221	0.020407	0.079468	-4.308458	6.856663	5.847482	6.777498	7.251614	6.491494	6.720881	6.609971	6.915387	6.820092	6.625725	6.940703	6.690057	6.917322	6.449379
Nup107	0.360758	4.973069	2.599273	0.020526	0.079905	-4.230726	4.566458	5.208484	4.985204	4.847241	5.008197	5.08423	5.261619	4.921495	5.134654	4.965917	4.85532	4.778284	4.906128	5.150119
Pdcd6	0.388347	5.64417	2.598689	0.02055	0.079971	-4.291764	5.752845	5.83579	5.954858	5.419585	6.138281	5.957198	5.54436	5.476045	5.506658	5.453434	5.808521	5.312249	5.488545	5.401689
Tes	0.522609	4.305683	2.597717	0.020589	0.080098	-4.183246	4.227114	4.393585	4.608118	4.177845	4.919359	4.959619	3.889373	4.193082	4.086326	3.901891	4.265502	4.674359	3.98464	4.079517
Ubqln2	-0.727441	7.428355	-2.597304	0.020606	0.080118	-4.310017	7.566651	6.323629	7.201876	8.119328	7.333763	7.602055	7.442931	7.547545	7.563109	7.153864	7.605679	7.26388	7.835454	7.446714
Bdh1	-0.369274	5.015611	-2.597159	0.020612	0.080118	-4.256393	5.206312	5.080024	4.67593	5.179216	4.810643	4.74387	5.234309	5.107976	5.144489	5.159982	5.043585	4.936411	4.874473	5.070381
Pxdc1	0.47542	3.674525	2.597058	0.020616	0.080118	-4.070087	3.279917	3.774588	4.214137	3.633342	3.706477	4.028875	3.496648	3.550909	3.510866	3.702612	3.609886	3.885525	3.48116	3.692011
Itpr1	-0.520485	9.004875	-2.596932	0.020621	0.080118	-4.285084	8.985961	7.852306	8.173321	8.608948	8.374659	8.71798	9.69163	9.318853	9.418964	9.047565	9.363136	9.322129	9.676393	9.519752
Nmi	0.697088	3.064628	2.596577	0.020636	0.080148	-4.045553	3.100876	3.368632	3.784955	3.431645	4.195107	4.490087	2.48486	2.80888	2.430637	2.655119	2.627988	3.065063	2.133689	2.530531
Ubr1	-0.547919	5.808429	-2.596109	0.020655	0.080187	-4.303679	5.710342	5.474531	5.852467	6.409214	5.131969	5.593102	6.040665	5.87889	6.078382	5.436012	5.846486	5.693731	6.246324	5.954705
Usp32	-0.558803	7.27464	-2.595913	0.020663	0.080187	-4.314717	7.427459	6.908275	7.435692	7.955039	6.809561	7.374986	7.24754	7.285593	7.218981	6.895179	7.381384	7.06304	7.628841	7.223713
Ppapdc3	-0.421473	4.496506	-2.59582	0.020666	0.080187	-4.171427	4.495729	3.879549	3.830814	4.235668	4.312723	3.751866	4.767901	4.937208	4.893527	4.871149	4.703619	4.803137	4.752072	4.789392
Serp2	-0.402908	5.853795	-2.595574	0.020676	0.080187	-4.314992	6.55921	5.930445	6.022889	6.232328	6.206916	5.810283	5.594038	5.488896	5.886604	5.628953	5.778723	5.325652	5.762308	5.753498
RGD1561328	-0.428543	4.515296	-2.595509	0.020679	0.080187	-4.177692	4.569741	4.175923	3.772191	4.249769	4.191499	3.798811	4.707794	4.688835	4.824848	4.941431	4.786679	4.7949	4.797792	4.985976
Pafah1b3	0.767542	2.618756	2.595325	0.020687	0.080191	-3.86181	2.620642	3.117121	3.540923	1.996424	2.496038	3.014396	2.467906	2.539194	2.381582	2.600561	2.761158	2.2368	2.348615	2.802815
Cd37	0.929901	3.610218	2.595015	0.020699	0.080214	-3.909201	2.694686	3.08236	3.028308	2.297273	4.074901	3.444586	2.787802	5.22269	3.392049	5.233536	4.79964	5.243299	2.269694	3.133933
Lrp11	-0.477413	8.128954	-2.594749	0.02071	0.08023	-4.311711	7.902647	6.934093	7.689558	7.964689	7.701981	7.504888	8.461358	8.421788	8.517305	8.320105	8.461225	8.610765	8.677819	8.643182
Sema6d	-0.373377	7.183483	-2.594526	0.020719	0.080239	-4.322529	7.067592	7.00483	6.98302	7.489671	6.845597	6.786406	7.156565	7.340391	7.250061	7.356261	7.12601	7.50512	7.296492	7.371713
Eif4e3	-0.402306	4.55356	-2.594096	0.020737	0.080281	-4.234098	4.686244	4.414697	4.248334	5.066232	4.753051	4.507149	4.718584	4.424739	4.571407	4.369831	4.543347	4.461895	4.582846	4.468825
Ncor1	-0.401463	7.852511	-2.59352	0.02076	0.080347	-4.309845	7.854668	7.580671	7.797694	8.340923	7.507445	7.896705	7.937916	7.951978	7.828225	7.719998	7.772727	7.889868	8.021881	7.841316
Wdr62	1.42081	0.44449	2.592939	0.020784	0.080413	-3.395289	-0.410969	1.991799	0.484899	0.105385	1.113405	1.353343	0.282279	1.020936	-0.080923	0.413143	-0.411343	1.045835	0.434663	0.119416
Syngap1	-0.522858	9.335688	-2.592565	0.020799	0.080426	-4.263109	9.552844	8.690595	9.369285	9.806091	8.986338	9.58625	9.374958	9.474218	9.298738	9.19611	9.329015	9.319858	9.45317	9.264628
Map2k1	-0.743228	8.095321	-2.592528	0.020801	0.080426	-4.308926	8.099479	6.731669	7.758053	8.520302	7.797065	7.983845	8.168224	8.457095	8.36632	8.052779	8.576967	8.238299	8.567283	8.023244
Samd14	0.562812	5.350598	2.59146	0.020845	0.080569	-4.276181	5.32763	5.87567	5.774609	4.7588	5.636749	5.116089	5.67752	5.025118	5.253025	5.312036	5.060912	5.042261	5.683818	5.403549
Thbs1	1.896572	2.656452	2.590947	0.020866	0.080625	-3.792813	2.488111	2.36878	4.825515	1.033666	3.523394	3.47381	3.706333	2.665313	1.867508	2.854798	1.762161	2.187314	2.701169	2.030937
Rtn4rl2	-0.855549	5.635864	-2.589582	0.020922	0.080809	-4.292231	5.469722	3.735969	5.203752	5.958469	5.328778	5.138162	5.699474	5.980965	6.291447	5.856985	5.877378	6.025968	6.133842	6.237293
Slc20a1	0.461846	7.38459	2.58946	0.020927	0.080809	-4.32981	7.137845	7.747349	7.828332	7.119629	7.162317	7.62438	7.354607	7.255827	7.244927	7.556297	7.141872	7.498795	7.210892	7.510689
Fundc2	-0.420051	4.278114	-2.588879	0.020951	0.080861	-4.196277	4.432216	4.010125	3.750662	4.473536	4.405982	3.988947	4.602986	4.123366	4.453722	4.12123	4.352504	4.249279	4.449555	4.561731
LOC498368	-0.512536	3.540463	-2.588807	0.020954	0.080861	-4.088164	4.119218	3.1924	3.070623	3.450082	3.409963	3.384299	3.436917	3.960684	3.130231	3.734632	4.008504	4.123371	3.237505	3.447025
RT1-A3	1.336234	2.071298	2.588317	0.020974	0.080913	-3.69121	1.644914	2.397344	2.528721	1.033666	2.630048	2.949304	2.165439	2.603631	1.717448	2.291729	1.199219	3.42668	1.407665	1.415936
Srsf9	0.478924	4.639971	2.5881	0.020983	0.080922	-4.185441	4.069061	4.830589	4.326082	4.102146	4.499426	4.616013	5.089416	4.761243	4.852113	5.064951	4.630068	4.808602	4.559811	4.814782
Cpped1	0.559156	3.046852	2.587831	0.020994	0.080926	-4.025329	3.263791	4.023929	3.763618	2.890108	3.273255	3.450478	2.613764	2.68033	2.625819	2.945148	3.149191	2.960934	2.575555	2.635584
Gareml	-0.738732	4.02007	-2.58775	0.020997	0.080926	-4.167231	4.920286	4.204491	3.253175	3.795925	3.500244	3.53054	4.040359	4.009066	3.96946	4.126066	4.052479	4.350161	4.161181	4.468825
Dmrt3	-1.304147	0.096237	-2.587578	0.021005	0.080927	-3.408027	1.108109	-0.687936	-0.974047	0.280432	0.371962	-0.750296	0.357848	0.449407	0.245823	-0.210791	1.507975	0.514819	0.182287	0.554014
Dnajc28	-0.935176	2.702973	-2.587282	0.021017	0.080948	-3.990452	3.026365	1.453108	2.345554	3.815017	2.742832	3.438669	2.415815	2.650138	2.720913	2.469694	2.739799	2.738492	2.944167	2.59708
Etnk1	-0.487484	8.142147	-2.587101	0.021024	0.080951	-4.323366	7.683394	7.42854	7.312537	8.438986	7.682509	7.863139	8.610022	8.330141	8.563399	8.227502	8.4442	8.298286	8.607553	8.505736
Lgals3bp	0.973953	6.084807	2.586211	0.021061	0.081067	-4.320044	6.675771	6.368502	6.496962	4.588954	6.78941	6.710484	5.038651	6.740154	5.262088	6.661145	6.908163	6.932093	4.680656	5.362053
Tmem159	0.935227	3.095114	2.585354	0.021097	0.081178	-3.968943	3.197423	4.275453	3.530855	2.213416	3.827669	2.854719	2.774074	2.835955	2.834633	3.258764	3.03984	3.35379	2.535634	2.992761
Dnal4	-0.377249	5.693683	-2.585155	0.021105	0.081184	-4.318066	6.005266	5.246268	5.38794	5.641698	5.614014	5.529113	5.54436	5.81569	5.885139	5.93071	5.982275	5.839671	5.623207	5.696968
Myo1d	-0.542343	5.818929	-2.584614	0.021127	0.081221	-4.324424	6.19686	5.709075	5.253581	5.865378	5.80678	5.195423	5.501266	6.133337	5.634294	5.960802	5.905225	6.443651	5.949913	5.938113
Ctu2	0.868478	4.085404	2.584598	0.021128	0.081221	-4.06487	3.446334	4.604273	4.23289	2.985885	3.965054	3.49677	4.56363	4.388622	4.538218	4.556968	3.967746	4.273193	3.713602	4.561731
Galnt13	-0.82527	2.513536	-2.582843	0.021201	0.081476	-3.907848	2.357438	1.854335	2.298852	3.474303	1.705671	2.613455	2.324585	2.695192	2.430637	2.614395	2.468337	2.691938	2.944167	3.002614
Siae	-0.423802	4.313508	-2.582512	0.021215	0.0815	-4.216526	4.608566	4.037603	3.952724	4.440107	4.312723	4.101824	4.667523	4.182573	4.121609	4.303064	4.387261	4.412503	4.349488	4.591416
MGC105567	1.103391	1.144002	2.582371	0.021221	0.0815	-3.572538	0.692264	1.655851	2.487668	0.823477	2.014259	1.107027	0.625837	1.49863	0.385075	1.104165	1.557647	2.153355	0.491415	0.190106
Rsbn1l	-0.453395	4.316844	-2.581046	0.021276	0.081686	-4.227418	4.421353	3.919485	4.248334	4.785664	3.96082	4.478599	4.301868	4.477275	4.389005	4.307328	4.265502	4.331301	4.512607	4.116286
Gm2a	0.457756	7.49137	2.580509	0.021299	0.081746	-4.339448	7.776907	8.009746	8.062801	7.34857	8.180572	7.832162	6.705276	7.650742	6.817789	7.503843	7.18893	7.675187	6.979399	7.156346
Epdr1	-0.53797	6.579552	-2.580344	0.021306	0.081747	-4.347207	6.603931	5.607302	6.237747	6.773047	6.569331	6.186323	6.537677	6.609939	6.848199	6.645229	6.684683	6.805934	7.012497	7.008928
Aurkb	3.360179	1.627038	2.579999	0.02132	0.081776	-3.340803	-0.310971	1.934482	0.914692	-1.388744	3.523394	2.772436	1.913281	2.436799	1.843557	0.591587	1.199219	3.440827	2.466543	2.310209
Slc30a3	-0.873348	7.850343	-2.579571	0.021338	0.081818	-4.344135	7.821313	5.82663	7.449852	7.816843	7.33785	7.17465	8.029592	8.340518	8.570256	8.525789	8.086344	8.339926	8.127765	8.46756
Frzb	-1.235565	5.217382	-2.579292	0.02135	0.081818	-4.273669	5.017259	2.439156	4.399853	5.808165	4.879544	4.932509	5.256692	5.89668	5.889532	5.932091	5.543201	6.057295	5.554953	5.493644
Ppib	0.437997	7.122717	2.579253	0.021351	0.081818	-4.3511	6.974055	7.251625	7.021281	6.516619	7.48219	6.973995	7.012008	7.135673	7.03361	7.464763	7.215285	7.353536	7.027566	7.267279
Samd4b	0.290398	6.667653	2.578946	0.021364	0.081841	-4.352242	6.845194	7.03184	7.175055	6.680436	6.994365	7.010849	6.396981	6.457017	6.313411	6.447858	6.472858	6.588879	6.456901	6.491098
Otub2	-0.751847	4.154218	-2.577934	0.021407	0.081978	-4.159882	4.218773	2.439156	3.580509	4.181527	3.973484	3.737479	4.43432	4.666384	4.372361	4.449167	4.72823	4.292824	4.689778	4.493492
Cd48	0.729482	5.463476	2.576616	0.021463	0.082138	-4.315757	5.453818	5.721836	5.799984	4.945809	6.352984	5.819419	4.238302	6.11963	4.930947	5.752952	5.235114	6.134393	5.176569	4.845219
Scn3b	-0.823072	9.946	-2.57658	0.021464	0.082138	-4.288875	9.737145	8.042611	9.10322	10.04561	9.423616	9.714285	10.31085	10.54658	10.42435	10.29929	10.42706	10.32601	10.48786	10.35736
Ndufs1	-0.506726	7.325456	-2.576455	0.021469	0.082138	-4.349525	7.57933	6.859067	7.131161	7.966825	7.477752	7.596087	7.200847	7.181252	7.310827	6.967699	7.357919	7.121294	7.547958	7.2683
Tspan5	-0.295655	7.609216	-2.575288	0.021519	0.082301	-4.354438	7.549709	7.101849	7.252918	7.58764	7.356501	7.38194	7.737202	7.819206	7.710258	7.883163	7.723252	7.833013	7.84297	7.7576
Nxph1	-0.583407	5.007363	-2.574586	0.021548	0.082388	-4.277986	4.816322	3.939046	4.43537	5.245807	4.817684	4.600198	5.143803	5.196815	5.339051	4.965917	5.084402	5.127817	5.759422	5.683248
Cspg5	-0.40415	7.63554	-2.57435	0.021558	0.082401	-4.351419	7.877036	7.59654	7.16329	7.815955	7.478863	7.529104	7.480397	7.575928	7.480101	7.806525	7.622917	7.994572	7.73364	7.750673
Nefl	-1.165637	9.038894	-2.57201	0.021658	0.082679	-4.317347	9.157917	6.749088	8.558078	9.692763	8.836839	8.906873	9.437799	9.349122	9.516626	8.922827	9.466609	9.101824	9.702262	9.149469
Nppc	-1.082971	2.142391	-2.571948	0.02166	0.082679	-3.715949	2.144973	-0.273517	1.061799	1.945353	1.840817	1.249022	2.703406	2.665313	2.508863	2.529327	2.892776	2.921125	3.3415	2.932182
Gbas	-0.468749	4.99518	-2.571914	0.021662	0.082679	-4.319942	5.397656	4.790772	4.913277	5.514617	4.8386	5.408679	4.952909	4.9924	4.755947	4.836158	4.997084	4.81947	5.176569	4.588148
Sult4a1	-0.378327	8.253077	-2.571805	0.021667	0.082679	-4.343194	8.423624	7.974635	8.039243	8.346879	8.051899	7.963901	8.333821	8.165472	8.510191	8.304456	8.569609	8.099174	8.573047	8.192331
Dnaaf3	1.125096	2.823864	2.571798	0.021667	0.082679	-3.900787	2.977763	3.769134	3.674966	1.454757	2.970456	2.734303	2.760215	3.575215	2.917273	2.325762	2.792614	2.502617	2.816716	2.503027

Cyb5d2	-0.646706	3.058436	-2.571471	0.021681	0.082679	-4.011563	3.109925	1.894965	2.691232	3.41297	2.978854	2.889836	3.137884	3.386664	3.416608	3.040455	3.401255	3.212521	3.372046	3.069767
Map3k5	-0.511025	4.621425	-2.571437	0.021682	0.082679	-4.255519	4.762823	4.364946	4.058135	4.667211	4.051203	4.358391	5.009657	4.81326	4.723506	4.421893	4.837836	4.686283	5.364902	4.645875
Cers5	0.35533	6.26782	2.571281	0.021689	0.082679	-4.358241	6.173313	6.558324	6.474812	5.915994	6.416191	6.14695	6.199304	6.232184	6.239624	6.304818	6.251153	6.324728	6.111386	6.421186
Add1	-0.382396	9.026494	-2.571177	0.021693	0.082679	-4.312139	9.302691	8.719691	8.92441	9.470348	9.136644	9.236379	8.883099	8.92059	8.930405	8.840619	9.023919	8.962914	9.107637	8.914594
Dapk1	-0.496247	6.825931	-2.571011	0.0217	0.08268	-4.36669	6.507933	6.122324	6.556487	7.221075	6.168883	6.625794	7.254354	7.113024	7.052016	7.105705	6.934841	6.973431	7.022761	6.918786
Slc16a7	-0.95824	2.948088	-2.570348	0.021729	0.082762	-3.990314	2.670424	1.279848	2.568639	3.870824	2.460496	2.99033	3.159163	3.159031	3.225976	2.945148	3.20481	3.220743	3.582598	3.160583
Scube1	0.701156	4.321756	2.568864	0.021792	0.08296	-4.198868	4.128153	4.438943	5.08147	3.633342	4.227186	4.507149	4.243293	4.58094	4.263751	3.715505	4.172914	4.458423	4.405935	4.728352
Wdr74	0.338137	4.77992	2.568807	0.021795	0.08296	-4.291195	4.830077	5.035287	5.27032	4.669835	5.089943	4.940905	4.689628	4.662608	4.765539	4.716194	4.655954	4.539538	4.58936	4.521176
Pole2	1.651047	0.74434	2.567174	0.021865	0.083201	-3.542289	0.176305	2.324846	0.604933	0.531921	2.640673	2.410784	0.952703	0.222004	0.245823	0.699359	0.535373	0.403657	-0.512812	0.257494
Cplx1	-0.491819	9.250618	-2.56676	0.021882	0.083242	-4.30552	9.843115	9.231929	9.517578	9.826595	9.526472	9.099764	8.948727	8.914451	9.252449	8.796551	9.315656	8.855634	9.439827	8.942537
Ap3m2	-0.519473	5.445224	-2.5666	0.021889	0.083242	-4.349067	5.629274	4.91203	5.416931	5.979775	5.320542	5.488167	5.256692	5.522617	5.43233	5.089921	5.590204	5.408965	5.867892	5.354378
Ndrg3	-0.50207	8.607972	-2.565878	0.02192	0.083334	-4.346792	8.550554	7.690419	8.13333	8.813694	8.368879	8.529219	9.098234	8.772547	8.864616	8.706986	8.834153	8.622642	8.829633	8.700872
Mfng	0.793687	3.290686	2.564461	0.021982	0.083539	-3.974286	3.09177	3.785434	3.184186	2.045748	3.551819	2.836835	3.270902	3.575215	2.995434	3.844356	3.678847	4.069761	3.203938	3.106782
Tbcel	-0.63255	3.195231	-2.563633	0.022017	0.083649	-4.05003	3.683012	2.631765	3.12518	3.083591	2.386673	2.791131	3.445603	3.449372	3.271566	2.998871	3.656224	3.454835	3.671242	3.262515
Kifap3	-0.59761	8.091437	-2.563126	0.022039	0.083706	-4.35916	8.161176	7.282579	7.94666	8.757284	8.020971	8.194899	8.040742	8.080645	8.156946	7.846635	8.286042	7.966734	8.433157	8.111544
Mboat2	-0.338583	5.875125	-2.561536	0.022108	0.083926	-4.367935	5.821932	5.562287	5.471906	6.062986	5.547816	5.839765	6.036359	6.030149	6.034097	5.93485	5.816195	5.945159	5.992314	6.183083
Ethe1	0.509997	3.760116	2.561471	0.022111	0.083926	-4.15996	3.896775	4.252185	4.02265	3.275	4.282511	3.775529	3.734959	3.622632	3.805565	3.820684	3.461211	3.625558	3.522595	3.661237
Cyp4f17	-0.835345	2.443435	-2.56123	0.022122	0.083939	-3.786935	2.445855	1.812527	0.785152	1.874319	1.78444	1.046069	3.337752	2.849305	3.188437	3.258764	3.065821	2.980434	2.783013	3.35002
Spidr	0.656343	2.356476	2.560336	0.02216	0.08406	-3.865461	1.80948	2.324846	2.877559	2.269859	2.484288	3.061358	2.206902	2.364261	2.066858	2.342482	2.133674	2.461578	2.393975	2.503027
Sv2b	-0.628779	8.915624	-2.559703	0.022188	0.084138	-4.350214	8.747867	7.636776	8.622164	9.20807	8.671172	8.470806	9.16747	8.926507	9.40484	8.777656	9.086067	9.022917	9.572644	9.507261
Stxbp1	-0.70406	9.890173	-2.558387	0.022245	0.084329	-4.300125	10.10091	9.143405	9.868567	10.855	9.779231	10.30291	9.660957	9.909723	9.802714	9.644018	9.996667	9.690632	10.11189	9.597495
Rpl26	1.038021	0.420848	2.558067	0.022259	0.084355	-3.391244	-0.899176	0.812964	0.158563	-0.02435	0.517419	0.61372	1.21902	0.137473	0.884903	0.799637	0.535373	0.618025	1.001683	0.753927
Rspo1	-0.704725	3.208738	-2.557738	0.022274	0.084383	-4.135354	3.32724	3.492948	2.654608	4.239206	3.482634	2.845805	3.211029	3.282262	3.548392	3.060806	2.782205	2.931181	3.169571	3.069767
Nup37	0.759189	3.275552	2.557009	0.022306	0.084477	-4.008478	2.753611	4.005493	3.47943	2.591079	3.26641	2.974059	3.616879	3.575215	3.555781	3.649853	3.141068	3.195935	2.933966	3.286911
Shank2	-0.738653	4.275314	-2.556225	0.02234	0.084581	-4.28331	4.993008	3.929299	4.432669	4.632654	3.452798	4.449473	4.171806	4.446862	4.254698	3.90752	4.272956	4.212646	4.934548	3.847716
Aldh9a1	-0.53641	4.647131	-2.556035	0.022348	0.084586	-4.339006	5.362121	4.357697	4.682767	5.304401	4.762811	5.373446	4.091065	4.60868	4.222559	4.4607	4.411103	4.496167	4.602301	4.388433
Spn	1.23348	2.257794	2.555601	0.022367	0.084631	-3.79876	1.071521	1.769471	2.853524	2.077719	2.919011	3.622139	1.962529	2.16515	2.653634	2.256873	2.066633	2.668085	1.943396	1.931282
Nrsn1	-0.497672	6.535017	-2.555278	0.022382	0.084655	-4.395591	7.417927	7.174302	6.933244	7.285803	6.519109	6.792798	6.094126	5.761998	6.390855	5.963506	6.095906	6.051545	6.780267	6.246652
Gramd1b	-0.41302	6.671245	-2.555141	0.022388	0.084655	-4.393369	6.466262	6.15719	6.440936	6.64019	5.891515	6.072488	6.957633	7.161989	6.938591	6.835157	6.963916	6.990962	7.16747	6.729289
Dact3	-0.400163	8.090496	-2.553279	0.02247	0.084933	-4.385219	8.328955	7.635652	7.789272	7.873151	7.609004	7.776665	8.508236	8.030986	8.309534	8.390133	8.024208	8.295613	8.223269	8.478193
Usp30	-0.295151	6.407928	-2.553145	0.022476	0.084933	-4.391897	6.163568	6.016972	5.986514	6.263935	5.88706	5.787187	6.596384	6.689098	6.692659	6.727888	6.624773	6.762948	6.706526	6.824718
Cd40	1.066446	0.834429	2.552714	0.022495	0.084979	-3.534093	0.428583	1.506527	1.874203	0.166125	0.985877	0.846031	0.429655	1.112357	0.837186	1.51148	1.135223	1.593679	-0.305233	0.499386
Napb	-1.187482	6.092125	-2.552151	0.022519	0.085046	-4.395529	6.34887	4.47624	6.316051	7.284519	5.419496	6.281123	6.026261	6.376322	6.244205	5.416404	6.580404	5.769947	6.853131	5.922627
Gabrb3	-1.236837	6.714711	-2.551752	0.022537	0.085086	-4.399673	6.747105	4.788078	6.745204	7.923499	5.976799	6.865716	6.264573	7.100518	7.037574	6.450752	6.98846	6.940829	7.418123	6.775998
MGC95210	0.454203	3.533423	2.551332	0.022556	0.085129	-4.083397	3.205888	3.86939	3.420663	2.943136	3.458815	3.359461	3.749062	3.749088	3.548392	3.721909	3.695584	3.778199	3.502026	3.604134
Senp7	-0.421449	4.548178	-2.550758	0.022581	0.085162	-4.323387	4.698293	4.309667	4.631863	5.244047	4.454966	4.814106	4.51494	4.370218	4.488889	4.320048	4.50904	4.482556	4.477919	4.425531
Specc1	-0.652032	5.708199	-2.55074	0.022582	0.085162	-4.391553	5.894422	4.936565	5.57472	6.507112	5.782998	5.895902	5.556439	5.95032	5.533105	5.312036	5.786556	5.654816	6.002112	5.558541
Tmprss9	1.189372	1.148492	2.550558	0.02259	0.085162	-3.584362	0.873216	1.558038	2.498042	0.166125	1.338932	1.107027	1.697535	0.705741	1.218642	1.14286	1.260496	0.760186	0.836265	1.659607
Dcdc2	-1.042863	1.875217	-2.55037	0.022598	0.085162	-3.928266	3.311637	0.973336	2.010869	2.389316	1.285769	2.845805	1.511505	2.030999	1.743567	0.938085	1.978199	1.848377	1.815874	2.030937
Abhd6	-0.532089	6.806342	-2.550127	0.022609	0.085162	-4.405675	6.638736	6.061157	5.951088	7.10419	6.567244	6.780571	6.97948	7.028264	7.091319	7.096493	6.994109	6.955068	6.851099	7.204632
Psmd10	-0.735388	2.090716	-2.54995	0.022617	0.085162	-3.893964	2.372556	1.279848	1.554575	2.677071	2.226624	2.160607	2.286414	1.80302	2.462438	2.274406	2.243983	1.738143	2.639729	1.931282
Kmo	1.703193	0.836965	2.549871	0.02262	0.085162	-3.39338	-0.518419	1.702379	0.158563	-0.81875	1.143601	0.734546	1.408645	1.775313	0.628769	2.758411	1.803924	2.082939	-0.211665	0.190106
Erc1	-1.18068	5.465199	-2.549857	0.022621	0.085162	-4.389912	5.960945	4.142566	5.582052	6.9153	5.165466	6.114845	5.127701	5.722645	5.084445	4.653926	5.588712	5.365131	6.185278	4.943127
RGD1311648	-0.393719	5.484573	-2.54896	0.022661	0.085251	-4.382263	5.671142	5.071186	5.267291	5.838805	5.618052	5.489598	5.497094	5.456551	5.38748	5.416404	5.68405	5.379891	5.621619	5.420187
Rabggtb	0.346319	6.265864	2.548745	0.02267	0.085251	-4.401846	6.092305	6.530388	6.466912	6.134076	6.560965	6.279469	6.410354	6.009488	6.361639	6.291963	6.187498	5.97827	6.095456	6.343842
Tnfrsf21	-0.8288	7.708878	-2.548703	0.022672	0.085251	-4.40044	7.555102	6.02042	7.329336	8.24783	7.357709	7.581729	7.887408	8.01996	8.002604	7.792142	7.869015	8.069038	8.123283	8.076986
Irs2	-0.56143	6.444152	-2.548688	0.022673	0.085251	-4.407495	6.503648	5.738676	6.493726	7.071815	6.33265	6.339443	6.424702	6.337772	6.471207	6.128094	6.655752	6.493703	6.827873	6.417508
Parp2	0.469457	4.686679	2.54838	0.022687	0.085276	-4.294024	4.526467	4.941423	4.596097	4.214255	4.964461	4.843511	4.851979	4.823442	4.686958	4.919311	4.573843	4.526331	4.345646	4.861554
Prmt7	0.291772	5.144059	2.548154	0.022697	0.085285	-4.344974	4.975787	5.244304	5.286867	4.906296	5.280338	5.114234	5.261619	5.082767	5.22317	5.114467	5.176705	4.975806	5.233825	5.185153
Smg7	-0.399141	7.263785	-2.548005	0.022703	0.085285	-4.406293	7.324816	6.925538	7.41907	7.781184	7.043736	7.226221	7.178225	7.352161	7.228838	7.169176	7.298899	7.171996	7.373999	7.209426
Adam17	0.431847	5.380774	2.547517	0.022725	0.085315	-4.369714	5.31007	5.6962	5.452035	5.175528	5.723019	5.82283	5.159727	5.366574	4.967421	5.434063	5.101249	5.762926	5.107901	5.289453
Pdxk	-0.517322	3.316844	-2.547509	0.022726	0.085315	-4.152516	3.799454	3.099845	3.228468	3.627954	3.044338	3.402651	3.026505	3.211662	3.298245	3.214035	3.586147	3.376046	3.73726	2.942457
Rap1a	0.454712	5.750788	2.546848	0.022755	0.085399	-4.399754	5.690875	5.94382	6.222188	6.034761	6.684205	6.421978	5.377362	5.478195	5.330461	5.368229	5.510468	5.743085	5.402381	5.333059
Pmm2	0.37293	5.858579	2.546508	0.02277	0.085403	-4.393452	5.78286	6.297347	5.955799	5.573263	6.127934	5.819419	5.852639	5.76904	5.911298	5.905614	5.872479	5.841005	5.576426	5.762225
Hey1	-0.527768	3.996693	-2.546395	0.022775	0.085403	-4.261499	4.231267	3.361401	4.275724	4.552446	3.939464	3.857641	3.734959	4.213873	3.91304	3.814705	4.116027	4.018805	4.11226	3.911867
Plcl2	-0.829628	5.718181	-2.546347	0.022777	0.085403	-4.399091	5.874648	4.57956	5.372519	6.694681	5.607258	6.251898	5.797727	5.733485	5.649915	5.606633	5.955889	5.831641	5.730244	5.399826
Dstyk	-0.376725	6.664594	-2.545979	0.022794	0.085438	-4.412642	6.484641	6.512546	6.350807	6.926864	6.083843	6.373181	6.976486	6.742839	6.742079	6.73977	6.791044	6.90811	6.879949	6.807958
Fbxo11	-0.417872	6.907779	-2.545286	0.022825	0.085527	-4.414795	6.971575	6.579511	6.923657	7.606254	6.972453	7.005856	6.96218	6.723939	6.913646	6.653628	6.853902	6.748092	6.894489	6.91292
Klhl7	-0.539216	6.184985	-2.543397	0.02291	0.085818	-4.416261	6.389381	5.713341	5.885437	6.964223	6.444513	6.509955	6.239818	5.849298	6.134978	5.862791	6.089588	5.998734	6.277922	6.251825
B3galt1	-0.862578	4.402064	-2.542449	0.022952	0.085923	-4.304517	4.428604	3.125682	4.299641	5.233442	3.846148	4.581525	4.263083	4.785751	4.60741	4.015725	4.714609	4.458423	4.944745	4.403387
Asic2	-0.678271	6.266105	-2.542267	0.02296	0.085923	-4.413119	6.396794	5.131939	5.471906	6.47746	6.377806	6.051319	6.711592	6.435051	6.442594	6.706251	6.18157	6.099149	6.639052	6.624706
Il34	-0.381844	5.707323	-2.541919	0.022976	0.085923	-4.391904	5.865327	5.246268	5.225764	5.501419	5.475469	5.253872	5.796029	5.709895	6.039378	5.842364	5.991341	5.943917	6.176652	5.865727
Kif2a	-0.412226	6.376485	-2.541917	0.022976	0.085923	-4.418353	6.281389	6.030712	6.125977	6.861793	6.107015	6.377827	6.656541	6.318704	6.418477	6.194024	6.430993	6.375322	6.680829	6.430341
Cxxc5	-0.558451	5.829003	-2.541831	0.02298	0.085923	-4.416293	6.200041	5.397089	5.853477	6.78943	6.196152	6.299194	5.290835	5.659633	5.405997	5.682467	5.862632	5.818157	5.597584	5.581733
Cd101	0.94949	1.12371	2.54181	0.022981	0.085923	-3.63271	0.873216	1.874793	2.067688	0.577356	1.390206	1.193889	0.202532	1.49863	0.571595	1.14286	1.456531	2.046399	1.040235	0.554014
Lrp6	-0.547332	5.247954	-2.541664	0.022988	0.085923	-4.390033	5.509586	4.941423	5.450701	5.909347	4.7917	5.463612	5.146469	5.438999	5.192685	4.850841	4.958312	5.237233	5.490287	5.132278
Hr	-0.612187	5.627175	-2.541097	0.023013	0.085992	-4.376734	5.671142	5.39532	4.432669	5.326191	5.158089	4.586885	6.057763	6.10857	5.874836	6.188254	5.877378	6.383585	5.666979	6.087944
Pdcd7	-0.365822	5.315667	-2.540733	0.02303	0.086026	-4.388391	5.369676	5.112689	5.098612	5.780507	5.242473	5.396521	5.317106	5.260653	5.15669	5.301385	5.521462	5.259352	5.324473	5.317354
Slc24a1	1.270265	-0.029609	2.539875	0.023068	0.086145	-3.48448	-0.129637	1.453108	0.715742	-0.166914	0.266146	1.821837	-0.399109	-0.511025	0.007959	-0.420466	-0.616402	-0.541385	0.434663	-0.621773
Fam151a	-0.923191	2.16272	-2.539611	0.02308	0.086162	-3.885316	2.967843	2.151237	0.851376	1.9796	2.030509	1.275818	2.659281	1.80302	2.639794	2.514649	2.243983	2.619163	2.408783	2.516844
Asrgl1	-0.432939	6.776317	-2.539243	0.023097	0.086198	-4.426642	7.448121	6.880723	6.411057	6.990178	6.963488	6.893016	6.737465	6.460284	6.645821	6.776379	6.48815	6.716462	6.770984	6.700822
Pdhb	-0.39467	7.029742	-2.538744	0.02312	0.086256	-4.42699	7.11836	6.560694	6.907211	7.462685	7.121652	6.992792	7.121083	6.915387	7.151084	6.958237	7.101101	6.797026	7.164201	7.057004
Glg1	-0.650592	7.037237	-2.537595	0.023172	0.086423	-4.429438	6.752171	5.905805	6.745749	7.42318	6.25179	6.841256	7.205329	7.429744	7.316276	6.945981	7.228763	7.424216	7.701677	7.362178
Col16a1	1.090681	7.724738	2.537302	0.023185	0.086423	-4.425619	6.767264	8.870085	7.012263	6.502703	7.810655	7.19058	7.979631	8.285724	8.074139	8.660862	7.603833	8.34769	7.056066	7.993288
Dgke	-0.590468	5.418555	-2.537196	0.02319	0.086423	-4.390745	5.195727	4.843622	5.438634	5.995551	4.567829	5.179547	5.658967	5.613437	5.812969	5.268953	5.439377	5.275228	5.867892	5.740308
Ddit4l	-0.789603	1.529009	-2.537103	0.023194	0.086423	-3.742714	1.594228	0.769921	1.143363	1.874319	0.691028	1.275818	1.862293	2.007376	1.937064	2.308845	2.166061	2.046399	1.465469	0.844371
Psenen	-0.400445	6.175214	-2.536963	0.023201	0.086423	-4.426341	6.57808	5.817411	5.894299	6.38626	6.335922	6.278641	5.979177	5.996048	6.196547	6.274643	6.253039	6.17092	6.154857	6.159054
Tbck	-0.503578	4.733728	-2.535225	0.02328	0.086691	-4.352322	4.816322	4.200444	4.195136	5.192053	4.803569	4.820946	4.822272	4.803005	4.720222	4.556968	4.908931	4.709839	5.019009	4.763548
Gadd45b	0.751481	3.907106	2.534552	0.02331	0.086779	-4.247088	3.460552	4.220564	5.20058	4.062753	4.27571	4.355253	3.383673	3.550909	3.592171	3.867646	3.448101	4.269235	3.794763	3.326676
Gnai3	0.446383	5.85	2.533896	0.02334	0.086863	-4.428584	5.996754	6.124461	6.521322	6.099456	6.497352	6.832258	5.377362	5.484625	5.24619	5.351806	5.51676	5.764333	5.634275	5.481365
Macrod1	-0.535156	3.685454	-2.5336	0.023354	0.086884	-4.195077	4.150251	3.52566	3.289459	3.480296	3.306999	2.982217	3.647317	3.891392	3.991425	4.010503	3.633241	3.895831	3.833693	4.084165
MGC112830	0.312412	6.094712	2.533461	0.02336	0.086884	-4.423858	5.952167	6.208464	6.058368	5.764636	6.260428	6.152383	6.316401	6.006512	6.202444	6.131661	6.002593	5.952583	6.035891	6.304554
Rundc3b	-0.56705	4.974592	-2.532272	0.023415	0.087059	-4.382891	5.014852	4.313419	5.069347	5.661611	4.638447	5.063233	5.003787	4.90241	5.002995	4.833203	5.242726	4.7949	5.231744	4.922513
Cdkn1a	1.092222	3.928178	2.531872	0.023433	0.08707	-4.308652	4.55325	5.724656	5.990193	3.686144	4.789315	4.271191	2.501617	3.501034	2.639794	3.214035	4.265502	3.816678	3.3415	2.825198
Rims3	-0.950454	2.282707	-2.531797	0.023437	0.08707	-4.044716	3.474632	1.506527	2.869592	2.925676	2.240661	2.271274	2.033384	2.121805	1.255413	1.755735	2.651054	2.419337	3.02328	1.755962
RGD1563888	-0.603361	2.70127	-2.531739	0.023439	0.08707	-4.067514	2.718545	2.724859	2.588191	3.567317	2.752661	2.217002	2.893159	2.505864	2.870631	2.469694	2.213317	2.794648	2.933966	2.814049
Nle1	0.522664	3.818606	2.529887	0.023525	0.087359	-4.184622	3.608352	4.134105	4.072089	3.16769	3.794748	3.595796	4.187425	3.683523	4.146293	3.87341	3.771221	3.805789	3.67737	4.056051
Ddhd1	-0.498445	6.557612	-2.529494	0.023543	0.087385	-4.441353	6.279385	5.894578	5.810426	6.910869	6.32031	6.366964	6.974987	6.777294	6.802341	6.965002	6.702733	6.718641	6.583031	6.717371
Ypel2	-0.683048	4.491172	-2.529414	0.023546	0.087385	-4.345776	4.566458	3.86939	4.287732	5.235215	4.082714	4.644565	4.166561	4.796129	4.37654	4.150009	4.50588	4.671363	5.098798	4.496982
Rce1	0.504135	4.976701	2.529261	0.023553	0.087385	-4.350451	4.686244	5.474531	4.790075	4.427757	5.114924	4.86573	5.038651	4.930944	5.171196	5.277674	4.967529	5.096883	4.725704	5.156753
Bnip3l	-0.507275	7.075037	-2.528706	0.023579	0.087453	-4.441423	7.44052	6.731669	6.979786	7.820981	7.322671	7.458129	7.065112	6.674173	6.917236	6.671107	7.13012	6.806617	7.166381	6.877882
Gnl3l	-0.71438	5.690396	-2.528307	0.023597	0.087495	-4.435232	5.865327	5.051103	5.846391	6.730945	5.482869	5.949937	5.44173	5.777793	5.66878	5.138601	5.737561	5.401751	6.093166	5.511007
Neto2	-0.766931	4.310215	-2.52809	0.023608	0.087505	-4.336073	4.667979	3.607396	3.733206	4.980046	4.051203	4.847983	3.975863	4.299035	4.227195	3.940842	4.073974	4.584831	4.911337	4.5246
Panx2	-0.470354	8.078109	-2.527371	0.023641	0.087601	-4.42796	8.461675	7.985259	7.588215	8.23504	8.246183	7.692155	8.214063	7.822594	8.116605	8.192193	7.865937	8.2032	8.081726	8.394599
Efs	0.491133	5.148846	2.526917	0.023662	0.087652	-4.400639	5.513001	5.616442	5.813544	4.713732	5.600471	5.353145	4.781687	4.843593	4.833994	5.06998	5.071637	5.149513	4.716806	5.051519
B4galt2	-0.35619	6.322975	-2.525624	0.023722	0.087832	-4.446766	6.649636	5.948654	6.23232	6.194556	6.016093	6.062905	6.380089	6.313897	6.440599	6.572681	6.311212	6.456763	6.46578	6.496338
Aplp2	-0.399799	9.690553	-2.525546	0.023725	0.087832	-4.385905	9.688141	8.964079	9.3474	9.731746	9.494704	9.439268	9.824203	9.77095	10.00351	9.788013	9.717008	9.886407	9.830413	10.18385
Nudcd2	0.412196	3.24621	2.525397	0.023732	0.087832	-4.17584	3.481621	3.655486	3.595078	3.160244	3.794748	3.848745	3.083269	2.926907	2.822431	3.030171	3.132898	2.951084	3.220819	2.91141
Aimp1	0.387988	5.077018	2.525155	0.023744	0.087846	-4.40159	5.026846	5.11484	5.646417	5.177373	5.679885	5.510901	4.760958	4.889545	4.992142	4.847916	4.857801	4.824874	4.969927	4.825924
Olfm2	0.741146	6.328773	2.525006	0.023751	0.087846	-4.450692	6.720028	8.051062	7.46903	6.387855	6.787021	6.866267	5.57635	5.432361	6.084781	5.986294	5.733515	5.681868	5.682296	6.165647
Ppia	-0.622123	6.05055	-2.5232	0.023835	0.088127	-4.451781	6.69469	5.774504	6.069714	6.447951	5.385155	6.565308	6.315216	5.951868	6.143586	5.89715	5.749633	5.863497	6.172319	5.701512
Pdk3	-0.407782	5.198221	-2.523025	0.023843	0.088127	-4.408534	5.428725	4.88707	5.09178	5.359083	5.278638	4.685111	5.2443	5.096827	5.304377	5.262379	5.43606	5.208581	5.428048	5.10738
Rassf5	0.395705	4.739393	2.522903	0.023849	0.088127	-4.401514	5.055231	5.640537	5.540002	5.023782	5.416407	5.143628	4.473071	4.026799	4.227195	4.417954	4.414477	4.451452	4.216936	4.365708
Man1a1	0.484243	5.330222	2.522343	0.023875	0.088196	-4.377553	4.878536	4.833205	5.071085	4.546747	5.273527	5.581066	5.428646	5.632865	5.616738	5.41047	5.526148	5.814088	5.462168	5.588291
Spag9	-0.41824	8.718387	-2.522157	0.023883	0.088201	-4.414538	8.862229	8.772266	8.712626	9.310317	8.302053	8.878198	8.835771	8.51085	8.638834	8.492935	8.494882	8.628254	8.845026	8.776942
Bbs7	0.42471	5.113887	2.521157	0.02393	0.088347	-4.401861	5.007607	5.755323	5.479778	4.97581	5.198203	5.23522	5.363679	4.866752	5.142036	5.011064	4.812484	4.851593	4.879797	5.060981
Setd4	0.759313	4.289868	2.520768	0.023949	0.088387	-4.240417	3.854329	4.853963	4.334789	3.268089	4.265449	3.789543	4.906476	4.455617	4.628589	4.613168	4.242906	4.216763	4.033233	4.680048
Kcnmb2	-2.227567	0.215793	-2.518951	0.024034	0.088645	-3.461422	0.243629	-3.260591	-0.130036	1.188094	0.15195	-0.862697	1.21902	0.377517	0.512061	1.104165	1.102128	1.252437	1.700256	1.501815
Lppr2	-0.544475	9.462939	-2.518848	0.024039	0.088645	-4.409681	9.468184	8.256744	9.243148	9.41384	9.157257	8.942718	9.622812	9.793462	9.880176	9.790015	9.600663	9.723008	9.679163	9.912316
Pls3	-0.601058	6.557276	-2.518545	0.024053	0.088645	-4.463528	6.626964	5.509296	6.061869	6.956183	6.688052	6.502175	6.731262	6.631348	6.82698	6.439139	6.461486	6.599953	6.9398	6.844714
Rnf219	-0.445466	4.035211	-2.518514	0.024054	0.088645	-4.302611	4.145858	3.557647	3.805981	4.409031	3.952316	4.020978	4.187425	3.904241	4.096495	3.867646	4.26176	3.980582	4.43154	3.968267
LOC606294	-0.668141	7.476186	-2.5185	0.024055	0.088645	-4.459687	7.499379	6.242317	7.161252	7.909966	7.47107	7.271027	7.505537	7.704067	7.779746	7.434865	7.814509	7.46372	7.863943	7.554469
Ptgfrn	0.388273	5.374567	2.518111	0.024073	0.088685	-4.448996	5.824683	6.078899	6.29017	5.662929	5.971549	6.181017	4.861747	4.685117	4.700356	4.910928	5.032648	5.259352	4.777968	5.046765
Htr2c	-1.432525	5.561304	-2.517607	0.024097	0.088745	-4.431882	7.078581	3.879549	4.893885	5.233442	5.487291	4.592225	6.017549	4.406794	6.045953	5.642519	6.568338	4.97094	6.165795	6.916182
Cdc14a	0.463365	3.850122	2.516482	0.02415	0.088914	-4.303656	4.012279	4.32833	4.377553	3.946362	4.282511	4.765303	3.727855	3.242343	3.350165	3.501417	3.454671	3.880344	3.45286	3.692011
Dlg4	-0.458251	10.12153	-2.516143	0.024166	0.088945	-4.378114	10.51327	9.489397	10.12076	10.32046	9.913231	10.32417	10.21424	10.09737	10.1484	10.10291	10.00417	10.14702	10.1455	10.16189
Zbtb8b	-0.751408	2.068145	-2.51451	0.024244	0.089203	-3.897504	2.054131	0.934883	1.687412	2.43939	1.822268	1.585258	1.962529	2.505864	2.204856	2.325762	2.401899	2.656009	2.838755	1.931282
Flrt3	-0.961579	5.144019	-2.513188	0.024306	0.089368	-4.404488	4.808006	3.125682	4.813106	5.886966	4.695613	4.878898	5.354483	5.361925	5.740245	5.257979	5.285734	5.355829	5.689893	5.813505
Rpl23	0.461605	7.319056	2.512961	0.024317	0.089368	-4.45231	7.950342	8.470936	8.284052	8.278674	8.463557	9.080776	6.528508	6.574206	6.468273	6.676887	6.459854	6.50721	6.30588	6.429428
Hmcn1	-0.689804	2.977101	-2.512804	0.024325	0.089368	-4.058544	2.906846	2.690649	2.663852	3.027403	1.72577	2.086865	4.085519	2.738882	3.991425	3.00938	2.360517	2.990087	3.658907	3.968267
Actn2	-0.837675	5.463099	-2.512784	0.024326	0.089368	-4.425841	5.254031	3.702025	4.648255	5.846944	5.488762	5.002363	6.054927	5.832592	6.123835	5.528417	5.726745	5.329459	5.867892	6.117717
Fam13a	-0.575716	3.564591	-2.512752	0.024327	0.089368	-4.264608	3.88628	3.159427	3.545931	4.136703	3.403739	3.637716	3.241276	3.845499	3.456633	3.267546	3.786926	3.331185	4.061622	3.278825
Atp2b1	-0.87753	9.153608	-2.512627	0.024333	0.089368	-4.433459	8.720065	7.460192	8.479116	9.657334	8.193709	9.109689	9.379923	9.875573	9.684871	9.223589	9.581186	9.514879	9.694352	9.579194
Chrna7	-1.212995	3.241987	-2.51223	0.024352	0.08941	-4.031606	2.072763	1.184734	2.09528	4.034524	2.139408	2.116816	3.993741	3.770297	4.409542	3.060806	3.822919	3.811244	4.806206	4.295302
Cgrrf1	-0.398814	4.50081	-2.511573	0.024383	0.089477	-4.41253	5.169999	4.822713	4.440757	5.138114	4.733331	5.038019	4.134688	4.061624	4.19443	4.026112	4.404331	4.288919	4.334055	4.295302
Bid	0.928884	2.239324	2.511536	0.024385	0.089477	-3.933294	2.246933	2.854122	3.474186	1.526009	2.60856	2.146157	1.607516	2.099634	1.843557	2.027955	2.481265	2.404977	2.423441	1.951771

Xk	-1.066409	2.635517	-2.511069	0.024407	0.089504	-3.9972	2.127257	0.632508	2.33402	3.52156	2.139408	1.993092	2.918339	2.926907	3.244385	2.543858	3.189137	2.859272	3.3415	3.425211
Fam160a1	-1.910604	0.676432	-2.510918	0.024415	0.089504	-3.681572	3.263791	0.369671	0.25957	0.280432	-0.613814	-0.548781	0.625837	0.76329	0.571595	1.355481	2.556491	0.84773	0.313987	0.606649
Wdtc1	-0.310612	7.046019	-2.510481	0.024436	0.089504	-4.480644	7.310169	6.757376	6.754969	6.972749	6.859876	6.952876	7.20277	7.008319	7.121511	7.152087	6.983925	7.09134	7.199227	7.289081
Gem	0.929622	2.464428	2.510458	0.024437	0.089504	-3.963797	2.357438	2.619695	3.484656	1.679737	2.800826	2.695134	2.582602	2.709903	2.834633	2.758411	1.783193	2.461578	1.837926	2.194616
Nf1	-0.564066	6.354226	-2.510448	0.024437	0.089504	-4.476345	6.192607	5.955873	6.153307	7.000638	5.731741	6.294288	6.617678	6.572195	6.46042	6.016563	6.493743	6.311316	6.804966	6.373572
Thra	-0.389943	9.361451	-2.510442	0.024437	0.089504	-4.415701	9.754109	8.943799	9.40452	9.563873	9.531922	9.205244	9.213651	9.257281	9.411601	9.326319	9.281534	9.277627	9.450713	9.440527
Ddx18	0.454396	4.146939	2.509835	0.024466	0.089567	-4.312667	3.84352	4.125594	4.636566	4.070718	4.695613	4.178379	4.166561	3.910622	4.070936	4.178226	4.069701	4.132116	4.089463	3.978289
Rps15	0.992746	6.544614	2.509665	0.024475	0.089567	-4.474789	6.864036	7.649818	7.040037	5.332829	7.525831	6.125933	6.187702	6.184265	6.491579	6.618878	6.484944	6.385415	6.182049	6.569427
Ube2ql1	-0.815771	8.123933	-2.509615	0.024477	0.089567	-4.451939	8.550554	7.018685	7.930954	8.976495	8.45474	8.389562	7.920497	8.113172	8.010692	7.840395	8.438204	7.881141	8.372838	7.843031
Sucla2	-0.343253	7.140358	-2.508682	0.024522	0.089704	-4.48024	7.325787	7.011774	7.129076	7.668921	7.242107	7.231792	7.216157	6.959142	7.19256	6.79168	7.072019	6.812749	7.287507	7.034968
Tapbp	0.515825	4.317766	2.506665	0.024619	0.090031	-4.266441	3.953171	4.060109	4.266652	3.275	4.187881	3.926902	4.380979	4.899205	4.485023	4.850841	4.515339	4.936411	4.212723	4.581589
Prex1	0.416092	7.539406	2.506346	0.024634	0.09006	-4.483645	7.555102	7.854561	7.838827	6.978053	7.489556	7.549836	7.44618	7.602285	7.443223	7.500587	7.514292	7.839368	7.502085	7.446263
Bend6	-0.401202	6.995862	-2.505491	0.024675	0.090183	-4.483434	7.563772	7.078337	7.370497	7.775104	7.368535	7.255966	6.642444	6.585219	6.707663	6.511157	6.926594	6.493703	7.038319	6.637411
Cobl	-0.374283	4.652852	-2.504322	0.024732	0.09033	-4.444225	5.371558	5.05559	5.298783	5.418025	4.866025	4.861313	4.267989	3.966821	4.405458	4.116377	4.168924	4.368779	4.599077	4.440107
Tctex1d2	-0.408368	3.674968	-2.504207	0.024737	0.09033	-4.310497	4.026686	3.61957	3.614277	4.239206	3.67581	4.009049	3.706333	3.169712	3.555781	3.463704	3.633241	3.516245	3.805994	3.537917
Kcnj5	-2.259353	-0.413954	-2.503943	0.02475	0.09033	-3.365657	0.785575	-3.260591	-1.909221	-1.075778	-0.718553	-1.612049	-0.279766	1.279465	-0.504027	1.180545	1.135223	2.027776	0.791767	0.499386
Kif1b	-0.615169	9.109879	-2.5039	0.024752	0.09033	-4.441588	9.091373	8.691136	9.132437	9.602073	8.221402	8.879018	9.416971	9.333622	9.326409	8.662944	9.044889	9.190048	9.714699	9.23424
Fahd2a	-0.372807	5.307996	-2.503878	0.024753	0.09033	-4.444502	5.603877	5.11484	5.03414	5.169977	5.124418	4.777073	5.54436	5.340814	5.604319	5.279845	5.535475	5.333256	5.453267	5.436635
Gpr162	-0.316215	7.764807	-2.503465	0.024773	0.090376	-4.488506	7.641885	7.410261	7.237136	7.50555	7.26975	7.113759	8.246113	7.777481	8.192342	8.09471	7.830068	7.951376	8.145556	8.298883
Kcnh4	-0.726019	3.833269	-2.502764	0.024807	0.090473	-4.263307	4.021899	3.472958	3.73759	3.893455	2.77212	2.940957	4.118484	4.234368	4.560429	4.421893	3.70112	3.970866	3.671242	4.266966
Dusp8	-0.433364	6.312984	-2.50224	0.024833	0.090532	-4.490282	6.510498	5.673024	6.411743	6.402918	5.893738	6.1102	6.362996	6.427283	6.39601	6.319671	6.409173	6.524748	6.659318	6.300566
Wdr81	0.533683	5.568093	2.502117	0.024838	0.090532	-4.431438	5.099062	5.97968	5.471906	4.685481	5.216075	5.028202	5.77033	5.901494	5.741865	5.914028	5.863867	6.013007	5.546608	5.756413
Prkaca	-0.429531	7.542334	-2.501772	0.024855	0.090566	-4.485922	7.743863	7.122847	7.440406	8.052776	7.516847	7.795306	7.405579	7.615042	7.43423	7.46524	7.703735	7.3276	7.581021	7.396682
Pank3	-0.486073	6.599103	-2.499602	0.024961	0.090923	-4.500647	6.885272	6.424217	6.663465	7.404788	6.560265	6.986722	6.511028	6.254932	6.187061	6.280078	6.465558	6.629588	6.736183	6.414743
Oaf	0.674422	5.587357	2.499292	0.024976	0.090951	-4.45157	5.56163	6.086593	5.846391	4.583397	5.799686	5.219778	5.49291	5.605591	5.166377	5.812672	5.764251	6.207579	5.549951	5.56021
Bsg	-0.455189	9.889516	-2.498611	0.025009	0.091044	-4.425004	10.24476	9.257472	9.458634	9.807358	9.79626	9.782076	9.769257	9.93097	10.13166	10.16474	10.04645	10.01097	9.919952	10.13436
Pid1	0.722491	3.156801	2.497951	0.025042	0.091134	-4.181622	3.342675	3.184227	3.763618	2.687469	3.785203	4.090548	2.518182	2.988532	2.834633	2.694725	3.310002	3.127617	3.40196	2.648193
Klhl28	-0.853	2.615053	-2.497254	0.025076	0.091231	-4.14396	2.694686	2.100024	2.434653	3.924553	2.212449	3.193712	2.286414	2.68033	2.508863	2.088679	2.684976	2.656009	2.994118	2.417223
Slc25a30	-0.530209	2.954734	-2.496891	0.025093	0.091268	-4.180877	3.652372	2.655608	3.070623	3.107017	2.651221	2.957603	2.930765	2.91426	3.09008	2.572488	3.258364	2.941167	2.944167	2.825198
Plcb1	-0.865651	7.345657	-2.496671	0.025104	0.091279	-4.499695	7.320437	6.130854	6.842031	8.201426	6.9619	7.639289	7.426022	7.729558	7.398716	7.060943	7.773384	7.313735	7.861591	7.189614
LOC498122	-0.370546	3.985725	-2.495093	0.025182	0.091506	-4.362564	4.361994	3.747109	4.079015	4.383678	4.018988	4.153309	3.882992	3.95452	3.991425	3.790537	3.949256	3.890687	4.047497	3.648741
LOC100233176	0.498704	2.726087	2.49509	0.025182	0.091506	-4.107095	2.555898	3.06466	2.853524	2.758225	3.359399	3.359461	2.67414	2.400986	2.568526	2.438927	2.782205	2.619163	2.466543	2.503027
Psmc3ip	0.591923	2.768131	2.494382	0.025217	0.091597	-4.07552	2.947796	3.117121	2.795835	2.013053	3.252623	3.02233	2.828218	2.80888	2.462438	2.694725	2.833517	2.827322	2.748504	2.635584
Acsl4	-0.743575	7.27305	-2.494071	0.025232	0.091597	-4.507323	7.050657	5.992608	6.982097	8.060297	6.929229	7.335332	7.565041	7.181252	7.611778	7.188956	7.168593	7.289512	7.714398	7.763769
Sf3a3	0.431658	6.39778	2.49389	0.025241	0.091597	-4.5078	6.334466	7.115344	6.841013	6.353172	6.483401	6.661793	6.459426	6.319903	6.303568	6.324939	6.031446	6.152772	5.978732	6.227874
Cep19	-0.47206	5.97498	-2.493876	0.025242	0.091597	-4.507264	6.338321	5.78805	5.890367	6.790033	6.36184	6.331483	5.763399	5.89668	5.63778	5.760748	6.024839	5.599141	5.894439	5.598073
Sri	-0.388296	5.420764	-2.493811	0.025245	0.091597	-4.492692	5.805316	5.246268	5.68211	6.051963	5.530781	5.699604	5.089416	5.27061	5.253025	5.052302	5.378487	5.419718	5.359196	5.088999
Rbfox1	-0.98654	8.761572	-2.493627	0.025254	0.091602	-4.456294	9.100624	7.46947	8.807556	10.06368	8.722243	9.385114	8.555645	8.742231	8.670024	8.430036	8.889906	8.516025	8.944449	8.36888
Sox18	0.930202	3.873659	2.493367	0.025267	0.091621	-4.272312	4.069061	4.46953	3.918379	2.77782	4.824689	4.08677	4.267989	3.526187	4.14139	3.968034	3.048553	3.78376	3.356854	4.107181
Wnk3	-0.820864	3.017483	-2.492662	0.025302	0.091718	-4.17178	2.853968	2.279532	3.035447	3.942027	2.139408	2.924117	3.116285	3.159031	2.962454	2.998871	3.288097	2.827322	3.805994	3.11589
Qars	0.462434	7.065122	2.492519	0.025309	0.091718	-4.512229	6.738379	7.345773	7.139053	6.398178	6.964546	6.670023	7.295175	7.230544	7.274899	7.362422	7.058065	7.205007	6.890538	7.351133
Lpgat1	-0.828295	6.022052	-2.492253	0.025322	0.091737	-4.510877	6.261224	5.350374	6.101525	7.332512	5.825528	6.595536	5.653354	5.857579	5.745101	5.347671	5.929011	5.981902	6.417173	5.935544
Adhfe1	-0.709838	5.337826	-2.491292	0.025369	0.091879	-4.482502	6.03882	5.779937	5.143906	5.477652	5.022485	4.264517	5.495004	5.113519	5.332613	5.495156	5.289414	5.456736	5.332262	5.528164
Cers4	-0.44466	5.811539	-2.491156	0.025376	0.091879	-4.508068	6.224202	5.908288	5.816656	6.405282	5.446997	6.312197	5.754688	5.627064	5.841969	5.396526	5.431072	5.568867	5.863867	5.79236
Zfp2	-0.54577	3.67339	-2.490998	0.025384	0.091879	-4.321829	3.799454	3.134193	3.560852	4.405886	3.568609	3.984893	3.654827	3.727562	3.81791	3.401235	3.728487	3.46871	3.817137	3.482664
Vangl2	-0.851947	3.463001	-2.490784	0.025395	0.09189	-4.223231	3.188908	2.801663	3.387569	4.132904	2.16907	2.940957	3.529702	4.279444	4.091419	3.501417	3.373787	3.864689	3.522595	3.853172
Dennd1c	0.907118	4.863432	2.48951	0.025458	0.092091	-4.392492	4.3544	6.072271	4.93455	4.026356	5.030587	4.355253	4.739927	5.354922	4.73657	5.301385	4.899331	5.119046	4.195746	5.025175
Tubb4b	-0.430295	8.17121	-2.489317	0.025467	0.092098	-4.489611	8.608168	7.920313	8.102857	8.697775	8.411188	8.457701	8.059254	7.876971	8.203852	7.763328	8.272858	7.873339	8.156017	7.998837
Rad54l	0.994618	1.70919	2.488993	0.025484	0.092129	-3.79366	0.915118	2.452828	1.245402	1.128291	2.335271	2.05628	2.033384	1.830204	1.891068	2.108365	1.740817	1.404538	1.316372	1.971974
Zfp192	-0.439459	5.677997	-2.488597	0.025503	0.092173	-4.497985	5.528269	5.527177	5.506998	6.158573	5.189184	5.407165	6.199304	5.756695	5.908415	5.606633	5.593185	5.376215	5.861178	5.904347
Sema3g	0.734616	4.515234	2.488168	0.025525	0.092201	-4.344169	4.304037	4.907073	4.371924	3.381296	4.5954	4.370872	5.26653	4.208703	5.403951	4.51702	4.128407	4.465359	4.42791	4.938001
Acly	-0.349671	7.968638	-2.488129	0.025527	0.092201	-4.500536	8.198505	7.803785	8.037022	8.449905	7.970538	8.086267	7.977761	7.63926	8.049487	7.502448	7.821197	7.68153	8.303214	8.046352
Ddx49	0.289849	5.69135	2.487281	0.025569	0.092315	-4.482412	5.272282	5.677398	5.559943	5.29763	5.607258	5.45631	5.870489	5.78302	5.820657	5.994252	5.941928	5.822216	5.855784	5.750578
Cltb	0.297276	7.564247	2.487158	0.025575	0.092315	-4.511874	7.801202	7.938952	7.911442	7.604541	8.072155	8.079391	7.246298	7.349814	7.377501	7.418694	7.459408	7.251895	7.229258	7.167338
Sec61b	0.480596	3.759529	2.486908	0.025588	0.092315	-4.307083	3.917539	3.943895	3.989949	3.374877	4.258567	4.254448	3.699086	3.599118	3.548392	3.832569	3.958531	3.447848	3.576053	3.35002
Selenbp1	-1.061255	0.933204	-2.486884	0.025589	0.092315	-3.743539	2.054131	1.248835	-0.067513	0.785264	0.371962	0.056669	1.17791	1.020936	1.579251	1.322149	0.961591	1.515634	0.698429	1.227132
RGD1309730	-0.405075	4.269357	-2.486436	0.025611	0.092318	-4.426014	4.70129	4.290759	4.102999	4.874798	4.598129	4.559885	3.96382	4.134313	4.044916	4.0968	4.341911	4.051439	4.250206	3.842239
Hsd11b1	0.547344	6.238155	2.486321	0.025617	0.092318	-4.518981	6.449429	7.068934	6.749552	5.823566	6.621221	6.288543	6.046387	5.593743	6.191812	6.143636	5.852718	5.9327	6.130496	6.462825
Slc25a39	0.41185	5.815853	2.486295	0.025618	0.092318	-4.502826	5.906157	6.127661	5.792629	5.312821	6.241356	5.9088	5.744165	5.813989	5.570064	5.940353	5.925468	5.790806	5.631121	5.744718
Rps23	0.514539	5.567202	2.48625	0.02562	0.092318	-4.488526	5.635554	6.171732	5.630004	4.984269	5.988284	5.491028	5.666417	5.561662	5.405997	5.677534	5.387099	5.333256	5.455051	5.586654
Dyrk3	-0.668589	2.551999	-2.485465	0.02566	0.092432	-4.106703	2.730329	1.79116	2.508341	3.175097	1.997824	2.854719	2.467906	2.964197	2.810125	2.529327	2.782205	2.461578	2.58862	2.341603
Phldb3	1.667483	0.797415	2.485187	0.025674	0.092455	-3.758717	1.895723	2.843782	3.077557	-0.411255	1.765147	1.402804	0.357848	-0.26106	0.091681	0.055728	1.375754	0.714338	-0.405292	-0.204094
Snx16	-0.597001	4.195463	-2.484622	0.025702	0.092508	-4.413338	4.268111	3.7962	3.941366	5.164405	4.272298	4.507149	4.263083	4.303891	4.272747	3.715505	4.017407	3.956168	4.318455	4.027379
Tsen15	0.382305	4.002725	2.484583	0.025704	0.092508	-4.370892	4.092076	4.364946	4.299641	4.022255	4.693063	4.392459	3.454237	3.698353	3.669138	3.940842	3.967746	3.911153	3.828196	3.803307
Casp3	0.421175	4.303728	2.484007	0.025733	0.092584	-4.40741	4.10118	4.610386	4.566831	4.549599	4.949583	4.881081	4.197745	3.904241	4.13647	4.224061	4.152854	4.212646	3.908538	3.937777
Bai3	-0.391644	7.182288	-2.483741	0.025746	0.092599	-4.530366	6.81577	6.328272	6.664041	7.235318	6.701435	6.841256	7.640745	7.52068	7.612222	7.41968	7.227325	7.453291	7.45649	7.646901
Diablo	0.453818	3.544272	2.483558	0.025755	0.092599	-4.260218	3.516065	3.874479	3.805981	3.182467	4.006719	3.491064	3.383673	3.668539	3.563133	3.360772	3.684447	3.38339	3.416687	3.417866
Fut10	-0.560154	2.248882	-2.483441	0.025761	0.092599	-4.111667	2.977763	2.100024	2.162035	2.853641	2.496038	2.675144	1.668148	2.030999	2.147322	1.73055	2.197736	2.390473	2.379013	2.011549
Sox2	0.40797	6.178245	2.483311	0.025768	0.092599	-4.5254	6.179773	6.562271	6.435549	6.229662	6.55255	6.899492	5.977681	5.953414	5.694114	5.898564	5.757625	6.24232	6.017894	6.116583
RGD1305235	-0.601225	4.887947	-2.48206	0.025831	0.092798	-4.476171	5.139392	4.129856	4.648255	5.567645	4.947445	5.282232	4.861747	4.792678	4.884753	4.663938	5.069498	4.701051	5.089637	4.707418
Elavl2	-1.246872	5.280114	-2.481843	0.025842	0.09281	-4.504966	5.627699	3.397198	5.021611	6.724648	5.52075	5.747056	5.626867	5.157111	5.094627	4.602796	5.450923	4.822175	6.058371	5.116484
Psma7	0.301173	6.670476	2.481453	0.025861	0.092853	-4.532572	6.630899	6.868661	6.890083	6.405282	6.796554	6.719662	6.672353	6.600102	6.696843	6.72231	6.789096	6.555139	6.547508	6.507627
Ncoa2	-0.640105	5.534728	-2.481045	0.025882	0.092899	-4.507072	5.536682	5.216521	5.519781	6.13027	4.656883	5.387335	5.538282	5.893462	5.741865	5.181051	5.789158	5.650258	5.948647	5.331105
Wbp2	-0.538146	8.356846	-2.479862	0.025942	0.093069	-4.487179	9.124122	8.266697	9.114957	9.458254	8.720053	8.916085	7.666051	8.124883	8.098378	7.762163	8.049927	7.856618	8.081149	7.761595
LOC100233213	-0.508618	3.281684	-2.479619	0.025954	0.093069	-4.33152	3.838085	2.991607	3.780713	4.203428	3.639189	3.622139	3.083269	2.939445	2.797714	2.783118	3.157269	2.910998	3.302384	3.060363
Nrg1	-0.548148	1.368481	-2.479487	0.025961	0.093069	-3.9448	1.996747	1.248835	1.825611	2.184345	1.745593	1.378286	1.297885	0.972957	1.218642	0.893396	0.998029	0.969826	1.60059	1.445134
Fut2	-0.793315	0.822578	-2.479432	0.025964	0.093069	-3.681531	0.955837	0.118359	0.20995	0.896996	0.606834	-0.214525	0.851758	1.532126	1.360416	1.569444	1.102128	1.515634	1.11438	0.844371
Etv4	0.614291	4.027596	2.479304	0.02597	0.093069	-4.363639	3.932918	4.616473	4.811028	3.838533	4.147475	4.401612	4.292269	3.349568	4.217909	3.608978	3.733898	3.631738	3.45286	4.450943
Sertad4	-0.516222	4.516713	-2.479188	0.025976	0.093069	-4.39039	4.197708	3.884602	3.719973	4.627263	3.596165	4.422747	4.893837	4.9924	4.928103	4.885482	5.021628	4.680333	4.725704	4.731318
Ptprcap	1.239815	0.730354	2.478767	0.025997	0.093094	-3.492382	-0.518419	0.895377	0.818644	-0.941548	0.15195	-0.291112	0.952703	2.121805	0.97585	2.14695	1.581856	2.100868	0.649393	0.753927
Myh2	-0.947074	1.15765	-2.478747	0.025998	0.093094	-3.870675	2.501925	1.047298	0.750864	1.327578	0.88224	1.193889	1.135593	1.393205	0.837186	0.938085	2.303426	1.082389	0.791767	0.753927
Smpd3	-0.457055	7.27685	-2.478131	0.02603	0.093178	-4.540299	7.205363	6.189194	6.84254	7.181624	7.022566	6.891393	7.638853	7.530036	7.480586	7.414253	7.570572	7.655609	7.71328	7.550696
Zfr	-0.599464	8.43251	-2.477819	0.026045	0.093207	-4.49946	8.686056	7.933477	8.519933	9.437903	8.451726	8.941019	8.383826	8.091218	8.259217	7.913204	8.222017	8.148762	8.632409	8.439044
Mettl10	-0.427964	2.927133	-2.476976	0.026088	0.093332	-4.173751	3.118917	2.570375	2.568639	3.107017	2.847434	2.772436	3.159163	2.964197	2.858731	2.866406	3.20481	2.931181	3.186856	3.031778
Thrb	-0.478484	5.595918	-2.476793	0.026098	0.093338	-4.529091	6.165739	5.61796	5.779933	6.059988	5.259808	5.878525	5.592083	5.295206	5.345461	5.26018	5.543201	5.370683	5.946111	5.261001
Nop9	0.365716	4.078332	2.476319	0.026122	0.093397	-4.356181	3.848935	4.12132	4.308508	3.861672	4.358498	4.082982	4.258161	4.171987	4.106593	3.994723	4.164923	3.990233	3.908538	4.012827
Znrd1	0.672088	3.127219	2.47608	0.026134	0.093412	-4.131888	2.718545	3.684748	3.436929	2.41457	3.188895	2.613455	3.29032	3.159031	3.518449	3.186517	3.31723	3.136336	3.079891	3.220915
Chek2	0.901043	1.536231	2.475315	0.026173	0.093498	-3.847904	1.212615	2.134368	1.574317	1.158502	1.877213	2.705027	0.563361	1.356279	1.360416	1.704918	1.652137	1.890216	1.651284	1.227132
Runx1t1	-0.851741	4.305874	-2.475305	0.026174	0.093498	-4.443595	4.546601	3.506122	4.302603	5.368806	3.97768	4.636834	3.999651	4.688835	4.329893	3.545408	4.530966	3.946285	4.874473	4.111741
Zfp316	-0.310249	4.699528	-2.473013	0.026291	0.093889	-4.438193	4.633881	4.263866	4.302603	4.638025	4.248182	4.490087	4.874668	4.803005	4.899348	5.008447	4.822678	4.872617	4.979878	5.017906
Atp6v0a1	-0.41358	9.721014	-2.472644	0.02631	0.093929	-4.484802	9.472356	8.929813	9.297886	9.910676	9.442905	9.439176	9.937456	9.853281	10.14151	9.867693	9.77106	9.895905	9.943184	10.19322
Nfkbia	0.769216	5.074781	2.471809	0.026353	0.094016	-4.511807	5.292294	6.780601	6.352237	5.130514	5.52075	5.265617	4.663805	4.823442	4.553063	4.687033	4.576858	4.647164	4.442376	4.361886
Aldh6a1	-0.392031	6.460646	-2.471585	0.026364	0.094016	-4.552746	7.325787	6.739359	6.831303	6.866376	6.586607	6.661158	6.081642	6.064895	5.948265	6.282246	6.088533	6.433075	6.318678	6.239378
Celf4	-0.390903	9.376294	-2.471585	0.026364	0.094016	-4.476955	9.888601	9.396502	9.468393	10.01517	9.441956	9.937133	9.337676	8.928866	9.178536	8.913241	9.064753	8.964598	9.487471	9.247638
Pcdh8	-1.022571	6.74736	-2.471394	0.026374	0.094016	-4.544556	6.414249	4.267739	5.755281	6.872654	5.92667	6.512774	7.677619	7.154615	7.613107	7.371615	7.00534	7.27478	7.007033	7.628723
Rrm2b	-0.741011	2.48419	-2.471325	0.026377	0.094016	-4.140413	2.694686	2.294795	2.298852	3.329127	2.09374	2.493017	2.467906	2.38274	2.539002	1.704918	2.802949	2.346065	3.310292	2.310209
Ccdc92	-0.428584	7.25585	-2.471252	0.026381	0.094016	-4.541116	7.963818	7.207949	7.664162	7.952347	7.905669	7.344063	6.774994	6.986585	6.88753	6.938439	7.217701	6.910018	7.022159	6.817061
Uxt	0.354183	4.117242	2.470001	0.026446	0.094199	-4.433873	4.421353	4.515857	4.673644	4.277563	4.784532	4.820946	3.577905	3.825375	3.924502	3.790537	3.963146	3.60686	3.789115	3.763295
Rabl6	-0.284292	8.049356	-2.469954	0.026448	0.094199	-4.536357	8.232822	8.053308	7.782923	8.204597	7.93186	7.968015	8.116469	8.008106	8.002942	8.172164	8.019231	8.041163	8.030883	8.132449
Gmps	0.507596	4.934859	2.469673	0.026462	0.094222	-4.501104	4.611755	5.391775	5.448028	5.439714	5.460554	5.856504	4.626095	4.775299	4.582303	4.386051	4.747086	4.492777	4.808999	4.514305
Rtn3	-0.545661	10.24508	-2.469383	0.026477	0.094248	-4.476695	9.936834	8.988486	9.649861	10.3024	9.889762	9.771083	10.364	10.4535	10.7074	10.5519	10.52946	10.62831	10.76651	10.893
Dctn1	-0.302721	9.686301	-2.468185	0.026539	0.094419	-4.486019	9.715076	9.376578	9.562905	9.978861	9.619378	9.616456	9.738882	9.72575	9.738617	9.665771	9.668466	9.656186	9.806499	9.740705
Stoml2	0.346206	5.992491	2.467993	0.026549	0.094419	-4.546413	6.076236	6.354913	6.226873	5.703196	6.333468	6.021942	6.029154	5.763762	5.893911	5.972933	5.987948	5.812728	5.842211	5.900399
Lrrn4cl	0.965999	0.326862	2.467992	0.026549	0.094419	-3.639247	0.176305	0.679782	1.004744	-0.02435	0.917619	1.302125	-0.279766	0.58326	0.317129	0.055728	0.764165	1.219993	-0.211665	-0.621773
Rab3il1	0.948332	3.188485	2.467617	0.026569	0.094433	-4.186916	3.303772	2.864389	4.287732	2.124384	3.89578	3.359461	2.534559	3.606999	2.894141	3.258764	3.414796	3.587916	3.079891	2.610029
Klhl11	-1.076885	4.444411	-2.467615	0.026569	0.094433	-4.459974	4.536569	3.309744	4.564365	5.656328	3.61245	4.576145	4.27775	4.823442	4.582303	3.838474	4.79447	4.331301	5.418933	3.978289
Ddx19a	0.313744	5.528165	2.467125	0.026594	0.094482	-4.541609	5.627699	5.871858	5.961432	5.788981	5.999679	6.256109	5.185883	5.232912	5.081888	5.345599	5.419364	5.257355	5.208658	5.191628
Adarb2	-0.7719	2.839512	-2.467046	0.026598	0.094482	-4.182874	2.765113	2.324846	2.379614	3.622545	2.308867	2.781814	2.659281	3.201289	2.639794	2.745897	3.189137	2.772447	3.743115	2.847239
Gart	0.333658	5.561958	2.466476	0.026628	0.094559	-4.534574	5.529955	5.693323	6.083556	5.511695	5.763686	5.870856	5.359088	5.522617	5.323984	5.307785	5.488226	5.361417	5.592729	5.491897
Mob3b	-1.109096	0.486046	-2.465489	0.026679	0.094713	-3.749076	1.245833	-0.366503	0.049949	1.300739	0.266146	0.915838	-0.279766	1.155984	0.736738	0.134646	0.378374	1.082389	1.407665	-0.03329
Osbpl2	-0.293807	6.523086	-2.46505	0.026702	0.094766	-4.562897	6.555903	6.331051	6.406934	6.779137	6.417736	6.34103	6.542746	6.702952	6.44856	6.486922	6.702733	6.482634	6.62406	6.517969
Trim16	0.88673	2.154727	2.464489	0.026731	0.094825	-4.106497	2.035255	3.643613	3.776458	2.546081	2.507694	2.791131	1.63815	1.318383	1.458292	1.828755	1.430105	1.950783	1.943396	1.684306
Avp	2.36619	-1.046654	2.464385	0.026737	0.094825	-3.465298	0.105685	1.79116	1.317458	-2.344749	-0.718553	0.949514	-2.558588	-0.992527	-0.929891	-1.33445	-1.719763	-1.073407	-1.047095	-1.899685
RGD1310852	-0.846492	3.338516	-2.464274	0.026742	0.094825	-4.281915	3.380557	2.200694	2.718102	4.04669	3.188895	3.402651	3.392685	3.884925	3.350165	2.807409	3.867914	3.744381	3.794763	3.12494
RT1-N3	0.778609	3.493736	2.464104	0.026751	0.094829	-4.242707	2.927466	3.595119	3.330668	3.051752	4.608991	3.547133	3.727855	3.606999	3.333065	3.566911	3.493474	3.755742	3.042398	3.468514
Ncln	0.519935	8.12903	2.463841	0.026765	0.094849	-4.558296	7.456127	8.359216	7.854302	7.452814	8.048163	7.630232	8.632306	8.228767	8.545184	8.551634	8.088194	8.239059	8.018264	8.708041
Wdr82	-0.571469	3.862246	-2.463669	0.026774	0.094853	-4.429585	4.17633	3.382986	3.986269	4.86567	4.220119	4.220367	3.742028	3.458111	3.525993	3.478908	3.78171	3.61312	3.98464	3.745801
Nabp1	0.550392	4.270977	2.462632	0.026828	0.095017	-4.451273	3.896775	4.598135	4.915329	4.773515	4.757939	5.303548	4.096591	3.845499	3.907275	4.066928	3.921068	4.018805	3.783444	3.993192
Cct5	0.326572	7.512374	2.461888	0.026867	0.095127	-4.564059	7.389412	7.435873	7.822669	7.448618	7.90181	7.824242	7.451581	7.38862	7.500348	7.361397	7.481291	7.280691	7.476396	7.418929
Cyfip2	-0.348963	10.86291	-2.461674	0.026878	0.095138	-4.448247	11.19035	10.61978	11.05585	11.20703	10.77033	10.95912	10.87735	10.73698	10.79937	10.60931	10.81305	10.7202	10.9464	10.77642
Rai2	-0.539639	3.277021	-2.461291	0.026898	0.095164	-4.352813	3.589067	3.091129	3.426105	4.314927	3.339971	3.830786	2.828218	3.012464	3.216682	3.223093	3.212584	3.037402	2.805569	3.11589
Tmc8	1.483893	1.276829	2.461236	0.026901	0.095164	-3.682774	0.369528	1.812527	1.317458	-0.093872	1.578815	1.496914	1.63815	1.934092	1.360416	2.469694	1.674823	2.153355	-0.628995	1.582867
Ubxn2b	-0.639043	4.37931	-2.460574	0.026936	0.095259	-4.426803	4.137033	3.967899	3.767911	4.819147	3.568609	4.109292	5.075493	4.424739	4.959085	4.613168	4.612548	4.018805	5.009325	4.307278
Heatr5b	-0.33297	6.275744	-2.460334	0.026948	0.095273	-4.563364	5.986964	5.658348	5.96611	6.39501	5.866839	5.942639	6.632969	6.514708	6.612548	6.300545	6.348731	6.403585	6.663955	6.588362
Maged1	-0.494002	8.913172	-2.460193	0.026956	0.095273	-4.530799	9.010933	7.990834	8.849806	9.116414	8.868258	8.57848	8.967508	9.119974	9.170587	8.987541	9.098968	8.880497	9.126517	9.021441
Psma2	0.313055	6.083294	2.459821	0.026975	0.09529	-4.56991	6.239743	6.388203	6.685189	6.349903	6.739003	6.617961	5.846094	5.834271	5.851008	5.654285	5.868795	5.636495	5.760865	5.718054

Zfp689	-0.313862	4.216825	-2.4598	0.026977	0.09529	-4.421461	4.380808	3.939046	4.068613	4.228565	3.973484	3.972661	4.339638	4.374841	4.342766	4.349296	4.457635	4.245254	4.156802	4.291288
Rtn4rl1	-0.75775	7.362406	-2.459493	0.026993	0.095319	-4.572608	7.178196	5.64203	7.019032	7.663006	7.008278	6.976549	7.505018	7.681854	7.803219	7.558088	7.629101	7.637299	7.751539	8.031088
Ncam2	-0.855789	5.547333	-2.456428	0.027154	0.095861	-4.560944	5.513001	4.91203	5.579612	6.790033	4.879544	5.811428	5.328891	5.726267	5.738623	4.941431	5.31852	5.323745	6.225564	5.609402
Map1a	-0.37025	10.88595	-2.456124	0.02717	0.09589	-4.460414	10.95617	10.73406	10.75667	11.35427	10.79558	10.84706	10.91395	10.77108	10.91679	10.56219	10.77442	10.86366	11.22486	10.93336
Pja2	-0.821643	9.43174	-2.455855	0.027185	0.095906	-4.514729	9.474438	8.102385	9.236871	10.22327	9.222255	9.550863	9.524521	9.623963	9.603868	9.072365	9.743742	9.373735	9.907868	9.386636
Dock7	0.342923	6.157223	2.455705	0.027192	0.095906	-4.570426	5.867997	6.28498	6.256198	6.005973	6.179853	6.39931	6.485984	6.049074	6.197728	6.112292	5.921916	6.162408	5.995996	6.303558
Mog	-0.755633	7.323375	-2.455586	0.027199	0.095906	-4.579751	7.558411	7.363072	6.178517	7.29688	7.120229	6.033765	7.418872	7.599326	7.444219	7.603013	7.401502	7.933041	7.7428	7.844401
Wscd1	-0.476036	7.989931	-2.455311	0.027213	0.095929	-4.570427	8.095498	7.776183	7.252152	7.858847	7.703882	7.271443	7.974013	8.231955	8.078635	8.211947	8.081046	8.611351	8.284267	8.434274
Tmem163	-0.683371	2.846147	-2.454997	0.02723	0.09596	-4.199609	3.460552	2.439156	2.225837	2.825673	2.883666	2.28453	2.550752	2.91426	2.834633	2.843096	2.782205	3.323571	3.286436	3.417866
Fam219a	-0.340341	7.102685	-2.454127	0.027276	0.096094	-4.583806	7.270367	7.123916	7.15348	7.320871	6.73529	6.809515	7.099918	7.022383	7.27434	7.048259	7.165092	7.09358	7.277456	7.054638
Pgm2l1	-0.946375	7.774226	-2.453378	0.027316	0.096185	-4.566454	7.896123	6.713326	7.38873	8.944342	7.485509	8.301025	7.77775	7.503368	7.637689	7.242831	8.033015	7.607039	8.535377	7.78069
Raver2	-0.403099	5.839157	-2.453342	0.027318	0.096185	-4.565817	5.834269	5.333844	5.297299	5.955246	5.628766	5.710694	6.136317	6.293288	6.071954	5.811171	5.953571	5.839671	6.075873	5.834346
Cd24	0.477993	6.138483	2.452933	0.02734	0.096233	-4.580559	6.226284	6.773809	6.475468	6.052969	6.529152	6.689462	5.742404	5.850958	5.368723	5.974275	5.941928	6.366086	6.194922	5.775217
Lppr1	-1.20752	4.01197	-2.452239	0.027377	0.09633	-4.358004	3.88628	1.117652	3.776458	4.549599	3.217567	3.817169	4.0114	4.803005	4.659784	4.487257	4.341911	4.509652	4.56643	4.558395
Fbxl4	-0.52066	4.223775	-2.451976	0.027391	0.09633	-4.427021	4.403064	3.142653	4.19832	4.249769	4.018988	3.803422	4.531353	4.374841	4.409542	4.294496	4.309659	4.327499	4.533027	4.623699
Alk	-0.550595	3.730154	-2.451967	0.027391	0.09633	-4.290982	3.742407	2.953644	2.992072	3.309066	3.203302	2.734303	4.306644	4.203515	4.096495	4.259708	3.853071	4.304475	3.892823	4.500463
Ppil1	0.355739	4.719988	2.450823	0.027452	0.096516	-4.522462	4.851815	5.039823	5.153781	4.926188	5.354824	5.426725	4.177031	4.464319	4.389005	4.571225	4.667312	4.335093	4.488413	4.295302
Abcg1	-0.38685	6.905168	-2.4505	0.027469	0.096537	-4.590801	6.678815	6.213492	6.529562	6.959941	6.410769	6.576802	6.918391	7.306311	6.94071	7.307595	7.081601	7.654851	7.04248	7.064667
Ptrf	0.814196	3.924449	2.45039	0.027475	0.096537	-4.360757	4.227114	3.96313	4.139901	2.77782	4.272298	4.726978	3.957761	4.166665	4.029076	3.808701	3.50618	4.208518	3.562873	3.704139
Uqcrc2	-0.280531	8.127535	-2.450259	0.027482	0.096537	-4.567095	8.404905	8.022409	8.151925	8.442059	8.211859	8.186759	8.205139	7.970804	8.201793	7.798216	8.045582	7.879843	8.155743	8.114103
Fam168a	-0.499874	7.781773	-2.449935	0.0275	0.096553	-4.574792	8.103732	7.458498	7.978726	8.339275	7.574092	7.877425	7.589741	7.763887	7.500826	7.405328	7.878513	7.834688	8.120756	7.526569
Anxa6	-0.595002	8.038291	-2.449875	0.027503	0.096553	-4.566128	8.408118	7.79274	7.485741	8.744582	8.576986	8.062899	8.104863	8.250312	7.688994	7.71479	7.969061	7.966428	7.924771	7.851915
Ngf	-0.865741	2.452668	-2.449649	0.027515	0.096567	-4.05683	2.387517	0.480615	1.858187	2.602113	2.308867	1.857753	2.828218	3.126505	2.694377	3.110461	2.604547	2.9008	3.042398	2.868948
Agtpbp1	-0.482867	8.350037	-2.448221	0.027591	0.096808	-4.566473	8.38291	7.833811	8.025191	8.887982	8.128444	8.618775	8.695117	8.334597	8.419437	8.266629	8.324212	8.183092	8.490185	8.315036
Spint2	-0.65221	5.305129	-2.447602	0.027625	0.096896	-4.551726	6.000408	4.687583	5.055367	5.097757	4.989615	4.825487	4.965043	5.350235	5.175999	5.834998	6.347849	5.588061	4.997129	5.397961
Pqlc2	0.877073	1.82887	2.447238	0.027644	0.096936	-3.856802	0.995438	1.914859	2.122353	1.006693	2.226624	1.136566	2.343298	2.099634	2.147322	1.964563	1.581856	2.252925	2.022549	2.261799
Tmem81	0.876106	1.044369	2.446663	0.027675	0.097016	-3.734462	0.692264	1.655851	1.220561	0.105385	1.312595	0.734546	1.297885	1.155984	1.255413	1.652245	1.403186	1.152831	0.836265	0.929479
Wash	0.585929	5.388588	2.446481	0.027685	0.097023	-4.542851	5.189338	6.145662	5.396283	4.802503	5.616707	5.165286	5.484505	5.391882	5.343328	5.526589	5.314913	5.448008	4.987296	5.666301
Frmd4a	-0.433279	4.974872	-2.446286	0.027695	0.097031	-4.543631	5.214725	5.037557	4.889952	5.396005	4.57061	5.004367	4.722163	5.324179	4.64946	4.809346	5.021628	5.37253	4.969927	4.716427
Kctd6	-0.701475	4.971471	-2.444875	0.027772	0.097269	-4.540934	5.048187	3.884602	4.673644	5.648366	4.912799	5.110518	4.818933	4.799571	5.154258	4.896847	5.300397	4.838295	5.519575	5.046765
Slc25a33	-0.353753	4.930324	-2.444213	0.027807	0.097361	-4.525877	5.105862	4.733113	4.53192	4.904069	4.762811	4.581525	5.178086	4.771798	5.225489	5.09979	5.198397	4.908688	5.047677	5.02759
Tpd52l1	-0.640588	3.81841	-2.444089	0.027814	0.097361	-4.433076	4.47136	3.929299	4.082466	4.1518	3.711525	2.957603	3.969854	3.272386	4.342766	3.62956	3.695584	2.970717	4.148003	4.242228
Mmp24	-0.291745	6.856832	-2.443653	0.027838	0.097377	-4.603796	6.964733	6.515807	6.458305	6.69081	6.679704	6.490784	6.981721	6.961449	6.997436	7.102641	6.956413	7.067605	7.01975	7.122292
Ap1ar	-0.582536	6.436936	-2.443566	0.027842	0.097377	-4.603278	6.72298	5.678853	6.476124	7.099818	6.402989	6.755207	6.419201	6.423941	6.511666	6.11351	6.526074	6.268334	6.605701	6.131254
Gstm1	-0.353713	7.7979	-2.443556	0.027843	0.097377	-4.581756	8.555323	8.020115	8.171297	8.168403	7.996621	7.851218	7.625059	7.600313	7.522673	7.673676	7.334066	7.542498	7.464928	7.651605
LOC360231	1.770462	0.700201	2.442738	0.027887	0.097504	-3.605084	0.243629	1.397635	1.386086	-1.223791	0.091273	1.519512	0.118118	1.747064	-0.504027	1.023513	1.347792	2.419337	1.1849	0.257494
Ltc4s	-1.347224	1.138156	-2.441977	0.027929	0.09762	-3.714674	1.764345	-0.103798	-0.974047	0.22441	0.15195	-0.214525	1.576217	2.709903	1.489496	2.795315	1.996325	2.838051	0.836265	1.868007
Tdrkh	-0.363845	4.738101	-2.440654	0.028	0.097843	-4.518023	4.621279	4.335728	4.443444	5.080109	4.633136	4.554424	4.877881	4.899205	4.867041	4.680472	4.840346	4.683311	5.028628	4.847954
Lpcat2	0.702105	4.52519	2.439717	0.028052	0.097993	-4.411412	3.997728	4.707679	4.284739	3.41297	4.213017	4.380163	4.77481	5.368893	4.441807	5.341446	4.650242	5.497911	3.91892	4.436477
Dlg2	-1.051144	6.619318	-2.439449	0.028066	0.098015	-4.604788	7.41656	5.998447	7.064673	8.177422	6.203337	7.709765	6.229796	6.417234	6.148482	5.548372	6.357522	6.190936	7.141108	6.084468
Pcdhb2	-0.708752	0.46587	-2.439298	0.028074	0.098016	-3.790994	0.995438	0.118359	0.818644	1.188094	0.091273	0.61372	0.563361	0.222004	0.512061	0.534523	0.432628	0.28321	0.836265	0.442608
Kcna2	-1.240754	4.116928	-2.438054	0.028142	0.098224	-4.487755	4.272147	3.588941	4.53192	5.607886	2.970456	3.722947	3.790562	4.523808	3.958351	3.100666	4.341911	4.327499	5.340009	3.661237
Cml5	-1.230394	1.671779	-2.43758	0.028168	0.098267	-3.943401	2.799078	1.453108	-0.411358	1.245516	1.20216	1.107027	2.033384	1.775313	2.00342	2.325762	2.627988	1.890216	1.724133	2.228598
Myom2	-0.606596	2.940028	-2.437531	0.028171	0.098267	-4.256731	3.016775	2.200694	2.984714	3.456176	2.530726	2.809587	3.410541	2.91426	3.31576	2.469694	3.250834	2.568523	3.36447	3.079111
Rnf208	-0.383088	7.178106	-2.437341	0.028181	0.098275	-4.611358	7.676177	6.867385	7.400873	7.420455	7.343958	7.054059	7.035212	7.031927	7.173473	7.078516	7.256758	6.923304	7.17236	7.069949
Bbs10	-0.452697	3.60277	-2.436963	0.028202	0.098319	-4.358547	3.718942	2.780133	3.301354	3.686144	3.416161	3.47381	3.96382	3.756192	3.805565	3.734632	3.728487	3.516245	3.97969	3.710165
Frem1	-1.000399	2.217856	-2.436639	0.02822	0.098339	-4.061754	3.05476	2.264105	1.493674	1.874319	0.470544	1.668504	2.8014	2.522625	2.347926	2.877922	2.968181	2.461578	2.508394	2.124152
Tmem179	-0.402461	6.520999	-2.436559	0.028224	0.098339	-4.603937	6.099138	5.449176	6.009355	6.106253	5.826691	5.510901	6.816603	6.977495	7.097029	7.192419	6.915326	6.969777	7.176152	7.166791
Cacnb4	-0.738764	5.242365	-2.436317	0.028237	0.098356	-4.583735	5.354528	5.244304	5.526131	6.184497	4.436792	4.932509	5.617289	5.237996	4.956296	4.680472	5.27278	5.035286	5.768061	5.189473
RGD1565616	-0.400404	7.230216	-2.43555	0.02828	0.098456	-4.619128	7.10258	7.021555	6.369995	7.038698	6.680992	6.611402	7.760884	7.598338	7.437234	7.474751	7.322439	7.669942	7.600904	7.544386
Med12l	-0.930751	2.445494	-2.435485	0.028283	0.098456	-4.10181	2.072763	2.046927	2.322392	3.114742	1.113405	1.327961	3.003159	3.252427	2.707706	2.291729	2.684976	2.594065	3.294432	2.733504
Naa10	0.400588	4.986751	2.435351	0.028291	0.098456	-4.53823	4.867907	5.344885	5.026994	4.594489	5.238981	4.909165	5.162364	4.860173	5.183174	5.057375	4.962928	4.90359	4.734547	5.017906
Gabrb1	-0.931478	3.885211	-2.435151	0.028302	0.098466	-4.425494	3.658553	2.780133	3.560852	4.904069	3.188895	3.905613	3.669732	4.346879	4.131534	3.649853	4.196624	3.811244	4.855682	3.847716
Cfh	0.696712	6.123787	2.434765	0.028323	0.098511	-4.60776	6.07739	6.076693	6.234648	5.607886	6.787618	7.045812	5.302835	6.55702	5.362416	6.375573	6.103242	7.115794	5.404229	5.706042
Rgd1310049	-0.487857	7.118637	-2.434125	0.028358	0.098577	-4.620892	7.025771	6.540027	6.507269	7.504815	7.228964	6.831694	7.456961	7.224794	7.168059	7.056516	7.336736	7.052138	7.514547	7.22424
Cbr4	-0.595963	3.343863	-2.43412	0.028358	0.098577	-4.372231	3.748215	2.864389	2.999392	3.857074	3.036313	3.935331	3.462819	3.340143	3.548392	2.966878	3.499841	3.110019	3.416687	3.186749
Sod3	-0.772572	6.233165	-2.43331	0.028403	0.098676	-4.621957	7.601554	6.146713	5.932087	6.627421	6.631245	6.663062	5.526049	5.634794	5.876312	6.367427	6.840168	5.980693	5.553287	5.90566
Slc22a17	-0.462744	10.1038	-2.433307	0.028403	0.098676	-4.55533	9.936675	9.049654	9.43276	9.576602	9.64236	9.061535	10.56727	10.37224	10.78284	10.65803	10.4976	10.48506	10.45663	10.93557
Lrrc1	-0.497361	3.380922	-2.433107	0.028414	0.098686	-4.358683	3.683012	3.519177	2.992072	3.665254	3.174343	3.076678	3.261094	3.377479	3.159628	3.602051	3.173291	3.761389	3.318158	3.722142
Irf7	1.035472	3.19821	2.43224	0.028462	0.09881	-4.16051	2.582145	3.108509	3.595078	1.910273	3.144791	3.121686	3.299932	3.972932	2.524011	3.913127	3.609886	5.032953	2.480628	2.673087
Atf2	-0.741046	5.878204	-2.432086	0.028471	0.09881	-4.621344	6.346958	5.462754	6.263817	7.071319	5.847478	6.296743	5.33825	5.907888	5.622027	5.162339	6.021524	5.430392	6.187427	5.363965
Stx18	-0.400065	5.497771	-2.431944	0.028478	0.09881	-4.61264	5.95468	5.337533	5.705797	6.263067	5.845183	5.945771	5.172864	5.31699	5.24619	5.27332	5.437719	5.058407	5.298866	5.147901
Pex7	-0.663041	2.52483	-2.431864	0.028483	0.09881	-4.25501	2.853968	1.934482	2.31067	3.533136	2.901447	3.038068	2.433388	2.307359	2.47808	2.221154	2.346454	2.529342	2.168911	2.570827
Ccdc135	2.132875	1.402798	2.431423	0.028507	0.098866	-3.659309	0.739674	1.480065	1.089503	-1.388744	2.240661	1.563674	2.144252	2.619301	1.393785	0.055728	1.740817	2.027776	2.66462	2.142093
Klf15	-0.373762	4.469078	-2.431076	0.028526	0.098904	-4.531753	5.014852	4.560742	4.397084	4.619139	4.439837	4.370872	4.494157	4.155962	4.241012	4.585342	4.473897	4.555878	4.442376	4.287263
Ralgps2	-0.83864	3.498808	-2.430554	0.028555	0.098976	-4.424384	3.460552	3.000943	3.525794	4.886126	2.995506	3.884005	3.537848	3.395791	3.742206	2.843096	3.512491	3.16218	3.944552	3.237699
Mapk12	0.80293	3.040362	2.430397	0.028564	0.098978	-4.212759	2.810225	3.69053	3.34223	2.241914	3.359399	2.863579	3.356296	3.000548	3.027678	3.52358	2.921517	3.127617	2.220182	3.278825
Nfrkb	0.644763	7.115121	2.430127	0.028579	0.099001	-4.625646	6.36029	7.588047	7.441078	6.438749	6.565851	6.581508	7.6497	7.170656	7.472312	7.354201	6.914135	7.307958	7.153251	7.625937
Zfp641	0.348985	3.821149	2.429264	0.028627	0.099095	-4.409891	3.652372	4.095404	3.948948	3.659984	4.043216	3.935331	3.889373	3.798099	3.709451	3.978769	3.673224	3.906064	3.589113	3.728093
Tssc4	0.483651	5.31276	2.429248	0.028628	0.099095	-4.56611	5.02206	5.89583	5.282373	4.792905	5.400866	4.988255	5.151787	5.655839	5.190313	5.815669	5.624113	5.53465	4.934548	5.130032
Pptc7	-0.408613	6.208718	-2.429195	0.028631	0.099095	-4.6238	6.200041	5.645011	6.067974	6.549765	6.002771	6.149669	6.356102	6.221957	6.212998	6.168464	6.418445	6.182542	6.603289	6.16455
Csdc2	-0.446747	6.467145	-2.428335	0.028679	0.099197	-4.628271	6.908147	6.298294	5.974492	6.221636	6.290258	5.909869	6.638661	6.520978	6.387753	6.767894	6.581905	6.631131	6.656994	6.770241
Iqsec2	-0.425298	8.164782	-2.428176	0.028688	0.099197	-4.610416	8.369786	7.740049	8.257394	8.365817	7.589256	8.188963	8.278378	8.318789	8.250421	8.074523	8.052095	8.342283	8.278231	8.206521
Acyp2	-0.569596	4.830168	-2.428106	0.028691	0.099197	-4.572143	5.210525	4.051149	4.725329	5.384868	4.870545	5.250499	4.906476	4.761243	4.975708	4.5783	4.882375	4.600669	4.893023	4.588148
Myo9b	0.551496	7.872015	2.427882	0.028704	0.099197	-4.629577	7.098039	8.200366	7.52082	7.110482	7.497615	7.397656	8.405135	8.308254	8.181933	8.40364	7.888253	8.252404	7.705055	8.245683
Spta1	1.409782	1.822618	2.427826	0.028707	0.099197	-3.846333	0.643243	1.972946	1.631973	0.705659	1.981199	2.602913	1.809437	3.301815	1.426399	1.652245	1.533025	2.931181	2.041677	1.779078
Laptm5	0.597073	6.158241	2.427551	0.028722	0.099197	-4.606228	5.917798	5.844892	6.031841	5.042128	6.395951	6.005026	5.22677	7.2552	5.405997	7.097109	7.114647	7.739903	5.396821	5.766569
Rpp40	0.41267	2.822756	2.42755	0.028722	0.099197	-4.285308	2.937667	3.492948	3.277465	2.968937	3.313654	3.384299	2.841442	2.709903	2.39812	2.529327	2.696109	2.447635	2.301782	2.446395
Htr4	-0.957497	1.415196	-2.42731	0.028736	0.099197	-3.865346	0.915118	0.480615	0.914692	1.962578	0.15195	0.61372	1.938115	2.207232	2.539002	1.755735	2.100543	1.643459	1.700256	1.556352
Lipe	0.845793	4.764257	2.42722	0.028741	0.099197	-4.52275	4.883821	5.908288	5.138943	3.795925	5.254629	4.389395	4.92212	4.420274	4.71693	4.894014	4.493172	4.606955	4.474404	4.861554
Lilrb3	3.100886	0.958284	2.427185	0.028743	0.099197	-3.681855	0.485316	3.06466	3.184186	-1.223791	2.295481	1.519512	-0.169545	3.567159	-1.536754	0.055728	0.263333	3.018661	-0.211665	0.499386
Aak1	-0.661589	8.779903	-2.426883	0.02876	0.099213	-4.589353	8.825965	8.138789	8.881846	9.561932	8.336159	8.770298	8.830185	8.915443	8.713413	8.33663	8.972771	8.588699	9.37143	8.678763
Npy5r	-1.09068	2.491796	-2.426806	0.028764	0.099213	-4.145798	2.127257	0.812964	1.089503	3.468286	2.732935	2.386402	2.918339	2.571773	3.253503	2.694725	2.506778	2.252925	3.160849	3.237699
Ccdc65	-0.446746	5.181783	-2.426329	0.028791	0.099276	-4.59146	5.566568	5.053348	4.634216	5.376859	5.33206	5.089903	5.469678	5.149037	5.232422	5.26018	5.331071	4.906142	5.06414	5.123274
Eif3l	0.401177	6.221205	2.425363	0.028845	0.099408	-4.628553	6.246939	6.227479	6.500191	5.797405	6.7623	6.195124	6.20571	6.202584	6.091152	6.239367	6.216781	6.162408	6.03231	6.238336
Nap1l2	-0.929118	5.944922	-2.425349	0.028845	0.099408	-4.630476	6.105938	4.619507	5.738974	7.167302	5.946075	6.515587	5.872101	5.895072	6.069375	5.485779	5.96742	5.720069	6.285965	5.867076
Cenpm	0.682255	1.335841	2.424923	0.028869	0.099462	-3.977858	1.957182	2.21681	1.966737	0.896996	2.139408	1.943815	0.68572	1.318383	1.102347	0.938085	1.319276	1.11804	0.698429	1.048408
Dlgap3	-0.460329	8.835881	-2.424569	0.028889	0.099502	-4.596147	9.357381	8.299935	8.697516	8.753423	8.532036	8.866126	9.037945	8.72423	8.893518	9.101359	8.669358	8.915408	8.855198	9.002691
Prrt1	-0.764433	9.274287	-2.424146	0.028913	0.099555	-4.56335	9.756542	8.402571	9.23251	10.43056	9.820338	9.862309	8.844828	9.190834	9.059311	9.102893	9.159789	9.066906	8.911522	9.00177
Celf5	-0.367837	9.784637	-2.423679	0.028939	0.099592	-4.562979	9.798861	9.497356	9.742682	10.19928	9.520462	9.761412	9.972907	9.777184	9.990367	9.77383	9.64523	9.648112	9.66757	9.991454
C8g	0.884278	2.35754	2.42366	0.02894	0.099592	-3.99647	1.874641	2.747223	2.654608	1.033666	2.16907	1.627481	2.943086	2.781287	2.759824	2.733273	2.428842	2.643831	2.423441	2.516844
Alg12	0.479528	4.505149	2.423166	0.028968	0.099659	-4.497738	4.315816	4.861671	4.57175	3.963576	4.772505	4.254448	4.599099	4.429191	4.621564	4.680472	4.421201	4.465359	4.310591	4.875027
Ddx21	0.565998	4.112363	2.423016	0.028976	0.099659	-4.483125	3.664706	4.167656	4.903672	4.351348	4.312723	4.921945	4.017238	3.942114	3.878097	3.879152	3.807603	3.946285	4.075609	3.797658
Pom121	-0.447137	7.410132	-2.421897	0.029039	0.099847	-4.638415	7.36977	6.814091	7.418729	7.90357	7.258152	7.266024	7.242565	7.501256	7.476698	7.349556	7.416303	7.50512	7.711788	7.517577
Angpt1	-1.049982	1.980652	-2.421563	0.029058	0.099865	-4.158265	2.896425	1.047298	1.033553	2.718219	1.947363	3.061358	2.343298	1.429209	1.489496	1.755735	1.456531	2.375822	2.522078	2.087586
Alkbh3	0.488809	4.980771	2.42151	0.029061	0.099865	-4.566852	5.005184	5.608829	5.241285	4.488478	5.086061	4.988255	5.178086	4.90241	4.989416	5.092395	4.911322	4.698109	4.656042	4.935431
Atl2	-0.37891	4.996104	-2.420828	0.0291	0.099969	-4.58203	5.09451	5.053348	4.895848	5.256321	4.484758	4.767664	5.236813	5.178422	5.137119	4.856672	4.786679	4.93141	5.157891	5.147901
Arhgef11	-0.294035	7.042216	-2.420352	0.029127	0.100016	-4.647132	6.941477	6.881355	6.638481	6.969025	6.490026	6.636817	7.457497	7.149903	7.353322	7.217562	7.174078	7.065895	7.305423	7.322409
Mospd3	0.371274	5.301337	2.420184	0.029136	0.100016	-4.61347	5.683317	5.752562	5.822858	5.109403	5.848624	5.626016	4.906476	5.225253	4.922398	5.279845	5.287575	4.958703	4.906128	4.930278
Fktn	-0.464116	5.344888	-2.420145	0.029138	0.100016	-4.605292	5.417836	4.943845	5.386545	5.618792	4.725866	5.158103	5.37963	5.326567	5.471927	5.131403	5.407561	5.50129	5.92952	5.46898
Rufy3	-0.418323	6.736769	-2.418766	0.029216	0.100255	-4.649861	6.777948	6.466954	6.64841	7.370111	6.570026	6.9367	6.740997	6.739258	6.748542	6.377603	6.69928	6.592052	6.919909	6.741844
Faah	-0.466407	4.920184	-2.418053	0.029257	0.100365	-4.537224	4.830077	4.010125	4.566831	4.53528	4.451953	3.808019	5.430835	5.273089	5.41415	5.14338	5.225542	5.194039	5.381888	5.674029
Mthfd1l	0.379169	5.165144	2.417497	0.029288	0.100445	-4.581142	4.859884	5.440624	5.396283	4.847241	5.137606	4.955481	5.34058	5.338449	5.347591	5.16704	5.099154	5.119046	5.274818	5.032408
Ache	-0.579318	6.300649	-2.417135	0.029309	0.100473	-4.641545	6.044731	5.028455	5.601424	6.4812	5.893738	6.206485	6.765486	6.354253	6.81163	6.585907	6.350494	6.47578	6.652335	6.97867
Ghdc	0.659911	3.866351	2.417057	0.029313	0.100473	-4.402635	3.816137	4.528825	4.068613	3.019194	4.128728	3.524966	3.882992	3.871902	3.930199	4.130887	3.684447	3.900956	3.640205	4.111741
Rgs8	-1.095054	6.053077	-2.416894	0.029323	0.100476	-4.647336	6.434199	4.294561	6.167186	7.224203	5.960989	6.492213	5.886526	6.30181	5.854008	5.655958	6.361024	5.969758	6.486868	5.680182
Ttc21b	0.497149	4.125647	2.416057	0.02937	0.100585	-4.487061	3.968177	4.965467	4.266652	3.950685	4.368121	4.247696	3.837517	4.213873	4.18493	3.957219	3.862983	3.727169	4.125768	4.174106
Bst2	1.014272	3.514003	2.416043	0.029371	0.100585	-4.278631	3.460552	3.588941	2.829082	1.910273	4.325951	3.901317	2.880403	4.406794	2.894141	4.421893	4.444493	4.781067	2.689088	2.825198
Hif1an	-0.658187	3.310752	-2.413494	0.029517	0.101055	-4.43822	3.748215	2.982209	3.34223	4.235668	3.020129	4.009049	3.251219	2.964197	3.04878	2.807409	3.074378	3.35379	3.754753	2.921834
Lox	2.807749	0.801393	2.413163	0.029536	0.101091	-3.793289	0.369528	0.812964	3.688616	-0.166914	2.014259	4.467019	0.282279	0.449407	0.512061	0.20947	-0.616402	0.007173	0.182287	0.190106
Tinagl1	1.165361	4.223069	2.412214	0.02959	0.101248	-4.513755	4.990561	5.871858	5.44669	2.994284	4.974995	4.188991	3.945565	3.591194	3.722643	4.21501	4.376922	3.767014	3.318158	3.814538
Gpd2	-0.477347	5.139752	-2.41188	0.029609	0.101285	-4.598758	5.043471	4.449211	5.04125	5.396005	4.478849	4.996333	5.246787	5.445606	5.442331	5.06243	5.323912	5.179349	5.642129	5.25483
Mbtps2	-0.952019	3.143156	-2.411164	0.02965	0.101388	-4.348973	3.171726	1.453108	3.084457	4.04669	2.856578	3.221545	2.787802	3.19084	3.416608	2.923085	3.50618	3.18757	3.743115	3.610591
Slc6a15	-0.615266	6.467451	-2.410982	0.029661	0.101388	-4.655643	5.985736	5.082224	6.105771	6.813956	5.818526	6.126853	6.843963	6.659091	6.969352	6.65279	6.690261	6.728765	7.043073	7.043345
Col18a1	0.65711	4.944201	2.410913	0.029665	0.101388	-4.562812	4.935636	4.882026	5.032356	4.005731	5.089943	5.441593	5.185883	4.836907	5.315302	5.217764	4.730939	4.941394	4.460257	5.195929
Id2	0.582569	5.854337	2.409941	0.029721	0.101551	-4.658956	6.001624	6.748396	7.420434	6.205449	6.214048	6.361501	5.290835	5.573776	5.134654	5.524759	5.427736	5.547781	5.366799	5.172115
RGD1359290	0.7907	0.76499	2.409274	0.029759	0.101641	-3.950358	1.212615	1.558038	1.669168	1.065902	2.295481	2.086865	-0.067151	0.449407	0.317129	-0.537962	0.631286	0.151783	0.111637	0.799858
Tnfaip8	-0.594021	3.980242	-2.40919	0.029764	0.101641	-4.591265	4.611755	4.354059	4.337679	5.613349	4.662107	4.760567	2.979429	3.559057	3.761508	3.538169	3.760656	3.195935	3.529386	3.169358
Ankrd34b	-0.763831	3.482183	-2.408856	0.029783	0.101678	-4.478141	3.439172	3.790827	3.458336	5.003121	3.500244	3.221545	3.419386	2.835955	3.375441	3.139454	3.408041	3.092205	3.817137	3.395603
Atpaf1	-0.428455	5.489671	-2.407944	0.029836	0.101828	-4.650674	5.7427	5.204449	5.6661	6.14826	5.532208	5.664547	5.47393	5.230363	5.399851	5.131403	5.476975	5.198209	5.775222	5.246561
Mpc1	-0.414334	6.421362	-2.407801	0.029844	0.101828	-4.669849	7.101446	6.287843	6.656536	7.145785	7.045236	6.84741	6.276793	5.907888	6.158224	6.101282	6.221604	5.991545	6.127142	6.049248
Fzd5	0.953265	0.386025	2.407094	0.029885	0.101939	-3.719105	0.105685	1.279848	0.715742	-0.243853	0.371962	0.949514	0.028455	0.923327	-0.080923	0.134646	0.631286	0.760186	0.491415	0.554014
Arl5b	-0.983616	3.03448	-2.406088	0.029943	0.102108	-4.434176	3.536344	1.914859	3.555895	4.549599	3.052318	3.68846	2.814871	2.471744	2.583064	2.256873	3.141068	2.715403	3.689548	2.721621
Brms1l	-0.713135	4.121097	-2.405651	0.029969	0.102166	-4.568824	4.380808	3.909605	4.162901	5.349294	3.716556	4.850213	3.857182	4.12885	4.101553	3.493953	4.073974	3.750073	4.233667	3.780579
Tmem169	-0.411925	4.285472	-2.404646	0.030027	0.102306	-4.535712	4.172016	3.7962	3.963992	4.706082	4.047215	4.326706	4.555628	4.224157	4.500423	4.31582	4.295089	4.224961	4.595845	4.35421
Raver1	0.347819	6.710192	2.404575	0.030031	0.102306	-4.673996	6.711879	7.076682	6.892051	6.358062	6.908497	6.650959	6.670502	6.712114	6.710149	6.818072	6.713045	6.598376	6.447968	6.689429

Slc29a4	-0.585412	4.941583	-2.404507	0.030035	0.102306	-4.604986	5.687857	4.582672	4.648255	4.667211	4.352047	4.753435	4.704179	5.170467	5.119774	5.511883	5.136409	5.247329	4.553161	5.100515
Epb41l1	-0.307948	9.779637	-2.404202	0.030053	0.102306	-4.599225	9.983045	9.843245	9.56306	9.939575	9.562191	9.642025	10.01437	9.576214	9.877782	9.760186	9.601496	9.684421	9.835303	10.03381
Ttl	-0.593731	4.458827	-2.404045	0.030062	0.102306	-4.597674	4.990561	4.010125	4.671355	5.179216	4.36492	4.902733	4.096591	4.402272	4.203867	3.940842	4.527854	4.433879	4.81457	3.958175
Limch1	-0.418627	7.204905	-2.403887	0.030071	0.102306	-4.672258	7.440071	7.078888	7.180298	7.716023	6.878512	7.499215	7.015658	7.360925	7.091955	7.075394	6.980515	7.27231	7.317244	6.972416
Ndfip1	-0.532337	8.330175	-2.403814	0.030076	0.102306	-4.659539	8.097206	6.99435	7.870111	8.346264	7.885709	8.012507	8.663821	8.45054	8.72534	8.711217	8.550568	8.677621	8.81007	8.832381
Ednra	0.707839	2.960592	2.403675	0.030084	0.102306	-4.2355	2.595092	2.631765	2.947351	2.255954	3.428477	3.432728	2.854546	3.180315	3.006262	2.966878	2.580719	3.489274	3.134365	3.151754
Vav2	0.405626	4.81223	2.403629	0.030086	0.102306	-4.541778	4.247758	4.817439	4.462108	4.196165	4.760377	4.475713	5.114144	5.199424	4.778229	5.277674	5.017197	5.162375	4.754973	5.165551
Clvs2	-1.175653	2.286496	-2.403293	0.030106	0.102343	-4.207575	2.43149	1.151583	2.445413	3.456176	0.731352	2.809587	2.078759	2.753156	2.772565	1.755735	2.833517	2.220493	2.84965	2.087586
Ankrd29	-0.534163	4.309416	-2.402299	0.030164	0.102458	-4.582511	4.592516	4.435504	3.797607	5.122873	4.633136	4.484354	4.207992	3.741948	4.12658	4.091865	4.376922	4.196062	4.330171	4.275119
Nek9	0.310341	6.299503	2.402279	0.030165	0.102458	-4.670096	6.132823	6.492828	6.37774	5.886966	6.298669	6.108337	6.377821	6.336588	6.262385	6.359234	6.463931	6.441014	6.263737	6.411048
Syngr2	0.627675	4.585475	2.402277	0.030165	0.102458	-4.545792	4.748409	4.777251	4.753743	3.633342	5.012294	4.649696	3.969854	4.866752	4.034376	4.874027	5.021628	5.544509	4.056929	4.323092
Hbb-b1	0.88609	2.910685	2.401712	0.030198	0.102541	-4.440444	3.793849	4.180039	4.188746	3.067759	5.13573	3.683465	2.247205	2.077116	2.95129	2.06872	2.197736	1.152831	1.79348	2.474989
B3galt2	-0.729055	5.874214	-2.401402	0.030216	0.102574	-4.665438	5.568211	4.841025	5.34405	6.788826	5.642047	5.972635	5.911821	5.738874	6.257862	5.815669	5.972007	5.831641	6.144941	6.437623
Mgat4a	-0.458039	4.425243	-2.400877	0.030247	0.102649	-4.537218	4.304037	4.077865	4.254466	4.529512	3.886882	3.632543	4.855242	4.446862	4.680213	4.294496	4.692545	4.738754	4.999576	4.636413
Gins1	1.714865	0.320985	2.400142	0.03029	0.102766	-3.79344	0.307952	2.47979	0.20995	-0.093872	2.564599	1.927009	0.118118	0.222004	-0.773548	0.134646	-0.149817	-0.070959	-0.405292	-0.505482
Ndufb10	-0.361095	7.139586	-2.39985	0.030307	0.102796	-4.678284	7.632888	7.083839	7.089323	7.643446	7.555613	7.380012	6.945436	6.905836	7.087501	7.022551	7.101624	6.78252	6.898429	6.836473
Tmeff2	-0.777359	6.595872	-2.399041	0.030355	0.102928	-4.683115	6.40876	5.136182	6.243153	7.266428	6.287725	6.569375	6.522363	6.760611	6.854205	6.7733	6.48975	7.005907	7.028765	7.013199
Tbpl1	-0.385579	5.349858	-2.398476	0.030388	0.103011	-4.639505	5.272282	4.758107	5.069347	5.542091	5.101527	5.159902	5.804495	5.250627	5.548002	5.414429	5.674232	5.261346	5.504144	5.576794
Gpr135	-0.772884	2.408269	-2.398305	0.030398	0.103016	-4.201577	2.529164	0.895377	2.067688	2.862845	2.124346	2.592292	2.598267	2.436799	2.917273	2.558244	2.415434	2.680061	2.838755	2.516844
Esf1	0.329362	5.491942	2.398137	0.030408	0.103021	-4.663745	5.466203	5.693323	6.052222	5.732361	5.9726	5.998002	5.175477	5.423464	5.232422	5.178725	5.39566	5.287022	5.27077	5.044382
Ablim2	-0.381883	6.444723	-2.397766	0.03043	0.103066	-4.682384	6.37068	6.397954	6.170433	6.4812	5.921234	5.692163	6.749791	6.610919	6.83003	6.569134	6.547745	6.476639	6.661638	6.765185
LOC689986	-0.48815	6.356168	-2.39745	0.030449	0.10308	-4.685917	6.256138	6.363986	6.29464	7.080717	5.951419	6.117625	6.500646	6.458107	6.365849	6.056381	6.550048	6.252381	6.4825	6.275385
Plxnd1	0.645676	5.944198	2.397401	0.030452	0.10308	-4.655896	5.983276	6.335671	5.88346	4.751386	5.956744	5.837519	6.069049	6.141498	6.079664	6.270281	6.056497	6.275258	5.495499	6.109761
Abhd17c	-0.548881	5.0768	-2.397107	0.030469	0.10311	-4.62454	5.089944	3.958345	4.790075	5.38167	4.840905	5.141808	5.268979	5.207221	5.13958	5.275498	5.385381	5.164508	5.202297	5.277328
Nxph3	-0.831407	3.384792	-2.395423	0.030568	0.103418	-4.580406	4.611755	4.749824	3.633224	5.082081	3.726565	4.044542	2.433388	1.239467	2.568526	3.080873	2.949696	3.204252	3.245776	3.002614
Nell1	-0.779514	6.222614	-2.394214	0.03064	0.103604	-4.663161	5.88259	3.972652	5.194214	5.798604	5.834811	5.214594	6.957633	6.633278	7.244356	6.710282	6.648607	6.617184	7.01975	7.415245
Tifab	0.545482	3.059966	2.393967	0.030655	0.103604	-4.235714	2.787845	2.904741	2.663852	1.9796	3.137307	2.915623	2.286414	4.532109	2.241976	4.349296	4.284065	4.447955	2.220182	2.310209
Rnf150	-0.748732	3.928293	-2.393882	0.03066	0.103604	-4.481853	3.522857	3.028596	3.409716	4.627263	2.987204	3.939526	3.951676	4.536242	4.14139	3.861859	4.380377	4.241218	4.47088	4.007943
Ramp2	0.56868	5.437046	2.393852	0.030661	0.103604	-4.646247	5.360227	5.693323	5.519781	4.849557	6.236111	5.226661	5.193638	5.314586	5.525598	5.519255	5.204257	5.37253	5.428048	5.71206
Mak16	0.314971	4.580241	2.393681	0.030672	0.103604	-4.604334	4.529842	4.859106	5.039476	4.7588	4.938861	5.002363	4.306644	4.548571	4.441807	4.417954	4.404331	4.191886	4.438773	4.311247
Pbx1	-0.62108	5.322337	-2.393503	0.030682	0.103604	-4.680733	5.810876	5.29072	5.660338	6.481947	5.161783	5.989933	4.958988	5.107976	4.827903	4.647212	5.250298	4.992709	5.541578	4.831464
Dfnb31	-0.62861	6.27194	-2.393478	0.030684	0.103604	-4.692244	6.792072	6.897067	5.757442	6.542624	5.855482	5.656923	6.128247	6.268659	6.253324	6.379629	6.289327	6.584108	6.021511	6.40177
Nxph4	1.006085	1.754354	2.39261	0.030735	0.103748	-4.118651	2.342161	2.582864	2.175023	1.128291	2.800826	3.214637	1.544223	0.645799	1.520039	1.652245	1.403186	0.081289	1.700256	2.310209
Iqsec3	-0.304021	8.992199	-2.392021	0.03077	0.103838	-4.650654	9.206181	8.973595	8.869789	9.094362	8.753785	8.788439	9.279199	8.807591	8.971043	8.964941	8.826258	8.939287	9.249994	9.169426
Grin2b	-0.908236	4.702235	-2.391605	0.030795	0.103892	-4.591838	4.17633	3.512664	4.287732	5.690329	3.808949	4.518413	4.77481	5.416754	4.824848	4.643844	5.090743	4.948838	5.526381	4.676974
Ipcef1	-0.983266	1.538873	-2.391333	0.030811	0.103918	-4.139802	2.072763	1.506527	1.472789	2.77782	0.649545	2.297664	1.21902	1.429209	1.255413	0.893396	1.883989	0.969826	2.466543	1.227132
Pnkd	-0.382538	8.350334	-2.391097	0.030825	0.103936	-4.671379	8.612552	7.79106	8.257394	8.57102	8.386544	8.408037	8.299495	8.324476	8.338742	8.221017	8.474014	8.381315	8.470469	8.373396
Mycbp2	-0.485949	8.411634	-2.390255	0.030876	0.104074	-4.682419	7.824065	7.947428	7.745863	8.741155	7.53796	7.943155	9.063035	8.750704	8.874622	8.635434	8.560691	8.56891	8.728498	8.846298
Rell2	-0.356191	4.649118	-2.390123	0.030883	0.104074	-4.595643	4.830077	4.528825	4.399853	4.580611	4.445908	4.024932	4.884284	4.67015	4.771898	4.797268	4.706374	4.738754	4.954871	4.817576
Pwp2	0.317746	5.15494	2.389616	0.030914	0.104147	-4.629322	4.82184	5.12555	5.393507	4.854179	5.074352	5.02228	5.335916	5.312177	5.192685	5.249138	5.269057	5.121244	5.14817	5.293472
Ptgs1	-0.62554	3.695381	-2.389	0.030951	0.104227	-4.406276	3.865059	3.317238	2.787402	3.247155	2.874693	2.753495	3.96382	4.332691	3.503242	4.259708	4.184818	4.835621	3.805994	4.138799
Gabra3	-0.596717	4.319541	-2.388911	0.030956	0.104227	-4.61656	4.920286	4.335728	4.664464	5.056238	4.109733	4.455345	3.569982	3.858761	4.461611	3.838474	3.867914	3.864689	4.844833	4.707418
Rtn1	-0.523811	10.59312	-2.388781	0.030964	0.104227	-4.596928	10.63961	9.772349	10.32883	11.06018	10.57119	10.63321	10.5807	10.46811	10.82841	10.68645	10.46136	10.64645	10.64591	10.98195
App	-0.480072	10.59743	-2.388394	0.030987	0.104266	-4.612683	10.27951	9.421923	10.05328	10.58418	10.13175	10.2043	10.9254	11.02851	11.16964	10.80164	10.6701	10.90598	10.98991	11.19896
Cd244	0.836665	1.749529	2.388297	0.030993	0.104266	-4.068995	1.178614	2.309899	2.654608	1.818649	1.912714	2.434761	1.258992	2.227821	1.326257	1.180545	1.978199	2.203999	1.700256	0.799858
Fbll1	-0.405745	5.360591	-2.388082	0.031006	0.104281	-4.674826	5.733947	4.909553	5.422387	5.625567	5.369271	5.382721	5.361385	5.191584	5.489397	5.208673	5.591695	5.140873	5.492026	5.167742
Pcdh20	-1.186476	6.526629	-2.387779	0.031024	0.104312	-4.698426	6.407843	3.848855	5.740067	7.170549	6.233481	6.560549	7.115651	6.968348	7.014296	6.689195	6.771443	6.695229	7.133885	7.04573
Nrcam	-0.536178	9.763509	-2.387577	0.031036	0.104324	-4.636493	9.375199	8.944857	9.36318	10.31691	9.240269	9.681937	10.03385	9.949973	10.12515	9.711842	9.787986	9.890197	10.15788	10.11178
Gdap1l1	-0.373329	6.106071	-2.386607	0.031094	0.104491	-4.700715	6.280387	5.653916	6.091285	6.344985	6.122259	5.888325	6.07466	6.243603	6.292553	6.052575	6.260557	5.852956	6.267804	6.082147
Megf10	0.608896	4.644708	2.386336	0.03111	0.104516	-4.582305	4.284188	4.774532	5.471906	4.221428	4.641095	4.529589	4.43432	4.879821	4.60741	4.320048	4.467414	4.9879	4.608729	4.861554
Slc30a7	0.594279	3.143392	2.386124	0.031123	0.10453	-4.390617	2.917193	3.159427	3.948948	3.211576	3.596165	3.888353	2.613764	3.012464	2.694377	2.720538	2.977336	3.397965	3.160849	2.890335
Nptxr	-0.829981	11.2753	-2.38593	0.031135	0.10454	-4.582386	11.42681	9.500034	10.87807	11.50411	10.87917	11.30435	11.34242	11.50552	11.62707	11.33185	11.4541	11.52943	11.85629	11.71568
Lppr4	-1.314831	8.886699	-2.385264	0.031175	0.104641	-4.672087	8.631528	6.057805	8.201761	9.695503	8.113027	9.026339	9.286017	9.44923	9.460893	9.168244	9.240064	9.202945	9.499626	9.385342
Plscr2	0.924157	1.387212	2.385142	0.031182	0.104641	-3.995828	1.787089	1.632013	2.067688	0.485009	2.212449	2.025031	1.668148	1.356279	1.520039	1.419918	1.03357	0.28321	0.962073	1.608903
Frs2	-0.384315	6.426776	-2.384761	0.031205	0.104689	-4.709067	6.667367	6.001938	6.41037	6.582863	6.00996	6.53928	6.553835	6.290844	6.408308	6.330188	6.301223	6.50721	6.738382	6.651571
Abr	-0.380658	9.650566	-2.384528	0.031219	0.104707	-4.646436	9.532377	9.035101	9.554466	9.81612	9.076774	9.51089	9.845998	9.883097	9.787984	9.797329	9.855403	9.78739	9.870914	9.756077
Pfkfb2	-0.381644	5.187585	-2.383828	0.031262	0.104819	-4.651012	5.085364	4.977341	4.996223	5.307775	4.460974	4.836779	5.617289	5.443407	5.399851	5.244698	5.314913	5.265326	5.274818	5.445693
Ccnl1	0.548483	6.350089	2.3836	0.031275	0.104836	-4.703405	5.827428	6.921242	6.450315	6.053974	6.452073	6.136937	6.73481	6.472197	6.36795	6.61802	6.151556	6.338017	5.895753	6.500691
Mtx1	-0.308638	6.405945	-2.38332	0.031292	0.104864	-4.708748	6.331568	5.806804	6.205669	6.466937	6.202441	6.14695	6.548805	6.513661	6.692659	6.514849	6.475284	6.589673	6.545834	6.660934
Sardh	0.662876	3.775573	2.382345	0.031351	0.105009	-4.480914	4.087502	4.515857	3.926082	2.925676	4.202298	3.94371	3.562015	3.282262	3.648551	3.784431	3.518775	3.926315	3.689548	3.96323
Ruvbl2	0.38877	5.579472	2.382314	0.031353	0.105009	-4.674618	5.492389	5.786701	5.602627	5.023782	5.766114	5.41773	5.854271	5.599679	5.772315	5.732483	5.444337	5.458476	5.517869	5.677109
Flot2	0.376918	7.169573	2.381424	0.031407	0.105161	-4.713613	7.464529	7.587272	7.889694	7.06136	7.630486	7.455568	6.604164	6.917765	6.873553	6.956881	6.941873	7.01952	7.084013	6.898479
Fam83d	-0.621667	2.235607	-2.380902	0.031439	0.105207	-4.200422	2.43149	1.655851	1.574317	2.511387	2.361201	1.875381	1.862293	2.753156	1.636128	2.694725	2.604547	2.941167	2.466543	2.278116
Kcnk12	-0.65304	2.939579	-2.380764	0.031447	0.105207	-4.241949	2.445855	1.506527	2.262807	2.916866	2.448452	1.927009	3.251219	3.242343	3.577725	3.310669	3.461211	3.778199	3.707626	3.551406
Cps1	-0.833521	0.702005	-2.380631	0.031455	0.105207	-3.886314	1.108109	-0.186163	0.750864	1.097434	-0.038319	0.65513	0.625837	1.279465	0.884903	0.699359	0.885831	0.666985	0.921344	1.473753
Nucb2	0.375522	5.708311	2.380621	0.031456	0.105207	-4.690878	5.499292	5.723247	5.756362	5.488098	6.015073	5.978764	5.476051	5.81058	5.642993	6.052575	5.532373	5.904905	5.357288	5.709055
Ebpl	0.835609	2.796754	2.380224	0.03148	0.105258	-4.219848	2.658139	3.151065	2.225837	1.593908	3.273255	3.099357	2.534559	2.951874	2.47808	3.167876	3.031075	3.212521	3.02328	2.992761
Isyna1	0.65961	6.377498	2.379692	0.031513	0.105337	-4.70783	6.041187	7.025563	5.953916	5.616073	6.785226	6.182788	6.604164	6.34132	6.572127	6.72231	6.413395	6.44891	5.935923	6.661712
Hcn1	-0.79675	5.415616	-2.379533	0.031522	0.10534	-4.681275	5.117126	4.005493	5.115554	6.267405	5.086061	5.371895	5.446065	5.665305	5.727214	5.206391	5.566136	5.567256	5.984921	5.731448
Lmbrd2	-0.9881	4.671224	-2.379123	0.031547	0.105395	-4.628261	4.406741	3.418258	4.685039	5.818845	3.67581	4.71479	4.571588	4.863466	5.000289	4.173561	4.791878	4.792144	5.599199	4.953324
Gne	-0.322023	5.504071	-2.378746	0.03157	0.105422	-4.677085	5.403188	4.919435	5.062374	5.413335	5.362868	5.000356	5.900809	5.603623	6.000646	5.64083	5.660089	5.594403	5.799304	5.731448
Fbxl15	-0.485065	4.134338	-2.378699	0.031573	0.105422	-4.494938	4.28818	3.263946	3.468922	3.492206	3.403739	3.420773	4.61073	4.485848	4.723506	4.633691	4.730939	4.338875	4.659142	4.458122
Cdc42ep5	0.709396	1.315951	2.378502	0.031585	0.105433	-4.050157	1.741237	1.702379	1.966737	1.000693	2.484288	1.993092	0.903114	1.020936	0.837186	0.646479	1.167576	1.045835	1.150071	1.415936
RT1-CE14	1.305026	2.572571	2.378225	0.031602	0.10546	-4.070188	1.741237	2.631765	2.356997	0.932396	3.751288	1.668504	2.433388	2.939445	2.185932	3.636356	3.474203	3.292705	2.348615	2.932182
Strbp	-0.403001	6.635762	-2.377629	0.031638	0.105552	-4.72445	6.545102	6.586505	6.442951	7.230438	6.338372	6.574106	6.92617	6.604045	6.81163	6.495357	6.666404	6.376243	6.670114	6.649221
Slc1a1	-0.365993	6.654663	-2.377367	0.031654	0.105564	-4.716001	6.093446	5.829253	6.160671	6.387058	5.692706	5.816	7.190549	7.195041	7.39357	7.106929	6.851415	6.96489	7.157641	7.3434
Ccsap	-0.558864	5.131949	-2.377284	0.03166	0.105564	-4.676073	5.169999	4.613433	5.22264	5.719753	4.511054	5.198928	5.159727	5.589771	5.313124	5.089921	5.345284	5.03994	5.096513	4.823147
Rpl13a	0.629922	5.395899	2.377083	0.031672	0.105571	-4.704067	6.010107	6.794762	6.078381	5.192053	5.988284	6.042569	5.2443	4.673906	4.720222	5.357987	4.942039	4.772703	4.740412	5.025175
Gucy1a2	-1.10836	2.293161	-2.376964	0.031679	0.105571	-4.291062	2.488111	1.833582	2.466695	3.659984	0.88224	2.800388	2.144252	2.419003	2.462438	1.755735	2.66245	2.118577	3.079891	1.684306
Cdc25c	1.017012	2.010793	2.376788	0.03169	0.105578	-4.096724	1.514695	2.884706	3.049621	1.478901	1.415175	2.613455	1.938115	2.077116	1.891068	1.964563	1.844511	2.170435	2.186205	1.529341
Itga6	0.425992	6.869048	2.375874	0.031746	0.1057	-4.726935	6.71781	6.946831	7.026217	6.63684	7.062132	7.376921	6.594433	6.909026	6.545465	6.989758	6.742875	7.494139	6.654666	6.483202
Slc52a2	0.585906	3.46365	2.375728	0.031755	0.1057	-4.361184	3.342675	3.375827	3.209656	2.310787	3.617838	3.334188	3.504983	3.690957	3.70281	3.740952	3.56201	3.761389	3.542874	3.942904
Fam110b	-0.491969	5.204183	-2.375727	0.031755	0.1057	-4.693769	5.566568	4.604273	5.354078	5.738624	5.196404	5.475219	5.021324	5.268128	5.227804	5.140992	5.311298	4.968501	5.242117	4.786544
Polr1a	0.457106	5.787375	2.375616	0.031762	0.1057	-4.692687	5.296263	6.11804	6.078381	5.389651	5.596383	5.410191	6.077457	5.999045	6.066791	5.899977	5.71448	5.740228	5.749277	5.916125
Hsd17b11	-0.373378	6.176689	-2.374945	0.031803	0.105807	-4.718706	6.295338	5.752562	5.562416	6.043894	6.092576	5.779016	6.176005	6.30181	6.275871	6.567357	6.300311	6.593636	6.304891	6.450279
Med31	-0.379592	3.358142	-2.374388	0.031837	0.105892	-4.497001	3.859704	3.176008	3.313151	3.761892	3.67581	3.552622	3.231264	3.126505	3.159628	3.401235	3.288097	3.16218	3.32598	3.133933
Bin1	-0.331565	9.052793	-2.373772	0.031875	0.105989	-4.686513	9.067912	8.930423	8.503294	9.095583	8.872913	8.688523	9.273265	9.15869	9.246325	9.299944	9.023919	9.156421	9.099388	9.325511
Tmem63c	-0.45381	6.214198	-2.37342	0.031897	0.106031	-4.722199	5.869329	5.735883	6.163932	6.603599	5.575768	5.659469	6.571799	6.33184	6.708492	6.079006	6.158615	6.185695	6.657769	6.719613
Suv39h1	0.369481	3.67528	2.371356	0.032024	0.106426	-4.549116	3.782575	4.037603	4.343444	3.942027	4.162299	4.373976	3.137884	3.591194	3.31576	3.360772	3.387587	3.454835	3.556237	3.133933
Tmem150c	-0.521839	7.216223	-2.370635	0.032069	0.106544	-4.737246	7.029353	5.965443	6.756049	7.298153	6.975604	6.868469	7.382016	7.362672	7.632456	7.520469	7.384399	7.461987	7.653919	7.747381
Ralgapb	-0.351589	7.521187	-2.370057	0.032105	0.106634	-4.733416	7.302296	7.176364	7.302955	7.866017	7.109274	7.338116	7.718509	7.573919	7.61355	7.470479	7.735106	7.602327	7.783848	7.712534
Nol4	-0.731408	5.452944	-2.3696	0.032133	0.106698	-4.723076	5.784274	4.640568	5.499273	6.564648	5.490232	6.132362	5.286006	5.304929	5.271094	5.003199	5.457481	4.985489	5.689893	5.269187
Grm4	-0.645137	4.510307	-2.369332	0.03215	0.106724	-4.666447	5.010026	5.397089	4.32899	5.316175	4.312723	4.098075	4.494157	3.871902	4.429792	4.567674	4.316888	4.350161	4.246089	4.475916
St6galnac5	-0.860636	6.310665	-2.368892	0.032177	0.106757	-4.732246	6.235615	4.502772	5.90895	6.847955	5.978894	6.317848	6.188995	6.583223	6.622259	6.393736	6.771443	6.666173	6.851099	6.502428
Snx7	-0.684435	3.889189	-2.368864	0.032179	0.106757	-4.624354	4.97826	3.240489	4.293698	4.538155	4.169655	4.524012	3.632178	3.526187	3.648551	3.377094	4.188764	3.516245	3.748946	3.178079
Zfp706	-0.41522	5.940024	-2.36865	0.032192	0.106757	-4.733727	6.366911	5.647985	6.021091	6.332618	5.929923	6.136937	5.816264	5.744244	5.861481	5.526589	6.14852	5.803178	6.129379	5.721041
Cnbd2	-0.660235	6.307076	-2.368598	0.032195	0.106757	-4.740546	6.780784	6.05108	6.5434	7.484836	6.249189	7.043864	5.952001	6.080544	6.007398	5.601432	6.271762	5.895948	6.531528	5.826046
Vopp1	-0.671254	7.091867	-2.368042	0.03223	0.106821	-4.737643	7.365998	6.28211	6.804913	7.862293	6.897464	7.784267	6.896384	7.016478	6.939298	7.022551	7.139835	7.040573	7.219317	7.026541
Gatad2b	-0.747315	4.59197	-2.368004	0.032232	0.106821	-4.668715	4.655673	4.046648	4.751577	5.771983	4.209453	4.872329	4.35355	4.820056	4.575048	3.957219	4.784073	4.581643	4.806206	4.169739
Txndc11	0.321397	5.537109	2.367456	0.032266	0.106889	-4.705253	5.423291	5.890816	5.613401	5.242285	5.623419	5.486734	5.342907	5.512163	5.534975	5.734068	5.55395	5.657847	5.406075	5.531571
Galnt6	0.817112	5.176538	2.367387	0.03227	0.106889	-4.64756	4.614937	6.038667	5.286867	4.367604	5.499017	4.410708	4.915882	5.615391	4.787674	5.597955	5.117902	5.889516	5.0524	5.323264
Neurod6	-1.483157	7.914189	-2.36644	0.03233	0.107055	-4.710752	8.769978	5.767682	7.213332	9.340279	8.390287	8.922458	7.819281	8.086646	7.82708	7.397349	8.164067	7.525176	8.301479	7.281513
Slc17a6	-1.176796	3.877802	-2.365505	0.032388	0.107219	-4.596166	3.417469	3.943895	3.370733	5.97129	3.482634	3.450478	3.837517	2.951874	3.799353	3.844356	3.901965	4.170823	4.028447	4.233886
Ns5atp9	2.22655	-1.183468	2.365284	0.032402	0.107235	-3.579806	-1.642648	0.769921	-1.223881	-1.575022	1.415175	0.61372	-1.21312	-0.992527	-0.929891	-0.420466	-1.500575	-1.925331	-1.638202	-2.228037
Btbd10	-0.545989	6.188274	-2.364398	0.032457	0.107389	-4.744886	6.493311	5.382875	6.276677	6.732828	6.226445	6.378599	6.229796	6.107182	6.270267	5.992929	6.425987	5.903629	6.226609	6.010707
Mgea5	-0.541735	6.612553	-2.36413	0.032474	0.107415	-4.748292	6.463617	6.156146	6.386141	7.291352	6.058313	6.743855	6.86192	6.758843	6.644955	6.297333	6.727359	6.618741	6.961306	6.622311
Fscn2	-1.061425	1.327148	-2.363639	0.032505	0.107487	-4.048521	2.569081	0.632508	1.430088	1.128291	0.15195	0.846031	1.135593	1.393205	1.102347	1.51148	2.544222	1.848377	1.493524	1.473753
Tubb5	0.334962	9.443271	2.362552	0.032573	0.107683	-4.681403	9.580063	9.694645	9.813825	9.555028	9.952739	10.15147	9.1075	9.108223	9.293775	9.079707	9.323753	9.172935	9.258322	9.116102
Prmt3	0.428073	4.324783	2.361619	0.032632	0.107848	-4.622683	3.997728	4.418186	4.792184	4.509141	4.720868	4.807235	4.447353	4.101219	4.023757	4.106622	4.192699	3.936334	4.456698	4.116286
Vegfb	-0.392587	5.707983	-2.36126	0.032654	0.107893	-4.736841	6.180847	5.763574	5.235097	5.739873	5.791366	5.486734	5.735337	5.63672	5.63952	5.916821	5.721307	5.738797	5.65616	5.699999
Crym	-0.630357	7.301874	-2.361116	0.032663	0.107893	-4.752598	7.181419	6.208464	6.816874	7.661688	7.497615	6.638754	7.695935	7.141789	7.999897	7.318729	7.593823	7.140923	7.646129	7.69479
Esam	0.901409	4.8784	2.36076	0.032686	0.107937	-4.630533	4.70428	5.812117	5.157058	3.727037	5.247695	4.199526	5.185883	4.548571	5.144489	5.190317	4.600749	4.906142	4.621498	5.309437
Aplnr	-1.096304	2.217667	-2.36047	0.032704	0.107943	-4.129827	2.843156	0.973336	0.442561	1.548999	1.20216	1.803538	2.828218	3.340143	2.127623	2.866406	2.544222	3.575147	2.494578	2.879681
Dcbld2	-0.493308	4.354359	-2.360449	0.032705	0.107943	-4.627693	4.391979	4.21255	4.43537	4.741441	3.649748	4.013036	4.51494	4.564846	4.325577	4.066928	4.383823	4.433879	4.780817	4.5246
Scarf2	1.637887	3.95577	2.359916	0.032739	0.108024	-4.578201	5.078466	6.701922	5.830061	2.363612	4.169655	4.495797	3.137884	3.449372	2.995434	3.861859	3.848089	3.397965	2.892424	3.286911
Zfp280d	-0.326782	6.177781	-2.35966	0.032755	0.108037	-4.753229	6.266292	6.144609	6.172053	6.738464	6.12699	6.262822	6.329365	5.985507	6.166694	6.039816	6.131194	5.858236	6.193853	6.094869
Car13	2.172031	-0.61838	2.359571	0.032761	0.108037	-3.74776	1.644914	1.082904	1.220561	-2.344749	1.082564	2.00915	-1.450335	-1.196868	-3.260591	0.134646	0.584127	-0.657145	-1.413173	-1.632384
Pcdhgb8	-0.773414	3.905429	-2.359365	0.032774	0.10805	-4.53853	3.84352	2.264105	3.883197	4.28785	3.252623	3.703341	3.908348	4.379449	4.329893	3.90752	4.176893	4.506292	4.306643	4.041787
Actg1	0.844076	1.806445	2.359066	0.032793	0.108083	-4.060857	1.48718	2.01041	1.841991	0.966949	2.308867	1.993092	2.746221	1.198329	1.959523	2.027955	1.783193	1.869448	1.493524	2.068949
Rars2	0.463505	3.892536	2.358454	0.032831	0.108181	-4.529073	3.481621	3.939046	4.235992	3.665254	4.140005	3.826262	4.192594	3.653399	4.236421	4.046664	3.633241	3.778199	3.671242	4.102607
Ndufaf1	-0.581273	3.216557	-2.35825	0.032844	0.108194	-4.487951	3.639931	2.439156	3.196978	3.721989	3.391209	3.340548	3.127125	3.282262	3.169295	2.831299	3.639021	3.10114	3.3415	2.982839
Skiv2l	0.330173	6.145635	2.357662	0.032882	0.108287	-4.740426	5.861314	6.120183	6.110849	5.485121	5.937483	5.835269	6.426897	6.276092	6.508814	6.39574	6.189469	6.304563	6.239092	6.370721
Sgpl1	0.547754	5.875444	2.357479	0.032893	0.108295	-4.722619	5.333436	6.319904	5.900178	5.217387	5.52075	5.551217	6.421404	5.835949	6.230419	6.125636	5.753634	6.126756	5.718405	6.228923
Crtc1	-0.409455	6.9655	-2.356808	0.032936	0.108406	-4.760232	7.429268	6.878825	7.362713	7.403606	6.779228	7.009851	6.794674	6.965286	6.865399	6.61802	6.811663	6.793586	7.170732	6.646866
Nceh1	-0.817497	6.007652	-2.356411	0.032961	0.108459	-4.747103	5.869329	4.332034	5.790521	6.59672	5.473985	6.042569	6.227279	6.133337	6.391887	5.74039	6.191437	6.219936	6.624853	6.499821
Fbln5	1.03746	2.172278	2.356092	0.032981	0.108466	-4.298395	2.718545	2.643736	2.607481	1.780306	4.031153	3.713177	2.305626	1.318383	1.891068	1.14286	1.803924	1.515634	1.28461	2.050068

Rps16	0.583789	6.634546	2.356034	0.032985	0.108466	-4.760513	6.857334	7.257967	6.667492	5.867665	6.961371	6.88814	6.563843	6.313897	6.568479	7.001649	6.572874	6.374401	6.351624	6.653136
Tmem18	-0.446219	3.656244	-2.35583	0.032998	0.108466	-4.538018	3.689063	3.33211	3.711083	4.066741	3.167012	3.53054	3.504983	3.698353	3.88398	3.478908	3.901965	3.662244	3.866246	3.820121
Tm4sf1	0.826083	3.307203	2.355689	0.033007	0.108466	-4.4131	3.197423	3.404252	3.118472	2.499634	4.237722	3.857641	3.537848	2.822481	3.472337	3.12019	3.116419	3.461789	2.805569	3.814538
RGD1311447	3.466705	-1.986408	2.355668	0.033008	0.108466	-3.464984	-2.659044	1.339943	-0.761158	-3.260591	-0.954037	-2.369898	-1.21312	-1.196868	-1.536754	-0.806258	-1.500575	-1.925331	-2.656794	-0.748265
Fam107a	-0.592732	9.879709	-2.355351	0.033028	0.108503	-4.669044	10.73874	10.57129	9.395791	10.3905	10.13991	9.770273	9.491609	9.487619	9.542892	9.921456	9.662537	9.928349	9.66603	9.610642
Dag1	-0.386414	7.80385	-2.354345	0.033092	0.108683	-4.749883	8.243911	7.505175	7.998954	8.077199	7.681545	7.917593	7.514853	7.788784	7.612222	7.57189	7.719506	8.002045	7.81545	7.811847
Nr3c2	-1.181972	7.350002	-2.353734	0.033131	0.108782	-4.760875	7.523706	4.892097	7.043583	8.093905	6.95127	7.610961	7.67254	7.786617	7.501305	7.37365	7.755204	7.551897	7.790578	7.364568
Ncdn	-0.616715	11.47226	-2.353486	0.033147	0.108789	-4.625425	11.77326	10.3642	11.50043	11.83208	11.42708	11.46808	11.32181	11.68667	11.54767	11.41172	11.72186	11.50713	11.66264	11.38761
Med14	-0.467874	6.71938	-2.353417	0.033152	0.108789	-4.768641	6.826102	6.034127	6.779625	7.194439	6.588666	6.765288	6.755042	6.885732	6.853456	6.438167	6.750237	6.531374	6.946793	6.737425
Sgk2	-1.083997	1.124273	-2.353062	0.033174	0.108833	-3.884181	1.108109	0.854759	-0.761158	0.74601	0.606834	-0.862697	1.938115	2.186344	1.743567	1.986005	1.652137	2.118577	1.521045	1.868007
Tdrp	-0.411257	4.630327	-2.352506	0.03321	0.108921	-4.673349	5.062241	4.557582	4.19832	4.572219	4.659497	4.209984	4.743453	4.415795	4.827903	4.626882	4.778847	4.555878	4.757867	4.922513
Fibp	0.310423	5.852116	2.351747	0.033259	0.10899	-4.757198	5.906157	6.10511	6.203294	5.775642	6.187119	6.105539	5.891303	5.770795	5.80524	5.713327	5.74562	5.480898	5.498963	5.768014
Xrcc6	0.420326	5.235414	2.351633	0.033266	0.10899	-4.72756	5.26014	6.061157	5.288362	5.155071	5.736702	5.432691	5.108686	4.949657	5.042981	5.157622	4.911322	5.044579	5.02142	5.167742
Insc	-0.855393	2.646447	-2.351521	0.033273	0.10899	-4.232215	2.555898	2.519311	0.945338	2.363612	2.09374	1.07687	3.180134	3.262441	3.216682	3.080873	2.771719	3.397965	3.333761	3.558103
Trmt2a	0.616326	5.497212	2.351509	0.033274	0.10899	-4.704489	5.005184	5.97495	5.313541	4.675069	5.589544	4.945084	5.875319	5.670955	5.817587	5.970246	5.468884	5.59282	5.233825	5.864377
Fuca2	-0.391872	4.997427	-2.35146	0.033277	0.10899	-4.717434	5.497569	4.551241	5.074555	5.115191	4.958104	5.049711	4.802122	4.998404	4.950701	4.938684	4.850346	5.177238	4.969927	5.07972
Zfp513	0.462581	6.220216	2.351207	0.033293	0.10899	-4.759787	5.991867	6.764246	6.372816	5.746104	6.197951	5.987909	6.26825	6.229634	6.397039	6.47465	6.237883	6.213771	5.889168	6.333144
Neurod1	-1.4546	4.559661	-2.351066	0.033302	0.10899	-4.697154	4.940716	1.894965	4.446125	6.217158	4.643738	5.435664	4.218166	4.803005	4.68359	4.361652	4.939699	4.502925	4.695828	4.14326
Eif1	0.33536	7.50691	2.351044	0.033304	0.10899	-4.763375	7.641105	8.048813	7.922556	7.512146	8.034883	7.6409	7.408913	7.193734	7.269853	7.37365	7.468769	7.139297	7.227171	7.223713
Lsm3	0.467705	4.230445	2.350294	0.033352	0.109118	-4.656696	4.28818	4.428602	4.680492	4.433945	5.130085	5.06899	4.107579	3.798099	4.081214	3.753509	4.111876	3.767014	3.989574	3.673625
Lypd6b	-0.833775	2.274872	-2.349956	0.033374	0.109159	-4.179521	1.936985	0.533043	1.340699	2.487785	1.840817	1.875381	3.003159	2.753156	2.446625	2.40749	2.544222	2.783591	3.278395	2.992761
Ubxn1	0.270024	7.031791	2.349132	0.033427	0.109303	-4.772915	7.353195	7.597695	7.535057	7.171012	7.555964	7.439734	6.628208	6.852971	6.744506	6.833679	6.760879	6.734519	6.516225	6.733732
Odf2l	0.671746	3.438464	2.348226	0.033485	0.109464	-4.401467	3.082606	3.904639	3.171281	2.427034	3.238702	3.353184	4.051782	3.070611	3.742206	3.918713	3.61576	3.397965	3.576053	3.739923
Dhx58	1.015665	2.730557	2.347345	0.033542	0.109599	-4.194332	2.035255	2.545069	3.091325	1.379803	2.901447	2.174914	2.905804	3.676051	2.185932	3.734632	3.099749	4.338875	2.151407	2.278116
Foxq1	-0.559698	2.991409	-2.347304	0.033544	0.109599	-4.367362	3.25566	2.425352	2.122353	2.666599	2.60856	2.446602	3.585784	2.976416	3.742206	3.432808	2.949696	3.212521	3.203938	3.461387
Olfml3	0.576547	2.782893	2.34716	0.033554	0.109599	-4.367839	3.188908	3.279374	2.947351	2.029493	3.144791	3.248852	2.380012	2.849305	2.508863	2.866406	2.86345	2.794648	2.508394	2.584013
Tbc1d10b	-0.297568	7.462133	-2.346844	0.033574	0.10961	-4.774284	7.539699	7.328263	7.157575	7.724252	7.29897	7.550865	7.467661	7.518593	7.559897	7.483258	7.533814	7.415736	7.536626	7.363612
Srgap2	-0.305791	6.350241	-2.346829	0.033575	0.10961	-4.772428	6.280387	5.96425	5.782056	6.25085	6.021185	6.074398	6.410354	6.896221	6.236179	6.686741	6.621127	6.918257	6.456901	6.324332
Gtf2f1	0.398854	7.610609	2.346054	0.033625	0.109744	-4.768358	7.720791	8.462512	8.185406	7.75238	8.012303	7.877698	7.357486	7.428077	7.423163	7.554056	7.20947	7.176758	7.051355	7.345337
Grin2a	-1.312157	6.179453	-2.345644	0.033652	0.1098	-4.760064	5.581282	4.086661	6.140948	7.180244	4.345567	5.664547	6.578725	7.271407	6.796114	5.983632	7.010365	6.451532	7.293005	6.156849
Flrt2	-0.728277	4.374674	-2.345413	0.033667	0.109819	-4.672386	4.633881	3.684748	4.217279	5.011421	3.61245	4.856883	4.358158	4.82682	4.272747	3.88487	4.421201	4.285003	4.924278	4.33484
Rom1	0.658066	3.546558	2.344592	0.03372	0.109963	-4.482769	3.556342	4.283127	3.871277	2.728325	3.794748	3.186669	3.479833	3.377479	3.408468	3.765957	3.684447	3.70975	3.372046	3.571405
Ampd2	-0.365838	6.979917	-2.344136	0.03375	0.11003	-4.783067	6.571545	6.081102	6.102375	6.71769	6.57834	6.355233	7.57695	7.352747	7.528319	7.425577	7.357043	7.295849	7.195496	7.594112
Mycn	-1.167535	2.21363	-2.343442	0.033795	0.110147	-4.231454	2.230432	-0.273517	1.408255	2.881078	2.322129	2.160607	2.186319	2.186344	2.625819	2.668442	2.802949	2.738492	3.079891	2.402413
Impg2	-1.411818	-0.012529	-2.343111	0.033816	0.110188	-3.825198	0.369528	0.118359	-0.411358	0.932396	0.15195	-2.070862	1.258992	0.04768	-0.27702	-0.027758	0.535373	-0.070959	0.375586	0.799858
Slc39a8	0.525225	2.95094	2.342569	0.033852	0.110266	-4.373658	2.8647	2.864389	3.013923	2.350586	3.137307	3.426763	3.241276	3.104407	2.797714	2.977621	3.022257	3.055902	2.639729	3.022122
Cwc27	0.329755	4.056493	2.342461	0.033859	0.110266	-4.636759	4.15463	4.393585	4.266652	4.082584	4.559454	4.568038	3.824257	4.17729	3.729193	3.989425	3.906764	4.04219	3.562873	3.629792
Klhl5	-0.345486	6.71277	-2.342166	0.033878	0.110299	-4.789292	7.138951	6.974345	6.610669	6.952416	6.534859	6.68322	6.656541	6.551926	6.628404	6.673587	6.434321	6.877885	6.689193	6.587544
Mrpl51	-0.434561	4.996746	-2.341788	0.033903	0.110318	-4.738766	5.178626	4.704825	4.747236	5.569051	4.997903	5.319734	5.050088	4.856872	4.768722	4.830243	5.128213	4.830257	5.174405	4.847954
Smyd3	-0.49862	3.002871	-2.341703	0.033908	0.110318	-4.452257	3.016775	2.519311	2.924461	3.589663	2.874693	2.940957	2.893159	3.000548	3.178898	3.080873	3.280721	2.8487	3.286436	2.802815
Opn3	0.922733	0.99053	2.341654	0.033911	0.110318	-3.932904	0.643243	1.397635	1.921212	0.280432	1.143601	0.846031	0.625837	0.705741	0.736738	1.253084	1.199219	1.186803	1.407665	1.32462
Aph1b	0.633728	2.626747	2.341287	0.033935	0.110356	-4.183208	1.853246	1.874793	2.487668	1.526009	2.553396	2.244393	3.094359	2.91426	3.01701	3.060806	2.792614	3.346294	3.254	3.031778
Acot4	-0.589195	2.14775	-2.341192	0.033942	0.110356	-4.278608	2.342161	1.339943	2.225837	2.656049	1.840817	2.230763	2.380012	2.186344	2.204856	2.027955	2.259075	2.027776	2.601568	2.105984
Arrdc1	0.638033	4.543252	2.34086	0.033963	0.110375	-4.652318	4.485336	5.370323	4.850017	3.810268	4.705768	4.185462	4.494157	4.455617	4.62508	4.766622	4.573843	4.619447	4.161181	4.571694
Kcng2	-1.110101	5.317546	-2.340823	0.033966	0.110375	-4.688545	5.150398	2.184396	4.421813	5.054231	5.169141	4.094316	6.195447	5.964191	6.055107	5.941725	5.861397	5.943917	6.198122	6.272334
Tmem125	-0.972116	2.713025	-2.340419	0.033992	0.110431	-4.285693	2.645748	2.100024	1.874203	2.708042	2.28197	0.695385	2.415815	3.475434	2.772565	3.129854	3.345785	3.859433	3.589113	3.380568
Sstr4	-0.855985	4.408302	-2.33969	0.03404	0.110556	-4.65772	4.210384	2.874583	3.651925	5.126698	4.184254	4.546193	4.527267	5.016268	4.639062	4.182876	4.624251	4.529644	5.073464	4.610873
Tusc3	-0.512945	7.116931	-2.339145	0.034076	0.110642	-4.793118	6.709648	5.627033	6.553382	6.914747	6.502458	6.511365	7.486197	7.465943	7.786828	7.625816	7.405322	7.478367	7.657024	7.925628
Zbtb38	-0.571734	5.427721	-2.338644	0.034108	0.110719	-4.76711	5.548378	4.866787	5.612208	6.053974	5.052636	5.381179	5.417651	5.702559	5.20918	5.008447	5.488226	5.475754	5.901	5.307451
Rabgap1	-0.340295	6.859799	-2.338449	0.034121	0.11073	-4.796018	6.795581	6.524735	6.734277	7.225541	6.629912	6.792218	6.909786	6.924087	6.897742	6.692459	6.986194	6.812749	7.162564	6.962984
Extl2	-0.446993	5.11416	-2.337728	0.034169	0.110854	-4.726246	4.915133	4.302134	4.79429	5.324526	4.654263	4.940905	5.138455	5.31939	5.230115	5.194928	5.434399	5.287022	5.663896	5.445693
Ero1lb	-0.381173	4.197	-2.337461	0.034186	0.110881	-4.639491	4.123692	4.216563	3.867281	4.458437	3.680967	3.922669	4.660078	4.044317	4.457672	4.281549	4.043791	4.060629	4.449555	4.578298
Araf	-0.262807	7.11107	-2.337086	0.034211	0.110931	-4.797791	7.110492	6.893939	6.897938	7.31171	7.055199	6.956503	7.254972	7.042862	7.239202	7.126369	7.223964	7.039993	7.179933	7.227919
Zbtb11	-0.646196	5.731109	-2.336716	0.034235	0.11098	-4.785398	5.611862	5.119134	5.557465	6.819285	5.546404	6.058089	5.75294	5.621239	5.673882	5.392518	5.746959	5.605434	6.013056	5.747651
Aldh1l1	-0.585348	4.679286	-2.336316	0.034262	0.111036	-4.683569	4.97331	4.758107	3.651925	4.443178	4.27571	3.848745	5.224248	4.863466	4.950701	4.971302	4.540262	4.938905	5.023827	5.111939
Grsf1	-0.337303	7.057299	-2.335725	0.034301	0.111104	-4.800319	7.095764	6.560694	6.865757	7.326287	7.056191	7.011845	7.357486	6.961449	7.199653	6.963651	7.117237	6.915727	7.156545	7.225818
Foxred2	-0.65044	2.471745	-2.335712	0.034301	0.111104	-4.308035	2.402324	1.607774	2.434653	2.825673	1.463853	2.3616	2.550752	2.650138	2.625819	2.600561	2.604547	2.921125	2.984265	2.868948
Asun	0.434838	5.845721	2.335576	0.03431	0.111104	-4.783096	5.629274	6.384641	5.911863	5.824744	6.119413	6.236634	6.002911	5.744244	5.800582	5.918216	5.4978	5.654816	5.316642	5.826046
Shc3	-0.6673	4.371378	-2.335429	0.03432	0.111106	-4.736369	5.507875	4.435504	4.921122	5.068223	4.223657	4.876712	4.107579	3.653399	3.842287	3.574008	3.84309	4.158035	5.105631	3.96323
Aatf	0.480798	3.976276	2.334997	0.034349	0.111162	-4.642749	3.832629	3.982112	4.760222	4.225001	4.695613	4.589557	3.734959	4.026799	3.627666	3.734632	3.690026	3.732929	3.627601	3.510555
Prrg1	-0.630707	2.670437	-2.334883	0.034356	0.111162	-4.537109	3.502386	2.582864	3.203331	3.967847	3.238702	3.402651	2.286414	2.287882	2.127623	1.451089	1.674823	2.581351	2.994118	2.357047
Synj2bp	-0.447975	6.064098	-2.334524	0.03438	0.111203	-4.798344	6.293354	5.673024	6.038962	6.771216	6.169801	6.456791	6.133632	5.795142	5.867432	5.796076	6.034738	5.792186	6.158147	5.940678
Cys1	-0.407232	3.953129	-2.334416	0.034387	0.111203	-4.621383	4.082914	3.7962	3.353699	4.192519	4.035185	3.780216	4.091065	4.078726	4.14139	3.87341	4.073974	3.859433	3.913738	4.174106
Megf9	-0.978759	6.537422	-2.334246	0.034398	0.111209	-4.800932	6.533379	5.22252	6.51814	7.631043	5.753932	6.910221	6.325841	6.850484	6.618735	6.006107	6.76815	6.690057	7.228736	6.484961
Gas7	-0.346069	9.220016	-2.333863	0.034424	0.11261	-4.737537	9.535843	9.291159	9.551667	9.821441	9.254067	9.231896	8.940685	9.09987	9.029932	8.959194	9.243506	8.962914	9.219609	8.941096
Lphn3	-0.432942	6.240301	-2.332253	0.03453	0.111568	-4.796376	5.88391	5.634551	5.575945	6.545485	5.721768	6.202126	6.645274	6.315101	6.679189	6.300545	6.213879	6.381753	6.598453	6.687903
Kcnt1	1.167084	6.247908	2.33215	0.034537	0.111568	-4.793684	5.831537	8.294725	7.274952	5.771983	6.49589	5.804544	6.559848	5.326567	6.40422	6.262614	5.510468	5.810006	5.591107	6.55527
Ip6k1	-0.45979	5.575381	-2.330408	0.034653	0.1119	-4.780429	5.66348	4.972603	5.575945	5.785355	5.145088	5.356287	5.651478	5.733485	5.56458	5.339365	5.795641	5.782499	6.131613	5.591559
Map3k10	-0.354216	8.355632	-2.330231	0.034665	0.1119	-4.781761	8.776031	8.119631	8.19241	8.378507	8.149754	8.437632	8.440598	8.265328	8.280837	8.536501	8.162563	8.437511	8.325091	8.481279
Slc8a1	-0.80978	6.721086	-2.330186	0.034668	0.1119	-4.808474	6.391238	5.344885	6.167186	7.456998	6.162446	6.779986	6.990651	7.073045	6.889724	6.423508	7.050495	6.825609	7.564584	6.991097
Gde1	-0.403912	6.864514	-2.329957	0.034683	0.111919	-4.808646	6.86604	5.831871	6.458305	6.460888	6.370643	6.373181	6.930044	7.240711	7.143134	7.259984	7.36796	7.418419	7.22194	7.174439
Dgat2	-0.620392	6.728234	-2.32907	0.034742	0.11208	-4.805936	6.367854	5.173818	6.554003	6.604286	6.028283	5.697128	6.955354	7.270787	7.311373	7.4407	7.097956	7.404501	7.031757	7.274413
Emc6	0.373152	4.351496	2.328374	0.034789	0.1122	-4.637039	4.082914	4.167656	4.334789	3.781437	4.519714	4.164107	4.543542	4.327931	4.575048	4.535316	4.585863	4.36136	4.649823	4.369521
Nptn	-0.694121	9.287859	-2.327877	0.034822	0.112277	-4.765553	9.091516	7.661641	8.70243	9.522186	9.006631	9.086936	9.481217	9.54707	9.80116	9.539361	9.472791	9.566092	9.679354	9.874416
Sdf2l1	0.759186	4.020322	2.327546	0.034844	0.112303	-4.601908	3.875708	3.982112	3.851187	3.355447	5.030587	4.578838	3.392685	3.979018	3.225976	4.336834	4.502714	4.66836	3.633916	3.973287
Akap6	-0.826921	7.472774	-2.327476	0.034849	0.112303	-4.806314	7.341718	6.445744	7.379642	8.489995	6.944318	7.579042	7.53585	7.695316	7.510841	6.854229	7.614507	7.485209	8.235521	7.516288
Wee1	-0.404049	4.637909	-2.327211	0.034867	0.112311	-4.733634	4.796842	4.320894	4.539472	5.138114	4.6725	4.729403	4.725734	4.532109	4.833994	4.349296	4.81759	4.372473	4.740412	4.425531
Prr11	1.659993	-0.800544	2.327159	0.03487	0.112311	-3.733647	-1.22254	-0.103798	0.158563	-0.600663	1.20216	1.165512	-1.73438	-0.380636	-2.155202	-2.176425	-0.731011	-0.24119	-0.405292	-0.748265
Herc2	-0.393875	8.163344	-2.326928	0.034886	0.112331	-4.803967	7.613533	7.711543	7.48867	8.314752	7.368535	7.704227	8.765532	8.475506	8.690728	8.241498	8.274019	8.296343	8.668428	8.67934
Isoc1	-0.311525	5.567697	-2.326409	0.03492	0.1124	-4.788508	5.569851	5.273489	5.258166	5.788981	5.588172	5.359422	5.568419	5.478195	5.589995	5.669274	5.682652	5.699625	5.797899	5.656972
Lpin2	-0.335995	6.848482	-2.326244	0.034931	0.1124	-4.816774	6.480286	6.419873	6.537755	7.05936	6.356211	6.424226	7.267273	7.038498	7.122133	7.09834	6.921268	6.959987	7.096643	7.11095
Snap25	-0.769119	11.16954	-2.326113	0.03494	0.1124	-4.66766	11.73143	10.36894	11.43759	12.32924	11.80539	11.43762	11.05841	10.78537	11.13518	10.4548	11.15956	10.5983	11.3208	10.75161
Fndc8	1.109074	0.646447	2.326051	0.034944	0.1124	-3.828553	-0.129637	1.184734	0.307539	-0.411255	0.88224	0.734546	1.091998	0.923327	1.142163	1.14286	0.676952	0.930264	0.111637	1.529341
Plod1	0.512026	5.904208	2.325141	0.035006	0.112567	-4.78405	5.587774	6.074484	5.648747	5.086016	6.081895	5.577032	5.979177	6.346036	5.895368	6.219139	5.879821	6.433959	5.627961	6.248723
Ccdc23	0.656026	2.538822	2.324694	0.035036	0.112607	-4.344209	2.753611	2.801663	2.908996	1.818649	3.273255	2.644629	2.324585	1.983359	2.313467	2.572488	2.739799	2.703718	2.423441	2.55752
Nup160	0.397971	4.306141	2.324677	0.035037	0.112607	-4.639412	3.799454	4.595055	4.299641	3.989017	4.140005	4.156917	4.704179	4.502842	4.508062	4.311581	4.250477	4.233112	4.294734	4.581589
Slitrk3	-1.212769	5.995142	-2.323743	0.0351	0.112748	-4.799911	5.665015	3.368632	5.58692	6.978582	5.293864	6.144226	6.310469	6.498913	6.562989	6.077758	6.213879	6.185695	6.561657	6.515391
F2r	0.3941	5.166192	2.323685	0.035104	0.112748	-4.766549	5.195727	5.140412	5.301747	4.899604	5.4917	5.813716	5.052933	5.045558	4.905145	5.087444	5.09285	5.218879	4.974911	5.150119
Ppard	0.39754	5.673557	2.323469	0.035118	0.112748	-4.804837	6.014933	6.250168	6.340035	5.448911	6.051362	5.862041	5.191057	5.5474	5.178394	5.528417	5.493021	5.65936	5.483308	5.412816
Sdr42e1	-0.721938	2.615353	-2.323468	0.035118	0.112748	-4.401829	3.188908	1.532513	2.924461	2.806722	2.411703	2.131561	2.582602	2.522625	2.734001	2.325762	2.883067	2.668085	3.356854	2.814049
Ifnar1	0.274623	6.816412	2.32294	0.035154	0.112819	-4.823105	6.705923	6.913847	7.062053	6.692746	6.887461	7.032125	6.735695	6.773798	6.704343	6.7733	6.604237	6.949513	6.793022	6.815664
Crebrf	-0.355484	5.480805	-2.322862	0.035159	0.112819	-4.790657	5.506163	5.326436	5.414195	5.732361	5.006144	5.314898	5.857529	5.350235	5.381255	5.517415	5.44928	5.523062	5.743447	5.644439
Hm13	0.387582	6.32779	2.321238	0.035269	0.113141	-4.817482	6.178698	6.387314	6.372816	5.861939	6.651087	6.215163	6.15762	6.246128	6.468273	6.413652	6.258681	6.413482	6.268819	6.715125
Pcsk7	0.594696	5.627374	2.320872	0.035294	0.113147	-4.771826	5.172161	6.170698	5.603828	4.835601	5.646007	4.969911	6.121487	5.844307	6.128798	5.955377	5.672824	5.704031	5.618437	6.002137
Kbtbd4	-0.34741	3.835721	-2.320798	0.035299	0.113147	-4.635444	4.012279	3.661386	3.859257	4.090441	3.660229	3.637716	3.975863	3.891392	3.786848	3.968034	3.838074	3.542721	4.033233	3.853172
Rnf24	-0.902983	1.219666	-2.320794	0.035299	0.113147	-4.076337	1.741237	0.118359	1.430088	1.353927	0.649545	0.386712	1.258992	1.393205	1.579251	1.064403	1.652137	1.404538	2.379013	1.386135
Drd1	-0.664289	1.857141	-2.31979	0.035367	0.113335	-4.194512	2.091157	1.310208	1.514261	1.856	0.731352	1.708392	1.697535	2.207232	2.260183	2.108365	2.259075	2.046399	2.220182	2.446395
Hmgcs2	-0.73262	3.64293	-2.319596	0.03538	0.113347	-4.585469	4.495729	3.613496	2.568639	3.295536	3.464807	3.014396	3.933266	3.923301	3.487872	3.721909	3.728487	4.083352	3.805994	3.998126
Zfp955a	-0.669495	5.525313	-2.318757	0.035437	0.1135	-4.816833	5.761485	4.909553	5.603828	6.640858	5.445483	6.163187	5.293243	5.398708	5.422258	5.059905	5.558532	5.271276	5.691408	5.16993
Fam20c	0.365579	6.560931	2.317872	0.035498	0.113663	-4.826125	6.157034	6.375697	6.325547	6.144491	6.804844	6.557822	6.558848	6.742839	6.618735	6.765571	6.582655	7.005312	6.441681	6.788867
Dfna5	-0.438482	3.560766	-2.317638	0.035514	0.113683	-4.588495	3.854329	3.69053	3.436929	3.686144	3.152236	3.069038	3.908348	3.340143	3.799353	3.772142	3.574129	3.412394	3.556237	3.73402
Scamp1	-0.737714	8.046072	-2.317136	0.035548	0.113763	-4.817314	7.880345	6.556742	7.676488	8.611168	7.633148	8.166989	8.16165	8.14701	8.305704	8.088529	8.217437	8.252404	8.543158	8.410647
Neu3	-0.518746	2.151819	-2.316457	0.035594	0.113874	-4.270362	2.295333	2.064844	1.669168	2.154676	1.664612	1.496914	2.033384	2.419003	2.223535	2.681644	2.949696	2.749899	2.097585	1.991897
Csmd1	-0.520874	6.467843	-2.316348	0.035602	0.113874	-4.827921	5.935721	5.741464	6.004816	6.843894	5.607258	6.127773	7.135247	6.866985	7.048092	6.666964	6.572874	6.346495	6.825116	6.846083
Kif3a	-0.637487	7.062913	-2.315873	0.035634	0.113948	-4.832362	7.078005	6.496959	7.02756	8.067031	6.771393	7.437139	6.989909	7.165996	7.213736	6.762468	6.981652	6.614847	7.375885	6.910305
Sfr1	0.271443	6.041603	2.315467	0.035662	0.114006	-4.83128	6.107068	6.369403	6.46955	6.329302	6.529152	6.590875	5.892891	5.800307	5.738623	5.829078	5.798227	5.734497	5.762308	5.655412
Nhs	-1.02857	0.930195	-2.315153	0.035683	0.11404	-4.061972	1.401336	0.310827	1.061799	1.548999	-0.613814	1.193889	1.443753	1.155984	1.549949	0.534523	1.135223	0.28321	1.922909	1.009844
Pik3cd	0.461457	5.279027	2.31488	0.035702	0.11404	-4.757094	4.725042	5.588845	4.858093	4.713732	5.137606	5.152692	5.482396	5.805453	5.119774	5.777752	5.557006	5.925172	4.887747	5.217242
Prkca	-1.20148	6.779872	-2.314783	0.035709	0.11404	-4.833622	7.16686	4.993802	6.682918	7.866875	6.613825	6.959089	6.644332	7.326736	6.535221	5.918216	7.286063	6.767164	7.735843	6.437623
Pip4k2a	-0.382518	6.751983	-2.314758	0.03571	0.11404	-4.83761	7.13175	6.864192	6.511115	7.402028	7.123547	7.037517	6.345699	6.644808	6.403196	6.536809	6.560745	6.733083	6.666267	6.581804
Snx5	0.30677	6.775425	2.314403	0.035735	0.114088	-4.838647	6.881974	7.136149	7.019932	6.763251	7.211253	7.148609	6.664934	6.666652	6.407287	6.733445	6.433489	6.780435	6.390377	6.632659
RGD1310429	-0.70569	4.505457	-2.312539	0.035863	0.114456	-4.739916	4.529842	3.60127	4.503885	5.254574	3.859855	4.772376	4.614587	4.604749	4.488889	4.369831	4.747086	4.387158	5.139318	4.275119
Robo3	-1.041666	6.314279	-2.312446	0.035869	0.114456	-4.812399	5.73833	4.522355	4.485756	6.280342	5.024515	5.848717	8.267091	6.631348	7.206125	7.491246	6.30668	6.937715	6.548344	7.138023
Rplp1	0.532503	4.279946	2.311179	0.035957	0.114704	-4.745158	4.889087	5.341214	4.700841	4.140492	4.964461	5.152692	3.933266	3.630385	3.895674	4.18751	3.650513	3.848862	3.536146	4.134325
Ezh2	0.534275	3.829319	2.311006	0.035968	0.114712	-4.628677	3.431974	4.200444	3.693138	3.847833	4.335792	4.512792	3.927077	3.891392	3.613573	3.721909	3.621611	3.838214	3.318158	3.769079
Mcu	-0.485034	4.325094	-2.309765	0.036054	0.114955	-4.733755	4.55325	3.7962	4.251403	4.833262	4.268877	4.512792	4.425566	4.313555	4.15118	4.10172	4.373459	4.372473	4.713828	3.96323
Bcat1	-0.611695	10.01515	-2.309575	0.036067	0.114963	-4.758361	10.12489	9.04881	9.968113	10.64011	9.968507	10.10249	9.986457	10.02413	10.02971	9.758629	10.25513	9.920168	10.42226	9.964215
Serinc2	1.078026	5.119574	2.309452	0.036076	0.114963	-4.723756	3.788223	6.170698	5.03592	4.591724	4.94316	4.990279	5.138455	5.414511	6.215333	5.70527	5.010524	4.946361	4.353321	5.420187
S100a16	0.669278	6.237971	2.308594	0.036135	0.115121	-4.832471	6.248988	6.822673	5.763903	5.190226	6.754372	6.193368	6.224758	6.271141	6.093693	6.659478	6.362771	6.548622	6.054845	6.16455
Gtl3	-0.281442	5.582397	-2.307276	0.036227	0.115382	-4.816859	5.586154	5.269631	5.198991	5.540658	5.453038	5.209391	5.802806	5.722645	5.885139	5.695542	5.826363	5.597563	5.700463	5.698484
Mark2	-0.292538	6.739177	-2.306722	0.036265	0.115474	-4.852779	6.740566	6.586505	6.702112	7.01722	6.363445	6.694436	6.843142	6.850484	6.774901	6.813582	6.78193	6.772767	6.744232	6.677945
Rab12	-0.528624	4.152005	-2.305952	0.036319	0.115593	-4.710319	4.399379	3.309744	4.299641	4.529512	3.930833	4.164107	4.107579	4.490115	4.286137	3.978769	4.417843	3.995035	4.258405	4.051312
Slco2b1	-0.41711	5.820258	-2.305909	0.036322	0.115593	-4.817812	5.785686	5.279255	5.140599	5.394419	4.979188	5.288824	5.532178	6.791195	5.553549	6.66281	6.467996	7.10028	5.668518	5.869771
Socs5	-0.366512	6.573408	-2.30563	0.036341	0.115624	-4.854232	6.674246	6.086593	6.585353	6.999074	6.492228	6.715998	6.748037	6.454836	6.573948	6.480327	6.450846	6.495398	6.740579	6.546877
Siglec1	3.145857	1.191776	2.305154	0.036374	0.115699	-3.838454	0.031429	1.310208	1.061799	-1.388744	3.877929	2.695134	0.563361	3.509467	-0.632503	2.06872	2.604547	4.288919	-0.893866	-1.040339
Slc7a14	-1.06444	5.366149	-2.304574	0.036415	0.115779	-4.779444	4.832812	3.375827	5.217941	5.83997	3.665442	4.786421	5.684875	6.276092	6.214166	5.082476	6.043481	5.858236	6.446174	5.850804

Ppp1r9a	-0.889282	6.086761	-2.30452	0.036419	0.115779	-4.849784	6.087732	5.630045	6.253904	7.158921	4.95385	6.099927	5.787514	6.64672	6.313411	5.535706	6.141411	6.110255	6.667807	5.852167
Fam65b	-0.294924	6.177873	-2.304206	0.036441	0.115818	-4.843171	6.010107	5.693323	5.676412	6.039842	5.622079	5.930042	6.673278	6.362423	6.620498	6.109853	6.316632	6.078939	6.759477	6.619913
Mrpl12	0.374088	5.484629	2.303428	0.036495	0.11596	-4.821165	5.504448	5.792089	5.562416	5.156942	5.975751	5.479548	5.548398	5.232912	5.403951	5.46111	5.602089	5.290932	5.387506	5.422024
Ncs1	-0.427756	7.037825	-2.30281	0.036538	0.116067	-4.858773	7.453907	6.740753	7.046678	7.15049	6.550438	7.163034	7.275822	6.746411	6.965889	6.895179	6.854523	7.10028	7.469791	7.128493
Ociad1	-0.371354	7.894651	-2.302521	0.036558	0.116078	-4.848516	7.813025	7.219993	7.419752	8.032529	7.792307	7.773731	7.956268	7.915953	8.100906	8.027152	8.058848	8.034473	8.255883	8.131046
Pink1	-0.399416	7.212954	-2.302484	0.036561	0.116078	-4.858381	7.47551	7.315224	7.01	7.306187	6.831174	6.811233	7.398326	7.164662	7.293251	7.399847	6.947706	7.312774	7.141108	7.585123
Ptpn14	-0.726342	2.237977	-2.302199	0.036581	0.116086	-4.452115	3.064101	1.812527	2.885483	3.043681	1.947363	2.613455	1.888013	1.934092	2.597457	1.14286	2.049375	1.989793	2.805569	1.910497
Syt4	-0.709117	7.637793	-2.302134	0.036586	0.116086	-4.853323	7.310659	6.276355	7.340908	8.310975	7.172879	7.611947	7.801628	7.637337	7.824023	7.79632	7.726988	7.822584	8.145556	8.159401
Igfbp4	-1.090872	5.560307	-2.302034	0.036593	0.116086	-4.841166	5.375315	6.076693	4.529394	7.193527	5.150675	5.078533	5.989609	4.736311	5.580969	5.854072	5.523026	5.964872	5.075785	5.752039
Smyd5	0.43434	4.522111	2.301152	0.036654	0.116252	-4.718092	4.346765	4.830589	4.605721	3.950685	4.7007	4.174824	4.798736	4.515458	4.496589	4.616609	4.672958	4.416088	4.539769	4.71343
Cdk5rap3	0.56259	6.124818	2.300422	0.036706	0.116377	-4.839692	5.78286	6.720409	6.034515	5.319521	5.994511	5.695888	6.455131	6.289621	6.328589	6.423508	6.069397	6.224032	5.92695	6.505896
Ccdc33	-0.735803	2.200969	-2.300315	0.036713	0.116377	-4.26918	2.054131	2.01041	1.386086	2.283631	1.85913	0.65513	3.105363	2.099634	3.280514	2.127786	1.959843	2.300247	2.794335	3.286911
Ggt5	1.164237	1.187555	2.299753	0.036753	0.116471	-4.03341	0.643243	2.324846	2.053692	0.43652	1.685287	0.61372	1.17791	0.645799	1.393785	1.704918	0.676952	1.284167	1.1849	1.529341
Apoc1	-0.942448	3.701252	-2.299545	0.036768	0.116487	-4.600952	3.948134	3.404252	2.423812	3.781437	3.726565	2.244393	4.267989	3.916976	3.09008	4.086912	3.882605	4.66836	4.314529	4.20003
Gnai1	-0.722387	6.733696	-2.298943	0.03681	0.11654	-4.865062	6.912033	5.855224	6.637308	7.786635	6.815434	7.192778	6.578725	6.630381	6.803893	6.063962	6.721923	6.515588	7.084589	6.688666
Als2	-0.31728	5.705069	-2.298672	0.036829	0.11654	-4.837937	5.630846	5.15303	5.506998	5.722283	5.328778	5.444548	6.029154	5.89185	5.848001	5.674236	5.879821	5.828954	6.142728	5.820486
Slmap	-0.594016	6.34818	-2.298636	0.036832	0.11654	-4.866008	6.641858	5.852648	6.613057	7.109999	5.941785	6.718442	6.232308	6.499972	6.159437	5.829078	6.435151	6.250375	6.555016	6.055178
Apold1	1.503364	3.556193	2.298631	0.036832	0.11654	-4.604349	3.287913	6.0031	5.44669	3.137672	3.846148	3.55809	3.981847	2.781287	3.87219	3.401235	2.346454	2.951084	2.748504	2.584013
Gopc	-0.558316	6.580559	-2.29862	0.036833	0.11654	-4.866986	6.636389	6.027289	6.440264	7.480731	6.47229	7.058888	6.51516	6.446076	6.517355	6.366405	6.613074	6.505528	6.662411	6.402701
Mas1	-1.481358	5.910713	-2.297784	0.036892	0.116659	-4.836744	5.899649	2.294795	5.378145	6.659463	5.721768	5.72048	6.185111	6.261188	6.573038	6.450752	6.362771	6.44891	6.392241	6.445772
Creb3l1	0.658282	4.453136	2.297776	0.036892	0.116659	-4.756039	4.716181	4.47624	5.499273	4.022255	4.680246	5.374996	3.554005	4.572916	3.748669	4.277207	4.555623	4.659314	4.134703	4.147707
Fut9	-0.974534	1.93521	-2.297586	0.036906	0.116659	-4.352722	1.936985	2.264105	1.723223	3.309066	0.88224	1.976852	1.913281	2.287882	1.867508	1.28803	1.864385	1.715048	2.794335	1.708589
Ifi27	0.348771	6.240707	2.297449	0.036915	0.116659	-4.851698	5.980811	6.164479	5.982826	5.508766	6.291944	5.924761	6.264573	6.556003	6.294763	6.757022	6.715783	6.732364	6.056021	6.161255
Sumo3	0.314651	6.491745	2.297403	0.036919	0.116659	-4.867799	6.651187	6.683919	6.913037	6.424834	6.916716	6.894638	6.220969	6.295728	6.342541	6.251591	6.593858	6.296807	6.335245	6.080985
Atp1a1	-0.622596	9.835095	-2.296893	0.036955	0.116732	-4.809077	9.492606	8.156391	9.337416	9.753945	9.318558	9.201864	10.45742	10.20805	10.59466	10.31519	10.01025	10.1956	10.10459	10.54674
Copg2	-0.33814	6.474305	-2.296801	0.036961	0.116732	-4.865422	6.361238	5.766314	6.305016	6.528235	6.246583	6.107405	6.64998	6.660038	6.663884	6.520371	6.723964	6.604673	6.850422	6.670238
Npas4	-1.215955	2.891522	-2.295063	0.037085	0.11709	-4.500169	3.821655	2.667383	2.356997	3.051752	2.154315	1.72793	4.685967	1.747064	2.973531	3.676473	3.189137	3.110019	2.725031	2.83626
Tcp11	2.261473	-0.64585	2.294591	0.037118	0.117165	-3.731247	-1.642648	1.369077	2.039558	-1.075778	-0.516166	-0.214525	-0.672244	-0.26106	-0.386063	-2.176425	-0.072212	-1.660823	-0.305233	-0.505482
Grap	0.88133	1.667209	2.293672	0.037183	0.117325	-4.136591	1.371547	2.466372	1.874203	0.705659	2.062471	1.165512	1.511505	1.596873	1.914249	1.73055	1.803924	1.97042	1.521045	2.159814
Msra	-0.384649	3.650256	-2.293606	0.037188	0.117325	-4.649246	3.932918	3.317238	3.365078	3.776576	3.596165	3.595796	3.889373	3.791198	3.735714	3.409193	3.822919	3.52291	3.903318	3.571405
Slc7a5	0.447634	6.942477	2.293393	0.037203	0.117342	-4.875558	7.034708	7.038089	7.537259	6.340051	6.960841	7.001849	6.728595	7.001609	6.845941	7.182006	6.865042	7.156543	6.65	6.865111
Xkr4	-0.884453	4.167985	-2.293131	0.037222	0.11737	-4.705892	3.848935	2.864389	4.004574	4.992678	3.26641	4.013036	4.118484	4.560794	4.36817	3.832569	4.585863	4.502925	5.165713	4.323092
Mapk10	-0.572479	7.638557	-2.292144	0.037292	0.11756	-4.864797	7.845555	6.989082	7.661281	8.324986	7.589941	7.811707	7.628401	7.759033	7.602443	7.220392	7.825639	7.343689	8.007664	7.338059
Ppox	0.668018	4.134566	2.292012	0.037302	0.11756	-4.643177	3.713016	4.793461	3.952724	3.288723	4.05518	3.866483	4.473071	4.239446	4.384862	4.616609	4.280371	4.444449	3.602057	4.266966
Mov10	0.656057	5.27546	2.291614	0.03733	0.117619	-4.779852	4.633881	5.674484	5.231993	4.334907	4.824689	4.820946	5.958085	5.423464	5.759573	5.729308	5.250298	5.502977	4.979878	5.775217
Mpp5	-0.715386	5.032419	-2.290807	0.037388	0.117766	-4.851395	5.423291	4.414697	5.216372	6.253476	4.987536	5.868657	4.599099	4.876565	4.632088	4.528025	4.969824	4.86739	5.208658	4.658395
Mob3c	0.716022	0.873414	2.290686	0.037397	0.117766	-4.066517	1.071521	1.632013	1.363571	0.386344	1.48759	1.136566	0.028455	0.972957	0.787836	1.104165	0.764165	0.930264	0.879431	0.554014
Sytl1	2.055797	-0.170425	2.290185	0.037432	0.117848	-3.882309	1.033981	2.943995	0.715742	-1.788967	1.48759	0.61372	-1.009457	-0.81357	-0.929891	-0.311823	0.378374	-0.78301	-1.638202	0.045082
Ibsp	-1.688125	0.575907	-2.289383	0.03749	0.117998	-4.176577	0.592497	1.632013	0.353965	3.527359	-0.613814	0.772673	-0.399109	-0.26106	-0.080923	1.481601	0.924208	1.691577	-0.405292	0.119416
Zfp191	0.392146	4.145564	2.289203	0.037503	0.118008	-4.719036	4.247758	4.661326	4.352047	3.771698	4.237722	4.329906	4.028845	4.095628	4.039656	4.086912	3.786926	4.149447	4.182881	4.156561
Pcdh17	-0.83015	5.530971	-2.288308	0.037567	0.118179	-4.8494	5.282322	3.904639	5.181399	6.267405	4.983368	5.695888	5.919635	5.776047	5.976071	5.159982	5.590204	5.565643	6.237019	5.931681
Atmin	-0.476494	3.669703	-2.288113	0.037581	0.118192	-4.69864	3.973144	3.099845	4.029817	4.298064	3.639189	3.839794	3.545949	3.550909	3.641623	3.52358	3.633241	3.46871	3.817137	3.43979
Ccnjl	-0.450305	3.587102	-2.287293	0.03764	0.118347	-4.680827	3.689063	3.613496	3.353699	4.298064	3.628553	3.648009	3.30948	3.413873	3.440756	3.276274	3.518775	3.767014	3.822677	3.571405
Meox2	-1.330755	0.941197	-2.286962	0.037664	0.118391	-4.044759	0.539902	-1.271663	-0.067513	2.013053	0.517419	1.014597	1.697535	1.747064	1.360416	1.704918	1.230182	1.375373	0.546019	1.80183
Bcl11b	-1.086799	6.989442	-2.286698	0.037683	0.118407	-4.882588	7.023378	5.337533	6.533349	8.392079	6.760474	7.83695	6.986193	7.174638	7.048746	6.877361	7.093227	7.023638	6.852454	6.925923
Abhd17b	-0.541002	3.973893	-2.286616	0.037689	0.118407	-4.704047	3.788223	3.134193	3.834912	4.563778	3.751288	3.835297	4.187425	3.93587	3.96946	3.838474	4.168924	4.158035	4.44597	4.125333
Cntf	0.627234	2.656694	2.28534	0.037781	0.118666	-4.427762	2.072763	3.512664	2.578448	2.613062	2.847434	3.038068	2.746221	2.875639	2.185932	2.614395	2.616315	2.619163	2.393975	2.733504
Plekhg1	-0.620567	5.12465	-2.284939	0.03781	0.118726	-4.837418	5.062241	4.610386	5.079744	5.742369	4.316041	5.130842	5.141132	5.589771	5.339051	4.794232	5.071637	5.227065	5.666979	5.020333
Limk2	0.425123	6.580103	2.284772	0.037822	0.118733	-4.887034	6.389381	7.054995	6.792851	6.093604	6.34894	6.467001	6.789566	6.59119	6.545465	6.809078	6.432658	6.56728	6.507652	6.748447
Ptk2b	-0.772172	9.961295	-2.284416	0.037848	0.118747	-4.807226	10.2125	8.451488	9.968987	10.44279	9.918425	9.900677	10.01737	10.31821	10.06881	9.872737	10.30103	9.929447	10.23631	9.821035
Il6r	0.438699	5.6362	2.284335	0.037854	0.118747	-4.844044	4.97826	5.377509	5.964241	5.294232	5.609964	5.34684	5.597939	5.936314	5.63778	5.83056	5.71858	6.226076	5.563249	5.857606
Sez6l2	-0.550507	9.466589	-2.284297	0.037856	0.118747	-4.844801	9.040074	7.989076	8.861779	9.417163	9.004962	8.86171	10.13086	9.787732	10.31402	9.827243	9.582405	9.748733	9.771658	10.19732
Papd7	-0.258256	6.014987	-2.284102	0.037871	0.118761	-4.877948	5.849207	5.659822	5.773542	6.098482	5.734224	5.698367	6.2159	6.028683	6.270267	6.180138	6.207086	6.131125	6.176652	6.210974
Tcf7l1	0.354808	4.209632	2.283176	0.037938	0.11892	-4.784961	4.65258	4.889586	4.636566	4.263733	4.770087	4.936713	3.713543	3.916976	3.930199	4.205901	3.911548	3.975732	3.515771	3.704139
Hspa4l	-0.53234	6.747593	-2.283127	0.037941	0.11892	-4.894765	6.777238	6.005422	6.571299	7.359991	6.672603	6.893557	6.77327	6.757074	6.805444	6.435247	6.905768	6.589673	7.209836	6.724831
Gdf11	-0.355299	6.25448	-2.282859	0.037961	0.11895	-4.892905	6.569086	6.168628	6.019292	6.686928	6.307864	6.597528	6.044959	6.009488	6.05641	6.184782	6.074738	6.264362	6.280944	6.318427
Dock1	0.374996	6.384542	2.281903	0.03803	0.119137	-4.895575	6.862698	7.015234	6.702112	6.242059	7.001081	6.985708	6.060593	6.015421	5.883671	6.119586	6.001472	6.267342	6.107988	6.137975
Zmynd15	1.383332	0.780739	2.281485	0.038061	0.119183	-3.994683	0.643243	2.082541	1.245402	-0.325128	0.691028	1.839907	0.202532	1.883083	0.317129	0.750369	0.484915	2.136071	-0.305233	0.321874
Pacrg	-0.883581	2.271009	-2.281302	0.038074	0.119183	-4.423383	3.342675	0.854759	1.858187	2.297273	2.411703	2.493017	2.227194	2.709903	2.024877	2.127786	2.750518	2.433555	2.601568	2.011549
Slc35f6	0.389772	5.765799	2.281291	0.038075	0.119183	-4.862293	5.592624	5.71476	5.823889	5.117115	5.92667	5.49531	5.713926	5.865813	5.861481	6.021763	5.704867	6.054998	5.788022	6.069312
Ywhag	-0.749881	9.51665	-2.28019	0.038155	0.119403	-4.817187	9.880527	8.674093	9.489423	10.63198	9.588755	10.27371	9.318889	9.238538	9.362014	9.118146	9.493378	9.178486	9.759976	9.227429
Nfatc4	0.653617	1.929836	2.27979	0.038184	0.119463	-4.261039	2.072763	1.972946	2.10888	1.033666	2.124346	2.422822	1.697535	2.099634	1.743567	2.274406	2.100543	2.170435	1.493524	2.124152
Ptpn4	-0.541049	5.780036	-2.278283	0.038294	0.119777	-4.895255	5.958442	5.476206	5.934953	6.766932	5.813839	6.063866	5.865643	5.268128	5.424278	5.290656	5.753634	5.403558	6.14162	5.788093
Cks1b	0.527915	1.921057	2.276591	0.038418	0.120133	-4.401861	2.127257	2.200694	2.795835	2.241914	2.936364	2.854719	1.372662	1.908813	1.426399	1.569444	1.762161	1.404538	1.28461	1.445134
Secisbp2l	-0.459449	7.886827	-2.276243	0.038444	0.120182	-4.897666	7.632888	7.753576	7.589125	8.058045	6.881875	7.315291	8.253243	8.185675	8.310899	7.956953	7.982194	8.137678	8.29427	8.070853
Gdap1	-0.663882	6.653553	-2.274968	0.038538	0.120444	-4.908852	6.757939	5.817411	6.62079	7.557135	6.767763	6.764105	6.848883	6.705707	6.605444	6.207777	6.898558	6.325681	6.946793	6.341903
Stxbp5	-0.472611	6.994095	-2.274642	0.038562	0.120488	-4.90996	6.62854	6.347613	6.801252	7.326287	6.150414	6.719662	7.46339	7.349227	7.267043	7.134793	7.208498	7.09414	7.375885	7.063491
Tfrc	-0.401349	7.158726	-2.274115	0.0386	0.120576	-4.911116	6.975912	6.662016	6.808043	7.255987	6.807794	6.580837	7.955508	6.739258	7.802831	7.209609	6.766831	7.19513	7.499061	7.974948
Traf3ip2	0.710895	2.257333	2.273986	0.03861	0.120576	-4.339921	2.279379	3.046741	2.466695	1.405223	2.183675	2.410784	2.247205	2.007376	1.891068	2.558244	2.213317	2.568523	2.186205	2.474989
Capza1	0.43766	6.116383	2.273231	0.038666	0.120718	-4.907309	6.014933	6.146713	6.776966	6.424057	6.861581	6.843497	5.756435	5.83091	5.720655	5.74039	6.06081	5.979482	5.885203	5.611013
Ogfr	0.251562	6.377142	2.272913	0.038689	0.120749	-4.910113	6.417897	6.66789	6.727681	6.402918	6.576958	6.674436	6.164213	6.330651	6.143586	6.475597	6.385299	6.354924	5.938477	6.038512
Capn6	-0.663016	3.107259	-2.272826	0.038696	0.120749	-4.622277	3.502386	3.631641	2.654608	3.701615	2.661692	2.924117	2.955303	2.926907	3.09008	3.508843	2.782205	3.78376	2.535634	3.031778
Efnb2	-0.656011	2.904909	-2.272272	0.038737	0.120846	-4.552072	2.753611	1.991799	2.877559	3.737082	2.586747	2.949304	2.814871	3.047633	3.159628	2.694725	2.771719	2.999674	3.349197	3.151754
Slc16a4	-0.56634	1.327907	-2.272056	0.038753	0.120864	-4.190914	1.669604	1.184734	0.851376	1.405223	1.05105	0.881356	2.05625	1.356279	1.690848	1.180545	0.998029	1.433124	1.626159	1.846283
Tmsbl1	0.54379	1.597985	2.271862	0.038767	0.120878	-4.343528	1.874641	2.557778	2.2133	1.874319	2.183675	2.705027	1.607516	0.301855	1.426399	1.064403	1.167576	0.930264	1.521045	1.501815
Cnn1	1.279211	0.498547	2.271542	0.038791	0.12092	-3.914784	0.643243	0.310827	0.25957	-1.223791	1.338932	1.378286	0.743216	0.76329	0.091681	1.104165	0.998029	0.666985	0.698429	0.442608
Rft1	0.408037	4.862273	2.271046	0.038827	0.121003	-4.835858	4.846411	5.501094	5.069347	4.629961	5.008197	5.004367	5.024227	4.643578	4.91667	4.850841	4.600749	4.712757	4.456698	4.861554
Cd80	1.176591	0.143355	2.27074	0.03885	0.121043	-3.863922	-1.052261	1.117652	0.604933	-0.166914	0.15195	0.117189	0.851758	0.645799	0.317129	0.20947	0.071497	0.84773	0.182287	-0.397871
Fam126b	-0.696511	3.887026	-2.270317	0.038881	0.121104	-4.725068	3.689063	3.000943	3.728808	4.690659	3.280067	3.988947	4.362751	3.904241	4.081214	3.335938	4.128407	3.686193	4.582846	4.070176
Zfat	0.38418	4.171709	2.270204	0.03889	0.121104	-4.715875	3.88628	4.073446	4.159638	3.649386	4.035185	4.307355	4.543542	4.182573	4.421726	4.164187	4.078235	4.357637	4.08486	4.548339
Tmem173	0.875834	2.677928	2.270025	0.038903	0.121114	-4.281971	2.179767	2.904741	2.298852	0.860705	2.109124	2.086865	2.100922	3.991112	2.330799	3.896239	3.70112	4.241218	2.253378	2.83626
Tmem106b	-0.593822	6.235438	-2.269843	0.038917	0.121124	-4.90824	6.001624	4.948678	5.90895	6.603599	5.892627	6.077257	6.169466	6.472197	6.63103	6.347893	6.445908	6.568086	6.682353	6.568598
Naaa	0.525283	4.203605	2.269591	0.038935	0.121151	-4.707091	3.676936	3.939046	4.004574	3.670505	4.319352	4.515605	3.742028	4.905609	3.755102	4.865376	4.615483	5.363275	3.536146	4.032198
Nova1	-0.418118	5.164354	-2.268865	0.038989	0.121288	-4.889498	5.317901	5.142523	5.262736	6.051963	5.214297	5.463612	4.986034	5.013306	4.992142	4.696819	5.146589	4.908688	5.210772	4.938001
Opa3	-0.344275	5.471678	-2.267081	0.039122	0.12167	-4.907606	5.869329	5.425102	5.730201	6.110122	5.673431	5.753028	5.183288	5.220124	5.180786	5.087444	5.417684	5.173007	5.594349	5.221467
Btbd1	-0.378025	8.001645	-2.266659	0.039154	0.121737	-4.895416	8.454366	7.825625	8.158275	8.720577	8.314963	8.539449	7.892579	7.55927	7.754279	7.728371	7.728344	7.677802	7.896485	7.778899
Cd9	0.57681	6.797211	2.265947	0.039207	0.121841	-4.922801	6.732532	6.761502	6.574366	6.156703	7.384032	7.35902	6.270696	7.169991	6.303568	7.120928	6.794286	7.627669	6.402451	6.517969
Cbfa2t2	-0.525647	4.928256	-2.265938	0.039208	0.121841	-4.865581	5.019661	4.489567	5.147205	5.556344	4.478849	4.934612	4.84543	5.27061	4.953501	4.483493	5.021628	4.811327	5.174405	4.861554
RGD1305455	-0.26615	6.491356	-2.265621	0.039232	0.121884	-4.92372	6.620647	6.222499	6.33136	6.589115	6.490026	6.311387	6.493334	6.584221	6.550097	6.566468	6.721923	6.424202	6.522196	6.469057
Lepre1	0.590783	4.458771	2.265112	0.03927	0.121971	-4.721764	4.110227	4.646529	4.156367	3.295536	4.335792	3.980827	4.955952	4.732714	4.700356	4.933176	4.431229	4.634911	4.477919	5.10738
Acss3	-1.028277	0.161763	-2.264786	0.039294	0.122015	-4.025041	1.48718	-0.572649	0.049949	0.280432	0.091273	-0.072565	-1.009457	0.871929	0.091681	0.750369	0.378374	0.969826	0.745852	-0.297732
Suclg1	-0.37527	6.810842	-2.264226	0.039336	0.122114	-4.928571	6.908147	6.237388	6.470867	6.939969	6.936793	6.550297	7.011277	6.840491	6.958939	6.807573	7.116719	6.755185	6.997268	6.835095
Dgkb	-1.351091	7.216128	-2.263306	0.039405	0.122297	-4.919212	7.489012	4.833205	6.7727	8.620525	7.400146	7.794437	7.278863	7.220306	7.43122	6.533172	7.526037	6.984941	7.874317	7.279995
Gcc2	-0.571604	6.450873	-2.262886	0.039437	0.122363	-4.930272	6.651187	5.738676	6.520686	7.233545	6.424669	6.796851	6.3981	6.410495	6.333971	6.006107	6.547745	6.318038	6.713252	6.237293
Selp	2.290122	-2.07331	2.262576	0.03946	0.122404	-3.766667	0.307952	-0.465897	0.818644	-2.731185	1.230571	0.231108	-2.558588	-2.550306	-2.604521	-2.176425	-0.411343	-3.260591	-3.260591	-2.228037
Gabrg1	-0.51257	5.049923	-2.262378	0.039475	0.122406	-4.865323	5.380933	4.554415	4.961037	5.021729	4.774918	4.446528	4.991976	4.97728	5.185557	4.766622	4.955998	5.39813	5.689893	5.642865
Cenpf	2.087508	1.051972	2.262297	0.039481	0.122406	-4.049481	0.176305	2.082541	0.353965	0.105385	3.167012	3.158148	0.282279	1.239467	0.385075	0.646479	0.584127	1.805289	0.836265	0.799858
Suv420h1	-0.388911	6.307278	-2.26193	0.039509	0.12246	-4.926462	6.135041	6.108354	6.019292	6.667364	5.665649	6.261985	6.65841	6.427283	6.504047	6.384683	6.281957	6.395437	6.437174	6.375469
Mrps26	0.415265	5.998351	2.261222	0.039562	0.122574	-4.919206	5.963443	6.560694	6.016589	5.668188	6.29783	6.045492	6.20187	5.912665	6.001999	6.104961	5.936071	5.786658	5.616844	5.888491
Cib2	-0.401373	4.781398	-2.261056	0.039575	0.122574	-4.862546	5.099062	4.622535	4.576652	5.084049	5.116828	4.452412	5.021324	4.442465	4.91667	4.663938	4.906537	4.565594	4.794977	4.734278
Pgls	0.460921	5.057089	2.261035	0.039576	0.122574	-4.857758	5.202087	5.346717	5.233545	4.344794	5.358047	4.959619	4.903326	4.921495	5.112276	5.159982	5.257831	5.105789	4.822887	5.118751
Lpar3	-2.122002	-1.624137	-2.260052	0.039651	0.122773	-3.834158	1.108109	-3.260591	-1.526232	-0.600663	-1.585779	0.846031	-2.088409	-0.992527	-2.604521	-0.961779	-1.142289	-1.243945	-1.638202	-0.886923
Slc30a10	-0.43222	4.169212	-2.257242	0.039864	0.1234	-4.7451	3.922683	4.055636	3.47943	4.188865	3.372207	3.653128	4.606864	4.468651	4.800172	4.382013	4.269233	4.346409	4.592606	4.323092
Sphkap	-0.843816	6.519841	-2.257013	0.039881	0.123409	-4.934605	6.220029	4.790772	6.388931	6.986498	5.431783	6.412199	6.886051	7.141789	6.983804	6.497225	6.955255	6.737387	7.212476	6.653136
Rabggta	0.525915	6.055625	2.256806	0.039897	0.123409	-4.914599	5.726612	6.329199	6.20883	5.26676	6.039366	5.499579	6.442169	6.189523	6.48482	6.178975	6.035834	6.007077	5.861178	6.533345
Syt11	-0.587421	9.070297	-2.25672	0.039903	0.123409	-4.890599	9.196126	8.073361	9.038735	9.468747	8.789284	9.087172	8.921549	9.305446	9.019949	8.879626	9.107462	9.361703	9.576543	9.161476
Vstm5	-0.378168	3.211166	-2.256662	0.039908	0.123409	-4.607915	3.311637	2.702143	3.138503	3.261145	2.828971	2.940957	3.410541	3.159031	3.592171	3.310669	3.42821	3.346294	3.302384	3.395603
Tmem154	1.537368	-0.668426	2.256528	0.039918	0.123409	-3.883381	-0.899176	0.480615	1.408255	-0.502853	0.266146	0.65513	-1.21312	-0.380636	-1.10526	-0.116374	-0.149817	0.081289	-0.755361	-3.260591
Vldlr	-0.350757	5.859694	-2.256381	0.039929	0.123412	-4.912467	5.633986	5.210498	5.390726	5.714678	5.383574	5.310045	6.077457	6.35191	6.221154	5.842364	5.920731	6.206544	6.405223	6.39524
Vstm2b	-0.44242	4.738746	-2.256061	0.039953	0.123456	-4.837513	4.64948	3.7962	4.287732	4.85187	4.511054	4.629061	5.006725	4.677653	5.275577	4.924873	4.675772	4.800396	5.023827	5.293472
Kcna4	-1.068303	4.582381	-2.255626	0.039987	0.123527	-4.809988	4.384541	1.934482	4.008208	5.222759	4.377679	4.548942	4.937596	4.937208	5.202134	4.908123	5.054439	4.797651	4.908735	5.015475
Pole3	0.320648	5.119797	2.255264	0.040014	0.12358	-4.906843	5.365903	5.51093	5.71583	5.314499	5.751483	5.658196	4.707794	4.58094	4.730053	4.830243	5.134364	4.824874	4.906128	4.692276
Sec13	0.320321	4.832983	2.254487	0.040073	0.123718	-4.880877	4.955851	5.110534	5.313541	4.932758	5.333698	5.292109	4.519061	4.544473	4.956296	4.398098	4.653101	4.581643	4.643576	4.482972
Pdha1	-0.502048	6.698485	-2.254376	0.040082	0.123718	-4.942497	7.293883	6.564634	7.014974	7.760405	6.92217	7.596751	6.310469	6.300596	6.32643	6.140054	6.423477	6.29972	6.601679	6.239378
Psmb3	0.331671	5.139048	2.254241	0.040092	0.123718	-4.906881	5.453818	5.519076	5.686652	5.162543	5.8691	5.470877	4.897007	4.843593	4.997579	4.960511	4.896921	4.76429	4.833902	4.636413
Csgalnact2	-0.667586	2.171016	-2.254137	0.0401	0.123718	-4.431798	2.372556	1.310208	2.423812	2.687469	1.415175	2.28453	2.467906	2.207232	2.55384	2.06872	2.149958	2.461578	2.041677	2.310209
Akap7	-0.541932	2.993379	-2.253787	0.040127	0.123745	-4.651994	3.145563	3.472958	2.969885	3.920151	2.672087	2.974059	3.01488	3.036005	3.079866	2.745897	2.729	2.880186	2.923692	2.544088
Alg2	-0.485978	7.891278	-2.253757	0.040129	0.123745	-4.93568	7.753279	6.870572	7.458446	8.138164	7.63647	7.874962	7.878615	8.054949	8.078635	8.170702	8.032741	8.133057	8.199248	8.204922
RGD1566386	-0.392588	4.067093	-2.253512	0.040148	0.123771	-4.791412	4.002595	3.884602	4.000932	4.621853	3.813652	4.017012	4.005537	4.015001	4.070936	4.020928	4.227643	3.854157	4.298715	4.20003
Ly96	0.678919	1.550198	2.25336	0.04016	0.123775	-4.283714	1.430522	2.046927	1.858187	1.430203	2.386673	2.101918	1.782265	1.112357	0.931093	1.678822	1.199219	2.027776	1.218908	1.048408
Cdh7	-0.784271	1.842261	-2.252703	0.04021	0.123898	-4.376949	1.741237	1.279848	1.825611	2.862845	1.72577	1.378286	1.962529	1.112357	2.00342	1.451089	1.740817	1.691577	2.575555	2.900911
Slc38a1	-0.638234	5.360232	-2.251779	0.040281	0.124085	-4.917069	5.367791	4.687583	5.534973	6.095557	4.989615	5.159902	5.114144	5.55353	5.199777	4.979343	5.385381	5.683357	5.99477	5.336959
Elmod1	-0.943387	8.535661	-2.251506	0.040302	0.124117	-4.922468	8.320454	6.437172	8.07887	9.15778	8.032615	8.637621	8.671477	8.903495	8.939084	8.857621	8.757017	8.829326	8.887274	8.994382
St3gal5	-0.657208	7.669378	-2.251144	0.040329	0.124158	-4.939278	7.963194	6.40764	7.43704	8.119088	7.846377	7.851218	7.57695	7.769164	7.628958	7.717196	7.724272	7.778359	7.782072	7.777824
Lysmd2	-0.347879	5.113714	-2.251064	0.040336	0.124158	-4.91143	5.504448	4.846214	5.192619	5.492552	5.354824	5.202424	5.032899	4.866752	4.986685	4.944172	5.166736	4.890765	5.278854	4.877707
Mid2	-0.50706	2.811479	-2.250671	0.040366	0.124219	-4.621305	3.00712	2.884706	3.215954	3.675737	2.661692	2.634313	2.550752	2.849305	2.707706	2.375352	2.750518	2.816513	2.913344	2.544088

Phb2	0.263246	6.399209	2.25054	0.040376	0.124219	-4.950121	6.465381	6.590376	6.788104	6.396595	6.824782	6.57208	6.232308	6.157685	6.221154	6.197475	6.353134	6.216857	6.256592	6.335095
Pld5	-1.477142	0.631374	-2.250271	0.040397	0.12423	-4.088116	1.717752	0.769921	0.56602	0.785264	0.422095	-2.070862	0.798508	0.58326	0.837186	1.023513	0.432628	1.515634	1.54805	1.32462
Ppp3ca	-1.11216	9.562786	-2.250198	0.040402	0.12423	-4.880038	9.691064	7.523917	9.175284	10.52508	9.163026	10.13184	9.602809	10.06725	9.784745	9.40267	9.959761	9.571828	9.896914	9.385225
Arx	-0.390449	4.680502	-2.250088	0.040411	0.12423	-4.863032	4.857199	4.368558	4.659851	4.897366	4.728358	4.188991	4.622269	4.572916	4.593117	4.616609	4.667312	4.885603	4.974911	4.955862
Tub	-0.504002	5.920923	-2.249634	0.040446	0.124306	-4.941075	5.886545	6.179975	5.721374	6.39501	5.06057	5.602394	6.120131	6.037457	6.169105	5.851154	5.827629	5.931448	6.081661	6.055178
Atp6v1c1	-0.448773	7.851597	-2.24935	0.040468	0.124312	-4.936486	7.985183	7.326872	7.807892	8.484602	7.985724	8.022678	7.752596	7.741241	7.966327	7.544604	7.894307	7.600753	8.058144	7.758327
Mx2	1.520954	4.737325	2.249339	0.040468	0.124312	-4.765053	4.646374	4.220564	5.557465	2.677071	5.046656	5.669608	4.42995	5.31939	4.213244	5.286341	5.037033	7.226091	3.866246	3.203933
Shisa4	-0.421782	7.310854	-2.248594	0.040526	0.124416	-4.947835	7.885294	7.094781	7.362003	7.6784	7.662446	7.325347	6.777576	7.363835	7.23748	7.050805	7.010365	7.461987	7.241718	7.209958
Osr1	-0.872217	2.144302	-2.248586	0.040526	0.124416	-4.515572	2.917193	0.854759	2.122353	3.019194	2.198134	3.165332	1.888013	1.830204	2.364852	1.51148	2.274011	1.826994	2.269694	2.159814
Tnpo1	0.30335	6.580314	2.248448	0.040537	0.124416	-4.955763	6.452989	6.802792	7.014523	6.831642	6.855892	7.110971	6.653733	6.258689	6.376321	6.260416	6.273621	6.228116	6.502484	6.518828
Prkci	-0.519803	6.47315	-2.248362	0.040544	0.124416	-4.95659	6.665067	5.999612	6.585961	7.384561	6.514062	6.918216	6.407022	6.194762	6.27139	6.055113	6.467184	6.287052	6.613712	6.27843
Cpne5	-0.446258	6.585082	-2.248196	0.040557	0.124424	-4.956875	7.201136	6.773809	6.676653	6.691456	6.161524	6.390142	6.219703	6.608958	6.397039	6.56112	6.731423	6.513916	6.827873	6.452977
Sec62	-0.483994	7.787205	-2.24805	0.040568	0.124427	-4.936943	8.041542	7.559501	7.529695	8.593136	7.873937	8.364425	7.531267	7.699928	7.551143	7.592568	7.442568	7.71974	7.753716	7.774952
Camsap2	-0.469219	8.349024	-2.247724	0.040593	0.124472	-4.927088	8.287932	7.881349	8.299516	9.069983	8.117313	8.534603	8.470165	8.300986	8.381474	8.055951	8.36881	8.166694	8.629844	8.326604
Pik3c3	-0.306206	5.478095	-2.24656	0.040683	0.124717	-4.923876	5.331503	5.146735	5.238194	5.774423	5.183745	5.429711	5.638278	5.605591	5.597175	5.515573	5.550887	5.54123	5.670055	5.50582
Slc7a6	0.418813	6.079744	2.246143	0.040715	0.124771	-4.934842	5.346894	6.112667	6.098972	5.73487	5.664348	5.971611	6.509993	6.155	6.384644	6.444957	6.105332	6.234221	5.941026	6.435806
Abhd11	0.547562	4.35324	2.24596	0.04073	0.124771	-4.804746	4.251852	5.095359	4.671355	3.82447	4.448934	4.109292	4.704179	4.12885	4.421726	4.421893	4.359523	4.060629	4.152409	4.373323
Fbxo21	-0.294227	6.521567	-2.245729	0.040748	0.124771	-4.957659	6.480286	6.140391	6.360077	6.551189	6.335922	6.05035	6.64998	6.631348	6.647551	6.647754	6.825669	6.627271	6.844988	6.526532
Actr3b	-0.498444	6.127215	-2.24553	0.040763	0.124771	-4.952638	6.220029	5.242337	5.687785	6.392629	6.156905	6.141497	6.314031	6.427283	6.32643	6.050032	6.507237	6.115777	6.120411	6.101761
LOC498154	0.349461	4.638038	2.245483	0.040767	0.124771	-4.842321	4.410408	4.904587	4.773092	4.33821	4.614391	4.600198	4.48576	4.836907	4.778229	4.922095	4.904139	4.584831	4.353321	4.489994
Oasl	1.44598	0.921664	2.245367	0.040776	0.124771	-3.997802	0.105685	0.725555	1.723223	-0.411255	1.48759	0.772673	1.17791	1.68885	0.512061	1.804823	1.430105	3.037402	-0.211665	0.045082
Vcan	0.493289	6.143618	2.245274	0.040783	0.124771	-4.950104	5.911342	6.372104	6.813763	5.87223	6.213159	6.136937	6.218437	6.178988	6.074529	6.119586	5.537023	6.413482	5.951178	6.220504
Kcnmb4	-0.342132	5.506093	-2.245262	0.040784	0.124771	-4.933544	6.002839	5.489533	5.27032	5.392832	5.389886	5.267287	5.742404	5.3896	5.56641	5.485779	5.385381	5.484316	5.703469	5.550167
Acbd4	-0.383601	6.769186	-2.244585	0.040836	0.1249	-4.962863	7.083183	6.802124	6.339314	6.577982	6.433862	6.215163	6.930818	7.026061	6.952655	7.000991	6.856385	6.987353	6.677011	6.900457
Lilrb3l	2.300033	2.067792	2.243643	0.04091	0.125002	-4.333993	1.787089	2.643736	3.99728	0.860705	3.563034	3.935331	1.000645	4.134313	-1.536754	1.51148	1.199219	4.519683	1.040235	1.048408
Apba2	-0.530132	7.900469	-2.243639	0.04091	0.125002	-4.947033	8.06616	7.246728	7.865361	8.414941	7.913905	7.817133	7.769126	8.083825	7.897111	7.496391	8.18422	7.799441	8.339654	7.719272
Slc36a1	-0.753518	4.564437	-2.243622	0.040911	0.125002	-4.851065	4.637014	3.200527	4.467397	4.984269	3.969275	4.548942	4.438678	4.70728	4.919536	4.14048	4.884809	5.140873	5.214991	4.719417
Prkar1b	-0.359339	9.001158	-2.243622	0.040911	0.125002	-4.91775	9.215381	8.588237	8.927412	9.079389	8.86373	8.779397	8.8827	8.996797	9.159413	8.982549	9.356134	8.952153	9.296521	8.939494
Grem2	-0.404542	3.831109	-2.243316	0.040935	0.125043	-4.809158	4.110227	4.069014	3.847136	4.517907	3.823012	3.94371	3.521509	3.614836	3.289407	3.683052	3.678847	3.816678	4.028447	3.803307
Wwox	-0.536163	2.51369	-2.243163	0.040947	0.125047	-4.588122	2.875353	2.100024	2.607481	3.374877	2.77212	2.907078	1.836107	2.471744	2.241976	2.40749	2.627988	2.252925	2.535634	2.460763
Zfp644	-0.51048	5.718025	-2.242898	0.040968	0.125078	-4.952549	5.764353	5.261885	5.615785	6.496064	5.570221	6.033765	5.756435	5.874	5.588194	5.271138	5.700728	5.616382	5.924376	5.609402
Idi1	-0.515662	4.352829	-2.242538	0.040996	0.125123	-4.915539	5.262171	4.204491	4.968516	5.539224	5.137606	5.218052	4.129307	3.282262	4.007682	3.393233	3.862983	3.42668	4.379868	4.212819
Gpalpp1	0.368484	4.789925	2.242446	0.041003	0.125123	-4.893768	4.71914	5.398856	5.355505	4.939298	4.930226	5.114234	4.778253	4.688835	4.830952	4.491011	4.524735	4.350161	4.47088	4.5246
Fut8	-0.456621	5.801866	-2.241915	0.041044	0.125217	-4.952338	5.831537	5.173818	5.582052	6.255225	5.499017	6.090524	5.731791	5.988526	5.898278	5.647573	5.740252	5.870046	5.970022	5.976117
RGD1560010	0.927902	0.878332	2.240927	0.041122	0.125421	-4.247928	1.341131	1.702379	1.386086	0.896996	2.361201	2.613455	0.357848	1.067371	-0.27702	0.134646	0.138317	0.34469	0.182287	1.009844
Qtrt1	0.550588	3.789678	2.240434	0.04116	0.125507	-4.674297	3.279917	4.042133	3.926082	3.114742	3.596165	3.408717	4.35355	3.818604	4.029076	4.237532	3.690026	3.761389	3.689548	4.225496
Homer1	-0.90215	5.995233	-2.24019	0.041179	0.125517	-4.967587	6.200041	4.889586	6.136805	7.474366	6.016093	6.690707	5.935138	5.899891	5.809882	5.376371	6.080059	5.551045	6.231823	5.66785
Cdkn2c	1.141993	1.013816	2.23994	0.041199	0.125517	-4.125808	0.955837	1.854335	0.642822	0.041976	2.335271	1.563674	0.798508	0.972957	0.787836	1.828755	0.885831	1.375373	0.375586	0.606649
Fitm2	-0.403106	4.894517	-2.239877	0.041204	0.125517	-4.878559	4.640141	4.155166	4.506456	5.070211	4.611694	4.543439	4.909618	5.157111	5.032426	4.922095	5.158711	5.19195	5.349634	5.329149
Snapc5	-0.588825	5.114931	-2.239823	0.041208	0.125517	-4.93526	5.53164	4.489567	4.996223	5.782933	5.325489	5.457773	4.750481	4.998404	5.005695	4.716194	5.43606	4.926391	5.486802	4.751911
Cdc26	-0.480162	5.002201	-2.239651	0.041222	0.125517	-4.947157	5.658863	4.763603	5.250517	5.995551	5.575768	5.794155	4.622269	4.643578	4.538218	4.706539	4.765698	4.53295	4.754973	4.479448
Ugt1a6	1.198105	0.80851	2.239593	0.041226	0.125517	-4.154691	1.831529	0.934883	1.775325	-0.502853	1.912714	2.217002	0.028455	0.705741	0.317129	0.591587	1.347792	1.186803	0.111637	-0.116166
Cchcr1	0.866903	4.998985	2.239179	0.041259	0.125584	-4.888175	4.646374	6.473676	5.416931	4.546747	5.022485	4.953408	5.108686	4.69993	5.211521	5.279845	4.695321	4.641051	4.148003	5.19378
Snx29	-0.462902	3.526413	-2.238546	0.041308	0.125703	-4.67777	3.562947	3.108509	3.319014	3.456176	2.81965	2.932562	3.562015	4.055878	3.375441	3.802672	3.882605	4.376159	3.811576	3.447025
Hspa12a	-0.607644	8.126206	-2.238235	0.041333	0.125746	-4.951227	8.076583	7.192752	8.198584	8.855212	8.006924	8.033513	8.135718	8.191583	8.169042	7.828575	8.423208	8.005618	8.528825	8.126547
Dhx40	-0.410099	5.481492	-2.237804	0.041367	0.125817	-4.954716	5.687857	5.232463	5.205336	6.098482	5.630099	5.871954	5.365968	5.491027	5.345461	5.194928	5.468884	5.359557	5.497232	5.32719
Ss18l1	-0.523729	3.980806	-2.23749	0.041391	0.125845	-4.786528	3.865059	3.52566	3.960246	4.412169	3.245679	3.747086	4.096591	4.101219	4.263751	3.765957	4.348982	3.816678	4.573018	4.111741
Naga	0.396662	5.916838	2.237422	0.041397	0.125845	-4.947521	5.689367	6.112667	5.866546	5.289121	5.895957	5.658196	5.692193	6.244866	5.865946	6.163768	5.957046	6.293887	5.889168	6.243539
Rnf165	-0.332793	6.859805	-2.237135	0.041419	0.125882	-4.976892	7.038268	6.476188	6.680643	7.117228	6.795366	7.027697	7.057341	6.779911	6.861677	6.661145	6.846427	6.684123	7.157093	6.867809
Hnrnpr	-0.713175	1.738549	-2.236314	0.041484	0.126035	-4.421966	1.874641	0.895377	1.981598	2.835056	1.664612	2.086865	1.697535	2.007376	1.663747	1.652245	1.674823	1.515634	1.770732	1.501815
Frem2	-0.783711	2.187779	-2.236229	0.041491	0.126035	-4.499047	3.271877	2.679063	1.792283	2.213416	1.285769	2.101918	2.324585	1.80302	2.430637	1.755735	2.604547	2.170435	2.379013	2.17732
Lrrc16a	-0.354408	5.770171	-2.235675	0.041534	0.126136	-4.960115	5.963443	5.830563	5.503141	5.853884	5.403987	5.486734	6.226019	5.740666	5.837429	5.93071	5.582726	5.751622	5.743447	5.95724
Pop5	-0.367377	4.648565	-2.235407	0.041556	0.126156	-4.904151	5.156961	4.357697	4.821391	4.917381	4.627805	4.850213	4.579502	4.481568	4.771898	4.42101	4.658802	4.597515	4.512607	4.594677
Ak1	-0.60787	5.852691	-2.235327	0.041562	0.126156	-4.975701	6.339284	5.552796	5.684382	6.91751	6.181673	6.666231	5.558443	5.425693	5.740245	5.378399	6.017092	5.338932	5.640562	5.524749
Llgl1	0.448747	6.26536	2.234775	0.041606	0.126256	-4.964083	6.128377	6.415516	6.200915	5.383269	6.341632	5.844248	6.496472	6.384369	6.32751	6.443989	6.216781	6.625724	6.375378	6.551919
Ddx31	0.40667	3.895243	2.234517	0.041626	0.126286	-4.705544	3.410162	3.833259	3.776458	3.275	3.86892	3.502454	4.32559	3.891392	4.19443	4.31582	4.065415	4.136468	3.903318	4.14326
Capn10	0.563505	5.617142	2.233575	0.041701	0.126472	-4.920195	5.187202	6.03299	5.515958	4.646043	5.322193	5.036061	6.114695	5.693335	6.073242	6.011344	5.814919	5.588061	5.573143	6.064616
Tmem181	-0.438156	5.933569	-2.23348	0.041708	0.126472	-4.956699	5.448478	4.919435	5.393507	6.112053	5.410211	5.649258	6.351488	6.23727	6.357417	5.972933	6.10846	6.139823	6.531528	6.465499
RT1-CE16	0.970554	3.145727	2.232956	0.04175	0.126566	-4.468791	2.957854	2.894758	3.599901	1.158502	2.865664	2.434761	2.717819	3.818604	2.667343	4.307328	4.302392	4.346409	2.954296	3.229331
Amd1	-0.383888	7.495547	-2.231731	0.041847	0.126829	-4.974056	7.771927	7.174818	7.50226	8.11042	7.677358	7.874689	7.33604	7.274502	7.333038	7.251156	7.513506	7.304578	7.489951	7.33221
Zfp322a	-0.402011	4.003283	-2.231342	0.041878	0.126869	-4.821409	4.055074	3.643613	3.847136	4.449301	3.686105	4.240912	4.14005	4.032662	3.901486	4.005262	3.958531	3.83286	4.258405	4.093415
Tnnt3	1.155702	0.170296	2.2313	0.041881	0.126869	-3.996609	0.031429	1.506527	0.307539	-0.502853	1.05105	0.571087	-1.21312	0.972957	-0.175642	1.481601	0.138317	0.567345	-0.405292	-0.397871
Tmed9	0.309279	6.484481	2.231128	0.041895	0.126878	-4.975012	6.058818	6.096426	6.17448	5.78172	6.483401	6.157795	6.562845	6.78513	6.662174	6.873052	6.737497	6.961215	6.67395	6.792422
Stau1	-0.243714	6.616594	-2.230877	0.041915	0.126888	-4.987284	6.700692	6.462737	6.566995	6.883999	6.561664	6.604151	6.525439	6.593175	6.557478	6.658643	6.719197	6.684866	6.646099	6.483202
Adamtsl3	0.739818	2.371362	2.230822	0.041919	0.126888	-4.484635	2.196854	3.176008	3.118472	2.093442	1.981199	3.214637	2.659281	2.287882	2.47808	1.920699	1.82436	1.783252	2.408783	2.372328
Src	-0.309668	6.682273	-2.230582	0.041938	0.126894	-4.987296	6.912033	6.531193	6.631431	6.611129	6.272434	6.372405	6.774994	6.787732	6.671557	6.757022	6.81422	6.781825	6.98682	6.662489
Rfesd	-0.549083	3.065784	-2.230475	0.041947	0.126894	-4.665559	3.127854	3.240489	2.744481	3.665254	2.640673	2.872384	3.083269	3.301815	2.858731	3.00938	3.401255	3.028062	3.515771	2.622863
Tmem252	1.844413	1.899346	2.230312	0.04196	0.126894	-4.471987	1.936985	5.298313	4.617663	1.837445	2.295481	2.685174	0.743216	1.020936	0.683763	1.51148	0.631286	1.643459	1.11438	1.122575
St8sia1	-0.946113	3.401016	-2.230268	0.041964	0.126894	-4.667649	3.32724	1.082904	3.013923	3.852461	2.962008	3.456346	3.797364	4.078726	3.620637	3.409193	3.765948	3.83286	3.964736	3.629792
Khdrbs3	-0.629643	8.008955	-2.228377	0.042115	0.127318	-4.964336	8.406283	7.481609	7.518274	8.83971	8.354428	8.614681	7.918159	8.043743	8.062839	7.538268	7.956385	7.708091	7.978182	7.711032
Atxn3	-0.35017	4.04422	-2.228238	0.042126	0.12732	-4.828691	4.031456	3.982112	3.910635	4.455398	3.696327	4.020978	4.34429	3.929599	4.290573	3.861859	3.967746	3.995035	4.221137	4.007943
RGD1305592	0.606244	6.358619	2.227429	0.04219	0.127484	-4.979927	5.744154	7.015234	6.179323	5.888093	6.476745	6.022931	6.780154	6.285944	6.596515	6.827011	6.127087	6.320909	6.028719	6.747715
Hip1	0.414408	6.528604	2.226899	0.042233	0.12758	-4.99074	6.542598	7.066713	6.651318	6.090669	6.730946	6.471354	6.615755	6.253678	6.409328	6.517613	6.185525	6.642652	6.505071	6.734472
Parn	0.33122	4.569016	2.226767	0.042244	0.12758	-4.849824	4.026686	4.658378	4.564365	4.314927	4.39974	4.40465	4.931426	4.782275	4.646003	4.620041	4.546426	4.671363	4.646703	4.820364
Btf3	0.347815	4.541948	2.226516	0.042264	0.127609	-4.925964	5.059908	5.119134	5.247446	4.711187	5.022485	5.522136	4.113042	4.313555	4.091419	4.259708	4.231474	4.149447	4.013992	3.803307
Hgf	0.81039	1.871549	2.224423	0.042432	0.128085	-4.389802	2.054131	1.953842	3.063656	1.273392	1.981199	2.654873	1.576217	1.464337	1.549949	1.481601	1.803924	1.542121	2.253378	1.991897
Sbk1	-0.533375	3.638178	-2.223756	0.042486	0.128215	-4.742698	3.92781	3.06466	2.812554	3.556013	3.601614	3.359461	3.908348	3.534475	3.59934	4.041554	3.609886	3.99982	3.850062	4.20003
Tenm3	-0.44048	7.11453	-2.223404	0.042514	0.128268	-5.000372	6.837723	6.625509	7.016779	7.638765	6.588666	6.957021	7.344192	7.020171	7.174673	7.021902	7.27914	7.140923	7.464485	7.505068
Mepe	-0.569331	2.047775	-2.222969	0.042549	0.128342	-4.466472	2.542593	1.453108	1.825611	2.255954	2.014259	2.00915	2.644268	2.14364	2.313467	2.308845	1.864385	1.715048	1.859646	2.105984
RT1-DOb	2.301949	-0.550961	2.222816	0.042562	0.128347	-3.798899	-0.217457	-0.186163	-0.007587	-2.731185	0.15195	0.949514	-0.529223	-0.26106	-1.304935	1.451089	-0.072212	1.930875	-1.904936	-1.632384
Tmem123	0.652777	4.305282	2.222298	0.042604	0.128432	-4.898496	4.912549	4.560742	5.110492	3.984807	5.33042	5.32776	3.545949	3.985077	3.416608	3.595091	3.986003	4.519683	4.156802	3.927469
Pttg1ip	0.542169	7.095989	2.222204	0.042611	0.128432	-5.001477	7.377284	7.489507	7.317305	6.234989	7.505629	7.074235	6.598333	7.250177	6.660461	7.2079	7.239266	7.628442	6.816812	6.955394
Krt26	-0.688001	1.961155	-2.221544	0.042665	0.128531	-4.479897	2.179767	1.151583	1.493674	2.797152	2.124346	2.515676	1.668148	2.099634	2.147322	2.048482	1.883989	1.930875	1.983515	1.846283
LOC654482	-1.016437	2.575219	-2.221531	0.042666	0.128531	-4.584777	3.978094	1.769471	1.890044	2.310787	2.712936	2.025031	2.746221	2.58779	2.330799	2.758411	3.939921	2.502617	2.269694	2.516844
Map2k5	-0.279573	5.843611	-2.220787	0.042726	0.128681	-4.997073	6.095726	5.949859	6.01749	6.39501	5.826691	6.072488	5.854271	5.591758	5.789655	5.751387	5.591695	5.54123	5.688377	5.672487
Eef2	0.376812	10.55883	2.219623	0.04282	0.128933	-4.909351	10.60047	10.85488	10.64263	9.967774	10.66899	10.4846	10.68313	10.40432	10.58674	10.6352	10.43253	10.63553	10.53249	10.69534
Pkn2	0.267621	5.731262	2.219422	0.042837	0.12895	-4.992873	5.772924	6.127661	6.16881	5.894839	6.015073	6.095233	5.710327	5.45873	5.379174	5.476341	5.47536	5.639565	5.483308	5.570183
Tmc5	-0.70052	1.729876	-2.219125	0.042861	0.128991	-4.311102	1.895723	0.812964	0.945338	1.548999	1.533923	0.846031	2.415815	1.775313	2.381582	2.274406	1.978199	1.869448	1.79348	2.660694
Scoc	-0.557047	7.158224	-2.218632	0.042901	0.129055	-5.003667	7.45124	6.462737	7.000455	7.871157	7.533334	7.418848	7.198279	6.982048	7.107123	6.829978	7.243066	6.857597	7.381995	6.888549
Cdkl2	-0.552764	5.445134	-2.218527	0.042909	0.129055	-4.991916	5.798336	5.136182	5.487608	6.289756	5.312258	6.028851	5.417651	5.178422	5.255296	4.847916	5.387099	5.145199	5.883878	5.100515
Fignl1	1.256687	-0.594611	2.218466	0.042914	0.129055	-3.936551	-1.22354	-0.572649	0.52603	-0.166914	0.649545	1.193889	-0.831021	-0.654381	-0.632503	-0.665882	-0.616402	-1.243945	-0.405292	-1.212031
Ccl12	4.342865	-3.545838	2.218235	0.042933	0.129079	-3.664112	-3.260591	-3.260591	-0.761158	-3.260591	1.20216	0.915838	-3.260591	-3.260591	-3.260591	-2.618976	-2.293564	-2.249472	-3.260591	-3.260591
Ift20	0.40601	5.442826	2.217836	0.042966	0.129145	-4.977874	5.545046	6.251146	5.515958	5.271951	5.842885	5.646694	5.44173	5.196815	5.213859	5.412451	5.263455	5.046893	5.223392	5.363965
Ctgf	0.658653	4.714157	2.217557	0.042989	0.129181	-4.939306	5.278315	5.56701	5.170091	4.291262	5.338601	5.654372	5.229287	3.386664	5.211521	4.320048	3.78171	3.951235	4.481425	4.403387
Pfas	0.524994	6.631815	2.217249	0.043014	0.129208	-4.996905	6.032885	6.905792	6.374224	5.564828	6.06917	5.938452	7.241318	6.884111	7.136989	7.062836	6.723284	6.879833	6.772416	7.276445
Ppp3r1	-0.955394	9.559928	-2.217184	0.043019	0.129208	-4.929487	9.813302	8.14696	9.501073	10.78388	9.482112	10.24687	9.523239	9.597508	9.553889	9.12948	9.799564	9.23805	9.823176	9.20213
Fam84a	-0.999656	6.349968	-2.215703	0.04314	0.129539	-5.012092	6.55921	4.801498	6.362208	7.730236	6.237862	7.167065	6.087204	6.587212	6.049883	6.071503	6.504073	6.380836	6.313769	6.068139
Fbxo41	-0.372777	6.605283	-2.21541	0.043164	0.129578	-5.010847	6.679574	6.244284	6.474155	6.373438	5.810314	6.084853	6.950785	6.794649	6.776483	6.730273	6.785843	6.854298	7.126625	6.805146
Osbpl5	0.361438	4.633545	2.21409	0.043272	0.12987	-4.897734	4.495729	5.060063	4.731936	4.249769	4.562251	4.576145	4.722163	4.564846	4.646003	4.66726	4.483567	4.683311	4.586106	4.904232
Map4k5	-0.359943	5.983094	-2.213306	0.043337	0.130031	-5.006975	5.869329	5.454283	5.878506	6.407642	5.826691	5.956163	6.258424	5.974888	6.169105	5.829078	6.009303	5.881759	6.184203	6.0891
Doc2b	-0.991337	5.527779	-2.212512	0.043402	0.130195	-4.970091	5.615043	2.991607	4.944066	5.676041	5.116828	5.502418	5.841165	6.161703	5.882203	5.703653	5.955889	5.692253	6.093166	6.25595
Ptprr	-0.627707	4.583142	-2.212312	0.043418	0.130195	-4.905551	4.495729	3.34683	4.485756	4.998953	3.935155	4.68261	4.633716	4.895992	4.812563	4.602796	4.493172	4.811327	4.987296	5.051519
Ippk	-0.511019	4.059603	-2.21222	0.043426	0.130195	-4.811053	4.012279	2.812308	3.863274	4.022255	3.500244	3.789543	4.35355	4.289273	4.433808	4.041554	4.441188	4.376159	4.595845	4.403387
Map10	-0.696116	1.01432	-2.212117	0.043434	0.130195	-4.226981	1.071521	0.426209	0.883381	1.478901	0.917619	0.231108	1.091998	1.429209	1.393785	0.750369	1.319276	1.593679	1.150071	1.260365
Efna2	-0.486314	2.753669	-2.210918	0.043533	0.130458	-4.563142	2.555898	1.833582	2.31067	2.87199	2.661692	2.131561	3.060831	3.059168	2.858731	3.080873	2.986432	3.065063	3.089114	3.229331
Cnrip1	-0.546732	6.923121	-2.210163	0.043596	0.130613	-5.020231	7.338835	6.412895	6.979323	7.770526	7.271032	7.366831	6.71429	6.741049	6.841413	6.467998	7.059262	6.445406	6.980019	6.547718
Nupr1l	-0.709735	2.337452	-2.209502	0.04365	0.130744	-4.542596	2.515609	1.117652	1.981598	2.881078	2.109124	2.753495	2.787802	2.454377	2.364852	2.655119	2.374443	2.170435	2.639729	2.245294
Amz2	-0.339939	6.886408	-2.209146	0.04368	0.130789	-5.0262	6.961612	6.526352	6.556487	7.171012	6.829434	6.993296	6.894799	6.88735	7.0015	6.759359	7.085311	6.778348	7.113126	6.865111
F10	1.896967	0.615773	2.209056	0.043687	0.130789	-3.951432	-0.217457	1.558038	0.20995	-1.388744	0.770579	1.136566	-1.73438	3.559057	-1.10526	3.12019	2.374443	4.032882	-1.047095	-0.748265
Dtx2	0.40585	3.65832	2.208749	0.043713	0.130833	-4.789721	3.664706	3.506122	4.153089	3.456176	4.090486	4.052313	3.40164	3.534475	3.548392	3.649853	3.56201	3.772618	3.48116	3.468514
Ercc1	0.296343	5.156382	2.208596	0.043725	0.130838	-4.987399	5.300221	5.444051	5.766051	5.331172	5.563257	5.668344	4.919005	4.968131	4.959085	4.919311	4.990317	4.597515	4.893023	4.914707
Dnajc17	0.443965	3.654553	2.207565	0.043811	0.131059	-4.780903	3.509242	4.240408	3.89109	3.462244	3.990197	3.606391	3.830902	3.676051	3.799353	3.615871	3.543639	3.496064	3.278395	3.35002
Fam72a	1.314726	0.150583	2.207324	0.043831	0.131059	-3.895104	-0.518419	-0.366503	-0.491181	-1.575022	0.562819	0.61372	1.135593	0.449407	0.512061	1.21727	0.998029	0.714338	0.375586	0.119416
Ppapdc2	-0.379493	6.196712	-2.207309	0.043832	0.131059	-5.022779	6.247964	5.805472	5.910893	6.532567	5.970496	6.358371	6.383483	6.266173	6.071954	6.200917	6.604237	6.29972	6.110254	6.013146
LOC499331	-0.447976	4.417459	-2.206975	0.04386	0.131072	-4.93896	4.630741	4.129856	4.263615	5.164405	4.762811	4.662444	4.425566	3.929599	4.31255	4.154751	4.404331	4.17506	4.640443	4.262873
Xpr1	-0.701072	5.005526	-2.20694	0.043863	0.131072	-4.978436	5.172161	4.046648	5.335399	5.791393	4.396609	5.324555	4.736391	5.204627	4.947896	4.719397	5.09285	5.173007	5.347714	4.839733
Adap1	-0.33871	7.705367	-2.206864	0.043869	0.131072	-5.023282	7.837406	7.616798	7.146491	7.489299	7.457988	7.078046	7.980005	7.898802	7.850178	7.951514	7.847022	7.939282	7.891551	7.898508
Phf3	-0.418197	6.680334	-2.206358	0.043911	0.131162	-5.030641	6.841803	6.504363	6.850658	7.555007	6.695078	7.06802	6.418098	6.628447	6.391887	6.442051	6.579653	6.673679	6.616107	6.275385
Celf2	-0.484183	6.957275	-2.206167	0.043927	0.131162	-5.030139	7.100879	6.594237	7.088036	7.513972	6.541961	7.067541	6.971232	7.161989	7.039552	6.653628	7.117755	6.733083	7.222987	6.607859
Hspa13	-0.590085	4.98476	-2.206111	0.043932	0.131162	-4.950922	4.859884	3.899656	4.940267	5.287413	4.140005	4.932509	5.003787	5.22781	5.317478	4.941431	5.03922	5.27917	5.589483	5.381063
Klk10	-1.333177	0.312991	-2.205577	0.043976	0.131262	-4.064625	-0.129637	-1.463779	-1.093563	1.454757	-0.18071	0.695385	0.282279	1.393205	0.884903	0.750369	1.068256	0.403657	1.150071	0.706485
Nckap1	-0.491628	9.307975	-2.204441	0.044071	0.131467	-4.97414	9.310561	8.483271	9.071881	9.777861	9.212161	9.443983	9.501145	9.300139	9.412491	9.062465	9.448311	9.240958	9.678399	9.370556
Arel1	-0.500261	7.019926	-2.204411	0.044073	0.131467	-5.03413	7.058273	6.028431	7.036928	7.210299	6.648457	6.820363	7.070036	7.314155	7.118396	6.866564	7.313882	7.205007	7.477274	7.123422
Dok4	-0.373921	4.510966	-2.204219	0.044089	0.131467	-4.941987	4.664912	4.095404	4.410874	5.038072	4.685386	4.724549	4.390007	4.351577	4.469458	4.524366	4.397527	4.444449	4.477919	4.548339
Thap4	-0.396492	4.536489	-2.204183	0.044092	0.131467	-4.958975	4.995452	4.240408	4.817254	5.097757	4.845505	4.685111	4.171806	4.455617	4.19443	4.298786	4.822678	4.342647	4.420622	4.19144
Ankrd17	-0.434027	7.74305	-2.204096	0.044099	0.131467	-5.019477	7.739133	7.50927	7.91701	8.45581	7.44857	7.774905	7.740294	7.805146	7.661002	7.370597	7.782552	7.593648	7.907931	7.704255

Gucy1b2	-0.786209	0.97932	-2.203839	0.044121	0.131498	-4.209832	1.245833	1.010791	-0.007587	1.065902	0.731352	-0.006501	1.511505	0.517885	1.579251	1.540753	1.03357	1.345607	1.465469	1.529341
Psma1	0.309634	6.55078	2.203185	0.044176	0.131629	-5.035208	6.564156	6.635285	6.897448	6.630792	7.121178	6.973995	6.45728	6.198678	6.453514	6.218007	6.500109	6.175157	6.504209	6.398042
Fbxl6	0.653097	4.633136	2.202673	0.044218	0.131724	-4.838606	4.055074	4.970229	4.136585	3.40669	4.296017	4.105563	5.268979	4.937208	5.050846	5.253565	4.730939	4.958703	4.505736	5.25689
Lyar	0.445136	4.124487	2.202181	0.04426	0.131813	-4.942145	4.406741	4.585778	4.923048	4.733936	5.101527	5.45191	3.545949	3.891392	3.35864	3.669864	3.66758	3.575147	3.262178	3.667444
Cxxc1	0.373176	6.225419	2.202053	0.04427	0.131813	-5.03005	6.036449	6.588829	6.389628	6.095557	6.584535	6.194246	6.208264	6.190834	6.236179	6.377603	6.034738	6.101377	5.773793	6.365003
Xpnpep1	0.260848	7.26589	2.201198	0.044342	0.131988	-5.037304	7.1206	7.45553	7.421797	7.28152	7.54434	7.424472	7.345933	7.2087	7.272098	7.175022	7.179045	6.954451	7.124944	7.22424
Rps6ka3	0.350326	4.733486	2.201093	0.044351	0.131988	-4.958372	4.519693	4.953495	5.30913	4.963029	4.995836	5.116089	4.893837	4.429191	4.765539	4.272852	4.313278	4.523011	4.749166	4.5246
Blvrb	-0.417669	3.957745	-2.200894	0.044368	0.132005	-4.896645	4.526467	3.894655	3.867281	4.314927	3.789984	4.452412	3.734959	3.734773	3.918782	4.020928	3.939921	3.61312	4.047497	3.655003
Cox7a2l	-0.399916	7.681875	-2.199894	0.044452	0.132222	-5.022012	8.128154	7.532393	7.723936	8.436291	7.940754	8.331079	7.503459	7.351574	7.461534	7.440215	7.551544	7.316615	7.48297	7.353542
Slitrk5	-0.941926	6.875761	-2.199359	0.044497	0.132323	-5.041132	6.674246	4.812145	6.499545	7.660369	6.574189	7.004354	7.391037	7.154615	7.323329	6.893052	6.989592	6.915727	7.062518	7.321425
Ociad2	0.339722	5.257011	2.198395	0.044578	0.132532	-5.000705	5.108122	5.309628	5.690049	5.357456	5.493166	5.795313	5.234309	4.886311	5.291156	5.044659	5.190547	4.885603	5.282878	5.070381
Tom1l1	-0.42138	3.678411	-2.198106	0.044602	0.132572	-4.865793	4.526467	3.570246	3.614277	3.815017	3.913413	3.857641	3.419386	3.567159	3.448717	3.595091	3.633241	3.941318	3.237505	3.482664
Tmem214	0.521646	6.846649	2.197626	0.044643	0.132659	-5.043166	6.599922	7.339346	7.072503	6.211766	6.915623	6.378599	7.119727	6.82706	7.106494	7.129383	6.683984	6.787372	6.569091	7.126241
Pgam5	0.309491	5.537351	2.197409	0.044661	0.132681	-5.015993	5.426916	5.796117	6.022889	5.510231	5.735463	5.55669	5.57635	5.331332	5.580969	5.328915	5.566136	5.304534	5.381888	5.438452
Serinc1	-0.719114	10.06295	-2.197132	0.044685	0.13269	-4.964117	9.850051	8.552569	9.594895	10.62578	9.840424	10.08987	10.33537	9.966243	10.52543	10.14886	9.998706	10.1526	10.41907	10.78304
Dcaf4	0.280405	4.341382	2.197111	0.044686	0.13269	-4.914161	4.280186	4.652466	4.443444	4.281	4.576156	4.576145	4.233294	4.294162	4.203867	4.505931	4.208334	4.316033	4.103184	4.182799
Trmt44	0.497721	3.543291	2.19666	0.044725	0.132769	-4.733997	3.118917	4.086661	3.776458	3.122426	3.293596	3.3278	3.749062	3.698353	3.824043	3.696121	3.518775	3.461789	3.445698	3.62342
Gabrr3	-1.842432	-0.277158	-2.196534	0.044735	0.132769	-3.945865	0.176305	-3.260591	-0.575682	0.105385	-1.235601	0.117189	1.297885	0.705741	0.449965	0.134646	0.584127	0.28321	0.375586	0.442608
Rnf144a	-0.475418	3.64492	-2.196152	0.044768	0.132811	-4.891092	4.197708	3.7962	3.98258	4.479531	3.574163	4.113012	3.496648	3.321106	3.341641	3.139454	3.057213	3.674268	3.934354	3.050898
Pnrc2	0.33726	5.550104	2.196109	0.044771	0.132811	-5.035963	5.908752	5.893325	5.61448	5.880183	6.436913	6.418225	4.868222	5.287872	4.902249	5.14338	5.416002	5.344586	5.134872	4.927694
Ash1l	-0.566581	7.521211	-2.195478	0.044825	0.132936	-5.040118	7.43468	7.003669	7.563116	8.336387	7.031677	7.673069	7.55052	7.695778	7.389957	7.224907	7.636705	7.554745	7.888583	7.321917
Dlx2	-0.707483	1.298924	-2.194863	0.044877	0.133058	-4.345765	1.278303	1.339943	1.004744	2.077719	1.018831	0.734546	1.135593	1.239467	1.255413	1.451089	1.102128	1.404538	2.133689	1.659607
Adra1d	-1.468743	6.487501	-2.194597	0.044899	0.133058	-5.034412	6.427881	2.667383	5.641746	6.916957	6.563061	5.883978	7.08053	7.031927	7.341079	6.97039	6.896146	6.978894	7.164747	7.292599
Slc2a12	-1.28543	3.169917	-2.194519	0.044906	0.133058	-4.795291	5.485452	2.200694	2.588191	2.768056	2.575715	3.808019	2.717819	2.988532	2.928701	3.352542	4.111876	3.046681	2.913344	3.088394
Heatr2	0.373314	4.055815	2.194473	0.04491	0.133058	-4.880083	4.239536	4.302134	4.284739	3.706736	4.57061	4.185462	4.074361	3.871902	3.799353	3.88487	3.949256	4.140807	3.908538	3.958175
Niacr1	0.8735	1.209156	2.19382	0.044966	0.13319	-4.342471	1.143792	1.339943	2.466695	1.245516	2.07819	2.217002	-0.672244	1.934092	0.317129	1.253084	1.375754	2.300247	0.546019	0.190106
Nr2c2	-0.707854	4.969715	-2.193664	0.044979	0.133196	-5.004281	5.350716	4.361326	5.462667	5.694201	4.316041	5.09931	4.980068	5.013306	4.790809	4.332655	5.126156	4.961159	5.561594	4.578298
Fdx1	0.426667	3.307091	2.193235	0.045015	0.133272	-4.726233	2.937667	3.643613	3.442311	3.197095	3.384903	3.53054	3.454237	3.492551	3.271566	3.302147	3.493474	2.859272	3.195422	3.25429
Snx10	-0.624524	6.324496	-2.192798	0.045053	0.133349	-5.053944	6.686396	5.681759	6.605881	7.33582	6.449054	6.806072	6.322308	5.997547	6.170309	5.693914	6.294829	5.868738	6.684637	5.96608
Insig1	-0.49554	4.310141	-2.192275	0.045097	0.133422	-4.916605	4.611755	4.095404	4.251403	4.367604	4.162299	3.462191	4.587372	3.705711	4.730053	3.994723	4.212217	4.412503	5.033414	4.797905
Ren	1.299618	0.359736	2.192183	0.045105	0.133422	-3.98818	-0.634519	0.812964	0.442561	-0.705589	1.463853	-0.548781	0.429655	1.393205	-0.386063	1.104165	1.167576	1.593679	0.249639	0.045082
Zc2hc1a	-0.675722	4.009556	-2.192119	0.04511	0.133422	-4.967951	4.59574	3.828023	4.472666	5.458049	4.165982	4.945084	3.577905	3.668539	3.655446	3.060806	3.981461	3.46871	4.156802	3.203933
Cacna1e	-0.912198	7.069583	-2.191821	0.045136	0.133464	-5.054127	6.497627	6.122324	6.764667	7.597666	5.167305	6.485054	7.605814	8.001022	7.725082	6.898009	7.689532	7.29	8.025188	7.118899
Idh3a	-0.483609	7.985422	-2.19155	0.045159	0.133499	-5.028054	8.43582	7.586497	8.219304	8.789012	8.350793	8.360519	7.714921	7.817934	7.748648	7.479011	8.027793	7.612908	8.029985	7.629121
Pkmyt1	0.804545	2.814384	2.191117	0.045196	0.133576	-4.616542	2.730329	3.848855	2.423812	2.108996	3.563034	3.069038	2.746221	2.822481	2.330799	3.12019	2.673757	2.880186	2.363894	2.952659
Mphosph8	-0.458006	6.453792	-2.190785	0.045224	0.133627	-5.057428	6.519014	5.935323	6.500191	7.207593	6.483401	6.701865	6.477539	6.538597	6.454503	6.191719	6.343431	6.215829	6.518787	6.283491
Smim17	-0.624903	1.323133	-2.18878	0.045396	0.134101	-4.405379	1.514695	1.310208	1.472789	2.227735	1.143601	1.193889	1.544223	1.429209	1.60797	1.064403	0.885831	0.760186	1.922909	1.085968
Rbpms2	0.436198	3.000101	2.188319	0.045436	0.134185	-4.757913	3.516065	3.763659	3.540923	2.862845	3.568609	3.568964	2.343298	2.539194	2.759824	2.988286	2.930971	2.668085	2.408783	2.745289
Pdgfrb	0.589569	6.399335	2.187568	0.0455	0.134343	-5.050056	6.251035	6.875022	6.408997	5.397589	6.399083	5.957198	6.627254	6.321101	6.613433	6.87449	6.339886	6.702586	6.14162	6.700822
Caskin1	-0.323959	8.514514	-2.187235	0.045529	0.134394	-5.035286	8.527313	8.592489	8.190612	8.621372	8.132684	8.080342	8.778697	8.476044	8.737982	8.671033	8.430517	8.53593	8.599306	8.833244
Grb14	-0.453826	5.433245	-2.186684	0.045576	0.134493	-5.029592	5.430532	5.108376	4.764524	5.799803	5.567439	5.226661	5.841165	5.434577	5.730483	5.613537	5.410943	5.523062	5.266712	5.384835
LOC314140	0.532883	2.624909	2.186549	0.045588	0.134493	-4.6795	2.445855	3.046741	2.947351	3.051752	3.470774	3.703341	2.12275	2.419003	2.185932	2.165862	2.651054	2.404977	2.168911	2.228598
Pou3f3	-0.382624	6.309061	-2.186456	0.045596	0.134493	-5.061286	6.383796	6.015821	5.848419	6.633483	6.181673	6.461905	6.460498	6.295728	6.235029	6.405719	6.120905	6.455021	6.226609	6.622311
Gpr27	-0.607032	3.046997	-2.186248	0.045614	0.134513	-4.564198	2.670424	1.339943	2.025285	2.475838	2.139408	2.323579	3.601415	3.645768	4.160904	3.344264	3.678847	3.827486	3.754753	3.901371
LOC500300	1.042738	0.973889	2.185738	0.045658	0.134578	-4.285404	0.955837	2.545069	2.10888	0.705659	1.600747	1.136566	0.68572	1.067371	1.549949	0.475109	0.378374	0.804622	-0.305233	0.799858
Taf10	0.286405	5.907623	2.185658	0.045665	0.134578	-5.055061	6.043551	6.170698	6.066232	5.821207	6.421592	6.212565	5.704911	5.702559	5.919913	5.762302	5.809803	5.693731	5.621619	5.782385
Hrasls	-0.525088	0.710502	-2.185605	0.045669	0.134578	-4.342484	1.568201	0.973336	1.004744	1.502648	1.082564	1.046069	0.118118	0.222004	0.571595	0.534523	0.631286	0.28321	0.879431	0.499386
Runx2	0.517584	2.91101	2.185244	0.0457	0.13458	-4.606407	2.387517	2.822876	2.837276	2.401998	2.781752	3.179592	3.148563	3.466799	3.140097	3.232094	2.90242	3.118845	2.535634	3.012401
Slc12a2	-0.419012	6.732131	-2.185087	0.045714	0.13458	-5.068242	7.019782	6.383749	6.542774	7.039205	6.776822	6.739049	6.543758	6.526183	6.477057	6.546763	6.602759	6.975254	7.120453	6.971162
Syt12	-0.427489	4.715333	-2.185019	0.04572	0.13458	-4.914213	4.460789	3.52566	4.153089	4.242735	4.20588	3.775529	5.050088	5.170467	5.255296	4.957801	5.32032	5.177238	5.42623	5.360138
Adipoq	-4.910797	-2.972809	-2.184973	0.045724	0.13458	-3.789997	3.109925	-3.260591	-3.260591	-2.731185	-3.260591	-3.260591	-2.558588	-2.550306	-3.260591	0.280604	2.684976	-2.249472	-3.260591	-1.632384
Mapk15	0.988842	2.939227	2.184946	0.045726	0.13458	-4.557188	3.05476	3.397198	3.222225	1.097434	2.883666	2.481552	3.36548	3.340143	3.140097	2.628098	2.580719	3.475597	3.169571	3.544677
Pigy	-0.347397	5.065999	-2.184745	0.045744	0.134598	-5.027993	5.466203	5.039823	4.959161	5.526249	5.370867	5.231803	5.015502	4.958923	4.846099	4.683756	5.164734	4.752996	5.134872	4.820364
Mab21l1	0.96546	-0.166408	2.184078	0.045801	0.134735	-4.207152	0.915118	1.632013	1.966737	0.041976	1.285769	0.61372	-1.21312	-0.654381	-1.10526	-0.665882	-0.731011	-0.434228	-0.211665	-1.632384
Taf6l	0.513763	4.570627	2.183931	0.045814	0.13474	-4.902287	4.172016	4.953495	4.380359	3.756964	4.519714	4.048433	4.874668	4.666384	4.824848	5.016284	4.606661	4.778284	4.488413	4.970998
Bicd1	-0.791613	4.134666	-2.183633	0.04584	0.134782	-4.943672	4.172016	3.63764	4.272706	5.252825	3.326873	4.469923	4.05746	4.35626	4.101553	3.478908	4.345431	3.794816	4.811788	3.901371
RGD1564719	0.54876	1.809463	2.183401	0.04586	0.134808	-4.431453	1.874641	2.232748	2.250588	1.327578	1.981199	2.025031	1.938115	1.532126	1.60797	2.14695	1.959843	1.760874	1.700256	1.445134
Cxxc4	-0.457622	4.823587	-2.183269	0.045871	0.134809	-5.032933	5.262171	4.741493	5.083193	5.88922	5.214297	5.443072	4.57555	4.198308	4.496589	4.294496	4.549498	4.229042	5.02623	4.584872
Wrnip1	-0.298339	5.293931	-2.182645	0.045925	0.134935	-5.026498	5.41236	4.953495	5.142254	5.389651	5.233727	5.078533	5.261619	5.443407	5.523715	5.224545	5.527707	5.183562	5.449691	5.331105
Galnt14	-0.488348	4.81812	-2.182007	0.045981	0.135065	-4.976881	4.464322	4.777251	4.421813	5.238754	4.299374	4.013036	5.097706	5.05423	5.410079	4.633691	4.946707	4.769904	5.165713	5.219356
Dusp7	-0.323795	6.893053	-2.181053	0.046064	0.135275	-5.072788	7.233064	6.751165	7.086748	7.567727	7.323083	7.144525	6.583652	6.741944	6.596515	6.708671	6.871808	6.774862	6.53912	6.593261
Tlcd1	-0.618464	4.019979	-2.180676	0.046096	0.135338	-4.886951	4.805223	3.758163	3.535898	3.578533	3.76592	3.186669	4.306644	3.614836	4.37654	4.349296	4.043791	4.158035	4.394821	4.5074
Strn	-0.586037	5.79592	-2.17987	0.046167	0.135508	-5.068302	6.001624	5.731683	6.007541	6.718958	5.251166	6.11113	5.64395	6.035999	5.591793	5.188006	5.576715	5.776934	6.035891	5.500615
Zfp414	0.370394	5.466774	2.179755	0.046177	0.135508	-5.048126	5.574761	5.959469	5.915737	5.389651	5.9726	5.55669	5.363679	5.312177	5.362416	5.360041	5.345284	5.140873	4.86645	5.4511
Fbxo3	-0.260699	6.052682	-2.17947	0.046201	0.135548	-5.073033	6.263253	6.255053	6.124304	6.604971	6.144827	6.173912	6.066236	5.786494	5.955267	5.870017	5.858922	5.692253	5.99477	5.971108
Cyb5r3	0.287845	7.137734	2.17922	0.046223	0.135578	-5.074242	7.495068	7.485356	7.597591	7.086621	7.722756	7.511597	6.648099	7.002356	6.875769	6.921151	7.003661	7.049256	6.7303	6.810064
Lrrc59	0.357777	6.881881	2.178772	0.046262	0.135654	-5.07821	6.806059	6.952255	7.389775	7.056856	7.382055	7.434912	6.496472	6.670417	6.522078	6.590288	6.948288	6.745245	6.849743	6.515391
Secisbp2	0.340386	4.840177	2.178668	0.046271	0.135654	-4.982912	4.751303	5.05559	4.930726	4.433945	4.753051	4.976052	4.955952	4.806432	4.843082	5.109591	4.927943	4.94388	4.512607	4.817576
Vamp4	-0.328278	6.022822	-2.177302	0.046391	0.135971	-5.075524	6.141674	5.935323	6.053981	6.624042	5.975751	6.25695	6.047814	5.817389	5.883671	5.986294	5.952411	5.750202	6.083969	5.834346
Hipk3	-0.59975	5.36569	-2.176918	0.046425	0.136006	-5.063444	5.739788	5.097537	5.766051	6.321812	4.919359	5.936354	5.157085	5.165139	5.239322	4.66726	5.192513	5.235205	5.665438	5.056258
Aadat	0.562917	3.917112	2.176774	0.046437	0.136006	-4.9496	4.391979	5.41815	4.511585	4.038591	4.901799	4.284446	3.29032	3.466799	3.027678	3.844356	3.434871	3.916225	3.107385	3.31881
Lingo3	-0.844178	3.977155	-2.176706	0.046443	0.136006	-4.762359	3.881004	1.425638	3.495051	3.247155	2.530726	3.248852	4.788531	4.771798	4.896441	4.509637	4.770972	4.936411	4.725704	4.591416
Ncoa1	-0.431279	7.610005	-2.176648	0.046448	0.136006	-5.072179	7.663173	7.154045	7.661569	8.108729	7.395057	7.607998	7.513821	7.81666	7.408952	7.422631	7.895212	7.515183	7.909884	7.475301
Stx2	0.587652	4.549005	2.175276	0.046569	0.136253	-4.902228	4.017097	4.982063	4.402616	3.675737	4.12116	4.220367	5.052933	5.010338	5.019122	4.908123	4.397527	4.562362	4.43154	4.955862
Riiad1	-0.589128	1.467151	-2.174993	0.046593	0.136365	-4.429753	2.10932	1.184734	1.293837	1.63746	1.258434	1.402804	2.206902	1.393205	1.360416	1.104165	1.740817	1.345607	1.493524	1.582867
Stx6	-0.417034	7.199493	-2.174802	0.04661	0.136381	-5.082509	7.422474	6.548807	7.311803	7.58347	7.061638	7.423724	6.998789	7.292939	7.129581	7.13179	7.278214	7.235758	7.287507	7.09722
Nde1	0.720086	3.047439	2.174132	0.046669	0.13652	-4.702065	3.035892	3.833259	2.977318	2.389316	3.913413	2.982217	2.854546	2.926907	2.583064	2.807409	3.493474	3.30822	2.639729	3.11589
Tmed10	0.394111	6.399995	2.173853	0.046694	0.136559	-5.079183	6.099138	6.432004	6.356519	6.011156	6.556062	6.45019	5.832912	6.597137	6.285903	6.508381	6.590879	6.892112	6.65389	6.352537
Ephx2	0.824386	1.686829	2.173518	0.046723	0.136612	-4.305683	1.143792	1.812527	1.996308	0.621404	1.622351	1.221719	2.467906	1.49863	2.166756	2.165862	1.996325	1.618784	1.521045	2.278116
Gfer	0.392401	3.493553	2.172627	0.046802	0.136788	-4.86151	3.827152	4.091039	4.179109	3.400384	3.917788	3.789543	2.991343	3.159031	3.178898	3.385186	3.568082	3.412394	3.294432	2.858134
Itm2a	-0.86686	6.231549	-2.172575	0.046806	0.136788	-5.084798	7.026966	5.938971	4.605721	6.39976	6.664165	6.19161	6.611901	5.844307	6.774901	6.238251	5.706244	6.177271	6.265772	6.819153
Mdm2	0.315578	5.58048	2.172153	0.046844	0.136859	-5.067294	5.546713	5.861645	5.961432	5.677346	5.706678	6.181017	5.630681	5.400976	5.241615	5.434063	5.441032	5.417931	5.262642	5.396093
Ero1l	-0.497949	5.528124	-2.172042	0.046854	0.136859	-5.068767	5.553362	5.046602	5.708032	6.251726	5.143221	5.719261	5.450387	5.559634	5.473878	5.162339	5.510468	5.475754	5.867892	5.50582
Rdh10	0.412156	3.255353	2.170292	0.047008	0.137278	-4.720352	3.109925	3.117121	3.145118	2.591079	3.365818	3.353184	3.537848	3.126505	3.383769	3.186517	3.273307	3.482452	3.445698	3.62342
Col3a1	1.493008	4.162957	2.169918	0.047042	0.137342	-4.889926	5.132748	4.037603	4.87411	2.029493	4.685386	6.34578	3.727855	4.224157	3.648551	4.21501	3.925804	4.200226	3.061266	4.283226
Eif2s1	0.381684	5.439567	2.168651	0.047154	0.137632	-5.076985	5.558328	5.727471	6.092997	5.815293	6.42774	6.02688	5.009657	4.81326	5.134654	5.07749	5.221695	4.985489	5.294885	5.005709
Traf3	-0.37444	6.327367	-2.168519	0.047166	0.137632	-5.091516	6.714847	5.919409	6.062743	6.16974	6.059303	6.226368	6.541734	6.133337	6.289232	6.347893	6.241687	6.437491	6.820278	6.638991
Dzip1l	-0.345849	5.242167	-2.16841	0.047176	0.137632	-5.046045	5.331503	5.155123	5.076287	5.352564	4.755497	5.004367	5.592083	5.35258	5.454242	5.54657	5.215906	5.143038	5.139318	5.313401
Psmd4	0.278666	6.905472	2.167838	0.047226	0.137714	-5.097801	6.917197	7.265734	7.060742	6.847955	7.224557	7.092249	6.813251	6.791195	6.983804	6.911379	6.801391	6.704786	6.596838	6.678713
Pag1	0.915654	0.879269	2.167783	0.047231	0.137714	-4.167053	-0.046857	1.425638	0.945338	0.33436	0.649545	1.107027	0.202532	1.318383	1.061401	0.893396	1.430105	1.989793	0.962073	0.929479
Zmym4	-0.31541	6.167837	-2.16765	0.047243	0.137714	-5.089522	5.982044	5.904562	5.87453	6.484931	5.816184	6.082958	6.462639	6.246128	6.258994	6.047485	6.313925	6.176214	6.370658	6.350609
Tbc1d22b	-0.288063	6.369001	-2.167582	0.047249	0.137714	-5.095503	6.4521	6.01467	6.246233	6.674568	6.386512	6.454594	6.341051	6.407114	6.381528	6.420559	6.417604	6.244338	6.371603	6.372622
Polr3c	0.264232	5.317129	2.16744	0.047262	0.137717	-5.055205	5.126074	5.647985	5.57104	5.391242	5.39617	5.485299	5.281162	5.207221	5.197417	5.257979	5.202306	5.16024	5.206541	5.348595
Atp5a1	-0.279895	9.062399	-2.166647	0.047332	0.137863	-5.052073	9.155461	8.65	8.899683	9.124448	9.016735	8.877651	9.261783	9.049434	9.277805	8.966291	9.215371	8.932889	9.25115	9.197848
Wdr43	0.400792	5.283953	2.166605	0.047336	0.137863	-5.064977	5.19147	5.309628	6.04251	5.537788	5.745343	5.962362	4.986034	5.204627	5.066451	4.844986	5.140489	5.076641	4.944745	4.96345
Cpne6	-0.608255	9.233351	-2.16649	0.047346	0.137863	-5.045996	9.273919	8.026701	8.787082	9.574766	9.534239	8.921265	9.515783	9.353813	9.68213	9.245423	9.348107	9.08925	9.376147	9.541037
Shisa7	-0.325707	8.983956	-2.165736	0.047414	0.138026	-5.064167	8.710801	8.269355	8.573487	8.980596	8.503033	8.734223	9.415037	9.19721	9.430817	9.224857	9.13111	9.039135	9.117249	9.451697
Fam210a	-0.325296	5.698555	-2.165407	0.047443	0.138078	-5.082095	5.677242	5.462754	5.646417	6.059988	5.428721	5.640264	5.831256	5.678453	5.553549	5.573377	5.95125	5.704031	5.875907	5.726997
Nat14	-0.462985	5.461022	-2.165167	0.047465	0.138107	-5.057609	5.393957	4.445797	4.907568	5.552083	5.287117	5.393466	5.454696	5.537125	5.834394	5.669274	5.851474	5.656333	5.81884	5.689362
Mtmr12	-0.580394	4.642009	-2.164866	0.047492	0.138152	-4.976545	4.29613	3.914553	4.438066	4.945809	3.372207	4.449473	5.072692	5.183701	4.97846	4.879766	4.978968	4.805872	5.057107	4.683115
Armc5	0.40628	6.310515	2.164477	0.047526	0.138211	-5.095205	6.28339	6.742146	6.508552	5.795003	6.380978	6.144226	6.258424	6.399194	6.248772	6.522206	6.287488	6.460239	5.891806	6.444869
Nhlh2	-1.007227	0.53103	-2.164382	0.047535	0.138211	-4.324676	0.873216	-0.273517	0.750864	2.077719	0.731352	0.881356	0.282279	-0.511025	0.683763	0.280604	0.805871	0.804622	1.316372	-0.116166
Snx30	-0.623139	4.699898	-2.164218	0.04755	0.138221	-5.006307	4.64948	3.702025	4.766671	5.155071	4.002607	4.623856	4.771359	5.157111	4.849109	4.357545	5.10543	4.851593	5.180889	4.689229
Gmpr	-0.286499	6.099474	-2.164084	0.047562	0.138222	-5.096398	6.375378	5.947447	5.984671	6.135026	6.136405	5.805694	6.102389	6.192145	6.03806	6.247158	6.405786	6.312278	5.911436	5.821878
Gulp1	-0.772781	2.38777	-2.163442	0.047619	0.138356	-4.780965	4.114729	2.184396	2.31067	2.890108	2.828971	3.49677	2.05625	1.747064	1.549949	1.780488	2.580719	2.284645	2.060555	1.868007
Rbm43	0.74078	2.464131	2.163205	0.04764	0.138364	-4.597983	2.582145	3.570246	2.770386	1.679737	2.564599	2.504391	2.227194	2.709903	2.39812	3.040455	2.442128	2.447635	1.770732	2.087586
Tceal1	-0.582905	4.673204	-2.163153	0.047645	0.138364	-5.030294	4.851815	3.833259	4.352047	5.383269	5.010247	4.945084	4.791941	4.600808	4.821787	4.349296	4.81759	4.338875	4.833902	4.558395
Mad2l1bp	0.512424	3.00042	2.162803	0.047677	0.138422	-4.718766	3.073383	2.790938	3.436929	2.43939	3.476716	3.242073	2.828218	3.262441	2.785194	2.998871	3.273307	2.980434	2.994118	2.622863
Nap1l4	-0.3081	6.759206	-2.161506	0.047793	0.138727	-5.109469	6.865372	6.581069	6.924138	7.259911	6.890247	6.622535	6.643388	6.647676	6.679189	6.633388	6.81422	6.633443	6.803566	6.645295
Ppp1r3e	-1.127231	0.489404	-2.161111	0.047829	0.138797	-4.179013	-0.129637	0.310827	-0.761158	1.679737	-0.613814	0.231108	1.21902	0.517885	1.180909	0.413143	1.290185	0.567345	1.407665	0.799858
Cd27	1.076528	-0.298579	2.16037	0.047895	0.138957	-4.08117	-0.634519	1.010791	0.353965	-0.166914	-0.038319	1.165512	-1.009457	0.222004	-0.504027	-1.136114	-0.231835	-0.24119	-0.755361	-0.204094
Rabep1	-0.350192	7.365066	-2.159858	0.047942	0.139029	-5.104217	7.481187	7.184069	7.2487	7.966558	7.351658	7.706999	7.333118	7.306311	7.163229	7.249495	7.274506	7.298279	7.391811	7.164598
Tor3a	0.549206	3.845254	2.15984	0.047943	0.139029	-4.825862	3.536344	4.317161	3.759312	2.977436	3.716556	3.402651	3.769961	4.055878	3.774234	4.12123	4.136602	4.597515	3.98464	3.797658
Paqr6	-0.676119	4.333444	-2.159351	0.047987	0.139121	-4.927009	4.536569	3.948728	3.319014	3.920151	3.994336	2.974059	4.53949	4.628172	4.867041	4.982013	4.287749	4.805872	4.621498	5.331105
Aebp2	-0.479339	3.94522	-2.15923	0.047998	0.139121	-4.96744	4.193457	3.588941	3.989949	4.667211	3.799498	4.434686	3.616879	3.997121	4.002283	3.595091	3.83304	3.961064	4.066299	3.591131
Lbp	3.827551	-0.137817	2.158867	0.048031	0.139173	-4.093568	2.10932	1.010791	3.89502	-2.731185	1.43972	4.724549	-1.009457	-0.654381	-2.604521	0.534523	-1.500575	-0.153565	-1.904936	-1.212031
Uck2	0.616156	3.103487	2.158775	0.04804	0.139173	-4.753921	2.957854	2.963229	3.887149	2.77782	3.122223	3.857641	2.73209	3.221962	2.759824	3.040455	3.345785	3.018661	3.302384	2.648193
Ccdc8	0.419334	2.80466	2.158392	0.048074	0.13924	-4.729606	3.035892	3.375827	3.049621	2.546081	3.188895	3.186669	2.550752	3.047633	2.462438	2.641672	2.696109	2.921125	2.562371	2.228598
Fto	-0.432366	5.703998	-2.157949	0.048114	0.139323	-5.095974	5.968427	5.226505	5.608623	5.909347	5.6394	5.545723	5.404346	5.919009	5.573708	5.445717	6.001472	5.872657	6.149356	5.622242
Zdhhc24	-0.404668	3.542883	-2.157682	0.048138	0.139359	-4.73436	3.073383	2.466372	2.735741	3.059778	2.874693	2.570814	3.939429	4.229271	4.146293	3.994723	4.140682	4.028205	4.116777	4.377115
RGD1302996	-0.305377	6.170145	-2.157537	0.048152	0.139364	-5.111975	6.403249	6.328272	6.169622	6.599476	6.206916	6.082958	6.111969	6.019856	6.272511	6.115944	6.138354	5.858236	5.85308	6.243539
Dnajb14	-0.658026	4.223456	-2.157073	0.048194	0.139452	-4.963899	3.997728	3.785434	4.026238	4.895125	3.568609	3.821723	4.213088	4.68139	4.417676	3.715505	4.316888	4.465359	5.098798	4.212819
Snx22	-1.050431	1.195989	-2.156841	0.048215	0.139479	-4.294601	1.48718	1.506527	0.945338	1.097434	-0.424712	-0.750296	1.938115	1.68885	1.549949	1.625169	1.347792	1.97042	1.859646	1.755962
Hivep1	-0.357604	4.730658	-2.155418	0.048344	0.13982	-5.065536	5.270265	5.220523	4.899765	5.334484	4.784532	4.889781	4.390007	4.112335	4.393136	4.585342	4.404331	4.73014	4.579577	4.695317
Poglut1	0.333361	5.337399	2.154701	0.048409	0.139943	-5.053271	4.960861	5.454283	5.123951	4.858787	5.258083	5.138162	5.737107	5.099622	5.6132	5.519255	5.39566	5.528039	5.420761	5.655412
Grik1	-0.505863	4.340916	-2.154693	0.04841	0.139943	-4.984093	4.255933	4.57956	3.550922	4.746422	3.96082	3.980827	4.736391	4.193082	4.526983	4.502215	4.043791	4.261285	4.592606	4.922513

Rbm7	0.315379	4.948277	2.154119	0.048462	0.140048	-5.063326	4.975787	5.414661	5.397668	5.086016	5.338601	5.236926	4.855242	4.816662	4.881816	4.585342	4.974403	4.665351	4.637302	4.461699
Rap1gap2	-0.47727	7.57641	-2.154038	0.04847	0.140048	-5.112839	7.919472	7.796767	7.649115	7.748351	6.758646	7.224073	7.854358	7.386904	7.581779	7.611229	7.47282	7.664301	7.665911	7.744448
Nfkbib	0.309876	4.626202	2.153773	0.048494	0.140084	-5.059729	4.96336	5.175881	5.496689	4.99058	5.345112	5.121639	3.914618	4.274505	4.12658	4.290193	4.627162	4.383501	3.949625	4.174106
Twsg1	-0.582259	2.864975	-2.153424	0.048526	0.140143	-4.748973	3.303772	2.100024	3.171281	3.051752	2.183675	2.845805	2.867532	3.036005	2.917273	2.586593	3.116419	3.083214	3.220819	2.847239
Wdr4	0.505202	3.141131	2.153087	0.048557	0.140198	-4.755343	3.180343	3.582736	3.319014	2.523044	3.320278	3.143674	2.955303	2.901501	3.333065	3.344264	3.408041	2.970717	2.871195	3.302949
Dpysl2	-0.507247	9.17553	-2.152725	0.04859	0.14026	-5.06788	9.092658	8.479728	8.800109	9.805867	9.084578	9.397177	9.152296	9.16621	9.226495	9.013385	9.236852	9.279473	9.55226	9.173112
Jag1	0.551207	3.278201	2.152137	0.048643	0.140381	-4.768854	2.947796	3.375827	3.283475	2.844379	3.488528	3.616909	4.166561	2.939445	3.341641	3.28495	2.986432	3.170693	3.042398	3.571405
Nell2	-0.748161	10.50588	-2.151468	0.048705	0.140508	-5.04108	10.2081	8.364651	9.771762	10.41827	10.06787	10.07284	10.96982	11.08871	11.08682	10.98931	10.91393	10.99989	11.0184	11.1132
Lif	2.292365	-1.295275	2.151401	0.048711	0.140508	-3.936776	-1.22254	-1.271663	2.175023	-1.788967	1.018831	-0.1418	-1.450335	-1.435005	-1.536754	-1.838237	-1.142289	-0.920914	-1.413173	-2.653844
Prdx6	-0.386813	7.013854	-2.150959	0.048751	0.140549	-5.123446	7.707812	7.00483	6.979786	7.34857	7.638128	6.946114	6.612866	6.842161	6.773317	6.841052	6.952937	6.995162	6.847707	6.715874
Rasgrf1	-0.368898	9.934029	-2.150863	0.04876	0.140549	-5.040919	10.01109	9.473032	10.10106	10.43659	9.911518	9.93569	9.915837	9.924382	10.04414	9.74425	9.767106	9.709469	10.02671	10.07715
Gpd1l	-0.493781	4.284225	-2.150863	0.04876	0.140549	-5.005823	4.608566	3.758163	4.438066	4.654018	4.078813	4.251076	4.063116	4.161323	4.413615	3.856048	4.451079	4.342647	4.863765	4.120817
Aoc3	-0.89772	0.85144	-2.149606	0.048875	0.140821	-4.298238	1.619793	0.118359	-0.067513	0.785264	0.320024	0.949514	1.913281	0.301855	1.426399	1.355481	1.135223	0.714338	0.836265	1.445134
Met	0.906864	3.564504	2.149579	0.048878	0.140821	-4.77161	3.064101	2.519311	4.419086	2.568755	3.590696	4.164107	3.804135	3.645768	4.023757	3.463704	3.621611	3.38339	3.754753	4.022545
RGD1565166	-0.767683	1.848489	-2.149171	0.048915	0.140868	-4.54325	2.620642	1.425638	1.723223	1.892408	1.643636	1.136566	2.415815	1.155984	2.870631	1.804823	1.403186	1.890216	1.626159	2.733504
Fam57b	-0.306098	6.200869	-2.148924	0.048938	0.140868	-5.126559	6.477667	6.464425	6.24161	6.479706	6.00996	5.977744	6.258424	6.056287	6.051191	6.207777	6.089588	6.136567	6.085122	6.297567
Sult1d1	-0.549277	3.292604	-2.148889	0.048941	0.140868	-4.934878	4.485336	3.302211	3.834912	3.752019	3.378569	3.68846	3.30948	1.983359	3.09008	3.050666	2.303426	2.970717	3.536146	3.584586
Zkscan1	-0.72967	4.553828	-2.148775	0.048952	0.140868	-5.058364	4.779933	4.138342	5.043022	5.660292	3.948045	4.832273	4.171806	4.70361	4.441807	3.940842	4.664481	4.472262	5.023827	4.003043
Tango2	-0.272648	6.417253	-2.148649	0.048963	0.140868	-5.12915	6.651961	6.51173	6.349375	6.583559	6.326082	6.195124	6.575761	6.268659	6.591131	6.342708	6.428492	6.077808	6.489482	6.468169
Mcm8	0.727346	2.623995	2.148638	0.048964	0.140868	-4.626907	2.035255	3.486315	3.118472	2.255954	2.198134	2.705027	2.867532	2.939445	2.694377	2.819403	2.604547	2.390473	2.186205	2.697558
Cenpu	1.168109	-0.378121	2.148394	0.048987	0.140899	-4.091682	0.031429	1.184734	-0.263851	-0.941548	0.808768	0.772673	-0.831021	-0.380636	-0.773548	-0.806258	-0.991788	0.34469	-0.893866	-0.397871
Nsg1	-0.512159	7.933336	-2.14709	0.049107	0.141211	-5.116711	8.032648	6.951654	8.011906	8.381712	7.839764	7.982066	7.782043	7.903209	8.088859	7.950833	7.971353	7.984347	8.151622	8.041282
Aebp1	0.898899	6.149257	2.146102	0.049198	0.14142	-5.114685	6.541762	6.455964	6.462946	4.638025	6.127934	6.851313	5.083863	7.222231	5.434336	6.738192	6.264302	7.430432	5.091933	5.77234
Ptger1	0.653512	3.62542	2.146047	0.049203	0.14142	-4.775629	3.016775	3.874479	3.359399	2.687469	3.770765	3.006419	4.39898	3.567159	3.991425	4.07195	3.549789	3.848862	3.556237	4.19144
Alg8	0.405316	3.870061	2.145113	0.04929	0.141635	-4.912188	3.582581	4.350411	3.89502	3.665254	4.098216	3.798811	3.993741	3.749088	4.044916	3.924277	3.627438	3.906064	3.602057	4.051312
Gsta4	-0.746971	2.800242	-2.144716	0.049327	0.141707	-4.714413	3.788223	2.294795	2.025285	2.227735	2.240661	2.386402	3.105363	3.301815	2.639794	3.293574	3.273307	2.794648	2.794335	3.278825
Tm2d3	-0.341415	3.262238	-2.144558	0.049341	0.141713	-4.837912	3.239259	3.224637	3.158259	3.644058	3.091571	2.982217	3.383673	3.282262	3.307029	3.167876	3.280721	3.277021	3.286436	3.510555
Ptdss2	-0.294244	6.656518	-2.144439	0.049352	0.141713	-5.13573	6.791369	6.425085	6.267611	6.352355	6.29531	6.122247	6.92927	6.709372	6.916519	6.748422	6.724644	6.915094	6.950593	7.059366
Tmem38b	-0.396413	3.63031	-2.144229	0.049372	0.141735	-4.911393	3.922683	3.24835	3.426105	3.800721	3.557438	3.616909	3.337752	3.567159	3.533498	3.530893	3.717602	3.805789	3.959716	3.927469
Srpx	0.890992	1.365073	2.144084	0.049385	0.141739	-4.400433	1.717752	2.439156	1.723223	0.43652	1.822268	1.688586	1.782265	1.020936	1.180909	1.51148	1.375754	0.930264	0.491415	1.634478
Naa60	-0.270158	6.93899	-2.143522	0.049438	0.141855	-5.139418	7.26278	6.844878	6.897448	7.270324	7.217033	7.026712	6.64904	6.857933	6.778853	6.906467	7.039611	6.84502	6.842263	6.72036
Afmid	-0.604437	3.507542	-2.14331	0.049457	0.141878	-4.843216	3.683012	3.519177	2.250588	3.425447	3.458815	2.675144	3.545949	3.904241	3.577725	3.861859	3.862983	3.980582	3.576053	3.932632
Ndufa1	-0.384735	5.92454	-2.143012	0.049485	0.141924	-5.139948	6.715589	6.048831	6.207251	6.806821	6.447542	6.802621	5.53625	5.191584	5.753159	5.597955	5.447634	5.24934	5.578065	5.588291
Arfgef2	-0.4242	5.868292	-2.142694	0.049514	0.141943	-5.118593	5.745606	5.50438	5.854487	5.940108	5.050645	5.265617	6.224758	6.23727	6.222315	5.794558	6.203189	5.962422	6.238055	5.940678
Jakmip2	-0.555423	5.568264	-2.142684	0.049515	0.141943	-5.120751	5.56163	4.974974	5.646417	6.345806	5.256357	5.714371	5.534216	5.890237	5.50284	5.229048	5.794347	5.423285	5.857134	5.258947
Oaz2	-0.288793	7.735291	-2.141948	0.049584	0.142106	-5.128154	8.104015	7.732362	7.632438	7.96148	7.745392	7.866999	7.675774	7.593883	7.728355	7.630929	7.897625	7.564873	7.725161	7.442195
Ptger2	1.037028	0.090508	2.14175	0.049602	0.142125	-4.124742	0.105685	-0.103798	1.245402	-0.705589	0.691028	0.61372	0.498056	-0.380636	0.245823	-0.806258	0.001431	0.460309	0.375586	0.554014
Dnlz	0.475521	4.440854	2.141229	0.049651	0.14223	-5.02576	4.840987	4.941423	4.796393	3.879919	4.955978	4.592225	4.464549	4.084383	4.334197	4.505931	4.427894	4.136468	4.182881	4.102607
Armcx3	-0.315729	7.098913	-2.140283	0.049739	0.142449	-5.144043	7.109928	6.913847	7.100003	7.621246	6.934096	7.249221	7.291566	6.856281	7.135141	6.78405	6.944209	6.943315	7.294999	7.218438
Jun	0.473543	7.313215	2.139642	0.049799	0.142586	-5.142727	6.908796	7.16239	8.176352	7.522346	7.717433	7.704536	6.882857	7.222872	7.20495	7.215294	7.268925	7.229151	7.164747	7.014417
Emc1	-0.347511	7.18249	-2.139273	0.049834	0.142651	-5.147941	6.879992	6.359457	6.741935	7.2112	6.704603	6.985708	7.658599	7.276357	7.69235	7.274216	7.215285	7.350261	7.430874	7.785335
Agap3	-0.285446	8.977625	-2.138876	0.049871	0.142724	-5.098963	9.22296	8.597114	9.008217	9.129342	8.934928	9.029787	8.968073	8.926703	9.031092	9.037171	8.959551	8.910033	9.015104	8.919804
Cks2	1.714569	-0.579143	2.138366	0.049919	0.142822	-4.099244	-0.634519	0.118359	-0.195392	-0.600663	2.198134	1.857753	-1.009457	-0.81357	-2.155202	-0.961779	0.001431	-0.657145	-1.047095	-1.406952
Man2a2	-0.310227	7.526561	-2.138256	0.049929	0.142822	-5.148747	7.214831	6.970786	7.106373	7.289647	6.613825	7.078046	7.991172	7.984102	7.937271	7.784515	7.733081	7.96765	7.789164	7.920439
Stk3	0.315568	4.206335	2.138055	0.049948	0.142842	-5.046756	4.621279	4.567042	4.884032	4.452353	5.026542	4.909165	3.797364	3.741948	3.641623	3.856048	4.017407	3.961084	3.871601	3.629792
Cstf2t	-0.681449	6.422105	-2.137696	0.049981	0.142904	-5.149507	6.95158	5.631549	6.793378	7.483717	6.484138	7.23008	5.980672	6.279794	6.144812	5.940353	6.475284	6.105823	6.425389	6.002137
Ppp2r2b	-0.491698	6.867842	-2.137446	0.050005	0.142937	-5.148055	7.325302	6.359457	6.931352	7.603855	7.234236	7.366052	6.78444	6.587212	6.672407	6.498158	6.816134	6.399064	7.008249	6.57769
Usp45	-0.746927	4.584466	-2.136876	0.050058	0.143026	-5.081541	4.698293	4.08227	4.882053	5.816478	4.070978	5.016334	4.587372	4.736311	4.519444	3.734632	4.765698	4.118979	4.911337	4.311247
Gbf1	-0.223123	7.306451	-2.136742	0.050071	0.143026	-5.149834	7.276408	7.101849	7.149784	7.459275	7.161856	7.164827	7.436409	7.378872	7.369661	7.303865	7.319292	7.357737	7.45827	7.362178
Tfpt	0.288701	4.361222	2.136734	0.050072	0.143026	-5.037213	4.460789	4.667202	4.79429	4.405886	4.779733	4.636834	4.253222	4.166665	4.236421	4.31582	4.32049	4.046822	3.929227	4.120817
Pcdhga12	-0.788346	3.278876	-2.136501	0.050094	0.143055	-4.841336	2.977763	1.894965	3.158259	4.155549	2.586747	3.502454	3.137884	3.591194	3.577725	3.250936	3.265855	3.419555	3.748946	3.432519
Nog	-1.010233	3.107978	-2.136211	0.050121	0.143099	-4.83641	3.127854	0.973336	2.718102	4.155549	3.259533	3.57437	3.159163	3.232189	3.280514	3.258764	2.90242	3.461789	3.152075	3.468514
Mylk	0.343763	5.156947	2.135998	0.050141	0.143122	-5.092728	5.266224	5.4978	5.176563	4.731426	5.2	5.479548	5.261619	5.188961	5.117279	5.131403	4.967529	5.116845	4.992221	5.114213
Slc25a46	-0.637452	6.104652	-2.135673	0.050171	0.143163	-5.148536	6.158125	5.18205	5.879498	6.977523	6.179853	6.479302	6.124195	5.862525	6.05641	5.842364	6.283803	5.892092	6.534907	6.036115
Tnfaip6	0.779381	1.281266	2.135579	0.05018	0.143163	-4.439969	1.459129	1.21714	2.122353	0.966949	2.460496	1.960428	0.357848	1.532126	0.571595	1.625169	0.924208	1.461155	0.836265	1.122575
Tcea3	1.434743	-0.41607	2.135464	0.050191	0.143163	-4.048263	-0.518419	1.010791	-0.491181	-1.223791	1.143601	0.117189	-0.529223	0.818632	-0.929891	-0.027758	-0.411343	-0.541385	-1.638202	-0.297732
Ddr1	0.498852	7.068926	2.134949	0.05024	0.143233	-5.15338	6.845194	7.39485	7.146491	6.299956	7.081271	6.657979	6.729485	7.472967	6.851956	7.266022	7.114128	7.851994	7.048993	7.215793
Zfp385d	-0.883208	1.954937	-2.134867	0.050247	0.143233	-4.671685	2.357438	0.533043	2.135702	3.197095	2.254562	2.665044	1.726334	2.007376	1.743567	1.322149	1.803924	1.950783	2.220182	1.889408
Zeb2	-0.532915	7.766528	-2.134823	0.050252	0.143233	-5.140885	7.741318	7.08713	7.627427	8.435713	7.486614	8.018965	7.602905	8.129335	7.771042	7.542796	7.827855	7.870402	8.047834	7.549437
Mrap2	-0.434547	4.607689	-2.133647	0.050268	0.143247	-5.041938	4.255933	4.077865	4.207831	5.087979	4.505252	4.199526	4.494157	4.863466	4.910919	4.775884	4.706374	4.859513	4.743336	4.885716
Prkacb	-0.7554	3.764633	-2.133671	0.05036	0.143462	-4.912334	3.556342	2.47979	3.74196	4.267203	2.847434	3.536092	3.654827	4.342165	3.996864	3.602051	4.26176	4.092341	4.637302	3.814538
Tspan9	0.345847	6.146408	2.133584	0.050369	0.143462	-5.141029	5.921657	6.095337	6.250841	5.636341	6.196152	5.948896	6.019005	6.133337	6.113858	6.233777	6.158615	6.39362	6.440781	6.529943
Trpv2	0.509525	4.377579	2.133395	0.050386	0.143462	-4.986448	4.045673	4.522355	4.113157	3.838533	4.854661	4.310598	4.498338	4.254576	4.46554	4.542569	4.078235	4.921355	4.326276	4.591416
Zfp507	-0.387892	3.583595	-2.133344	0.050391	0.143462	-4.894787	3.556342	3.317238	3.495051	3.761892	2.987204	3.301961	3.981847	3.966821	3.634661	3.552611	3.673224	3.536147	3.822677	3.716166
Apba1	-0.547161	6.96761	-2.132324	0.050488	0.143702	-5.1558	7.510526	6.66128	7.427913	7.58972	6.654368	7.271027	6.731262	6.839655	6.703511	6.363336	6.937189	6.785294	7.424968	6.659378
Plg	1.984962	-0.436722	2.130732	0.050639	0.144098	-4.062526	-1.052261	2.36878	1.758166	-0.81875	-0.718553	0.284862	-1.21312	-0.81357	-0.929891	-0.806258	-0.616402	-0.24119	-0.893866	-0.03329
Gmcl1	-0.317597	4.998238	-2.130384	0.050672	0.144158	-5.099162	4.997891	4.673054	4.878087	5.32286	5.080218	4.748661	5.067074	4.754164	5.127233	5.036976	5.075905	4.875224	5.246245	5.141226
Hes5	-0.865546	2.532181	-2.129841	0.050723	0.14427	-4.605172	3.136736	1.914859	1.317458	1.720809	2.062471	0.949514	3.251219	2.307359	3.178898	2.707689	3.165302	3.179156	3.467079	3.417866
Me1	-0.348745	6.837469	-2.12921	0.050783	0.144405	-5.165184	7.013768	6.479532	6.668066	7.227324	6.95925	6.980118	6.980227	6.502086	6.803117	6.634237	6.914135	6.693015	7.074177	6.809362
Rnf181	0.332751	5.756544	2.129092	0.050795	0.144405	-5.139453	5.861314	5.926776	6.031841	5.259809	5.87923	5.716818	5.636383	5.747812	5.661948	5.773134	5.919544	5.775539	5.832633	5.598073
Tgfa	-0.371395	5.083773	-2.128775	0.050825	0.144456	-5.105788	5.380933	5.037557	4.862114	5.082081	4.86149	4.680104	4.785113	5.326567	4.797058	5.208673	5.20815	5.603863	5.294885	5.09131
Sesn1	-0.51159	5.66144	-2.128646	0.050837	0.144457	-5.150691	5.851906	4.982063	5.769266	6.325978	5.428721	6.121323	5.402117	5.611479	5.627297	5.485779	5.688238	5.49113	5.855784	5.650719
Grm6	-0.451471	3.294856	-2.128472	0.050854	0.14447	-4.800952	2.957854	2.724859	2.901201	3.361953	2.936364	2.323579	3.749062	3.550909	3.709451	3.62956	3.373787	3.212521	3.924083	3.942904
Lancl1	-0.486754	6.216395	-2.127752	0.050922	0.144631	-5.160044	6.209544	5.442339	6.32409	6.549765	5.77699	6.023919	6.35495	6.459196	6.546392	5.979629	6.501696	6.016554	6.661638	6.204585
Nab2	-0.309461	5.846222	-2.127597	0.050937	0.144639	-5.149241	5.799735	5.256049	5.671838	6.011156	5.732983	5.72048	5.894478	5.939438	5.865946	6.081498	6.111581	6.145232	5.724337	5.920029
Tp53i11	-0.528741	6.784264	-2.127263	0.050969	0.144671	-5.167872	6.963485	6.225489	7.091037	6.930152	5.908101	6.445772	6.504808	6.931166	6.783581	7.20904	6.867506	7.09078	7.098357	6.945212
Kif5c	-0.37374	9.07964	-2.127225	0.050973	0.144671	-5.113188	9.272534	8.998717	9.204478	9.468465	8.612334	9.17164	9.243688	9.001849	9.173291	8.621373	8.80633	8.896624	9.48758	9.159
Dexi	-0.287209	5.675764	-2.126613	0.051031	0.144756	-5.146209	5.900953	5.464442	5.425108	5.6695	5.757597	5.343677	5.721099	5.680322	5.775484	5.849693	5.850228	5.639565	5.709462	5.704534
Arf3	-0.422049	10.86972	-2.12655	0.051037	0.144756	-5.053467	11.07999	10.03467	10.88028	10.96359	10.70416	10.79421	10.95597	10.88002	11.04008	10.8335	11.07903	10.84848	11.15277	10.93014
Tp53inp2	-0.361372	7.897958	-2.126537	0.051038	0.144756	-5.154228	8.23334	8.038366	7.493862	7.921023	7.626816	7.703919	8.051451	7.63252	7.998881	8.021323	7.722912	8.030386	7.92638	8.176912
Orc3	-0.392007	3.964862	-2.12624	0.051067	0.144802	-5.010509	4.096635	3.828023	3.960246	4.488478	3.590696	4.244308	4.0114	4.032662	3.958351	3.566911	3.916315	3.822092	4.130242	3.96323
Rpl36al	0.523146	3.091033	2.125414	0.051146	0.144992	-4.803161	3.127854	3.53854	3.387569	2.568755	3.623205	2.881136	3.231264	3.024282	2.995434	3.14899	3.380703	2.89053	2.860463	2.802815
Ikzf3	-1.079648	0.294703	-2.125187	0.051168	0.144999	-4.265223	0.873216	-1.10215	0.484899	0.966949	-0.33871	0.527154	0.028455	1.356279	-0.175642	-0.027758	1.03357	0.28321	1.001683	0.606649
Nup85	0.379358	4.593695	2.125136	0.051173	0.144999	-5.01988	4.193457	4.658378	4.421813	4.026356	4.662107	4.209984	5.015502	4.750611	4.887683	4.938684	4.576858	4.559124	4.605518	4.872343
Hfe	-1.36221	1.670908	-2.124626	0.051222	0.145104	-4.457533	3.171726	0.24948	0.484899	0.785264	1.082564	0.527154	1.726334	2.007376	2.147322	2.614395	3.574129	1.950783	1.859646	1.824228
Exoc2	-0.353223	6.650424	-2.123639	0.051316	0.145338	-5.17282	6.40876	6.319904	6.094706	6.975934	6.328548	6.617961	7.028001	6.660985	6.861677	6.823293	6.715783	6.605458	6.793022	6.887885
Samd15	-0.473715	1.700332	-2.122775	0.0514	0.145516	-4.675684	2.32672	2.117298	2.135702	2.718219	1.822268	2.18908	1.335758	1.658838	1.255413	1.388059	1.581856	1.082389	1.724133	0.970221
Abcd1	0.390197	4.592904	2.122732	0.051404	0.145516	-5.036623	4.428604	4.664267	4.287732	3.980586	4.908409	4.498643	4.403445	4.856872	4.575048	4.79119	4.781463	5.056112	4.491894	4.642728
Tenm4	-0.548042	6.013816	-2.121293	0.051542	0.145875	-5.166127	5.774348	5.71901	6.111694	6.675222	5.39617	5.486734	6.274358	5.942556	6.352122	5.508182	6.129142	5.745936	6.800762	6.302561
Ntn4	-0.647047	2.746314	-2.121128	0.051558	0.145885	-4.727624	3.516065	2.309899	1.981598	2.154676	2.335271	2.05628	3.003159	2.80888	2.894141	3.060806	2.873292	3.195935	3.220819	3.286911
Rnf187	-0.373352	8.980684	-2.120855	0.051585	0.145926	-5.131297	9.311542	8.474291	8.67612	9.024188	9.19063	8.844473	8.8815	8.970885	9.078357	9.040698	9.18124	9.001901	9.151632	8.905268
Eif2a	0.283235	5.580611	2.119153	0.051749	0.146357	-5.153208	5.346894	5.73728	5.661493	5.656328	5.928839	5.819419	5.57635	5.565712	5.549853	5.44765	5.561578	5.292883	5.420761	5.596447
Bag4	-0.754943	4.855072	-2.118961	0.051768	0.146359	-5.12004	4.881181	3.758163	5.112182	5.834133	4.469939	5.055522	4.746971	4.971187	4.878873	4.336834	5.267192	4.840964	5.286892	4.588148
Zfp763	-0.520861	3.959572	-2.11878	0.051786	0.146359	-5.006562	3.827152	3.1924	3.763618	4.627263	3.756182	4.135132	4.046082	4.117861	4.121609	3.728285	4.204441	4.014082	4.13915	3.864023
Smc4	0.407181	4.619475	2.118769	0.051787	0.146359	-5.111183	4.707265	5.080024	4.841896	4.97581	5.535058	5.549845	4.085519	4.254576	4.055381	4.224061	4.327666	4.440934	4.337929	4.323092
Angptl4	1.340998	4.181889	2.117852	0.051875	0.146576	-4.978544	4.193457	5.655395	6.234648	2.968937	3.639189	4.020978	3.969854	4.527965	3.907275	4.086912	3.586147	4.183498	3.805994	3.864023
Kcnd2	-1.108254	7.036789	-2.117568	0.051903	0.146619	-5.182098	6.744202	4.335728	6.450315	7.660369	6.33265	7.24117	7.394967	7.63252	7.496513	7.17327	7.44834	7.453291	7.702428	7.465093
RGD1564036	0.523955	2.525407	2.1172	0.051939	0.14668	-4.717391	2.765113	3.142653	3.104962	1.9796	2.630048	2.602913	2.398025	2.522625	2.539002	2.291729	2.401899	2.489067	2.220182	2.544088
Lace1	-0.443037	3.176547	-2.117098	0.051949	0.14668	-4.88517	3.357948	3.046741	2.673036	3.600707	3.060255	3.426763	3.496648	2.695192	3.367065	3.070874	3.338699	2.941167	3.254	3.31881
Slmo2	0.291126	5.45794	2.116669	0.051991	0.146763	-5.162829	5.684832	5.802805	5.692309	5.53203	6.01303	6.084853	5.268979	5.08559	5.315302	5.169384	5.359357	5.058407	5.212883	5.167742
Sms	-0.648079	3.707964	-2.116212	0.052035	0.146854	-5.042218	4.132599	3.24835	3.656563	4.87707	3.789984	4.75105	3.684484	3.501034	3.456633	3.501417	3.474203	2.980434	3.5956	3.388105
Prom2	1.635769	0.475451	2.115763	0.052079	0.146943	-4.102729	-0.634519	2.324846	0.715742	-0.941548	-0.038319	-0.006501	1.668148	0.222004	0.837186	1.104165	0.721218	0.969826	0.313987	0.706485
Hist3h2ba	-0.434166	3.230509	-2.11512	0.052141	0.147077	-4.930316	3.417469	3.037697	3.131857	3.976352	3.196117	3.732651	3.072094	3.159031	3.225976	3.223093	2.940364	2.921125	3.237505	3.12494
Clec11a	-0.291585	4.501505	-2.115023	0.052151	0.147077	-5.104848	5.110378	4.557582	4.509023	4.706082	4.718362	4.670039	4.519061	4.117861	4.393136	4.298786	4.400933	4.269235	4.40224	4.418187
Pcdha6	-1.850793	0.527664	-2.114292	0.052222	0.147222	-4.232772	0.692264	-2.680539	-0.33572	1.327578	-0.424712	1.046069	1.607516	2.16515	1.843557	0.20947	0.584127	0.618025	2.253378	0.442608
Olr1462	-2.272527	1.300978	-2.114249	0.052226	0.147222	-4.328784	0.739674	-3.260591	1.593793	2.748327	0.266146	1.014597	1.913281	2.307359	2.667343	2.668442	2.213317	2.220493	1.377871	2.245294
Pcnx	-0.39088	7.463365	-2.113955	0.052255	0.147232	-5.18786	7.2749	6.79073	7.447782	7.707108	6.88969	7.272275	7.786323	7.618944	7.759089	7.243944	7.511144	7.489043	7.932157	7.773154
Clcn3	-0.49625	7.037426	-2.11392	0.052259	0.147232	-5.190842	6.979002	6.288796	6.92606	7.477365	6.596192	7.114223	6.909001	7.195695	7.096395	6.766346	7.133707	7.21533	7.557126	7.281007
Pex11a	0.572636	3.768836	2.113835	0.052267	0.147232	-4.977548	3.742407	5.000799	3.945162	3.567317	4.090486	3.901317	3.699086	3.526187	3.456633	3.588098	3.58015	4.009344	3.278395	3.496677
Npy1r	-0.580118	4.072139	-2.11358	0.052292	0.147268	-4.971261	3.718942	2.545069	3.780713	4.239206	3.741449	3.49677	4.451671	4.224157	4.642537	4.4607	4.188764	4.273193	4.152607	4.836982
Pcdh12	-1.106591	0.156582	-2.112987	0.05235	0.147387	-4.151784	0.428583	-1.947514	-0.33572	0.041976	-0.718553	-0.072565	1.000645	-0.150641	1.360416	0.134646	0.321999	0.969826	1.377871	1.556352
Pemt	0.957595	0.708543	2.112897	0.052359	0.147387	-4.153021	0.176305	0.973336	0.52603	-0.941548	0.210178	0.175273	1.836107	1.279465	1.426399	1.023513	0.924208	0.889587	1.040235	1.473753
Per2	-0.352401	6.545463	-2.112698	0.052378	0.147407	-5.190898	6.674246	6.412021	6.108312	6.582863	6.287725	6.299194	7.139268	6.417234	6.761382	6.798513	6.380132	6.318038	6.582215	6.892529
Fundc1	-0.481537	4.283468	-2.111862	0.05246	0.147603	-5.098116	4.569741	3.833259	4.254466	5.138114	4.502342	4.907024	4.272878	4.003106	4.081214	4.081942	4.116027	4.037544	4.250206	4.003043
Fsd1	-0.315749	4.272117	-2.111699	0.052476	0.147614	-5.082011	4.499177	4.256089	4.089343	4.768627	4.463969	4.487224	4.192594	4.17729	4.389005	4.10172	3.877725	4.060629	4.216936	4.311247
Map9	-0.887092	5.722464	-2.110773	0.052567	0.147804	-5.187901	6.03882	4.914502	5.994779	7.04174	5.268397	6.425722	5.321831	5.964191	5.6308	4.842049	5.933722	5.49113	6.166885	5.111939
Ltv1	0.329815	5.415768	2.110691	0.052575	0.147804	-5.159523	5.249943	5.758078	5.773542	5.441251	5.752708	5.422235	5.658967	5.220124	5.508563	5.257979	5.176705	5.094648	5.110168	5.432996
Ptprn2	-0.274537	7.996307	-2.110634	0.052581	0.147804	-5.186227	7.824065	7.438452	7.812055	7.859422	7.377298	7.644117	8.281717	8.179743	8.274133	8.142933	8.120057	8.146876	8.424074	8.429716
Dars	0.355865	5.662796	2.109	0.052742	0.148225	-5.178193	5.535003	5.703367	5.787668	5.758485	6.155054	6.365405	5.57239	5.480341	5.462129	5.297103	5.518329	5.472315	5.595968	5.606174
Gpr26	-0.798924	2.704524	-2.108745	0.052767	0.148258	-4.68964	2.742017	1.655851	2.487668	2.499634	1.463853	1.648138	2.930765	3.526187	3.09008	2.572488	3.474203	3.323571	3.855477	2.868948
Pdpk1	-0.899432	4.985301	-2.108493	0.052792	0.148293	-5.154013	5.18292	4.014741	5.339731	6.102373	4.348811	5.253872	4.874668	5.149037	4.893527	4.07195	5.33997	4.90359	5.817453	4.555051
Srfbp1	0.288317	4.65003	2.106971	0.052942	0.148681	-5.131105	4.765689	4.972603	5.294326	4.973688	5.221393	5.141808	4.362751	4.576933	4.263751	4.417954	4.477127	4.200226	4.266558	4.229697
Fbxo34	-0.373434	6.390086	-2.106642	0.052975	0.148738	-5.197145	6.212697	5.627033	6.118433	6.532567	5.977847	6.272833	6.755915	6.759727	6.641486	6.601619	6.661443	6.460239	6.532374	6.326295
Cpt1b	0.726726	3.394678	2.106265	0.053012	0.148808	-4.808461	2.682606	4.023929	3.228468	2.634716	3.210452	3.106838	4.063116	3.081965	3.901486	3.918713	3.065821	3.625558	3.212403	3.927469
Mtmr1	-0.310486	5.950898	-2.105257	0.053112	0.149027	-5.19032	5.899649	5.786701	5.582052	6.058988	5.56186	5.751835	6.393618	6.054847	6.10381	6.228164	6.106375	5.92643	5.954966	5.9291
Nedd4l	-0.451468	6.932349	-2.105078	0.05313	0.149027	-5.206167	6.868708	6.313361	6.924138	7.558552	6.833491	6.974506	7.119048	7.104003	6.967275	6.698966	7.071485	6.764355	7.077077	6.791001

Zfp703	0.376957	5.258026	2.105041	0.053133	0.149027	-5.181226	5.298244	5.667172	5.890367	5.822387	5.891515	6.308957	4.425566	5.033913	4.54566	5.084962	5.22362	5.366984	4.498832	4.597931
Rps26	0.958865	1.02744	2.104975	0.05314	0.149027	-4.261669	0.830061	1.425638	1.033553	-0.600663	0.470544	0.982423	1.862293	0.923327	1.60797	1.597576	1.347792	1.284167	1.347449	1.122575
Mmp15	-0.477423	6.245005	-2.104212	0.053216	0.149205	-5.202424	6.82953	6.034127	5.57104	6.13027	6.385723	6.019662	6.076059	6.150963	6.127559	6.425471	6.217747	6.542896	6.447071	6.492847
Cbln2	-0.663574	3.259615	-2.103532	0.053283	0.149329	-4.918899	3.180343	3.232585	2.924461	4.04669	3.26641	2.504391	3.40164	2.665313	2.95129	3.608978	3.141068	3.502822	3.882251	3.496677
Pus1	0.344734	4.254391	2.103519	0.053285	0.149329	-5.050372	4.22295	4.432057	4.416354	3.920151	4.643738	4.149692	4.063116	4.327931	4.421726	4.294496	4.235294	4.132116	4.250206	4.134325
Cxcl14	0.299352	6.876976	2.103184	0.053318	0.149387	-5.209094	7.146666	7.260884	7.193124	6.747188	7.33785	7.191899	6.382352	6.764139	6.523963	6.80154	6.645021	7.003526	6.688435	6.604628
Trim14	0.712791	1.205721	2.10293	0.053343	0.149423	-4.455795	1.245833	1.369077	2.162035	0.860705	1.390206	1.943815	0.952703	1.318383	1.393785	1.14286	1.102128	0.514819	1.377871	0.799858
Il3ra	0.801627	4.523788	2.102672	0.053369	0.149461	-4.988383	3.848935	5.02617	4.085909	3.275	4.542557	3.780216	5.172864	4.876565	4.899348	5.374339	4.404331	4.832942	4.23782	5.051519
LOC680254	0.543962	3.032196	2.10246	0.05339	0.149485	-4.779548	3.00712	2.991607	2.804219	2.077719	3.365818	3.076678	2.854546	3.660989	2.962454	3.552611	3.678847	3.33876	2.466543	2.814049
Rfc5	0.292977	4.244034	2.102249	0.053411	0.149509	-5.065123	4.15463	4.506054	4.576652	4.199801	4.43374	4.361521	4.287446	4.299035	4.106593	4.116377	4.280371	4.087853	4.182881	3.906629
Schip1	-0.389132	7.102742	-2.101997	0.053436	0.149545	-5.210502	7.246973	6.494482	6.9275	7.409506	7.286761	7.047757	7.195064	7.094925	7.222468	6.902244	7.208984	6.923304	7.433592	7.056413
Tspyl1	-0.593646	7.27437	-2.101692	0.053467	0.149559	-5.206196	7.506677	6.275394	7.31217	7.887314	7.33785	7.489231	7.161187	7.324946	7.298767	6.995055	7.538461	7.135497	7.576519	7.01259
Ptprk	-0.314078	6.36114	-2.101634	0.053473	0.149559	-5.203676	6.193671	5.913241	6.146729	6.294865	5.6394	6.021942	6.900339	6.650538	6.750153	6.502814	6.374945	6.378082	6.579763	6.729289
Gnaq	-0.881829	5.572417	-2.101532	0.053483	0.149559	-5.198386	5.972153	4.541677	5.937813	6.704312	5.315577	6.010022	5.135774	5.834271	5.351842	4.785087	5.8651	5.492828	6.206621	4.896325
Tcf3	0.429163	5.353416	2.101448	0.053491	0.149559	-5.150663	5.156961	5.775864	5.316475	4.7588	5.224928	5.223224	5.60958	5.35726	5.442331	5.689019	5.322117	5.49113	5.031023	5.588291
Ap5b1	0.307266	4.107938	2.101303	0.053506	0.149564	-5.016585	3.917539	3.972652	4.308508	3.742078	4.223657	4.009049	4.085519	4.264575	4.15118	4.066928	4.265502	4.249279	4.225326	4.120817
Nbr1	-0.256397	7.592837	-2.101014	0.053535	0.14958	-5.20518	7.643054	7.291167	7.550401	7.794472	7.409884	7.599074	7.714921	7.663079	7.611335	7.470479	7.628012	7.574127	7.739143	7.618747
Nfia	-0.413424	4.880953	-2.100998	0.053536	0.14958	-5.176079	5.331503	4.96785	5.166843	5.817662	4.947445	5.571636	4.494157	4.918332	4.441807	4.425821	4.558676	4.689249	4.746254	4.311247
Dis3	0.3792	4.188096	2.10087	0.053549	0.149581	-5.061207	3.88628	4.459406	4.808946	4.328278	4.248182	4.440619	4.34429	4.101219	4.245589	3.838474	3.925804	3.99982	4.130242	3.96323
Mpeg1	0.743161	6.226914	2.099852	0.053651	0.149789	-5.201888	6.274363	6.052203	6.765204	5.349294	6.754372	6.624491	5.159727	7.026796	5.163961	6.60075	6.492945	7.942082	5.604033	5.390475
Kdelr3	0.609666	2.130528	2.099796	0.053656	0.149789	-4.68435	2.474162	2.439156	2.36835	1.658753	2.962008	2.809587	2.05625	1.658838	1.458292	1.964563	2.100543	2.187314	1.747621	2.310209
Col4a3bp	-0.387239	6.880148	-2.099753	0.05366	0.149789	-5.214682	6.915262	6.696904	6.890083	7.664322	6.811325	7.208511	6.934679	6.668536	6.754173	6.60075	6.808461	6.735236	6.901049	6.746249
Cndp1	0.696384	2.751443	2.099524	0.053684	0.149794	-4.749106	2.706665	2.167912	3.222225	1.9796	3.293596	3.282273	1.888013	3.377479	2.024877	3.167876	3.42821	3.703897	2.301782	2.228598
Ankrd33b	-0.640746	3.778208	-2.099487	0.053687	0.149794	-5.051592	4.137033	3.055728	4.334789	4.597249	3.540516	3.935331	3.392685	4.095628	3.824043	3.344264	4.056804	3.542721	3.944552	3.212449
Pnlip	-0.704453	2.570045	-2.098533	0.053783	0.150026	-4.730892	2.810225	2.667383	1.363571	2.534609	2.361201	1.627481	2.67414	2.875639	2.846732	2.866406	2.639567	3.555779	2.575555	2.868948
Wipf2	-0.572717	5.283661	-2.097361	0.053901	0.150263	-5.197427	5.548378	5.039823	5.638234	6.319307	5.2	5.567575	4.652595	5.326567	4.936619	4.735311	5.419364	5.263337	5.587858	4.777963
Zc3hc1	0.287577	4.526996	2.097345	0.053903	0.150263	-5.077797	4.264063	4.563896	4.454139	4.159289	4.556652	4.41674	4.571588	4.677653	4.635579	4.712982	4.80736	4.416088	4.662235	4.548339
Tfpi	0.350826	5.634315	2.097314	0.053906	0.150263	-5.196157	5.443117	5.755323	6.17448	5.758485	5.692706	6.186323	5.443899	5.526777	5.549853	5.504473	5.314913	5.572084	5.343866	5.646012
Bex2	-0.472988	7.359112	-2.094282	0.054211	0.15108	-5.209962	7.8019	7.12445	7.599398	8.385708	7.675422	8.082477	7.043103	7.012033	7.331424	6.935687	7.098481	6.860889	7.221416	6.865111
Pdss2	-0.375382	4.484402	-2.092732	0.054368	0.151474	-5.152363	4.830077	4.588877	4.685039	5.256321	4.361713	5.049711	4.506663	4.101219	4.250151	4.250879	4.352504	4.229042	4.221137	4.169739
Raph1	-0.597963	5.25364	-2.092639	0.054378	0.151474	-5.202436	5.350716	4.871884	5.553741	6.404494	4.93455	5.761348	5.103206	5.278033	5.05867	4.751052	5.054439	5.138705	5.415271	4.917314
Wdr3	0.378916	5.13142	2.092477	0.054394	0.151484	-5.138139	4.442997	5.248229	5.100316	4.854179	4.895159	4.887611	5.6496	5.275563	5.436339	5.339365	4.999333	5.183562	5.101079	5.472529
Tmem60	-0.423932	4.060082	-2.091429	0.054501	0.151746	-5.080125	4.276172	3.439014	3.945162	4.488478	4.154906	4.284446	3.933266	3.811801	4.086326	4.173561	4.107714	3.895831	4.028447	4.311247
Rps17	1.483212	3.02454	2.09129	0.054515	0.15175	-4.652231	2.311111	3.96313	3.240874	1.065902	3.930833	1.402804	3.410541	2.888628	3.480126	3.448339	3.53126	3.136336	3.070609	3.704139
Plekhf2	0.361981	4.505028	2.090575	0.054588	0.151917	-5.140067	4.865238	4.92435	4.79429	4.418424	5.056608	5.216324	4.063116	4.411301	3.716062	4.414005	4.733642	4.789383	3.822677	3.917086
Ddx41	0.380865	5.087531	2.090387	0.054607	0.151935	-5.177125	5.22309	5.607302	5.521053	4.921791	5.605903	5.06899	4.771359	5.082767	4.893527	5.208673	4.91609	4.830257	4.579577	5.041995
Dync2li1	-0.367582	4.999082	-2.090083	0.054638	0.151986	-5.186658	5.29428	4.960691	4.787963	5.567645	5.040651	5.478106	5.198785	4.879821	4.942268	4.630291	4.837836	4.619447	4.863765	4.935431
Steap2	-0.625855	3.426568	-2.089946	0.054652	0.15199	-5.024529	4.60537	3.06466	3.604709	3.561676	3.540516	3.524966	2.943086	3.180315	3.038267	3.249928	4.111876	2.999674	3.459987	3.237699
Fam189b	-0.262974	7.260363	-2.089361	0.054711	0.15212	-5.232887	7.310169	6.911992	7.033812	7.138225	7.0646	6.836202	7.443473	7.324946	7.596185	7.325052	7.123951	7.329503	7.49256	7.724864
Ylpm1	-0.445433	7.920441	-2.088063	0.054844	0.152433	-5.215841	7.753279	7.798775	7.865361	8.804171	7.67413	7.983083	7.925939	8.040473	7.999559	7.716395	7.832592	7.676308	7.962271	7.860407
Kcnb1	-0.383052	6.166497	-2.088011	0.054849	0.152433	-5.223982	6.098001	5.802805	6.248538	6.198196	5.374054	5.633805	6.323487	6.435051	6.344676	6.309077	6.237883	6.423312	6.541641	6.383033
Atp6v1a	-0.691166	7.869871	-2.087778	0.054873	0.152433	-5.218827	8.011678	6.577173	7.698019	8.484974	8.040661	7.916796	7.865324	7.978038	8.051122	7.481372	8.157789	7.680413	8.370712	7.871204
Srd5a3	0.347589	4.598705	2.087665	0.054884	0.152461	-5.116453	4.380808	4.486247	4.792184	4.374055	5.002029	4.605489	4.177031	4.736311	4.429792	4.677181	4.714609	4.926391	4.502288	4.642728
Etv1	-0.512322	6.575591	-2.087023	0.05495	0.152608	-5.234137	6.930665	7.044875	6.570685	8.071752	7.178814	7.151324	6.263345	5.749593	6.36795	6.067738	6.115732	5.9327	6.39131	6.239378
Scd1	-0.631038	4.878998	-2.086304	0.055024	0.152743	-5.158881	5.478482	3.806886	4.377553	4.728912	4.685386	4.974008	5.167624	4.519639	4.858103	4.815347	4.730939	5.023584	5.752183	5.443886
Begain	-0.315841	8.491879	-2.086303	0.055024	0.152743	-5.209791	8.590296	8.231436	8.413844	8.531888	8.207845	8.130752	8.737954	8.524957	8.796658	8.756758	8.423627	8.413054	8.355983	8.775686
Snx2	0.380946	6.14224	2.085436	0.055113	0.152948	-5.237198	6.135041	6.392644	6.821528	6.687576	6.868377	7.088475	5.670127	5.751372	5.715715	5.692284	5.841482	5.880462	5.866551	5.602939
Vapb	-0.310319	5.693626	-2.085332	0.055124	0.152948	-5.224624	5.88259	5.393549	5.686652	6.092626	5.692706	5.935304	5.57635	5.680322	5.607878	5.555561	5.618273	5.672907	5.847656	5.498875
Slc6a17	-0.379822	9.248913	-2.084795	0.055179	0.153066	-5.187374	9.0261	8.647215	9.168603	9.633753	8.831922	9.149964	9.366121	9.182453	9.501639	9.154551	9.284188	9.29111	9.604449	9.645355
Sc5d	-0.439039	6.558757	-2.084068	0.055254	0.153209	-5.242309	7.004094	6.412021	6.919322	7.321289	6.508272	7.054059	6.253486	5.94877	6.490615	5.864239	6.147507	6.213771	6.895146	6.807255
Mrpl38	0.287132	5.759962	2.084047	0.055256	0.153209	-5.224754	5.874648	6.053325	6.041624	5.641698	6.250923	5.829628	5.719309	5.621239	5.727214	5.613537	5.726745	5.467141	5.529772	5.571839
Ddx25	-0.283642	5.18875	-2.083345	0.055328	0.153374	-5.185856	5.266224	4.833205	4.985204	5.193877	5.018417	4.945084	5.697657	5.088407	5.385408	5.341446	5.154681	5.049203	5.312711	5.414663
Rnf113a1	-0.383148	3.332046	-2.082994	0.055364	0.153416	-5.023986	3.84352	3.134193	3.500221	3.959292	3.61245	3.817169	3.003159	3.047633	3.464507	3.070874	3.273307	2.941167	3.03287	3.106782
Itpr3	0.725839	3.800216	2.082904	0.055373	0.153416	-4.986391	3.689063	4.889586	4.116527	3.059778	3.716556	3.648009	4.243293	3.449372	4.131534	4.051757	3.310002	3.542721	3.459987	4.012827
Bbc3	0.531254	2.925473	2.082737	0.055391	0.153416	-4.794373	2.501925	3.317238	3.240874	2.401998	2.484288	2.863579	2.703406	3.449372	2.834633	3.747244	3.324422	3.433771	2.437952	2.431883
Zc3h6	-0.468205	4.592975	-2.082701	0.055394	0.153416	-5.143202	4.417714	4.49288	4.564365	5.181057	3.836938	4.467019	4.8055	4.836907	4.707008	4.491011	4.784073	4.472262	4.716806	4.594677
Pon1	-3.965988	-3.448256	-2.082545	0.05541	0.153426	-3.931374	2.342161	-3.260591	-2.432145	-2.731185	-3.260591	-3.260591	-3.260591	-3.260591	-3.260591	-0.420466	1.605665	-2.249472	-3.260591	-3.260591
Gins2	-0.348335	6.206993	-2.082088	0.055458	0.153521	-5.229814	5.835634	5.633051	5.674127	6.1492	5.899279	5.376544	6.420303	6.583223	6.497346	6.754682	6.357522	6.637287	6.364025	6.738163
Mib1	-0.664616	5.571903	-2.081669	0.055501	0.153605	-5.221242	5.661942	5.060063	5.771406	6.165098	4.505252	5.653096	5.60183	5.744244	5.717364	5.398527	5.546281	5.696681	6.295957	5.223575
Gpr6	-0.752768	0.725593	-2.081155	0.055554	0.153715	-4.402787	1.594228	0.369671	0.679743	0.621404	0.210178	0.175273	0.429655	1.020936	1.061401	1.253084	0.535373	0.84773	1.28461	1.048408
Smo	-0.453926	4.105986	-2.081037	0.055566	0.153715	-5.037639	4.280186	3.613496	3.295418	3.701615	3.649748	3.579757	4.785113	4.351577	4.347031	4.4607	4.265502	4.588013	4.070962	4.591416
Arf6	0.455597	5.431869	2.080771	0.055594	0.153756	-5.226455	5.501013	5.469496	6.263817	5.760949	6.197052	6.413708	4.980068	5.102412	5.159118	4.902496	5.213971	5.321836	5.040563	4.757741
Incenp	0.426327	4.360932	2.080616	0.05561	0.153765	-5.149842	4.414065	5.273489	4.443444	4.690659	4.95385	5.275609	3.882992	4.193082	3.96946	4.010503	4.030659	4.233112	3.969738	3.791988
Hs2st1	0.362092	6.606258	2.080229	0.05565	0.153841	-5.247215	6.300288	6.537623	6.949398	6.769381	6.98084	7.121627	6.247289	6.530333	6.329667	6.607683	6.398138	6.690797	6.555847	6.484961
Mapre2	-0.4954	8.313882	-2.079961	0.055678	0.153882	-5.203236	8.917712	8.188412	8.861402	9.381086	8.577677	8.990263	7.639326	8.069637	7.898928	7.708758	8.235671	7.919845	8.267101	7.743713
Dlc1	-0.465903	5.094693	-2.079693	0.055706	0.153924	-5.218277	5.388389	4.827969	5.596606	6.004935	5.026542	5.469427	5.024227	4.873301	4.950701	4.425821	4.85532	4.613215	5.338076	4.978507
LOC502894	-0.495244	4.927331	-2.078769	0.055801	0.154153	-5.166411	5.002757	3.848855	4.617663	4.967302	4.728358	4.736655	5.206471	5.194202	5.05867	5.029251	5.427736	5.028276	5.44431	4.746057
Vamp8	0.53794	3.33495	2.078374	0.055842	0.154231	-5.009614	4.078311	3.99151	3.754994	2.934433	4.230707	4.094316	2.398025	3.282262	2.797714	3.070874	3.480655	3.390696	2.379013	2.972849
Traf7	0.519453	6.710497	2.077504	0.055933	0.154445	-5.247811	6.39031	7.192752	6.895978	5.969161	6.505368	6.198629	7.049528	6.801533	6.900647	7.128781	6.688869	6.803883	6.424478	7.01259
Gramd1a	0.419065	7.672288	2.076599	0.056027	0.15466	-5.252954	7.256682	7.893619	7.382444	6.869804	7.556665	7.092249	8.089011	7.845674	7.99922	8.081698	7.712328	7.788765	7.695274	8.156646
Zfp532	-0.257256	6.577044	-2.076511	0.056036	0.15466	-5.25209	6.517315	6.438031	6.328457	6.573784	6.325259	6.063866	6.796373	6.795511	6.784368	6.818819	6.696511	6.654839	6.661638	6.640569
Txnrd3	-0.327509	4.370139	-2.076373	0.05605	0.154664	-5.148412	4.664912	4.302134	4.544485	4.728912	4.059146	4.568038	4.3855	4.117861	4.504248	4.377964	4.103539	4.296718	4.212723	4.392186
Cd72	1.493884	-0.214208	2.075454	0.056147	0.154893	-4.093726	-1.417928	0.310827	-0.263851	-0.941548	0.027932	0.571087	-0.399109	2.054242	-2.155202	1.21727	0.998029	2.433555	-1.218554	-1.632384
Hmbs	0.447656	4.155508	2.075161	0.056177	0.154942	-5.069232	4.110227	4.382912	3.89502	3.584109	4.725866	4.149692	4.263083	4.198308	4.002283	4.303064	4.387261	4.170823	4.028447	4.065483
Hspb6	0.246914	5.808688	2.074585	0.056237	0.155038	-5.256042	6.491582	6.807455	6.565762	6.426386	6.595509	6.854094	5.069886	5.245588	5.146937	5.305655	5.419364	5.204441	5.105631	5.114213
Sephs2	-0.363011	5.57333	-2.074581	0.056238	0.155038	-5.236819	5.710342	5.194311	5.497982	5.99241	5.644688	5.616876	5.534216	5.603623	5.339051	5.364141	5.675639	5.681868	5.863867	5.340848
Rgcc	-0.434101	4.153014	-2.074252	0.056272	0.155097	-5.070105	4.3275	3.61957	3.228468	3.897939	4.035185	3.835297	4.696922	4.459975	4.473365	4.453021	4.188764	4.224961	4.360956	4.432837
Ralgapa1	-0.364551	7.483189	-2.073699	0.05633	0.155222	-5.258049	7.015575	7.189183	6.930854	7.752689	6.881875	7.07137	8.175748	7.555203	7.990726	7.492183	7.579617	7.399078	7.814755	7.92498
Tnnc2	-0.610118	2.569573	-2.072999	0.056404	0.155388	-4.929401	3.569521	3.452688	2.445413	3.204354	3.00376	2.28453	2.01015	2.099634	2.04602	3.223093	2.332254	2.461578	1.943396	2.17732
Snca	-0.848905	8.314523	-2.072696	0.056435	0.15544	-5.223512	8.728352	6.893312	8.253381	9.30716	8.652427	8.882699	8.221036	8.237043	8.53096	7.873489	8.440895	7.830329	8.482331	8.075236
Rgs17	-0.804755	3.620214	-2.072346	0.056472	0.155506	-5.099536	3.865059	2.801663	4.126591	5.036039	3.568609	4.079183	3.241276	3.475434	3.400282	2.707689	3.760656	3.269114	4.250206	3.237699
Coa5	-0.405821	7.173513	-2.072035	0.056505	0.15556	-5.258597	7.502818	6.863552	6.894016	7.595598	7.329665	7.486366	7.069333	6.952972	7.013625	6.954163	7.366219	7.001141	7.440815	6.969907
Nck2	-0.319283	5.871476	-2.071646	0.056546	0.155637	-5.250662	5.96967	5.670101	5.591771	6.189078	5.721768	6.06098	6.130942	5.839298	6.066791	5.771592	5.932545	5.641098	5.811893	5.830202
Abi2	-0.639362	7.863049	-2.071238	0.056589	0.155719	-5.246171	7.955998	6.914464	7.898775	8.576974	7.483297	8.213643	7.77215	8.088054	7.82975	7.517711	8.056421	7.835358	8.328745	7.618346
Lpcat4	-0.293721	7.15721	-2.071114	0.056602	0.155719	-5.264588	7.068753	6.898941	6.896958	7.176097	6.855322	6.664331	7.456961	7.260831	7.451665	7.423122	7.084781	7.2584	7.134998	7.581018
Phactr1	-0.459349	7.123236	-2.070968	0.056617	0.155726	-5.256658	7.920758	7.122847	7.601204	7.611724	6.974029	7.536741	6.901917	6.889775	6.788294	6.426452	6.812942	7.018341	7.364536	6.767354
RGD1565819	-0.777401	4.225917	-2.070577	0.056658	0.155803	-5.079858	3.788223	2.943995	3.772191	4.773515	3.203302	4.109292	4.489964	5.074265	4.628589	3.826639	4.706374	4.526331	5.059455	4.35421
Tmx2	-0.374818	7.023275	-2.069	0.056825	0.156225	-5.268229	6.906201	6.343949	6.721608	7.22018	6.907947	6.779401	6.827443	7.23881	7.252905	7.069129	7.139325	7.154399	7.349263	7.42764
Ttbk2	-0.705633	4.297238	-2.068824	0.056843	0.156241	-5.163361	4.485336	4.180039	4.742881	5.258066	3.452798	4.304105	4.085519	4.429191	4.101553	3.765957	4.49954	4.092341	4.967428	3.880148
Apol3	0.679129	2.344137	2.067975	0.056933	0.156452	-4.751259	2.582145	3.216645	2.877559	1.63746	2.597694	2.349037	2.467906	2.227821	2.381582	2.720538	1.996325	2.064785	1.724133	2.294252
Zhx1	-0.486039	6.778696	-2.067474	0.056986	0.156561	-5.269492	7.0047	6.317103	6.911097	7.631379	6.770184	7.326548	6.516191	6.613855	6.523963	6.518533	6.783236	6.609377	6.923781	6.46639
Fhit	-0.513402	2.770023	-2.06724	0.057011	0.156594	-4.825763	3.073383	2.151237	2.200654	2.546081	2.484288	2.323579	2.566765	3.201289	3.31576	2.923085	3.250834	2.921125	3.270309	2.791492
Tmx4	-0.603554	7.785589	-2.067101	0.057025	0.156598	-5.261788	7.453463	6.4837	7.464078	8.30191	7.416469	7.730496	7.836307	7.777481	8.052102	7.881734	7.930107	8.005023	8.316036	8.356927
Hp1bp3	-0.248883	7.991651	-2.066144	0.057127	0.156841	-5.255815	8.069063	7.664581	7.956332	8.150665	7.956786	7.8681	7.952464	8.066069	8.047195	7.917046	8.145659	8.038839	8.047242	8.00926
Rps29	0.657459	6.173005	2.065972	0.057145	0.156842	-5.257094	6.349825	6.99084	6.002996	5.145675	6.703337	5.900214	6.166842	5.928474	6.223476	6.355121	6.255863	5.975843	6.064229	6.382089
Rpia	0.416298	3.601473	2.065835	0.05716	0.156842	-4.983173	3.230989	3.696289	3.746318	3.309066	4.047215	3.255598	3.790562	3.798099	3.592171	3.683052	3.656224	3.496064	3.576053	3.673625
Calu	0.339482	7.104032	2.065774	0.057166	0.156842	-5.270668	7.184635	7.177908	7.469689	7.168694	7.58066	7.838355	6.664934	6.8637	6.864655	6.809078	6.809743	7.073292	6.939163	7.02352
Ldb1	0.340486	6.89611	2.065339	0.057212	0.156933	-5.273091	6.760097	7.20189	7.161659	6.454815	6.705236	6.720881	6.848064	7.139074	6.902821	7.154456	6.963916	7.001738	6.635909	6.90834
Zfp598	0.407142	6.751335	2.0646	0.057291	0.157112	-5.272992	6.358393	7.176364	6.924619	6.541192	6.737766	6.589541	6.939299	6.802391	6.939298	6.945981	6.439298	6.67293	6.434463	7.031964
Fbxo6	0.241689	5.512451	2.0642	0.057334	0.157193	-5.24939	5.624546	5.902072	5.847405	5.492552	5.649957	5.797626	5.309988	5.412264	5.38748	5.524759	5.419364	5.407164	5.183045	5.250702
Dgkg	-1.000606	7.069968	-2.062838	0.057479	0.157516	-5.275472	6.95535	4.899604	6.959755	7.844691	7.07394	6.650959	7.415008	7.514933	7.4064	6.613722	7.621458	6.969777	7.855185	7.212613
Fkbp11	0.880054	2.225634	2.06274	0.057489	0.157516	-4.721005	2.372556	3.702025	2.61703	1.405223	2.240661	2.434761	2.598267	1.429209	2.087398	2.499674	2.166061	1.667719	1.983515	2.310209
Scg2	-1.147551	8.65407	-2.062728	0.057491	0.157516	-5.246661	8.364597	5.893325	8.146784	9.423008	8.28987	8.659724	8.778482	9.111169	9.282267	9.002906	8.793592	8.923003	9.044885	9.448547
Hic2	-0.513432	2.727888	-2.062075	0.05756	0.157671	-4.885778	2.821286	2.570375	2.812554	3.275	1.930143	2.872384	2.644268	2.951874	2.462438	2.998871	2.940364	2.772447	2.933966	2.446395
Lmf2	0.537819	6.118718	2.061551	0.057616	0.157788	-5.261668	6.055309	6.693309	5.833137	5.312821	6.430804	5.91627	6.1747	6.180309	6.083504	6.450752	5.948925	6.385415	5.820225	6.399907
Enthd2	0.623352	5.634886	2.061327	0.05764	0.157803	-5.213953	5.033995	6.244284	5.519781	4.669835	5.335334	4.80033	6.196734	5.726267	5.977447	6.061439	5.74428	5.783886	5.604033	6.22472
Stx7	-0.415861	4.802913	-2.06115	0.057659	0.157803	-5.184409	4.573017	3.982112	4.446125	5.080109	4.6725	4.548942	4.815587	5.143629	4.878873	4.916522	5.130266	5.121244	5.237977	4.751911
Pp3111	0.376393	5.349872	2.061131	0.057661	0.157803	-5.22058	5.002757	5.818732	5.322324	4.971562	5.18556	5.136335	5.632584	5.396437	5.499012	5.597955	5.343515	5.239258	5.276837	5.514455
Dpm1	-0.490299	5.62507	-2.060898	0.057686	0.157836	-5.272745	5.97587	5.348546	5.605028	6.643531	5.876985	6.449454	5.478169	5.292766	5.466056	5.376371	5.325705	5.251348	5.40053	5.293472
Eif4a2	-0.480858	9.029639	-2.060188	0.057762	0.157982	-5.220718	9.262552	8.594803	8.993055	9.928729	9.283498	9.583153	8.997168	8.690092	8.970352	8.67124	8.911408	8.608612	9.077399	8.845956
Pcdhb6	-1.000254	1.816708	-2.060154	0.057766	0.157982	-4.696526	2.01613	0.679782	1.116684	2.697792	1.510943	2.244393	2.854546	1.856886	0.628769	2.184529	1.697158	2.170435	1.837926	2.431883
Dpp7	0.532994	6.99461	2.059951	0.057788	0.158006	-5.27752	6.503648	7.171721	6.59686	5.842299	6.785226	6.263659	7.280078	7.484777	7.203187	7.692954	7.219149	7.825283	6.718462	7.350651
C1galt1c1	-0.506914	4.923938	-2.059333	0.057854	0.158151	-5.219834	5.25607	4.313419	4.204668	5.195699	5.016379	5.343677	4.851979	4.860173	5.132184	4.824303	4.736341	4.898474	5.154774	5.200217
Blnk	0.857088	4.504922	2.058622	0.05793	0.158323	-5.120473	4.361994	4.625556	3.702138	3.480296	5.661742	5.070904	3.647317	5.480341	3.930199	5.159982	5.146589	5.559173	3.536146	3.786295
Got2	-0.401634	7.704805	-2.058444	0.057949	0.15834	-5.277509	7.778327	6.925538	7.563116	7.75794	7.711146	7.271027	7.70862	7.81666	7.897838	7.741038	8.132147	7.795662	8.075069	7.700855
Tmem66	-0.384659	7.793446	-2.058176	0.057978	0.158382	-5.278905	7.7935	6.913847	7.348452	7.639769	7.638128	7.432309	7.815516	7.910391	8.084075	8.038741	7.907237	8.2032	8.043392	8.347051
LOC100158225	0.378756	2.826046	2.057141	0.05809	0.158651	-4.842741	2.582145	2.953644	2.812554	2.451642	3.036313	2.762997	2.828218	3.093229	2.917273	2.866406	2.986432	2.951084	2.562371	2.982839
Fam73b	-0.274179	7.011276	-2.056453	0.058164	0.158817	-5.287038	6.622229	6.588055	6.295384	6.62201	6.392811	6.118551	7.536867	7.360343	7.49026	7.477119	7.332729	7.354471	7.360257	7.619948
Anxa11	-0.493082	7.953373	-2.056309	0.058179	0.158824	-5.272112	8.120619	6.961252	7.859591	8.278244	8.018936	7.991948	7.93907	8.096475	8.030054	7.963386	8.274484	7.893408	8.124966	7.8013
Napa	-0.404614	8.197514	-2.056149	0.058197	0.158829	-5.273431	8.336444	7.491992	7.644745	7.922949	8.156248	7.613589	8.268927	8.602144	8.467487	8.512691	8.816089	8.239817	8.470249	8.227417
Trip11	-0.544605	5.268877	-2.055938	0.058219	0.158829	-5.256771	5.315947	4.88455	5.419662	6.07887	4.750601	5.562143	5.159727	5.423464	5.05867	4.927646	5.176705	5.164508	5.757977	5.12553
Klhdc2	-0.319255	5.869587	-2.055821	0.058232	0.158829	-5.279276	5.974632	5.530404	6.004816	6.318471	5.835967	5.937404	5.921193	5.746029	5.982939	5.639139	5.871252	5.511379	6.070063	5.857606
Snrpd2	0.517866	4.311845	2.055748	0.05824	0.158829	-5.125305	4.536569	4.724685	4.363439	3.443962	4.745687	4.127796	4.39898	4.303891	4.405458	4.373903	4.451079	4.118979	4.161181	4.291288
Tpd52l2	0.296404	5.248608	2.055681	0.058247	0.158829	-5.244178	5.290305	5.492845	5.554984	5.132418	5.672137	5.303548	5.100459	5.232912	5.204486	4.856672	5.28389	5.018877	5.210772	5.167742
Gstz1	-0.338636	5.578398	-2.054992	0.058322	0.158974	-5.267693	5.967183	5.742856	5.430534	5.61062	5.460554	5.170651	5.653354	5.416754	5.733744	5.766954	5.560056	5.440987	5.478052	5.698484
LOC499602	-0.55472	3.251793	-2.054944	0.058327	0.158974	-4.971242	3.32724	3.390109	2.837276	3.515737	2.732935	2.592292	4.223227	3.232189	3.510866	3.530893	3.013384	3.083214	3.203938	3.503633

Enpep	0.727034	2.028626	2.054427	0.058383	0.159075	-4.728287	2.091157	1.833582	2.548818	1.700419	3.00376	2.957603	2.343298	1.239467	2.00342	1.942797	1.557647	1.760874	1.493524	2.325991
Chmp7	-0.207996	7.104326	-2.054355	0.058391	0.159075	-5.29179	7.261258	7.027278	7.03915	7.266861	7.049728	7.108644	7.14261	7.054435	7.073411	7.125766	7.131659	7.008283	7.078235	7.104673
Sh3bgr	-0.449822	2.325708	-2.052598	0.05858	0.159558	-4.836161	2.832262	2.36878	1.874203	2.591079	2.553396	2.297664	2.518182	2.326577	2.568526	2.39151	2.181985	2.00891	2.203293	2.159814
Rnf11l1	-0.436221	3.719302	-2.052314	0.058612	0.159569	-5.077243	3.938008	3.134193	3.570714	3.924553	3.601614	3.653128	3.749062	3.475434	3.811751	3.814705	4.107714	3.662244	4.216936	3.531125
Kxd1	0.334954	5.280433	2.052175	0.058627	0.159569	-5.251453	5.331503	5.779937	5.467954	5.111335	5.799686	5.175995	5.052933	5.258153	5.099692	5.224545	5.252185	5.302599	4.962418	5.147901
Zfp37	-0.5708	1.693709	-2.052041	0.058642	0.159569	-4.770829	2.196854	1.248835	2.31067	2.718219	1.705671	2.28453	1.443753	1.532126	1.769222	1.481601	1.456531	1.375373	1.493524	1.193114
Nupl1	-0.434979	4.599034	-2.051919	0.058655	0.159569	-5.216333	4.680181	4.368558	4.78373	5.329514	4.519714	4.621246	4.595201	4.402272	4.523219	4.111508	4.711869	4.458423	4.984828	4.361886
Cdk2ap1	0.335337	6.744537	2.051919	0.058655	0.159569	-5.294472	7.211156	7.46947	7.248317	7.090544	7.893504	7.331346	6.194159	6.09182	6.236179	6.417603	6.399841	6.30939	6.187427	6.358304
Swap70	0.332514	5.405169	2.051829	0.058665	0.159569	-5.252288	5.419656	5.48455	5.62528	5.034003	5.457552	5.651818	5.34058	5.471736	5.230115	5.642519	5.334638	5.762926	4.937104	5.317354
Pik3r4	-0.270341	5.847379	-2.051062	0.058748	0.159753	-5.270429	5.545046	5.29072	5.4941	5.824744	5.419496	5.457773	6.164213	6.149614	6.207144	5.821644	6.131194	6.061879	6.235981	6.087944
Endog	-0.475876	2.698611	-2.050962	0.058759	0.159753	-4.850199	3.180343	2.279532	2.379614	2.389316	2.373993	2.146157	2.688847	2.781287	2.680923	3.00938	2.995472	2.951084	3.160849	3.012401
Wasf1	-0.86232	9.408838	-2.050231	0.058839	0.159898	-5.227298	9.654618	7.775163	9.296309	10.33449	9.609017	9.85899	9.288885	9.650776	9.487581	9.16531	9.572812	9.276641	9.535069	9.220585
Wdr48	-0.302403	6.40836	-2.050223	0.05884	0.159898	-5.29877	6.664299	6.188172	6.647828	6.824594	6.2673	6.664331	6.317584	6.283487	6.231573	6.267	6.433489	6.229135	6.416257	6.300566
Arhgap29	0.403406	3.858229	2.050031	0.058861	0.159898	-5.131028	4.315816	4.515857	4.391531	3.659984	4.031153	4.576145	3.957761	3.466799	3.748669	3.377094	3.243265	3.536147	3.614885	3.692011
Foxj3	-0.749331	6.017809	-2.049983	0.058866	0.159898	-5.297787	6.35078	5.271561	6.648992	7.336646	5.899279	6.552353	5.509574	6.062031	5.756369	5.292808	6.084302	5.728743	6.22974	5.551845
Epb41l4b	-0.501721	3.819557	-2.049798	0.058886	0.159907	-5.157576	4.15463	3.939046	4.549481	4.771073	3.428477	3.939526	3.756062	3.591194	3.464507	3.622732	3.42821	3.594258	3.866246	3.482664
Ager	0.824813	4.188772	2.04971	0.058896	0.159907	-5.062684	3.488576	5.088807	4.31439	3.443962	4.198707	3.585123	4.667523	4.388622	4.429792	4.693564	3.944596	4.149447	3.569478	4.772214
Clip1	-0.396823	6.921975	-2.049523	0.058916	0.15991	-5.301051	6.82129	6.38107	6.820495	7.463441	6.737766	7.040938	7.022206	7.126108	6.978316	6.782519	6.962764	6.821561	7.121016	6.841286
Fam126a	-0.747392	2.96801	-2.049456	0.058923	0.15991	-4.959659	2.947796	1.769471	3.013923	3.857074	2.496038	3.353184	2.760215	3.252427	2.870631	2.819403	2.843564	3.145002	3.6527	2.982839
Pwp1	0.332518	4.388203	2.04916	0.058956	0.159937	-5.171853	4.15463	4.332034	4.620039	4.440107	4.899589	4.670039	4.330288	4.388622	4.334197	4.311581	4.176893	4.158035	4.225326	4.468825
Imp4	0.307616	5.129426	2.049121	0.05896	0.159937	-5.22677	5.069216	5.086616	5.258166	4.600004	5.280338	4.917698	5.094948	5.162468	5.173599	5.284179	5.331071	5.218879	5.145962	5.234069
Sidt1	-0.303074	7.030817	-2.048524	0.059025	0.160078	-5.302559	6.843838	6.881987	6.383346	6.872654	6.493694	6.464819	7.394406	7.308729	7.425181	7.391837	7.086898	7.283146	7.176693	7.436754
Gabarapl2	-0.364138	4.902938	-2.048333	0.059046	0.160099	-5.224428	5.134966	4.21255	4.762375	4.874798	4.85009	4.71234	5.193638	4.655026	5.114779	4.930413	5.13845	4.911231	5.178731	5.025175
Itgae	0.53024	2.702591	2.047842	0.0591	0.160181	-4.862434	2.682606	3.324693	2.829082	2.310787	3.068148	2.863579	2.880403	2.505864	2.597457	2.843096	2.823399	2.726993	1.881043	2.745289
Rnf13	-0.55817	5.144279	-2.04774	0.059111	0.160181	-5.274212	5.622966	4.57644	5.245908	5.917099	5.174635	5.840887	4.678618	4.921495	4.69032	4.735311	4.923213	5.348344	5.310741	5.07972
Sgsm1	-0.30927	7.601161	-2.047612	0.059125	0.160181	-5.297003	7.584208	7.682099	7.369791	7.779666	7.354888	7.080423	7.872589	7.509162	7.970134	7.447478	7.511932	7.41484	7.773516	8.073777
Med11	0.539243	3.069272	2.047474	0.05914	0.160181	-4.963801	3.543041	3.607396	3.545931	2.43939	3.706477	3.143674	2.774074	3.211662	2.95129	2.988286	3.124682	2.838051	2.452318	2.83626
Ado	-0.526969	3.268959	-2.047408	0.059147	0.160181	-5.032491	3.516065	2.545069	3.171281	3.82447	3.500244	3.353184	3.105363	3.282262	3.23521	2.819403	3.448101	3.331185	3.748946	3.050898
Smad2	0.340541	4.467946	2.047325	0.059156	0.160181	-5.206599	4.579546	4.588877	5.048326	4.577819	4.912799	5.108656	4.362751	4.161323	4.290573	3.962637	4.363019	4.241218	4.398536	4.027379
Itch	-0.549234	5.343147	-2.045827	0.059321	0.16059	-5.285144	5.589393	5.035287	5.706915	6.265671	5.165466	5.603716	4.915882	5.423464	5.24619	4.609719	5.22362	5.24128	5.721374	5.09592
Zfp105	0.489288	3.797381	2.045621	0.059343	0.160615	-5.083897	3.627383	4.522355	4.39431	3.611667	3.523394	3.99299	3.797364	3.93587	3.641623	3.765957	3.603988	3.864689	3.4385	3.558103
Bcor	-0.280153	4.842318	-2.045225	0.059387	0.160697	-5.235252	4.878536	4.661326	4.864121	5.192053	4.735811	4.767664	4.767901	4.873301	4.80328	4.738473	4.698092	5.032953	4.906128	4.927694
Slc35e2b	-0.502483	3.938204	-2.044451	0.059472	0.160891	-5.091075	3.614724	3.42521	3.604709	4.294667	3.011968	3.812601	4.134688	4.123366	4.250151	3.957219	4.284065	4.04219	4.659142	4.027379
Zfp868	0.288719	4.922426	2.043643	0.059561	0.161095	-5.241864	4.894334	5.494499	5.224203	4.919588	5.070428	5.000356	4.858498	4.806432	4.673435	4.874027	4.80736	4.822175	4.683703	4.836982
Ikbkb	0.417353	5.724482	2.042957	0.059637	0.161264	-5.271524	5.350716	6.174829	5.566119	5.197519	5.487291	5.540207	6.033481	5.956501	5.909857	6.057647	5.692413	5.858236	5.453267	5.895119
Tpcn2	0.643234	4.581657	2.041684	0.059778	0.161608	-5.089376	3.695089	4.704825	4.051108	3.462244	4.302723	3.830786	5.365968	4.930944	4.919536	5.159982	4.667312	5.210647	4.456698	5.458279
Dhps	0.276611	5.232479	2.04118	0.059833	0.161722	-5.267758	5.19785	5.35766	5.612208	5.20839	5.26325	5.678421	5.249269	4.961999	5.124751	5.185692	5.071637	5.076641	5.123695	5.185153
Olfml1	-0.448779	5.690505	-2.040928	0.059861	0.161761	-5.303766	6.497627	5.773142	5.319403	5.85619	5.983075	5.838643	5.393164	5.312177	5.2845	5.550173	5.599127	5.923914	5.728769	5.638132
Trim36	-0.520398	3.356038	-2.040021	0.059962	0.161962	-5.017248	3.09177	3.019437	3.12518	3.937678	3.129785	2.791131	3.479833	3.763261	3.383769	3.129854	3.082885	3.594258	3.760537	3.853172
Chkb	0.537917	5.931492	2.040016	0.059962	0.161962	-5.277389	5.511294	6.384641	5.807301	5.021729	5.721768	5.293749	6.460498	5.991539	6.276989	6.402732	5.968568	6.049238	5.806312	6.371672
Pcm1	-0.335083	7.459829	-2.039727	0.059994	0.161962	-5.311706	7.252091	7.325479	7.213725	7.981158	7.199623	7.380784	7.740735	7.515979	7.674217	7.389325	7.295244	7.325219	7.538731	7.614335
RGD1311899	-0.287781	7.960911	-2.039666	0.060001	0.161962	-5.299134	8.250575	7.754956	7.830129	8.110179	8.184662	7.803098	7.935221	7.883474	8.070597	7.935761	8.075462	7.75873	7.980662	7.885586
Enkur	-0.60984	3.724081	-2.039652	0.060003	0.161962	-5.11539	4.674093	3.432128	2.977318	3.538889	3.794748	3.747086	3.993741	4.003106	3.503242	3.530893	4.188764	3.70975	3.608485	3.558103
Sstr1	-0.413711	4.176879	-2.039385	0.060032	0.162005	-5.188526	4.255933	4.290759	4.175882	4.807278	4.109733	3.939526	4.134688	3.501034	4.500423	3.994723	3.88747	3.83286	4.314529	4.820364
Ndufa12	-0.524612	4.672016	-2.039085	0.060066	0.162059	-5.299468	5.655776	4.941423	5.235097	6.318471	5.516429	6.159595	3.920861	3.871902	3.96946	3.924277	4.136602	3.70975	3.994491	4.125333
Ndufs5	-0.588802	2.853267	-2.038371	0.060145	0.162205	-5.057584	3.453461	2.801663	2.820842	4.030446	2.95351	4.090548	2.644268	2.488905	2.493553	2.239124	2.627988	2.475388	2.393975	2.660694
Catsperg1	0.590917	3.992279	2.03812	0.060173	0.162205	-5.111901	4.087502	5.019295	4.175882	3.295536	3.86892	4.028875	4.253222	3.668539	4.076084	3.832569	3.639021	3.60686	3.994491	4.44734
Hcfc1r1	-0.308955	7.589796	-2.038062	0.06018	0.162205	-5.296064	8.440983	7.807448	7.986345	8.364806	8.286913	8.298373	7.095095	7.144499	7.258577	7.144956	7.307545	7.001141	7.085742	7.043345
Ipo9	-0.218817	7.241399	-2.037933	0.060194	0.162205	-5.31787	7.385691	7.215989	7.21883	7.406362	7.090989	7.183521	7.400562	7.148554	7.359141	6.994394	7.142381	7.120195	7.444413	7.278981
Arfgef1	-0.454247	7.023825	-2.037885	0.060199	0.162205	-5.319739	6.787849	6.677383	6.763593	7.786937	6.640539	7.24626	7.217426	6.929596	7.183645	6.851311	7.056446	6.918889	7.239649	7.046326
Csf1r	0.45946	7.833593	2.037821	0.060206	0.162205	-5.31677	7.565418	7.70406	7.691254	6.833984	7.805057	7.438252	7.200847	8.962464	7.180063	8.598282	8.537862	9.361354	7.32556	7.473531
Mllt3	-0.456878	4.445276	-2.03775	0.060214	0.162205	-5.235094	4.602167	4.200444	4.424534	5.312821	4.387176	5.010363	4.421169	4.299035	4.31255	4.173561	4.369987	3.946285	4.592606	4.25465
Lats2	-0.524583	6.181336	-2.037497	0.060242	0.162244	-5.312281	6.424259	5.095359	5.884449	6.232328	6.050366	6.174802	6.855416	6.246128	6.097495	6.053845	6.123999	6.16667	6.763803	6.392432
Nfib	-1.156921	4.897606	-2.03683	0.060317	0.162408	-5.280899	5.055231	3.33211	5.316475	6.683686	4.282511	5.881799	4.53949	5.175775	4.723506	4.237532	5.11997	4.686283	5.409761	4.182799
Akap9	-0.429655	7.108181	-2.036595	0.060343	0.162442	-5.322692	6.74783	6.506004	6.838974	7.594562	6.529866	7.088475	7.412241	7.429189	7.317907	7.125766	7.165593	7.131687	7.424513	7.213674
Rgs19	0.361922	5.317444	2.035693	0.060444	0.162677	-5.297432	5.495845	5.409412	6.036296	5.570457	6.186213	5.941593	4.897007	5.188961	5.013766	5.036976	5.124097	4.86739	4.833902	4.883051
Myom1	0.91708	0.820065	2.035541	0.060461	0.162686	-4.440878	0.307952	1.369077	1.430088	0.531921	0.371962	2.04074	0.429655	0.645799	0.787836	1.253084	0.721218	1.082389	0.491415	0.929479
Zfp638	-0.398414	6.937058	-2.034604	0.060566	0.162932	-5.326082	6.708904	6.61946	6.68235	7.635413	6.737148	7.053091	7.08679	6.945222	6.99404	6.817325	6.919488	6.930214	7.050765	6.951584
Tsr3	0.364739	4.649169	2.033192	0.060724	0.163321	-5.205769	4.495729	4.946264	4.71425	4.203428	4.646377	4.537915	4.818933	4.685117	4.961869	4.879766	4.582868	4.461895	4.42791	4.789392
Zbtb20	-1.116655	3.709046	-2.032843	0.060763	0.163377	-5.061977	2.997401	2.151237	2.837276	4.895125	2.575715	3.862069	4.028845	4.775299	3.774234	3.58107	4.196624	4.056042	4.906128	3.432519
Loxl4	1.527087	1.508361	2.032613	0.060789	0.163377	-4.73638	2.402324	3.279374	4.740698	0.860705	1.85913	2.374054	-0.672244	1.628188	0.683763	1.481601	1.783193	1.252437	-0.040977	0.257494
RGD1305939	0.901101	6.02387	2.032541	0.060797	0.163377	-5.280255	4.791228	6.996686	6.244694	5.314499	5.322193	5.275609	6.233562	6.570181	6.343609	6.761692	6.238835	5.796318	5.674658	6.797383
Ostm1	-0.489419	6.058823	-2.032524	0.060799	0.163377	-5.32145	6.112706	5.261885	5.937813	6.582863	5.890403	6.340237	5.784094	6.230909	5.996579	5.854072	6.131194	6.301659	6.258637	6.16455
Pfkp	-0.275536	8.86661	-2.03234	0.06082	0.163396	-5.273603	9.241863	8.956451	9.488764	8.911949	9.265708	8.651204	8.547143	8.849851	8.529445	8.503225	8.488837	8.748464	8.728039
Msh2	0.282672	4.63677	2.0321	0.060847	0.163432	-5.211946	4.28818	4.91203	4.529394	4.520817	4.725866	4.605489	4.802122	4.721869	4.743058	4.609719	4.53717	4.625653	4.707853	4.649015
Rhbdd2	-0.476739	5.060067	-2.031646	0.060898	0.163532	-5.26975	5.333436	4.155166	4.97038	5.182896	5.178286	4.795709	4.785113	5.143629	4.964647	5.119326	5.202306	5.261346	5.440712	5.3563
Tsr2	-0.471826	3.933205	-2.031143	0.060954	0.163541	-5.251775	4.748409	4.192317	4.678213	5.304401	4.244704	5.106791	3.356296	3.484018	3.350165	3.129854	3.338699	3.277021	3.772036	3.195366
Trim44	-0.684505	5.815314	-2.031134	0.060955	0.163541	-5.328406	6.215844	5.616442	6.280438	7.220627	5.529352	6.709256	5.188472	5.81569	5.525598	5.114467	5.64437	5.460213	5.923087	5.200217
Osbp	-0.219738	6.586721	-2.031076	0.060962	0.163541	-5.328806	6.486379	6.215499	6.463608	6.563942	6.272434	6.270337	6.797221	6.704789	6.771731	6.645229	6.734801	6.734519	6.804266	6.765909
Mrps7	0.387549	5.362876	2.030854	0.060987	0.163541	-5.280263	5.460908	5.727471	5.397668	4.748906	5.644688	5.179547	5.377362	5.314586	5.426295	5.368229	5.51519	5.292883	5.221296	5.443886
Stx4	0.354288	5.907859	2.030851	0.060987	0.163541	-5.31802	5.967183	6.520683	5.925377	5.709586	6.250056	6.072488	5.886526	5.772547	5.784947	6.013956	5.613877	5.890805	5.502419	5.826046
Ppl	-1.359437	3.674374	-2.030789	0.060994	0.163541	-4.966904	3.724844	0.04804	3.035447	3.468286	1.390206	4.304105	3.810873	4.725493	4.68359	4.690302	4.407721	4.758654	4.298715	4.33484
Rcan2	-0.320375	7.158647	-2.030711	0.061003	0.163541	-5.327379	7.506249	7.561475	7.134905	7.663006	7.169214	7.188378	6.838203	6.914593	6.702679	7.092792	7.124981	7.311812	7.10576	6.918135
Zfp445	-0.293667	6.50572	-2.030651	0.06101	0.163541	-5.329741	6.286386	6.256029	6.252373	6.850271	6.327726	6.267002	6.670502	6.620683	6.559317	6.441081	6.700662	6.57932	6.737649	6.548559
Pigs	-0.369828	6.202492	-2.030514	0.061025	0.163546	-5.321267	6.28838	5.49119	5.642915	5.995551	6.218488	5.732621	6.276793	6.36591	6.444585	6.435247	6.3452	6.524748	6.388511	6.706862
Edn3	-1.019009	-0.262703	-2.030079	0.061074	0.163641	-4.198436	0.105685	-0.950476	-1.223881	-0.705589	-0.954037	-1.612049	0.202532	0.871929	-0.27702	1.104165	0.321999	1.375373	-0.040977	0.321874
Hpcal4	-0.480142	6.365982	-2.029709	0.061116	0.163642	-5.333453	6.988846	6.13404	6.57314	6.704952	6.139218	6.61927	6.053507	6.218103	6.218828	5.970246	6.359274	6.357722	6.891197	5.914821
Tfeb	0.365109	4.540701	2.029682	0.061119	0.163642	-5.202918	4.499177	4.830589	4.402616	4.054744	4.890715	4.428729	4.223227	4.643578	4.413615	4.839106	4.765698	4.895909	4.274664	4.475916
Kcnk13	0.757558	1.957686	2.029492	0.06114	0.163642	-4.640585	0.995438	1.397635	2.345554	1.63746	2.09374	2.374054	1.091998	2.539194	1.959523	2.359011	2.317912	2.816513	1.747621	2.159814
Sec11c	-0.37597	7.086984	-2.029355	0.061156	0.163642	-5.335056	7.008334	6.478697	6.480054	7.266861	7.158625	6.928807	7.11497	7.074467	7.319535	7.327154	7.09428	7.163491	7.472877	7.34146
Map7d3	-1.154898	0.086511	-2.02913	0.061181	0.163642	-4.306748	0.915118	-1.463779	-0.216022	-0.166914	-0.33871	-0.862697	0.118118	0.137473	0.091681	1.104165	0.998029	1.950783	0.249639	0.383504
Oma1	0.648728	2.397426	2.029118	0.061183	0.163642	-4.804828	2.357438	1.914859	2.709201	1.780306	3.00376	3.076678	2.305626	2.91426	2.024877	2.40749	2.729	2.680061	1.881043	2.087586
Fbxo25	-0.402421	5.870203	-2.029102	0.061185	0.163642	-5.317968	5.812263	5.02159	5.597812	6.138819	5.729254	5.938452	5.870489	6.019856	6.012778	5.894318	6.141411	5.948875	6.127142	5.95724
Trmt1	0.530921	6.594801	2.029045	0.061191	0.163642	-5.321967	5.968427	6.925538	6.074054	5.709586	6.528437	5.949937	7.225013	6.781652	7.052669	7.123349	6.61234	6.783214	6.474604	7.135787
Sowahc	0.515242	3.102499	2.028989	0.061197	0.163642	-5.045173	3.627383	3.232585	3.8018	2.881078	3.836938	4.082982	2.324585	3.070611	2.00342	3.040455	3.258364	3.331185	2.736815	2.402413
Nub1	-0.309134	6.80478	-2.028152	0.061292	0.163859	-5.337202	6.873365	6.38107	6.809605	7.007398	6.709027	6.625143	6.698025	7.026796	6.821626	6.675238	7.017039	6.817501	7.058415	6.760838
Slc4a8	-0.752228	4.646522	-2.027555	0.06136	0.164003	-5.19941	4.031456	3.986819	4.331892	5.169977	3.020129	4.105563	5.2443	5.31939	5.213859	4.782026	4.969824	4.747316	5.595968	4.604416
Tsen2	0.442379	4.589688	2.027216	0.061398	0.164038	-5.20536	4.346765	5.016996	4.377553	4.140492	4.703236	4.649696	4.897007	4.692543	4.953501	4.85376	4.287749	4.383501	4.360956	4.658395
Ralgds	0.460181	7.108804	2.027198	0.0614	0.164038	-5.33749	7.072808	7.838376	7.407767	6.602913	7.128274	6.812949	7.001737	6.780782	6.851205	7.129985	6.79752	6.996959	6.780267	7.074043
Fam69b	-0.343214	5.596157	-2.026633	0.061464	0.164173	-5.285659	5.487189	4.938996	5.107108	5.124787	5.183745	4.610761	5.998491	5.784758	6.078382	5.955377	5.901623	5.952583	5.958745	6.298567
Capn8	2.28615	-2.321777	2.025879	0.06155	0.164365	-4.011412	-2.235785	1.339943	-0.974047	-2.344749	-0.516166	-1.612049	-2.558588	-3.260591	-1.813089	-1.136114	-3.260591	-2.249472	-1.904936	-1.040339
Emg1	0.280318	4.76797	2.025468	0.061597	0.164454	-5.270091	4.922855	5.068969	5.147205	4.842596	5.189184	5.238629	4.667523	4.532109	4.726784	4.402091	4.678581	4.542821	4.579577	4.271049
Ppp4c	0.377504	5.245833	2.024391	0.061719	0.164744	-5.278402	5.134966	5.3631	5.43459	4.738944	5.609964	4.832273	5.246787	5.385024	5.2845	5.482012	5.388815	5.342704	5.134872	5.105095
Dclre1a	0.335665	4.659548	2.023818	0.061784	0.164882	-5.24118	4.485336	4.938996	4.544485	4.577819	5.13948	4.8591	4.481542	4.850248	4.596704	4.494755	4.632967	4.468815	4.539769	4.686175
Camsap1	-0.294518	7.223865	-2.023146	0.061861	0.16505	-5.344053	7.12004	6.77517	7.128659	7.508485	6.965074	7.20938	7.310709	7.375416	7.259143	7.210179	7.330052	7.322834	7.428151	7.201427
Ttll10	1.870659	0.307278	2.022804	0.0619	0.165117	-4.140675	-0.310971	-0.186163	0.398943	-2.344749	0.606834	-0.291112	1.544223	1.393205	1.360416	-0.311823	-0.731011	1.568131	1.815874	1.73247
Zbtb42	0.819958	1.192947	2.022352	0.061952	0.165211	-4.594601	1.401336	1.894965	2.31067	0.705659	1.895073	1.193889	1.297885	0.972957	-0.080923	0.981432	1.230182	1.284167	1.252133	1.085968
Slc1a2	-0.732386	7.690879	-2.022256	0.061963	0.165211	-5.323833	8.765153	7.963933	8.356744	8.193925	7.148889	7.530844	7.252499	7.289883	7.191968	7.008214	7.541164	7.74668	8.391829	7.298611
Crip2	0.466239	6.642995	2.022063	0.061985	0.165233	-5.345827	7.096902	7.293543	6.737564	6.1492	7.366937	6.950279	6.323487	6.243603	6.492541	6.681823	6.49454	6.508889	6.233904	6.444869
Ssbp3	-0.284347	6.825329	-2.021739	0.062022	0.165295	-5.345457	7.252602	6.95706	7.024873	7.524885	7.13016	7.304352	6.408134	6.433944	6.664739	6.392733	6.627683	6.460239	6.751512	6.635829
Trhde	-0.751482	5.593066	-2.021431	0.062057	0.165332	-5.309238	5.917798	3.854016	4.766671	5.477652	5.228454	5.90129	6.166842	5.854273	5.788087	5.396526	5.95125	5.895948	6.164705	5.976117
Pcsk6	-0.508024	4.977703	-2.02138	0.062063	0.165332	-5.254158	5.029233	4.658378	4.337679	4.753862	4.745687	3.808019	4.958988	5.465247	5.081888	5.380424	5.03922	5.801809	5.284886	5.396093
C1qtnf3	-4.03231	-0.641017	-2.020712	0.062139	0.165499	-4.194808	4.082914	-3.260591	-1.367151	-1.388744	-2.049163	-2.070862	-0.399109	-0.81357	-0.080923	1.704918	3.366837	-0.24119	-1.413173	-0.03329
Gnat2	2.21962	-0.222675	2.020512	0.062162	0.165518	-4.15202	-0.760787	1.010791	1.905712	-2.040259	-0.107758	-0.862697	-1.450335	0.137473	0.091681	1.828755	1.102128	-0.153565	-1.218554	-0.204094
Dclre1b	0.328365	4.056767	2.020408	0.062174	0.165518	-5.22751	4.388265	4.719039	4.975957	4.334907	4.387176	4.662444	3.40164	3.645768	3.59934	3.676473	3.901965	3.744381	3.800389	3.655003
Rab5b	-0.276829	8.355471	-2.019214	0.062311	0.165846	-5.31697	8.742604	8.294962	8.548129	8.748777	8.56239	8.472979	8.073684	8.189288	8.183127	8.095943	8.391458	8.186769	8.408502	8.082804
Scp2	-0.286699	7.214955	-2.018816	0.062357	0.165909	-5.347212	7.765857	7.419429	7.18472	7.39689	7.198724	7.378467	7.150601	6.844663	7.105236	7.256129	7.04343	7.095817	7.03534	7.144713
Sept4	-0.648136	7.870136	-2.018769	0.062363	0.165909	-5.34403	7.845555	8.17816	6.73318	7.851931	7.271887	6.613373	8.27015	8.166139	8.207082	8.409603	7.838884	8.322364	8.044282	8.436774
Ndst2	0.558535	5.658688	2.018068	0.062443	0.166087	-5.289762	4.96336	6.154055	5.580833	4.842596	5.161783	4.955481	6.136317	5.813989	6.126319	6.184782	5.700728	5.883055	5.602423	6.149107
Rarb	-0.417073	2.329204	-2.017557	0.062502	0.166207	-4.891412	2.542593	2.184396	2.162035	2.825673	2.28197	2.493017	2.613764	1.934092	2.347926	2.06872	2.243983	2.375822	2.689088	2.159814
Depdc5	-0.25427	6.130731	-2.016923	0.062575	0.166364	-5.350747	6.253078	6.249189	6.177711	6.585645	6.017113	6.220346	6.077457	6.04037	5.995221	5.912629	6.098006	6.007077	6.186353	6.032513
Taf9b	-0.653028	6.063394	-2.016645	0.062607	0.166413	-5.355125	6.467142	5.476206	6.106618	7.409113	6.571415	6.984693	6.054927	5.387314	5.757972	5.550173	5.796935	5.539588	6.039464	5.770899
Cnnm3	-0.320542	6.302385	-2.016284	0.062649	0.166487	-5.344388	6.152662	6.006581	5.727999	6.034761	5.566046	5.79647	6.72146	6.4808	6.573948	6.712696	6.471239	6.658627	6.413506	6.937527
Prpsap1	0.339289	5.501134	2.016051	0.062676	0.16652	-5.294406	5.167834	5.440624	5.102017	4.775953	5.471011	5.221502	5.847733	5.63672	5.792785	5.922393	5.540116	5.675901	5.544933	5.912209
Pogz	-0.241938	6.468986	-2.015724	0.062714	0.166585	-5.352124	6.263253	6.210477	6.112538	6.488651	6.09064	6.125933	6.827443	6.682587	6.698513	6.647754	6.675572	6.542076	6.51537	6.70309
Ppm1d	-0.306383	4.678071	-2.014521	0.062853	0.166918	-5.29117	4.909961	4.567042	4.712023	5.285703	5.032606	4.86573	4.376444	4.515458	4.628589	4.475935	4.681385	4.516347	4.533027	4.454537
Asgr1	1.640501	-0.060383	2.014024	0.062911	0.167034	-4.30221	0.539902	1.310208	0.945338	-1.575022	-0.516166	1.892796	-0.399109	0.76329	0.091681	-0.210791	-0.411343	0.514819	-1.218554	-0.621773
Dgki	-0.684077	5.19262	-2.013579	0.062962	0.167059	-5.312567	4.958358	4.801498	5.128966	6.060988	4.043216	5.353145	5.448228	5.630934	5.469972	4.989994	5.414317	5.087922	5.442513	4.912095
Mbnl2	-0.470681	7.685977	-2.013448	0.062977	0.167059	-5.342505	7.879353	7.348518	7.89461	8.641065	7.800622	8.110426	7.563047	7.581438	7.581779	7.138388	7.639591	7.27626	7.784203	7.372189
Yes1	0.481022	2.811115	2.013419	0.062981	0.167059	-4.966568	2.730329	3.117121	3.393138	2.738361	2.712936	3.595796	2.450751	2.665313	2.720913	2.572488	2.506778	2.703718	3.013624	2.660694
Hyal1	-0.720632	2.531649	-2.013392	0.062984	0.167059	-4.989356	4.10118	2.570375	2.726949	2.41457	2.322129	2.386402	2.100922	2.030999	2.47808	2.103365	2.750518	2.749899	2.480628	2.503027
Chuk	0.268548	5.375337	2.013343	0.062989	0.167059	-5.318302	5.225173	5.610355	5.56118	5.299326	5.50339	5.450441	5.657098	5.08559	5.469972	5.297103	5.170732	5.078905	5.256515	5.627027
Lrrc4b	-0.340976	8.917743	-2.012967	0.063033	0.167138	-5.323755	8.851311	8.128976	8.536854	8.90437	8.838715	8.647273	9.148144	9.06543	9.229386	9.106874	8.909168	9.15602	9.010105	9.319118
Atp6v1e1	-0.380562	8.49737	-2.012745	0.063059	0.167169	-5.32988	8.751846	7.978185	8.326505	8.674443	8.631272	8.399686	8.487618	8.556329	8.649665	8.376498	8.760335	8.28683	8.793239	8.295129
Ghitm	-0.364285	7.238384	-2.011953	0.063151	0.167367	-5.36161	7.309678	6.691147	7.121129	7.532475	7.202315	7.21285	7.207246	7.194388	7.498911	7.025142	7.486911	7.176758	7.673212	7.295608
Dio3	0.927314	0.530371	2.011864	0.063161	0.167367	-4.444156	0.873216	0.632508	1.631973	-0.325128	0.808768	1.302125	0.028455	0.222004	0.245823	0.134646	0.378374	0.403657	0.921344	1.29285

Vps9d1	0.616388	6.134905	2.011141	0.063246	0.167553	-5.33403	5.658863	6.742146	6.043396	5.147559	5.983075	5.298657	6.578725	6.309075	6.606334	6.580631	6.163637	6.145232	6.015477	6.63978
Eml1	-0.372508	5.978603	-2.010503	0.06332	0.167713	-5.349637	6.035262	6.031851	5.250517	5.913781	5.700344	5.440113	6.246047	6.180309	6.182295	6.013956	5.861397	6.325681	6.239092	6.305549
Fam210b	-0.398426	3.5841	-2.009282	0.063462	0.168054	-5.14428	4.163349	3.287027	3.447672	3.617117	3.359399	3.826262	3.677127	3.475434	3.324439	3.471326	3.792123	3.811244	3.45286	3.604134
Msto1	0.344326	4.483473	2.009075	0.063487	0.168059	-5.232595	4.193457	4.482919	4.559421	4.291262	4.921539	4.383247	4.746971	4.429191	4.571407	4.509637	4.534071	4.265266	4.314529	4.636413
Atp2c1	-0.52367	7.859472	-2.009027	0.063492	0.168059	-5.355685	7.640324	6.932264	7.358093	8.430113	7.628151	8.123855	8.032838	7.943833	7.983895	7.798973	7.812272	7.964594	8.160125	8.230303
Gfm1	0.225746	5.844699	2.008795	0.063519	0.168085	-5.357825	6.057649	6.235412	6.064489	5.865378	6.286034	6.160494	5.809551	5.532995	5.751551	5.539336	5.557006	5.533	5.663896	5.796614
Lrpap1	-0.317759	7.123682	-2.008704	0.06353	0.168085	-5.369875	7.047717	6.508461	6.680074	7.055853	6.953402	6.794536	6.897967	7.438052	7.32927	7.439729	7.338957	7.441907	7.335282	7.48192
Slu7	-0.274001	6.404029	-2.007181	0.063708	0.168519	-5.372126	6.713364	6.394416	6.656536	7.030553	6.549029	6.792218	6.059178	6.404856	6.111352	6.18941	6.258681	6.209646	6.230782	6.07516
Tgoln2	-0.437331	7.496058	-2.006508	0.063787	0.168691	-5.366163	7.653148	6.681018	7.460768	7.767467	7.465104	7.489231	7.167107	7.626233	7.433729	7.531904	7.577361	7.696715	7.843992	7.559902
Wdr5	0.307339	5.245094	2.006191	0.063825	0.168753	-5.300548	4.990561	5.302095	5.137285	4.71881	5.280338	4.919823	5.517835	5.309765	5.462129	5.542957	5.332856	5.283102	5.198041	5.477837
Cnppd1	0.288689	5.222602	2.005258	0.063934	0.169005	-5.314642	5.108122	5.302095	5.583271	4.921791	5.183745	5.130842	5.170246	5.240531	5.114779	5.3205	5.296745	5.425065	5.059455	5.301477
Psip1	-0.587173	7.791149	-2.005134	0.063949	0.169007	-5.346703	8.148607	7.502713	8.04655	9.134695	7.940216	8.720878	7.548001	7.680455	7.551605	7.253921	7.445457	7.216359	7.606535	7.287571
Pik3r3	-0.614434	3.885744	-2.004643	0.064007	0.169122	-5.244573	4.15463	3.418258	4.320248	5.046174	3.607042	4.600198	3.419386	3.966821	3.83015	3.393233	3.50618	3.732929	3.98464	3.531125
Galnt16	-0.37487	8.41485	-2.004401	0.064035	0.16916	-5.358517	8.046264	7.352626	8.123313	8.345444	8.038905	7.772261	8.613881	8.758906	8.799774	8.724831	8.644072	8.762436	8.834782	8.995307
Csrp2bp	0.312815	4.004751	2.00394	0.064089	0.169231	-5.220855	4.007445	4.407694	4.581538	4.185201	4.3745	4.274516	4.040359	3.763261	3.767885	3.844356	3.760656	3.926315	3.777752	3.454224
Lmo2	-0.436549	5.002456	-2.003934	0.06409	0.169231	-5.295689	4.859884	3.943895	4.475294	5.130514	4.879544	4.928293	5.108686	5.275563	5.243904	5.249138	5.352337	5.458476	4.964926	5.215125
Atp6v0a4	-0.531064	2.603432	-2.003497	0.064142	0.16933	-4.790309	1.9771	1.339943	1.554575	2.350586	2.062471	1.648138	3.231264	2.822481	3.440756	3.463704	3.173291	3.083214	3.160849	3.43979
Mrpl15	-0.417601	4.722789	-2.003119	0.064186	0.169411	-5.31498	5.038741	4.368558	4.59368	5.362331	4.923716	5.250499	4.718584	4.420274	4.697018	4.311581	4.730939	4.53295	4.797792	4.432837
Jmjd7	0.588567	2.312092	2.002557	0.064253	0.169549	-4.754501	1.741237	2.894758	1.966737	1.571628	2.062471	2.071653	2.629097	2.58779	2.858731	2.325762	2.401899	2.461578	2.333172	2.791492
Sgol2	2.37464	-1.170612	2.002001	0.064318	0.169685	-4.117467	-1.642648	-0.103798	-1.526232	-2.040259	1.143601	1.353343	-0.279766	-0.81357	-1.813089	-0.806258	-2.697527	-0.78301	-1.413173	-0.621773
Pi4k2b	0.438028	2.467641	2.00153	0.064374	0.169791	-4.932466	2.387517	2.595246	3.070623	2.591079	2.762423	3.242073	2.450751	2.488905	2.04602	2.127786	2.149958	2.284645	2.220182	2.417223
Trerf1	-0.365891	5.864129	-2.001423	0.064387	0.169791	-5.363806	5.696893	5.409412	5.652234	6.195466	5.593651	5.67087	6.27801	6.043277	6.209488	6.047485	5.760279	5.689294	5.724337	6.154642
Ptplad1	-0.335882	7.748442	-2.001034	0.064433	0.169863	-5.374522	7.693211	7.060029	7.221571	7.745866	7.65853	7.595754	7.927878	7.793971	7.872911	8.058483	7.753207	7.991873	7.968219	8.144181
Irf2	0.267116	4.295038	2.000954	0.064442	0.169863	-5.23112	4.22295	4.320894	4.556942	4.074684	4.348811	4.413727	4.134688	4.481568	4.055381	4.294496	4.400933	4.575244	4.187182	4.14326
Smcr8	-0.682042	4.335607	-2.000812	0.064459	0.16987	-5.266378	4.664912	3.411272	4.586407	4.863379	3.930833	4.358391	3.857182	4.718236	4.259232	3.808701	4.80736	4.465359	5.016594	4.032198
Lancl2	-0.541636	5.827549	-2.000561	0.064489	0.169911	-5.37499	6.091164	5.157212	6.020192	6.662102	5.873611	6.284426	5.630681	5.847636	5.743484	5.286341	5.907622	5.417931	6.058371	5.633383
Vps13a	-0.464074	6.238233	-2.00036	0.064513	0.169937	-5.374358	5.810876	6.015821	5.989274	6.774877	5.389886	5.931096	6.80568	6.367071	6.609	6.004794	6.241687	6.090202	6.786659	6.540127
Pm20d1	1.072834	0.706128	1.999898	0.064568	0.170044	-4.463251	1.212615	1.812527	1.293837	-0.705589	0.517419	1.221719	0.68572	0.871929	0.17081	0.280604	1.230182	1.11804	0.249639	0.970221
Chchd3	-0.243806	6.225522	-1.999308	0.064638	0.170191	-5.38254	6.551758	6.146713	6.22921	6.605657	6.505368	6.458985	6.156298	5.997547	6.121347	6.042377	6.116768	5.967317	6.163614	6.115448
Atp5g3	-0.308934	7.151384	-1.999069	0.064666	0.170217	-5.383573	7.294876	6.826616	7.124899	7.458137	7.252535	7.066582	7.351143	6.91618	7.342682	6.89447	7.320641	6.805251	7.260718	7.215793
Rnf112	-0.3443	8.994735	-1.998985	0.064676	0.170217	-5.350988	8.608168	8.236138	8.488901	8.91104	8.69574	8.238546	9.727464	9.125299	9.627292	9.334439	8.99435	9.058186	9.25845	9.625564
Prps1	-0.476224	6.404686	-1.998788	0.0647	0.170242	-5.385933	6.80884	5.810791	6.738658	7.143898	6.874016	6.57478	6.220969	6.141498	6.29034	5.986294	6.352254	6.030652	6.578127	6.133497
Sars2	0.477242	4.505564	1.998612	0.06472	0.17026	-5.254501	4.369549	5.116989	4.841896	4.125276	4.801203	4.131469	4.685967	4.485848	4.589521	4.683756	4.140682	4.357637	4.250206	4.568381
Mvd	-0.310864	4.522292	-1.99816	0.064774	0.170364	-5.288066	4.975787	4.535265	4.380359	4.560953	4.677668	4.230676	4.685967	4.239446	4.771898	4.210463	4.421201	4.296718	4.803407	4.591416
Ssr2	0.363932	5.646108	1.997649	0.064835	0.170487	-5.325777	5.099062	5.273489	5.206917	4.849557	5.695256	5.167077	5.675675	5.883764	5.882203	6.263712	5.973152	6.068727	5.87324	6.166743
Scube3	-0.680037	1.402696	-1.997442	0.06486	0.170514	-4.666684	2.279379	0.854759	0.818644	1.033666	0.770579	1.402804	1.576217	1.856886	1.636128	1.481601	1.959843	1.404538	1.651284	1.529341
Tyro3	-0.308616	7.864706	-1.997293	0.064878	0.170524	-5.380964	7.933557	7.521486	7.303325	7.467595	7.524039	7.222352	8.075434	7.945	8.175952	8.198521	8.026691	8.266372	8.129163	8.323414
Dnmt3a	-0.427862	4.710676	-1.996704	0.064948	0.170671	-5.275866	4.272147	4.368558	4.220415	5.072196	4.006719	4.431711	5.271424	5.039747	5.151822	4.763521	4.661644	4.995108	4.847553	4.909479
Prr22	0.564896	2.18024	1.99651	0.064971	0.170677	-4.8036	1.853246	2.667383	2.487668	1.818649	2.484288	1.943815	2.450751	1.830204	2.347926	2.572488	2.149958	2.064785	1.815874	2.387449
Wipf3	-1.035908	9.23582	-1.996365	0.064988	0.170677	-5.324805	9.562764	7.154568	9.017373	10.1342	9.130768	9.964607	9.565317	9.534728	9.125984	9.118904	9.578087	9.168825	9.307428	8.940936
Hsph1	-0.609807	8.451513	-1.996329	0.064993	0.170677	-5.359637	8.411095	7.157702	8.258538	8.999105	8.426428	8.54152	8.466435	8.640083	8.672575	8.344951	8.735765	8.411933	8.81303	8.446733
Alg6	0.445761	3.170209	1.995084	0.065141	0.17103	-5.006537	2.742017	3.418258	3.515619	2.835056	2.810268	3.179592	3.632178	3.047633	3.307029	3.00938	3.082885	3.261164	3.160849	3.558103
Coro7	-0.281747	7.348121	-1.993841	0.06529	0.171348	-5.394688	7.195302	6.821356	6.838464	6.916957	6.796554	6.64262	7.955508	7.826397	7.686051	7.733134	7.602354	7.648389	7.478591	7.742244
Crmp1	-0.471043	7.810737	-1.993834	0.065291	0.171348	-5.379773	7.853658	6.913847	7.881299	8.290872	7.771308	7.842001	7.687721	7.918726	7.970825	7.736301	8.006991	7.729136	8.036257	7.718525
Amy1a	3.598144	-1.924655	1.993549	0.065325	0.171368	-3.97978	-2.659044	-0.950476	1.874203	-3.260591	-1.798946	-1.823292	-2.088409	-1.720374	-2.155202	-1.33445	1.260496	0.514819	-2.232431	-2.653844
RGD1312005	-0.469449	6.204471	-1.993535	0.065327	0.171368	-5.391888	6.413335	5.570149	6.17448	6.885693	6.400647	6.57478	5.922749	6.149614	6.226951	6.035967	6.300311	5.973412	6.354495	5.901716
Rdh14	-0.529577	4.620943	-1.993067	0.065383	0.171478	-5.268341	4.421353	3.1924	4.106393	4.785664	4.499426	4.475713	4.92212	4.816662	5.037713	4.876899	4.706374	4.941394	4.947283	5.032408
Cnih4	0.353559	4.532715	1.992434	0.065459	0.17164	-5.329686	4.865238	4.859106	5.32524	4.969434	5.303926	5.58375	3.927077	4.055878	3.918782	4.091865	3.930525	4.31219	4.212723	4.174106
Caln1	-0.608951	3.316278	-1.992102	0.065499	0.171678	-5.164368	3.748215	3.224637	3.826705	4.094353	2.72297	3.47381	3.049479	3.413873	3.059216	2.707689	3.401255	3.037402	3.98464	2.847239
Scn3a	-0.440189	5.842204	-1.991962	0.065516	0.171678	-5.374258	5.459139	5.476206	5.685517	6.241177	4.974995	5.515125	6.43237	6.016901	6.307951	5.887213	5.898012	5.953816	5.973761	5.997217
Syne3	0.496393	2.452233	1.991873	0.065527	0.171678	-4.899357	2.41698	2.982209	2.626517	2.077719	2.361201	2.965855	2.8014	2.400986	2.493553	1.804823	2.317912	2.447635	2.408783	2.516844
Cpeb4	-0.600933	6.550535	-1.991838	0.065531	0.171678	-5.395952	6.322839	6.173797	6.697056	7.33044	5.536481	6.497203	6.787859	6.819449	6.820092	6.218007	6.696511	6.328537	6.907581	6.58918
Zfand2a	0.359311	5.30568	1.991267	0.0656	0.17182	-5.354109	5.384666	5.423367	6.055737	5.068223	5.471011	5.370341	5.183288	5.119041	5.084445	5.194928	5.278346	5.229104	5.3611	5.09592
Acox2	0.948592	0.373607	1.990909	0.065643	0.171896	-4.384588	-0.760787	0.973336	0.158563	0.33436	1.018831	0.695385	0.563361	0.818632	0.512061	0.847278	0.378374	1.252437	-0.405292	0.119416
Rhog	0.3551	6.095706	1.990488	0.065694	0.171958	-5.391713	6.29831	6.473676	6.327003	5.859643	6.714067	6.216028	5.368254	6.150963	5.687401	6.141249	6.107418	6.439254	5.70497	5.875144
Zdhhc14	-0.482227	6.639008	-1.990452	0.065698	0.171958	-5.397149	6.540926	5.36852	6.374928	6.793045	6.483401	6.508544	6.881257	6.867805	6.910047	6.695716	6.714415	6.877235	6.96632	6.980541
Rhbdl3	-0.37665	5.824928	-1.990358	0.065709	0.171958	-5.373641	5.735409	5.161381	5.840289	5.780507	5.361263	5.152692	6.173393	5.709895	5.935576	6.021763	6.040209	5.957511	6.337182	6.370721
Sdcbp	-0.327277	8.210659	-1.989727	0.065785	0.172119	-5.376808	8.240053	7.715093	8.01168	8.581859	8.238989	8.367932	8.231119	8.146334	8.308167	8.104852	8.261896	8.2238	8.325091	8.197702
Mitd1	0.359963	4.098773	1.989421	0.065822	0.172167	-5.236117	4.064414	4.452618	4.317322	3.980586	4.463969	4.291029	4.39898	3.884925	3.935874	4.061889	3.897149	4.196062	3.582598	3.948012
Ppp1r7	-0.497236	6.891115	-1.989338	0.065832	0.172167	-5.398095	7.288414	6.43889	7.190728	7.773579	6.999018	7.505242	6.541734	6.744626	6.600093	6.486922	6.868736	6.625724	7.029364	6.396174
Glud1	-0.288085	8.642563	-1.989148	0.065855	0.17219	-5.366266	8.97718	8.502092	8.3082	8.571897	8.522014	8.617957	8.750276	8.556329	8.541699	8.720841	8.576214	8.736508	8.76995	8.84869
Ost4	0.495183	5.231401	1.988011	0.065993	0.172512	-5.373591	5.649584	5.675942	5.477159	5.22989	6.656987	5.915205	4.648839	4.856872	4.852113	4.815347	5.067356	4.761475	4.939655	4.737232
RGD1563325	-0.407455	3.03131	-1.986436	0.066184	0.172974	-5.053544	3.197423	2.769246	2.787402	3.233029	2.553396	3.136381	3.127125	3.242343	3.130231	2.923085	3.189137	2.761217	3.522595	3.060363
St3gal1	0.724054	2.148597	1.985557	0.066291	0.173215	-4.911578	2.488111	2.667383	3.484656	1.92792	2.268331	3.143674	1.478026	2.227821	1.891068	1.322149	1.482482	2.419337	2.022549	1.634478
Ptprj	-0.532295	7.740288	-1.985241	0.066329	0.173271	-5.402028	7.294876	6.462737	7.571122	8.122923	7.104484	7.566549	8.104177	7.992022	8.144441	7.844803	7.842959	8.031263	8.092658	8.196898
Cpne9	-0.470349	4.007796	-1.985144	0.066341	0.173271	-5.184408	3.875708	4.155166	3.070623	3.933316	3.464807	3.143674	4.579502	4.101219	4.575048	4.320048	4.136602	4.153748	4.221137	4.482972
Lrrc27	-0.431216	1.862983	-1.984857	0.066376	0.173295	-4.854877	2.32672	1.953842	1.858187	2.124384	1.765147	1.627481	1.754571	1.856886	1.937064	1.964563	1.803924	1.515634	2.269694	1.755962
Crabp1	0.686553	2.966699	1.984831	0.066379	0.173295	-5.151464	3.652372	3.302211	4.166157	3.130069	4.427618	4.304105	1.335758	1.934092	2.55384	2.745897	2.66245	2.100868	2.816716	2.635584
Angptl6	0.920294	2.024867	1.984144	0.066463	0.173469	-4.764343	1.48718	3.125682	2.812554	1.502648	2.373993	1.327961	2.05625	1.80302	2.493553	2.108365	2.117203	1.593679	1.626159	2.325991
Elp2	-0.277215	5.964065	-1.983916	0.06649	0.173469	-5.393933	5.812263	5.388222	5.768195	6.067969	5.793748	5.70084	6.118774	6.018379	6.249911	5.921002	6.13529	5.946399	6.349707	6.251825
Prkar1a	-0.595578	8.066896	-1.983866	0.066497	0.173469	-5.37735	8.366014	7.5336	8.244937	9.22824	8.091647	8.932098	7.702292	7.8733	7.923413	7.636872	7.786138	7.752009	8.147213	7.724864
Tmtc4	-0.354715	4.676417	-1.983811	0.066503	0.173469	-5.315101	4.59574	4.57956	4.311452	5.029924	4.345567	4.629061	4.559635	4.544473	4.896441	4.494755	4.725516	4.978233	4.919116	4.922513
Pafah1b1	-0.644475	8.723699	-1.983673	0.06652	0.173475	-5.360453	8.844381	8.009746	8.849173	9.804749	8.625934	9.231468	8.558651	8.634311	8.494895	8.174501	8.821024	8.532012	9.141832	8.412957
Galnt1	0.333636	5.738735	1.983374	0.066557	0.173532	-5.391328	5.808099	5.87567	6.077517	5.556344	5.900385	6.200378	5.386413	5.516354	5.43233	5.611814	5.530819	6.001123	5.753633	5.721041
Plec	0.421271	8.56113	1.983044	0.066597	0.1736	-5.392221	7.762633	8.857933	8.376557	8.207309	8.153468	8.226363	9.048497	8.884979	8.939967	8.846492	8.434677	8.727881	8.500159	8.893235
Dapp1	0.595893	3.169723	1.981671	0.066765	0.173999	-4.970379	2.8647	2.631765	2.845423	2.093442	3.280067	3.414757	2.629097	4.572916	2.47808	4.425821	4.111876	4.653252	2.022549	2.55752
Aspdh	0.934459	3.071407	1.981332	0.066806	0.17407	-4.916642	2.474162	4.099756	3.253175	1.818649	2.828971	2.101918	3.356296	3.19084	3.510866	3.715505	2.86345	3.245131	3.143247	3.604134
Tanc1	-0.898369	7.190632	-1.981021	0.066844	0.174095	-5.413077	6.752171	5.254098	6.953644	8.028189	6.300345	7.444539	7.26052	7.711396	7.668264	7.263281	7.528764	7.427327	7.76491	7.323884
Rgl1	-0.37764	6.922372	-1.981018	0.066845	0.174095	-5.414594	7.054763	6.541627	7.005462	7.542533	6.886904	7.248376	6.903494	7.025326	6.792209	6.5964	6.903969	6.780435	6.956905	6.687903
Smoc2	1.483955	0.567313	1.980702	0.066883	0.174117	-4.395144	0.428583	2.439156	0.715742	-1.075778	-0.18071	1.221719	0.903114	0.377517	0.385075	1.21727	0.535373	1.433124	0.434663	0.321874
Tyms	0.402967	2.41632	1.980702	0.066883	0.174117	-4.99648	2.682606	3.224637	2.901201	2.591079	2.828971	3.200721	1.809437	1.934092	2.066858	1.920699	2.303426	1.97042	2.452318	2.245294
Actn4	0.279813	8.124487	1.980594	0.066897	0.174117	-5.396325	8.23515	8.474501	8.293015	7.841203	8.374659	8.13144	8.037514	7.976898	8.155427	8.127942	7.967052	8.095267	7.909884	8.128517
Phc2	0.304169	6.740135	1.980458	0.066913	0.174123	-5.414399	6.675771	6.861631	7.02756	6.293164	6.817191	6.445034	6.759403	6.825372	6.596515	6.909276	6.885245	6.849666	6.737649	6.693236
Ints12	0.413673	2.859119	1.980251	0.066939	0.174151	-5.075264	3.230989	3.33211	3.656563	2.806722	3.452798	3.214637	2.518182	2.471744	2.364852	2.499819	2.912	2.555581	2.923692	2.310209
Polm	-0.471348	4.204217	-1.979291	0.067057	0.174383	-5.217373	3.907194	4.064569	3.253175	4.185201	3.691225	3.37813	4.591292	4.632039	4.676828	4.738473	4.394114	4.613215	4.061622	4.763548
Btrc	-0.470019	5.763612	-1.979287	0.067057	0.174383	-5.408537	6.019742	5.286909	5.887411	6.502703	5.890403	6.098051	5.443899	5.796866	5.452264	5.305655	5.93019	5.567256	6.019101	5.519611
Ocln	-0.663324	4.457987	-1.979067	0.067084	0.174416	-5.297328	5.339219	4.180039	3.404211	4.07864	4.584435	4.005051	4.67123	4.527965	4.707008	4.839106	4.561722	5.056112	4.089463	4.443728
Sspo	-1.222719	0.858586	-1.978746	0.067124	0.174461	-4.397993	-0.129637	0.369671	-0.491181	0.966949	-1.798946	-0.548781	2.324585	1.429209	2.493553	1.986005	1.456531	1.515634	1.436856	2.387449
Cant1	-0.332613	5.80888	-1.978691	0.06713	0.174461	-5.387909	5.806708	4.907073	5.408707	5.485121	5.543576	5.376544	5.873711	6.063464	6.062907	6.102509	6.164639	6.264362	6.107988	6.186329
Gemin4	0.508262	3.405862	1.9783	0.067178	0.174504	-5.056874	3.026365	3.953545	3.404211	2.738361	3.306999	2.898483	3.593621	3.349568	3.540964	3.676473	3.66758	3.755742	3.627601	3.294952
Specc1l	-0.324797	5.629613	-1.978283	0.067181	0.174504	-5.404242	5.824683	5.425102	5.795786	6.14826	5.616707	5.809137	5.456846	5.539186	5.420236	5.301385	5.667178	5.589649	5.834006	5.418348
Tubgcp3	0.259954	5.939567	1.9782	0.067191	0.174504	-5.406586	5.809488	6.276355	6.281189	5.955246	5.941785	6.071533	5.985148	5.796866	5.995221	5.898564	5.835201	5.725858	5.753633	5.853529
Gpr12	-0.999574	4.575005	-1.97791	0.067227	0.17455	-5.31633	4.870572	2.134368	4.162901	5.201152	4.481806	5.282232	4.696922	4.771798	4.723506	4.744776	4.773602	4.677349	4.911337	4.698352
Gspt2	-0.62811	4.973319	-1.977821	0.067238	0.17455	-5.359377	4.958358	3.758163	4.868125	5.677346	5.113018	5.008367	5.038651	5.194202	5.195053	4.680472	5.186605	4.73014	5.4116	4.858845
Smarcal1	0.377359	4.11294	1.976804	0.067363	0.174838	-5.223757	3.932918	4.525594	4.308508	3.665254	4.023054	3.835297	4.223227	4.145178	4.231815	4.369831	4.254248	4.200226	3.959716	3.998126
Slc6a5	0.807564	1.309559	1.976662	0.06738	0.174845	-4.825667	1.341131	3.000943	3.151703	2.124384	1.947363	2.086865	0.028455	0.137473	0.571595	0.646479	0.924208	0.666985	1.150071	1.29285
Ppp3cb	-0.752434	8.29424	-1.975692	0.0675	0.175114	-5.391883	8.414979	6.963044	8.157457	9.196856	8.337384	8.757136	8.294695	8.358426	8.233241	7.928512	8.539408	8.171474	8.736775	8.035294
MGC116197	-0.342656	3.962838	-1.97559	0.067513	0.175114	-5.245625	4.012279	3.817493	3.871277	4.277563	4.027109	3.519371	4.33497	3.811801	4.263751	3.747244	3.981461	3.656194	4.318455	3.942904
LOC690918	-1.318459	3.663862	-1.975431	0.067532	0.175117	-5.094924	4.163349	-0.273517	3.259286	3.36843	3.339971	3.242073	4.320877	4.527965	4.575048	3.850214	4.404331	3.83286	4.806206	4.120817
Car7	-0.451162	5.024889	-1.975344	0.067543	0.175117	-5.359617	5.229331	4.397125	4.707561	5.162543	4.895159	4.969911	5.469678	5.340814	5.332613	5.244698	5.341744	4.797651	4.780817	4.728352
Ttc5	-0.237937	5.64198	-1.975006	0.067585	0.175187	-5.399604	5.821932	5.514194	5.408707	5.607886	5.557661	5.426725	5.837869	5.763762	5.714065	5.616977	5.781339	5.536298	5.70497	5.726997
Bcl6b	0.512858	3.11166	1.973946	0.067716	0.17549	-5.057131	2.718545	3.466233	3.750662	2.977436	3.044338	3.172479	3.337752	3.252427	2.772565	3.241039	2.739799	3.433771	2.725031	3.11589
Csrnp3	-0.904473	3.194807	-1.973408	0.067783	0.175592	-5.120287	2.977763	2.466372	3.277465	4.294667	2.030509	3.22842	3.261094	3.997121	3.375441	2.614395	3.603988	3.018661	4.023644	2.745289
Irak1	0.359491	7.720022	1.973363	0.067789	0.175592	-5.419883	7.659325	8.203402	8.018451	7.34857	7.668948	7.563154	7.949413	7.615042	7.767466	7.752812	7.515471	7.597994	7.51583	7.911968
Ptgr2	-0.327004	6.020232	-1.973275	0.067799	0.175592	-5.422206	6.637172	6.185104	5.907005	6.122628	6.00071	6.118551	6.024813	5.605591	5.81451	5.963506	5.748297	6.077808	6.009418	6.092564
Entpd1	0.726949	1.606831	1.973112	0.06782	0.175606	-4.774628	2.10932	1.747452	2.47722	0.932396	2.109124	2.386402	1.408645	1.356279	1.489496	1.322149	1.068256	2.203999	1.493524	0.929479
Il12rb2	-0.610563	1.457133	-1.972877	0.067849	0.175644	-4.660921	1.033981	1.310208	0.52603	1.760745	0.606834	0.949514	2.100922	1.908813	2.024877	1.678822	1.803924	1.869448	1.881043	1.556352
Phkg1	-0.944305	2.394648	-1.970911	0.068094	0.176239	-4.809382	2.765113	2.028784	0.20995	1.593908	2.183675	0.695385	3.105363	3.093229	3.271566	3.070874	2.771719	3.30822	2.748504	3.079111
Vdac1	-0.483314	5.9913	-1.970352	0.068163	0.17637	-5.431538	6.527481	5.662767	6.396575	6.997508	6.28857	6.76765	5.726455	5.774298	5.695787	5.294957	5.893184	5.475754	5.839481	5.563542
Ppp1r14a	-0.509626	5.313956	-1.970262	0.068174	0.17637	-5.399208	5.770073	5.466129	4.586407	5.563417	5.77458	4.825487	4.980068	5.260653	5.353963	5.41047	5.287575	5.630336	5.132643	5.394223
Itm2c	-0.430854	10.02725	-1.969897	0.06822	0.17637	-5.361068	10.07616	8.916152	9.651387	9.651703	9.793212	9.988667	10.06157	10.27172	10.479	10.38002	10.37362	10.32034	10.40286	10.61667
Patz1	-0.24513	5.04478	-1.969852	0.068226	0.17637	-5.396524	5.16132	4.846214	4.919194	5.220971	4.949583	5.070904	4.943741	5.039747	4.870008	5.074991	5.276493	5.155958	5.134872	5.0106
Fgfr4	-1.731422	-0.080945	-1.969838	0.068227	0.17637	-4.3396	0.243629	-3.260591	-0.195392	0.33436	-0.516166	-0.072565	0.563361	1.067371	0.317129	1.51148	0.924208	0.567345	-0.211665	0.844371
Gca	-0.31773	5.319641	-1.969695	0.068245	0.17637	-5.407025	5.627699	5.315253	5.17333	5.805781	5.683743	5.432691	5.603771	4.816662	5.199777	4.859579	5.144558	5.125629	5.330318	5.396093
Chac2	-0.547919	2.607989	-1.969684	0.068247	0.17637	-5.051732	3.045357	1.812527	2.548818	3.211576	2.828971	3.114281	2.73209	2.539194	2.611707	2.308845	2.81321	2.136071	2.701169	2.372328
S100pbp	-0.413666	4.937426	-1.969504	0.068269	0.17639	-5.362531	4.788412	4.506054	4.819324	5.405483	4.352047	5.06899	5.157085	5.292766	5.066451	4.850841	5.030451	4.916302	5.0524	4.869653
Ccdc137	0.304521	4.439963	1.969183	0.068309	0.176455	-5.299671	4.036211	4.690471	4.561895	4.479531	4.641095	4.386324	4.468816	4.36558	4.445789	4.640468	4.30603	4.350161	4.387364	4.472374
Ly6e	-0.431613	7.616023	-1.96846	0.068399	0.176651	-5.430098	7.726686	6.676655	7.333324	7.605912	7.707676	7.226221	7.355183	7.794834	7.836591	7.920182	7.832277	7.981628	7.850108	7.785335
Fat3	-0.644466	4.943229	-1.968203	0.068432	0.176696	-5.34427	4.47136	4.993802	4.462108	5.451963	3.644478	4.240912	5.370537	5.526777	5.405997	4.763521	5.263455	4.948838	5.616844	5.100515
Creb3	0.244076	5.576452	1.967238	0.068553	0.17696	-5.410488	5.635554	5.677398	5.877513	5.438176	5.813839	5.745858	5.326541	5.488896	5.63778	5.455357	5.39907	5.508024	5.485056	5.61423
Sgsm3	-0.226035	6.240064	-1.967152	0.068563	0.17696	-5.429312	6.180847	6.107273	6.024683	6.193644	5.888175	5.827366	6.557847	6.335402	6.502136	6.314384	6.319335	6.209646	6.402451	6.518828
Plcb4	-0.393191	4.769559	-1.966205	0.068682	0.177228	-5.365761	4.857199	5.129812	4.725329	5.265025	4.460974	4.392459	4.974076	4.803005	4.852113	4.719397	4.454361	4.656286	4.576302	4.96597
Olr34	-0.874076	0.589121	-1.965091	0.068822	0.177552	-4.441754	0.592497	-0.273517	-1.223881	0.22441	0.027932	-0.006501	1.754571	0.923327	1.393785	1.355481	1.230182	1.045835	0.879431	1.582867
Zbtb16	-0.599637	4.731225	-1.964782	0.068861	0.177614	-5.388907	5.057571	5.123415	5.071085	5.758485	4.27571	4.793392	4.626095	4.873301	4.571407	4.224061	4.770972	4.051439	5.094225	4.007943
Slc25a28	0.373943	6.045589	1.964463	0.068901	0.177679	-5.428196	5.934448	6.642014	6.340035	5.715949	6.0038	5.80799	6.027708	6.049074	5.989775	6.218007	6.130169	6.020091	5.75073	6.032513
Fabp7	0.728423	7.201556	1.964281	0.068924	0.1777	-5.440374	6.785733	7.087678	6.673796	6.927413	8.294923	8.280291	6.505846	7.758148	6.773317	7.411779	7.096382	7.913836	6.662411	6.661712

RGD1566380	0.590167	3.66296	1.964102	0.068947	0.17772	-5.222114	4.045673	4.502772	4.519244	3.1898	3.886882	3.839794	3.601415	3.791198	3.761508	3.669864	3.352837	2.89053	3.089114	3.270693
Cers2	0.476795	6.489028	1.963821	0.068982	0.177773	-5.431148	6.152662	6.656118	6.28194	5.589986	6.686771	5.758975	6.57675	6.769415	6.546392	6.89447	6.629136	7.06304	6.39131	6.867809
Ube2d1	-0.626683	5.257465	-1.963074	0.069076	0.17795	-5.424739	5.481971	4.452618	5.216372	6.431808	5.682458	5.96648	5.027123	5.059983	5.092089	4.899674	5.20815	5.138705	5.080417	4.909479
Gna11	-0.365957	5.55226	-1.962975	0.069089	0.17795	-5.413509	5.798336	4.902098	5.674127	5.5073	5.308931	5.240331	5.517835	5.630934	5.6061	5.606633	5.894392	5.702564	5.877239	5.498875
Caly	-0.475967	8.45559	-1.962926	0.069095	0.17795	-5.419629	8.525412	7.475343	7.981964	8.490922	8.654722	8.022183	8.31942	8.523136	8.844959	8.730797	8.570176	8.616034	8.717017	8.910838
Gtf3c4	-0.46209	4.445594	-1.962797	0.069111	0.177954	-5.340363	4.519693	4.180039	4.749408	5.013489	4.098216	4.196023	4.267989	4.600808	4.37654	4.07195	4.603708	4.365074	4.957391	4.311247
Htr3a	0.634132	2.618661	1.962196	0.069187	0.178111	-4.99181	2.645748	3.532115	3.247038	2.184345	2.936364	2.434761	2.450751	1.958936	2.653634	2.359011	2.274011	1.869448	3.03287	3.35002
Alg11	-0.3225	4.448975	-1.961247	0.069307	0.178382	-5.322682	4.377065	4.064569	4.397084	4.693241	3.998482	4.395516	4.618433	4.433629	4.656351	4.178226	4.564762	4.519683	4.806206	4.655275
Ctbp2	-0.328198	5.769618	-1.960534	0.069398	0.178576	-5.424604	5.687857	5.281173	5.280872	5.851575	5.83943	5.370341	6.183813	5.888621	6.107586	5.972933	5.704867	5.714258	5.827132	6.093717
Agpat5	-0.355166	5.63552	-1.960271	0.069431	0.178588	-5.426911	5.71628	4.996138	5.645251	5.927005	5.487291	5.728989	5.64395	5.476045	5.663659	5.521092	5.581226	5.814088	5.85308	5.875144
Aga	-0.30065	5.53253	-1.95995	0.069472	0.178588	-5.419036	5.686345	5.5207	5.115554	5.583041	5.312258	5.396521	5.592083	5.657738	5.525598	5.867131	5.510468	5.677395	5.292891	5.752039
Nipa2	0.311369	4.881756	1.959933	0.069474	0.178588	-5.349884	4.421353	4.57644	4.975957	4.706082	5.008197	4.93881	4.851979	4.899205	4.83703	4.879766	5.082283	5.19195	4.984828	5.044382
Rab40b	-0.800399	5.556086	-1.959916	0.069476	0.178588	-5.421104	5.507875	3.774588	5.411453	6.294015	5.128199	6.060017	5.708524	5.812285	5.788087	5.601432	5.899217	5.557551	5.715431	5.563542
Btbd9	-0.633949	5.517007	-1.959912	0.069476	0.178588	-5.435336	5.88127	4.613433	5.914769	6.441823	5.475469	6.088636	5.003787	5.473892	5.190313	5.235776	5.47536	5.389039	5.862523	5.227782
Daxx	0.312703	5.598933	1.95927	0.069558	0.178759	-5.421063	5.57149	5.98792	5.701315	5.373643	5.876985	5.571636	5.597939	5.561662	5.485533	5.901388	5.412631	5.581691	5.282878	5.511007
Tmem30a	-0.52998	7.245543	-1.958928	0.069602	0.178833	-5.450627	7.014973	6.125528	7.007279	7.695564	6.889133	7.275183	7.321367	7.226714	7.614435	6.967025	7.281913	7.453291	7.837508	7.738563
Ran	0.348126	6.8476	1.958159	0.069699	0.17904	-5.452492	6.857334	6.895191	7.24601	7.01825	7.574092	7.429702	6.587582	6.602074	6.741269	6.335418	6.794933	6.444529	6.798655	6.55527
Ctsd	0.485057	9.209885	1.958058	0.069712	0.17904	-5.417589	8.946569	8.901738	8.798011	8.063543	9.352886	8.91808	8.659394	10.01315	8.788841	10.04997	9.955062	10.70403	8.738058	9.05201
Epc2	-0.63912	4.064775	-1.957467	0.069787	0.179157	-5.366678	4.53321	3.479652	4.521788	5.501419	4.355276	5.149073	3.720717	3.690957	3.682701	3.440594	3.771221	3.744381	3.754753	3.661237
Tubgcp5	-0.293094	5.227763	-1.957467	0.069787	0.179157	-5.395068	5.080769	5.148837	4.762375	5.341085	4.95385	4.830014	5.780665	5.243062	5.499012	5.294957	5.285734	5.112433	5.370586	5.528164
Fam173b	-0.35883	4.332873	-1.956917	0.069858	0.179299	-5.329638	4.272147	4.459406	3.887149	4.812037	4.299374	4.149692	4.371894	4.472969	4.355524	4.406073	4.411103	4.074306	4.416965	4.350357
Gas6	-0.453589	7.751508	-1.956609	0.069897	0.179343	-5.448307	8.280938	7.158745	7.211757	7.202617	7.617433	7.175984	7.736317	7.938769	7.847922	8.048327	8.371416	8.152528	7.803242	7.982748
LOC500124	-0.595732	2.581883	-1.956547	0.069905	0.179343	-5.013846	2.718545	2.963229	2.626517	2.985885	2.046579	1.496914	3.01488	2.753156	2.694377	2.758411	2.707156	2.433555	2.676906	2.544088
St3gal3	-0.290293	4.819227	-1.956291	0.069937	0.179389	-5.356931	4.82184	4.361326	4.509023	4.693241	4.630473	4.361521	4.736391	4.943446	5.048229	4.913728	5.11997	5.143038	5.154774	5.088999
Camk2d	0.570369	4.910099	1.955043	0.070097	0.179721	-5.411232	5.610268	6.03299	6.120113	4.669835	5.256357	5.396521	4.150713	4.145178	4.473365	4.250879	4.857801	4.735888	4.646703	4.450943
Ttll1	-0.284618	5.229798	-1.955033	0.070098	0.179721	-5.415042	5.32763	4.889586	5.028784	5.540658	5.364471	5.319734	5.261619	5.265641	5.216192	5.307785	5.29125	5.103568	5.221296	5.121014
Gli1	1.216995	2.276561	1.954928	0.070112	0.179721	-5.05319	3.522857	5.075612	3.179711	1.548999	2.640673	3.353184	2.361772	-0.150641	2.066858	1.14286	1.456531	0.151783	2.701169	2.721621
Rpp38	0.405876	2.712595	1.95478	0.07013	0.179731	-4.999203	2.542593	2.643736	3.049621	2.363612	2.962008	2.714852	2.787802	2.951874	2.720913	2.681644	3.048553	2.703718	2.689088	2.357047
Cgnl1	-0.612263	5.643586	-1.954179	0.070207	0.179879	-5.446094	6.913971	5.731683	5.304705	5.464109	5.501934	5.764899	5.722886	4.983347	5.82983	5.655958	5.816195	5.24934	5.372476	5.731448
Nmt2	-0.385031	5.367774	-1.954096	0.070218	0.179879	-5.426566	5.237611	5.028455	5.244369	6.020439	5.242473	5.476663	5.49291	5.385024	5.422258	5.054841	5.362854	5.342704	5.640562	5.236159
Ogt	-0.319289	8.375817	-1.953179	0.070336	0.180141	-5.437124	8.170667	8.226718	7.791909	8.651392	8.106814	8.231505	8.895238	8.268437	8.679356	8.364013	8.312505	8.293909	8.506411	8.767402
Prkcz	-0.214575	7.616302	-1.952955	0.070364	0.180145	-5.450613	7.873387	7.695459	7.576638	7.820095	7.557717	7.698978	7.529226	7.543445	7.444219	7.620685	7.614507	7.524761	7.6473	7.489823
Gen1	0.836956	0.910912	1.952935	0.070367	0.180145	-4.582423	0.369528	1.425638	0.484899	0.577356	1.85913	1.450626	1.297885	1.112357	1.255413	0.893396	0.721218	0.514819	1.150071	0.499386
Cnih3	-0.525471	3.077068	-1.952603	0.07041	0.180215	-5.126063	3.016775	2.354284	2.901201	3.665254	3.238702	2.809587	2.659281	2.862532	3.525993	3.158464	3.258364	3.153617	3.262178	3.403062
Gpr45	-0.349188	2.805866	-1.952215	0.070459	0.180305	-5.014555	2.645748	2.309899	2.47722	2.825673	2.436306	2.349037	2.967416	2.753156	3.216682	3.070874	3.022257	3.118845	3.212403	3.106782
Prkcg	-0.469365	9.991746	-1.951979	0.07049	0.180344	-5.375236	10.22732	9.101162	9.904103	10.34771	10.09462	10.18273	10.03735	10.03248	10.24012	9.821291	10.11467	9.72337	10.10175	9.957344
Atp5s	-0.373793	3.404888	-1.951576	0.070542	0.180438	-5.168169	3.562947	2.679063	3.098159	3.425447	3.238702	3.432728	3.951676	3.321106	3.811751	3.249928	3.373787	3.405197	3.608485	3.661237
Snap23	0.304349	4.327993	1.951443	0.070559	0.180443	-5.344843	4.373312	4.774532	4.687307	4.412169	4.548211	4.779416	4.3855	4.055878	4.023757	4.015725	3.862983	4.136468	4.070962	4.544972
Pdk2	-0.299003	8.018237	-1.951095	0.070603	0.18052	-5.437139	8.54264	8.05499	8.316833	8.498693	8.393432	8.124776	7.575961	7.787917	7.779351	7.687647	8.060464	7.717926	7.998209	7.72263
Acss1	-0.460225	6.071392	-1.950716	0.070652	0.180601	-5.450427	6.383796	6.205438	5.227324	5.752308	5.627431	5.38426	6.322308	6.461371	5.973314	6.467998	6.312117	6.674427	6.017894	6.214158
Sfrp2	-1.114254	2.521026	-1.950615	0.070665	0.180601	-4.905896	2.765113	0.854759	1.169557	2.337441	1.840817	1.910004	4.864988	2.80888	2.834633	3.100666	2.303426	3.028062	2.860463	2.952659
Hba2	0.766684	5.876308	1.950444	0.070687	0.180619	-5.462824	7.019182	7.410699	6.84254	5.973416	8.053391	6.732414	4.842144	5.048455	5.870398	5.067468	5.188577	4.506292	4.477919	5.269187
Chgb	-0.543203	10.50209	-1.949953	0.070751	0.180714	-5.362746	10.38816	9.444553	10.30688	11.0769	10.32252	10.68466	10.32106	10.77316	10.75357	10.66749	10.42529	10.54446	10.60169	10.72
Gng8	-0.774595	2.048151	-1.949922	0.070755	0.180714	-4.859172	2.460078	2.21681	1.089503	1.892408	1.745593	0.695385	2.566765	2.054242	2.204856	2.543858	1.959843	2.816513	2.022549	2.825198
Pole4	0.310766	4.529991	1.949468	0.070813	0.180821	-5.356956	4.602167	4.601207	4.608118	4.33821	5.058591	4.836779	4.407896	4.161323	4.57868	4.483493	4.470659	4.323687	4.56643	4.450943
Pbx2	-0.223774	6.796089	-1.94936	0.070827	0.180821	-5.468188	6.878669	6.58029	6.821528	7.005321	6.721593	6.749841	6.915268	6.718494	6.774109	6.864395	6.689565	6.723712	6.796545	6.920086
Nsdhl	-0.381062	5.064948	-1.949248	0.070842	0.180821	-5.414916	5.237611	4.496185	5.016208	5.425809	5.290494	4.994318	4.946803	4.840254	5.434336	4.803319	4.955998	4.916302	5.485056	5.114213
Sf3a2	0.305176	5.865169	1.948848	0.070893	0.180834	-5.453005	5.796936	6.306788	6.291662	5.824744	6.06917	5.79647	5.574371	5.901494	5.709103	5.992929	5.891974	5.704031	5.462168	5.817698
Mbd6	0.474016	7.686768	1.948835	0.070895	0.180834	-5.461012	7.424742	8.317556	8.129581	7.412251	7.54009	7.580386	7.807536	7.652647	7.765879	7.910052	7.381384	7.652574	7.138334	7.910333
Cabp7	-0.500274	9.244954	-1.948757	0.070905	0.180834	-5.417385	9.412246	8.098313	8.669395	9.180271	9.095118	9.333637	9.754259	9.363295	9.28338	9.539814	9.303879	9.435419	9.521507	9.441546
Xiap	-0.583133	3.99721	-1.948644	0.07092	0.180834	-5.357514	4.421353	3.86939	4.669061	5.101649	3.523394	4.649696	3.857182	3.811801	3.907275	3.471326	3.493474	3.71558	4.200009	3.372991
Galm	-0.507918	4.415581	-1.948577	0.070928	0.180834	-5.352664	5.195727	3.780021	4.015447	4.357872	4.5954	4.639415	4.228269	4.313555	4.286137	4.449167	4.946707	4.568818	4.24196	4.275119
Csrnp1	0.574326	3.649223	1.948339	0.070959	0.180834	-5.224257	3.481621	3.854016	4.841896	3.486263	4.002607	3.479584	3.328389	3.550909	3.440756	3.636356	3.999546	3.60686	3.356854	3.151754
Hat1	0.435019	4.28909	1.94831	0.070963	0.180834	-5.355969	4.272147	4.343088	4.629506	4.555288	5.09188	5.329359	4.102096	4.155962	3.907275	3.90752	3.939921	4.023513	4.013992	3.858608
Ulk3	0.586	3.51115	1.948204	0.070977	0.180834	-5.138289	3.09177	4.324616	3.580509	2.951788	3.384903	3.165332	3.96382	3.534475	3.627666	3.747244	3.441501	3.461789	3.169571	3.853172
Lrrc8a	0.281065	6.855936	1.94816	0.070982	0.180834	-5.469401	7.005306	7.30866	7.295541	6.778529	7.062132	7.024738	6.629161	6.663821	6.546392	6.616302	6.597574	6.978894	6.797249	6.692476
Tbc1d19	-0.391394	4.204927	-1.947754	0.071035	0.18093	-5.338339	4.395683	3.613496	4.251403	4.624561	4.415294	4.181925	4.118484	3.979018	4.338488	3.924277	4.196624	4.092341	4.627841	4.195741
Brix1	0.31339	5.282019	1.947253	0.0711	0.181057	-5.441719	5.176474	5.613402	5.888397	5.802197	5.840583	5.880709	5.086642	4.934079	4.983948	4.982013	4.967529	4.968501	5.098798	4.766442
Cbr3	0.584301	1.782595	1.945875	0.071278	0.181473	-4.894628	2.196854	2.894758	2.487668	1.548999	2.411703	1.960428	1.576217	1.020936	1.29127	1.828755	1.605665	1.152831	1.770732	1.684306
Abhd13	-0.533315	5.271205	-1.945668	0.071305	0.181483	-5.425202	5.073848	4.159341	5.274852	5.830619	4.901799	5.332553	5.281162	5.321786	5.436339	5.176396	5.43606	5.504661	5.651498	5.458279
Ccdc53	0.342429	3.87391	1.945597	0.071315	0.181483	-5.298601	4.163349	4.073446	4.287732	3.761892	4.319352	4.492945	3.763028	3.509467	3.774234	3.683052	3.897149	3.727169	3.32598	3.56477
Wisp2	1.34539	0.731829	1.945496	0.071328	0.181483	-4.623309	1.831529	1.047298	2.162035	-0.705589	0.845972	2.863579	0.68572	0.76329	0.17081	0.475109	0.263333	0.618025	-0.123798	0.442608
Kdelc2	0.367137	5.311067	1.944485	0.071459	0.181752	-5.437193	5.302196	5.532015	5.289856	5.334484	5.848624	6.071533	5.032899	5.285418	4.939446	5.155258	5.037033	5.502977	5.033414	5.030001
Eps15	-0.342813	7.581586	-1.944405	0.07147	0.181752	-5.467058	7.629353	7.074473	7.320596	7.917439	7.65198	7.674957	7.724321	7.664495	7.551143	7.390833	7.61157	7.478367	7.896485	7.564483
Mybpc2	0.576995	1.969133	1.944335	0.071479	0.181752	-4.826808	1.80948	2.33964	2.09528	1.327578	1.685287	2.374054	2.186319	2.121805	1.914249	2.221154	2.317912	2.046399	1.377871	2.159814
Slc25a23	-0.421553	7.881577	-1.9441	0.071509	0.181792	-5.463975	8.280187	7.830542	7.900731	7.649109	7.151677	7.297013	8.163296	7.795265	7.950996	7.957293	7.880039	8.098338	8.256139	8.138327
Srcin1	-0.352906	7.702327	-1.94389	0.071537	0.181823	-5.464254	8.0029	7.892679	7.739047	7.890692	7.285492	7.45886	7.62983	7.777917	7.660145	7.497325	7.56489	7.711741	8.108336	7.620348
Tspan3	-0.303748	8.19205	-1.943683	0.071564	0.181851	-5.465727	8.067031	7.392191	7.571429	7.691696	7.813886	7.530496	8.511083	8.315787	8.598929	8.576583	8.538056	8.566896	8.625493	8.894889
Dyrk1a	-0.508333	6.231698	-1.943531	0.071584	0.181851	-5.473851	6.238712	5.647985	6.521957	7.006879	5.820863	6.467001	6.011712	6.344858	6.141132	5.938979	6.417604	6.24333	6.449759	6.014364
Vstm4	0.613845	2.580756	1.943454	0.071594	0.181851	-4.969379	2.765113	2.184396	2.908996	1.615848	2.919011	2.982217	2.987806	2.753156	2.330799	2.484835	2.580719	3.055902	2.614401	2.622863
Ankrd50	-0.409195	4.145664	-1.942981	0.071655	0.181945	-5.34089	4.110227	4.134105	4.360599	4.828572	3.761059	4.020978	3.987806	4.015001	4.091419	3.957219	4.086719	4.241218	4.477919	4.056051
Aurkaip1	0.397604	5.558521	1.942941	0.07166	0.181945	-5.450918	5.770073	6.211483	5.843343	5.263289	6.131705	5.459235	5.435203	5.359594	5.438339	5.432112	5.671415	5.290932	5.150375	5.396093
Tmem39b	0.365106	4.627287	1.942406	0.07173	0.182083	-5.338422	4.197708	4.69623	4.506456	4.210655	4.720868	4.358391	4.934514	4.60868	4.997579	4.957801	4.609607	4.631832	4.420622	4.995876
Pcp4l1	-0.422481	4.377031	-1.942224	0.071754	0.182105	-5.391485	5.254031	4.838423	4.521788	4.594489	4.525459	4.116722	4.187425	4.101219	4.263751	4.219542	4.655954	4.118979	3.964736	3.993192
Cbs	-0.560705	6.265506	-1.941197	0.071888	0.182408	-5.474625	6.894466	6.482867	5.658028	6.028641	6.248321	5.221502	6.43237	6.230909	6.364798	6.569134	6.257742	6.444529	6.40891	6.496338
Bmyc	-0.377053	4.777432	-1.940737	0.071949	0.182522	-5.387684	4.96336	4.180039	4.424534	4.72387	4.815341	4.431711	4.952909	4.843593	5.190313	4.916522	5.132316	4.647164	5.068809	4.652149
Patl1	0.258218	5.846915	1.940575	0.07197	0.182537	-5.467919	5.771499	6.155101	6.340035	5.953093	5.949284	6.038663	5.907112	5.717195	5.799026	5.669274	5.593185	5.559173	5.627961	5.803676
Adamts4	0.536164	5.485831	1.93875	0.072209	0.183106	-5.429996	5.059908	5.821369	5.733497	4.996864	5.701613	5.000356	5.259158	5.78302	5.304377	5.508182	5.117902	6.16134	5.578065	5.812105
Trpc4	-0.777506	5.632257	-1.938186	0.072284	0.183247	-5.433213	5.28032	3.27168	4.847991	5.738624	5.333698	5.438632	6.179914	6.16304	6.238477	5.940353	6.06081	5.951348	6.231823	6.214158
Ube2q2l	-0.334518	3.338008	-1.938094	0.072296	0.183247	-5.219212	3.543041	3.263946	3.184186	3.706736	3.238702	3.502454	3.419386	3.311493	3.408468	3.14899	3.603988	3.30822	3.245776	3.002614
Sez6	-0.282615	9.235712	-1.937943	0.072315	0.183259	-5.444342	9.009725	8.660901	8.627932	8.913534	8.826842	8.601502	9.850808	9.432281	9.794538	9.554913	9.343468	9.531396	9.356827	9.798056
Ncl	0.400741	7.514844	1.937784	0.072336	0.183273	-5.468555	7.590687	7.922771	8.484506	8.250463	8.331248	8.545999	6.726815	7.366739	7.020318	6.941872	7.140345	7.039412	7.061347	6.79455
Fnta	0.301566	5.999412	1.936583	0.072495	0.183636	-5.479346	6.136148	6.275394	6.044281	5.932479	6.73529	6.286074	5.787514	5.796866	5.781799	5.849693	5.811084	5.904905	5.816065	5.858963
Fmo3	-0.875584	-0.43971	-1.936173	0.072549	0.183734	-4.582527	1.594228	0.04804	0.642822	0.22441	-0.257548	-0.291112	-0.067151	-0.654381	-0.632503	-0.311823	-0.991788	-0.78301	-0.755361	-1.632384
Wdpcp	-0.630017	2.322591	-1.9359	0.072585	0.183766	-4.9832	2.917193	2.279532	1.195284	2.227735	2.28197	2.086865	2.415815	2.121805	2.694377	2.308845	3.013384	2.390473	2.494578	2.417223
Xpo5	0.209077	6.559375	1.935845	0.072592	0.183766	-5.484894	6.215844	6.520683	6.542774	6.344164	6.533434	6.359937	6.74628	6.601088	6.703511	6.646071	6.639265	6.604673	6.628021	6.762288
Ptchd1	-0.881245	1.156775	-1.935238	0.072672	0.18393	-4.747773	0.915118	-0.025882	1.220561	2.350586	0.606834	1.378286	0.743216	1.020936	1.843557	1.12717	0.924208	1.515634	1.574559	1.659607
Mknk2	0.286842	6.559065	1.934846	0.072724	0.184022	-5.487122	6.548434	6.656857	6.568841	6.052969	6.682278	6.437637	6.638661	6.518892	6.469252	6.746068	6.568338	6.783908	6.492092	6.679481
Pcdh11x	-1.003449	0.915329	-1.934733	0.072739	0.184022	-4.792371	1.874641	-0.465897	2.135702	1.92792	0.606834	1.275818	0.498056	0.818632	0.97585	-0.311823	0.202178	0.618025	1.770732	1.846283
Taok3	-0.372533	6.357866	-1.934227	0.072806	0.184152	-5.491502	6.363131	6.364891	6.509834	7.178403	6.121311	6.600181	6.291322	6.337772	6.385681	6.188254	6.211941	6.150622	6.262719	6.063439
Slc47a1	1.582339	-1.225347	1.934051	0.072829	0.184173	-4.408727	0.369528	-0.103798	1.169557	-1.575022	0.091273	1.766229	-0.529223	-0.81357	-1.813089	-1.838237	-3.260591	-1.925331	-1.638202	-1.632384
Cox18	0.416314	3.898864	1.933382	0.072918	0.184358	-5.204867	3.145563	4.010125	3.74196	3.41297	3.706477	3.359461	4.358158	3.93587	4.417676	4.386051	3.976904	3.941318	4.013992	4.287263
Kcnj10	-0.617598	5.223718	-1.933236	0.072937	0.184368	-5.464989	6.426071	5.585746	5.113869	5.192053	5.145088	4.915569	4.983054	4.768288	4.881816	4.643844	5.037033	5.392683	5.813285	5.277328
Zfp329	-0.390744	4.561637	-1.932975	0.072972	0.184417	-5.402551	4.519693	4.368558	4.284739	5.213795	4.390327	4.894111	4.767901	4.446862	4.541944	4.475935	4.527854	4.273193	4.763639	4.461699
Trpc6	-0.531886	5.510868	-1.932635	0.073017	0.184492	-5.457611	5.1416	4.525594	5.316475	6.098482	5.064521	5.45191	5.624957	5.763762	5.826779	5.846766	5.380213	5.877865	5.378131	5.891146
Kpnb1	0.395784	7.229137	1.932206	0.073074	0.184563	-5.491521	6.982701	7.381955	7.73768	7.534636	7.583072	7.748664	6.864351	7.211287	6.903546	6.715105	7.109452	7.057886	7.365959	6.856308
Mesdc2	-0.363239	6.809747	-1.932191	0.073076	0.184563	-5.496038	7.165775	6.311486	6.773767	7.101763	6.848464	7.148609	6.419201	6.721219	6.725793	6.824782	6.76485	6.844355	6.782401	6.918135
Chga	-0.350407	8.100543	-1.931604	0.073154	0.184721	-5.48321	7.913347	7.629646	7.468371	8.081393	7.858375	7.628934	8.620369	8.085941	8.482348	8.570608	7.975071	8.256659	8.160672	8.681833
Rab14	-0.608707	5.957258	-1.930545	0.073295	0.185036	-5.493247	6.173313	5.210498	6.014784	6.933431	5.881472	6.670023	5.59599	5.97641	5.86446	5.542957	6.028146	5.846329	6.17557	5.514455
Recql	0.249331	5.040269	1.93034	0.073322	0.185036	-5.430232	4.915133	5.127683	5.076287	4.932758	5.30058	5.188389	5.178086	5.019224	5.171196	5.059905	4.784073	4.898474	4.937104	5.022756
Selrc1	-0.389525	3.483311	-1.930321	0.073325	0.185036	-5.301775	3.92781	3.354134	3.772191	4.170451	3.470774	4.03673	3.585784	3.211662	3.341641	3.195748	3.50618	3.18757	3.237505	2.91141
Tmem33	-0.611964	5.105222	-1.929947	0.073374	0.185123	-5.459839	5.292294	4.069014	4.97038	5.948777	5.354824	5.633805	4.952909	4.83019	5.154258	4.620041	4.872595	4.983074	5.472777	5.365875
Pds5b	-0.475657	7.392446	-1.92958	0.073423	0.185208	-5.49325	7.325302	6.636034	7.387684	8.102186	7.305663	7.620465	7.57695	7.318961	7.39666	7.071638	7.445044	7.156543	7.72257	7.438117
Zbtb41	-0.603143	3.123427	-1.929458	0.07344	0.18521	-5.27147	3.439172	2.953644	3.830814	4.443178	2.892584	3.698398	2.905804	3.070611	2.785194	2.484835	3.048553	2.691938	3.270309	2.402413
Nagk	0.396682	4.621041	1.92887	0.073518	0.185369	-5.377223	4.611755	5.216521	4.736324	4.054744	4.436792	4.509973	4.798736	4.464319	4.872969	4.626882	4.411103	4.451452	4.722744	4.845219
Gla	-0.293054	5.348298	-1.928464	0.073572	0.185467	-5.452672	5.390247	4.719039	5.125624	5.316175	5.156239	5.228377	5.298047	5.418994	5.497094	5.539336	5.500978	5.465412	5.558277	5.701512
Lrp12	-0.633646	6.405416	-1.928103	0.073621	0.18555	-5.497542	6.051792	5.02617	6.280438	7.118189	5.953551	6.541352	6.617678	6.515755	6.721692	6.572681	6.370609	6.615626	6.616107	6.693997
Hpca	-0.802197	10.99278	-1.92726	0.073734	0.185762	-5.382837	11.51764	9.136273	10.70681	10.99722	11.09382	10.91635	11.33023	11.422	11.21001	11.09064	11.50689	10.96983	11.29852	10.70353
Map2k6	-0.496025	4.792715	-1.927239	0.073736	0.185762	-5.461209	5.087656	4.613433	4.860105	5.959542	4.993765	5.66074	4.652595	4.459975	4.703686	4.281549	4.480351	4.277141	4.772253	4.35421
Tomm34	-0.367882	6.949068	-1.927119	0.073752	0.185762	-5.504407	7.22163	6.321768	6.727681	6.925218	7.126858	6.646475	7.095785	7.079432	7.105236	6.862947	7.21238	6.902371	7.22194	6.850181
Nanp	-0.392522	3.170117	-1.927011	0.073767	0.185762	-5.257984	3.569521	2.934281	3.404211	3.950685	3.506066	3.632543	3.200804	2.901501	2.984524	2.911926	3.124682	2.960934	2.933966	2.544088
Pask	0.704615	4.326586	1.926736	0.073804	0.185765	-5.339882	3.788223	5.738676	4.569292	4.109897	4.023054	4.342635	4.53949	4.264575	4.632088	4.417954	4.061116	3.995035	4.028447	4.14326
Nlk	-0.39521	3.71788	-1.926729	0.073805	0.185765	-5.332686	3.963192	3.492948	3.93756	4.464495	3.780406	4.075375	3.593621	3.798099	3.592171	3.14899	3.639021	3.412394	3.855477	3.417866
Arl5c	0.942318	1.276752	1.926425	0.073845	0.185765	-4.55097	0.307952	1.679303	0.52603	-0.093872	1.48759	0.386712	2.247205	1.658838	1.636128	1.942797	2.031907	1.643459	1.316372	1.846283
Etfa	0.360848	5.186494	1.926389	0.07385	0.185765	-5.457539	5.619802	5.630045	5.510845	4.733936	5.622079	5.357855	5.035778	4.934079	4.983948	5.013676	5.052274	4.96361	4.977397	5.219356
H6pd	0.440287	3.711463	1.92632	0.07386	0.185765	-5.222463	3.42474	3.899656	4.226666	3.197095	3.26641	3.563537	3.734959	3.878428	3.767885	3.90752	3.78171	3.941318	3.850062	3.642453
LOC304396	0.429499	5.209991	1.926309	0.073861	0.185765	-5.414868	4.794038	5.44747	4.961037	4.473536	5.105367	4.734242	5.611511	5.456551	5.6132	5.575146	5.255952	5.253353	5.011753	5.690887
Bmpr2	-0.549158	6.191781	-1.926093	0.07389	0.185799	-5.499575	6.002839	5.590392	6.266095	6.938881	5.533634	6.298378	6.217169	6.462457	6.430586	5.766954	6.191437	6.204473	6.686157	6.117717
Lnp	-0.567086	4.658582	-1.925963	0.073907	0.185804	-5.390687	4.284188	3.317238	4.408127	5.158812	4.451953	4.434686	4.750481	4.965068	5.037713	4.531675	4.889666	4.81947	5.141536	5.09592
Ppp2r5e	-0.38789	6.763924	-1.924889	0.074052	0.186105	-5.507729	6.910739	6.347613	6.826681	7.456998	6.876828	7.13083	6.603194	6.642892	6.701014	6.417603	6.772758	6.521424	6.910185	6.590814
Pgrmc2	-0.396069	6.812116	-1.924842	0.074058	0.186105	-5.507849	6.913971	6.254078	6.858227	7.433254	6.877951	7.11793	6.537677	6.738361	6.810086	6.690828	6.752239	6.760835	6.955014	6.683315
Rbbp8	0.303209	4.740526	1.924457	0.07411	0.186194	-5.414273	4.646374	5.206468	4.97038	4.608236	4.914989	4.634248	4.84543	4.647404	4.867041	4.66726	4.664481	4.575244	4.671475	4.5074
LOC652955	-0.342937	5.543179	-1.924348	0.074124	0.186194	-5.475925	5.735409	5.131939	5.27937	5.51899	5.554855	5.165286	5.276301	5.65013	5.304377	5.854072	5.765573	6.159203	5.763748	5.479602
Rnf146	-0.421938	5.386166	-1.924004	0.074171	0.186272	-5.475346	5.339219	4.7275	5.217941	5.935753	5.408657	5.512311	5.484505	5.51426	5.410079	5.162339	5.594673	5.288978	5.637422	5.210881
Pcdhga10	-0.542548	4.185241	-1.923297	0.074266	0.186413	-5.36852	4.110227	3.404252	4.257522	4.874798	3.706477	4.431711	3.876582	4.451246	4.421726	4.010503	4.017407	4.170823	4.579577	4.369521
Cmc1	0.688413	1.257386	1.923251	0.074272	0.186413	-4.751009	1.430522	1.874793	1.996308	0.705659	1.822268	1.165512	1.258992	1.393205	0.736738	1.540753	1.430105	0.714338	0.791767	1.415936

Arg2	-0.450841	3.276521	-1.923144	0.074287	0.186413	-5.191322	3.502386	2.690649	3.215954	3.309066	3.26641	2.613455	3.691803	3.340143	3.448717	2.998871	3.728487	3.323571	3.621257	3.286911
Ilfg3	0.587019	5.285498	1.923047	0.0743	0.186413	-5.427086	5.284322	5.759454	5.026994	4.062753	5.437888	4.772376	5.224248	5.469576	5.334762	5.689019	5.579724	5.835662	5.004459	5.558541
Dclre1c	0.435213	3.922535	1.923011	0.074304	0.186413	-5.202106	3.205888	4.023929	3.595078	3.145235	3.416161	3.420773	4.502506	4.318363	4.18493	4.531675	4.200538	4.40891	3.833693	4.238063
Ttc30a1	-1.064284	0.757484	-1.922743	0.074341	0.186418	-4.713482	1.071521	-1.463779	0.975347	1.892408	1.20216	1.165512	0.743216	0.818632	0.884903	0.591587	0.805871	0.714338	1.218908	1.085968
Lin7b	-0.479889	5.241592	-1.922657	0.074352	0.186418	-5.439192	5.425104	5.206468	4.413617	4.819147	4.641095	4.323498	5.976183	5.473892	5.712413	5.738812	5.483415	5.261346	5.310741	5.641289
Nmrk1	-0.342968	3.076082	-1.92265	0.074353	0.186418	-5.143919	3.136736	2.631765	2.718102	3.114742	2.692656	3.091837	3.554005	2.926907	3.375441	3.258764	3.057213	3.10114	3.237505	3.357718
Stag1	0.293265	4.844853	1.922191	0.074415	0.186535	-5.433543	4.576285	4.835816	5.271833	5.013489	5.150675	5.09931	4.893837	4.677653	4.700356	4.754179	4.644506	4.816761	4.713828	4.734278
Cul4b	-0.311839	6.059892	-1.921441	0.074516	0.186732	-5.512057	6.658143	6.150913	6.534609	6.770604	6.260428	6.663697	5.870489	5.579795	5.655084	5.489538	5.961666	5.591235	5.878569	5.798029
Lrrc48	-0.264107	4.216146	-1.921378	0.074525	0.186732	-5.389735	4.519693	4.244344	4.363439	4.541025	4.082714	4.373976	4.489964	4.117861	4.189688	3.946322	3.944596	3.906064	4.182881	4.208569
Mllt11	-0.529342	8.014624	-1.921	0.074576	0.186821	-5.483918	8.355115	7.479106	8.2891	8.986923	8.219852	8.578649	7.847411	7.807713	7.901105	7.511718	8.061003	7.505962	8.128045	7.539318
Wdr92	-0.310861	4.252573	-1.920843	0.074597	0.186836	-5.368664	4.119218	3.843675	4.068613	4.621853	4.090486	4.257812	4.380979	4.193082	4.425765	4.433645	4.373459	4.208518	4.28674	4.315206
Rasgef1c	0.775395	2.135306	1.919507	0.074778	0.18725	-4.975004	2.706665	3.411272	3.042552	1.300739	2.399242	2.086865	1.938115	1.775313	1.867508	2.359011	1.94125	2.187314	1.54805	1.708589
Edn1	0.866732	2.360436	1.919278	0.074809	0.187289	-4.933715	2.263247	2.631765	1.936547	1.327578	3.273255	2.695134	3.180134	1.71825	3.341641	2.499819	1.844511	2.027776	2.133689	2.503027
Cpe	-0.288055	11.54782	-1.919022	0.074844	0.187336	-5.3877	11.46033	10.90485	11.06705	11.16742	11.05364	11.08552	11.97404	11.63142	12.02003	11.86371	11.56585	11.82012	11.83035	12.22575
Slc29a1	0.550073	3.887792	1.918903	0.07486	0.187338	-5.25959	3.522857	4.563896	3.875261	3.309066	4.078813	3.359461	3.975863	3.891392	3.799353	4.285878	3.858035	4.220868	3.589113	4.208569
Sema6b	-0.237832	7.747704	-1.917142	0.0751	0.18776	-5.517778	7.444549	7.196821	7.214905	7.357144	7.143296	7.09836	8.236134	8.014416	8.254116	8.240662	7.898228	8.084607	8.013125	8.279257
Pyroxd2	-0.609721	4.564988	-1.917078	0.075108	0.18776	-5.376296	4.940716	4.286948	3.253175	4.058754	4.567829	3.479584	4.746971	4.989389	4.771898	5.451509	5.174716	5.058407	4.505736	4.698352
Lonp1	0.246944	7.066158	1.917033	0.075115	0.18776	-5.519884	6.903601	7.283536	7.261696	7.09787	7.226762	7.218041	7.089564	6.884111	7.084949	7.049532	6.854523	6.942694	6.806365	7.234728
Ajap1	-0.480979	5.76458	-1.916972	0.075123	0.18776	-5.475364	5.441326	4.146778	5.282373	5.5248	5.397737	5.156301	6.507921	6.050519	6.182295	6.077758	6.042391	6.119079	6.340082	6.467279
Aamdc	-0.525707	1.868091	-1.91689	0.075134	0.18776	-4.928115	2.387517	2.046927	1.089503	1.910273	1.997824	1.450626	1.913281	1.983359	1.891068	2.202958	1.922414	2.046399	1.700256	2.050068
Elk4	0.24226	6.134247	1.916874	0.075136	0.18776	-5.509081	5.788507	6.196323	6.135145	6.112053	6.063258	6.379372	6.218437	6.178988	6.113858	6.178975	6.084302	6.194071	6.16143	6.09717
Slc16a14	-0.849777	4.382069	-1.916857	0.075138	0.18776	-5.354683	4.247758	2.028784	4.413617	4.682885	3.736505	4.17126	4.407896	4.983347	4.950701	4.502215	4.87014	4.741613	5.125938	4.578298
Hecw2	-0.567861	4.713423	-1.916526	0.075184	0.1878	-5.452781	4.881181	5.110534	4.899765	5.51899	3.780406	4.774727	4.877881	4.778791	4.71693	4.116377	4.477127	4.492777	5.033914	4.591416
Wdr20	-0.229906	4.726341	-1.916511	0.075186	0.1878	-5.442506	4.920286	4.735912	4.731936	4.980046	4.669909	4.748661	4.678618	4.620407	4.65291	4.5783	4.852835	4.659314	4.797792	4.601177
Camkv	-0.295648	9.20395	-1.915871	0.075273	0.187886	-5.476333	9.210789	8.56816	8.829542	8.9511	9.129708	8.620899	9.552684	9.351616	9.579605	9.377159	9.427275	9.20463	9.485399	9.56949
Rilpl2	-0.331823	4.525483	-1.915656	0.075302	0.187886	-5.406163	4.683216	3.986819	4.099597	4.549599	4.436792	4.605489	4.587372	4.628172	4.60741	4.800297	4.850346	4.778284	4.549825	4.262873
Atp6v0d1	-0.287048	8.845154	-1.915632	0.075305	0.187886	-5.481542	9.02251	8.374561	8.643493	8.853193	8.933713	8.657497	8.924465	8.906092	8.936433	8.877476	9.088577	8.805779	9.008739	8.803079
Map2k2	0.420773	6.600104	1.915613	0.075308	0.187886	-5.516952	6.619855	6.853279	6.499545	5.739873	6.828853	6.212565	6.799764	6.527221	6.83687	6.83294	6.741533	6.621848	6.614511	6.689429
Irf5	0.403506	4.616267	1.915527	0.07532	0.187886	-5.400005	4.598957	4.462789	4.968516	4.086518	4.981279	4.521215	4.213088	5.07142	3.87219	5.140992	5.032648	5.549414	4.066299	4.129836
LOC100911827	0.576967	2.542516	1.915476	0.075327	0.187886	-4.999486	2.488111	2.690649	2.626517	1.760745	2.399242	2.974059	2.787802	2.80888	2.759824	2.681644	2.843564	1.989793	2.269694	2.791492
Shisa8	-0.769294	1.835775	-1.915456	0.07533	0.187886	-4.838007	1.693879	1.480065	1.089503	1.9796	1.664612	0.231108	1.478026	2.326577	1.867508	2.807409	2.083688	2.859272	2.408783	2.228598
Fbxo17	0.907764	0.471242	1.91508	0.075381	0.187917	-4.552986	0.105685	1.310208	0.883381	-0.02435	0.649545	0.772673	1.21902	0.517885	0.449965	-0.537962	0.924208	0.460309	-0.040977	1.085968
Trmt61a	0.411122	4.259133	1.915068	0.075383	0.187917	-5.36996	4.128153	4.155166	4.817254	4.066741	4.733331	4.294309	4.017238	4.015001	4.180157	4.005262	4.630068	4.140807	4.318455	4.208569
Adrbk1	-0.214853	7.975018	-1.915022	0.075389	0.187917	-5.503598	8.291913	8.051343	8.07216	8.158627	7.891282	8.029091	7.866538	7.825975	7.86624	7.877438	7.80811	7.933979	8.009487	7.974323
Pcdha11	-1.872583	0.147849	-1.914191	0.075502	0.188161	-4.412202	-0.310971	-3.260591	-1.909221	0.823477	-0.257548	1.193889	1.000645	0.972957	1.663747	0.591587	0.998029	1.082389	1.675978	1.193114
Ccdc176	-0.342856	3.573625	-1.914055	0.075521	0.188169	-5.282766	3.608352	3.713429	3.313151	3.906865	3.280067	3.414757	3.677127	3.727562	3.613573	3.867646	3.58015	3.300483	3.576053	3.584586
Kcnb2	-0.720602	2.773075	-1.913522	0.075594	0.188285	-5.141678	2.555898	2.279532	2.999392	3.805502	1.643636	3.006419	2.582602	3.000548	2.917273	2.423294	2.912	2.970717	3.143247	2.825198
Fmc1	-0.526078	2.806345	-1.913485	0.075599	0.188285	-5.10448	3.410162	1.972946	2.345554	2.475838	2.60856	2.644629	2.967416	2.571773	3.038267	3.00938	3.302737	2.9008	3.061266	3.212449
Acot7	-0.354747	7.442936	-1.913332	0.07562	0.188292	-5.521799	7.611544	6.923699	7.145666	7.520893	7.716492	7.062259	7.487249	7.413547	7.751868	7.419187	7.67202	7.314215	7.638689	7.532958
Th	1.097435	0.845996	1.913192	0.075639	0.188292	-4.670914	0.592497	2.493084	2.200654	0.485009	1.258434	0.481842	1.047044	0.377517	0.628769	0.799637	-0.318799	0.804622	0.649393	1.29285
Rb1cc1	-0.36423	6.87045	-1.913122	0.075649	0.188292	-5.524494	7.002272	6.923085	6.970038	7.835078	6.955531	7.363712	6.723247	6.554985	6.654451	6.521289	6.570608	6.506369	6.919263	6.700065
Hddc3	-0.385771	4.490116	-1.911328	0.075895	0.188865	-5.441719	5.18292	4.758107	4.072089	4.632654	4.803569	4.46411	4.192594	4.229271	4.384862	4.341	4.49954	4.53295	4.424271	4.414502
Ssx2ip	-0.411731	6.186295	-1.911177	0.075916	0.188878	-5.519461	6.025731	5.288816	5.998437	6.382265	5.714243	6.163187	6.40591	6.673235	6.365849	6.285492	6.584154	6.191982	6.419917	6.131254
Zwint	-0.441754	11.60486	-1.909988	0.076079	0.189246	-5.372668	12.07691	11.09068	11.84307	12.14377	12.0258	11.72467	11.41134	11.48151	11.61142	11.2259	11.64955	11.18599	11.71715	11.28073
Msantd4	-0.428145	5.561881	-1.909566	0.076138	0.189351	-5.519664	5.841078	5.11484	5.687785	6.404494	5.908101	6.065786	5.422059	5.245588	5.275577	5.257979	5.457481	5.287022	5.659259	5.273263
Gltp	0.40462	6.302948	1.909424	0.076157	0.189361	-5.525491	6.411507	6.645739	6.126813	5.797405	6.846744	6.410689	5.935138	6.442777	5.970552	6.457483	6.223529	6.816823	6.040653	6.135738
Rimbp2	-0.423611	8.947914	-1.909198	0.076188	0.189399	-5.493286	8.656446	8.821346	8.641301	9.231241	7.990664	8.617303	9.392893	9.211333	9.436958	9.138933	9.003061	8.928817	8.950629	9.253194
Mfge8	-0.603721	7.382849	-1.909006	0.076215	0.189426	-5.53073	8.148881	7.337966	6.766814	6.822826	7.116428	6.319459	7.557548	7.08014	7.772629	7.503843	7.535365	7.573325	7.786331	8.048434
Rps21	0.381496	2.929793	1.908833	0.076239	0.189446	-5.196737	3.100876	3.719097	3.393138	2.994284	3.372207	3.235263	2.8014	2.505864	3.140097	2.758411	2.580719	2.529342	2.326894	2.733504
Ppm1e	-0.963404	7.405	-1.908117	0.076338	0.189652	-5.525705	7.400978	5.610355	7.362003	8.485719	7.039728	7.901821	7.528715	7.811128	7.441229	6.78405	7.79878	7.277246	8.103508	7.135227
Uhmk1	-0.665965	3.603892	-1.907621	0.076406	0.189783	-5.316272	3.799454	3.176008	3.656563	4.249769	2.865664	3.747086	3.727855	3.550909	3.641623	2.733273	3.93523	3.33876	4.692807	3.475607
RGD1559442	0.360097	6.638131	1.907165	0.075469	0.189868	-5.531471	6.329633	7.011197	6.621384	6.154831	6.263009	6.512774	7.055214	6.739258	6.854954	6.78787	6.659311	6.648758	6.462235	6.849499
Fam46a	-1.036998	1.497864	-1.907043	0.076486	0.189868	-4.934047	3.388015	-0.025882	1.936547	1.353927	1.415175	1.72793	0.952703	2.268138	0.091681	1.964563	2.616315	2.419337	1.218908	0.321874
Cep63	0.288885	4.663419	1.907033	0.076487	0.189868	-5.426462	4.589284	4.989118	4.652905	4.367604	4.730847	4.692588	4.835551	4.778791	4.635579	4.763521	4.681385	4.482556	4.467348	4.683115
Tmem72	-5.684563	-1.264551	-1.906528	0.076557	0.190002	-4.44576	6.043551	-1.463779	-2.147119	-0.093872	-3.260591	-1.26448	-2.558588	-3.260591	-2.155202	2.956054	4.768337	-2.668207	-1.638202	-2.228037
Fez2	0.324344	4.779526	1.905624	0.076683	0.190274	-5.48095	5.119368	5.044346	5.221076	4.960888	5.588172	5.590436	4.34429	4.632039	4.250151	4.250879	4.612548	4.444449	4.533027	4.380898
Lrrc8b	-0.434233	5.332506	-1.905252	0.076734	0.190362	-5.491289	5.189338	5.012387	5.36687	5.644369	4.424547	5.134507	5.428646	5.733485	5.56641	5.183373	5.464008	5.496219	5.706469	5.344726
Scrn3	-0.49727	3.854062	-1.90483	0.076793	0.190446	-5.377279	3.978094	3.34683	3.986269	4.656666	3.61245	4.342635	3.824257	3.784265	3.878097	3.424979	4.030659	3.433771	4.107729	3.661237
Krt28	1.300834	-0.111878	1.904781	0.0768	0.190446	-4.514328	-0.217457	2.466372	1.089503	-0.411255	0.027932	0.175273	0.357848	-0.992527	-0.080923	-0.210791	-1.310338	-0.334482	-0.211665	-0.03329
Fance	0.799759	5.172966	1.904467	0.076843	0.190515	-5.426504	4.730919	6.072271	5.597812	3.937678	4.587184	4.240912	5.570406	5.571764	5.416182	5.682467	5.369823	5.316091	4.903517	5.472529
Tomm70a	-0.763631	4.79206	-1.90255	0.07711	0.191107	-5.474694	4.782765	3.390109	4.915329	5.784144	4.508156	5.24203	4.750481	5.033913	4.746291	4.491011	5.069498	4.712757	5.308768	4.414502
Slc4a1	1.488659	-0.879953	1.902524	0.077114	0.191107	-4.434446	-1.052261	1.58312	-0.067513	-0.502853	0.210178	0.809818	-1.450335	-0.26106	-0.27702	-0.027758	-1.310338	0.007173	-3.260591	-2.228037
Zbtb24	-0.357626	4.240869	-1.902175	0.077163	0.191153	-5.410641	4.167689	3.899656	4.229782	4.790495	3.994346	4.383247	4.223227	4.379449	4.189688	4.394093	4.334806	4.028205	4.334055	4.107181
Cd99	0.543186	5.282062	1.902161	0.077164	0.191153	-5.467313	5.026846	5.951064	5.128966	4.473536	5.388311	4.697551	5.43302	5.441205	5.416182	5.814171	5.200353	5.653298	5.033414	5.333059
Ssbp4	0.343735	7.972662	1.901844	0.077209	0.191218	-5.536058	7.658555	8.141166	7.902685	7.440957	7.772517	7.756889	8.350682	7.988633	8.265991	8.337673	7.980772	7.984347	7.743165	8.299632
Vegfa	-0.371097	7.76793	-1.901745	0.077222	0.191218	-5.53113	8.122297	7.981726	7.763437	7.936362	7.512154	7.376148	8.240821	7.258956	8.254968	7.624108	7.569059	7.29	7.696783	8.131608
Pggt1b	-0.385439	4.613809	-1.901385	0.077273	0.19128	-5.428805	4.373312	4.099756	4.047581	4.883868	4.234219	4.621246	5.038651	4.806432	5.061268	4.910928	4.434557	4.575244	4.686744	4.885716
Lyst	-0.679018	6.908512	-1.901281	0.077287	0.19128	-5.544646	6.293354	5.871858	6.783871	7.520893	5.52649	6.728176	7.671152	7.38862	7.516533	7.169176	7.088483	6.829647	7.239131	7.106388
Cdh6	-0.590753	3.232424	-1.901226	0.077295	0.19128	-5.277989	3.388015	3.167741	3.535898	4.04669	2.348294	3.49677	3.221182	3.42283	3.09008	2.641672	3.173291	3.509549	3.45286	2.932182
Zfp292	-0.394693	5.745979	-1.901049	0.07732	0.191302	-5.529967	5.568211	5.527177	5.821826	6.421723	5.283731	5.776673	6.118774	5.687772	5.753159	5.637447	5.699345	5.59282	5.744907	5.839853
Ankrd39	0.660774	1.188883	1.900521	0.077394	0.191445	-4.776252	1.143792	1.991799	1.613009	0.896996	1.43972	1.606524	1.000645	1.393205	1.579251	1.481601	0.378374	1.284167	0.698429	0.844371
Ctnnd1	0.227716	6.725787	1.900166	0.077443	0.191528	-5.547724	6.748555	7.104558	6.955527	6.778529	6.888019	7.016321	6.511028	6.580224	6.547319	6.562013	6.436812	6.776258	6.613712	6.65704
Akr1c19	-0.856136	2.659768	-1.899808	0.077493	0.191599	-5.005595	2.987615	2.769246	0.851376	2.013053	1.840817	1.275818	3.471351	3.201289	2.94004	3.574008	3.196995	3.496064	2.480628	3.447025
Ino80	0.330767	5.919114	1.899734	0.077504	0.191599	-5.530358	5.649584	6.435451	6.201708	5.821207	5.825528	5.806842	6.101015	5.86417	5.945455	5.975615	5.814919	5.862183	5.688377	5.901716
Ywhab	-0.34817	9.999777	-1.899286	0.077566	0.191715	-5.456519	10.19487	9.570609	10.00637	10.54138	10.32581	10.17752	9.968017	9.822942	9.948348	9.766782	9.971619	9.741073	10.05691	9.906178
Taok2	-0.228299	7.504998	-1.898813	0.077633	0.191839	-5.5436	7.59109	7.445308	7.528747	7.65077	7.154461	7.442693	7.582867	7.542418	7.510366	7.49919	7.485708	7.56002	7.605732	7.479276
Psmc3	0.225538	7.447166	1.898068	0.077737	0.192058	-5.54182	7.538024	7.690058	7.833461	7.682954	7.878994	7.941325	7.143945	7.280058	7.308642	7.238929	7.30482	7.10028	7.138334	7.189614
Pdzrn4	-0.503051	2.022934	-1.897908	0.07776	0.192074	-5.026763	2.072763	2.117298	2.262807	2.806722	1.664612	1.803538	1.913281	1.983359	2.066858	1.920699	1.978199	1.890216	2.522078	1.708589
Fbxo7	0.238754	5.977261	1.896997	0.077888	0.192331	-5.541357	5.903558	6.206447	6.430142	6.003895	6.04037	6.09241	5.785805	5.965724	5.837429	6.026944	5.843986	5.864809	5.834006	5.871116
Hmg20a	-0.396834	6.698666	-1.89687	0.077906	0.192331	-5.553448	6.837042	6.066725	6.560823	7.143427	6.854181	6.890851	6.491238	6.796373	6.515461	6.581512	7.018149	6.638055	6.82925	6.573565
Tmem170b	-0.756917	1.756749	-1.896827	0.077912	0.192331	-4.905977	1.371547	1.184734	1.574317	2.62393	1.622351	0.772673	2.05625	2.121805	2.185932	0.981432	2.133674	1.989793	2.480628	1.991897
Cmbl	-0.361185	3.774893	-1.896318	0.077984	0.192468	-5.392749	4.478365	3.996186	3.545931	4.074684	3.89578	4.227248	3.790562	3.321106	3.59934	3.595091	3.387587	3.744381	3.522595	3.786295
Maml3	-0.512557	2.542249	-1.896136	0.078009	0.192492	-5.157743	2.906846	1.914859	2.947351	3.197095	2.411703	2.854719	1.938115	2.76729	2.166756	2.342482	2.940364	2.8487	2.601568	2.030937
Msx1	-1.255027	3.69505	-1.89602	0.078025	0.192493	-5.380366	6.202158	2.607522	3.453014	3.067759	3.751288	3.508115	3.180134	3.591194	2.882434	4.076955	5.28389	3.60686	3.286436	3.372991
Tcp11l2	-0.301739	4.661478	-1.8955	0.078099	0.192578	-5.433161	4.509471	4.028502	4.397084	4.605497	4.067045	4.521215	4.736391	5.059983	4.867041	4.891176	4.79447	4.938905	4.885102	5.022756
Clec9a	-0.975637	0.414629	-1.895435	0.078108	0.192578	-4.596377	-0.129637	-1.271663	0.307539	1.502648	0.371962	-0.214525	0.625837	0.871929	0.736738	1.253084	0.676952	1.152831	0.745852	0.499386
Plekhh1	-0.713438	7.032648	-1.895434	0.078108	0.192578	-5.553192	6.740566	6.966622	5.323783	6.895256	6.662863	5.480988	7.644051	7.522244	7.617086	7.601712	6.941873	7.742761	7.399709	7.932087
Yod1	-0.621445	2.081995	-1.895315	0.078125	0.19258	-5.097378	2.295333	1.953842	2.379614	3.288723	1.685287	2.753495	2.033384	1.983359	1.914249	1.451089	2.259075	1.618784	2.333172	1.582867
Npepps	-0.257571	8.184797	-1.895057	0.078161	0.19258	-5.529696	8.305516	8.180473	8.011001	8.620186	8.207399	8.416904	8.258798	8.035372	8.126254	7.962372	8.159801	7.997566	8.181302	8.129642
Cbx8	-0.334777	3.110673	-1.895043	0.078163	0.19258	-5.219943	3.303772	2.780133	2.837276	3.002635	2.838232	2.800388	3.36548	3.349568	3.289407	3.552611	3.220315	3.390696	3.061266	2.942457
Kif26b	-0.565389	3.63642	-1.894973	0.078173	0.19258	-5.246268	2.885927	2.982209	3.104962	4.082584	2.586747	3.114281	4.134688	4.351577	4.096495	3.820684	3.911548	3.767014	4.383621	3.825682
Pcmt1	-0.391541	7.271956	-1.89446	0.078246	0.192719	-5.554445	7.388483	6.500254	7.229764	7.4956	7.331715	7.142252	7.213617	7.277592	7.437234	7.070384	7.515471	7.256401	7.659347	7.29961
Tmem200a	-0.396727	4.937613	-1.894079	0.078299	0.192809	-5.468441	4.671039	3.982112	4.678213	5.132418	4.516833	4.836779	5.221722	5.08559	5.171196	4.824303	5.128213	5.155958	5.288894	5.48664
Syn3	-0.408167	2.840108	-1.893974	0.078314	0.192809	-5.1596	2.885927	2.643736	2.820842	3.114742	2.139408	2.791131	2.930765	3.211662	3.169295	2.720538	2.833517	2.921125	2.954296	2.847239
Snd1	0.246471	7.099722	1.89342	0.078393	0.19296	-5.551686	6.753616	6.893312	6.940869	6.85777	7.279981	7.094132	7.075642	7.250177	7.258577	7.092792	7.245436	7.162958	7.180473	7.321917
Exoc8	-0.699655	4.862521	-1.893314	0.078408	0.19296	-5.501937	4.97826	3.7962	4.819324	5.836471	4.5954	5.611626	4.667523	5.036833	4.733316	4.417954	4.969824	4.843629	5.280867	4.544972
Kcnf1	-0.47414	3.700167	-1.893064	0.078443	0.193008	-5.37668	4.235407	3.551306	4.179109	4.121446	3.716556	3.340548	3.654827	2.951874	3.842287	3.050666	2.986432	3.644018	4.200009	4.454537
Eogt	0.473584	4.309853	1.892633	0.078504	0.193119	-5.351259	3.793849	4.75259	4.357754	3.63871	3.775594	3.875271	4.641297	4.560794	4.830952	4.491011	4.095154	4.523011	4.522853	4.561731
Ccdc94	0.238545	4.822338	1.892039	0.078588	0.193286	-5.482024	4.846411	5.140412	5.148852	4.766176	5.046656	4.839026	4.579502	4.876565	4.710323	4.785087	4.81759	4.803137	4.519446	4.689229
Traf2	0.37641	3.858085	1.891875	0.078612	0.193295	-5.298387	3.753999	3.479652	3.941366	3.122426	4.051203	3.693437	3.90205	4.117861	3.924502	4.114177	4.276668	4.105722	3.892823	3.745801
Bcam	0.672886	5.286363	1.891721	0.078633	0.193295	-5.497439	5.791322	5.882	5.527397	3.980586	5.529352	5.221502	5.283586	4.995405	5.485533	5.519255	5.300397	5.287022	4.811788	5.436635
Orc2	0.347085	5.154707	1.891579	0.078654	0.193295	-5.497963	4.835542	5.640537	5.301747	5.080109	5.050645	5.24203	5.408795	5.127283	5.2845	5.292808	4.946707	4.880423	4.919116	5.200217
Fkbpl	-0.42652	4.902827	-1.89156	0.078656	0.193295	-5.532202	5.446693	4.646529	5.38375	5.990312	5.428721	5.704543	4.443022	4.523808	4.560429	4.643844	4.644506	4.379835	4.495367	4.403387
G3bp2	-0.55672	6.685335	-1.890933	0.078745	0.193474	-5.562514	6.935127	5.932887	6.819978	7.426287	6.644503	7.095073	6.395861	6.664765	6.537089	6.126843	6.824402	6.650281	7.138889	6.418428
Snapc2	0.428673	6.311158	1.890601	0.078793	0.193548	-5.557091	6.449429	7.001345	6.327003	5.86995	6.922714	6.098051	5.947422	6.362423	6.055107	6.575336	6.454128	6.395437	5.877239	6.040905
Dgcr8	0.323021	5.845358	1.890494	0.078808	0.193548	-5.543887	5.487189	6.149865	6.240838	5.915994	5.76247	5.953053	5.985148	5.652036	5.826779	5.93071	5.737561	5.695207	5.574785	5.950893
Oat	-0.29541	6.303982	-1.890152	0.078856	0.193628	-5.559372	6.474167	5.78805	6.120952	6.434125	6.285188	6.43912	6.418098	6.239806	6.47511	6.208917	6.415922	6.235236	6.478996	6.262116
Setmar	-0.617599	2.531292	-1.889217	0.078989	0.193882	-5.167138	2.896425	1.369077	2.626517	3.275	2.856578	2.99033	2.186319	2.471744	2.47808	2.454393	2.782205	2.284645	2.58862	2.460763
Hebp1	0.345683	3.602762	1.889081	0.079009	0.193882	-5.35992	3.707065	3.854016	4.143209	3.82447	3.990197	4.443576	3.299932	3.40486	3.480126	3.232094	3.537463	3.269114	3.40196	2.982839
Best1	0.828394	0.499778	1.88907	0.07901	0.193882	-4.531324	0.369528	1.047298	0.484899	-0.81875	0.091273	0.435061	0.625837	1.067371	1.218642	0.750369	0.676952	0.969826	0.698429	0.554014
Sycp3	-0.673415	1.294359	-1.888972	0.079024	0.193882	-4.918278	1.853246	1.425638	1.825611	2.093442	1.143601	0.881356	1.809437	1.596873	1.180909	0.799637	0.885831	0.567345	0.698429	2.030937
Ppp1r1a	-0.392595	7.2012	-1.888794	0.07905	0.193905	-5.560351	8.049795	7.307249	7.454789	7.227324	6.888019	7.343272	6.806523	7.211287	7.032286	7.346969	7.476457	7.081216	6.768116	6.834406
Ptpn9	0.291872	4.883281	1.888336	0.079115	0.194022	-5.486505	4.865238	4.989118	5.280872	4.688073	5.016379	4.969911	5.009657	4.732714	4.864068	4.670575	4.847853	4.781067	5.112432	4.591416
Atg13	-0.236839	7.086043	-1.888235	0.07913	0.194022	-5.565267	7.237715	7.204418	7.213725	7.46684	6.895247	7.147702	7.081227	7.019433	7.072124	6.860047	6.996362	6.882427	7.141662	6.99727
Rpe65	-0.869634	1.7164	-1.888003	0.079163	0.194063	-4.909936	2.196854	2.519311	1.004744	1.799605	1.113405	0.117189	2.100922	2.14364	1.549949	2.256873	2.117203	2.461578	1.040235	2.142093
Gtf3c5	0.365965	5.331628	1.887479	0.079237	0.194207	-5.528865	5.388389	6.084399	5.82595	5.21918	5.593651	5.174216	5.027123	5.212396	5.151822	5.188006	5.213971	5.130001	5.130412	5.342788
Slitrk4	-0.610553	4.461411	-1.887109	0.07929	0.194297	-5.431921	4.10118	2.991607	4.102999	5.019674	4.132497	4.565325	4.7114	4.628172	4.815644	4.382013	4.632967	4.526331	5.101079	4.825924
Igsf8	-0.304463	7.415362	-1.886379	0.079395	0.194513	-5.570549	7.091202	6.816076	6.68859	7.024415	6.901887	6.608111	7.956647	7.659294	7.925553	7.821138	7.62583	8.060188	7.555463	8.09062
Trim35	-0.276643	7.840196	-1.885562	0.079512	0.19476	-5.560334	7.823377	7.263311	7.525581	7.898265	7.818572	7.731101	8.071581	7.848585	7.98868	7.967434	8.046126	7.971614	7.963212	7.852256
Dlx5	-0.851634	1.995948	-1.88534	0.079544	0.194799	-5.047956	2.311111	0.118359	1.981598	2.899083	2.542105	2.3616	2.324585	1.958936	1.981639	1.451089	1.844511	1.97042	2.253378	2.387449
Rexo2	0.418588	6.156844	1.88495	0.0796	0.194896	-5.567277	6.215844	6.430277	6.081833	6.242941	7.22588	6.852982	5.947422	5.931615	5.969169	5.875772	5.899217	5.810006	5.828509	5.906972
Abcc9	0.528222	4.530428	1.884419	0.079676	0.195043	-5.414677	4.158996	4.953495	4.687307	3.911307	4.412197	4.188991	5.122294	4.616508	5.021793	4.803319	3.949256	4.631832	4.337929	4.70138
Myo5a	-0.641548	7.233991	-1.884296	0.079693	0.195046	-5.567218	7.358425	6.799451	7.574189	8.078187	6.630579	7.300279	7.14261	7.384614	7.145585	6.437194	7.334956	7.074994	8.014336	7.01137
Usp20	-0.201885	7.049796	-1.883366	0.079827	0.195333	-5.57453	6.839765	6.544024	6.514312	6.685632	6.666117	6.519096	7.480925	7.356842	7.343749	7.281279	7.345158	7.331402	7.429513	7.371713
Cmip	-0.350805	7.282456	-1.882605	0.079937	0.195562	-5.573307	7.397287	6.859067	7.392557	7.50408	6.986057	7.163931	7.212345	7.394323	7.240349	7.074768	7.506804	7.37211	7.728484	7.132425
Mc4r	-0.630298	0.806064	-1.882312	0.079979	0.195625	-4.726692	0.739674	0.310827	0.158563	1.379803	1.082564	0.386712	1.258992	0.645799	1.102347	0.750369	0.484915	1.152831	1.252133	1.501815

Prlr	-3.047604	-1.413341	-1.882102	0.080009	0.195659	-4.443019	3.431974	-0.813242	-1.909221	-1.575022	-2.352077	-2.369898	-2.088409	-1.720374	-2.155202	0.646479	2.494078	-2.249472	-1.413173	-1.899685
Bai2	-0.31748	10.03666	-1.881635	0.080076	0.195784	-5.51212	9.555744	9.18892	9.425645	9.704009	9.242015	9.394027	10.74941	10.42884	10.72794	10.61513	10.16649	10.39563	10.20199	10.71911
Peak1	-0.696967	5.298807	-1.881431	0.080106	0.195816	-5.537366	5.174319	4.171795	5.46399	6.109156	4.854661	5.278924	5.191057	5.704396	5.204486	4.879766	5.679851	5.484316	6.045399	4.983491
Ift43	0.387323	3.395649	1.880683	0.080214	0.196019	-5.271727	3.37306	3.97739	3.277465	3.019194	3.590696	3.491064	3.554005	3.475434	3.280514	3.352542	3.461211	3.195935	3.186856	3.454224
Bgn	0.608466	5.96669	1.88063	0.080221	0.196019	-5.562328	6.306205	6.082202	5.812506	5.147559	6.595509	6.823204	5.554433	6.214239	5.632548	6.122009	5.776103	6.39271	5.152576	5.948346
Basp1	-0.717708	9.011571	-1.88033	0.080265	0.196085	-5.513637	9.309334	8.158739	8.953911	10.23942	9.118705	9.992578	8.598872	8.977739	8.955065	8.598499	9.00502	8.651059	9.121469	8.484798
Hsd17b8	0.334285	4.948187	1.880213	0.080282	0.196087	-5.493977	4.899562	5.331995	4.872118	4.512069	5.141352	4.807235	5.003787	4.927801	5.124751	5.155258	4.904139	4.758654	4.81457	5.072721
Dpf2	0.342363	6.395642	1.879801	0.080341	0.196192	-5.572859	6.31699	6.531193	6.513034	5.87109	6.730324	5.965451	6.361849	6.488286	6.442594	6.534992	6.533851	6.383585	6.364974	6.519686
Stox2	-0.590752	5.956939	-1.879257	0.08042	0.196345	-5.574735	6.248988	5.64947	6.28793	6.672607	5.097676	6.443558	5.955046	5.855927	5.687401	5.49889	5.762929	5.91634	6.528989	5.817698
Rabep2	0.416061	4.45397	1.879063	0.080448	0.196373	-5.421038	4.137033	5.003124	4.360599	3.950685	4.511054	3.97675	4.527267	4.600808	4.485023	4.760414	4.549498	4.659314	4.28674	4.620503
Alkbh2	-0.318388	2.896655	-1.878131	0.080583	0.196663	-5.180312	2.927466	2.557778	2.869592	2.968937	2.597694	2.493017	3.211029	2.862532	3.04878	3.019813	3.108108	3.037402	2.994118	3.069767
Pold4	0.575546	2.623624	1.877901	0.080617	0.196666	-5.089817	2.843156	3.34683	2.654608	1.679737	2.692656	2.549011	2.380012	2.68033	2.430637	3.195748	2.81321	2.703718	2.549064	2.474989
Eif3k	0.547537	5.440688	1.877898	0.080617	0.196666	-5.519831	5.406864	6.06004	5.23974	4.4153	5.608612	4.900582	5.615366	5.478195	5.653363	5.846766	5.68405	5.570476	5.278854	5.4493
Rab34	0.460255	4.898519	1.877467	0.08068	0.196778	-5.451021	4.481855	5.202427	4.59126	4.001571	4.675086	4.326706	5.397647	5.059983	5.308757	5.468746	5.132316	5.060699	4.786497	5.141226
Slc5a5	-1.748651	2.473953	-1.877263	0.080709	0.196811	-5.14723	5.227254	2.151237	1.472789	1.720809	1.510943	1.585258	2.518182	1.71825	2.539002	3.48645	4.942039	2.404977	1.316372	2.402413
Nudcd1	-0.553069	3.476542	-1.876519	0.080817	0.197035	-5.341147	3.589067	2.411416	3.550922	4.086518	3.384903	3.761378	3.488265	3.606999	3.555781	3.28495	3.518775	3.361247	3.97969	3.237699
Hs3st2	-0.378235	3.597251	-1.876034	0.080888	0.197129	-5.325774	3.652372	3.532115	3.138503	3.696477	3.075998	3.479584	4.074361	3.349568	3.761508	3.88487	3.592118	3.936334	3.646466	3.673625
Neurl1	-0.484966	5.956327	-1.876027	0.080889	0.197129	-5.580914	6.79488	5.678853	6.299838	6.132174	5.702881	6.22465	5.837869	5.516354	5.544292	5.618694	5.647241	6.17304	6.342012	5.901716
Nme1	0.249142	5.967077	1.87573	0.080932	0.197195	-5.577976	6.16899	6.313361	6.197736	5.928101	6.368248	6.308146	5.932051	5.625125	6.03145	5.785415	5.828894	5.610136	5.816065	5.650719
Egfem1	-0.609105	1.901096	-1.87551	0.080964	0.197225	-5.001598	1.831529	2.01041	2.187895	2.591079	0.985877	1.353343	2.01015	1.934092	2.330799	1.704918	1.978199	2.187314	1.837926	2.105984
Itgb8	-0.722024	3.639433	-1.875419	0.080978	0.197225	-5.407477	4.388265	3.49955	4.092769	4.214255	2.530726	4.156917	3.180134	3.484018	3.613573	2.707689	3.70112	3.692118	4.322371	3.503633
Cacnb2	-0.620668	6.687286	-1.874774	0.081072	0.197414	-5.588336	6.785027	5.472854	6.589603	7.357551	6.604357	7.13083	6.953071	6.909823	6.800009	6.463228	6.835142	6.409891	6.853131	6.473492
Rhot1	-0.349016	6.091272	-1.874453	0.081118	0.197488	-5.584302	6.152662	5.794776	5.987435	6.755859	6.226445	6.415968	6.203151	5.939438	5.9299	5.770048	6.077933	5.938943	6.0966	6.011927
Tsc22d4	0.57551	2.557069	1.873786	0.081216	0.19764	-5.104118	2.776524	3.1924	3.151703	1.818649	2.661692	2.3616	2.361772	2.186344	2.241976	2.720538	2.823399	2.749899	2.508394	2.516844
Vip	-0.432873	3.666065	-1.873729	0.081224	0.19764	-5.272521	3.670834	2.884706	2.954902	3.145235	3.245679	2.781814	4.202878	3.668539	3.901486	4.341	3.981461	4.074306	4.229503	4.377115
Kbtbd3	-0.499286	3.326787	-1.873588	0.081245	0.19764	-5.326677	3.100876	3.099845	3.063656	4.294667	3.167012	3.57437	3.639767	2.91426	3.548392	3.28495	3.235656	3.009199	3.556237	3.246018
Ptpn13	0.309006	5.321775	1.873575	0.081247	0.19764	-5.542381	5.405027	5.56858	5.569811	4.958743	5.194602	5.61294	5.503348	5.338449	5.081888	5.357987	5.252185	5.312249	5.12145	5.267145
Myo9a	-0.517873	5.794301	-1.872739	0.081369	0.197897	-5.574744	5.504448	5.45088	5.864543	6.561825	5.080218	5.673392	5.982166	6.066325	5.919913	5.445717	5.816195	5.644157	6.365923	5.773779
Spata22	2.293192	-1.679777	1.872452	0.081411	0.197959	-4.205873	-3.260591	1.117652	-1.705055	-1.075778	-0.516166	-1.26448	-1.450335	-1.720374	-1.813089	-0.961779	-2.293564	-0.657145	-1.413173	-0.505482
Pus7	0.388931	4.595914	1.871758	0.081513	0.19816	-5.495313	4.380808	4.879497	5.103716	4.753862	4.633136	5.216324	4.874668	4.327931	4.726784	4.49849	4.073974	4.229042	4.182881	4.528015
Acp5	0.550183	1.862795	1.871663	0.081526	0.19816	-4.948488	1.717752	2.21681	2.08155	1.478901	2.496038	1.747207	0.851758	2.007376	1.255413	2.40749	2.428842	2.738492	1.54805	1.556352
Ptprn	0.236267	9.452911	1.870332	0.081722	0.198554	-5.54715	8.951297	9.035527	9.346593	8.999757	9.165788	9.302721	9.889023	9.486685	9.826604	9.559389	9.379952	9.522797	9.760878	10.01606
Npy2r	-2.262885	2.578308	-1.870319	0.081724	0.198554	-5.036803	2.843156	-3.260591	2.861581	3.696477	3.099296	2.705027	3.410541	3.232189	3.88398	2.375352	3.165302	2.726993	3.949625	3.591131
Bicc1	0.633942	2.536414	1.870137	0.08175	0.198554	-5.228788	3.239259	3.1924	3.633224	2.451642	2.752661	4.199526	1.809437	2.400986	2.024877	2.14695	2.049375	2.203999	1.651284	2.050068
Dll4	0.647166	2.344829	1.870107	0.081755	0.198554	-5.051096	2.41698	2.854122	2.829082	1.615848	2.702832	2.146157	3.01488	2.14364	2.720913	2.469694	1.482482	1.783252	2.11575	2.858134
Stxbp4	-0.606089	1.954642	-1.869763	0.081805	0.198637	-5.063597	2.896425	1.369077	1.613009	2.029493	1.96438	2.410784	2.227194	2.227821	1.743567	1.355481	2.149958	1.910689	2.151407	1.73247
Ptplad2	1.590107	-0.481409	1.869249	0.081881	0.198781	-4.343114	0.105685	-1.10215	-0.665442	-2.731185	0.422095	0.284862	-0.529223	0.705741	-1.304935	0.534523	0.764165	1.11804	-1.047095	-0.297732
Nts	-1.123018	4.657148	-1.868583	0.081979	0.198958	-5.474731	3.614724	4.538474	2.770386	6.161373	3.188895	4.777073	5.164996	5.170467	4.511866	5.364141	5.424393	5.549414	4.178567	4.864259
Tradd	0.583418	2.456643	1.86842	0.082003	0.198958	-5.018928	1.619793	2.943995	1.858187	2.108996	2.77212	2.323579	2.450751	2.888628	2.446625	2.795315	2.639567	2.9008	2.220182	2.721621
Osbpl6	-0.35836	6.024802	-1.868415	0.082004	0.198958	-5.584541	5.765785	6.011209	5.678694	6.321812	5.457552	5.608994	6.569814	6.192145	6.557478	6.028237	5.896807	5.913807	6.115906	6.253889
Itga7	-0.34267	5.990798	-1.868296	0.082021	0.198961	-5.55588	5.369676	5.216521	4.866124	5.543523	5.226692	5.163494	6.888442	6.461371	6.838386	6.602487	6.22449	6.291938	6.313769	6.892529
Crbn	-0.441154	5.61693	-1.867728	0.082105	0.199124	-5.583595	5.603877	5.062295	5.700192	6.433353	5.657824	5.88941	5.558443	5.613437	5.534975	5.213226	5.642933	5.385387	5.825753	5.548486
Tacr2	-1.503717	0.912089	-1.867135	0.082193	0.199295	-4.66682	1.48718	-2.680539	-0.067513	0.664146	0.562819	1.136566	0.851758	1.830204	1.819202	1.481601	1.719153	2.170435	2.186205	1.29285
Comt	0.24543	6.636665	1.867014	0.08221	0.199298	-5.599566	6.626964	6.728862	6.904776	6.436439	6.826528	6.646475	6.2159	6.712114	6.557478	6.714303	6.767491	6.84502	6.371603	6.575216
Mocs3	-0.513639	3.231214	-1.866833	0.082237	0.199323	-5.317636	3.460552	2.506257	3.253175	3.761892	2.919011	3.693437	2.905804	2.976416	3.341641	3.186517	3.525031	3.009199	3.665088	3.203933
Usp9x	-0.450382	8.327536	-1.866607	0.082271	0.199334	-5.572544	8.096637	7.812428	8.2347	9.140501	8.088013	8.527826	8.490245	8.292764	8.358154	7.995126	8.350872	8.146336	8.639097	8.417798
Rpusd3	0.548216	4.239825	1.866564	0.082277	0.199334	-5.393509	4.123692	4.738705	4.419086	3.288723	4.282511	3.519371	4.421169	4.393186	4.628589	4.353427	4.454361	4.118979	4.387364	4.315206
Cort	-0.601864	1.788115	-1.866467	0.082291	0.199334	-5.052903	2.843156	1.991799	1.705429	1.92792	1.745593	1.668504	2.343298	1.020936	1.549949	1.920699	1.903329	2.027776	1.218908	1.634478
RGD1311345	0.214659	4.804625	1.865488	0.082436	0.199644	-5.52172	4.748409	5.071186	4.907568	4.812037	4.995836	5.010363	4.622269	4.823442	4.639062	4.806336	4.91609	4.692208	4.665321	4.610873
RGD1307235	-0.393699	6.289685	-1.865151	0.082486	0.199725	-5.597511	6.011315	5.690441	6.101525	6.744701	5.937483	6.21083	6.604164	6.491483	6.166694	6.146019	6.496133	6.340848	6.720689	6.41382
Sel1l	-0.384094	7.230468	-1.864577	0.08257	0.19989	-5.605271	6.837723	6.449158	6.939918	7.427063	6.587294	7.015328	7.448343	7.605239	7.562651	7.060943	7.488112	7.449798	7.822379	7.543543
Nkx6-2	-0.482109	4.476395	-1.864268	0.082617	0.199961	-5.493609	4.725042	4.585778	3.550922	4.695818	4.801203	4.032808	4.14005	4.736311	4.380707	4.585342	4.638748	4.958703	4.44597	4.465266
Cd300lf	0.505304	3.088749	1.864096	0.082642	0.199983	-5.067094	2.035255	2.532247	2.568639	2.108996	2.919011	2.271274	3.116285	4.433629	2.928701	4.150009	3.862983	4.405307	2.860463	3.262515
Dgcr2	-0.49803	3.405645	-1.863525	0.082727	0.200147	-5.330011	3.652372	2.643736	3.595078	3.63871	3.326873	2.974059	2.930765	3.676051	3.440756	3.195748	3.695584	3.761389	3.882251	3.417866
Paxip1	-0.199748	6.112894	-1.863359	0.082752	0.200167	-5.601343	6.324783	6.191234	6.235424	6.387855	6.032323	6.168559	6.162897	5.93475	6.16307	5.980965	5.948925	5.974628	6.067732	6.030106
Mterfd3	-0.427326	2.611806	-1.862962	0.082811	0.200262	-5.108912	2.196854	1.812527	2.122353	2.87199	2.361201	2.203108	3.169687	2.901501	3.110295	2.866406	2.792614	2.63155	2.881849	2.91141
Mtmr4	-0.259138	6.989591	-1.86287	0.082825	0.200262	-5.608297	6.845194	6.663487	6.787575	7.265994	6.819531	6.915023	7.174311	7.08579	7.115898	6.783285	6.991852	6.915727	7.327022	7.176072
Irs3	0.790889	0.259849	1.861796	0.082984	0.200608	-4.646876	0.692264	0.895377	0.715742	-0.411255	0.606834	1.193889	-0.067151	0.871929	-0.632503	0.591587	-0.072212	0.151783	-0.040977	0.499386
Rab30	-0.363837	3.280752	-1.861115	0.083086	0.200764	-5.306671	3.350332	3.1924	3.265371	3.54462	3.060255	2.845805	3.347054	3.599118	3.207329	3.267546	3.592118	3.153617	3.589113	3.079111
Serpinb2	3.761184	-4.007832	1.86111	0.083086	0.200764	-4.149632	-3.260591	-3.260591	2.700244	-3.260591	-2.049163	-1.26448	-3.260591	-3.260591	-3.260591	-3.260591	-3.260591	-3.260591	-3.260591	-2.228037
Pcdhac1	-1.388373	-0.063806	-1.861028	0.083099	0.200764	-4.572931	0.307952	-2.680539	-0.263851	0.664146	-0.257548	0.435061	0.282279	0.58326	0.091681	-0.537962	0.805871	0.666985	1.1849	0.119416
Phlpp2	-0.484028	5.216201	-1.860788	0.083135	0.20081	-5.556575	4.960861	5.220523	5.133963	5.733616	4.319352	4.81182	5.64395	5.555567	5.598964	4.944172	5.274637	5.19195	5.509306	5.172115
Ssrp1	0.242447	7.731949	1.860602	0.083162	0.200836	-5.602357	7.43468	7.78904	7.829359	7.739322	7.779745	7.917062	7.843308	7.820478	7.753074	7.834867	7.647856	7.620696	7.542093	7.703123
Ing3	-0.412891	4.340353	-1.859907	0.083266	0.20102	-5.533348	4.777095	3.97739	4.712023	5.258066	4.779733	4.942996	3.882992	4.044317	4.096495	3.95178	4.235294	3.985416	4.258405	3.942904
Mettl20	0.659903	2.078145	1.85987	0.083272	0.20102	-4.98305	2.372556	2.702143	2.10888	0.932396	2.28197	1.976852	2.206902	2.419003	2.04602	2.342482	2.544222	2.203999	1.436856	1.910497
Atp7b	-0.541813	3.178353	-1.859495	0.083328	0.201114	-5.199479	3.239259	2.758277	2.845423	2.728325	1.981199	2.160607	3.545949	3.93587	3.216682	3.545408	3.525031	4.216763	3.45286	3.537917
Abca5	-0.489266	4.108376	-1.859212	0.08337	0.201154	-5.419949	3.488576	3.780021	3.851187	4.716273	3.403739	3.508115	4.855242	4.095628	4.363967	4.056832	4.073974	4.17506	4.689778	4.555051
Mrps31	0.314196	4.643355	1.859057	0.083393	0.201154	-5.514348	4.280186	4.946264	4.854061	4.863379	4.728358	5.043877	4.746971	4.632039	4.632088	4.49849	4.612548	4.433879	4.405935	4.392186
Tspan13	-0.755416	7.619983	-1.859048	0.083394	0.201154	-5.609538	7.382428	5.574846	6.639067	7.835663	7.560517	7.636707	7.817609	7.930157	8.003279	7.958988	7.998304	8.064762	8.087778	8.199042
Evl	-0.207895	7.565727	-1.858715	0.083444	0.201205	-5.602338	7.82921	7.621346	7.606307	7.940443	7.672836	7.806548	7.470855	7.420272	7.432224	7.564783	7.430953	7.389993	7.381995	7.360742
Phf11	0.857492	1.05066	1.858685	0.083449	0.201205	-4.709707	0.428583	1.558038	1.534559	0.33436	1.018831	0.695385	1.782265	1.564863	0.17081	1.704918	1.167576	1.568131	0.598632	1.386135
Kpna5	-0.463441	3.075884	-1.858356	0.083498	0.201283	-5.293042	2.917193	2.943995	3.177748	3.861672	2.484288	3.179592	3.094359	3.093229	3.244385	2.854798	2.968181	2.869767	3.40196	3.160583
Ccnb2	1.617869	-0.935764	1.857925	0.083563	0.201398	-4.435625	-0.899176	0.480615	-1.223881	-1.388744	1.85913	0.284862	-1.450335	-0.81357	-1.304935	-0.961779	-1.719763	-0.657145	-1.413173	-0.621773
Abl2	-0.634834	4.300775	-1.857724	0.083593	0.20143	-5.485599	4.045673	3.909605	4.603321	5.193877	3.333437	4.124114	4.421169	4.572916	4.656351	4.056832	4.460902	3.936334	4.850267	4.129836
Ube2b	-0.342692	7.009653	-1.857549	0.083619	0.201453	-5.614074	7.130639	6.770401	6.898427	7.676117	7.163238	7.407534	7.084011	6.773798	6.963113	6.80305	6.990722	6.668429	6.984968	6.833026
Adamts2	0.783118	3.137522	1.857249	0.083664	0.201463	-5.22861	3.303772	4.379337	3.47943	2.108996	3.346477	2.675144	3.624549	2.345542	3.487872	2.988286	3.03984	2.619163	3.089114	3.629792
B4galt6	-0.472229	7.232542	-1.857192	0.083672	0.201463	-5.616817	6.911386	5.913241	6.915457	7.419286	6.804254	7.136324	7.53585	7.314155	7.516059	7.456627	7.616705	7.51058	7.712161	7.504634
Dnajc18	-0.399362	4.858057	-1.857187	0.083673	0.201463	-5.554889	4.955851	4.472889	5.09178	5.48363	4.608991	5.108656	4.667523	4.833553	4.833994	4.494755	4.736341	4.733017	5.254467	4.792236
Porcn	-0.290559	7.102265	-1.856497	0.083777	0.201584	-5.61735	6.681093	6.450011	6.538383	6.867519	6.607068	6.34103	7.637906	7.438604	7.694862	7.589069	7.192865	7.413496	7.2443	7.747747
Usp16	0.287182	5.380199	1.856447	0.083784	0.201584	-5.567521	5.048187	5.659822	5.405955	5.256321	5.388311	5.314898	5.626867	5.214976	5.618503	5.530243	5.294916	5.275228	5.208658	5.517894
Vsx2	1.071541	1.147473	1.856374	0.083795	0.201584	-4.753457	0.031429	2.248512	2.225837	1.000693	0.808768	1.014597	1.607516	1.155984	1.180909	1.964563	1.290185	0.760186	0.313987	1.227132
Msrb2	-0.421845	4.097131	-1.856366	0.083796	0.201584	-5.454068	4.432216	4.350411	3.330668	4.121446	3.8553	3.931123	4.451671	4.198308	3.974983	4.365748	4.33124	4.241218	3.871601	3.998126
Enah	-0.431395	6.127377	-1.856295	0.083807	0.201584	-5.618421	6.71855	6.098602	6.62079	7.243709	6.484138	6.994305	5.57635	5.832592	5.477774	5.458779	5.809803	5.727301	6.136069	5.601319
Spata6l	-0.507697	2.948453	-1.855476	0.08393	0.201839	-5.16485	2.460078	2.36878	1.996308	3.067759	2.295481	2.481552	3.713543	3.517851	3.333065	3.669864	3.099749	3.220743	2.923692	3.35002
Golga4	-0.332337	7.411258	-1.855074	0.083991	0.201873	-5.612355	7.334499	7.24329	7.406047	8.098782	7.242543	7.639934	7.424375	7.424737	7.294356	7.287229	7.282375	7.307476	7.481658	7.29961
Mdga2	-0.400715	3.843636	-1.855048	0.083995	0.201873	-5.446375	3.859704	3.673115	3.906747	4.546747	3.511865	4.153309	3.727855	3.852146	3.729193	3.409193	3.760656	3.698019	4.080242	4.012827
Mkks	-0.416804	5.237947	-1.855029	0.083998	0.201873	-5.589464	5.536682	4.7275	5.108801	5.843462	5.624757	5.631214	5.146469	5.001397	5.07419	5.079985	5.28389	4.81947	5.420761	5.075058
Pskh1	0.561436	2.553669	1.854935	0.084012	0.201873	-5.081661	2.445855	3.117121	2.508341	1.679737	2.586747	2.217002	2.266943	2.471744	2.667343	3.040455	2.81321	2.869767	2.452318	2.890335
Zfp202	0.580406	4.075713	1.854139	0.084132	0.202121	-5.365789	3.365524	4.707679	3.719973	3.474303	3.8553	3.770827	4.53949	4.393186	4.321248	4.602796	3.999546	4.158035	3.701625	4.548339
Rbfox2	-0.561476	5.661775	-1.853976	0.084156	0.202139	-5.614507	6.070454	5.437189	6.223752	6.757711	5.487291	6.260309	5.295647	5.695184	5.424278	4.824303	5.597644	5.099115	5.930803	5.19378
Cdo1	0.502614	7.012598	1.853768	0.084188	0.202139	-5.620524	6.977149	6.943808	7.586089	6.940512	7.581694	7.738043	7.548001	6.875166	6.469252	6.655301	6.973098	6.510567	6.864589	6.525678
Kcna3	-0.801971	1.593347	-1.853699	0.084198	0.202139	-4.958944	1.48718	0.679782	2.067688	2.43939	0.649545	1.327961	1.21902	1.958936	1.981639	1.28803	1.82436	1.691577	2.562371	1.684306
Klhl12	-0.317832	4.895977	-1.853638	0.084207	0.202139	-5.53919	4.725042	4.604273	4.600917	5.140008	4.451953	4.820946	5.312364	4.980316	5.13958	4.871149	5.200353	4.73014	5.105631	4.914707
Cacfd1	-0.51068	3.430678	-1.853531	0.084223	0.202139	-5.385581	4.031456	2.631765	3.505372	3.706736	3.286847	3.931123	3.30948	3.440579	3.01701	3.030171	3.555912	3.619352	3.5956	3.517445
Arhgap4	0.494983	5.43739	1.853336	0.084253	0.20217	-5.549532	4.870572	5.59964	4.839859	4.738944	5.635421	5.132675	5.897647	5.869094	5.586392	6.057647	5.530819	5.751622	4.957391	5.69393
Rps6kb2	0.552606	6.874927	1.85237	0.084399	0.20248	-5.621337	6.442282	7.627763	6.923176	6.307559	6.642522	6.514181	7.271554	6.82706	7.255744	7.265475	6.650038	6.760835	6.43084	7.343885
Rlbp1	-0.428128	5.034246	-1.852178	0.084428	0.202509	-5.555656	5.444906	5.200402	4.207831	4.830919	4.635794	4.81182	5.22677	4.889545	5.347591	5.169384	4.939699	5.306467	5.163531	5.354378
Eef1a1	0.298849	8.636737	1.852019	0.084452	0.202526	-5.577331	8.953969	8.878816	9.267701	8.89739	9.160144	9.598325	8.150403	8.471195	8.210598	8.30712	8.277498	8.484362	8.151347	8.109551
Ric8b	-0.307894	3.882745	-1.85164	0.084509	0.202564	-5.458139	3.891537	3.607396	3.797607	4.022255	3.391209	3.803422	4.129307	3.966821	3.958351	3.802672	4.204441	3.895831	4.125768	3.869418
Nob1	0.394298	5.934758	1.851635	0.08451	0.202564	-5.596621	5.732483	6.222499	5.911863	5.233442	5.997614	5.365672	6.284076	5.987017	6.173915	6.211195	5.97886	5.890805	5.890488	6.233114
Asb3	-0.441293	3.978709	-1.851524	0.084527	0.202564	-5.474034	4.064414	3.466233	4.11989	4.690659	3.716556	4.446528	3.927077	4.106787	4.060584	3.62956	3.906764	3.686193	4.018826	3.96323
Giot1	-0.641345	0.84614	-1.851361	0.084551	0.202564	-4.788256	1.143792	0.426209	0.883381	1.128291	0.422095	0.336685	1.335758	-0.048075	1.29127	1.180545	1.290185	0.804622	1.077784	1.473753
Dirc2	-0.311798	6.34195	-1.851356	0.084552	0.202564	-5.618755	6.24386	5.607302	6.041624	6.366175	6.214937	6.107405	6.270696	6.508411	6.546392	6.549466	6.424314	6.628044	6.528989	6.76952
Fubp3	0.301704	5.98149	1.8507	0.084652	0.202741	-5.615852	5.959694	6.341195	6.401418	5.94228	5.889289	6.377053	5.903964	5.930045	6.101288	5.839422	5.628478	5.846329	5.654608	5.950893
Rab2a	-0.684359	7.105967	-1.850648	0.084659	0.202741	-5.61669	7.462764	6.29924	7.307386	8.452384	7.375709	8.107632	6.740115	6.91221	6.944233	6.566468	6.992416	6.732364	7.077077	6.525678
Lrfn4	0.288082	5.443173	1.850432	0.084692	0.202779	-5.565306	5.048187	5.352198	5.515958	4.939298	5.230214	5.020301	5.756435	5.555567	5.689082	5.756075	5.47536	5.678888	5.45148	5.77234
Fam168b	-0.539915	7.726916	-1.849704	0.084803	0.203003	-5.609675	8.049207	6.976713	8.213611	8.512465	7.623807	8.155086	7.404465	7.666853	7.535811	7.265475	7.776665	7.617196	7.948085	7.439024
Tmem134	0.525414	4.832217	1.848505	0.084985	0.203398	-5.519856	4.909961	5.437189	4.942168	3.933316	5.089943	4.274516	4.743453	4.983347	4.922398	5.15289	4.906537	5.090167	4.576302	4.746057
Abcb7	-0.37778	3.382528	-1.847946	0.08507	0.203562	-5.346874	3.287913	3.151065	3.203331	3.810268	3.036313	3.34688	3.299932	3.211662	3.533498	3.186517	3.408041	3.529544	3.964736	3.537917
Mrpl2	0.422382	4.309145	1.847056	0.085206	0.203845	-5.463694	4.33909	4.804167	4.43537	3.633342	4.384017	3.972661	4.306644	4.374841	4.575048	4.390078	4.373459	4.208518	4.13915	4.472374
Cnksr1	1.596542	-0.664597	1.846775	0.085248	0.203885	-4.568691	0.643243	1.914859	1.220561	-1.575022	0.210178	0.336685	-1.009457	-0.81357	-1.536754	-0.806258	0.001431	-0.434228	-3.260591	-0.116166
Timm23	0.300775	3.500334	1.846686	0.085262	0.203885	-5.35621	3.431974	3.53854	3.505372	3.322471	3.76592	3.884005	3.479833	3.567159	3.432752	3.360772	3.661913	3.52291	3.423994	3.246018
Marc2	-0.310268	5.52195	-1.846486	0.085293	0.203885	-5.593854	5.705873	5.37392	5.008973	5.285703	5.223162	5.136335	5.513711	5.627064	5.700795	5.933471	5.527707	5.990343	5.576426	5.738835
Dhfr	0.640327	2.370673	1.846409	0.085304	0.203885	-5.159293	2.295333	3.390109	2.10888	2.350586	3.306999	3.121686	2.361772	1.775313	2.223535	2.423294	2.066633	2.064785	1.675978	2.341603
Dcaf7	-0.280115	7.304254	-1.846284	0.085323	0.203885	-5.631026	7.364581	6.945018	7.424179	7.542533	7.07443	7.251754	7.248161	7.272026	7.32062	7.169176	7.516257	7.292928	7.546284	7.301607
Hic1	1.195469	-0.029144	1.84628	0.085324	0.203885	-4.533496	-1.052261	1.339943	-0.761158	-0.243853	0.517419	0.982423	-0.279766	-0.380636	0.091681	0.646479	0.432628	0.151783	-0.123798	-0.03329
Grhpr	-0.33702	4.852684	-1.846121	0.085348	0.203892	-5.552495	4.870572	4.414697	4.096187	4.520817	4.760377	4.167688	5.138455	5.183701	5.259827	5.253565	5.280196	5.09241	4.916528	5.039604
Sec11a	0.296501	5.270509	1.845922	0.085379	0.203892	-5.574356	4.846411	5.093178	5.160327	5.184732	5.375645	5.626016	5.154438	5.333708	5.299983	5.453434	5.22362	5.410762	5.282878	5.38295
Lgi4	0.461999	7.494606	1.84583	0.085393	0.203892	-5.631321	7.39266	8.300171	7.07467	6.969558	7.646719	7.542269	7.403351	7.564844	7.243212	7.798973	7.382677	7.880492	7.275438	7.458396
Nudt9	0.228019	6.503315	1.845818	0.085395	0.203892	-5.634601	6.607928	6.855211	6.983943	6.792443	6.801297	7.072803	6.138997	6.376322	6.202444	6.285492	6.377541	6.275258	6.29097	6.003364
Cpsf3l	0.321916	5.38526	1.845133	0.085499	0.204101	-5.581449	5.117126	5.834485	5.356931	5.056238	5.362868	5.088014	5.515774	5.387314	5.622027	5.443781	5.248409	5.453251	5.326424	5.619042
Mxra8	0.565108	4.261398	1.844776	0.085554	0.204191	-5.461149	4.637014	4.267739	4.340564	3.197095	4.553844	4.639415	3.742028	4.833553	3.584966	4.581825	4.334806	5.096883	3.924083	4.012827
Prkar2b	-0.347161	5.403222	-1.844431	0.085607	0.204276	-5.621154	5.77577	5.234443	5.743341	6.204544	5.697802	5.894822	4.92833	4.863466	5.183174	5.217764	5.250298	5.069831	5.272795	5.346662
Trappc2	-0.479748	5.773767	-1.84337	0.085769	0.204624	-5.625986	5.832904	5.02617	5.589347	6.469951	5.794938	6.273664	5.991093	5.543299	5.895368	5.396526	5.805955	5.414351	6.033505	5.795197
Syngr3	-0.316765	6.688396	-1.843216	0.085793	0.20464	-5.632077	6.469781	5.959469	5.892335	6.183579	6.420821	5.767261	6.998051	6.917765	7.155954	7.293154	7.075753	7.091901	7.20772	7.22055
Adat3	0.417048	2.727929	1.842998	0.085826	0.204679	-5.15122	2.213741	3.046741	2.691232	2.427034	2.672087	2.570814	2.867532	2.724465	3.140097	2.988286	2.739799	2.999674	2.480628	2.868948

Galnt5	-1.665111	0.444052	-1.842349	0.085926	0.204841	-4.56484	0.592497	-3.260591	0.049949	0.280432	0.210178	-0.291112	1.000645	1.71825	1.743567	1.322149	1.290185	1.667719	1.747621	1.048408
Dennd2c	0.547177	2.334846	1.842334	0.085928	0.204841	-5.048742	2.246933	2.324846	1.996308	1.430203	2.424057	2.685174	1.938115	2.939445	1.549949	2.934159	3.004456	3.412394	1.922909	2.211707
Cisd3	0.547015	3.922021	1.841932	0.08599	0.204947	-5.400031	4.031456	4.432057	3.555895	3.002635	4.412197	3.798811	3.975863	3.811801	3.985965	4.061889	4.324082	3.794816	3.974722	3.853172
Vps29	-0.586071	6.752337	-1.841565	0.086047	0.205018	-5.636857	7.46981	6.038667	7.050647	7.779666	7.352466	7.713751	6.563843	6.382074	6.536155	6.169636	6.522952	6.181489	6.546671	6.24042
Cnbp	0.272808	7.992283	1.841516	0.086054	0.205018	-5.616197	8.209333	8.317322	8.667242	8.286817	8.49244	8.609588	7.780328	7.485846	7.67549	7.609935	7.934226	7.637299	7.70393	7.488509
Tmed1	0.483783	4.309266	1.841401	0.086072	0.20502	-5.431932	3.992844	4.716207	4.033387	3.425447	4.361713	3.642872	4.56363	4.420274	4.347031	4.709764	4.655954	4.811327	4.20426	4.528015
Mapk1	-0.780012	7.60158	-1.840289	0.086243	0.205358	-5.626177	7.797006	6.452565	7.874348	8.775065	7.429548	8.26804	7.271554	7.693929	7.461534	6.918366	7.798135	7.40134	8.061955	7.227394
Lamc3	-0.630951	3.401475	-1.840259	0.086248	0.205358	-5.211367	3.271877	2.545069	1.705429	2.57996	2.496038	2.469996	4.161298	3.891392	4.308181	4.373903	3.78171	4.170823	3.576053	4.472374
Nxpe3	-0.307498	5.540653	-1.840052	0.08628	0.205367	-5.594038	5.218914	4.619507	5.030571	5.474653	5.223162	5.282232	5.945892	5.857579	5.859989	5.748254	5.842734	5.793565	5.977491	5.729965
Nacad	0.416322	7.209319	1.840011	0.086286	0.205367	-5.643181	6.933217	7.910128	7.146903	6.881171	7.395449	6.835639	7.231306	7.353918	7.049401	7.423614	7.041249	7.486914	6.956275	7.296109
Ccdc71	-0.351165	4.629119	-1.839403	0.08638	0.20555	-5.564673	4.97331	4.220564	4.749408	4.986375	4.820023	4.709886	4.376444	4.65122	4.504248	4.429738	4.867681	4.606955	4.806206	4.169739
Sbno1	-0.596584	6.483421	-1.839107	0.086425	0.205618	-5.645293	6.623809	5.921869	6.750637	7.521257	6.106057	7.121166	6.247289	6.546814	6.319935	5.93071	6.511181	6.230154	6.825116	6.13013
Popdc3	0.812359	1.283524	1.838426	0.086531	0.205827	-4.802397	0.539902	2.36878	1.061799	0.785264	1.78444	0.734546	1.443753	0.871929	1.867508	1.180545	1.068256	1.11804	1.79348	2.030937
Ccdc28b	-0.460455	3.896042	-1.83791	0.086611	0.205976	-5.458128	4.206171	4.064569	3.319014	4.038591	3.969275	3.34688	4.150713	4.009066	4.290573	4.076955	3.487079	3.816678	3.614885	4.262873
RGD1561161	-1.155379	0.093411	-1.837302	0.086705	0.206159	-4.772054	2.474162	-0.813242	0.307539	-0.02435	-0.038319	0.65513	-0.399109	-0.380636	0.091681	0.348395	0.998029	-0.541385	0.249639	-0.03329
Gorasp2	-0.463953	7.437082	-1.836983	0.086754	0.206236	-5.635757	7.803644	6.822673	7.64766	8.226821	7.759475	7.978248	7.128519	7.218378	7.331424	6.935687	7.43345	7.09414	7.41858	7.329766
Cpne3	0.457959	3.165151	1.836057	0.086898	0.206501	-5.278672	3.082606	3.649562	3.426105	2.645422	2.847434	3.321383	3.577905	3.159031	3.375441	2.988286	2.771719	3.228918	3.423994	2.992761
Zfp35	-0.387828	3.880339	-1.836043	0.0869	0.206501	-5.472455	4.007445	3.588941	4.116527	4.341506	3.623205	3.817169	3.810873	3.985077	3.786848	3.696121	4.160911	3.70975	4.250206	3.537917
Aph1a	0.258274	6.271757	1.83565	0.086961	0.206605	-5.63624	6.011315	6.056687	6.196144	5.67212	6.254387	5.885066	6.360702	6.462457	6.371095	6.683464	6.467184	6.631131	6.293964	6.479679
Dcun1d4	-0.237034	6.45289	-1.835327	0.087011	0.206683	-5.647887	6.28239	6.205438	6.189759	6.703032	6.304527	6.326685	6.730374	6.378626	6.567565	6.420559	6.570608	6.487753	6.618497	6.572738
Hexdc	0.614801	4.530218	1.835029	0.087057	0.206712	-5.451208	4.145858	5.112689	4.380359	3.329127	4.187881	3.68846	4.842144	4.82682	4.806381	5.15289	4.650242	4.822175	4.481245	5.070381
Rbm8a	0.243948	5.683148	1.835028	0.087058	0.206712	-5.642326	5.922941	6.078899	6.308704	6.13027	6.352176	6.342615	5.321831	5.403241	5.399851	5.335194	5.416002	5.225023	5.314678	5.044382
Gfpt2	0.349127	2.456099	1.834847	0.087086	0.206715	-5.168804	2.474162	2.790938	2.804219	2.255954	2.651221	2.570814	2.659281	2.345542	2.278164	2.469694	2.401899	2.555581	2.236876	2.17732
Zer1	-0.262846	8.459209	-1.834799	0.087093	0.206715	-5.626715	8.617319	8.200872	8.346735	8.260726	8.279709	7.883427	8.688729	8.431528	8.65913	8.470768	8.570365	8.543529	8.745368	8.735259
Sec14l1	-0.20466	7.812618	-1.83446	0.087146	0.206799	-5.641708	7.867397	7.570909	7.669335	7.824224	7.775231	7.550522	7.840841	7.928979	7.802831	7.938513	7.945057	7.966123	7.953776	7.749942
Sp4	-0.491453	3.390251	-1.834171	0.087191	0.206865	-5.43442	3.608352	3.019437	3.922236	4.308205	3.129785	3.794184	2.943086	3.630385	3.244385	2.977621	3.366837	3.16218	3.431265	3.079111
Akt2	-0.254555	6.288382	-1.833768	0.087253	0.206952	-5.650817	6.541762	6.314297	6.010262	6.520986	6.430039	6.355233	6.262117	6.202584	6.330764	6.214605	6.173627	6.366086	6.011844	6.323349
Rngtt	-0.409474	4.742238	-1.833681	0.087267	0.206952	-5.597436	5.050539	4.290759	4.945961	5.533472	4.938861	5.351571	4.579502	4.429191	4.477262	4.531675	4.675772	4.390807	4.769388	4.486487
RGD1305420	-0.476733	1.463396	-1.833603	0.087279	0.206952	-5.004405	1.895723	1.184734	1.004744	1.892408	1.765147	1.785004	1.21902	1.393205	1.489496	1.597576	1.740817	1.404538	1.493524	1.193114
Stmn4	-0.287701	8.095661	-1.833141	0.087351	0.207074	-5.635296	8.210911	8.126045	7.76907	8.252871	8.170067	7.707922	7.788032	8.197466	8.071564	8.398652	8.125986	8.347221	8.10635	8.0729
Map2k4	-0.567093	5.160289	-1.833053	0.087365	0.207074	-5.602309	5.041108	4.10842	5.187821	5.774423	5.006144	5.053587	5.114144	5.331332	5.220848	4.712982	5.607995	5.210647	5.768061	5.152334
Tceb2	0.403451	5.96262	1.832822	0.087401	0.207119	-5.648009	6.45654	6.578732	6.357231	5.736122	6.966131	6.088636	5.590126	5.603623	5.740245	5.685747	5.874931	5.53465	5.597584	5.692409
Spire1	-0.199307	7.881402	-1.8326	0.087436	0.207132	-5.638921	8.049207	7.96751	7.835763	8.136505	7.907045	7.862034	7.935606	7.665439	7.812503	7.779919	7.76614	7.863198	7.89221	7.873219
Cxcl10	3.636491	0.425677	1.832564	0.087441	0.207132	-4.43628	-0.634519	0.769921	4.634216	-2.344749	0.606834	0.386712	-0.399109	2.054242	-0.773548	1.064403	0.961591	3.625558	-0.628995	-1.212031
Notch2	-0.394358	5.575335	-1.832129	0.087509	0.207224	-5.626867	6.143878	5.248229	5.622912	5.263289	5.078266	5.262271	5.562442	5.717195	5.740245	5.445717	5.92665	5.738797	5.563249	5.775217
Lmbr1	-0.268505	5.127438	-1.832	0.087529	0.207224	-5.587213	4.846411	4.613433	4.942168	5.302711	4.863759	4.814106	5.34058	5.165139	5.397796	5.064951	5.182653	5.281137	5.507587	5.507551
Asgr2	1.05537	0.867305	1.831984	0.087532	0.207224	-4.838397	1.594228	2.264105	0.604933	-0.093872	2.030509	2.101918	0.952703	0.04768	0.736738	1.28803	0.584127	0.218993	0.111637	0.65743
Tmem128	0.317924	4.259476	1.831424	0.087619	0.20739	-5.438373	3.816137	3.96313	3.989949	3.572936	4.296017	3.775529	4.567614	4.323155	4.534483	4.630291	4.421201	4.698109	4.618316	4.510857
Acta1	-0.325226	3.920747	-1.831278	0.087642	0.207404	-5.474557	4.391979	3.752647	3.826705	3.843191	3.904623	3.653128	4.023054	3.871902	4.316905	3.968034	3.939921	3.761389	3.949625	3.791988
Tmem135	-0.328293	5.595654	-1.831142	0.087663	0.207413	-5.63732	5.728082	5.10405	5.554984	5.985053	5.588172	5.862041	5.538282	5.441205	5.604319	5.279845	5.635724	5.608571	5.790851	5.650719
Rcn3	-0.414565	5.500419	-1.830691	0.087734	0.207522	-5.61913	5.543377	4.506054	4.968516	5.537788	5.424116	5.594433	5.370537	5.877262	5.684033	5.862791	5.674232	5.698154	5.597584	5.703024
Lsr	0.413415	4.664398	1.830627	0.087744	0.207522	-5.557036	4.925421	5.294522	4.78373	4.132904	4.970791	4.6901	4.42995	4.576933	4.824848	4.763521	4.558676	4.66836	4.1699	4.575
Rnf44	-0.29464	6.914574	-1.830241	0.087804	0.207624	-5.656314	7.362217	6.883881	7.435692	7.570187	7.008278	7.360195	6.572791	6.76502	6.665593	6.425471	6.680485	6.660139	6.769551	6.65782
Mef2d	-0.542176	6.856393	-1.829834	0.087868	0.207704	-5.655894	7.228917	6.540827	7.509328	7.94803	6.754372	7.378467	6.177309	6.8637	6.565736	6.287653	6.930134	6.708445	6.894489	6.216276
Mapre3	-0.23545	8.503903	-1.829805	0.087873	0.207704	-5.624693	8.690398	8.354444	8.495711	8.811765	8.708895	8.503412	8.425036	8.398991	8.581839	8.443419	8.479058	8.307001	8.332633	8.526374
Rnf25	0.306596	5.799803	1.829511	0.087919	0.207772	-5.637097	5.772924	6.15719	5.870544	5.442786	5.982031	5.641552	5.928957	5.711724	5.896824	5.945834	5.760279	5.568867	5.592729	5.953435
Psmf1	0.297222	5.617539	1.829323	0.087948	0.207801	-5.644435	5.96967	5.972579	6.140948	5.594136	6.314516	5.878525	5.125	5.340814	5.381255	5.275498	5.706244	5.412558	5.353466	5.213005
Igfbp7	0.511517	7.157215	1.828921	0.088011	0.207909	-5.66049	7.225798	7.461038	7.146078	6.221636	7.599844	6.726356	7.007615	7.380022	7.152303	7.548212	7.04343	7.536335	6.765242	7.398549
Acp6	0.479384	4.577779	1.828747	0.088038	0.207933	-5.493324	4.284188	4.904587	4.511585	3.670505	4.478849	3.901317	4.989008	4.955841	4.881816	4.957801	4.647377	4.647164	4.43154	4.896325
Hba1	0.748555	7.213529	1.8284	0.088093	0.20802	-5.642454	8.354639	8.64051	8.387409	7.344059	9.384908	7.984353	6.017549	6.450463	7.171671	6.47465	6.546208	5.830298	5.792263	6.624706
Tinf2	0.411748	4.389803	1.828246	0.088117	0.208032	-5.506141	4.180631	4.998471	4.617663	3.972106	4.299374	4.03673	4.674929	4.318363	4.473365	4.502215	4.348982	4.331301	4.20426	4.575
Rnf216	-0.317603	5.713159	-1.828067	0.088145	0.208032	-5.645777	5.915219	5.244304	5.879498	5.970226	5.560462	5.80799	5.461136	5.78302	5.544292	5.693914	6.00035	5.812728	5.86521	5.47607
Gpr182	0.791044	1.903104	1.828037	0.08815	0.208032	-5.056707	2.091157	2.046927	2.250588	1.353927	1.947363	3.637716	1.668148	1.80302	1.218642	1.875459	2.014226	2.100868	1.60059	1.473753
Pygm	0.433855	6.766705	1.827503	0.088234	0.20819	-5.662724	7.228397	7.966021	7.160844	6.644865	7.386007	6.962186	6.539707	6.060597	6.529603	6.49909	6.370609	6.465438	6.30291	6.632659
Fuom	-0.596307	1.167801	-1.825775	0.088506	0.208755	-4.922145	1.619793	1.369077	0.20995	1.430203	1.338932	0.982423	1.372662	1.393205	1.663747	1.180545	0.846404	1.252437	1.11438	1.29285
Mtpn	-0.652869	6.961062	-1.825761	0.088509	0.208755	-5.662398	7.407873	6.179975	7.241387	7.794773	6.705869	7.666123	6.676049	6.972166	6.853456	6.265905	7.145937	6.780435	7.380117	6.398042
Cerk	0.223454	6.911622	1.82521	0.088596	0.20892	-5.66747	6.87004	7.173786	7.069028	6.988076	7.35287	7.013339	6.611901	6.905037	6.591131	6.846923	6.940118	7.001141	6.784531	6.627893
Tasp1	-0.320546	3.548533	-1.824569	0.088697	0.209117	-5.442034	3.771212	3.1924	3.715535	3.928941	3.706477	3.491064	3.669732	3.368235	3.35864	3.241039	3.609886	3.496064	3.713602	3.551406
Mtmr6	-0.485159	7.117542	-1.823442	0.088875	0.209496	-5.663692	7.301802	6.598856	7.241773	8.114038	7.219693	7.827359	7.093024	6.694656	6.932927	6.73186	6.973098	6.74667	7.207191	6.974295
Ino80d	-0.717277	2.907708	-1.823215	0.088911	0.20954	-5.307954	2.694686	2.452828	3.177748	3.884445	1.930143	2.809587	2.67414	3.583227	3.01701	2.558244	3.265855	2.703718	3.529386	2.648193
Snrk	-0.364134	4.984186	-1.822411	0.089038	0.209799	-5.613322	5.080769	4.793461	5.245908	5.418025	4.600852	4.902733	4.861747	5.178422	4.961869	4.640468	5.097056	4.803137	5.316642	4.927694
Lsg1	0.209992	4.892746	1.82178	0.089139	0.209994	-5.58836	4.69529	5.048854	5.001701	4.809659	4.901799	4.902733	4.80887	4.965068	4.867041	4.992645	4.840346	4.997502	4.842108	4.877707
Tm9sf1	0.270869	5.313665	1.821628	0.089163	0.210009	-5.603003	5.126074	5.361289	5.132299	4.677679	5.143221	5.039974	5.469678	5.375829	5.491325	5.610089	5.455844	5.559173	5.378131	5.611013
Xpo7	-0.345482	7.024205	-1.820996	0.089263	0.210205	-5.673581	7.083183	6.454265	7.078563	7.449382	6.892471	7.257227	7.097853	7.000862	6.967967	6.819566	7.138816	6.92582	7.204541	6.980541
Atxn7l3b	-0.439682	7.915458	-1.819454	0.089508	0.210741	-5.651679	8.28244	7.441027	8.208093	8.652885	8.06601	8.399876	7.582375	7.677183	7.688154	7.498258	8.001392	7.666184	8.110602	7.548596
Mal2	-0.846448	7.7722	-1.81921	0.089547	0.210792	-5.661171	7.746404	5.928	7.402945	8.721842	7.905669	8.314213	7.641218	7.729106	7.911582	7.644898	7.929812	7.936165	8.131397	7.875567
Pdhx	-0.431729	4.953922	-1.818935	0.089591	0.210841	-5.630219	5.235546	4.462789	5.043022	5.630964	5.180108	5.318124	4.579502	4.9088	5.021793	4.410044	5.099154	4.526331	5.075785	4.914707
Map7d2	-0.586317	6.694152	-1.818859	0.089603	0.210841	-5.675108	6.958484	6.609576	6.784401	7.956115	6.675189	7.407912	6.130942	6.611898	6.593826	5.937604	6.793638	6.072139	6.961934	6.24042
Adra2c	-0.41989	4.629531	-1.818323	0.089689	0.211001	-5.501965	4.361994	3.382986	3.590238	4.038591	4.01491	4.105563	5.180689	4.937208	5.066451	5.366186	4.911322	5.357694	5.078103	5.493644
Rdh11	-0.311978	5.317963	-1.817983	0.089743	0.211087	-5.633386	5.506163	4.812145	4.990724	5.321191	5.370867	5.224944	5.047237	5.361925	5.495173	5.468746	5.368084	5.499602	5.446106	5.578442
Tti2	-0.234478	3.86593	-1.817356	0.089843	0.211223	-5.5278	4.210384	4.155166	4.12993	4.383678	4.031153	3.952041	3.742028	3.734773	3.669138	3.669864	3.755344	3.536147	3.576053	3.685908
Psmd13	0.213571	6.803333	1.817299	0.089853	0.211223	-5.679172	6.690172	6.804126	6.828224	6.546199	6.894137	6.799162	7.065817	6.741049	6.902097	6.861497	6.826303	6.69744	6.743502	6.861055
Cstf1	0.225716	5.03636	1.817292	0.089854	0.211223	-5.604216	4.851815	5.007763	5.17333	4.842596	5.176461	4.928293	4.822272	5.196815	5.021793	5.09979	5.204257	5.290932	4.967428	4.973505
Lztr1	0.269103	6.731256	1.81649	0.089982	0.211483	-5.679218	6.683368	7.022701	6.563912	6.278624	6.814261	6.596865	6.901128	6.737464	6.768555	6.777917	6.738844	6.621072	6.862574	6.885224
Cdc16	0.20156	5.601645	1.816219	0.090025	0.211505	-5.648332	5.406864	5.675942	5.592981	5.480644	5.693981	5.6208	5.775507	5.567732	5.699127	5.541148	5.593185	5.575293	5.524682	5.707549
Lamc2	0.679426	2.06713	1.816105	0.090044	0.211505	-5.210746	2.474162	3.833259	3.084457	2.198954	2.254562	2.889836	1.511505	1.356279	1.142163	2.325762	1.199219	1.738143	1.521045	1.824228
Tnk1	1.255032	-0.089351	1.816102	0.090044	0.211505	-4.598294	0.369528	1.453108	-0.007587	-1.388744	-0.18071	1.136566	0.357848	0.04768	-0.080923	0.534523	-0.991788	-0.070959	-0.628995	0.045082
Tex26	-0.852549	0.146287	-1.815779	0.090096	0.211579	-4.634371	0.539902	-0.366503	-0.575682	-0.093872	0.210178	-1.427823	1.047044	0.705741	1.326257	-0.027758	0.378374	0.403657	0.649393	0.887553
C4a	0.831924	4.042567	1.815679	0.090112	0.211579	-5.371345	3.342675	4.010125	3.240874	2.816229	3.8553	4.501484	4.023054	5.549446	3.383769	4.719397	4.540262	5.848983	3.107385	3.769079
Scarf1	0.664196	3.724541	1.815577	0.090128	0.211579	-5.323899	3.171726	4.091039	3.510504	2.57996	3.654998	2.872384	4.263083	3.804966	4.146293	4.494755	3.750012	4.212646	3.474137	4.24638
Mrgprf	1.283764	0.551301	1.814677	0.090273	0.211876	-4.766364	2.091157	0.632508	1.293837	-1.388744	1.643636	1.960428	-0.067151	0.517885	0.245823	0.750369	0.138317	0.84773	-0.305233	0.65743
Gnpat	-0.259322	6.20521	-1.814302	0.090333	0.211976	-5.677963	6.244887	5.89833	5.80417	6.38626	6.29783	6.227226	6.433462	6.243603	6.255595	6.182462	6.229288	6.16134	6.263737	6.266212
Stat5b	0.263847	3.675059	1.814155	0.090356	0.21199	-5.46596	3.713016	4.069014	4.102999	3.706736	3.89578	3.821723	3.545949	3.517851	3.408468	3.409193	3.434871	3.581545	3.73726	3.629792
LOC246295	-0.193784	6.743624	-1.813787	0.090416	0.212088	-5.68572	6.865372	6.633035	6.778562	6.80324	6.568636	6.582179	6.956114	6.664765	6.823923	6.665304	6.651469	6.677418	6.849065	6.906374
Ola1	-0.51105	6.745989	-1.812328	0.09065	0.21258	-5.687048	6.980236	5.999612	6.864755	7.60865	6.951803	7.313676	6.662142	6.672296	6.678343	6.297333	6.723964	6.437491	6.865932	6.402701
Adra1a	-0.615232	0.69948	-1.812263	0.09066	0.21258	-4.841332	0.643243	0.369671	0.353965	1.526009	0.517419	0.846031	0.952703	0.705741	0.736738	0.413143	0.676952	1.008331	1.407665	0.606649
Mrpl23	0.348735	4.71311	1.81203	0.090698	0.212626	-5.619108	5.204201	5.412913	5.021611	4.583397	5.519311	4.998346	4.36733	4.337436	4.389005	4.661844	4.451079	4.327499	4.24196	4.584872
Dynlt3	-0.618109	7.018235	-1.811719	0.090749	0.212703	-5.67934	7.488578	6.354002	7.254831	8.282751	7.636138	7.823391	6.894006	6.335402	6.782007	6.316501	6.908762	6.351184	7.225603	6.615104
Erf	0.264133	6.094684	1.811539	0.090778	0.212729	-5.683256	6.146079	6.364891	6.77803	6.282058	6.310362	6.458254	5.812911	6.060597	5.838944	6.041898	5.958202	5.981902	5.488545	5.786668
Kctd18	0.336292	5.003133	1.81121	0.090831	0.212812	-5.627476	4.782765	5.551208	5.183007	5.181057	5.12631	5.432691	5.125	4.840254	4.950701	5.011064	4.725516	4.913769	4.463807	4.806368
Gab1	-0.278502	6.436022	-1.811095	0.090849	0.212814	-5.686798	6.432397	6.291652	6.180933	6.684984	6.342446	6.308957	6.366431	6.527221	6.288123	6.452678	6.268037	6.826957	6.538278	6.613497
Casp12	1.111018	-0.52836	1.809646	0.091083	0.213287	-4.577869	-0.634519	-0.687936	0.20995	-0.705589	0.470544	1.327961	-0.672244	0.04768	-0.504027	-0.420466	-1.142289	0.151783	-1.047095	-1.406952
Trmt10a	0.288607	3.155047	1.809559	0.091098	0.213287	-5.350617	3.05476	3.184227	3.420663	3.059778	3.313654	3.438669	3.127125	2.976416	3.130231	3.195748	3.250834	2.931181	3.212403	3.050898
Ahi1	-0.381838	6.758566	-1.809517	0.091104	0.213287	-5.692369	6.529169	6.815415	6.610071	7.341181	6.246583	6.54411	7.1769	6.891389	7.004204	6.593784	6.659311	6.427758	7.087469	6.766632
Fam117b	-0.34788	6.918699	-1.809145	0.091165	0.213386	-5.692957	6.864704	6.471998	7.122806	7.46533	6.791199	6.881068	7.063702	6.811798	6.920817	6.715105	7.12034	6.793586	7.043073	6.809362
Cds2	-0.65116	5.480096	-1.808836	0.091215	0.213457	-5.654317	5.110378	4.625556	5.414195	6.185414	4.390327	5.563503	5.615366	6.051963	5.722297	5.136206	5.71858	5.656333	6.176652	5.39235
Apoo	-0.400103	4.40127	-1.80874	0.09123	0.213457	-5.579523	4.633881	3.649562	4.191945	4.703523	4.705768	4.509973	4.652595	4.171987	4.526983	4.237532	4.549498	4.110154	4.553161	4.496982
Oas2	1.317158	-0.895772	1.808365	0.091291	0.213557	-4.344653	-2.235785	-0.025882	-1.093563	-1.788967	-1.798946	-0.862697	-1.73438	1.067371	-1.536754	1.180545	0.764165	1.433124	-1.638202	-0.505482
Cbx2	-0.565306	1.980434	-1.807972	0.091355	0.213665	-5.078934	2.035255	1.607774	1.089503	2.108996	1.997824	1.450626	2.48486	1.934092	2.583064	2.543858	1.82436	2.118577	1.79348	2.570827
Map3k3	0.330768	6.001235	1.807621	0.091412	0.213693	-5.667652	5.576394	6.317103	5.848419	5.473152	5.670842	5.587765	6.350332	6.185581	6.143586	6.36436	6.132219	6.227096	5.934645	6.23102
Fam89b	0.255404	5.947619	1.807589	0.091417	0.213693	-5.68535	6.138361	6.019271	6.226094	5.892594	6.362643	6.469905	5.699474	5.591758	5.735372	5.832041	5.805955	5.838336	5.883878	5.796614
St7l	-0.431818	4.922937	-1.80757	0.09142	0.213693	-5.621655	4.997891	4.482919	4.424534	5.177373	5.004088	4.724549	4.842144	5.099622	4.710323	4.599322	4.946707	5.062987	5.266712	5.634968
Hdac1	0.372511	3.693294	1.80745	0.091439	0.213697	-5.444619	3.589067	4.117033	3.715535	3.41297	4.128728	3.536092	3.624549	3.622632	3.620637	3.88487	3.56201	3.816678	3.333761	3.864023
Plek2	1.447435	-0.180064	1.806733	0.091556	0.213909	-4.658004	0.243629	2.411416	0.398943	-0.941548	-0.33871	1.585258	-0.399109	0.449407	-0.386063	-0.665882	-1.310338	-0.153565	-0.628995	-0.748265
Cdc42se2	-0.34737	5.786882	-1.806672	0.091566	0.213909	-5.669044	5.873321	4.853963	5.656871	5.574664	5.560462	5.413212	6.026261	5.835949	6.022144	5.877207	6.151556	5.948875	6.25044	6.000908
Rab36	-0.28184	5.199088	-1.8064	0.09161	0.213935	-5.648361	5.471478	5.39	4.926892	5.238754	4.8773	5.106791	5.49291	5.22269	5.259827	5.210951	5.08016	5.1581	5.101079	5.293472
Rnf135	0.382754	2.66062	1.806347	0.091619	0.213935	-5.251297	2.742017	3.108509	3.091325	2.324175	2.682409	2.72461	2.613764	2.634802	2.639794	2.600561	2.761158	2.880186	2.220182	2.474989
Csnk2a1	-0.441552	6.185123	-1.806274	0.091631	0.213935	-5.693448	6.311117	5.532015	6.326275	6.810987	6.068186	6.550297	6.120131	6.218103	6.260125	5.735651	6.325621	6.008265	6.362124	5.984843
Acaca	-0.330147	6.386726	-1.805643	0.091733	0.214119	-5.692203	6.357444	6.234422	6.246233	6.40134	5.726763	5.991955	6.623431	6.640975	6.505955	6.213469	6.401542	6.656356	6.96005	6.473492
Ism1	-0.509027	2.152575	-1.805572	0.091745	0.214119	-5.117905	2.488111	1.425638	1.514261	1.945353	1.578815	2.28453	2.73209	2.121805	2.260183	2.06872	2.592682	2.220493	2.701169	2.570827
Slc38a2	0.279916	7.064242	1.805307	0.091788	0.214161	-5.698008	6.87004	7.118565	7.479538	7.18713	7.321846	7.315291	7.062291	6.678854	6.955451	6.828496	6.584154	6.980106	7.205602	7.323884
Atp6v1b2	-0.362793	8.92999	-1.805133	0.091816	0.214161	-5.652375	9.077169	8.306774	8.982936	9.162679	8.806048	8.940365	8.969767	8.898286	9.054266	8.697473	9.192216	8.718839	9.344244	8.872059
Popdc2	0.977765	1.743693	1.805132	0.091817	0.214161	-4.903706	0.915118	2.854122	1.340699	0.785264	2.030509	1.107027	2.717819	1.318383	2.241976	2.165862	2.066633	1.848377	1.407665	2.124152
Lgi3	-0.570027	7.329052	-1.804769	0.091876	0.214258	-5.699248	7.325302	7.332891	6.68859	7.120588	6.934096	5.66201	7.512271	7.707278	7.654564	7.685192	7.452039	7.934291	7.683905	7.924656
Lias	-0.224822	4.682472	-1.804654	0.091894	0.21426	-5.621974	5.026846	4.804167	4.65754	4.895125	4.805931	4.680104	4.622269	4.527965	4.614504	4.433645	4.692545	4.42323	4.789329	4.642728
Capn7	-0.199124	5.925075	-1.803957	0.092008	0.214484	-5.68515	5.809488	5.740071	5.696819	6.063984	5.77699	5.74226	6.165528	5.936314	6.16186	5.947201	5.921916	5.868738	6.043028	6.102907
Fem1b	-0.502222	4.6305	-1.803657	0.092057	0.214524	-5.628213	4.788412	4.073446	4.964781	5.45653	4.567829	4.870133	4.498338	4.677653	4.461611	3.984107	4.714609	4.368779	5.107901	4.358053
Fam222b	-0.276469	6.270624	-1.803631	0.092061	0.214524	-5.701702	6.750002	6.318037	6.78228	7.021336	6.703337	6.632936	5.86078	5.944112	5.808336	5.904206	6.153577	5.981902	6.149356	5.799443
Foxk2	-0.276892	6.464304	-1.803496	0.092084	0.214534	-5.700121	6.503648	6.058923	6.425395	6.836322	6.454334	6.634231	6.546788	6.385514	6.377364	6.357179	6.538497	6.411688	6.570738	6.417508
Mcc	0.701332	1.328182	1.803134	0.092143	0.214628	-5.011103	1.594228	2.411416	2.700244	1.158502	1.463853	1.606524	1.091998	0.923327	0.884903	1.14286	1.102128	1.152831	1.574559	0.442608
Bpnt1	-0.353592	5.929094	-1.803032	0.092159	0.214628	-5.693442	6.277378	5.428566	6.03985	6.216261	6.057322	6.041593	5.881734	5.869094	5.967785	5.698792	6.189469	5.621048	6.078191	5.666301
Tm9sf2	-0.449791	7.634858	-1.802571	0.092235	0.214762	-5.69426	7.530882	6.726753	7.336579	8.089992	7.492491	7.886418	7.684056	7.478347	7.966327	7.636872	7.472011	7.726252	7.758782	8.110405
Pak3	-0.945586	4.659902	-1.802388	0.092265	0.21479	-5.64299	4.788412	3.302211	5.276359	6.174368	4.173318	5.359422	4.192594	4.980316	4.481148	3.879152	4.918469	4.43741	5.312711	4.032198
Apba3	0.423502	5.033968	1.802182	0.092299	0.214801	-5.607264	4.827336	5.198375	5.060625	4.331596	5.251166	4.478599	5.295647	5.039747	5.199777	5.441843	5.0566	5.018877	4.871804	5.452898
Cyth2	0.204295	6.601577	1.80214	0.092305	0.214801	-5.702294	6.64653	6.742146	6.859233	6.441055	6.76473	6.624491	6.466912	6.554985	6.549172	6.686741	6.606451	6.541255	6.502484	6.452078
Lrfn3	-0.371845	4.681334	-1.801924	0.092341	0.214842	-5.579333	4.464322	3.673115	4.659851	4.667211	4.339058	4.075375	4.955952	4.873301	5.137119	4.957801	4.720073	4.918831	5.00202	5.158958

Pomt1	0.318131	5.700107	1.800776	0.092529	0.215238	-5.662721	5.284322	5.806804	5.233545	5.224545	5.770958	5.485299	5.945892	5.784758	5.846496	5.967554	5.791755	6.007077	5.594349	6.0891
Man1b1	0.302823	7.509904	1.799853	0.092681	0.215549	-5.706722	7.250046	7.540019	7.251769	6.715151	7.350041	6.997327	7.785041	7.611129	7.735287	7.893483	7.661764	7.792907	7.679714	7.883921
Sarm1	-0.375609	5.276019	-1.799584	0.092725	0.215581	-5.655016	5.350716	5.563863	5.067607	5.312821	4.720868	4.492945	5.439557	5.425693	5.444323	5.307785	5.409253	5.385327	5.51445	5.470756
Hars	0.237673	6.369633	1.799551	0.09273	0.215581	-5.705027	6.403249	6.471998	6.846097	6.441055	6.627911	6.691951	6.162897	6.284716	6.354242	6.072757	6.280109	6.075543	6.346826	6.134618
Csnk1g3	-0.401457	6.20689	-1.799346	0.092764	0.215618	-5.704958	6.216891	5.775864	6.272151	6.964757	6.19885	6.512774	6.274358	6.223239	6.126319	5.791517	6.276405	5.931448	6.339116	6.014364
Slc13a3	-0.859407	4.138809	-1.798638	0.09288	0.215811	-5.580415	5.574761	3.24835	3.967729	3.933316	3.137307	5.108656	3.844102	4.055878	3.854323	4.150009	4.196624	4.346409	3.876936	4.746057
Cldn23	1.137738	-0.276366	1.798623	0.092883	0.215811	-4.697284	-0.310971	1.184734	1.921212	-0.02435	0.210178	0.284862	-0.529223	-0.992527	-1.536754	0.348395	-1.142289	0.403657	-1.218554	-0.297732
Hist3h2a	-0.381127	4.504776	-1.797939	0.092996	0.216031	-5.629826	5.024455	4.267739	4.207831	4.954445	4.964461	4.994318	4.253222	4.117861	4.553063	4.345154	4.44779	4.200226	4.519446	4.287263
Rmdn2	-0.40406	2.751769	-1.797752	0.093026	0.216061	-5.322893	3.073383	2.134368	2.568639	3.043681	2.742832	3.150929	2.814871	2.781287	2.834633	2.720538	2.707156	2.606669	2.58862	2.992761
Ccnt1	-0.49988	3.721476	-1.797402	0.093084	0.216122	-5.551059	3.92781	3.557647	4.263615	4.753862	3.333437	4.223812	3.299932	3.832115	3.464507	3.267546	3.414796	3.562264	3.882251	3.43979
Alox12	0.617435	2.249258	1.797319	0.093098	0.216122	-5.033343	1.741237	2.184396	1.430088	1.158502	2.60856	1.993092	2.01015	3.330656	1.743567	3.559779	3.461211	3.71558	1.436856	1.501815
Ngdn	0.415659	5.849251	1.797242	0.09311	0.216122	-5.6844	5.582908	6.116967	5.965176	5.455009	5.940711	5.711921	6.249771	5.860878	6.165487	6.261516	5.546281	5.6257	5.409761	6.025281
Pqlc1	-0.354205	5.277826	-1.796985	0.093153	0.216122	-5.63292	5.221003	4.259983	4.886008	4.780817	4.949583	4.458273	5.701288	5.486762	5.588194	5.818659	5.83142	5.698154	5.512737	5.740308
Rnase1	3.152134	-4.108304	1.796761	0.09319	0.216122	-4.228276	-3.260591	1.310208	-3.260591	-3.260591	-2.352077	-0.548781	-3.260591	-3.260591	-3.260591	-3.260591	-3.260591	-3.260591	-3.260591	-2.228037
Wbscr16	0.292204	4.475478	1.796742	0.093193	0.216122	-5.568376	4.342933	4.769077	4.556942	4.062753	4.409093	4.234096	4.67123	4.552657	4.659784	4.546183	4.591836	4.496167	4.413298	4.421864
Cd1d1	1.129385	1.392848	1.796688	0.093202	0.216122	-4.860694	1.644914	0.583632	1.408255	-0.411255	2.062471	2.386402	1.888013	1.908813	1.489496	2.06872	1.652137	1.950783	0.375586	1.193114
Morn4	-0.247924	5.749812	-1.79668	0.093203	0.216122	-5.700213	6.233547	5.94382	5.839269	5.920408	5.726763	5.805694	5.599886	5.543299	5.685718	5.571605	5.799517	5.432163	5.844936	5.580088
Kif16b	-0.337672	3.886233	-1.79661	0.093215	0.216122	-5.550965	4.280186	3.445867	3.989949	4.263733	3.986037	4.287741	3.632178	3.818604	3.88398	3.566911	3.786926	3.811244	3.974722	3.786295
Cept1	-0.390782	5.134332	-1.796207	0.093281	0.216235	-5.654742	5.216821	4.320894	4.815182	5.312821	5.266683	5.08423	5.203913	4.82682	5.234726	5.06243	5.282044	5.300661	5.471014	5.528164
Tmem192	0.295182	4.675499	1.796033	0.09331	0.216238	-5.618331	4.830077	4.965467	4.811028	4.418424	5.178286	4.704965	4.213088	4.673906	4.589521	4.663938	4.744407	4.727257	4.416965	4.581589
Kctd13	-0.395837	6.943078	-1.795982	0.093319	0.216238	-5.71277	7.365054	6.234422	7.189128	7.183462	7.171505	6.920871	6.665863	6.938211	6.991999	6.820312	7.168094	6.865484	6.942983	6.758659
Rab9b	-0.643519	5.179156	-1.795043	0.093474	0.216556	-5.689	5.304169	4.361326	5.496689	6.474462	5.203588	5.91414	4.802122	4.82682	5.253025	4.630291	5.180673	4.800396	5.409761	4.896325
Wasl	-0.58001	7.433153	-1.794863	0.093504	0.216583	-5.659978	7.816829	6.650938	7.605108	8.471124	7.8083	8.192923	7.576456	7.255827	7.065021	6.947348	7.326922	7.031838	7.311838	7.013199
Qprt	0.804279	1.034634	1.794489	0.093566	0.216685	-4.878014	1.48718	1.79116	1.669168	-0.02435	1.463853	0.915838	0.202532	0.76329	0.736738	0.981432	1.507975	1.252437	1.1849	1.355706
Elmo2	0.264411	7.713012	1.794292	0.093598	0.216719	-5.709703	7.598744	7.843899	7.897307	7.284519	7.581349	7.503826	7.963468	7.78965	7.776982	7.821511	7.825006	7.697083	7.600501	7.80588
RGD1304595	0.599322	4.661165	1.793911	0.093662	0.216824	-5.539768	4.082914	5.208484	4.827573	3.706736	4.258567	3.668376	4.700555	5.312177	4.768722	5.286341	4.978968	5.29872	4.353321	4.872343
Ddx59	0.256655	3.848948	1.79338	0.09375	0.216986	-5.528166	3.932918	4.150978	4.320248	3.857074	3.956574	4.160517	3.577905	3.884925	3.748669	3.753509	3.644778	3.750073	3.725479	3.531125
Zbtb10	-0.86576	1.475061	-1.79324	0.093773	0.216998	-5.144025	2.144973	-0.103798	1.981598	2.656049	1.622351	2.613455	1.754571	1.628188	1.180909	0.799637	1.03357	1.568131	1.316372	1.085968
Aurkc	0.675257	3.745004	1.793013	0.093811	0.217043	-5.373198	2.906846	4.607333	3.515619	2.968937	3.333437	3.061358	4.202878	3.904241	4.034376	4.398098	3.760656	3.732929	3.73726	4.392186
Rgma	0.354986	6.795648	1.792755	0.093854	0.217092	-5.718066	7.158162	7.46146	6.908183	6.458614	7.237739	7.04045	6.3981	6.572195	6.458451	6.657809	6.629136	6.911289	6.473724	6.788155
Nup210	0.351822	4.826447	1.79267	0.093868	0.217092	-5.583162	4.17633	4.963081	4.622412	4.324952	4.685386	4.160517	5.317106	4.937208	5.137119	5.036976	4.889666	5.149513	4.952346	5.275297
Sorbs2	-0.2735	7.583639	-1.79253	0.093891	0.217104	-5.709571	7.681119	7.243782	7.720895	7.868303	7.325555	7.717116	7.74953	7.597843	7.733251	7.345414	7.522915	7.344629	7.657411	7.67143
Cebpb	0.497685	4.405276	1.791666	0.094035	0.217346	-5.514378	3.37306	3.61957	4.773092	4.090441	4.180618	4.339463	4.371894	4.958923	4.71693	4.882627	4.949035	4.727257	4.539769	4.229697
Plekhm1	0.213477	5.946563	1.791634	0.09404	0.217346	-5.709806	5.979578	6.244284	6.396575	6.028641	6.197951	6.030818	5.715723	5.869094	5.710759	5.749822	5.852718	5.897231	5.783768	5.820486
Gpatch2l	-0.364983	4.277697	-1.79147	0.094067	0.217346	-5.592512	4.28818	3.879549	4.337679	4.780817	3.935155	4.53237	4.301868	4.299035	4.321248	3.913127	4.431229	4.216763	4.477919	4.25465
Htr2a	-1.193542	0.70069	-1.791466	0.094068	0.217346	-4.877428	1.178614	-1.685466	0.851376	1.548999	0.091273	1.668504	1.047044	0.301855	0.736738	0.055728	0.721218	0.804622	2.379013	1.355706
Col5a1	0.637143	6.490771	1.791108	0.094128	0.217442	-5.713576	5.983276	7.535609	6.409684	6.132174	6.296151	6.540662	7.213617	5.746029	7.151694	6.543151	5.717214	6.149545	6.184203	7.286563
Mesp2	-0.730411	0.547186	-1.790688	0.094198	0.217562	-4.858021	1.071521	-0.366503	0.56602	1.065902	1.143601	0.117189	0.282279	0.449407	0.837186	0.20947	1.199219	0.930264	0.249639	1.009844
Abcb9	-0.310593	5.984625	-1.790432	0.09424	0.21761	-5.69183	5.741245	5.681759	5.170091	5.573263	5.468031	5.078533	6.491238	6.186896	6.483852	6.476544	6.293914	6.403585	6.176652	6.585907
Ficd	-0.409731	4.309271	-1.79027	0.094267	0.217639	-5.575707	4.414065	3.256169	4.210987	4.405886	4.094356	4.392459	4.506663	4.402272	4.473365	4.332655	4.204441	4.482556	4.680656	4.555051
Cnksr2	-0.727909	7.80943	-1.788791	0.094514	0.218167	-5.706635	7.963818	6.400602	7.994611	8.616449	7.551748	8.307938	7.675312	8.076039	7.863637	7.403337	8.073062	7.671068	8.256906	7.484999
Dtd1	-0.466954	5.748179	-1.78849	0.094565	0.218212	-5.713161	5.88127	4.970229	5.7542	6.470704	5.876985	6.228942	5.708524	5.693335	5.722297	5.542957	5.822558	5.453251	5.854432	5.524749
Loxl3	0.567922	3.463421	1.788457	0.09457	0.218212	-5.414384	3.683012	4.138342	3.070623	2.602113	3.956574	3.622139	3.40164	3.542716	3.197914	3.344264	3.750012	3.542721	3.134365	3.648741
Gdpd2	-0.907488	3.30165	-1.787946	0.094656	0.218368	-5.305678	3.342675	2.833367	0.52603	2.825673	3.068148	2.504391	4.040359	4.095628	3.88398	3.709073	3.574129	3.911153	3.671242	4.461699
Tmco4	0.364499	2.808014	1.787529	0.094726	0.218487	-5.378766	3.239259	3.472958	3.468922	2.77782	3.476716	3.02233	2.186319	2.634802	1.959523	2.934159	2.977336	3.046681	1.963595	2.387449
LOC499339	0.345762	3.429403	1.786825	0.094844	0.218717	-5.467778	3.127854	3.735969	3.929918	3.786283	3.639189	3.94371	3.454237	3.466799	3.207329	3.327565	3.228006	3.055902	3.186856	3.069767
Prune	-0.3172	4.70533	-1.78657	0.094886	0.218774	-5.634292	4.813555	4.208526	4.773092	4.840268	4.553844	4.487224	4.815587	4.943446	4.700356	4.505931	4.892088	4.683311	5.085035	4.633245
Pdgfra	-0.458436	6.281939	-1.786408	0.094914	0.218789	-5.727181	7.122278	6.78871	6.196941	6.455575	5.989324	6.349726	6.047814	5.674709	6.05641	5.809668	6.133244	6.461974	6.548344	6.333144
Alg1	0.329397	3.845966	1.786314	0.094929	0.218789	-5.413579	3.357948	3.684748	3.540923	3.002635	3.428477	3.353184	4.519061	4.12885	4.286137	4.150009	4.128407	4.037544	4.013992	4.327019
Smap2	-0.392366	8.264183	-1.785927	0.094994	0.218897	-5.695436	8.542222	7.750466	8.446515	8.943393	8.526139	8.681181	7.936762	8.246848	8.194711	7.971471	8.330477	8.018056	8.135576	7.979945
Ndfip2	-0.517299	6.684375	-1.785726	0.095028	0.218933	-5.728829	6.71633	5.404143	6.617226	7.089074	6.695715	6.820363	6.72414	6.684451	6.823157	6.550366	6.738171	6.785987	7.022159	6.925923
Atp13a1	0.448164	6.889797	1.785559	0.095056	0.218955	-5.723526	6.344085	7.105099	6.712171	5.972354	6.589352	6.015994	7.441846	7.210641	7.337868	7.36447	6.961611	7.162958	6.818892	7.434026
Atat1	-0.238244	6.026719	-1.78491	0.095165	0.219139	-5.727056	6.52156	5.970204	6.362918	6.47296	6.334287	6.366964	5.712128	5.939438	5.82983	5.855529	5.664347	5.737365	5.816065	5.814904
Mfap3	-0.473676	3.540042	-1.784869	0.095172	0.219139	-5.455221	3.509242	2.667383	3.550922	3.776576	3.083806	3.34688	3.30948	3.972932	3.620637	3.401235	3.872828	3.885525	4.178567	3.524301
Zfp84	-0.253822	5.318714	-1.784354	0.095259	0.219296	-5.693437	5.272282	5.194311	5.379549	5.737374	5.07631	5.364112	5.219191	5.343175	5.334762	5.4065	5.357605	5.261346	5.341939	5.213005
Phactr2	-0.677401	2.730721	-1.783723	0.095365	0.219499	-5.456771	4.180631	2.397344	3.484656	3.288723	3.106978	3.106838	2.415815	1.464337	2.278164	2.165862	3.609886	2.082939	2.713149	2.194616
Cenpq	0.50907	1.159379	1.783295	0.095437	0.219581	-5.074098	1.541696	1.953842	1.687412	1.571628	2.373993	2.230763	0.798508	0.923327	0.245823	0.20947	0.721218	0.460309	1.11438	1.158276
Cited1	-0.594834	0.802521	-1.783294	0.095437	0.219581	-5.072508	1.787089	0.632508	2.025285	2.029493	1.173177	1.402804	0.028455	0.04768	0.17081	0.413143	0.885831	0.760186	-0.040977	0.887553
Erh	0.33451	5.227277	1.782945	0.095496	0.219675	-5.700944	5.262171	5.428566	5.56118	5.585823	6.070152	5.978764	5.162364	4.743479	4.961869	4.785087	5.130266	4.814046	4.850267	4.89103
Sv2c	-0.644487	1.795287	-1.782446	0.095581	0.219826	-5.189904	1.831529	1.279848	2.412888	3.010938	1.556544	1.927009	1.544223	1.596873	2.107651	1.064403	1.102128	1.950783	2.11575	2.105984
Prx	0.53806	2.100901	1.782302	0.095605	0.21984	-5.167639	2.179767	2.801663	2.744481	1.720809	2.553396	1.785004	2.206902	2.007376	2.204856	1.804823	1.996325	2.187314	1.859646	1.73247
RT1-M1-2	1.07094	2.81697	1.781377	0.095761	0.220114	-5.062394	1.108109	3.368632	1.705429	1.615848	2.198134	2.559953	4.118484	2.634802	3.149895	3.90752	3.310002	3.179156	2.902922	3.953102
LOC690344	0.327754	3.883967	1.781274	0.095779	0.220114	-5.560347	4.150251	4.509329	3.929918	3.776576	4.319352	4.398567	3.521509	3.638097	3.503242	3.832569	4.128407	3.822092	3.536146	3.417866
Sumo1	-0.408774	6.189759	-1.781272	0.095779	0.220114	-5.735763	6.565801	5.879471	6.458969	7.093479	6.402989	6.941415	5.979177	5.829227	5.970552	5.8929	6.142429	5.778327	6.033505	5.710558
Meis1	0.538116	1.930685	1.780972	0.09583	0.220189	-5.216912	2.41698	3.382986	2.434653	1.892408	2.472441	2.469996	1.668148	1.318383	1.061401	1.597576	1.605665	1.805289	1.407665	1.931282
Gba	-0.278592	7.478567	-1.779681	0.096049	0.220649	-5.734305	7.447675	6.908275	7.335495	7.62294	7.420328	7.364102	7.393845	7.558254	7.597976	7.44167	7.494901	7.630372	7.799736	7.69327
Rpl23a	0.358733	5.486689	1.779521	0.096076	0.22067	-5.711404	5.817796	6.223497	5.646417	5.21918	5.701613	5.924761	5.426454	5.124541	5.168788	5.64589	5.352337	5.337043	5.016594	5.244487
Anp32a	-0.514376	6.807519	-1.77885	0.09619	0.220889	-5.735414	7.126186	6.505184	6.882184	8.082132	7.146561	7.818558	6.372137	6.644808	6.497346	6.53953	6.613808	6.50805	6.372548	6.210974
Uchl1	-0.352034	9.877659	-1.77833	0.096278	0.22103	-5.651784	10.2726	9.391408	9.998969	10.26106	10.22946	10.04828	9.90643	9.704444	9.988662	9.644861	9.846688	9.448058	9.859843	9.688152
Ufsp2	-0.301373	5.941327	-1.778271	0.096288	0.22103	-5.736229	6.270333	5.860363	5.934953	6.623365	6.29699	6.492213	5.804495	5.724457	5.705785	5.660966	5.778723	5.564028	5.65771	5.830202
Tmem14c	0.23598	5.235207	1.777481	0.096422	0.221297	-5.716562	5.632417	5.63605	5.697944	5.53203	6.076036	5.857613	4.685967	4.856872	4.787674	4.952365	4.962928	4.90359	4.852977	4.901601
Aldh3a2	-0.237382	5.971065	-1.77706	0.096494	0.221419	-5.735027	6.28838	5.814767	6.007541	6.218951	6.020168	6.221207	5.726455	5.746029	5.838944	5.874336	5.982275	5.941432	6.029917	5.909593
Tmem35	-0.610981	6.982268	-1.776539	0.096583	0.22158	-5.743335	6.932579	5.333844	6.566379	7.170549	7.176534	6.681342	6.981721	7.045764	7.441229	7.097109	7.063448	7.107504	7.545028	7.622346
Tcta	0.390924	5.449419	1.776247	0.096633	0.22165	-5.703063	5.661942	5.986746	5.734594	4.886126	5.731741	5.184859	5.333578	5.304929	5.220848	5.528417	5.555479	5.342704	5.505866	5.350525
Usp48	-0.373079	5.309016	-1.776143	0.09665	0.22165	-5.707905	5.19785	5.254098	5.349789	5.95417	4.757939	5.469427	5.300443	5.443407	5.356081	5.171725	5.231293	5.189857	5.481558	5.208755
Ervfrd-1	-0.513147	2.37978	-1.775756	0.096716	0.221759	-5.202255	1.741237	1.679303	2.010869	3.002635	1.745593	2.131561	2.467906	2.619301	2.653634	2.783118	2.707156	2.643831	2.701169	2.745289
Tmem203	-0.402343	3.309589	-1.77547	0.096765	0.221812	-5.362021	3.357948	2.21681	2.924461	2.816229	2.507694	2.998397	3.454237	3.818604	3.627666	3.901891	3.712129	3.869926	3.589113	3.710165
Fv1	0.290995	5.941643	1.775404	0.096777	0.221812	-5.7307	5.898344	6.281153	6.456312	5.77686	5.99658	5.776673	5.758179	5.865813	5.938405	6.020465	5.911209	5.775539	5.843574	5.909593
Isca1	-0.271699	7.456257	-1.775297	0.096795	0.221812	-5.740041	7.769788	7.187651	7.356669	7.411075	7.465104	7.270195	7.496161	7.347464	7.514638	7.449409	7.778958	7.344159	7.637904	7.36743
LOC688613	0.352903	5.606205	1.774987	0.096848	0.221891	-5.723534	5.887861	6.289749	6.082695	5.362331	6.055338	5.472326	5.422059	5.484625	5.559075	5.333104	5.416002	5.49113	5.252416	5.410968
Lrrtm2	-0.538967	5.828682	-1.774868	0.096868	0.221896	-5.730654	5.633986	4.763603	5.937813	6.551901	5.570221	5.934253	5.765135	5.820781	5.874836	5.526589	5.867564	5.847657	6.252494	6.283491
Rad51	1.008187	-0.087136	1.774413	0.096946	0.222031	-4.813298	0.031429	0.426209	0.484899	0.43652	1.895073	2.025031	-1.73438	-0.048075	-0.504027	-0.806258	-0.318799	-0.24119	-0.040977	-0.886923
RGD1359108	-0.23028	5.061635	-1.774161	0.096989	0.222088	-5.67955	4.950824	4.874426	4.71425	5.215592	4.824689	5.014346	5.092185	5.237996	4.922398	5.27332	5.225542	5.321836	5.167891	5.075058
LOC497899	0.682729	0.770013	1.773893	0.097035	0.22215	-4.865785	1.071521	1.702379	1.195284	0.041976	1.018831	0.846031	0.743216	0.645799	0.736738	1.253084	0.584127	0.969826	0.111637	0.799858
Pnck	-0.289663	6.300322	-1.773617	0.097082	0.222155	-5.737746	6.321866	5.94382	5.816656	5.988211	6.081895	5.619493	6.948495	6.133337	6.88019	6.485981	6.223529	6.123471	6.650779	7.007705
Scgb1c1	-2.230357	0.344291	-1.773606	0.097084	0.222155	-4.849233	3.771212	-0.366503	-1.367151	-0.411255	-0.038319	-0.371994	0.118118	0.222004	-0.504027	2.375352	3.518775	0.007173	-0.512812	0.119416
Cit	-0.457817	6.087085	-1.773556	0.097092	0.222155	-5.746213	6.71781	7.060587	6.266095	6.684984	5.860036	5.795313	6.153649	5.56975	5.889532	5.625814	5.549353	5.852956	6.158147	6.049248
Pick1	-0.236708	5.414209	-1.772992	0.097189	0.222288	-5.714159	5.501013	5.489533	5.160327	5.598275	5.214297	5.39957	5.60571	5.378133	5.479717	5.571605	5.388815	5.381725	5.328076	5.329149
Akap11	-0.398988	8.127036	-1.772919	0.097202	0.222288	-5.732645	7.748217	7.350345	8.034797	8.537287	7.520447	8.066979	8.473089	8.218837	8.441544	7.891709	8.150473	8.176238	8.624304	8.550085
Pgr	-0.656487	2.540463	-1.772893	0.097206	0.222288	-5.373925	3.035892	2.01041	2.984714	3.394049	2.542105	2.685174	2.227194	2.634802	2.278164	1.625169	2.930971	2.118577	3.474137	1.910497
Pycr1	0.532895	2.329544	1.772532	0.097268	0.222388	-5.237115	2.32672	2.383133	3.021133	2.061822	2.198134	3.193712	2.450751	2.227821	2.241976	2.127786	2.468337	2.284645	2.236876	1.708589
Scd	-0.357023	11.77303	-1.771931	0.097371	0.222581	-5.59806	12.08153	11.7703	11.43509	11.74057	11.46356	11.54868	12.01063	11.41938	11.84389	11.86933	11.33989	12.01544	12.06563	12.21899
Smek1	0.19153	6.108642	1.771593	0.097429	0.222671	-5.742756	6.016136	6.310548	6.328457	6.162305	6.209595	6.275325	6.118774	6.080544	6.091152	6.004794	5.946597	6.005889	5.958745	6.034915
Ppp1r32	1.3084	2.658419	1.771023	0.097527	0.222853	-5.061032	2.10932	1.480065	2.298852	0.43652	3.488528	2.665044	3.471351	3.770297	3.069578	1.597576	2.556491	3.38339	3.805994	3.410483
Dlk1	1.590241	-0.158182	1.769527	0.097785	0.22338	-4.628916	-0.518419	0.118359	0.818644	-1.075778	1.338932	0.231108	-0.279766	-0.992527	0.007959	0.413143	0.071497	-2.668207	1.11438	1.708589
Mrpl46	0.242668	4.383496	1.769468	0.097795	0.22338	-5.641883	4.506048	4.851385	4.603321	4.433945	4.836291	4.562608	4.223227	4.15058	4.15605	4.341	4.338363	4.123371	4.047497	4.271049
Leprel4	0.409943	4.106577	1.768484	0.097965	0.223725	-5.585333	4.3275	4.532048	4.054626	3.633342	4.677668	4.261168	3.96382	3.749088	4.01306	4.046664	3.744661	4.390807	3.994491	4.195741
Las1l	0.446177	6.97983	1.768375	0.097983	0.223725	-5.754382	6.447645	7.553962	6.902338	6.54834	6.74209	6.577475	7.494592	7.016478	7.327652	7.276393	6.803966	6.863517	6.742041	7.434026
Aco2	-0.225096	9.545163	-1.767711	0.098098	0.223934	-5.684966	9.886299	9.650832	9.571679	9.67066	9.586353	9.406963	9.599043	9.226599	9.552159	9.464721	9.425708	9.393189	9.522465	9.677715
Gpi	-0.247406	9.734172	-1.767631	0.098112	0.223934	-5.676327	10.06319	9.653055	9.890572	9.976809	9.977048	9.573904	9.682474	9.591935	9.801743	9.519739	9.691942	9.408004	9.729427	9.720417
Slc9a1	-0.233126	7.098013	-1.767266	0.098175	0.224001	-5.758576	6.835679	6.679565	6.77803	7.066348	6.934096	6.480742	7.404465	7.206109	7.433228	7.19932	7.136264	7.243345	7.423146	7.563651
Dck	-0.588371	3.007541	-1.767244	0.098179	0.224001	-5.499983	3.247483	2.570375	3.203331	4.301452	3.00376	4.013036	2.967416	2.709903	2.720913	2.438927	3.013384	2.315682	3.107385	2.697558
Ccdc51	-0.307527	3.589528	-1.766712	0.098271	0.224169	-5.520026	3.881004	3.506122	3.4152	3.670505	3.670635	3.282273	3.797364	3.668539	3.464507	3.401235	3.633241	3.656194	3.89808	3.43979
Hook2	0.652	5.23451	1.766214	0.098357	0.22432	-5.673323	4.808006	6.309609	5.071085	4.452353	4.970791	4.786421	5.827938	5.170467	5.738623	5.604901	5.103341	5.025932	4.887747	5.570183
Coro1c	0.223245	7.534485	1.766112	0.098375	0.22432	-5.752389	7.450349	7.497362	7.835763	7.579289	7.843506	7.776958	7.345933	7.553674	7.324411	7.486552	7.569059	7.477939	7.496031	7.25445
Snap47	-0.363033	5.048257	-1.765907	0.09841	0.224359	-5.682872	5.202087	4.236461	4.716472	4.84492	5.044657	4.618632	5.22677	5.307349	5.262088	5.128996	5.530819	4.992709	5.460392	5.152334
Shank3	-0.323633	8.753681	-1.765782	0.098432	0.224366	-5.726232	9.061804	8.360123	8.577773	8.399806	8.283951	8.419535	9.047606	8.78736	8.774661	8.941125	8.653918	9.100989	9.167909	8.978711
Ypel5	-0.305817	6.861177	-1.765343	0.098508	0.224497	-5.761249	6.957231	6.534412	6.866257	7.326703	6.926518	7.016818	6.788713	6.791195	6.888993	6.637628	7.087954	6.683379	6.966945	6.597331
Fam20b	-0.430638	6.938983	-1.764772	0.098607	0.22468	-5.761819	6.924267	6.062272	6.807	7.477739	7.007765	7.20154	6.957633	6.893001	6.910047	6.76712	6.992416	7.053865	7.125505	6.97867
Edem3	-0.395721	5.334824	-1.764254	0.098697	0.224802	-5.710611	5.09451	4.779965	5.289856	5.607886	4.573386	5.18309	5.419857	5.512163	5.636038	5.188006	5.332856	5.650258	5.966272	5.493644
Ap5s1	-0.4139	2.264505	-1.764248	0.098698	0.224802	-5.226953	2.445855	1.679303	2.025285	2.310787	1.96438	2.174914	2.144252	2.207232	2.611707	2.274486	2.802949	2.691938	2.269694	2.431883
Thoc3	-0.265354	5.227153	-1.763525	0.098824	0.22502	-5.728503	5.555019	5.112689	5.358355	5.557761	5.280338	5.408679	5.138455	5.217552	5.112276	5.021485	5.334638	5.164508	5.244183	4.716427
Nup62	0.213907	5.908514	1.763481	0.098831	0.22502	-5.756958	6.101408	6.195307	6.369289	6.225209	6.50682	6.436895	5.321831	5.754923	5.523715	5.682467	5.753634	5.760108	5.587858	5.526457
Allc	-0.599537	0.646378	-1.762935	0.098927	0.225194	-4.971623	1.212615	0.369671	0.785152	1.454757	0.649545	1.221719	1.258992	0.04768	0.683763	0.475109	0.764165	0.804622	0.375586	-0.03329
Dph3	0.295218	4.609952	1.762749	0.098959	0.225225	-5.688268	4.85451	4.788078	4.9741	4.783243	5.374054	5.308423	4.3855	4.166665	4.272747	4.277207	4.540262	4.153748	4.353321	4.373323
Mmp16	-0.448917	4.757376	-1.762501	0.099002	0.225281	-5.679079	4.566458	4.125594	4.59368	5.339438	4.27571	5.0341	4.547582	5.178422	4.913797	4.693564	4.832801	4.744468	5.059455	4.757741
Zfp68	-0.24406	5.393099	-1.761105	0.099246	0.225792	-5.737432	5.45204	5.322718	5.300266	5.817662	5.428721	5.522136	5.499182	5.217552	5.183174	5.337281	5.388815	5.194039	5.422586	5.454694
Ssh3	0.594857	7.089935	1.760467	0.099357	0.226003	-5.764568	6.458313	7.763896	6.823591	6.1492	6.800113	6.202998	7.77775	7.210641	7.619733	7.59954	6.919488	7.164024	6.96005	7.822316
Pnpla3	-0.451769	2.305328	-1.760136	0.099415	0.226092	-5.267255	2.488111	2.643736	2.010869	2.57996	2.240661	1.541762	2.518182	2.14364	2.667343	1.828755	2.346454	2.046399	2.860463	2.685374
Mtss1l	-0.310463	8.809133	-1.758304	0.099736	0.226739	-5.727004	9.280446	8.975077	8.464975	8.816805	8.837705	8.676161	8.879097	8.587034	8.78354	8.906756	8.488493	8.836032	8.796585	9.002691
Chsy1	0.351705	5.000645	1.758294	0.099737	0.226739	-5.70556	5.092229	4.75259	5.449365	4.766176	5.386733	5.500999	4.622269	5.036833	4.666626	4.896847	5.073772	5.390862	4.740412	4.683115
Klhl9	-0.525526	6.42476	-1.758099	0.099772	0.226774	-5.77183	6.546769	5.640537	6.412428	7.345291	6.643183	6.983678	6.229796	6.316303	6.315589	5.93071	6.465558	6.200321	6.65389	6.281469
Gpr85	-0.426433	4.990918	-1.757361	0.099901	0.227026	-5.699954	4.835542	3.953545	4.731936	5.347656	4.829341	5.138162	5.038651	4.921495	5.220848	5.084962	5.196438	5.114641	5.219198	5.291464
Polr2k	-0.345751	3.841652	-1.757026	0.09996	0.227052	-5.665364	4.576285	4.000847	4.269682	4.816781	4.342316	4.791072	3.647317	3.093229	3.662308	3.276274	3.592118	3.16218	3.474137	3.195366
Ahcy	0.320967	5.630332	1.756975	0.099969	0.227052	-5.756882	5.748506	5.633051	6.089571	5.786564	6.23961	6.387073	5.201351	5.503746	5.458191	5.124169	5.478587	5.294831	5.498963	5.412816
Sirt6	0.254186	5.692608	1.756942	0.099975	0.227052	-5.751871	5.772924	6.100775	5.947307	5.534912	5.933168	5.646694	5.670127	5.565712	5.618503	5.793038	5.638612	5.575293	5.378131	5.551845

Dhrs7	-0.461924	3.61148	-1.756865	0.099988	0.227052	-5.640188	4.29216	3.812199	4.162901	4.756333	3.517641	4.765303	2.717819	3.358932	3.188437	3.019813	3.212584	3.35379	3.229186	3.310901
Tmco3	-0.274233	5.227923	-1.7566	0.100035	0.227115	-5.713935	5.159142	4.869338	4.762375	5.224545	5.008197	5.038019	5.501266	5.11075	5.548002	5.517415	5.188577	5.353961	5.240048	5.71206
Plekhg5	-0.389308	8.520925	-1.756476	0.100057	0.227121	-5.752106	8.0225	7.523917	8.113648	8.714871	8.027564	8.24936	8.994766	8.929063	9.034567	8.881415	8.578472	8.63788	8.590606	8.99885
Cbln4	0.831859	3.589243	1.756151	0.100114	0.227208	-5.580977	4.785591	5.05559	4.768815	2.708042	3.649748	4.657358	3.211029	2.539194	2.894141	2.807409	3.776475	2.805622	3.502026	3.237699
Dnajc10	-0.323492	7.23481	-1.755914	0.100155	0.227218	-5.775676	6.966602	6.406762	6.828224	7.361615	7.052217	7.074235	7.387661	7.28129	7.430215	7.281279	7.389122	7.43574	7.756251	7.646901
Klhl20	-0.233111	4.75711	-1.755911	0.100156	0.227218	-5.69972	4.782765	4.735912	4.785848	5.192053	4.656883	4.854663	4.750481	4.655026	4.743058	4.599322	4.686975	4.701051	4.858381	4.655275
Sipa1l2	-0.306921	6.030166	-1.75547	0.100234	0.227325	-5.761144	5.72514	5.56071	6.000262	6.274319	5.714243	5.499579	6.160261	6.175018	6.323186	6.232656	6.088533	6.312278	6.016686	6.364048
Dusp26	0.517599	5.06767	1.755428	0.100241	0.227325	-5.725168	5.733947	6.155101	5.639406	4.452353	5.445483	5.163494	4.874668	4.337436	4.975708	4.79119	4.678581	4.536248	5.096513	5.116484
Habp4	-0.430983	7.066029	-1.755186	0.100284	0.227379	-5.770871	7.432878	6.766985	7.256741	7.861432	7.007765	7.833008	6.803148	6.949102	6.842923	6.650274	7.071485	6.70332	7.136667	6.620713
Lyrm7	-0.476949	4.962992	-1.755062	0.100306	0.227386	-5.748214	5.460908	4.613433	5.205336	6.051963	5.586799	5.711921	4.543542	4.374841	5.086997	4.369831	4.889666	4.300602	4.81457	4.5246
Napepld	-0.498671	3.090215	-1.754902	0.100334	0.227406	-5.442733	3.045357	2.167912	3.049621	3.659984	3.011968	3.193712	2.760215	3.395791	3.333065	2.966878	3.189137	3.074167	3.582598	3.022122
Slc39a4	1.815386	-0.56052	1.754796	0.100353	0.227406	-4.468278	-0.518419	0.632508	0.049949	-2.731185	-0.718553	-1.26448	-0.672244	1.067371	-0.504027	0.534523	0.321999	1.593679	-2.232431	-0.03329
Spop	-0.219696	7.568078	-1.754623	0.100383	0.227433	-5.76831	7.757964	7.618695	7.408798	7.754854	7.488821	7.629583	7.769126	7.419712	7.507987	7.649524	7.390408	7.451546	7.524354	7.590033
Zfp772	0.297799	3.2237	1.754154	0.100466	0.227545	-5.441931	3.127854	3.563961	3.4152	3.035565	3.068148	3.462191	3.148563	3.282262	3.038267	3.276274	3.295436	3.220743	3.286436	3.079111
Txlna	0.239103	5.462364	1.754127	0.10047	0.227545	-5.716053	5.007607	5.45088	5.32524	5.00935	5.07631	5.139986	5.894478	5.629	5.795909	5.700414	5.478587	5.642628	5.660807	5.698484
Neu1	0.312549	5.600225	1.753809	0.100527	0.227629	-5.743384	5.543377	5.778581	6.038962	5.22811	5.570221	5.391936	5.476051	5.573776	5.604319	5.786943	5.494616	5.904905	5.44431	5.599697
Cutc	0.319466	3.821455	1.752968	0.100675	0.227897	-5.550708	3.601952	4.175923	4.036949	3.670505	3.756182	3.862069	4.07995	3.923301	3.87219	3.88487	3.858035	3.502822	3.822677	3.56477
Gripap1	-0.232445	8.029571	-1.752925	0.100683	0.227897	-5.760243	8.087504	7.931037	7.916285	8.352811	7.954925	8.14581	8.178682	7.935643	8.17205	8.061327	7.911124	7.777314	7.930234	8.065281
Fam134a	-0.269966	8.490579	-1.752022	0.100843	0.228174	-5.762559	8.379173	7.682099	8.119119	8.011475	8.094549	7.813994	8.75618	8.823108	8.923638	8.868124	8.706474	8.872692	8.786345	9.035757
Rpl27a	0.557845	5.290744	1.751958	0.100854	0.228174	-5.73898	5.831537	6.560694	5.655713	4.600004	5.466539	5.379636	5.047237	5.207221	5.045607	5.268953	4.904139	5.05151	4.934548	5.158958
Spsb4	-0.486995	1.800519	-1.751911	0.100862	0.228174	-5.100839	1.957182	0.895377	1.669168	1.615848	1.285769	1.302125	2.144252	1.747064	2.147322	1.852296	2.197736	2.027776	2.676906	2.159814
Ncoa6	-0.285212	7.016248	-1.751553	0.100926	0.228249	-5.779507	7.148863	7.015234	7.223526	7.709661	6.976652	7.358237	6.723247	7.034851	6.88313	6.723108	6.894939	6.822236	6.931492	6.79455
Mkrn1	-0.414132	4.981395	-1.75151	0.100933	0.228249	-5.705126	4.894334	3.909605	4.944066	5.193877	4.888487	4.755816	5.018416	5.173124	5.24619	5.029251	5.293084	5.105789	5.385636	4.953324
Chmp2a	0.225941	6.676545	1.751394	0.100954	0.228253	-5.782508	6.935127	7.047693	6.85369	6.658142	7.139555	7.047271	6.483878	6.487219	6.434599	6.640166	6.463931	6.425092	6.308843	6.561949
Minos1	0.299328	4.354421	1.750009	0.1012	0.228722	-5.656537	4.467845	4.710528	4.363439	4.185201	4.695613	4.724549	4.192594	4.026799	4.51566	4.324262	4.363019	4.118979	4.337929	4.012827
Prpf31	0.193851	5.503755	1.749938	0.101212	0.228722	-5.76402	5.713314	5.865483	5.73788	5.686446	5.957806	6.013011	5.241808	5.307349	5.282274	5.290656	5.238925	5.288978	5.219198	5.244487
Ing5	0.33063	3.664661	1.749902	0.101219	0.228722	-5.500101	3.365524	3.953545	3.867281	3.302317	3.346477	3.485336	3.783728	3.93587	3.634661	3.669864	3.776475	3.732929	3.701625	3.874793
Ndufa4	-0.273463	7.369655	-1.749515	0.101287	0.228834	-5.780697	7.583802	7.020982	7.210575	7.504448	7.645071	7.208076	7.527181	7.124736	7.547905	7.325578	7.433034	7.150638	7.409405	7.493322
Pde1a	-0.692833	5.985284	-1.748614	0.101448	0.229153	-5.780435	6.101408	4.838423	5.980978	6.977523	5.912492	6.647758	5.824612	6.169707	5.893911	5.309912	6.230245	5.762926	6.438978	5.731448
Ldb3	-0.372603	3.220416	-1.748109	0.101538	0.229314	-5.471435	3.388015	3.167741	3.111733	3.400384	3.106978	2.72461	3.026505	3.458111	2.94004	3.48645	3.048553	3.433771	3.302384	3.661237
Ltbp3	0.361629	6.255772	1.74795	0.101566	0.229334	-5.777689	6.300288	6.541627	5.959557	5.674735	6.594143	6.294288	6.286495	6.281026	6.212998	6.35409	6.253981	6.370712	5.938477	6.539281
Bckdhb	-0.349794	5.023067	-1.747255	0.10169	0.229571	-5.745259	5.640246	4.841025	4.988886	5.273677	5.223162	5.36255	5.100459	4.568886	4.824848	4.830243	5.341744	4.744468	4.836642	4.795073
Zfp91	-0.223369	7.603427	-1.746949	0.101744	0.229651	-5.775343	7.807474	7.752195	7.815688	8.141713	7.566799	7.867825	7.634586	7.200911	7.519843	7.264926	7.272648	7.330453	7.598888	7.6822
Creld1	-0.234923	7.149042	-1.746154	0.101886	0.229928	-5.790865	6.962861	6.97138	6.754969	7.25904	6.859876	6.917684	7.411687	7.137034	7.403331	7.356775	7.110492	7.209145	7.194962	7.548596
Ppme1	-0.25033	7.61683	-1.745938	0.101925	0.229954	-5.777969	7.946243	7.534002	7.878076	7.923774	7.75245	7.57736	7.286135	7.602778	7.546514	7.509869	7.734769	7.389537	7.605732	7.356426
ST7	-0.283919	4.650693	-1.745871	0.101937	0.229954	-5.688326	4.713215	4.146778	4.496143	4.743933	4.725866	4.401612	4.851979	4.768288	4.818719	4.797268	4.457635	4.588013	4.677602	4.985976
Cyp2j10	0.584197	3.832316	1.74577	0.101955	0.229954	-5.518375	3.350332	4.619507	3.855228	3.315784	3.886882	3.340548	3.471351	4.299035	3.947156	4.150009	3.574129	4.603816	3.495104	3.858608
Tmem132a	-0.27333	7.9484	-1.745546	0.101995	0.230001	-5.781078	7.979948	7.581061	7.768266	7.677096	7.519008	7.357061	8.204499	8.014416	8.288065	8.260318	8.044493	8.185457	7.963839	8.44063
Rad23a	0.264407	5.759104	1.745209	0.102055	0.230031	-5.78186	6.189409	6.408517	6.28194	5.818845	6.262149	6.116699	5.214116	5.510064	5.595383	5.743541	5.55395	5.346466	5.300852	5.315379
Igfbp1	2.14265	-1.653319	1.745191	0.102058	0.230031	-4.45091	-1.642648	0.480615	1.317458	-2.344749	-2.352077	-0.291112	-0.831021	-2.076466	-1.813089	-1.564464	-1.978322	-1.437379	-2.232431	-0.886923
Atp5g2	0.339349	6.895028	1.745149	0.102066	0.230031	-5.791743	7.200606	7.169653	7.138639	6.437979	7.469208	6.853538	6.619598	6.70754	6.8399	6.858594	6.906367	6.84834	6.637481	6.856308
Sncaip	-0.435567	3.315952	-1.744501	0.102182	0.230232	-5.518909	3.652372	3.167741	2.597868	3.606198	3.446756	3.611659	3.562015	3.115498	3.120298	3.28495	3.220315	3.750073	3.169571	3.278825
Unc119	-0.445175	4.258773	-1.744437	0.102194	0.230232	-5.663576	5.03637	4.208526	3.697645	4.074684	4.445908	4.071557	4.311404	4.003106	4.160904	4.369831	4.700858	4.179285	4.208498	4.238063
Kcnk9	-0.579127	2.40644	-1.743794	0.102309	0.230419	-5.24454	1.619793	2.064844	1.981598	3.107017	1.643636	1.648138	2.828218	2.454377	3.289407	2.221154	2.274011	2.489067	3.169571	3.220915
LOC100365935	-0.562643	3.363912	-1.743732	0.10232	0.230419	-5.524555	3.32724	2.963229	3.614277	4.082584	2.472441	3.547133	3.585784	3.720315	3.35864	2.911926	3.42821	3.16218	3.91892	3.160583
Mina	0.31893	2.950196	1.743559	0.102351	0.230419	-5.434312	3.035892	3.08236	3.47943	2.890108	3.114621	3.39656	2.828218	3.000548	2.583064	2.600561	2.823399	2.921125	2.871195	2.879681
Mapk4	-0.407707	6.41679	-1.743547	0.102353	0.230419	-5.794117	7.297848	6.154055	6.471526	6.39976	6.409993	6.726356	6.181215	6.333029	6.134978	6.3867	6.892522	6.138738	6.159242	6.167838
Pcgf3	-0.56233	4.689389	-1.743403	0.102379	0.230434	-5.752279	5.210525	4.294561	5.354078	5.906012	4.715852	5.610311	4.177031	4.696241	4.18493	3.901891	4.570823	4.327499	4.763639	4.003043
Abca2	-0.393606	9.595671	-1.743237	0.102409	0.230458	-5.741044	9.345937	9.523202	8.871162	9.320687	8.930469	8.425156	10.03915	9.962025	10.02289	9.991705	9.698531	10.09709	9.850636	10.26297
Aimp2	0.266569	4.948196	1.74274	0.102498	0.230458	-5.728562	4.922855	4.882026	5.399053	4.956595	5.338601	5.19191	4.884284	4.853564	5.040349	4.744776	4.892088	4.750159	4.786497	4.683115
Lag3	1.080373	-0.482957	1.742686	0.102508	0.230458	-4.456382	-1.642648	-0.813242	-1.093563	-2.040259	-0.424712	-1.612049	-1.73438	2.364261	-1.304935	2.572488	2.066633	2.594065	-0.893866	-1.406952
Pde1c	-0.576657	1.67002	-1.742633	0.102517	0.230458	-5.211284	2.311111	2.167912	1.493674	2.124384	1.78444	1.221719	1.297885	1.71825	1.690848	1.804823	0.631286	1.97042	1.724133	1.951771
Pdcd11	0.367629	6.902476	1.742582	0.102526	0.230458	-5.79234	6.232511	7.172238	6.487232	6.437209	6.665467	6.487206	7.485671	7.177949	7.224788	7.303332	6.79299	7.0295	6.873291	7.278981
Bora	0.622176	3.512037	1.742515	0.102538	0.230458	-5.40815	2.694686	4.155166	3.145118	2.787519	3.320278	2.898483	4.32559	3.832115	3.974983	4.046664	3.448101	3.346294	3.423994	3.917086
Hdgf	0.210249	6.796423	1.742472	0.102546	0.230458	-5.796285	6.843838	6.972567	7.023078	6.887386	7.196476	7.110971	6.522363	6.729364	6.540818	6.646913	6.791693	6.637287	6.78169	6.479679
LOC100365504	0.290121	4.754228	1.742411	0.102557	0.230458	-5.751457	5.108122	5.173818	5.43998	5.368806	5.894848	5.606358	4.197745	4.182573	4.351284	4.328465	4.302392	4.216763	4.191471	4.258767
Lmx1a	-2.872302	-1.175296	-1.742383	0.102562	0.230458	-4.64035	3.145563	-1.685466	-1.705055	-1.575022	-2.352077	-1.823292	-1.009457	-0.81357	-1.813089	0.893396	3.116419	-1.925331	-1.047095	-2.653844
Rnd3	0.375943	4.721854	1.742262	0.102584	0.230464	-5.721191	4.59574	4.604273	5.600221	5.034003	5.201795	5.34684	4.543542	4.424739	4.425765	4.588849	4.454361	4.578447	4.368551	4.399663
Akr7a3	-0.588876	1.674878	-1.742032	0.102625	0.230514	-5.150641	2.488111	1.769471	1.340699	1.454757	1.72577	0.695385	1.938115	1.68885	1.717448	1.704918	1.456531	2.064785	1.902128	2.011549
Erc2	-1.003935	6.151539	-1.741883	0.102652	0.230531	-5.794702	6.48985	4.49288	6.708268	7.701027	6.08287	7.066102	5.548398	6.281026	5.911298	5.226798	6.358399	5.985526	6.737649	5.55687
Nln	0.281016	5.056296	1.741572	0.102708	0.230598	-5.736763	4.965854	5.18205	5.524863	5.068223	5.167305	5.311664	4.707794	5.194202	4.925253	4.979343	4.827749	5.22093	5.089637	4.670808
Hbb	0.636316	5.17865	1.741503	0.10272	0.230598	-5.779635	6.179773	5.882	6.20883	5.211996	7.05867	6.152383	4.61073	4.604749	5.302182	4.505931	4.242906	3.772618	3.91892	4.901601
G2e3	0.452635	3.963354	1.740964	0.102817	0.230773	-5.524285	3.064101	4.228535	3.847136	3.644058	3.644478	3.57437	4.791941	4.038501	4.409542	4.111508	3.812726	3.946285	3.944552	4.534822
Fubp1	-0.363087	6.43724	-1.740708	0.102863	0.230833	-5.798832	6.511351	6.211483	6.454981	7.259911	6.471546	6.952876	6.45728	6.39237	6.421514	6.051304	6.133244	6.083455	6.463122	6.275385
Ppp1r10	-0.238175	6.418856	-1.740387	0.102921	0.23092	-5.79789	6.529169	6.250168	6.4989	6.791238	6.536993	6.403113	6.483878	6.487219	6.410347	6.213469	6.445908	6.227096	6.387577	6.217334
Tbl1xr1	-0.472265	6.851835	-1.740252	0.102946	0.230931	-5.798257	6.874693	6.300186	7.078995	7.833031	6.78283	7.35902	6.75766	6.658143	6.609888	6.544958	6.887067	6.638055	7.006424	6.608666
RGD1304587	-0.271303	4.534788	-1.74006	0.10298	0.230966	-5.688111	4.73969	4.271601	4.106393	4.538155	4.418385	4.672562	4.567614	4.732714	4.71363	4.502215	4.647377	4.594355	4.536402	4.514305
Chordc1	-0.637353	5.650922	-1.739841	0.10302	0.231012	-5.793288	5.877301	5.108376	5.487608	7.07825	6.211378	6.557822	5.454696	5.324179	5.518052	4.888332	5.508891	5.143038	5.827132	5.161159
Gstm6	-0.979222	3.695008	-1.739284	0.10312	0.231194	-5.540393	4.026686	0.725555	3.393138	3.893455	4.248182	3.408717	3.933266	3.966821	4.437813	3.856048	4.069701	3.698019	3.994491	4.24638
Npsr1	-1.140679	-0.461173	-1.738858	0.103197	0.231316	-4.836723	-0.129637	1.184734	-0.095192	1.615848	-0.613814	-1.26448	-0.672244	-1.435005	-1.10526	0.534523	0.001431	0.218993	-1.413173	-0.748265
Chrna4	-0.498604	3.631519	-1.738678	0.10323	0.231316	-5.645059	4.569741	4.432057	4.065129	4.159289	3.706477	3.275651	3.200804	2.400986	3.662308	3.167876	3.099749	3.447848	3.871601	3.917086
Gltpd1	-0.280855	4.765327	-1.738661	0.103233	0.231316	-5.729838	5.083068	4.390036	4.802683	5.019674	5.07631	4.80033	4.696922	4.696241	4.720222	4.623466	4.87014	4.581643	4.797792	4.61409
Coro1a	0.292447	6.827973	1.738233	0.10331	0.231447	-5.801035	6.623809	6.669354	6.837954	6.423279	7.227643	6.526785	6.871621	7.064486	6.99336	7.000991	7.005899	6.964278	6.663183	6.732993
U2af1	0.271168	6.540999	1.737906	0.10337	0.231536	-5.801699	6.822666	7.325943	7.347735	7.383762	7.276578	7.545714	5.913387	6.125128	6.079664	6.18594	6.024839	5.921394	5.755083	5.8845
Polr2e	0.278549	6.019808	1.737577	0.103429	0.231612	-5.794715	6.259192	6.447452	6.196941	5.849261	6.563759	6.118551	5.899229	5.706232	5.844988	6.011344	6.042391	5.875263	5.663896	5.823268
Zfp428	-0.266917	4.947652	-1.737352	0.10347	0.231612	-5.759946	5.488925	4.926801	5.181399	5.352564	5.078266	5.419233	4.633716	4.782275	4.846099	4.663938	4.974403	4.584831	4.81457	4.571694
Angel1	0.264825	4.707702	1.737348	0.103471	0.231612	-5.688958	4.424983	4.871884	4.870123	4.464495	4.499426	4.597545	4.798736	4.677653	4.875924	4.797268	4.573843	4.851593	4.671475	4.993408
Rcan3	0.690185	0.888133	1.737202	0.103497	0.231612	-4.95625	1.033981	1.339943	1.269823	0.386344	1.685287	1.136566	0.563361	0.705741	1.01926	0.799637	0.676952	1.461155	1.347449	-0.116166
Capn5	-0.339757	5.054599	-1.737081	0.103519	0.231612	-5.757188	5.337294	4.984419	5.427823	5.462596	4.854661	4.969911	4.771359	5.132753	4.797058	4.824303	5.03922	5.065272	5.417104	4.728352
Mfsd1	0.316115	5.72529	1.73698	0.103537	0.231612	-5.774827	5.643365	5.528791	5.814582	5.327853	5.874737	5.973658	5.524	5.648222	5.607878	5.738812	5.667178	6.229135	5.803513	5.802266
Abi1	-0.494427	7.572717	-1.736922	0.103548	0.231612	-5.788183	7.866062	6.880091	7.865361	8.462447	7.673483	8.262817	7.536867	7.422506	7.475238	7.064097	7.487712	7.208628	7.559616	7.261648
Cd47	-0.524933	6.042099	-1.736868	0.103558	0.231612	-5.795154	5.695391	5.252144	6.066232	6.895256	5.517871	6.245981	5.754688	6.153655	6.175115	5.982299	6.103242	6.342733	6.275904	6.153536
Sav1	0.293112	4.592497	1.736174	0.103683	0.231841	-5.728456	4.904771	4.774532	4.988886	4.721342	5.176461	5.466522	4.272878	4.327931	4.101553	4.219542	4.502714	4.376159	4.298715	4.229697
L2hgdh	-0.270569	3.381083	-1.736033	0.103709	0.231841	-5.551184	3.633671	3.33211	3.365078	3.716922	3.352952	3.579757	3.577905	3.047633	3.487872	3.258764	3.324422	3.153617	3.495104	3.160583
RGD1564149	1.920324	0.952933	1.735983	0.103718	0.231841	-4.815289	1.401336	0.04804	1.386086	-1.575022	2.630048	2.131561	0.68572	2.364261	0.449965	-0.665882	0.378374	1.738143	2.003164	1.556352
Rnf34	-0.264046	5.888563	-1.735595	0.103788	0.231947	-5.799046	6.072769	5.771779	5.980053	6.47296	6.060293	6.225509	5.832912	5.680322	5.663659	5.632356	5.832682	5.650258	5.937201	5.653849
Hirip3	0.269736	5.943201	1.73551	0.103804	0.231947	-5.800529	6.036449	6.509279	6.231543	6.306717	6.489291	6.537897	5.660833	5.708064	5.700795	5.915425	5.587217	5.546146	5.296877	5.704534
Pdk4	-0.711906	1.767134	-1.734171	0.104047	0.232448	-5.356471	2.620642	3.046741	2.845423	3.107017	1.258434	1.585258	1.258992	1.112357	0.931093	1.481601	1.03357	1.488652	2.220182	1.260365
Rangap1	-0.266981	7.82061	-1.73389	0.104099	0.232519	-5.799185	7.765857	7.266218	7.465401	7.861719	7.776135	7.680608	7.939839	7.953524	8.026069	7.795941	8.133939	7.831336	8.140298	7.858713
Snx12	0.287279	3.385894	1.733625	0.104147	0.232584	-5.515679	3.431974	3.519177	3.599901	3.16769	3.623205	3.568964	3.29032	3.40486	3.383769	3.090803	3.487079	3.292705	3.549571	3.142871
Pald1	0.427528	3.75202	1.733265	0.104212	0.232668	-5.520938	3.536344	4.196386	3.580509	3.035565	3.546179	3.513754	3.706333	4.106787	3.555781	4.2553	4.048142	4.440934	3.220819	3.906629
Desi1	-0.249842	6.634711	-1.733206	0.104223	0.232668	-5.810635	7.07512	6.579511	6.584745	6.688223	6.78103	6.582179	6.584636	6.470039	6.383606	6.538624	6.594602	6.687834	6.809856	6.541817
Maob	-0.346505	6.59183	-1.733052	0.104251	0.232687	-5.808922	6.904902	6.542427	5.849432	6.474462	6.530581	6.453128	6.646217	6.619709	6.615203	6.880942	6.497725	6.972214	6.556679	6.758659
Gtf2f2	0.241298	3.50014	1.731659	0.104505	0.233163	-5.581046	3.556342	3.859159	3.98258	3.732068	3.850732	3.8532	3.261094	3.321106	3.207329	3.463704	3.338699	3.405197	3.318158	2.992761
Cdh3	-2.434463	0.834375	-1.731566	0.104522	0.233163	-5.004136	4.851815	0.185409	-0.067513	-0.325128	-0.516166	-0.548781	0.68572	0.04768	0.17081	2.06872	4.012962	0.804622	0.962073	0.606649
Rab11fip2	-0.721	4.234685	-1.731564	0.104523	0.233163	-5.684233	4.502616	3.774588	4.80059	5.581648	3.574163	5.132675	3.769961	4.464319	3.755102	3.58107	4.464162	3.869926	4.553161	3.551406
Eif4g3	-0.236663	8.746729	-1.731359	0.10456	0.233204	-5.775244	8.598557	8.557126	8.594032	9.042276	8.469715	8.70833	8.823536	8.922762	8.77565	8.669999	8.78482	8.810383	8.884297	8.816408
Pnma2	-0.415246	5.680513	-1.730796	0.104663	0.233386	-5.802046	5.88391	5.212508	5.932087	6.417045	5.874737	5.918397	5.428646	5.407759	5.661948	5.504169	5.652966	5.407164	6.138292	5.528164
Lnpep	-0.816702	1.039007	-1.7307	0.104681	0.233386	-5.007739	0.830061	0.24948	1.143363	1.892408	-0.107758	1.07687	1.21902	1.68885	1.061401	0.750369	1.167576	1.461155	2.133689	0.799858
Oprl1	-0.292723	4.527336	-1.730338	0.104747	0.23349	-5.698699	4.395683	4.421666	4.511585	4.819147	4.279114	4.071557	4.736391	4.234368	4.950701	4.524366	4.272956	4.196062	4.949817	5.088999
Hdac11	-0.340418	7.252205	-1.729384	0.104922	0.23378	-5.812879	7.67808	7.62701	6.97283	7.281949	7.230284	6.729388	7.12582	7.292328	7.117147	7.346451	7.34869	7.229151	7.351181	7.21102
Med12	0.436922	7.06129	1.729351	0.104928	0.23378	-5.815366	6.572363	7.76321	7.201082	6.85316	6.823617	6.808941	7.273385	7.198957	7.272098	7.268759	6.735475	7.083472	6.713997	7.301607
Atp13a5	-0.538578	3.610777	-1.729208	0.104954	0.23378	-5.505055	3.870393	3.091129	2.538805	3.099251	3.224646	2.924117	4.704179	3.386664	4.457672	4.0968	3.250834	3.71558	3.871601	4.461699
St5	0.582155	7.116095	1.729203	0.104955	0.23378	-5.814382	6.713364	7.592686	7.666751	6.354804	6.418508	6.784658	7.706815	7.355673	7.686051	7.400346	7.037971	6.899173	6.722912	7.297611
Hhatl	-0.852387	2.682971	-1.728688	0.105049	0.233947	-5.359568	3.22267	3.151065	2.10888	2.57996	2.16907	0.881356	3.241276	2.555575	3.120298	3.463704	2.428842	3.447848	2.220182	3.25429
Slc9a4	-0.89979	5.404518	-1.728356	0.105111	0.234009	-5.748119	5.117126	2.519311	4.725329	5.588599	5.07631	5.423733	5.814589	6.06918	5.946861	5.511883	5.985681	5.836999	6.237019	5.861672
Atp5b	-0.246717	10.19831	-1.728326	0.105116	0.234009	-5.71659	10.54515	10.03978	10.30471	10.55475	10.47247	10.39978	10.11649	9.861025	10.16775	9.98215	10.15582	9.869102	10.1207	10.18797
Ahsa2	0.337735	5.506518	1.728201	0.105139	0.234016	-5.782092	5.204201	5.993778	5.258166	5.536351	5.752708	5.845367	5.797727	5.354922	5.573708	5.468746	5.313107	5.263337	5.246245	5.517894
N4bp2l1	-0.354928	4.044995	-1.727472	0.105273	0.234271	-5.628585	3.922683	4.03306	4.113157	4.235668	3.403739	3.37813	4.207992	4.161323	4.347031	4.10172	4.223802	4.233112	4.103184	4.262873
LOC679149	1.131858	-0.300582	1.72702	0.105356	0.234413	-4.698207	-0.410969	0.24948	-0.491181	-0.941548	1.533923	0.175273	0.118118	0.137473	-0.773548	0.534523	0.138317	-0.334482	-1.638202	-0.397871
Ksr1	-0.509425	5.066506	-1.726268	0.105494	0.234606	-5.76968	5.110378	4.339413	5.137285	5.704476	4.794082	5.318124	4.980068	5.557602	5.071615	4.744776	5.259708	4.89334	5.490287	4.578298
Trim23	-0.539538	6.075079	-1.726261	0.105496	0.234606	-5.816061	6.165739	5.108376	6.107465	6.920818	6.208702	6.587537	6.168154	5.906292	6.100025	5.603168	6.152567	5.669908	6.357361	6.019226
Slc5a11	-0.794883	2.937471	-1.726128	0.10552	0.234606	-5.352955	2.832262	3.232585	1.363571	2.797152	2.81965	0.915838	3.756062	2.939445	3.689436	3.214035	2.843564	3.284884	3.582598	4.111741
Ptpdc1	0.325149	4.841793	1.726124	0.105521	0.234606	-5.726179	4.54993	5.459372	4.893885	4.682885	4.690509	4.74387	4.818933	4.946555	5.037713	4.693564	4.784073	4.733017	4.977397	4.828697
RGD1305938	-0.505015	5.171631	-1.725342	0.105665	0.234883	-5.798595	5.478482	4.628571	5.469272	6.261328	5.638075	5.719261	4.919005	4.921495	4.846099	4.535316	5.274637	4.792144	5.296877	4.667715
Polr2g	-0.384855	5.732832	-1.725126	0.105705	0.234897	-5.813382	6.182992	5.250188	5.861534	6.311766	6.020168	6.313005	5.67752	5.265641	5.802136	5.301385	5.732163	5.389039	5.697451	5.484884
Rp2	0.340588	2.810393	1.725006	0.105727	0.234897	-5.547189	3.334978	3.42521	3.763618	3.425447	3.601614	4.067729	2.165439	2.121805	2.185932	1.875459	2.544222	2.475388	2.408783	2.194616
Renbp	0.356332	4.120053	1.724897	0.105747	0.234897	-5.640222	4.064414	4.560742	3.910635	3.756964	4.292652	4.304105	3.585784	4.60868	3.761508	4.592349	4.223802	4.671363	3.45286	3.988241
Ufc1	0.242181	5.970368	1.724821	0.105761	0.234897	-5.810912	6.04591	6.115893	6.141776	5.820027	6.230847	6.207355	6.007318	5.742456	5.924202	5.990279	6.068326	5.848983	5.885203	5.581733
Tgm4	0.682935	1.656264	1.7247	0.105784	0.234897	-5.021303	1.071521	2.117298	1.631973	0.74601	1.78444	0.772673	1.913281	2.030999	1.579251	2.342482	1.762161	2.284645	1.574559	2.105984
Ntan1	-0.391628	4.725983	-1.724674	0.105789	0.234897	-5.763217	5.002757	4.407694	4.534442	5.479149	5.283731	5.184859	4.682297	4.477275	4.593117	4.332655	4.615483	4.416088	4.789329	4.425531
Sesn3	-0.36536	5.483312	-1.72452	0.105817	0.234917	-5.803765	6.042369	5.50438	5.873535	5.756017	5.145088	5.603716	5.241808	5.418994	5.277813	5.054841	5.307674	5.645685	5.757977	5.172115
Hnrnph2	-0.472175	7.00548	-1.723956	0.105921	0.235068	-5.818407	7.314089	6.465268	7.351315	8.027422	7.20545	7.771084	6.598333	6.882489	6.758183	6.617162	6.825669	6.720092	6.938525	6.6143
Crtc2	0.313576	6.665442	1.723939	0.105924	0.235068	-5.823998	6.564156	7.199864	7.115666	6.861793	6.907397	6.883792	6.593456	6.527221	6.630156	6.648594	6.219677	6.39089	6.081661	6.707615
Mettl9	0.222368	5.641788	1.723181	0.106065	0.235336	-5.808232	5.919085	5.883263	5.862538	5.634999	6.144827	6.099927	5.368254	5.345532	5.339051	5.575146	5.530819	5.528039	5.402381	5.38295
Slc12a4	0.445572	5.836076	1.722931	0.106111	0.235395	-5.808223	6.287384	6.398838	6.091285	5.17183	5.900385	6.28525	5.463276	5.729681	5.41821	5.875772	6.257742	5.964872	5.123695	5.765122

Usp34	-0.333552	7.234401	-1.722439	0.106202	0.235554	-5.827087	6.70816	6.926151	6.748467	7.52416	6.493694	6.961154	7.880218	7.419153	7.657572	7.29959	7.235932	7.189905	7.628841	7.619548
Mtmr9	-0.46544	4.933048	-1.721289	0.106415	0.235983	-5.765493	5.073848	4.400657	5.103716	5.394419	4.767666	4.795709	4.645073	5.079939	4.902249	4.382013	5.240827	4.95131	5.60564	4.772214
Crim1	-0.293979	6.220159	-1.72099	0.106471	0.236063	-5.810625	6.035262	5.435469	5.36687	5.77686	5.60319	6.03964	6.539707	6.760611	6.661318	6.456524	6.492945	6.641121	6.649221	6.646081
Ankfy1	0.311482	6.968847	1.720329	0.106594	0.236291	-5.829514	6.605531	7.454681	7.144016	6.836906	6.775617	6.756396	7.226904	7.014257	7.030298	7.052076	6.871194	6.898533	6.853131	7.057004
Pitrm1	0.219477	6.491583	1.720181	0.106621	0.236302	-5.827751	6.502789	6.796773	6.759826	6.411567	6.702703	6.446509	6.560848	6.437263	6.458451	6.368448	6.225451	6.396344	6.32942	6.503296
Stx1b	-0.419412	7.906775	-1.720093	0.106638	0.236302	-5.805381	8.221647	7.872474	8.328323	8.698257	7.640777	8.319454	7.58778	7.814535	7.766273	7.340741	7.839197	7.571719	8.215394	7.484999
Eftud1	0.304033	4.613413	1.719302	0.106785	0.236584	-5.732271	4.611755	5.292622	4.930726	4.549599	4.782135	4.540679	4.531353	4.564846	4.54566	4.613168	4.272956	4.379835	4.51603	4.521176
Spata5l1	0.421194	2.558809	1.717964	0.107034	0.237093	-5.326516	2.279379	2.934281	2.908996	2.213416	2.692656	2.116816	2.688847	2.603631	2.583064	2.668442	2.442128	2.680061	2.466543	2.814049
Knstrn	1.811826	-0.686941	1.717758	0.107072	0.237134	-4.695586	-0.129637	0.934883	-1.223881	-1.575022	1.930143	1.627481	-1.73438	-0.654381	-1.536754	-1.838237	-0.411343	0.28321	-1.413173	-0.748265
Emp2	0.408073	2.856081	1.716816	0.107249	0.23748	-5.443018	3.05476	3.382986	2.744481	2.511387	3.167012	3.444586	2.703406	2.665313	2.539002	2.795315	2.782205	2.921125	2.480628	3.012401
Txnrd2	0.34966	4.131203	1.716519	0.107304	0.23756	-5.594105	3.865059	4.095404	3.772191	3.261145	4.169655	3.57437	4.443022	4.393186	4.245589	4.345154	4.579866	4.426789	4.42791	4.330935
Ppp2r5c	-0.306808	7.514426	-1.716113	0.10738	0.237622	-5.818737	8.023099	7.678467	7.804762	8.28318	7.749386	8.151699	7.223751	7.25708	7.152303	6.962976	7.356604	7.149023	7.497763	6.920736
Fuca1	0.421044	6.30256	1.716009	0.107399	0.237622	-5.828511	6.70144	6.432867	6.45365	5.441251	6.543378	6.522596	5.615366	6.640015	5.692438	6.698966	6.750237	6.856938	5.765187	6.142438
Blcap	-0.32811	7.117622	-1.715896	0.107421	0.237622	-5.83623	7.268348	6.449158	7.123643	7.22331	7.205002	6.894638	6.998789	7.092121	7.074698	7.238371	7.509962	7.115242	7.35166	7.113226
Lonp2	-0.308316	5.362667	-1.715871	0.107425	0.237622	-5.802469	5.57149	4.904587	5.295814	5.607886	5.556259	5.36255	5.211572	5.25314	5.362416	5.092395	5.493021	5.365131	5.706469	5.333059
Arl6ip1	-0.294031	7.797236	-1.715838	0.107431	0.237622	-5.828146	7.752557	7.119101	7.396721	7.749902	7.75612	7.55941	7.678079	7.862231	7.985263	8.020998	7.977925	8.041743	8.075069	8.193407
Gpatch4	0.731537	5.458853	1.715457	0.107503	0.237737	-5.808406	5.315947	7.46947	6.404179	5.557761	5.529352	5.283883	5.393164	4.870031	5.213859	5.370269	4.80736	4.901034	5.068809	5.277328
Tcerg1l	-0.445684	2.767665	-1.71452	0.107678	0.238081	-5.549432	3.736577	2.736085	3.818451	3.400384	3.217567	2.634313	2.880403	1.596873	3.110295	1.755735	2.066633	1.890216	3.079891	3.079111
Il17re	1.31572	-0.081172	1.713804	0.107813	0.238294	-4.671555	0.031429	0.04804	0.307539	-1.788967	-0.718553	1.165512	0.798508	-0.26106	0.449965	1.14286	-0.318799	0.460309	-0.040977	-0.505482
Ctsa	0.368373	7.609638	1.713796	0.107814	0.238294	-5.838268	7.444996	7.422038	7.3463	6.659463	7.757341	7.15629	7.493546	8.037196	7.666559	8.098099	7.867785	8.320214	7.417208	7.856337
Tspan17	-0.300242	7.064321	-1.713633	0.107845	0.238314	-5.838864	6.861359	6.544822	6.153307	6.556162	6.725341	6.212565	7.716267	7.236907	7.542801	7.594314	7.241167	7.489468	7.420408	7.618346
Thap1	-0.346416	2.700979	-1.71344	0.107881	0.238314	-5.424811	2.787845	2.397344	2.345554	3.019194	2.962008	2.570814	2.760215	2.964197	2.870631	2.558244	2.739799	2.555581	2.827778	2.697558
Kcnc4	-0.433001	6.458074	-1.713431	0.107883	0.238314	-5.838648	6.299299	5.701936	6.610669	7.139645	6.401428	6.431692	6.291322	6.535503	6.68088	6.491614	6.365389	6.47578	6.245293	6.760838
Pacs1	-0.278665	6.983519	-1.713277	0.107912	0.238334	-5.83967	7.241323	6.63828	7.21883	7.339122	7.020025	7.170639	6.818276	7.028997	6.824688	6.760914	7.077883	6.814787	7.048402	6.779584
Tbxa2r	0.818326	1.285327	1.71261	0.108037	0.238567	-4.961953	1.278303	1.702379	1.195284	-0.166914	1.510943	0.809818	2.078759	1.747064	1.426399	1.481601	0.676952	1.568131	1.377871	2.011549
Mocs1	0.584457	3.618887	1.712309	0.108094	0.238648	-5.516815	3.509242	4.390036	3.393138	2.62393	3.751288	3.038068	3.662299	3.991112	3.748669	4.026112	3.802462	4.16231	3.116435	3.584586
Usb1	0.306985	3.349005	1.711882	0.108174	0.238782	-5.527274	3.188908	3.317238	3.510504	3.182467	3.808949	3.314938	3.280644	3.413873	3.35864	3.377094	3.42821	3.18757	3.502026	3.169358
Dnajb4	-0.461318	5.406815	-1.711671	0.108214	0.2388	-5.816956	5.473232	4.755351	5.481086	6.125498	5.258083	5.854284	5.377362	5.443407	5.403951	5.029251	5.667178	5.183562	5.629542	5.051519
Il1rl1	0.633781	0.797905	1.711627	0.108222	0.2388	-4.949919	0.830061	1.279848	1.143363	0.22441	1.285769	0.772673	0.563361	1.356279	-0.504027	1.322149	1.674823	2.082939	0.249639	-0.116166
Gtpbp2	0.31542	6.393592	1.711462	0.108253	0.238825	-5.832636	5.960945	6.632284	6.200915	5.978717	6.223797	6.0858	6.772407	6.436157	6.655311	6.709477	6.326516	6.52972	6.219277	6.798091
Ap3s1	-0.677106	5.418879	-1.710812	0.108376	0.239051	-5.840303	6.178698	5.028455	5.779933	7.143427	6.041373	7.084691	5.221722	4.76477	4.864068	4.475935	4.942039	4.461895	5.132643	4.786544
Scx	-0.541108	1.070222	-1.710403	0.108453	0.239178	-4.983619	1.108109	0.583632	0.398943	0.932396	0.027932	1.014597	1.443753	1.279465	1.579251	1.51148	1.533025	1.252437	1.28461	1.824228
Atg16l2	0.672358	3.564851	1.710266	0.108479	0.239191	-5.442798	3.263791	4.099756	3.013923	2.077719	3.452798	2.743931	4.263083	3.871902	3.761508	4.224061	3.695584	4.00459	3.556237	4.027379
Rhobtb3	-0.289488	3.895575	-1.710152	0.1085	0.239194	-5.658422	4.031456	4.051149	3.702138	4.125276	3.785203	3.653128	4.362751	3.599118	3.941526	3.890566	3.812726	3.562264	3.91892	4.208569
Dynll1	-0.492341	7.461794	-1.709832	0.108561	0.239284	-5.831639	7.895142	6.849408	7.275329	8.239905	8.086313	8.090518	7.386533	7.116478	7.452655	7.074143	7.419665	6.993364	7.48908	7.105817
Dync1li1	-0.418627	5.684714	-1.709679	0.10859	0.239304	-5.83048	5.722193	5.007763	5.803125	6.345806	5.616707	6.028851	5.554433	5.733485	5.557236	5.434063	5.852718	5.648735	5.901	5.410968
Slc27a3	0.429153	4.612226	1.70957	0.10861	0.239305	-5.666314	4.243653	4.544872	4.427251	3.589663	4.244704	4.116722	5.047237	4.961999	4.959085	4.81235	4.348982	5.053812	4.825648	5.463639
Slc38a3	-0.568104	6.944964	-1.709401	0.108642	0.239332	-5.846187	7.646556	7.15718	6.352951	6.320143	6.373831	5.783691	7.033773	7.159312	7.104606	7.410789	7.463485	7.318052	6.873291	7.24566
Snrpf	0.36748	4.595874	1.709204	0.108679	0.23937	-5.798304	4.917711	5.108376	5.46399	5.410199	5.460554	6.097113	3.876582	4.12885	4.096495	3.984107	4.061116	4.145134	3.866246	3.797658
Pde9a	0.513312	5.896485	1.708431	0.108826	0.239648	-5.83197	5.748506	7.147228	5.938766	5.78172	5.929923	6.103671	5.998491	5.478195	5.977447	5.97159	5.569167	5.477471	5.343866	6.112039
Tmem70	-0.308383	4.768306	-1.708301	0.10885	0.239658	-5.772177	4.824591	4.357697	4.574203	5.204776	5.012294	4.889781	4.915882	4.433629	4.778229	4.703306	4.757751	4.647164	4.974911	4.74018
Pgm5	-0.60876	3.328494	-1.707605	0.108982	0.239905	-5.571464	4.040949	2.309899	2.568639	3.342347	3.601614	3.789543	3.445603	3.492551	3.197914	3.327565	3.116419	3.990233	3.245776	3.294952
Gabarap	0.302105	7.288407	1.707213	0.109057	0.239982	-5.846678	7.528354	7.403676	7.425877	6.929057	7.896001	7.399941	7.022931	7.326736	7.049401	7.311847	7.554606	7.310368	7.034147	6.854948
Fut11	-0.369041	2.749566	-1.707128	0.109073	0.239982	-5.458585	3.163058	2.452828	2.939761	2.908002	2.484288	2.743931	2.450751	2.539194	2.894141	2.558244	2.892776	2.921125	3.107385	2.673087
Rtn4	-0.520451	9.553964	-1.707103	0.109077	0.239982	-5.778901	9.515983	8.435653	9.262197	10.16051	9.759487	9.81542	9.481745	9.545405	9.600746	9.383106	9.510722	9.64401	9.863039	9.779639
Cnih2	-0.514686	9.629972	-1.706962	0.109104	0.239997	-5.782294	9.93158	8.379268	9.038846	9.567484	9.597985	9.940343	9.762538	9.79087	9.82094	9.964031	9.708099	9.86296	9.654815	9.801941
Macf1	-0.32761	9.371207	-1.70651	0.10919	0.240119	-5.804011	8.851142	9.120566	8.562718	9.372553	8.542875	8.896119	10.07326	9.797987	9.846773	9.595583	9.442435	9.589298	9.663524	9.844595
RGD1311188	-0.402787	1.836672	-1.706459	0.1092	0.240119	-5.313194	2.501925	2.248512	1.905712	2.227735	2.240661	1.496914	1.443753	1.71825	1.867508	2.007132	1.697158	1.618784	1.574559	1.608903
Lcn12	0.786382	2.966149	1.706166	0.109255	0.240198	-5.34826	2.460078	4.060109	2.977318	1.892408	2.519256	2.28453	3.545949	3.232189	3.341641	3.566911	2.930971	2.794648	2.713149	3.425211
Ldlrad4	-0.313314	4.187577	-1.705562	0.10937	0.240406	-5.737578	4.816322	3.9244	4.483148	4.449301	4.176973	4.652254	3.957761	3.972932	3.924502	4.18751	4.180861	4.179285	4.033233	3.774841
Tmem209	0.344482	4.069071	1.705061	0.109465	0.240572	-5.667019	4.026686	3.848855	4.116527	3.766803	4.542557	4.41674	3.920861	4.044317	3.799353	4.159476	3.906764	4.277141	3.98464	4.250521
Fbxo22	0.216315	5.478474	1.70467	0.10954	0.240691	-5.824306	5.538358	5.842297	5.887411	5.491069	5.619395	5.572987	5.497094	5.220124	5.341191	5.335194	5.416002	5.259352	5.298866	5.414663
RGD1359127	0.318861	4.342481	1.704137	0.109641	0.24087	-5.715555	4.432216	4.425138	4.764524	4.042646	4.662107	4.333098	4.218166	4.067347	4.044916	4.453021	4.686975	4.398075	4.302685	4.041787
Grp	-0.705266	2.784743	-1.703845	0.109697	0.240948	-5.499855	3.045357	2.452828	2.804219	3.611667	3.452798	1.857753	2.380012	1.532126	3.392049	2.239124	3.116419	2.941167	3.212403	3.195366
Lmln	-0.448358	3.984808	-1.703668	0.109731	0.240978	-5.604275	3.311637	2.943995	3.89502	4.214255	3.00376	3.432728	4.3855	4.411301	4.508062	4.264103	4.324082	4.433879	4.463807	4.303297
Tsc1	-0.270237	5.545214	-1.703331	0.109795	0.241075	-5.817105	5.416013	5.459372	5.410081	5.592754	4.8478	5.228377	5.787514	5.720831	5.775484	5.713327	5.47536	5.651779	5.855784	5.732928
Sorcs3	-0.379734	6.08894	-1.702523	0.109949	0.24137	-5.840971	5.686345	5.292622	5.88346	6.325146	5.375645	5.952015	6.507921	6.278561	6.742079	6.107409	6.343431	5.839671	6.364025	6.571083
Larp1b	-0.284522	5.230521	-1.701909	0.110067	0.241539	-5.834025	5.495845	5.284999	5.481086	6.051963	5.456049	5.74346	5.069886	4.918332	4.986685	4.905312	5.017197	4.948838	5.161347	4.748987
Fam134b	-0.415905	6.512028	-1.701909	0.110067	0.241539	-5.855588	6.307188	5.667172	6.406246	7.032084	6.393597	6.548238	6.439997	6.420591	6.534286	6.473701	6.434321	6.769268	6.848386	6.910959
Letm1	-0.33969	6.644493	-1.701565	0.110132	0.24164	-5.85816	6.569906	6.183055	6.647828	7.122984	6.305362	6.890851	6.584636	6.775547	6.65703	6.485981	6.589387	6.774862	6.885253	6.566108
Mdga1	-0.438764	4.974865	-1.700408	0.110354	0.242081	-5.73596	4.435818	3.551306	3.851187	4.756333	4.6725	4.559885	5.497094	5.601653	5.553549	5.15289	5.478587	5.573689	5.591107	5.429348
Dbp	-0.511989	7.323758	-1.70003	0.110426	0.242196	-5.852713	8.414295	7.430268	6.879211	7.148139	7.581349	7.195851	7.259905	7.378296	7.310827	7.709161	7.442155	7.074994	6.639052	7.079291
Papss1	-0.214149	6.449079	-1.699919	0.110448	0.242199	-5.859618	6.690172	6.300186	6.436898	6.663419	6.575574	6.537897	6.315216	6.361258	6.447568	6.160235	6.350494	6.518092	6.472843	6.475263
Lhfpl5	-0.310118	3.636894	-1.699812	0.110468	0.2422	-5.583704	3.562947	2.894758	3.184186	3.584109	3.563034	3.402651	3.933266	3.606999	3.836231	3.940842	3.70112	3.761389	4.013992	4.060775
Srsf6	0.277434	7.666679	1.699647	0.1105	0.242225	-5.854973	7.334016	7.902053	7.410858	7.529227	7.847237	7.670231	8.087625	7.604255	7.765476	7.657481	7.47686	7.591668	7.556295	7.908041
Dus3l	0.478042	5.612147	1.699167	0.110592	0.242383	-5.813889	5.302196	6.265744	5.692309	4.988479	5.476952	5.057453	5.933595	5.615391	5.908415	5.980965	5.521462	5.641098	5.254467	5.964821
Irf2bp1	-0.252094	5.475499	-1.699037	0.110617	0.242393	-5.824556	5.600671	4.892097	5.30913	5.430459	5.476952	5.36255	5.428646	5.559634	5.580969	5.65261	5.677044	5.6257	5.600812	5.49539
Elovl1	0.441384	6.291346	1.698638	0.110693	0.242473	-5.848136	6.118322	6.677383	6.135975	5.514617	6.530581	5.678421	6.324664	6.459196	6.403196	6.61802	6.194384	6.638055	6.095456	6.711375
LOC294154	-0.242633	8.259248	-1.698596	0.110702	0.242473	-5.841688	8.507548	8.164204	7.933823	8.028189	8.072401	7.934509	8.463498	8.17809	8.337402	8.423421	8.33827	8.436629	8.443756	8.372922
Slc25a4	-0.357725	9.387803	-1.698531	0.110714	0.242473	-5.782679	9.814946	9.18726	9.605427	10.24433	9.757505	10.13722	9.114156	9.100742	9.226495	9.002413	9.18569	8.925992	9.168045	8.961448
Ywhaz	-0.609769	9.85856	-1.697998	0.110817	0.242626	-5.777225	9.926468	8.65998	9.806475	10.62903	9.846604	10.36447	9.7646	9.939772	9.905702	9.544112	10.11842	9.70883	10.23125	9.575898
Prkcb	-0.318188	8.25199	-1.697868	0.110842	0.242626	-5.837353	8.449474	8.152207	8.281239	8.520665	7.79558	8.439856	8.348368	8.290014	8.134914	7.92955	8.348669	8.106126	8.600315	8.136091
C2cd2l	-0.324444	8.544927	-1.697853	0.110844	0.242626	-5.836841	8.422262	7.677012	8.580978	8.586901	8.2027	8.267205	8.609056	8.772109	8.822926	8.709405	8.612655	8.778364	8.673227	8.918503
Otx1	0.457379	2.188261	1.697248	0.110961	0.242837	-5.441218	3.073383	3.151065	3.330668	2.184345	2.732935	3.006419	1.754571	1.198329	1.458292	1.755735	1.978199	1.715048	1.493524	2.17732
Fh	0.201931	5.910388	1.696345	0.111135	0.243173	-5.847992	5.789915	5.88956	5.953916	5.729848	6.167047	5.834142	5.900809	5.845972	5.98841	5.963506	5.99021	5.917605	5.862523	5.938113
Dcx	-0.335929	5.37854	-1.696215	0.11116	0.243184	-5.838459	5.423291	5.637547	5.291347	5.997642	5.062547	5.529113	5.688539	5.045558	5.277813	5.185692	5.368084	4.880423	5.544933	5.405408
Fam175a	0.524995	2.457452	1.695723	0.111255	0.243347	-5.316001	2.01613	3.08236	2.187895	2.029493	2.72297	2.244393	2.979429	2.505864	2.446625	2.923085	2.531849	2.390473	2.079189	2.55752
Pgm2	0.218398	4.814186	1.695408	0.111316	0.243436	-5.813028	5.195727	5.192275	5.331054	5.040101	5.295546	5.515125	4.629911	4.323155	4.582303	4.483493	4.50588	4.496167	4.364758	4.500463
Has2	1.344773	-0.113	1.694953	0.111403	0.243584	-4.8834	0.176305	-0.813242	1.966737	-0.243853	1.600747	1.993092	-0.399109	-0.26106	-0.632503	-1.136114	-0.855515	0.28321	-0.893866	-0.397871
Ttc17	-0.251646	6.359621	-1.694617	0.111468	0.243681	-5.864321	6.240773	6.132979	6.424035	6.652183	5.986203	6.238338	6.350332	6.441676	6.39601	6.420559	6.28288	6.483489	6.407989	6.596518
Card10	0.837465	2.138029	1.69435	0.11152	0.24375	-5.237329	2.41698	3.27168	2.434653	0.823477	2.519256	1.426914	2.629097	1.68885	2.653634	2.007132	1.783193	2.2368	2.041677	2.387449
Fcnb	2.731471	-0.721581	1.693991	0.11159	0.243857	-4.631261	-1.417928	-2.267963	2.466695	-1.575022	1.912714	1.648138	-2.558588	0.04768	-2.155202	0.646479	0.924208	1.045835	-1.638202	-2.653844
Lamb2	0.574941	6.780933	1.692552	0.111868	0.244422	-5.864014	6.830214	7.204418	6.265336	5.273677	6.534859	6.484336	7.12582	6.988095	7.061132	7.257782	7.019811	7.209145	6.513659	7.18042
Pepd	0.353686	5.598209	1.692284	0.11192	0.244476	-5.828925	5.655776	5.36852	5.46399	4.837937	6.021185	5.51653	5.454696	5.791689	5.529356	5.802133	5.825096	6.01419	5.492026	5.634968
Spry4	-0.421082	3.910369	-1.692118	0.111952	0.244476	-5.711306	4.064414	3.279374	3.772191	4.543889	4.368121	4.120423	3.824257	3.492551	3.866259	3.508843	3.892318	3.869926	4.246089	4.003043
Ttc23	0.437981	2.490304	1.692109	0.111954	0.244476	-5.430489	3.064101	3.125682	2.735741	2.029493	3.00376	2.974059	2.659281	2.099634	2.00342	2.529327	2.274011	2.187314	2.203293	2.261799
Med19	0.236819	5.192381	1.691783	0.112017	0.24457	-5.822231	5.19147	5.10405	5.44401	5.138114	5.484345	5.562143	5.133088	5.013306	4.972951	5.233536	5.265325	5.090167	5.057107	5.046765
Tle3	0.255795	6.194144	1.691544	0.112064	0.244577	-5.862888	6.152662	5.888303	6.380546	5.829446	6.440718	6.269504	6.149668	6.347213	6.106329	6.232656	6.324724	6.330438	6.070063	6.217334
Stk36	0.369486	3.686597	1.691521	0.112068	0.244577	-5.572354	3.543041	4.000847	3.495051	2.943136	3.596165	3.3278	3.957761	3.871902	3.761508	3.861859	3.776475	3.980582	3.608485	4.012827
Efcab10	-0.650587	0.45796	-1.691396	0.112093	0.244577	-5.067971	1.741237	0.632508	0.715742	0.896996	1.173177	0.175273	0.282279	-0.150641	0.385075	0.280604	1.03357	-0.434228	0.745852	0.119416
Ptprg	-0.364383	6.813136	-1.691348	0.112102	0.244577	-5.873877	6.705923	6.203417	6.930854	7.15096	6.58248	6.538589	6.976486	6.940552	7.193153	6.275732	6.911749	6.638055	7.312331	7.037965
Stt3a	0.296004	5.921256	1.690614	0.112245	0.244844	-5.861432	5.661942	5.829253	6.171243	6.103344	6.348129	6.369298	5.645836	5.731684	5.741865	5.687384	5.874931	6.056147	5.77093	5.931681
Ankrd46	-0.440741	7.456814	-1.690487	0.112269	0.244853	-5.864603	7.703335	6.671548	7.516043	8.058545	7.755203	7.821403	7.267886	7.324348	7.447202	7.202758	7.606785	7.193043	7.618528	7.217909
Armc1	-0.477744	5.196064	-1.690222	0.112321	0.244921	-5.840956	5.459139	4.305905	5.349789	5.848103	5.388311	5.644125	5.31947	5.099622	5.016447	4.778959	5.261583	4.94388	5.428048	4.945683
Gan	-0.661014	1.978968	-1.689986	0.112367	0.244977	-5.327256	2.279379	2.134368	2.390791	2.557463	0.770579	1.803538	1.986536	2.38274	1.769222	1.875459	1.456531	2.36102	2.62712	1.73247
Vcpip1	-0.68637	5.332601	-1.689777	0.112408	0.245021	-5.849499	5.35833	4.482919	5.633536	6.437209	4.928059	5.791836	5.069886	5.557602	5.25075	4.468337	5.651537	5.200289	5.924376	4.943127
Prdm2	-0.388361	6.855091	-1.689568	0.112448	0.245065	-5.875548	6.966602	6.501899	7.166951	7.627672	6.797741	7.166171	6.577738	6.978255	6.568479	6.574452	6.888281	6.718641	7.036533	6.415665
Ctcf	-0.338181	6.395348	-1.689427	0.112476	0.245081	-5.87664	6.655056	6.004262	6.608875	7.116747	6.648457	6.928279	6.228538	6.248649	6.130036	6.119586	6.305772	6.195115	6.262719	6.101761
Ccdc93	0.302086	4.678825	1.689115	0.112537	0.245126	-5.765108	4.55325	4.459406	4.764524	4.37727	4.886257	4.934612	4.606864	5.042656	4.46554	4.760414	4.709125	5.140873	4.631001	4.233886
Cbfb	0.468855	4.120724	1.68906	0.112547	0.245126	-5.768759	4.114729	4.204491	4.9741	4.797712	5.078266	5.450441	3.562015	3.517851	3.533498	3.545408	3.916315	3.975732	3.713602	3.403062
Rpl31	0.48301	2.726238	1.689007	0.112558	0.245126	-5.430536	2.620642	3.390109	2.682163	2.389316	3.464807	2.434761	2.814871	2.634802	2.858731	2.514649	2.823399	2.346065	2.736815	2.697558
Colgalt1	0.271848	5.989693	1.688477	0.112661	0.245307	-5.849749	5.467963	5.515823	5.687785	5.578859	6.033331	5.942639	6.024813	6.348388	6.079664	6.315443	6.238835	6.523918	5.858483	6.265189
Ptprm	0.261208	5.543446	1.687742	0.112804	0.245547	-5.849456	5.605478	5.94382	5.950144	5.391242	5.523623	5.614253	5.566429	5.364251	5.345461	5.324714	5.289414	5.559173	5.488545	5.67557
Gdi1	-0.277175	10.02874	-1.687702	0.112812	0.245547	-5.788243	10.15383	9.671718	10.02661	10.42047	10.30916	10.03278	9.921783	10.0374	10.05901	9.830118	10.03697	9.812682	10.04955	10.04175
Rnf26	-0.288274	5.575888	-1.687598	0.112833	0.245547	-5.839379	5.652684	4.827969	5.34405	5.331172	5.434839	5.188389	5.730014	5.751372	5.548002	5.812672	5.772163	6.001123	5.901	5.800855
Rbm42	0.288847	6.002208	1.68738	0.112875	0.245595	-5.864149	6.073926	6.016972	6.239293	5.557761	6.350559	5.799936	5.802806	6.105792	6.065498	6.108631	6.154586	5.945159	5.859831	5.974866
Pigq	-0.320145	6.829546	-1.68719	0.112912	0.245631	-5.878706	7.035302	6.364891	6.098972	6.39976	6.770184	6.355233	7.062997	6.93743	7.011612	6.938439	7.05212	7.213272	7.141108	7.246697
Hif1a	0.309338	6.571429	1.686859	0.112977	0.245728	-5.880191	6.573181	6.65464	7.30591	6.989127	7.065093	7.339308	6.257191	6.37286	6.260125	6.039816	6.281033	6.291938	6.401526	6.185247
Top1	-0.322391	4.78018	-1.686298	0.113087	0.24592	-5.816234	4.960861	4.449211	5.018011	5.346016	4.779733	5.074724	4.416758	4.876565	4.632088	4.650573	4.733642	4.591187	4.957391	4.493492
Chrm4	-0.325286	5.377036	-1.686197	0.113107	0.24592	-5.835887	5.268246	5.080024	5.093491	5.567645	5.346735	4.863523	5.554433	5.079939	5.481658	5.569831	5.25407	5.423285	5.640562	6.093717
Mir3072	1.147562	0.678946	1.685903	0.113164	0.246001	-4.789419	-0.217457	1.558038	0.642822	-0.81875	0.371962	-0.371994	1.782265	0.645799	1.326257	1.704918	0.924208	0.460309	0.698429	1.951771
Cdk17	-0.445166	7.214583	-1.68531	0.11328	0.246209	-5.879013	7.174966	6.38107	7.278341	7.893501	7.132044	7.562474	7.26052	7.305101	7.17767	7.030956	7.326026	7.069883	7.392277	7.030159
Nav3	-0.446156	6.438071	-1.684701	0.1134	0.246424	-5.882636	6.546769	6.690426	6.655957	7.042246	5.620738	6.430202	6.323487	6.616785	6.342541	6.184782	6.503281	6.250375	6.76092	6.183083
Cd83	-0.543677	4.364274	-1.68443	0.113453	0.246461	-5.716215	4.243653	2.904741	3.834912	4.380478	4.519714	3.780216	3.939429	4.958923	4.12658	4.885482	5.012752	5.458476	4.698844	4.440107
Cul3	-0.407395	7.455837	-1.684405	0.113458	0.246461	-5.872344	7.573209	6.947434	7.577555	8.29534	7.664074	7.916796	7.293973	7.278209	7.261404	7.05715	7.478473	7.216873	7.584693	7.24566
Adamts15	-0.414478	2.657544	-1.684112	0.113515	0.246542	-5.427293	2.730329	2.747223	2.508341	2.738361	1.912714	2.160607	3.169687	2.471744	3.069578	2.758411	2.718119	2.542521	2.639729	3.294952
Zfp365	-0.447055	7.616316	-1.68379	0.113579	0.246634	-5.865453	8.426343	8.164204	7.903904	8.118367	7.11023	8.125927	7.469791	7.188491	7.09386	7.149713	7.291579	7.484782	7.846374	7.263186
Arhgap8	-1.096667	0.047013	-1.682612	0.11381	0.247093	-4.933891	1.594228	0.185409	1.033553	0.105385	-0.107758	-1.823292	0.625837	0.137473	0.17081	0.055728	0.202178	-0.920914	0.491415	0.706485
Trim2	-0.545334	8.507242	-1.682098	0.113912	0.247229	-5.853955	8.538875	7.315691	8.356566	9.027517	8.297653	8.986846	8.659627	8.647982	8.442043	8.321423	8.700613	8.542299	8.900596	8.368166
Orai2	-0.453972	7.539198	-1.682084	0.113914	0.247229	-5.881731	7.456127	6.506004	7.782127	7.857697	6.945925	7.581729	7.425474	7.707737	7.54094	7.654136	7.735106	7.87757	7.794459	7.692509
Tp53i13	0.693451	3.124357	1.681304	0.114068	0.247518	-5.450776	2.917193	3.967899	3.035447	2.077719	3.217567	2.504391	3.328389	3.349568	2.984524	3.471326	3.288097	3.875144	2.379013	3.537917
Adipor1	0.28203	6.887417	1.680864	0.114155	0.24766	-5.88868	6.878007	6.58573	7.001822	6.387855	7.081759	6.987229	6.719671	6.903438	6.772524	6.960947	7.116719	7.236772	6.905624	6.899138
Trim17	-0.491282	2.240889	-1.680575	0.114212	0.24766	-5.309441	1.895723	1.506527	1.613009	2.523044	1.840817	2.00915	2.760215	2.505864	2.785194	2.911926	1.903329	2.346065	2.689088	2.431883
Aagab	-0.289146	4.250815	-1.680566	0.114214	0.24766	-5.740488	4.150251	4.171795	4.23289	4.529512	3.706477	4.116722	4.371894	4.485848	4.425765	4.324262	4.427894	4.338875	4.23782	4.074854
Cript	-0.413925	5.157287	-1.680441	0.114238	0.24766	-5.867658	5.622966	4.777251	5.397668	6.093604	5.69653	5.90129	5.044381	4.406794	4.855111	4.546183	4.892088	4.758654	5.225485	5.030001
Dmrta2	0.507938	2.026292	1.680355	0.114255	0.24766	-5.343114	2.372556	2.631765	2.812554	1.740915	1.912714	2.781814	1.408645	1.775313	1.636128	2.127786	1.629088	2.300247	1.724133	1.910497

P2rx3	-0.641338	1.56551	-1.680348	0.114257	0.24766	-5.153926	1.541696	0.310827	1.004744	1.679737	1.258434	1.353343	1.782265	2.099634	1.743567	1.51148	1.978199	2.390473	2.508394	1.32462
Terf2ip	-0.316344	5.88864	-1.680188	0.114288	0.247683	-5.882325	6.132823	5.492845	6.030948	6.407642	6.156905	6.133278	5.558443	5.822474	5.712413	5.608362	5.899217	5.858236	6.033505	5.620643
Trappc5	-0.289311	5.236321	-1.680047	0.114316	0.247699	-5.854962	5.502732	4.879497	5.551253	5.617434	5.282035	5.359422	4.915882	5.121793	5.071615	5.097329	5.39566	5.125629	5.334202	5.09592
RGD1566265	-0.716467	3.595385	-1.679908	0.114344	0.247714	-5.72346	3.922683	2.506257	4.068613	4.858787	3.86892	4.220367	3.30948	3.705711	3.510866	2.586593	3.862983	3.16218	4.013992	2.879681
Mblac1	-0.325765	3.156854	-1.67955	0.114414	0.247822	-5.65184	3.718942	2.934281	3.505372	3.847833	3.686105	3.683465	3.094359	2.738882	2.810125	2.819403	3.004456	2.951084	2.860463	2.721621
Fbxo31	-0.205043	6.522361	-1.679156	0.114493	0.247945	-5.886783	6.518165	6.10186	6.355093	6.254351	6.181673	6.083906	6.792973	6.658143	6.733144	6.793961	6.783236	6.717915	6.632759	6.723342
Tmem104	0.288553	4.002973	1.679056	0.114512	0.247945	-5.67098	3.582581	3.859159	3.918379	3.805502	4.067045	4.094316	3.969854	4.161323	4.039656	4.066928	4.039427	4.584831	4.018826	3.932632
Slc16a8	-2.426147	-0.511448	-1.678946	0.114534	0.247947	-4.800543	2.799078	-1.463779	-1.909221	-1.075778	-0.718553	-1.612049	-0.529223	-0.048075	-2.604521	1.755735	3.639021	-0.334482	-0.628995	-0.886923
Pdp2	-0.447016	2.551076	-1.678452	0.114632	0.248031	-5.545963	3.481621	2.21681	2.984714	3.083591	2.661692	3.39656	2.518182	2.471744	2.024877	2.088679	2.083688	2.447635	2.522078	2.011549
Casr	-1.156075	-0.159846	-1.678437	0.114635	0.248031	-4.836761	-0.046857	-0.687936	-1.526232	0.705659	0.027932	-0.457681	0.68572	1.239467	-0.929891	-0.537962	0.263333	1.008331	0.921344	-0.886923
Slc25a1	0.398666	5.604248	1.678267	0.114668	0.248031	-5.852965	5.747057	6.05108	5.479778	4.823867	5.882591	5.300289	5.744165	5.577792	5.757972	5.667616	5.737561	5.567256	5.422586	5.732928
LOC287167	0.687577	2.072581	1.678258	0.11467	0.248031	-5.493202	3.522857	3.27168	3.442311	1.856	3.956574	2.634313	1.297885	0.923327	2.364852	1.064403	1.135223	0.930264	1.316372	1.755962
Htr7	-0.682016	1.620793	-1.678229	0.114676	0.248031	-5.18944	1.831529	-0.186163	1.514261	1.720809	1.705671	1.302125	2.48486	1.830204	2.107651	1.388059	1.347792	1.152831	2.701169	2.372328
Ulk2	-0.242063	6.758474	-1.677466	0.114827	0.248314	-5.895131	6.940844	6.617185	6.883174	7.064355	6.604357	6.937749	6.876447	6.576215	6.59203	6.409691	6.631312	6.720816	6.955014	6.824024
Zfp148	-0.466325	5.620627	-1.676298	0.115059	0.248761	-5.88353	5.788507	5.106215	5.928256	6.511508	5.620738	6.075351	5.349864	5.78302	5.448299	5.136206	5.664347	5.350218	5.851726	5.10738
Kdm5b	-0.235842	6.458746	-1.676217	0.115075	0.248761	-5.892848	6.293354	6.392644	6.235424	6.711972	6.087731	6.356018	6.548805	6.694656	6.583921	6.494422	6.538497	6.372558	6.606504	6.523969
Fam214a	0.299528	6.230682	1.675969	0.115125	0.248822	-5.888652	5.931899	6.855211	6.227653	6.246461	6.133587	6.344198	6.27801	6.241073	6.233878	6.260416	6.159621	6.049238	6.019101	6.270296
Iah1	0.375127	4.773315	1.675493	0.115219	0.248982	-5.786086	4.82184	5.119134	4.915329	4.086518	4.923716	4.323498	4.61073	4.937208	4.775067	5.036976	5.052274	5.032953	4.435161	4.814782
Akip1	0.402536	3.204477	1.675221	0.115273	0.249054	-5.565633	3.543041	3.563961	3.484656	2.62393	3.551819	3.248852	3.419386	3.070611	3.23521	3.241039	3.20481	3.083214	2.676906	3.088394
Rftn1	0.430074	2.902199	1.67486	0.115345	0.249165	-5.441774	2.263247	2.466372	2.795835	2.687469	3.099296	3.255598	3.137884	2.822481	2.905753	2.807409	3.074378	2.703718	3.45286	3.372991
Ap2m1	-0.276501	9.633052	-1.674574	0.115402	0.249243	-5.816498	10.02633	9.450528	9.83367	10.08786	9.969693	9.872974	9.320218	9.465575	9.554696	9.387267	9.662094	9.32189	9.61855	9.293385
Vsig2	-0.557573	1.905336	-1.673934	0.11553	0.249438	-5.328029	1.874641	1.425638	1.363571	2.666599	1.664612	2.526873	2.12275	1.883083	2.347926	1.986005	1.903329	1.461155	1.651284	2.228598
C2cd2	-0.276418	3.142911	-1.673903	0.115536	0.249438	-5.573827	3.188908	3.08236	3.104962	3.437817	2.892584	3.099357	3.083269	2.988532	3.333065	2.966878	3.031075	3.212521	3.495104	3.262515
Coq10b	0.380009	3.991807	1.67381	0.115555	0.249438	-5.766762	4.050381	4.095404	4.923048	4.48252	4.606283	4.951331	3.706333	3.301815	3.577725	3.636356	3.822919	3.489274	3.855477	3.489688
Dcaf5	-0.196323	6.522441	-1.673689	0.115579	0.249445	-5.898861	6.610322	6.425085	6.571913	6.716421	6.336739	6.535127	6.583652	6.542711	6.444585	6.391729	6.619666	6.450659	6.597646	6.50503
MGC125239	-0.248831	5.027804	-1.673019	0.115712	0.249688	-5.808306	4.563167	4.459406	4.645925	5.056238	4.713338	4.443576	5.439557	5.237996	5.485533	5.305655	5.250298	5.127817	5.385636	5.325228
Aven	0.329138	3.376828	1.672899	0.115736	0.249696	-5.596058	3.388015	3.678943	3.387569	3.075697	3.886882	3.275651	3.280644	3.583227	3.35864	3.352542	3.280721	3.212521	3.445698	3.220915
Strn4	-0.211578	8.512055	-1.672766	0.115763	0.249708	-5.880974	8.310921	8.092047	8.13104	8.160961	8.014858	7.861758	8.954063	8.80095	8.953667	8.881415	8.802794	8.655236	8.700939	8.852611
Cand1	-0.417405	6.86256	-1.672444	0.115827	0.249802	-5.902193	6.779367	6.100775	6.819461	7.473615	6.830594	7.082796	6.865161	6.946775	6.851205	6.557544	6.979946	6.765057	7.252019	6.785304
Slc7a11	-0.414011	6.725236	-1.672069	0.115902	0.249919	-5.902883	7.051244	6.045451	6.472841	7.033613	6.759865	7.234783	6.745401	6.676983	6.25105	6.782519	6.645739	6.987353	6.679303	6.802328
Tsta3	0.437539	5.282002	1.671767	0.115963	0.250004	-5.827254	5.128302	5.690441	5.294326	4.464495	5.26325	4.667512	5.59599	5.309765	5.586392	5.623833	5.32032	5.381725	5.028628	5.634968
Gimap5	0.562149	1.474134	1.67151	0.116014	0.25007	-5.129166	1.48718	1.607774	1.574317	0.664146	1.685287	1.519512	1.668148	1.356279	1.867508	2.221154	1.260496	1.082389	1.747621	1.473753
Tmem47	-0.430416	6.525518	-1.671367	0.116043	0.250087	-5.901554	7.399134	6.39972	6.898917	7.422402	7.080296	7.543993	6.071857	5.798587	5.817587	6.111073	5.746959	6.246353	6.367819	6.470833
Vars2	0.431082	6.111219	1.670755	0.116165	0.250306	-5.885242	5.8546	6.675198	6.30723	5.529143	5.841734	5.66201	6.401452	6.257438	6.401147	6.333329	5.874931	6.013007	5.879898	6.5494
Wwp2	0.210362	4.724069	1.670457	0.116225	0.25039	-5.79804	4.643261	4.610386	4.78373	4.427757	4.750601	4.81867	4.851979	4.76477	4.707008	4.85376	4.741723	4.772703	4.649823	4.820364
Nrip3	-0.77481	9.075768	-1.670079	0.116301	0.250508	-5.849713	9.310316	7.256506	8.92897	9.688968	9.191986	9.538382	9.277528	9.25085	8.987872	8.728811	9.410054	8.961995	9.680498	8.851249
Tdrd1	1.140565	0.037812	1.669628	0.116391	0.250659	-4.769266	-1.22354	1.702379	0.442561	-0.243853	-0.424712	-0.291112	-0.399109	0.645799	0.736738	0.750369	0.138317	-0.070959	0.375586	0.045082
Tef	-0.223019	8.162895	-1.669502	0.116417	0.250668	-5.886991	8.327259	7.874063	8.175544	8.064541	7.889334	7.958742	8.275641	8.222048	8.143215	8.199668	8.383087	8.210702	8.400884	8.161327
Cdc42ep2	-0.414559	4.716045	-1.669314	0.116454	0.250704	-5.79541	4.85451	4.294561	4.089343	4.543889	4.962345	3.798811	4.674929	4.747049	4.887683	5.011064	4.972116	5.175124	4.934548	5.141226
Akr1c14	0.931731	0.95515	1.668569	0.116604	0.250977	-5.243806	2.694686	1.972946	2.356997	0.280432	1.622351	3.268997	-0.169545	-0.26106	0.571595	0.475109	0.001431	0.666985	0.434663	0.442608
Dync1li2	-0.211445	8.231659	-1.668477	0.116623	0.250977	-5.882673	8.210648	8.197069	8.082968	8.600376	8.134799	8.355621	8.334113	8.1149	8.265709	8.117961	8.097669	8.104183	8.325091	8.307355
Rph3al	-0.482175	3.029698	-1.668051	0.116708	0.251116	-5.57091	3.357948	3.479652	3.138503	3.302317	2.436306	2.386402	2.644268	2.976416	2.611707	3.440594	3.678847	3.118845	2.838755	3.203933
Ywhaq	-0.386069	7.947484	-1.667471	0.116825	0.251322	-5.880308	8.36507	7.613378	8.206315	8.814436	8.382989	8.612712	7.628877	7.513361	7.699457	7.402341	7.763496	7.590479	8.036257	7.642181
Wdr37	-0.252664	6.03696	-1.667295	0.11686	0.251354	-5.901334	5.958442	5.790744	6.09812	6.51297	5.950352	6.096173	6.156298	5.920591	6.133744	5.958092	6.043481	5.772746	6.085122	6.064616
Bysl	0.247861	4.413843	1.666311	0.117059	0.251735	-5.799705	4.424983	4.554415	4.678213	4.541025	4.972895	4.717236	4.129307	4.239446	4.338488	4.233056	4.373459	4.092341	4.438773	4.134325
Ube2e2	-0.33605	5.937162	-1.665969	0.117128	0.251839	-5.90787	6.336395	5.687553	6.27517	6.715151	6.2801	6.515587	5.584238	5.680322	5.749941	5.642519	5.81747	5.463681	5.91533	5.483125
March1	-0.635728	1.798396	-1.665524	0.117218	0.251987	-5.274603	1.895723	0.854759	1.981598	2.169587	1.082564	1.668504	1.408645	2.505864	1.636128	1.597576	2.627988	1.848377	2.423441	1.951771
Vps18	-0.263196	5.256703	-1.665409	0.117241	0.251991	-5.868508	5.32763	4.838423	5.15542	5.514617	5.349976	5.287179	5.143803	5.328951	5.183174	5.171725	5.563099	5.222978	5.363002	5.185153
RGD1309104	-0.274788	5.583955	-1.665175	0.117288	0.252048	-5.902481	6.095726	5.659822	5.683247	6.213565	6.017113	6.160494	5.246787	5.398708	5.202134	5.128996	5.407561	5.355829	5.549951	5.088999
Tmprss13	1.038725	0.824077	1.664909	0.117342	0.252118	-5.08841	1.108109	2.354284	2.744481	0.386344	1.556544	0.284862	0.903114	0.137473	1.218642	0.055728	0.321999	0.460309	0.598632	0.383504
Sh2d3c	0.346924	5.39441	1.664788	0.117366	0.252126	-5.874687	5.346894	6.019271	5.980053	5.29763	5.60319	5.074724	5.211572	5.290321	5.227804	5.398527	5.192513	5.461948	5.204421	5.250702
Rpl36	1.10556	0.339395	1.664198	0.117486	0.252287	-4.884043	0.915118	1.532513	0.56602	-1.223791	1.082564	0.056669	0.429655	-0.150641	0.571595	0.847278	0.378374	0.760186	-0.628995	1.009844
Commd4	0.492317	6.601656	1.664185	0.117488	0.252287	-5.906673	6.38193	7.298757	6.424035	5.773203	6.549029	6.003023	7.052374	6.570181	6.828506	6.991746	6.634209	6.619518	6.327473	6.986139
Exnef	0.62521	0.969497	1.664106	0.117504	0.252287	-4.999709	0.485316	1.21714	0.851376	0.386344	1.622351	0.481842	1.297885	1.596873	1.01926	1.064403	1.03357	1.284167	1.252133	0.799858
Uqcr11	-0.311387	7.299827	-1.663079	0.117712	0.252649	-5.915593	7.620278	6.956461	6.881689	6.84215	7.127802	6.717831	7.744699	7.260206	7.752672	7.523222	7.457773	7.344629	7.482533	7.49594
Adamts3	-0.484781	5.24372	-1.663067	0.117715	0.252649	-5.824496	4.435818	3.702025	4.617663	5.397589	4.559454	4.85244	5.765135	6.034539	5.848001	5.466841	5.682652	5.54123	5.886526	5.669397
Nrarp	-0.301059	4.294043	-1.662521	0.117826	0.252841	-5.784081	4.85451	4.208526	4.109779	4.102146	4.195107	4.164107	4.750481	3.871902	4.585917	4.18751	4.039427	4.16231	4.43154	4.534822
RGD1562407	-0.417422	6.397418	-1.661629	0.118007	0.253184	-5.917502	6.970333	6.381964	6.798108	7.563147	6.859876	7.354707	5.86726	6.086193	5.838944	5.533887	6.130169	6.031821	6.327473	5.839853
Pak4	0.266363	4.679726	1.661178	0.118098	0.253304	-5.818518	4.799641	5.080024	4.919194	4.367604	4.753051	4.629061	4.52317	4.747049	4.453722	4.806336	4.79447	4.906142	4.250206	4.548339
Sos2	-0.297696	6.091657	-1.661146	0.118105	0.253304	-5.915188	6.211647	6.010053	6.213559	6.829883	6.112751	6.559186	5.994057	6.114111	5.960844	5.829078	5.761604	5.846329	5.992314	5.871116
Dnaaf1	0.806622	1.023803	1.660821	0.118171	0.253401	-5.028519	1.48718	0.725555	1.293837	-0.411255	1.48759	1.402804	0.903114	1.658838	1.01926	0.893396	0.631286	1.488652	1.493524	1.085968
Surf6	0.238461	3.571014	1.660593	0.118217	0.253455	-5.66167	3.576066	3.730367	3.763618	3.431645	3.623205	3.830786	3.454237	3.698353	3.341641	3.545408	3.684447	3.440827	3.495104	3.510555
Stk38	0.41566	6.0353	1.660014	0.118335	0.253663	-5.897297	5.831537	6.51173	5.747694	5.372033	6.008935	5.794155	6.469044	6.006512	6.279223	6.482214	6.020417	6.082327	5.649941	6.263141
Dusp18	-0.229851	6.086035	-1.659834	0.118372	0.253696	-5.917438	6.460083	6.073378	6.163932	6.571681	6.416191	6.491499	5.673828	6.041824	5.848001	5.855891	5.899217	6.013007	5.957486	5.757868
Ccdc32	-0.304232	6.131279	-1.659317	0.118477	0.253877	-5.914766	6.291366	5.622504	6.263817	6.379061	6.007909	6.226368	6.162897	6.171036	6.195364	5.972933	6.338999	5.931448	6.371603	5.925219
Clec1a	-0.533176	2.470453	-1.659165	0.118508	0.253897	-5.489415	2.213741	2.532247	2.508341	3.561676	2.062471	2.469996	1.836107	2.603631	2.364852	2.239124	2.481265	2.772447	2.713149	2.516844
Depdc1b	1.160539	-0.723581	1.658838	0.118575	0.253995	-4.706065	-1.052261	-0.103798	-1.093563	-1.223791	1.082564	-0.214525	-1.450335	0.301855	-0.27702	-0.027758	-0.510232	0.218993	-1.047095	-1.899685
Slc7a3	0.613551	2.21135	1.658215	0.118702	0.254211	-5.307377	2.196854	3.06466	2.225837	1.273392	1.85913	2.160607	2.48486	1.49863	2.810125	2.239124	1.762161	1.890216	2.676906	3.178079
Ofd1	0.330858	4.527133	1.658136	0.118718	0.254211	-5.799993	4.064414	4.946264	4.79429	4.651364	4.380852	4.670039	4.785113	4.592894	4.571407	4.472141	4.366508	4.458423	4.208498	4.486487
LOC100360552	-1.196426	2.137898	-1.658032	0.118739	0.254212	-5.221141	2.947796	-1.463779	0.642822	1.128291	1.912714	2.203108	2.644268	2.80888	3.09008	2.911926	3.228006	2.859272	2.652228	3.041369
Psmd2	0.188776	7.833387	1.657882	0.11877	0.254232	-5.912838	7.622255	7.726746	7.890925	7.754854	7.967904	7.923695	7.833414	7.893174	7.864753	7.784897	7.937748	7.765771	7.893527	7.814646
Mmachc	-0.354035	3.466311	-1.656859	0.118979	0.254634	-5.721448	3.978094	3.08236	3.623781	4.030446	3.969275	3.901317	3.026505	3.047633	3.448717	3.129854	3.467722	3.397965	3.356854	3.212449
Taf1d	0.430228	4.35774	1.656511	0.11905	0.254741	-5.77736	3.84352	5.014693	4.792184	4.424653	4.409093	4.08677	4.958988	4.274505	4.656351	4.015725	3.995046	3.936334	4.250206	4.429189
Cdk5rap2	0.309473	5.48452	1.655614	0.119234	0.255089	-5.884385	5.239674	5.833178	5.069347	5.294232	5.691429	5.724133	5.41323	5.577792	5.665368	5.635752	5.31852	5.509703	5.428048	5.418348
Slco1a5	-5.620337	-1.562099	-1.655463	0.119265	0.255109	-4.618321	5.169999	-3.260591	-3.260591	-1.788967	-2.049163	-2.747572	-2.088409	-2.076466	-2.155202	2.90068	4.543347	-2.249472	-1.218554	-1.899685
Ift81	-0.284574	5.423559	-1.654329	0.119498	0.255548	-5.904316	5.603877	5.256049	5.247446	5.913781	5.493166	5.906658	5.419857	5.434577	5.393679	5.157622	5.168735	5.177238	5.424409	5.369687
Cyp4b1	1.003049	0.705438	1.654257	0.119512	0.255548	-4.964978	0.692264	0.679782	0.158563	-0.411255	2.124346	1.327961	1.258992	0.923327	0.385075	0.938085	0.885831	1.515634	-0.211665	0.65743
Zfand6	-0.269737	5.568135	-1.654154	0.119533	0.255548	-5.906964	5.684832	5.159298	5.462667	5.92261	5.688871	5.823965	5.653354	5.382731	5.458191	5.500753	5.603568	5.477471	5.638993	5.529868
Ikbke	0.424197	3.97342	1.653445	0.119679	0.255763	-5.667724	3.509242	4.159341	3.436929	3.348912	3.994346	3.839794	4.32559	4.123366	4.231815	4.277207	4.073974	4.416088	3.569478	4.425531
Dram2	0.415794	3.557888	1.653402	0.119688	0.255763	-5.692453	3.334978	3.418258	4.029817	3.800721	4.345567	4.138786	3.445603	3.272386	3.392049	3.223093	3.116419	3.83286	3.178239	3.417866
Psmc1	0.211575	7.727785	1.653354	0.119698	0.255763	-5.909528	7.956312	8.167058	8.371277	8.237917	8.22626	8.469356	7.232562	7.381171	7.334112	7.399348	7.465926	7.300705	7.399709	7.254965
Acp2	0.302082	6.278172	1.653246	0.11972	0.255765	-5.909656	5.933174	6.057805	5.88049	5.418025	6.302856	5.658196	6.546788	6.609939	6.624896	6.624871	6.463116	6.614847	6.399674	6.781016
Tlk1	-0.398519	7.196522	-1.652953	0.11978	0.255812	-5.921973	7.388483	6.995518	7.384542	8.210016	7.259445	7.959259	7.144612	7.014998	6.826216	6.849119	6.923045	6.840359	7.109731	6.856987
Rsrc1	-0.462904	3.715875	-1.652927	0.119786	0.255812	-5.806821	4.150251	3.74155	4.123244	5.058242	4.027109	4.702498	3.337752	3.272386	3.464507	2.694725	3.592118	3.261164	3.683472	3.041369
Dcn	0.661114	4.887925	1.652828	0.119806	0.255812	-5.808187	5.421475	4.848802	4.682767	3.175097	4.908409	5.247117	5.015502	5.065713	5.213859	5.235776	5.041404	4.890765	4.646703	5.09592
Tsga10	-0.437737	4.805201	-1.652396	0.119895	0.255956	-5.815266	4.481855	4.57644	4.099597	4.833262	3.746377	4.501484	5.796029	5.113519	5.405997	5.07749	4.960622	4.76429	4.817348	5.158958
Prima1	-0.534589	4.398449	-1.652144	0.119947	0.256022	-5.760634	4.28818	4.563896	3.277465	4.253272	4.094356	3.106838	4.583442	4.540363	4.793937	4.859579	4.627162	5.078905	4.481425	5.111939
Slc45a1	-0.270498	6.387785	-1.651636	0.120052	0.2562	-5.92497	6.343126	5.825316	6.399345	6.184497	6.030304	5.714371	6.608038	6.537566	6.728247	6.558439	6.412551	6.56486	6.679303	6.862408
Sdk2	-0.359821	4.896665	-1.651089	0.120164	0.256395	-5.855251	4.835542	5.071186	4.835775	5.175528	4.176973	4.526803	5.188472	5.042656	5.166377	4.821324	4.784073	5.179349	5.031023	4.772214
Ppdpf	0.342648	6.256458	1.650847	0.120214	0.256456	-5.930204	6.72298	6.903305	6.549023	5.930291	6.930854	6.287721	5.938219	6.049074	6.10507	6.195175	6.057577	6.028312	5.859831	6.053994
Adamts5	0.802333	0.472016	1.650719	0.120241	0.256467	-4.956744	0.995438	1.279848	1.220561	-0.705589	0.091273	0.772673	0.903114	0.645799	0.571595	0.20947	0.138317	0.28321	0.546019	0.844371
Zfand5	-0.392983	6.718233	-1.650609	0.120264	0.25647	-5.933834	6.547601	5.775864	6.677224	7.09592	6.503914	6.756396	6.994356	6.762376	6.878718	6.626578	7.026992	6.776955	7.003988	6.643721
Raf1	0.307716	6.521441	1.648965	0.120604	0.257149	-5.930268	6.337359	6.801456	6.655957	5.987159	6.377806	6.041593	6.821616	6.628447	6.646686	6.753901	6.621857	6.588085	6.438978	6.616708
Zfp112	-0.340001	3.339848	-1.648608	0.120677	0.257261	-5.661358	3.357948	3.317238	3.12518	3.815017	3.137307	3.468012	3.569982	3.42283	2.858731	3.352542	3.408041	3.195935	3.582598	3.302949
Tmem50a	-0.362231	7.077852	-1.648377	0.120725	0.257318	-5.937638	7.1206	6.252124	6.712171	7.278514	7.184725	7.199792	7.03665	6.98734	7.303713	7.147337	6.999735	7.313255	7.205602	7.360263
Sephs1	0.23084	5.621992	1.648235	0.120755	0.257335	-5.90865	5.497569	5.965443	5.606227	5.529143	5.701613	5.708237	5.789221	5.591758	5.677274	5.698792	5.557006	5.53465	5.366799	5.516176
Srd5a2	-0.957885	-0.651561	-1.647716	0.120862	0.257518	-4.962168	0.105685	-1.463779	0.914692	1.245516	-0.516166	0.231108	-0.399109	-0.26106	-1.304935	-0.961779	-1.310338	-0.657145	-1.638202	-0.397871
Wnt9a	-0.314239	3.884497	-1.647492	0.120909	0.257532	-5.689686	4.012279	3.125682	3.679531	3.450082	3.440689	3.34688	4.292269	4.17729	4.222559	4.086912	4.341911	4.00459	4.148003	4.16536
Ap1g1	-0.321259	5.918038	-1.64745	0.120917	0.257532	-5.928121	5.939533	5.607302	6.046933	6.360501	5.820863	5.840887	5.806182	6.009488	5.858496	5.580442	6.107418	5.893379	6.308843	5.698484
Aktip	-0.177496	6.493972	-1.647374	0.120933	0.257532	-5.938738	6.770121	6.536018	6.518777	6.722754	6.627911	6.59354	6.427994	6.199981	6.430586	6.312264	6.434321	6.407192	6.479873	6.47172
Itsn1	-0.297014	6.999321	-1.64718	0.120973	0.257563	-5.937496	7.179272	6.748396	7.13532	7.577543	7.017989	7.422975	6.847245	6.952972	6.800009	6.765571	6.868121	6.802515	7.16311	6.721852
Spats1	-0.636375	1.162498	-1.647066	0.120997	0.257563	-5.239876	1.568201	1.184734	1.723223	1.962578	0.091273	1.606524	1.607516	0.871929	1.489496	1.28803	0.961591	0.889587	0.791767	0.970221
Gnb1	-0.288203	6.958319	-1.646898	0.121032	0.257563	-5.939903	7.151056	6.726753	6.981173	7.208044	6.725965	7.132206	6.786151	7.045764	6.876507	6.728684	7.109452	6.879833	7.316754	6.760838
Mettl23	-0.371918	3.65384	-1.646891	0.121033	0.257563	-5.664528	3.467609	2.758277	3.313151	3.747057	3.61245	3.255598	4.145391	3.690957	3.996864	4.031278	3.717602	3.848862	3.5956	4.102607
Uap1	0.247494	3.890101	1.646615	0.121091	0.25764	-5.712762	3.736577	3.713429	4.008208	3.606198	3.965054	3.956189	4.107579	3.95452	3.854323	4.020928	4.052479	3.800313	3.833693	3.958175
Btg3	-0.451526	4.487257	-1.646368	0.121142	0.257703	-5.833569	4.342933	3.544937	4.638911	5.162543	4.548211	4.467019	4.674929	4.568886	4.618038	4.66726	4.655954	4.368779	4.254311	4.380898
Aff4	-0.47594	7.436018	-1.646238	0.121169	0.257715	-5.932102	7.36269	6.778567	7.662146	8.260291	7.080784	7.818843	7.383146	7.487981	7.257444	7.092792	7.544248	7.407206	7.759504	7.217909
Ttc12	0.531452	2.851701	1.64543	0.121337	0.258027	-5.50404	3.380557	3.159427	2.877559	1.720809	3.159643	2.949304	2.867532	3.311493	2.47808	2.866406	2.968181	3.018661	2.601568	2.791492
Adora2a	0.46446	2.199149	1.64515	0.121395	0.258105	-5.342693	2.179767	2.532247	2.187895	1.502648	2.09374	2.323579	2.534559	1.958936	2.508863	2.586593	2.332254	2.447635	1.859646	2.087586
Fam220a	-0.408092	5.02461	-1.644947	0.121438	0.258149	-5.88929	5.212627	4.24827	4.866124	5.46562	5.237232	5.364112	4.855242	5.028055	4.939446	4.916522	5.140489	4.961159	5.383763	4.775092
Hdhd3	-0.28941	3.38223	-1.644378	0.121556	0.258345	-5.663072	3.713016	3.34683	3.056656	3.387687	3.523394	3.121686	3.251219	3.301815	3.432752	3.393233	3.421519	3.529544	3.627601	3.395603
Rev3l	-0.323193	6.981858	-1.644298	0.121573	0.258345	-5.943634	6.50193	6.738662	6.415168	7.205785	6.365048	6.607451	7.733216	7.14111	7.4728	7.177353	6.96334	6.970996	7.130539	7.335625
Rtp4	0.835208	2.138101	1.644096	0.121615	0.258368	-5.273671	2.196854	1.506527	2.916749	0.74601	1.85913	2.570814	2.286414	2.822481	1.458292	2.795315	2.761158	3.600573	1.11438	1.708589
Aup1	0.257608	7.057121	1.64404	0.121627	0.258368	-5.944082	6.953466	7.126584	7.005462	6.665393	7.239488	6.894638	7.111563	7.12405	7.163229	7.351106	7.029195	7.114139	6.778843	7.25445
Pdcd4	-0.369712	6.228829	-1.643615	0.121715	0.258494	-5.941912	6.144979	6.006581	6.163118	7.068339	6.073097	6.70987	6.044959	6.276092	6.040696	6.091424	6.230245	6.301659	6.173403	5.900399
Polr1c	0.258933	4.560817	1.64355	0.121729	0.258494	-5.809615	4.421353	4.435504	4.770955	4.144271	4.545387	4.383247	4.718584	4.572916	4.752736	4.7573	4.760405	4.447955	4.512607	4.695317
Mrpl21	0.259928	4.004826	1.643016	0.121841	0.258685	-5.789843	4.092076	4.361326	4.047481	4.155549	4.611694	4.526803	3.790562	3.770297	3.696138	3.92982	3.750012	3.822092	3.860871	3.751656
Tssc1	-0.286808	4.676207	-1.642305	0.121989	0.258942	-5.842944	4.730919	4.014741	4.419086	4.713732	4.633136	4.659903	4.909618	4.710941	4.945085	4.751052	4.937355	4.549364	4.852977	4.70138
Tmem68	-0.416772	5.262121	-1.642231	0.122005	0.258942	-5.89257	4.983193	4.324616	4.872118	5.67865	4.910606	5.555324	5.419857	5.278033	5.50284	5.4065	5.252185	5.27917	5.724337	5.524749
Abracl	0.350944	4.516583	1.641195	0.12222	0.259356	-5.84591	4.214585	4.655425	4.664464	4.86567	5.050645	5.221502	4.551611	4.49437	4.397255	4.345154	4.400933	4.183498	4.278701	3.978289
H2afz	0.300335	3.961124	1.641054	0.122251	0.259373	-5.687458	3.683012	3.859159	3.619037	3.261145	3.939464	3.642872	4.567614	4.101219	4.290573	3.968034	4.246696	3.900956	4.233667	4.24638
Srf	0.206549	6.657016	1.64078	0.122308	0.259449	-5.947478	6.727396	6.550397	6.8354	6.379061	6.866681	6.783492	6.652795	6.596148	6.619617	6.641011	6.601279	6.610942	6.667807	6.681783
Slc38a6	0.511924	3.038663	1.64063	0.12234	0.25947	-5.492753	2.279379	3.667262	2.853524	2.62393	2.810268	2.602913	3.639767	2.988532	3.59934	3.158464	3.132898	2.990087	2.84965	3.544677
Cc2d1b	0.366513	6.427346	1.640347	0.122399	0.25955	-5.943069	6.244887	7.044875	6.438245	6.068963	6.382561	6.228084	6.728595	6.361258	6.618735	6.620593	6.278258	6.259382	6.053667	6.673325
Tfap2c	-1.636172	-0.252065	-1.640214	0.122427	0.259563	-4.794306	-0.634519	-3.260591	-0.411358	0.785264	-0.613814	-0.1418	-0.279766	0.04768	1.218642	1.023513	0.202178	1.186803	-0.211665	1.009844
Rogdi	-0.223995	7.969286	-1.639908	0.122491	0.259653	-5.930064	8.218513	7.965126	8.02564	8.228157	8.229343	7.777544	7.944059	7.84234	8.065753	7.864472	7.886431	7.629986	7.863943	8.034994

Slc39a14	0.349059	5.801614	1.639752	0.122524	0.259677	-5.917582	5.066895	5.63605	6.289424	5.77686	5.436364	5.733829	5.933595	5.898287	5.985677	5.898564	5.826363	5.925172	5.854432	5.989805
Fra10ac1	-0.514472	4.826187	-1.639629	0.12255	0.259686	-5.896086	4.745509	4.622535	4.337679	5.977659	5.004088	5.470877	5.030014	4.620407	4.670035	4.862481	4.763054	4.496167	4.519446	4.503936
Aldh1a2	0.977368	2.927535	1.639453	0.122586	0.259718	-5.698907	4.899562	3.667262	4.191945	1.892408	3.930833	5.34526	2.165439	2.030999	2.223535	2.359011	2.083688	2.461578	1.493524	2.503027
Cdk19	-0.343544	6.626799	-1.639041	0.122673	0.259856	-5.951005	6.50193	5.976134	6.5817	7.121068	6.41387	6.90003	6.605133	6.662876	6.542678	6.625725	6.798165	6.815466	6.664726	6.581804
Hectd2	0.391979	5.996147	1.638857	0.122712	0.259892	-5.940655	5.615043	6.171732	6.850152	6.276043	5.997614	6.335468	6.416994	5.524698	6.127559	5.789994	5.722668	5.446256	5.843574	5.853529
Mapk8	-0.27258	6.952673	-1.638551	0.122776	0.259982	-5.949773	7.089487	7.366688	7.139053	7.71634	6.9777	7.033107	7.016387	6.556003	6.842168	6.478437	6.716466	6.506369	7.053123	6.859022
Kin	0.282667	4.211437	1.638332	0.122822	0.260034	-5.816423	4.055074	4.525594	4.331892	4.476537	4.723369	4.641992	4.14005	4.269548	4.044916	4.150009	3.976904	3.926315	3.713602	4.070176
Phf13	-0.363092	3.998837	-1.638147	0.122861	0.26007	-5.762931	4.002595	3.055728	4.040502	4.188865	3.804231	3.988947	3.837517	4.254576	4.034376	4.135691	4.172914	4.319865	4.221137	4.027379
Snn	-0.332721	8.542093	-1.637294	0.12304	0.260405	-5.923695	8.737869	7.733763	8.459511	8.485347	8.290713	8.643902	8.479975	8.909282	8.493694	8.612784	8.821182	8.780451	8.733102	8.412034
Ets1	0.275244	5.168799	1.637063	0.123089	0.260462	-5.907993	5.362121	5.144631	5.588134	5.082081	5.506298	5.728989	5.221722	4.850248	4.936619	4.987339	4.906537	5.196126	4.992221	4.904232
L3hypdh	-0.421054	1.853033	-1.636486	0.123211	0.260673	-5.326477	2.054131	1.874793	1.269823	1.910273	1.78444	1.402804	2.398025	1.628188	2.313467	2.127786	1.740817	2.027776	1.881043	1.971974
Plcb2	0.552477	3.628014	1.634891	0.123547	0.261333	-5.566506	2.832262	3.1924	3.013923	2.677071	3.808949	3.193712	3.742028	4.782275	3.495578	4.927646	4.090943	4.901034	2.838755	3.43979
Rem1	0.815776	1.009566	1.634763	0.123575	0.261333	-4.984166	0.915118	1.21714	1.169557	-0.600663	0.952152	0.175273	1.607516	1.464337	1.180909	2.06872	1.347792	1.618784	0.698429	1.193114
Trmt11	0.585817	2.284864	1.634699	0.123588	0.261333	-5.335755	2.162475	2.843782	2.298852	1.273392	2.062471	1.910004	2.880403	2.58779	2.446625	2.877922	2.455292	2.433555	1.815874	2.278116
Iqcg	-1.866298	1.126459	-1.634481	0.123634	0.261385	-5.087343	2.906846	-3.260591	1.143363	0.43652	1.338932	1.165512	2.078759	2.030999	1.636128	1.253084	2.506778	1.691577	1.902128	1.473753
Acot13	-0.316995	4.056634	-1.634305	0.123671	0.261418	-5.856496	4.788412	4.425138	3.98258	4.706082	4.576156	4.753435	3.927077	3.466799	3.836231	3.790537	3.877725	3.315916	3.777752	3.667444
Selt	-0.485174	6.904453	-1.634028	0.12373	0.261481	-5.953609	7.376816	6.317103	7.250619	7.878265	7.313154	7.689044	6.39474	6.564122	6.57849	6.203207	6.747565	6.674427	6.996043	6.692476
Cyb5b	-0.327466	6.407709	-1.633948	0.123747	0.261481	-5.958232	6.666601	6.057805	6.699867	6.886822	6.435388	6.602829	6.324664	6.223239	6.266894	5.970246	6.582655	6.140906	6.677011	6.191722
Nupl2	0.344397	4.110482	1.633855	0.123767	0.261481	-5.75262	3.793849	4.479583	4.153089	3.742078	4.051203	3.775529	4.394501	4.269548	4.405458	4.353427	4.26176	3.975732	3.844626	4.138799
Tpbg	0.509116	3.463759	1.63366	0.123808	0.261522	-5.626406	2.885927	3.763659	3.570714	3.204354	3.818339	3.099357	3.797364	2.364261	3.83015	3.545408	3.243265	3.323571	3.748946	4.44734
Slc6a20	1.078022	3.388495	1.63334	0.123876	0.261619	-5.663347	4.380808	3.696289	4.775226	1.892408	2.883666	5.158103	2.979429	2.951874	3.244385	3.636356	2.428842	3.179156	2.816716	3.591131
Ryk	0.323871	5.111837	1.632752	0.124	0.261836	-5.91244	5.121607	5.039823	5.569811	5.240521	5.440931	5.958232	4.743453	4.958923	4.790809	4.830243	4.847853	5.151664	4.86645	5.051519
Lsm4	0.273997	5.815683	1.632478	0.124058	0.261913	-5.953639	6.317966	6.427683	6.235424	5.932479	6.663514	6.266167	5.417651	5.425693	5.597175	5.592723	5.699345	5.218879	5.372476	5.281381
Cbx7	-0.354981	6.245582	-1.632146	0.124129	0.262016	-5.948621	6.084293	6.356732	5.743341	6.095557	5.488762	5.364112	6.619598	6.364749	6.558398	6.72231	6.407481	6.521424	6.375378	6.757932
Dnajc15	-0.269178	5.192085	-1.631984	0.124163	0.262042	-5.950063	5.933174	5.541644	5.734594	6.266538	5.890403	6.157795	4.781687	4.468651	4.778229	4.456866	4.765698	4.338875	4.861076	4.760648
Lrch1	0.352247	3.700249	1.631109	0.124349	0.262388	-5.750236	3.799454	4.155166	4.432669	3.659984	3.926497	3.784887	3.261094	3.466799	3.836231	3.559779	3.250834	3.885525	3.349197	3.558103
Ropn1l	0.75486	0.243911	1.630839	0.124406	0.262464	-4.884483	0.031429	0.310827	0.442561	-0.705589	0.091273	0.481842	0.118118	1.356279	0.512061	0.134646	0.846404	0.007173	0.491415	0.65743
Ppp1r9b	-0.29226	10.93326	-1.630506	0.124477	0.262567	-5.84456	11.16136	10.6212	10.48174	10.90925	10.76582	11.10707	11.25787	10.89363	11.00639	11.07259	10.70814	11.02343	10.92965	11.12836
Dtx3	-0.250031	7.574265	-1.630212	0.12454	0.262653	-5.95977	7.659325	7.49406	7.254066	7.408328	7.310246	7.078046	7.920108	7.638299	7.850929	7.798973	7.626194	7.563257	7.505534	7.940762
Tbce	0.278337	5.666026	1.62998	0.124589	0.262711	-5.921109	5.296263	5.864205	5.627643	5.224545	5.454545	5.211127	6.109239	5.684052	5.949668	5.852614	5.761604	5.627247	5.689893	6.003364
Zfp483	-0.514599	6.083078	-1.629524	0.124686	0.26287	-5.9548	5.739788	6.097515	6.044281	6.760176	4.919359	5.893742	6.393618	6.432836	6.421514	5.932091	6.158615	5.889516	6.481625	6.022862
Ankrd54	0.366609	3.921752	1.629314	0.124731	0.262888	-5.728846	3.953171	4.060109	3.822584	3.225913	4.202298	3.683465	3.981847	3.720315	3.952765	4.130887	4.258009	3.869926	3.91892	4.229697
Crispld1	-0.81779	1.239557	-1.629233	0.124748	0.262888	-5.188569	1.430522	0.118359	-0.33572	1.571628	1.05105	2.131561	2.033384	1.532126	1.393785	1.569444	1.135223	1.760874	1.1849	1.529341
Ghr	-0.333716	3.893993	-1.629142	0.124768	0.262888	-5.716693	3.402817	3.046741	3.724397	4.058754	3.359399	3.47381	4.52317	3.884925	4.372361	4.12123	3.963146	3.961084	4.298715	4.436477
Dhx38	0.206218	5.78259	1.629071	0.124783	0.262888	-5.942502	5.591009	5.778581	5.976348	5.674735	5.753932	5.846484	5.846094	5.87889	5.902631	5.926557	5.83646	5.735932	5.594349	5.642865
Pik3ca	-0.307887	5.297788	-1.628664	0.12487	0.26302	-5.930932	5.212627	5.112689	5.084914	5.914888	5.252899	5.593102	5.395408	5.13548	5.132184	5.14338	5.332856	5.212709	5.229661	5.456487
Dnajc2	0.282691	5.866932	1.628574	0.124889	0.26302	-5.959007	5.816415	6.213492	6.421994	6.397387	6.265585	6.661158	5.731791	5.687772	5.570064	5.594469	5.432737	5.403558	5.591107	5.377281
Fzd3	-0.704261	3.577075	-1.628469	0.124911	0.263021	-5.711735	3.342675	2.411416	3.711083	4.354614	2.874693	3.579757	3.383673	4.055878	3.675936	2.988286	4.056804	3.680242	4.659142	3.447025
Ccdc50	0.311356	4.642011	1.627632	0.12509	0.263351	-5.865991	4.586045	5.206468	4.998051	4.520817	4.55103	4.704965	4.656342	4.799571	4.614504	4.429738	4.316888	4.542821	4.777968	4.346493
Tnks	-0.441842	5.851562	-1.627207	0.125181	0.263496	-5.949037	5.731017	5.605773	5.993863	6.135975	4.881785	5.488167	6.19802	6.219389	5.995221	5.627248	5.910014	5.831641	6.564139	5.768014
Emc4	-0.245193	6.188847	-1.627052	0.125214	0.263519	-5.960017	6.231475	5.611879	5.99935	6.16974	6.156905	6.05035	6.19287	6.06918	6.453514	6.283329	6.338111	6.32282	6.413506	6.372622
Ttc22	0.49431	3.216573	1.626857	0.125256	0.263561	-5.586686	3.467609	3.49955	3.145118	2.169587	3.470774	3.014396	3.116285	3.292072	3.487872	2.911926	3.53126	3.195935	3.262178	3.642453
Slc35a4	0.317586	5.828836	1.625886	0.125464	0.263952	-5.940616	5.777191	5.590392	5.913801	5.182896	6.174378	5.489598	5.772058	5.926901	5.835912	5.878641	6.147507	6.032988	5.937201	5.972362
Mef2a	-0.664601	6.586857	-1.625365	0.125575	0.264141	-5.969403	6.854645	5.926776	7.102555	7.994042	6.455839	7.544681	6.001439	6.601088	6.16307	5.856985	6.588641	6.39271	6.83885	5.912209
Vps13d	-0.271675	7.394522	-1.624908	0.125673	0.264301	-5.971457	7.021581	7.078337	7.138224	7.502609	6.636563	7.107246	7.862084	7.672497	7.668264	7.359858	7.580368	7.513929	7.707303	7.684114
Lmbr1l	0.626504	3.568012	1.624552	0.125749	0.2644	-5.623094	3.035892	4.506054	3.74196	2.835056	3.403739	2.623922	3.981847	3.517851	3.836231	3.656554	3.586147	3.668268	3.569478	4.129836
Pllp	-0.473391	6.765202	-1.624483	0.125764	0.2644	-5.971794	7.183028	6.60805	5.904083	6.447951	6.948065	5.912006	6.493334	7.02753	6.803893	7.05968	6.857005	7.409006	6.9398	7.134667
Krit1	0.278773	6.376504	1.62434	0.125795	0.264418	-5.968569	6.136148	6.800788	6.781219	6.457095	6.384933	6.33706	6.676971	6.116873	6.397039	6.277907	6.050004	6.058442	6.233904	6.581804
Reep4	0.423969	3.110275	1.622829	0.12612	0.265054	-5.591372	2.875353	3.404252	3.402215	2.768056	3.716556	2.82781	3.159163	3.413873	2.759824	3.636356	3.157269	3.419555	2.508394	3.041369
Cxcl9	2.215699	-2.398735	1.622597	0.12617	0.265089	-4.647945	-1.908947	-2.267963	2.162035	-1.788967	-0.831496	1.046069	-3.260591	-0.81357	-3.260591	-2.176425	-1.978322	-1.073407	-3.260591	-3.260591
Ogg1	0.341428	3.208706	1.622547	0.12618	0.265089	-5.624816	2.927466	3.263946	3.158259	2.568755	3.196117	2.705027	3.504983	3.169712	3.298245	3.574008	3.537463	3.502822	3.278395	3.410483
Ube2h	-0.226622	7.506374	-1.621722	0.126358	0.265416	-5.972512	7.461439	7.015234	7.424858	7.552876	7.188351	7.496013	7.673927	7.646925	7.442227	7.637296	7.717459	7.708091	7.64652	7.486755
Cat	-0.250842	7.013504	-1.621352	0.126438	0.265484	-5.9732	7.524129	7.196313	7.133242	7.487441	7.536894	7.153584	6.585618	7.109561	6.758983	6.815429	6.602759	6.883075	6.817506	6.596518
Pgam1	-0.354614	8.515794	-1.621349	0.126438	0.265484	-5.94587	8.528368	7.73061	8.344222	8.785084	8.602007	8.535123	8.701024	8.488362	8.668107	8.378779	8.841848	8.344637	8.813551	8.463775
RGD1309586	-0.509041	1.707712	-1.621211	0.126468	0.265484	-5.293834	1.48718	1.117652	1.116684	2.077719	0.985877	1.993092	2.165439	2.030999	2.313467	1.322149	1.605665	1.910689	2.022549	2.261799
Dcaf15	0.343208	5.450244	1.620929	0.126529	0.265484	-5.901556	4.997891	5.457677	5.22264	4.627263	5.205378	4.726978	6.010249	5.51426	5.86446	5.887213	5.596159	5.537944	5.566555	6.126755
Mid1	-0.441945	2.424609	-1.620787	0.12656	0.265484	-5.537851	2.529164	2.046927	2.735741	3.130069	2.212449	2.581593	2.100922	2.345542	2.166756	2.088679	2.580719	2.433555	2.994118	2.294252
Ranbp1	0.350747	5.646625	1.620776	0.126562	0.265484	-5.976225	6.16899	6.464425	6.726579	6.809798	6.929229	7.242869	4.725734	5.048455	4.930947	4.609719	5.026046	4.706916	5.035801	4.664615
Kctd15	0.304793	5.503838	1.620757	0.126566	0.265484	-5.929659	5.555019	5.474531	5.405955	4.809659	5.695256	5.34684	5.733565	5.488896	5.385408	5.789994	5.724028	5.562412	5.460392	5.656972
Parp6	-0.175776	7.590882	-1.620683	0.126582	0.265484	-5.968754	7.809211	7.576774	7.500004	7.70583	7.620791	7.630232	7.685432	7.454524	7.580874	7.540534	7.553458	7.389537	7.660121	7.573187
Cldn2	-5.647508	0.440663	-1.620649	0.126589	0.265484	-4.969047	7.099176	-1.10215	-1.705055	0.105385	-2.352077	-1.823292	0.202532	-0.81357	-0.080923	4.051757	6.526074	-0.920914	0.491415	-0.505482
Pdpr	-0.331283	4.089624	-1.620284	0.126668	0.265603	-5.802906	3.987945	3.939046	4.14651	4.351348	3.596165	3.693437	4.61073	4.370218	4.303799	3.88487	4.152854	3.911153	4.349488	4.051312
Nutf2	0.272305	5.953423	1.620134	0.126701	0.265625	-5.970185	6.226284	6.070055	6.200915	5.888093	6.660907	6.380144	5.768601	5.71355	5.63778	5.868575	5.954731	5.666902	5.824373	5.512732
Mtmr2	0.244492	6.784109	1.619948	0.126741	0.265663	-5.979108	6.752894	7.437593	7.338024	7.173326	7.020534	7.033597	6.668648	6.530333	6.61697	6.3867	6.526074	6.513916	6.51023	6.483202
Zw10	0.33117	3.758056	1.619553	0.126826	0.265796	-5.723085	3.136736	3.63764	3.952724	3.829173	3.965054	3.737479	3.824257	4.050109	3.930199	3.608978	3.70112	3.562264	4.012508	3.751656
Tmx1	-0.338661	5.984955	-1.619328	0.126875	0.265851	-5.961655	5.695391	5.144631	5.687785	6.152018	5.589544	5.91627	5.936679	6.006512	6.201266	6.107409	6.146493	6.251378	6.475483	6.504163
Slc25a18	-0.531709	5.823568	-1.61905	0.126935	0.26593	-5.962758	6.633254	6.06004	5.43459	5.411768	5.688871	4.78409	6.092744	5.405502	6.047264	6.131661	5.69519	5.660872	6.291969	6.221559
Asb7	-0.625353	2.828266	-1.618354	0.127086	0.266127	-5.710589	3.163058	2.33964	3.393138	4.291262	2.856578	3.844276	2.286414	3.201289	2.127623	1.780488	2.556491	2.516041	2.881849	2.59708
F12	-0.473391	2.953154	-1.618323	0.127092	0.266127	-5.477126	2.810225	2.582864	1.981598	2.283631	1.556544	2.203108	3.699086	3.517851	3.448717	3.88487	3.53126	3.433771	3.079891	3.56477
Ccdc19	0.864254	2.404104	1.618309	0.127095	0.266127	-5.352963	2.927466	2.167912	2.538805	0.386344	2.472441	2.458346	2.144252	3.012464	2.204856	2.165862	2.531849	2.8487	3.294432	2.847239
Cdh22	0.335728	5.235361	1.617881	0.127188	0.266275	-5.916058	5.000326	5.587296	5.376741	4.86567	4.897375	5.211127	5.532178	5.091219	5.389549	5.286341	5.113756	5.374373	4.949817	5.661644
Tmem248	0.328767	5.912581	1.617682	0.127231	0.266319	-5.956415	5.919085	5.830563	6.110004	5.188397	6.024231	5.541588	5.684875	6.096026	5.936991	6.132863	6.255863	6.231172	5.719891	6.131254
Dis3l2	-0.219118	5.429117	-1.617048	0.127369	0.26656	-5.951754	5.439533	5.433746	5.195808	5.753545	5.361263	5.533284	5.484505	5.599679	5.375002	5.34974	5.407561	5.412558	5.288894	5.409117
Slc39a13	0.311568	5.752663	1.615543	0.127696	0.267058	-5.94268	5.608673	5.878205	5.452035	4.912957	5.664348	5.278924	5.994057	5.922171	6.012778	6.01526	5.896807	5.947637	5.814676	6.168932
Rtkn	-0.23427	5.003936	-1.615447	0.127717	0.267058	-5.928448	5.18292	5.240367	5.051851	5.258066	4.733331	4.930402	5.000844	5.045558	4.933786	4.809346	4.837836	5.105789	5.031023	4.943127
Spem1	0.528661	1.407954	1.615445	0.127717	0.267058	-5.142697	0.955837	1.679303	1.430088	0.621404	1.230571	0.846031	1.836107	1.532126	1.636128	1.755735	1.135223	1.715048	1.902128	2.050068
Cwf19l2	-0.418571	4.505279	-1.615417	0.127723	0.267058	-5.913035	4.664912	4.294561	4.576652	5.520445	4.545387	5.300289	4.407896	4.254576	4.19443	3.924277	4.387261	4.308338	4.553161	4.212819
RGD1307704	-0.438952	4.111386	-1.615294	0.12775	0.267058	-5.82536	4.22295	3.028596	4.320248	4.421542	4.223657	3.909896	3.993741	4.134313	4.199156	4.086912	4.486775	4.212646	4.409621	4.003043
Ccdc17	0.441128	3.419784	1.615257	0.127758	0.267058	-5.638997	3.417469	3.854016	3.138503	2.57996	3.529124	3.193712	3.337752	3.638097	3.456633	3.728285	3.939921	3.78376	2.860463	3.571405
Kctd10	0.192974	6.13628	1.615135	0.127785	0.267058	-5.980833	6.276374	6.300186	6.39102	6.213565	6.446028	6.612716	6.134975	5.834271	6.070665	5.940353	5.821288	5.902352	5.901	6.085628
Tead1	0.48419	2.580197	1.614972	0.12782	0.267058	-5.392367	2.488111	3.108509	3.838998	3.027403	2.712936	3.288866	2.305626	2.345542	2.524011	1.852296	2.243983	1.950783	2.760099	1.951771
Zfp189	-0.658388	5.463129	-1.614938	0.127827	0.267058	-5.960328	5.388389	3.97739	5.805215	6.393423	5.358047	5.763716	5.375091	5.697031	5.588194	5.2885	5.647241	5.344586	5.820225	5.075058
Depdc7	-0.476684	2.480944	-1.614932	0.127829	0.267058	-5.511227	2.474162	2.690649	1.723223	2.862845	2.484288	2.025031	2.905804	2.345542	2.347926	2.40749	2.133674	2.931181	2.689088	3.002614
Lrrc45	0.381449	6.451041	1.614765	0.127865	0.267087	-5.9772	6.125033	6.941991	6.436898	5.87109	6.268157	5.869757	6.798917	6.600102	6.617853	6.872332	6.437641	6.47578	6.297947	6.719613
LOC500227	-0.298374	3.680389	-1.614032	0.128025	0.267374	-5.750551	4.017097	3.390109	3.171281	3.556013	3.617838	3.756629	3.654827	3.904241	3.83015	3.7783	3.73929	3.61312	3.805994	3.814538
Toe1	0.447994	3.485526	1.613412	0.12816	0.267597	-5.646457	3.171726	4.042133	3.545931	2.862845	3.313654	2.957603	3.783728	3.630385	3.780555	3.913127	3.586147	3.516245	3.195422	3.642453
Alpk3	-0.645285	2.407022	-1.613339	0.128176	0.267597	-5.415118	2.32672	2.100024	0.883381	2.269859	1.43972	2.349037	2.518182	3.211662	2.568526	3.195748	2.782205	3.931333	2.151407	2.310209
Fau	0.319795	4.576441	1.611868	0.128497	0.268221	-5.872694	4.661839	5.099711	4.554459	4.14804	4.608991	4.621246	4.689628	4.332691	4.541944	4.827276	4.570823	4.506292	4.318455	4.655275
Adar	-0.246681	6.719752	-1.61067	0.128759	0.268721	-5.990051	6.446753	6.568564	6.449647	6.66605	6.078969	6.145135	6.95231	7.080847	7.016975	6.790157	6.975955	7.00769	7.084013	6.828878
Rtn4ip1	-0.25106	4.695603	-1.610464	0.128804	0.268731	-5.885515	4.539921	4.332034	4.31439	4.800109	4.576156	4.469923	5.000844	4.775299	5.035072	4.913728	4.960622	4.588013	4.698844	4.795073
Mrfap1	-0.324563	8.689797	-1.610445	0.128808	0.268731	-5.938986	9.120209	8.56285	8.922606	9.508415	9.005476	9.467692	8.35789	8.359593	8.410039	8.32721	8.604554	8.320214	8.459843	8.23449
Tspan11	0.446484	2.834658	1.610204	0.128861	0.26878	-5.526878	2.402324	3.33211	3.013923	2.451642	2.692656	2.469996	2.67414	2.795149	2.797714	3.241039	2.940364	3.631738	2.66462	2.802815
Snx19	-0.203316	7.003579	-1.610096	0.128885	0.26878	-5.994105	6.774395	6.45766	6.783341	6.989652	6.7623	6.711711	7.216157	7.265195	7.196702	7.105093	7.04234	7.236265	7.246363	7.27543
Fiz1	0.273096	4.710216	1.610031	0.128899	0.26878	-5.890155	4.805223	4.846214	4.947854	4.298064	4.981279	4.492945	4.464549	4.816662	4.710323	4.785087	4.857801	4.689249	4.659142	4.649015
Pkp1	0.442668	2.040747	1.608964	0.129133	0.269222	-5.458683	2.402324	2.822876	2.401882	1.837445	2.702832	2.271274	1.21902	2.186344	2.147322	1.755735	1.629088	2.027776	1.54805	2.011549
Far1	-0.459864	4.687948	-1.608607	0.129212	0.269323	-5.935304	4.725042	4.462789	4.880071	5.798604	4.584435	5.303548	4.648839	4.294162	4.765539	3.844356	4.555623	4.372473	5.02623	4.432837
Cdc27	-0.458588	5.253053	-1.608538	0.129227	0.269323	-5.967119	5.382801	4.758107	5.461342	6.109156	5.084116	5.845367	5.22677	5.268128	5.124751	4.571225	5.204257	5.085673	5.556616	4.906858
Rrp8	0.243859	3.596821	1.608309	0.129277	0.269356	-5.789176	3.84352	3.763659	4.116527	3.742078	4.059146	4.174824	3.270902	3.321106	3.280514	3.195748	3.31723	3.405197	3.549571	3.447025
Ier5	0.234072	5.673644	1.608192	0.129303	0.269356	-5.970413	5.600671	5.680307	6.030055	5.550659	5.884827	5.637684	5.193638	5.749593	5.519942	5.864239	5.858922	6.013007	5.553287	5.325228
Daw1	1.28816	0.344109	1.608127	0.129317	0.269356	-4.890389	0.830061	-0.465897	0.398943	-1.788967	1.173177	1.046069	0.202532	0.972957	0.007959	-0.210791	0.805871	1.152831	1.465469	0.753927
Kctd11	0.578877	1.342683	1.608014	0.129342	0.269356	-5.289389	1.996747	1.397635	1.687412	0.74601	2.124346	2.349037	0.903114	1.020936	0.683763	1.21727	1.430105	1.542121	1.077784	1.260365
Srp14	-0.362117	6.15843	-1.607952	0.129356	0.269356	-5.99721	6.7565	6.099689	6.595653	7.18438	6.615846	7.121166	5.578326	5.653939	5.892453	5.578679	5.945431	5.630336	5.976249	5.612623
Phc1	0.283439	6.598659	1.607854	0.129377	0.269356	-5.994483	6.50021	7.098591	6.769492	6.383864	6.488556	6.544799	6.684329	6.597137	6.593826	6.753119	6.401542	6.519759	6.30588	6.756477
Cltc	-0.312579	9.393336	-1.607083	0.129547	0.269663	-5.936456	9.210263	8.817883	9.298165	9.816342	9.27734	9.4063	9.554065	9.437538	9.597393	9.047246	9.466202	9.272321	9.743825	9.564198
Spats2	-0.173735	5.711127	-1.606018	0.129781	0.270103	-5.979805	5.789915	5.581085	5.753117	5.85619	5.574383	5.688428	5.728235	5.735283	5.692438	5.674236	5.809803	5.715713	5.734658	5.652285
Ly6h	-0.259223	8.369942	-1.60592	0.129803	0.270103	-5.975053	8.421808	7.830215	8.268987	8.382712	8.390681	8.087213	8.371628	8.273088	8.670236	8.50366	8.274019	8.365644	8.548809	8.795305
Cdc42ep3	-0.274137	3.53075	-1.605443	0.129908	0.270275	-5.81268	4.264063	3.780021	4.040502	3.888957	3.546179	3.808019	3.40164	3.311493	2.995434	3.538169	3.549789	3.46871	3.003904	2.972849
Mir421	-0.999758	0.151984	-1.605239	0.129953	0.270311	-4.939349	0.105685	0.533043	-0.130036	0.43652	-0.516166	-2.070862	0.903114	0.58326	1.142163	0.938085	0.484915	0.567345	-0.040977	0.970221
Klf16	-0.298407	6.222152	-1.605103	0.129983	0.270311	-5.988501	6.225243	5.391775	6.229988	5.945532	5.874737	5.640264	6.349175	6.551926	6.44558	6.507455	6.741533	6.459371	6.464894	6.304554
Ap2b1	-0.42699	4.998604	-1.605059	0.129993	0.270311	-5.929101	5.019661	3.986819	4.852041	5.204776	4.805931	5.074724	5.103206	5.265641	5.027119	4.613168	5.287575	5.116845	5.589483	5.084367
Zdhhc21	-0.65895	3.43961	-1.604948	0.130018	0.270315	-5.752905	3.127854	3.176008	3.8018	4.591724	2.212449	3.693437	3.529702	3.653399	3.510866	2.923085	3.408041	3.221521	3.964736	3.503633
B4galnt4	0.394841	7.478158	1.604358	0.130148	0.270539	-5.999703	7.147216	8.152469	7.617054	7.077262	7.187445	7.066582	7.891388	7.369058	7.761487	7.692139	7.227325	7.308923	7.395533	7.809393
Ndufaf7	-0.253912	4.678485	-1.603968	0.130234	0.270672	-5.91455	4.627594	4.613433	4.485756	5.097757	4.573386	4.788749	5.024227	4.402272	4.778229	4.637083	4.655954	4.440934	4.752072	4.683115
Matr3	-0.334723	3.477631	-1.603695	0.130295	0.270751	-5.821322	3.695089	3.643613	3.637922	4.563778	4.05518	3.905613	3.29032	3.272386	3.350165	2.854798	3.065821	2.869767	3.3415	3.286911
Chrnb2	-0.378943	5.313058	-1.603423	0.130355	0.270784	-5.938324	4.997891	4.236461	5.171711	5.355827	4.95385	4.81639	5.417651	5.646311	5.677274	5.188006	5.605045	5.589649	6.037083	5.731448
RGD1308134	0.310203	5.103173	1.603418	0.130356	0.270784	-5.943157	5.300221	5.322718	5.373927	4.738944	5.563257	5.036061	5.058606	5.143629	5.076761	5.24692	5.162729	4.758654	4.719778	4.988458
RGD1309540	-0.242043	7.052546	-1.603167	0.130412	0.270853	-6.002995	7.320437	6.80812	6.903801	7.203976	7.40249	6.967332	6.978732	6.947551	7.068254	7.089082	7.126525	6.915094	7.074757	6.941375
Sra1	0.48127	5.888493	1.602688	0.130517	0.271026	-5.962793	5.384666	6.411146	5.431887	5.107469	5.892627	5.177772	6.498561	6.044728	6.130036	6.435247	5.929011	6.001123	5.665438	6.357344
Pccb	-0.256283	5.130458	-1.602571	0.130543	0.271033	-5.959535	5.632417	5.242337	4.968516	5.17368	5.388311	4.984199	4.937596	5.065713	5.146937	5.018886	5.065211	5.145199	4.908735	5.19378
Arid4a	-0.372881	5.694816	-1.601811	0.130712	0.271336	-5.990824	5.528269	5.595024	5.710265	6.541192	5.359656	5.993973	5.668273	5.803739	5.707445	5.436012	5.57822	5.614823	5.799304	5.422024
Sdc3	-0.270898	8.234674	-1.600987	0.130895	0.271669	-5.983522	8.380342	7.907648	8.48418	8.337832	7.997138	7.970067	8.068773	8.12076	8.366845	8.218189	8.141084	8.293666	8.444879	8.559294
Snx24	0.227662	5.338281	1.600688	0.130961	0.27176	-5.961213	5.270265	5.462754	5.380951	5.202965	5.336968	5.673392	5.246787	5.278033	5.282274	5.506329	5.184631	5.54123	5.085035	5.323264
Cep85	0.402287	5.53487	1.60041	0.131023	0.271842	-5.953311	4.920286	6.078899	5.566119	5.222759	5.070428	5.200677	6.023363	5.630934	5.893911	5.826109	5.419364	5.474036	5.391239	5.805084
RGD1307621	-0.298019	2.13024	-1.600158	0.131079	0.271867	-5.527867	2.501925	2.082541	2.456093	2.687469	2.484288	2.174914	2.247205	1.830204	2.127623	1.986005	1.762161	1.848377	1.983515	2.011549
Skp1	-0.416477	9.059651	-1.600069	0.131099	0.271867	-5.923636	9.623461	8.903762	9.398303	10.22097	9.605208	10.11403	8.70193	8.482751	8.706998	8.500172	8.775174	8.459174	8.866137	8.480178
Zfp949	-0.379364	3.481315	-1.60005	0.131103	0.271867	-5.756507	3.388015	3.843675	3.436929	4.066741	2.95351	3.255598	3.684484	3.458111	3.735714	3.050666	3.518775	3.405197	3.658907	3.425211

Rgl2	0.45274	6.427933	1.599831	0.131152	0.271921	-5.994236	5.851906	6.904549	5.992029	5.792597	6.244843	5.96133	6.912138	6.547838	6.684255	6.941872	6.407481	6.777652	6.010632	6.981164
Cab39l	-0.435898	4.808662	-1.599705	0.13118	0.271932	-5.952582	5.906157	4.929249	4.751577	4.72387	4.997903	4.809529	4.743453	4.635896	4.457672	4.502215	5.33997	4.506292	4.599077	4.475916
Prim1	0.294095	4.228602	1.599106	0.131313	0.272161	-5.891724	4.62444	4.936565	4.432669	4.207046	4.815341	4.644565	4.292269	3.798099	3.958351	4.116377	3.802462	4.00459	3.731382	3.922287
Trim46	-0.223534	6.544964	-1.598404	0.13147	0.272376	-6.006856	6.51391	6.080001	6.385443	6.445656	6.411545	6.14695	6.811572	6.7659	6.904269	6.735029	6.61087	6.430419	6.62406	6.781016
Zbtb3	-0.308172	2.312315	-1.59839	0.131473	0.272376	-5.522342	2.555898	2.100024	2.31067	2.568755	2.198134	2.410784	2.305626	2.364261	2.597457	2.048482	2.274011	2.2368	2.575555	2.142093
Lrrc57	-0.234614	4.728642	-1.598335	0.131485	0.272376	-5.934465	5.033995	4.622535	4.716472	4.939298	4.995836	4.670039	4.80887	4.527965	4.534483	4.513333	4.879936	4.584831	4.686744	4.746057
Kdr	0.458823	4.711565	1.597754	0.131615	0.272598	-5.910703	4.891713	4.462789	4.796393	4.177845	5.237232	5.422235	4.955952	4.397736	5.084445	4.585342	4.269233	4.40891	4.254311	5.07972
Tpk1	-0.353572	2.229904	-1.597446	0.131684	0.272694	-5.595602	2.645748	2.324846	2.322392	3.16769	2.732935	2.898483	2.165439	1.775313	1.867508	1.625169	2.083688	2.046399	2.060555	1.846283
Mrps11	0.356298	4.72673	1.597251	0.131727	0.272737	-5.903283	4.824591	5.062295	4.605721	4.136703	5.146953	4.507149	4.965043	4.552657	4.793937	4.997931	4.64163	4.698109	4.631001	4.670808
Rnf6	-0.368319	6.26045	-1.596822	0.131823	0.272888	-6.009454	6.272349	5.723247	6.22921	6.843313	6.241356	6.565308	6.203151	6.412745	6.166694	6.02306	6.467184	6.117979	6.505071	5.896441
Fbxw11	-0.3237	7.161827	-1.596476	0.1319	0.272983	-6.009666	7.315557	6.664956	7.365902	7.693309	7.215257	7.477737	6.955354	7.10609	6.93647	6.981775	7.300724	7.049256	7.289008	6.925276
Ppp2ca	-0.473592	4.838762	-1.596354	0.131927	0.272983	-5.943829	4.777095	3.874479	4.725329	5.53203	4.810643	5.310045	4.958988	4.568886	4.981207	4.609719	5.084402	4.800396	5.014175	4.751911
Sptbn4	-0.281786	8.800167	-1.596277	0.131945	0.272983	-5.984113	8.354401	8.542214	8.228289	8.794434	8.262183	8.128227	9.495927	9.039642	9.381118	9.081265	8.850592	8.971923	8.917521	9.158312
Dnttip1	0.2729	5.662799	1.59621	0.13196	0.272983	-5.987329	5.744154	6.16136	5.856504	5.375252	5.734224	5.571636	5.671979	5.555567	5.72885	5.814171	5.682652	5.504661	5.428048	5.481365
Smim5	-0.961901	0.217076	-1.596036	0.131999	0.273017	-4.917293	0.955837	0.24948	-0.665442	-0.705589	-0.718553	-1.823292	1.047044	1.198329	1.360416	1.023513	0.846404	0.567345	0.791767	0.65743
Erlec1	-0.232127	6.887747	-1.595233	0.132178	0.273341	-6.015884	6.928749	6.506004	6.876729	7.101763	6.882994	6.866818	6.760273	6.701112	6.912927	6.932239	6.925412	6.9795	6.945524	7.121727
Dpp8	-0.218521	8.293484	-1.594684	0.132301	0.27353	-5.98588	8.425211	8.160563	8.297662	8.715665	8.380613	8.566831	8.239885	8.143966	8.165726	8.075148	8.213084	8.173858	8.384122	8.171463
Oxa1l	0.278399	6.594154	1.594624	0.132315	0.27353	-6.013443	6.639517	6.946227	6.838974	6.217158	6.754372	6.284426	6.540721	6.72575	6.63103	6.611999	6.616008	6.575318	6.346826	6.606245
Ngrn	-0.190999	6.302143	-1.594494	0.132344	0.273543	-6.013305	6.576449	6.412021	6.166373	6.47296	6.437675	6.320264	6.212087	6.185581	6.241917	6.250484	6.371478	6.122375	6.315735	6.16455
Get4	0.200104	5.734167	1.594094	0.132434	0.273671	-5.996387	5.820555	5.846187	6.087855	5.589986	5.915776	5.763716	5.64395	5.571764	5.653363	5.665956	5.71448	5.74736	5.731717	5.555197
Spag5	0.46496	3.478145	1.594016	0.132451	0.273671	-5.697992	3.145563	3.982112	2.869592	3.107017	3.969275	3.601103	3.810873	3.599118	3.641623	3.552611	3.31723	3.220743	3.270309	3.751656
Gpr153	-0.645929	2.24617	-1.593823	0.132495	0.273691	-5.407029	1.957182	2.493084	1.705429	2.324175	1.556544	0.435061	2.703406	2.436799	2.797714	2.783118	2.317912	2.9008	2.423441	2.992761
Slc7a2	0.574234	2.880661	1.593731	0.132515	0.273691	-5.605389	3.171726	3.08236	3.948948	2.154676	2.630048	3.099357	2.613764	3.211662	2.47808	2.514649	2.823399	3.245131	2.902922	2.673087
Gclc	0.221748	6.297217	1.593667	0.13253	0.273691	-6.014352	6.314056	6.496134	6.923176	6.481947	6.496621	6.56259	6.186407	6.116873	6.022144	6.010037	6.076869	6.02714	6.143835	6.323349
Tmie	-0.301862	3.472839	-1.59264	0.13276	0.27412	-5.781692	3.997728	3.224637	3.365078	3.584109	3.785203	3.547133	2.774074	3.645768	3.169295	3.622732	3.728487	3.674268	3.32598	3.31881
Rgs2	-0.465463	4.149615	-1.592514	0.132789	0.274132	-5.922427	4.495729	3.557647	4.588836	5.220971	4.659497	4.755816	3.577905	3.884925	3.91304	3.709073	4.086719	3.911153	4.254311	3.571405
Mlph	-0.864106	-0.563193	-1.592412	0.132812	0.274132	-4.801327	-0.310971	-0.687936	-1.705055	-0.81875	-1.798946	-0.862697	-0.067151	0.923327	-0.929891	1.51148	1.068256	0.930264	-1.413173	-0.886923
Ahsa1	-0.33556	7.185153	-1.592252	0.132848	0.274159	-6.015829	7.304268	6.663487	7.038706	7.70295	7.504903	7.463967	7.167764	7.071622	7.224208	6.937752	7.296616	7.0148	7.248422	6.963615
Fkbp4	-0.364799	7.646827	-1.592083	0.132886	0.274191	-6.002624	7.92525	7.373443	7.43704	8.378306	8.200682	8.133044	7.257441	7.439156	7.536278	7.275304	7.637066	7.453727	7.599291	7.417548
Arl8b	-0.524301	8.897836	-1.59162	0.13299	0.274358	-5.9543	9.341262	8.241071	9.189355	10.06424	9.429947	9.855663	8.49208	8.503745	8.512803	8.374721	8.682886	8.532631	8.902886	8.449888
Pms2	0.266748	5.166572	1.591244	0.133075	0.274486	-5.953676	4.948303	5.487874	5.297299	4.930571	5.169141	4.874522	5.448228	5.255649	5.084445	5.318389	5.126156	4.997502	5.187345	5.250702
Lama5	0.783262	4.529642	1.590657	0.133207	0.274712	-5.83927	4.586045	5.59964	4.833729	3.075697	4.237722	3.68846	4.90017	4.384043	4.693673	4.879766	5.219768	4.735888	3.954679	4.70138
Psmb6	0.259814	2.966146	1.590543	0.133232	0.274718	-5.699901	3.357948	3.49955	3.458336	2.985885	3.482634	3.248852	2.717819	2.665313	2.797714	2.543858	2.843564	2.581351	2.689088	2.858134
Zfp458	-0.468952	1.804293	-1.590218	0.133306	0.274822	-5.450731	1.916502	1.769471	2.187895	2.57996	1.338932	1.892796	1.938115	1.628188	1.393785	1.355481	1.783193	1.826994	2.348615	1.755962
Paip2	-0.280246	6.439267	-1.589394	0.133492	0.275158	-6.022356	6.712622	6.055567	6.264576	6.676527	6.810737	6.526088	6.498561	6.22452	6.491579	6.261516	6.575137	6.208613	6.491223	6.370721
RGD1304810	-0.400681	2.808204	-1.588665	0.133657	0.275446	-5.636809	2.8647	3.184227	2.578448	3.247155	2.226624	2.734303	3.105363	2.888628	2.822431	2.600561	3.141068	2.475388	2.964355	2.70964
Srsf7	0.31665	5.842089	1.588572	0.133678	0.275446	-5.983704	5.18292	5.83579	5.490208	5.396005	5.736702	5.373446	6.472235	6.051963	6.270267	6.293039	5.702109	5.839671	5.879898	6.293559
Csnk2b	0.228084	6.789128	1.588106	0.133783	0.275548	-6.025515	6.94843	6.913229	7.047561	6.838656	7.426482	7.098829	6.571799	6.588208	6.674106	6.637628	6.763527	6.415274	6.578127	6.560282
Mnt	-0.280905	6.349257	-1.588093	0.133786	0.275548	-6.024844	6.587829	6.150913	6.783341	6.930152	6.262149	6.715386	5.959602	6.35191	6.096229	6.170806	6.327412	6.190936	6.148254	6.23416
Mfsd5	0.260368	5.381964	1.588025	0.133801	0.275548	-5.970969	5.317901	5.140412	5.477159	4.897366	5.579915	5.250499	5.236813	5.407759	5.534975	5.582203	5.419364	5.617939	5.363002	5.56021
Ythdf1	-0.27987	6.204615	-1.587948	0.133819	0.275548	-6.021376	6.230438	5.806804	6.27064	6.699183	6.213159	6.45019	6.046387	6.219389	6.172714	6.042377	6.32293	6.120179	6.280944	6.010707
Magi3	-0.248607	5.879687	-1.58759	0.1339	0.275668	-6.019464	6.334466	5.934106	6.317516	6.288903	5.815012	6.183672	5.78238	5.484625	5.589995	5.504473	5.757625	5.720069	5.965021	5.66475
Phospho2	-0.330055	4.620224	-1.587116	0.134007	0.275842	-5.938288	4.582799	4.091039	4.729737	5.190226	4.539721	4.867933	4.693279	4.332691	4.541944	4.472141	4.757751	4.539538	4.725704	4.683115
Mark3	0.198447	6.809628	1.586816	0.134075	0.275935	-6.026084	6.587829	6.926151	6.75713	6.546913	6.703337	6.676322	7.058049	6.870263	6.928665	7.021253	6.840795	6.731645	6.775276	6.925276
Atp6v1d	-0.329189	8.254427	-1.58585	0.134294	0.276319	-5.993986	8.677522	7.905164	8.538739	8.823303	8.735779	8.51048	8.015806	7.955068	8.21702	7.793283	8.296137	7.821233	8.290277	7.987407
Sf3b3	0.236807	6.405264	1.585789	0.134308	0.276319	-6.023437	6.007689	6.221501	6.537755	6.48344	6.481185	6.690707	6.454055	6.479728	6.465333	6.320727	6.419285	6.329488	6.388511	6.412897
Grk6	0.264585	6.265186	1.585429	0.13439	0.27644	-6.016918	6.121682	6.360364	6.196144	5.722283	6.261288	5.922643	6.591501	6.346036	6.462388	6.546763	6.24832	6.364232	6.126023	6.463717
Eif2s3x	0.199205	6.972103	1.584916	0.134507	0.276553	-6.029773	6.832266	7.065601	7.222353	7.191246	7.239488	7.316501	6.878052	6.785998	6.788294	6.767894	6.888888	6.847013	6.96882	6.82957
Tyk2	0.380773	5.678771	1.584836	0.134525	0.276553	-5.972805	5.154777	5.976134	5.34405	4.919588	5.30058	5.055522	6.148338	5.937877	6.039378	6.213469	5.664347	6.096917	5.581336	6.102907
Mis18bp1	0.668469	0.866733	1.584811	0.134531	0.276553	-5.271006	0.592497	1.21714	1.613009	1.63746	2.308867	1.993092	0.952703	-0.511025	0.245823	0.413143	0.805871	1.11804	0.249639	0.442608
Fam102b	-0.21986	5.784659	-1.584784	0.134537	0.276553	-6.003552	5.497569	5.469496	5.303227	5.886966	5.484345	5.64541	5.829598	6.090416	5.761172	6.079006	6.055417	6.283131	5.883878	5.744718
Fam49a	-0.600302	5.972795	-1.584609	0.134576	0.276587	-6.026826	6.252057	5.106215	6.388931	7.041233	5.959929	6.725749	5.532178	6.175018	5.746716	5.305655	5.983411	5.704031	6.296952	5.427521
Rbm17	0.219866	6.273746	1.584277	0.134652	0.276695	-6.029634	6.363131	6.560694	6.821528	6.745945	6.751311	6.965276	6.023363	5.973365	5.965013	6.072757	5.991341	5.904905	5.86521	5.849439
Acat1	-0.210445	6.801207	-1.584115	0.134689	0.276724	-6.031825	7.145566	6.82596	7.04314	6.935068	6.657641	6.793957	6.701655	6.779911	6.809313	6.821804	6.805894	6.576119	6.596838	6.738163
Asns	-0.446761	6.414233	-1.583967	0.134723	0.276745	-6.029439	6.361238	5.499448	6.621384	7.046291	6.402209	6.502884	6.206988	6.618735	6.506909	6.212333	6.638543	6.306496	6.72143	6.173301
Dctpp1	0.572358	0.513705	1.583804	0.13476	0.276775	-5.156513	1.143792	1.279848	1.363571	0.166125	1.20216	0.846031	0.429655	0.137473	0.449965	-0.116374	0.484915	0.218993	0.921344	-0.204094
Ptprz1	0.236539	8.945848	1.583076	0.134926	0.277032	-5.973778	9.301086	9.421814	9.359895	9.207167	9.518483	9.663586	8.66056	8.442856	8.510904	8.445113	8.360306	8.789462	8.742996	8.82094
Akirin1	-0.340546	5.126767	-1.583051	0.134931	0.277032	-5.988047	5.395808	4.595055	5.320864	5.482138	5.240728	5.223224	4.958988	5.140918	4.964647	4.806336	5.274637	5.053812	5.517869	4.845219
Hibch	-0.232143	4.199048	-1.582945	0.134955	0.277034	-5.876709	4.218773	4.055636	3.847136	4.324952	4.063101	4.213454	4.519061	4.203515	4.121609	4.298786	4.212217	4.281077	4.326276	4.187126
Dhx36	-0.263804	6.34278	-1.582304	0.135102	0.277242	-6.025475	5.899649	5.98086	5.969842	6.576584	5.928839	6.024907	6.961423	6.385514	6.768555	6.35615	6.472858	6.2148	6.553351	6.725575
Tanc2	-0.600918	6.421772	-1.582171	0.135132	0.277242	-6.031581	6.208491	6.28498	6.647246	7.278944	5.172806	6.47063	6.474359	6.820297	6.576675	6.019165	6.569852	6.350247	6.956905	6.093717
Wdr13	-0.236613	6.240841	-1.582124	0.135143	0.277242	-6.026673	6.448537	6.214496	6.193753	6.101401	5.692706	6.051319	6.387996	6.304236	6.41949	6.22027	6.218712	6.257386	6.504209	6.37831
Cpq	-0.362736	4.98108	-1.582097	0.135149	0.277242	-5.952794	5.202087	4.625556	4.496143	4.753862	4.522589	4.807235	5.317106	4.955841	5.216192	5.322609	4.711869	5.296777	4.855682	5.703024
Rqcd1	0.221505	5.589054	1.58143	0.135301	0.277494	-6.002388	5.369676	5.915712	5.652234	5.536351	5.730498	5.408679	5.657098	5.579795	5.483597	5.634055	5.682652	5.506344	5.615248	5.507551
Tmem194b	0.476482	2.927589	1.581357	0.135318	0.277494	-5.553331	1.9771	3.361401	2.877559	2.718219	2.682409	2.469996	3.270902	2.888628	3.367065	3.401235	3.03984	3.083214	2.736815	3.326676
Cep57l1	0.309832	2.937612	1.581022	0.135394	0.277584	-5.68827	2.753611	3.404252	3.324853	3.302317	3.114621	3.552622	2.629097	2.939445	2.639794	2.988286	2.604547	2.680061	2.575555	2.825198
Fam172a	-0.403826	5.530469	-1.580963	0.135408	0.277584	-6.025397	5.841078	5.159298	5.834161	6.577283	5.844035	6.382457	5.221722	5.204627	5.114779	5.084962	5.210093	5.181457	5.407919	5.397961
Selm	-0.334328	6.93699	-1.580798	0.135446	0.277605	-6.035608	7.131194	6.052203	6.466912	6.495325	6.925432	6.468453	7.088871	7.198305	7.268168	7.15623	7.382677	7.117996	7.339152	7.040359
Gsk3a	-0.253607	8.123716	-1.580716	0.135464	0.277605	-6.004493	8.651226	8.097226	8.603671	8.604154	8.353016	8.449082	7.663727	7.986369	7.948542	7.773382	8.073596	7.804578	8.029087	7.700098
Hist1h4b	-0.438854	3.314906	-1.580457	0.135524	0.277679	-5.770366	4.150251	2.97275	2.700244	3.225913	3.741449	3.536092	2.967416	3.40486	2.962454	3.608978	3.678847	3.555779	2.923692	3.142871
Pafah1b2	-0.491388	6.46196	-1.580326	0.135554	0.277686	-6.036731	6.793477	5.785351	6.783871	7.477365	6.760474	7.242444	6.092744	6.273619	6.143586	5.907019	6.425151	6.086832	6.608911	6.105195
Nudt18	-0.245178	5.278912	-1.580241	0.135573	0.277686	-5.998172	5.77861	5.167613	5.323783	5.217387	5.362868	5.139986	5.309988	5.378133	5.173599	5.194928	5.400772	5.143038	5.089637	5.2651
Itpkb	0.420212	5.416245	1.580009	0.135626	0.277747	-6.004396	5.999191	5.751179	6.153307	4.828572	5.673431	5.715595	4.855242	5.595724	4.768722	5.131403	5.524588	5.767143	5.264678	4.836982
Ptbp3	0.470597	3.371187	1.578889	0.135883	0.278225	-5.797115	3.380557	3.472958	4.634216	3.696477	3.746377	4.138786	2.943086	3.292072	2.772565	2.628098	2.977336	3.594258	3.254	2.825198
Vipr2	-0.566103	2.544644	-1.578062	0.136073	0.278464	-5.505168	2.753611	2.812308	1.792283	2.13961	2.030509	0.982423	2.629097	3.012464	2.858731	3.110461	2.86345	3.204252	2.676906	3.060363
Bfsp1	0.503876	1.089827	1.578024	0.136081	0.278464	-5.2329	1.212615	1.702379	1.451597	0.705659	1.143601	1.450626	1.335758	1.067371	0.97585	1.28803	0.998029	1.375373	0.249639	1.048408
Slc9b2	0.660894	3.715357	1.578013	0.136084	0.278464	-5.731912	3.279917	4.92435	3.495051	2.994284	3.409963	3.408717	4.371894	3.492551	4.15605	4.086912	3.273307	3.529544	3.45286	4.266966
Mir27b	0.566028	1.153433	1.577978	0.136092	0.278464	-5.289969	1.143792	2.248512	2.175023	1.21709	1.364797	1.165512	1.478026	0.76329	1.01926	0.893396	0.584127	1.186803	0.879431	0.753927
Gemin5	0.239375	4.56366	1.576834	0.136355	0.278954	-5.912628	4.315816	4.724685	4.785848	4.357872	4.361713	4.431711	4.812232	4.498613	4.639062	4.700066	4.64163	4.546096	4.621498	4.521176
Slc40a1	-0.616451	0.723666	-1.576089	0.136526	0.279219	-5.165561	1.310059	0.769921	0.56602	0.705659	0.027932	0.284862	1.511505	0.871929	1.489496	-0.027758	-0.318799	1.082389	1.252133	1.634478
Cbll1	-0.379158	5.211693	-1.57607	0.136531	0.279219	-6.020352	5.41236	4.96785	5.622912	6.273457	5.433312	5.833015	4.871449	4.952752	4.994863	4.677181	5.160721	4.750159	5.134872	4.922513
Dpcd	-0.258365	6.01465	-1.574823	0.136818	0.279759	-6.035567	6.344085	6.193272	5.761753	6.067969	6.095476	5.74346	6.088591	5.920591	6.00605	6.016563	6.211941	5.698154	6.075873	6.005816
Ndor1	0.399035	5.792195	1.574669	0.136854	0.279777	-6.009366	5.690875	6.197339	5.642915	5.046174	5.787786	5.428219	6.288911	5.767283	6.136211	6.138858	5.702109	5.696681	5.493764	6.102907
Meis3	0.435209	5.587783	1.574522	0.136888	0.279777	-6.008099	5.584532	6.452565	5.851456	5.086016	5.496095	5.273948	5.819609	5.452183	5.642993	5.788469	5.31852	5.594403	5.200171	5.701512
Map2	-0.421805	9.895276	-1.574292	0.136941	0.279777	-5.955531	10.27241	10.05071	10.18893	10.32515	9.256875	10.01294	10.00943	9.717811	9.871041	9.19107	9.699222	9.662614	10.56997	9.70739
Cpox	-0.25331	5.245647	-1.574283	0.136943	0.279777	-6.011438	5.288313	5.226505	5.256639	5.863086	5.335334	5.486734	4.96806	5.366574	5.166377	4.987339	5.050107	5.183562	5.176569	5.12553
Rab5a	-0.409948	5.039251	-1.574281	0.136943	0.279777	-6.027591	5.638683	4.889586	5.788409	6.302495	5.56186	5.995989	4.527267	4.544473	4.526983	4.272852	4.733642	4.613215	4.874019	4.315206
Cln3	0.33203	5.033269	1.573872	0.137038	0.279922	-5.93783	4.60537	4.951089	4.503885	4.383678	5.030587	4.81867	5.361385	5.204627	5.336908	5.504473	5.037033	5.212709	4.911337	5.653849
Chd6	-0.301866	7.016406	-1.573414	0.137144	0.280089	-6.046752	6.684883	7.055555	6.615441	7.091523	6.209595	6.468453	7.554038	7.147203	7.369137	7.203902	7.068811	7.181505	7.200292	7.392004
Ciao1	-0.228567	5.097017	-1.573317	0.137166	0.280089	-6.004615	5.296263	4.982063	5.213227	5.577462	5.34186	5.300289	4.92833	5.059983	4.930947	4.751052	5.065211	4.814046	5.128176	5.015475
Mtap	0.337634	2.307727	1.572917	0.137258	0.28023	-5.514101	2.144973	2.466372	2.853524	2.184345	2.460496	2.18908	2.433388	2.14364	2.364852	2.184529	2.360517	2.252925	2.168911	2.417223
Ly6g5b	0.893667	0.425852	1.572768	0.137293	0.28023	-5.015849	-0.518419	1.655851	0.307539	0.22441	1.018831	-0.006501	0.851758	0.705741	0.683763	0.847278	0.071497	0.28321	-0.123798	1.193114
Pkp2	-0.401199	5.851425	-1.572715	0.137305	0.28023	-6.023126	5.693887	4.637578	5.396283	5.924809	5.876985	5.710694	6.370998	6.08901	6.342541	6.021763	5.996978	5.687812	6.109121	6.090256
Gamt	-0.40718	5.475611	-1.571923	0.137489	0.280557	-5.998024	5.787097	5.259942	4.586407	4.969434	5.478434	4.559885	5.626867	5.663417	5.736998	5.954017	5.749633	5.816802	5.591107	5.916125
Clptm1l	0.248702	7.158311	1.570975	0.137708	0.280922	-6.049909	6.952837	6.769718	7.125317	6.573083	7.286761	6.863509	7.133904	7.28375	7.271537	7.426557	7.348249	7.453727	7.306412	7.432205
Mrgpre	-0.498442	2.490714	-1.57095	0.137714	0.280922	-5.451959	2.196854	1.117652	2.298852	2.124384	1.72577	1.450626	2.717819	2.976416	3.23521	2.586593	2.995472	3.145002	3.379583	3.237699
Frmd3	-0.500974	1.161791	-1.570817	0.137745	0.280937	-5.341725	1.764345	1.21714	1.687412	1.615848	0.88224	0.982423	0.903114	0.449407	1.255413	1.14286	1.167576	0.804622	1.983515	1.122575
Gpr108	0.329544	5.609727	1.570382	0.137846	0.281033	-5.981896	5.055231	5.620991	5.04656	4.751386	5.414861	4.850213	5.992576	5.899891	5.942639	6.203207	5.808521	6.076676	5.730244	6.177657
Adam19	-0.260757	6.058743	-1.570376	0.137847	0.281033	-6.034214	5.594237	5.593481	5.652234	6.279483	5.846331	5.623411	6.247289	6.478654	6.289232	6.306949	6.127087	6.3845	6.223471	6.20031
Crhr2	1.31805	1.513855	1.570312	0.137862	0.281033	-5.224209	2.474162	3.019437	2.250588	-0.941548	1.173177	1.193889	1.697535	0.923327	1.218642	1.804823	2.556491	0.760186	1.700256	2.087586
Iba57	0.420367	2.06314	1.570003	0.137934	0.281132	-5.409697	1.874641	2.439156	1.809043	1.478901	1.997824	2.116816	2.165439	2.099634	2.147322	2.438927	2.428842	2.220493	2.363894	1.684306
Cdkl1	-0.308476	3.742736	-1.569888	0.137961	0.281139	-5.803819	3.664706	3.702025	3.151703	3.791112	3.333437	3.547133	4.292269	3.852146	4.160904	3.765957	3.901965	3.686193	4.013992	3.655003
Arl1	-0.449235	5.864465	-1.568541	0.138274	0.281699	-6.04669	6.037635	5.175881	6.102375	6.744079	6.017113	6.468453	5.482396	5.829227	5.745101	5.553767	5.845237	5.681868	6.02512	5.422024
Ptafr	0.477455	4.398661	1.568505	0.138282	0.281699	-5.908086	4.342933	4.051149	4.815182	3.963576	4.735811	4.8591	3.36548	5.028055	3.197914	4.830243	4.845355	5.765739	4.004274	3.858608
Trap1	0.181453	6.218356	1.568286	0.138333	0.281754	-6.04399	6.071612	6.219503	6.233097	5.999729	6.369047	6.045492	6.338721	6.238539	6.39601	6.233777	6.174622	6.078939	6.336214	6.342873
Pwwp2a	-0.225992	5.7431	-1.568167	0.138361	0.281763	-6.026865	5.613453	5.570149	5.505713	5.758485	5.194602	5.575685	6.027708	5.903095	6.01546	6.007418	5.946597	5.799066	5.688377	5.827432
Champ1	-0.360754	5.616561	-1.567848	0.138435	0.28183	-6.040619	5.863991	5.030736	5.863541	6.264804	5.860036	6.053257	5.314737	5.599679	5.253025	5.341446	5.641494	5.619495	5.646821	5.311421
Ctif	-0.388227	4.162557	-1.567824	0.138441	0.28183	-5.938229	4.432216	4.175923	4.69409	4.858787	3.823012	4.339463	3.850657	4.078726	4.01306	3.545408	4.144751	4.065203	4.546481	3.797658
Serpina11	0.745507	-0.461178	1.567692	0.138472	0.281845	-4.997486	-0.129637	1.453108	0.105278	-0.325128	-0.107758	0.527154	0.028455	-1.196868	-0.27702	-1.33445	-0.855515	-0.78301	-0.893866	-0.397871
Zc3h12c	-0.542653	2.494074	-1.567298	0.138563	0.281984	-5.632654	2.569081	1.914859	3.111733	3.342347	1.85913	2.772436	2.12275	2.875639	2.166756	2.221154	2.604547	2.447635	2.902922	2.294252
Hapln3	-0.495942	1.312669	-1.567147	0.138599	0.282008	-5.301042	1.619793	1.369077	0.642822	1.353927	0.731352	1.327961	2.100922	1.020936	1.579251	1.73055	0.924208	1.375373	1.521045	1.73247
Xab2	0.239955	5.760438	1.566744	0.138693	0.28214	-6.031418	5.646478	5.94382	5.885437	5.559177	5.912492	5.627317	5.801115	5.817389	5.938405	5.956735	5.66151	5.702564	5.42623	5.796614
Pnkp	0.374498	5.150234	1.566668	0.13871	0.28214	-5.985798	5.108122	5.467813	5.211652	4.48252	5.258083	4.719677	5.463276	4.998404	5.495173	5.508182	4.967529	5.103568	4.957391	5.407263
Trpv6	0.252887	4.843331	1.566386	0.138776	0.282226	-5.953235	4.481855	5.161381	4.773092	4.632654	4.542557	4.781755	5.035778	4.729108	5.05867	4.85376	4.781463	4.883016	4.919116	5.227782
LOC685909	-0.452863	1.702108	-1.565608	0.138958	0.282522	-5.441137	2.246933	1.310208	1.472789	1.874319	1.930143	1.708392	0.952703	2.030999	1.769222	1.780488	1.883989	1.643459	1.521045	2.194616
Etf1	0.347949	5.931625	1.565562	0.138969	0.282522	-6.050483	5.957189	5.864205	6.705473	6.278624	6.563759	6.728176	5.640171	5.518445	5.627297	5.437958	5.812363	5.740228	5.858483	5.336959
Agap2	-0.259937	11.22422	-1.565263	0.139039	0.282617	-5.929705	11.48969	10.90018	11.11114	11.0169	10.80266	11.17147	11.50774	11.17776	11.30076	11.42328	11.07973	11.35349	11.39722	11.40765
Mon1a	-0.211977	5.104507	-1.564694	0.139172	0.282839	-6.003527	5.3218	5.095359	4.905621	5.160679	5.088003	5.030171	5.214116	4.968131	5.056067	5.324714	5.170732	4.928903	5.059455	5.185153
LOC689064	0.647777	2.551183	1.564458	0.139227	0.282904	-5.726676	3.516065	3.445867	3.336461	2.534609	4.565043	3.242073	1.782265	1.775313	2.995434	2.165862	2.117203	1.045835	1.521045	1.991897
Oxsm	-0.310273	3.821544	-1.564114	0.139308	0.282979	-5.898921	4.335237	3.747109	4.229782	4.207046	4.195107	3.648009	3.504983	3.567159	3.689436	3.456042	3.972332	3.581545	3.969738	3.510555
Msln	3.416997	-1.06143	1.564099	0.139311	0.282979	-4.76078	0.995438	-1.463779	2.635942	-3.260591	-0.424712	3.502454	0.563361	0.137473	-0.504027	-1.136114	-3.260591	-3.260591	-1.047095	-0.505482
Stard8	0.324452	3.827937	1.562806	0.139614	0.283547	-5.857159	4.150251	4.037603	3.933744	3.431645	4.241217	4.188991	3.769961	3.466799	3.748669	4.020928	3.480655	3.875144	3.542874	3.814538
Ppm1l	-0.372909	6.432032	-1.562062	0.139789	0.283854	-6.059204	6.07739	6.090972	6.459633	7.03514	5.745343	6.44208	6.269474	6.493609	6.417463	6.544055	6.532299	6.701118	6.654666	6.603819
Nudcd3	-0.470054	5.115613	-1.561492	0.139923	0.284066	-6.003261	5.066895	4.146778	5.098612	5.480644	4.587184	5.36723	5.044381	5.505855	5.132184	4.850841	5.338195	5.365131	5.736127	4.945683
Parm1	-0.479245	3.774252	-1.561416	0.139941	0.284066	-5.912154	4.105711	3.151065	4.503885	4.703523	3.913413	4.101824	3.094359	3.583227	3.518449	3.186517	3.441501	3.827486	3.974722	3.853172
Rbm26	-0.324791	6.082326	-1.56102	0.140034	0.284207	-6.056552	5.877301	5.809463	6.021091	6.712608	5.818526	6.237487	6.264573	6.249908	6.210659	5.751387	6.095906	5.941432	6.210852	5.974866

LOC497940	0.45246	1.890033	1.560916	0.140059	0.284209	-5.500787	2.263247	2.801663	1.905712	1.740915	2.448452	2.644629	1.607516	1.80302	1.520039	1.569444	1.697158	2.100868	1.040235	1.755962
Nrd1	-0.205714	7.664448	-1.560758	0.140096	0.284236	-6.055225	7.587451	7.450428	7.434679	7.928439	7.562963	7.758077	7.884217	7.656449	7.752271	7.579404	7.684301	7.608606	7.697537	7.725235
Slc15a4	-0.339483	4.711897	-1.560436	0.140172	0.284342	-5.951824	4.73969	4.134105	3.918379	4.569411	4.708296	4.504319	4.965043	4.97728	4.939446	4.588849	4.698092	5.251348	4.929422	5.105095
Cntnap5c	-0.539053	1.590004	-1.55988	0.140303	0.284528	-5.329848	1.48718	1.151583	1.220561	1.679737	0.422095	1.473956	2.01015	2.522625	1.891068	1.481601	2.031907	1.593679	2.11575	1.73247
Grin2d	-0.283209	4.261815	-1.559847	0.14031	0.284528	-5.837983	3.870393	3.582736	3.353699	3.795925	3.886882	3.39656	4.912754	4.620407	4.693673	4.643844	4.394114	4.492777	5.00202	5.109662
Krt8	2.380063	1.192736	1.559667	0.140353	0.284565	-4.917573	3.136736	-0.273517	1.116684	-3.260591	1.415175	1.976852	1.297885	1.883083	0.931093	1.964563	3.448101	2.153355	1.943396	1.684306
Sin3b	0.294293	6.166364	1.559001	0.14051	0.284836	-6.052551	5.967183	6.42335	6.471526	5.723547	5.947145	5.711921	6.334051	6.336588	6.405243	6.288731	6.139374	6.131125	6.200251	6.271316
Gsto1	0.290733	6.698038	1.558527	0.140622	0.285015	-6.065783	6.627752	6.356732	6.735374	6.344985	7.103045	6.909687	6.824949	6.70754	6.880926	6.845457	6.788446	6.580917	6.469316	6.612693
Mccc2	-0.255081	5.374719	-1.557955	0.140757	0.285241	-6.026017	5.648032	5.359475	5.454701	5.233442	4.951718	4.965803	5.49291	5.4974	5.389549	5.362092	5.459115	5.542871	5.404229	5.523038
Scpep1	0.408883	5.744287	1.557625	0.140835	0.28535	-6.031736	5.576394	5.540043	5.304705	5.091898	5.979941	6.130528	5.511644	6.307866	5.481658	6.148398	5.966271	6.833003	5.091933	5.48664
Atp6ap1	-0.266158	9.266929	-1.557181	0.14094	0.285515	-6.016738	9.173106	8.619828	8.974371	9.342131	9.190291	9.18374	9.201522	9.349709	9.533566	9.398679	9.310685	9.41317	9.3732	9.6756
Ascl2	0.652476	1.470985	1.556875	0.141012	0.285614	-5.265403	0.785575	2.100024	1.514261	1.065902	1.664612	1.046069	2.227194	1.020936	1.60797	2.027955	1.456531	1.593679	1.040235	2.030937
Fbxw8	0.270753	6.679469	1.556525	0.141095	0.285733	-6.06159	6.047088	6.610339	6.425395	6.060988	6.170717	6.093352	7.298774	6.845496	7.244356	7.039313	6.675572	6.801145	6.853131	7.363134
Acsl5	-0.368097	6.920351	-1.556378	0.14113	0.285756	-6.070813	6.76225	5.751179	6.630252	7.034631	6.885229	6.852426	7.041672	7.110254	7.140065	7.076643	7.086898	7.09078	7.219842	7.216323
Vrk1	0.353447	4.140275	1.555652	0.141302	0.286043	-5.877899	3.481621	4.200444	4.051108	4.203428	4.39974	4.188991	4.531353	4.213873	4.203867	4.357545	4.188764	4.018805	3.719553	4.295302
Lrrc49	-0.233581	6.81713	-1.555578	0.14132	0.286043	-6.072189	7.017981	6.575611	7.034258	7.252927	6.95978	7.038006	6.747159	6.723033	6.752567	6.703827	6.810383	6.582513	6.7717	6.484082
Slc30a4	-0.308813	6.636093	-1.554662	0.141537	0.286393	-6.072921	6.573999	6.082202	6.753887	7.045281	6.411545	6.755207	6.628208	6.611898	6.441597	6.480327	6.752905	6.805251	6.876624	6.702334
D2hgdh	-0.245582	4.13172	-1.554649	0.14154	0.286393	-5.878884	4.231267	4.023929	3.679531	3.815017	3.706477	3.698398	4.33497	4.429191	4.425765	4.353427	4.427894	4.200226	4.290742	4.319155
Sgce	-0.290549	5.744985	-1.554442	0.141589	0.286399	-6.057103	5.831537	5.261885	5.597812	6.079857	5.922323	5.877432	5.443899	5.869094	5.722297	5.864239	5.540116	5.788042	5.731717	5.9291
Sec61a2	-0.269604	6.947866	-1.554435	0.141591	0.286399	-6.074003	6.821978	6.590376	6.71662	7.353068	6.854752	7.10911	7.166451	6.760611	7.217817	6.934998	6.796227	6.850991	6.861229	7.248769
Mapk14	0.198063	6.864222	1.554107	0.141669	0.286458	-6.072759	6.444072	6.80679	6.867258	6.691456	6.704603	6.68572	7.213617	6.907432	7.050055	7.018004	6.897955	6.89018	6.847028	7.088573
Zfp410	0.184836	4.948511	1.554066	0.141679	0.286458	-5.993547	4.771404	5.068969	5.026994	4.84492	4.890715	4.988255	5.097706	4.915162	4.950701	4.894014	5.078034	4.95131	4.908735	4.943127
Ubl4a	-0.25191	5.678723	-1.554012	0.141692	0.286458	-6.058623	6.100273	5.81609	5.667249	5.831792	5.960989	5.413212	5.586203	5.541244	5.56641	5.378399	5.748297	5.449758	5.847656	5.625434
Adcy5	-0.258658	7.030551	-1.553832	0.141734	0.286496	-6.074868	7.1273	6.801456	6.644913	6.739714	6.407662	6.846293	7.555544	6.976735	7.236905	7.187222	6.819956	7.216359	7.387612	7.492448
Tbccd1	-0.26687	4.082326	-1.553207	0.141883	0.286748	-5.910461	4.247758	3.582736	3.871277	4.207046	4.180618	4.192511	4.134688	4.090016	4.189688	3.88487	4.227643	4.078836	4.278701	4.079517
RGD1308147	-0.367718	4.188716	-1.553053	0.14192	0.286774	-5.959992	4.536569	3.719097	4.275724	4.756333	4.427618	4.680104	4.123906	3.942114	4.060584	3.696121	4.334806	3.880344	4.47088	3.825682
Bag2	-0.406887	3.93489	-1.552694	0.142005	0.286899	-5.939333	4.247758	3.390109	4.008208	4.72387	4.241217	4.672562	3.727855	3.683523	4.01306	3.622732	3.944596	3.56872	3.83917	3.510555
Commd8	-0.205595	5.453254	-1.552216	0.142119	0.287078	-6.058861	5.824683	5.472854	5.558705	5.948777	5.878108	5.816	5.273864	5.077105	5.202134	5.271138	5.250298	5.162375	5.312711	5.333059
Hspe1	0.357353	5.707646	1.552121	0.142141	0.287078	-6.070156	5.919085	6.011209	6.243923	6.374242	6.74763	7.014831	5.402117	4.927801	5.328305	5.27332	5.210093	5.011787	5.33614	5.138994
Hmox2	-0.270168	6.627493	-1.55196	0.14218	0.287107	-6.077672	7.002272	6.366697	6.87225	7.050829	7.10016	6.686344	6.33171	6.466794	6.538022	6.39974	6.711675	6.31324	6.53912	6.422104
Bend5	-0.268919	3.246184	-1.551688	0.142245	0.28715	-5.819321	3.707065	3.439014	3.693138	3.622545	3.273255	3.165332	3.003159	3.000548	3.23521	3.14899	3.235656	2.838051	3.013624	3.237699
Fndc1	-0.506922	2.52828	-1.551542	0.142279	0.28715	-5.614145	3.127854	2.134368	2.122353	2.389316	1.78444	2.915623	2.518182	2.619301	2.508863	3.241039	2.303426	2.816513	2.285827	2.91141
Ccnd2	-0.524583	5.573692	-1.551494	0.142291	0.28715	-6.066507	5.922941	5.095359	6.124304	6.546913	5.627431	5.986896	5.015502	5.456551	5.20918	4.725784	5.762929	5.327557	6.117033	5.147901
Rpl12	1.224835	2.405995	1.55147	0.142297	0.28715	-5.348821	1.212615	2.117298	4.11989	1.065902	1.85913	1.107027	2.415815	3.734773	2.381582	2.529327	2.696109	2.715403	3.318158	2.768575
Ppp1r21	-0.187617	5.798647	-1.550702	0.14248	0.287472	-6.057723	5.849207	5.540043	5.646417	5.746104	5.816184	5.435664	5.928957	5.893462	5.934159	5.858438	5.919544	5.86743	5.942299	5.831584
Cct7	0.18865	7.614779	1.548969	0.142894	0.28817	-6.071964	7.566651	7.574431	7.796906	7.672524	7.930778	7.931616	7.55052	7.504951	7.606451	7.513103	7.501262	7.444542	7.552549	7.468652
Arhgdig	-0.217954	5.392243	-1.548936	0.142902	0.28817	-6.022268	5.388389	4.96785	5.120598	5.0052	5.062547	4.75105	5.796029	5.543299	5.925628	5.532066	5.43606	5.433932	5.785187	5.782385
Lhpp	-0.227798	6.118278	-1.548859	0.142921	0.28817	-6.080747	6.907499	6.735869	6.449647	6.563237	6.544085	6.301641	5.471805	5.763762	5.722297	5.812672	5.905225	5.911269	5.799304	5.790939
Entpd2	0.39474	5.996002	1.548854	0.142922	0.28817	-6.05419	5.924225	5.960666	5.469272	5.119037	6.277549	5.937404	5.754688	6.495733	5.808336	6.3867	6.038023	6.816145	5.866551	6.115448
Eif2ak1	-0.274262	6.149252	-1.548723	0.142953	0.288185	-6.077039	6.339284	5.796117	6.251607	6.556162	6.084816	6.560549	5.970175	6.198678	6.148482	5.880073	6.11262	5.969758	6.218227	6.025281
Rab6a	-0.41175	9.413626	-1.548547	0.142995	0.288221	-6.002207	9.819607	8.936062	9.727884	10.23367	9.844666	9.955637	9.187674	9.211656	9.205654	8.825459	9.411954	8.988571	9.529575	8.915083
Noc2l	0.402339	7.851965	1.547435	0.143262	0.28871	-6.077159	7.323844	8.346455	7.6782	7.442876	7.560517	7.550522	8.442769	7.904009	8.225443	8.210525	7.622187	7.749525	7.534517	8.343179
Idh3B	-0.193151	7.293549	-1.546643	0.143452	0.289045	-6.078753	7.55303	7.303009	7.345941	7.604884	7.578244	7.316098	7.278255	7.012033	7.322246	7.022551	7.298443	6.998155	7.243784	7.243065
Lztfl1	-0.267181	4.227769	-1.546423	0.143505	0.289103	-5.948101	4.478365	3.889638	4.210987	4.390058	4.424547	4.127796	4.477313	4.044317	4.076084	4.290193	4.427894	4.018805	4.125768	4.291288
Smarcd3	0.380343	6.170426	1.54617	0.143566	0.289135	-6.066343	5.922941	6.60346	6.080971	5.498469	5.904799	5.768441	6.72146	6.053406	6.562072	6.486922	6.052172	5.946399	6.063059	6.744048
Csnk1g1	-0.65425	2.026026	-1.546155	0.143569	0.289135	-5.566229	1.831529	1.58312	2.538805	3.275	1.415175	2.160607	2.305626	2.099634	2.653634	1.678822	1.883989	1.568131	2.452318	1.32462
Arfgap1	0.290952	6.279991	1.545984	0.14361	0.28917	-6.067953	5.871991	6.514992	6.14508	5.649696	5.766114	5.779016	6.688909	6.540656	6.590232	6.635086	6.455767	6.37348	6.383834	6.546035
RGD1562658	-0.854949	3.371219	-1.545449	0.143739	0.28938	-5.841965	5.105862	2.184396	2.939761	2.899083	3.813652	3.456346	3.169687	3.440579	2.822431	2.998871	4.585863	3.245131	3.6527	3.050898
Itpripl1	-1.078926	1.427444	-1.545233	0.143791	0.289437	-5.578569	4.53321	1.339943	1.705429	1.158502	2.109124	1.585258	0.498056	0.449407	0.571595	0.981432	2.696109	1.315214	0.791767	0.929479
Adprhl2	0.281082	5.007274	1.544169	0.144047	0.289904	-5.978987	4.637014	4.989118	4.847991	4.361123	4.85009	4.422747	5.359088	5.275563	5.259827	5.398527	5.419364	5.042261	4.992221	5.29748
Cftr	-1.382717	1.647746	-1.54356	0.144194	0.290151	-5.577092	1.459129	4.150978	0.398943	4.242735	1.463853	0.61372	1.372662	0.137473	2.846732	2.586593	1.347792	1.542121	0.249639	1.32462
Eef2k	0.238579	5.831229	1.543031	0.144322	0.290359	-6.067064	5.885228	6.144609	5.861534	5.410199	5.797314	5.732621	5.826276	5.893462	5.715715	5.908424	5.81747	6.01419	5.701967	5.955973
Tcaim	-0.251812	4.037644	-1.542402	0.144474	0.290604	-5.900432	3.987945	4.03306	3.674966	4.109897	3.891338	3.62735	4.3855	3.852146	4.457672	4.061889	4.168924	3.931333	4.353321	4.088797
Bbx	-0.513474	2.557639	-1.542326	0.144492	0.290604	-5.649616	2.41698	2.383133	2.726949	3.288723	1.556544	2.772436	2.613764	2.781287	2.905753	2.342482	2.213317	2.36102	3.178239	2.544088
Pusl1	0.35199	3.822266	1.541265	0.144749	0.291072	-5.876473	3.932918	4.279295	4.429962	3.504019	3.680967	3.844276	3.632178	3.599118	4.034376	3.832569	3.621611	3.875144	3.488149	3.869418
Gsn	0.579905	8.005128	1.540739	0.144876	0.291259	-6.087297	7.578108	8.507425	7.3556	6.935614	8.386346	7.089419	8.043605	8.893058	7.980467	8.293749	7.943306	8.856953	7.776731	8.438136
Rtcd1	0.172433	5.460038	1.540679	0.144891	0.291259	-6.070135	5.600671	5.738676	5.794734	5.712135	5.983075	5.799936	5.26653	5.127283	5.25075	5.176396	5.233205	5.136534	5.359196	5.29748
Cep89	0.309674	4.513905	1.540444	0.144947	0.291325	-5.961579	4.442997	4.989118	4.475294	4.1518	4.614391	4.348958	4.8055	4.264575	4.666626	4.785087	4.366508	4.53295	4.398536	4.421864
Exosc3	-0.392383	2.621768	-1.539941	0.145069	0.291522	-5.687912	2.810225	2.064844	2.548818	3.145235	2.507694	3.22842	2.8014	2.555575	2.508863	2.359011	2.750518	2.726993	2.452318	2.503027
Ywhah	-0.492848	10.56562	-1.539573	0.145158	0.291652	-5.970571	10.94441	9.87382	10.82409	11.48148	11.14163	11.04544	10.14934	10.43929	10.49572	10.0134	10.65515	10.1351	10.68768	10.03321
Prkaa1	-0.366125	5.6555	-1.539032	0.14529	0.291867	-6.082292	5.7427	5.296419	5.803125	6.445656	5.592283	6.221207	5.816264	5.154424	5.705785	5.416404	5.696576	5.285063	5.553287	5.479602
Itpa	-0.233456	6.108476	-1.538349	0.145456	0.292151	-6.085973	6.241803	5.662767	6.008449	6.040856	5.99658	5.959266	6.229796	6.157685	6.32535	6.219139	6.459038	5.997539	6.226609	6.016797
Ccdc130	0.207569	5.070973	1.537829	0.145582	0.292325	-6.040437	4.983193	5.324578	5.285371	5.206584	5.372461	5.23522	5.092185	4.943446	5.048229	5.092395	4.88724	4.744468	4.780817	5.044382
Xrcc6bp1	0.347718	2.974096	1.537793	0.145591	0.292325	-5.676475	2.896425	3.479652	2.901201	2.451642	2.692656	2.998397	2.979429	3.19084	3.069578	3.276274	3.220315	3.170693	2.676906	2.83626
RGD1310313	-0.283916	7.797245	-1.537313	0.145708	0.292438	-6.086347	7.86673	7.169653	7.582133	7.884774	7.864054	7.755105	7.703197	7.646447	7.891646	7.949469	7.848582	7.975263	7.84297	8.188559
Pik3c2a	-0.23248	5.630401	-1.537229	0.145728	0.292438	-6.079873	5.809488	5.601176	5.755281	5.977659	5.380408	5.9088	5.859156	5.416754	5.50284	5.412451	5.27092	5.499602	5.740523	5.722532
Rasa3	-0.191067	6.250583	-1.537224	0.145729	0.292438	-6.096559	6.630899	6.422481	6.539011	6.690164	6.425437	6.491499	5.961117	6.056287	6.078382	5.827594	5.996978	6.031821	6.335245	6.042099
Tatdn3	0.477116	4.529216	1.537162	0.145744	0.292438	-5.943152	4.082914	5.259942	4.852881	4.074684	4.598129	4.090548	5.032899	4.729108	4.890608	4.844986	4.444493	4.308338	4.080242	4.734278
Tpm1	-0.237915	7.711506	-1.536467	0.145914	0.292729	-6.083335	7.993763	7.541619	7.652891	8.097077	7.949327	8.08674	7.634586	7.710939	7.697788	7.558982	7.602724	7.418419	7.57077	7.453016
Kcnk5	-0.506948	1.239536	-1.535471	0.146156	0.293167	-5.28623	1.278303	0.533043	0.945338	1.21709	0.422095	1.107027	1.809437	1.49863	1.218642	1.898256	0.998029	1.618784	1.747621	1.755962
Smim1	-0.522094	1.048806	-1.535144	0.146236	0.293278	-5.325379	2.035255	1.151583	1.004744	0.823477	0.808768	0.61372	1.511505	1.464337	1.01926	1.023513	0.998029	1.152831	0.836265	1.009844
Tmem80	-0.283561	5.63578	-1.534366	0.146426	0.29361	-6.079235	5.933174	5.5207	5.030571	5.666875	5.768538	5.750642	5.528095	5.543299	5.678967	5.849693	5.648674	5.627247	5.773793	5.612623
Thada	-0.200124	4.745101	-1.533641	0.146604	0.293891	-5.986609	4.539921	4.462789	4.308508	4.613698	4.475885	4.274516	5.078288	5.045558	5.109768	4.992645	4.92558	4.789383	4.919116	4.955862
Rbm47	1.052787	0.466543	1.533593	0.146615	0.293891	-5.373214	3.073383	1.972946	2.126301	-0.600663	2.109124	2.217002	-2.088409	0.137473	-1.813089	0.750369	2.083688	0.930264	-0.628995	-1.632384
Ube4b	-0.236793	7.117913	-1.533359	0.146673	0.293957	-6.103339	6.872036	6.740056	7.134073	7.46684	6.888576	6.966818	7.291566	7.185863	7.234029	7.059048	7.21238	7.109719	7.308881	7.192307
Zdhhc23	-0.292226	3.829274	-1.53319	0.146714	0.29399	-5.791718	3.25566	2.747223	3.228468	3.538889	3.122223	3.321383	4.443022	4.424739	4.384862	4.111508	4.33124	4.257294	4.416965	4.147707
Calhm2	-0.401639	3.797776	-1.532943	0.146774	0.294063	-5.763674	3.502386	2.519311	2.787402	3.099251	3.452798	2.800388	3.763028	4.402272	4.421726	4.690302	4.647377	4.848943	4.152409	4.212819
Med1	-0.438502	6.087951	-1.53248	0.146888	0.294241	-6.100795	6.200041	5.726064	6.574979	6.943226	5.716756	6.514884	5.827938	6.2258	5.835912	5.611814	6.02263	6.036486	6.350666	5.66785
Abhd15	0.571815	1.388312	1.532353	0.146919	0.294254	-5.35578	1.278303	2.248512	1.451597	1.097434	1.745593	1.585258	0.625837	1.958936	0.837186	1.355481	1.629088	2.715403	0.698429	0.844371
Wnt7a	-0.327468	5.070596	-1.53215	0.146969	0.294292	-6.036622	5.247895	5.155123	4.4568	4.982159	4.715852	4.839026	5.188472	4.989389	4.775067	5.162339	5.219768	5.482608	5.442513	5.379173
Rab5c	0.214216	8.005773	1.532077	0.146987	0.294292	-6.074563	8.421354	8.36035	8.585849	8.440715	8.756688	8.880928	7.384276	7.643576	7.475238	7.64279	7.764819	7.702597	7.590388	7.437663
Rnf103	-0.309022	5.407352	-1.531945	0.147019	0.294307	-6.065657	5.119368	4.625556	4.975957	5.594136	5.150675	5.444548	5.521948	5.412264	5.653363	5.599695	5.362854	5.633419	5.752183	5.895119
Pnma1	-0.466431	1.752487	-1.531756	0.147065	0.294351	-5.487535	2.035255	0.812964	1.705429	2.093442	1.840817	2.00915	1.335758	1.80302	2.066858	1.597576	2.133674	1.805289	1.881043	1.889408
Wdyhv1	0.411819	3.846405	1.531061	0.147236	0.294607	-5.885861	3.676936	4.897106	4.044046	3.659984	3.691225	3.727807	3.993741	3.583227	3.935874	3.808701	3.73929	3.71558	3.689548	3.797658
Parg	-0.319005	5.435749	-1.531035	0.147242	0.294607	-6.085231	5.504448	5.161381	5.532452	6.182661	5.462053	5.946814	5.229287	5.326567	5.277813	5.044659	5.512044	5.308397	5.359196	5.289453
Ccdc153	1.145938	2.365213	1.530921	0.14727	0.294614	-5.380754	2.035255	1.248835	2.10888	0.33436	3.068148	2.493017	2.828218	3.330656	2.539002	1.21727	2.049375	3.170693	3.665088	3.410483
Prpf38b	0.262975	7.117334	1.530344	0.147412	0.294848	-6.103968	6.990071	7.688253	7.396028	7.558906	7.451968	7.607998	6.997313	6.979773	7.011612	7.013444	6.769468	6.784601	6.577308	6.827493
Mpzl1	0.263418	6.29155	1.530143	0.147462	0.294899	-6.098193	6.166823	6.437172	6.243153	5.892594	6.478226	6.011019	6.132288	6.312693	6.397039	6.549466	6.154586	6.681891	6.265772	6.379256
Adal	0.354996	3.152013	1.530023	0.147491	0.294908	-5.683653	2.501925	3.411272	2.885483	2.797152	3.075998	2.705027	3.691803	3.386664	3.416608	3.241039	3.273307	3.065063	3.203938	3.655003
Tnni3	0.850573	1.206957	1.52973	0.147563	0.295001	-5.20948	1.245833	1.833582	1.408255	-0.243853	1.390206	0.481842	1.697535	2.38274	0.931093	1.104165	1.864385	1.186803	0.836265	1.529341
Cyp19a1	-0.586497	0.540475	-1.529636	0.147586	0.295001	-5.255032	0.915118	1.082904	0.56602	1.273392	0.606834	-0.006501	0.68572	0.645799	1.255413	-0.210791	0.676952	-0.334482	1.150071	0.383504
Wbp1l	0.190671	6.672478	1.52941	0.147642	0.295062	-6.106976	6.504506	6.563059	6.803868	6.52389	6.765337	6.686344	6.51928	6.692806	6.434599	6.86873	6.778661	6.976469	6.708771	6.603819
Lemd2	0.304546	5.639268	1.528658	0.147827	0.295383	-6.050037	5.154777	5.717595	5.165217	4.897366	5.359656	5.082333	6.094126	5.85923	5.918481	6.143636	5.715848	6.007077	5.671591	6.196023
Prosc	-0.269534	4.633587	-1.528535	0.147857	0.295395	-6.0111	4.637014	4.522355	4.678213	4.883868	4.589928	4.185462	4.8055	4.792678	4.614504	4.879936	4.526331	4.847553	4.6173	
Ccdc125	0.360783	2.851562	1.527077	0.148217	0.296043	-5.625849	2.279379	3.028596	2.718102	2.451642	2.752661	2.434761	2.905804	3.559057	2.639794	3.728285	3.173291	3.38339	2.408783	2.685374
Pck2	0.4127	5.321811	1.52702	0.148231	0.296043	-6.03505	4.995452	5.94503	5.037699	4.580611	5.109198	4.71479	5.688539	5.385024	5.756369	5.672584	5.434399	5.275228	5.266712	5.684779
Chm	-0.327012	4.425488	-1.526474	0.148366	0.296263	-6.030383	4.640141	4.305905	4.767325	5.26155	4.525459	4.911303	4.32559	4.193082	4.268256	3.62956	4.180861	4.056042	4.704856	4.266966
Fam89a	0.430946	3.062023	1.525936	0.148499	0.296479	-5.70119	3.00712	3.24835	2.744481	2.376521	3.517641	2.940957	3.383673	2.571773	3.375441	3.424979	2.90242	3.204252	3.013624	3.35002
Med7	-0.362646	3.662576	-1.524953	0.148742	0.296916	-5.962796	4.158996	3.404252	3.989949	4.546747	3.994346	4.565325	3.677127	3.19084	3.197914	3.52358	3.265855	3.179156	3.423994	3.286911
Gal3st1	-0.601688	3.163964	-1.524456	0.148865	0.297016	-5.675228	3.188908	2.924502	1.825611	2.591079	3.144791	1.353343	3.699086	3.395791	3.689436	3.709073	3.568082	4.018805	3.549571	3.847716
Ets2	-0.193012	5.871775	-1.524221	0.148923	0.297179	-6.101204	5.745606	5.691883	5.809385	6.180822	5.787786	5.797626	5.855901	5.959582	5.7739	6.017865	5.8956	6.051545	5.871905	5.79236
Kansl2	-0.215217	5.625986	-1.523776	0.149034	0.297316	-6.090074	5.651135	5.200402	5.499273	5.738624	5.52075	5.694648	5.79433	5.829227	5.685718	5.746684	5.652966	5.547781	5.660807	5.573492
Prmt1	0.691131	3.001937	1.523745	0.149042	0.297316	-5.667231	1.80948	3.655486	2.820842	3.075697	2.856578	3.353184	3.593621	3.431732	2.024877	2.889346	3.394437	2.643831	3.40196	3.286911
Zeb1	0.311803	5.848008	1.52336	0.149137	0.297457	-6.101793	6.026926	6.502721	6.114225	5.601028	5.933168	5.946814	5.880133	5.473892	5.861481	5.890059	5.244623	5.893379	5.692921	5.838478
Tspan18	-0.686374	5.163249	-1.5231	0.149201	0.297536	-6.056472	5.304169	3.125682	5.458689	5.4149	5.58405	4.458273	5.146469	5.245588	5.330461	5.511883	5.602089	5.439227	5.310741	5.403549
Kcnj2	-0.509865	2.049189	-1.522839	0.149266	0.297616	-5.499596	1.80948	1.082904	1.705429	2.337441	1.20216	2.31068	2.305626	2.76729	2.508863	2.108365	2.317912	2.529342	2.236876	1.868007
Spopl	-0.431485	2.768667	-1.522623	0.14932	0.297674	-5.726075	2.595092	2.425352	2.735741	3.50989	2.226624	3.172479	3.026505	2.964197	2.680923	2.484835	2.66245	2.960934	2.902922	2.648193
Tas1r1	0.427348	3.115205	1.522447	0.149364	0.297688	-5.713451	3.016775	3.643613	3.365078	2.43939	2.901447	2.665044	3.562015	3.221962	3.495578	3.232094	2.986432	2.794648	3.125428	3.35002
Sfmbt1	-0.217434	5.130687	-1.522394	0.149377	0.297688	-6.057269	4.975787	5.057828	5.050089	5.352564	4.698159	4.996333	5.221722	5.235456	5.166377	5.331011	5.113756	5.162375	5.260602	5.252767
Prrt2	-0.305741	8.370306	-1.522047	0.149463	0.297811	-6.079031	8.553459	8.228458	8.738558	9.251749	8.546587	8.86984	8.012886	8.169801	8.28751	8.081076	8.163315	7.955077	8.294021	8.036793
Trmt5	-0.318875	3.686336	-1.521779	0.14953	0.297894	-5.909119	3.771212	3.099845	3.719973	4.132904	3.965054	3.74229	3.797364	3.683523	3.848317	3.649853	3.706635	3.587916	3.646466	3.380568
Hyou1	-0.269285	7.694578	-1.521243	0.149663	0.298111	-6.114275	7.470249	6.924312	7.220397	7.747419	7.600525	7.612932	7.879417	7.845674	7.914459	7.816283	7.770754	7.926144	7.879309	8.124011
Zfp870	-0.323944	3.249686	-1.52079	0.149776	0.298269	-5.760139	3.136736	3.184227	3.013923	3.197095	2.892584	2.323579	3.537848	3.431732	3.518449	3.430116	3.684447	3.361247	3.522595	3.551406
Fxyd1	-0.52747	6.412248	-1.520725	0.149792	0.298269	-6.119102	7.613136	6.737266	5.841308	5.906012	6.492228	5.858722	5.902387	6.880052	6.055107	6.893761	6.721242	6.923933	5.844936	6.121113
Hnrnpa2b1	-0.34589	7.580252	-1.520444	0.149862	0.298359	-6.104139	7.809211	7.544016	7.534112	8.484602	7.743236	8.376275	7.550016	7.357426	7.517479	7.307063	7.141363	7.252397	7.19976	7.315014
Cmas	-0.424909	6.595715	-1.520045	0.149962	0.298507	-6.121216	6.619855	5.548027	6.383346	7.031064	6.795366	6.873412	6.592479	6.594167	6.606334	6.53135	6.747565	6.544534	6.964442	6.524823
Pcdhb20	0.401889	2.428658	1.519916	0.149994	0.298514	-5.499634	1.48718	1.914859	2.412888	2.045748	2.062471	2.271274	2.8014	2.80888	2.653634	2.707689	2.761158	2.761217	2.466543	3.151754
Pvrl2	0.318934	3.390261	1.519833	0.150014	0.298514	-5.78415	3.342675	3.544937	3.259286	2.816229	3.326873	3.420773	3.51327	3.449372	3.262563	3.493953	3.461211	3.916225	3.178239	3.629792
Agl	-0.283265	6.272874	-1.519605	0.150072	0.298578	-6.1206	6.475918	6.182029	6.004816	6.806821	6.41928	6.829435	6.305706	6.084783	6.042012	6.002166	6.094855	6.091323	6.319658	6.181999
Phf23	0.183657	5.705128	1.519234	0.150164	0.298712	-6.103898	5.795534	5.818732	5.878506	5.689036	6.078969	5.923702	5.521948	5.583794	5.560912	5.832041	5.619735	5.636495	5.402381	5.56021
Trmt112	0.255787	5.202514	1.519002	0.150222	0.298778	-6.104254	5.828799	5.687553	5.82492	5.629617	6.382561	6.037684	4.460269	4.70728	4.71363	4.741628	4.958312	4.718575	4.569728	4.620503
Btbd16	-1.169049	2.062092	-1.518244	0.150411	0.299105	-5.590483	2.529164	-1.463779	2.954902	3.002635	1.912714	2.940957	2.206902	2.054242	2.625819	2.529327	2.049375	1.826994	2.097585	2.245294
Lgalsl	-0.288071	6.413168	-1.518154	0.150444	0.29912	-6.124742	6.614302	6.413769	6.728232	7.133004	6.33019	6.870668	6.373276	6.101615	6.149703	6.114728	6.350494	6.099149	6.505932	6.018012
Igbp1	0.253873	5.126736	1.517358	0.150633	0.299447	-6.068229	5.235546	5.571717	5.010785	4.90852	5.497557	5.223224	5.055772	4.924651	5.011081	5.162339	5.142525	5.009416	4.887447	5.178649
Pdzk1	-0.321865	3.758862	-1.5164	0.150873	0.299874	-5.85857	3.481621	3.74155	3.158259	3.761892	3.529124	2.854719	4.161298	3.832115	4.06577	3.968034	3.921068	4.078836	3.969738	4.221283
Msx2	-0.84912	0.551976	-1.515137	0.15119	0.300454	-5.298393	2.246933	-0.273517	0.25957	0.43652	0.517419	1.353343	0.563361	0.58326	-0.773548	0.280604	1.375754	1.008331	0.313987	1.009844

Pcp4	-0.340955	6.726554	-1.514225	0.151419	0.30086	-6.128868	7.159252	7.038655	6.750637	7.481852	7.254266	6.872864	7.208522	6.254932	6.573948	6.156694	6.576644	5.98914	6.529836	6.339961
Nek7	-0.25898	5.180338	-1.51378	0.151531	0.300984	-6.091942	5.249943	5.068969	5.22264	5.751069	5.101527	5.58375	5.290835	4.886311	5.271094	4.919311	4.653101	5.004662	5.378131	5.187315
Trim66	-0.490982	1.906118	-1.513678	0.151556	0.300984	-5.67894	2.515609	2.780133	2.962413	3.075697	1.912714	1.648138	1.544223	1.596873	1.489496	1.023513	1.230182	1.783252	1.837926	1.73247
Mdc1	0.292108	3.660731	1.513653	0.151563	0.300984	-5.919991	3.718942	4.204491	3.978882	3.795925	4.043216	3.984893	3.241276	3.972932	3.432752	3.019813	3.480655	3.625558	3.542874	3.334499
Lrrc24	-0.312713	5.260572	-1.513507	0.151599	0.300984	-6.029072	4.933089	4.435504	4.405374	4.566597	4.713338	4.185462	5.999966	5.579795	6.116358	5.909827	5.338195	5.578496	5.683818	6.248723
Thnsl1	-0.422849	3.684379	-1.513479	0.151606	0.300984	-5.935928	3.670834	3.108509	3.4152	4.491448	3.770765	4.377073	3.639767	3.386664	3.716062	3.360772	3.603988	3.575147	3.850062	3.739923
Pex16	0.26015	4.857606	1.513073	0.151709	0.301137	-6.032135	4.751303	4.690471	5.060625	4.440107	5.07631	4.568038	4.897007	4.836907	5.132184	5.049759	4.79447	4.859513	4.852977	5.051519
Igfbp2	1.636723	5.592565	1.512023	0.151973	0.301612	-6.095532	7.877367	6.80812	6.812205	2.568755	7.010328	6.594206	3.987806	4.647404	3.889839	6.295187	8.164818	4.975806	3.725479	4.998341
Lrba	-0.237117	5.358852	-1.511785	0.152033	0.301681	-6.101319	5.419656	5.538441	5.505713	5.78172	5.018417	5.404132	5.411014	5.165139	5.317478	5.092395	5.213971	5.263337	5.563249	5.367782
Cdc37l1	-0.294708	5.859589	-1.510666	0.152316	0.302078	-6.127513	5.989418	5.843595	5.941619	6.603599	5.826691	6.400071	5.942828	5.605591	5.746716	5.474446	5.748297	5.480898	5.91792	5.540053
Plekha8	-0.200768	4.829262	-1.510608	0.15233	0.302078	-6.042121	4.643261	4.646529	4.62478	4.947973	4.412197	4.71234	5.159727	4.886311	5.037713	4.859579	4.80736	4.848943	4.962418	5.116484
Rfx1	0.423837	5.715561	1.510596	0.152333	0.302078	-6.092594	5.319852	6.421612	5.500563	5.17368	5.500476	5.268955	6.188995	5.709895	6.032774	6.043656	5.504148	5.680379	5.497232	6.206718
Alas2	0.578414	1.266959	1.510595	0.152334	0.302078	-5.497153	2.279379	1.894965	2.322392	1.065902	2.781752	1.785004	0.952703	0.818632	1.360416	0.938085	1.230182	0.460309	0.249639	0.321874
Sik1	0.708127	2.542809	1.510108	0.152457	0.302229	-5.66369	2.091157	4.077865	3.575619	2.591079	2.240661	2.374054	2.165439	2.345542	2.204856	2.819403	2.083688	2.794648	2.508394	2.011549
Vps37a	0.244903	3.88651	1.510043	0.152473	0.302229	-5.917687	3.608352	3.919485	4.254466	3.847833	3.751288	3.964448	3.882992	3.923301	3.963917	3.784431	3.897149	3.772618	4.094051	3.853172
Nrip1	-0.469411	5.40162	-1.509995	0.152485	0.302229	-6.095119	5.313991	4.117033	5.250517	5.786564	5.207167	5.732621	5.713926	5.803739	5.385408	5.297103	5.473744	5.599141	5.616844	5.363965
Taf9	0.261276	5.827372	1.509894	0.152511	0.30223	-6.114582	5.654231	5.552796	6.045165	5.68515	6.179853	5.940547	5.809551	5.872367	6.010091	5.526589	5.985681	5.678888	5.937201	5.732928
Gpx8	0.407854	4.133596	1.509617	0.152581	0.302314	-5.961027	4.47136	4.509329	3.830814	3.443962	4.332519	4.731825	3.756062	4.187837	3.675936	4.272852	4.421201	4.288919	3.887547	4.152141
U2af1l4	-0.20057	5.070316	-1.509528	0.152603	0.302314	-6.081458	5.313991	5.064523	5.005341	5.17368	4.958104	5.143628	5.052933	5.140918	5.310942	4.963216	5.034842	4.816761	4.977397	5.075058
Cd302	0.332197	4.312658	1.509171	0.152694	0.302442	-6.000129	4.579546	4.452618	4.549481	3.928941	4.836291	4.458273	3.593621	4.303891	3.96946	4.373903	4.324082	4.89334	3.994491	4.20003
Vwa5b1	-0.6791	2.059965	-1.509073	0.152719	0.302442	-5.41151	2.607924	0.854759	1.116684	0.823477	1.173177	0.846031	2.967416	2.888628	2.39812	2.291729	3.243265	2.827322	2.760099	2.503027
RGD1304624	-0.472772	1.675667	-1.508681	0.152818	0.302589	-5.474721	1.936985	1.702379	1.004744	1.799605	1.390206	1.585258	2.450751	1.934092	1.60797	2.06872	1.456531	1.738143	1.316372	1.971974
Fkbp9	-0.438993	6.433737	-1.508276	0.15292	0.302742	-6.1292	6.683368	5.186149	5.624096	5.883579	5.940711	6.614685	6.665863	6.922509	6.713457	6.790919	6.674869	6.856278	6.667807	6.867809
Hpgd	0.494193	2.468163	1.508056	0.152976	0.302803	-5.737843	2.658139	3.046741	2.812554	2.797152	3.326873	3.708268	3.148563	1.983359	1.843557	1.828755	2.259075	1.618784	2.301782	1.529341
Lyrm1	-0.281711	4.12454	-1.507857	0.153027	0.302853	-6.040977	4.680181	4.03306	4.462108	4.874798	4.822358	4.659903	3.783728	3.599118	3.985965	3.622732	4.008504	3.674268	3.860871	3.769079
Crcp	0.212365	5.320546	1.507743	0.153056	0.30286	-6.113839	5.627699	5.98792	5.998437	5.570457	5.719264	5.538825	4.861747	5.013306	4.953501	5.112031	5.242726	5.021232	4.989761	4.89103
Abhd10	-0.331778	3.305941	-1.507534	0.153109	0.302915	-5.903035	3.42474	3.479652	3.495051	4.177845	3.224646	3.74229	3.116285	3.292072	3.31576	2.745897	3.338699	2.990087	3.169571	2.932182
Nars	0.244569	7.985207	1.507015	0.153241	0.303126	-6.115142	8.011979	8.164723	8.582599	8.448187	8.527571	8.622857	7.643107	7.782274	7.756285	7.551361	7.756534	7.549859	7.831682	7.569878
Ndufaf5	-0.231487	4.719057	-1.5069	0.15327	0.303134	-6.081862	5.050539	4.958296	4.71425	5.368806	5.13573	5.123484	4.455977	4.318363	4.659784	4.51702	4.558676	4.285003	4.58936	4.392186
Slc25a16	-0.312132	4.960075	-1.506614	0.153342	0.303227	-6.097215	5.35643	4.640568	5.596606	5.544953	5.072391	5.228377	4.884284	4.688835	4.700356	4.556968	4.955998	4.549364	5.006894	4.710427
Atp1b3	0.217348	6.532407	1.506265	0.153431	0.303353	-6.136198	6.349825	6.403245	6.340035	6.218951	6.588666	6.673176	6.568821	6.511563	6.500222	6.858594	6.573629	6.858256	6.382897	6.642934
Rnf19a	-0.287588	5.842894	-1.505019	0.153748	0.30393	-6.131776	5.893112	5.419891	5.915737	6.378259	5.830177	6.226368	5.988123	5.625125	5.795909	5.597955	5.848982	5.613262	5.935923	5.759322
Ift57	-0.351062	5.52773	-1.504508	0.153878	0.304137	-6.120662	5.869329	5.244304	5.752034	6.420165	5.856622	6.321068	5.116866	5.31939	5.38748	5.16704	5.483415	5.19195	5.292891	5.000801
Apln	-0.334173	4.98675	-1.504002	0.154007	0.304342	-6.09974	5.732483	5.238395	4.862114	5.149441	5.475469	4.847983	4.848708	4.485848	4.553063	4.803319	4.92558	5.206513	4.952346	4.783689
Phlda3	0.331803	3.872518	1.503781	0.154064	0.304388	-5.970941	4.201945	4.466163	4.156367	3.771698	4.767666	3.866483	3.221182	3.871902	3.280514	3.734632	3.995046	3.926315	3.48116	3.584586
Eml5	-0.561085	6.786389	-1.503713	0.154081	0.304388	-6.140971	5.867997	6.354913	6.189759	7.396097	5.327135	6.423477	7.687264	7.302676	7.639429	7.019955	6.926594	6.716462	6.917322	7.25651
Pcdhga7	-0.454737	4.764657	-1.503485	0.154139	0.304416	-6.053755	4.810783	3.661386	5.018011	5.019674	4.418385	4.702498	4.547582	5.124541	4.989416	4.341	5.103341	5.110222	5.011753	4.906858
Lrrc73	-0.236741	5.557021	-1.503457	0.154146	0.304416	-6.105426	5.672669	5.366716	5.170091	5.289121	5.367673	5.06899	5.88493	5.619293	5.72885	5.951295	5.71311	5.644157	5.528077	5.827432
Pdgfa	-0.231409	5.155702	-1.503318	0.154182	0.304437	-6.07054	5.247895	4.88707	4.680492	4.768627	4.856941	4.675081	5.426454	5.230363	5.395739	5.553767	5.186605	5.425065	5.472777	5.418348
Slc26a6	0.454154	4.92008	1.503124	0.154231	0.304484	-6.01346	4.519693	5.23642	4.622412	4.005731	4.677668	4.317063	5.49291	5.11075	5.299983	5.675886	4.932657	5.166638	4.563124	5.315379
Rin2	0.241974	6.140162	1.502833	0.154306	0.304569	-6.136011	6.150471	6.241332	6.269126	5.902669	6.085789	6.473526	6.033481	6.32827	5.677274	6.419574	6.310307	6.723712	5.607246	5.762225
Klf7	-0.40592	4.53111	-1.502757	0.154325	0.304569	-6.084189	4.808006	4.551241	5.174948	5.651025	4.487704	5.091789	4.123906	4.464319	4.116621	3.709073	4.434557	4.17506	4.662235	4.056051
Snrnp35	-0.252649	5.028089	-1.502505	0.154389	0.304646	-6.114151	5.457367	5.179997	5.093491	5.754782	5.425653	5.724133	4.633716	4.83019	4.68359	4.728967	5.060912	4.789383	4.569728	4.510857
Brsk1	-0.271317	9.230888	-1.501232	0.154715	0.305213	-6.088852	9.753838	9.4167	9.195443	9.102155	9.017627	9.004727	9.342771	8.997359	9.169383	9.319342	9.006977	9.22022	9.316904	9.371626
Mlh3	-0.303241	4.180935	-1.501183	0.154727	0.305213	-5.985682	3.912376	3.982112	3.763618	4.591724	3.886882	4.220367	4.477313	4.451246	4.231815	4.111508	4.483567	4.12775	4.282726	4.098018
Tspan14	0.318664	4.917638	1.500765	0.154835	0.305375	-6.023931	4.563167	4.866787	4.758065	4.14804	4.779733	4.271191	4.838851	5.333708	5.104739	5.220028	5.010524	5.624151	5.14817	5.234069
Plvap	-0.467368	1.864412	-1.500122	0.154999	0.30563	-5.611172	2.8647	2.082541	2.33402	1.962578	1.664612	1.627481	1.047044	2.38274	0.97585	2.39151	2.995472	2.082939	0.836265	1.32462
Sh3bp1	0.30339	5.391946	1.50006	0.155015	0.30563	-6.097076	5.28032	5.717595	5.517234	4.969434	5.302254	5.168865	5.552424	5.605591	5.43233	5.706885	5.348815	5.680379	4.858381	5.384835
Col6a4	0.998936	-0.181026	1.499929	0.155049	0.305643	-4.982758	-0.634519	1.632013	-0.575682	-0.941548	-0.18071	-0.214525	-0.169545	0.222004	0.007959	0.348395	-0.731011	-0.24119	0.111637	0.706485
Mms19l	0.311135	5.400781	1.499836	0.155072	0.305643	-6.086948	5.132748	5.769049	5.219509	4.833262	5.271819	4.917698	5.772058	5.43679	5.649915	5.714933	5.49939	5.511379	5.266712	5.653849
Ttc9	0.212533	5.557912	1.499607	0.155131	0.305661	-6.129438	5.747057	5.809463	6.114225	5.644369	5.770958	5.932149	5.094948	5.410013	5.293368	5.500753	5.651537	5.257355	5.396821	5.221467
Fosb	-0.403142	2.865485	-1.499514	0.155155	0.305661	-5.814529	2.987615	2.506257	3.118472	3.649386	2.586747	3.47381	2.774074	2.454377	2.759824	2.572488	3.031075	2.715403	3.070609	2.635584
Nosip	0.318566	5.176761	1.499503	0.155158	0.305661	-6.060084	5.048187	5.259942	5.026994	4.298064	4.923716	4.724549	5.497094	5.312177	5.462129	5.726126	5.427736	5.235205	5.161347	5.416507
Plekhb2	-0.247469	8.397303	-1.499345	0.155199	0.305691	-6.116501	8.577306	8.040914	8.632059	8.828156	8.498477	8.653193	8.312611	8.143289	8.388733	8.194497	8.417545	8.176238	8.398799	8.305117
Tmem206	-0.28391	4.548206	-1.499033	0.155279	0.305755	-6.038752	4.576285	4.073446	4.337679	4.711187	4.5954	4.431711	4.416758	4.370218	4.666626	4.830243	4.348982	4.761475	4.674541	4.948235
Hcfc1	-0.376131	5.460466	-1.499019	0.155282	0.305755	-6.131054	5.803923	5.283087	6.074054	5.998686	5.080218	5.654372	5.116866	5.482485	5.336908	4.992645	5.329285	5.416142	5.816065	5.098219
RGD1309188	-0.214823	6.353748	-1.498738	0.155355	0.305848	-6.147637	6.429689	6.078899	6.09556	6.52389	6.355404	6.519096	6.346858	6.279794	6.364798	6.320727	6.554642	6.29875	6.511088	6.292556
Ar	-1.144684	1.632207	-1.498594	0.155391	0.30587	-5.504482	1.717752	-0.813242	1.874203	2.748327	1.173177	2.18908	1.607516	2.634802	1.843557	0.134646	2.531849	1.252437	3.195422	1.445134
Pitpnb	0.184256	6.420006	1.49848	0.155421	0.305878	-6.14882	6.342166	6.437172	6.616631	6.496064	6.739621	6.619923	6.329365	6.243603	6.273632	6.349962	6.530745	6.335179	6.355451	6.228923
Rmdn1	-0.238773	4.424616	-1.498356	0.155453	0.305891	-6.042416	4.671039	4.796145	4.169406	4.627263	4.302723	4.401612	4.614587	4.229271	4.401362	4.228565	4.316888	4.296718	4.364758	4.597931
Dlat	-0.441147	4.454425	-1.498078	0.155524	0.305981	-6.064519	4.716181	3.843675	4.787963	5.166265	4.573386	4.779416	4.282606	4.446862	4.409542	3.715505	4.741723	4.136468	4.828405	4.007943
Xcl1	-2.609767	-3.398202	-1.497488	0.155676	0.306229	-4.681638	-0.129637	-3.260591	-2.787702	-2.344749	-3.260591	-2.070862	-3.260591	-3.260591	-3.260591	0.413143	1.903329	-2.668207	-3.260591	-3.260591
Dusp16	-0.263513	3.073796	-1.497175	0.155756	0.306338	-5.863014	3.502386	3.019437	3.555895	3.492206	3.286847	3.045873	2.828218	3.059168	2.95129	2.655119	3.048553	2.951084	2.902922	2.921834
Mapk9	-0.314244	7.553209	-1.496966	0.15581	0.306393	-6.139257	7.791393	7.380614	8.006694	8.266155	7.570971	7.900477	7.314863	7.420831	7.32387	6.999674	7.526817	7.268349	7.721088	7.261648
Zfp830	-0.301224	3.741698	-1.496822	0.155847	0.306416	-5.974872	3.963192	3.432128	3.715535	4.318277	4.070978	4.149692	3.137884	3.676051	3.662308	3.7783	3.633241	3.761389	3.658907	3.544677
Atp6ap2	-0.374396	7.185858	-1.49646	0.15594	0.306549	-6.152413	6.536738	5.502738	6.388931	6.777313	6.773808	6.462634	7.7574	7.573919	8.090767	7.639411	7.511144	7.612908	7.841948	8.145015
RGD1305587	-0.26043	3.907972	-1.496148	0.156021	0.306657	-6.009437	4.643261	4.252185	4.229782	4.090441	4.031153	3.888353	3.895725	3.690957	3.716062	3.790537	3.690026	3.35379	3.811576	3.73402
Mpc2	-0.215934	5.045133	-1.49593	0.156077	0.306718	-6.0954	5.225173	4.874426	4.852041	5.143788	5.163626	4.984199	5.108686	4.696241	5.330461	4.979343	5.152662	4.843629	5.204421	5.121014
Ing2	-0.226848	4.65996	-1.495648	0.15615	0.30681	-6.076513	4.917711	4.397125	4.716472	4.988479	4.808289	4.976052	4.443022	4.639742	4.600281	4.725784	4.768337	4.523011	4.424271	4.373323
Bud31	0.231256	5.612069	1.495416	0.15621	0.306878	-6.138139	5.770073	5.716178	5.890367	5.758485	6.144827	6.227226	5.708524	5.378133	5.426295	5.297103	5.375027	5.140873	5.326424	5.442077
Mus81	0.423569	4.934434	1.495018	0.156313	0.30703	-6.02437	4.377065	5.34305	4.59126	4.155549	4.680246	4.156917	5.623044	5.077105	5.408039	5.394523	4.96523	5.065272	4.844833	5.454694
Paqr3	-0.26651	4.458185	-1.494402	0.156472	0.30728	-6.017837	4.428604	3.735969	4.28174	4.399576	4.436792	4.113012	4.682297	4.351577	4.723506	4.31582	4.564762	4.581643	4.937104	4.935431
Tdrd7	-0.160397	5.7473	-1.494286	0.156502	0.30728	-6.136658	5.870661	5.671563	5.645251	5.717218	5.673431	5.542968	5.827938	5.807164	5.826779	5.805152	5.699345	5.772746	5.772362	5.858963
Edem2	0.291077	4.670273	1.494228	0.156517	0.30728	-6.004473	4.526467	4.54806	4.317322	3.756964	4.600852	4.220367	4.595201	5.183701	4.69032	5.208673	4.976688	5.300661	4.716806	4.806368
Smyd4	-0.262557	3.240855	-1.494051	0.156562	0.307286	-5.815251	3.271877	3.08236	2.812554	3.197095	3.174343	2.845805	3.270902	3.14827	3.634661	3.377094	3.324422	3.228918	3.689548	3.482664
LOC100361087	-0.462155	3.062859	-1.494018	0.156571	0.307286	-5.701719	2.41698	2.545069	2.345554	3.107017	2.399242	2.025031	3.981847	2.939445	3.991425	3.622732	3.50618	2.960934	3.294432	3.953102
Prpf19	-0.191132	8.22465	-1.493789	0.15663	0.307352	-6.128961	8.459906	8.109686	8.388803	8.604497	8.388318	8.478937	7.993025	8.128993	8.113792	8.060695	8.148955	7.998164	8.198449	8.078152
Tnfrsf25	-0.646557	5.037577	-1.493522	0.156699	0.307434	-5.980797	3.870393	2.874583	3.111733	4.698391	4.525459	4.08677	6.384613	5.551489	6.152143	6.053845	5.172725	5.757285	5.666979	6.688666
Slc35f1	-0.319873	6.293583	-1.49343	0.156723	0.307434	-6.153426	6.094586	5.758078	5.946361	6.745945	6.318657	6.382457	6.300927	5.967255	6.389821	6.287653	6.169639	6.35399	6.65	6.765185
Pcdhb21	-0.61165	1.227087	-1.49308	0.156814	0.307561	-5.368125	0.739674	0.118359	1.061799	2.029493	0.731352	1.327961	0.952703	1.775313	0.97585	1.73055	1.456531	1.826994	1.837926	1.32462
Rad54b	0.766307	0.160856	1.492935	0.156851	0.307585	-5.096075	-0.310971	0.812964	-0.195392	-0.166914	0.091273	1.302125	1.135593	-0.380636	0.449965	0.280604	-0.149817	0.34469	0.037347	0.442608
Irf2bpl	0.229786	5.950344	1.492486	0.156967	0.307763	-6.145127	5.866663	5.80147	6.337871	5.827097	5.9726	6.188969	5.701288	6.044728	5.893911	5.89715	5.889551	6.090202	6.053667	5.765122
Tceb1	-0.263493	4.834291	-1.492286	0.157019	0.307814	-6.127641	5.52658	4.938996	5.282373	5.676041	5.443967	5.682182	4.61073	4.12885	4.263751	4.386051	4.348982	4.204378	4.683703	4.561731
Rfc1	-0.267979	6.24708	-1.491929	0.157112	0.307926	-6.157226	6.159216	6.083301	6.189759	6.861793	6.150414	6.550982	6.152323	6.319903	6.208317	6.06648	6.232158	6.180436	6.283959	6.040905
Gars	0.24945	7.108956	1.491868	0.157127	0.307926	-6.15767	7.16469	7.139323	7.649115	7.354292	7.714607	7.535009	6.767219	6.982805	6.937177	6.683464	7.03633	6.74382	7.043073	6.786017
Scaf8	-0.208043	6.61393	-1.491505	0.157222	0.30806	-6.16189	6.843838	6.571701	6.984405	6.992276	6.535571	6.748646	6.494381	6.655296	6.44558	6.360261	6.523733	6.497937	6.564139	6.393368
Hdlbp	-0.206002	8.30031	-1.491361	0.157259	0.308083	-6.13789	8.18973	7.994052	8.169676	8.507155	8.135034	8.269082	8.426682	8.492888	8.347816	8.187284	8.473812	8.324988	8.454055	8.237101
Nedd4	-0.217158	9.579611	-1.490619	0.157452	0.308391	-6.091234	9.576395	9.272849	9.50967	9.903517	9.561842	9.745175	9.513461	9.605766	9.498646	9.512716	9.647024	9.609985	9.674096	9.485467
Samm50	0.180112	5.874717	1.490559	0.157467	0.308391	-6.140735	5.755731	6.011209	5.820794	5.595517	5.898172	5.690919	6.005851	5.813989	6.001999	6.010037	5.825096	5.855598	5.970022	6.018012
Ocrl	-0.201209	5.849007	-1.490262	0.157544	0.308491	-6.141213	5.72514	5.51093	5.688917	5.844623	5.640724	5.49531	6.105133	6.070605	5.846496	5.809668	6.114696	5.841005	6.157051	6.063439
Vamp7	-0.474097	4.116054	-1.489096	0.157848	0.309035	-6.03792	4.319721	3.317238	4.402616	4.870241	4.086605	4.654809	3.81758	4.24955	3.716062	3.608978	4.327666	4.032882	4.539769	3.774841
Necab3	-0.520615	5.054257	-1.488739	0.157941	0.309167	-6.09496	5.339219	5.800134	4.503885	4.99058	4.348811	3.972661	5.347548	5.149037	5.277813	5.478234	5.27278	5.112433	4.800602	5.416507
Riok3	0.197064	6.55723	1.4886	0.157977	0.309187	-6.162301	6.271342	6.501077	6.816874	6.544055	6.57834	6.523295	6.711592	6.593175	6.589332	6.614583	6.624045	6.435726	6.454227	6.560282
Dgka	0.362005	7.173239	1.488309	0.158053	0.309285	-6.162368	6.28838	7.131906	6.323361	6.422501	6.78043	6.635525	8.052162	7.548057	7.84189	7.769137	7.139325	7.309405	7.248422	7.946835
Cmtm5	-0.441033	6.309922	-1.487552	0.15825	0.309621	-6.159744	6.600725	6.486195	5.271833	6.144491	6.432334	5.547098	6.168154	6.520978	6.399094	6.715105	6.297573	6.875283	6.316717	6.584267
Rpp14	-0.232273	4.763552	-1.487276	0.158322	0.309711	-6.081689	4.796842	4.375753	4.629506	4.97581	4.738286	4.872329	4.874668	4.576933	4.809475	4.716194	4.976688	4.662336	4.982355	4.760648
Msl3	0.246987	5.79042	1.487031	0.158386	0.309779	-6.151817	5.648032	6.343949	5.977275	5.989262	5.905901	6.043544	6.014634	5.543299	5.625543	5.642519	5.612409	5.463681	5.471014	5.813505
Slc26a1	-0.314777	2.892861	-1.486946	0.158408	0.309779	-5.729396	2.706665	2.864389	2.135702	2.881078	2.460496	2.549011	3.231264	3.115498	2.973531	3.00938	3.004456	3.277021	3.152075	3.357718
Kl	-3.688063	0.742393	-1.48583	0.1587	0.310299	-5.455937	7.358899	0.185409	-0.263851	-0.093872	-0.33871	-0.1418	-0.831021	-0.511025	-0.632503	3.978769	5.998103	-0.920914	-0.305233	-1.212031
Zc3h7a	0.2812	6.109869	1.485592	0.158762	0.31037	-6.1533	5.563278	6.231451	6.070583	6.032724	5.986203	6.037684	6.434553	6.218103	6.300273	6.470852	5.999227	6.145232	5.786605	6.284501
Mamdc4	0.453558	1.966376	1.485461	0.158796	0.310386	-5.429821	1.310059	1.702379	1.386086	1.21709	1.947363	1.803538	2.659281	1.958936	2.087398	2.375352	2.049375	2.461578	2.236876	2.756979
Ap1b1	0.186997	7.614017	1.484835	0.15896	0.310656	-6.166091	7.388018	7.688614	7.717292	7.348161	7.437949	7.376534	7.854358	7.629138	7.733659	7.669126	7.723252	7.597994	7.637511	7.802711
Dnajb6	-0.175168	5.313525	-1.483759	0.159242	0.311157	-6.139683	5.417836	5.364909	5.401818	5.732361	5.43941	5.402613	5.103206	5.321786	5.282274	5.249138	5.25407	5.214769	5.1192	5.12553
LOC691995	0.372313	5.393085	1.483329	0.159355	0.311302	-6.143036	5.616631	5.590392	5.56118	5.344374	5.728009	6.519797	5.003787	4.69993	5.586392	4.874027	5.010524	4.755828	5.775222	5.47607
Sema4f	0.234302	6.234929	1.483279	0.159368	0.311302	-6.145828	5.576394	6.132979	5.734594	5.544953	5.656516	5.659469	6.858672	6.369389	6.801564	6.624871	6.392161	6.508889	6.534063	6.916833
Tnrc18	-0.169143	7.523736	-1.4829	0.159468	0.311444	-6.169421	7.383361	7.322224	7.3195	7.534276	7.245591	7.270611	7.72744	7.714591	7.672094	7.713184	7.451628	7.671817	7.614142	7.700477
Prkcsh	0.242618	8.307395	1.482302	0.159625	0.311666	-6.156457	7.991317	8.292113	8.092198	7.922949	8.381208	8.031549	8.442769	8.412263	8.55028	8.612353	8.236386	8.432653	8.160399	8.750141
Gpc5	-0.331594	5.667663	-1.482272	0.159633	0.311666	-6.152054	6.262239	5.752562	5.554984	5.405483	5.346735	5.34684	5.751189	5.387314	5.660235	5.756075	5.460748	5.946399	5.686859	6.061084
Ogfod1	-0.399665	4.789015	-1.481654	0.159795	0.311932	-6.10929	5.092229	4.379337	5.394896	5.268492	4.436792	4.889781	4.477313	4.886311	4.549366	4.31582	4.946707	4.701051	5.204421	4.561731
Degs1	-0.315735	6.893353	-1.481196	0.159916	0.312117	-6.175989	7.27992	6.382857	6.965373	6.87493	7.011864	6.687592	6.324664	7.003849	6.848951	7.020604	6.897955	7.210178	6.970069	7.041554
Hs6st1	-0.194965	6.213213	-1.481024	0.15996	0.312142	-6.168888	6.372561	5.842297	6.303539	6.201827	6.130763	6.088636	6.190288	6.267417	6.183488	6.211195	6.222567	6.339905	6.331364	6.320398
LOC500034	-0.461549	2.748011	-1.480951	0.15998	0.312142	-5.78964	2.742017	1.894965	2.761803	3.456176	2.828971	3.084277	2.467906	2.91426	2.810125	2.325762	2.892776	2.594065	3.278395	2.660694
Xbp1	0.247444	7.145413	1.480088	0.160207	0.312534	-6.177062	6.813002	6.718288	7.095741	6.889639	7.450836	7.13083	6.977984	7.238176	7.181855	7.33918	7.268925	7.349324	7.222464	7.370763
Alpl	0.405924	4.009493	1.479468	0.160371	0.312664	-6.010091	4.519693	4.528825	4.39431	3.504019	3.943761	4.726978	4.046082	3.458111	3.980484	4.015725	3.812726	3.447848	3.646466	4.208569
Evi5	-0.19215	6.222391	-1.479445	0.160377	0.312664	-6.174076	6.431495	6.338435	6.367875	6.49828	6.091609	6.294288	6.246047	5.94877	6.03806	6.090187	6.086419	6.271306	6.311801	6.119982
Lat	0.637502	1.471545	1.479443	0.160378	0.312664	-5.311666	0.873216	2.028784	0.715742	0.485009	1.463853	1.046069	2.305626	1.532126	1.914249	2.184529	1.533025	1.404538	1.54805	2.17732
RGD1565784	-0.243977	4.543906	-1.479406	0.160387	0.312664	-6.105144	4.851815	4.49288	4.817254	5.213795	4.93239	4.917698	4.161298	4.402272	4.429792	4.111508	4.518478	4.237171	4.409621	4.187126
Gpn1	0.18739	4.797663	1.479193	0.160443	0.312664	-6.077098	4.559869	4.560742	4.864121	4.635342	4.890715	4.81639	4.919005	4.90241	4.919536	4.747917	4.85532	4.814046	4.885102	4.85341
Tdg	0.181263	5.786447	1.479179	0.160447	0.312664	-6.159376	5.665015	5.734484	6.084417	5.830619	6.015073	5.881799	5.811232	5.722645	5.741865	5.71814	5.778723	5.674405	5.659259	5.721041
Rbms2	0.328736	4.56201	1.479145	0.160456	0.312664	-6.08111	4.89695	5.432022	4.682767	4.301452	5.032606	4.554424	4.371894	4.274505	4.250151	4.588849	4.376922	4.426789	4.270617	4.475916
Tvp23a	-0.33306	4.051289	-1.478796	0.160548	0.312751	-5.938739	3.549707	3.551306	3.89502	4.018141	3.500244	2.889836	4.301868	4.485848	4.485023	4.449167	4.363019	4.365074	4.420622	4.544972
Pdia4	0.357237	6.100916	1.478773	0.160554	0.312751	-6.165224	5.928067	5.903318	5.694566	5.801	6.660254	6.625794	5.991093	6.182948	5.890993	6.151959	5.947762	6.388155	5.983686	6.286519
Ifnlr1	0.501283	1.165039	1.478681	0.160579	0.312751	-5.363412	1.459129	1.558038	1.809043	0.43652	1.05105	1.165512	1.000645	1.279465	0.837186	1.540753	1.167576	1.667719	1.077784	0.970221
Kpna1	-0.538394	4.945894	-1.478191	0.160708	0.312866	-6.128091	4.988109	3.929299	5.259691	5.849261	4.8478	5.472326	4.614587	5.059983	4.852113	4.502215	5.107516	4.761475	5.456834	4.594677
Exoc3l4	0.466041	2.324766	1.478188	0.160709	0.312866	-5.511165	2.144973	1.934482	1.858187	1.000693	1.981199	2.203108	2.703406	3.311493	2.539002	2.988286	3.004456	2.738492	2.203293	2.278116
Dpy30	0.318385	4.203901	1.478162	0.160716	0.312866	-6.022419	4.026686	4.08227	4.156367	4.177845	4.901799	4.543439	4.272878	4.139756	4.259232	3.984107	4.21995	4.014082	4.314529	3.847716

Meaf6	-0.229729	5.974734	-1.477328	0.160936	0.313244	-6.177704	6.393092	6.093156	6.080108	6.666707	6.546206	6.483618	5.759921	5.758465	5.71901	5.647573	5.740252	5.460213	5.710957	5.612623
Lemd3	-0.238636	4.764527	-1.477058	0.161008	0.313333	-6.054956	4.304037	4.051149	4.554459	4.713732	4.154906	4.323498	5.067074	5.088407	5.218522	4.973987	4.88724	5.065272	5.163531	5.198074
Hmgn3	-0.242929	6.773639	-1.476731	0.161094	0.31345	-6.180185	7.173888	7.138795	6.851164	7.47699	6.945925	7.391926	6.749791	6.417234	6.61697	6.550366	6.323827	6.26038	6.359268	6.58918
Zfp467	-0.236577	5.076054	-1.476549	0.161143	0.313494	-6.136226	5.276307	5.246268	5.178177	5.499945	5.089943	5.103056	4.835551	5.036833	5.102217	5.213226	5.082283	5.081164	4.405935	4.960925
Ccnb1	0.754504	0.591922	1.475716	0.161363	0.313872	-5.21788	0.105685	0.24948	0.307539	0.33436	2.014259	1.221719	0.429655	0.972957	0.317129	0.591587	0.584127	0.930264	1.040235	0.257494
Eif2d	0.298367	4.461559	1.475603	0.161393	0.31388	-6.026944	4.243653	4.88455	4.166157	3.984807	4.355276	4.254448	4.729295	4.552657	4.639062	4.670575	4.558676	4.509652	4.254311	4.731318
Pigl	0.355682	3.33552	1.475113	0.161523	0.314081	-5.754044	2.853968	3.159427	3.077557	2.41457	2.77212	2.898483	3.895725	3.683523	3.456633	3.747244	3.568082	3.52291	3.621257	4.19144
Slc18a2	-0.344892	3.180243	-1.47478	0.161612	0.314203	-5.791771	3.073383	2.397344	2.744481	2.985885	2.936364	2.644629	3.280644	3.232189	3.324439	3.7783	3.359854	3.549265	3.459987	3.937777
Kcnk2	-0.371224	4.243	-1.474409	0.16171	0.314341	-6.059104	4.492273	3.649562	4.204668	4.635342	4.051203	4.845749	4.663805	4.213873	3.947156	4.246444	4.056804	4.153748	4.274664	4.051312
Fchsd2	0.211136	5.804744	1.474315	0.161735	0.314341	-6.166847	5.774348	6.257977	5.831087	5.751069	5.893738	5.915205	5.897647	5.742456	5.523715	5.664295	5.678448	5.871352	5.709462	5.783814
Uap1l1	0.336603	4.442679	1.474132	0.161784	0.314385	-6.031587	4.559869	4.4112	4.188746	3.696477	4.757939	4.446528	4.102096	4.68139	4.294995	4.922095	4.661644	4.997502	4.047497	4.503936
Pou3f1	-0.417965	6.008022	-1.474005	0.161818	0.3144	-6.165262	5.626123	4.92435	5.005341	6.205449	5.950352	6.117625	6.136317	6.464627	6.291447	6.475597	6.453308	6.415274	5.967523	6.105195
Cntfr	-0.206687	6.664449	-1.473833	0.161863	0.314437	-6.184004	6.688663	6.430277	6.29464	6.587728	6.644503	6.357587	6.503768	6.753528	6.718404	6.973077	6.747565	6.909382	6.807064	6.901774
Zfp319	-0.394559	3.492561	-1.473092	0.16206	0.314746	-5.936682	3.701089	3.037697	3.684081	3.815017	3.238702	3.468012	3.026505	3.784265	3.271566	3.129854	3.621611	3.926315	4.070962	3.262515
Cd82	0.379185	6.058323	1.47304	0.162074	0.314746	-6.159947	5.959694	5.797457	6.004816	5.113264	6.344072	5.489598	5.846094	6.454836	6.027469	6.398741	6.207086	6.707714	6.034699	6.455669
Nudt2	-0.258356	4.280749	-1.472259	0.16228	0.315098	-6.043639	4.611755	4.240408	3.945162	4.140492	4.387176	3.892687	4.292269	4.294162	4.170562	4.433645	4.53717	4.338875	4.456698	4.271049
Rnf168	-0.252075	4.878473	-1.471976	0.162358	0.315194	-6.130293	5.055231	4.804167	4.770955	5.392832	5.068462	5.257237	4.732848	4.870031	4.755947	4.602796	4.877493	4.581643	4.994677	4.588148
Grm2	-0.45612	6.271853	-1.471877	0.162384	0.315194	-6.178977	6.273357	6.675198	5.662646	6.282916	5.855482	5.112377	6.51516	6.367071	6.718404	6.661145	6.182559	6.485196	6.144941	6.891204
RGD1307947	-0.36764	4.541649	-1.471522	0.162479	0.315327	-6.129012	4.912549	4.482919	4.897808	5.569051	4.675086	5.446024	4.481542	4.284367	4.070936	3.826639	4.235294	3.99982	4.637302	4.134325
Ackr2	-0.524295	1.249592	-1.470849	0.162658	0.315583	-5.34528	1.212615	-0.186163	0.785152	1.065902	0.649545	1.221719	1.754571	1.775313	1.959523	1.451089	1.922414	1.345607	1.700256	1.556352
Rab33b	-0.347241	4.792012	-1.47083	0.162663	0.315583	-6.113765	4.757075	4.129856	4.718692	5.312821	4.928059	4.976052	4.674929	4.80985	4.771898	4.653926	5.0566	4.870006	5.06414	4.421864
Ppp1r12a	-0.32063	6.666484	-1.470687	0.162701	0.315606	-6.190065	6.476793	6.294502	6.629073	7.32795	6.40844	6.995313	6.694385	6.685381	6.775692	6.500022	6.662862	6.521424	6.859209	6.515391
LOC361635	0.171523	5.90008	1.469659	0.162976	0.31605	-6.171341	5.613453	5.817411	5.80417	5.837638	5.951419	6.018971	6.021912	5.945666	5.928478	5.932091	5.876155	5.984319	6.003332	5.892471
Ipp	-0.409199	3.37219	-1.469635	0.162983	0.31605	-5.979008	3.701089	2.963229	3.633224	4.249769	3.378569	4.287741	3.299932	2.988532	3.140097	2.733273	3.273307	3.145002	3.522595	3.050898
Hibadh	-0.20319	6.076254	-1.469429	0.163038	0.316105	-6.187946	6.530854	6.205438	6.110004	6.436439	6.433862	6.373181	5.958085	5.676582	5.672184	5.851165	5.947762	5.940188	5.973761	6.015581
Shroom3	-0.395151	3.620613	-1.469301	0.163072	0.316121	-5.963263	3.776905	2.769246	3.929918	3.984807	3.617838	3.57437	3.577905	3.777297	3.947156	3.167876	3.723055	3.419555	4.056929	3.496677
RGD1306151	-0.174167	7.667974	-1.468612	0.163256	0.316427	-6.189014	7.496363	7.230945	7.257504	7.411467	7.418786	7.21285	7.84413	7.785749	7.99922	7.88352	7.76746	7.880168	8.013125	8.1583
Clvs1	-0.382844	5.465009	-1.467971	0.163428	0.316709	-6.171414	5.485452	4.769077	5.730201	6.219847	5.552043	5.821694	5.342907	5.43679	5.436339	5.059905	5.546281	5.177238	5.610452	5.35822
Herc4	0.236922	4.84607	1.467848	0.163461	0.316722	-6.105379	4.495729	5.060063	4.790075	4.926188	4.962345	5.004367	5.191057	4.789219	4.908035	4.722594	4.717344	4.721475	4.640443	4.970998
Lrp1	0.22191	10.05585	1.46775	0.163487	0.316722	-6.122415	9.531746	10.03119	9.916059	9.6551	9.556591	9.753614	10.42423	10.25731	10.36512	10.24904	10.05606	10.43177	10.18287	10.37264
Kcnk16	1.850321	0.915669	1.46741	0.163578	0.316847	-4.958622	0.830061	-0.273517	-0.067513	-2.731185	1.463853	0.117189	2.717819	2.207232	2.04602	-0.420466	1.135223	2.375822	2.652228	2.70964
Ttr	-5.064076	4.702692	-1.467208	0.163632	0.316901	-5.958291	12.89039	2.134368	1.951721	1.740915	3.028244	2.00915	3.529702	3.509467	2.905753	10.45516	12.78437	2.315682	3.431265	3.365375
Cep41	-0.364468	2.9863	-1.466939	0.163705	0.316969	-5.864371	3.614724	2.493084	3.084457	3.067759	2.810268	3.432728	2.659281	3.000548	2.995434	2.758411	3.295436	2.761217	3.03287	3.002614
Urgcp	-0.193963	6.094668	-1.46688	0.16372	0.316969	-6.17483	5.862653	5.763574	5.726897	5.780507	5.692706	5.329359	6.427994	6.252422	6.408308	6.434273	6.44178	6.398158	6.389444	6.44125
Entpd4	-0.178119	6.757774	-1.466404	0.163848	0.317166	-6.194662	6.639517	6.589603	6.681212	6.722754	6.372238	6.370076	6.860297	6.799815	6.944937	6.895179	6.825669	6.870065	6.989285	7.062902
Fank1	0.592847	0.77807	1.466164	0.163913	0.317223	-5.250649	0.539902	1.117652	0.945338	0.166125	1.312595	0.284862	1.297885	1.067371	0.884903	0.20947	0.924208	1.11804	0.698429	1.260365
Cdc40	-0.497176	6.428373	-1.466038	0.163947	0.317223	-6.191063	6.406925	4.934131	5.980978	6.611129	6.394382	6.731204	6.848883	6.711201	6.61697	6.720712	6.629862	6.445406	6.697509	6.287527
RGD1563666	-0.679866	1.236333	-1.465998	0.163957	0.317223	-5.438889	0.739674	1.425638	1.245402	2.350586	0.091273	0.982423	2.05625	1.464337	1.843557	1.14286	0.924208	1.045835	1.700256	1.009844
Fkbp5	0.268249	6.615468	1.465559	0.164075	0.317401	-6.1967	6.554246	6.925538	7.379291	7.008953	6.783429	7.113759	6.551825	6.354253	6.330744	6.249376	6.504073	6.099149	6.552517	6.225772
Syncrip	0.384307	5.017754	1.465371	0.164126	0.317447	-6.16415	4.997891	5.311505	5.8992	5.831792	5.595018	6.404631	4.531353	4.643578	4.457672	4.49849	4.512193	4.588013	4.766516	4.262873
Arhgef15	0.434206	3.31023	1.464534	0.164351	0.317832	-5.874119	3.334978	4.042133	3.436929	2.899083	3.665442	3.053636	3.783728	3.159031	3.655446	3.249928	2.696109	3.16218	2.871195	3.496677
Dffb	0.46337	3.452251	1.464164	0.164451	0.317974	-5.802121	2.730329	4.060109	3.209656	2.77782	2.865664	2.791131	3.987806	3.838823	3.947156	3.7783	3.598065	3.581545	3.416687	3.901371
Erlin1	0.208904	5.362253	1.463826	0.164542	0.318098	-6.145811	5.057571	5.086616	5.361199	5.220971	5.482869	5.459235	5.239313	5.510064	5.462129	5.46111	5.300397	5.460213	5.449691	5.558541
Shc4	0.37834	2.280843	1.463687	0.164579	0.318099	-5.760776	2.63325	2.724859	3.575619	2.546081	2.742832	2.743931	2.186319	1.68885	2.107651	1.73055	1.347792	2.100868	2.151407	1.991897
Sesn2	0.467935	4.462397	1.463581	0.164608	0.318099	-6.094302	4.45014	4.438943	5.861534	4.53528	4.735811	4.767664	3.734959	4.60868	4.029076	3.896239	4.460902	4.603816	4.368551	4.056051
Tcf19	0.507944	2.116733	1.463499	0.16463	0.318099	-5.79381	2.555898	3.780021	2.401882	2.835056	3.601614	3.143674	1.047044	1.155984	1.579251	2.027955	1.629088	1.950783	0.791767	1.556352
Stip1	-0.304624	7.921289	-1.463431	0.164648	0.318099	-6.179178	8.057124	7.429836	7.900731	8.469995	8.39559	8.10903	7.827611	7.792244	7.93373	7.652042	7.912616	7.629986	7.940462	7.853618
Prelid1	0.20671	6.862581	1.462927	0.164784	0.318311	-6.198797	7.328697	7.220492	7.319865	6.987024	7.622132	7.297013	6.449744	6.531368	6.551022	6.534082	6.738171	6.448035	6.561657	6.500691
LOC100302465	-0.5456	2.618774	-1.461927	0.165054	0.318781	-5.692453	2.460078	3.125682	1.613009	2.634716	2.046579	1.426914	2.501617	3.19084	2.810125	3.267546	2.696109	3.575147	2.760099	2.83626
Zdhhc22	-0.523425	3.1986	-1.461407	0.165195	0.319001	-5.983848	3.536344	3.324693	4.438066	4.430854	2.692656	3.390443	2.518182	2.695192	3.207329	2.600561	2.995472	3.055902	2.923692	3.151754
Foxg1	-0.466686	7.561152	-1.461072	0.165286	0.31908	-6.186832	7.718944	6.823331	7.620322	8.554962	7.827319	8.408605	7.324902	7.457251	7.489778	7.134193	7.537301	7.228132	7.422234	7.317483
Snapc1	0.226831	3.441012	1.46106	0.165289	0.31908	-5.91479	3.287913	3.563961	3.679531	3.419222	3.397487	3.678453	3.159163	3.517851	3.333065	3.393233	3.467722	3.482452	3.542874	3.395603
Rsad2	1.393471	2.438515	1.459964	0.165585	0.319601	-5.559067	1.741237	1.934482	4.498728	1.21709	2.07819	2.634313	2.227194	2.939445	1.255413	2.966878	2.86345	5.325652	1.407665	1.415936
Brd1	-0.208216	6.158429	-1.459559	0.165695	0.319739	-6.197343	6.087732	5.940185	6.113381	6.421723	5.848624	6.286074	6.259656	6.344858	6.214166	6.111073	6.144462	6.187794	6.158147	6.122243
Calm1	-0.255729	10.35455	-1.459504	0.16571	0.319739	-6.098282	10.67339	10.08129	10.57104	10.69746	10.43966	10.63374	10.27429	10.204	10.27118	9.949353	10.31785	10.19417	10.55383	10.10364
Tshr	0.666909	1.21665	1.45932	0.16576	0.319784	-5.325069	0.105685	1.425638	1.593793	1.128291	0.985877	1.107027	1.17791	2.24812	1.01926	1.104165	0.961591	2.082939	1.574559	1.227132
RGD1305089	-0.220894	3.728034	-1.459219	0.165787	0.319786	-5.990139	3.748215	3.588941	3.789185	4.026356	3.686105	3.616909	3.585784	3.929599	3.70281	3.622732	3.627438	3.789298	3.701625	3.896095
Tmtc2	-0.342716	3.286572	-1.458875	0.165881	0.319915	-5.917669	3.799454	3.099845	3.240874	3.281878	3.044338	3.359461	2.991343	3.292072	3.271566	2.923085	3.022257	3.703897	3.683472	3.461387
Chrna2	-0.389609	3.272761	-1.457767	0.166181	0.320443	-5.824406	2.843156	1.934482	3.12518	3.342347	2.847434	2.881136	3.624549	3.690957	3.774234	3.636356	3.480655	3.637891	3.45286	3.722142
Ppp1r11	-0.310759	6.568361	-1.457236	0.166326	0.320524	-6.208627	7.026966	6.125528	6.655957	6.971154	7.010328	6.960638	6.246047	6.429507	6.442594	6.246048	6.679784	6.364232	6.610513	6.204585
Dock9	-0.248681	8.788495	-1.457227	0.166328	0.320524	-6.170858	8.544309	8.864182	8.470417	9.055506	8.327965	8.530263	9.243065	8.911073	9.02395	8.729606	8.668828	8.659968	9.018578	8.994845
Rraga	-0.358048	6.690315	-1.457221	0.16633	0.320524	-6.208103	6.631684	5.855224	6.598669	7.137751	6.798335	6.852982	6.735695	6.644808	6.962418	6.498158	6.941288	6.445406	6.94489	6.632659
Wdfy2	0.228959	6.430182	1.457202	0.166335	0.320524	-6.198494	5.910048	6.485364	6.420631	6.158573	6.046376	6.103671	6.853786	6.607977	6.711804	6.546763	6.496929	6.615626	6.407989	6.675637
Usp7	-0.253452	7.635788	-1.457103	0.166362	0.320524	-6.199557	7.40879	7.332429	7.532536	8.077446	7.30441	7.785142	7.918159	7.625748	7.775005	7.547761	7.525647	7.5877	7.705055	7.783193
Capn1	0.340137	4.513303	1.456841	0.166433	0.320524	-6.073878	4.110227	5.082224	4.223544	4.418424	4.695613	4.46411	4.822272	4.36558	4.673435	4.680472	4.258009	4.394445	4.233667	4.834225
Tp73	-1.432984	1.8787	-1.456836	0.166435	0.320524	-5.374103	3.562947	-1.685466	0.642822	-0.243853	0.731352	0.881356	3.200804	3.211662	2.330799	2.600561	3.83304	2.656009	2.522078	2.972849
Haus7	0.358252	5.255523	1.456829	0.166437	0.320524	-6.141016	5.033995	5.728876	4.988886	4.792905	5.270109	5.104925	5.765135	5.143629	5.555394	5.566277	4.920843	5.032953	5.014175	5.701512
Mobp	-0.733856	9.180857	-1.456383	0.166558	0.320667	-6.167528	9.308229	9.791475	7.883031	8.85766	8.533997	7.172869	9.84651	9.438367	9.885684	9.710031	8.789537	9.658363	9.457739	10.20187
Pigw	0.237886	3.61368	1.45636	0.166564	0.320667	-5.966455	3.576066	3.707738	3.767911	3.606198	3.775594	4.056182	3.562015	3.501034	3.860303	3.393233	3.537463	3.269114	3.409342	3.698087
Cdipt	-0.287469	7.359114	-1.456018	0.166657	0.320795	-6.208804	7.331601	6.424217	7.076402	7.249861	7.319781	7.194974	7.324902	7.500727	7.68563	7.608208	7.428452	7.581728	7.579385	7.69327
Tmem57	-0.201334	6.440953	-1.455127	0.1669	0.321211	-6.206716	6.227323	6.132979	6.297612	6.660123	6.171634	6.370076	6.346858	6.544764	6.560236	6.592911	6.492945	6.559198	6.573205	6.661712
Gtse1	0.747326	1.98366	1.454987	0.166938	0.321233	-5.54211	2.832262	1.972946	1.792283	0.22441	2.919011	2.00915	1.836107	2.207232	2.024877	2.202958	2.360517	2.046399	1.79348	1.991897
Ebi3	0.364027	2.60368	1.454684	0.167021	0.321236	-5.80578	2.620642	2.557778	3.575619	2.87199	2.987204	3.255598	2.324585	2.077116	2.446625	2.543858	2.544222	2.315682	2.348615	2.245294
Pxmp2	-0.3765	3.363333	-1.454678	0.167023	0.321236	-5.956513	4.021899	3.986819	2.753168	3.41297	3.446756	3.22842	3.374605	3.201289	3.367065	3.424979	3.352837	3.315916	3.143247	3.212449
Dnajc30	0.207374	3.988383	1.454675	0.167024	0.321236	-6.073595	4.29216	4.256089	4.669061	4.263733	4.576156	4.419747	3.639767	3.526187	3.848317	3.734632	3.706635	3.594258	3.794763	3.61702
Tppp3	-0.426369	4.284751	-1.454533	0.167062	0.321236	-6.062325	4.851815	3.86939	3.604709	3.950685	4.641095	3.756629	4.223227	4.342165	4.488889	4.268484	4.192699	4.916302	4.460257	4.503936
Man2c1	0.445715	6.970262	1.454491	0.167074	0.321236	-6.206617	6.589447	7.41376	6.641408	5.850418	6.538414	6.063866	7.577938	7.152598	7.548368	7.455666	6.986194	7.173056	6.964442	7.641787
Nlrx1	0.31253	4.252763	1.453603	0.167316	0.321629	-5.969589	3.793849	4.335728	3.74196	3.342347	3.818339	3.616909	4.606864	5.007364	4.508062	4.650573	4.573843	4.647164	4.40224	4.581589
Sept11	0.228699	7.063896	1.453489	0.167347	0.321629	-6.207611	7.324816	7.646847	7.624175	7.499293	7.354888	7.935823	6.668648	6.908229	6.672407	6.669451	6.752905	6.681891	6.770268	6.397109
Ndufv1	-0.176362	7.384905	-1.453388	0.167375	0.321629	-6.21093	7.418383	7.180992	7.206627	7.32878	7.307748	7.094602	7.591699	7.324946	7.713152	7.390833	7.54886	7.266861	7.462268	7.562402
Tmem199	-0.212291	5.119451	-1.453351	0.167385	0.321629	-6.152855	5.225173	4.704825	5.08147	5.128607	5.078266	4.982166	5.141132	5.302504	5.163961	5.024078	5.276493	5.025932	5.402381	5.18082
Ppp2r3b	0.276329	4.58819	1.452852	0.167521	0.321789	-6.102217	4.539921	5.051103	4.903672	4.412169	4.409093	4.641992	4.606864	4.485848	4.809475	4.653926	4.431229	4.523011	4.208498	4.623699
Nr2f1	-0.215195	6.420348	-1.452851	0.167522	0.321789	-6.211862	6.587829	6.2364	6.27517	6.604971	6.677771	6.336264	6.538692	6.35776	6.363746	6.416616	6.237883	6.397251	6.227653	6.645295
Hddc2	0.336727	3.259446	1.452667	0.167572	0.321799	-5.972108	3.460552	3.595119	3.767911	3.675737	4.165982	4.090548	3.038038	3.115498	3.006262	2.877922	3.108108	2.880186	2.974345	2.050068
Cntn3	-0.320416	3.793604	-1.452635	0.167581	0.321799	-6.004777	3.799454	4.073446	3.945162	4.109897	3.099296	3.462191	4.0114	3.727562	3.91304	3.559779	3.678847	3.738666	4.009141	4.098018
RGD1310371	2.129343	-0.158832	1.452511	0.167615	0.321813	-4.849963	0.692264	-1.685466	-0.130036	-3.260591	1.113405	-0.291112	0.498056	0.871929	-1.10526	0.20947	0.631286	1.045835	1.60059	1.29285
Slc39a3	-0.239586	4.41151	-1.451565	0.167874	0.322259	-6.019115	4.026686	3.874479	3.81015	3.989017	3.943761	3.456346	4.674929	4.677653	5.000289	4.850841	4.757751	4.885603	4.885102	5.008157
Tardbp	0.20353	6.953014	1.451462	0.167902	0.322261	-6.215497	6.511351	6.901436	6.707709	6.850271	6.973504	6.955468	7.336624	6.969112	7.243212	7.065357	6.847676	6.898533	6.984968	7.109811
Hsd17b1	0.529343	0.659376	1.451084	0.168006	0.322374	-5.233949	0.243629	0.632508	0.398943	0.166125	0.606834	1.193889	0.68572	1.239467	0.512061	1.322149	0.805871	1.284167	0.182287	0.970221
Apex2	0.347182	2.479566	1.451052	0.168015	0.322374	-5.68689	2.501925	2.619695	2.345554	1.874319	2.762423	2.349037	2.450751	2.287882	2.446625	2.720538	2.455292	2.726993	2.676906	2.780079
Stk19	0.317488	2.866865	1.450955	0.168041	0.322374	-5.776559	2.607924	3.1924	2.893363	2.57996	2.874693	2.695134	3.211029	3.000548	3.04878	3.090803	2.750518	3.028062	2.522078	2.858134
Sde2	0.234369	5.024547	1.450666	0.16812	0.322474	-6.166445	4.927981	5.204449	5.499273	5.4149	5.249432	5.677165	4.591292	4.816662	4.771898	4.818339	4.955998	4.961159	4.746254	4.757741
Gnl3	0.282246	5.455271	1.450111	0.168273	0.322716	-6.16972	5.09451	5.687553	5.653395	5.29763	5.298904	5.280579	5.919635	5.312177	5.658521	5.716538	5.200353	5.316091	5.284886	5.689362
Pla2g4b	0.502314	4.766559	1.449754	0.168371	0.322852	-6.069699	4.137033	5.464442	4.391531	4.106027	4.646377	4.028875	5.302835	4.853564	5.202134	5.220028	4.597784	4.752996	4.55649	5.533271
Pld3	-0.250646	9.090814	-1.449502	0.16844	0.322902	-6.178006	9.066605	8.489503	8.904565	8.725946	8.800137	8.398162	9.274789	9.157181	9.617615	9.325794	9.174167	9.214442	9.385886	9.7396
Jdp2	-0.223971	4.604591	-1.449464	0.168451	0.322902	-6.133604	4.805223	4.522355	4.725329	4.973688	4.484758	4.930402	4.494157	4.552657	4.222559	4.821324	4.703619	4.536248	4.502288	4.25465
Metap1	-0.208562	5.89867	-1.448552	0.168701	0.323331	-6.217236	5.908752	5.549619	5.865545	6.200013	5.928839	6.03964	5.94895	5.880517	5.984309	5.824622	5.975438	5.812728	5.91792	5.770899
Rbks	0.259899	3.064716	1.448338	0.16876	0.323368	-5.903283	3.287913	3.354134	3.184186	3.114742	3.654998	3.637716	2.717819	2.80888	2.707706	2.911926	3.173291	2.869767	2.771602	2.900911
Snx14	-0.29729	6.464996	-1.448285	0.168775	0.323368	-6.216207	6.224202	6.228473	5.906032	6.76877	6.197951	6.47063	7.069333	6.395786	6.76776	6.630838	6.234069	6.334232	6.404299	6.895838
Parva	0.365831	3.030503	1.448043	0.168841	0.323444	-5.924865	3.556342	3.368632	3.674966	3.010938	3.590696	3.879645	2.814871	2.38274	2.772565	2.375352	2.940364	2.581351	3.40196	2.278116
Insrr	1.733733	-0.763978	1.447827	0.168901	0.323507	-4.875889	1.108109	-0.572649	-0.575682	-3.260591	-1.235601	0.734546	-0.831021	-0.048075	-0.504027	-0.420466	-0.318799	-0.24119	-0.893866	0.554014
Acn9	-0.387897	2.246275	-1.447573	0.168971	0.323585	-5.751886	2.957854	2.01041	2.298852	2.427034	2.79132	2.071653	2.033384	2.186344	2.260183	2.165862	2.288794	1.738143	2.66462	1.889408
Fam174a	-0.318332	5.080506	-1.447483	0.168996	0.323585	-6.163293	5.017259	4.37216	4.889952	5.476153	5.058591	5.393466	4.991976	5.05423	5.257563	4.992645	5.246517	5.229104	5.193772	5.000801
Clcc1	0.241208	5.948782	1.447328	0.169038	0.323615	-6.199455	5.651135	6.207456	5.93113	5.6695	5.735463	5.735037	6.170776	5.835949	6.106329	6.135264	5.760279	6.164541	5.924376	6.281469
Sugt1	0.267059	5.218695	1.44712	0.169096	0.323626	-6.16792	4.95334	4.991462	5.35265	5.280562	5.690151	5.502418	5.159727	4.965068	5.286722	5.124169	5.419364	5.05151	5.237977	5.088999
Clcnkb	0.7819	1.812504	1.447064	0.169111	0.323626	-5.480559	1.541696	3.000943	1.7408	0.823477	1.745593	1.327961	2.380012	1.564863	2.04602	2.499819	2.133674	1.11804	1.377871	2.55752
Atp6v0e1	0.336214	4.37318	1.446943	0.169145	0.323626	-6.087767	4.733849	4.421666	4.405374	3.819751	4.879544	4.68261	4.051782	4.161323	4.091419	4.494755	4.812484	4.468815	4.023644	4.25465
Disp2	-0.210205	9.932497	-1.446784	0.169188	0.323626	-6.155135	9.370382	9.348282	9.363357	9.872527	9.557906	9.36712	10.39685	10.12001	10.4956	10.1391	10.04753	10.14594	10.28064	10.55141
Zcchc17	-0.166204	5.729849	-1.446701	0.169211	0.323626	-6.210381	6.066973	5.831871	5.886424	6.043894	5.928839	5.909869	5.524	5.526777	5.475827	5.495156	5.688238	5.630336	5.740523	5.498875
Tmem129	0.292956	4.957004	1.446672	0.169219	0.323626	-6.137482	4.933089	5.033013	4.74506	4.538155	5.4917	4.832273	4.764433	4.80985	4.830952	5.067468	5.142525	5.170887	4.982355	5.10738
Gmfb	-0.217666	6.386036	-1.446625	0.169232	0.323626	-6.219886	6.423351	6.327345	6.380546	6.717056	6.102218	6.602168	6.410354	6.248649	6.302471	6.252697	6.190453	6.441014	6.617701	6.407344
Lrrc8d	-0.341973	6.547874	-1.446186	0.169353	0.323806	-6.217826	6.373501	5.320855	6.217488	6.504174	6.326082	6.518395	6.77068	6.942889	6.707663	6.690011	6.768809	6.908746	6.828561	6.810064
Zfp46	-0.379937	6.150039	-1.445661	0.169498	0.324031	-6.221648	6.415162	5.688998	6.450315	6.921919	6.118463	6.895718	5.768601	6.29938	5.886604	5.834998	6.303954	5.948875	5.98739	5.602939
Aff1	0.356059	3.872191	1.445457	0.169555	0.324087	-6.014192	3.748215	3.801553	4.472666	3.756964	3.741449	4.377073	3.863678	4.193082	3.774234	3.368956	3.537463	3.931333	4.018826	3.73402
Pnpt1	0.185056	5.442734	1.444639	0.169781	0.324369	-6.197057	5.416013	5.633051	5.784177	5.682553	5.740411	5.783691	5.57239	5.162468	5.259827	5.183373	5.090743	5.121244	5.286892	5.517894
Adck5	0.426315	4.954661	1.444622	0.169786	0.324369	-6.10223	4.611755	5.461064	4.74506	4.07864	4.592667	4.277834	5.542337	5.102412	5.332613	5.434063	4.845355	5.085673	4.858381	5.4511
RGD1562747	-0.324001	3.407345	-1.444482	0.169824	0.324369	-6.005322	3.633671	3.512664	3.863274	4.159289	3.052318	3.884005	3.116285	3.377479	3.059216	3.440594	3.31723	3.18757	3.349197	2.900911
Srsf12	-0.295456	3.034118	-1.444451	0.169833	0.324369	-5.861875	3.016775	2.963229	2.770386	3.275	2.672087	3.045873	3.251219	3.242343	3.262563	2.945148	3.265855	2.761217	3.3415	2.858134
Dnaja2	-0.337698	7.242126	-1.444437	0.169837	0.324369	-6.220459	7.187309	6.73377	7.265116	7.958263	7.288874	7.64925	7.235695	7.189804	7.27434	6.916971	7.309358	6.989159	7.353097	7.0493
Elovl5	0.288412	5.256644	1.443891	0.169988	0.324606	-6.169578	5.403188	5.445761	5.528663	4.751386	5.307264	5.154498	5.018416	5.300076	5.117279	5.206391	5.012752	5.526382	5.483308	5.379173
Tmem2	-0.224639	5.165991	-1.443441	0.170112	0.324792	-6.16009	4.970829	4.960691	5.125624	5.231667	4.829341	4.600198	5.511644	5.140918	5.499012	5.316274	5.084402	5.325652	5.290894	5.481365
Mcts1	-0.480085	5.05237	-1.443325	0.170145	0.324802	-6.198552	5.371558	4.335728	5.20058	6.163237	5.586799	6.021942	5.251748	4.49437	5.016447	4.414005	4.714609	4.426789	5.00202	4.783689
Ccdc132	-0.330414	5.294215	-1.443091	0.17021	0.324849	-6.181692	5.225173	4.535265	5.255111	5.625567	5.118729	5.465068	5.363679	5.343175	5.487466	4.955085	5.48502	5.214769	5.772362	5.313401
Adam10	-0.282198	6.238797	-1.443041	0.170223	0.324849	-6.224372	6.420627	6.030712	6.262296	6.717056	6.163367	6.691329	5.873711	6.209071	5.996579	6.002166	6.005952	6.505528	6.292967	6.192799
Micalcl	-0.412312	2.242493	-1.441885	0.170544	0.325385	-5.762811	2.529164	2.679063	2.518567	2.881078	1.96438	2.086865	2.550752	1.68885	2.260183	2.291729	1.652137	2.082939	1.747621	2.802815
Ndufs2	0.180734	7.762038	1.441834	0.170558	0.325385	-6.217948	7.793851	7.925225	7.866112	7.595252	8.026298	7.684363	7.872187	7.578435	7.837728	7.752421	7.624374	7.609781	7.684666	7.825096

Herpud1	-0.275444	6.124098	-1.441687	0.170599	0.325411	-6.218174	6.468902	5.494499	5.879498	5.804587	6.14576	5.915205	6.046387	6.190834	6.139903	6.382664	6.328306	6.39271	6.192784	6.37831
Coch	-1.777604	-0.083043	-1.441513	0.170648	0.325437	-5.254219	2.832262	-3.260591	0.442561	-0.411255	-0.831496	2.526873	0.68572	0.04768	0.245823	-0.027758	-0.149817	0.151783	-0.405292	0.383504
Mag	-0.476827	8.57526	-1.441439	0.170668	0.325437	-6.204931	8.653548	8.583001	7.50226	8.259855	8.48103	7.348409	8.556898	9.088657	8.723906	9.033958	8.484687	9.333784	8.804306	9.203866
Atox1	0.375701	4.983212	1.441345	0.170694	0.325437	-6.154619	5.341141	5.423367	4.870123	4.31157	5.620738	4.861313	4.906476	4.792678	4.950701	5.176396	5.060912	4.840964	4.797792	4.861554
Pfkfb4	0.416272	2.695963	1.440797	0.170846	0.325676	-5.771738	2.488111	3.486315	2.947351	2.475838	2.308867	2.872384	3.060831	2.58779	2.772565	2.90068	2.531849	2.680061	2.535634	2.341603
Enpp4	-0.322414	6.173155	-1.440695	0.170875	0.325678	-6.223838	6.232511	6.066725	6.135145	6.475212	6.045377	5.881799	5.918076	6.274856	5.987045	5.837949	5.940759	6.647996	6.611314	6.391495
Alas1	-0.195968	5.880378	-1.440108	0.171038	0.325938	-6.219278	6.117201	5.631549	5.991111	6.020439	6.001741	5.863146	5.903964	5.911074	5.912737	5.71814	6.018201	5.762926	5.736127	5.763674
Gpr88	0.603456	4.040955	1.439674	0.171159	0.326116	-6.128894	5.165666	5.646499	5.452035	3.810268	4.209453	4.982166	2.967416	3.104407	2.720913	3.51623	3.771221	3.936334	4.148003	3.25429
Chst9	-1.121501	3.620673	-1.439168	0.1713	0.326333	-5.990176	3.502386	-0.025882	3.95649	4.792905	3.859855	4.192511	3.221182	4.026799	4.263751	3.448339	3.66758	3.885525	4.28674	3.831222
Ikbip	0.321807	3.013125	1.438757	0.171414	0.326459	-5.849503	3.082606	3.200527	3.171281	2.645422	3.028244	3.288866	2.398025	3.159031	3.207329	2.866406	2.930971	3.179156	3.061266	3.160583
Celf1	-0.281914	5.687958	-1.438736	0.17142	0.326459	-6.201712	5.296263	5.101882	5.599017	5.967029	5.29218	5.413212	6.010249	6.008001	5.922774	5.64589	5.900421	5.711343	6.120411	5.674029
Dolpp1	0.230574	4.605291	1.438544	0.171474	0.326479	-6.107202	4.45014	4.646529	4.569292	4.284429	4.487704	4.677595	4.233294	4.662608	4.504248	4.882627	4.744407	4.870006	4.833902	4.692276
Isg20l2	0.629265	0.62506	1.438504	0.171485	0.326479	-5.344521	1.178614	1.310208	1.451597	0.041976	0.985877	0.949514	0.798508	1.067371	0.091681	-0.311823	0.535373	1.152831	-0.040977	0.606649
Nsd1	-0.31421	6.909412	-1.437907	0.171651	0.326744	-6.234459	6.547601	6.558324	6.870754	7.321289	6.294469	6.758179	7.08053	7.291106	7.080473	6.765571	7.086369	7.007096	7.275438	6.807958
Fastkd1	0.361377	5.275475	1.43757	0.171746	0.326872	-6.15331	4.566458	5.778581	4.985204	4.941472	4.8728	4.861313	5.837869	5.314586	5.685718	5.664295	5.210093	5.353961	5.231744	5.59482
Uba3	-0.279311	6.12388	-1.436885	0.171937	0.327184	-6.234688	6.56086	6.038667	6.411057	6.932885	6.649115	6.771773	5.747682	5.887004	5.840457	5.48766	5.847735	5.647211	6.113648	5.821878
Lonrf3	0.25365	3.986889	1.436648	0.172003	0.327258	-6.003681	3.562947	3.780021	4.096187	3.732068	3.639189	4.079183	4.494157	4.106787	4.15118	4.010503	3.999546	4.078836	4.130242	4.056051
Pcolce	0.845445	2.599705	1.435448	0.172339	0.327845	-5.771168	3.973144	3.117121	2.726949	0.785264	3.144791	3.49677	2.286414	2.326577	2.260183	2.558244	3.065821	2.581351	1.465469	2.91141
Khdrbs1	-0.280279	6.901746	-1.43525	0.172394	0.327899	-6.236043	7.11836	6.624	6.994514	7.576495	7.242543	7.406019	6.531571	6.726654	6.734773	6.614583	6.885853	6.735954	6.860556	6.585907
Cog6	-0.199588	4.558761	-1.435123	0.17243	0.327915	-6.11885	4.543265	4.585778	4.19832	4.566597	4.345567	4.313834	4.732848	4.572916	4.646003	4.51702	4.493172	4.755828	4.861076	4.757741
Cebpz	0.197437	6.169829	1.434844	0.172508	0.328011	-6.233237	6.058818	6.363082	6.621976	6.503439	6.384933	6.546175	6.039231	6.10022	5.95247	6.026944	5.884694	5.987936	6.035891	5.893796
Mrpl52	0.254663	4.91936	1.434379	0.172639	0.328203	-6.152668	4.988109	5.284999	4.854061	4.476537	4.979188	4.81639	4.90017	5.099622	4.97846	5.206391	4.830277	4.877826	4.740412	4.89103
Pelo	0.203078	4.036335	1.43429	0.172664	0.328203	-6.072817	4.045673	4.159341	4.397084	4.098255	4.345567	4.188991	4.102096	3.825375	3.958351	3.896239	3.999546	3.936334	3.959716	3.692011
Ptpn1	0.205288	5.561	1.433692	0.172831	0.32847	-6.202975	5.360227	5.414661	5.692309	5.419585	5.656516	5.61294	5.528095	5.760233	5.412116	5.583962	5.685448	5.656333	5.803513	5.301477
Usp8	-0.195417	6.877462	-1.433417	0.172909	0.328509	-6.238501	7.12116	6.987908	7.110182	7.529588	7.112617	7.307602	6.669575	6.721219	6.501179	6.599012	6.648607	6.625724	6.756585	6.607052
Ptpn5	0.344295	8.60566	1.433369	0.172922	0.328509	-6.214182	8.202211	8.90205	8.233342	8.259855	8.689677	8.467178	9.341754	8.419273	8.930938	8.872088	8.261428	8.460042	8.382247	9.061321
Tmc7	-0.262957	3.616509	-1.433327	0.172934	0.328509	-5.949822	3.633671	3.452688	3.301354	3.361953	3.114621	3.069038	3.654827	4.061624	3.985965	3.765957	3.723055	3.99982	3.964736	3.673625
Zbp1	1.111674	-0.034331	1.433128	0.17299	0.32853	-5.041911	-1.052261	-0.103798	-0.86368	-0.81875	0.952152	0.386712	-0.672244	2.207232	-1.536754	2.14695	1.719153	3.195935	-1.904936	-1.632384
Ipmk	-0.241695	5.421279	-1.433093	0.173	0.32853	-6.220463	5.533322	5.435469	5.588134	6.1492	5.471011	5.912006	5.370537	5.173124	5.166377	5.268953	5.246517	5.267312	5.165713	5.187315
Rae1	0.224444	5.555419	1.432834	0.173072	0.328585	-6.204598	5.576394	5.576408	5.923454	5.211996	5.612665	5.34526	5.578326	5.520532	5.622027	5.493286	5.696576	5.568867	5.471014	5.612623
Rab28	-0.212032	5.848117	-1.432794	0.173084	0.328585	-6.230261	6.075082	5.570149	5.859524	6.162305	6.09451	6.100864	5.908683	5.583794	5.778645	5.677534	5.847735	5.622601	5.822992	5.796614
Rbm41	0.480028	2.01646	1.432602	0.173138	0.328636	-5.646932	1.741237	2.953644	2.322392	1.996424	1.510943	2.654873	1.754571	1.883083	2.147322	1.852296	1.883989	2.00891	1.963595	1.951771
Inpp4a	-0.180919	7.768546	-1.432124	0.173272	0.328839	-6.233311	7.608753	7.753576	7.525264	7.809722	7.351658	7.479539	8.13135	7.759916	7.957288	7.821511	7.796843	7.73704	8.021881	8.012616
Ell	0.26481	5.253937	1.431443	0.173464	0.329151	-6.196971	5.076159	5.771779	5.774609	5.391242	5.172806	5.285532	5.180689	5.292766	5.199777	5.266765	5.109599	5.168764	4.831156	5.075058
Cotl1	0.392577	7.483773	1.431342	0.173492	0.329153	-6.237925	7.565418	7.047693	7.455122	7.044776	8.096238	8.197967	7.602905	7.655974	7.119643	7.518171	7.718142	7.642663	7.16747	6.949675
Htr6	-0.41272	2.182776	-1.430283	0.173791	0.329647	-5.690296	2.43149	2.046927	2.187895	2.213416	1.143601	2.257897	2.247205	2.522625	2.41447	2.308845	2.133674	1.950783	2.639729	2.417223
Zmat2	-0.324664	7.460992	-1.430224	0.173807	0.329647	-6.226713	7.989786	7.318961	7.729174	8.32457	7.951195	8.331478	7.042388	7.169991	7.161414	7.004935	7.365783	6.983734	7.267843	6.821938
Med8	0.28732	3.073536	1.429914	0.173895	0.32976	-5.839943	2.987615	3.019437	3.042552	2.557463	3.217567	2.907078	3.436917	3.024282	3.262563	3.090803	3.265855	3.046681	2.933966	3.425211
Yy1	-0.323933	5.489171	-1.429731	0.173946	0.329806	-6.226945	5.735409	5.057828	5.734594	6.11687	5.614014	5.996996	5.133088	5.589771	5.291156	5.024078	5.390529	5.294831	5.629542	5.275297
Fhod3	-0.387091	4.405602	-1.429266	0.174078	0.330003	-6.183528	5.519806	5.344885	5.108801	4.919588	4.180618	4.6901	3.908348	3.653399	3.924502	4.051757	4.21995	3.805789	4.43154	3.998126
Zfp692	0.464726	6.869125	1.428584	0.17427	0.330316	-6.238995	6.161393	7.199357	5.813544	6.010121	6.875704	6.315429	7.70365	7.006086	7.475238	7.411779	6.902768	6.981316	6.752963	7.573187
Mrpl17	0.224347	6.064484	1.428469	0.174303	0.330317	-6.230784	5.979578	5.851358	6.019292	5.658972	6.376217	5.91946	6.101015	6.067753	6.15701	6.112292	6.355768	6.025968	6.20344	6.098319
Sdpr	-0.267079	3.365027	-1.428389	0.174325	0.330317	-6.070194	4.380808	4.159341	3.754994	3.915736	3.574163	4.032808	3.454237	2.709903	3.120298	2.628098	2.873292	2.568523	2.994118	3.106782
Rab8a	0.181988	6.363818	1.427709	0.174518	0.330623	-6.247458	6.735458	6.739359	6.737564	6.678483	6.957657	7.120242	5.900809	6.185581	5.808336	6.131661	6.251153	6.121277	5.957486	5.788093
Mir568	-0.55429	1.669698	-1.427623	0.174542	0.330623	-5.39292	0.830061	0.812964	0.25957	1.353927	0.517419	0.772673	3.026505	2.287882	2.611707	2.342482	2.243983	2.330954	1.881043	2.70964
Lpp	0.668785	0.189455	1.426729	0.174795	0.330965	-5.272008	-0.129637	0.854759	1.493674	0.531921	0.091273	0.915838	-0.279766	0.58326	0.571595	0.134646	-0.231835	-0.153565	0.313987	-0.621773
Zfp111	-0.239749	4.149598	-1.42668	0.174809	0.330965	-6.052868	4.172016	3.919485	3.651925	3.852461	3.850732	3.668376	4.238302	4.274505	4.338488	4.588849	4.324082	4.741613	4.233667	4.330935
Rnf114	0.194718	5.787485	1.426636	0.174822	0.330965	-6.228872	5.904858	5.775864	6.047816	5.521898	6.04037	5.756599	5.60183	5.847636	5.544292	5.820152	5.899217	5.907454	5.683818	5.701512
Tspan15	-0.371943	4.291599	-1.426596	0.174833	0.330965	-6.015131	3.827152	3.397198	3.453014	3.843191	4.070978	2.949304	4.52317	4.799571	4.833994	4.800297	4.624251	5.198209	4.808999	5.044382
Chtf8	-0.272005	6.084712	-1.426485	0.174864	0.330973	-6.240884	6.387522	5.519076	5.849432	6.089689	6.27158	6.221207	6.078853	5.982481	5.931321	6.106186	6.218712	6.223009	6.292967	6.037314
Pbx4	0.357992	3.812556	1.425472	0.175152	0.33138	-5.99998	3.516065	4.335728	3.838998	3.515737	3.397487	3.94371	4.14005	3.770297	3.91304	4.07195	3.755344	3.70975	3.4385	4.138799
Rap1gds1	0.235447	8.24301	1.425469	0.175153	0.33138	-6.223405	8.09065	8.309594	8.494416	8.541413	8.676829	8.721639	8.052162	8.124197	8.044243	7.906543	8.249198	8.005023	8.26583	7.925628
Tnrc6b	-0.326654	6.759052	-1.425419	0.175167	0.33138	-6.249384	6.214796	6.317103	6.287183	7.200804	6.188025	6.737243	7.116331	7.189148	6.810086	6.800783	6.952937	6.757307	7.085742	6.984275
Srd5a1	0.339282	5.240426	1.425338	0.17519	0.33138	-6.204781	5.348806	5.842297	5.140599	5.072196	6.050366	5.168865	4.732848	5.230363	4.875924	5.114467	5.128213	5.463681	5.178731	5.060981
Chst11	-0.47773	4.72755	-1.425139	0.175246	0.331434	-6.162429	4.838267	3.806886	5.120598	5.062243	4.332519	4.592225	4.301868	4.81326	4.959085	4.150009	4.906537	4.862143	5.511022	4.990935
Rnf32	-0.338483	2.413768	-1.425039	0.175275	0.331436	-5.761008	2.460078	2.167912	2.445413	2.687469	1.765147	2.581593	2.566765	2.753156	2.185932	2.529327	2.639567	2.315682	2.575555	2.417223
Bace2	0.553536	2.296959	1.424564	0.17541	0.3316	-5.748899	2.387517	2.309899	3.177748	2.124384	3.083806	2.549011	1.836107	2.419003	1.60797	1.704918	1.347792	2.516041	2.437952	2.992761
Adprm	0.221254	3.651083	1.424463	0.175438	0.3316	-6.024317	3.621067	3.69053	3.952724	3.742078	4.002607	3.956189	3.647317	3.501034	3.584966	3.602051	3.586147	3.300483	3.695599	3.357718
Nfatc3	0.212992	4.119188	1.424442	0.175444	0.3316	-6.110362	4.231267	4.294561	4.556942	4.263733	4.418385	4.559885	3.870144	3.897831	3.780555	3.802672	3.999546	4.323687	3.850062	3.911867
Engase	0.328199	4.494531	1.423977	0.175577	0.331769	-6.075067	4.17633	4.749824	4.493553	3.747057	3.986037	3.909896	4.689628	5.077105	4.564098	4.827276	4.804791	4.96361	4.330171	4.676974
Lrrc61	-0.230922	4.290054	-1.423915	0.175594	0.331769	-6.127411	4.53321	4.037603	4.195136	4.49737	4.5954	4.304105	4.187425	4.17729	4.227195	4.173561	4.50588	4.253292	4.383621	4.070176
Fzr1	0.18557	5.330739	1.423836	0.175617	0.331769	-6.195981	5.231406	5.283087	5.389334	4.967302	5.380408	5.074724	5.486611	5.423464	5.50284	5.441843	5.287575	5.281137	5.483308	5.436635
Bloc1s2	0.203352	5.202948	1.423534	0.175702	0.331879	-6.201301	5.187202	5.042086	5.441325	5.136218	5.501934	5.462154	5.293243	5.105197	5.112276	5.131403	5.180673	5.225023	4.999576	5.065689
Pla2g5	-2.020798	0.643283	-1.422999	0.175855	0.332051	-5.495169	4.816322	0.310827	-0.665442	-0.243853	0.952152	0.175273	-0.399109	0.301855	-0.386063	2.14695	4.518478	-0.24119	-0.405292	-0.397871
Gtf2ird1	0.446306	3.769614	1.422952	0.175868	0.332051	-6.002892	3.771212	4.716207	4.149803	3.218762	3.409963	3.468012	3.999651	3.599118	3.878097	3.790537	3.733898	3.680242	3.529386	3.948012
Gtf2a2	0.378018	2.616391	1.422923	0.175876	0.332051	-5.821523	2.246933	2.557778	2.837276	2.977436	3.144791	3.450478	2.501617	2.287882	2.430637	2.291729	2.696109	2.703718	2.452318	2.310209
Ddx27	0.207088	6.156319	1.422788	0.175915	0.332072	-6.249385	6.05648	6.520683	6.466252	6.51297	6.379393	6.604151	6.050663	6.034539	6.071954	6.051304	5.780032	5.909999	5.783768	5.988566
Arid3a	0.373512	3.222897	1.422679	0.175946	0.332079	-5.953578	2.799078	3.479652	3.784955	3.696477	3.231692	4.03673	3.116285	3.059168	3.079866	3.040455	2.892776	3.083214	2.689088	3.302949
Wnt5b	-0.563335	1.622321	-1.422063	0.176121	0.332357	-5.435608	1.669604	0.679782	0.442561	0.860705	0.691028	1.014597	2.880403	1.775313	2.166756	2.454393	1.94125	2.284645	1.859646	2.584013
Irf3	0.414519	7.17889	1.421269	0.176348	0.332733	-6.255027	6.782907	7.642753	6.562059	6.407642	7.168755	6.659887	7.717164	7.380597	7.58404	7.746152	7.078414	7.282656	6.904319	7.598585
RGD1306502	-0.299143	5.754509	-1.421064	0.176406	0.332754	-6.247482	5.894422	5.428566	5.998437	6.546199	6.064245	6.194246	5.61344	5.491027	5.579158	5.414429	5.675639	5.449758	5.828509	5.414663
Ube2k	-0.247279	7.244562	-1.421035	0.176415	0.332754	-6.25016	7.39266	6.989082	7.379642	7.859135	7.596096	7.548464	7.120405	7.005341	7.094494	6.982442	7.236409	7.007096	7.21353	7.009539
Tomm22	0.20543	6.137035	1.42047	0.176576	0.333006	-6.250234	6.226284	6.179975	6.403489	6.090669	6.629912	6.23919	6.081642	5.928474	6.028797	6.056381	6.238835	5.940188	6.039464	5.857606
Thop1	0.225435	5.79587	1.420101	0.176682	0.333153	-6.230769	5.672669	5.494499	6.007541	5.447382	6.010984	5.615565	5.855901	5.825855	6.032774	5.763854	5.943097	5.70696	5.85037	5.943239
RGD1565512	-1.530507	-0.962426	-1.419824	0.176761	0.33325	-4.951771	0.643243	-3.260591	-1.093563	-1.788967	-1.087961	-1.612049	-0.831021	-0.654381	0.512061	-0.420466	1.290185	-0.920914	0.249639	0.119416
Fem1a	-0.236098	4.827989	-1.41858	0.177117	0.333853	-6.178846	5.017259	4.528825	4.78373	4.858787	4.808289	4.675081	4.736391	4.863466	4.775067	4.633691	5.021628	4.933912	5.256515	4.754829
RGD1307155	-0.298555	3.020678	-1.418459	0.177152	0.333853	-5.872395	3.239259	2.953644	2.645305	2.835056	2.962008	2.398644	3.083269	3.242343	3.375441	3.424979	3.031075	3.10114	3.051863	3.142871
Cope	0.258299	4.807804	1.418374	0.177176	0.333853	-6.122195	4.492273	4.637578	4.427251	4.038591	4.845505	4.164107	5.038651	5.127283	5.190313	5.059905	5.142525	5.007041	5.075785	5.121014
Abtb2	-0.352291	3.116036	-1.418319	0.177192	0.333853	-5.917557	3.357948	3.375827	2.645305	3.175097	3.060255	2.634313	2.880403	2.888628	3.280514	3.409193	2.968181	3.440827	3.134365	3.558103
Pc	-0.222815	6.917078	-1.418183	0.177231	0.33386	-6.260206	6.962236	6.889547	6.428109	6.625394	6.69444	6.273664	7.221224	7.118547	7.142521	7.016703	7.060759	7.115794	7.107463	7.196069
Polr2f	-0.220719	5.409228	-1.418111	0.177251	0.33386	-6.242455	5.73833	5.41815	5.527397	6.039842	5.73794	5.992964	5.288423	5.11075	5.218522	5.206391	5.146589	5.185663	5.152576	5.003257
Igf2r	-0.26249	6.904298	-1.418008	0.177281	0.333864	-6.25932	6.45121	6.110512	6.850152	6.927413	6.274994	6.471354	7.428763	7.175301	7.420635	6.96027	6.959881	7.087411	7.208778	7.347272
Cog2	0.344701	6.155886	1.417542	0.177415	0.334063	-6.238149	5.687857	6.582624	6.090428	5.546382	5.720517	5.454845	6.674202	6.272381	6.605444	6.507455	6.22449	6.179381	6.184203	6.475263
Spire2	-0.171545	4.762953	-1.417427	0.177448	0.334073	-6.172663	4.742602	4.747052	4.687307	4.919588	4.651639	4.565325	4.980068	4.592894	4.905145	4.815347	4.739035	4.66836	4.879797	4.845219
Ubqln1	-0.324423	7.437985	-1.416641	0.177673	0.334445	-6.250083	7.6796	7.025563	7.731923	8.180876	7.697217	7.969811	7.122438	7.262079	7.300967	6.905764	7.315688	7.186236	7.549211	7.213674
Brap	-0.2119	5.811864	-1.41637	0.177751	0.334539	-6.248941	5.823308	5.591938	5.793682	6.184497	5.706678	6.078208	5.721099	5.860878	5.656803	5.760748	5.991341	5.772746	5.81884	5.633383
Ptpro	-0.453659	5.85747	-1.416082	0.177834	0.334643	-6.229889	5.341141	4.167656	5.692309	5.993458	5.468031	5.506666	6.329365	6.09182	6.444585	6.050032	5.796935	6.280184	6.327473	6.545192
Steap3	-0.318257	3.7026	-1.415757	0.177927	0.334766	-6.03105	4.007445	3.397198	3.138503	3.644058	3.917788	3.57437	3.632178	3.734773	3.525993	4.005262	3.848089	4.153748	3.372046	4.007943
Mterf	0.350698	2.571055	1.415414	0.178026	0.334899	-5.75644	2.279379	2.506257	2.262807	2.283631	2.742832	3.014396	2.930765	2.522625	2.462438	2.694725	2.428842	2.529342	2.562371	3.041369
Taco1	-0.636428	1.870348	-1.414813	0.178199	0.335172	-5.686056	2.144973	0.426209	1.340699	2.463791	2.212449	2.714852	2.01015	2.186344	1.867508	1.942797	2.133674	1.488652	2.301782	1.415936
Aars2	-0.290785	4.481714	-1.414599	0.17826	0.335235	-6.111535	4.391979	4.479583	4.089343	4.239206	3.799498	3.751866	5.012583	4.639742	4.80328	4.839106	4.700858	4.677349	4.442376	4.950782
Usp14	-0.471297	7.242196	-1.414157	0.178388	0.335422	-6.259784	7.212207	6.213492	7.294425	8.112351	7.545754	7.707922	7.287948	6.94211	7.330886	6.777917	7.268925	6.948894	7.542932	7.215793
RGD1561648	0.664839	0.488115	1.413159	0.178675	0.335911	-5.262644	0.105685	1.679303	0.25957	0.041976	0.470544	0.527154	0.68572	-0.150641	1.29127	0.646479	0.321999	0.151783	0.546019	1.415936
March11	-0.42406	1.340062	-1.412909	0.178748	0.335994	-5.466696	0.955837	0.426209	0.851376	1.615848	1.05105	1.221719	1.862293	1.429209	1.794428	1.419918	1.290185	1.848377	1.79348	1.846283
Hspa4	-0.284846	8.291737	-1.411882	0.179044	0.336499	-6.242407	8.235667	7.681011	8.132081	8.686318	8.431791	8.458798	8.240821	8.262213	8.337938	8.098406	8.430309	8.226609	8.52352	8.343906
Me2	0.301327	4.778239	1.411688	0.1791	0.336552	-6.179549	4.559869	4.601207	4.764524	4.812037	5.378822	5.207652	4.531353	4.955841	4.363967	4.690302	4.784073	5.251348	4.608729	4.443728
Kbtbd2	-0.304201	5.102941	-1.411152	0.179255	0.33679	-6.217751	4.990561	4.610386	4.979663	5.656328	5.074352	5.423733	5.058606	5.010338	5.122264	4.766622	5.063063	5.208581	5.370586	5.152334
Abcc2	-0.475202	0.719384	-1.410857	0.17934	0.336898	-5.36557	0.785575	0.369671	0.20995	0.932396	0.210178	0.881356	0.952703	0.137473	1.180909	1.023513	0.764165	1.186803	1.28461	1.122575
Ppm1a	-0.315778	5.902633	-1.410064	0.17957	0.337276	-6.261942	5.908752	5.596564	5.992946	6.538322	5.796127	6.247674	5.679362	5.93475	5.820657	5.530243	6.027045	5.852956	6.189572	5.548486
Smpd4	0.292653	5.606776	1.409307	0.17979	0.337636	-6.231502	5.495845	6.029572	5.630004	5.117115	5.549226	5.18309	5.914952	5.702559	5.715715	5.794558	5.664347	5.509703	5.40053	5.820486
Cuta	0.253569	5.646641	1.409001	0.179878	0.33775	-6.237355	5.726612	5.726064	5.578391	5.087979	5.888175	5.432691	5.636383	5.63672	5.733744	5.954017	5.830157	5.714258	5.433489	5.706042
Bmf	-0.593929	1.92188	-1.40879	0.17994	0.337812	-5.571799	2.501925	1.117652	0.642822	1.065902	1.643636	1.302125	2.033384	2.80888	1.663747	2.438927	2.651054	3.38339	2.11575	2.011549
Per3	-0.297026	5.563126	-1.40832	0.180076	0.337984	-6.229407	5.568211	5.573282	5.174948	5.301019	4.936707	4.872329	6.062005	5.97641	5.962235	5.944466	5.692413	5.581691	5.455051	5.817698
Fnbp4	0.382796	7.39549	1.408281	0.180087	0.337984	-6.271357	6.916552	8.101029	7.663298	7.27162	7.094375	7.043864	7.595118	7.561806	7.528788	7.717196	7.195809	7.420651	6.932133	7.504201
Mmd	-0.354671	7.693357	-1.407695	0.180258	0.33825	-6.263131	7.714502	6.750473	7.711453	8.052022	7.603244	8.054218	7.796969	7.500198	7.755483	7.609935	7.739148	7.668816	7.961958	7.796352
Arfip1	0.245773	3.637319	1.407236	0.180391	0.338422	-6.046227	3.736577	3.707738	3.74196	3.606198	3.956574	4.240912	3.462819	3.653399	3.533498	3.335938	3.421519	3.594258	3.67737	3.380568
Stxbp5l	-0.606955	4.852033	-1.407186	0.180406	0.338422	-6.191068	4.399379	4.482919	4.944066	5.700631	3.633881	4.746268	5.100459	5.204627	4.939446	4.377964	5.38366	4.578447	5.871905	4.623699
Gtpbp3	0.483603	4.677377	1.407056	0.180443	0.33844	-6.116828	4.29216	5.31899	4.808946	3.82447	4.325951	3.703341	5.024227	4.836907	5.159118	5.008447	4.830277	4.769904	4.51603	5.130032
Ighmbp2	-0.159636	5.2585	-1.406664	0.180557	0.338598	-6.22804	5.290305	5.119134	5.219509	5.38167	5.190992	5.177772	5.286006	5.212396	5.356081	5.238011	5.332856	5.151664	5.455051	5.248633
Npr2	0.408841	6.080427	1.406536	0.180595	0.338598	-6.246949	5.513001	6.624755	5.710265	5.464109	5.519311	5.74226	6.570807	6.337772	6.44558	6.553959	5.861397	6.319953	5.838114	6.649221
Rasip1	0.389944	4.763745	1.406477	0.180612	0.338598	-6.160297	4.777095	5.302095	4.736324	4.058754	4.720868	4.446528	5.064257	4.70728	5.053459	5.150519	4.594813	4.625653	4.379868	5.13452
Wdr24	0.236062	5.703093	1.405909	0.180777	0.338816	-6.238928	5.386529	5.973765	5.653395	5.349294	5.516429	5.277267	6.019005	5.860878	5.921344	5.791517	5.843986	5.647211	5.691408	5.941959
Dnah1	1.208775	2.772854	1.405884	0.180785	0.338816	-5.475049	2.529164	1.151583	1.554575	-0.705589	2.651221	2.398644	4.150713	4.055878	3.577725	2.274406	3.537463	3.946285	4.052221	4.079517
Acp1	0.25193	2.766022	1.405696	0.180839	0.338835	-5.853593	2.957854	2.963229	2.969885	2.43939	2.856578	2.949304	2.828218	2.76729	2.928701	2.668442	2.627988	2.529342	2.771602	2.697558
Tnik	-0.414867	6.998857	-1.405656	0.180851	0.338835	-6.276644	6.831583	7.221491	7.149373	7.622263	6.042375	6.833386	7.243188	7.265195	7.338403	6.720712	6.879763	6.816145	7.197096	6.835784
G3bp1	0.178141	7.049443	1.404788	0.181104	0.339256	-6.277568	6.990684	6.997269	7.317671	7.104675	7.232481	7.357061	6.967467	6.929596	6.872814	6.833679	7.064522	7.044053	7.021557	6.970535
Cirh1a	0.205009	5.1848	1.404562	0.18117	0.339304	-6.244642	5.367791	5.326436	5.864543	5.516077	5.756376	5.635099	4.815587	4.968131	5.005695	4.794232	4.972116	4.848943	4.937104	4.823147
Grwd1	0.205347	4.224361	1.404507	0.181186	0.339304	-6.099377	3.992844	4.228535	4.380359	3.976352	4.132497	4.009049	4.34429	4.393186	4.347031	4.341	4.431229	4.346409	4.270617	4.032198
Msl3l2	-0.286161	3.386299	-1.40281	0.18168	0.34018	-6.021563	3.639931	2.943995	3.409716	3.701615	3.568609	3.595796	3.148563	3.509467	3.432752	2.966878	3.414796	3.549265	3.474137	3.203933
Ubr2	-0.251079	7.065492	-1.40261	0.181741	0.340236	-6.280753	6.560035	6.917549	6.509834	7.051332	6.30953	6.485772	7.701838	7.448506	7.555757	7.26383	7.124466	7.159219	7.379648	7.461971
Htr5b	-0.670633	4.441577	-1.402206	0.181859	0.340399	-6.032597	4.342933	1.854335	2.597868	3.431645	4.27571	4.03673	5.125	5.183701	5.38748	5.378399	5.276493	5.119046	4.982355	5.295477
Fam195b	0.303637	5.530635	1.402119	0.181884	0.340399	-6.254557	5.972153	5.999612	5.963305	5.120956	5.989324	5.42523	5.312364	5.326567	5.41415	5.353869	5.662929	5.206513	5.391239	5.325228
MGC112715	0.356049	6.021461	1.401794	0.181979	0.340506	-6.265403	6.072769	5.614923	6.012072	5.480644	6.46334	6.423477	5.251748	6.504198	5.471927	6.380641	6.36626	7.033006	5.544933	5.706042
Akap8	0.331939	7.434023	1.401731	0.181998	0.340506	-6.280382	6.9023	7.936826	7.341628	7.267294	7.102564	7.280568	7.819698	7.481566	7.615319	7.802754	7.235932	7.374876	7.157641	7.766663
Nadsyn1	0.275392	4.284844	1.400443	0.182375	0.341159	-6.111308	3.938008	4.515857	4.136585	4.07864	4.424547	4.082982	4.678618	4.264575	4.481148	4.382013	4.111876	4.216763	4.047497	4.710427
Tusc5	0.404888	1.310017	1.399724	0.182587	0.341445	-5.52803	1.278303	1.480065	1.534559	1.158502	1.877213	1.541762	1.511505	1.112357	1.102347	1.540753	0.535373	1.284167	1.54805	1.473753

Cd200	-0.320051	6.903072	-1.399659	0.182606	0.341445	-6.285201	6.759378	6.046578	6.538383	7.164514	6.943245	7.038496	6.890034	6.833791	7.026314	6.955523	6.917705	7.010656	7.173986	7.357866
S100b	-0.293124	11.01568	-1.399632	0.182613	0.341445	-6.148992	11.814	11.43403	11.29113	11.06623	11.02548	10.84796	10.47138	10.92765	10.60628	11.14991	10.90781	11.16903	10.72759	10.78188
Chrnb4	1.001685	0.305246	1.399313	0.182707	0.341567	-5.368439	-0.518419	2.843782	1.061799	1.300739	0.422095	1.378286	-1.009457	0.137473	-0.386063	-0.027758	0.263333	-0.78301	-0.040977	1.122575
RGD1306941	-0.262669	4.749788	-1.399069	0.182779	0.341648	-6.222022	4.870572	4.591969	4.89192	5.436636	4.897375	5.193668	4.667523	4.337436	4.564098	4.398098	4.650242	4.552625	4.942203	4.561731
Exosc5	0.282614	4.133229	1.398861	0.18284	0.341709	-6.115727	4.432216	4.538474	4.217279	3.622545	4.3745	4.06389	4.051782	4.015001	4.116621	4.145252	4.324082	3.961084	4.08486	4.007943
Map1lc3b	0.216458	8.069951	1.398491	0.182949	0.341859	-6.260804	8.451032	8.509471	8.477315	8.070511	8.695899	8.229793	7.65017	7.799999	7.776587	7.849563	8.054259	7.849677	7.922516	7.64847
LOC301124	0.363802	6.122559	1.397823	0.183145	0.342173	-6.277773	6.510498	6.590376	6.3938	5.526249	6.707133	5.8251	5.985148	5.680322	6.24649	6.11351	6.155594	5.902352	6.016686	6.085628
Mgat1	0.241538	5.702956	1.396966	0.183398	0.342576	-6.247743	5.633986	5.794776	5.562416	5.007277	5.454545	5.420735	5.883333	5.813989	5.789655	6.034681	5.955889	6.130034	5.594349	5.796614
Wrap53	0.289551	3.886553	1.396898	0.183418	0.342576	-6.074342	4.002595	4.286948	3.993619	3.486263	4.184254	3.642872	4.085519	3.798099	3.87219	4.020928	3.906764	3.789298	3.569478	3.880148
Sufu	-0.263958	3.268011	-1.39658	0.183512	0.342698	-6.004446	3.627383	3.099845	3.409716	3.437817	3.174343	3.359461	3.231264	3.431732	3.120298	3.258764	3.434871	3.18757	3.445698	2.697558
Shbg	-0.426243	3.425481	-1.396322	0.183588	0.34277	-5.917324	3.188908	3.263946	2.434653	3.114742	3.060255	2.230763	4.040359	3.979018	3.96946	4.281549	3.621611	3.619352	3.40196	3.911867
Rbm18	-0.256616	5.624067	-1.396256	0.183607	0.34277	-6.267057	5.733947	5.248229	5.814582	5.868808	5.649957	5.460695	5.801115	5.410013	5.743484	5.333104	5.936071	5.523062	5.775222	5.470756
Dbnl	0.153147	7.003579	1.395874	0.18372	0.342927	-6.289992	6.830214	6.955861	6.860239	6.844475	7.113571	7.031633	7.067928	7.08579	7.024984	7.159772	7.025338	7.124584	6.951858	6.986139
Gng5	0.320814	2.373476	1.395423	0.183853	0.343122	-5.798791	2.620642	2.963229	2.47722	2.108996	2.553396	2.714852	2.078759	2.287882	2.462438	2.600561	2.317912	2.136071	2.301782	1.910497
Tank	-0.291759	4.305939	-1.394691	0.18407	0.343473	-6.194139	4.579546	4.028502	4.438066	4.998953	4.617084	4.909165	4.023054	4.259584	3.985965	3.861859	4.345451	4.277141	4.143583	3.896095
Zadh2	-0.210717	4.530967	-1.394527	0.184118	0.34351	-6.195001	4.611755	4.649501	4.579097	4.917381	4.244704	4.760567	4.494157	4.511265	4.441807	4.571225	4.380377	4.412503	4.409621	4.517745
Ube3a	-0.425362	5.459397	-1.394219	0.184209	0.343626	-6.276886	5.348806	5.440624	5.667249	6.765093	5.333698	6.098051	5.708524	5.159792	5.161541	4.876899	5.088633	4.946361	5.649941	5.223575
Nostrin	0.44019	3.047907	1.394108	0.184242	0.343634	-5.972812	3.601952	4.364946	3.365078	2.687469	3.137307	3.438669	3.328389	2.454377	2.962454	2.572488	2.494078	2.703718	2.689088	3.069767
Cct3	0.258578	7.038076	1.393672	0.184371	0.343787	-6.292009	6.832266	6.758752	7.394988	7.187588	7.477382	7.445646	6.890829	6.916973	6.972806	6.83294	7.09112	6.796339	7.059589	6.887885
Add3	-0.183035	8.01901	-1.393638	0.184381	0.343787	-6.268723	8.32944	8.283292	7.982887	8.365008	8.158329	8.22915	7.77215	7.844425	7.751466	7.919834	7.738139	8.103071	7.913458	7.881587
Skp2	0.33933	3.386288	1.393539	0.18441	0.343788	-5.960679	2.917193	3.97739	3.215954	3.281878	3.306999	3.334188	3.824257	3.395791	3.627666	3.559779	3.022257	3.38339	3.32598	3.388105
Trim34	0.311025	3.97211	1.393217	0.184506	0.343913	-6.037245	3.881004	3.919485	3.489863	3.130069	3.935155	3.870884	3.844102	4.662608	3.824043	4.741628	4.421201	4.656286	3.416687	3.922287
Prpf18	-0.183363	6.265455	-1.3929	0.1846	0.344034	-6.286134	6.132823	6.213492	5.904083	6.373438	6.025245	6.141497	6.606102	6.171036	6.333971	6.352027	6.263366	6.338961	6.371603	6.509356
RGD1311558	-0.25634	6.161985	-1.39259	0.184692	0.344142	-6.286708	6.022141	5.631549	6.290916	6.555453	5.991401	6.216028	6.236068	6.148265	6.426561	6.225913	6.069397	6.138738	6.090872	6.246652
Pdcd2	0.287269	3.855505	1.39244	0.184736	0.344142	-6.09219	3.88628	4.216563	4.079015	3.747057	4.251652	3.862069	4.123906	3.690957	3.81791	3.790537	4.061116	3.277021	3.731382	3.551406
Ntn1	-0.639312	1.716035	-1.392386	0.184752	0.344142	-5.616626	2.246933	-0.186163	1.613009	1.571628	1.463853	2.05628	1.888013	1.856886	2.107651	1.780488	1.719153	2.475388	2.236876	1.73247
Mitf	0.333689	2.518681	1.392318	0.184772	0.344142	-5.885809	3.127854	3.354134	3.215954	2.363612	2.762423	2.907078	2.48486	2.24812	1.843557	2.39151	2.481265	2.330954	1.983515	2.050068
Tbcd	-0.190196	6.009599	-1.392154	0.18482	0.34418	-6.278974	5.995534	5.842297	5.729101	5.888093	5.861172	5.575685	6.409244	6.115493	6.252187	6.004794	6.154586	6.209646	5.992314	6.129006
Hmgn2	0.22651	4.600138	1.391861	0.184908	0.344239	-6.213755	4.757075	4.646529	4.983359	4.828572	5.084116	5.353145	4.330288	4.498613	4.12658	4.491011	4.431229	4.458423	4.148003	4.330935
Caap1	-0.361114	3.64292	-1.391772	0.184934	0.344239	-6.112979	3.742407	3.208609	3.62851	4.546747	4.124949	4.20302	3.734959	3.282262	3.472337	3.204921	3.609886	3.331185	3.536146	3.503633
Pxmp4	-0.317244	4.189474	-1.391756	0.184939	0.344239	-6.173599	4.754192	4.298352	4.149803	4.402734	4.329239	4.261168	3.850657	4.388622	4.523219	3.802672	4.49954	3.875144	3.602057	4.003043
Marf1	-0.283577	3.095003	-1.391565	0.184996	0.344292	-5.973375	3.522857	3.108509	2.885483	3.175097	3.333437	2.932562	3.36548	2.488905	3.09008	3.177227	2.921517	2.880186	3.152075	3.482664
Fam76a	-0.210542	5.511351	-1.391431	0.185035	0.344312	-6.274242	5.708854	5.386442	5.779933	5.89708	5.380408	5.820557	5.497094	5.285418	5.306568	5.46111	5.508891	5.460213	5.446106	5.25483
Mfsd12	-0.267578	4.871101	-1.390625	0.185275	0.344667	-6.169863	4.395683	3.909605	4.31439	4.638025	4.598129	4.174824	5.193638	5.297643	5.149382	5.114467	5.122035	5.564028	5.44431	5.336959
Trip12	-0.236912	7.683861	-1.390571	0.185291	0.344667	-6.285738	7.532566	7.508043	7.709223	8.145725	7.370133	7.817703	7.888603	7.673905	7.74259	7.417215	7.67202	7.567293	7.841948	7.69479
Tgds	0.359327	5.672628	1.390501	0.185312	0.344667	-6.24424	5.202087	6.082202	5.179789	5.021729	5.443967	5.190151	6.419201	5.667191	6.074529	6.053845	5.71311	5.638031	5.581336	6.181999
Eaf1	0.242972	5.067226	1.390062	0.185442	0.344825	-6.248915	5.069216	5.121276	5.811466	5.334484	5.403987	5.435664	4.812232	4.803005	4.71363	4.815347	5.067356	5.112433	4.874473	4.61409
Rnf121	-0.217477	3.866165	-1.390022	0.185454	0.344825	-6.092296	3.953171	3.61957	3.784955	3.946362	4.01491	3.508115	3.876582	3.749088	4.101553	3.973412	3.838074	3.800313	3.97969	4.088797
H2afx	0.274636	5.424259	1.389733	0.18554	0.344931	-6.280881	5.654231	5.551208	5.933043	5.910457	6.461094	6.282775	4.802122	5.016268	4.91667	5.092395	5.202306	5.22093	4.916528	5.017906
Fcgrt	0.555835	5.129484	1.388821	0.185812	0.345383	-6.205361	5.499292	5.131939	5.354078	3.68095	5.445483	4.546193	4.997894	5.121793	5.353963	5.726126	5.252185	5.922654	4.659142	5.16993
Rangrf	0.24778	3.709551	1.388707	0.185846	0.345393	-6.086806	3.793849	4.236461	3.688616	3.732068	4.220119	3.918424	3.577905	3.349568	3.487872	3.734632	3.56201	3.555779	3.445698	3.751656
Btbd6	-0.158374	6.347584	-1.388526	0.1859	0.345437	-6.294961	6.511351	6.151961	6.311647	6.333445	6.258704	6.332281	6.427994	6.289621	6.329667	6.367427	6.496133	6.283131	6.395962	6.396174
Kdelc1	0.291453	5.081931	1.388433	0.185927	0.345437	-6.227473	4.859884	5.64947	4.924971	4.899604	5.192798	4.936713	5.439557	4.983347	5.248472	5.190317	4.730939	4.789383	4.939655	5.409117
Pum1	-0.256851	6.355089	-1.387741	0.186134	0.345697	-6.298415	6.388452	6.021567	6.545902	6.871515	6.304527	6.62123	6.179914	6.380926	6.182295	6.150773	6.41508	6.29875	6.525596	6.104052
Satb2	-0.401265	3.231076	-1.387689	0.186149	0.345697	-6.086952	4.119218	3.176008	3.793402	3.82447	3.665442	3.595796	2.361772	3.660989	3.383769	2.342482	3.181236	2.619163	3.186856	2.503027
Ddx5	-0.213218	9.589638	-1.387674	0.186154	0.345697	-6.230492	9.474985	9.438445	9.426918	9.9198	9.360439	9.703917	9.879839	9.662285	9.709822	9.531749	9.511904	9.443784	9.58135	9.611749
Pde8a	-0.340096	4.859707	-1.387338	0.186254	0.34583	-6.20951	4.70428	5.108376	4.331892	5.017615	4.484758	4.209984	5.127701	5.05423	5.099692	5.059905	4.518478	5.357694	4.858381	5.158958
Pafah2	0.467603	1.254848	1.386802	0.186414	0.346074	-5.518264	0.739674	2.064844	1.669168	1.430203	0.88224	1.648138	1.335758	1.393205	0.884903	1.322149	1.375754	1.252437	1.040235	1.193114
Them4	-0.323601	4.100194	-1.386504	0.186503	0.346132	-6.159643	4.304037	3.486315	4.182328	4.526619	4.299374	4.380163	3.927077	4.020912	3.83015	3.796617	4.454361	4.032882	4.306643	3.948012
Loxhd1	1.246301	0.10016	1.386434	0.186524	0.346132	-5.353489	0.243629	2.519311	2.298852	0.166125	-0.831496	1.708392	-0.279766	-1.435005	-0.386063	-0.116374	0.263333	-0.78301	-0.305233	0.119416
March5	-0.307401	5.771612	-1.386409	0.186531	0.346132	-6.291359	5.974632	5.411164	5.972634	6.3351	5.702881	6.300826	5.712128	5.601653	5.661948	5.251354	5.809803	5.597563	5.995996	5.504087
Mgme1	0.32812	2.043956	1.386113	0.18662	0.346228	-5.793989	2.488111	2.493084	2.726949	2.093442	2.553396	2.623922	1.697535	2.099634	1.180909	1.355481	2.031907	2.187314	1.79348	1.684306
Calr3	-0.337513	2.123375	-1.386043	0.186641	0.346228	-5.720709	2.127257	1.854335	1.340699	2.213416	1.947363	2.271274	2.305626	2.207232	2.347926	2.256873	2.442128	2.390473	2.301782	2.087586
RGD1307752	0.204155	5.093344	1.385626	0.186766	0.346406	-6.226795	4.849116	4.909553	4.905621	4.858787	5.424116	4.9923	5.141132	5.130021	5.15669	5.233536	5.259708	5.13436	5.219198	5.138994
Lpin1	-0.300803	4.684427	-1.385496	0.186805	0.346424	-6.207993	4.689266	5.095359	4.643591	4.926188	4.140005	4.185462	4.753981	4.820056	4.670035	4.623466	4.638748	4.956243	4.803407	4.698352
Trappc6b	-0.281731	5.494015	-1.384485	0.187107	0.346932	-6.283397	5.644922	5.116989	5.509564	6.019411	5.608612	5.965451	5.375091	5.178422	5.568238	5.26018	5.582726	5.296777	5.612053	5.213005
Angel2	-0.174431	5.544301	-1.384228	0.187184	0.347021	-6.271447	5.460908	5.39532	5.326696	5.653679	5.254629	5.562143	5.758179	5.56975	5.775484	5.627248	5.561578	5.482608	5.680771	5.545119
Grid2ip	-0.428496	1.991892	-1.383948	0.187268	0.347124	-5.629552	1.594228	2.151237	1.340699	2.013053	1.173177	1.014597	2.943086	1.80302	2.41447	2.239124	2.014226	2.203999	2.66462	2.745289
Tbc1d24	-0.33375	5.686327	-1.383698	0.187343	0.347187	-6.289215	5.765785	5.496151	5.959557	6.186331	5.212518	5.90129	5.617289	5.587782	5.551703	5.197228	5.725387	5.560793	6.266789	5.611013
Dcakd	0.2747	6.481154	1.383641	0.18736	0.347187	-6.304858	6.473291	6.528775	6.583528	6.774267	7.235988	7.245837	5.77033	6.704789	6.02081	6.538624	6.508026	6.552699	5.988622	5.828818
Strada	0.228068	6.065234	1.383433	0.187423	0.347249	-6.292845	6.001624	6.281153	6.386839	5.814108	5.829016	6.042569	6.042098	6.197374	6.118855	6.317559	6.213879	6.009452	5.730244	5.952165
Slc25a29	-0.290402	4.15038	-1.382735	0.187632	0.347525	-6.138691	4.576285	4.256089	3.906747	3.857074	3.956574	3.568964	4.320877	4.084383	4.131534	4.277207	4.49954	4.368779	4.178567	4.212819
Scap	-0.184256	7.787485	-1.38269	0.187646	0.347525	-6.301367	7.635632	7.256019	7.484763	7.48558	7.444028	7.323742	8.207375	7.942277	8.128733	8.01905	7.810993	8.106126	7.9167	8.271127
Gpr107	0.167559	6.642503	1.382648	0.187658	0.347525	-6.303072	6.28838	6.662016	6.549647	6.426386	6.372238	6.516992	6.825781	6.653395	6.825452	6.690011	6.664988	6.825609	6.733244	6.976797
Atp2a1	-1.132031	1.080526	-1.382209	0.18779	0.347716	-5.455225	0.955837	-2.267963	1.858187	1.799605	0.985877	0.772673	0.68572	1.429209	2.313467	2.007132	1.533025	1.783252	0.879431	1.659607
Agap1	-0.249905	6.592315	-1.38178	0.187919	0.347901	-6.30732	6.692433	6.660544	6.660582	6.780958	6.263868	6.363845	6.523389	6.543738	6.52019	6.53953	6.376676	6.892112	7.003379	6.487594
Rps6ka6	-0.479813	2.224526	-1.381418	0.188028	0.348049	-5.731308	1.717752	2.383133	1.269823	2.738361	1.822268	1.766229	3.231264	2.227821	2.278164	2.202958	2.415434	2.187314	2.549064	2.70964
RGD1561795	-2.398967	-1.825389	-1.381322	0.188057	0.348049	-4.949072	2.41698	-2.680539	-1.909221	-2.731185	-2.352077	-2.747572	-1.21312	-0.992527	-1.813089	-0.116374	1.996325	-2.668207	-1.638202	-1.632384
Ube2j2	0.177401	6.105517	1.381136	0.188113	0.348099	-6.304242	6.353639	6.257977	6.452983	6.252601	6.630579	6.578148	5.910253	5.802024	5.81451	5.983632	6.021524	5.781109	5.839481	5.821878
Mecom	0.481904	2.676385	1.380955	0.188167	0.348146	-5.845891	3.05476	3.287027	2.718102	1.856	3.137307	2.504391	3.383673	2.227821	3.159628	2.668442	1.697158	2.346065	2.639729	3.050898
Arhgef1	0.398282	7.318211	1.380768	0.188224	0.348197	-6.308457	6.792072	7.713319	6.939442	6.487908	7.031172	6.464819	7.835894	7.574924	7.72139	7.868807	7.318392	7.709552	7.088044	7.91979
Rnpepl1	0.319728	5.993672	1.380618	0.188269	0.348227	-6.282941	5.698394	6.280195	5.662646	5.399171	6.101257	5.428219	6.15762	6.218103	6.144812	6.433297	6.154586	6.324728	5.618437	6.315465
Zfyve20	-0.263253	5.802321	-1.379923	0.188478	0.348503	-6.293278	5.646478	5.809463	5.893317	6.203639	5.245957	5.708237	5.857529	5.903095	5.803689	5.684108	5.899217	5.693731	6.115906	5.796614
Qk	-0.373653	4.220036	-1.379899	0.188485	0.348503	-6.230619	4.640141	4.535265	4.467397	5.355827	4.244704	5.278924	3.699086	4.123366	3.548392	3.676473	3.525031	4.12775	4.161181	3.786295
Lurap1l	-0.367457	2.46215	-1.379834	0.188505	0.348503	-5.770008	2.460078	1.279848	2.175023	2.283631	2.399242	2.101918	2.930765	2.436799	2.917273	2.543858	2.892776	2.475388	2.994118	2.879681
Ap5m1	-0.250715	4.165482	-1.379583	0.188581	0.348589	-6.182389	4.180631	4.313419	4.044046	4.802503	4.169655	4.484354	4.464549	3.763261	4.096495	3.740952	3.990531	3.926315	4.250206	4.178459
Crb2	0.865095	1.326899	1.379221	0.18869	0.348737	-5.402653	1.401336	0.533043	0.914692	-0.411255	1.745593	1.803538	1.443753	1.983359	1.393785	0.750369	1.557647	2.529342	2.041677	1.608903
Thoc1	0.444746	5.776825	1.37902	0.18875	0.348759	-6.279155	5.364014	6.703352	5.642915	5.461082	5.604547	5.492457	6.396981	5.697031	6.032774	6.096361	5.369823	5.448008	5.442513	6.153536
Peli1	-0.414292	2.740474	-1.378988	0.18876	0.348759	-5.841002	2.230432	2.248512	2.607481	3.145235	1.705671	2.613455	3.083269	3.126505	2.95129	2.854798	3.235656	2.980434	3.195422	2.635584
Farp1	0.361628	6.707939	1.378475	0.188915	0.348939	-6.31028	6.623809	7.578334	6.801776	6.325978	6.521986	6.44208	6.926946	6.474353	6.752567	6.946664	6.3452	6.74382	6.565792	6.877213
Tpgs2	-0.293699	6.140113	-1.378474	0.188915	0.348939	-6.310068	6.488115	5.756701	6.269126	6.740338	6.611126	6.644548	5.792629	5.95032	5.909857	5.834998	6.145478	5.815445	6.1885	5.837102
Ccdc47	-0.271903	8.242907	-1.377697	0.18915	0.349319	-6.28915	8.105995	7.77278	8.04124	8.667566	8.057362	8.584354	8.316168	8.184029	8.408254	8.154223	8.053989	8.212507	8.290277	8.557206
Abhd5	-0.275056	3.126385	-1.377432	0.18923	0.349389	-6.023798	3.821655	3.418258	3.289459	3.197095	2.970456	3.221545	2.73209	2.939445	3.038267	2.923085	2.86345	3.145002	3.195422	3.195366
Cpsf2	-0.334848	6.352994	-1.377352	0.189254	0.349389	-6.312456	6.375378	5.800134	6.564529	7.048814	6.329369	6.814663	6.162897	6.464627	6.292553	6.101282	6.349613	6.145232	6.45957	6.052809
Kif3b	-0.265212	6.656151	-1.377072	0.189339	0.349389	-6.313625	6.623809	6.089878	6.712728	6.905869	6.629246	6.565987	6.614792	6.756188	6.634524	6.403729	6.95757	6.648758	7.006424	6.652354
Tubb2a	-0.238568	6.7837	-1.37701	0.189358	0.349389	-6.313152	6.620647	6.067836	6.565762	6.767545	6.511893	6.675694	7.095785	6.741944	7.189594	6.659478	7.15605	6.828975	7.154899	6.950312
Ruvbl1	0.194631	4.499659	1.377001	0.189361	0.349389	-6.199023	4.439412	4.544872	4.605721	4.49737	4.845505	4.654809	4.425566	4.58892	4.526983	4.21501	4.549498	4.43741	4.442376	4.291288
Acy1	0.305349	5.212114	1.376994	0.189363	0.349389	-6.25874	5.401346	5.670101	5.102017	4.768627	5.730498	5.047769	5.236813	4.995405	5.218522	5.3205	4.91609	5.168764	5.066477	5.369687
Gipc1	0.225133	6.11608	1.376546	0.189498	0.349389	-6.302843	6.216891	6.2433	6.130151	5.67212	6.372238	5.924761	6.20954	6.067753	6.170309	6.198623	6.274549	6.089079	5.92952	6.149107
Nqo2	0.42367	3.053751	1.376498	0.189513	0.349389	-5.939467	3.180343	3.466233	2.787402	2.568755	4.023054	2.898483	3.072094	2.926907	2.973531	3.040455	3.031075	3.10114	2.760099	3.11589
Rbm38	0.242273	3.75731	1.376479	0.189519	0.349389	-6.140571	4.064414	3.843675	4.166157	3.950685	4.508156	4.452412	3.280644	3.542716	3.448717	3.58107	3.61576	3.656194	3.178239	3.432519
Cebpg	0.219877	4.751318	1.376468	0.189522	0.349389	-6.226532	4.621279	4.567042	5.044792	4.814411	4.972895	5.158103	4.531353	4.778791	4.635579	4.716194	4.804791	4.733017	4.754973	4.443728
Tmem37	0.80435	0.299231	1.376393	0.189545	0.349389	-5.3039	0.830061	-0.186163	1.195284	-0.705589	0.917619	1.014597	-1.21312	0.923327	-0.504027	0.646479	0.721218	1.783252	-0.040977	0.257494
Timp2	0.358225	7.578828	1.376376	0.18955	0.349389	-6.308266	7.832972	7.450854	7.991631	7.020308	7.839476	7.86755	6.878052	7.464859	7.28605	7.487022	7.632003	7.905253	7.699797	7.756144
Grin3a	-0.348191	2.707005	-1.376323	0.189566	0.349389	-5.855862	2.501925	2.439156	1.996308	2.925676	2.586747	2.537984	3.280644	2.077116	3.440756	2.06872	2.401899	2.941167	3.522595	3.432519
Pim3	0.440809	5.31975	1.37612	0.189627	0.349448	-6.256381	5.195727	6.359457	5.271833	4.809659	5.500476	4.652254	5.469678	5.119041	5.349718	5.573377	5.182653	5.344586	5.391239	5.29748
Lrif1	-0.254249	4.004183	-1.375848	0.18971	0.349547	-6.211763	4.384541	4.188236	4.519244	5.034003	4.545387	4.586885	3.987806	3.201289	3.555781	3.368956	3.555912	3.454835	3.969738	3.808933
Palmd	-0.291802	5.803517	-1.375478	0.189822	0.3497	-6.307118	6.189409	5.341214	5.99935	6.239412	6.168883	6.175691	5.790926	5.609519	5.803689	5.412451	5.827629	5.316091	5.889168	5.514455
Amigo3	0.411108	2.324886	1.374838	0.190016	0.350004	-5.674091	2.035255	2.383133	1.996308	1.353927	2.183675	1.785004	3.060831	2.454377	2.694377	2.795315	2.455292	2.542521	2.269694	2.868948
Slc31a1	-0.416473	4.49148	-1.374335	0.190169	0.350185	-6.246224	5.843793	4.502772	4.811028	4.39641	4.695613	4.746268	3.863678	4.171987	4.060584	4.328465	5.032648	4.323687	4.233667	3.942904
Paox	0.209963	4.690851	1.374321	0.190173	0.350185	-6.196734	4.62444	4.843622	4.566831	4.207046	4.669909	4.407682	4.67123	4.820056	4.71693	4.824303	4.85532	4.872617	4.895654	4.757741
Ndel1	-0.335934	6.389077	-1.374038	0.190259	0.35029	-6.318574	6.668133	5.983217	6.955997	7.287085	6.598919	7.026712	6.047814	6.073452	6.150923	6.043656	6.309401	6.063022	6.291969	5.96608
Nsun5	0.284822	4.254956	1.373714	0.190357	0.350417	-6.138569	4.064414	4.678883	4.172648	3.847833	4.28928	3.830786	4.464549	4.264575	4.316905	4.574767	4.208334	4.316033	4.148003	4.475916
Vcp	0.169008	8.353306	1.373469	0.190432	0.350501	-6.2975	8.092648	8.0717	8.326142	8.167939	8.421237	8.379946	8.444124	8.481144	8.447267	8.295092	8.48087	8.449586	8.493445	8.399968
Chrm5	-0.908864	2.181922	-1.373346	0.190469	0.350516	-5.694425	1.957182	-1.10215	1.613009	2.523044	2.460496	2.398644	2.582602	2.709903	2.962454	1.920699	2.556491	2.703718	2.902922	2.900911
Pin1	-0.204348	6.435139	-1.373136	0.190533	0.35058	-6.317848	6.82816	6.266712	6.398653	6.473711	6.630579	6.488638	6.423604	6.192145	6.503091	6.397741	6.568338	6.197199	6.428117	6.313488
RGD1565033	-0.215344	3.878443	-1.373039	0.190563	0.350581	-6.134372	4.031456	4.138342	3.789185	4.113757	3.670635	3.862069	3.797364	3.727562	3.895674	3.989425	4.004032	3.772618	3.665088	3.948012
Tcam1	-0.638922	1.66025	-1.372613	0.190692	0.350677	-5.48893	1.108109	-0.687936	1.245402	1.405223	0.422095	1.519512	2.450751	2.58779	2.524011	2.499819	2.117203	2.170435	2.236876	2.294252
Pvrl3	-0.406187	4.181099	-1.372518	0.190721	0.350677	-6.160268	3.987945	3.34683	3.95649	4.643375	4.01082	4.333098	3.969854	4.438054	4.36817	3.721909	4.338363	4.285003	4.921699	4.303297
Rbmx2	0.230395	3.552014	1.372491	0.190729	0.350677	-6.106416	3.502386	4.028502	3.914512	4.014017	3.917788	4.135132	3.200804	3.449372	3.510866	3.249928	3.302737	3.433771	3.116435	3.088394
Mfsd10	0.516772	4.906742	1.372476	0.190734	0.350677	-6.154294	4.304037	5.38466	4.419086	3.69132	4.622454	3.62735	5.538282	5.196815	5.41415	5.580442	4.944375	5.401751	5.006894	5.622242
Mien1	-0.231022	3.707023	-1.372276	0.190795	0.350677	-6.194732	4.481855	4.037603	4.072089	4.543889	4.412197	4.608127	3.072094	3.047633	3.197914	3.302147	3.331578	3.18757	3.502026	3.229331
Acvr1b	-0.277053	5.838542	-1.372259	0.1908	0.350677	-6.27958	5.392103	5.116989	5.716941	5.560592	4.989615	4.967859	5.974683	6.420591	6.239624	6.067738	6.233114	6.28215	6.45957	6.347713
Adsl	0.2413	6.708872	1.372195	0.190819	0.350677	-6.315546	6.330601	6.621732	6.304278	6.214464	6.686771	6.44208	7.070738	6.991109	6.963113	7.036747	6.722604	6.695967	6.608109	7.251354
Rbl2	-0.168983	6.302714	-1.371906	0.190908	0.350785	-6.316886	6.426071	6.446598	6.131818	6.471456	6.369845	6.173912	6.44542	6.150963	6.266894	6.260416	6.207086	6.271306	6.22974	6.406417
Rps12	0.574019	0.367613	1.371391	0.191064	0.35102	-5.277416	-0.310971	1.117652	0.398943	0.166125	0.691028	-0.214525	0.357848	0.517885	0.628769	0.938085	0.484915	0.34469	0.649393	0.606649
RGD1560289	0.397366	3.118815	1.371253	0.191106	0.351043	-5.928028	2.742017	3.96313	2.954902	2.835056	2.781752	3.114281	3.428178	3.282262	3.298245	3.030171	3.013384	2.970717	2.923692	3.510555
Rrad	0.624574	2.074555	1.37093	0.191205	0.351142	-5.642916	2.01613	2.943995	2.025285	0.966949	2.308867	1.107027	2.05625	2.14364	2.611707	2.14695	1.996325	2.433555	2.11575	2.570827
Trim41	-0.159655	6.395606	-1.370885	0.191219	0.351142	-6.316122	6.296329	6.219503	6.321902	6.343342	6.118463	5.981819	6.51516	6.491483	6.540818	6.631689	6.537724	6.534675	6.466665	6.557778
Upf2	-0.28401	6.139339	-1.370644	0.191292	0.351202	-6.318777	6.098001	6.03299	6.083556	6.918062	6.136405	6.648399	6.07466	6.027216	5.978822	5.805152	6.024839	5.920132	6.227653	6.005816
Senp2	-0.288871	5.27429	-1.370501	0.191336	0.351202	-6.287742	5.365903	5.108376	5.599017	5.78172	5.06057	5.373446	5.075493	5.463078	5.241615	4.830243	5.39907	5.155958	5.493764	4.932857
LOC687399	-0.465978	1.348954	-1.370491	0.191339	0.351202	-5.620988	1.895723	0.632508	1.631973	1.571628	1.113405	1.627481	0.903114	1.393205	1.520039	0.981432	1.674823	1.219993	2.022549	1.32462
Atp5c1	-0.189722	7.465468	-1.369528	0.191633	0.351688	-6.317342	7.599548	7.194788	7.3326	7.633733	7.711461	7.469781	7.577938	7.306311	7.587198	7.37365	7.500071	7.265868	7.501654	7.470872
Med6	0.221294	3.886772	1.368273	0.192017	0.352338	-6.150419	3.953171	4.104095	4.058135	4.058754	4.371314	4.380163	3.830902	3.713031	3.682701	3.478908	3.901965	3.692118	3.828196	3.468514
Nav2	0.277974	7.129301	1.368032	0.192091	0.352338	-6.325589	6.644196	7.358086	7.399836	7.184839	6.794177	7.218473	7.409469	7.2552	7.317907	7.183747	7.11309	6.967336	6.827184	7.148046
B3galnt1	0.22433	5.409934	1.367986	0.192104	0.352338	-6.270542	4.938178	5.064523	5.458689	5.278844	5.50775	5.30192	5.417651	5.359594	5.56641	5.522927	5.348815	5.595984	5.578065	5.837102

Nr2f2	-0.376164	4.605775	-1.367985	0.192105	0.352338	-6.275067	5.218914	4.313419	5.187821	5.516077	5.114924	5.33574	4.248266	3.550909	4.815644	3.401235	4.287749	3.662244	5.059455	4.839733
Hyal3	0.317294	4.033901	1.367515	0.192249	0.352501	-6.147733	4.193457	4.75259	4.477916	3.721989	4.12116	3.727807	3.939429	3.942114	3.980484	3.826639	3.958531	3.869926	4.116777	3.942904
P2rx2	-0.613517	0.737077	-1.367504	0.192252	0.352501	-5.468963	1.48718	0.310827	1.340699	0.785264	0.808768	-0.371994	1.091998	0.871929	1.180909	0.981432	0.805871	0.007173	0.598632	1.415936
Crtac1	-0.328985	7.350628	-1.367025	0.192399	0.352717	-6.326517	7.099176	7.313351	6.991305	7.440572	7.184271	6.273664	7.568525	7.423064	7.927691	7.556745	7.284681	7.233221	7.58347	8.039189
Katnb1	0.270437	5.736107	1.366678	0.192505	0.352858	-6.296283	5.774348	5.87567	5.918636	5.164405	5.884827	5.262271	5.962031	5.808873	5.890993	5.818659	5.762929	5.591235	5.706469	5.913516
Nsmce4a	0.209401	4.102832	1.366509	0.192557	0.352899	-6.167146	3.94308	4.138342	4.421813	4.274118	4.424547	4.294309	3.987806	3.910622	4.170562	3.721909	4.082483	3.975732	3.994491	4.19144
Ppp2r2d	-0.192255	4.24716	-1.366253	0.192636	0.352989	-6.196524	4.307974	4.019342	4.263615	4.591724	4.36492	4.389395	4.14005	4.101219	4.175368	4.210463	4.254248	4.170823	4.282726	4.271049
Zfp867	-0.387946	2.623703	-1.365623	0.192829	0.35329	-5.885117	2.765113	2.082541	2.487668	2.853641	2.254562	2.854719	2.854546	3.000548	3.01701	2.707689	3.065821	2.100868	2.575555	2.372328
Tmem180	0.274314	5.527178	1.364475	0.193182	0.353821	-6.260295	5.099062	5.440624	5.197401	4.716273	5.361263	4.719677	5.894478	5.839298	6.065498	5.929327	5.678448	5.862183	5.607246	6.005816
Mtif3	0.286323	3.433292	1.364453	0.193189	0.353821	-6.085685	3.718942	3.459476	3.656563	3.425447	4.128728	4.14243	3.105363	2.964197	3.027678	3.327565	3.414796	3.346294	3.318158	3.178079
RGD1310587	-0.309071	4.63765	-1.364392	0.193207	0.353821	-6.242465	5.210525	4.796145	4.287732	4.566597	4.430682	4.798021	4.884284	4.055878	4.953501	4.51702	4.518478	4.634911	4.40224	4.935431
Fbxl3	-0.255681	5.696918	-1.364076	0.193304	0.353886	-6.315379	5.652684	5.39	5.749866	6.282058	5.615361	6.095233	5.740641	5.545351	5.555394	5.576914	5.581226	5.728743	5.645259	5.628619
Lyplal1	-0.316723	3.548689	-1.363996	0.193329	0.353886	-6.156374	3.953171	3.317238	3.656563	4.405886	4.165982	4.323498	3.410541	3.137429	3.271566	3.249928	3.394437	3.018661	3.294432	3.220915
Ikzf5	-0.313568	4.963067	-1.36399	0.193331	0.353886	-6.300312	5.323746	5.046602	5.611014	5.983999	5.016379	5.690919	4.421169	4.771798	4.65291	4.307328	4.755092	4.606955	4.893023	4.454537
Slc38a4	0.700346	0.680123	1.362782	0.193703	0.354493	-5.362597	0.243629	1.047298	0.750864	0.22441	1.258434	0.571087	1.091998	-1.196868	2.024877	0.591587	0.378374	-0.434228	1.700256	2.446395
Kdm5a	-0.205872	5.884206	-1.362721	0.193722	0.354493	-6.319809	5.791322	5.73728	6.012072	6.279483	5.630099	5.942639	5.914952	5.870731	5.917047	5.774675	5.81747	5.871352	6.058371	5.788093
Pf4	1.004003	-0.361692	1.362476	0.193797	0.354577	-5.252973	0.692264	-0.572649	1.705429	-1.223791	0.027932	1.014597	-0.529223	-0.81357	-0.27702	-1.33445	-0.149817	0.403657	-0.893866	-0.886923
Insr	-0.309452	4.97601	-1.362313	0.193848	0.354615	-6.262616	4.865238	4.655425	5.273343	5.304401	4.265449	4.907024	5.203913	5.096827	5.227804	4.680472	4.913708	4.997502	5.338076	4.985976
RGD1563692	-3.184011	-3.275426	-1.362147	0.193899	0.354655	-4.834669	2.035255	-3.260591	-2.787702	-3.260591	-3.260591	-3.260591	-2.558588	-3.260591	-3.260591	0.280604	2.707156	-2.249472	-3.260591	-2.653844
Smtn	0.351173	3.870092	1.361961	0.193956	0.354706	-6.110295	4.092076	4.47624	3.772191	3.261145	3.948045	3.875271	3.993741	3.804966	4.050158	4.051757	3.487079	3.869926	3.542874	4.065483
Cog4	-0.19763	5.961266	-1.361807	0.194004	0.354739	-6.311436	5.907455	5.58264	5.372519	5.639022	5.898172	5.44897	6.188995	6.114111	6.214166	6.213469	6.279184	6.130034	6.252494	6.2425
Myl9	0.618574	4.739047	1.361319	0.194154	0.35496	-6.203395	5.11488	4.904587	3.797607	3.36843	5.592283	5.055522	4.798736	4.540363	4.970188	5.183373	4.79447	4.752996	4.55649	4.981001
Slc25a37	0.348652	2.181837	1.361068	0.194232	0.354988	-5.753094	1.874641	2.854122	2.2133	2.045748	2.386673	1.839907	2.206902	2.307359	2.127623	2.256873	2.149958	2.220493	2.11575	2.294252
Mrpl37	0.191956	5.632156	1.361032	0.194243	0.354988	-6.313822	5.768645	5.647985	6.012072	5.622861	6.06917	5.813716	5.424258	5.559634	5.589995	5.557352	5.564618	5.271276	5.490287	5.490147
Adipor2	-0.316599	4.327081	-1.360982	0.194258	0.354988	-6.217893	4.707265	4.200444	4.617663	4.529512	4.082714	4.284446	4.051782	4.166665	4.029076	4.145252	4.148808	4.811327	4.780817	4.102607
Murc	-0.5684	0.550603	-1.360887	0.194288	0.354988	-5.331658	0.176305	-0.103798	0.105278	0.74601	-0.33871	0.117189	0.498056	1.68885	1.061401	1.704918	0.676952	0.889587	0.879431	0.753927
Gpr101	-0.992282	0.390578	-1.360695	0.194347	0.355011	-5.288372	-0.310971	-2.267963	0.25957	1.188094	0.15195	0.175273	0.563361	1.067371	1.393785	0.591587	0.535373	0.28321	1.815874	1.708589
Serpinb6b	-0.489519	0.868701	-1.360656	0.194359	0.355011	-5.494955	1.212615	1.369077	-0.067513	1.065902	0.952152	0.571087	1.047044	1.155984	1.180909	1.023513	0.676952	1.082389	0.649393	1.122575
Sox17	0.469539	3.553071	1.360317	0.194464	0.355148	-6.013238	3.556342	3.812199	2.924461	2.687469	4.094356	3.49677	3.939429	3.221962	3.87219	3.984107	3.373787	3.78376	3.310292	3.825682
Wiz	0.15425	6.638213	1.359914	0.194589	0.355321	-6.335238	6.752171	6.804792	6.714953	6.437209	6.748244	6.725749	6.613829	6.638093	6.552869	6.607683	6.512756	6.614847	6.583847	6.643721
Psmd14	0.214858	5.779843	1.359584	0.194691	0.355454	-6.318239	5.622966	5.624015	5.967977	5.882448	6.14576	6.133278	5.671979	5.674709	5.854008	5.564496	5.855203	5.653298	5.722856	5.573492
Brd8	-0.196276	6.508344	-1.359178	0.194816	0.355629	-6.337379	6.550928	6.548011	6.740844	7.092012	6.484138	6.729388	6.44542	6.398059	6.469252	6.365383	6.380994	6.259382	6.330392	6.339961
Gpr22	-0.652278	6.325389	-1.359077	0.194848	0.355633	-6.329509	5.948388	4.146778	6.105771	6.938337	6.238736	6.656705	6.642444	6.597137	6.675802	6.384683	6.675572	6.294861	6.709519	6.563614
Vti1a	-0.338372	3.836676	-1.358619	0.19499	0.355837	-6.194726	4.184919	3.673115	4.051108	4.638025	4.195107	4.383247	3.169687	3.770297	3.963917	3.158464	3.916315	3.482452	3.913738	3.326676
Psap	-0.312423	11.24336	-1.35852	0.19502	0.35584	-6.22077	11.38184	10.33784	10.79289	10.56276	11.01518	10.51391	11.39812	11.62975	11.58019	11.59118	11.69293	11.87876	11.26133	11.77107
Uba7	0.754236	3.130412	1.358137	0.195139	0.356002	-5.765661	2.843156	3.302211	3.028308	1.000693	2.308867	1.821837	3.554005	4.481568	2.894141	4.502215	4.039427	4.913769	2.333172	3.041369
LOC100365008	0.296469	3.328907	1.357832	0.195234	0.356074	-5.987521	3.035892	3.719097	3.570714	3.010938	3.011968	2.982217	3.383673	3.386664	3.392049	3.463704	3.243265	3.461789	3.536146	3.56477
RT1-M3-1	-0.244801	4.225698	-1.357818	0.195238	0.356074	-6.157937	4.082914	4.12132	3.468922	4.132904	3.926497	3.980827	4.506663	4.299035	4.421726	4.595839	4.444493	4.568818	4.195746	4.500463
Cops5	0.180627	5.734693	1.357175	0.195437	0.356384	-6.315351	5.543377	5.724656	5.746607	5.698063	5.931005	5.805694	5.809551	5.648222	5.879261	5.573377	5.879821	5.622601	5.874574	5.578442
Cldn4	0.68408	0.143734	1.356931	0.195513	0.356421	-5.553298	0.692264	2.01041	2.356997	1.353927	1.390206	1.046069	-1.450335	-1.435005	-1.536754	0.591587	-0.731011	0.403657	-1.047095	0.321874
Fam211b	-0.477067	1.327579	-1.356919	0.195517	0.356421	-5.619955	1.936985	0.854759	1.169557	1.379803	1.803478	0.881356	1.372662	1.239467	1.29127	0.981432	1.430105	1.593679	2.203293	1.085968
Suv39h2	-0.231629	3.214882	-1.35679	0.195557	0.35644	-6.064707	3.402817	3.240489	3.525794	3.721989	3.500244	3.076678	3.231264	3.047633	3.059216	2.877922	3.099749	3.065063	3.278395	3.050898
Slc25a38	0.256278	4.457526	1.355245	0.196037	0.357261	-6.203635	4.573017	4.822713	4.483148	3.950685	4.481806	4.257812	4.438678	4.429191	4.334197	4.502215	4.714609	4.401696	4.51603	4.571694
Srek1ip1	-0.238553	3.962074	-1.354843	0.196162	0.357435	-6.244399	4.618111	4.167656	4.427251	4.897366	4.829341	4.657358	3.554005	3.431732	3.383769	3.471326	3.853071	3.56872	3.445698	3.270693
Abhd14a	-0.295208	3.714417	-1.354744	0.196193	0.357437	-6.112373	4.137033	3.812199	3.171281	3.52156	3.706477	3.450478	3.850657	3.550909	3.634661	3.734632	4.017407	3.625558	3.892823	4.017694
Abcc1	0.515789	5.226086	1.354596	0.196239	0.357467	-6.241657	4.573017	5.843595	5.512125	4.461469	3.97768	4.80033	5.933595	5.512163	5.820657	5.457277	5.305858	5.261346	5.324473	5.427521
Mcmdc2	-0.274039	3.328231	-1.354264	0.196343	0.357601	-6.007874	3.073383	3.01022	2.753168	3.474303	3.122223	3.179592	3.844102	3.567159	3.641623	3.424979	3.461211	3.245131	3.278395	3.67978
Gdf15	1.381399	-1.044986	1.353799	0.196488	0.35781	-5.135294	0.739674	-0.813242	1.451597	-2.344749	0.266146	-0.291112	-2.088409	-0.654381	-2.155202	-0.537962	1.290185	0.151783	-3.260591	-1.212031
St3gal6	-0.35317	4.252525	-1.353545	0.196567	0.357901	-6.200697	4.611755	3.96313	3.359399	4.214255	4.460974	4.467019	4.197745	4.182573	4.086326	4.592349	4.570823	4.695162	4.018826	4.20003
Emc9	-0.241612	3.970753	-1.353273	0.196652	0.358001	-6.138403	4.069061	4.055636	3.575619	3.829173	3.607042	3.611659	4.161298	3.818604	4.421726	4.205901	4.048142	3.956168	4.033233	4.299305
Hexim2	0.296846	3.513545	1.353128	0.196697	0.358029	-6.069618	3.576066	4.028502	3.843073	3.233029	3.546179	3.365711	3.479833	3.542716	3.392049	3.552611	3.812726	3.10114	3.372046	3.482664
LOC691931	-0.363889	1.838972	-1.352665	0.196841	0.358194	-5.694148	1.717752	1.047298	1.905712	2.077719	1.840817	1.353343	1.888013	1.983359	1.959523	1.852296	2.100543	2.082939	2.333172	2.050068
Man1c1	0.257708	5.624355	1.352648	0.196847	0.358194	-6.288852	5.178626	5.179997	5.438634	4.986375	5.425653	5.30192	5.505426	6.074873	5.777065	6.107336	5.821288	6.468031	5.488545	6.024072
Chmp5	-0.262925	5.954422	-1.352499	0.196893	0.358224	-6.338285	6.18192	5.522322	5.891351	6.381465	6.225563	6.434668	5.899229	5.885385	5.777065	5.746684	5.945431	5.758697	5.962514	5.775217
Usp18	0.766137	2.246951	1.35224	0.196974	0.358317	-5.61172	1.853246	1.991799	1.825611	0.621404	2.212449	1.766229	2.828218	3.413873	1.843557	3.186517	2.519368	4.16231	1.465469	2.159814
LOC689574	0.18589	5.056509	1.351322	0.197261	0.358767	-6.324716	5.827428	5.690441	5.772474	5.495514	5.979941	5.936354	4.223227	4.639742	4.380707	4.693564	4.884809	4.546096	4.372333	4.399663
Nkap	-0.281229	3.734319	-1.351205	0.197298	0.358767	-6.150976	3.870393	3.486315	4.089343	4.185201	3.428477	3.972661	3.662299	3.741948	3.722643	3.417108	3.901965	3.600573	3.866246	3.454224
Eif2b1	0.19645	4.810031	1.351162	0.197311	0.358767	-6.254731	4.765689	4.934131	4.886008	4.594489	5.103448	4.576145	4.92833	4.710941	4.768722	4.81235	4.820137	4.778284	4.908735	4.809178
Rhbdd3	0.295259	4.054378	1.350693	0.197458	0.358926	-6.049182	3.474632	3.934181	3.347976	3.091442	3.741449	3.288866	4.567614	4.360928	4.384862	4.683756	4.309659	4.674359	4.449555	4.555051
Tfg	0.184714	6.146429	1.350692	0.197458	0.358926	-6.34655	6.488983	6.509279	6.655378	6.596031	6.975604	6.768239	5.733565	5.795142	5.777065	5.665956	5.847735	5.603863	5.921797	5.734407
Arf1	-0.346719	7.777807	-1.35044	0.197537	0.359015	-6.330554	8.247247	7.060029	8.072594	8.094638	8.019445	8.155988	7.449423	7.773985	7.687313	7.479011	7.936575	7.585314	7.896485	7.43857
Gsr	-0.375806	5.222434	-1.35001	0.197672	0.359172	-6.318537	5.306139	4.766343	5.202167	6.062986	5.2	6.072488	5.224248	4.986371	5.086997	4.894014	5.293084	4.851593	5.200171	5.0106
Ccdc61	0.384408	3.490054	1.349973	0.197683	0.359172	-6.014819	3.163058	4.046648	3.565791	3.002635	3.391209	2.881136	3.756062	3.599118	3.408468	3.784431	3.537463	3.52291	3.318158	4.027379
Zfp563	-0.318496	3.22604	-1.349511	0.197828	0.35938	-6.055422	3.334978	2.570375	3.376367	3.670505	3.238702	3.53054	2.991343	3.292072	2.962454	3.158464	3.352837	3.284884	3.529386	3.041369
Tada3	0.192833	6.031956	1.348451	0.198161	0.35993	-6.332308	5.898344	6.068946	5.868547	5.607886	6.104139	5.737448	6.186407	6.251165	6.15701	6.190565	6.150545	6.042296	5.952442	6.25595
Gng3	-0.394542	7.3207	-1.348193	0.198242	0.359982	-6.334391	7.902322	7.123916	7.592154	8.392079	7.999978	8.294078	6.905857	6.965286	7.232877	6.485981	7.167094	6.568086	7.219842	6.650788
Amigo2	-0.452001	4.729319	-1.34817	0.198249	0.359982	-6.216161	4.759952	3.894655	3.505372	4.277563	5.028566	3.844276	5.688539	4.927801	4.855111	4.987339	5.117902	4.659314	5.280867	5.4493
Thap11	-0.206758	4.427307	-1.34779	0.198369	0.360127	-6.210804	4.460789	3.958345	4.179109	4.308205	4.390327	4.167688	4.606864	4.523808	4.670035	4.505931	4.83532	4.49955	4.463807	4.486487
Rab21	-0.223731	5.922451	-1.347725	0.198389	0.360127	-6.34203	5.977107	5.563863	5.949199	6.334273	6.101257	6.113917	5.902387	5.704396	5.9925	5.824622	5.758953	5.863497	6.117033	5.737361
Sntb2	-0.50787	0.816616	-1.347135	0.198574	0.360409	-5.423907	0.955837	0.118359	0.945338	0.621404	-0.516166	0.61372	1.258992	1.155984	0.931093	1.253084	1.290185	1.152831	1.377871	1.227132
Dtnbp1	0.221582	4.651554	1.346811	0.198676	0.36054	-6.23223	4.499177	4.827969	4.448801	4.328278	4.8386	4.428729	4.760958	4.584936	4.881816	4.856672	4.850346	4.641051	4.569728	4.667715
Stambpl1	-0.224465	5.534232	-1.345938	0.198951	0.360984	-6.329586	5.497569	5.596564	5.341172	5.979775	5.544991	5.590436	5.768601	5.601653	5.477774	5.532066	5.591695	5.310324	5.442513	5.238245
Gpr83	-0.543708	4.867363	-1.345639	0.199045	0.3611	-6.214994	4.502616	2.667383	3.963992	4.555288	4.955978	4.297582	5.354483	5.165139	5.491325	5.064951	5.182653	5.053812	5.948647	6.005816
Impact	-0.23308	8.684145	-1.345197	0.199185	0.361298	-6.317716	8.54681	8.806613	8.563336	9.04569	8.368879	8.503589	9.096167	8.453821	8.892059	8.724433	8.568473	8.372579	8.683539	8.955911
Tjp1	-0.217928	8.157142	-1.343988	0.199566	0.361935	-6.328246	8.276173	8.273695	8.185967	8.743336	7.995846	8.513825	8.083457	8.103456	8.052102	7.867003	7.795873	8.015695	8.084899	8.020821
Cox16	0.255702	2.67062	1.343619	0.199682	0.362092	-6.002977	3.197423	3.216645	3.184186	2.853641	3.529124	3.158148	2.550752	1.983359	2.185932	2.325762	2.442128	2.568523	2.236876	2.211707
Clptm1	-0.22908	8.399529	-1.343089	0.19985	0.362341	-6.33521	8.346527	7.682099	8.056673	8.278029	8.385162	8.213643	8.434339	8.53867	8.594227	8.612784	8.458367	8.666754	8.523945	8.80695
Apaf1	0.378077	4.544144	1.342872	0.199919	0.362388	-6.258982	4.15463	4.432057	5.102017	4.986375	4.777328	5.499579	4.36733	4.584936	4.351284	3.850214	4.53717	4.365074	4.449555	4.229697
Glyr1	-0.267904	6.676222	-1.34281	0.199938	0.362388	-6.358593	6.978384	6.532803	7.092321	7.185756	6.644503	6.987229	6.288911	6.726654	6.500222	6.202063	6.741533	6.496245	6.796545	6.309524
Dnajb9	-0.305257	5.203267	-1.342578	0.200011	0.362388	-6.331523	5.641806	4.744275	5.554984	5.806973	5.726763	5.637684	4.812232	4.658822	4.945085	4.941431	4.882375	5.023584	5.252416	5.261001
Tmem194	0.521437	0.938846	1.342558	0.200018	0.362388	-5.390588	-0.046857	1.369077	0.642822	0.621404	0.770579	0.481842	1.862293	1.020936	1.061401	1.28803	1.199219	0.969826	1.150071	1.608903
Armc8	-0.169658	5.921252	-1.342472	0.200045	0.362388	-6.341618	5.7514	5.573282	5.798935	6.045916	5.741646	5.804544	6.136317	6.006512	6.08988	5.922393	6.104287	5.885643	6.094312	5.968596
Slc24a5	-0.504181	1.365557	-1.342435	0.200057	0.362388	-5.611844	1.310059	1.914859	0.642822	1.760745	1.082564	0.734546	2.033384	1.747064	1.663747	1.780488	1.375754	1.045835	1.077784	1.582867
Mapk8ip3	-0.257753	9.066455	-1.341934	0.200215	0.362581	-6.311668	8.868575	9.351479	8.781387	9.333881	8.501759	8.724681	9.245088	9.179483	9.214872	9.085151	9.073583	9.105298	9.262786	9.205333
Plekha1	-0.194264	6.875993	-1.341893	0.200228	0.362581	-6.359789	6.658143	6.902682	6.687457	7.163583	6.642522	6.635525	7.186669	6.878425	6.972116	6.888078	6.761542	6.915727	6.9398	7.045134
Vezt	0.155894	5.945963	1.341813	0.200254	0.362581	-6.340097	5.707365	5.992608	5.936861	5.782933	5.884827	5.791836	6.128247	5.930045	6.083504	6.013956	5.814919	5.992746	5.997222	6.212036
Mbp	-0.654524	13.02542	-1.341356	0.200398	0.362788	-6.169251	13.22872	13.53628	11.73943	12.61291	12.48624	11.28031	13.70951	13.22651	13.69243	13.70364	12.63512	13.49986	13.01102	13.99416
Upf3b	0.223083	5.969007	1.341174	0.200456	0.362838	-6.35457	5.926787	6.588055	6.396575	6.542624	6.263009	6.588873	5.88812	5.719014	5.661948	5.724532	5.600609	5.54123	5.531464	5.619042
Wnt5a	0.447213	1.591334	1.340719	0.2006	0.363045	-5.622471	1.401336	1.453108	2.10888	1.188094	1.143601	2.131561	1.697535	1.658838	1.843557	1.28803	1.260496	1.738143	2.186205	1.708589
Ubb	-0.213011	8.363616	-1.340117	0.200791	0.363335	-6.333764	8.553044	7.941682	8.379014	8.421185	8.533284	8.248937	8.434066	8.341699	8.57094	8.243447	8.475024	8.195399	8.494747	8.262952
Slc26a10	0.499476	4.053784	1.339974	0.200837	0.363347	-6.110983	3.516065	4.63158	3.719973	3.450082	4.191499	3.426763	4.781687	4.213873	4.64946	4.49849	3.882605	3.900956	3.542874	4.44734
Smim8	-0.280028	3.028347	-1.339896	0.200861	0.363347	-5.985141	2.937667	2.324846	2.795835	3.233029	3.099296	3.006419	3.270902	3.159031	3.333065	3.080873	3.181236	2.910998	3.152075	3.106782
Naprt1	0.392206	4.580122	1.339703	0.200923	0.363347	-6.182956	4.319721	4.763603	3.843073	3.556013	4.606283	4.08677	4.571588	5.019224	4.778229	5.339365	4.904139	5.151664	4.24196	5.0106
LOC691153	-0.437562	2.931506	-1.339697	0.200925	0.363347	-5.990593	3.821655	2.991607	3.021133	2.534609	2.373993	2.469996	3.094359	2.555575	2.870631	3.090803	3.149191	3.055902	3.467079	2.756979
Nrn1l	-0.825916	-0.012358	-1.33962	0.200949	0.363347	-5.295651	1.48718	-0.813242	-0.195392	-0.81875	0.091273	-1.117764	-0.399109	0.301855	0.091681	0.847278	1.403186	0.666985	-0.211665	0.257494
Tctn2	-0.236115	4.57628	-1.339483	0.200993	0.363371	-6.244096	4.816322	4.707679	4.293698	4.33821	4.268877	4.094316	4.835551	4.65122	4.759151	4.66726	4.661644	4.581643	4.576302	4.883051
Pfkl	-0.216982	7.962967	-1.339169	0.201092	0.363471	-6.348026	7.989479	7.437593	7.898286	8.011993	7.829352	7.973907	7.906016	8.079583	8.145666	7.881019	8.168569	7.918582	8.290527	7.957323
Phb	0.194413	5.009343	1.339118	0.201109	0.363471	-6.319283	5.292294	5.259942	5.317939	5.282277	5.770958	5.574336	4.641297	4.658822	4.676828	4.696819	4.867681	4.744468	4.789329	4.607648
Tmem138	0.235493	3.650559	1.338863	0.201189	0.36353	-6.083146	3.582581	3.874479	3.426105	3.211576	3.706477	3.513754	3.830902	3.698353	3.722643	3.850214	3.925804	3.698019	3.608485	3.584586
Marveld1	0.405395	3.306343	1.338825	0.201202	0.36353	-6.008756	3.730722	3.08236	3.203331	2.255954	3.639189	3.49677	3.231264	3.292072	3.307029	3.559779	3.434871	3.656194	3.116435	3.447025
Rab38	-0.566685	1.112386	-1.338284	0.201374	0.363786	-5.711874	1.212615	1.369077	0.642822	2.687469	1.96438	1.839907	0.282279	0.871929	0.091681	0.981432	1.260496	1.691577	0.491415	0.970221
Pgm1	-0.217029	6.226702	-1.337574	0.201599	0.364139	-6.359447	6.625388	6.339356	5.984671	6.142603	6.433098	5.910938	6.169466	6.176342	6.20597	6.204351	6.291163	6.253384	6.25044	6.207783
Mir6315	-0.417077	1.043199	-1.33747	0.201633	0.364145	-5.605233	1.594228	0.725555	1.293837	1.353927	0.649545	1.541762	1.372662	1.020936	0.97585	0.699359	0.805871	1.315214	1.316372	0.706485
Gsk3b	-0.483065	5.442807	-1.337278	0.201694	0.364175	-6.352976	5.799735	5.196344	6.166373	6.558288	5.280338	6.135109	4.771359	5.412264	4.884753	4.657271	5.549353	5.181457	5.904922	4.74018
Socs2	-0.370485	2.577224	-1.337198	0.201719	0.364175	-6.001209	2.896425	2.582864	3.077557	3.456176	2.424057	3.165332	2.165439	2.738882	2.446625	1.804823	2.673757	2.284645	2.66462	1.971974
Emc10	0.234715	6.971825	1.337131	0.20174	0.364175	-6.364575	6.750726	6.722527	6.771631	6.507112	7.237302	6.720881	6.852153	7.021646	7.239202	7.147931	7.019811	7.314696	6.996655	7.316002
LOC306766	-0.156141	7.437357	-1.336692	0.201881	0.364373	-6.354592	7.984875	7.56502	7.674775	7.673178	7.758561	7.69743	7.183427	7.173312	7.194337	7.255025	7.280989	7.222513	7.26835	7.200357
Cbln1	0.625317	3.557348	1.336109	0.202066	0.364654	-6.258973	5.069216	5.916945	5.393507	3.810268	3.97768	4.909165	2.841442	2.14364	1.914249	2.274406	3.695584	2.461578	2.964355	2.610029
Pgap1	-0.343959	4.17084	-1.335626	0.20222	0.364877	-6.177177	3.713016	3.889638	4.026238	4.458437	3.280067	3.821723	4.595201	4.490115	4.508062	3.784431	4.390692	4.281077	4.844833	4.399663
Map4k3	-0.283504	7.192039	-1.335256	0.202339	0.365036	-6.36641	6.977767	6.606522	7.072503	7.66498	7.054206	7.41584	7.596094	7.158642	7.302615	6.985772	7.229242	6.935843	7.428606	7.27085
Mrpl3	-0.183905	5.892306	-1.335106	0.202386	0.365067	-6.362641	6.266292	6.008897	6.176904	6.48642	6.132647	6.452394	5.497094	5.634794	5.648188	5.49889	5.748297	5.627247	5.731717	5.609402
Tgfb3	-0.380602	4.263481	-1.334523	0.202573	0.365348	-6.213312	4.251852	3.099845	3.929918	4.433945	4.725866	4.067729	4.648839	3.966821	4.639062	4.311581	4.095154	4.204378	4.722744	4.676974
Ptk7	0.344667	4.07144	1.333887	0.202776	0.36566	-6.149135	4.280186	4.073446	4.139901	3.122426	3.917788	3.94371	4.150713	4.254576	3.895674	4.19213	4.345451	4.647164	3.794763	4.338735
Thumpd2	-0.266469	3.683889	-1.332824	0.203116	0.366218	-6.1205	3.652372	3.724743	3.196978	3.670505	3.378569	3.3278	4.233294	3.741948	3.842287	4.061889	3.70112	3.83286	3.542874	3.791988
Cyp51	-0.256184	7.705806	-1.332714	0.203151	0.366227	-6.349518	8.232563	7.734463	7.755623	8.323529	8.19416	8.403108	7.700932	6.747302	7.726719	7.05968	6.922453	7.240315	7.854171	7.994214
Hsd17b7	-0.443375	1.680347	-1.331914	0.203408	0.366634	-5.684936	1.717752	1.655851	1.905712	1.837445	1.143601	0.695385	1.809437	2.007376	2.204856	1.451089	2.100543	1.805289	2.11575	1.582867
Atg10	-0.310338	2.04852	-1.3317	0.203477	0.366703	-5.839509	2.357438	1.894965	1.936547	2.401998	2.198134	2.271274	1.726334	2.077116	1.579251	2.127786	2.442128	2.475388	2.220182	1.355706
Ndufc2	-0.274797	6.065446	-1.331554	0.203523	0.366727	-6.367511	6.347914	5.678853	6.019292	6.517348	6.386512	6.546175	5.970175	5.447802	6.034097	5.990279	5.888339	5.883055	6.104581	6.125629
Eea1	-0.283473	6.266753	-1.331468	0.203551	0.366727	-6.36971	6.155943	5.870585	6.463608	6.994894	6.248321	6.587537	6.366431	6.194762	6.201266	5.960802	6.048919	6.036486	6.369712	6.25595
Pdzd7	0.559688	4.69724	1.331211	0.203633	0.36682	-6.236122	4.264063	6.018122	4.462108	4.140492	4.581681	4.028875	5.193638	4.498613	5.185557	4.922095	4.411103	4.376159	4.44597	5.29748
Ugcg	-0.346684	7.20921	-1.331101	0.203669	0.366829	-6.371693	6.913325	6.272505	7.260554	7.75238	7.139555	7.269362	7.10541	7.243241	7.318993	7.191843	7.239266	7.356804	7.44576	7.430837
Hras	-0.277126	6.300466	-1.33088	0.203739	0.366902	-6.372039	6.696193	5.93654	6.301319	6.711972	6.612476	6.856315	6.419201	6.066325	6.202444	6.120798	6.276405	5.917605	6.185278	5.923924
Zfp622	-0.192267	3.139826	-1.330393	0.203896	0.367129	-6.066458	3.431974	3.134193	3.104962	3.348912	3.320278	3.235263	3.094359	3.137429	3.120298	3.129854	3.065821	2.921125	3.098279	2.992761
Cops2	-0.286401	6.413358	-1.329478	0.20419	0.367581	-6.375306	6.693938	6.077797	6.664617	7.117708	6.839268	6.895178	6.200588	6.150963	6.166694	5.915425	6.369741	6.038813	6.534907	6.140208
L1cam	-0.204161	8.474928	-1.329421	0.204208	0.367581	-6.353155	8.150253	8.298989	8.150898	8.411221	8.039657	7.811993	8.957294	8.560394	9.015434	8.625008	8.403181	8.479017	8.69113	9.05911
Ulk1	-0.187619	7.096347	-1.328843	0.204394	0.367861	-6.375475	7.111055	7.256019	7.026665	7.298153	7.005712	6.778816	7.212981	6.972929	7.188406	7.17327	7.062373	6.975862	7.001548	7.29661

Pde7a	0.299265	3.89922	1.328637	0.204461	0.367925	-6.170742	3.529616	4.57956	4.123244	4.050723	3.836938	3.879645	4.145391	3.95452	3.952765	3.702612	3.603988	3.761389	3.602057	3.973287
Atg9a	-0.180621	8.087021	-1.328421	0.20453	0.367947	-6.364815	7.877367	7.609187	7.71006	7.816843	7.869992	7.478097	8.338201	8.313381	8.468222	8.398402	8.156782	8.338983	8.266084	8.582679
Ap1m2	0.896998	-0.824759	1.32841	0.204534	0.367947	-5.217091	-0.634519	1.79116	-0.263851	-0.705589	-0.257548	-0.457681	-0.279766	-1.720374	-1.536754	-0.665882	-0.510232	-0.920914	-1.413173	-1.040339
F3	0.229144	5.08423	1.327793	0.204732	0.368249	-6.345749	5.665015	5.517451	5.68211	5.292531	5.532208	6.083906	4.498338	4.58892	4.564098	4.933176	4.832801	4.941394	4.409621	4.686175
Hn1	-0.233866	6.209217	-1.327538	0.204815	0.368342	-6.369534	6.397718	5.71192	6.029161	6.106253	6.260428	6.066746	6.372137	6.219389	6.35106	6.282246	6.583404	6.182542	6.40891	5.978616
Ptgs2	-0.599517	5.012071	-1.327367	0.20487	0.368385	-6.311489	4.713215	3.354134	5.202167	5.802197	4.355276	5.692163	5.032899	5.302504	5.343328	4.879766	5.166736	5.044579	5.272795	5.060981
Ppip5k1	-0.256852	7.357584	-1.326929	0.205011	0.368584	-6.375885	7.124512	7.576774	6.905264	7.663664	6.929229	7.131747	7.744259	7.300248	7.703205	7.435839	7.338957	7.222001	7.374942	7.565314
Itga10	-1.103008	0.200375	-1.326682	0.205091	0.368673	-5.631573	1.741237	3.24835	1.451597	1.799605	-0.954037	-0.548781	-0.279766	-1.435005	0.736738	1.21727	-0.855515	0.007173	-1.638202	0.257494
Lrrc47	-0.179667	6.022978	-1.326438	0.205169	0.368731	-6.359217	5.926787	5.699071	5.826978	5.738624	5.705414	5.378091	6.362996	6.32827	6.207144	6.25601	6.335443	6.186745	6.237019	6.157952
Zfp90	0.258301	3.852529	1.326326	0.205206	0.368731	-6.140421	3.689063	4.271601	3.750662	3.533136	3.780406	3.68846	4.118484	3.741948	4.106593	4.12123	3.897149	3.744381	3.748946	3.853172
Cdyl2	-0.425314	2.268268	-1.326296	0.205216	0.368731	-5.857671	2.311111	2.01041	2.466695	2.634716	1.43972	2.297664	1.726334	2.555575	2.539002	2.342482	2.568656	2.100868	2.902922	2.194616
Pkig	0.199785	6.472332	1.326165	0.205258	0.368752	-6.379147	7.14996	7.046567	7.125735	6.747188	7.019516	7.401463	5.873711	6.049074	6.011435	6.341669	6.187498	6.010638	5.838114	5.828818
Bphl	-0.330738	2.74387	-1.325899	0.205344	0.36883	-5.935232	3.118917	2.279532	2.122353	2.463791	2.77212	2.634313	3.049479	2.988532	2.834633	3.139454	2.995472	2.529342	2.923692	2.802815
Ercc2	0.293068	5.583926	1.32584	0.205363	0.36883	-6.324929	5.16132	5.822686	5.392117	5.038072	5.44245	4.919823	6.010249	5.78302	5.788087	5.955377	5.590204	5.811368	5.449691	6.044486
Adc	0.319405	3.626974	1.325579	0.205447	0.368927	-6.090558	3.474632	3.953545	4.02265	3.182467	3.326873	3.255598	3.804135	3.413873	3.729193	3.867646	3.858035	3.854157	3.646466	3.517445
Serpinf1	0.562008	3.663605	1.32515	0.205586	0.369121	-6.165328	4.235407	3.287027	4.139901	3.130069	4.436792	4.961683	2.48486	4.090016	3.027678	4.020928	3.280721	4.114573	2.62712	3.591131
Shq1	0.411994	1.020314	1.324866	0.205678	0.369231	-5.610271	1.245833	1.279848	1.936547	1.097434	1.05105	1.943815	0.798508	1.279465	0.736738	0.799637	0.885831	0.714338	0.546019	0.753927
Spata5	0.288796	3.10787	1.324233	0.205882	0.369512	-6.043428	2.967843	2.884706	3.540923	3.218762	3.546179	3.524966	2.688847	2.781287	3.09008	3.019813	3.212584	3.092205	3.152075	2.972849
Igfbp3	0.621941	3.6897	1.324191	0.205896	0.369512	-6.228519	3.907194	3.184227	5.261214	4.232121	4.718362	5.540207	2.967416	2.926907	3.006262	2.966878	3.480655	2.869767	3.445698	3.286911
Hrh1	0.569754	3.091353	1.32389	0.205993	0.369632	-6.028206	3.474632	2.904741	4.617663	2.43939	3.210452	3.143674	2.67414	3.012464	2.95129	2.529327	2.958968	3.16218	3.409342	2.982839
Ifngr2	0.203944	6.292942	1.323631	0.206078	0.369727	-6.375376	6.080846	6.189194	6.574979	6.249096	6.50682	6.203871	5.875319	6.404856	6.164279	6.407706	6.466371	6.690057	6.27186	6.036115
Cdk13	-0.183953	6.09781	-1.323483	0.206126	0.369758	-6.374674	5.983276	6.040932	6.161487	6.48642	5.933168	6.072488	6.258424	5.980965	6.16186	6.01526	6.075804	6.035321	6.008203	6.178743
Col27a1	-0.364775	7.529251	-1.323366	0.206163	0.369766	-6.379013	7.653535	7.495712	6.953173	7.364047	6.861581	7.266859	8.16888	6.972929	8.130281	7.60518	8.012845	7.355405	7.407103	8.170917
Tmem98	-0.518583	3.91527	-1.323279	0.206191	0.369766	-6.108956	4.22295	3.375827	2.238266	3.137672	4.043216	3.006419	4.228269	4.134313	4.15118	4.494755	4.493172	4.800396	4.094051	4.514305
Kat2a	0.268856	7.296447	1.322341	0.206496	0.370257	-6.383759	6.878007	7.401915	6.846605	6.634155	7.27146	6.576802	7.712674	7.550613	7.625889	7.676151	7.39212	7.552304	7.310853	7.731174
Foxo4	-0.338888	5.367404	-1.322227	0.206533	0.370267	-6.33133	5.613453	5.536837	4.586407	5.036039	4.989615	4.843511	5.817937	5.214976	5.568238	5.821644	5.180673	5.657847	5.453267	5.863025
Med30	0.237873	3.839281	1.322074	0.206582	0.370267	-6.187111	3.870393	3.914553	4.061636	3.852461	4.531181	3.879645	3.706333	3.492551	3.742206	3.808701	3.797302	3.822092	3.713602	3.667444
Tamm41	0.246942	3.758787	1.321983	0.206612	0.370267	-6.169198	3.896775	3.649562	3.851187	3.606198	4.237722	4.213454	3.669732	3.734773	3.824043	3.602051	3.673224	3.536147	3.783444	3.461387
Oser1	0.226505	4.264004	1.32185	0.206655	0.370267	-6.27475	4.464322	4.382912	4.666764	4.555288	4.836291	5.049711	3.96382	4.123366	3.81791	3.7783	4.172914	4.132116	3.924083	3.911867
Kcnh7	-0.641184	3.281722	-1.321849	0.206656	0.370267	-6.045067	3.026365	2.758277	2.999392	3.761892	1.643636	3.678453	3.537848	4.12885	3.620637	2.438927	3.656224	3.440827	4.156802	3.278825
Abhd2	-0.402541	5.263952	-1.321712	0.2067	0.370291	-6.337595	5.926787	4.699101	5.495395	4.939298	4.779733	5.09931	4.96806	5.418994	5.491325	4.815347	5.519896	5.641098	5.730244	5.213005
Tmem164	-0.204275	5.574857	-1.321424	0.206793	0.370404	-6.36106	5.719239	5.352198	5.806258	5.768314	5.242473	5.750642	5.430835	5.56975	5.385408	5.608362	5.70762	5.721519	5.453267	5.565205
Map6	-0.164054	6.841603	-1.321291	0.206837	0.370426	-6.385847	6.780076	6.7663	6.71662	7.049318	6.765337	6.755207	6.838203	6.798095	6.917236	6.983109	6.662862	6.920151	6.834743	7.008928
Triqk	-0.409829	2.87132	-1.320848	0.206981	0.370628	-6.021026	3.334978	1.914859	2.893363	3.059778	3.313654	2.949304	3.049479	2.76729	3.253503	2.529327	3.082885	2.284645	3.278395	2.70964
Nop16	0.237469	5.113795	1.319283	0.20749	0.371485	-6.34844	5.298244	5.234443	5.618164	5.383269	5.8691	5.585089	4.965043	4.750611	4.881816	4.865376	5.103341	4.444449	4.877138	4.763548
Tram1	0.226496	6.576394	1.318732	0.20767	0.371751	-6.388431	6.647307	6.695467	6.986708	6.889076	6.951803	7.345645	6.016092	6.384369	6.209488	6.359234	6.263366	6.590467	6.319658	6.427601
Aaed1	-0.420092	0.921316	-1.318444	0.207764	0.371823	-5.495462	1.071521	0.480615	0.484899	0.74601	0.470544	0.571087	1.17791	1.198329	1.180909	0.799637	1.652137	1.252437	1.651284	1.009844
Cenpc	-0.258105	4.528624	-1.31842	0.207772	0.371823	-6.286427	4.388265	4.24827	4.311452	5.062243	4.469939	4.867933	4.760958	4.552657	4.57868	4.341	4.411103	4.372473	4.621498	4.482972
Elmod3	0.323732	3.350544	1.318295	0.207812	0.371826	-6.025838	3.410162	3.53854	3.222225	2.523044	3.313654	3.014396	3.529702	3.321106	3.577725	3.232094	3.487079	3.827486	3.4385	3.629792
Mgat5b	-0.376008	5.890976	-1.318225	0.207835	0.371826	-6.365467	6.001624	5.853937	6.008449	5.630964	5.231972	4.66498	6.236068	5.964191	6.241917	6.274643	5.889551	6.119079	5.976249	6.408271
Tmem158	-0.381589	4.5452	-1.317844	0.207959	0.371993	-6.152266	3.601952	2.631765	3.863274	4.054744	3.696327	3.55809	5.006725	5.074265	5.343328	5.09979	5.422719	5.509703	5.485056	5.369687
Wars	0.190589	7.207331	1.317722	0.207999	0.372009	-6.386159	7.192643	7.285925	7.727797	7.431323	7.589256	7.408669	6.930818	7.079432	7.122133	6.865841	7.189422	7.00947	7.100069	6.980541
Tma16	0.390855	2.124692	1.316967	0.208246	0.372394	-5.907711	2.035255	2.064844	2.893363	2.738361	2.910256	3.22842	1.697535	1.883083	1.769222	1.597576	1.883989	1.783252	2.079189	1.556352
Lsm5	0.394169	1.282983	1.316527	0.208389	0.372596	-5.62902	1.245833	2.01041	1.269823	1.158502	1.578815	1.541762	1.754571	0.517885	1.218642	1.14286	1.375754	1.219993	1.252133	1.32462
Hsbp1	0.192996	8.169861	1.316315	0.208459	0.372664	-6.363589	8.455254	8.469465	8.439804	8.247391	8.846767	8.423098	7.995615	7.816235	7.988339	7.861938	8.111247	7.824609	8.050197	7.853618
Gchfr	0.682294	-0.057802	1.315505	0.208724	0.373083	-5.348836	0.485316	-0.186163	0.049949	-0.600663	1.143601	1.165512	-0.279766	-0.81357	-0.080923	-0.420466	-0.072212	-0.334482	0.698429	0.119416
Acd	0.306052	6.721317	1.315054	0.208872	0.37326	-6.390782	6.55921	7.213984	6.638481	6.350721	6.896356	6.294288	6.980227	6.685381	7.004204	6.978436	6.572119	6.560008	6.390377	6.989859
Lrrc28	-0.178243	4.310067	-1.315012	0.208885	0.37326	-6.258001	4.323616	4.112733	4.308508	4.512069	4.329239	4.213454	4.425566	4.406794	4.254698	4.290193	4.480351	4.179285	4.345646	4.238063
Ngfrap1	-0.276681	7.504512	-1.31392	0.209243	0.373844	-6.376951	7.996205	7.337505	7.996898	8.276954	8.042164	8.06602	7.079833	7.284365	7.413535	6.9623	7.363167	6.955683	7.306412	6.991097
Cask	-0.264319	4.159928	-1.313641	0.209335	0.373952	-6.264591	4.346765	4.192317	4.475294	4.627263	3.922149	4.307355	3.927077	4.123366	4.06577	3.676473	4.116027	3.995035	4.579577	3.973287
Etfb	-0.274016	5.202474	-1.312693	0.209646	0.374452	-6.346306	5.187202	5.037557	4.899765	5.499945	5.122525	5.233512	4.92212	5.449994	5.117279	5.535706	5.02825	4.94388	5.65771	5.242409
Mpg	0.279942	2.699625	1.312334	0.209764	0.374608	-5.909405	2.645748	2.943995	2.568639	2.154676	2.752661	2.422822	2.534559	2.988532	2.870631	2.90068	3.004456	2.838051	2.816716	2.59708
Acot3	-0.193832	4.107416	-1.312235	0.209796	0.37461	-6.234599	4.17633	4.279295	3.784955	4.267203	4.027109	4.056182	4.034614	4.055878	4.096495	4.336834	4.168924	4.105722	4.08486	4.120817
Fgf22	-0.327206	2.422727	-1.312087	0.209845	0.374641	-5.797223	2.387517	1.972946	1.451597	1.818649	1.947363	1.747207	2.905804	2.849305	2.858731	2.514649	2.739799	3.065063	3.013624	2.962789
Pxdn	-0.328266	6.782017	-1.311808	0.209937	0.374749	-6.382874	5.668082	5.88956	5.892335	6.406069	5.469522	5.595763	7.789739	7.431964	7.987656	7.655391	7.068276	7.164024	7.158189	7.789254
Mark4	-0.148755	7.666534	-1.311712	0.209968	0.37475	-6.385572	7.81821	7.617557	7.861601	7.870586	7.586511	7.718643	7.582867	7.643098	7.615319	7.588193	7.692662	7.530973	7.632789	7.580196
Zmynd11	-0.176211	8.338749	-1.311492	0.210041	0.374798	-6.368635	8.335238	8.294962	8.333401	8.689393	8.230442	8.481637	8.487356	8.248738	8.455192	8.26991	8.192842	8.094987	8.223531	8.409721
Rab4b	0.228192	6.102569	1.31144	0.210058	0.374798	-6.38202	6.182992	6.125528	6.173672	5.474653	6.158755	5.756599	6.188995	6.153655	6.1811	6.214605	6.368001	6.2403	6.128261	6.112039
Lpcat3	0.17277	5.886435	1.311104	0.210168	0.37494	-6.374577	5.695391	5.783999	5.88049	5.595517	5.796127	5.80799	5.865643	5.936314	5.934159	6.148398	5.838973	6.254385	5.849014	6.050436
Wfdc2	0.746935	1.100091	1.310824	0.210261	0.374998	-5.617683	2.246933	2.200694	2.039558	-0.093872	1.705671	0.949514	0.498056	0.818632	1.255413	1.14286	1.978199	0.618025	0.598632	0.257494
Slc35d1	0.21691	4.007055	1.310816	0.210263	0.374998	-6.197358	4.073693	4.155166	4.159638	3.617117	3.973484	3.922669	4.085519	3.825375	4.029076	3.913127	4.021838	4.265266	4.052221	4.102607
E4f1	0.38826	5.772046	1.310683	0.210307	0.375009	-6.352886	5.319852	6.284024	5.394896	5.091898	5.497557	5.207652	6.461569	5.805453	6.172714	6.270281	5.733515	5.883055	5.465713	6.250792
Smad3	-0.369727	6.138298	-1.310518	0.210361	0.375009	-6.385867	5.867997	4.919435	6.144254	6.337577	5.556259	6.300826	6.238569	6.550905	6.275871	6.410682	6.624045	6.461974	6.008203	6.263141
Hgs	0.162307	7.322103	1.310424	0.210393	0.375009	-6.394792	7.412452	7.451705	7.683606	7.295606	7.601885	7.330148	7.056632	7.258331	7.222468	7.246167	7.267526	7.170936	7.265302	7.25651
Fbxw2	-0.193299	6.460898	-1.310417	0.210395	0.375009	-6.398607	6.599119	6.531999	6.690287	6.939969	6.338372	6.745654	6.408134	6.333029	6.47023	6.063962	6.355768	6.287052	6.455119	6.251825
Avil	-0.378608	2.530636	-1.310158	0.21048	0.375106	-5.925941	2.670424	1.854335	2.225837	2.677071	2.72297	2.374054	2.286414	2.695192	2.41447	2.795315	2.228732	3.300483	2.838755	2.622863
Plcxd3	-0.650604	1.753881	-1.309782	0.210604	0.375271	-5.845971	2.488111	1.010791	2.434653	2.376521	1.85913	1.747207	2.078759	1.532126	1.326257	0.20947	2.556491	0.804622	3.02328	1.634478
Arsi	0.748365	0.943814	1.309646	0.210649	0.375288	-5.431268	0.692264	-0.273517	1.493674	-0.166914	0.649545	1.785004	1.478026	1.279465	1.393785	1.322149	1.230182	1.738143	0.879431	0.606649
Cmtm6	0.461253	1.227302	1.309564	0.210676	0.375288	-5.527876	0.995438	0.812964	1.825611	0.485009	1.113405	0.846031	1.258992	1.71825	1.255413	1.253084	1.605665	1.760874	1.436856	1.501815
Fam129c	0.660331	2.329733	1.309387	0.210734	0.375336	-5.915963	2.230432	4.117033	3.545931	2.389316	2.226624	2.04074	2.467906	1.908813	2.759824	1.828755	1.102128	1.515634	2.133689	2.697558
Sqrdl	0.341511	2.512993	1.308885	0.2109	0.375575	-5.989374	3.303772	3.117121	2.901201	2.169587	3.174343	3.006419	2.033384	2.24812	1.663747	2.291729	2.442128	2.461578	2.236876	2.417223
Hdgfrp3	-0.303481	5.685762	-1.307614	0.21132	0.376267	-6.392764	5.88127	5.397089	6.177711	6.516619	5.905901	6.099927	5.471805	5.456551	5.452264	5.128996	5.634277	5.359557	5.862523	5.287439
Lrrc18	-1.631601	-2.162497	-1.307276	0.211431	0.37641	-4.991074	1.108109	-3.260591	-1.705055	-2.731185	-2.049163	-2.070862	-2.558588	-1.435005	-1.813089	-0.961779	0.584127	-1.660823	-1.413173	-2.228037
Acsm3	-0.68671	0.953279	-1.30697	0.211533	0.376535	-5.501022	2.035255	0.185409	0.398943	-0.02435	-0.038319	0.571087	1.372662	1.393205	0.787836	1.21727	2.228732	1.691577	1.54805	0.887553
Acadsb	-0.33606	4.77004	-1.306193	0.21179	0.376937	-6.294674	4.60537	5.097537	4.275724	4.768627	3.761059	4.320284	5.388667	4.952752	5.28894	4.868265	4.534071	4.712757	5.033414	5.234069
mrpl9	0.169927	5.178051	1.30579	0.211924	0.377119	-6.356638	5.325689	5.55755	5.307656	5.130514	5.50775	5.218052	5.100459	4.968131	5.079327	5.148143	5.136409	5.025932	5.057107	4.973505
Acot2	-0.268503	2.753404	-1.305475	0.212028	0.377239	-5.981227	3.109925	2.324846	2.673036	2.62393	2.892584	2.446602	2.629097	2.951874	2.905753	3.080873	2.771719	2.89053	2.713149	2.768575
Zbtb6	-0.450396	3.7583	-1.305397	0.212054	0.377239	-6.303225	4.276172	3.519177	4.700841	5.336137	4.113552	5.08423	3.049479	3.466799	3.307029	2.956054	3.181236	3.195935	3.536146	3.022122
R3hdm4	-0.215295	8.598321	-1.305278	0.212093	0.377253	-6.380909	8.503262	7.997846	8.19759	8.239684	8.321169	8.113679	9.145314	8.685912	9.125053	8.897946	8.582414	8.714842	8.646144	9.209859
Scrib	0.293838	7.231009	1.305024	0.212177	0.377326	-6.405202	7.081459	7.684636	7.160844	6.766319	7.239488	6.782324	7.414455	7.35801	7.361778	7.435839	6.86689	7.467612	7.092637	7.532533
Mrpl53	0.266024	5.160003	1.304966	0.212197	0.377326	-6.328443	5.06457	5.307749	4.949745	4.591724	5.318889	4.788749	5.526049	5.149037	5.444323	5.418377	5.296745	5.028276	4.997129	5.403549
C1qtnf9	0.483068	2.93822	1.3047	0.212285	0.377383	-6.076086	3.180343	4.390036	3.623781	2.977436	3.540516	2.72461	1.668148	3.104407	2.347926	3.417108	2.519368	3.30822	2.097585	2.460763
Slfnl1	0.406047	2.360674	1.304679	0.212292	0.377383	-5.719023	1.787089	2.354284	1.65069	1.245516	1.685287	1.960428	2.991343	2.709903	3.130231	2.934159	2.415434	2.8487	2.575555	3.097617
Rsrc2	-0.203003	6.664387	-1.304559	0.212332	0.377398	-6.406441	6.900997	6.618703	7.019032	7.346111	6.917809	7.126236	6.485984	6.484013	6.506909	6.398741	6.459854	6.351184	6.435367	6.266212
Fndc3b	-0.397959	4.249918	-1.304437	0.212372	0.377414	-6.272976	4.214585	3.752647	4.666764	4.814411	3.696327	4.342635	4.123906	4.58094	4.981207	4.168882	3.925804	4.12775	4.165547	4.022545
Otos	-0.58939	0.607883	-1.304225	0.212442	0.377421	-5.434302	1.033981	0.632508	0.049949	0.22441	-0.257548	-0.1418	0.563361	0.818632	1.426399	0.534523	1.430105	1.219993	0.546019	1.529341
Usf1	-0.146245	6.632631	-1.304169	0.212461	0.377421	-6.405424	6.704431	6.554366	6.468891	6.598788	6.607068	6.391674	6.814928	6.636169	6.781219	6.660311	6.721923	6.599165	6.621679	6.712126
Zfp423	-0.244757	5.303111	-1.304141	0.21247	0.377421	-6.362633	5.392103	5.080024	5.527397	5.442786	5.280338	4.798021	5.60958	5.22781	5.295576	5.414429	5.088633	5.414351	5.174405	5.538361
Fam133b	-0.234336	5.03814	-1.303986	0.212522	0.377457	-6.370974	5.344979	4.861671	5.403198	5.73487	5.354824	5.60107	5.018416	4.761243	4.846099	4.687033	4.81759	4.622553	4.833902	4.695317
Hsh2d	0.543093	0.938482	1.302895	0.212884	0.378045	-5.36568	0.105685	0.533043	0.785152	-0.166914	0.371962	0.175273	0.952703	2.522625	0.385075	2.438927	2.197736	3.009199	0.375586	0.442608
Uqcrh	-0.205821	7.191199	-1.302548	0.213	0.378078	-6.395656	7.901345	7.632277	7.393252	7.942072	8.007694	7.82849	6.855416	6.523062	6.875031	6.81508	6.783888	6.498783	6.818892	6.812867
Nyap1	-0.178188	4.934276	-1.302511	0.213012	0.378078	-6.329425	4.899562	4.782675	4.659851	4.973688	4.740757	4.8591	5.239313	4.729108	5.094627	5.159982	5.023839	4.911231	4.992221	5.065689
Ythdc1	-0.20343	6.698326	-1.302468	0.213026	0.378078	-6.409245	6.743476	6.690426	6.837443	7.39054	6.901335	7.024245	6.685246	6.481872	6.544537	6.514849	6.511181	6.42153	6.502484	6.542662
Slc35a1	0.185821	4.644455	1.302462	0.213028	0.378078	-6.268907	4.403064	4.393585	4.391531	4.140492	4.659497	4.367762	4.736391	4.850248	4.878873	4.871149	4.776227	4.916302	4.722744	4.978507
Eif3j	0.287133	4.872819	1.302025	0.213174	0.37828	-6.367097	5.024455	5.326436	5.453368	5.540658	5.370867	6.13785	4.567614	4.239446	4.397255	4.182876	4.272956	4.506292	4.852977	4.403387
Taf12	0.272041	4.103243	1.301596	0.213317	0.378477	-6.290958	4.141452	4.466163	4.443444	4.690659	4.895159	4.974008	3.569982	3.991112	3.729193	3.478908	3.944596	3.668268	3.989574	3.558103
Lasp1	0.190681	7.890569	1.301311	0.213412	0.378585	-6.392655	8.15217	8.124178	8.020252	7.795674	8.200682	8.316029	7.647819	7.629621	7.578609	7.88673	7.742508	8.063334	7.714398	7.60264
Wbp5	-0.389642	5.809996	-1.30109	0.213485	0.378585	-6.410175	6.605531	5.674484	6.4794	7.106613	6.518389	7.19146	5.268979	5.143629	5.460161	5.024078	5.470506	4.961159	5.483308	4.983491
Eri1	0.244561	4.769581	1.301068	0.213493	0.378585	-6.354379	4.935636	4.907073	5.336845	5.252825	5.362868	5.745858	4.339638	4.464319	4.397255	4.2553	4.530966	4.468815	4.553161	4.283226
Ccdc58	0.233801	2.715543	1.301036	0.213503	0.378585	-6.049766	2.997401	2.97275	3.12518	3.002635	3.384903	3.432728	2.518182	2.419003	2.347926	2.484835	2.604547	2.268872	2.508394	2.194616
Zfp637	-0.162951	5.611344	-1.300647	0.213633	0.378759	-6.391063	5.8546	5.455981	5.73788	5.784144	5.700344	5.726563	5.54436	5.473892	5.636038	5.592723	5.703489	5.433932	5.460392	5.48664
Sgta	-0.16987	7.097854	-1.300525	0.213674	0.378775	-6.409858	7.317999	6.83708	7.130744	7.26556	7.281679	7.239895	6.958392	7.012775	7.084949	7.033533	7.151003	7.015981	6.972563	7.079291
Igf2bp2	0.511079	0.04514	1.300233	0.213771	0.378892	-5.471364	0.307952	1.632013	1.033553	0.531921	0.952152	0.117189	-0.399109	-0.380636	-0.386063	0.055728	-0.411343	-0.334482	-0.211665	-0.297732
Emd	0.265854	4.673299	1.299319	0.214076	0.379377	-6.344687	4.948303	4.622535	5.253581	4.926188	5.374054	5.541588	4.218166	4.438054	4.469458	4.290193	4.600749	4.319865	4.357143	4.129836
Adpgk	0.281604	6.164605	1.299178	0.214124	0.379405	-6.39315	5.831537	6.351267	5.692309	5.589986	6.19885	5.726563	6.023363	6.64672	5.982939	6.614583	6.222567	6.944557	6.158147	6.343842
Taf5	-0.245516	3.233882	-1.299074	0.214158	0.37941	-6.085048	3.016775	3.037697	2.916749	3.589663	3.196117	3.150929	3.577905	3.070611	3.31576	3.327565	3.265855	3.145002	3.254	3.578011
Ube2d4	-0.653673	-0.083245	-1.298688	0.214287	0.379541	-5.539913	0.369528	-0.186163	0.484899	1.780306	-0.038319	1.648138	-0.169545	0.137473	-0.175642	-0.420466	-0.731011	-0.434228	-0.405292	-1.212031
Nfic	-0.218479	4.911635	-1.298664	0.214295	0.379541	-6.375426	5.569851	5.194311	5.545013	5.430459	5.028566	5.348419	4.421169	4.761243	4.227195	4.49849	4.64163	4.928903	4.749166	4.472374
Ddx20	0.223779	4.847392	1.298431	0.214373	0.379623	-6.319874	4.598957	5.273489	4.753743	4.721342	4.892938	4.636834	5.100459	4.853564	5.032426	4.862481	4.837836	4.797651	4.668401	4.888375
Mroh1	-0.292838	3.577441	-1.298315	0.214412	0.379635	-6.149639	3.633671	3.939046	3.387569	3.649386	3.167012	2.957603	4.028845	3.763261	3.866259	3.310669	3.592118	3.509549	3.777752	3.636136
Abcd4	0.447381	4.207558	1.298211	0.214447	0.379641	-6.155024	3.509242	4.661326	3.93756	3.394049	3.691225	3.334188	4.757474	4.757708	4.523219	4.879766	4.099353	4.523011	4.08486	4.845219
Rgs5	-0.416041	4.895718	-1.298101	0.214483	0.379651	-6.319733	5.360227	4.670131	4.169406	4.374055	4.633136	4.317063	5.397647	5.102412	5.694114	5.148143	4.152854	5.275228	4.984828	5.317354
Clock	-0.521492	3.74255	-1.297864	0.214563	0.379735	-6.24363	3.683012	3.452688	4.448801	4.888381	2.865664	4.310598	3.537848	3.838823	3.709451	2.90068	3.568082	3.529544	4.156802	3.629792
Vps36	0.193025	4.253879	1.297124	0.21481	0.380117	-6.255445	4.069061	4.180039	4.133261	4.170451	4.617084	4.326706	4.207992	4.384043	4.272747	4.382013	4.280371	4.261285	4.208498	4.14326
Mdm4	-0.412987	5.385725	-1.296976	0.21486	0.380149	-6.375116	4.955851	5.541644	5.048326	6.103344	4.352047	5.570283	5.822946	5.524698	5.670483	5.266765	5.305858	5.408965	5.435298	5.432996
Epas1	0.304181	7.570265	1.296864	0.214898	0.38016	-6.401345	8.267354	8.321753	8.295433	7.32878	7.814179	7.986129	7.383711	6.884922	7.487847	7.10754	6.843927	7.460252	7.442615	7.367907
Cdyl	0.226552	3.138429	1.296522	0.215012	0.380307	-6.086167	3.197423	3.317238	3.580509	3.107017	3.224646	3.359461	2.854546	3.159031	2.984524	2.843096	3.235656	3.397965	2.944167	2.91141
Itih4	-0.665004	0.245627	-1.295287	0.215426	0.380983	-5.39401	0.243629	-0.366503	-1.223881	0.577356	0.691028	0.284862	0.903114	0.449407	1.520039	-0.027758	0.484915	-0.541385	0.879431	1.048408
Pik3cb	-0.276694	4.468767	-1.294763	0.215603	0.381188	-6.32157	4.506048	4.275453	4.913392	5.072196	4.209453	4.654809	4.134688	4.68139	4.329893	4.196735	4.407721	4.430338	4.543129	4.279179
Psmb1	0.163964	6.496561	1.294752	0.215606	0.381188	-6.41748	6.619063	6.674469	6.753887	6.617939	6.942709	6.758179	6.493334	6.189523	6.397039	6.198623	6.49055	6.128942	6.353539	6.351573
Polr3h	0.262606	4.353288	1.294638	0.215644	0.3812	-6.270609	4.546601	4.699101	4.410874	3.838533	4.525459	4.149692	4.583442	4.532109	4.254698	4.328465	4.421201	4.426789	4.20426	4.102607
Gab2	0.252698	4.585564	1.294196	0.215793	0.381385	-6.338332	4.96336	5.157212	5.379549	4.624561	4.705768	4.921945	4.380979	4.332691	4.31255	4.250879	3.916315	4.575244	4.318455	4.425531
Ilkap	0.233533	5.639984	1.294138	0.215813	0.381385	-6.384426	5.444906	6.013517	5.362618	5.394419	5.755155	5.489598	5.952001	5.595724	5.819123	5.741966	5.529264	5.602291	5.43891	5.852167
Arfgap2	0.259119	6.615077	1.293122	0.216154	0.381933	-6.414866	6.50021	6.839034	6.693111	5.978717	6.399083	6.059053	6.866779	6.696503	6.833074	6.885228	6.700662	6.74382	6.548344	6.883892
Jkamp	-0.254885	5.86254	-1.293007	0.216193	0.381945	-6.40791	5.824683	5.324578	5.897242	6.310085	5.998646	6.028851	5.584238	5.803739	5.938405	5.824622	5.768213	5.805913	5.923087	6.070483
Metrn	-0.350823	5.553878	-1.292651	0.216313	0.382101	-6.375148	6.012522	5.405902	4.796393	4.849557	5.362868	4.741469	5.836219	5.670955	5.641258	6.07526	5.812363	6.015372	5.648382	5.921329

Itih3	0.491139	8.822683	1.292412	0.216393	0.382187	-6.381492	9.077602	10.19185	9.034622	8.142894	8.766726	8.408794	9.1566	8.209162	8.961165	8.792944	8.300018	8.282172	8.810767	9.385931
Pdcd6ip	0.170158	7.520135	1.292013	0.216528	0.382368	-6.411597	7.47332	7.673367	7.823185	7.847012	7.879555	7.942894	7.261751	7.283135	7.205538	7.32084	7.377932	7.483502	7.340601	7.377878
Spata1	0.3999	0.942682	1.291559	0.216681	0.382582	-5.517951	0.785575	1.369077	0.750864	0.43652	0.649545	1.221719	1.297885	1.067371	1.255413	1.104165	1.068256	1.11804	0.921344	0.970221
Snx17	0.258137	5.727222	1.291435	0.216723	0.3826	-6.388321	5.752845	5.616442	5.696819	5.097757	6.071135	5.32776	5.61921	5.812285	5.838944	5.970246	6.057577	5.796318	5.806312	5.747651
Adam32	1.19577	0.427873	1.290504	0.217037	0.383098	-5.302973	0.955837	-0.950476	0.642822	-1.788967	1.72577	0.61372	1.607516	0.517885	-0.080923	1.14286	0.535373	0.930264	0.111637	1.556352
Car5b	-0.360395	1.689912	-1.290317	0.2171	0.383153	-5.799338	2.402324	1.894965	1.996308	1.63746	1.364797	1.327961	1.913281	1.80302	1.743567	1.51148	1.82436	1.643459	1.377871	1.708589
Ilvbl	0.313717	4.848141	1.290119	0.217167	0.383215	-6.311052	4.875886	5.159298	4.760222	4.04669	4.708296	4.437656	4.952909	4.980316	5.050846	5.286341	4.797057	5.085673	4.627841	5.161159
Smurf2	-0.15273	5.871183	-1.289888	0.217245	0.383297	-6.413421	5.97587	5.85908	5.957679	6.205449	5.902594	6.034746	5.785805	5.680322	5.710759	5.84823	5.692413	5.88435	5.849014	5.837102
Tekt2	-0.799819	0.999277	-1.289111	0.217508	0.383704	-5.54226	1.957182	1.047298	0.975347	0.280432	0.027932	-0.750296	1.726334	1.356279	0.787836	0.591587	2.181985	1.315214	1.815874	1.608903
Slc39a5	0.298531	4.135365	1.288936	0.217567	0.383752	-6.209057	4.017097	4.393585	3.929918	3.443962	4.162299	3.606391	4.297077	4.264575	4.523219	4.324262	4.276668	4.208518	3.994491	4.544972
Fads2	-0.288026	6.837282	-1.28847	0.217725	0.383974	-6.426098	7.660866	6.871845	6.829251	6.69081	7.021042	6.833386	6.478597	6.540656	6.673257	6.570909	6.690261	6.886309	7.107463	6.881224
Gpr37l1	-0.325766	7.872954	-1.287651	0.218002	0.384407	-6.418943	8.137034	7.635652	6.888112	7.32961	7.869145	7.24032	7.976638	7.974996	8.01539	8.327735	8.074129	8.37304	8.104361	8.282294
Scand1	0.309666	5.411403	1.287361	0.2181	0.384524	-6.349137	5.401346	5.206468	5.17333	4.214255	5.07631	4.959619	5.806182	5.601653	5.844988	5.974275	5.786556	5.653298	5.505866	5.59482
Bmpr1b	-0.379839	2.416773	-1.28652	0.218386	0.384968	-6.001398	3.188908	2.822876	2.726949	2.591079	2.139408	2.323579	1.962529	2.650138	1.914249	1.875459	2.066633	2.816513	2.701169	2.357047
Inca1	-0.499399	0.840746	-1.28641	0.218423	0.384968	-5.618769	1.278303	0.04804	1.493674	1.327578	0.770579	0.881356	0.498056	1.564863	0.787836	0.893396	0.805871	0.34469	1.316372	0.65743
B4galt7	0.300763	4.768923	1.286334	0.218449	0.384968	-6.272739	4.403064	5.014693	4.32899	3.838533	4.316041	4.017012	5.089416	5.042656	5.056067	5.185692	4.939699	5.099115	5.172237	5.323264
Ankrd10	0.330607	5.833529	1.285637	0.218685	0.385326	-6.38099	5.1936	6.237388	5.400436	5.160679	5.359656	5.049711	6.437822	5.970313	6.340403	6.381653	6.021524	5.996342	5.925664	6.223667
Arpc5l	0.26399	3.616902	1.285547	0.218716	0.385326	-6.121141	3.303772	3.551306	3.670387	3.1898	3.628553	3.172479	3.81758	3.526187	3.83015	4.076955	4.008504	3.575147	3.6527	3.763295
Ctdspl2	-0.320466	3.439245	-1.285257	0.218815	0.385441	-6.207008	3.633671	3.08236	4.026238	4.136703	3.217567	3.914167	3.328389	3.368235	3.038267	3.195748	3.441501	3.25317	3.508915	3.151754
Sharpin	0.214937	6.207965	1.285098	0.218869	0.385441	-6.425712	6.507077	6.683195	6.448979	6.111088	6.807204	6.154189	5.958085	6.116873	6.082225	6.096361	6.142429	6.02714	5.86521	5.932969
Mmp3	2.242385	-2.583719	1.285072	0.218877	0.385441	-4.869326	-1.642648	-3.260591	1.386086	-3.260591	-1.235601	-0.984601	-2.558588	-3.260591	-1.536754	-1.136114	-2.697527	-2.249472	-1.413173	-1.632384
Ppp1r12c	0.235701	7.737227	1.284823	0.218962	0.385534	-6.426826	7.431976	7.840003	7.528114	7.162186	7.503084	7.258487	8.15736	7.855217	8.109404	8.098714	7.722912	7.831672	7.6473	8.182341
Sdhd	-0.200951	5.981867	-1.284514	0.219067	0.385662	-6.419039	6.278382	5.798796	5.755281	5.866522	6.148554	5.780186	6.114695	5.923749	6.065498	5.868575	6.234069	5.871352	6.078191	5.987326
C2cd4c	-0.453836	2.555599	-1.283323	0.219473	0.386291	-6.001424	2.810225	1.480065	3.283475	3.002635	2.16907	2.781814	2.078759	2.522625	2.822431	2.641672	2.639567	2.9008	2.535634	2.387449
Piwil2	-0.377673	1.831601	-1.283277	0.219488	0.386291	-5.763957	1.541696	1.425638	1.841991	2.124384	1.173177	1.450626	2.343298	2.287882	1.819202	1.652245	1.903329	2.046399	2.494578	1.991897
Zfp3	-0.301697	2.932297	-1.283055	0.219564	0.386354	-6.073325	3.136736	2.279532	3.056656	3.225913	3.091571	3.053636	2.433388	3.221962	2.905753	3.00938	3.196995	2.772447	2.923692	2.952659
Lrp4	-0.267525	7.234631	-1.282983	0.219589	0.386354	-6.432303	7.357001	7.274903	6.998173	7.122026	6.693165	6.923521	7.131214	7.309937	6.96866	7.541439	7.23879	7.974656	7.315772	7.446263
Zfp259	0.196878	6.440344	1.282712	0.219681	0.386365	-6.431128	6.356494	6.357641	6.757669	6.644865	6.862148	6.812949	6.407022	6.266173	6.35106	6.345821	6.264302	6.199281	6.11816	6.439438
Tgfbr2	0.419714	4.043879	1.28259	0.219723	0.386365	-6.244203	4.614937	3.958345	4.352047	3.137672	4.348811	4.380163	3.677127	4.24955	3.735714	4.061889	4.128407	4.9879	3.627601	3.454224
Bhlhe40	0.209024	6.164867	1.282534	0.219742	0.386365	-6.42969	6.345043	6.469478	6.970504	6.387058	6.358626	6.500046	5.836219	6.096026	5.953869	5.998214	6.048919	6.0958	5.766625	5.504087
C4bpb	0.621852	0.817402	1.282532	0.219742	0.386365	-5.359916	-0.046857	0.725555	0.679743	-0.325128	0.027932	0.117189	2.033384	1.155984	1.663747	1.898256	1.135223	0.714338	1.11438	1.556352
Dnajc3	0.264637	5.134159	1.282492	0.219756	0.386365	-6.409331	5.444906	5.477878	5.806258	5.779292	5.825528	6.39931	4.306644	4.743479	4.504248	4.683756	4.621334	4.936411	4.677602	4.719417
Pten	-0.359416	5.602173	-1.282202	0.219855	0.386483	-6.428337	6.036449	5.720423	6.243923	6.831056	5.807959	6.527482	5.216656	5.162468	4.91667	4.630291	5.400772	5.206513	5.703469	5.060981
Cygb	-0.225219	5.323027	-1.282107	0.219887	0.386484	-6.398464	5.638683	5.127683	5.481086	5.453487	5.611315	5.053587	5.130397	5.143629	5.299983	5.190317	5.439377	5.03994	5.543256	5.409117
Asf1a	-0.194134	4.153171	-1.281932	0.219947	0.386532	-6.330096	4.529842	4.346754	4.501309	4.85187	4.424547	4.724549	3.699086	3.734773	3.930199	3.689602	3.995046	3.838214	4.056929	3.911867
Supt6h	-0.293638	8.614557	-1.281511	0.220091	0.386728	-6.392073	8.635645	8.140374	8.826329	9.294994	8.650128	9.068975	8.450341	8.677049	8.478708	8.351161	8.599931	8.435526	8.671117	8.327584
LOC690911	-0.209251	6.938129	-1.281398	0.220129	0.386739	-6.434816	6.860689	6.991426	7.038262	7.457758	6.722218	7.046785	6.731262	6.954517	6.76935	6.900834	6.809743	7.04521	7.034744	6.783876
Ppil4	-0.262542	6.122672	-1.281259	0.220177	0.386766	-6.429614	6.096864	5.709075	6.268369	6.710698	6.08092	6.507838	5.944361	6.09182	6.196547	5.964857	6.176611	5.955049	6.152659	5.8845
Unc13c	-0.525611	3.515337	-1.281153	0.220213	0.386773	-6.212044	4.499177	2.934281	3.967729	3.456176	3.011968	3.756629	3.844102	2.753156	2.95129	3.14899	2.912	3.916225	4.463807	3.745801
Mrps18c	0.234675	3.701292	1.280687	0.220372	0.386996	-6.245257	3.963192	4.10842	3.989949	3.847833	4.380852	4.075375	3.383673	3.321106	3.555781	3.702612	3.461211	2.9008	3.608485	3.642453
Eif4a3	0.170916	6.301281	1.28041	0.220467	0.387106	-6.43027	6.342166	6.175859	6.599873	6.152957	6.52629	6.367742	6.162897	6.400328	6.198908	6.362312	6.353134	6.191982	6.126023	6.277416
Hunk	-0.574744	3.481003	-1.280313	0.2205	0.387108	-6.112125	3.495497	1.558038	2.829082	3.425447	3.083806	3.988947	3.727855	4.455617	3.627666	3.440594	4.250477	3.650119	3.887547	3.475607
Twist1	-0.68452	1.332506	-1.280094	0.220575	0.387182	-5.729642	1.764345	-0.687936	1.408255	1.856	1.48759	2.271274	1.047044	0.923327	1.819202	1.481601	1.260496	1.691577	1.436856	1.608903
Tdp2	0.28704	5.451242	1.279905	0.22064	0.387239	-6.387891	5.130527	6.10186	5.382351	5.242285	5.192798	5.23522	5.868875	5.297643	5.588194	5.650933	5.362854	5.355829	5.340009	5.604558
Ces5a	-0.725412	0.684419	-1.279753	0.220692	0.387274	-5.423819	0.830061	0.973336	0.20995	0.166125	-1.400091	-0.371994	1.335758	0.818632	1.458292	1.804823	1.068256	1.045835	1.040235	1.824228
Tob2	-0.227329	4.555605	-1.279531	0.220768	0.387303	-6.349702	4.927981	4.777251	4.802683	4.809659	4.457973	4.526803	4.091065	4.612599	4.081214	4.433645	4.661644	4.715669	4.752072	4.195741
Rad17	0.182017	4.490918	1.279467	0.22079	0.387303	-6.339053	4.439412	4.591969	4.907568	4.823867	4.854661	4.907024	4.438678	4.219024	4.321248	4.311581	4.269233	4.331301	4.208498	4.319155
Rps10	1.484433	-0.32644	1.279392	0.220815	0.387303	-5.21098	-0.518419	-0.813242	-0.263851	-1.575022	3.174343	-0.984601	0.118118	-0.81357	0.007959	0.591587	-0.510232	-0.24119	-0.512812	0.045082
Slc25a3	0.155509	8.066502	1.279328	0.220837	0.387303	-6.422128	7.948136	8.012634	7.98381	7.826284	8.208068	7.966987	8.237386	8.026953	8.198551	8.066997	8.255326	7.98193	8.106066	8.117791
Hbp1	-0.198848	5.479085	-1.279054	0.220931	0.387411	-6.412137	5.644922	5.277336	5.43459	5.782933	5.532208	5.816	5.469678	5.400976	5.410079	5.439902	5.336417	5.513054	5.125938	5.558541
Scmh1	-0.200388	5.655458	-1.278921	0.220977	0.387435	-6.406136	5.7427	5.734484	5.442668	5.453487	5.26325	5.152692	5.928957	5.812285	5.820657	5.894318	5.697961	5.772746	5.686859	5.805084
Txndc5	0.22824	6.284042	1.278524	0.221113	0.387617	-6.426178	6.118322	5.822686	6.240066	5.769538	6.41387	6.202998	6.043529	6.518892	6.323186	6.455563	6.421802	6.699648	6.422655	6.544349
Igf2	-1.518358	6.337236	-1.278194	0.221226	0.387758	-6.43245	9.745969	4.977341	5.82595	5.0052	5.004088	7.643153	5.450387	5.76904	5.481658	7.035462	9.559349	5.757285	4.898279	6.596518
RGD1308706	-0.284245	4.291016	-1.278049	0.221275	0.387789	-6.341797	4.566458	3.958345	4.59368	5.013489	4.643738	4.823218	4.197745	4.139756	4.01306	3.978769	4.250477	3.895831	4.341792	3.739923
Ccdc116	0.701488	0.525262	1.277385	0.221503	0.388047	-5.565231	1.245833	2.151237	1.890044	0.105385	0.88224	0.386712	0.743216	0.517885	0.245823	-0.961779	0.138317	0.151783	0.182287	0.887553
Flnb	0.380858	5.588569	1.277346	0.221517	0.388047	-6.391267	5.276307	6.247229	5.857511	5.058242	4.960226	5.129006	6.008784	5.925326	6.065498	5.478234	5.424393	5.463681	5.846296	5.533271
Mrgprx3	-0.745139	0.548285	-1.277337	0.22152	0.388047	-5.458254	0.873216	-1.271663	-0.575682	0.43652	1.173177	0.527154	-0.831021	2.16515	0.007959	1.875459	1.674823	2.300247	-0.040977	0.753927
Lrrc56	-0.291208	3.202444	-1.276677	0.221746	0.388319	-6.115889	3.707065	2.934281	2.837276	2.985885	3.488528	2.915623	3.392685	3.262441	3.159628	3.090803	3.189137	3.277021	3.195422	3.571405
Tfb2m	0.212778	4.29908	1.276633	0.221762	0.388319	-6.295869	4.132599	4.544872	4.166157	4.318277	4.693063	4.373976	4.595201	4.073048	4.170562	4.154751	4.269233	4.060629	4.334055	4.380898
Nenf	0.33354	4.941346	1.276551	0.22179	0.388319	-6.344243	5.066895	5.246268	4.928811	4.155549	5.152532	4.413727	4.835551	4.83019	5.230115	5.333104	4.92558	4.9879	4.942203	5.182988
Thg1l	0.271924	3.15089	1.276508	0.221804	0.388319	-6.176022	3.22267	3.466233	3.776458	3.711838	3.736505	3.99299	2.703406	3.180315	2.858731	2.514649	2.940364	2.568523	3.079891	2.544088
Fam20a	-0.370725	2.158889	-1.276326	0.221867	0.388372	-5.858352	2.091157	2.383133	1.386086	2.269859	2.124346	1.496914	2.206902	2.054242	2.381582	2.529327	2.149958	2.643831	2.285827	2.584013
Camk2n2	-0.333365	4.312926	-1.276049	0.221962	0.388482	-6.296197	4.757075	4.37216	4.519244	4.04669	3.799498	3.547133	4.531353	4.117861	4.600281	4.336834	4.338363	4.060629	4.942203	4.493492
Cldn19	-0.710712	1.628585	-1.275896	0.222015	0.388518	-5.778638	3.197423	1.725091	0.52603	0.823477	1.364797	1.473956	1.335758	2.099634	0.787836	1.852299	3.065821	2.516041	1.252133	1.355706
Ring1	0.172147	5.711479	1.275764	0.22206	0.388541	-6.423822	5.897038	6.108354	5.88148	5.661611	5.920144	5.89266	5.450387	5.505855	5.568238	5.837949	5.53857	5.642628	5.413437	5.672487
Timm10b	0.317686	2.7901	1.275657	0.222097	0.388548	-6.015796	3.05476	2.843782	3.049621	2.297273	3.245679	2.734303	3.01488	2.58779	2.611707	2.668442	3.132898	2.489067	2.713149	2.847239
Agrn	0.34966	8.441786	1.275334	0.222208	0.388686	-6.428399	7.946558	8.779913	8.126033	7.562441	7.710201	7.79733	9.166321	8.606819	9.10819	8.896178	8.298193	8.555564	8.476193	9.159964
Sep15	0.262972	7.727816	1.27513	0.222278	0.38873	-6.428057	7.867063	7.538817	8.05316	7.757632	8.502851	8.212126	7.298774	7.350988	7.496513	7.574546	7.688139	7.633069	7.683524	7.539741
Slc25a44	-0.163469	6.475738	-1.275073	0.222298	0.38873	-6.440281	6.611915	6.196323	6.462946	6.546913	6.523422	6.480022	6.292526	6.476505	6.420502	6.479382	6.503281	6.537146	6.650779	6.496338
Arid5b	-0.312694	4.883355	-1.274418	0.222524	0.389068	-6.40529	5.302196	4.874426	5.776741	5.769538	4.715852	5.517933	4.425566	4.866752	4.54566	4.528025	4.689763	4.659314	4.512607	4.238063
Hs6st3	-0.549787	4.331466	-1.273958	0.222682	0.389288	-6.304674	4.078311	4.091039	4.421813	4.973688	3.068148	4.361521	4.171806	4.934079	4.299404	3.772142	4.789281	4.412503	5.328373	4.022545
Azin1	-0.376276	6.624429	-1.273742	0.222757	0.389362	-6.443077	6.913971	6.309609	7.044468	7.712527	6.895247	7.495301	6.33522	6.194762	6.256728	5.858438	6.546977	6.237264	6.795841	6.162354
Fgd1	-0.25068	3.291856	-1.273648	0.222789	0.389362	-6.178513	3.595524	3.055728	3.73759	3.659984	3.137307	3.541623	2.814871	3.292072	3.169295	3.14899	3.235656	3.261164	3.294432	3.302949
Prcp	0.31822	4.363027	1.273554	0.222821	0.389362	-6.277953	3.963192	3.801553	4.284739	4.125276	4.348811	4.994318	4.258161	4.729108	3.947156	4.663938	4.612548	4.978233	4.134703	4.319155
Ankrd9	0.269026	3.011816	1.272054	0.223339	0.39021	-6.051718	3.136736	3.08236	3.228468	2.534609	3.231692	2.800388	2.955303	2.753156	3.110295	3.050666	3.165302	2.9008	3.286436	3.12494
Capsl	-1.003078	0.663208	-1.271488	0.223534	0.390495	-5.563192	2.569081	-0.950476	0.25957	-0.093872	1.664612	-0.214525	0.743216	1.934092	0.007959	0.055728	1.403186	1.152831	1.574559	0.383504
Txlng	-0.357967	2.720088	-1.271354	0.223581	0.390519	-6.05616	2.41698	2.452828	2.861581	3.665254	2.542105	2.974059	2.880403	2.603631	2.962454	2.274406	2.771719	2.447635	2.736815	2.733504
Usp33	-0.251256	7.419379	-1.271063	0.223682	0.390638	-6.444463	7.305745	6.833818	7.193922	7.545036	7.184725	7.484573	7.910729	7.570397	7.791921	7.297448	7.507594	7.377636	7.204541	7.672588
Hapln2	0.684312	6.864474	1.270819	0.223766	0.390729	-6.439291	6.319917	8.215984	6.651318	5.722283	6.686771	5.263945	7.297575	6.75264	7.404866	7.35317	6.508816	7.268845	6.951858	7.720392
Angpt4	0.724789	0.320814	1.270723	0.223799	0.39073	-5.328261	-0.129637	1.010791	-0.007587	-0.705589	0.320024	-0.291112	0.498056	0.137473	-0.080923	1.322149	1.605665	1.404538	0.111637	0.65743
Ranbp2	-0.256924	7.594271	-1.270457	0.223891	0.390766	-6.441497	7.19211	7.227469	7.436029	8.079668	7.168296	7.6781	7.875806	7.700389	7.793875	7.433402	7.639591	7.570513	7.824797	7.708024
RT1-CE6	0.82093	0.596943	1.270444	0.223896	0.390766	-5.454034	0.592497	0.04804	0.442561	-0.325128	2.226624	0.809818	-0.279766	0.517885	0.007959	1.451089	1.959843	0.889587	0.649393	0.499386
Rnf125	0.978132	-0.575797	1.270382	0.223917	0.390766	-5.363611	-0.899176	0.812964	1.809043	0.280432	-0.516166	0.336685	-2.558588	-1.435005	-0.773548	-0.806258	-0.855515	-0.541385	0.037347	-0.116166
Mad2l1	0.521843	2.178721	1.269802	0.224118	0.391008	-5.946914	1.874641	1.812527	2.548818	2.666599	3.280067	3.524966	1.754571	2.227821	1.60797	1.920699	2.360517	2.027776	2.003164	1.260365
Mpv17l2	0.289967	4.647867	1.269793	0.224121	0.391008	-6.284039	4.319721	4.649501	4.551972	3.805502	4.427618	3.751866	4.871449	4.876565	4.930947	5.159982	4.967529	5.044579	4.763639	5.015475
Pkn1	0.27725	6.797844	1.269604	0.224187	0.391066	-6.445185	6.37819	7.151427	6.489833	6.284629	6.500272	6.294288	7.256824	6.913005	7.126482	7.2079	6.752239	6.959373	6.679303	7.190692
RGD1311739	0.303856	4.994014	1.269122	0.224354	0.391266	-6.374582	5.352623	5.315253	5.123951	4.33821	5.327135	4.791072	5.097706	4.60868	5.24619	5.026667	4.96523	4.752996	4.842108	5.178649
Rpf1	0.240884	4.344449	1.269085	0.224366	0.391266	-6.354225	4.3275	4.522355	4.868125	4.952291	5.030587	5.117941	4.253222	4.067347	3.918782	3.924277	3.88747	4.074306	3.969738	3.988241
RGD1306820	-0.205693	5.543577	-1.268421	0.224597	0.39161	-6.401045	5.010026	5.248229	4.961037	5.634999	5.145088	5.141808	6.183813	5.486762	6.03674	5.623833	5.619735	5.631879	5.862523	6.058724
Cyld	-0.421048	4.14709	-1.268204	0.224672	0.391685	-6.272076	3.922683	2.894758	4.308508	4.476537	3.726565	4.14243	4.3855	4.35626	4.213244	3.968034	4.700858	4.187698	4.794977	4.074854
Rbm28	0.174285	5.22824	1.26805	0.224725	0.391721	-6.391498	4.955851	5.097537	5.438634	5.182896	5.118729	5.318124	5.317106	5.43679	5.308757	5.213226	5.27278	5.173007	5.229661	5.174296
F8a1	-0.2387	4.329277	-1.267651	0.224864	0.391906	-6.293692	4.406741	3.719097	4.092769	4.140492	4.445908	3.8532	4.447353	4.490115	4.618038	4.410044	4.695321	4.316033	4.58936	4.465266
Scara5	-0.777622	2.141941	-1.267468	0.224928	0.39196	-5.918156	3.516065	0.426209	1.758166	1.679737	2.762423	2.101918	2.534559	1.883083	2.066858	2.048482	3.555912	1.542121	2.133689	2.387449
Tfb1m	-0.195079	4.25179	-1.266899	0.225125	0.392202	-6.348689	4.464322	4.435504	4.561895	4.921791	4.619772	4.395516	4.068749	4.078726	3.895674	3.88487	4.082483	3.941318	4.294734	3.96323
Dgat1	0.273241	5.45662	1.266879	0.225132	0.392202	-6.366215	4.805223	5.397089	4.96665	4.651364	5.036634	4.610761	6.088591	5.644398	6.108843	6.079006	5.552419	5.714258	5.660807	6.115448
Slco1a2	-0.447442	5.928757	-1.266787	0.225164	0.392202	-6.438476	6.389381	6.284024	4.629506	5.805781	6.006883	5.702076	6.554839	5.469576	6.397039	5.938979	5.375027	5.922654	5.973761	6.580982
Ptbp2	-0.238889	6.428885	-1.26662	0.225223	0.392246	-6.450333	6.259192	6.369403	6.532718	6.918613	5.951419	6.550297	6.665863	6.37286	6.637139	6.317559	6.34166	6.216857	6.438076	6.451179
LOC690871	0.349424	3.841925	1.266322	0.225326	0.392367	-6.247045	4.260004	4.601207	3.702138	3.302317	4.258567	3.922669	3.933266	3.386664	3.958351	3.879152	3.882605	3.529544	3.627601	3.655003
Mif	-0.233683	6.242128	-1.266232	0.225357	0.392367	-6.448981	6.733264	6.156146	6.207251	6.184497	6.592092	5.942639	6.172085	6.15231	6.316677	6.095129	6.377541	5.95628	6.312785	6.212036
Tbx19	0.594449	1.499871	1.265695	0.225544	0.392614	-5.56216	1.143792	1.894965	0.818644	0.105385	1.230571	0.809818	1.782265	2.419003	1.769222	1.652245	2.197736	2.187314	1.252133	2.357047
Ttc27	0.209387	4.577192	1.265637	0.225565	0.392614	-6.311555	4.276172	4.661326	4.440757	4.214255	4.396609	4.323498	5.175477	4.624294	4.964647	4.574767	4.644506	4.555878	4.624673	4.670808
Rffl	-0.257944	4.645655	-1.265316	0.225676	0.392752	-6.341968	4.667979	4.861671	4.109779	4.600004	4.36492	4.20302	4.606864	4.873301	4.589521	4.637083	4.675772	5.046893	4.817348	5.049144
Vprbp	-0.233856	5.42632	-1.265186	0.225721	0.392774	-6.427572	5.362121	5.388222	5.609819	6.031704	5.170974	5.694648	5.352175	5.414511	5.487466	5.092395	5.231293	5.24934	5.568205	5.352453
Rhob	0.225578	8.451004	1.265071	0.225762	0.392787	-6.424186	8.332583	8.225225	8.773803	8.506788	8.884134	8.702015	8.250457	8.397856	8.318793	8.1666	8.585037	8.411933	8.545672	8.217668
Rab7l1	0.21672	5.337368	1.264905	0.22582	0.392831	-6.403239	5.448478	5.727471	5.250517	4.881605	5.33042	5.209391	5.317106	5.516354	5.2845	5.457277	5.530819	5.312249	5.210772	5.285422
Cul1	-0.147237	7.45731	-1.264596	0.225927	0.392961	-6.451066	7.334981	7.205428	7.448451	7.603855	7.315229	7.319322	7.580898	7.532107	7.507511	7.429984	7.531486	7.489468	7.604928	7.507665
Naa30	-0.365898	5.310856	-1.263604	0.226273	0.393506	-6.439046	5.483713	4.914502	5.704677	6.44259	5.601831	6.167666	5.050088	4.83019	5.112276	4.716194	5.293084	4.973375	5.154774	4.948235
Pramef8	-0.454871	2.062028	-1.263372	0.226354	0.39359	-5.855248	2.072763	0.812964	1.890044	2.227735	2.212449	1.839907	2.12275	2.555575	2.462438	2.06872	2.506778	2.284645	2.652228	1.556352
Fam135a	-0.249603	5.208395	-1.26306	0.226463	0.393685	-6.395661	4.843702	4.991462	5.050089	5.511695	4.617084	5.07663	5.726455	5.220124	5.559075	5.277674	5.047936	5.138705	5.223392	5.677109
Meis2	0.28756	5.590083	1.263028	0.226474	0.393685	-6.444359	6.324783	5.818732	6.258488	5.496992	6.402209	6.296743	5.241808	5.194202	5.310942	4.891176	5.603568	5.046893	5.296877	5.111939
Trub2	0.215844	4.822603	1.262746	0.226573	0.393799	-6.348648	4.633881	5.033013	4.839859	4.412169	4.625132	4.452412	5.000844	4.889545	4.922398	4.944172	5.13845	4.695162	5.047677	4.938001
Abhd8	-0.268971	7.703725	-1.261907	0.226866	0.394219	-6.451795	8.046853	7.666783	7.323151	7.237531	7.561566	6.946635	8.0209	7.63974	7.910142	7.945028	7.863471	7.736681	7.964466	7.996989
Aatk	-0.288826	8.53491	-1.261868	0.22688	0.394219	-6.43569	8.306008	8.638829	7.845697	8.372277	8.100574	7.607668	8.915896	8.749592	8.779009	8.897062	8.489893	9.005623	8.697928	9.087164
Dqx1	0.526937	3.593437	1.261456	0.227024	0.394412	-6.021399	2.917193	4.060109	2.663852	2.198954	3.106978	2.526873	4.48576	4.101219	4.222559	4.264103	3.872828	3.980582	3.608485	4.450943
Whsc1l1	-0.306127	6.118665	-1.26116	0.227128	0.394535	-6.45332	5.903558	6.058923	6.248538	6.850271	5.605903	6.37628	6.152323	6.180309	6.189438	5.782355	6.058655	6.018913	6.336214	5.922627
Yipf6	-0.357679	4.697746	-1.260344	0.227414	0.394974	-6.395641	4.960861	4.167656	5.032356	5.396005	4.651639	5.419233	4.36733	4.515458	4.610962	3.968034	4.609607	4.447955	5.066477	4.6173
Cntrob	0.402137	2.498863	1.260012	0.22753	0.395119	-5.972458	2.569081	3.432128	2.654608	2.154676	2.77212	2.203108	2.48486	2.186344	2.625819	2.694725	2.802949	2.00891	2.203293	2.474989
Pdlim5	0.293314	4.07319	1.259701	0.227639	0.395216	-6.372489	5.045831	4.54806	4.913392	4.357872	4.960226	5.509491	3.454237	3.232189	3.555781	3.335938	3.387587	3.35379	3.949625	3.524301
Cdk6	0.576581	0.737804	1.259666	0.227651	0.395216	-5.296742	0.830061	0.812964	2.108881	0.664146	1.364797	0.809818	0.625837	1.020936	1.102347	-0.665882	0.484915	0.714338	0.745852	0.706485
Dcaf8	-0.192114	6.913262	-1.259186	0.22782	0.395451	-6.461941	6.955977	7.143018	7.006371	7.308294	6.633906	6.976549	6.955354	6.952199	7.005553	6.78022	6.752239	6.658627	6.840898	6.82957
Ntpcr	-0.238313	4.231033	-1.258985	0.22789	0.395451	-6.260639	4.145858	3.833259	3.47943	3.819751	3.956574	3.713177	4.637512	4.459975	4.632088	4.560545	4.820137	4.31219	4.624673	4.327019
Il6st	0.198737	7.74254	1.258907	0.227917	0.395451	-6.452535	7.595929	7.64536	7.832693	7.648776	7.769797	8.037678	7.495115	7.936425	7.41861	7.723991	7.75354	8.255159	7.647691	7.642181
Fam101b	-0.580938	2.173349	-1.258904	0.227918	0.395451	-5.966241	2.753611	0.480615	3.013923	2.591079	2.014259	2.685174	2.398025	2.054242	1.937064	1.804823	2.259075	2.489067	2.11575	2.211707
Rspo3	-0.36759	3.536126	-1.258172	0.228175	0.395794	-6.197895	3.303772	3.595119	3.776458	4.026356	3.075998	2.809587	3.038038	3.852146	3.786848	3.656554	3.78171	3.502822	3.929227	3.510555
Dnajb12	-0.14936	6.271889	-1.258154	0.228182	0.395794	-6.457032	6.365967	6.095337	6.274416	6.284629	6.196152	6.136937	6.002911	6.362423	6.198908	6.38972	6.452488	6.550254	6.307856	6.208847
Acr	0.870747	1.119282	1.257738	0.228328	0.395974	-5.550927	0.592497	2.425352	2.010869	0.33436	0.15195	0.65513	1.372662	1.464337	1.663747	1.678822	0.885831	0.930264	0.313987	1.991897

Txnip	0.281552	6.542154	1.257671	0.228352	0.395974	-6.462591	6.930665	6.945018	7.096167	6.285485	6.660907	6.852426	5.730014	6.871081	5.740245	6.606818	6.399841	7.087973	6.167973	6.233114
Smg6	-0.175973	5.633185	-1.256961	0.228601	0.396302	-6.443363	5.622966	5.595024	5.892335	5.972354	5.593651	5.410191	5.57239	5.680322	5.551703	5.674236	5.68405	5.589649	5.497232	5.56021
LOC499770	0.264048	5.092161	1.256947	0.228606	0.396302	-6.375438	4.950824	5.370323	4.996223	4.455398	4.854661	4.634248	5.495004	5.028055	5.268848	5.457277	5.238925	5.170887	5.035801	5.381063
Stag2	-0.190448	6.250839	-1.256691	0.228696	0.3964	-6.463505	6.502789	6.149865	6.488533	6.832813	6.531294	6.740252	6.15762	5.926901	5.967785	5.967554	5.97658	6.064165	6.131613	6.094869
Rcor3	0.262385	3.031924	1.256595	0.22873	0.396402	-6.057265	2.682606	2.790938	3.376367	2.862845	2.883666	3.053636	3.127125	3.19084	3.341641	2.783118	3.243265	2.980434	3.254	3.069767
Cox8b	-1.977425	-2.03793	-1.256077	0.228913	0.396611	-5.082935	1.831529	-2.680539	-3.260591	-2.731185	-1.798946	-0.646023	-1.450335	-2.076466	-2.604521	-0.537962	1.430105	-1.243945	-2.656794	-1.406952
Sirt5	-0.191232	6.501482	-1.256064	0.228917	0.396611	-6.460833	6.530854	6.385533	6.002085	6.302495	6.416964	6.098989	6.796373	6.617761	6.806218	6.730273	6.754237	6.458502	6.606504	6.531645
Adora1	-0.28487	6.568716	-1.255521	0.229108	0.396885	-6.466651	6.859348	6.185104	6.855204	7.079237	6.748858	6.947677	6.083034	6.632313	6.20597	6.183622	6.414238	6.651042	6.809856	6.323349
Myadml2	-0.420358	1.724183	-1.255313	0.229182	0.396941	-5.751947	1.108109	0.679782	1.514261	2.213416	1.48759	1.378286	2.247205	1.908813	2.278164	2.14695	2.066633	1.950783	1.747621	1.910497
Zbtb43	-0.228567	4.791048	-1.255243	0.229206	0.396941	-6.383543	4.70129	4.343088	4.936458	5.130514	4.539721	4.928293	4.861747	4.930944	4.849109	4.818339	4.988055	4.872617	4.686744	4.544972
Zbed3	0.176966	4.161883	1.254711	0.229394	0.397208	-6.352773	4.55325	4.681789	4.740698	4.4153	4.598129	4.613389	3.328389	4.187837	3.87219	3.994723	4.216089	4.065203	3.467079	3.62342
Trim13	-0.272216	2.576258	-1.254509	0.229465	0.397275	-6.072907	2.8647	2.309899	2.588191	3.137672	2.919011	3.114281	2.566765	2.436799	2.364852	2.108365	2.303426	2.581351	2.549064	2.489076
Gcsh	0.207147	5.013358	1.2544	0.229504	0.397284	-6.388213	5.057571	5.208484	4.990724	4.586178	5.192798	4.709886	5.141132	4.934079	5.066451	5.138601	5.128213	4.995108	5.066477	5.020333
Uck1	0.250267	5.367084	1.25417	0.229585	0.397367	-6.396537	5.212627	5.515823	5.23974	4.594489	5.114924	4.726978	5.568419	5.522617	5.542434	5.692284	5.615344	5.621048	5.505866	5.706042
Psma5	0.190146	5.67355	1.254035	0.229633	0.397393	-6.438303	5.684832	5.482885	5.927297	5.357456	5.840583	5.578378	5.77033	5.607557	5.84348	5.722937	5.778723	5.549414	5.715431	5.601319
Cbx3	-0.279129	3.825907	-1.253816	0.22971	0.39747	-6.203634	3.402817	3.117121	3.070623	3.870824	3.628553	3.663311	4.35355	4.020912	3.996864	4.277207	4.298745	3.956168	4.125768	3.896095
Usp47	-0.229043	7.480459	-1.253679	0.229759	0.397496	-6.459935	7.397749	7.24329	7.439733	8.039901	7.412987	7.859268	7.433683	7.517025	7.41861	7.262183	7.436357	7.409006	7.519672	7.345821
Pcdhga5	-0.460503	2.435375	-1.253019	0.229992	0.397806	-5.917807	2.41698	0.973336	2.225837	2.324175	1.822268	2.537984	2.286414	2.91426	2.905753	2.499819	2.718119	2.691938	3.372046	2.721621
Cubn	0.563427	1.360302	1.252984	0.230004	0.397806	-5.674244	1.957182	1.117652	1.65069	0.166125	1.600747	1.766229	1.544223	2.268138	0.931093	1.51148	1.068256	1.805289	1.077784	1.227132
Invs	0.362429	5.063939	1.251766	0.230435	0.398495	-6.384589	4.618111	5.883263	4.899765	4.792905	4.8478	4.675081	5.542337	4.863466	5.422258	5.2885	4.884809	4.86739	4.855682	5.502352
Carf	-0.308115	3.948692	-1.250778	0.230785	0.399029	-6.278479	3.788223	3.713429	4.143209	4.324952	3.306999	3.848745	4.455977	4.095628	4.397255	3.683052	4.069701	3.555779	3.91892	4.084165
Slc18a1	0.482704	3.603232	1.250705	0.230811	0.399029	-6.173453	3.32724	4.664267	3.265371	3.145235	3.372207	3.632543	4.316148	3.169712	4.018418	3.940842	3.181236	3.277021	3.229186	4.041787
Ntrk2	-0.252505	9.851618	-1.250394	0.230921	0.399115	-6.384623	10.43931	10.02648	10.09419	9.909774	9.716269	9.861134	9.446885	9.859789	9.368978	9.673432	9.715128	10.14378	9.909415	9.759804
RGD1308428	0.230851	5.690389	1.250378	0.230927	0.399115	-6.448747	5.680283	6.212488	6.093852	5.580254	5.666949	5.470877	5.754688	5.541244	5.660235	5.667616	5.400772	5.704031	5.519575	5.74325
Tollip	-0.216195	6.97884	-1.250227	0.23098	0.399121	-6.47315	7.088915	6.534412	7.031583	6.858921	6.83407	6.630992	7.14261	7.012775	6.942825	6.881657	7.318842	7.021875	7.402486	7.014417
Kmt2e	-0.255741	8.131773	-1.250181	0.230997	0.399121	-6.446086	8.109947	8.50722	8.283677	8.802976	7.822369	8.178351	8.132023	8.141256	8.100274	8.035531	7.81929	7.942082	7.819958	8.155542
Zfp36l2	0.428233	2.166593	1.249698	0.231168	0.399322	-6.209583	3.402817	3.232585	4.065129	3.474303	3.470774	4.639415	0.357848	2.38274	0.449965	0.750369	1.199219	1.848377	0.921344	0.65743
Cnnm4	-0.179247	4.968856	-1.249642	0.231188	0.399322	-6.378276	4.698293	4.785379	4.561895	4.84492	4.439837	4.600198	5.295647	5.25314	5.234726	5.229048	4.994832	5.329459	5.054755	5.293472
Capza2	-0.338179	7.742236	-1.249573	0.231212	0.399322	-6.460087	7.824408	6.850054	7.77548	8.119808	7.902362	8.013005	7.881019	7.648357	7.773421	7.442155	7.951458	7.662039	8.081726	7.473531
Gpbp1	-0.252289	6.701476	-1.249257	0.231325	0.399458	-6.473536	6.8479	6.565421	6.895488	7.553587	7.124494	7.2059	6.535645	6.354253	6.385681	6.250484	6.616008	6.375322	6.663955	6.461932
Spata2	-0.359212	5.489647	-1.248915	0.231446	0.399611	-6.455153	5.808099	4.735912	6.06012	6.036796	5.878108	5.560782	5.050088	5.615391	5.20918	5.14338	5.596159	5.37253	5.703469	5.121014
Fcgbp	0.543841	2.340271	1.248255	0.231681	0.399958	-5.826362	2.246933	1.854335	1.792283	1.065902	2.046579	2.915623	2.518182	1.983359	3.375441	2.641672	2.133674	2.027776	2.994118	3.510555
Ythdf3	-0.281615	5.561045	-1.247916	0.231801	0.400107	-6.464382	5.799735	5.414661	5.915737	6.47746	5.988284	6.11113	5.288423	5.391882	5.237026	4.885482	5.455844	5.162375	5.615248	5.145679
Rock2	-0.286031	8.187982	-1.247826	0.231834	0.400107	-6.445977	8.185723	7.828905	8.512117	8.940272	8.412932	8.350706	8.126632	8.075684	8.0426	7.731548	8.219126	7.82663	8.397872	7.986477
Wfs1	0.573592	8.529113	1.247491	0.231953	0.400225	-6.469674	8.409722	7.187651	8.378488	6.162305	7.461363	8.432804	9.068141	9.636409	8.983426	9.136089	9.344906	9.242095	8.840596	9.129233
Lypd8	2.000501	-3.69195	1.247447	0.231968	0.400225	-4.861849	-3.260591	-3.260591	-1.909221	-2.344749	1.082564	-1.612049	-2.558588	-3.260591	-3.260591	-1.564464	-2.697527	-3.260591	-3.260591	-3.260591
Hif3a	1.344227	0.653953	1.246865	0.232175	0.400491	-5.575668	0.873216	3.445867	3.259286	-0.02435	-0.424712	0.61372	0.625837	0.137473	0.449965	-0.311823	0.631286	-0.070959	0.962073	0.190106
Kiss1r	-0.561775	1.266632	-1.246796	0.2322	0.400491	-5.630754	1.459129	0.533043	1.169557	0.896996	1.082564	-0.291112	1.372662	1.279465	2.330799	1.481601	1.719153	1.890216	1.377871	2.159814
Rsbn1	-0.207785	4.593346	-1.246732	0.232223	0.400491	-6.39366	4.559869	4.604273	4.843931	5.251074	4.713338	4.652254	4.56363	4.455617	4.553063	4.290193	4.404331	4.398075	4.686744	4.39593
Stra6	-0.830976	3.360649	-1.246203	0.232412	0.400759	-6.121097	4.748409	1.934482	2.2133	2.108996	3.181638	2.818727	4.267989	2.835955	4.473365	3.826639	4.597784	3.037402	3.203938	3.988241
Fbxl20	-0.400751	2.398726	-1.245968	0.232495	0.400846	-5.945456	2.295333	1.702379	2.067688	2.546081	1.895073	2.434761	2.518182	2.951874	2.107651	2.184529	2.616315	2.749899	3.318158	2.503027
Slc9a9	-0.332315	4.405655	-1.245719	0.232584	0.400941	-6.316617	4.485336	3.397198	3.614277	4.082584	4.405982	4.355253	4.468816	4.757708	4.504248	4.51702	4.647377	5.00228	4.934548	4.584872
Slc44a5	-0.396739	1.99208	-1.245321	0.232726	0.401129	-5.854043	1.401336	1.79116	1.890044	2.634716	1.312595	1.648138	2.343298	2.007376	2.493553	1.828755	2.415434	2.2368	2.11575	2.17732
Shprh	-0.264533	5.925101	-1.244982	0.232847	0.40128	-6.466595	5.618218	5.882	5.812506	6.54834	5.513542	6.079159	6.164213	5.999045	6.177512	5.763854	5.766893	5.666902	5.966272	6.019226
Mrpl32	0.238207	3.833149	1.244822	0.232904	0.40132	-6.318632	3.912376	4.046648	4.326082	4.298064	4.675086	4.336284	3.713543	3.386664	3.35864	3.588098	3.786926	3.30822	3.6527	3.388105
Letm2	-0.290922	4.568483	-1.244189	0.23313	0.401652	-6.375338	4.55325	5.044346	4.239088	4.881605	4.377679	4.101824	4.832242	4.374841	4.614504	4.782026	4.338363	4.40891	4.474404	5.003257
Gfod2	-0.225252	3.356427	-1.243549	0.233359	0.401989	-6.209087	3.562947	3.134193	3.442311	3.611667	3.428477	3.432728	3.318966	3.301815	3.178898	3.019813	3.414796	3.461789	3.683472	3.151754
RGD1307325	-0.308639	3.557463	-1.243306	0.233446	0.402081	-6.271022	3.569521	3.27168	3.859257	4.341506	3.574163	3.905613	3.127125	3.583227	3.375441	3.177227	3.61576	3.461789	3.882251	3.195366
Fnip1	-0.271286	5.570693	-1.242876	0.2336	0.402287	-6.458076	5.365903	5.17794	5.671838	6.202733	5.29218	5.917334	5.759921	5.510064	5.591793	5.362092	5.465635	5.392683	5.719891	5.59319
Myeov2	-0.225918	6.243479	-1.242785	0.233632	0.402287	-6.479042	6.806059	6.373003	6.006633	6.366984	6.683563	6.379372	6.2658	5.964191	6.270267	6.048759	6.196346	5.877865	6.251467	5.939396
Ppp3cc	-0.183242	4.883594	-1.242122	0.23387	0.402582	-6.427193	5.174319	4.730309	5.112182	5.309459	5.128199	5.258917	4.739927	4.886311	4.676828	4.66726	4.770972	4.578447	4.621498	4.769331
Tpp1	-0.254385	6.758382	-1.242118	0.233871	0.402582	-6.478379	6.80048	6.317103	5.939717	6.182661	6.564457	6.126853	6.762013	7.353918	6.842923	7.186643	7.070416	7.595625	6.797952	7.092038
RGD1562608	0.281377	2.513267	1.241926	0.23394	0.402643	-6.007884	2.342161	2.982209	2.744481	2.568755	2.348294	2.898483	2.893159	2.400986	2.223535	2.291729	2.360517	2.252925	2.494578	2.660694
Ccdc148	-0.356365	2.29702	-1.241677	0.234029	0.402739	-5.97878	2.091157	2.294795	2.401882	2.951788	2.124346	1.943815	2.841442	2.400986	2.446625	2.600561	2.181985	2.330954	1.881043	1.991897
Hk1	-0.174442	9.493838	-1.240911	0.234304	0.403139	-6.409291	9.732758	9.34313	9.720152	9.861019	9.650187	9.779738	9.231026	9.362422	9.395861	9.3254	9.394441	9.27158	9.506957	9.341249
Dlst	-0.158964	7.401565	-1.240842	0.234328	0.403139	-6.476325	7.501099	7.353993	7.4026	7.791463	7.557366	7.633473	7.413348	7.215804	7.336796	7.181425	7.388264	7.168813	7.291507	7.39528
Ccdc41	-0.279298	5.017048	-1.240442	0.234472	0.403281	-6.42472	5.000326	5.093178	4.438066	5.424255	5.276936	4.959619	5.395408	4.688835	5.037713	5.235776	4.87014	4.814046	4.86645	5.187315
Tmem65	-0.25382	5.446626	-1.240312	0.234519	0.403281	-6.472508	5.907455	5.51093	5.860529	6.277764	5.642047	6.341823	5.214116	4.757708	5.021793	5.190317	5.242726	4.9879	5.264678	5.070381
Rer1	0.188943	6.179214	1.240286	0.234528	0.403281	-6.473773	6.075082	5.954672	6.097267	5.934662	6.369047	6.350514	5.953525	6.08901	6.233878	6.349962	6.179588	6.343674	6.265772	6.33412
Sox13	0.241721	4.340203	1.240239	0.234545	0.403281	-6.348698	4.54993	4.673054	4.498728	4.030446	4.608991	4.320284	4.407896	4.032662	4.401362	4.592349	4.061116	4.212646	4.018826	4.432837
Dld	-0.246641	6.562887	-1.240028	0.234621	0.403353	-6.485098	6.637172	6.264775	6.511755	7.188961	6.869507	7.019793	6.73658	6.189523	6.485787	6.249376	6.440126	6.14739	6.561657	6.594891
Sgms1	-0.194468	5.337007	-1.239676	0.234747	0.403512	-6.445047	5.535003	5.324578	5.462667	5.384868	4.95385	5.314898	5.259158	5.314586	5.481658	5.072488	5.285734	5.479185	5.539897	5.348595
Ubap2	-0.160041	6.873279	-1.239032	0.234979	0.403853	-6.484521	7.133415	6.980848	7.058115	7.452814	7.157701	7.334536	6.653733	6.655296	6.584824	6.601619	6.596089	6.569696	6.764523	6.696275
Dgcr6	0.269841	6.332127	1.238107	0.235312	0.404367	-6.483067	6.629326	7.072813	6.550893	6.036796	6.692527	6.09241	6.190288	6.2258	6.263514	6.280078	6.355768	6.044614	6.115906	6.11885
Ap3m1	-0.182767	5.757153	-1.237724	0.235449	0.404515	-6.471282	5.979578	5.740071	5.527397	5.87109	6.044377	5.66074	5.607646	5.812285	5.525598	5.680825	5.846486	5.938943	5.825753	5.568526
Has1	0.507935	1.78229	1.237608	0.235491	0.404515	-5.706398	0.873216	2.264105	1.792283	1.327578	0.808768	1.606524	2.48486	2.007376	2.39812	2.359011	1.844511	1.618784	1.79348	2.261799
Col9a1	0.948516	1.376375	1.237588	0.235498	0.404515	-5.774583	1.669604	4.028502	2.010869	0.896996	0.470544	2.434761	1.668148	0.76329	0.449965	1.21727	0.676952	1.186803	1.040235	1.445134
Aspa	-0.283587	4.28474	-1.237325	0.235593	0.404558	-6.342186	4.3275	4.667202	3.814306	4.4153	4.051203	3.875271	4.14005	4.112335	4.281688	4.324262	4.287749	4.800396	4.460257	4.510857
Gabre	0.98335	-0.600461	1.237246	0.235622	0.404558	-5.220538	-0.899176	-0.273517	0.442561	-1.388744	-0.954037	-0.457681	-1.009457	-0.511025	-1.304935	1.355481	-0.731011	-0.657145	0.546019	-0.03329
Mis12	-0.375747	3.003637	-1.237237	0.235625	0.404558	-6.194941	3.439172	2.354284	3.158259	3.627954	3.372207	3.713177	2.841442	2.901501	2.95129	2.221154	2.696109	2.990087	3.416687	2.570827
Map4k4	0.277679	8.724941	1.236748	0.235801	0.404783	-6.447869	8.630742	9.485661	8.831081	8.8076	8.823494	8.835636	8.726194	8.521574	8.647722	8.819873	8.05696	8.78132	8.431118	8.753974
Ccar1	0.212956	6.491819	1.236671	0.235829	0.404783	-6.487577	6.362185	6.938351	6.796007	6.805628	6.58798	6.867368	6.692562	6.219389	6.365849	6.416616	6.035834	6.180436	6.148254	6.486717
Ramp3	-0.458141	2.32153	-1.236593	0.235857	0.404783	-5.898624	2.776524	2.134368	2.250588	1.63746	1.390206	1.136566	2.659281	2.400986	2.39812	3.070874	2.592682	2.8487	2.913344	2.635584
Tmem55a	-0.25172	5.737975	-1.236324	0.235954	0.404892	-6.480965	6.026926	5.523942	5.921529	6.467691	6.326082	6.211698	5.59599	5.382731	5.489397	5.335194	5.51519	5.346466	5.85308	5.365875
Josd1	-0.200258	5.704839	-1.236219	0.235992	0.404899	-6.469153	5.959694	5.565437	5.943517	5.699348	5.446997	5.552587	5.701288	5.593743	5.555394	5.689019	5.866333	5.680379	5.993542	5.650719
Cnot2	-0.158308	6.004655	-1.236107	0.236032	0.404911	-6.481504	6.070454	5.990266	6.126813	6.382265	5.969443	6.188088	6.007318	6.000542	6.052497	5.839422	5.908818	5.88435	5.851726	5.817698
Ankrd52	-0.273524	5.298873	-1.235986	0.236076	0.404928	-6.438597	5.062241	5.140412	5.27032	5.592754	4.767666	5.053587	5.439557	5.583794	5.466056	5.000567	5.414317	5.319923	5.930803	5.182988
Zfp652	-0.219178	3.748451	-1.235834	0.236131	0.404964	-6.332047	4.15463	3.838476	4.235992	4.37727	3.850732	4.257812	3.356296	3.698353	3.262563	3.276274	3.487079	3.650119	3.748946	3.403062
Frmd6	-0.141825	5.031171	-1.235309	0.23632	0.405231	-6.414527	4.955851	4.838423	4.811028	4.97581	4.779733	4.870133	5.300443	4.9924	5.24619	5.157622	5.078034	5.175124	5.112432	5.191628
Ush2a	-0.54127	2.437014	-1.235173	0.236369	0.405258	-5.945623	3.263791	1.991799	2.225837	1.615848	1.72577	1.496914	3.137884	2.76729	2.810125	1.828755	2.149958	3.118845	3.416687	2.890335
Eya4	-0.343572	2.208903	-1.23503	0.236421	0.405288	-5.954034	1.895723	2.309899	2.039558	2.844379	2.046579	1.857753	2.398025	2.795149	2.204856	1.964563	2.049375	2.315682	2.285827	2.261799
LOC497934	-0.211902	4.752244	-1.234518	0.236606	0.405548	-6.380203	4.794038	4.077865	4.48836	4.476537	4.743225	4.355253	4.877881	5.059983	4.896441	4.930413	5.217838	5.025932	4.919116	4.728352
Bloc1s1	0.251779	5.496707	1.233968	0.236805	0.405799	-6.465089	5.967183	6.114818	5.597812	5.155071	5.958868	5.563503	5.146469	5.297643	5.410079	5.455357	5.375027	5.308397	5.231744	5.407263
Soat1	0.321715	4.23261	1.233925	0.23682	0.405799	-6.330436	4.110227	3.879549	4.650582	4.04669	4.387176	4.639415	3.713543	4.360928	4.086326	3.901891	4.284065	4.811327	4.42791	4.041787
Sf3a1	-0.221515	7.04219	-1.233615	0.236933	0.405915	-6.489632	7.123396	6.870572	7.19671	7.688788	7.171505	7.497437	6.775855	7.049383	6.891185	6.78405	7.01148	6.869412	6.963815	6.70912
Adck1	-0.241315	3.92096	-1.233552	0.236956	0.405915	-6.242882	3.932918	3.046741	3.8018	3.498125	3.585206	3.384299	4.263083	4.123366	4.359752	4.135691	4.291424	4.114573	4.290742	4.174106
Utp23	0.231342	2.933253	1.233263	0.23706	0.406037	-6.199833	3.073383	3.492948	3.458336	3.589663	3.633881	3.708268	2.467906	2.634802	2.278164	2.558244	2.771719	2.346065	2.84965	2.417223
Utp18	0.210956	4.67455	1.232821	0.23722	0.406204	-6.376688	4.28818	4.375753	4.886008	4.621853	4.646377	4.753435	4.788531	4.754164	4.71693	4.647212	4.867681	4.66836	4.969927	4.521176
Exoc3l1	0.396494	3.672465	1.232756	0.237244	0.406204	-6.176962	3.582581	4.064569	3.458336	2.768056	3.76592	2.957603	4.091065	3.599118	4.081214	4.106622	3.58015	3.822092	3.488149	4.178459
Gzf1	-0.148337	4.934343	-1.232713	0.237259	0.406204	-6.424567	4.980729	4.841025	4.833729	5.120956	4.949583	4.9923	5.092185	4.836907	4.852113	4.924873	4.904139	4.883016	5.042938	4.877707
Abhd14b	-0.278568	4.598216	-1.232484	0.237342	0.406249	-6.417178	5.52489	4.755351	4.464755	4.491448	4.949583	4.722115	4.202878	4.342165	4.303799	4.623466	4.733642	4.43741	4.420622	4.468825
Kcnn2	-0.283344	5.411189	-1.232453	0.237354	0.406249	-6.442939	5.214725	5.625525	4.629506	5.447382	5.305596	4.670039	5.735337	5.557602	5.855505	5.578679	5.437719	5.412558	5.52128	5.803676
Ptp4a1	-0.317004	2.352393	-1.232317	0.237403	0.406276	-5.983933	2.32672	1.655851	2.47722	2.656049	2.295481	2.257897	2.467906	2.555575	2.539002	2.239124	2.568656	2.36102	2.794335	2.050068
Elac2	0.204678	6.005899	1.231873	0.237564	0.406493	-6.471735	5.672669	6.215499	5.904083	5.639022	5.793748	5.531895	6.401452	6.173692	6.185871	6.198623	6.013759	6.045771	6.035891	6.295565
Rtf1	-0.232138	7.142622	-1.231453	0.237716	0.406606	-6.485954	7.486408	7.155613	7.534427	8.001353	7.44289	7.9128	6.775855	6.892195	6.766964	6.712696	7.003661	6.779044	6.887237	6.65704
Pcdhb12	0.378492	1.565002	1.231432	0.237723	0.406606	-5.694299	0.785575	1.702379	1.631973	1.327578	0.88224	1.541762	2.033384	1.830204	2.00342	1.419918	1.605665	1.568131	1.881043	2.245294
Mrps12	0.19818	4.265393	1.231405	0.237734	0.406606	-6.36957	4.655673	4.489567	4.655224	4.117607	4.630473	4.524012	3.945565	3.923301	4.217909	4.159476	4.302392	4.00459	4.130242	4.041787
Fam63a	0.223148	4.438426	1.231247	0.237791	0.406606	-6.366079	4.602167	4.452618	4.806861	4.098255	4.753051	4.237508	4.455977	4.429191	4.281688	4.298786	4.427894	4.53295	4.368551	4.465266
Zbtb46	-0.322788	2.106928	-1.231087	0.237849	0.406606	-5.861884	2.263247	1.58312	1.613009	1.679737	1.364797	1.993092	2.659281	2.207232	2.583064	2.694725	2.228732	2.187314	2.494578	2.325991
Pcdha8	-1.221957	-0.201642	-1.231075	0.237853	0.406606	-5.284961	-0.310971	-3.260591	0.105278	-0.093872	-0.954037	-0.006501	0.028455	0.972957	0.449965	0.280604	1.403186	0.081289	1.316372	0.554014
Ndst1	-0.29663	5.799621	-1.231039	0.237866	0.406606	-6.476651	5.875975	5.31899	5.964241	5.924809	5.303926	5.819419	5.717517	6.059162	5.745101	5.378399	5.989079	6.066448	6.327473	5.732928
Ston1	0.422496	2.291034	1.230293	0.238137	0.407011	-6.053313	2.977763	2.324846	2.893363	2.169587	2.987204	3.62735	1.697535	2.030999	1.663747	1.704918	1.740817	2.489067	2.423441	1.684306
Desi2	0.251621	3.78509	1.230149	0.238189	0.407042	-6.310389	3.94308	4.37216	4.413617	3.915736	3.686105	4.244308	3.392685	3.475434	3.533498	3.424979	3.673224	3.750073	3.871601	3.410483
LOC500684	0.43329	2.015217	1.23002	0.238237	0.407065	-5.841851	1.514695	2.184396	2.578448	1.780306	1.705671	1.821837	2.787802	2.24812	1.981639	2.221154	1.844511	2.203999	1.493524	2.245294
Arhgap26	-0.357675	3.940029	-1.229453	0.238443	0.407303	-6.350077	4.069061	4.12132	4.490959	4.711187	3.639189	3.905613	3.479833	3.606999	3.87219	3.14899	4.172914	3.838214	4.405935	3.803307
Nmur2	-1.974273	-0.560618	-1.229451	0.238443	0.407303	-5.109369	0.592497	-2.680539	-2.787702	-2.040259	-3.260591	0.809818	3.261094	0.517885	-0.080923	0.799637	-0.231835	1.315214	0.546019	1.158276
Azi2	-0.165584	5.19108	-1.229111	0.238567	0.407456	-6.451893	5.247895	5.02159	5.17333	5.482138	5.372461	5.23522	5.185883	5.138201	5.097162	5.266765	5.296745	5.042261	5.170065	4.988458
Gna13	0.38816	3.144508	1.228597	0.238754	0.407717	-6.222504	3.509242	3.806886	4.391531	3.261145	2.95351	3.875271	2.73209	2.835955	2.74697	2.499819	2.604547	3.292705	3.051863	2.648193
Emc3	0.178257	7.063627	1.227919	0.239001	0.408081	-6.498981	6.894466	6.748396	7.029349	6.582863	7.037217	6.851313	6.97048	7.167329	7.104606	7.297448	7.20947	7.34557	7.292007	7.372189
Gatc	-0.170853	5.778108	-1.226934	0.23936	0.408583	-6.493182	6.172233	5.982039	5.907005	6.369407	6.351367	6.16229	5.60958	5.452183	5.485533	5.502614	5.584225	5.368835	5.594349	5.381063
Armc9	0.168072	6.462386	1.226811	0.239405	0.408583	-6.496643	6.29831	6.508461	6.677224	6.439518	6.45659	6.505717	6.656541	6.449367	6.638009	6.540437	6.352254	6.286073	6.26068	6.422104
Asap1	-0.223268	6.457414	-1.226804	0.239407	0.408583	-6.501047	6.893811	7.018111	6.878219	7.100791	6.45659	6.744455	5.94895	6.39351	6.133744	5.982299	6.314828	6.205509	6.444379	5.906972
Dnpep	0.273031	6.9152	1.226739	0.239431	0.408583	-6.499085	6.797683	7.175849	6.627302	6.310926	7.033694	6.469179	7.269721	6.878425	7.171671	7.223216	6.837657	6.955068	6.725133	7.350651
Ncoa5	-0.174887	6.213394	-1.226352	0.239573	0.408713	-6.494842	6.18192	5.992608	6.037185	6.467691	6.184399	6.387841	6.383483	6.144209	6.190625	6.35409	6.240737	6.114674	6.104581	6.22472
Tmem189	-0.218566	6.612411	-1.226344	0.239575	0.408713	-6.498656	6.796282	6.220503	6.507269	6.384663	6.692527	6.064826	6.446503	6.784261	6.601879	6.84619	6.748902	7.068744	6.698262	6.729289
Zswim3	0.189983	4.130001	1.225641	0.239832	0.409093	-6.294676	3.742407	4.200444	4.004574	3.884445	4.059146	3.770827	4.421169	4.234368	4.175368	4.394093	4.280371	4.12775	4.250206	4.365708
Ttc39b	-0.317726	3.379552	-1.225388	0.239925	0.409193	-6.249947	3.529616	3.279374	4.000932	3.915736	2.77212	3.55809	3.428178	3.440579	3.253503	2.934159	3.181236	3.292705	3.515771	3.365375
Wdr75	0.200741	5.315513	1.225235	0.23998	0.40923	-6.445872	4.94325	5.435469	5.292838	5.26155	5.459054	5.039974	5.706718	5.199424	5.469972	5.48766	5.25407	5.19195	5.274818	5.440265
Srprb	-0.238972	5.312633	-1.224763	0.240153	0.409466	-6.45891	5.426916	4.798824	5.030571	5.410199	5.328778	5.553956	4.986034	5.326567	5.271094	5.506329	5.265325	5.605434	5.490287	5.416507
Cryl1	-0.32411	3.756057	-1.224098	0.240396	0.409823	-6.270533	4.087502	3.167741	3.330668	3.578533	4.023054	3.47381	3.691803	3.960684	4.175368	3.676473	4.235294	3.419555	4.103184	3.780579
Mettl25	0.326562	2.922853	1.223773	0.240515	0.409897	-6.046792	2.41698	3.397198	2.853524	2.62393	2.81965	2.374054	3.454237	3.159031	2.810125	3.12019	2.995472	2.951084	2.944167	3.212449
Lnx1	-0.157047	4.628927	-1.223737	0.240529	0.409897	-6.381301	4.523084	4.290759	4.399853	4.506207	4.234219	4.501484	4.946803	4.628172	4.983948	4.788142	4.647377	4.706916	4.769388	4.943127
Cherp	0.157637	7.127671	1.223699	0.240542	0.409897	-6.503232	6.979619	7.255043	7.365194	7.229104	7.283375	7.144071	7.139268	7.075887	7.166853	7.125162	7.005899	6.984941	6.85786	7.186376
Senp8	-0.375416	1.872924	-1.223389	0.240656	0.410033	-5.936564	1.853246	2.134368	1.905712	2.645422	1.643636	1.943815	1.297885	2.121805	1.959523	1.898256	1.94125	1.643459	2.408783	1.260365
Ggh	-0.231321	4.688413	-1.223056	0.240778	0.410183	-6.402854	4.633881	3.986819	4.514142	4.816781	4.6725	4.791072	4.743453	4.515458	4.905145	4.663938	4.684183	4.838295	4.908735	5.025175
MAST1	-0.189431	7.743123	-1.222316	0.241049	0.410587	-6.493712	7.687933	7.949238	7.572656	8.053279	7.585136	7.611289	8.024166	7.59537	7.927335	7.798973	7.467551	7.539626	7.707677	7.890901

Tbl1x	-0.154723	6.71399	-1.221829	0.241228	0.410833	-6.506902	6.908147	6.669354	6.644913	6.976464	6.823034	7.013339	6.629161	6.654346	6.474135	6.550366	6.613808	6.651802	6.710266	6.691715
Dhx15	0.178856	6.534038	1.221152	0.241476	0.411198	-6.507032	6.483771	6.836428	6.885645	7.021336	6.899677	7.10538	6.503768	6.234729	6.411366	6.221401	6.22449	6.065307	6.300927	6.299567
Umps	0.199369	4.475323	1.221044	0.241516	0.411207	-6.396283	4.474867	4.598135	4.354903	4.541025	4.930226	4.953408	4.272878	4.337436	4.351284	4.336834	4.467414	4.376159	4.43154	4.299305
Gnpda2	-0.270628	4.028564	-1.22085	0.241587	0.41127	-6.385321	4.206171	3.953545	4.260572	4.90852	4.154906	4.80033	3.830902	3.804966	3.91304	3.759747	3.56201	3.70975	4.004274	3.629792
Gpatch11	0.195014	4.946203	1.220221	0.241819	0.411605	-6.458334	4.935636	5.381089	5.297299	5.453487	5.240728	5.655648	4.795342	4.49437	4.746291	4.754179	4.627162	4.610089	4.749166	4.558395
Mx1	1.068044	3.388839	1.219507	0.242081	0.411989	-5.994084	2.263247	2.200694	4.223544	1.945353	3.922149	2.131561	2.433388	4.438054	3.159628	4.31582	3.776475	6.269325	3.416687	3.142871
Sh3rf1	-0.298253	5.69161	-1.219317	0.242151	0.411989	-6.492038	6.073926	5.057828	5.905058	5.811733	5.488762	6.109269	5.560444	5.885385	5.557236	5.491413	5.733515	5.628793	6.021511	5.388597
Ddi2	0.206186	4.989212	1.219262	0.242171	0.411989	-6.430099	4.592516	5.129812	5.198991	5.050208	4.789315	5.008367	4.949859	5.105197	5.002995	4.797268	5.08016	5.078905	5.240048	4.875027
Kpna6	-0.302674	4.952754	-1.219235	0.242181	0.411989	-6.451404	5.029233	4.425138	5.191021	5.461082	4.955978	5.19191	4.874668	4.961999	4.60385	4.513333	4.942039	4.968501	5.462168	4.809178
Lhb	-0.715324	0.460548	-1.219041	0.242253	0.412053	-5.600205	1.957182	-0.687936	0.049949	0.041976	0.562819	1.07687	0.202532	0.76329	0.17081	0.413143	1.629088	0.151783	0.375586	0.970221
Dvl1	0.288698	7.259693	1.218708	0.242376	0.412203	-6.509808	6.949061	7.642007	7.138639	6.664077	6.906847	6.690707	7.769558	7.288046	7.645502	7.617683	7.097956	7.26388	7.197096	7.775311
Tfe3	0.165233	5.666125	1.218249	0.242544	0.412432	-6.49164	5.900953	5.735883	6.157403	5.61062	5.83943	5.940547	5.515774	5.669074	5.299983	5.625541	5.702109	5.602291	5.380011	5.377281
Mmadhc	-0.177401	5.263705	-1.218112	0.242595	0.412457	-6.473026	5.435939	4.946264	5.442668	5.550659	5.471011	5.396521	5.278733	5.065713	5.257563	5.14338	5.233205	5.076641	5.288894	5.145679
Atg5	0.201665	4.739712	1.217993	0.242639	0.412457	-6.431731	4.904771	4.655425	5.04125	4.748906	5.203588	5.193668	4.506663	4.560794	4.445789	4.693564	4.827749	4.735888	4.646703	4.250521
Ldb2	-0.330867	4.971424	-1.21793	0.242662	0.412457	-6.465053	5.163495	4.335728	5.291347	5.682553	5.655207	5.080435	4.678618	4.616508	5.166377	4.650573	4.781463	4.597515	5.167891	4.783689
Opa1	-0.262727	6.661795	-1.217485	0.242826	0.412657	-6.511051	6.503648	6.16344	6.648992	7.079237	6.436151	6.866818	6.718776	6.759727	6.746122	6.316501	6.736824	6.572911	7.13277	6.598956
Exoc5	-0.345988	5.49949	-1.217425	0.242848	0.412657	-6.497244	5.765785	4.934131	6.033624	6.3351	5.618052	6.22293	5.193638	5.230363	5.204486	5.000567	5.412631	5.24128	5.683818	5.152334
Supt20	0.209816	6.653503	1.217194	0.242934	0.412743	-6.510192	6.37819	6.921242	6.739205	6.704952	6.609099	6.737845	6.966713	6.566144	6.696843	6.685104	6.445908	6.521424	6.304891	6.88722
Pdcd2l	0.258084	3.190196	1.216726	0.243106	0.412978	-6.141767	3.180343	2.963229	3.47943	2.728325	3.346477	2.974059	3.488265	3.047633	3.225976	3.463704	3.280721	3.220743	3.134365	3.302949
Morn5	-0.374967	3.869932	-1.216425	0.243218	0.413109	-6.375077	4.802435	4.667202	4.015447	4.140492	4.421469	3.295428	3.488265	4.003106	3.533498	3.409193	3.661913	3.269114	3.999391	3.584586
LOC100362431	-0.446888	1.035592	-1.215913	0.243407	0.413372	-5.743755	1.245833	0.118359	1.033553	1.658753	1.48759	1.426914	0.798508	0.517885	0.97585	1.355481	1.260496	1.252437	1.347449	0.799858
Islr	0.802762	2.720315	1.215715	0.24348	0.413437	-6.100713	3.736577	2.631765	2.778919	1.526009	2.944963	5.078533	2.324585	2.14364	2.241976	2.484835	3.022257	2.375822	2.269694	2.791492
Pcdhb8	0.638094	2.015649	1.215227	0.24366	0.413627	-5.71642	0.995438	0.725555	2.010869	1.033666	1.78444	1.910004	2.05625	2.58779	3.408468	1.678822	1.996325	2.447635	2.760099	3.270693
Exosc8	0.330962	4.220354	1.215207	0.243668	0.413627	-6.340273	3.832629	4.670131	3.847136	4.140492	4.502342	4.304105	4.842144	4.155962	4.405458	4.382013	3.925804	3.811244	3.844626	4.5074
Fancc	0.599507	1.607636	1.215135	0.243694	0.413627	-5.726919	1.033981	2.854122	1.245402	1.065902	1.685287	0.915838	1.986536	1.279465	1.769222	2.308845	1.403186	1.805289	1.574559	2.124152
Sla2	-0.594559	0.826142	-1.214838	0.243804	0.413754	-5.494049	0.692264	-0.366503	-0.974047	0.166125	0.470544	0.435061	1.754571	1.112357	2.278164	0.893396	1.629088	1.186803	1.407665	1.971974
Vwc2l	-0.713076	0.017571	-1.214675	0.243865	0.413798	-5.80778	1.178614	2.354284	1.936547	2.401998	0.562819	-0.214525	-0.831021	-2.076466	-1.10526	-0.806258	-0.231835	0.081289	-0.405292	-0.505482
Ankmy2	-0.17257	6.779743	-1.214147	0.24406	0.414072	-6.514691	7.040636	6.851345	7.106797	7.412251	6.990736	7.266024	6.53259	6.573201	6.591131	6.423508	6.624773	6.396344	6.629602	6.491973
Hacl1	-0.208074	3.956966	-1.213748	0.244208	0.41421	-6.302091	3.838085	3.667262	3.436929	3.954995	3.850732	3.826262	4.519061	3.798099	4.250151	4.091865	3.807603	4.092341	4.116777	4.250521
Wdr53	-0.189682	3.548377	-1.213741	0.24421	0.41421	-6.300658	3.805036	3.843675	3.702138	3.959292	3.422332	3.826262	3.504983	3.559057	3.440756	3.432808	3.414796	3.204252	3.40196	3.294952
Dsc2	-0.531525	0.131433	-1.21276	0.244574	0.414728	-5.581391	1.310059	-0.273517	-0.130036	0.280432	0.808768	0.65513	0.028455	0.222004	-0.175642	-0.665882	0.631286	0.151783	-0.040977	0.499386
Hspbp1	0.339325	5.939389	1.21273	0.244585	0.414728	-6.491847	6.129489	6.128726	5.996609	5.027879	6.254387	5.263945	5.989609	6.172365	6.06161	6.042377	6.258681	5.86743	6.097743	5.887162
Ctns	0.213153	3.890582	1.212587	0.244638	0.41476	-6.276363	3.782575	3.894655	3.711083	3.450082	3.818339	3.870884	4.14005	4.032662	3.793114	4.130887	3.862983	4.335093	3.754753	3.998126
Sap30bp	0.141785	6.274958	1.212446	0.244691	0.414789	-6.51652	6.602329	6.570917	6.831816	6.653509	6.867812	6.807794	5.883333	6.10857	5.908415	6.013956	6.003714	5.873961	5.886526	5.857606
Sycp2	0.430908	1.294916	1.212355	0.244724	0.414789	-5.717615	0.785575	2.046927	1.245402	1.300739	1.643636	1.046069	2.078759	1.067371	1.867508	1.28803	0.584127	0.714338	1.347449	1.779078
Smim14	0.200974	5.88898	1.212254	0.244762	0.414794	-6.503092	5.974632	5.752562	6.345072	5.769538	6.010984	6.174802	5.768601	5.774298	5.873358	5.654285	6.019309	5.768545	5.962514	5.623839
RGD1562865	-0.380357	1.846125	-1.212016	0.24485	0.414885	-5.978461	2.32672	2.264105	2.508341	2.43939	1.705671	1.563674	1.335758	1.658838	1.743567	1.14286	1.82436	2.315682	2.079189	1.386135
Arl14ep	-0.186717	5.158742	-1.211864	0.244907	0.414922	-6.466582	5.085364	5.150935	5.09178	5.496992	4.884023	5.3068	5.246787	5.321786	5.237026	5.011064	5.160721	5.030616	5.195908	5.046765
Elof1	0.251818	5.073911	1.211706	0.244965	0.414963	-6.478649	5.745606	5.598103	5.286867	4.928381	5.964165	5.483864	4.606864	4.949657	4.635579	4.79119	4.92558	4.76429	4.831156	4.571694
LOC683897	-0.203592	4.898606	-1.211553	0.245022	0.415001	-6.477892	5.284322	5.228494	4.96291	5.626918	5.207167	5.626016	4.622269	4.556731	4.621564	4.670575	4.773602	4.444449	4.488413	4.521176
Sparcl1	-0.408496	12.13694	-1.210938	0.245251	0.415308	-6.331186	12.8431	12.32277	12.47452	12.62199	12.14604	12.35951	11.15624	11.14053	12.22007	12.13188	11.70222	12.17093	12.14906	12.47864
Arf5	-0.156144	7.79657	-1.210879	0.245272	0.415308	-6.500504	8.231527	7.772099	8.0525	8.045217	8.061569	8.047674	7.668372	7.506005	7.757087	7.686829	7.658211	7.51727	7.519672	7.635072
Lrp5	0.193278	4.744396	1.210614	0.245371	0.415389	-6.447548	4.968344	5.17794	4.802683	4.85187	5.212518	5.223224	4.494157	4.313555	4.582303	4.369831	4.460902	4.803137	4.51603	4.704402
Fah	-0.373422	5.660721	-1.210564	0.24539	0.415389	-6.468538	5.564924	5.783999	4.448801	5.160679	5.278638	4.478599	6.136317	6.060597	6.073242	6.216874	5.782645	6.184645	5.759422	6.355423
Ctdnep1	0.206912	6.825864	1.210207	0.245522	0.415547	-6.517501	6.695441	6.900189	6.584745	6.229662	6.858739	6.331483	6.882857	6.920139	7.002853	7.180844	7.066668	7.048679	6.830625	7.043345
Ganab	-0.189407	8.167005	-1.209942	0.245621	0.415547	-6.505306	7.97686	7.728152	7.574802	8.020242	7.881516	8.051071	8.564395	8.286338	8.544256	8.396902	8.21502	8.340634	8.139743	8.623761
Arl6ip4	0.204517	5.784969	1.209914	0.245632	0.415547	-6.504506	5.935721	6.081102	6.143429	5.723547	6.109886	5.797626	5.715723	5.535061	5.802136	5.630656	5.979999	5.290932	5.713941	5.558541
Pnma3	-0.318303	5.806897	-1.209905	0.245635	0.415547	-6.504642	5.476734	5.96425	5.974492	6.506378	5.187373	5.58375	5.992576	5.857579	6.088607	5.91961	5.473744	5.578496	5.733188	5.988566
Hs6st2	-0.291104	4.297889	-1.209848	0.245656	0.415547	-6.362356	3.948134	3.390109	4.047581	4.629961	4.173318	4.389395	4.394501	4.35626	4.656351	4.135691	4.427894	4.461895	4.592606	4.649015
Ormdl3	-0.209023	5.463952	-1.209576	0.245757	0.41566	-6.471169	5.471478	4.690471	5.285371	5.186565	5.26153	5.219778	5.33825	5.854273	5.580969	5.667616	5.789158	5.816802	5.706469	5.663198
Purg	-0.61002	1.318983	-1.208346	0.246216	0.416272	-5.842159	1.341131	-0.025882	1.574317	2.427034	1.338932	2.271274	0.851758	1.198329	1.360416	1.023513	1.230182	1.488652	2.220182	0.844371
Rbbp4	-0.349167	1.109886	-1.208344	0.246216	0.416272	-5.84397	1.541696	1.769471	1.613009	2.029493	0.952152	1.519512	0.429655	1.020936	0.97585	0.348395	0.885831	0.969826	1.001683	1.227132
Swt1	0.206994	4.289157	1.208327	0.246223	0.416272	-6.38473	4.137033	4.716207	4.483148	4.430854	4.355276	4.434686	4.587372	3.897831	4.160904	4.228565	3.822919	4.196062	4.24196	4.436477
Cep70	-0.266767	3.847907	-1.208102	0.246307	0.416355	-6.313705	3.724844	4.064569	3.767911	4.163019	3.523394	3.414757	4.362751	3.690957	4.254698	3.709073	3.467722	3.99982	3.777752	4.060775
Rwdd4	-0.289444	4.833804	-1.207814	0.246414	0.416478	-6.478754	5.225173	4.46953	5.147205	5.645703	5.305596	5.656923	4.637512	4.548571	4.485023	4.361652	4.739035	4.482556	4.637302	4.388433
Vkorc1	0.311028	4.198001	1.207599	0.246494	0.416509	-6.352054	4.428604	4.585778	4.004574	3.606198	4.801203	3.888353	4.028845	4.182573	4.096495	4.349296	4.411103	4.273193	3.994491	4.208569
Cblb	-0.298586	4.716435	-1.207579	0.246502	0.416509	-6.436047	4.45014	4.670131	5.003522	5.342731	4.078813	4.667512	4.842144	4.905609	4.887683	4.690302	4.700858	4.703986	4.495367	4.652149
Rnf31	0.243673	5.582547	1.206743	0.246814	0.416953	-6.481653	5.249943	5.820051	5.846391	5.337788	5.436364	5.05165	5.724671	5.749593	5.868916	5.695542	5.51676	5.681868	5.417104	5.79236
Atp10a	-0.379379	4.239953	-1.206689	0.246834	0.416953	-6.390306	4.67714	5.097537	3.661186	4.321618	3.909025	3.875271	4.57555	3.798099	4.621564	4.345154	3.750012	4.316033	4.116777	4.380898
Tuba1a	0.273833	3.744182	1.206582	0.246874	0.416962	-6.201454	3.431974	3.294639	3.215954	2.925676	3.746377	3.502454	4.348928	3.599118	4.222559	3.962637	4.073974	3.83286	3.944552	4.440107
Styx	0.459846	1.065812	1.206093	0.247057	0.417212	-5.630266	0.369528	1.047298	1.841991	0.932396	0.562819	0.915838	1.511505	1.393205	1.426399	1.180545	1.03357	1.152831	1.347449	0.970221
Spata6	0.336038	3.042133	1.205851	0.247147	0.417306	-6.210885	3.838085	3.576505	3.359399	2.57996	3.706477	3.420773	2.629097	2.634802	2.680923	2.681644	3.421519	2.921125	2.701169	2.635584
Sostdc1	-4.392247	-0.533233	-1.205485	0.247284	0.417479	-5.557717	7.586236	-1.463779	-1.367151	-1.388744	-1.798946	-0.750296	-1.21312	-1.720374	-2.155202	4.410044	6.622587	-3.260591	-1.904936	-2.653844
Sh3glb2	-0.156547	8.295945	-1.205239	0.247376	0.417576	-6.499121	8.503047	8.257718	8.380943	8.349746	8.327759	8.11623	8.287768	8.214653	8.330957	8.339756	8.157789	8.381774	8.097808	8.402296
Mtfp1	-0.252552	3.155741	-1.204959	0.247481	0.417677	-6.20705	3.516065	2.97275	3.021133	3.204354	3.346477	3.069038	3.003159	2.76729	3.35864	3.158464	3.380703	2.703718	3.4385	3.417866
Prss22	-0.785309	0.74259	-1.204893	0.247506	0.417677	-5.621353	0.785575	-0.950476	1.116684	1.245516	1.981199	-0.984601	1.607516	0.517885	1.914249	0.534523	0.961591	0.34469	1.218908	1.260365
Tmem26	0.626097	0.283298	1.203774	0.247925	0.418303	-5.652302	0.031429	1.991799	0.715742	1.327578	1.20216	1.450626	0.028455	-0.150641	0.512061	-0.027758	-0.149817	0.84773	-1.638202	-0.621773
Elf2	0.142609	5.341555	1.203717	0.247947	0.418303	-6.488279	5.354528	5.643521	5.441325	5.384868	5.56186	5.407165	5.122294	5.438999	5.151822	5.46111	5.274637	5.292883	5.06414	5.221467
LOC498265	0.281381	2.702377	1.203219	0.248133	0.418546	-6.092631	2.582145	3.33211	2.861581	2.687469	2.865664	2.644629	2.979429	2.539194	2.810125	2.770817	2.332254	2.594065	2.285827	2.791492
Slx4ip	-0.385741	1.08098	-1.203147	0.24816	0.418546	-5.675116	0.592497	1.047298	0.715742	1.548999	0.606834	0.571087	1.478026	1.356279	1.60797	1.419918	1.102128	1.186803	1.574559	1.085968
Mdp1	-0.173491	4.2372	-1.203022	0.248207	0.418567	-6.382492	4.335237	4.252185	4.015447	4.33821	4.162299	4.237508	4.425566	4.203515	4.329893	4.429738	4.298745	4.083352	4.075609	4.217057
Pcdhgb7	-0.28488	4.132749	-1.202203	0.248514	0.419026	-6.363014	4.141452	3.673115	4.290718	4.321618	3.506066	4.297582	3.783728	4.259584	4.504248	4.130887	4.111876	4.368779	4.182881	4.377115
Magt1	0.382624	2.921886	1.202088	0.248558	0.41904	-6.200125	3.516065	3.117121	3.599901	2.728325	3.252623	3.922669	2.05625	2.964197	2.185932	2.202958	2.627988	3.228918	3.061266	2.660694
Tkt	0.160983	7.850104	1.201862	0.248642	0.41911	-6.517766	7.92525	7.851983	7.940969	7.560674	8.085584	7.741051	7.677619	7.838161	7.845663	7.853213	7.938919	7.946737	7.866292	7.836159
Wipi2	-0.171127	5.501948	-1.201791	0.248669	0.41911	-6.498262	5.485452	5.224514	5.644084	5.698063	5.322193	5.489598	5.599886	5.565712	5.519942	5.271138	5.567652	5.470592	5.68534	5.517894
Nhp2l1	0.269408	2.727781	1.201573	0.248751	0.41919	-6.138866	2.765113	2.679063	3.131857	2.934433	3.534831	3.106838	2.286414	2.709903	2.539002	2.600561	2.823399	2.346065	2.614401	2.357047
Derl1	-0.222591	6.349916	-1.201189	0.248896	0.419331	-6.522455	6.206383	5.494499	6.196941	6.28634	6.165208	6.234075	6.369857	6.376322	6.556557	6.479382	6.576644	6.67891	6.606504	6.690953
Zdhhc4	0.204226	4.267795	1.201165	0.248905	0.419331	-6.347924	4.145858	4.361326	4.089343	3.786283	4.285899	3.931123	4.228269	4.294162	4.441807	4.66726	4.50904	4.506292	4.20426	4.380898
Crb3	-0.906061	0.715764	-1.200884	0.24901	0.419389	-5.834711	3.263791	1.894965	0.883381	0.664146	0.952152	0.386712	0.282279	-0.150641	-0.386063	1.419918	2.995472	-0.153565	-0.628995	-0.204094
Cse1l	-0.22827	6.175403	-1.200883	0.249011	0.419389	-6.524629	6.194735	5.668637	5.952031	6.484931	6.294469	6.531657	6.244803	6.137423	6.238477	5.936228	6.179588	5.999929	6.335245	6.279444
Ap4b1	-0.15587	4.520824	-1.200795	0.249044	0.419389	-6.406637	4.592516	4.324616	4.374741	4.412169	4.390327	4.287741	4.781687	4.632039	4.65291	4.653926	4.555623	4.430338	4.522853	4.748987
Tcp11l1	-0.406726	4.784684	-1.199881	0.249388	0.419909	-6.474716	4.975787	4.63158	5.527397	5.609254	4.230707	5.150884	4.316148	4.523808	4.697018	4.228565	4.717344	4.529644	5.488545	4.418187
Lnx2	0.201241	3.930135	1.199781	0.249426	0.419914	-6.357921	4.28818	4.294561	4.38316	3.761892	4.067045	4.116722	3.810873	3.791198	3.774234	3.896239	3.644778	3.936334	3.562873	3.797658
Ywhae	-0.348828	10.40527	-1.199681	0.249463	0.419919	-6.407964	10.8373	10.04529	10.7009	11.47205	11.06881	11.40803	9.848559	10.10678	10.04521	9.840546	10.22633	9.985334	10.18367	9.906178
Pax1	2.088693	-3.825088	1.199487	0.249536	0.419982	-4.898554	-3.260591	-0.813242	-3.260591	-2.731185	-2.73597	1.07687	-2.558588	-3.260591	-3.260591	-3.260591	-2.697527	-1.925331	-3.260591	-3.260591
Cul2	-0.247305	6.078828	-1.199203	0.249643	0.420104	-6.525358	6.049442	5.437189	6.265336	6.519532	6.243972	6.200378	6.158941	5.898287	6.216499	5.794558	6.019309	5.88435	6.328446	6.1109
Ndufab1	-0.208826	5.539005	-1.198975	0.249729	0.420163	-6.509596	5.843793	5.218524	5.510845	5.676041	5.935327	5.531895	5.675675	5.08559	5.723938	5.279845	5.582726	5.255355	5.65771	5.602939
Trmt12	-0.281337	2.765533	-1.198906	0.249755	0.420163	-6.19446	3.100876	2.595246	3.063656	3.443962	3.075998	3.235263	2.227194	2.795149	2.653634	2.239124	2.81321	2.153355	2.933966	2.622863
Psmd3	0.144107	7.499901	1.198817	0.249789	0.420163	-6.525751	7.569933	7.579503	7.871359	7.512146	7.724942	7.567227	7.348251	7.31716	7.41709	7.384287	7.523305	7.348855	7.471996	7.371238
Pcbp4	0.222358	7.750232	1.198738	0.249818	0.420163	-6.519962	8.092933	8.267422	8.003739	7.473615	8.043916	7.710379	7.530247	7.433073	7.582232	7.863387	7.591589	7.73453	7.483845	7.69972
Pglyrp1	1.072743	-0.111347	1.198543	0.249892	0.420181	-5.341817	-0.518419	0.369671	1.220561	-1.075778	0.770579	-1.612049	0.743216	-0.81357	0.17081	0.981432	0.676952	0.151783	-0.628995	-0.03329
Gnb2	0.169241	7.262239	1.198525	0.249899	0.420181	-6.532014	7.36269	7.572867	7.326429	6.97009	7.307748	7.134952	7.218693	7.193734	7.276018	7.332395	7.383108	7.207077	7.201355	7.194458
Eif3c	0.233017	7.650215	1.197905	0.250133	0.420458	-6.522078	7.547213	7.665682	8.081459	7.912185	8.029081	8.07463	7.182128	7.46269	7.888358	7.395847	7.489312	7.541678	7.620119	7.22055
Mfsd2a	0.44461	4.78188	1.197903	0.250133	0.420458	-6.435534	4.968344	5.724656	5.407332	4.159289	4.886257	3.984893	4.881086	4.342165	4.983948	4.818339	4.216089	4.603816	4.869129	5.161159
RGD1305743	-0.199359	4.128482	-1.197626	0.250238	0.420575	-6.354143	4.105711	3.953545	3.719973	4.098255	3.948045	4.005051	4.579502	3.979018	4.222559	4.31582	4.254248	4.405307	4.094051	4.208569
Lactb2	-0.218215	4.378523	-1.197156	0.250416	0.420814	-6.442279	4.674093	4.208526	4.725329	4.943642	4.622454	4.80033	4.403445	4.17729	4.15605	3.935342	4.180861	4.145134	4.47088	3.932632
Tsen54	0.357207	4.17778	1.196668	0.2506	0.421066	-6.272624	3.695089	4.47624	3.733206	3.218762	3.785203	3.262313	4.84543	4.393186	4.809475	4.751052	4.184818	4.31219	4.318455	4.797905
Rab3d	-0.188124	4.80414	-1.195775	0.250938	0.421574	-6.457345	4.94325	4.472889	4.911454	4.941472	4.827017	4.793392	4.57555	4.986371	4.936619	4.827276	5.078034	4.718575	4.737483	4.56506
Nexn	0.416173	1.8499	1.195646	0.250986	0.421597	-5.9576	1.9771	1.894965	1.951721	1.962578	2.507694	3.150929	1.888013	1.318383	1.769222	1.51148	1.697158	1.082389	1.377871	2.261799
RGD1561157	0.550383	0.86401	1.195374	0.25109	0.421711	-5.569955	0.739674	1.397635	0.818644	-0.093872	1.018831	0.117189	1.135593	1.596873	0.884903	1.451089	0.961591	1.848377	0.249639	0.887553
Brd7	0.144311	6.780151	1.19518	0.251163	0.421775	-6.537546	6.801877	6.993766	7.045352	7.109999	7.129217	7.232647	6.617678	6.582224	6.538955	6.623161	6.621857	6.523918	6.481625	6.634245
Usp49	-0.309602	4.215975	-1.194514	0.251415	0.422089	-6.355283	3.917539	4.421666	4.051108	4.324952	3.403739	3.432728	4.618433	4.584936	4.703686	4.449167	4.298745	3.995035	4.481425	4.429189
Cd46	0.408566	1.467759	1.194501	0.25142	0.422089	-5.771139	0.830061	1.953842	1.775325	1.548999	1.082564	1.627481	1.782265	1.067371	1.914249	1.597576	1.167576	1.515634	1.252133	2.011549
RGD1310769	-0.248631	5.514077	-1.194124	0.251563	0.42227	-6.508641	5.624546	4.841025	5.508281	5.668188	5.668248	5.560782	5.216656	5.3896	5.591793	5.625541	5.462379	5.521398	5.728769	5.824658
Fam163a	-0.334601	5.02921	-1.194002	0.251609	0.422289	-6.492354	5.812263	5.571717	5.026994	5.019674	5.174635	4.548942	4.940672	4.279444	4.925253	4.984678	5.142525	5.123438	5.110168	4.797905
Isca2	0.220855	3.400532	1.193505	0.251798	0.422546	-6.26281	3.417469	3.806886	3.609501	3.425447	3.731543	3.432728	3.51327	3.232189	3.408468	3.588098	3.331578	3.083214	3.203938	2.972849
Mn1	-0.274964	3.791782	-1.192836	0.252052	0.422913	-6.332699	4.172016	3.200527	3.978882	3.761892	3.808949	3.708268	3.734959	3.985077	3.555781	3.530893	3.963146	3.911153	4.208498	3.67978
Tmem101	-0.189947	4.496042	-1.192648	0.252123	0.422974	-6.399684	4.346765	4.275453	4.029817	4.4153	4.220119	4.257812	4.425566	4.572916	4.69032	4.856672	4.546426	4.888187	4.615127	4.875027
RGD1306227	0.414765	0.996912	1.192393	0.25222	0.423077	-5.662086	1.033981	1.480065	1.143363	0.43652	0.422095	1.450626	1.335758	0.76329	1.360416	0.938085	1.068256	1.045835	1.11438	1.158276
Slc4a3	-0.288513	7.271461	-1.192277	0.252264	0.423092	-6.541298	7.046539	7.35217	6.880203	7.022876	6.470802	6.284426	8.01617	7.170656	7.864381	7.578522	7.116201	7.474505	7.559616	7.974323
Mrps10	-0.239495	3.788296	-1.192078	0.252339	0.42316	-6.412897	4.255933	3.972652	4.179109	4.738944	4.371314	4.526803	3.471351	3.749088	3.400282	3.080873	3.603988	3.18757	3.502026	3.11589
Fam83h	0.426476	3.198705	1.191763	0.252459	0.423302	-6.19029	3.31946	4.196386	3.647273	2.677071	3.036313	2.791131	3.554005	2.862532	3.503242	3.167876	2.912	2.656009	2.902922	3.73402
Scyl1	0.230699	6.826084	1.191395	0.252599	0.423477	-6.541119	6.968469	7.234412	6.823076	6.327641	6.882994	6.570729	6.899549	6.910619	6.785939	7.085361	6.77997	6.835013	6.589545	6.88589
Myh14	-0.234239	6.578603	-1.191162	0.252688	0.423567	-6.539505	6.600725	6.698339	5.864543	6.555453	6.539124	6.278641	6.695296	6.659091	6.663029	6.868008	6.460671	6.877885	6.521344	6.835095
Slc4a11	0.466615	4.001838	1.190702	0.252863	0.423801	-6.28547	3.239259	3.899656	4.819324	3.686144	3.665442	3.321383	4.595201	4.244507	4.600281	4.228565	3.858035	3.956168	3.536146	4.479448
Trove2	-0.369587	3.677302	-1.190523	0.252931	0.423856	-6.350895	3.718942	3.724743	4.266652	4.458437	3.122223	3.789543	3.554005	3.818604	3.350165	2.934159	3.574129	3.581545	4.191471	3.524301
Dpysl3	-0.308922	4.117699	-1.190287	0.253021	0.423948	-6.380613	4.17633	3.479652	4.153089	4.357872	3.900209	4.277834	3.850657	4.411301	4.044916	3.832569	4.099353	4.237171	4.825648	4.093415
Sema6c	-0.270203	5.126889	-1.190036	0.253116	0.424048	-6.431732	4.716181	4.919435	4.371924	4.577819	4.380852	3.862069	5.806182	5.387314	5.834394	5.763854	5.323912	5.408965	5.556616	5.917427
Pex14	-0.206825	5.888428	-1.189818	0.2532	0.424107	-6.524431	6.043551	5.3631	6.002085	5.670811	5.608612	5.61294	5.79433	5.85923	6.069375	6.231534	6.048919	6.096917	6.014267	6.049248
Ntn5	-0.603117	0.302138	-1.189759	0.253222	0.424107	-5.481512	0.369528	1.117652	-0.575682	0.105385	-0.954037	-0.646023	0.118118	1.318383	0.683763	1.14286	0.721218	0.804622	0.745852	0.706485
Ate1	-0.423122	3.798023	-1.189645	0.253266	0.424121	-6.36461	3.788223	3.368632	4.201497	4.600004	3.372207	4.149692	3.593621	3.871902	3.889839	2.770817	4.180861	3.71558	4.290742	3.496677
Cpeb1	-0.209429	6.045638	-1.189304	0.253396	0.424253	-6.538419	6.072769	5.953471	6.258488	6.64019	5.888175	6.490784	6.030598	5.800307	5.980196	5.84823	5.923101	5.760108	6.063059	5.953435
Atp6v1h	-0.251734	6.619941	-1.189252	0.253415	0.424253	-6.542681	6.427881	5.831871	6.741389	6.895817	6.416191	6.64968	6.831591	6.70754	6.854954	6.457483	6.717833	6.52972	6.928926	6.7046
Snrnp48	-0.222552	4.829117	-1.188578	0.253672	0.424615	-6.43732	4.369549	4.724685	4.308508	4.958743	4.387176	4.395516	5.406572	5.001397	5.216192	5.09979	4.845355	4.822175	4.926852	5.202356
Zfp13	-0.227726	4.446737	-1.1885	0.253702	0.424615	-6.426571	4.950824	4.554415	4.459457	4.203428	4.105904	4.247696	4.301868	4.666384	4.316905	4.585342	4.81759	4.468815	4.40224	4.24638
Ccna1	-0.316123	2.662926	-1.188357	0.253757	0.424648	-6.063266	2.753611	2.167912	1.809043	2.475838	2.81965	2.422822	2.955303	2.951874	2.894141	3.00938	3.132898	2.668085	2.974345	2.503027

Mfn2	-0.222268	4.85207	-1.188177	0.253825	0.424703	-6.443005	4.794038	4.324616	4.72312	4.643375	4.451953	4.467019	5.122294	5.146336	5.142036	4.602796	5.170732	4.911231	5.492026	4.993408
Ttc25	0.586673	1.804537	1.188042	0.253877	0.42473	-5.827405	2.357438	1.953842	1.143363	0.386344	2.240661	2.244393	2.12275	2.054242	1.326257	1.986005	2.592682	1.848377	2.022549	1.473753
Hcn4	-0.575558	0.65186	-1.187068	0.254249	0.42521	-5.725893	1.178614	0.583632	1.65069	1.405223	-0.516166	1.221719	0.282279	0.222004	0.512061	-0.116374	0.631286	0.760186	1.252133	1.122575
Akap13	0.18138	6.396356	1.187025	0.254265	0.42521	-6.537478	5.950908	5.92064	6.392411	6.176215	6.402209	6.263659	6.686163	6.645764	6.425553	6.447858	6.533851	6.639588	6.660092	6.423022
RGD1307365	0.349705	4.501655	1.187014	0.25427	0.42521	-6.386726	3.788223	5.146735	4.579097	4.364367	3.909025	4.131469	5.012583	4.624294	4.759151	4.687033	4.454361	4.492777	4.334055	4.811983
Cnot1	-0.173682	7.116004	-1.186832	0.254339	0.425267	-6.547417	6.862028	6.80012	7.173033	7.303655	6.774412	6.889225	7.343031	7.231819	7.258577	7.081632	7.210441	7.159754	7.371167	7.176616
Tmem261	-0.242805	4.161694	-1.186533	0.254453	0.425353	-6.399983	4.424983	3.774588	3.81015	4.277563	4.508156	4.333098	4.390007	3.997121	4.096495	4.224061	4.334806	3.921279	4.134703	4.125333
Mpnd	0.2985	6.290036	1.186513	0.254461	0.425353	-6.53498	6.162481	6.792075	6.009355	5.676041	6.217601	5.848717	6.704372	6.189523	6.601879	6.639321	6.188484	6.245346	6.093166	6.712876
Slc13a4	-1.253922	3.075717	-1.186109	0.254616	0.425552	-6.318544	6.60713	2.151237	3.131857	2.045748	2.460496	4.185462	1.836107	1.958936	2.364852	4.046664	5.478587	2.390473	1.724133	2.932182
Med16	0.225782	6.265868	1.185782	0.254741	0.425702	-6.537525	6.333501	6.281153	6.514312	5.656328	6.244843	5.831887	6.422504	6.241073	6.410347	6.373541	6.223529	6.355857	6.314752	6.539281
Rasal1	0.296436	7.326224	1.18568	0.25478	0.425708	-6.547488	6.851951	7.292118	7.247548	6.393423	6.902991	6.23151	8.032117	7.544471	7.87032	7.773768	7.48651	7.396358	7.574468	7.980257
Htatip2	-0.370026	2.903928	-1.185157	0.25498	0.425945	-6.189538	3.271877	2.134368	2.498042	3.145235	3.00376	3.68846	3.060831	2.753156	2.94004	2.977621	2.986432	2.990087	2.974345	2.446395
Oacyl	0.440004	0.56793	1.185125	0.254992	0.425945	-5.53324	0.369528	0.812964	0.25957	-0.093872	0.320024	0.809818	0.903114	0.301855	0.787836	1.14286	0.805871	0.969826	0.546019	1.085968
Rsph9	-0.486394	2.657257	-1.184998	0.255041	0.425967	-5.985874	3.180343	2.082541	2.067688	1.502648	1.72577	1.402804	3.105363	2.862532	3.271566	3.327565	3.633241	2.827322	3.03287	3.468514
Crabp2	0.843141	1.467508	1.18487	0.25509	0.425989	-5.897468	3.263791	1.914859	2.412888	-0.02435	1.578815	3.340548	0.952703	0.972957	1.142163	1.652245	1.883989	0.460309	0.182287	1.501815
Ddit4	-0.295669	5.604693	-1.184604	0.255192	0.4261	-6.522252	5.897038	6.126595	5.491507	5.526249	5.187373	4.870133	5.540311	5.672833	5.519942	5.777752	5.667178	5.892092	5.825753	5.504087
Agpat1	-0.214278	8.305722	-1.18434	0.255293	0.426202	-6.529195	8.439189	7.766293	7.890187	8.032274	8.410412	8.025148	8.132695	8.443131	8.364217	8.5235	8.575084	8.607437	8.510493	8.565124
Hspd1	0.296631	6.011259	1.184207	0.255344	0.426202	-6.545963	5.895731	6.0398	6.478091	6.740962	6.859308	7.086111	5.5803	5.609519	5.632548	5.522927	5.765573	5.588061	5.728769	5.655412
Brd3	-0.187379	5.537433	-1.184167	0.25536	0.426202	-6.531704	5.644922	5.471176	5.818726	6.092626	5.539323	5.899138	5.2443	5.407759	5.349718	5.286341	5.410943	5.484316	5.558277	5.350525
Ap2a2	-0.226587	8.367782	-1.183981	0.255431	0.426262	-6.523112	8.465425	7.880717	8.493121	8.471688	8.39657	8.203865	8.392836	8.410575	8.402118	7.993142	8.467126	8.297558	8.865131	8.413881
Mmp2	-0.601531	3.786026	-1.183795	0.255502	0.426322	-6.303411	5.101332	3.01022	2.548818	2.656049	3.660229	3.737479	3.116285	4.732714	3.225976	4.528025	5.323912	4.665351	3.302384	3.531125
Nars2	0.245475	2.702236	1.183605	0.255575	0.426384	-6.1152	2.620642	2.758277	2.837276	2.677071	3.091571	2.881136	3.038038	2.619301	2.223535	2.600561	2.506778	2.738492	2.783013	2.697558
Dhtkd1	-0.174061	5.16161	-1.183388	0.255659	0.426414	-6.494596	5.317901	5.071186	5.191021	5.124787	4.989615	4.930402	5.49291	4.918332	5.211521	5.312036	4.994832	5.125629	5.180889	5.445693
Zscan10	-1.831803	-2.071495	-1.183374	0.255664	0.426414	-5.22165	1.693879	-1.685466	-2.787702	-2.344749	-2.049163	-1.427823	-1.73438	-1.196868	-3.260591	0.413143	2.604547	-1.925331	-2.656794	-3.260591
Srr	-0.237536	6.054551	-1.18289	0.25585	0.426665	-6.545147	6.030504	5.625525	6.178517	6.629444	6.184399	6.382457	6.027708	6.079129	6.032774	5.923782	6.077933	5.718619	6.020307	5.876484
Trem2	0.408337	4.712623	1.182642	0.255945	0.426741	-6.365856	4.516294	4.541677	3.754994	3.275	4.836291	4.017012	3.876582	6.249908	3.924502	6.218007	5.868795	6.465438	4.023644	4.482972
Exosc4	0.20785	3.542287	1.18251	0.255996	0.426741	-6.246638	3.467609	3.459476	3.656563	3.099251	3.523394	3.275651	3.662299	3.668539	3.748669	3.7783	3.858035	3.581545	3.508915	3.43979
Pex6	-0.192294	5.112134	-1.182494	0.256002	0.426741	-6.45977	5.012441	4.830589	4.574203	4.600004	4.608991	4.437656	5.653354	5.331332	5.464094	5.583962	5.27278	5.288978	5.351551	5.607789
Tpd52	-0.16382	5.397509	-1.18231	0.256073	0.426799	-6.521982	5.441326	5.412913	5.390726	5.778076	5.506298	5.483864	5.333578	5.250627	5.375002	5.052302	5.180673	5.312249	5.694433	5.390475
Ercc8	-0.159841	5.109531	-1.182151	0.256134	0.426842	-6.483087	5.019661	5.171753	4.798493	5.078135	4.868287	4.734242	5.521948	5.13548	5.339051	5.03183	5.124097	5.099115	5.318604	5.338905
Pnldc1	0.482799	1.139238	1.182007	0.256189	0.426876	-5.650412	0.739674	1.607774	1.061799	0.485009	1.338932	0.336685	1.938115	1.393205	1.218642	0.699359	1.260496	1.375373	1.436856	1.80183
Flt4	0.392742	1.953314	1.181793	0.256271	0.426953	-5.972724	2.41698	2.354284	2.924461	1.548999	1.895073	2.160607	2.659281	1.198329	2.524011	1.481601	1.068256	1.515634	1.922909	2.105984
Ccdc181	-0.173157	6.325026	-1.181238	0.256485	0.42725	-6.553033	6.626964	6.447452	6.312382	6.870944	6.825946	6.719052	6.005851	6.176342	6.073242	6.18941	6.20903	6.083455	6.127142	5.903032
Zic1	-0.427519	3.322628	-1.181084	0.256544	0.427288	-6.3007	4.543265	2.702143	3.4152	3.010938	3.224646	3.884005	3.280644	3.211662	2.95129	3.676473	3.574129	3.16218	2.725031	3.31881
Elavl1	0.221467	4.571412	1.180948	0.256596	0.427288	-6.43221	4.172016	4.382912	4.402616	4.646043	4.868287	4.889781	4.696922	4.736311	4.441807	4.505931	4.664481	4.715669	4.56643	4.377115
Taf5l	0.3891	1.016473	1.180846	0.256636	0.427288	-5.65566	0.692264	1.248835	1.430088	0.621404	0.952152	0.481842	1.21902	1.067371	1.180909	1.388059	1.507975	1.345607	0.791767	1.085968
Tex264	-0.20185	6.347742	-1.180808	0.25665	0.427288	-6.543291	6.397718	5.766314	6.028266	5.83997	6.078969	5.787187	6.305706	6.627479	6.660461	6.743709	6.599798	6.812749	6.489482	6.750641
LOC100302372	0.263402	2.260046	1.180461	0.256784	0.427452	-5.978867	2.263247	2.167912	2.423812	1.799605	2.399242	2.101918	2.12275	2.505864	2.166756	2.308845	2.288794	2.715403	2.363894	2.341603
Papola	-0.169101	7.07602	-1.180234	0.256872	0.427538	-6.553693	7.064687	6.907034	6.9342	7.440572	7.198724	7.292509	7.231306	6.951425	7.057232	6.945297	7.034686	6.915094	7.008856	7.094344
Chka	0.310082	5.947219	1.180141	0.256907	0.427538	-6.519519	5.460908	6.308669	5.552497	5.327853	5.623419	5.340508	6.630114	6.033077	6.29034	6.495357	6.083243	6.086832	5.725816	6.329234
Thrsp	0.365595	4.736408	1.179897	0.257001	0.427636	-6.470765	5.26014	5.670101	5.315009	4.199801	4.690509	4.66498	4.481542	4.044317	4.864068	4.754179	4.579866	4.613215	4.704856	4.528015
Lrrcc1	0.145603	4.574538	1.179622	0.257107	0.427704	-6.457765	4.573017	4.670131	4.833729	4.72387	4.827017	4.845749	4.696922	4.244507	4.676828	4.433645	4.223802	4.269235	4.529643	4.561731
Pcyt2	0.277356	6.602424	1.179553	0.257134	0.427704	-6.546744	6.245913	6.843581	6.063616	5.900436	6.576266	5.918397	7.067928	6.547838	6.971425	7.023847	6.735475	6.663913	6.700521	7.191769
Actr2	-0.279318	7.124396	-1.179514	0.257149	0.427704	-6.5548	7.172269	6.451714	7.204253	7.336646	6.997469	7.248376	7.145945	7.408483	7.120889	6.729478	7.36273	7.160288	7.517966	6.896498
Fam160a2	-0.203397	5.384554	-1.179135	0.257295	0.427858	-6.511461	5.371558	5.196344	5.486306	5.42736	4.822358	5.275609	5.408795	5.652036	5.362416	5.353869	5.588712	5.433932	5.709462	5.333059
Rps4y2	0.532667	1.211025	1.179045	0.25733	0.427858	-5.791198	1.936985	1.854335	1.004744	0.43652	2.240661	1.606524	0.743216	0.871929	1.579251	1.780488	0.961591	0.760186	0.879431	1.009844
Plaa	0.22885	5.80523	1.178997	0.257348	0.427858	-6.548902	5.847855	6.096426	6.520686	6.460888	6.270725	6.647758	5.478169	5.545351	5.466056	5.06243	5.606521	5.410762	5.522982	5.365875
Foxp1	-0.326163	4.788725	-1.178591	0.257505	0.428059	-6.509349	5.150398	4.445797	5.153781	5.655004	5.146953	5.55669	4.34429	4.81326	4.60385	4.051757	4.739035	4.323687	4.895654	4.221283
Cndp2	0.176564	6.857925	1.178337	0.257603	0.428163	-6.555892	6.686396	6.635285	6.941344	6.542624	7.07345	6.512774	6.740997	7.175964	6.849703	7.060943	7.141872	7.227622	6.795137	6.640569
Fads1	-0.283368	8.088211	-1.177767	0.257823	0.428409	-6.534392	8.871903	8.271285	7.895101	7.814177	8.105856	7.976717	8.018719	7.628654	7.859538	8.124623	8.024484	8.137678	8.263032	8.24905
Hps1	0.359006	4.868514	1.177739	0.257834	0.428409	-6.422906	4.529842	5.214516	4.524328	4.00988	4.777328	3.99299	5.326541	5.25314	5.218522	5.386484	5.128213	5.206513	4.44597	5.202356
Trafd1	0.352196	7.807632	1.177677	0.257858	0.428409	-6.554076	7.342678	8.391965	7.529063	7.104675	7.287184	7.088947	8.334406	8.011821	8.151164	8.366825	7.7178	8.042033	7.620119	8.325623
Dyrk2	-0.428063	5.341263	-1.17722	0.258035	0.428589	-6.520216	5.163495	4.023929	5.142254	6.001814	5.563257	5.882889	5.469678	5.391882	5.213859	5.240244	5.429405	5.27917	5.616844	5.399826
Rnmt	-0.305269	4.552184	-1.177212	0.258038	0.428589	-6.455626	4.406741	3.86939	4.755906	5.087979	4.176973	4.961683	4.633716	4.502842	4.57868	4.365748	4.730939	4.479133	4.752072	4.496982
Tubd1	-0.326374	2.057559	-1.176886	0.258164	0.428689	-6.022382	2.246933	1.679303	1.809043	2.511387	2.014259	2.743931	1.962529	2.099634	1.937064	2.108365	2.031907	1.715048	2.437952	1.889408
Tmem41b	-0.228103	6.072121	-1.176861	0.258173	0.428689	-6.549977	5.999191	5.52556	6.083556	6.447951	6.101257	6.27117	5.905539	5.920591	6.100025	6.072757	5.979999	6.036486	6.210852	6.37831
Fam122a	-0.292249	4.373714	-1.17668	0.258244	0.428689	-6.470817	4.909961	3.899656	4.72312	4.870241	4.941012	4.798021	3.975863	4.193082	4.259232	4.116377	4.661644	4.023513	4.20426	3.73402
Vps28	0.195645	6.756465	1.17661	0.258271	0.428689	-6.558243	6.804666	7.056115	6.861746	6.534728	7.036211	6.505717	6.72146	6.690026	6.742888	6.919759	6.734126	6.701118	6.447071	6.849499
Med26	-0.205781	3.544475	-1.176565	0.258288	0.428689	-6.320571	3.562947	3.519177	3.805981	3.950685	3.306999	3.59047	3.280644	3.395791	3.584966	3.440594	3.644778	3.475597	3.633916	3.56477
Pi4k2a	-0.254875	5.668083	-1.176503	0.258312	0.428689	-6.539335	5.841078	5.150935	6.092997	5.88922	5.443967	5.739856	5.624957	5.774298	5.483597	5.478234	5.86016	5.616382	5.944841	5.443886
Mon1b	-0.191893	4.496895	-1.176203	0.258428	0.428822	-6.438628	4.713215	4.320894	4.529394	4.380478	4.469939	4.079183	4.506663	4.647404	4.347031	4.585342	4.561722	4.549364	4.797792	4.538213
Xrcc1	0.300364	7.719273	1.176001	0.258506	0.428853	-6.556962	7.315557	8.014652	7.348093	7.042753	7.461738	7.088947	8.312018	7.88469	8.120348	8.226095	7.676593	7.789802	7.502517	8.293876
LOC691849	-0.401336	2.23027	-1.175971	0.258518	0.428853	-6.117992	3.136736	1.58312	2.175023	2.546081	3.075998	2.845805	1.962529	2.24812	1.393785	2.202958	2.506778	1.869448	2.203293	1.824228
H2afy	0.151309	7.023005	1.175723	0.258614	0.428953	-6.559269	7.029949	7.106181	6.997259	7.096896	7.388767	7.365662	6.781012	6.982048	6.897742	7.007558	6.933666	6.932093	6.835428	6.979918
Sqle	-0.163777	6.462762	-1.175546	0.258682	0.429007	-6.556866	6.448537	6.061157	6.45365	6.575185	6.532008	6.344989	6.753294	6.116873	6.870593	6.208917	6.103242	6.278216	6.756585	6.99357
Sema3b	-0.403211	3.699815	-1.175359	0.258755	0.429068	-6.225988	3.92781	3.117121	3.042552	2.768056	2.712936	2.818727	3.554005	4.58094	3.716062	4.696819	4.056804	5.121244	3.701625	4.120817
Cpsf6	-0.361186	6.850482	-1.175109	0.258852	0.42917	-6.557679	6.845871	6.480366	7.032475	8.048245	7.070015	7.753616	6.951547	6.524103	6.636268	6.362312	6.493743	6.31708	6.795841	6.609472
Fyn	-0.193818	7.259084	-1.174994	0.258897	0.429185	-6.554898	7.444996	7.057235	7.569893	7.693309	7.339889	7.536048	6.894006	7.333877	7.024318	7.048896	7.182513	7.354938	7.252533	6.905061
RGD1566320	-0.258594	3.551223	-1.174595	0.259051	0.429298	-6.268431	3.782575	3.24835	3.500221	3.211576	3.286847	2.863579	4.005537	3.282262	3.860303	3.896239	3.678847	3.656194	3.866246	3.716166
Gstm4	-0.17067	4.600403	-1.174498	0.259089	0.429298	-6.436129	4.539921	4.350411	4.352047	4.461469	4.390327	4.227248	4.955952	4.806432	5.002995	4.830243	4.612548	4.588013	4.599077	4.754829
Bcat2	0.317513	4.170524	1.174478	0.259097	0.429298	-6.359061	4.331374	4.496185	3.967729	3.302317	4.147475	3.866483	4.633716	4.24955	4.417676	4.453021	4.212217	4.179285	3.866246	4.35421
Pik3r2	-0.140078	7.933569	-1.174449	0.259108	0.429298	-6.547455	8.072249	7.774142	7.915801	7.789051	7.706729	7.769907	8.043248	7.981073	8.030386	8.07983	8.032741	7.892765	7.943643	8.044862
Lekr1	-0.374216	1.56649	-1.173321	0.259545	0.429738	-5.837641	1.644914	1.79116	1.430088	1.571628	0.917619	0.881356	2.305626	1.958936	1.843557	1.51148	1.674823	1.890216	1.700256	1.355706
Mbd3	0.163232	6.45995	1.173251	0.259573	0.429738	-6.559822	6.588638	6.630782	6.364336	6.297413	6.862148	6.565987	6.271918	6.385514	6.403196	6.530438	6.497725	6.480924	6.262719	6.315465
Hmg20b	0.283493	5.043279	1.173182	0.259599	0.429738	-6.481577	5.069216	5.494499	4.629506	4.494412	5.256357	4.876712	5.178086	5.116283	4.925253	5.464933	5.03922	5.225023	4.680656	5.204492
Gpkow	0.160595	5.60829	1.173122	0.259623	0.429738	-6.540581	5.633986	6.048831	5.972634	5.723547	5.701613	5.637684	5.564437	5.563688	5.468016	5.474446	5.51676	5.355829	5.355378	5.531571
Eif3f	0.169439	7.443465	1.173116	0.259625	0.429738	-6.559691	7.488145	7.584558	7.407079	7.073798	7.516847	7.293739	7.562049	7.394323	7.541405	7.667053	7.469174	7.401792	7.314298	7.503333
Nmbr	-0.716517	0.972791	-1.172994	0.259673	0.429738	-5.78689	0.307952	-0.273517	1.386086	2.748327	1.230571	1.107027	0.563361	0.972957	0.683763	1.253084	0.764165	0.714338	1.001683	2.030937
Pnrc1	-0.308154	5.511137	-1.172899	0.259709	0.429738	-6.549436	5.777191	4.909553	5.991111	6.334273	6.051362	6.030818	5.172864	5.503746	5.122264	5.26018	5.434399	5.430392	5.237977	4.935431
Npas1	-0.255032	4.607919	-1.17288	0.259717	0.429738	-6.391864	4.197708	4.479583	3.805981	4.16674	3.922149	3.547133	5.278733	4.816662	5.109768	5.109591	4.594813	5.016518	5.217096	5.319327
Ppp2r3a	-0.228208	4.486063	-1.172866	0.259722	0.429738	-6.46887	4.499177	4.882026	4.652905	5.091898	4.180618	4.592225	4.292269	4.155962	4.401362	4.264103	4.295089	4.416088	4.634155	4.517745
Gpr116	0.270806	5.925773	1.172843	0.259731	0.429738	-6.550712	6.086587	6.679565	6.06012	5.768314	5.932087	6.119475	6.124195	5.698876	6.243062	5.719741	5.113756	5.822216	5.701967	5.917427
Ptms	-0.208696	10.16645	-1.172641	0.259809	0.429809	-6.451916	10.74565	10.21761	10.55774	10.80683	10.64968	10.84964	9.62926	10.0099	9.969079	9.883488	10.063	9.756442	9.641163	9.552192
Ntmt1	0.256006	3.799265	1.172511	0.25986	0.429834	-6.292528	3.633671	3.982112	3.838998	3.254167	3.789984	3.165332	3.863678	3.942114	4.018418	4.154751	4.136602	3.985416	3.866246	3.673625
Mphosph10	0.242742	5.211933	1.171465	0.260267	0.430447	-6.535271	5.156961	5.366716	5.794734	5.868808	5.711726	6.160494	4.743453	4.9924	4.864068	5.057375	4.765698	4.911231	4.9009	4.716427
LOC100912292	-0.367836	5.73332	-1.170258	0.260737	0.431165	-6.539572	6.180847	6.084399	4.97038	5.334484	5.750257	4.767664	5.94895	5.591758	6.039378	6.169636	5.736214	5.876565	5.834006	6.013146
Canx	-0.275591	9.643463	-1.169776	0.260925	0.431416	-6.48723	9.748507	9.090542	9.669641	10.16923	9.863356	10.11153	9.387268	9.406549	9.571084	9.336136	9.38598	9.665063	9.759795	9.845793
Rassf9	-0.397703	1.899512	-1.169306	0.261108	0.431609	-6.014202	2.987615	2.046927	1.408255	1.700419	1.947363	2.323579	2.247205	1.658838	2.00342	1.852296	1.290185	1.989793	1.724133	1.846283
Brd4	-0.207039	8.126988	-1.169293	0.261113	0.431609	-6.535797	8.229712	8.328258	8.39124	8.96265	8.190549	8.637459	7.813001	8.046281	7.878078	7.831169	7.983898	7.874316	7.866628	7.750308
Slc25a35	-0.230795	2.677576	-1.168822	0.261297	0.431779	-6.191691	3.045357	3.091129	3.049621	3.107017	2.619344	2.644629	2.613764	2.795149	2.087398	2.600561	2.428842	2.643831	2.423441	2.584013
Tm2d1	-0.279575	5.202766	-1.168771	0.261317	0.431779	-6.516422	5.227254	4.386479	4.917262	5.42891	5.377234	5.505251	5.103206	5.010338	5.22317	5.439902	5.27092	5.353961	5.266712	5.371589
Aldoc	-0.314793	9.836415	-1.168701	0.261344	0.431779	-6.481034	10.58164	10.38846	9.863311	9.752553	9.930991	9.231896	9.810188	9.325585	9.912643	9.738636	9.54133	9.742233	9.843501	10.0486
Zfp94	0.188903	4.193804	1.16861	0.26138	0.431779	-6.377852	4.040949	4.256089	4.257522	3.805502	3.808949	4.105563	4.3855	4.299035	4.281688	4.456866	4.298745	4.482556	4.195746	4.125333
Dnajb1	-0.238001	5.572104	-1.168569	0.261396	0.431779	-6.537901	5.651135	4.866787	5.59419	5.633655	5.642047	5.499579	5.484505	5.646311	5.525598	5.569831	5.983411	5.630336	5.882553	5.432996
Kctd3	-0.179587	6.77346	-1.168378	0.26147	0.431843	-6.568394	6.70144	6.884511	6.844066	6.996986	6.360234	6.591542	6.875644	6.924875	6.907883	6.778685	6.782583	6.772767	6.763083	6.658599
Trmt13	0.335695	2.957745	1.168034	0.261605	0.432005	-6.172572	2.529164	3.801553	3.381979	3.075697	2.732935	2.714852	3.231264	2.80888	3.188437	2.707689	2.673757	2.827322	2.871195	3.069767
Mlx	0.137072	5.961139	1.166983	0.262016	0.432625	-6.55962	5.995534	6.047705	6.234648	6.088709	6.29699	6.136937	5.932051	5.789959	5.876312	5.768502	5.955889	5.699625	5.847656	5.810704
Lman2	0.213618	6.102279	1.166554	0.262183	0.432842	-6.550523	6.037635	5.677398	5.761753	5.4149	6.167966	6.136023	6.182515	6.249908	6.053802	6.324939	6.435982	6.501316	6.192784	6.318427
Hp	0.537212	3.21479	1.16641	0.26224	0.432876	-6.096633	2.607924	3.934181	3.171281	2.184345	2.60856	2.025031	4.05746	3.340143	3.81791	3.90752	3.394437	3.346294	3.061266	3.739923
Hdac2	-0.247292	6.843847	-1.165829	0.262467	0.433129	-6.571613	6.650412	6.346698	6.657115	7.349389	6.77622	7.229652	6.852153	6.910619	6.931508	6.948031	6.903969	6.744533	6.773847	6.753562
Ict1	0.21742	4.818376	1.165739	0.262503	0.433129	-6.481526	4.970829	5.148837	4.696344	4.449301	5.210736	4.641992	4.909618	4.81326	4.827903	4.910928	4.752428	4.775496	4.671475	4.734278
Kcnk15	0.329659	3.343704	1.165707	0.262515	0.433129	-6.246991	3.453461	4.228535	3.171281	2.853641	3.174343	3.365711	3.669732	3.036005	3.35864	3.493953	3.082885	3.245131	3.32598	3.510555
Cisd1	-0.173411	5.613329	-1.16565	0.262538	0.433129	-6.535947	5.626123	5.331995	5.354078	5.435094	5.500476	5.238629	5.919635	5.571764	5.851008	5.722937	5.986815	5.775539	5.783768	5.521326
Atp13a3	-0.61542	0.228239	-1.165486	0.262602	0.433135	-5.542178	-0.518419	0.24948	0.307539	1.327578	-0.516166	-0.548781	0.625837	1.155984	0.245823	0.134646	0.484915	0.403657	1.001683	0.257494
Klhdc10	-0.268902	4.374	-1.165368	0.262648	0.433135	-6.45192	4.516294	4.421666	4.574203	4.643375	3.859855	4.392459	4.498338	4.490115	4.397255	3.790537	4.424552	4.200226	4.895654	4.208569
Ptgds	-0.93543	8.220661	-1.165364	0.262649	0.433135	-6.54203	10.36183	7.134559	8.252998	7.127287	6.543378	9.367023	7.378051	8.019591	7.692769	8.784364	9.157651	8.411933	7.854509	9.009884
Dhx34	0.219642	3.835745	1.164493	0.262991	0.433639	-6.3216	3.736577	3.986819	3.510504	3.381296	4.070978	3.541623	3.742028	4.182573	3.854323	4.168882	3.999546	3.99982	3.800389	3.836741
Slc39a6	0.261435	6.23789	1.164345	0.263049	0.433676	-6.565708	5.877301	5.864205	6.590209	6.429487	6.393597	6.811805	5.965653	6.12787	6.249911	6.056381	6.076869	6.314201	6.17557	6.418428
Cck	0.261109	7.832886	1.164147	0.263126	0.433744	-6.559005	8.20749	7.959451	8.038355	7.37936	8.327965	7.900477	7.367805	7.51807	8.013378	7.708758	7.865013	7.585314	7.831682	7.964273
Laptm4a	-0.204326	8.185395	-1.163693	0.263305	0.433978	-6.552116	8.512035	7.859381	7.932867	7.997964	8.228683	8.189184	8.101088	8.111788	8.186106	8.22525	8.150473	8.407888	8.154919	8.54335
Unc45a	0.218717	6.379366	1.163177	0.263507	0.434252	-6.561987	6.179773	6.490345	6.086996	5.715949	6.174378	5.909869	6.855416	6.482943	6.68594	6.753901	6.462302	6.538791	6.313769	6.680249
Phf20	-0.354444	6.185397	-1.163058	0.263554	0.43427	-6.572834	6.380061	5.741464	6.766277	7.080224	6.125099	6.868469	5.817937	6.16571	5.883671	5.532066	6.16564	6.015372	6.374435	5.701512
Dpt	-0.749152	0.000485	-1.162355	0.26383	0.434666	-5.586128	0.485316	-0.687936	0.105278	0.785264	0.808768	-0.457681	-1.21312	-0.992527	0.245823	0.348395	1.557647	1.082389	-0.755361	0.442608
Tmed2	0.236865	7.454958	1.161901	0.264009	0.4349	-6.565085	7.529619	7.388639	7.88723	7.762865	7.978413	8.280498	6.836553	7.130215	7.131436	7.234457	7.242591	7.459384	7.302452	7.214204
Dctn6	-0.196281	6.307344	-1.161528	0.264156	0.435083	-6.575863	6.739837	6.230459	6.60288	6.866376	6.847891	6.745654	5.965653	6.090416	6.217664	5.964857	6.232158	5.842338	6.097743	5.879161
Nat9	0.194228	4.343155	1.161108	0.264321	0.435295	-6.437734	4.495729	4.616473	4.226666	4.066741	4.713338	4.333098	4.36733	4.424739	4.160904	4.237532	4.348982	4.277141	4.270617	4.342619
Fmr1	-0.264783	5.207138	-1.160371	0.264612	0.435714	-6.553859	5.352623	5.242337	5.609819	6.208159	5.278638	5.941593	5.083863	5.065713	5.045607	4.549787	4.812484	4.830257	5.045309	4.877707
Cdkl3	-0.176766	4.077894	-1.160245	0.264661	0.435736	-6.444845	4.323616	4.32833	4.408127	4.558123	4.018988	4.313834	4.118484	3.749088	3.952765	3.896239	3.822919	3.869926	4.107729	3.716166
Aste1	-0.466374	0.53205	-1.160031	0.264746	0.435815	-5.558483	0.307952	0.185409	0.105278	0.485009	-0.18071	-0.214525	1.21902	0.871929	1.458292	1.51148	0.961591	0.666985	0.836265	0.383504
Ndufa4l2	0.53722	2.133223	1.159866	0.264811	0.435816	-5.915259	2.555898	2.924502	1.7408	0.664146	2.226624	1.519512	2.415815	2.326577	2.430637	2.543858	2.213317	2.568523	2.133689	1.991897
LOC501038	-0.606498	1.499644	-1.159763	0.264851	0.435816	-5.830934	0.995438	-0.572649	1.220561	2.227735	1.600747	1.875381	1.697535	2.077116	1.690848	1.920699	1.674823	1.568131	1.651284	2.011549
Eif4g2	0.158381	7.376519	1.159736	0.264862	0.435816	-6.566887	7.74059	7.657588	7.869862	7.669249	7.957849	7.970323	7.074943	6.821144	6.926529	7.080386	7.206067	7.087973	7.168558	7.049894
Cxcl17	0.515848	1.230272	1.159661	0.264891	0.435816	-5.807617	1.212615	1.874793	1.65069	1.097434	2.014259	1.107027	0.625837	2.326577	0.628769	1.569444	2.442128	2.805622	-0.211665	-1.040339
Zfp668	0.161134	3.851454	1.159482	0.264962	0.435873	-6.331959	3.646165	3.661386	3.674966	3.556013	3.939464	3.761378	3.969854	3.991112	3.980484	3.92982	4.061116	3.895831	4.009141	3.953102
Med21	0.222031	3.860226	1.159315	0.265028	0.435922	-6.457005	4.467845	4.073446	4.755906	4.361123	4.767666	4.913438	3.593621	3.211662	3.341641	3.204921	3.352837	3.195935	3.508915	3.410483
Chd5	-0.270558	8.547486	-1.159063	0.265127	0.436025	-6.547277	8.333549	8.807944	8.467451	8.906736	7.768889	8.34261	8.948536	8.490227	8.908747	8.630762	8.275875	8.401121	8.631423	8.75525

Drd2	0.55826	2.05685	1.158942	0.265175	0.436044	-6.025504	2.645748	3.752647	2.635942	1.454757	2.373993	1.606524	2.05625	1.628188	1.393785	1.652245	1.82436	1.989793	2.151407	2.030937
Mrps21	-0.189248	5.000685	-1.158819	0.265224	0.436064	-6.549835	5.603877	5.31899	5.135625	5.676041	5.623419	5.730201	4.614587	4.58892	4.80328	4.833203	4.567795	4.516347	4.579577	4.468825
Ace	-0.926958	4.7523	-1.158551	0.26533	0.436179	-6.529222	7.547213	5.053348	4.736324	3.706736	4.368121	4.557157	4.272878	3.852146	4.338488	5.169384	6.583404	4.16231	4.1699	4.084165
Mecr	0.340143	3.807518	1.158429	0.265378	0.436198	-6.302368	3.771212	4.192317	3.684081	3.010938	3.917788	3.091837	4.248266	3.865347	4.116621	4.196735	4.008504	3.738666	3.683472	3.896095
Phtf1	-0.167812	6.419206	-1.158192	0.265471	0.436292	-6.573511	6.198981	6.329199	5.953916	6.443357	6.206916	6.128692	6.861108	6.436157	6.768555	6.526786	6.421802	6.445406	6.419003	6.747715
Crot	0.158347	4.974293	1.158049	0.265528	0.436326	-6.507352	4.97331	5.252144	4.915329	4.854179	5.066493	5.057453	5.251748	4.768288	4.919536	4.933176	4.884809	4.877826	4.903517	5.032408
Dcp2	-0.586672	0.764574	-1.157904	0.265585	0.43636	-5.735794	1.108109	0.725555	0.353965	1.273392	0.027932	1.473956	0.282279	1.239467	1.142163	0.646479	0.924208	-0.070959	2.11575	0.442608
Ckap4	0.27021	4.906741	1.157599	0.265706	0.436461	-6.504134	4.637014	4.506054	5.023408	5.044152	5.43941	5.473773	4.722163	4.873301	4.746291	4.924873	4.670138	5.096883	4.662235	4.927694
Vps33a	-0.199276	4.845495	-1.157421	0.265776	0.436461	-6.504497	4.975787	4.459406	5.043022	4.994772	4.786925	4.839026	4.704179	4.961999	4.870008	4.623466	5.006058	4.862143	5.094225	4.670808
Cnpy3	0.198982	6.767326	1.15739	0.265788	0.436461	-6.579315	6.817842	6.563059	6.617821	6.332618	7.068048	6.844057	6.409244	6.941331	6.603663	7.034176	6.908163	7.201895	6.679303	6.735949
Arhgef37	-0.253328	4.010549	-1.157381	0.265792	0.436461	-6.317966	3.742407	3.53854	3.203331	3.54462	3.306999	3.524966	4.884284	4.536242	4.508062	4.453021	4.188764	4.475702	4.229503	4.116286
Ovol2	-0.691008	-0.718286	-1.157284	0.26583	0.436465	-5.660111	1.143792	1.79116	0.307539	0.664146	-1.235601	0.336685	-1.009457	-2.076466	-0.773548	-0.210791	-1.500575	-1.660823	-1.638202	-1.040339
Hltf	-0.292131	5.253619	-1.157032	0.26593	0.436568	-6.547363	5.214725	4.804167	5.09178	5.981889	5.510649	5.830758	5.422059	4.840254	5.084445	4.965917	5.168735	5.183562	5.330318	5.163357
Foxj2	0.178133	6.441129	1.15685	0.266002	0.436627	-6.576257	6.271342	6.699773	6.651318	6.312606	6.217601	6.312197	6.574772	6.303023	6.420502	6.525871	6.374079	6.520592	6.343939	6.666369
Axin1	0.147347	5.634682	1.156652	0.26608	0.436696	-6.553898	5.594237	5.800134	5.896262	5.549235	5.564652	5.61294	5.662696	5.753148	5.442331	5.737232	5.597644	5.595984	5.591107	5.519611
Aloxe3	0.565986	3.691737	1.155995	0.26634	0.437063	-6.304198	3.32724	5.302095	3.883197	3.322471	3.320278	3.136381	3.945565	3.431732	4.034376	3.747244	3.474203	3.941318	2.984265	3.96323
Ebag9	-0.213547	4.914847	-1.154793	0.266817	0.4377	-6.532876	5.143805	4.601207	5.198991	5.48363	5.275232	5.321343	4.57555	4.782275	4.65291	4.677181	4.812484	4.843629	4.9009	4.591416
Chmp1b	-0.230776	4.868585	-1.154789	0.266818	0.4377	-6.544084	5.421475	4.771807	5.078016	5.546382	5.445483	5.713147	4.3855	4.596856	4.263751	4.546183	4.667312	4.683311	4.627841	4.468825
Taf11	0.17246	4.361636	1.15474	0.266838	0.4377	-6.477895	4.60537	4.926801	4.821391	4.523721	4.490643	4.707427	4.36733	4.078726	4.121609	4.182876	4.03505	4.037544	3.954679	4.287263
Col1a1	0.674727	4.558316	1.15463	0.266882	0.437712	-6.44906	5.348806	3.774588	4.526863	3.075697	5.066493	5.985882	4.248266	4.750611	4.263751	4.81235	4.632967	4.752996	3.608485	5.044382
Cars2	0.266767	4.622782	1.15425	0.267032	0.4379	-6.441985	4.414065	5.042086	4.432669	4.054744	4.345567	4.185462	5.052933	4.515458	4.947896	4.927646	4.741723	4.562362	4.546481	5.015475
Rbm22	0.149037	5.608633	1.153905	0.267169	0.438065	-6.561427	5.563278	5.627033	6.027371	5.758485	5.809137	5.775499	5.542337	5.583794	5.525598	5.420347	5.576715	5.405362	5.453267	5.484884
Rxfp1	-0.586288	2.165595	-1.153592	0.267294	0.438208	-6.125112	2.682606	4.10842	2.135702	2.916866	1.018831	1.839907	2.078759	1.68885	2.107651	1.920699	1.844511	1.869448	2.363894	2.124152
Hpdl	0.364144	1.585151	1.153276	0.267419	0.438354	-5.876547	1.916502	1.972946	1.921212	1.00693	1.96438	1.302125	1.335758	1.393205	1.794428	1.942797	1.719153	1.488652	1.28461	1.684306
Fbln1	-0.387781	4.861933	-1.153094	0.267491	0.438413	-6.496522	5.646478	4.171795	4.201497	4.192519	4.439837	5.250499	4.858498	5.130021	4.765539	5.185692	5.082283	5.208581	4.769388	5.221467
Nmral1	0.312221	3.014073	1.152728	0.267637	0.438561	-6.164056	2.8647	3.339488	2.753168	2.523044	3.446756	2.493017	3.072094	2.926907	3.324439	3.377094	3.220315	3.127617	2.933966	2.992761
Abca1	0.303829	5.874378	1.152683	0.267655	0.438561	-6.5438	5.408698	5.703367	5.54876	5.158812	5.325489	5.764899	5.603771	6.934302	5.438339	6.352027	6.258681	7.405854	5.782347	5.585015
Itga1	-0.475496	2.007332	-1.152463	0.267742	0.438579	-6.025668	2.515609	2.264105	2.238266	2.154676	0.770579	2.174914	2.206902	2.38274	2.639794	1.481601	1.375754	1.848377	2.760099	1.708589
Kirrel	0.40546	2.431908	1.152412	0.267762	0.438579	-6.123036	3.271877	2.874583	3.247038	1.720809	2.308867	2.957603	2.01015	2.077116	2.313467	2.088679	2.149958	2.581351	2.452318	2.294252
Chchd1	0.18527	4.667651	1.152249	0.267827	0.438579	-6.510908	4.938178	4.984419	4.787963	4.807278	5.130085	5.326158	4.656342	4.411301	4.46554	4.357545	4.512193	4.166573	4.294734	4.571694
Sphk2	-0.167426	6.066014	-1.152242	0.26783	0.438579	-6.580006	6.323811	5.78805	6.268369	6.297413	6.372238	6.141497	5.790926	6.027216	5.9664	5.963506	6.011533	6.008265	6.028719	5.959772
Mta1	0.146546	7.278459	1.15214	0.267871	0.438579	-6.583067	7.23565	7.479523	7.45179	7.396097	7.537249	7.379626	7.355759	7.197653	7.234029	7.219826	7.073087	7.048679	6.985586	7.314025
RGD621098	-0.157413	5.013963	-1.152096	0.267888	0.438579	-6.513425	4.799641	4.866787	4.864121	5.206584	4.801203	4.867933	5.201351	4.958923	5.262088	5.036976	5.158711	4.985489	5.233825	5.000801
Ing1	-0.17242	4.707378	-1.152013	0.267921	0.438579	-6.508292	5.126074	4.755351	4.798493	4.773515	4.941012	4.602846	4.828927	4.244507	4.670035	4.556968	4.689763	4.472262	4.746254	4.757741
Tspyl2	-0.149951	8.021565	-1.151827	0.267995	0.438613	-6.559532	8.249296	8.31849	8.341344	8.644404	8.247269	8.275527	7.894563	7.758148	7.915537	7.725187	7.765809	7.508062	7.859234	7.805176
Gtpbp1	-0.155501	5.745573	-1.151777	0.268015	0.438613	-6.574831	5.948388	5.703367	6.048698	6.152957	5.903697	5.924761	5.309988	5.751372	5.527478	5.64589	5.629196	5.662382	5.715431	5.551845
Ptrh2	0.182228	4.002316	1.151013	0.268319	0.439051	-6.462963	4.350588	4.244344	4.605721	4.440107	4.7917	4.657358	3.40164	3.741948	3.533498	3.545408	3.728487	3.698019	3.817137	3.578011
Mmp11	0.409455	1.494112	1.150748	0.268425	0.439162	-5.809772	1.48718	1.679303	1.004744	0.823477	1.705671	1.803538	1.576217	1.883083	1.458292	2.048482	1.605665	1.869448	0.745852	1.80183
Cav1	0.205262	5.817585	1.150598	0.268485	0.439162	-6.578712	6.153757	6.280195	5.934953	5.949857	6.389665	6.421978	5.465413	5.260653	5.577343	5.437958	5.329285	5.895948	5.692921	5.684779
Prkg2	-0.422882	1.885069	-1.150568	0.268496	0.439162	-6.131413	3.22267	2.643736	2.795835	2.337441	1.230571	2.685174	1.697535	0.871929	1.549949	1.355481	1.290185	1.252437	2.097585	1.824228
Vps33b	0.183257	5.055125	1.150318	0.268596	0.439265	-6.506425	4.674093	5.106215	4.893885	4.919588	5.022485	4.913438	5.41323	5.178422	5.317478	5.266765	4.937355	4.916302	4.954871	5.305462
Gorasp1	-0.179372	5.621686	-1.15022	0.268635	0.439269	-6.563369	5.947126	5.506021	5.474535	5.479149	5.768538	5.378091	5.538282	5.561662	5.56458	5.639139	5.832682	5.570476	5.793674	5.681716
Fscn3	0.686317	-0.0457	1.14952	0.268914	0.439666	-5.365329	-1.642648	0.310827	-0.007587	-0.02435	-0.257548	-0.862697	0.68572	0.222004	0.091681	1.104165	0.484915	0.218993	0.037347	0.799858
Casc3	-0.199051	6.119367	-1.149138	0.269067	0.439815	-6.587259	6.26528	6.171732	6.467572	6.86294	6.189835	6.633584	5.862403	6.076293	5.806789	5.800621	5.803383	5.836999	6.00577	5.910902
Smtnl2	0.524095	0.293368	1.147574	0.269692	0.440817	-5.469853	0.031429	-0.366503	-0.263851	-0.705589	0.422095	0.695385	-0.169545	1.239467	0.385075	0.893396	0.998029	1.345607	0.313987	0.65743
Myh8	0.815026	-1.027029	1.147472	0.269733	0.440824	-5.552671	-0.217457	1.248835	0.642822	0.105385	-0.831496	1.927009	-1.21312	-1.720374	-1.304935	-2.176425	-0.991788	-1.660823	-1.413173	-2.653844
Tbc1d5	-0.191844	5.508126	-1.146994	0.269924	0.441076	-6.573219	5.824683	5.354021	5.793682	5.885838	5.664348	5.836394	5.335916	5.460906	5.255296	5.074991	5.311298	5.523062	5.627961	5.200217
Vom1r90	-0.423025	0.793498	-1.146544	0.270104	0.44131	-5.75178	1.568201	1.117652	0.56602	0.74601	0.470544	0.809818	0.282279	0.871929	0.787836	0.750369	0.432628	0.714338	1.493524	1.415936
Sptssa	-0.201701	4.82861	-1.146164	0.270256	0.441499	-6.53954	5.369676	4.902098	4.802683	5.166265	5.26325	5.293749	4.858498	4.269548	4.429792	4.623466	4.770972	4.568818	4.707853	4.63007
Pik3r6	0.514108	1.676128	1.145809	0.270398	0.441671	-5.768257	1.341131	2.046927	1.451597	0.531921	1.285769	0.915838	1.888013	2.454377	2.185932	2.308845	2.066633	2.680061	0.879431	1.971974
Usp13	-0.322064	4.755184	-1.145677	0.270451	0.441698	-6.52228	4.556564	4.499482	5.012595	5.605147	4.216572	5.32776	4.739927	4.58892	4.840059	4.4607	4.588853	4.600669	4.86645	4.728352
Pfdn6	0.193757	5.434686	1.145397	0.270563	0.441821	-6.562746	5.635554	5.713341	5.726897	5.346016	5.846331	5.443072	5.198785	5.116283	5.422258	5.314157	5.635724	5.087922	5.433489	5.202356
Npb	-0.399648	1.633808	-1.144903	0.270761	0.442034	-5.928191	2.162475	0.679782	1.905712	1.593908	1.643636	1.563674	1.21902	1.429209	1.717448	1.51148	2.049375	1.950783	2.186205	1.779078
Crlf3	-0.199967	4.176721	-1.144802	0.270802	0.442034	-6.423959	4.050381	4.163505	3.95649	4.284429	4.051203	3.732651	4.667523	4.239446	4.338488	4.328465	4.327666	4.170823	4.107729	4.14326
Aqp4	0.254903	9.947358	1.144795	0.270804	0.442034	-6.50526	10.27637	10.17706	10.19671	9.815083	10.32915	10.50832	9.312226	10.02588	9.095095	9.862268	9.803752	10.47897	9.71656	9.667202
Rap2c	-0.253834	5.574438	-1.144699	0.270843	0.442037	-6.587368	6.023338	5.588845	5.915737	6.546913	6.106057	6.51629	5.497094	4.870031	5.024458	5.026667	5.194477	5.196126	5.398676	5.172115
Repin1	0.260585	5.521742	1.144481	0.270931	0.442065	-6.552825	5.506163	6.126595	5.62528	5.078135	5.408657	5.047769	5.634485	5.443407	5.772315	5.627248	5.594673	5.327557	5.524682	5.622242
Kdm1a	0.136978	6.334641	1.144465	0.270937	0.442065	-6.58791	6.135041	6.440607	6.257725	6.214464	6.383352	6.166771	6.589543	6.379776	6.456478	6.31014	6.376676	6.220961	6.407989	6.365003
Eapp	-0.152268	5.159792	-1.144344	0.270986	0.442065	-6.551008	5.247895	5.046602	5.267291	5.521898	5.275232	5.34526	5.175477	5.121793	5.163961	4.797268	5.02825	5.018877	5.191633	5.07972
Tie1	0.371092	5.288918	1.14429	0.271007	0.442065	-6.519232	5.499292	5.444051	5.028784	4.140492	5.400866	4.839026	5.651478	5.336081	5.677274	5.827594	5.115831	5.502977	4.982355	5.641289
Mier3	-0.24944	4.524997	-1.143292	0.271408	0.442616	-6.520647	4.589284	4.313419	4.886008	5.419585	4.713338	5.119791	4.447353	4.323155	4.303799	4.12123	4.21995	4.220868	4.357143	4.384671
Sdhaf2	-0.182976	6.004981	-1.143265	0.271419	0.442616	-6.591953	6.541762	5.946239	6.233873	6.316798	6.487085	6.315429	5.584238	5.857579	5.665368	5.729308	6.003714	5.733061	5.986156	5.69393
Lmo7	-0.233691	7.299795	-1.142473	0.271737	0.443075	-6.597556	6.930665	6.63003	7.334772	7.300695	6.560265	7.003352	7.768693	7.666382	7.82975	7.514026	7.356166	7.329027	7.543771	7.439931
Sh3bgrl	-0.313022	6.782585	-1.142211	0.271842	0.443148	-6.590172	7.668159	6.610339	7.23907	7.715705	7.888498	7.782224	6.30809	5.951868	6.087333	6.103736	6.330987	6.239288	6.616107	6.430341
Rsph6a	-0.349789	0.724355	-1.142178	0.271856	0.443148	-5.772802	1.071521	1.480065	0.883381	1.300739	0.320024	0.809818	0.851758	0.377517	0.787836	0.591587	0.764165	0.218993	0.836265	0.799858
Snapc3	0.205051	4.884296	1.14136	0.272185	0.443625	-6.544442	4.85451	5.155123	5.390726	5.413335	5.226692	5.594433	4.8055	4.655026	4.733316	4.620041	4.609607	4.40891	4.318455	4.649015
Rps6kb1	-0.17685	5.428687	-1.140759	0.272427	0.443959	-6.578459	5.566568	5.464442	5.706915	6.046925	5.4917	5.862041	5.484505	5.214976	5.190313	5.131403	5.231293	5.081164	5.286892	5.279355
Neurl1b	-0.402318	2.725106	-1.140571	0.272503	0.444023	-6.172515	3.350332	2.383133	2.992072	2.475838	2.109124	2.446602	2.48486	2.68033	2.707706	2.586593	2.455292	3.10114	3.766298	2.858134
Tnfrsf11a	0.207855	4.722718	1.140424	0.272562	0.444059	-6.522574	4.70428	5.5207	4.796393	4.988479	4.987536	4.942996	4.306644	4.658822	4.433808	4.700066	4.480351	4.792144	4.314529	4.551699
Chpf	-0.19052	7.022253	-1.139751	0.272833	0.444441	-6.59784	6.787144	6.174829	6.558347	6.351538	6.627244	6.151479	7.455349	7.287433	7.525499	7.446028	7.365348	7.579332	7.389946	7.625139
Son	-0.214396	8.248967	-1.139648	0.272875	0.444448	-6.581169	7.883976	8.242793	8.048316	8.479749	7.602225	7.968271	8.573092	8.5119	8.574582	8.117051	8.226823	8.325941	8.527554	8.408564
Fbn1	-0.344562	5.675262	-1.139372	0.272986	0.444569	-6.532526	4.813555	4.864231	5.04656	5.442786	4.319352	4.924064	6.880457	6.338956	6.638879	6.314384	5.954731	5.945159	5.692921	6.313488
RGD1306063	-0.184775	4.541471	-1.13922	0.273048	0.444609	-6.532207	5.071534	4.943845	4.53192	5.060244	5.146953	4.915569	4.3855	4.187837	4.286137	4.285878	4.369987	4.092341	4.349488	4.022545
Cpne2	-0.315568	4.622504	-1.13873	0.273246	0.444871	-6.527782	5.292294	4.40418	5.062374	4.821509	4.949583	4.515605	4.481542	3.818604	4.768722	3.856048	4.942039	3.921279	5.035801	4.912095
Loh12cr1	0.185326	4.925075	1.138566	0.273312	0.444918	-6.539893	5.101332	5.188194	5.342612	4.977929	5.340231	5.020301	4.579502	4.761243	4.919536	4.677181	5.054439	4.526331	4.903517	4.610873
Ubl7	0.171415	7.247241	1.138296	0.273421	0.445036	-6.593305	7.492476	7.565413	7.753189	7.682629	8.079252	7.637353	6.856231	7.092121	6.752567	6.926706	7.191391	6.942073	6.863246	6.637411
Rev1	0.261907	6.162048	1.137687	0.273667	0.445376	-6.585872	5.819176	6.538425	6.128483	5.763408	5.792558	5.75541	6.688909	6.300596	6.508814	6.444957	6.162634	6.109149	5.882553	6.396174
Skiv2l2	-0.255942	5.901158	-1.137265	0.273838	0.445579	-6.595328	5.912636	5.426835	6.173672	6.62877	6.070152	6.388608	5.811232	5.702559	5.848001	5.594469	5.826363	5.698154	5.802112	5.759322
Fbxo46	0.170109	3.83213	1.137195	0.273866	0.445579	-6.367221	3.771212	3.702025	3.906747	3.54462	3.952316	3.718071	3.691803	4.055878	3.811751	3.832569	4.128407	4.009344	3.860871	3.774841
Med23	0.193187	6.003707	1.137052	0.273924	0.445613	-6.574551	5.548378	6.015821	5.679833	5.5248	5.556259	5.67087	6.602223	6.050519	6.532415	6.286573	6.008187	6.083455	6.041841	6.476147
Hdac5	-0.216927	7.378056	-1.136706	0.274064	0.44578	-6.602026	7.486843	7.431995	6.755509	7.177481	7.149819	7.123473	7.791443	7.300856	7.354382	7.748506	7.400652	7.494563	7.406642	7.680282
Lppr3	-0.270289	5.406828	-1.136579	0.274115	0.445804	-6.538019	5.268246	5.328292	4.897808	4.994772	4.625132	4.602846	6.036359	5.541244	5.874836	5.852614	5.215906	5.526382	5.623207	6.347713
Dok6	-0.524768	4.231254	-1.135921	0.274382	0.446144	-6.523114	5.029233	4.46953	4.897808	5.070211	3.231692	5.488167	3.999651	3.878428	3.81791	3.135864	3.838074	3.755742	5.091933	3.604134
Prpf8	-0.158953	8.782473	-1.135878	0.274399	0.446144	-6.569822	8.560489	8.431343	8.806122	8.815917	8.451726	8.428332	9.093406	8.924143	9.117739	8.664191	8.836041	8.813614	8.952683	9.062499
Slc48a1	-0.215712	7.654956	-1.135776	0.27444	0.446146	-6.595375	7.978405	7.613378	7.297398	7.532114	7.922094	7.327349	7.311898	7.813258	7.458087	7.774538	7.734769	8.034182	7.690737	7.689081
Lrp2	-0.415786	2.619561	-1.135693	0.274474	0.446146	-5.993508	1.48718	1.702379	1.981598	2.687469	1.533923	1.606524	3.593621	3.475434	3.548392	2.889346	2.346454	2.761217	3.495104	3.869418
Ppt2	-0.250216	5.758133	-1.135574	0.274522	0.446164	-6.56534	5.599065	5.472854	4.802683	5.373643	5.592283	5.089903	6.013174	6.093224	6.064203	6.253802	6.164639	5.970977	5.713941	6.440344
Kdm4c	-0.199143	4.476259	-1.135428	0.274582	0.4462	-6.477107	4.214585	4.354059	4.506456	4.753862	4.035185	4.271191	4.868222	4.600808	4.797058	4.425821	4.387261	4.273193	4.618316	4.633245
Bud13	0.158471	4.541682	1.135017	0.274748	0.44641	-6.513279	4.598957	4.719039	4.905621	4.797712	4.803569	5.000356	4.394501	4.464319	4.393136	4.520698	4.313278	4.269235	4.094051	4.377115
Slirp	0.185597	3.901018	1.134639	0.274901	0.446552	-6.477995	4.323616	4.37216	4.427251	4.412169	4.641095	4.772376	3.609168	3.115498	3.696138	3.64312	3.568082	3.237047	3.349197	3.558103
Myl12b	-0.238381	8.22654	-1.134618	0.27491	0.446552	-6.563405	8.880026	8.267664	8.527548	9.012606	8.999555	9.038981	7.614506	7.99315	7.952047	7.771454	8.096882	7.689328	7.790224	7.543121
Ptgis	0.563598	1.412809	1.134464	0.274972	0.446554	-5.735817	1.619793	0.854759	1.514261	-0.093872	1.578815	0.982423	1.576217	1.596873	1.489496	2.438927	2.592682	1.488652	1.316372	1.473753
Pola1	0.366857	3.051954	1.134357	0.275016	0.446554	-6.279611	3.136736	3.459476	2.635942	3.122426	3.873432	4.056182	2.867532	3.14827	2.720913	2.514649	2.86345	2.726993	3.051863	2.745289
Phc3	-0.393678	4.706158	-1.13431	0.275035	0.446554	-6.541224	4.65258	4.80949	5.262736	5.717218	3.939464	5.177772	4.57555	4.886311	4.663209	3.968034	4.486775	4.401696	4.957391	4.450943
Zfp382	-0.19981	5.038981	-1.134249	0.27506	0.446554	-6.522119	4.69529	4.989118	4.798493	5.086016	4.368121	4.634248	5.450387	5.287872	5.336908	5.337281	5.148616	5.028276	5.268742	5.165551
Dnai1	-0.500668	2.078324	-1.134083	0.275127	0.446603	-5.917391	2.263247	1.854335	1.089503	1.353927	2.124346	0.231108	2.582602	2.619301	2.47808	2.088679	2.616315	2.816513	2.783013	2.635584
Pcdhb4	0.38766	1.928735	1.133918	0.275194	0.446651	-5.870667	1.514695	1.453108	1.613009	1.158502	2.030509	1.708392	1.782265	2.603631	2.797714	1.920699	2.149958	2.447635	1.943396	2.310209
Strap	0.156565	6.635295	1.133765	0.275256	0.446692	-6.607007	6.952209	6.854567	7.241283	6.996986	7.124494	7.305571	6.296133	6.401461	6.200088	6.200917	6.435982	6.248365	6.425389	6.226823
Ccdc62	0.308095	4.737889	1.133559	0.27534	0.446747	-6.488899	4.33909	5.377509	4.360599	4.488478	4.627805	4.562608	5.452543	4.666384	4.989416	4.971302	4.579866	4.53295	4.533027	4.909479
Srsf4	0.151925	6.835628	1.133498	0.275365	0.446747	-6.607474	6.755058	7.152474	6.832329	6.989127	7.081759	7.028682	6.781012	6.779039	6.715108	6.946664	6.764189	6.689316	6.489482	6.708368
Dnajc12	-0.269268	3.616572	-1.132788	0.275653	0.447155	-6.368979	3.782575	2.833367	3.697645	3.781437	3.86892	3.601103	3.647317	3.575215	3.930199	3.48645	3.827988	3.489274	3.822677	3.417866
Gpm6b	-0.208539	9.182809	-1.132237	0.275877	0.447389	-6.549878	9.61007	9.259297	9.170833	9.16489	9.251686	9.039959	9.03525	8.937486	9.124898	9.131887	8.739637	9.371659	9.285781	9.438713
Tecta	-0.287476	2.962724	-1.132215	0.275886	0.447389	-6.234795	3.188908	3.368632	2.645305	3.019194	2.77212	2.537984	2.841442	3.804966	2.446625	3.636356	3.771221	3.482452	1.79348	2.387449
Dph6	0.1899	3.91989	1.132159	0.275909	0.447389	-6.44361	3.854329	4.180039	4.346317	4.318277	4.140005	4.458273	3.90205	3.501034	3.901486	3.64312	3.848089	3.60686	3.725479	3.558103
Clic2	0.418232	1.300726	1.131745	0.276077	0.447502	-5.826846	1.212615	1.047298	1.574317	1.065902	2.030509	1.473956	2.227194	1.279465	1.981639	0.699359	0.631286	0.666985	1.077784	1.910497
Lin37	0.191591	4.512352	1.131743	0.276078	0.447502	-6.501957	4.683216	4.755351	4.71425	4.409031	5.036634	4.431711	4.306644	4.35626	4.469458	4.49849	4.417843	4.479133	4.13915	4.544972
Armc7	0.225116	3.721109	1.131573	0.276147	0.447502	-6.397531	3.689063	4.091039	4.033387	3.866255	3.886882	4.013036	3.981847	3.660989	3.253503	4.010503	3.499841	3.61312	3.134365	3.482664
Tmem196	-0.424054	1.14523	-1.131524	0.276167	0.447502	-5.907082	1.541696	1.953842	1.493674	1.892408	0.562819	1.302125	0.282279	0.972957	0.628769	1.023513	1.068256	0.889587	1.521045	1.634478
Prdm1	0.715176	0.203063	1.131443	0.2762	0.447502	-5.511007	-0.310971	-1.271663	0.750864	-0.243853	0.985877	1.014597	0.028455	1.067371	-0.080923	0.893396	0.764165	1.315214	-0.040977	-0.505482
Sdad1	0.205469	4.234949	1.131432	0.276205	0.447502	-6.439216	3.765497	4.055636	4.432669	4.328278	4.070978	4.467019	4.438678	4.318363	4.469458	4.205901	4.008504	4.17506	4.294734	4.342619
Tdrd3	-0.187324	4.171817	-1.131279	0.276267	0.447502	-6.478998	4.184919	4.320894	4.116527	4.728912	4.223657	4.458273	4.046082	4.155962	3.811751	4.2553	4.136602	3.941318	4.066299	4.046557
Crk	-0.294496	5.459558	-1.131255	0.276277	0.447502	-6.600261	5.88391	5.52556	6.022889	6.523165	5.71801	6.370853	5.006725	5.181064	4.925253	4.609719	5.305858	4.983074	5.564903	4.850685
Ddrgk1	-0.193821	7.161579	-1.131126	0.276329	0.447527	-6.607068	7.338835	7.143018	6.816874	7.465708	7.416855	7.454835	6.896384	7.087903	7.144973	7.274216	6.894939	7.202933	7.043073	7.092615
Hira	-0.166641	4.796637	-1.13088	0.276429	0.447606	-6.492287	4.640141	4.535265	4.405374	4.479531	4.230707	4.401612	5.345229	5.07142	5.056067	5.133806	4.899331	4.803137	5.06414	5.145679
Mat2a	0.231228	5.524778	1.130823	0.276453	0.447606	-6.577479	5.247895	5.879471	6.094706	5.661611	5.192798	5.586428	5.84937	5.31699	5.559075	5.257979	5.257831	5.257228	5.548281	5.653849
Fasn	-0.137583	8.391475	-1.130511	0.27658	0.447752	-6.590839	8.248016	8.128443	7.990024	8.098052	8.072646	7.951229	8.664519	8.487028	8.641224	8.405381	8.531856	8.594049	8.92816	8.74483
Rwdd2b	-0.204582	4.384721	-1.130117	0.276741	0.447951	-6.495637	4.543265	3.958345	4.272706	4.646043	4.664713	4.657358	4.362751	4.219024	4.342766	4.527698	4.345451	4.212646	4.420622	4.295302
Mmp23	-0.403234	2.329113	-1.129944	0.276811	0.448005	-5.87134	1.459129	0.583632	1.65069	1.679737	1.173177	1.249022	3.049479	3.368235	3.207329	3.649853	2.958968	3.42668	2.549064	3.002614
Slc26a4	0.382384	2.159875	1.129804	0.276868	0.448037	-5.91558	1.541696	2.082541	1.792283	1.454757	1.96438	1.668504	3.094359	2.099634	2.984524	2.543858	2.317912	2.082939	2.041677	2.942457
Mrpl27	0.171206	5.332789	1.129708	0.276907	0.44804	-6.589222	5.859974	6.061157	5.596606	5.491069	6.023216	5.702076	4.994938	4.989389	5.07419	5.042103	5.063063	4.948838	4.974911	4.877707
Alkbh7	0.261099	3.623001	1.129518	0.276985	0.448106	-6.34384	3.907194	3.948728	3.711083	3.019194	3.711525	3.456346	3.699086	3.542716	3.842287	3.753509	3.822919	3.323571	3.508915	3.604134
Il10ra	0.354966	2.386633	1.129275	0.277084	0.448205	-6.079868	2.311111	2.046927	2.558762	2.077719	2.79132	2.714852	1.443753	2.976416	1.937064	2.720538	2.729	3.656194	2.133689	1.634478
Pbdc1	0.213588	4.94857	1.12872	0.277311	0.448512	-6.583995	5.317901	5.523942	5.645251	5.753545	5.600471	6.234928	4.171806	4.70728	4.363967	4.472141	4.489977	4.426789	4.453131	4.174106
RGD1565059	-0.206316	3.226081	-1.128491	0.277404	0.448602	-6.286644	3.136736	3.279374	3.184186	3.474303	2.919011	3.061358	3.392685	3.395791	3.244385	3.51623	3.091342	3.228918	3.051863	3.357718
Rbm45	-0.240685	4.836485	-1.12793	0.277633	0.448913	-6.51809	4.713215	4.21255	4.210987	4.870241	5.122525	4.667512	5.31947	4.905609	4.989416	4.933176	4.962928	4.805872	5.031023	5.022756
Map3k4	-0.195159	6.086858	-1.12758	0.277776	0.449084	-6.60447	5.913928	5.804139	6.090428	6.429487	5.734224	6.280296	6.098263	6.336588	6.169105	6.219139	6.048919	6.10249	5.975005	6.037314

Stk35	-0.216113	3.749329	-1.127482	0.277816	0.449088	-6.419656	3.891537	3.780021	3.929918	4.1518	3.422332	4.101824	3.40164	3.904241	3.669138	3.463704	3.73929	3.619352	3.817137	3.716166
Tmem27	-3.440064	-2.955834	-1.127282	0.277898	0.44916	-5.177849	4.54993	-2.267963	-3.260591	-3.260591	-1.798946	-3.260591	-3.260591	-2.550306	-3.260591	1.451089	3.414796	-3.260591	-3.260591	-3.260591
RT1-CE5	0.447998	3.940001	1.12699	0.278018	0.449292	-6.364862	4.082914	3.099845	4.787963	2.960388	3.990197	3.798811	3.060831	4.455617	3.225976	4.650573	4.892088	5.035286	3.45286	3.780579
LOC681766	-0.627635	0.009869	-1.126699	0.278136	0.449424	-5.567975	1.371547	-0.465897	0.049949	-0.600663	0.091273	-0.750296	0.563361	0.449407	0.007959	-0.027758	1.102128	-0.153565	0.249639	-0.116166
Mapk13	1.685319	-1.259387	1.126574	0.278188	0.449447	-5.29145	1.80948	-0.950476	0.52603	-3.260591	0.470544	-0.750296	-2.558588	-1.435005	-2.604521	0.20947	2.303426	-0.541385	-2.232431	-2.228037
Iars	0.161348	6.338736	1.126274	0.278311	0.449542	-6.608834	5.930623	6.237388	6.512395	6.490879	6.398301	6.343407	6.552831	6.267417	6.452525	6.210057	6.240737	6.246353	6.378202	6.500691
Csnk1a1	-0.276891	6.534307	-1.126247	0.278322	0.449542	-6.615614	6.79488	6.094247	6.92654	7.06036	6.635235	6.945593	6.368716	6.517847	6.41949	5.8929	6.546208	6.270316	6.82925	6.196023
Derl3	0.212884	3.976617	1.126145	0.278363	0.449549	-6.390857	3.816137	4.267739	3.986269	3.600707	3.939464	3.491064	4.123906	4.020912	4.007682	4.19213	3.944596	4.078836	4.125768	4.178459
Sema3d	0.315511	3.367246	1.126033	0.278409	0.449563	-6.402621	3.595524	4.244344	3.967729	3.897939	3.500244	4.565325	2.361772	3.036005	2.759824	2.758411	2.90242	3.52291	3.333761	2.858134
Gkap1	0.16563	5.241001	1.125657	0.278563	0.449751	-6.578664	5.365903	5.71476	5.342612	5.424255	5.556259	5.636392	5.024227	5.059983	5.002995	5.364141	5.099154	4.880423	4.842108	5.102807
Hexb	-0.278391	7.196313	-1.125247	0.278731	0.449961	-6.616788	7.109928	6.956461	6.011167	6.791238	6.778627	6.945071	7.200206	7.888736	6.908605	7.772226	7.629101	8.276514	7.097786	7.394345
Pex10	-0.253404	3.395152	-1.124818	0.278907	0.450185	-6.289507	3.365524	2.619695	3.247038	3.281878	3.416161	2.957603	3.699086	3.272386	3.341641	3.530893	3.760656	3.644018	3.844626	3.704139
Lrrc36	-0.504937	1.884263	-1.124204	0.279159	0.450531	-5.833331	1.644914	0.973336	0.20995	1.188094	1.463853	1.107027	2.930765	2.76729	2.381582	1.652245	2.014226	2.703718	2.689088	3.169358
Orc1	0.321377	1.809717	1.122913	0.279689	0.451326	-5.933173	1.245833	1.833582	1.451597	1.856	2.335271	1.943815	2.186319	1.775313	2.00342	1.964563	1.557647	1.691577	2.097585	1.846283
Cetn4	0.320953	1.938086	1.12275	0.279756	0.451338	-6.181471	2.620642	2.324846	2.626517	2.62393	3.326873	3.456346	1.091998	1.393205	1.102347	1.322149	1.533025	1.186803	1.626159	1.355706
Plekhm2	0.222042	7.341466	1.122712	0.279772	0.451338	-6.617124	7.45124	7.835769	7.49451	6.910315	7.236426	7.043864	7.445098	7.220948	7.262534	7.445061	7.193847	7.437505	7.367855	7.44536
Ncaph2	0.162048	6.093364	1.122183	0.27999	0.451629	-6.60591	6.07739	6.332901	6.107465	5.726071	5.8691	5.941593	6.199304	6.081959	6.225794	6.258215	6.050004	6.087956	6.166885	6.205652
Suclg2	0.269666	6.076716	1.121783	0.280154	0.451833	-6.607735	6.484641	6.517434	6.200121	5.36719	6.119413	5.928987	5.900809	5.968785	5.941229	6.224787	6.042391	6.331388	5.904922	6.165647
Wnt10a	0.360122	2.119715	1.121636	0.280214	0.451857	-5.959253	2.091157	2.100024	1.574317	1.21709	2.046579	2.244393	1.668148	2.926907	2.066858	2.628098	2.259075	2.838051	2.269694	2.124152
Uqcrq	0.190199	6.131927	1.121565	0.280244	0.451857	-6.61453	6.426071	6.405005	6.170433	6.039842	6.671308	6.442819	6.158941	5.779538	6.03674	6.06648	6.092751	5.654816	5.885203	6.039709
Wdr73	-0.171188	4.275011	-1.121132	0.280422	0.452084	-6.455731	4.421353	3.790827	4.214137	3.984807	4.031153	4.044542	4.421169	4.351577	4.523219	4.369831	4.460902	4.365074	4.477919	4.475916
Slc25a40	-0.266426	2.252567	-1.120632	0.280628	0.452355	-6.095352	2.10932	2.383133	2.135702	2.687469	1.822268	2.28453	2.746221	2.436799	2.508863	1.964563	2.014226	1.869448	2.535634	2.372328
Emc2	-0.205634	6.360292	-1.119219	0.281211	0.453233	-6.6235	6.81577	6.464425	6.695367	7.276793	6.984494	7.217609	5.986636	5.819086	5.973314	5.929327	5.952411	5.839671	6.164705	5.944517
Arih2	-0.130638	6.167159	-1.119058	0.281277	0.45328	-6.617417	6.226284	6.083301	6.304278	6.438749	6.123206	6.297561	6.095506	6.090416	6.075814	6.077758	6.215814	6.084581	6.170148	6.078658
Fbxl12	0.22571	3.121429	1.117841	0.28178	0.453997	-6.233112	2.957854	3.339488	3.171281	2.768056	3.091571	2.743931	2.930765	3.292072	3.35864	3.070874	3.408041	3.30822	3.186856	3.25429
Unc5b	-0.281999	4.986367	-1.117798	0.281798	0.453997	-6.55134	5.212627	4.57956	5.028784	4.886126	4.979188	4.461194	4.746971	5.181064	4.890608	4.803319	4.840346	5.677395	5.355378	5.217242
R3hdml	-0.737413	0.089861	-1.117508	0.281918	0.454129	-5.486348	0.692264	-0.103798	0.158563	-0.705589	-1.798946	-0.984601	-0.169545	0.377517	0.837186	0.699359	0.631286	1.315214	1.11438	1.009844
RGD1306001	0.340627	3.096376	1.117172	0.282056	0.454243	-6.231307	2.742017	3.506122	3.359399	2.890108	3.224646	2.559953	3.003159	3.301815	3.424703	3.232094	3.487079	2.951084	3.394539	2.460763
Zfyve26	-0.161955	5.057837	-1.117155	0.282064	0.454243	-6.543883	4.824591	4.931692	4.889952	4.996864	4.553844	4.631657	5.276301	5.309765	5.107255	5.162339	5.229378	5.465412	5.282878	5.195929
Tmem69	-0.314005	4.478885	-1.117045	0.282109	0.454255	-6.565841	5.139392	4.08227	4.798493	5.321191	5.298904	5.470877	3.895725	4.050109	4.029076	3.890566	4.295089	3.946285	4.413298	4.147707
Pdk1	-0.18626	4.82089	-1.116886	0.282175	0.454267	-6.566506	5.101332	4.704825	4.957283	5.280562	5.06057	5.295387	4.791941	4.639742	4.51566	4.487257	4.789281	4.603816	4.855682	4.465266
Mcoln1	-0.162514	6.592673	-1.116843	0.282192	0.454267	-6.620234	6.334466	6.179975	6.236973	6.398969	6.372238	6.028851	6.816603	6.744626	7.111517	6.78405	6.651469	6.732364	6.726611	7.195533
Tubg1	0.136462	5.488515	1.116122	0.282491	0.454687	-6.595211	5.65732	5.778581	5.553741	5.357456	5.630099	5.607677	5.446065	5.421231	5.383333	5.472549	5.364599	5.29872	5.40053	5.502352
Rcbtb1	-0.146581	4.993513	-1.115512	0.282744	0.455033	-6.551041	4.875886	4.984419	4.80059	5.058242	4.817684	4.695072	5.143803	5.025118	5.197417	4.902496	5.008293	5.19195	5.225485	5.032408
Otud5	-0.133137	7.93646	-1.115348	0.282811	0.455081	-6.610503	8.071091	7.947428	7.92808	8.033293	8.003069	7.797908	7.925551	7.850661	7.912302	8.077647	7.942722	7.848682	7.768861	8.009565
Asb8	-0.142923	6.140489	-1.114954	0.282975	0.455283	-6.617737	6.198981	5.967826	5.991111	6.060988	6.18893	5.799936	6.092744	6.36591	6.130036	6.177811	6.379269	6.271306	6.27186	6.092564
Htr1f	-0.571046	0.322854	-1.114793	0.283042	0.455322	-5.614261	-0.518419	0.310827	0.158563	1.454757	-0.831496	0.284862	1.258992	0.222004	0.683763	0.055728	0.321999	0.34469	1.040235	1.085968
Tfdp2	-0.176918	4.186408	-1.114712	0.283075	0.455322	-6.469165	4.28818	4.014741	4.068613	4.203428	3.904623	4.281144	4.394501	4.415795	4.13647	4.150009	4.216089	4.110154	4.456698	4.056051
Ajuba	0.490345	2.398582	1.114541	0.283146	0.455376	-6.039823	2.937667	2.506257	1.669168	0.932396	2.640673	2.665044	2.398025	2.38274	2.55384	2.889346	2.930971	2.475388	2.363894	2.55752
Mir2985	-0.502734	2.53509	-1.114354	0.283224	0.45544	-5.947643	1.514695	2.466372	1.195284	2.13961	0.266146	1.627481	3.677127	3.340143	3.805565	3.214035	3.065821	2.9008	3.186856	3.461387
Prkcq	-0.21817	4.616801	-1.114159	0.283305	0.455502	-6.531433	5.101332	4.625556	4.446125	4.43703	4.675086	4.437656	4.42995	4.647404	4.15605	4.609719	4.892088	4.880423	4.786497	4.575
Bves	-0.467393	1.091198	-1.114045	0.283352	0.455502	-5.696067	0.539902	-0.465897	0.56602	1.033666	1.173177	0.117189	2.01015	0.818632	2.185932	1.355481	1.674823	1.152831	2.097585	1.868007
Sypl1	0.194089	5.177555	1.113987	0.283376	0.455502	-6.559216	4.948303	4.958296	5.176563	4.932758	5.044657	5.349996	5.122294	5.24811	5.310942	5.052302	5.021628	5.390862	5.713941	5.258947
Prkrir	0.158703	5.883883	1.113581	0.283545	0.455712	-6.616844	5.933174	5.879471	6.245463	5.969161	6.196152	6.115772	5.950476	5.611479	5.642993	5.548372	5.821288	5.826263	5.938477	5.722532
Rprd1b	-0.134969	5.086084	-1.113278	0.283671	0.455853	-6.585135	5.362121	5.088807	5.224203	5.392832	5.436364	5.219778	4.851979	4.958923	4.950701	4.888332	4.976688	4.990306	5.038184	4.872343
Inpp5e	0.214517	5.096597	1.113113	0.283739	0.455902	-6.552817	4.96336	5.402383	5.023408	4.733936	5.158089	4.670039	5.419857	5.146336	5.122264	5.307785	5.030451	5.187762	4.913935	5.319327
RGD1308106	-0.133519	4.649494	-1.11287	0.28384	0.455967	-6.523846	4.65258	4.541677	4.503885	4.654018	4.565043	4.472821	4.778253	4.823442	4.628589	4.824303	4.765698	4.698109	4.51603	4.731318
Mob1b	-0.341852	3.768536	-1.112833	0.283855	0.455967	-6.440798	3.827152	3.224637	4.320248	4.37727	3.286847	4.185462	3.810873	3.904241	3.81791	3.223093	3.930525	3.346294	4.052221	3.571405
Rn28s	0.51108	8.910334	1.112542	0.283976	0.4561	-6.606605	8.689267	9.507116	8.368276	7.462685	8.068963	8.403298	7.908374	9.92645	9.74913	9.448522	9.451291	9.411487	9.100529	9.253065
Chst10	0.161217	6.657141	1.112442	0.284018	0.456106	-6.62834	6.55921	6.95706	6.659427	6.450243	6.610451	6.436895	6.608038	6.651491	6.570304	6.84399	6.606451	6.728765	6.683115	6.850181
Elk1	-0.143132	6.242408	-1.112328	0.284065	0.456121	-6.626186	6.415162	6.159277	6.445633	6.431035	6.144827	6.369298	6.132288	6.210364	6.012778	6.18941	6.333662	6.115777	6.314752	6.140208
Zfp287	-0.151422	4.34527	-1.112103	0.284159	0.456211	-6.51243	4.460789	4.535265	4.366273	4.638025	4.342316	4.367762	4.192594	4.303891	4.263751	4.0968	4.287749	4.308338	4.526252	4.221283
Wdr44	-0.218644	5.123781	-1.111848	0.284265	0.456278	-6.568856	5.143805	4.866787	5.267291	5.67212	4.983368	5.64541	5.309988	4.876565	5.173599	4.640468	4.730939	4.908688	5.248305	5.311421
Cadm4	-0.197985	7.245105	-1.11182	0.284277	0.456278	-6.630044	7.412909	7.338886	7.126571	7.12442	6.888576	6.930389	6.981721	7.394892	7.10838	7.516331	7.165593	7.655988	7.386677	7.410626
Ssna1	-0.209666	3.18772	-1.111509	0.284406	0.456371	-6.344597	3.595524	3.142653	3.145118	3.419222	3.196117	3.693437	3.049479	3.252427	3.271566	3.050666	3.212584	2.869767	3.02328	2.879681
Tmem256	0.222789	3.112309	1.111453	0.284429	0.456371	-6.388019	3.664706	4.112733	3.623781	3.533136	3.551819	4.009049	2.979429	2.539194	2.680923	2.783118	2.639567	2.542521	2.236876	2.868948
Ndufa5	-0.196852	5.691306	-1.1112	0.284535	0.456371	-6.602947	5.674195	5.35766	5.230438	5.624215	5.785394	5.438632	6.140335	5.857579	5.89973	5.674236	5.961666	5.448008	5.903616	5.713561
Pias3	0.162253	5.770336	1.111138	0.28456	0.456371	-6.614462	5.836997	6.222499	6.09556	5.753545	5.712985	5.798782	5.899229	5.579795	5.754765	5.672584	5.62557	5.508024	5.676189	5.677109
Zfp865	-0.154189	3.813389	-1.111136	0.284561	0.456371	-6.403554	3.838085	3.763659	3.530855	3.756964	3.563034	3.727807	3.908348	3.997121	3.675936	3.946322	4.073974	3.965983	3.871601	3.880148
Idua	0.359466	7.41473	1.111132	0.284563	0.456371	-6.63106	6.910739	7.872792	7.084599	6.440286	6.791795	6.386305	8.008496	7.787917	7.884695	8.052779	7.447105	7.701129	7.329943	8.117791
Agpat4	-0.183791	6.699047	-1.11096	0.284635	0.456426	-6.625539	6.166823	6.246248	5.909922	6.451768	6.394382	5.957198	6.930818	7.052272	7.062429	7.212455	6.882202	7.165622	7.056653	7.314025
Cep44	0.24492	3.670276	1.110288	0.284914	0.456813	-6.281236	2.947796	3.61957	3.138503	3.137672	3.238702	3.200721	4.489964	3.997121	4.250151	3.968034	3.807603	3.890687	3.772036	4.056051
Spata24	-0.323732	1.175162	-1.109486	0.285249	0.45724	-5.804414	1.371547	1.082904	0.56602	0.966949	1.230571	0.571087	1.135593	1.393205	1.102347	1.625169	1.762161	1.910689	1.218908	1.227132
Iffo1	0.268103	7.210319	1.109467	0.285257	0.45724	-6.633474	6.931304	7.851983	7.115666	6.820466	6.886346	6.718442	7.448883	7.478347	7.305358	7.534635	7.190899	7.358202	6.976296	7.338546
Chmp3	-0.221707	6.482047	-1.109366	0.285299	0.457247	-6.63379	6.638736	6.12979	6.475468	7.01722	6.793582	7.026219	6.312845	6.449367	6.409328	6.242711	6.404939	6.30069	6.404299	6.161255
Ten1	0.331826	2.409821	1.108933	0.285479	0.457475	-6.125072	2.445855	2.383133	2.691232	2.154676	3.217567	2.174914	2.100922	2.077116	2.625819	2.795315	2.468337	2.046399	2.423441	2.431883
Ankrd12	-0.308199	6.493498	-1.108533	0.285646	0.457642	-6.634711	6.335431	6.391757	6.837443	7.421624	6.088702	6.964246	6.497517	6.502086	6.27139	6.028237	6.391305	6.296807	6.657769	6.2425
Abce1	0.173866	6.976834	1.108501	0.28566	0.457642	-6.634686	6.731799	6.851345	7.316572	7.191703	7.248633	7.127616	7.012739	6.847992	6.916519	6.681001	7.010923	6.825609	7.008249	6.917484
Nup155	-0.176521	4.840373	-1.10766	0.286011	0.458144	-6.535116	4.464322	4.462789	4.519244	4.973688	4.478849	4.731825	5.214116	4.924651	5.166377	4.650573	4.911322	4.880423	5.3611	5.082046
Cish	0.380534	0.715349	1.10749	0.286082	0.458197	-5.857052	1.310059	1.082904	2.2133	1.033666	1.20216	1.541762	0.028455	0.645799	-0.175642	0.348395	0.846404	0.930264	-0.405292	0.442608
Pigm	-0.213095	4.325501	-1.107024	0.286277	0.458447	-6.505186	4.45014	3.943895	4.472666	4.485502	4.28928	4.294309	4.085519	4.264575	4.393136	3.935342	4.444493	4.430338	4.713828	4.432837
Ints2	0.232328	5.29828	1.106555	0.286473	0.458701	-6.561312	4.488808	5.254098	5.197401	5.226329	4.93455	5.004367	6.023363	5.456551	5.831353	5.290656	5.362854	5.053812	5.420761	5.672487
Stradb	-0.142182	5.595151	-1.106388	0.286543	0.458752	-6.610442	5.702886	5.659822	5.527397	5.644369	5.620738	5.321343	5.747682	5.55353	5.689082	5.502614	5.693802	5.428618	5.553287	5.719548
Tmem127	-0.156293	6.954347	-1.106114	0.286658	0.458874	-6.637335	6.895774	6.556742	6.64841	6.715151	6.556062	6.835076	6.895592	7.132946	6.863911	7.286149	7.209955	7.382683	7.165837	7.229494
Mrps17	0.17758	4.539043	1.105728	0.286819	0.459072	-6.550621	4.883821	4.771807	4.729737	4.610969	5.223162	4.961683	4.263083	4.234368	4.208563	4.509637	4.515339	4.208518	4.337929	4.156561
Smurf1	0.169486	6.454875	1.105478	0.286924	0.459178	-6.633981	6.322839	6.882619	6.603481	6.387855	6.281798	6.333078	6.492286	6.465711	6.442594	6.526786	6.406634	6.414379	6.297947	6.528238
Fbxl7	-0.312801	1.652189	-1.10532	0.28699	0.459207	-6.048104	2.035255	1.117652	1.841991	2.269859	2.386673	2.00915	1.726334	1.020936	1.937064	0.893396	1.290185	1.375373	1.770732	1.971974
Wipi1	-0.184388	5.895713	-1.105252	0.287019	0.459207	-6.618949	5.862653	5.89833	5.606227	5.828272	5.682458	5.463612	6.161579	5.965724	5.985677	6.161414	5.700728	6.297779	5.867892	6.084468
Pou6f1	-0.158886	5.335869	-1.104937	0.287151	0.459334	-6.59909	5.466203	5.576408	5.373927	5.450438	5.078266	5.175995	5.44173	5.237996	5.479717	5.297103	5.206205	5.319923	5.191633	5.445693
Gls2	-0.332007	5.406539	-1.104831	0.287195	0.459334	-6.582261	5.506163	5.851358	4.506456	5.042128	5.004088	4.437656	6.019005	5.290321	5.789655	5.690653	5.526148	5.49113	5.599199	5.977367
Cyyr1	0.265329	4.907344	1.104789	0.287213	0.459334	-6.559861	4.993008	5.624015	4.882053	4.629961	4.888487	4.915569	5.377362	4.647404	5.016447	4.549787	4.291424	4.727257	4.752072	5.461855
Rilp	-0.364818	1.759186	-1.104511	0.287329	0.4594	-5.970689	1.996747	2.294795	1.061799	1.679737	1.05105	1.606524	2.380012	1.49863	1.690848	1.898256	1.978199	1.805289	2.097585	2.068949
Ndufa8	-0.185453	6.608645	-1.104509	0.28733	0.4594	-6.639624	7.174427	6.821356	6.631431	6.750911	7.10016	6.556457	6.484931	6.180309	6.638879	6.347893	6.550048	6.196157	6.537436	6.566939
Adnp2	-0.17407	4.690977	-1.104233	0.287446	0.459524	-6.5324	4.467845	4.228535	4.638911	4.85187	4.424547	4.654809	4.678618	4.937208	4.867041	4.690302	4.752428	4.769904	4.906128	4.866959
Vps25	0.156254	6.307592	1.104132	0.287488	0.45953	-6.637685	6.649636	6.439749	6.807521	6.420945	6.910144	6.606132	5.899229	6.157685	6.071954	6.102509	6.260557	6.013007	6.026321	5.961036
Hccs	-0.236448	4.034507	-1.103494	0.287756	0.459878	-6.494742	4.307974	3.684748	4.242177	4.424653	4.463969	4.113012	4.046082	3.871902	3.59934	3.747244	4.363019	3.822092	4.08486	3.808933
Aoc2-ps1	-0.272449	2.826053	-1.103431	0.287783	0.459878	-6.124616	2.213741	2.690649	2.162035	2.677071	2.154315	1.803538	3.496648	3.159031	3.533498	3.080873	3.149191	2.738492	3.409342	3.537917
RGD1308601	-0.223861	4.268269	-1.10174	0.288493	0.460952	-6.522319	4.342933	4.442374	4.600917	4.783243	3.935155	4.398567	4.390007	3.942114	4.268256	3.721909	4.216089	4.12775	4.56643	4.102607
Xpo4	0.202392	4.546379	1.101184	0.288727	0.461265	-6.516376	3.94308	4.582672	4.564365	4.807278	4.508156	4.702498	4.750481	4.536242	4.534483	4.51702	4.527854	4.426789	4.698844	4.6173
Alg3	0.193536	5.211925	1.100978	0.288814	0.461342	-6.608098	5.443117	5.204449	5.786295	5.418025	5.860036	5.633805	4.591292	5.175775	5.037713	5.003199	5.026046	4.90359	4.911337	5.015475
Pla2g16	0.26726	5.178198	1.100745	0.288912	0.461433	-6.568735	5.237611	5.607302	4.825515	4.491448	5.172806	4.909165	5.167624	5.214976	5.127233	5.604901	5.202306	5.699625	4.967428	5.311421
Klra1	0.694063	0.634793	1.10066	0.288948	0.461433	-5.700456	0.428583	-0.103798	1.089503	0.386344	1.364797	1.839907	0.68572	1.628188	-0.504027	1.51148	0.924208	2.447635	-1.413173	-0.116166
Cox6a1	0.250517	8.614165	1.100128	0.289172	0.461674	-6.616412	8.702787	8.748731	8.531498	7.907745	8.784501	8.164751	8.986791	8.394445	9.041328	8.780151	8.511126	8.46589	8.544835	9.037855
Serpina1	0.395582	1.145242	1.100039	0.28921	0.461674	-5.90415	1.957182	1.769471	1.472789	0.705659	1.78444	1.708392	1.836107	0.705741	0.837186	0.646479	0.676952	0.889587	0.491415	1.29285
Trappc11	-0.151888	6.282767	-1.100029	0.289213	0.461674	-6.636015	6.148277	5.759454	6.140948	6.308402	6.155054	6.249365	6.558848	6.422825	6.429581	6.241598	6.390448	6.285093	6.44348	6.445772
Upf3a	-0.179457	6.525266	-1.099065	0.28962	0.462212	-6.644747	6.968469	7.02842	6.84559	7.383762	6.784627	7.326548	6.190288	6.192145	6.159437	6.304818	6.034738	6.041136	6.119286	5.99228
Pdia2	-0.400883	3.099416	-1.09893	0.289677	0.462212	-6.23355	3.026365	3.411272	1.858187	2.899083	2.564599	2.549011	3.987806	2.951874	3.641623	3.52358	2.604547	3.153617	3.310292	4.111741
Wsb1	0.219577	7.935679	1.098872	0.289702	0.462212	-6.636444	7.45124	8.127112	7.535372	7.692664	7.732101	7.7643	8.339366	8.185675	8.133062	8.374467	7.880039	8.131423	7.645739	8.113535
Jrkl	-0.218957	2.724271	-1.098865	0.289705	0.462212	-6.276585	2.821286	2.582864	2.804219	3.394049	3.099296	3.114281	2.380012	2.571773	2.47808	2.600561	2.707156	2.680061	2.522078	2.622863
Bri3	0.184646	5.188963	1.098562	0.289832	0.462293	-6.58337	5.392103	5.15303	5.137285	4.682885	5.378822	5.19191	5.246787	5.265641	5.227804	5.4065	5.28389	5.183562	5.038184	5.100515
Ephb6	-0.1315	6.647534	-1.098562	0.289832	0.462293	-6.640659	6.444072	6.422481	6.176904	6.337577	6.1532	6.286074	6.909786	6.803249	7.012954	6.723108	6.799456	6.841025	6.99788	7.173894
Senp3	0.131892	6.418073	1.098346	0.289923	0.462377	-6.639534	6.315035	6.38107	6.354379	6.152957	6.509721	6.216893	6.637714	6.387803	6.549172	6.4888	6.480927	6.376243	6.415341	6.605437
Rheb	-0.183422	6.292457	-1.097799	0.290155	0.462684	-6.640777	6.208491	5.852648	6.183346	6.52389	6.335105	6.360719	6.503768	6.194762	6.377364	6.273554	6.483338	6.198241	6.490353	6.129006
Setd5	-0.190353	7.630085	-1.096869	0.290547	0.463215	-6.638911	7.508389	7.794084	7.70643	7.891816	7.157238	7.377308	7.878615	7.685115	7.832034	7.560769	7.619997	7.477081	7.623297	7.71703
Narf	-0.190297	5.875332	-1.096824	0.290567	0.463215	-6.636951	5.929345	6.303962	6.047816	6.321812	5.685027	5.713147	5.796029	5.672833	5.832874	5.695542	5.902825	5.588061	5.933365	5.858963
Mir504	-0.429867	-0.133827	-1.096737	0.290603	0.463215	-5.759067	0.592497	0.973336	-0.195392	1.300739	0.320024	1.249022	-0.831021	-0.048075	-0.632503	-0.420466	-0.616402	-0.434228	-0.893866	-0.397871
Nmd3	0.183453	4.848064	1.095927	0.290946	0.46366	-6.586827	4.791228	4.861671	5.441325	5.295932	5.325489	5.282232	4.729295	4.568886	4.663209	4.630291	4.579866	4.475702	4.627841	4.655275
Susd3	0.29742	2.382163	1.095895	0.290959	0.46366	-6.072485	2.582145	2.232748	2.187895	1.478901	2.530726	2.217002	2.206902	2.822481	2.295923	2.668442	2.556491	2.816513	2.58862	2.474989
Gss	0.23298	4.009854	1.095216	0.291247	0.463954	-6.437851	4.050381	4.267739	4.000932	3.515737	4.187881	3.579757	4.177031	4.061624	4.007682	4.010503	4.284065	3.931333	3.903318	4.258767
Exog	-0.196812	3.757243	-1.095189	0.291258	0.463954	-6.431334	3.978094	3.397198	3.793402	3.776576	3.623205	3.870884	3.810873	3.825375	3.648551	3.702612	3.939921	3.78376	4.070962	3.496677
Mansc1	-0.280679	3.673531	-1.095186	0.29126	0.463954	-6.388773	4.114729	3.34683	3.910635	3.240109	3.159643	3.069038	3.529702	4.009066	3.793114	3.814705	3.872828	4.123371	3.671242	3.901371
Gipc2	0.391044	0.336767	1.094517	0.291543	0.464326	-5.708766	0.830061	0.583632	1.340699	0.166125	0.808768	0.61372	0.357848	0.137473	0.449965	-0.116374	0.321999	0.514819	-0.305233	0.257494
Akap1	-0.129632	6.102301	-1.094423	0.291583	0.464326	-6.633616	5.911342	5.675942	5.926337	5.901553	5.736702	5.76608	6.463708	6.2258	6.514513	6.143636	6.100103	6.329488	6.325523	6.434897
Zbed4	0.196471	3.331642	1.09431	0.291631	0.464326	-6.331521	3.127854	3.256169	3.719973	3.315784	3.238702	3.438669	3.221182	3.395791	3.448717	3.167876	3.228006	3.502822	3.372046	3.365375
Anapc5	0.138875	7.811721	1.09427	0.291648	0.464326	-6.64185	7.72411	7.787692	7.624767	7.433254	7.856667	7.602055	8.033918	7.775734	7.948542	7.98318	7.853562	7.876269	7.860918	8.010481
Ddx51	0.302974	5.29368	1.093995	0.291765	0.46445	-6.575396	4.819083	5.840998	5.197401	4.872521	4.968684	4.608127	5.821279	5.368893	5.616738	5.65261	5.329285	5.187762	5.123695	5.746185
Pklr	-0.520883	-0.010812	-1.09366	0.291907	0.464615	-5.589204	0.243629	0.426209	-0.575682	0.22441	-0.424712	-0.291112	0.202532	-0.150641	-0.386063	0.348395	0.071497	-0.334482	0.921344	1.193114
Acbd3	0.195857	5.67221	1.093541	0.291957	0.464634	-6.638966	5.70438	5.86676	5.99935	6.212666	6.238736	6.517693	5.456846	5.285418	5.195053	5.370269	5.47536	5.327557	5.449691	5.342788
Naa40	0.220294	4.983268	1.092968	0.292201	0.46496	-6.56485	4.713215	5.296419	4.9741	4.766176	4.815341	4.765303	5.061434	4.968131	5.306568	5.322609	4.822678	5.067553	4.671475	5.2651
Gpr3	-0.380193	0.866378	-1.092588	0.292362	0.465155	-5.820909	0.873216	1.117652	0.914692	1.478901	0.691028	0.61372	1.135593	0.377517	0.512061	0.646479	0.535373	1.404538	1.252133	1.445134
Hnrnpa1	0.195464	5.18624	1.092266	0.292499	0.465311	-6.58703	4.785591	5.452583	5.014403	5.195699	5.247695	5.055522	5.657098	4.892772	5.362416	5.199524	4.985788	5.058407	5.156968	5.586654
Nudt5	0.216056	3.422961	1.092059	0.292587	0.465358	-6.374241	3.417469	3.294639	3.376367	3.419222	3.952316	3.901317	3.318966	3.377479	3.307029	3.385186	3.394437	3.179156	3.474137	3.270693
Gltscr1l	-0.222098	5.803191	-1.092015	0.292606	0.465358	-6.628429	5.506163	5.986746	5.552497	5.994505	5.054623	5.540207	6.219703	5.874	6.144812	5.837949	5.823827	5.790806	5.89969	6.048059
Crls1	-0.158643	4.756857	-1.091749	0.292719	0.465462	-6.560724	4.69529	4.749824	4.549481	4.897366	4.633136	4.636834	4.815587	4.515458	4.790809	4.821324	4.946707	4.73014	4.794977	5.077391
Siah3	-0.357545	0.824459	-1.091678	0.292749	0.465462	-5.950303	1.310059	1.812527	1.631973	2.077719	1.285769	0.772673	0.028455	0.377517	0.449965	0.055728	0.846404	0.760186	0.745852	0.321874
Slc35f5	-0.172915	4.775148	-1.091446	0.292847	0.465557	-6.508382	4.197708	4.232503	4.278735	4.494412	4.234219	3.96856	5.411014	4.995405	5.41415	5.199524	4.972116	5.132182	5.061799	5.321296

Arl4a	0.292859	3.498254	1.09132	0.292901	0.465581	-6.43345	4.105711	4.125594	4.337679	3.335752	3.529124	3.980827	3.105363	3.012464	3.149895	3.267546	3.760656	3.292705	3.431265	2.685374
Coil	-0.181672	4.218492	-1.090719	0.293157	0.465926	-6.542582	4.365777	4.286948	4.503885	4.87707	4.36492	4.621246	4.063116	4.055878	4.029076	3.918713	3.953901	3.767014	4.368551	3.968267
Syap1	-0.150106	4.468218	-1.090055	0.29344	0.466314	-6.558779	4.97826	4.619507	4.514142	4.538155	4.705768	4.578838	4.263083	4.360928	4.259232	4.398098	4.441188	4.454942	4.379868	4.134325
Arhgef12	-0.2083	6.63494	-1.089739	0.293574	0.466466	-6.654462	6.572363	6.63828	6.793378	6.948639	6.118463	6.660523	6.731262	6.926451	6.645821	6.346858	6.608662	6.575318	6.763083	6.576041
RGD1561145	0.327631	1.196965	1.0895	0.293676	0.466541	-5.772583	0.873216	1.480065	0.715742	0.621404	1.05105	1.046069	1.258992	1.564863	1.218642	1.704918	1.719153	1.618784	1.001683	1.582867
Mex3b	-0.402285	4.764992	-1.089445	0.293699	0.466541	-6.57048	4.909961	3.354134	4.634216	5.023782	4.720868	5.503835	4.8055	5.036833	4.646003	4.946908	5.250298	4.975806	4.624673	4.338735
Synj2	-0.213515	6.596358	-1.088921	0.293923	0.466835	-6.653992	6.564979	6.815415	6.329909	6.690164	6.044377	6.47063	6.705276	6.879239	6.685098	6.393736	6.671348	6.612505	7.002159	6.500691
Anks6	0.373868	4.919421	1.088709	0.294013	0.466875	-6.534863	4.395683	5.744246	4.71425	4.354614	4.531181	4.040642	5.419857	5.079939	5.195053	5.360041	4.918469	5.028276	4.769388	5.375386
RGD1305110	-0.22269	5.215562	-1.088625	0.294049	0.466875	-6.625202	5.344979	5.370323	5.760676	5.893717	5.068462	5.394994	5.146469	5.007364	5.097162	4.581825	5.058757	4.862143	5.458614	5.015475
Mrpl54	0.184013	5.393041	1.088588	0.294065	0.466875	-6.619484	5.661942	5.71901	5.338289	5.140008	5.669545	5.602394	5.488714	5.039747	5.271094	5.472549	5.375027	5.185663	5.296877	5.279355
Klf3	-0.185901	6.004935	-1.088301	0.294188	0.466924	-6.651139	6.100273	5.916945	6.27064	6.653509	6.079945	6.498625	5.824612	5.987017	5.685718	5.74039	5.891974	5.885643	5.747821	5.810704
Ppp2r3c	-0.171763	4.242983	-1.088242	0.294213	0.466924	-6.526896	4.424983	4.086661	4.166157	4.503267	4.567829	4.352109	4.182238	4.003106	4.15118	4.106622	4.295089	4.158035	4.43154	4.056051
Pkia	-0.327335	5.844606	-1.088099	0.294274	0.466924	-6.649303	5.924225	5.608829	6.49178	6.709423	5.572997	6.284426	5.501266	5.877262	5.636038	5.268953	6.013759	5.547781	6.077033	5.338905
Tmem39a	0.208436	3.783943	1.088076	0.294283	0.466924	-6.40825	3.627383	3.833259	3.855228	3.527359	3.86892	3.57437	4.248266	3.749088	4.039656	3.984107	3.480655	3.744381	3.614885	3.942904
Theg	-0.675741	0.283417	-1.088061	0.29429	0.466924	-5.472001	0.176305	-0.186163	-1.909221	-0.705589	-0.613814	-0.548781	1.607516	1.658838	1.360416	0.799637	0.631286	0.666985	0.962073	1.846283
Mgat4b	0.181587	6.778464	1.087931	0.294345	0.46695	-6.652016	6.537576	6.611101	6.655378	6.055981	6.480446	6.16229	7.069333	6.997118	7.007576	7.199893	6.965642	7.139839	6.85786	7.173349
Ndufaf4	-0.22226	4.516447	-1.087736	0.294428	0.46702	-6.579435	4.664912	4.279295	4.835775	5.282277	4.866025	5.030171	4.306644	4.219024	4.303799	4.182876	4.50588	4.220868	4.44597	4.156561
Grin2c	-0.319126	7.657372	-1.087613	0.294481	0.467042	-6.657565	7.4042	7.521892	6.254669	6.782172	6.87289	6.450925	8.250767	8.166139	8.207669	8.592615	8.034112	8.4916	7.833055	8.349708
Cst3	-0.352244	10.39512	-1.086865	0.294801	0.467442	-6.576464	10.71378	10.08362	9.887863	9.363868	9.832191	8.961151	10.19288	11.3156	10.38529	11.41577	11.29869	11.27291	10.15905	10.65041
Tmem205	-0.202285	4.503729	-1.086841	0.294811	0.467442	-6.492937	4.410408	4.332034	3.81015	4.00988	4.035185	3.862069	4.718584	4.870031	4.858103	5.082476	4.820137	4.662336	4.665321	4.988458
Arglu1	0.215792	7.441856	1.08636	0.295017	0.467697	-6.65547	7.083183	7.902364	7.316572	7.438265	7.291826	7.388861	7.708169	7.627686	7.600211	7.625816	7.231635	7.281182	7.221416	7.477953
Acsf2	-0.183259	6.333916	-1.086283	0.29505	0.467697	-6.658808	7.092344	6.839034	6.551515	6.58495	6.488556	6.742655	5.79433	6.08901	5.867432	6.289809	5.985681	6.367938	5.855784	6.145777
Fcgr2a	0.670021	-0.018748	1.085925	0.295203	0.467878	-5.471062	-0.634519	0.426209	0.398943	-0.705589	0.470544	-1.427823	-0.067151	0.645799	-0.080923	0.893396	0.631286	1.375373	-0.123798	-0.297732
Mrpl48	-0.162273	5.713311	-1.085601	0.295341	0.468036	-6.630894	5.674195	5.546434	5.194214	5.613349	5.725516	5.466522	6.029154	5.629	5.941229	5.8453	5.850228	5.704031	5.75073	6.046869
Cbwd1	-0.267682	2.687612	-1.085222	0.295504	0.468232	-6.22273	2.445855	2.064844	2.744481	3.16769	2.575715	2.800388	2.918339	2.522625	3.059216	2.707689	2.673757	2.761217	2.860463	2.570827
Wdr5b	0.326297	2.122838	1.084872	0.295653	0.468407	-6.092715	1.9771	1.725091	2.286937	2.124384	2.411703	3.030221	1.888013	2.326577	1.60797	2.359011	2.014226	2.36102	1.922909	2.050068
Nedd9	-0.331001	4.853818	-1.084457	0.295832	0.468608	-6.607068	5.010026	4.091039	5.433239	5.625567	4.901799	5.503835	4.519061	4.799571	4.646003	4.513333	4.79447	4.591187	4.992221	4.588148
Trim24	-0.159343	4.795062	-1.084394	0.295858	0.468608	-6.572901	4.707265	4.610386	4.872118	4.977929	4.755497	4.501484	5.130397	4.69993	4.930947	4.896847	4.681385	4.755828	4.806206	4.861554
Myh7	-0.408922	0.862763	-1.083864	0.296085	0.46886	-5.751921	1.071521	0.24948	-0.195392	0.664146	1.113405	0.772673	1.297885	1.198329	0.837186	1.451089	1.375754	0.930264	0.921344	1.29285
Rab43	0.419372	1.471949	1.083841	0.296096	0.46886	-5.859518	1.430522	1.747452	1.858187	0.705659	0.952152	1.249022	1.335758	2.14364	1.180909	1.388059	2.100543	2.529342	1.316372	1.260365
Ccz1	0.177793	6.045839	1.083668	0.29617	0.468884	-6.640204	5.722193	6.095337	5.705797	5.673428	6.018132	5.685933	6.528508	6.15231	6.35953	6.236016	6.137333	6.057295	5.956227	6.338017
Baiap2l1	-0.6776	1.035843	-1.083594	0.296201	0.468884	-5.996458	3.205888	1.248835	1.430088	0.823477	1.018831	1.402804	0.282279	0.137473	0.317129	0.981432	2.86345	0.460309	1.077784	0.119416
Nap1l1	0.24234	7.453254	1.083486	0.296248	0.468884	-6.651022	7.252091	7.553168	7.645328	8.054283	7.981812	8.395492	7.334287	7.190459	7.169264	7.138986	7.112571	7.123488	7.263267	7.140257
Taf15	0.184475	4.518572	1.083442	0.296266	0.468884	-6.477373	3.92781	4.167656	3.986269	3.993213	4.393472	4.044542	5.224248	4.714593	4.846099	4.930413	4.730939	4.744468	4.734547	4.89368
Krtcap2	0.193968	5.235236	1.083348	0.296307	0.468886	-6.597431	5.172161	5.090994	5.090066	4.837937	5.593651	5.008367	5.164996	5.102412	5.364521	5.542957	5.452566	5.314172	5.246245	5.354378
Ccdc60	1.418151	-1.032235	1.082641	0.29661	0.469249	-5.292285	1.430522	-1.685466	0.049949	-3.260591	0.091273	-0.750296	-0.067151	-1.435005	-1.813089	-0.420466	0.535373	-0.657145	-1.047095	-0.621773
Prelp	-0.438772	5.356513	-1.082556	0.296647	0.469249	-6.642882	6.727396	5.22252	4.709794	5.153196	5.848624	6.209094	4.56363	5.057109	4.784533	5.390509	5.852718	5.644157	4.836642	5.032408
Clasrp	0.304317	8.063027	1.08253	0.296658	0.469249	-6.650355	7.704829	8.675004	7.92472	7.707108	7.831383	7.602385	8.5619	8.120416	8.496336	8.399651	7.775682	7.884379	7.718118	8.486555
Irf6	-0.517312	2.115419	-1.08245	0.296692	0.469249	-6.339005	2.810225	4.184143	2.861581	4.018141	2.240661	2.537984	1.21902	0.04768	1.843557	1.780488	2.049375	2.252925	1.001683	1.227132
Bdp1	-0.437881	1.458095	-1.082268	0.296771	0.469311	-6.03245	1.568201	1.369077	2.379614	2.487785	0.845972	1.821837	1.511505	1.68885	1.218642	0.646479	1.430105	1.542121	1.700256	0.799858
Ccdc77	0.266486	2.64691	1.082019	0.296878	0.469374	-6.180298	2.445855	2.894758	2.578448	2.41457	2.856578	2.469996	3.060831	2.709903	2.611707	3.030171	2.883067	2.284645	2.408783	2.660694
Sema3f	0.432545	2.789462	1.081993	0.296889	0.469374	-6.291307	3.402817	3.982112	3.283475	2.401998	3.313654	2.705027	2.582602	1.747064	2.720913	2.854798	3.414796	2.284645	2.168911	2.431883
Creg1	0.243046	5.116925	1.081757	0.29699	0.469472	-6.60926	5.239674	5.161381	5.518508	5.00935	5.380408	5.391936	4.316148	5.452183	4.449761	4.850841	5.385381	5.835662	4.863765	4.828697
Spg20	-0.197839	6.084064	-1.081521	0.297091	0.469522	-6.66171	6.459198	6.055567	6.367168	6.758327	6.40844	6.812949	5.556439	5.940998	5.712413	5.67093	5.874931	5.93395	5.973761	5.67557
Zfp787	0.161199	3.614598	1.081502	0.2971	0.469522	-6.401333	3.529616	3.563961	3.746318	3.504019	3.823012	3.563537	3.585784	3.614836	3.424703	3.728285	3.872828	3.631738	3.640205	3.503633
Mtr	0.213806	4.067629	1.08112	0.297264	0.469712	-6.461886	3.689063	4.382912	4.308508	3.989017	3.623205	3.922669	4.287446	4.17729	4.250151	4.000002	3.981461	4.065203	4.13915	4.225496
Tmem56	0.308185	3.178461	1.081041	0.297298	0.469712	-6.360856	3.064101	3.232585	4.015447	3.468286	3.167012	3.857641	2.880403	3.000548	2.962454	2.375352	2.81321	3.065063	3.658907	3.11589
Pard3b	0.315643	1.371398	1.080057	0.297722	0.470319	-6.058905	2.246933	1.972946	2.548818	1.548999	1.96438	2.217002	0.563361	0.517885	0.628769	0.981432	0.846404	0.889587	1.60059	1.32462
Clic3	-0.491723	0.971104	-1.079819	0.297824	0.47042	-5.8915	1.936985	1.480065	1.169557	1.000693	0.210178	1.046069	1.258992	-0.150641	1.255413	1.28803	1.740817	0.760186	0.836265	0.606649
B4galt4	-0.161079	4.751643	-1.079198	0.298091	0.47078	-6.58046	4.802435	4.664267	4.600917	4.926188	5.004088	4.554424	4.637512	4.532109	4.824848	4.775886	4.830277	4.859513	4.737483	4.831464
Ndrg2	-0.228238	10.71607	-1.078865	0.298235	0.470945	-6.534782	11.55601	11.38421	10.97008	11.04307	11.04626	10.88753	10.47768	10.00692	10.45734	10.50352	10.00487	10.55364	10.42511	10.70975
Rnf2	-0.33276	5.304579	-1.07846	0.298409	0.471111	-6.64447	5.492389	4.625556	5.806258	6.164167	5.596383	5.942639	4.861747	5.394161	5.241615	4.859579	5.338195	4.948838	5.284886	4.748987
RGD1310553	-0.17248	4.83898	-1.078439	0.298419	0.471111	-6.602507	5.096788	4.570182	4.915329	5.136218	5.130085	5.152692	4.767901	4.600808	4.697018	4.564114	4.717344	4.792144	4.964926	4.695317
Jade2	-0.25928	5.454887	-1.078294	0.298481	0.471148	-6.649503	5.785686	5.501094	6.036296	6.048943	5.207167	5.885066	5.072692	5.432361	5.104739	4.922095	5.287575	5.243299	5.800709	5.077391
Maoa	0.17032	5.814936	1.077942	0.298633	0.471305	-6.654074	5.77577	5.876938	5.916704	6.048943	6.091609	6.447982	5.807867	5.609519	5.879261	5.345599	5.555479	5.475754	5.887848	5.718054
Rarres1	0.566341	1.861366	1.07781	0.29869	0.471305	-5.95547	1.541696	0.973336	1.340699	1.245516	2.847434	2.570814	1.297885	2.454377	1.326257	2.359011	2.031907	3.083214	1.407665	2.050068
Thumpd1	-0.190017	6.611252	-1.077791	0.298698	0.471305	-6.668168	6.751449	6.555159	6.860239	7.341181	6.870071	7.139063	6.370998	6.433944	6.425553	6.232656	6.497725	6.274271	6.528989	6.292556
Tiprl	-0.259478	5.17915	-1.077319	0.298902	0.471513	-6.642013	5.315947	4.993802	5.512125	6.131223	5.459054	5.89806	5.273864	4.490115	5.000289	4.933176	4.958312	4.616334	5.042938	4.927694
Nfkbil1	0.267224	4.074725	1.077304	0.298909	0.471513	-6.478124	4.331374	4.567042	4.299641	3.480296	4.147475	3.62735	4.113042	4.078726	4.096495	4.233056	4.204441	4.158035	3.989574	3.814538
Mreg	0.484456	0.505266	1.076617	0.299205	0.471919	-5.719816	0.485316	0.583632	1.534559	0.386344	0.470544	0.915838	0.282279	1.239467	0.317129	0.413143	0.805871	0.403657	1.001683	-0.621773
Higd2a	0.255513	4.902375	1.076384	0.299306	0.472011	-6.534166	4.71914	4.506054	4.631863	3.875379	4.930226	4.08677	5.372815	5.154424	5.426295	5.255774	5.325705	5.069831	5.134872	5.200217
Ap5z1	0.199177	6.140856	1.076301	0.299342	0.472011	-6.651295	5.845148	6.388203	5.928256	5.699348	5.818526	5.751835	6.411463	6.309075	6.391887	6.486922	6.184537	6.23625	6.00577	6.537588
Mtf1	-0.256482	3.250071	-1.075545	0.299669	0.472464	-6.355979	3.100876	2.801663	3.336461	3.670505	2.970456	3.426763	2.867532	3.458111	3.262563	3.267546	3.165302	3.562264	3.608485	3.169358
Megf8	-0.21232	8.440602	-1.075334	0.29976	0.472489	-6.652266	7.92461	8.265487	8.077142	8.335768	7.559118	7.763709	9.05401	8.600418	9.139879	8.48213	8.676401	8.767531	8.640274	8.886765
Mecp2	-0.395088	3.828352	-1.075327	0.299763	0.472489	-6.546567	4.193457	4.256089	4.847991	4.99058	3.259533	4.443576	3.221182	3.878428	3.197914	3.100666	3.448101	3.562264	4.191471	3.12494
Eno4	-0.276162	3.48805	-1.074731	0.300021	0.472797	-6.313729	3.529616	3.208609	2.61703	2.844379	3.020129	2.791131	4.187425	3.832115	3.742206	3.957219	3.967746	3.549265	3.602057	4.134325
Akap17a	0.134831	6.919129	1.074693	0.300038	0.472797	-6.671069	6.919129	7.16291	7.063363	7.02544	7.094375	7.107246	6.836553	6.860407	6.803893	7.060943	6.833882	6.845685	6.485995	6.781016
Tpp2	-0.2698	6.326222	-1.074561	0.300095	0.472826	-6.667851	6.118322	5.650954	6.254669	6.964223	6.466329	6.715998	6.631066	6.056287	6.399094	6.085228	6.270831	6.052697	6.444379	6.477031
Abt1	-0.193547	3.897143	-1.074328	0.300196	0.472922	-6.459126	3.793849	3.397198	3.941366	4.018141	3.86892	3.632543	3.969854	4.073048	4.018418	4.061889	4.284065	3.906064	4.009141	3.692011
Usp51	0.3271	0.794767	1.073688	0.300473	0.473298	-5.820282	0.592497	1.117652	0.975347	1.158502	1.390206	1.353343	0.68572	0.972957	0.628769	0.134646	0.321999	0.618025	1.001683	1.085968
Foxk1	-0.324109	3.866693	-1.072895	0.300817	0.473777	-6.526639	4.573017	3.859159	4.740698	4.217846	3.563034	4.06389	3.148563	3.929599	3.655446	3.276274	3.967746	3.916225	4.130242	3.203933
Camlg	-0.199786	4.915677	-1.071	0.30164	0.474985	-6.59486	5.052887	4.361326	4.843931	4.830919	5.084116	4.657358	4.753981	4.949657	5.07419	4.965917	5.124097	4.953779	5.276837	4.943127
Becn1	-0.145459	6.954288	-1.070948	0.301663	0.474985	-6.675376	6.972195	6.793419	6.885151	7.128241	6.867812	7.072803	7.063702	7.060187	7.048092	6.88879	7.050495	6.797714	7.01189	6.732253
Phax	-0.218238	5.239045	-1.070242	0.30197	0.475406	-6.64013	5.315947	4.838423	5.267291	5.769538	5.611315	5.58375	5.064257	5.191584	5.102217	5.018886	5.37156	4.966058	5.262642	5.025175
Aar2	-0.255688	4.415588	-1.069478	0.302302	0.475779	-6.521449	4.485336	3.570246	4.354903	4.125276	4.448934	3.770827	4.438678	4.576933	4.593117	4.468337	4.920843	4.724369	4.895654	4.521176
Tmem216	0.217364	3.938501	1.0694	0.302336	0.475779	-6.444956	3.821655	4.046648	3.746318	3.515737	4.184254	3.55809	4.017238	4.032662	4.023757	4.246444	4.242906	3.895831	3.73726	4.174106
Gucd1	0.279146	1.429915	1.069389	0.302341	0.475779	-5.940832	1.594228	1.397635	1.905712	1.158502	1.72577	1.473956	1.258992	1.564863	1.102347	1.322149	1.652137	1.404538	1.651284	1.386135
RGD1311703	-0.220329	6.778574	-1.069333	0.302366	0.475779	-6.672876	7.217973	6.837731	7.398799	7.715388	7.078832	7.669915	6.274358	6.487219	6.486754	6.237134	6.575891	6.361445	6.389444	6.184165
Itm2b	-0.180424	9.99736	-1.069217	0.302416	0.475796	-6.601444	9.899324	9.357397	9.472097	9.736135	9.921152	9.803456	10.07125	10.18841	10.22144	10.27731	10.09575	10.36935	10.12456	10.42699
Kptn	-0.213394	3.08367	-1.069009	0.302507	0.475877	-6.301936	3.100876	2.790938	3.035447	3.059778	3.020129	2.613455	3.488265	3.137429	3.634661	3.050666	3.099749	2.960934	2.994118	3.372991
Cops6	-0.134605	7.059485	-1.068774	0.302609	0.475975	-6.675765	7.365998	7.128715	6.999999	7.203523	7.16462	7.308007	7.151265	6.800674	6.99268	7.030311	6.922453	6.773466	6.951226	7.051675
Lage3	0.177562	5.466092	1.068527	0.302717	0.476082	-6.635672	5.607076	5.449176	5.364037	5.038072	5.761253	5.408679	5.588166	5.366574	5.593589	5.573377	5.564618	5.271276	5.485056	5.490147
Slc4a1ap	-0.191251	6.178892	-1.068419	0.302764	0.476094	-6.671203	6.133932	5.717595	6.093852	6.501967	6.2801	6.415968	6.252249	6.271141	6.287014	6.058912	6.293914	6.098034	6.129379	5.99228
Stk11ip	0.16949	5.163654	1.068237	0.302843	0.476104	-6.597327	4.881181	5.254098	5.153781	4.761263	4.715852	4.836779	5.542337	5.225253	5.356081	5.437958	5.172725	5.218879	5.370586	5.409117
Ocel1	0.208687	4.270236	1.06815	0.30288	0.476104	-6.518511	4.460789	4.541677	4.029817	3.791112	4.487704	4.254448	4.32559	4.219024	4.165741	4.509637	4.338363	4.166573	4.216936	4.358053
Tmem11	-0.206727	4.205025	-1.068011	0.302942	0.476104	-6.611365	4.983193	4.4112	5.021611	5.21918	4.843207	5.292109	3.669732	3.630385	3.722643	3.615871	3.944596	3.516245	3.582598	3.510555
Cog8	-0.140976	5.238294	-1.067984	0.302953	0.476104	-6.629696	5.348806	4.993802	5.163589	5.295932	5.378822	5.184859	5.276301	5.343175	5.336908	5.157622	5.345284	5.24934	5.240048	5.063337
Usp40	0.320033	5.790208	1.06795	0.302968	0.476104	-6.640679	5.35643	6.373003	5.323783	5.355827	5.52649	5.496734	6.33171	5.835949	6.136211	6.265905	5.750968	5.805913	5.306793	6.226823
Slc7a10	-0.300283	5.921885	-1.067453	0.303185	0.476382	-6.660416	6.308172	6.242317	5.15214	5.526249	5.780598	5.30192	6.454055	5.412264	6.388787	6.04621	5.93019	5.790806	6.107988	6.491973
Fap	0.834288	-0.555548	1.06692	0.303418	0.476676	-5.71881	1.80948	2.064844	1.363571	-1.075778	0.731352	0.056669	0.498056	-0.26106	-2.155202	-1.33445	-1.500575	-1.437379	-2.232431	-1.040339
Gnpda1	0.166587	4.99822	1.066844	0.303451	0.476676	-6.597249	4.89695	4.835816	4.89192	4.713732	5.290494	4.8591	4.767901	5.074265	4.992142	5.087444	5.364599	5.277201	5.00202	4.970998
Slc25a11	-0.18207	5.973262	-1.06656	0.303575	0.476808	-6.657665	5.889175	5.464442	5.591771	5.677346	5.979941	5.364112	6.319948	6.129238	6.287014	6.007418	6.435151	6.004699	6.300927	6.20031
Hemk1	0.214714	2.928143	1.066277	0.303699	0.47694	-6.228779	2.730329	2.934281	2.548818	2.451642	3.060255	2.675144	3.072094	3.012464	3.280514	3.267546	2.977336	3.110019	3.003904	3.079111
Col9a2	-0.45929	5.480765	-1.065971	0.303832	0.477087	-6.609529	5.180775	5.970204	4.397084	5.097757	4.548211	3.870884	5.989609	5.850958	6.08988	6.593784	5.527707	6.029482	5.409761	6.216276
Suox	-0.14199	5.200862	-1.065692	0.303954	0.477217	-6.628962	5.298244	5.062295	5.062374	5.258066	5.282035	5.138162	5.044381	5.304929	5.183174	5.4065	5.034842	5.235205	5.223392	5.321296
Fam213a	-0.208432	4.836054	-1.065241	0.304152	0.477464	-6.604003	5.388389	4.704825	4.705324	4.572219	5.089943	4.559885	4.725734	4.750611	4.818719	4.963216	4.872595	4.822175	4.9009	4.885716
RGD1563348	0.358134	2.539387	1.064502	0.304475	0.477909	-6.087532	2.387517	2.864389	2.225837	1.47891	2.240661	1.766229	3.003159	3.036005	2.928701	3.050666	3.004456	2.447635	2.783013	2.622863
Cldn3	-0.967765	-0.36197	-1.064282	0.304571	0.477998	-5.600865	1.341131	-0.572649	0.307539	-0.600663	0.210178	-2.070862	-0.672244	-2.076466	-0.080923	0.591587	1.230182	-1.073407	0.375586	0.606649
Nab1	-0.145	5.57445	-1.063995	0.304697	0.478133	-6.660658	5.689367	5.697636	5.454701	5.865378	5.746573	5.638975	5.787514	5.105197	5.56458	5.40451	5.462379	5.39813	5.701967	5.558541
Zbtb2	-0.214053	4.274765	-1.063744	0.304807	0.478186	-6.565875	4.467845	3.806886	4.687307	4.711187	4.384017	4.600198	4.096591	4.193082	4.076084	4.224061	4.030659	4.372473	4.056929	4.221283
Gtf2a1	-0.336083	3.184828	-1.063736	0.30481	0.478186	-6.428004	3.431974	2.864389	3.843073	4.00988	2.936364	3.879645	2.717819	3.211662	3.149895	2.484835	3.116419	3.118845	3.431265	2.570827
Jmjd1c	-0.233981	7.467743	-1.063326	0.30499	0.478405	-6.679471	7.082034	7.440598	7.461762	7.825401	6.719715	7.252176	7.763492	7.803432	7.666985	7.569673	7.450396	7.461554	7.522653	7.537625
Cd320	0.426602	4.000456	1.063185	0.305052	0.478431	-6.415756	4.243653	4.445797	3.776458	2.613062	4.102065	2.982217	4.339638	4.084383	4.111616	4.377964	4.493172	4.220868	4.023644	4.299305
Hdc	-0.911353	0.194334	-1.063107	0.305086	0.478431	-5.536071	1.853246	-1.463779	-0.575682	-1.575022	-0.107758	-1.26448	1.135593	0.137473	0.97585	1.51148	1.605665	0.889587	0.791767	0.706485
Purb	-0.374845	5.883995	-1.062833	0.305206	0.478542	-6.675896	5.936993	5.56858	6.263057	6.716421	5.393031	6.583521	5.597939	5.903095	5.525598	5.374339	5.959358	5.824915	6.469316	5.287439
Tm6sf1	0.199993	4.628786	1.062764	0.305236	0.478542	-6.583307	4.304037	4.788078	4.053829	4.91074	4.941012	4.930402	4.473071	4.429191	4.299404	4.592349	4.558676	4.97094	4.438773	4.626888
Prr3	0.183441	6.238761	1.062199	0.305484	0.478831	-6.672233	6.118322	6.372104	6.054859	5.805781	6.379393	5.77315	6.385741	6.363586	6.493504	6.433297	6.473667	6.180436	6.256592	6.273352
RT1-CE4	0.388454	2.391811	1.062141	0.30551	0.478831	-6.170355	2.947796	1.991799	3.151703	1.615848	2.542105	2.734303	1.938115	2.287882	1.914249	2.923085	3.091342	2.691938	1.881043	2.087586
Foxo3	-0.300567	4.503614	-1.062071	0.30554	0.478831	-6.607575	4.891713	4.354059	5.165217	5.140008	4.258567	5.067073	4.107579	4.438054	4.034376	3.968034	4.49636	4.733017	4.710843	3.757487
Shoc2	-0.336312	4.904169	-1.061631	0.305733	0.479071	-6.647313	5.266224	4.541677	5.4941	5.845784	5.101527	5.733829	4.425566	4.782275	4.618038	3.90752	4.88724	4.613215	5.134872	4.361886
Commd3	0.122221	6.302657	1.061472	0.305803	0.479116	-6.682227	6.491582	6.501077	6.507269	6.434125	6.761083	6.570052	6.134975	6.09182	6.16307	6.227039	6.152567	6.022445	6.144941	6.055178
Rrn3	-0.249171	3.892986	-1.061385	0.305842	0.479116	-6.494719	3.788223	3.570246	4.136585	4.331596	3.464807	4.079183	4.113042	3.871902	3.924502	3.463704	4.095154	3.750073	4.233667	3.786295
Rasl10a	-0.215631	5.206013	-1.061092	0.30597	0.479216	-6.636613	5.143805	4.7275	5.147205	5.542091	5.335334	5.30192	5.092185	5.146336	5.446313	5.337281	5.375027	5.049203	4.86645	5.416507
Adamtsl4	0.635859	3.544345	1.061059	0.305985	0.479216	-6.365585	4.502616	4.064569	3.118472	1.740915	3.751288	3.844276	2.918339	3.676051	3.140097	4.290193	5.276493	3.970866	2.285827	3.203933
Ccny	-0.205804	6.160025	-1.060638	0.30617	0.479442	-6.683787	6.461851	5.989094	6.582309	6.766932	6.261288	6.800892	5.924304	6.008001	5.759573	5.763854	6.043481	5.889516	6.140512	5.871116
Mbnl1	-0.267896	4.285551	-1.060264	0.306334	0.479637	-6.5688	4.417714	4.256089	4.691832	4.783243	3.939464	4.446528	4.123906	4.70361	4.199156	3.759747	4.302392	4.31219	4.44597	3.698087
Cyb561	-0.270544	5.25082	-1.060083	0.306414	0.479699	-6.61445	5.616631	4.69623	5.001701	4.512069	4.757939	4.672562	5.312364	5.207221	5.591793	5.611814	5.938417	5.454995	5.653054	5.528164
Mss51	0.453659	3.078975	1.059858	0.306513	0.479792	-6.170743	2.342161	3.570246	2.345554	2.124384	2.226624	2.654873	3.987806	3.340143	3.648551	3.911274	3.181236	3.30822	2.838755	3.831222
Nfat5	-0.229188	7.456718	-1.059128	0.306834	0.480204	-6.683913	7.020382	7.389972	7.196312	7.878832	6.921626	7.366831	8.012156	7.587923	7.699874	7.46094	7.432618	7.322357	7.412164	7.701233
Spdya	-0.227925	3.236465	-1.059078	0.306856	0.480204	-6.413874	3.342675	3.404252	3.767911	3.815017	2.987204	3.268997	3.410541	2.888628	3.280514	3.050666	3.258364	2.910998	3.061266	3.031778
Med13	-0.327365	5.609016	-1.058909	0.306931	0.480258	-6.670863	5.386529	5.546434	5.989274	6.54834	5.078266	5.957198	5.584238	5.765523	5.586392	5.047212	5.549353	5.387214	5.878569	5.25483
Sparc	0.289579	10.06875	1.058393	0.307158	0.48053	-6.606198	10.4763	10.15253	10.15271	9.187157	10.13287	10.06883	9.710222	10.26403	10.02522	10.13198	10.03868	10.54348	9.752104	10.32791
Ube2z	-0.190528	6.730977	-1.058334	0.307184	0.48053	-6.68826	6.840444	6.270577	6.895978	6.812176	6.666117	6.709256	6.56484	6.795511	6.568479	6.624871	7.034686	6.803199	7.037724	6.624706
Alkbh8	-0.326689	2.405665	-1.05784	0.307401	0.480807	-6.211499	2.311111	1.874793	2.908996	2.985885	1.912714	2.592292	2.828218	2.603631	2.347926	2.06872	2.531849	2.187314	2.575555	2.211707
Ovol1	-0.342336	2.435484	-1.057623	0.307497	0.480855	-6.142357	2.488111	2.279532	1.593793	2.198954	2.335271	1.857753	3.159163	2.227821	2.810125	3.030171	2.346454	2.594065	2.285827	3.195366
Blzf1	-0.166585	4.080315	-1.057589	0.307512	0.480855	-6.57092	4.439412	4.099756	4.498728	4.680285	4.409093	4.562608	3.830902	3.95452	4.023757	3.740952	3.862983	3.772618	3.772036	3.571405

Ccdc127	-0.24909	4.024744	-1.057376	0.307606	0.480939	-6.526446	4.050381	3.532115	4.008208	4.43703	4.213017	4.227248	3.742028	4.145178	4.044916	3.676473	4.090943	3.990233	4.505736	3.780579
Adam9	0.168106	6.377585	1.057196	0.307685	0.480982	-6.686606	6.587829	6.291652	6.763593	6.304185	6.529152	6.867919	6.138997	6.232184	6.189438	6.166118	6.214847	6.474062	6.27186	6.273352
Pias2	-0.244086	5.111784	-1.057048	0.307751	0.480982	-6.653189	5.367791	4.779965	5.499273	5.712135	5.305596	5.59177	4.788531	4.940331	4.80328	4.602796	5.212033	4.851593	5.366799	4.789392
Rspry1	-0.2087	4.265766	-1.057042	0.307753	0.480982	-6.548761	4.255933	4.263866	4.506456	4.523721	3.813652	4.149692	4.320877	4.145178	4.485023	3.946322	4.407721	4.187698	4.640443	4.156561
Vps4b	-0.145774	5.400784	-1.056827	0.307848	0.481067	-6.654827	5.462675	5.086616	5.559943	5.594136	5.349976	5.52772	5.437382	5.307349	5.403951	5.257979	5.429405	5.417931	5.456834	5.3563
Myt1	-0.247254	4.642536	-1.056736	0.307888	0.481068	-6.560106	3.997728	4.309667	4.326082	4.973688	3.943761	4.428729	5.119582	4.863466	5.079327	4.979343	4.527854	4.572035	4.839377	5.100515
Lman1	-0.187032	4.777996	-1.056066	0.308184	0.481434	-6.615284	4.930537	4.425138	4.90172	5.052221	4.753051	5.127168	4.56363	4.60868	4.710323	4.693564	4.546426	4.908688	4.800602	4.927694
Hsdl2	-0.186511	5.52994	-1.055943	0.308238	0.481434	-6.667386	5.843793	5.585746	5.248982	5.666875	6.002771	5.441593	5.307608	5.499519	5.368723	5.464933	5.429405	5.641098	5.320563	5.631797
Pomt2	0.34329	5.558876	1.055933	0.308243	0.481434	-6.60959	5.148204	5.916945	5.276359	4.390058	4.808289	4.431711	6.222233	5.939438	6.189438	6.004794	5.689631	5.814088	5.755083	6.275385
Galnt10	0.266908	2.949403	1.05554	0.308417	0.481643	-6.253611	2.937667	2.884706	3.077557	2.350586	3.122223	2.481552	2.880403	3.272386	3.207329	2.866406	3.048553	3.489274	3.03287	2.847239
Clcn5	-0.369857	0.756371	-1.054989	0.30866	0.481936	-5.728372	0.643243	0.118359	0.785152	0.621404	-0.038319	0.231108	1.21902	1.596873	0.97585	0.799637	0.924208	0.889587	1.377871	1.415936
Camsap3	-0.197882	5.855842	-1.054933	0.308685	0.481936	-6.671392	5.7514	6.125528	5.597812	5.89596	5.410211	5.38426	6.242313	5.700719	6.200088	6.021763	5.81747	5.761518	5.902308	6.198168
Stard7	-0.15537	5.459256	-1.054778	0.308753	0.481937	-6.642149	5.373438	5.238395	5.303227	5.242285	4.995836	5.06899	5.668273	5.655839	5.593589	5.548372	5.832682	5.638031	5.875907	5.431174
Rest	0.281494	2.712632	1.05468	0.308797	0.481937	-6.298229	2.8647	2.833367	3.171281	2.816229	2.919011	3.519371	2.078759	2.964197	2.107651	2.291729	2.761158	2.794648	3.013624	2.087586
Polr3f	-0.152242	5.427583	-1.054661	0.308805	0.481937	-6.652341	5.189338	5.288816	5.211652	5.715949	5.310595	5.408679	5.692193	5.394161	5.544292	5.436012	5.486624	5.243299	5.573143	5.528164
Fam207a	0.246552	4.723717	1.054503	0.308875	0.481983	-6.57511	4.774252	5.044346	4.709794	4.117607	4.879544	4.113012	4.812232	4.685117	4.730053	4.971302	5.075905	4.843629	4.731605	4.704402
Nop56	0.164633	6.823179	1.054198	0.30901	0.482131	-6.692631	6.636389	6.893312	7.157166	7.122506	7.070015	7.056959	6.91683	6.573201	6.888993	6.796997	6.436812	6.468894	6.623267	6.897159
Emcn	0.279854	4.13544	1.054037	0.309081	0.482179	-6.575197	4.519693	5.080024	4.008208	4.341506	5.006144	4.770022	4.134688	3.567159	3.842287	4.076955	3.401255	3.827486	3.529386	3.885483
Col2a1	0.756474	1.443529	1.053399	0.309364	0.482529	-5.748309	0.785575	2.801663	0.750864	-0.02435	0.027932	0.949514	2.286414	1.596873	2.166756	2.543858	1.557647	1.826994	1.28461	2.357047
Vps54	0.159724	5.582348	1.053349	0.309386	0.482529	-6.66862	5.425104	5.818732	5.8992	5.820027	5.599109	5.820557	5.647719	5.350235	5.424278	5.560929	5.368084	5.610136	5.274818	5.566866
Mnat1	-0.270248	4.357269	-1.052506	0.309759	0.483049	-6.601818	4.637014	3.854016	4.57175	5.031965	4.908409	4.913438	4.330288	4.171987	4.290573	3.924277	4.383823	3.755742	4.334055	3.973287
Ctr9	-0.167824	6.992905	-1.051867	0.310043	0.483429	-6.694793	6.900345	6.851345	6.887126	7.429775	6.917262	7.331745	7.038087	6.98129	7.004204	6.919063	6.820592	6.940207	6.944254	6.947764
LOC288978	-0.297432	1.737641	-1.051697	0.310118	0.483483	-6.061669	1.787089	1.425638	1.340699	2.13961	1.96438	2.00915	2.227194	1.393205	1.549949	2.048482	1.375754	1.667719	1.963595	1.910497
Wdr41	0.118758	5.54939	1.051211	0.310334	0.483724	-6.668092	5.671142	5.724656	5.7542	5.489584	5.719264	5.593102	5.402117	5.551489	5.42831	5.382447	5.527707	5.494525	5.526381	5.460068
Ankrd33	0.176461	3.50508	1.051167	0.310353	0.483724	-6.450049	3.556342	4.117033	3.8018	3.644058	3.623205	3.59047	3.624549	3.262441	3.555781	3.195748	3.157269	3.331185	3.318158	3.432519
Tmod2	-0.208527	5.71387	-1.050967	0.310442	0.4838	-6.688601	6.188342	5.978499	5.940668	6.370214	6.105098	6.261147	5.256692	5.31939	5.173599	5.119326	5.675639	5.741657	5.875187	5.109662
Me3	-0.246098	6.542259	-1.050782	0.310524	0.483825	-6.68548	6.090021	5.912005	5.621726	6.190906	6.372238	5.677165	7.40112	6.594167	7.317363	7.018655	6.903969	6.67293	6.605701	7.232113
Mok	-0.179182	4.148033	-1.05075	0.310539	0.483825	-6.528101	4.319721	4.224555	3.879235	4.04669	4.043216	3.875271	4.339638	4.112335	4.13647	4.528025	4.316888	4.023513	4.061622	4.25465
Cpne7	0.297663	8.270011	1.050413	0.310688	0.483995	-6.685599	7.749303	8.202896	7.958916	7.534276	8.461123	7.133122	9.224889	8.058186	9.169684	8.404386	8.146421	7.809697	8.69642	9.23646
Tpst2	0.191628	4.998406	1.050287	0.310744	0.483999	-6.588384	4.805223	4.853963	4.652905	4.274118	4.923716	4.472821	5.305224	5.22269	5.295576	5.41047	5.255952	5.432163	4.908735	5.215125
RGD1306746	0.175567	4.671004	1.050227	0.310771	0.483999	-6.564594	4.45014	4.817439	4.363439	4.228565	4.656883	4.227248	5.000844	4.90241	4.809475	4.885482	4.786679	4.735888	4.766516	4.825924
Erg	0.246298	3.725592	1.049746	0.310985	0.484269	-6.51007	4.276172	4.506054	4.143209	3.584109	4.043216	3.97675	3.876582	2.951874	3.907275	3.432808	2.940364	3.38339	3.302384	3.958175
Itpk1	-0.171295	5.277279	-1.048759	0.311423	0.484838	-6.654971	5.519806	5.090994	5.35265	5.29763	5.187373	5.3068	5.167624	5.258153	5.092089	5.136206	5.468884	5.294831	5.642129	5.10738
Lrguk	0.426231	1.291746	1.048743	0.311431	0.484838	-5.856005	1.278303	1.310208	1.723223	0.531921	1.556544	0.571087	1.607516	1.830204	1.458292	0.699359	1.199219	1.433124	1.943396	1.608903
Hdac10	0.368509	5.575746	1.048354	0.311604	0.485045	-6.626841	5.052887	6.042063	4.926892	4.682885	5.124418	4.863523	6.384613	5.837624	6.269144	6.157876	5.432737	5.648735	5.322519	6.350609
Dohh	-0.151625	5.775905	-1.047514	0.311978	0.485564	-6.68922	6.02812	5.662767	6.043396	6.215363	6.048372	6.125012	5.452543	5.609519	5.704124	5.613537	5.770848	5.518066	5.632699	5.467202
Ddx28	-0.198033	3.634968	-1.046403	0.312474	0.486272	-6.469505	3.793849	3.108509	3.759312	3.810268	3.804231	3.708268	3.445603	3.645768	3.748669	3.856048	3.661913	3.502822	3.640205	3.531125
Rnasek	-0.174034	7.992211	-1.04622	0.312555	0.486286	-6.684391	8.090935	7.518239	7.752919	8.008628	8.261108	7.973907	7.965356	7.922678	8.139224	8.136356	8.01507	8.017761	8.000044	8.094944
Trps1	-0.406312	4.058225	-1.046202	0.312563	0.486286	-6.584978	4.300089	4.332034	4.331892	5.025832	3.511865	4.887611	3.699086	4.145178	3.298245	3.186517	4.13251	3.864689	4.775113	3.425211
Neil1	0.236372	4.018731	1.046016	0.312646	0.486352	-6.487348	3.799454	4.479583	4.139901	3.732068	3.873432	3.53054	4.171806	4.318363	4.096495	4.14048	4.017407	4.065203	3.766298	4.229697
Nfxl1	0.342827	5.694686	1.045686	0.312794	0.486518	-6.662135	5.216821	6.506823	5.932087	5.425809	5.022485	5.212861	6.176005	5.860878	6.08988	5.871458	5.491424	5.518066	5.492026	5.940678
Nfasc	-0.182653	9.283747	-1.04541	0.312917	0.48664	-6.649898	8.900215	9.301586	8.911736	9.469218	8.857908	8.922723	9.438615	9.55422	9.403048	9.324874	9.273312	9.651442	9.459629	9.506503
Acad11	-0.180413	4.11159	-1.045329	0.312953	0.48664	-6.534999	4.428604	4.24827	3.918379	3.959292	3.775594	4.101824	4.213088	4.101219	4.175368	4.382013	4.200538	4.216763	3.949625	3.983274
Trim28	0.1304	7.741891	1.04506	0.313073	0.486764	-6.69027	7.643443	7.688975	7.929518	7.842076	7.914452	7.959776	7.678079	7.708652	7.733251	7.662899	7.668138	7.633839	7.517112	7.813597
Ahctf1	-0.147926	6.251926	-1.044703	0.313233	0.486949	-6.694756	5.978343	6.084399	6.17448	6.503439	5.953551	6.164084	6.512062	6.310282	6.403196	6.323887	6.16864	6.28215	6.313769	6.375469
Dtd2	-0.23052	2.692355	-1.044399	0.313368	0.487038	-6.361118	3.214303	2.439156	2.969885	3.281878	3.159643	3.47381	2.433388	2.488905	2.347926	2.558244	2.274011	2.375822	2.522078	2.402413
Cr1l	0.198463	4.996973	1.04421	0.313453	0.487038	-6.652238	5.132748	4.996138	5.300266	5.240521	5.497557	5.739856	4.51494	4.943446	4.827903	4.888332	4.627162	5.00228	4.44597	4.850685
Wdfy1	0.188652	4.665442	1.04414	0.313484	0.487038	-6.607641	4.707265	5.016996	5.050089	4.616421	4.405982	4.921945	4.718584	4.757708	4.417676	4.449167	4.512193	4.7671	4.582846	4.454537
Iffo2	0.291004	2.730203	1.044086	0.313508	0.487038	-6.347664	2.810225	3.184227	3.81015	3.225913	2.752661	3.47381	2.550752	2.505864	1.819202	2.274406	2.506778	2.816513	2.562371	2.17732
Mrps24	0.170574	4.449657	1.044071	0.313515	0.487038	-6.57403	4.435818	4.730309	4.343444	4.294667	4.733331	4.342635	4.663805	4.323155	4.453722	4.31582	4.591836	4.253292	4.360956	4.5246
Pqbp1	0.153342	5.875093	1.044021	0.313538	0.487038	-6.697474	6.142777	6.125528	6.466912	6.381465	6.529152	6.539971	5.454696	5.693335	5.512366	5.542957	5.675639	5.376215	5.53653	5.301477
Limd2	-0.207909	6.476234	-1.043941	0.313573	0.487038	-6.693134	6.541762	5.595024	5.983749	5.758485	6.283494	5.895902	6.725924	6.949877	6.721692	6.997036	7.08054	6.91952	6.685397	6.547718
Ap4s1	0.190232	3.849934	1.043672	0.313694	0.487162	-6.487871	3.676936	3.864284	3.759312	3.857074	4.262012	3.960324	4.177031	3.583227	3.848317	3.7783	3.976904	3.619352	3.929227	3.716166
Tbc1d9b	0.200967	7.575128	1.043355	0.313835	0.487318	-6.698776	7.716354	7.919698	7.600903	7.050829	7.593022	7.226221	7.737202	7.530036	7.506081	7.574104	7.452859	7.711012	7.691873	7.749942
Baiap2l2	-0.351415	2.764761	-1.043051	0.313971	0.487412	-6.243518	3.100876	3.01022	2.298852	2.363612	2.335271	1.892796	2.644268	2.724465	2.667343	3.802672	3.324422	3.269114	2.466543	3.050898
Fastk	0.239178	7.033222	1.04296	0.314012	0.487412	-6.704083	7.187309	7.510088	7.189128	6.547627	7.054702	6.670023	7.254354	6.886541	7.201421	7.218128	6.849546	6.889536	6.753688	7.265234
Zbtb44	-0.316134	3.562625	-1.04295	0.314017	0.487412	-6.466172	3.263791	3.382986	3.595078	4.328278	3.174343	3.839794	3.601415	3.818604	3.076062	2.945148	3.621611	3.42668	4.061622	3.237699
Nvl	0.32501	4.516695	1.042695	0.314131	0.487526	-6.564854	4.172016	5.346717	4.738513	4.304833	4.209453	3.99299	5.003787	4.806432	4.752736	4.574767	4.411103	4.316033	4.28674	4.388433
Gmeb1	-0.178629	4.351867	-1.042116	0.31439	0.487736	-6.568083	4.123692	4.302134	4.136585	4.688073	4.20588	4.307355	4.614587	4.523808	4.549366	4.468337	4.30603	4.233112	4.229503	4.315206
Atxn2l	0.209613	9.025389	1.04209	0.314402	0.487736	-6.660585	8.734764	9.442199	9.11622	8.956752	8.859899	8.794822	9.274941	9.002409	9.262645	9.186881	8.785309	8.88147	8.7501	9.310096
Tnnt1	0.678938	0.989892	1.042047	0.314421	0.487736	-5.847723	1.341131	1.769471	1.7408	0.22441	0.517419	1.585258	-0.279766	3.081965	1.489496	1.51148	0.885831	1.375373	-0.040977	-0.397871
Cacna1b	-0.186755	7.705203	-1.041964	0.314458	0.487736	-6.70164	7.230992	7.659431	7.289955	7.654087	7.052715	7.016321	8.434884	7.863876	8.221674	7.788715	7.734431	7.680413	8.070129	8.183154
Slc35e3	-0.256461	6.041086	-1.04194	0.314469	0.487736	-6.696061	5.984506	5.254098	5.910893	6.427162	6.120362	6.509555	5.910253	6.028683	5.995221	5.967554	6.041301	6.163475	6.210852	6.07516
Kcnip3	-0.230717	5.017944	-1.041348	0.314734	0.488084	-6.665709	5.599065	5.709075	5.490208	5.349294	4.866025	4.911303	4.732848	4.294162	5.048229	4.800297	4.778847	4.783844	4.931987	5.005709
Krt27	-0.523076	1.125398	-1.041173	0.314813	0.488143	-5.836981	0.307952	0.185409	0.105278	1.837445	0.691028	1.473956	1.938115	1.68885	2.04602	1.355481	1.375754	1.461155	0.921344	1.158276
Lphn2	0.253344	6.061479	1.041054	0.314867	0.488163	-6.693413	5.934448	6.911992	5.919601	5.907124	5.791366	6.079159	6.118774	6.002037	6.266894	6.144828	6.053254	6.017734	5.692921	6.044486
Hnrnpa3	0.156494	7.871669	1.040375	0.315171	0.488481	-6.690222	7.708184	7.927369	8.030337	8.177652	8.27588	8.160039	7.828857	7.563326	7.618851	7.695397	7.823103	7.717563	7.848412	7.835125
Sytl3	-1.509315	-0.86823	-1.040019	0.31533	0.488481	-5.536834	2.445855	-1.463779	-0.86368	-1.788967	-1.400091	-1.26448	-1.009457	-1.196868	-0.773548	1.180545	2.921517	-2.249472	-1.218554	-1.632384
Il12a	-0.333523	2.594673	-1.039995	0.315342	0.488481	-6.287564	2.810225	3.582736	2.31067	2.994284	2.226624	2.297664	3.083269	2.539194	2.539002	2.375352	1.996325	2.375822	2.480628	2.982839
Wibg	0.248656	4.315262	1.039993	0.315343	0.488481	-6.573044	4.576285	5.136182	4.655224	4.030446	4.309397	4.094316	4.253222	4.269548	4.259232	4.394093	4.295089	4.136468	4.018826	4.065483
Cir1	-0.207352	5.531521	-1.039917	0.315377	0.488481	-6.697066	5.861314	5.616442	5.932087	6.418606	5.842885	6.341823	4.994938	5.314586	5.275577	5.126584	5.393952	5.094648	5.322519	4.940566
Snhg4	-0.491635	0.151993	-1.039877	0.315395	0.488481	-5.638815	0.176305	0.185409	-1.093563	0.22441	0.15195	-0.072565	0.851758	-0.380636	1.102347	0.475109	0.676952	0.34469	0.791767	0.190106
RGD1305645	-0.849371	0.515021	-1.039823	0.315419	0.488481	-5.588069	1.644914	-1.947514	-0.86368	-1.223791	-0.038319	0.175273	0.743216	2.16515	-0.386063	2.438927	1.94125	2.489067	0.745852	1.085968
Spg7	-0.119063	5.984437	-1.039788	0.315435	0.488481	-6.689716	5.945863	5.758078	5.88148	5.784144	5.586799	5.754219	6.129595	6.050519	6.152143	6.150773	6.090643	6.12785	6.205562	6.189567
Amer1	-0.367421	1.696384	-1.039781	0.315438	0.488481	-6.103648	2.144973	1.655851	2.067688	2.093442	1.20216	2.071653	1.443753	1.934092	1.663747	0.938085	1.783193	2.170435	2.151407	0.929479
Rassf3	0.226192	4.212941	1.039259	0.315673	0.488782	-6.552836	4.495729	4.414697	4.061636	3.810268	4.325951	4.677595	4.238302	4.050109	4.189688	4.21501	4.309659	4.383501	3.707626	4.187126
Nt5c3a	-0.192496	5.532389	-1.038994	0.315791	0.488903	-6.669651	5.421475	4.721865	5.380951	5.550659	5.589544	5.470877	5.636383	5.63672	5.663659	5.672584	5.602089	5.654816	5.789437	5.696968
Matk	-0.255522	8.552164	-1.038586	0.315975	0.489124	-6.686712	8.41817	8.600188	7.83474	8.165617	8.033119	7.673384	9.340881	8.732359	9.10709	9.029931	8.508168	8.559621	8.593039	9.138479
Terf2	-0.207304	5.078069	-1.037665	0.316389	0.489703	-6.679608	5.337294	4.960691	5.453368	5.925907	5.514986	5.771975	4.682297	4.961999	4.659784	4.782026	4.944375	4.692208	4.836642	4.6173
Arl5a	-0.223251	3.901605	-1.037327	0.316542	0.489829	-6.558051	4.231267	3.747109	4.308508	4.39641	4.074901	4.216915	3.601415	3.825375	3.70281	3.158464	4.004032	3.721386	4.130242	3.610591
Mkln1	-0.15407	5.60804	-1.037302	0.316553	0.489829	-6.689755	5.635554	5.619476	5.697944	5.990312	5.52649	5.797626	5.77033	5.522617	5.510466	5.277674	5.478587	5.292883	5.879898	5.545119
Slc7a8	0.166206	6.980776	1.036818	0.316771	0.490104	-6.710013	6.801179	7.165507	6.955527	6.568872	6.68613	6.596865	7.055214	7.057314	7.186623	7.250049	7.054826	7.127867	6.955014	7.282524
RGD1562726	-0.382183	2.479168	-1.03598	0.317149	0.490624	-6.331882	2.947796	1.725091	3.151703	3.302317	3.152236	2.889836	2.078759	1.71825	2.127623	1.625169	2.627988	1.97042	3.160849	2.530531
B3galnt2	0.254819	5.08322	1.035891	0.317189	0.490624	-6.628647	4.730919	5.601176	4.977811	4.812037	4.817684	4.729403	5.653354	5.25314	5.368723	5.290656	4.765698	5.035286	4.903517	5.273263
Tube1	0.31569	3.334433	1.034822	0.317671	0.491262	-6.363857	3.197423	3.769134	2.645305	2.881078	3.680967	3.365711	3.783728	3.201289	3.525993	3.508843	3.004456	3.127617	3.229186	3.922287
Dyx1c1	-0.241212	1.43541	-1.034742	0.317707	0.491262	-6.009799	1.80948	1.58312	1.451597	1.526009	1.463853	1.221719	1.297885	1.80302	1.489496	1.253084	1.319276	1.461155	1.436856	1.556352
LOC498750	0.192617	4.040255	1.034701	0.317726	0.491262	-6.502691	3.658553	3.97739	4.226666	3.942027	3.981865	3.794184	4.36733	3.852146	4.160904	4.015873	4.272956	4.212646	4.306643	3.890799
Rbck1	0.213672	6.266269	1.034615	0.317765	0.491262	-6.697837	6.178698	6.465268	5.869546	5.536351	6.216714	5.726563	6.618638	6.329461	6.476084	6.595529	6.448379	6.496245	6.28897	6.502428
Fam103a1	-0.231689	4.284165	-1.034408	0.317858	0.491343	-6.597891	4.47136	3.752647	4.346317	4.746422	4.760377	4.639415	4.316148	4.134313	4.14139	4.173561	4.265502	3.906064	4.364758	4.041787
Cpz	-0.75699	0.475718	-1.033852	0.31811	0.491668	-5.783832	2.488111	-0.465897	0.679743	-0.502853	-0.516166	0.881356	-0.399109	0.923327	0.091681	1.540753	1.199219	0.760186	-0.123798	1.415936
Rnf139	-0.235402	5.355431	-1.033148	0.318428	0.492097	-6.67859	5.25607	4.851385	5.273343	5.838805	5.282035	5.846484	5.193638	5.105197	5.49325	5.136206	5.194477	5.379891	5.52128	5.642865
Yeats4	0.139971	4.800028	1.032948	0.318518	0.492173	-6.648042	4.891713	5.048854	4.93264	5.095807	5.323842	5.238629	4.618433	4.464319	4.65291	4.609719	4.675772	4.572035	4.618316	4.514305
Ror1	-0.325356	0.367394	-1.03206	0.31892	0.492731	-5.862017	1.033981	0.24948	0.750864	1.128291	0.917619	1.136566	-0.169545	0.449407	0.317129	0.280604	-0.411343	0.403657	0.249639	0.045082
Ide	0.147277	5.392603	1.031559	0.319147	0.493018	-6.682352	5.375315	5.652436	5.35122	5.550659	5.605903	5.839765	5.321831	5.220124	5.206835	5.148143	5.233205	5.319923	5.485056	5.223575
Bclaf1	-0.239701	7.485721	-1.031387	0.319225	0.49307	-6.705702	7.34938	7.489922	7.786894	8.263986	7.145163	7.840039	7.489876	7.516502	7.506081	7.193572	7.35967	7.24284	7.44845	7.176616
Zzz3	-0.152332	5.91106	-1.031304	0.319262	0.49307	-6.701532	5.696893	5.782647	5.963305	6.232328	5.649957	5.859829	6.136317	5.917426	6.022144	5.929327	5.8651	5.838336	5.871905	6.015581
Fbxl16	-0.216009	8.295729	-1.030984	0.319408	0.493231	-6.685923	8.840988	8.209203	8.864788	8.298099	8.241609	8.113215	8.155376	8.146334	8.079597	8.021972	8.208718	8.269843	8.529249	8.166267
Doc2g	-0.352255	2.759943	-1.03085	0.319468	0.493246	-6.201286	2.832262	2.713546	1.705429	2.13961	2.322129	1.839907	3.537848	2.964197	3.159628	3.440594	2.940364	2.921125	2.760099	3.61702
Sirt3	-0.208533	5.836428	-1.030781	0.319499	0.493246	-6.687773	6.047088	5.64947	5.578391	5.26155	5.478434	5.063233	6.1747	5.958043	6.116358	6.100053	6.2699	5.974628	5.979972	6.086786
Smug1	0.322527	1.333543	1.030548	0.319605	0.493308	-5.867932	1.178614	1.58312	1.195284	0.621404	1.312595	0.734546	1.443753	1.356279	1.794428	1.419918	1.533025	1.542121	1.983515	1.608903
Cstf2	0.281152	7.541065	1.030511	0.319622	0.493308	-6.712351	7.321898	8.210713	7.808934	7.332926	7.168755	7.24202	8.036077	7.302676	7.876235	7.644055	7.041794	7.262388	7.312823	8.022336
Snw1	-0.199685	5.434564	-1.029788	0.31995	0.493751	-6.695647	5.574761	5.146735	5.640576	5.998686	5.780598	5.780186	5.003787	5.414511	5.336908	5.339365	5.457481	5.121244	5.446106	5.07972
Dnaja1	-0.317521	7.906301	-1.029485	0.320088	0.4939	-6.696959	7.880014	7.124984	7.957272	8.674443	8.263043	8.49203	7.814259	7.830609	7.916254	7.432914	7.973928	7.617975	8.048129	7.669112
Anapc4	0.164715	6.155891	1.02908	0.320271	0.49412	-6.70334	5.781445	6.308669	5.979128	5.945532	5.987244	5.843129	6.707084	6.140141	6.465333	6.277907	6.098006	5.979482	6.198122	6.493721
Notch3	0.292404	5.172821	1.028805	0.320396	0.494204	-6.624729	5.154777	5.501094	4.806861	4.144271	4.720868	4.707427	5.524	5.290321	5.72885	5.402519	5.030451	5.645685	5.189491	5.619042
Chrd	0.323946	7.024256	1.028779	0.320408	0.494204	-6.71437	6.312097	7.401915	6.342196	6.292313	6.661559	6.1018	7.797817	7.270787	7.703621	7.603447	6.954676	7.184661	6.91797	7.807989
Nubpl	-0.22457	2.276246	-1.028373	0.320592	0.494425	-6.133111	2.32672	1.833582	1.966737	1.962578	1.822268	2.025031	2.893159	2.454377	2.364852	2.628098	2.556491	2.529342	2.494578	2.341603
Tmbim6	-0.176603	7.355538	-1.027889	0.320813	0.494701	-6.718512	7.418838	6.766985	7.093177	7.11193	7.254266	7.243717	7.159868	7.49171	7.514638	7.507091	7.459408	7.722638	7.598888	7.644149
Armc3	-0.604613	1.216777	-1.027611	0.320939	0.494832	-5.792012	1.48718	-0.572649	-0.491181	0.280432	1.143601	1.07687	1.668148	2.227821	1.393785	1.451089	1.652137	2.330954	2.423441	1.824228
Uhrf2	0.160128	6.628211	1.027502	0.320989	0.494832	-6.717212	6.284389	6.881987	6.549647	6.608395	6.444513	6.553038	7.009081	6.686311	6.909326	6.522206	6.52919	6.488605	6.580581	6.763738
Tmem106c	-0.146498	4.995069	-1.027284	0.321088	0.494832	-6.669146	5.319852	5.023882	5.048326	5.252825	5.338601	5.14726	4.637512	4.662608	4.902249	4.957801	4.797057	5.00228	4.877138	5.01304
Atp5i	-0.18988	6.058911	-1.027277	0.321091	0.494832	-6.715834	6.612711	6.492001	5.848419	6.263067	6.448298	6.205614	6.020459	5.625125	6.127559	5.926557	5.93019	5.698154	5.92952	5.721041
Hars2	0.165354	5.890223	1.02725	0.321103	0.494832	-6.699715	5.741245	6.199368	5.808344	5.694201	5.779396	5.748252	6.254722	5.802024	6.040696	6.02306	5.911209	5.737365	5.747821	6.002137
Celf3	-0.16228	8.192473	-1.027067	0.321187	0.494897	-6.701243	8.146684	7.958852	8.182395	8.170721	7.988066	7.858161	8.524469	8.254708	8.466998	8.405133	8.071994	8.158693	8.010701	8.502485
Rpo1-3	0.155211	5.077491	1.02688	0.321272	0.494963	-6.670569	5.172161	5.099711	5.313541	5.242285	5.422577	5.608994	5.061434	4.836907	4.843082	5.059905	4.988055	4.7671	4.969927	4.746057
Ndufb5	-0.147464	6.310718	-1.02672	0.321345	0.494963	-6.718372	6.603931	6.175859	6.28793	6.518804	6.691888	6.499336	6.19287	6.044728	6.333971	6.187098	6.299399	6.034155	6.264755	6.235205
Nme3	-0.200502	5.105006	-1.026702	0.321353	0.494963	-6.664574	5.581282	5.317123	4.755906	4.888381	4.8728	5.184859	5.012583	5.167806	5.112276	5.426242	5.088633	5.121244	4.919116	5.068037
Rtfdc1	0.143488	5.954618	1.026229	0.321568	0.495231	-6.708166	6.130601	6.055567	6.188959	5.729848	6.174378	5.868657	5.759921	5.925326	5.921344	5.980965	6.103242	5.86743	5.799304	5.8845
Rere	-0.161672	8.211631	-1.025351	0.321968	0.495783	-6.692344	8.263558	8.278021	8.514674	8.799385	8.14323	8.53893	8.085195	8.178751	8.049814	8.08201	7.95812	8.068468	7.947451	8.060572
Bicd2	-0.176921	7.165107	-1.024914	0.322168	0.496027	-6.717794	7.300815	6.990254	7.363067	7.728663	7.406775	7.590428	7.03089	6.965286	6.965889	6.711892	7.03633	7.001738	7.237576	6.992952
Rc3h2	-0.344377	4.137099	-1.024381	0.322411	0.496337	-6.585629	3.870393	4.228535	4.352047	5.048192	3.391209	4.548942	4.34429	4.279444	4.208563	3.530893	4.227643	3.906064	4.58936	3.988241
Nptx2	0.27191	5.839225	1.024289	0.322453	0.496338	-6.65779	5.121607	4.502772	5.771406	4.821509	5.361263	5.293749	6.27801	5.959582	6.5992	6.327041	6.234069	6.512242	6.478996	6.518828
Gpr146	-0.271383	3.499829	-1.024081	0.322548	0.49642	-6.459681	4.214585	3.439014	3.614277	3.099251	3.403739	2.99033	3.261094	3.440579	3.31576	3.734632	3.797302	4.056042	3.4385	3.334499
Mri1	0.164006	4.695376	1.023992	0.322588	0.49642	-6.616956	4.569741	4.664267	5.062374	4.552446	4.633136	4.526803	4.460269	4.81326	4.80328	4.763521	4.822678	4.703986	4.825648	4.594677
Cct8	0.182928	7.469327	1.023795	0.322678	0.496495	-6.717446	7.196895	7.242307	7.600602	7.731806	7.864337	7.880429	7.433683	7.315959	7.446705	7.227723	7.400226	7.2579	7.538731	7.442195
Phpt1	0.135264	6.06272	1.022982	0.32305	0.497003	-6.714534	6.229401	6.28498	6.113381	5.865378	6.171634	6.156895	6.154974	5.926901	6.05641	6.147209	6.009303	5.870046	5.91792	5.997217

Snrpg	-0.524207	0.746477	-1.022839	0.323115	0.497006	-6.090539	1.48718	0.679782	1.269823	2.602113	1.173177	3.076678	0.202532	-0.511025	0.736738	0.893396	-0.616402	0.151783	0.037347	0.383504
Zfp496	0.204481	4.687611	1.022796	0.323135	0.497006	-6.588393	4.630741	4.856537	4.561895	3.967847	4.415294	4.153309	4.937596	4.921495	4.755947	5.039542	4.918469	4.923876	4.707853	4.898966
Wdfy3	-0.18785	8.123982	-1.022549	0.323247	0.497116	-6.710616	7.543461	7.873428	7.800839	8.401583	7.594389	7.860928	8.518564	8.376116	8.385626	8.152743	8.162563	8.272812	8.447346	8.351154
Manbal	-0.185825	6.291826	-1.022282	0.32337	0.497226	-6.718376	6.599922	5.846187	6.172863	5.977659	6.372238	6.014006	6.060593	6.427283	6.389821	6.439139	6.557697	6.461974	6.492961	6.293559
RGD735065	0.125922	6.331374	1.022099	0.323453	0.497226	-6.713647	6.066973	6.061157	6.120952	5.888093	6.177118	6.051319	6.288911	6.616785	6.436602	6.684284	6.517471	6.718641	6.406145	6.624706
Slc11a2	-0.162408	6.021242	-1.022088	0.323458	0.497226	-6.70417	5.7514	5.573282	6.066232	5.829446	5.472498	5.396521	6.218437	6.292067	6.276989	6.303751	6.196346	6.324728	6.257615	6.363093
Arf4	0.214465	6.954747	1.022032	0.323484	0.497226	-6.719615	7.42202	7.066157	7.675632	7.439804	7.95386	7.890488	6.369857	6.454836	6.435601	6.32914	6.835142	6.523087	6.614511	6.36977
Noc4l	0.193425	5.71356	1.021739	0.323618	0.497368	-6.691205	5.41053	5.598103	5.96891	5.455009	5.763686	5.170651	5.927408	5.708064	5.949668	5.922393	5.691023	5.650258	5.686859	6.117717
Prlhr	0.430266	0.487427	1.02158	0.323691	0.49741	-5.812044	0.592497	0.24948	1.574317	0.621404	0.985877	1.165512	0.118118	0.517885	-0.504027	0.413143	0.263333	1.315214	0.111637	0.554014
Tnpo2	-0.11875	7.934409	-1.021499	0.323728	0.49741	-6.712213	7.921401	7.904543	7.940494	7.929534	7.641769	7.74045	8.132695	7.973854	8.026069	7.916348	7.956675	7.850008	8.109186	8.04516
Eif2b5	0.127669	6.044604	1.021315	0.323812	0.497475	-6.717672	6.023338	6.379282	6.065361	6.199104	6.177118	6.33706	6.021912	6.031614	5.965013	5.949932	5.894392	5.832982	5.941026	5.830202
Trpm2	0.251311	5.824585	1.020161	0.324341	0.498224	-6.648273	4.59574	5.548027	4.964781	5.025832	5.062547	4.707427	5.68304	7.602778	5.559075	7.316615	7.028094	7.769979	5.233825	5.481365
Smad5	0.204026	4.237784	1.019953	0.324436	0.498306	-6.609461	4.300089	4.275453	4.996223	4.594489	4.402865	4.917698	3.857182	4.203515	4.018418	3.913127	3.83304	4.087853	4.070962	3.942904
Pcdha13	-0.948881	1.178274	-1.019808	0.324503	0.498346	-5.802272	0.955837	-2.680539	1.089503	1.128291	0.985877	1.541762	1.258992	2.16515	2.55384	1.023513	1.697158	1.783252	2.269694	2.261799
Klhl25	-0.267252	2.749989	-1.018912	0.324914	0.498826	-6.248356	2.843156	2.264105	2.200654	2.389316	2.472441	2.434761	2.629097	3.093229	2.785194	2.681644	3.288097	3.587916	3.160849	2.91141
Klf10	-0.263486	4.866329	-1.018878	0.324929	0.498826	-6.668581	5.099062	4.240408	5.277865	5.312821	5.13948	5.091789	4.320877	4.68139	4.778229	4.472141	4.845355	4.872617	5.165713	4.885716
Scn9a	0.273519	2.498654	1.018853	0.324941	0.498826	-6.284042	2.595092	3.208609	3.042552	2.613062	2.254562	3.006419	2.955303	2.24812	2.330799	2.274406	1.697158	1.950783	2.58862	2.503027
Vmo1	0.598161	0.00194	1.018689	0.325016	0.498878	-5.571156	-0.518419	1.506527	-0.575682	-0.502853	-0.33871	-0.214525	0.282279	0.377517	0.317129	0.534523	-0.231835	0.460309	-0.040977	0.606649
Smim11	0.219898	3.573828	1.017682	0.325478	0.499524	-6.495907	3.854329	4.150978	3.661186	3.456176	3.981865	3.698398	3.616879	3.606999	3.548392	3.545408	3.345785	3.405197	2.827778	3.468514
Lama1	0.29627	2.3773	1.017522	0.325552	0.499573	-6.158252	2.357438	1.991799	2.423812	1.740915	2.268331	2.570814	2.991343	2.16515	2.94004	2.733273	2.332254	2.118577	2.236876	2.721621
Dcps	-0.172027	4.42756	-1.017127	0.325733	0.499782	-6.635179	4.958358	4.449211	4.71425	4.586178	4.718362	4.495797	4.118484	4.279444	4.175368	4.046664	4.295089	4.253292	4.689778	4.279179
RGD1562310	0.234265	4.872067	1.016946	0.325817	0.499782	-6.610243	4.421353	5.071186	4.860105	4.4153	4.553844	4.135132	5.135774	5.331332	5.192685	5.194928	4.667312	5.014154	5.123695	5.147901
Smarca4	-0.144201	8.279208	-1.016868	0.325853	0.499782	-6.707379	8.142005	7.957953	8.145753	8.474505	8.105856	8.44503	8.401512	8.357551	8.328262	8.322477	8.384163	8.273059	8.330448	8.245424
Gda	-0.426682	5.424533	-1.016852	0.32586	0.499782	-6.701104	5.050539	4.554415	6.074054	6.398178	5.016379	5.541588	5.271424	5.709895	5.570064	4.657271	5.615344	5.233174	6.037083	5.252767
Def8	0.236121	5.834026	1.016614	0.325969	0.499782	-6.694408	5.462675	6.18715	5.691179	5.316175	5.570221	5.059383	6.249771	5.988526	6.20597	6.191719	6.014871	5.856918	5.813285	6.09717
Asb2	-0.343161	1.956189	-1.01661	0.325971	0.499782	-5.926872	1.619793	1.117652	0.851376	0.966949	1.230571	0.61372	2.828218	3.115498	2.41447	3.080873	2.892776	2.691938	2.022549	2.431883
Slco4c1	-0.2748	1.354743	-1.016592	0.325979	0.499782	-5.952632	1.178614	1.425638	0.945338	1.548999	0.731352	1.378286	1.754571	1.112357	1.819202	1.419918	1.482482	1.433124	1.626159	1.73247
Nr2c1	-0.166034	4.70192	-1.016167	0.326175	0.500019	-6.650584	4.667979	4.747052	4.804774	5.168122	4.53688	4.93881	4.781687	4.58892	4.861089	4.606261	4.53717	4.296718	4.582846	4.769331
Hipk1	-0.127681	7.462751	-1.015733	0.326375	0.500261	-6.724204	7.652761	7.517426	7.587304	7.638765	7.349231	7.619485	7.476164	7.349814	7.27434	7.262183	7.302091	7.445858	7.609743	7.402276
Snrnp70	0.234639	8.998299	1.015169	0.326634	0.500595	-6.690773	8.498747	9.463882	8.984896	9.016477	8.663215	8.842654	9.332417	9.12667	9.296948	9.202134	8.717784	8.855964	8.774602	9.202932
Commd10	-0.277391	4.965261	-1.014994	0.326715	0.500655	-6.691258	5.282322	4.37216	5.274852	5.662929	5.469522	5.653096	4.739927	4.754164	4.849109	4.417954	5.010524	4.461895	4.937104	4.680048
Ccrn4l	-0.287019	4.531163	-1.014255	0.327056	0.501113	-6.635488	4.70129	3.74155	4.919194	4.87707	4.720868	4.592225	4.618433	4.452657	4.549366	4.086912	4.850346	4.060629	4.885102	4.350357
Ptrhd1	0.24034	2.051166	1.014045	0.327153	0.501198	-6.140231	2.144973	1.812527	2.262807	1.799605	2.295481	2.386402	2.100922	1.71825	1.891068	2.14695	2.259075	1.97042	2.060555	2.245294
Pelp1	0.180088	7.259291	1.013793	0.327269	0.501311	-6.731898	7.056519	7.693301	7.305541	7.335407	7.193772	7.307602	7.351143	7.175964	7.452655	7.32084	7.017039	7.066465	6.919263	7.444908
Nadk	0.151018	6.314326	1.013677	0.327323	0.50133	-6.724363	6.273357	6.201394	6.176096	5.850418	6.364247	6.104605	6.570807	6.411621	6.317764	6.534992	6.418445	6.502159	6.164705	6.529943
Srsf11	0.196919	7.807448	1.013297	0.327498	0.501513	-6.72244	7.527087	8.214229	7.783188	7.931996	7.699759	8.00101	8.063845	7.88631	7.933021	7.841498	7.651078	7.610954	7.404334	7.763045
Myadm	-0.293244	6.913897	-1.013236	0.327526	0.501513	-6.734585	6.885272	6.015821	6.952702	7.404788	7.337033	7.100235	6.552831	6.68817	7.161414	6.78787	6.744887	6.886309	7.075337	7.215264
Sugp1	0.172727	5.774106	1.012792	0.327731	0.501764	-6.712567	5.678764	6.2433	5.833137	5.6695	5.608612	5.707007	5.977681	5.819086	5.817587	5.8929	5.737561	5.595984	5.558277	5.726997
Faf2	-0.154058	5.556952	-1.012694	0.327776	0.501769	-6.715261	5.594237	5.574846	5.645251	6.054978	5.547816	5.921583	5.290835	5.503746	5.430321	5.333104	5.424393	5.446256	5.623207	5.440265
Zfp263	0.166404	4.64449	1.012314	0.327952	0.501974	-6.620775	4.519693	4.936565	4.534442	4.455398	4.617084	4.537915	4.757474	4.785751	4.60741	5.016284	4.706374	4.830257	4.250206	4.531422
Cx3cr1	0.321048	5.334572	1.011984	0.328104	0.502105	-6.687037	5.626123	5.144631	5.34405	4.743933	5.810314	5.687181	4.263083	5.991539	4.421726	5.553767	5.652966	6.615626	5.073464	4.797905
Tfap4	0.28281	3.262035	1.011948	0.328121	0.502105	-6.392208	3.109925	4.000847	3.091325	3.035565	3.091571	3.288866	3.251219	3.630385	3.169295	3.802672	3.057213	3.179156	2.816716	3.310901
Mgat5	-0.335153	4.646298	-1.011334	0.328405	0.502475	-6.633562	4.474867	3.929299	4.770955	5.040101	4.05518	4.92618	4.403445	5.051345	4.628589	4.086912	4.969824	4.901034	5.280867	4.594677
Rhbdd1	0.384592	1.691091	1.011068	0.328528	0.502599	-6.077164	1.9771	1.679303	2.36835	1.353927	1.78444	2.203108	1.408645	1.71825	0.97585	0.938085	1.581856	2.447635	1.983515	1.755962
Atf6	-0.307616	5.268514	-1.010423	0.328826	0.502992	-6.698102	5.35833	4.71337	5.900178	5.679952	4.993765	5.221502	4.764433	5.421231	5.129711	4.85376	5.305858	5.433932	5.846296	5.178649
Mrps18a	0.163919	5.712701	1.010288	0.328889	0.503023	-6.711051	5.865327	5.771779	5.688917	5.317849	5.945004	5.602394	5.883333	5.805453	5.591793	5.756075	5.894392	5.575293	5.692921	5.61744
Acvr2b	-0.395816	0.7613	-1.010083	0.328984	0.503104	-5.846639	1.071521	1.21714	0.750864	0.823477	-0.33871	0.695385	0.68572	0.871929	1.061401	0.348395	1.03357	1.082389	1.150071	1.158276
LOC257650	0.310474	3.592059	1.009674	0.329173	0.503295	-6.419047	3.118917	4.283127	3.228468	3.247155	3.452798	3.091837	3.895725	3.575215	3.941526	3.946322	3.84309	3.625558	3.220819	3.953102
Pphln1	0.135233	5.198355	1.009531	0.329239	0.503295	-6.699137	5.278315	5.419891	5.521053	5.476153	5.679885	5.432691	5.259158	4.873301	4.964647	4.772804	5.086519	4.90359	5.110168	5.041995
Enkd1	0.323605	2.688979	1.009489	0.329259	0.503295	-6.21546	2.542593	3.142653	2.298852	1.945353	2.781752	2.04074	3.049479	3.201289	3.01701	2.889346	2.853541	2.643831	2.480628	3.012401
Rps6ka4	0.186551	5.570576	1.009452	0.329276	0.503295	-6.697221	5.615043	5.851358	5.44669	5.050208	5.4917	5.277267	5.675675	5.559634	5.508563	5.898564	5.654393	5.864809	5.435298	5.692409
Ctdp1	0.113199	5.285143	1.008927	0.329519	0.503603	-6.684935	5.137181	5.190235	5.414195	5.195699	5.276936	5.233512	5.180689	5.445606	5.259827	5.396526	5.416002	5.346466	5.292891	5.246561
Stpg1	-0.336673	3.26096	-1.008665	0.32964	0.503725	-6.471317	4.214585	3.452688	3.642605	3.107017	3.060255	3.045873	2.841442	3.180315	2.772565	2.988286	4.21995	3.10114	3.089114	3.106782
Mospd2	0.177119	4.158311	1.008521	0.329708	0.503763	-6.573098	4.369549	4.350411	4.490959	3.810268	4.086605	4.094316	4.150713	3.991112	4.081214	4.150009	4.30603	4.220868	4.028447	4.174106
Tgfbr1	-0.331745	5.478997	-1.008289	0.329815	0.503863	-6.716651	5.586154	4.817439	5.133963	6.057986	5.74903	6.261147	5.047237	5.632865	4.771898	5.603168	5.473744	6.377163	5.110168	5.121014
Ube2d2	-0.233536	6.036579	-1.008167	0.329872	0.503885	-6.738491	6.4351	5.850067	6.566379	6.989127	6.527006	6.970411	5.653354	5.611479	5.627297	5.513729	5.74562	5.605434	5.840847	5.601319
Vps4a	-0.143389	6.740135	-1.00802	0.32994	0.503926	-6.740168	6.912033	6.617185	6.980711	7.156584	6.9777	6.988242	6.511028	6.726654	6.651364	6.442051	6.71715	6.561627	6.658544	6.476147
Mlf1	1.340091	0.739106	1.007823	0.330031	0.503953	-5.579992	2.230432	-1.10215	0.484899	-2.731185	1.822268	0.61372	1.047044	2.030999	0.17081	0.348395	2.031907	2.00891	2.133689	1.260365
Slc8a3	-0.316128	4.209437	-1.007801	0.330041	0.503953	-6.617704	4.026686	4.907073	4.583975	5.115191	3.943761	3.668376	3.981847	3.966821	4.170562	4.106622	3.877725	4.212646	4.08486	4.373323
Clcn7	0.156722	6.176815	1.007644	0.330114	0.503997	-6.713207	5.610268	5.894578	5.996609	5.556344	5.532208	5.534671	6.482823	6.466794	6.670707	6.551265	6.503281	6.690057	6.361172	6.647651
Nos1ap	-0.230962	6.394188	-1.007558	0.330154	0.503997	-6.733382	6.133932	5.905805	6.321172	6.493104	5.948215	6.155993	6.680655	6.913005	6.700181	6.281163	6.884028	6.203436	6.747149	6.170026
Arid3b	-0.218844	3.567463	-1.007201	0.33032	0.504141	-6.445385	3.495497	3.758163	3.353699	3.437817	3.036313	2.881136	3.783728	3.904241	3.735714	4.056832	3.695584	3.843548	3.431265	3.667444
Atp6v0c	-0.195524	10.335	-1.007174	0.330333	0.504141	-6.642554	10.43791	9.796678	10.31785	10.39408	10.52108	10.25426	9.937263	10.33373	10.4824	10.49747	10.3793	10.46054	10.43294	10.44573
Taok1	-0.352793	5.860947	-1.006832	0.330491	0.504319	-6.73342	5.761485	5.700505	6.166373	6.888513	5.533634	6.49364	5.94895	5.837624	5.882203	4.839106	5.71174	5.788042	6.183126	5.346662
Dusp14	-0.299332	1.048728	-1.005323	0.331193	0.505326	-5.93141	1.619793	0.679782	1.089503	0.896996	0.917619	1.07687	0.68572	1.067371	1.29127	1.180545	1.581856	1.404538	1.150071	0.799858
Crh	-0.858635	0.346053	-1.005174	0.331262	0.505368	-5.69398	0.915118	-2.680539	-0.007587	0.041976	0.470544	1.221719	0.118118	0.705741	0.449965	1.14286	0.924208	1.045835	0.879431	1.582867
Fkbp14	-0.243464	2.709093	-1.004258	0.331689	0.505954	-6.347366	3.214303	2.532247	2.401882	2.77782	2.781752	3.268997	2.398025	2.849305	2.330799	2.499819	2.739799	2.941167	2.676906	2.756979
Gtf2i	-0.153267	8.618221	-1.003986	0.331816	0.506024	-6.71473	8.472239	8.719161	8.258538	8.529361	8.215861	8.190725	8.999751	8.750704	8.861824	8.810514	8.635062	8.71902	8.676864	8.819547
Guf1	-0.147389	4.971224	-1.003979	0.331819	0.506024	-6.688533	5.033995	4.98677	4.999877	5.424255	5.064521	5.288824	5.167624	4.799571	4.919536	4.571225	4.845355	4.653252	4.959907	4.932857
Map3k1	0.387	1.13699	1.003751	0.331925	0.506087	-5.881441	1.033981	1.702379	1.363571	0.621404	1.258434	0.481842	1.443753	1.356279	1.393785	0.981432	1.290185	1.805289	0.791767	1.122575
Sec22b	-0.195824	5.241507	-1.003705	0.331947	0.506087	-6.698479	5.41053	4.738705	5.316475	5.357456	5.346735	5.33574	4.989008	5.373521	5.197417	5.057375	5.563099	5.204441	5.483308	5.049144
Ccdc101	0.167878	4.2237	1.003619	0.331986	0.506087	-6.593435	4.247758	4.104095	4.551972	4.155549	4.548211	4.247696	4.27775	4.061624	4.334197	4.241995	4.196624	3.970866	3.954679	4.323092
RGD1307439	0.671361	0.226664	1.003299	0.332136	0.50625	-5.692675	0.243629	1.532513	1.386086	-0.325128	-1.087961	0.571087	0.563361	0.04768	0.787836	0.055728	0.001431	0.218993	0.182287	0.442608
Cfp	0.29668	4.446055	1.002795	0.33237	0.506544	-6.65221	3.870393	4.658378	3.963892	3.527359	3.89578	3.47381	5.286006	4.624294	4.956296	4.971302	4.579866	4.758654	4.536402	5.221467
Atp5sl	0.244127	5.929011	1.002509	0.332504	0.506683	-6.734897	6.165739	6.725346	6.521957	5.855038	6.180763	5.587765	5.777228	5.689629	5.974693	5.65261	6.002593	5.644157	5.629542	5.625434
Nif3l1	0.210322	3.856072	1.002281	0.332611	0.506781	-6.518473	3.805036	4.086661	3.656563	3.589663	4.244704	3.648009	4.051782	3.858761	4.055381	3.90752	3.717602	3.727169	3.576053	4.169739
Lrrc14	0.18978	4.56362	1.001827	0.332823	0.507014	-6.579559	4.050381	4.652466	4.266652	4.109897	4.227186	3.956189	4.90017	4.83019	5.042981	4.916522	4.913708	4.701051	4.549825	4.842478
Etfdh	-0.13458	5.541215	-1.001773	0.332848	0.507014	-6.723478	5.830169	5.608829	5.44401	5.683852	5.90149	5.536057	5.419857	5.359594	5.549853	5.378399	5.550887	5.390862	5.505866	5.4511
March8	-0.156861	6.557777	-1.00134	0.33305	0.507204	-6.745407	6.693185	6.716163	6.266095	6.692101	6.691888	6.471354	6.558848	6.495733	6.417463	6.669451	6.295744	6.750224	6.442581	6.664818
Gpt	-0.310424	5.669174	-1.001325	0.333057	0.507204	-6.701995	5.696893	5.742856	4.472666	5.177373	5.403987	4.909165	6.413678	5.700719	6.256728	6.141249	5.576715	5.704031	5.701967	6.504163
Pcdha4	-0.415414	1.131205	-1.001181	0.333124	0.507242	-5.846423	0.592497	0.118359	0.914692	1.245516	0.091273	0.982423	1.888013	1.393205	1.326257	0.847278	1.581856	1.783252	2.168911	1.684306
Sall1	-0.177353	6.001764	-1.000357	0.333509	0.507764	-6.735569	6.252057	5.904562	5.626462	5.89147	5.977847	6.067704	5.293243	6.583223	5.319649	6.528613	6.380994	6.822912	5.673125	5.728482
Agk	-0.16118	5.029337	-0.999954	0.333698	0.507987	-6.687492	5.212627	4.766343	5.115554	5.124787	5.290494	4.847983	5.018416	5.113519	5.032426	4.984678	5.182653	4.846288	5.128176	4.795073
Ostc	0.235013	5.561633	0.999534	0.333894	0.508221	-6.73727	5.819176	5.681759	5.933043	6.159507	6.519109	6.764697	5.167624	5.028055	5.008391	5.169384	5.006058	5.164508	5.161347	5.315379
Tns1	0.307963	5.099096	0.999364	0.333974	0.508278	-6.695489	5.842436	5.797457	5.583271	4.4153	5.148815	5.170651	4.989008	4.747049	5.271094	4.722594	4.703619	5.243299	5.057107	4.743122
Tmem186	0.197184	4.57048	0.99908	0.334107	0.508416	-6.627914	4.759952	4.882026	4.596097	4.098255	4.745687	4.254448	4.498338	4.60868	4.523219	4.513333	4.809924	4.536248	4.695828	4.531422
Mtfr1l	0.126307	6.025185	0.998892	0.334195	0.508485	-6.736323	6.137255	6.026147	6.182542	5.774423	6.164288	5.948896	5.962631	5.985507	5.967785	6.00085	6.134267	6.024794	6.068898	5.998448
Syngr4	-0.47719	-0.032089	-0.998694	0.334287	0.508562	-5.754236	0.428583	-0.572649	1.169557	0.664146	0.210178	-0.457681	-0.831021	-0.048075	0.317129	-0.210791	0.001431	-0.24119	0.313987	0.442608
Med4	0.15322	4.173288	0.997937	0.334642	0.509037	-6.559835	4.007445	3.972652	4.123244	3.866255	4.28928	3.956189	4.438678	4.244507	4.46554	4.285878	4.254248	4.296718	4.233667	4.079517
Mlf2	-0.168232	9.195486	-0.996879	0.335139	0.509728	-6.683997	9.624448	9.02441	9.527898	9.546129	9.578085	9.546314	8.930859	9.07842	9.084596	8.925431	9.209314	8.877737	9.106313	8.769567
Styxl1	0.318846	2.90037	0.996399	0.335364	0.510006	-6.244946	2.445855	3.432128	2.538805	2.184345	2.124346	2.469996	3.383673	3.413873	3.01701	3.401235	2.873292	3.269114	3.003904	3.270693
RGD1308117	0.426422	2.002308	0.996063	0.335521	0.510151	-6.075409	1.996747	2.963229	1.723223	1.158502	1.912714	1.402804	2.343298	1.934092	1.743567	2.628098	2.332254	1.826994	2.003164	2.474989
Pih1d2	0.219232	2.653907	0.996014	0.335544	0.510151	-6.2868	2.947796	2.769246	2.578448	2.124384	2.828971	2.762997	2.501617	2.555575	2.55384	2.720538	2.949696	2.749899	2.66462	2.697558
Gfra4	0.393755	2.390666	0.995294	0.335882	0.510601	-6.038579	1.619793	2.47979	1.981598	1.245516	1.415175	1.402804	3.356296	2.571773	3.120298	3.214035	2.930971	2.63155	2.549064	3.294952
Kank3	0.345615	4.249018	0.995094	0.335976	0.510624	-6.550217	4.110227	5.064523	3.867281	3.504019	3.986037	3.703341	4.822272	4.044317	4.73657	4.595839	3.792123	4.376159	4.233667	4.737232
Rpap3	0.220273	6.594824	0.995081	0.335983	0.510624	-6.747174	6.182992	7.071706	6.27517	6.331789	6.349749	6.21862	7.15458	6.644808	6.877981	6.843256	6.496133	6.538791	6.522196	6.836473
Kat8	-0.120684	4.883214	-0.994931	0.336053	0.510667	-6.66511	4.777095	4.788078	4.673644	4.886126	4.677668	4.71479	5.114144	4.921495	4.925253	5.072488	5.037033	4.848943	4.942203	5.039604
Riok2	0.162166	4.225154	0.994451	0.336279	0.510945	-6.621955	4.439412	4.099756	4.734132	4.267203	4.5954	4.594888	4.074361	4.090016	4.160904	4.015725	4.116027	3.931333	4.070962	4.046557
Ranbp17	0.291888	2.481155	0.994268	0.336365	0.511012	-6.22999	2.054131	2.914655	2.795835	2.43939	2.295481	2.18908	3.231264	2.16515	2.446625	2.600561	2.243983	2.461578	2.348615	2.83626
Socs1	0.341811	1.373637	0.994147	0.336422	0.511033	-5.993401	1.371547	1.425638	2.262807	1.158502	1.510943	1.107027	1.21902	1.020936	1.520039	1.419918	1.674823	1.848377	1.040235	1.260365
Efnb1	0.21374	4.263586	0.993855	0.336559	0.511098	-6.591605	4.260004	3.919485	4.467397	3.954995	4.606283	4.244308	3.882992	4.239446	4.259232	4.290193	4.180861	4.565594	4.330171	4.571694
Mtx2	-0.182591	4.149684	-0.993778	0.336596	0.511098	-6.577321	4.007445	3.649562	3.89109	4.246256	4.147475	4.094316	4.468816	3.960684	4.338488	4.14048	4.483567	4.028205	4.51603	4.212819
E2f3	-0.280314	3.155094	-0.993737	0.336615	0.511098	-6.409278	2.957854	2.690649	3.301354	3.538889	2.640673	3.255598	2.943086	3.395791	3.130231	2.956054	3.474203	3.261164	3.822677	2.982839
Pitpna	-0.195448	8.063387	-0.993695	0.336635	0.511098	-6.721026	8.419763	8.079984	8.41641	8.858667	8.61014	8.666543	7.626014	7.84067	7.744208	7.505236	7.882173	7.706994	8.027289	7.509394
Efcab2	-0.365526	1.427939	-0.99336	0.336792	0.511273	-5.968163	1.143792	1.480065	1.613009	1.593908	0.15195	0.881356	1.478026	2.186344	1.549949	1.852296	1.762161	1.667719	1.747621	1.501815
Ganc	-0.250722	4.923892	-0.992204	0.337337	0.512035	-6.669548	4.785591	5.190235	4.957283	5.003121	4.01082	4.440619	5.188472	5.328951	5.152036	5.201817	5.058757	4.96361	4.879797	4.836982
Akr1e2	-0.162163	4.387789	-0.991421	0.337706	0.512531	-6.62881	4.559869	4.652466	4.210987	4.399576	4.387176	4.03673	4.498338	4.327931	4.457672	4.502215	4.454361	4.187698	4.357143	4.472374
Izumo1	0.503392	0.860755	0.990336	0.338219	0.513236	-5.908724	1.033981	2.619695	1.245402	0.664146	1.258434	0.336685	0.952703	0.923327	1.218642	0.280604	0.676952	0.567345	0.375586	0.799858
Trip4	-0.157914	4.07891	-0.990255	0.338257	0.513236	-6.631674	4.388265	4.08227	4.440757	4.600004	4.409093	4.410708	3.756062	3.94833	3.780555	3.772142	3.949256	3.859433	4.178567	3.62342
Dlk2	-0.292951	5.259373	-0.989601	0.338566	0.5136	-6.662768	5.007607	5.218524	4.308508	4.616421	4.651639	3.926902	6.004382	5.460906	6.04464	5.895735	5.445986	5.621048	5.387506	6.087944
Ubac2	-0.168281	6.46061	-0.989565	0.338583	0.5136	-6.748908	6.394944	5.576408	6.110004	5.956321	6.197951	6.136937	6.778436	6.711201	6.709321	6.824038	6.669231	6.717189	6.788075	6.897159
Pltp	-0.343465	7.047346	-0.989373	0.338674	0.5136	-6.755148	7.2577	6.65464	5.691179	5.914888	6.645163	5.979783	7.214252	7.973092	7.327652	7.931623	7.445457	8.102793	6.888558	7.650039
Otud6b	-0.327683	5.399593	-0.98932	0.338699	0.5136	-6.7	5.960945	5.302095	6.265336	6.885129	5.987244	6.826606	4.77481	4.961999	4.913797	4.421893	4.937355	4.680333	5.163531	4.555051
Gin1	0.227439	3.107878	0.989286	0.338715	0.5136	-6.387497	2.742017	3.445867	3.164784	3.175097	3.152236	3.038068	2.854546	3.070611	3.464507	3.186517	3.250834	2.643831	3.416687	3.088394
Srpk1	-0.158925	6.052137	-0.989099	0.338803	0.5136	-6.751802	6.051792	5.900826	6.046933	6.533287	6.180763	6.409177	5.86078	6.022804	6.045953	5.83506	5.955889	5.980693	6.079349	5.854889
Arhgap21	-0.149544	9.567197	-0.989047	0.338827	0.5136	-6.6947	9.28632	9.444553	9.369461	9.7162	9.206809	9.377982	10.00374	9.665826	9.913991	9.588811	9.468537	9.42878	9.623915	9.847929
Cnot7	-0.214459	6.113816	-0.989024	0.338839	0.5136	-6.757605	6.44049	5.902072	6.478745	6.989127	6.783429	6.837327	5.927408	5.693335	5.794348	5.610089	5.786556	5.696681	5.887848	5.789517
Pbx3	0.220313	3.896922	0.988839	0.338926	0.513668	-6.581685	4.218773	4.368558	4.123244	3.786283	4.361713	4.017012	2.905804	3.599118	3.729193	4.000002	4.13251	4.285003	3.627601	3.510555
Gipr	0.542574	4.269538	0.988604	0.339037	0.513678	-6.593424	3.917539	6.162401	4.217279	4.074684	3.639189	4.220367	4.527267	3.475434	4.824848	4.588849	3.656224	4.468815	3.719553	4.369521
Txnl1	-0.17803	6.45668	-0.988568	0.339054	0.513678	-6.759339	6.714847	6.331051	6.726027	7.051332	6.890247	6.872864	6.427994	6.155	6.307951	5.881504	6.319335	6.059589	6.541641	6.132376
Htra2	0.265333	5.879241	0.988505	0.339084	0.513678	-6.712557	5.600671	6.021567	5.595399	4.858787	5.50339	4.845749	6.426897	6.06918	6.377364	6.387707	6.081121	6.106932	6.03231	6.431253
Fxyd6	0.401553	5.133646	0.988279	0.339191	0.513678	-6.712209	6.02812	6.138277	5.950144	4.351348	5.088003	5.134507	4.443022	4.468651	4.913797	5.044659	4.872595	4.755828	5.294885	5.434817
Stk32a	-0.38641	0.962736	-0.988254	0.339203	0.513678	-5.877001	0.873216	0.533043	1.061799	1.097434	0.091273	0.772673	0.743216	1.464337	1.102347	0.750369	1.068256	1.433124	1.963595	1.355706
Arhgef18	-0.128501	5.988894	-0.988226	0.339216	0.513678	-6.749471	6.26528	5.919409	5.987435	5.994505	6.052357	6.043544	5.865643	5.825855	5.764364	6.048759	6.191437	5.977057	5.963768	5.969852
Lcmt1	0.158123	5.684283	0.988154	0.33925	0.513678	-6.746148	5.841078	5.73728	6.248538	6.003895	6.162446	6.17213	5.570406	5.387314	5.436339	5.388498	5.618273	5.281137	5.554953	5.208755
Nt5c3b	0.168517	5.198205	0.988102	0.339274	0.513678	-6.723955	5.392103	6.028431	5.437287	5.455009	5.402427	5.512311	4.937596	4.921495	4.875924	5.222288	5.073772	4.883016	4.695828	4.981001

Pla2g6	-0.164763	5.871828	-0.988008	0.339319	0.513681	-6.731415	5.752845	5.727471	5.171711	5.550659	5.692706	5.364112	5.988123	6.104401	6.211829	6.252697	6.197325	6.046928	5.916626	6.25595
Tmem86b	0.387471	1.977541	0.987892	0.339374	0.513699	-6.071793	1.310059	2.466372	2.250588	1.760745	1.643636	1.378286	2.01015	2.307359	2.858731	2.40749	1.740817	1.869448	1.770732	2.325991
Lmo1	0.157849	5.084442	0.987379	0.339617	0.513947	-6.686355	5.041108	4.853963	5.165217	4.763722	5.305596	4.896271	5.105949	4.915162	5.458191	5.112031	5.134364	5.096883	5.152576	5.227782
Aldoa	-0.1867	10.22291	-0.987366	0.339623	0.513947	-6.664214	10.42907	9.913449	10.18276	10.29163	10.63253	9.971347	10.21384	10.16591	10.49578	9.9829	10.32178	9.981332	10.34671	10.19295
Ppa2	-0.180905	4.7296	-0.987133	0.339734	0.514049	-6.665713	4.870572	4.339413	4.31439	4.690659	4.945304	4.793392	4.881086	4.60868	5.042981	4.891176	4.747086	4.549364	4.754973	4.845219
Bmp6	-0.306035	4.026024	-0.986661	0.339957	0.514323	-6.567914	4.748409	3.512664	3.550922	3.400384	3.506066	4.40465	4.085519	4.112335	3.96946	4.228565	4.287749	4.390807	4.004274	4.262873
Arpc1a	-0.165019	7.733126	-0.986244	0.340155	0.514557	-6.747844	7.824408	7.378825	7.761555	8.017927	7.873937	8.012009	7.804163	7.716414	7.798555	7.600843	7.813551	7.536747	7.644175	7.488071
Mettl13	0.141282	4.575571	0.985907	0.340315	0.514734	-6.643069	4.358202	4.368558	4.742881	4.563778	4.675086	4.618632	4.729295	4.743479	4.492744	4.687033	4.664481	4.565594	4.481425	4.432837
LOC498675	0.447802	0.597276	0.985348	0.34058	0.515071	-5.784617	0.428583	0.812964	-0.130036	0.22441	1.685287	0.734546	0.68572	0.705741	0.628769	0.750369	0.584127	0.403657	0.745852	1.158276
Mprip	-0.109991	8.330453	-0.984895	0.340795	0.515331	-6.739357	8.287932	8.075572	8.156639	8.293639	8.166854	8.325278	8.526517	8.438446	8.446274	8.387617	8.298878	8.401347	8.401347	8.424915
Gpc2	-0.369882	2.606808	-0.984261	0.341096	0.515721	-6.209234	2.658139	2.790938	1.841991	1.996424	1.556544	1.688586	3.30948	3.349568	2.973531	3.344264	2.81321	3.277021	2.220182	2.962789
Dhdds	-0.15782	5.567975	-0.984017	0.341212	0.515822	-6.738357	5.548378	5.230479	5.522324	5.86995	5.636749	5.817141	5.417651	5.642482	5.586392	5.551971	5.693802	5.48943	5.629542	5.348595
Minpp1	-0.280817	3.296076	-0.98394	0.341249	0.515822	-6.401049	3.073383	1.972946	3.171281	3.268089	3.020129	3.334188	3.374605	3.475434	3.448717	3.48645	3.639021	3.637891	3.689548	3.722142
Usp36	0.193633	6.159068	0.982884	0.341751	0.516516	-6.755181	5.915219	6.633786	6.47678	6.206353	5.946075	5.965451	6.43891	6.131972	6.32643	6.052575	5.957046	5.969758	5.952442	6.276401
Slc35b1	0.118764	6.596915	0.982384	0.341989	0.516811	-6.76159	6.426976	6.548807	6.428787	6.38626	6.590723	6.533046	6.77327	6.453744	6.774901	6.786343	6.623316	6.624949	6.59522	6.8268
Ap2a1	-0.14315	8.019484	-0.982218	0.342068	0.516865	-6.751425	8.008052	7.727449	8.034129	7.851931	7.892949	7.496725	8.264025	8.148023	8.332302	8.01905	8.159801	7.898222	8.236559	8.209715
Phka2	-0.270532	3.810391	-0.982081	0.342133	0.516899	-6.488426	3.188908	3.34683	3.767911	3.963576	2.800826	3.353184	4.425566	4.351577	4.37654	3.676473	4.03505	3.738666	4.314529	4.125333
Ccdc146	0.295004	3.375116	0.981699	0.342315	0.517109	-6.357074	2.957854	3.472958	2.31067	2.568755	3.273255	3.214637	4.14005	3.720315	3.641623	3.417108	3.695584	3.529544	3.640205	3.831222
Rnf8	-0.170915	5.096944	-0.980716	0.342783	0.517607	-6.69297	5.174319	5.326436	4.9741	5.076158	4.705768	4.820946	5.162364	5.151733	5.326146	5.416404	5.252185	5.007041	5.061799	4.948235
Ubc	0.267846	5.377303	0.980675	0.342803	0.517607	-6.609862	5.002757	5.457677	4.907568	4.249769	4.897375	4.294309	5.992576	5.849298	5.76755	6.065221	5.876155	5.754456	5.437105	5.77234
Dmap1	0.181013	4.557709	0.980669	0.342806	0.517607	-6.638744	4.569741	4.982063	4.598509	4.174153	4.342316	4.281144	4.637512	4.604749	4.787674	4.571225	4.612548	4.475702	4.649823	4.588148
Atp9b	0.217225	6.339588	0.980615	0.342832	0.517607	-6.75352	5.755731	6.719702	6.041624	6.060988	6.111796	5.638975	6.549813	6.533437	6.684255	6.633388	6.371478	6.674427	6.366871	6.631866
Taf1b	-0.148301	3.452644	-0.980555	0.34286	0.517607	-6.480442	3.453461	3.200527	3.426105	3.527359	3.372207	3.365711	3.577905	3.583227	3.662308	3.602051	3.324422	3.447848	3.379583	3.558103
Hsp90b1	-0.249806	9.519174	-0.980255	0.343003	0.517758	-6.701516	9.396733	8.846326	8.893189	9.768173	9.782683	9.804175	9.442146	9.573578	9.644613	9.635563	9.487183	9.656471	9.485726	9.854065
Mrp63	0.188154	4.61563	0.980078	0.343088	0.517798	-6.671606	4.862563	4.449211	4.955402	4.458437	5.109198	4.843511	4.595201	4.323155	4.730053	4.494755	4.720073	4.296718	4.379868	4.465266
Mtdh	-0.28244	4.948451	-0.979982	0.343133	0.517798	-6.710151	4.907368	4.298352	5.331054	5.592754	4.695613	5.538825	4.622269	5.077105	4.928103	4.696819	4.781463	4.992709	5.110168	4.757741
Cacybp	-0.349511	6.545607	-0.979921	0.343162	0.517798	-6.766803	6.99984	5.773142	6.918357	7.528865	7.660163	7.305978	6.293729	6.043277	6.304665	5.911228	6.3452	5.927686	6.365923	6.27843
Atp5h	-0.177149	6.091977	-0.979822	0.34321	0.517798	-6.766064	6.666601	6.248209	6.210408	6.619976	6.761691	6.644548	6.1747	5.579795	5.908415	5.799108	5.790457	5.435699	5.665438	5.806491
Armc10	-0.134168	5.632656	-0.979748	0.343245	0.517798	-6.740352	5.513001	5.257997	5.559943	5.802197	5.646007	5.631214	5.670127	5.78302	5.730483	5.682467	5.769531	5.576895	5.629542	5.636551
Coa3	-0.166778	5.456316	-0.979554	0.343338	0.517873	-6.714558	5.812263	5.175881	5.575945	5.567645	5.822031	5.506666	5.268979	5.119041	5.487466	5.380424	5.512044	5.179349	5.52128	5.49539
Gapdh	0.246711	5.43263	0.979226	0.343494	0.518002	-6.702777	5.416013	5.624015	5.213227	4.541025	5.219622	4.820946	5.60571	5.307349	5.823721	5.895735	5.813642	5.568867	5.456834	5.788093
Prkar2a	-0.25172	4.710266	-0.979127	0.343542	0.518002	-6.723673	5.276307	4.771807	5.475847	5.556344	4.904006	5.498157	4.192594	4.616508	4.131534	3.772142	4.477127	4.357637	4.861076	4.116286
Usp22	-0.295592	6.821564	-0.979103	0.343553	0.518002	-6.767691	6.878007	5.908288	7.140709	6.692101	6.334287	6.570052	6.766353	7.233093	6.886797	6.476544	7.265192	7.056164	7.512835	6.795967
Gimap8	0.244972	2.636346	0.978456	0.343862	0.518403	-6.348958	2.742017	3.452688	2.761803	2.708042	2.682409	3.02233	2.967416	2.077116	2.694377	2.668442	2.213317	2.300247	2.203293	2.673087
Lmcd1	0.29236	3.702834	0.978133	0.344017	0.518571	-6.517368	4.040949	4.418186	3.793402	3.043681	3.76592	3.308464	3.749062	3.431732	3.641623	3.973412	3.499841	3.970866	3.562873	3.763295
Rbm34	0.163003	5.689854	0.978042	0.34406	0.518571	-6.752895	5.680283	5.620991	6.146729	5.964894	6.183491	5.990944	5.759921	5.282961	5.506658	5.326816	5.635724	5.325652	5.686859	5.576794
Insl3	1.543213	-3.041963	0.977881	0.344137	0.518622	-5.097778	-3.260591	1.184734	-1.526232	-2.344749	-2.73597	-3.260591	-2.088409	-2.076466	-1.813089	-2.176425	-2.293564	-2.668207	-2.656794	-2.653844
Gpld1	-0.333467	6.058639	-0.977743	0.344203	0.518657	-6.74123	5.72514	6.446598	5.178177	5.689036	5.111109	4.793392	6.894006	6.318704	6.635396	6.698966	6.071536	6.379918	6.17557	6.73003
Ndufb2	-0.195716	5.469333	-0.977573	0.344284	0.518714	-6.744749	5.974632	5.86676	5.371108	5.474653	5.622079	5.257237	5.534216	5.175775	5.591793	5.420347	5.555479	4.938905	5.345791	5.477837
Serinc3	-0.229694	8.617961	-0.977456	0.34434	0.518733	-6.731479	8.537828	8.012634	8.703411	9.096436	8.714563	8.919673	8.303084	8.664124	8.610285	8.536047	8.360743	8.681164	8.630239	8.885268
Slc35c2	0.174553	5.213266	0.977053	0.344533	0.518933	-6.673595	4.878536	5.057828	4.870123	4.427757	4.829341	4.537915	5.384156	5.418994	5.549853	5.824622	5.476975	5.662382	5.467482	5.644439
Pde3a	0.33277	2.288023	0.976969	0.344573	0.518933	-6.129817	1.787089	1.747452	2.626517	1.720809	2.014259	1.857753	2.501617	2.939445	2.430637	2.14695	2.197736	2.447635	3.229186	2.721621
MGC94282	-0.224578	3.318474	-0.976895	0.344609	0.518933	-6.607053	4.012279	3.382986	4.015447	4.452353	4.402865	4.227248	2.629097	3.081965	2.772565	2.758411	2.968181	2.761217	2.363894	2.802815
Pcdhga1	1.076764	0.888994	0.976731	0.344687	0.518933	-5.675203	-0.310971	-0.103798	1.089503	-0.325128	-0.424712	2.116816	0.743216	0.58326	2.462438	0.055728	2.149958	2.252925	0.546019	2.697558
Slc2a8	0.271958	5.860092	0.976652	0.344725	0.518933	-6.718329	5.492389	5.994946	5.53119	4.792905	5.400866	4.719677	6.350332	6.180309	6.357417	6.466092	6.088533	6.24333	6.019101	6.433987
Nebl	-0.195187	4.969941	-0.976576	0.344761	0.518933	-6.684649	4.53321	4.782675	4.718692	5.210194	4.567829	4.670039	5.590126	5.077105	5.202134	5.255774	4.953681	4.911231	4.921699	5.236159
Eif2b4	0.123251	5.547091	0.976547	0.344775	0.518933	-6.749471	5.611862	5.818732	5.858518	5.808165	5.714243	5.944728	5.43302	5.510064	5.389549	5.439902	5.323912	5.153813	5.330318	5.3563
Slc22a14	0.515743	0.159227	0.97641	0.344841	0.518967	-5.614282	0.031429	0.769921	-0.86368	-0.941548	-0.038319	0.231108	0.851758	0.301855	0.571595	0.280604	0.378374	0.218993	0.598632	1.355706
Wdr54	0.209514	6.785989	0.976281	0.344903	0.518995	-6.767108	6.424259	7.008885	6.542147	6.280342	6.524856	6.17213	7.314863	6.806675	7.323329	7.148525	6.721242	6.680401	6.78595	7.28505
Col4a3	-0.476196	3.228911	-0.976009	0.345033	0.519102	-6.365652	3.064101	3.929299	2.356997	2.977436	1.705671	2.336365	4.074361	3.668539	4.060584	3.820684	2.823399	3.732929	3.02328	3.825682
Spsb3	0.271706	6.449016	0.975952	0.34506	0.519102	-6.761265	6.324783	6.945622	6.3938	5.700631	6.219374	5.574336	6.752419	6.520978	6.719227	6.852041	6.533075	6.533025	6.446174	6.789579
Spryd4	0.206713	3.257664	0.975647	0.345206	0.51925	-6.422692	3.239259	3.037697	3.489863	2.916866	3.259533	3.282273	3.038038	3.311493	3.456633	3.158464	3.760656	3.195935	3.379583	3.246018
Gimap6	-0.319092	1.121223	-0.975314	0.345366	0.519416	-5.977128	1.514695	1.833582	0.52603	1.065902	0.808768	0.846031	1.258992	0.972957	1.426399	1.023513	1.260496	1.219993	1.377871	1.29285
Eif1a	0.291231	4.779775	0.975245	0.345399	0.519416	-6.736032	5.059908	4.798824	6.026476	5.715949	5.891515	5.998002	4.223227	4.234368	4.039656	3.734632	4.348982	4.132116	4.543129	4.233886
Tsnax	-0.113456	7.37067	-0.975064	0.345486	0.519456	-6.770032	7.317999	7.276826	7.307386	7.345701	7.259445	7.030159	7.513305	7.405662	7.511316	7.287229	7.509962	7.368415	7.587137	7.478394
LOC688390	0.34687	0.770808	0.97501	0.345512	0.519456	-5.901485	1.033981	1.607774	0.883381	0.577356	1.43972	0.65513	1.335758	0.818632	0.449965	0.591587	0.631286	0.403657	0.791767	0.499386
Phf1	0.278848	6.213457	0.974583	0.345716	0.519699	-6.75281	5.988191	6.761502	5.922492	5.433551	5.872485	5.398047	6.559848	6.436157	6.488686	6.702208	6.333662	6.395437	6.087425	6.631072
Dcun1d1	-0.204318	3.575582	-0.974011	0.345991	0.520047	-6.569077	3.730722	3.354134	3.651925	4.185201	3.827669	4.196023	3.488265	3.242343	3.289407	3.129854	3.434871	3.482452	3.536146	3.642453
Thtpa	-0.147795	7.119934	-0.973817	0.346084	0.520122	-6.773575	7.281923	7.152474	6.850658	6.951338	7.140491	6.732414	7.173657	7.073756	7.09386	7.384287	7.214801	7.279707	7.077077	7.28404
Phtf2	-0.174754	3.989435	-0.973071	0.346442	0.520595	-6.550077	3.608352	3.678943	3.376367	4.04669	3.827669	3.784887	4.42995	4.203515	4.303799	4.086912	4.099353	3.951235	4.11226	4.443728
Alox15	0.338011	0.727712	0.972901	0.346524	0.520652	-5.99938	1.341131	1.184734	1.317458	1.065902	2.322129	1.275818	0.118118	-0.048075	0.628769	0.799637	0.071497	0.081289	0.375586	0.65743
Rb1	0.163396	6.158657	0.972536	0.346699	0.52085	-6.769168	6.149375	6.188172	6.160671	6.378259	6.597556	6.763514	6.162897	6.080544	5.901181	5.938979	6.023735	6.086832	5.948647	5.863025
Tarbp2	0.218585	4.88881	0.972236	0.346844	0.521002	-6.63838	4.62444	4.861671	4.346317	3.963576	4.633136	4.188991	5.458992	5.096827	5.401902	5.430158	5.140489	4.913769	5.098798	5.340848
Zswim4	0.162786	5.085558	0.972031	0.346942	0.521064	-6.686338	4.849116	4.889586	5.030571	4.621853	4.728358	4.928293	5.381895	5.340814	5.436339	5.497024	4.976688	5.181457	5.05004	5.333059
Pofut1	0.222746	2.841318	0.97197	0.346971	0.521064	-6.296922	2.474162	2.884706	2.885483	2.463791	2.79132	2.203108	3.038038	3.14827	2.962454	2.934159	2.940364	3.25317	3.186856	2.83626
Dctd	-0.197992	3.584008	-0.971551	0.347173	0.521302	-6.508479	3.536344	3.224637	3.098159	3.670505	3.680967	3.642872	3.127125	4.193082	3.408468	3.962637	3.958531	4.357637	3.409342	3.041369
Dusp2	0.308727	1.23362	0.97061	0.347626	0.521858	-5.971569	1.371547	0.934883	1.841991	0.823477	1.664612	1.014597	1.047044	1.393205	1.360416	1.451089	1.507975	1.219993	1.150071	1.158276
Slbp	0.253257	3.877723	0.9706	0.34763	0.521858	-6.629824	3.953171	3.874479	4.248334	4.458437	4.801203	4.921945	3.783728	3.440579	3.216682	3.471326	3.690026	3.390696	3.731382	3.417866
Uba2	0.137682	4.026161	0.970373	0.34774	0.521957	-6.558767	3.788223	3.909605	4.051108	3.752019	3.841551	3.817169	4.238302	4.171987	4.146293	4.061889	4.30603	4.074306	4.258405	4.046557
Abcf3	0.126419	6.421244	0.970002	0.347919	0.522161	-6.770664	6.365022	6.39972	6.422675	6.073925	6.395166	6.163187	6.65467	6.453744	6.609	6.467996	6.424202	6.390377	6.502428	
Sema7a	-0.154037	8.118263	-0.968334	0.348723	0.523295	-6.768675	7.814064	7.516205	7.437714	7.717926	7.734583	7.760155	8.75487	8.53867	8.333646	8.590648	8.261428	8.600759	8.251269	8.349708
Bzw1	0.208344	6.267481	0.968254	0.348761	0.523295	-6.777197	6.357444	6.3676	6.929417	6.723385	6.607745	7.090835	5.842809	6.043277	5.857002	5.743541	6.089588	5.962422	6.40891	5.74178
Phf10	0.17169	5.335433	0.968139	0.348817	0.523312	-6.747019	5.218914	5.37392	5.601424	5.752308	5.604547	6.058089	5.236813	5.077105	5.081888	5.185692	5.103341	5.1581	5.087338	5.195929
Inha	0.289167	4.081972	0.968041	0.348864	0.523318	-6.603695	4.158996	5.051103	4.352047	3.829173	4.124949	3.616909	4.192594	3.550909	4.236421	4.173561	3.949256	3.755742	3.908538	4.342619
Strn3	-0.220858	6.087103	-0.967797	0.348982	0.523417	-6.775062	6.029313	5.738676	6.402109	6.891326	6.215826	6.60349	6.095506	5.834271	6.042012	5.634055	5.917167	5.772746	6.061888	6.004591
Uqcc2	0.247866	3.733059	0.967724	0.349018	0.523417	-6.506649	3.891537	4.091039	3.381979	2.994284	3.891338	3.359461	3.951676	3.749088	3.81791	4.081942	3.838074	3.674268	3.89808	3.763295
Rpp30	0.136784	4.905493	0.967619	0.349068	0.523428	-6.726401	4.968344	5.148837	5.323783	5.372033	5.33206	5.431202	4.736391	4.548571	4.65291	4.735311	4.615483	4.475702	4.56643	4.823147
Rnd2	-0.248341	6.848568	-0.967308	0.349218	0.523588	-6.780023	7.507105	7.364429	6.913037	6.699183	6.85817	6.286898	6.697116	6.604045	6.94212	7.017354	6.569852	6.74382	6.725872	6.964876
Abcg3l3	0.559813	0.587043	0.967092	0.349323	0.523634	-5.696136	0.307952	1.506527	0.25957	-0.600663	0.320024	-0.548781	1.21902	1.68885	0.317129	1.023513	0.885831	1.515634	0.649393	0.844371
Rasl11a	-0.509376	2.262105	-0.967064	0.349336	0.523634	-6.231053	2.311111	0.118359	2.869592	2.534609	2.60856	1.927009	2.905804	1.983359	2.707706	2.14695	2.823399	2.100868	2.736815	2.278116
Glb1l	-0.254554	4.526533	-0.966797	0.349465	0.523762	-6.6445	4.922855	4.652466	3.834912	3.972106	4.173318	4.101824	4.771359	4.710941	4.752736	4.946908	4.899331	4.750159	4.178567	4.775092
Usp25	-0.158433	4.900521	-0.966179	0.349764	0.523966	-6.712869	5.076159	4.953495	5.039476	5.062243	4.664713	4.984199	4.739927	4.863466	4.861089	4.595839	4.939699	4.97094	5.204421	4.704402
RGD1305350	0.133022	5.392988	0.966008	0.349847	0.523966	-6.740825	5.163495	5.012387	5.170091	4.785664	5.240728	4.988255	5.679362	5.571764	5.548002	5.647573	5.672824	5.656333	5.607246	5.796614
Dbn1	-0.174938	9.451544	-0.965885	0.349906	0.523966	-6.716384	9.301827	9.291873	9.373868	9.764879	9.29626	9.465329	9.620896	9.494671	9.690756	9.588921	9.18606	9.309777	9.183601	9.755167
Rap2b	-0.298518	2.786669	-0.965863	0.349917	0.523966	-6.359945	2.607924	2.064844	2.578448	3.182467	2.640673	3.129052	2.828218	3.180315	3.188437	2.586593	3.173291	2.921125	2.954296	2.211707
Znhit2	-0.192532	4.131529	-0.965847	0.349925	0.523966	-6.586826	4.167689	3.361401	4.02265	3.875379	4.117361	3.761378	4.330288	4.411301	4.259232	4.398098	4.638748	4.196062	4.246089	4.147707
Fgfr1op2	-0.209628	7.028273	-0.965741	0.349976	0.523966	-6.776967	7.229436	6.779923	7.277965	7.759789	7.407164	7.674013	6.827443	6.943667	6.886065	6.612861	6.891311	6.566474	6.928926	6.62311
Trappc2l	0.125859	4.291122	0.965634	0.350028	0.523966	-6.658785	4.566458	4.522355	4.519244	4.298064	4.584435	4.589557	4.207992	4.17729	4.175368	4.086912	4.235294	4.009344	3.97969	4.204306
Leprotl1	-0.228562	5.17348	-0.965634	0.350028	0.523966	-6.734395	5.062241	4.573314	5.171711	5.683852	5.328778	5.508079	4.802122	5.212396	5.132184	4.973987	5.194477	5.357694	5.341939	5.130032
Acss2	0.184863	6.711174	0.96559	0.350049	0.523966	-6.779509	6.699943	7.151951	6.623162	6.338402	6.78223	6.257791	7.061585	6.439471	6.915083	6.664473	6.542358	6.621072	6.880613	6.99357
Golt1b	-0.237888	4.056548	-0.965566	0.350061	0.523966	-6.608151	3.854329	3.27168	4.162901	4.485502	3.973484	4.313834	4.0114	4.038501	4.160904	3.838474	3.999546	4.179285	4.310591	4.287263
Wasf2	0.192086	3.885242	0.965439	0.350122	0.523966	-6.635507	4.335237	4.138342	4.718692	4.125276	4.151195	4.639415	3.200804	3.997121	3.079866	3.832569	3.549789	4.110154	3.431265	3.195366
Kat7	-0.147704	6.650848	-0.965434	0.350124	0.523966	-6.781015	6.715589	6.225489	6.803345	6.75215	6.685489	6.629045	6.616717	6.731168	6.641486	6.538624	6.831991	6.619518	6.795137	6.541817
Dhx35	0.198266	4.9942	0.965252	0.350213	0.524032	-6.695189	4.922855	5.393549	5.012595	4.575022	4.638447	4.739064	5.44173	5.079939	5.341191	5.013676	4.935008	4.848943	4.947283	5.07972
Sike1	-0.125962	5.523094	-0.964957	0.350355	0.524181	-6.752909	5.502732	5.281173	5.556225	5.739873	5.647325	5.498157	5.522776	5.503746	5.540573	5.502614	5.613877	5.497911	5.429864	5.414663
Mboat4	-0.452031	0.353257	-0.964367	0.350641	0.524543	-5.797983	0.739674	-0.950476	-0.007587	0.43652	0.517419	1.046069	0.118118	0.449407	1.142163	0.699359	0.432628	0.403657	0.546019	0.65743
Abhd4	0.205398	6.974358	0.96375	0.35094	0.524925	-6.782814	7.391733	7.513761	7.145254	6.605657	7.341111	6.814092	6.456206	7.072334	6.441597	7.22716	7.007576	7.358668	6.574848	6.703845
Alkbh1	0.148894	4.331859	0.963547	0.351038	0.525007	-6.64326	4.276172	4.681789	4.416354	4.291262	4.109733	4.53237	4.489964	4.244507	4.277224	4.277207	4.302392	4.292824	4.221137	4.311247
Slc4a5	-2.05201	1.333185	-0.962722	0.351439	0.52554	-6.134542	7.155979	0.533043	0.105278	-1.223791	1.364797	0.571087	0.028455	0.705741	-0.080923	3.918713	5.793051	1.284167	0.375586	-0.621773
Ccm2	0.178528	5.905952	0.962397	0.351596	0.525693	-6.765598	6.166823	6.004262	5.873535	5.344374	6.071135	5.770798	6.066236	5.760233	5.838944	6.081498	5.846486	5.871352	5.962514	6.051623
Abcg2	-0.299079	2.873503	-0.962255	0.351665	0.525693	-6.459308	3.901994	3.747109	3.111733	2.806722	2.60856	2.714852	2.967416	2.14364	3.038267	2.641672	2.066633	2.529342	3.125428	3.050898
Atp5j	-0.140267	6.541777	-0.962241	0.351672	0.525693	-6.783843	6.838404	6.614147	6.516865	6.568872	6.6524	6.469905	6.570807	6.268659	6.711804	6.522206	6.673462	6.381753	6.499029	6.313488
Apopt1	0.129049	4.987851	0.962103	0.351739	0.525728	-6.721433	5.348806	5.303982	5.348356	4.996864	5.325489	5.216324	4.864988	4.620407	4.771898	4.876899	4.972116	4.701051	4.78366	4.748987
Impdh1	-0.12599	6.128733	-0.961991	0.351793	0.525743	-6.777215	6.339284	6.270577	6.063616	6.122628	6.234358	5.85206	6.226019	6.072029	6.197728	6.125636	6.062962	6.050392	6.098885	6.108621
Polrmt	0.318848	5.797117	0.961782	0.351895	0.52583	-6.732738	5.29428	6.334748	5.449365	4.965167	5.383574	4.636834	6.415889	6.006512	6.263514	6.305884	5.939588	5.948875	5.8105	6.435806
Nsun6	0.222288	2.761138	0.961171	0.352192	0.526207	-6.361155	2.694686	3.263946	2.691232	2.728325	2.672087	3.143674	2.828218	2.400986	3.006262	2.877922	2.696109	2.761217	2.379013	2.745289
Nono	0.169285	7.375443	0.961028	0.352261	0.526246	-6.776524	7.443207	7.362167	7.684174	7.788749	8.033119	8.061215	6.972735	7.277592	7.129581	7.100799	7.204118	7.138212	7.044852	7.025333
Vtn	-0.352299	5.210328	-0.960563	0.352487	0.526518	-6.724693	5.897038	5.341214	4.334789	4.543889	4.968684	4.81639	5.621128	5.304929	5.495173	5.818659	4.776227	5.366984	4.939655	5.765122
Zfyve21	-0.23712	3.733987	-0.959957	0.352782	0.526893	-6.587113	4.464322	4.000847	3.540923	3.538889	4.109733	3.37813	3.908348	3.475434	3.495578	3.740952	3.574129	3.662244	3.766298	3.739923
Lbh	-0.143926	4.688087	-0.959641	0.352936	0.527057	-6.695158	4.889087	4.619507	4.712023	4.703523	4.690509	4.578838	4.595201	4.36558	4.621564	4.690302	4.655954	4.926391	4.839377	4.806368
Neu4	-0.404393	3.656075	-0.959418	0.353045	0.527154	-6.468858	3.753999	4.263866	2.861581	3.075697	3.106978	1.892796	3.889373	4.003106	4.13647	4.303064	4.012962	4.327499	3.713602	3.988241
Cdk8	-0.160616	5.613027	-0.959069	0.353214	0.52733	-6.773068	5.7514	5.673024	5.607425	6.182661	5.77699	6.174802	5.660833	5.3896	5.397796	5.388498	5.462379	5.202367	5.465713	5.481365
Zic3	0.419981	0.60519	0.958995	0.35325	0.52733	-5.855443	1.108109	1.655851	0.642822	-0.02435	0.770579	0.65513	0.68572	0.137473	0.449965	0.981432	-0.072212	0.804622	0.649393	1.085968
Ints7	0.177105	4.606954	0.958253	0.353612	0.527804	-6.669781	4.3544	4.955898	4.852041	4.543889	4.553844	4.160517	4.622269	4.673906	4.64946	4.574767	4.64163	4.715669	4.608729	4.655275
Polg	0.123688	5.23599	0.957737	0.353863	0.528114	-6.721142	5.031616	5.053348	5.043022	4.932758	5.21785	5.104925	5.478169	5.418994	5.356081	5.114467	5.309487	5.48943	5.428048	5.367782
Inhbb	-0.502071	3.606298	-0.957476	0.353991	0.528239	-6.4212	3.608352	0.895377	2.916749	2.87199	3.891338	3.069038	3.920861	4.145178	4.170562	4.228565	3.872828	4.40891	4.233667	4.429189
Gmeb2	0.188745	3.329278	0.956792	0.354324	0.528671	-6.491435	3.118917	3.735969	3.728808	3.527359	3.091571	3.524966	3.356132	3.340143	3.271566	3.060806	3.295436	3.220743	3.278395	3.262515
Tusc2	-0.155776	6.600456	-0.956352	0.354539	0.528891	-6.790751	7.079157	6.579511	6.775367	7.00116	7.158163	7.024738	6.356102	6.131972	6.384644	6.334374	6.537724	6.220961	6.55751	6.281469
Smarcb1	0.111824	6.504988	0.956303	0.354563	0.528891	-6.788424	6.531696	6.540827	6.686324	6.530402	6.763515	6.580165	6.210814	6.60306	6.482883	6.490677	6.535402	6.461107	6.384771	6.28551
Tpi1	-0.184582	5.078841	-0.956147	0.354639	0.528891	-6.706557	5.002757	4.438943	4.71425	4.788082	5.040651	4.634248	5.426454	5.132753	5.436339	5.159982	5.555479	5.119046	5.618437	5.084367
Calcrl	0.318687	2.624625	0.956129	0.354648	0.528891	-6.38741	3.016775	2.758277	3.295418	2.568755	3.259533	3.038068	2.165439	2.326577	2.330799	1.388059	2.428842	3.118845	3.02328	2.294252
Chchd4	-0.191448	2.247163	-0.955541	0.354935	0.52921	-6.340062	2.718545	2.493084	2.735741	2.728325	2.484288	2.410784	1.836107	2.030999	2.278164	1.852296	2.259075	1.667719	2.348615	1.951771
Jade1	-0.217481	4.770412	-0.955459	0.354975	0.52921	-6.727008	4.70428	4.582672	5.261214	5.567645	4.794082	5.047769	4.778253	4.49437	4.639062	4.21501	4.722797	4.610089	4.842108	4.584872
Atp6v1g2	-0.189765	9.047841	-0.955341	0.355033	0.52921	-6.725201	9.602877	9.128441	9.445753	9.541211	9.741476	9.218362	8.648855	8.724004	8.925957	8.612569	9.067568	8.443232	8.96244	8.61013
RGD1305464	0.180173	4.565145	0.955331	0.355038	0.52921	-6.684017	4.414065	4.407694	4.899765	4.688073	4.951718	4.662444	4.171806	4.635896	4.445789	4.31582	4.717344	4.838295	4.579577	4.250521
Ly6g6e	-0.35354	1.060291	-0.95509	0.355156	0.529275	-5.915164	1.033981	0.854759	0.975347	0.966949	0.985877	-0.1418	1.544223	0.58326	1.891068	1.064403	1.290185	0.969826	1.521045	2.105984

Bach1	0.278083	1.901828	0.955061	0.35517	0.529275	-6.241835	2.263247	2.532247	2.820842	2.029493	2.124346	2.160607	1.21902	2.268138	1.60797	1.355481	1.605665	1.783252	2.097585	1.193114
Ctps2	-0.176086	5.416129	-0.954781	0.355307	0.529414	-6.772717	5.528269	5.414661	5.719159	6.170667	5.596383	5.969561	4.983054	5.181064	5.173599	5.238011	5.150641	5.288978	5.402381	5.046765
Rhox5	0.901492	-0.118124	0.954677	0.355358	0.529424	-5.684098	-0.310971	-1.10215	2.379614	-0.325128	-0.516166	1.165512	0.68572	0.76329	-0.504027	0.591587	0.202178	-0.657145	-1.413173	-0.621773
Sult5a1	0.313725	2.068248	0.954561	0.355414	0.529442	-6.13852	1.619793	2.151237	2.162035	1.780306	2.424057	1.327961	1.668148	2.619301	1.914249	2.614395	2.197736	2.252925	2.133689	2.474989
Narg2	0.194149	2.840834	0.95367	0.35585	0.52991	-6.368764	2.607924	3.046741	2.744481	2.835056	3.083806	2.82781	2.930765	2.964197	3.09008	2.668442	2.883067	2.931181	2.466543	2.91141
Wnt3a	-1.154026	-0.206833	-0.953665	0.355853	0.52991	-5.352773	0.830061	-3.260591	-1.705055	-2.731185	-2.73597	-0.006501	0.903114	1.856886	1.180909	1.625169	1.762161	1.738143	1.377871	1.501815
Fgfr1	-0.175483	7.931634	-0.953649	0.35586	0.52991	-6.783161	7.942132	7.118565	7.700267	7.567375	7.71272	7.794148	8.109653	8.281729	8.096162	8.087289	7.927747	8.326656	8.201375	8.183695
Cabin1	-0.112468	7.133727	-0.953388	0.355988	0.530035	-6.792617	7.096902	6.977896	7.181103	7.283235	7.191968	6.959606	7.055923	7.198957	7.206125	7.201613	7.107368	7.114139	7.110863	7.197679
Polr3e	0.15495	5.48551	0.953079	0.356139	0.530194	-6.744325	5.392103	5.509296	5.550007	5.052221	5.403987	5.0341	5.765135	5.652036	5.665368	5.625541	5.599127	5.49113	5.417104	5.67557
Stx16	-0.134095	6.609015	-0.95292	0.356217	0.530244	-6.792233	6.512205	6.553573	6.280438	6.782172	6.611801	6.609428	6.865161	6.491483	6.823157	6.633388	6.642865	6.425981	6.555016	6.755748
Rnf145	-0.149454	6.756417	-0.952569	0.356389	0.5304	-6.789993	6.322839	5.963056	6.333534	6.589808	6.402209	6.528875	6.909001	7.115098	7.059183	7.085361	6.931901	7.074427	7.034744	7.254965
Obfc1	0.134785	4.58633	0.952527	0.35641	0.5304	-6.65549	4.3275	4.570182	4.272706	4.274118	4.436792	4.469923	4.868222	4.673906	4.910919	4.865376	4.618412	4.519683	4.680656	4.786544
Atg4d	0.238552	5.687119	0.952435	0.356455	0.530401	-6.75247	5.605478	6.111589	5.491507	5.015553	5.585425	5.002363	6.043529	5.644398	6.001999	6.039816	5.780032	5.686328	5.637422	6.005816
Uba5	0.121564	5.993139	0.951863	0.356735	0.530694	-6.782946	5.834269	5.952268	6.005725	6.138819	6.223797	6.253584	6.020459	5.87889	6.01546	5.922393	5.958202	5.846329	5.967523	5.910902
Ubn2	-0.363126	4.864562	-0.951733	0.356799	0.530694	-6.713405	4.492273	5.042086	5.079744	5.474653	3.596165	4.804937	5.151787	5.359594	5.206835	4.513333	4.847853	4.603816	5.33614	4.652149
Jam3	-0.221301	5.482469	-0.951667	0.356832	0.530694	-6.751374	5.67572	5.620991	4.762375	5.168122	5.465045	4.963744	5.446065	5.597703	5.43233	5.757634	5.472126	5.964872	5.756531	5.707549
RGD1560556	0.53264	-0.445956	0.951433	0.356946	0.530694	-5.748676	-0.634519	1.632013	0.604933	0.664146	0.422095	-0.371994	-0.672244	-0.992527	-0.080923	-0.806258	-1.500575	-1.660823	-0.628995	0.190106
Foxd1	0.826457	-0.025166	0.951395	0.356965	0.530694	-5.664212	0.692264	-0.366503	1.195284	-1.388744	-1.087961	1.473956	1.000645	0.301855	0.628769	0.348395	-0.855515	0.007173	-0.628995	0.190106
Eif4e2	0.235551	4.823591	0.951164	0.357078	0.530694	-6.75705	5.03637	5.106215	5.62528	5.691621	5.485819	6.188088	4.145391	4.548571	4.203867	4.303064	4.609607	4.338875	4.416965	3.890799
Sfrp1	-0.520924	3.389396	-0.951125	0.357097	0.530694	-6.609444	5.457367	3.34683	3.986269	2.977436	3.028244	4.220367	2.566765	3.000548	2.882434	2.945148	4.324082	2.555581	3.089114	3.237699
Ei24	-0.14665	6.90782	-0.951042	0.357138	0.530694	-6.792993	7.199547	6.903927	7.209392	7.509951	7.305245	7.413579	6.393618	6.612877	6.639749	6.632539	6.695818	6.779044	6.695245	6.732253
RGD1311783	-0.124501	5.696571	-0.950941	0.357188	0.530694	-6.77815	5.813648	5.831871	5.897242	5.94228	5.655207	5.63251	5.597939	5.559634	5.570064	5.625541	5.665763	5.53465	5.895753	5.56021
Prpf4	0.142501	4.856767	0.950915	0.3572	0.530694	-6.705505	4.658759	4.681789	5.17333	4.800109	4.735811	4.887611	5.075493	4.840254	4.925253	4.973987	4.784073	4.7671	4.911337	4.834225
Timm8b	-0.155513	5.924127	-0.950857	0.357229	0.530694	-6.787823	6.255119	6.008897	5.987435	6.223424	6.20781	6.131446	5.844452	5.35258	6.113858	5.64589	6.004833	5.468867	5.968773	5.750578
Med20	-0.165402	4.250145	-0.950823	0.357245	0.530694	-6.658259	4.467845	4.305905	4.11989	4.33821	4.358498	4.17126	3.999651	4.346879	4.091419	3.957219	4.591836	4.245254	4.456698	4.134325
Prkag1	0.215435	6.993689	0.950787	0.357263	0.530694	-6.795504	6.817152	7.455106	6.834377	6.747188	7.01646	6.682594	7.428763	6.873533	7.265353	7.192995	6.751572	6.760835	6.699768	7.398549
Adam4l1	-0.366144	1.646397	-0.950773	0.35727	0.530694	-6.039383	1.108109	1.894965	0.818644	1.856	1.258434	0.734546	2.450751	2.077116	2.166756	2.088679	1.629088	1.826994	1.465469	2.211707
Smndc1	0.204276	3.623255	0.950604	0.357353	0.530752	-6.660128	4.017097	4.188236	4.48836	4.588954	4.402865	4.965803	2.955303	3.431732	3.149895	3.030171	2.986432	2.772447	2.838755	3.050898
Serbp1	0.223269	7.622875	0.950442	0.357433	0.530804	-6.780846	7.533407	7.548002	8.05206	8.174191	8.191679	8.515231	7.104724	7.586429	7.303164	7.314498	7.504827	7.45547	7.475517	6.969279
Tada2b	-0.133947	6.217216	-0.950275	0.357515	0.530861	-6.792322	6.346001	6.121254	6.483321	6.575884	6.238736	6.464819	6.199304	6.024276	5.95107	6.037251	6.186512	6.053848	6.298941	6.080985
Top1mt	-0.1384	4.5788	-0.950094	0.357603	0.530875	-6.693303	4.716181	4.332034	4.586407	4.659309	4.808289	4.461194	4.764433	4.50706	4.618038	4.520698	4.567795	4.519683	4.689778	4.418187
Acox1	0.174253	6.095036	0.950075	0.357613	0.530875	-6.785098	6.261224	6.385533	6.459633	5.723547	5.936405	5.877432	6.107871	6.070605	6.13251	5.899977	6.086419	6.206544	6.122658	6.083308
Mir3577	0.403648	0.644384	0.949572	0.35786	0.531131	-5.738708	-0.046857	0.583632	-0.130036	0.041976	0.470544	0.65513	1.478026	1.239467	0.683763	0.847278	0.631286	1.315214	0.836265	1.473753
Homer3	-0.200093	6.125074	-0.949543	0.357874	0.531131	-6.782033	5.907455	5.161381	6.054859	6.046925	5.945004	5.938452	6.19802	6.371704	6.328589	6.205494	6.418445	6.464573	6.588732	6.144665
Arpc5	-0.301185	7.386817	-0.949099	0.358093	0.53139	-6.785422	7.686043	6.647227	7.808153	8.09537	7.837746	8.07081	7.246919	7.320759	7.084311	6.818819	7.449574	7.052714	7.451135	6.855628
March7	-0.172992	5.580767	-0.948941	0.35817	0.531397	-6.776289	5.569851	5.45088	5.847405	6.030684	5.335334	5.878525	5.588166	5.488896	5.62905	5.164691	5.599127	5.348344	5.718405	5.514455
Slc25a17	-0.131963	6.181025	-0.94891	0.358185	0.531397	-6.791572	6.342166	6.214496	6.418585	6.32348	6.273288	5.896982	6.145676	5.979449	6.120102	6.187098	6.215814	6.054998	6.138292	6.245615
N4bp2l2	0.132861	4.729349	0.948336	0.358468	0.53175	-6.722946	4.843702	4.779965	5.168468	4.947973	5.107284	5.05165	4.555628	4.768288	4.421726	4.475935	4.689763	4.482556	4.563124	4.414502
Phf7	0.171323	4.012542	0.947412	0.358923	0.532359	-6.566817	3.782575	4.060109	3.609501	3.578533	3.922149	3.821723	4.330288	4.303891	4.355524	4.298786	4.026255	3.941318	3.989574	4.25465
Akap3	0.477121	0.29687	0.947022	0.359114	0.532578	-5.76921	0.485316	-0.103798	0.975347	-0.243853	0.422095	0.809818	0.202532	1.279465	0.884903	-0.116374	0.631286	-0.24119	0.598632	-0.116166
Map7d1	-0.172104	8.936708	-0.946914	0.359167	0.532591	-6.742497	9.05611	8.773969	9.217778	9.578262	9.118111	9.468599	8.564395	8.906092	8.760741	8.768405	8.848411	8.785137	8.724435	8.546721
Spata32	-0.443954	0.360563	-0.94639	0.359426	0.532908	-5.873822	0.307952	1.047298	1.061799	1.327578	0.091273	-0.457681	0.563361	-0.150641	0.571595	0.534523	0.846404	0.081289	0.434663	0.045082
Cryga	0.302432	2.047751	0.946262	0.359489	0.532936	-6.13657	1.430522	2.184396	2.10888	1.892408	2.062471	1.541762	2.534559	2.419003	2.446625	1.898256	2.468337	2.118577	1.626159	2.325991
Kpna3	-0.270579	5.74917	-0.945425	0.359902	0.533482	-6.789988	5.792727	5.273489	6.172863	6.50123	5.891515	6.165876	5.381895	5.659633	5.699127	5.054841	5.884694	5.508024	6.07239	5.460068
Usp6nl	-0.153252	5.754521	-0.945174	0.360025	0.533555	-6.781322	5.819176	6.007739	5.777806	5.836471	5.359656	5.559419	5.870489	5.740666	5.72885	5.765405	5.624113	5.846329	5.665438	5.991043
Wls	-0.223284	6.190458	-0.945145	0.36004	0.533555	-6.798437	6.904902	6.005422	6.278935	6.260457	6.305362	6.842937	5.826276	5.753148	6.055107	5.908424	6.313925	6.015372	5.87324	6.344811
Ccne1	-0.239156	3.787798	-0.944997	0.360113	0.533592	-6.555658	3.380557	2.894758	3.436929	4.086518	3.886882	3.784887	4.028845	4.112335	4.007682	3.92982	3.84309	3.78376	3.822677	4.147707
Spag8	-0.415323	3.410651	-0.944915	0.360154	0.533592	-6.430502	4.193457	3.037697	2.225837	2.184345	3.188895	2.526873	4.0114	3.676051	3.648551	3.64312	4.33124	3.703897	3.562873	3.983274
Traf1	0.45287	-0.014451	0.944814	0.360203	0.5336	-5.674312	0.105685	0.118359	-0.33572	-0.705589	0.091273	0.527154	-0.529223	0.04768	-0.632503	0.699359	0.924208	1.152831	-0.405292	0.383504
Sfxn1	-0.204909	6.088739	-0.944495	0.360361	0.533741	-6.796953	6.253078	5.699071	6.462284	6.608395	6.429273	6.311387	5.910253	5.939438	5.876312	5.635752	6.131194	5.893379	6.323571	5.79236
G6pc3	0.282656	6.419113	0.944442	0.360387	0.533741	-6.78842	6.291366	6.615667	6.013881	5.508766	6.614499	5.478106	6.715188	6.466794	6.819325	6.83958	6.500902	6.530547	6.407989	7.084519
Deaf1	-0.118459	5.961846	-0.944218	0.360498	0.53382	-6.785701	5.870661	5.965443	5.733497	5.947696	5.770958	5.698367	6.063417	6.070605	6.13251	6.181301	6.059733	6.053848	5.824373	6.11885
Reck	0.394297	5.67655	0.944113	0.360549	0.53382	-6.766367	5.950908	6.880091	5.900178	4.797712	5.198203	5.074724	6.20187	5.471736	5.980196	5.757634	5.454206	5.479185	5.417104	5.940678
Zfp281	-0.279079	4.702101	-0.944063	0.360574	0.53382	-6.737426	4.840987	4.525594	5.453368	5.46713	4.384017	5.132675	4.14005	4.768288	4.303799	4.210463	4.733642	4.581643	4.999576	4.350357
Adck3	-0.16433	5.091469	-0.943315	0.360944	0.534302	-6.708698	4.965854	4.874426	4.79429	4.600004	4.581681	4.216915	5.509574	5.438999	5.436339	5.474446	5.300397	5.263337	5.394962	5.477837
Ankrd44	0.176805	4.598538	0.942838	0.361179	0.534498	-6.688714	4.358202	4.856537	4.516696	4.613698	4.866025	4.437656	4.579502	4.604749	4.481148	4.556968	4.345451	4.985489	4.484924	4.757741
Cdhr5	0.513295	0.270485	0.942814	0.361191	0.534498	-5.708574	-0.899176	1.082904	0.105278	0.531921	0.470544	-0.291112	1.17791	0.377517	0.091681	0.534523	0.631286	-0.24119	0.745852	0.844371
Rab3gap2	-0.136572	6.615047	-0.94271	0.361243	0.534498	-6.800036	6.345043	6.662752	6.348659	6.653509	6.137343	6.306523	7.012739	6.698349	6.956847	6.639321	6.577397	6.557576	6.815423	6.91553
Timm8a1	0.163214	4.523873	0.942687	0.361255	0.534498	-6.721262	4.859884	5.028455	4.787963	4.753862	5.07631	4.988255	4.421169	3.653399	4.189688	4.345154	4.417843	4.308338	4.270617	4.303297
Fcrl2	0.50103	5.768204	0.942512	0.361341	0.534561	-6.729318	5.204201	5.337533	3.89502	4.4153	6.119413	5.883978	4.718584	8.093323	4.433808	7.87923	7.244488	8.87855	4.134703	4.561731
Nudt13	0.188637	2.993084	0.942371	0.361411	0.534598	-6.374276	2.896425	2.991607	2.61703	2.511387	2.944963	2.998397	3.374605	3.126505	2.894141	3.276274	3.280721	3.127617	3.070609	2.992761
Gramd3	0.215351	5.699341	0.94214	0.361525	0.534701	-6.781751	6.058818	6.330126	5.976348	5.285703	5.673431	5.55669	5.713926	5.557602	5.237026	5.719741	5.809803	5.862183	5.549951	5.490147
Pax6	0.156456	3.590644	0.941872	0.361658	0.534832	-6.581172	3.92781	3.707738	3.8018	3.550328	3.998482	4.032808	3.30948	3.542716	3.188437	3.615871	3.359854	3.816678	3.294432	3.25429
Abcg3	0.660398	-0.37914	0.941574	0.361805	0.534984	-5.597446	-0.634519	1.425638	-0.974047	-0.81875	-0.257548	-0.457681	0.357848	0.222004	-0.27702	0.055728	-0.855515	0.218993	-1.047095	-0.116166
Banf1	0.185339	5.977613	0.941326	0.361928	0.535099	-6.801529	6.366911	6.231451	6.340035	6.480453	7.01595	6.847968	5.469678	5.561662	5.661948	5.474446	5.820016	5.509703	5.587858	5.344726
Pycrl	0.180249	4.258495	0.940226	0.362473	0.535829	-6.649564	4.307974	4.452618	4.323168	4.00988	4.703236	3.866483	4.207992	4.17729	4.231815	4.277207	4.327666	4.212646	4.357143	4.24638
Ddx1	-0.198004	7.212475	-0.94015	0.362511	0.535829	-6.802316	7.080884	6.850699	7.139467	7.786333	7.283375	7.665489	7.242565	7.189148	7.206712	7.029021	7.219149	6.92896	7.289508	7.074043
Chd2	-0.202769	6.323963	-0.939984	0.362593	0.535885	-6.803639	6.247964	6.721821	6.514951	6.949719	5.925585	6.427964	6.436733	6.384369	6.279223	6.004794	6.11262	6.083455	6.343939	6.122243
Riok1	0.138196	4.875172	0.939377	0.362894	0.536264	-6.724023	4.742602	4.996138	5.15214	4.87707	4.635794	5.008367	5.018416	4.792678	4.992142	4.859579	4.827749	4.781067	4.760756	4.861554
Rundc3a	-0.132023	8.839159	-0.93874	0.36321	0.536646	-6.762437	9.163767	8.959896	8.818073	8.762211	8.896101	8.653991	8.966753	8.680321	8.880703	8.90658	8.741317	8.677435	8.789354	8.855163
Poc1a	0.272846	1.773432	0.938676	0.363242	0.536646	-6.098622	1.717752	1.914859	2.039558	1.273392	1.822268	1.221719	2.165439	2.007376	1.981639	1.986005	1.94125	1.910689	1.837926	1.473753
Ppp1r3d	0.201422	3.395747	0.938254	0.363451	0.536889	-6.556417	3.701089	3.986819	4.102999	3.437817	3.61245	3.353184	3.211029	3.012464	2.917273	3.040455	3.280721	3.284884	3.423994	3.326676
Rgs4	-0.188895	6.372616	-0.937917	0.363619	0.537071	-6.80796	6.839085	6.834471	6.810646	7.396493	6.954999	7.114223	6.026261	5.35726	6.057711	6.003481	6.208058	5.799066	5.961258	5.873803
Seli	-0.165675	5.30458	-0.937005	0.364073	0.537675	-6.750026	4.985653	5.324578	5.224203	5.394419	4.625132	4.942996	5.662696	5.347885	5.717364	5.157622	5.265325	5.271276	5.649941	5.735885
Prrc2a	-0.125164	9.655357	-0.936578	0.364285	0.537922	-6.732336	9.984583	9.695454	9.788761	9.679721	9.599603	9.750782	9.59551	9.462999	9.490596	9.621182	9.47784	9.735256	9.644002	9.650652
Ak8	-0.397813	0.462204	-0.936099	0.364523	0.538208	-5.825074	1.278303	-0.186163	0.679743	-0.093872	0.027932	0.117189	0.625837	0.58326	1.01926	0.348395	0.805871	0.567345	1.316372	0.554014
Bmp7	-0.712452	2.329756	-0.935588	0.364778	0.538456	-6.450423	4.832812	1.812527	2.322392	1.92792	2.424057	3.803422	1.258992	0.76329	2.204856	2.628098	3.949256	1.375373	1.28461	2.417223
Bmx	0.416886	-0.382102	0.935581	0.364781	0.538456	-5.776781	0.243629	1.082904	0.914692	-0.02435	-0.038319	0.056669	-0.067151	-0.654381	-0.929891	-1.136114	-0.616402	-1.243945	-0.893866	0.119416
Fnip2	0.230795	3.917238	0.935289	0.364926	0.538605	-6.583599	3.799454	4.091039	4.360599	3.595195	3.534831	3.693437	3.876582	4.171987	3.689436	3.784431	4.099353	4.451452	4.156802	3.642453
Zc4h2	-0.159142	4.464844	-0.934881	0.36513	0.538839	-6.728797	4.765689	4.544872	4.905621	5.050208	4.822358	4.724549	4.213088	4.020912	4.31255	4.015725	4.609607	4.032882	4.383621	4.178459
Nck1	0.210348	4.120823	0.934126	0.365506	0.539328	-6.67998	4.239536	3.894655	4.57175	4.43703	4.93239	4.777073	3.857182	3.871902	3.996864	3.649853	3.911548	4.023513	3.805994	3.814538
Ube2f	0.132249	4.750367	0.934025	0.365557	0.539336	-6.736837	4.85451	4.80949	4.96665	4.945809	5.333698	5.018319	4.739927	4.519639	4.575048	4.531675	4.621334	4.509652	4.569728	4.568381
Fan1	0.188176	3.29137	0.933911	0.365613	0.539354	-6.449369	2.694686	3.459476	3.330668	3.329127	2.95351	3.121686	3.616879	3.358932	3.448717	3.417108	3.373787	3.292705	3.502026	3.34228
Zfp161	-0.156554	3.469131	-0.933372	0.365882	0.539684	-6.554543	3.576066	3.411272	3.780713	3.711838	3.196117	3.552622	3.318966	3.614836	3.432752	3.478908	3.543639	3.496064	3.178239	3.417866
Manea	-0.255943	2.935008	-0.933213	0.365962	0.539728	-6.469152	3.035892	2.411416	3.301354	3.468286	2.800826	3.450478	2.746221	2.80888	2.870631	2.359011	2.930971	2.816513	3.387081	2.91141
Snx1	0.148983	4.20737	0.933133	0.366001	0.539728	-6.706831	4.47136	4.538474	4.703085	4.680285	4.625132	5.0341	4.0114	4.003106	3.860303	3.7783	3.66758	3.961084	3.964736	3.692011
Etnk2	0.429124	1.970166	0.932957	0.366089	0.539761	-6.086063	1.936985	2.279532	0.945338	0.823477	2.448452	1.519512	2.266943	2.307359	1.426399	2.600561	2.481265	2.827322	1.881043	2.278116
Bai1	-0.186518	9.937059	-0.932895	0.36612	0.539761	-6.739329	9.714428	9.921538	9.321077	9.806315	9.71462	9.327947	10.37449	10.05551	10.36032	10.29077	9.839411	10.02145	9.898718	10.4739
Mob4	-0.276225	5.343995	-0.932751	0.366192	0.539761	-6.78994	5.444906	4.675972	5.702436	6.161373	5.80678	5.91627	5.458992	4.899205	5.109768	4.772804	5.359357	4.921355	5.483308	5.143455
Cul5	-0.197522	5.251247	-0.932728	0.366204	0.539761	-6.764464	4.997891	5.129812	5.38375	5.690329	4.762811	5.326158	5.261619	5.343175	5.302182	4.989994	5.357605	5.127817	5.637422	5.248633
Arl8a	-0.133629	8.465205	-0.932476	0.366329	0.53988	-6.770158	8.81321	8.459764	8.918389	9.007947	8.807081	8.925634	8.0209	8.314885	8.192342	8.242334	8.271928	8.24007	8.237337	8.065574
Maf1	0.158001	6.641193	0.932248	0.366443	0.539981	-6.808197	6.518165	6.766985	6.484626	6.086747	6.464835	6.123169	6.875644	6.677919	6.913646	6.916273	6.769468	6.761539	6.685397	6.948402
Acap2	-0.197907	5.989987	-0.931253	0.366941	0.540648	-6.804452	5.947126	5.747023	6.312382	6.383864	5.570221	6.240891	6.267025	6.090416	5.973314	5.599695	5.764251	5.936449	6.110254	5.941959
Col4a4	0.370911	0.435969	0.931149	0.366993	0.540658	-5.799463	0.428583	1.010791	0.442561	-0.02435	0.422095	0.336685	0.429655	-0.048075	0.787836	0.799637	0.535373	1.11804	0.182287	0.844371
Fhl4	0.407086	0.335382	0.930991	0.367072	0.540702	-5.803506	0.031429	-0.103798	0.398943	0.386344	0.917619	1.165512	0.952703	0.449407	0.385075	0.646479	0.535373	0.403657	0.182287	-0.397871
Acaa1a	0.526804	0.852131	0.930909	0.367112	0.540702	-5.757862	0.485316	1.184734	0.642822	-0.502853	0.731352	-0.646023	1.938115	1.464337	1.142163	1.451089	1.456531	0.930264	1.001683	1.659607
Cdc25b	0.268176	5.490693	0.930763	0.367186	0.540744	-6.762782	5.235546	6.250168	5.166843	5.066232	5.375645	4.902733	5.945892	5.371209	5.874836	5.682467	5.300397	5.423285	5.357288	5.953435
Lpin3	0.442697	4.849781	0.930614	0.36726	0.540787	-6.725309	4.891713	6.506823	5.166843	4.424653	4.633136	4.113012	5.239313	4.055878	5.393679	4.706539	4.359523	4.237171	4.652936	5.575144
Hint3	-0.173508	3.267803	-0.930451	0.367342	0.540808	-6.475473	3.303772	2.963229	2.837276	3.175097	3.217567	3.248852	3.521509	3.252427	3.280514	3.360772	3.644778	3.245131	3.576053	3.286911
Art3	-0.328168	2.307997	-0.930318	0.367408	0.540808	-6.295622	2.694686	3.167741	2.175023	2.227735	1.840817	1.426914	2.598267	2.227821	2.625819	2.256873	2.166061	2.529342	2.133689	2.570827
Med24	0.199864	7.442816	0.930316	0.367409	0.540808	-6.81379	7.153793	7.789377	7.405702	7.045786	7.080296	6.920871	7.861273	7.538817	7.80632	7.632205	7.360108	7.37211	7.331401	7.91066
Ccdc39	-0.195934	5.695745	-0.929901	0.367617	0.541048	-6.775505	5.304169	5.544839	4.983359	5.628268	5.403987	5.174216	6.461569	5.788228	6.045953	6.101282	5.721307	5.680379	5.710957	6.223667
Trim54	-0.335871	0.929504	-0.929637	0.367749	0.541176	-6.204338	2.295333	2.184396	2.487668	1.874319	1.05105	1.249022	0.357848	0.04768	0.317129	1.104165	0.138317	0.28321	0.111637	0.442608
Rab39a	-0.26988	2.559144	-0.929476	0.36783	0.541228	-6.374578	2.387517	2.309899	2.804219	3.197095	2.322129	2.705027	2.534559	2.58779	2.313467	2.007132	2.66245	2.680061	3.169571	2.417223
Myo1b	-0.190794	3.464953	-0.92932	0.367908	0.541277	-6.556706	3.676936	3.287027	3.863274	3.622545	3.122223	3.524966	3.639767	3.377479	3.59934	3.28495	3.074378	3.397965	3.855477	3.326676
Galnt11	-0.107923	6.358589	-0.929161	0.367988	0.541328	-6.806532	6.209544	6.208464	5.992029	6.073925	5.946075	5.989933	6.645274	6.485083	6.559317	6.629136	6.500109	6.625724	6.544158	6.631072
Slc39a11	0.23601	2.906765	0.928514	0.368312	0.541565	-6.359637	2.787845	2.724859	3.215954	2.350586	2.72297	2.469996	2.659281	3.252427	2.734001	3.223093	3.013384	3.489274	3.061266	3.203933
Prss53	0.343399	4.688609	0.928513	0.368312	0.541565	-6.632898	4.031456	5.131939	3.670387	3.815017	4.140005	4.17126	5.511644	4.879821	5.25075	5.506329	4.672958	4.901034	4.592606	5.432996
Urb1	0.30085	5.525338	0.928486	0.368326	0.541565	-6.743925	4.904771	5.902072	5.614594	4.847241	4.641095	4.557157	6.253486	5.903095	6.116358	5.884361	5.606521	5.660872	5.569853	5.930391
Prep	-0.227264	5.545731	-0.92848	0.368329	0.541565	-6.791309	5.516408	4.835816	5.752034	5.947696	5.421037	6.001017	5.486611	5.585789	5.519942	5.294957	5.619735	5.460213	5.77665	5.456487
Fbxw17	0.176451	3.199886	0.927988	0.368576	0.54183	-6.471043	3.05476	3.411272	3.370733	3.137672	3.422332	2.898483	3.105363	3.201289	3.120298	3.538169	3.265855	3.368666	2.871195	3.203933
Doc2a	0.327286	3.940243	0.927941	0.368599	0.54183	-6.629383	4.22295	5.317123	4.102999	3.584109	3.680967	3.830786	3.933266	3.484018	4.13647	4.005262	3.512491	3.644018	3.91892	3.896095
RGD1311847	0.193748	3.477298	0.926944	0.369099	0.542498	-6.490848	3.453461	3.696289	3.336461	2.916866	3.333437	3.121686	3.876582	3.622632	3.432752	3.838474	3.678847	3.625558	3.445698	3.447025
Nrtn	0.589046	0.171939	0.926683	0.369231	0.542625	-5.68808	-0.217457	1.339943	0.20995	-0.502853	0.470544	-1.117764	0.202532	-0.150641	0.884903	0.893396	0.263333	0.714338	0.313987	0.606649
Wdsub1	0.232228	3.918861	0.926515	0.369315	0.542677	-6.592933	3.854329	4.637578	3.614277	3.54462	3.948045	3.55809	4.051782	4.055878	3.805565	4.135691	3.877725	4.114573	3.783444	3.988241
Tmem53	-0.166458	2.737204	-0.926433	0.369356	0.542677	-6.382613	2.853968	2.532247	2.682163	2.718219	2.712936	2.537984	2.703406	2.665313	2.917273	2.668442	2.930971	3.074167	2.701169	2.858134
Tial1	-0.191375	6.821954	-0.926177	0.369485	0.542799	-6.817275	6.382863	6.674469	6.29464	6.92247	6.300345	6.580837	7.406135	6.905037	7.362832	7.15623	6.608662	6.804567	6.812642	7.310063
Dync1i2	0.12792	7.741579	0.925949	0.369599	0.542901	-6.806429	7.615123	8.065039	7.73357	7.944512	7.929153	7.896975	7.886611	7.611618	7.87771	7.536906	7.540778	7.500907	7.602112	7.64847
Trappc4	-0.173227	4.378992	-0.925566	0.369792	0.543117	-6.639266	4.369549	3.934181	3.838998	3.838533	4.173318	3.751866	4.750481	4.451246	4.870008	4.728967	4.884809	4.513003	4.621498	4.658395
Icmt	-0.150328	4.312044	-0.925217	0.369967	0.543309	-6.627295	4.132599	3.730367	3.986269	3.902409	3.917788	3.808019	4.481542	4.743479	4.639062	4.398098	4.546426	4.584831	4.921699	4.658395
Rab3b	0.188302	4.832033	0.925071	0.37004	0.54335	-6.762877	5.156961	4.760857	5.631182	5.054231	5.454545	5.303548	4.403445	4.429191	4.316905	4.643844	4.480351	4.559124	4.803407	4.707418
Rbm48	-0.187902	3.756769	-0.924683	0.370236	0.543496	-6.686304	4.167689	3.934181	4.498728	4.708637	4.01082	4.562608	3.462819	3.567159	3.456633	3.335938	3.387587	3.083214	3.245776	3.294952
Serpinb10	1.003525	-3.466436	0.924628	0.370263	0.543496	-5.40625	-0.899176	-2.680539	-0.195392	-2.344749	-1.585779	1.165512	-3.260591	-3.260591	-3.260591	-3.260591	-2.697527	-3.260591	-3.260591	-3.260591
Ppp6c	-0.177473	5.413032	-0.924538	0.370309	0.543496	-6.797508	5.658863	5.202427	5.644084	5.86995	5.736702	5.761348	5.154438	5.295206	5.375002	4.815347	5.37156	5.225023	5.591107	5.118751
Wrap73	0.173327	3.452504	0.924514	0.370321	0.543496	-6.458911	3.214303	3.151065	3.447672	2.844379	3.137307	3.014396	3.810873	3.638097	3.848317	3.478908	3.817832	3.732929	3.748946	3.597647
Hnrnpdl	0.159752	8.118638	0.924037	0.370561	0.543782	-6.796897	7.987026	8.544411	8.331589	8.446275	8.129859	8.49203	8.188741	8.045919	8.167234	7.982514	7.668138	7.815476	7.81545	8.051997

Brpf3	-0.161191	4.502199	-0.923483	0.37084	0.544125	-6.689176	4.546601	4.499482	4.451472	4.341506	4.070978	4.105563	4.652595	4.80985	4.504248	4.677181	4.678581	4.73014	4.794977	4.238063
Tcn2	-0.276533	5.490502	-0.922231	0.371471	0.544984	-6.761938	5.984506	5.093178	4.709794	4.433945	5.159937	4.729403	5.370537	5.983995	5.499012	6.277907	6.222567	6.275258	5.413437	5.752039
Mzf1	0.230683	6.815029	0.92202	0.371577	0.545073	-6.817396	6.415162	7.172238	6.386839	6.118792	6.345696	6.086746	7.320187	7.007575	7.359141	7.270944	6.872421	6.902371	6.806365	7.360742
Slc9b1	0.193782	3.209784	0.921818	0.371679	0.545157	-6.444122	2.8647	3.607396	3.056656	3.002635	2.810268	3.084277	3.504983	3.272386	3.253503	3.440594	3.31723	3.228918	3.203938	3.461387
Glrx	0.1671	5.619156	0.921375	0.371902	0.545417	-6.803556	5.777191	5.604242	6.080971	5.701914	6.134527	5.799936	5.26653	5.3896	5.598964	5.264574	5.725387	5.333256	5.719891	5.303471
Cnot3	-0.131831	6.792437	-0.921122	0.37203	0.545539	-6.823604	6.860689	6.834471	6.956468	7.348161	7.066079	7.032616	6.73658	6.657195	6.677497	6.635086	6.524514	6.514752	6.486868	6.777434
Gli4	0.285309	3.163477	0.920579	0.372305	0.545819	-6.436805	3.287913	3.439014	3.151703	2.463791	3.623205	2.374054	3.159163	3.232189	3.253503	3.310669	3.20481	3.516245	3.134365	3.31881
Dync1h1	-0.146089	9.769773	-0.920563	0.372313	0.545819	-6.762218	9.175128	9.494777	9.401587	9.710635	9.08907	9.191347	10.24419	10.09877	10.2524	9.898583	9.889368	9.994429	10.15047	10.18797
Kdelr1	0.133052	6.629546	0.920393	0.372398	0.545878	-6.820426	6.616684	6.464425	6.514312	6.162305	6.659602	6.447982	6.400336	6.793786	6.659604	6.886654	6.811663	6.937715	6.707275	6.767354
Kcnh6	0.300446	2.87565	0.919659	0.372769	0.54623	-6.323879	2.875353	3.073537	2.778919	1.837445	2.77212	1.910004	3.669732	3.024282	3.408468	2.770817	2.883067	2.715403	3.294432	3.475607
Plk2	-0.17302	7.653989	-0.919649	0.372774	0.54623	-6.816689	7.66125	7.222489	7.576332	7.696852	7.251669	7.976973	7.76436	7.858935	7.838864	7.584684	7.898529	7.553525	7.669374	7.609911
Pex3	-0.163316	5.137615	-0.919649	0.372774	0.54623	-6.779479	5.174319	4.766343	5.43459	5.576063	5.356436	5.287179	5.067074	5.059983	5.161541	5.109591	5.047936	4.911231	5.128176	4.89103
Cpsf1	0.217778	6.601804	0.919028	0.373088	0.546623	-6.816848	6.107068	6.871209	6.263057	6.082814	6.356211	5.763716	7.063702	6.913799	7.044156	6.984441	6.675572	6.687093	6.546671	7.082778
Fam216a	-0.125378	5.709464	-0.918677	0.373266	0.546817	-6.805544	5.789915	5.590392	5.686652	5.822387	5.914682	5.531895	5.802806	5.729881	5.746716	5.550173	5.855203	5.603863	5.769496	5.568526
Ppp1r36	0.261376	4.501786	0.918573	0.373318	0.546827	-6.704442	5.017259	5.046602	4.116527	3.800721	4.757939	4.717236	4.171806	4.692543	4.111616	4.585342	4.972116	4.581643	4.246089	4.279179
Pygo2	0.122089	6.005876	0.918378	0.373417	0.546905	-6.807863	5.936993	6.01467	5.922492	5.636341	5.980986	5.711921	6.007318	6.097425	6.024809	6.093895	6.254922	6.154919	6.240127	6.030106
Tbc1d7	-0.218927	2.451467	-0.917647	0.373787	0.54738	-6.289982	2.387517	2.01041	2.498042	2.324175	2.139408	1.875381	2.67414	2.862532	2.524011	2.342482	2.940364	2.516041	2.871195	2.648193
Trim27	0.116755	5.163387	0.917133	0.374047	0.547694	-6.766034	4.94325	5.123415	5.110492	5.195699	5.231972	5.287179	5.030014	5.282961	5.084445	5.128996	5.250298	5.1581	5.280867	5.223575
Myo19	0.249738	5.054693	0.916808	0.374212	0.547869	-6.745318	4.707265	5.81609	5.010785	4.854179	4.827017	4.498643	5.454696	5.151733	5.375002	5.159982	4.755092	4.772703	5.071138	5.360138
Pfdn5	0.150644	6.181337	0.916713	0.374261	0.547873	-6.82267	6.574816	6.721821	6.25925	6.039842	6.405327	6.442819	6.059178	6.077712	6.042012	6.141249	6.10846	5.800438	5.91533	5.972362
Lamtor3	0.132415	5.22667	0.916516	0.37436	0.547952	-6.777551	5.237611	5.167613	5.438634	5.190226	5.56186	5.209391	5.178086	5.099622	5.280045	5.15289	5.39907	5.00228	5.163531	5.13452
Serf2	0.173768	6.794925	0.916065	0.374589	0.548075	-6.828135	7.193175	7.284492	6.661736	6.667364	7.28083	7.185731	6.626299	6.627479	6.650142	6.824038	6.803322	6.607811	6.365923	6.365003
Zc3h3	0.167236	5.23303	0.916014	0.374615	0.548075	-6.77083	5.341141	5.584194	5.206917	4.867958	5.430253	4.8591	5.164996	5.188961	5.275577	5.353869	5.240827	5.350218	5.202397	5.238245
Fkbp1a	-0.348544	9.545044	-0.91601	0.374617	0.548075	-6.748529	9.703634	8.470936	9.782808	10.26193	9.988856	10.30459	9.27768	9.532791	9.546606	9.128426	9.772622	9.174127	9.571719	9.116102
Fam46c	0.449815	0.531798	0.915978	0.374633	0.548075	-5.890028	1.401336	0.533043	1.033553	-0.325128	0.731352	1.275818	-0.529223	1.464337	-0.929891	0.981432	1.482482	2.203999	0.037347	-0.621773
Rnf5	-0.135851	6.733408	-0.91583	0.374708	0.548075	-6.828697	7.179809	6.710484	6.770562	6.944852	7.244721	6.980627	6.459426	6.501029	6.568479	6.586784	6.697896	6.523918	6.634335	6.479679
Hmces	-0.150198	4.409558	-0.915726	0.374761	0.548075	-6.688392	4.519693	4.184143	4.326082	4.239206	4.475885	3.914167	4.567614	4.393186	4.538218	4.581825	4.543347	4.288919	4.553161	4.683115
Vps26a	-0.205743	5.462206	-0.915721	0.374763	0.548075	-6.814931	5.708854	5.256049	6.034515	6.331789	5.95568	6.011019	5.122294	5.300076	5.076761	4.99529	5.314913	5.090167	5.406075	4.904232
Vdac3	-0.136884	7.723188	-0.915063	0.375097	0.548497	-6.81366	7.933238	7.531185	7.877082	8.055287	8.041162	7.982066	7.634111	7.547545	7.644636	7.470004	7.722231	7.465884	7.749723	7.477953
Ppm1n	0.449709	0.608785	0.914825	0.375218	0.548606	-5.813964	0.539902	0.583632	0.484899	-0.166914	1.556544	-0.214525	0.429655	1.020936	0.17081	1.597576	1.102128	1.515634	0.111637	0.887553
Ext1	0.126323	5.264391	0.914529	0.375368	0.548735	-6.782026	5.41053	5.467813	5.462667	5.095807	5.161783	5.414719	5.111417	5.382731	5.066451	5.136206	5.259708	5.261346	5.237977	5.273263
Rac1	0.154343	7.063905	0.914472	0.375397	0.548735	-6.829593	6.946537	6.824646	7.217261	6.990178	7.30232	7.147702	6.78187	7.265195	7.005553	6.914178	7.198258	7.230678	7.280479	6.801622
Sun1	-0.141311	6.374392	-0.91435	0.375459	0.548759	-6.818136	6.187273	6.225489	5.934953	5.946615	5.819695	5.724133	6.828274	6.637132	6.833834	6.712696	6.469618	6.613286	6.511945	6.816362
Slc9a2	-0.306695	6.176567	-0.913868	0.375704	0.54905	-6.79989	5.878625	4.283127	5.348356	5.459566	5.967334	5.817141	7.149937	6.610919	6.99947	6.644386	6.557697	6.315161	6.747877	6.718119
Klhl36	-0.16927	3.359708	-0.913534	0.375874	0.549231	-6.521933	3.664706	3.466233	3.131857	3.114742	3.188895	3.076678	3.392685	3.413873	3.400282	3.463704	3.728487	3.440827	3.45286	3.25429
Tbca	0.122582	4.985338	0.913332	0.375976	0.549295	-6.786852	5.206312	5.240367	5.332504	5.408629	5.579915	5.570283	4.835551	4.628172	4.71693	4.732143	4.618412	4.519683	4.82012	4.636413
Cap1	-0.200332	8.582422	-0.913268	0.376009	0.549295	-6.789111	8.752569	8.107527	8.761208	9.045563	8.877692	9.052846	8.081716	8.678453	8.383392	8.43881	8.751858	8.59207	8.492793	8.141675
Smpdl3b	-0.339719	5.289304	-0.913004	0.376143	0.549424	-6.756131	4.692281	3.479652	5.224203	5.419585	4.845505	5.283883	5.58227	5.708064	5.948265	5.582203	5.457481	5.619495	5.509306	5.74325
Tm2d2	-0.139286	6.337239	-0.912845	0.376224	0.549455	-6.825787	6.557557	5.88956	6.264576	6.176215	6.296151	6.455327	6.30928	6.315101	6.389821	6.385692	6.370609	6.47148	6.458681	6.400839
Tead2	-0.500476	0.74195	-0.912783	0.376256	0.549455	-5.919029	1.48718	0.632508	-0.974047	0.33436	1.258434	0.915838	1.372662	0.645799	0.837186	1.064403	0.924208	1.315214	0.649393	0.970221
LOC100125364	-0.167865	5.097895	-0.912308	0.376497	0.549741	-6.785756	5.251989	4.735912	5.262736	5.488098	5.393031	5.411702	4.725734	4.915162	4.997579	4.894014	4.999333	5.187762	5.150375	5.003257
Nphp1	0.138591	5.165583	0.911989	0.37666	0.549872	-6.774944	5.350716	5.3631	5.044792	4.886126	5.434839	5.175995	5.309988	5.065713	5.159118	5.124169	5.172725	4.995108	5.101079	5.178649
Ppapdc1a	-0.452001	3.758596	-0.911894	0.376708	0.549872	-6.478769	3.188908	1.453108	3.177748	3.374877	3.607042	2.998397	3.951676	4.171987	5.262088	4.738473	3.877725	3.843548	4.502288	4.620503
Dguok	-0.212411	3.539748	-0.911863	0.376724	0.549872	-6.505744	3.417469	3.582736	2.528721	3.204354	3.252623	3.099357	3.969854	3.660989	3.907275	3.890566	3.84309	3.859433	3.695599	3.786295
Sec24c	0.100274	7.08324	0.911589	0.376863	0.550008	-6.832408	6.934491	6.946227	7.189928	6.999596	7.07345	7.060334	7.125144	7.08438	7.174673	7.089701	7.225405	7.037086	7.116512	7.120031
Nop10	-0.210516	2.931232	-0.911051	0.377138	0.550286	-6.445242	2.810225	2.439156	2.568639	3.233029	3.280067	2.998397	3.200804	3.000548	3.333065	2.795315	2.802949	2.738492	3.03287	3.012401
Stab2	0.368206	2.375978	0.911036	0.377145	0.550286	-6.160713	2.10932	2.843782	1.966737	1.128291	1.72577	1.353343	3.060831	2.76729	3.069578	3.080873	2.531849	2.36102	2.713149	2.890335
Foxc2	0.706113	-0.19476	0.910659	0.377337	0.550499	-5.820702	1.669604	-0.366503	1.669168	-1.223791	-0.038319	1.606524	-0.399109	-0.654381	-0.632503	-0.420466	-0.991788	-0.920914	-0.512812	0.554014
Upk2	0.265162	1.226654	0.91039	0.377474	0.550632	-6.13881	1.644914	2.264105	1.841991	1.454757	1.803478	1.353343	0.952703	1.596873	0.97585	0.799637	0.885831	0.930264	0.921344	0.442608
LOC361985	-0.100994	5.938047	-0.910248	0.377547	0.550671	-6.814898	5.919085	5.764945	5.669545	5.753545	5.812665	5.693406	6.021912	5.974888	6.057711	6.109853	6.150545	6.159203	6.065397	6.005816
Fscn1	0.154651	7.594004	0.909791	0.37778	0.550944	-6.820648	7.797707	7.550389	7.920629	7.801669	8.223833	8.125697	6.973486	7.545496	7.290486	7.372633	7.622552	7.647627	7.243784	7.208362
Nphs1	-0.5315	0.232774	-0.908444	0.378467	0.551814	-5.954861	1.874641	0.725555	0.818644	0.280432	-0.038319	0.571087	0.625837	-0.81357	-0.175642	0.413143	1.82436	-0.070959	-0.893866	-0.397871
Marcks	-0.275748	6.655397	-0.908366	0.378507	0.551814	-6.835594	6.531696	6.242317	6.596257	7.600764	6.82711	7.493875	6.062005	6.702033	6.225794	6.533172	6.570608	6.945798	6.435367	6.424855
Zdhhc20	-0.181934	4.423231	-0.908327	0.378527	0.551814	-6.722856	4.243653	4.196386	4.477916	4.984269	4.36492	4.71479	4.481542	4.203515	4.481148	4.005262	4.144751	4.342647	4.833902	4.5246
Carm1	0.121593	6.705406	0.908263	0.37856	0.551814	-6.835486	6.878007	7.132968	7.283223	6.913086	6.821867	6.83113	6.467978	6.59119	6.488686	6.484099	6.54928	6.476639	6.483375	6.489347
Klhdc3	-0.110118	7.879588	-0.908137	0.378624	0.551841	-6.819296	7.96132	7.963336	7.94737	8.069021	7.817402	7.891029	7.909552	7.80643	7.923056	7.835605	7.953199	7.593648	7.789871	7.860068
Eid1	-0.187515	7.734871	-0.908041	0.378673	0.551845	-6.816717	7.86773	7.570909	7.850259	8.430306	8.070928	8.350706	7.501377	7.518593	7.659288	7.449891	7.657143	7.470201	7.660508	7.23786
Igf1r	-0.175405	4.277453	-0.9079	0.378745	0.551883	-6.696143	4.040949	4.112733	4.43537	4.736442	4.018988	4.313834	4.27775	4.269548	4.441807	3.978769	4.276668	4.140807	4.631001	4.291288
Ddx17	-0.178601	8.185271	-0.907663	0.378866	0.551993	-6.813153	8.087504	8.500447	8.110483	8.4974	7.749692	8.086267	8.475211	8.083472	8.400067	8.201961	7.988716	8.126781	7.948085	8.343179
Sdcbp2	0.332286	1.442954	0.907294	0.379055	0.552185	-6.031617	1.212615	1.655851	1.775325	0.966949	1.173177	1.014597	2.305626	1.393205	1.937064	1.780488	1.430105	1.568131	1.077784	1.501815
Sars	-0.153856	7.802794	-0.90718	0.379113	0.552185	-6.814205	8.035025	7.779238	8.076709	8.488137	8.132684	8.440226	7.485144	7.593883	7.559438	7.470004	7.573971	7.450235	7.682763	7.478835
Vps35	-0.209172	7.390614	-0.907136	0.379136	0.552185	-6.829988	7.447229	6.721821	7.371907	7.754236	7.745699	7.728982	7.373506	7.184547	7.344283	7.085982	7.338957	7.354471	7.638689	7.387781
Zranb2	-0.18064	7.996833	-0.906734	0.379342	0.552417	-6.811978	7.993152	8.296858	8.146784	8.736937	7.960766	8.322469	8.31083	7.707737	8.069307	7.720798	7.56185	7.406755	7.789517	7.938197
Zfp523	-0.135523	6.798975	-0.906314	0.379556	0.552663	-6.83617	6.855318	6.73447	6.464931	6.530402	6.616519	6.413708	7.178225	6.798955	7.009595	7.050805	6.740861	6.858915	6.812642	7.134667
Pcgf2	0.164957	4.658088	0.906191	0.379619	0.552688	-6.77053	5.052887	5.528791	5.178177	4.917381	4.801203	5.095555	4.213088	4.442465	4.492744	4.51702	4.44779	4.308338	3.939462	4.342619
Rapgef3	0.245854	6.702477	0.906017	0.379708	0.55275	-6.833308	6.664299	7.211975	6.562677	6.014257	6.487085	6.076304	7.104039	6.842996	6.742079	7.117289	6.559984	7.0295	6.525596	6.912266
Anks4b	0.672443	-0.25546	0.905677	0.379882	0.552937	-5.653892	-0.518419	1.151583	0.25957	-0.705589	0.210178	-1.427823	0.851758	-0.511025	0.245823	0.413143	-0.855515	-0.153565	-0.755361	0.257494
Ndn	-0.237473	7.181934	-0.905088	0.380184	0.553285	-6.83755	7.071072	6.226485	7.167764	7.400054	7.440232	7.158542	7.037368	7.115788	7.505127	7.236136	7.302091	6.910018	7.517539	7.469984
Btg1	-0.180071	5.760752	-0.904888	0.380286	0.553285	-6.816488	5.630846	5.010077	5.815619	5.93902	5.784196	5.802242	5.655227	5.820781	5.707445	6.01526	6.130169	6.114674	5.594349	5.660089
Pspc1	-0.250481	5.952206	-0.904884	0.380309	0.553285	-6.8293	5.919085	5.512563	6.263057	6.872084	6.077992	6.728782	5.704911	5.869094	5.867432	5.623833	5.850228	5.589649	5.824373	5.653849
Chrac1	0.231848	2.760688	0.904762	0.380351	0.553285	-6.389959	2.843156	2.519311	3.042552	2.376521	3.036313	2.675144	2.629097	2.964197	2.694377	3.302147	3.220315	2.461578	2.535634	2.584013
Pcdha2	-0.540207	0.332407	-0.904761	0.380352	0.553285	-5.806363	0.176305	-1.463779	0.158563	0.664146	-0.038319	1.046069	0.563361	0.222004	0.884903	0.646479	-0.318799	1.11804	1.407665	1.009844
Plrg1	-0.126217	5.093625	-0.904208	0.380635	0.55363	-6.786123	5.237611	4.861671	5.113869	5.275401	5.237232	5.303548	5.122294	4.9088	5.112276	5.008447	5.128213	4.854238	5.202297	4.990935
Cox5a	-0.114411	5.910846	-0.904104	0.380688	0.55364	-6.814477	5.668082	5.554382	5.403198	5.742369	5.7649	5.64541	6.366431	6.054847	6.322103	5.93071	6.040209	5.860869	6.245293	6.17983
Gltscr2	0.166508	6.360578	0.90312	0.381193	0.554308	-6.831489	6.375378	6.433729	6.381246	5.843462	6.375422	5.907729	6.479655	6.594167	6.373187	6.811332	6.652184	6.580119	6.022715	6.237293
Klc3	-1.054375	-0.975554	-0.902995	0.381257	0.554328	-5.609143	1.178614	-1.463779	-1.909221	-1.575022	-0.613814	-0.862697	-1.009457	-0.654381	-0.773548	0.475109	1.674823	-2.668207	-0.123798	-1.406952
Morf4l2	-0.181742	7.973043	-0.902912	0.3813	0.554328	-6.817392	8.050089	7.712609	8.068685	8.542308	8.234613	8.439856	7.868961	7.79138	7.99922	7.589069	7.988433	7.564469	8.0555	7.723747
Smarcd1	0.128044	7.298474	0.902637	0.381441	0.554467	-6.837279	7.407873	7.57404	7.641239	7.190332	7.386402	7.108178	7.137259	7.2552	7.186623	7.257782	7.354849	7.232713	7.225603	7.230542
Zfp438	0.173784	2.712299	0.902477	0.381523	0.554519	-6.434838	2.917193	3.224637	2.924461	2.718219	2.712936	3.099357	2.227194	2.665313	2.347926	2.600561	2.958968	2.726993	2.614401	2.474989
Zfp266	-0.130755	7.021626	-0.902376	0.381575	0.554527	-6.840669	6.575633	6.739359	6.741389	7.029021	6.49589	6.711711	7.46125	7.260831	7.369661	7.222651	7.107368	7.142547	7.11256	7.345337
Ctxn1	-0.152103	9.149521	-0.901659	0.381943	0.554968	-6.770073	9.881518	9.381948	9.453146	9.509331	9.694204	9.651648	8.655188	8.839924	9.066281	8.776503	9.03108	8.604495	8.823774	8.72711
Pcbd1	-0.224167	3.036326	-0.901606	0.381971	0.554968	-6.478281	3.380557	3.037697	2.47722	2.908002	3.259533	2.915623	3.231264	2.862532	3.400282	2.843096	3.228006	2.461578	3.556237	3.142871
Naf1	-0.23849	3.769883	-0.901027	0.382268	0.555333	-6.673654	3.759759	3.61957	4.23289	4.646043	3.846148	4.185462	3.706333	3.756192	3.350165	2.934159	3.656224	3.625558	3.954679	3.62342
Rbak	0.145349	3.496676	0.900794	0.382388	0.55544	-6.54308	3.145563	3.551306	3.393138	3.52156	3.470774	3.541623	3.720717	3.638097	3.408468	3.62956	3.66758	3.433771	3.488149	3.482664
Sema5b	0.23653	4.573164	0.900447	0.382566	0.555632	-6.723219	4.22295	5.252144	4.620039	4.632654	4.508156	4.323498	4.481542	4.289273	4.765539	4.637083	4.235294	5.030616	4.349488	4.743122
Lrp3	-0.136458	6.710621	-0.900103	0.382743	0.555822	-6.836883	6.472414	6.3676	6.395882	6.196377	6.140154	5.88724	7.143278	6.915387	7.104606	7.074143	6.888281	7.073292	7.044259	7.261648
Inpp5a	-0.117331	5.182888	-0.899563	0.383022	0.556159	-6.78683	5.386529	5.138299	5.071085	5.025832	5.024515	5.112377	5.298047	5.08559	5.213859	5.16704	5.388815	5.1581	5.276837	5.25689
Csgalnact1	-0.152073	5.070179	-0.899042	0.38329	0.556481	-6.784337	5.185062	4.710528	5.397668	5.17368	4.843207	5.139986	5.193638	5.151733	5.089545	5.034405	4.847853	5.103568	5.028628	5.130032
Csnk1d	-0.131411	7.386649	-0.898504	0.383567	0.556816	-6.841611	7.436029	6.945018	7.389078	7.286658	7.398191	7.19102	7.364374	7.502312	7.340544	7.437299	7.621093	7.425995	7.584286	7.248769
Spcs1	0.155068	6.139576	0.898177	0.383735	0.556944	-6.839108	6.226284	6.174829	6.177711	6.327641	6.754372	6.62123	5.673828	5.86417	6.045953	6.02306	6.013759	6.121277	6.031114	5.921329
Med9	-0.12519	4.815773	-0.898132	0.383759	0.556944	-6.777308	5.057571	4.793461	4.864121	5.080109	5.026542	5.089903	4.812232	4.464419	4.666626	4.754179	4.837836	4.741613	4.763639	4.5246
Rnf43	-0.230972	3.655141	-0.89802	0.383816	0.556944	-6.652626	3.95819	4.400657	3.902849	4.125276	3.416161	3.57437	2.943086	3.720315	3.244385	3.424979	3.352837	3.995035	3.549571	3.692011
Krr1	-0.161679	4.448576	-0.897973	0.38384	0.556944	-6.746968	4.464322	4.343088	4.576652	5.023782	4.519714	4.907024	4.187425	4.438054	4.325577	4.205901	4.53717	4.233112	4.360956	4.229697
Cacul1	-0.22464	5.735103	-0.897851	0.383904	0.556968	-6.839135	6.012522	5.686107	6.239293	6.742833	6.007909	6.680715	5.397647	5.394161	5.24619	5.082476	5.564618	5.396317	5.677718	5.19378
Ska2	0.242367	1.205762	0.897523	0.384073	0.557147	-6.04665	1.178614	1.279848	1.143363	1.097434	1.48759	1.585258	1.000645	1.198329	0.837186	1.755735	1.844511	1.345607	0.879431	0.929479
Slc35c1	-0.161716	5.559173	-0.896983	0.384351	0.55748	-6.823929	5.767216	5.142523	5.734594	5.803392	5.95568	5.653096	5.127701	5.56975	5.422258	5.54657	5.557006	5.570476	5.498963	5.512732
Ggct	-0.156014	3.830087	-0.896807	0.384442	0.55748	-6.733636	4.421353	4.496185	4.413617	4.79531	4.27571	4.647132	3.624549	2.964197	3.416608	3.385186	3.621611	3.028062	3.262178	3.388105
Tbc1d2	0.284026	2.974951	0.896683	0.384506	0.55748	-6.403149	3.016775	3.159427	3.34223	2.227735	2.883666	2.146157	3.410541	2.901501	3.262563	2.854798	3.331578	3.028062	3.070609	3.220915
Rnmtl1	0.20646	2.828732	0.896675	0.384511	0.55748	-6.439697	2.967843	2.631765	3.265371	2.656049	3.333437	2.762997	2.8014	2.436799	2.74697	2.600561	2.940364	2.816513	2.984265	2.879681
Selo	0.181056	4.440761	0.896628	0.384534	0.55748	-6.633838	3.983028	4.407694	4.011832	3.606198	3.827669	3.648009	5.038651	4.816662	4.890608	4.955085	4.684183	4.709839	4.749166	4.919916
Inpp5b	-0.147507	5.587891	-0.896498	0.384602	0.55751	-6.803077	5.469722	5.634551	5.224203	5.339438	5.178286	5.000356	6.034921	5.539186	6.053802	5.818659	5.561578	5.607003	5.772362	6.030106
Edc4	0.222614	7.417402	0.896181	0.384765	0.55768	-6.846216	7.0047	7.687169	7.236362	6.735336	6.94646	6.498625	8.00299	7.567371	7.910142	7.756326	7.480084	7.5047	7.493862	8.029281
Mutyh	0.299649	4.430258	0.896056	0.38483	0.557707	-6.661598	4.180631	4.827969	4.195136	3.68095	4.319352	3.57437	4.983054	4.596856	4.749517	5.036976	4.302392	4.634911	4.075609	4.943127
Ormdl1	-0.232559	1.022544	-0.895587	0.385072	0.55799	-6.023446	1.108109	1.339943	0.750864	1.245516	0.917619	0.881356	1.335758	0.871929	1.01926	1.104165	1.260496	1.045835	1.077784	1.122575
Smim22	-0.471817	-0.118079	-0.895238	0.385253	0.558185	-5.703584	0.105685	0.310827	-1.367151	-0.325128	-0.613814	-0.291112	-0.067151	0.222004	0.091681	0.20947	1.102128	0.151783	-0.123798	0.753927
Fbxw5	0.220133	6.778193	0.894991	0.38538	0.558292	-6.84265	6.678054	6.930433	6.418585	5.928101	6.73467	6.005026	7.179527	6.925663	7.081753	7.243944	6.943041	6.905562	6.739847	7.195533
Slc16a12	-0.70718	1.210157	-0.894915	0.38542	0.558292	-6.042079	3.32724	1.184734	0.604933	-0.325128	0.952152	0.056669	2.01015	0.972957	1.180909	1.704918	2.374443	1.186803	0.745852	1.779078
Cct2	0.114466	6.819828	0.894204	0.385788	0.558757	-6.848231	6.924267	6.783309	7.062053	6.921369	7.169214	7.134952	6.616717	6.659091	6.765371	6.526786	6.679748	6.659383	6.859209	6.73003
Ago3	-0.383633	2.762124	-0.894086	0.385849	0.558778	-6.340698	1.916502	2.702143	2.538805	3.137672	1.48759	2.00915	3.545949	3.614836	3.130231	2.720538	2.90242	2.619163	3.766298	2.83626
Rpn2	0.155574	7.810035	0.893867	0.385962	0.558811	-6.836592	7.663557	7.614899	7.626245	7.804059	8.105616	8.20974	7.76826	7.671088	7.834314	7.847002	7.644268	7.851332	7.683905	8.023244
Tmed4	-0.124349	5.908627	-0.893863	0.385964	0.558811	-6.826376	5.891801	5.667172	5.719159	5.639022	5.712985	5.460695	5.864024	5.94877	5.9925	6.251591	6.140393	6.157062	6.114777	6.187409
Ipo13	-0.106306	7.347928	-0.893499	0.386152	0.559016	-6.848382	7.104845	6.921242	7.139467	7.216595	7.186539	6.970923	7.673465	7.527962	7.632456	7.480428	7.410823	7.438827	7.476396	7.700855
Chd8	-0.150974	7.424727	-0.892873	0.386477	0.559418	-6.845456	7.145566	7.418123	7.384193	7.743999	7.021042	7.34762	7.70365	7.566866	7.643336	7.394846	7.300724	7.360995	7.410326	7.514137
Gas2	-0.224199	3.039018	-0.892276	0.386786	0.559598	-6.423633	2.906846	2.97275	2.528721	2.787519	2.874693	2.160607	3.850657	3.169712	3.392049	3.223093	3.022257	2.9008	3.229186	3.728093
Bnip3	-0.189586	4.995547	-0.892232	0.386809	0.559598	-6.778041	5.212627	5.000799	5.008973	4.858787	5.006144	4.361521	5.138455	5.042656	5.112276	4.859579	5.357605	4.851593	5.334202	4.842478
Adat2	-0.146879	3.138944	-0.892219	0.386815	0.559598	-6.504006	3.118917	3.294639	3.006676	3.275	2.936364	3.006419	3.169687	3.081965	3.432752	3.060806	3.141068	3.145002	3.356854	3.097617
Spred1	-0.208157	4.101808	-0.892216	0.386817	0.559598	-6.707181	4.15463	3.763659	4.323168	4.602753	4.070978	4.524012	3.96382	3.916976	4.055381	3.649853	4.078235	4.056042	4.56643	3.791988
LOC502684	0.382061	1.399863	0.892184	0.386834	0.559598	-6.05175	1.957182	1.79116	0.945338	0.280432	1.803478	1.275818	1.697535	1.658838	1.061401	1.14286	1.533025	1.643459	1.770732	1.659607
Fpgt	-0.179126	3.104772	-0.891942	0.386959	0.559712	-6.552079	3.365524	2.812308	3.47943	3.527359	3.384903	3.365711	2.905804	2.926907	2.858731	2.866406	3.359854	2.827322	3.079891	2.890335
Impa1	-0.242839	5.350577	-0.891842	0.387011	0.55972	-6.822923	5.365903	4.75259	5.522324	6.031704	5.586799	5.960298	5.408795	4.952752	5.271094	4.847916	5.32032	5.200289	5.621619	5.105095
Nr1i2	-1.397783	-1.559629	-0.891593	0.38714	0.559839	-5.230984	0.307952	-3.260591	-2.787702	-3.260591	-1.400091	-3.260591	1.091998	-0.26106	0.091681	-1.136114	1.230182	-0.920914	-0.040977	0.887553
Crybb1	-0.334154	0.801084	-0.891427	0.387226	0.559878	-5.877444	0.739674	1.082904	-0.263851	0.43652	0.470544	-0.072565	1.408645	0.871929	1.218642	1.104165	1.290185	1.252437	1.28461	1.355706
Cd55	-0.349006	2.302048	-0.891361	0.38726	0.559878	-6.459804	2.402324	1.725091	3.131857	3.711838	2.762423	3.242073	1.754571	2.14364	2.127623	1.852296	1.581856	1.568131	1.983515	2.584013
RGD1311863	-0.220218	2.553287	-0.891126	0.38382	0.559954	-6.356989	2.072763	2.47979	2.401882	2.994284	2.046579	2.434761	2.867532	2.436799	2.772565	2.308845	2.750518	2.568523	3.003904	2.879681
Nhlrc1	-0.232473	2.265125	-0.89108	0.387406	0.559954	-6.253459	2.072763	1.425638	2.162035	2.13961	1.96438	1.910004	1.938115	2.709903	2.508863	2.572488	2.696109	2.738492	2.794335	2.417223
LOC691807	0.131843	5.984522	0.890846	0.387528	0.560062	-6.847645	6.38566	6.321768	6.337871	6.434125	6.886346	6.620577	5.570406	5.528853	5.63778	5.539336	5.648674	5.695207	5.553287	5.649152

Golph3	-0.243724	5.944386	-0.890534	0.387689	0.560181	-6.845517	6.100273	5.3631	6.30723	6.643531	6.389665	6.450925	5.68304	5.860878	5.705785	5.493286	5.982275	5.743085	6.089724	5.434817
Tceal8	0.141965	5.378345	0.890417	0.38775	0.560181	-6.839651	6.002839	5.723247	6.125141	5.950937	6.346508	6.276155	4.832242	4.940331	4.864068	4.871149	5.08016	4.843629	4.879797	4.601177
Tmem147	0.176466	5.143929	0.890382	0.387768	0.560181	-6.755544	4.948303	5.086616	4.634216	4.374055	5.109198	4.507149	5.347548	5.300076	5.368723	5.562714	5.450923	5.401751	5.381888	5.588291
RGD1311745	0.158455	3.536316	0.890329	0.387796	0.560181	-6.661399	4.026686	3.735969	4.257522	3.847833	4.234219	4.113012	3.049479	3.180315	3.159628	3.100666	3.487079	2.941167	3.372046	3.142871
Pum2	-0.145145	7.818831	-0.889975	0.38798	0.560338	-6.832514	7.987026	7.675555	8.075627	8.32561	8.050157	8.243645	7.86411	7.421948	7.534409	7.536452	7.625103	7.564469	7.855523	7.711032
Trrap	-0.197619	8.6041	-0.88994	0.387998	0.560338	-6.833922	7.882327	8.339572	7.977569	8.357295	7.643421	7.719863	9.494489	9.069892	9.402536	9.008483	8.742492	8.695799	8.820144	9.307986
Zfp444	-0.139192	4.735497	-0.889537	0.388207	0.560573	-6.756227	4.870572	4.390036	4.588836	4.640703	4.735811	4.734242	4.599099	4.778791	4.809475	4.660609	5.060912	4.786616	4.957391	4.743122
Tst	-0.336836	5.833856	-0.888797	0.388592	0.561061	-6.822625	6.277378	6.0645	4.703085	5.076158	5.899279	4.702498	6.109239	6.041824	6.150923	6.441081	5.929011	6.16667	5.878569	6.264165
Zbtb22	-0.130197	5.661957	-0.888205	0.388899	0.561437	-6.83257	5.863991	5.41815	5.916704	5.721019	5.468031	5.836394	5.552424	5.751372	5.560912	5.692284	5.734865	5.602291	5.663896	5.516176
Wnt1	0.420139	0.12196	0.887979	0.389017	0.561517	-5.803384	0.105685	0.679782	0.307539	-0.02435	-0.107758	0.881356	0.498056	-0.150641	-0.080923	0.534523	0.071497	-0.434228	1.1849	-0.297732
Haus4	0.249275	2.426966	0.887919	0.389048	0.561517	-6.35482	2.529164	2.894758	2.010869	2.213416	3.011968	2.592292	2.566765	2.345542	2.446625	2.614395	2.274011	2.220493	2.022549	2.530531
Clybl	-0.216629	3.620801	-0.887822	0.389099	0.561523	-6.674976	4.377065	3.914553	3.619037	3.875379	4.442875	3.653128	3.569982	2.849305	3.31576	3.463704	3.525031	2.990087	3.423994	3.803307
Wdr12	0.148874	4.764935	0.887391	0.389323	0.561779	-6.763398	4.640141	4.719039	5.142254	4.800109	4.947445	4.662444	4.618433	4.943446	4.68359	4.778959	4.872595	4.653252	4.898279	4.407101
Ireb2	-0.183581	4.78841	-0.887167	0.38944	0.56188	-6.781469	4.70428	4.643552	4.999877	5.332829	4.606283	5.103056	4.760958	4.65122	4.824848	4.259708	4.609607	4.775496	5.128176	4.695317
Mad1l1	0.179218	3.955449	0.887073	0.389489	0.561883	-6.67552	4.087502	3.758163	4.051108	3.920151	4.5954	4.297582	3.720717	4.015001	3.662308	3.790537	3.78171	3.975732	3.766298	4.056051
Sod1	-0.130343	7.303363	-0.886967	0.389544	0.561895	-6.847826	7.560063	7.342104	7.250619	7.631379	7.674453	7.593426	7.30833	7.010549	7.293804	7.09032	7.281451	6.930841	7.238095	7.051675
Txnl4b	0.150292	4.355522	0.886838	0.389611	0.561925	-6.737164	4.576285	4.346754	4.685039	4.364367	4.930226	4.495797	4.150713	4.055878	4.213244	4.219542	4.457635	4.224961	4.306643	4.027379
Ndst3	0.212244	4.084981	0.886441	0.389818	0.562117	-6.598178	3.214303	3.763659	3.585381	3.847833	3.506066	4.174824	4.648839	4.632039	4.60385	4.353427	4.184818	4.265266	4.195746	4.311247
Ubox5	-0.150325	4.501564	-0.886402	0.389838	0.562117	-6.740208	4.658759	4.364946	4.755906	4.512069	4.292652	4.307355	4.394501	4.519639	4.511866	4.441427	4.850346	4.335093	4.529643	4.6173
Polr2c	0.132184	5.500287	0.886263	0.389911	0.562154	-6.816786	5.535003	5.45088	5.641746	5.245807	5.618052	5.36723	5.68304	5.343175	5.63778	5.608362	5.675639	5.363275	5.387506	5.481365
Dcaf17	-0.425885	1.506297	-0.886138	0.389976	0.562181	-6.088098	0.995438	1.632013	1.534559	1.945353	0.091273	1.107027	1.809437	2.268138	2.241976	0.750369	2.031907	1.568131	1.963595	1.755962
Helq	-0.185374	3.337247	-0.886034	0.39003	0.562192	-6.533418	3.342675	3.492948	3.184186	3.309066	3.224646	2.695134	3.647317	3.211662	3.540964	3.51623	3.639021	3.204252	3.431265	3.43979
Zdhhc9	-0.19599	6.519456	-0.885587	0.390263	0.562359	-6.853738	6.68564	6.246248	6.139293	6.533287	6.938947	6.329885	6.110605	6.594167	6.293659	6.541342	6.542358	6.917625	6.729563	6.687139
Csrnp2	-0.124064	5.014978	-0.885575	0.390269	0.562359	-6.754973	4.556564	4.704825	4.753743	4.847241	4.380852	4.487224	5.331236	5.275563	5.295576	5.286341	5.402472	5.231141	5.374363	5.333059
Dbt	0.102233	4.939642	0.885541	0.390287	0.562359	-6.816779	5.362121	5.457677	5.582052	5.413335	5.33206	5.586428	4.531353	4.673906	4.449761	4.574767	4.630068	4.616334	4.576302	4.421864
Dimt1	-0.178997	4.129089	-0.885355	0.390384	0.562432	-6.698474	3.932918	3.943895	3.993619	4.577819	4.109733	4.297582	4.583442	4.078726	4.165741	4.219542	3.972332	3.732929	4.047497	4.242228
Igfbp5	-0.4396	4.483884	-0.884902	0.39062	0.562705	-6.721601	4.358202	2.545069	5.248982	4.713732	4.067045	4.41674	4.32559	5.019224	4.57868	4.210463	4.470659	5.22093	4.850267	4.828697
Cyp2t1	-0.3198	2.239957	-0.884452	0.390855	0.562889	-6.256061	2.658139	1.934482	1.472789	1.593908	2.507694	1.275818	2.930765	2.522625	2.166756	2.514649	2.428842	2.300247	2.639729	2.768575
Mfrp	-3.484635	1.41328	-0.884427	0.390868	0.562889	-5.944826	8.406742	-2.267963	-0.491181	-2.344749	-0.257548	-0.457681	0.282279	1.71825	0.628769	5.763854	7.678699	1.315214	1.1849	0.929479
Mrpl35	-0.156556	4.588458	-0.884388	0.390889	0.562889	-6.763655	4.849116	4.175923	4.680492	4.731426	4.951718	4.704965	4.460269	4.564846	4.582303	4.394093	4.709125	4.489378	4.595845	4.414502
Eif5b	0.206266	3.526573	0.883999	0.391092	0.563114	-6.619698	3.37306	3.588941	3.830814	3.906865	3.770765	4.216915	3.981847	3.272386	3.188437	3.368956	2.912	3.261164	3.294432	3.544677
Cyfip1	-0.123144	7.363103	-0.883659	0.391269	0.563241	-6.852409	7.410165	7.156135	7.278341	7.606939	7.499075	7.607668	7.496683	7.349814	7.445214	7.141974	7.172584	7.262885	7.322142	7.3434
Eif4enif1	-0.16142	5.32029	-0.883651	0.391273	0.563241	-6.830464	5.466203	5.382875	5.88049	5.880183	5.249432	5.536057	5.030014	5.438999	5.104739	5.084962	5.13845	5.185663	5.278854	4.866959
Ccdc59	-0.153846	4.133091	-0.88302	0.391603	0.563648	-6.732177	4.307974	4.019342	4.340564	4.675069	4.487704	4.509973	3.987806	3.979018	3.980484	4.005262	4.172914	3.71558	4.038003	3.73402
Ppp2cb	-0.129115	7.200771	-0.882665	0.391789	0.563757	-6.853574	7.477259	7.073367	7.330425	7.512877	7.533691	7.52806	7.124468	6.980532	7.117147	6.915575	7.244962	6.911924	7.09321	6.978046
Srrm3	-0.168426	6.598849	-0.882647	0.391798	0.563757	-6.85554	6.268314	6.556742	6.110004	6.806821	6.159678	6.656068	6.982467	6.664765	6.935762	6.757022	6.540815	6.522256	6.555847	6.883892
Tbc1d25	0.189396	4.764067	0.882606	0.39182	0.563757	-6.75546	4.661839	5.23642	5.088351	4.514991	4.39974	4.386324	4.842144	4.930944	4.824848	4.803319	4.744407	4.744468	4.746254	4.831464
LOC688765	-0.166497	2.530606	-0.882489	0.391881	0.563777	-6.458062	2.821286	3.028596	2.578448	3.067759	2.77212	2.800388	2.05625	2.364261	2.278164	2.359011	2.684976	2.268872	2.480628	2.142093
Sympk	0.135523	7.039592	0.882216	0.392023	0.563915	-6.858986	6.990684	7.213984	6.922695	6.69081	7.108317	6.658615	7.259905	7.014257	7.247782	7.17327	7.060221	6.993964	7.029364	7.202496
Supv3l1	0.144907	5.482998	0.881433	0.392433	0.564437	-6.817918	5.460908	5.64947	5.499273	5.211996	5.570221	5.202424	5.733565	5.295206	5.76755	5.491413	5.500978	5.389039	5.296877	5.728482
Zfp746	-0.121442	5.129291	-0.881341	0.392481	0.564439	-6.79689	5.212627	4.948678	5.271833	5.038072	4.966574	4.81639	5.067074	5.340814	5.211521	5.208673	5.314913	5.1581	5.174405	5.12553
Naa25	-0.179366	4.539126	-0.881236	0.392536	0.56445	-6.714154	4.026686	4.332034	4.440757	4.661948	3.785203	4.138786	4.848708	4.892772	4.819797	4.49849	4.700858	4.727257	4.919116	4.731318
Ube4a	-0.126029	6.874853	-0.881142	0.392586	0.564453	-6.859963	6.627752	6.491173	6.760903	7.048814	6.687411	6.910221	6.93853	6.849654	6.987907	6.83958	6.952937	7.021286	7.078235	7.067017
Lrig2	-0.198203	5.197859	-0.880699	0.392817	0.564631	-6.778275	4.519693	5.086616	4.817254	5.316175	4.251652	4.767664	5.719309	5.460906	5.756369	5.299246	5.265325	5.410762	5.455051	5.689362
Slc33a1	-0.124457	5.042905	-0.880668	0.392834	0.564631	-6.77442	4.782765	4.435504	4.811028	4.928381	4.784532	4.887611	5.300443	5.154424	5.259827	5.121749	5.117902	5.155958	5.393102	5.516176
Ube2g1	-0.194741	5.350539	-0.88061	0.392864	0.564631	-6.838786	5.723667	4.938996	5.816656	5.877915	5.985161	5.672131	4.977075	4.989389	5.146937	4.944172	5.190547	5.042261	5.407919	5.234069
Btg2	0.164886	4.622967	0.880547	0.392897	0.564631	-6.765788	4.608566	4.75259	5.285371	4.748906	4.738286	4.589557	4.583442	4.548571	4.596704	4.595839	4.467414	4.778284	4.258405	4.233886
Htr5a	-0.244321	2.947856	-0.879681	0.393351	0.565215	-6.535906	2.706665	3.151065	3.190596	3.942027	2.936364	3.158148	2.566765	2.68033	2.834633	2.39151	2.729	2.749899	3.098279	3.34228
Ncapd3	0.167996	4.79698	0.879569	0.39341	0.565232	-6.759219	4.754192	5.082224	4.882053	4.455398	4.608991	4.509973	5.312364	4.806432	4.896441	4.818339	4.899331	4.656286	4.772253	4.760648
Car4	-0.207198	5.416338	-0.878799	0.393813	0.565683	-6.821224	5.714798	5.123415	5.457361	5.20839	5.527922	4.889781	5.422059	5.5474	5.893911	5.264574	4.87014	5.359557	5.549951	6.037314
Stx5	0.163135	5.719358	0.878792	0.393817	0.565683	-6.834009	5.693887	6.185104	5.661493	5.435094	5.688871	5.376544	5.841165	5.767283	5.7739	5.901388	5.822558	5.689294	5.485056	5.779522
Dtymk	0.144112	4.334348	0.878528	0.393955	0.565759	-6.696846	4.235407	4.522355	4.169406	3.972106	4.339058	3.960324	4.481542	4.548571	4.477262	4.382013	4.524735	4.357637	4.302685	4.486487
Wfikkn1	0.318328	2.302332	0.878451	0.393996	0.565759	-6.191923	1.787089	2.631765	1.574317	1.478901	2.16907	1.473956	2.518182	2.76729	2.611707	3.005666	2.481265	2.816513	2.203293	3.012401
Vps53	-0.102946	6.083982	-0.878421	0.394012	0.565759	-6.855166	6.22316	6.116967	6.299838	6.297413	6.124153	6.088636	5.854271	6.056287	6.070665	5.991604	6.129142	5.966095	6.07239	5.908283
Usmg5	-0.151349	4.039719	-0.878269	0.394092	0.565806	-6.766046	4.846411	4.506054	4.612898	4.667211	4.698159	4.594888	3.882992	2.988532	3.941526	3.463704	3.802462	3.028062	3.6527	3.973287
Zfp354a	-0.138947	3.041835	-0.877882	0.394295	0.565976	-6.544493	3.263791	2.991607	3.271431	3.400384	3.114621	3.390443	3.127125	2.665313	3.027678	2.819403	2.940364	2.827322	2.964355	2.972849
Rpn1	-0.18743	8.711727	-0.877864	0.394304	0.565976	-6.827676	8.554288	7.94622	8.427815	8.841602	8.819555	8.850613	8.652845	8.779755	8.848536	8.884496	8.665292	8.770159	8.86865	9.055712
Magoh	0.215434	4.488381	0.877715	0.394382	0.566021	-6.804176	4.692281	4.848802	5.186218	5.455009	5.256357	5.879617	3.945565	3.929599	4.086326	3.968034	4.156888	3.956168	3.929227	3.62342
Ppil2	0.130814	5.849285	0.877492	0.394499	0.566121	-6.840991	5.693887	5.915712	5.903108	5.692911	5.982031	5.496734	5.914952	5.882141	6.057711	5.964857	5.78395	5.75304	5.778076	6.098319
Khsrp	-0.106608	6.0628	-0.87694	0.394789	0.56647	-6.846313	5.780028	5.700505	5.893317	5.973416	5.626095	5.862041	6.190288	6.37286	6.316677	6.194024	6.211941	6.253384	6.304891	6.223667
Rnls	0.219551	2.209145	0.875761	0.395409	0.567291	-6.26285	2.402324	2.184396	2.010869	1.502648	2.183675	2.203108	2.659281	2.227821	2.493553	2.39151	2.288794	2.153355	2.168911	2.402413
Pnpo	0.155775	5.971666	0.875569	0.39551	0.567368	-6.851513	5.915219	6.166555	6.333534	5.93902	6.143894	5.687181	5.831256	5.749593	6.241917	6.021763	5.855203	5.921394	6.081661	5.740308
Lig3	-0.155865	5.327162	-0.875178	0.395716	0.567596	-6.817696	5.152589	5.303982	5.437287	5.533472	4.789315	5.228377	5.54436	5.505855	5.448299	5.190317	5.188577	5.486023	5.389374	5.422024
Pik3ip1	-0.157871	5.798545	-0.874908	0.395858	0.567732	-6.837543	5.622966	5.878205	5.499572	5.692911	5.21785	5.36255	5.797727	6.034539	5.919913	6.197475	5.81747	6.259382	5.752183	6.102907
Aes	-0.135155	9.86831	-0.874656	0.395991	0.567855	-6.77424	10.43875	10.1752	10.05965	9.993534	10.25037	9.844169	9.618378	9.621777	9.759268	9.767749	9.787905	9.568007	9.665259	9.608022
Chic2	0.194756	3.079851	0.874444	0.396102	0.567947	-6.474925	2.515609	2.884706	2.924461	3.240109	3.068148	3.468012	3.026505	3.126505	3.09008	3.214035	3.434871	3.261164	3.098279	2.952659
Anapc13	-0.158364	4.873969	-0.874223	0.396219	0.568046	-6.82339	5.555019	5.186149	4.882053	5.275401	5.45153	5.506666	4.543542	4.318363	4.659784	4.809346	4.594813	4.594355	4.322371	4.591416
Farp2	-0.137812	4.814621	-0.874127	0.396269	0.568051	-6.755341	4.680181	4.80949	4.454139	4.552446	4.355276	4.307355	5.386413	4.934079	5.029775	4.963216	4.894507	4.856878	5.038184	5.200217
Srebf1	0.271219	7.051051	0.874007	0.396333	0.56807	-6.863973	7.130639	7.190714	6.977934	5.851575	6.68613	6.186323	7.103353	7.561299	7.069545	7.525512	7.265659	7.939905	6.942347	7.29661
Wdr16	-0.365312	3.096858	-0.873922	0.396377	0.56807	-6.49596	4.141452	2.769246	2.31067	2.213416	2.77212	3.248852	3.521509	3.159031	3.01701	2.977621	3.84309	3.145002	3.245776	3.186749
Man1a2	-0.239911	5.350185	-0.873715	0.396487	0.56809	-6.835462	5.306139	5.080024	5.702436	5.953093	4.93455	5.844248	4.949859	5.396437	5.308757	4.865376	5.255952	5.414351	5.70497	5.22568
Setd2	-0.135879	6.790639	-0.87365	0.396521	0.56809	-6.86685	6.602329	6.781956	6.736469	7.222416	6.669364	6.919278	6.665863	7.009806	6.644089	6.746853	6.725323	6.870065	6.780267	6.70912
Fbxw9	0.193379	4.664832	0.873627	0.396533	0.56809	-6.711369	4.369549	4.943845	4.214137	3.954995	4.202298	4.067729	5.162364	4.82682	5.161541	4.984678	4.770972	4.715669	4.850267	5.147901
Hspbap1	0.192704	2.902094	0.873412	0.396646	0.568184	-6.411194	2.645748	2.914655	2.691232	2.523044	2.661692	2.836835	3.072094	3.272386	3.04878	3.545408	2.986432	2.838051	2.736815	3.069767
Det1	0.378887	1.52627	0.87316	0.396779	0.568213	-6.117336	1.108109	0.583632	1.825611	1.502648	2.014259	2.217002	1.809437	0.923327	1.458292	1.569444	2.014226	1.667719	1.881043	1.355706
Uqcrc1	0.145554	7.286883	0.873157	0.396781	0.568213	-6.865511	7.492476	7.190204	7.430962	6.811582	7.47107	7.101172	7.453735	7.160652	7.466443	7.237254	7.305275	7.22507	7.309374	7.371238
RGD1562963	-0.264918	1.486052	-0.873105	0.396808	0.568213	-6.188828	1.568201	1.991799	1.195284	1.9796	1.78444	1.221719	1.091998	1.908813	1.360416	1.28803	1.482482	1.11804	1.770732	1.608903
Zfp51	0.326622	1.427057	0.872929	0.396901	0.568278	-6.055321	0.830061	2.01041	0.785152	1.273392	1.415175	1.426914	1.607516	1.908813	1.981639	0.938085	1.167576	1.488652	1.700256	2.050068
Ass1	0.15841	2.624796	0.87251	0.397122	0.568478	-6.474189	2.977763	3.184227	3.209656	2.787519	2.883666	2.863579	2.582602	2.14364	2.278164	2.291729	2.274011	2.461578	2.66462	2.402413
Pcdh15	-0.303473	3.496859	-0.872409	0.397175	0.568478	-6.59076	3.529616	4.361326	3.330668	3.644058	2.361201	3.179592	3.691803	3.583227	3.774234	3.478908	3.196995	3.502822	3.876936	3.591131
Cdc34	0.154086	6.054329	0.872334	0.397214	0.568478	-6.857612	6.438695	6.120183	6.304278	5.701914	6.361038	6.1018	5.980672	5.765523	6.148482	6.012651	6.077933	5.758697	6.021511	5.991043
Zbtb45	-0.182923	4.604932	-0.872305	0.39723	0.568478	-6.776857	4.925421	4.073446	4.775226	4.726393	4.7917	4.898428	4.263083	4.532109	4.461611	4.357545	4.804791	4.606955	4.722744	4.594677
Wfdc1	0.318642	4.632528	0.872117	0.397329	0.568553	-6.760342	5.25607	5.611879	4.519244	3.781437	4.718362	4.452412	4.704179	4.568886	4.680213	4.643844	4.148808	4.650211	4.258405	4.927694
Uros	-0.220084	2.797023	-0.871904	0.397442	0.568646	-6.426693	2.8647	2.064844	2.992072	2.708042	2.856578	2.28453	2.854546	2.91426	2.995434	2.889346	3.157269	2.761217	3.220819	2.825198
Smc1a	-0.210901	7.596721	-0.8718	0.397497	0.568657	-6.855063	7.464088	7.237378	7.676773	8.362171	7.750306	8.139669	7.198279	7.609168	7.611335	7.427047	7.43137	7.430432	7.451135	7.573187
Txndc12	0.143866	5.969942	0.871683	0.397558	0.568677	-6.860062	6.126148	5.915712	6.164747	6.142603	6.441478	6.588873	5.456846	5.805453	5.72885	5.922393	5.83646	5.972195	5.746365	5.756413
Gpr173	0.314346	1.140778	0.871497	0.397656	0.56875	-5.88285	0.307952	0.895377	0.851376	0.386344	0.371962	0.481842	1.372662	1.49863	2.127623	1.920699	1.347792	1.760874	1.60059	1.824228
Hist1h2bh	-0.272294	4.57915	-0.871053	0.397891	0.568976	-6.797146	5.45026	4.418186	4.149803	4.688073	5.55345	5.047769	4.171806	4.274505	4.106593	4.390078	4.733642	4.665351	4.456698	4.070176
Gmppa	0.220303	5.427473	0.870973	0.397933	0.568976	-6.804158	5.249943	5.652436	4.78373	4.741441	5.472498	4.957551	5.801115	5.561662	5.817587	5.870017	5.513618	5.555927	5.214991	5.830202
Kcnj14	-0.268986	4.206465	-0.870929	0.397956	0.568976	-6.658632	3.821655	4.263866	2.969885	4.054744	3.96082	3.59047	4.881086	4.208703	4.294995	4.797268	4.324082	4.616334	4.326276	4.872343
Papd5	-0.140285	6.078128	-0.87056	0.398151	0.569135	-6.863271	6.126148	6.055567	6.452317	6.54477	5.915776	6.358371	6.081642	5.922171	5.877787	5.888637	5.803383	5.977057	6.048948	6.064616
Zic2	-0.23606	4.767153	-0.870539	0.398163	0.569135	-6.816584	5.707365	4.535265	4.895848	4.895125	5.054623	5.737448	4.510807	4.318363	4.46554	4.574767	4.483567	4.659314	4.254311	4.707418
Leo1	-0.149508	5.890693	-0.869844	0.39853	0.569566	-6.86517	6.127263	6.106192	6.024683	6.703032	6.484875	6.451659	5.497094	5.599679	5.636038	5.493286	5.780032	5.539588	5.561594	5.491897
RGD1305713	-0.192737	3.210831	-0.869789	0.398559	0.569566	-6.585761	3.431974	2.843782	3.545931	3.611667	3.579695	3.321383	3.30948	3.19084	3.207329	3.139454	3.373787	2.772447	3.003904	2.791492
Dhodh	0.154898	3.618167	0.869049	0.39895	0.570058	-6.560296	3.154337	3.404252	3.387569	3.400384	3.574163	3.384299	4.129307	3.838823	3.996864	3.844356	3.598065	3.46871	3.707626	3.896095
Slc35a2	0.185569	5.371044	0.868767	0.3991	0.570203	-6.783421	5.085364	5.204449	4.951633	4.409031	5.044657	4.507149	5.759921	5.512163	5.948265	5.823134	5.588712	5.75304	5.642129	6.005816
Abo	0.209612	2.206762	0.868456	0.399265	0.570346	-6.29267	2.279379	2.33964	2.401882	1.818649	2.154315	2.071653	1.862293	2.24812	2.347926	2.543858	2.415434	2.046399	2.203293	2.503027
Dennd6b	0.208688	6.335238	0.868399	0.399295	0.570346	-6.866009	6.037635	6.711195	6.068844	5.946615	6.176206	5.849832	6.763751	6.334216	6.751763	6.674413	6.137333	6.232189	6.131613	6.897819
Clk3	0.210323	7.238752	0.868243	0.399377	0.570396	-6.870293	7.000449	7.930732	6.936585	7.122026	7.24385	6.968872	7.506575	7.258331	7.335723	7.507091	7.094805	7.140381	6.908232	7.399482
Dhcr7	-0.141396	5.469329	-0.86814	0.399432	0.570406	-6.827212	5.543377	5.062295	5.30913	5.233442	5.43941	5.163494	5.761661	5.237996	5.919913	5.508182	5.194477	5.589649	5.651498	5.99228
Sdf2	-0.12956	6.163824	-0.867549	0.399745	0.570786	-6.861906	6.277378	5.921869	5.780995	5.988211	6.210487	6.098051	6.034921	6.159026	6.245348	6.498158	6.292081	6.277231	6.249412	6.28248
Slc4a7	-0.251212	4.565859	-0.867057	0.400005	0.57109	-6.723833	3.859704	3.894655	4.454139	4.837937	3.680967	4.392459	5.188472	4.820056	5.195053	4.126066	4.776227	4.357637	5.43891	4.970998
Ndufb8	-0.137542	6.998118	-0.866876	0.400102	0.571159	-6.872691	7.344596	6.986734	6.971435	6.780958	7.038724	6.705565	7.065112	6.82199	7.056581	7.13179	7.113609	6.913192	6.955645	7.100092
Mlec	-0.212079	5.272652	-0.866757	0.400165	0.571165	-6.825875	5.41053	4.649501	5.22264	5.341085	5.359656	5.407165	5.006725	5.350235	5.07419	5.044659	5.57822	5.477471	5.838114	5.098219
Gadd45gip1	0.115663	5.843844	0.866689	0.400201	0.571165	-6.865427	6.267303	6.28498	6.087855	6.113018	6.512616	6.33706	5.528095	5.432361	5.549853	5.615258	5.692413	5.416142	5.546608	5.458279
Zbtb1	-0.156709	4.091983	-0.866204	0.400458	0.571372	-6.67634	3.827152	3.466233	3.805981	4.155549	4.051203	4.020978	4.35355	4.106787	4.091419	4.268484	4.26176	4.323687	4.208498	4.440107
Zfp295	-0.140687	4.926058	-0.866156	0.400483	0.571372	-6.801916	4.698293	4.897106	4.775226	5.35093	4.784532	5.106791	5.035778	4.799571	5.076761	4.830243	4.913708	4.7671	4.989761	4.990935
Tcf12	-0.133169	5.434299	-0.866146	0.400489	0.571372	-6.849401	5.648032	5.496151	5.889383	5.818845	5.475469	5.538825	5.386413	5.407759	5.291156	5.03183	5.219768	5.363275	5.442513	5.10738
Tab2	-0.155361	6.83653	-0.865801	0.400672	0.57153	-6.877561	7.277412	6.835124	7.278718	7.481852	7.265894	7.518626	6.117416	6.83127	6.258994	6.457483	6.709616	6.863517	6.502484	6.327275
Ttk	0.534928	1.663818	0.865759	0.400694	0.57153	-6.119327	1.541696	1.953842	0.105278	0.932396	2.553396	2.549011	1.607516	2.488905	0.683763	2.499819	2.388236	2.738492	1.040235	0.799858
Idh3g	-0.136477	7.652321	-0.865163	0.40101	0.571913	-6.864088	7.744953	7.467366	7.590034	7.741818	7.941828	7.429329	7.830101	7.519637	7.810959	7.657481	7.599393	7.407656	7.496031	7.903775
Lef1	-0.152551	3.196059	-0.865043	0.401074	0.571919	-6.625069	3.917539	3.339488	3.500221	3.468286	3.557438	3.718071	2.841442	2.939445	3.09008	2.866406	2.843564	3.118845	2.639729	3.079111
Amt	0.273531	5.606334	0.864976	0.401109	0.571919	-6.809065	5.313991	5.926776	4.62478	4.638025	5.5092	4.942996	6.26825	5.800307	6.084781	6.160235	5.686843	5.378042	5.587858	6.191722
Zc3h10	-0.125932	4.182955	-0.864192	0.401526	0.572445	-6.745006	4.54993	4.051149	4.380359	4.357872	4.439837	4.41674	3.939429	4.139756	3.860303	4.386051	4.298745	4.132116	3.944552	3.751656
Ap2s1	0.18142	6.664801	0.863925	0.401668	0.57258	-6.873827	7.035896	6.953458	6.762518	6.166957	6.912338	6.497914	6.707986	6.429507	6.833074	6.619736	6.81422	6.379	6.691466	6.518828
Lamb3	-0.365864	0.459091	-0.863367	0.401965	0.572902	-5.936075	0.785575	1.425638	0.604933	0.705659	-0.18071	-0.1418	0.357848	0.449407	1.01926	0.413143	0.378374	0.514819	1.001683	0.257494
Gprc5c	-0.451371	0.912335	-0.86332	0.40199	0.572902	-5.986317	1.936985	0.310827	0.20995	0.105385	0.562819	1.046069	0.952703	1.155984	0.931093	0.280604	1.740817	1.643459	1.465469	1.32462
Glycam1	1.867874	-1.306843	0.863154	0.402078	0.57296	-5.718286	3.683012	0.632508	1.033553	-3.260591	0.917619	0.734546	-3.260591	-3.260591	-3.260591	2.586593	4.008504	-3.260591	-1.904936	-2.653844
Rdm1	-0.335821	2.012332	-0.862958	0.402183	0.573041	-6.253969	2.10932	2.425352	0.715742	1.454757	1.705671	1.07687	2.247205	2.488905	2.597457	2.572488	2.388236	2.375822	1.983515	2.460763
Mdk	-0.758374	4.464279	-0.862816	0.402258	0.57308	-6.739559	6.715589	4.313419	3.599901	2.62393	4.285899	3.288866	4.091065	4.244507	3.980484	5.766954	6.865658	4.637984	3.887547	4.291288
Opn4	0.63412	-0.071359	0.862446	0.402455	0.573293	-5.680704	0.176305	1.310208	-0.007587	-1.388744	-0.038319	-1.427823	0.282279	-0.150641	-0.080923	0.847278	0.071497	0.28321	0.546019	0.442608
Cenpk	0.369973	0.875199	0.862293	0.402536	0.573341	-6.129115	0.873216	1.151583	1.004744	1.740915	2.139408	2.481552	0.563361	0.517885	0.17081	0.646479	0.631286	0.403657	0.879431	-0.03329
Ppp1cb	-0.21655	7.210784	-0.862042	0.40267	0.573464	-6.869317	7.382895	6.818718	7.565584	7.916887	7.530123	7.819981	7.11769	6.785998	7.036914	6.482214	7.153529	6.901093	7.419494	7.030761
Tbc1d8	0.18613	4.208265	0.861037	0.403205	0.574158	-6.699865	4.069061	4.719039	4.217279	3.946362	4.124949	3.732651	4.42995	4.234368	4.175368	4.361652	4.208334	4.17506	4.12279	4.486487
Kcnab3	-0.189641	3.928605	-0.860892	0.403282	0.5742	-6.735849	4.442997	4.782675	4.266652	4.31157	3.952316	3.775529	3.908348	3.449372	3.780555	3.440594	3.84309	3.721386	3.627601	3.803307
Spcs3	-0.277878	3.823386	-0.860162	0.403672	0.574686	-6.696467	3.765497	2.97275	4.068613	4.494412	3.827669	4.524012	3.462819	3.904241	3.748669	3.48645	3.706635	3.767014	4.161181	3.751656

Slmo1	-0.251137	4.92169	-0.859471	0.40404	0.575142	-6.783211	4.674093	5.398856	4.467397	4.91074	4.074901	4.277834	5.611511	5.165139	5.401902	5.07749	4.894507	4.851593	4.831156	5.321296
Cd84	-0.453015	2.050235	-0.859276	0.404144	0.575159	-6.166803	1.996747	1.21714	0.52603	1.430203	2.014259	1.353343	0.68572	3.94833	1.326257	3.195748	3.499841	4.552625	1.651284	1.80183
Pdcl	-0.201788	3.389102	-0.85923	0.404169	0.575159	-6.660405	3.736577	3.06466	3.822584	4.018141	3.596165	4.192511	2.979429	3.252427	3.110295	3.070874	3.250834	3.065063	3.409342	3.031778
Atp6v1c2	0.382552	2.377906	0.85918	0.404196	0.575159	-6.334268	2.607924	3.512664	2.718102	1.679737	1.765147	2.05628	2.67414	2.326577	2.539002	2.694725	2.442128	2.330954	1.651284	2.610029
S100a1	-0.22156	4.540397	-0.858659	0.404474	0.575487	-6.816924	5.597458	5.467813	4.576652	4.721342	5.036634	4.670039	3.993741	4.12885	4.231815	4.564114	3.916315	4.257294	4.182881	4.291288
Dok5	0.192197	3.345853	0.858445	0.404588	0.575581	-6.661432	4.002595	4.005493	4.440757	3.515737	3.61245	3.683465	2.760215	3.059168	3.059216	3.167876	3.20481	2.816513	2.838755	2.83626
Fam96b	0.155493	4.693646	0.858127	0.404758	0.575672	-6.807755	5.092229	5.023882	4.687307	4.751386	5.433312	5.158103	4.416758	4.308732	4.585917	4.588849	4.486775	4.338875	4.376105	4.5246
Ndufb4	-0.204663	2.339627	-0.858117	0.404763	0.575672	-6.363835	2.387517	2.655608	1.809043	2.475838	2.28197	2.257897	2.814871	2.030999	2.759824	2.184529	2.303426	2.100868	2.713149	2.294252
Cryz	0.173693	3.122407	0.858011	0.40482	0.575672	-6.507111	3.350332	3.361401	3.049621	2.602113	3.152236	2.982217	3.060831	3.232189	3.110295	3.293574	3.116419	3.397965	3.116435	3.069767
Abcb6	0.300137	7.2992	0.857941	0.404857	0.575672	-6.879454	6.69469	7.714029	6.528297	6.648861	7.052217	6.590208	7.593654	7.678586	7.942213	8.153631	7.168593	7.475364	6.914731	8.044564
Med15	0.139053	7.061799	0.857877	0.404891	0.575672	-6.880124	6.862028	7.050506	7.481826	7.132053	7.117855	6.895718	7.025106	7.136354	7.182452	7.086603	7.093227	6.980106	6.763803	7.069363
Snph	-0.126941	9.075109	-0.857759	0.404954	0.575693	-6.834562	9.252117	8.839511	8.823368	8.733492	8.979946	8.828583	9.393174	8.975458	9.175392	9.226547	8.982891	9.14944	9.340629	9.353934
Hspb11	-0.208689	3.513623	-0.857498	0.405094	0.575824	-6.728218	3.922683	3.655486	3.843073	4.685481	4.28928	4.629061	3.169687	2.976416	3.23521	3.019813	2.823399	2.715403	3.195422	3.178079
Lamtor4	-0.123119	5.3089	-0.856944	0.40539	0.576177	-6.84798	5.629274	5.499448	5.242827	5.477652	5.402427	5.578378	5.34058	5.263149	5.304377	5.345599	5.084402	5.164508	4.949817	5.082046
Mpzl2	0.967928	0.245564	0.856786	0.405475	0.576229	-5.98367	3.026365	0.04804	2.039558	-1.575022	0.371962	2.998397	-1.21312	-0.654381	-0.632503	1.73055	1.230182	0.007173	-1.904936	0.045082
Abcg3l1	0.456947	0.669686	0.856412	0.405675	0.576445	-5.911801	0.785575	1.725091	0.398943	-0.02435	0.15195	1.014597	1.135593	0.517885	1.01926	1.419918	0.885831	0.84773	-0.305233	0.844371
Wdr26	-0.162043	7.494294	-0.856083	0.405851	0.576566	-6.870388	7.503247	7.448723	7.76639	8.14902	7.505629	7.932406	7.378051	7.308729	7.291593	7.181425	7.522524	7.273299	7.529868	7.138023
Tra2b	0.148395	5.984006	0.856073	0.405856	0.576566	-6.880333	6.39587	6.29924	6.821012	6.75153	6.726589	7.041914	5.592083	5.361925	5.525598	5.364141	5.566136	5.300661	5.471014	5.585015
Csrp2	0.339709	3.252816	0.855976	0.405908	0.576572	-6.471073	3.589067	3.42521	2.761803	1.92792	3.506066	2.570814	3.471351	3.221962	3.824043	3.656554	3.633241	3.376046	3.079891	3.673625
Sox5	-0.214258	3.09608	-0.855381	0.406227	0.576908	-6.616378	3.595524	4.042133	3.381979	3.633342	2.987204	3.248852	2.828218	2.849305	3.169295	2.469694	3.099749	2.619163	3.079891	2.530531
Taf13	-0.141502	5.082592	-0.855354	0.406241	0.576908	-6.861867	5.733947	5.538441	5.45459	5.873369	5.744111	5.935304	4.89066	3.997121	4.739818	4.549787	4.534071	4.581643	4.86645	4.786544
Hmmr	0.335563	1.657272	0.855214	0.406316	0.576947	-6.281327	1.764345	1.747452	1.554575	2.061822	2.865664	2.854719	0.68572	1.318383	0.97585	1.540753	1.456531	1.667719	1.815874	1.415936
Ccdc97	-0.094334	6.156204	-0.85502	0.40642	0.576962	-6.880289	6.562509	6.338435	6.397961	6.536167	6.553253	6.530267	5.829598	5.903095	5.868916	5.932091	6.040209	5.892092	5.957486	5.867076
Map3k12	0.158064	8.154558	0.855014	0.406423	0.576962	-6.868717	7.736579	8.34737	8.029667	7.922949	7.90732	7.661685	8.57705	8.197793	8.479922	8.454517	8.078123	8.167758	8.097808	8.510923
Ptk2	-0.123321	7.209875	-0.854579	0.406656	0.577182	-6.882678	7.002272	7.325943	7.01	7.34857	6.990736	6.879976	7.452658	7.256453	7.413027	7.295839	7.288364	7.175173	7.182629	7.327318
Fam76b	0.174327	4.833987	0.854547	0.406674	0.577182	-6.804206	4.627594	5.292622	5.194214	5.050208	4.675086	4.911303	5.170246	4.666384	4.933786	4.744776	4.387261	4.634911	4.536402	4.906858
Stub1	-0.104469	6.043744	-0.854198	0.40686	0.577319	-6.876319	6.363131	6.040932	6.164747	6.152018	6.204233	6.200378	6.070454	5.83091	6.02081	5.983632	6.027045	5.75304	5.907531	5.917427
Vimp	0.12316	6.000126	0.854187	0.406866	0.577319	-6.870114	6.041187	6.102944	6.215132	5.822387	5.900385	5.996996	5.745925	6.109958	6.097495	6.086469	5.858922	6.126756	5.840847	6.080985
Sco1	0.164697	3.782465	0.853841	0.407052	0.577514	-6.663919	3.765497	3.843675	3.867281	3.742078	4.113552	3.844276	3.756062	3.653399	3.307029	3.508843	4.012962	3.750073	4.018826	3.885483
Ptdss1	0.111378	5.989463	0.853718	0.407118	0.577539	-6.851694	5.504448	5.502738	5.674127	5.494034	5.720517	5.541588	6.243558	6.190834	6.210659	6.17548	6.264302	6.448035	6.466665	6.44125
Ago4	-0.239482	2.671213	-0.853439	0.407267	0.577637	-6.344604	2.035255	2.713546	2.09528	2.645422	1.643636	2.086865	3.51327	2.781287	3.324439	2.831299	2.833517	3.018661	3.042398	3.097617
Pkdcc	0.347784	3.415319	0.853218	0.407386	0.577637	-6.511704	3.695089	3.99151	3.098159	2.169587	3.346477	2.592292	3.374605	3.386664	3.577725	4.015725	3.967746	3.419555	3.474137	3.864023
Eed	0.141791	4.493859	0.853082	0.407459	0.577637	-6.790685	4.399379	4.646529	4.825515	4.984269	4.833978	5.047769	4.39898	4.187837	4.189688	4.290193	4.454361	4.346409	4.254311	4.125333
Lin7c	-0.252325	3.902982	-0.852999	0.407504	0.577637	-6.767166	4.276172	3.889638	4.691832	4.921791	3.998482	4.804937	3.083269	3.818604	3.480126	3.186517	3.897149	3.376046	4.066299	3.262515
Usp12	-0.264252	1.509355	-0.852956	0.407527	0.577637	-6.220438	1.831529	1.369077	1.613009	1.856	1.578815	1.766229	1.668148	0.449407	1.326257	1.253084	2.049375	1.345607	1.902128	1.684306
Gnptg	0.139634	6.466406	0.852929	0.407541	0.577637	-6.876711	6.308172	6.573266	6.136805	5.990312	6.424669	6.019962	6.642444	6.586216	6.745314	6.477884	6.582655	6.557576	6.454227	6.751372
Bcl7b	-0.125672	6.088127	-0.852881	0.407567	0.577637	-6.8833	6.525792	6.200381	6.566379	6.78943	6.665467	6.695676	5.568419	5.73708	5.660235	5.849693	5.963971	5.699625	5.698958	5.636551
Nkx2-2	0.216626	2.750607	0.852874	0.407571	0.577637	-6.523723	3.287913	2.801663	3.164784	2.758225	3.534831	3.371934	2.48486	2.454377	1.981639	2.819403	2.346454	3.153617	2.203293	2.387449
Bmi1	-0.162075	5.200047	-0.852633	0.4077	0.577752	-6.850291	5.239674	5.228494	5.354078	5.844623	5.159937	5.790676	5.154438	4.995405	5.146937	4.785087	4.773602	5.121244	5.170065	5.07972
Cdk12	-0.193481	5.281455	-0.852112	0.40798	0.57808	-6.859378	5.364014	5.322718	5.725795	6.053974	5.317234	5.704543	5.030014	5.361925	5.056067	4.653926	5.170732	5.042261	5.40053	4.777963
Sumo2	-0.235788	5.476271	-0.851811	0.408141	0.57824	-6.869441	5.809488	4.902098	5.795786	6.100429	6.0038	6.165876	5.37963	5.255649	5.280045	5.06998	5.541659	5.19195	5.393102	4.814782
Elmo3	0.271805	3.53887	0.851452	0.408334	0.578446	-6.53849	3.549707	3.780021	3.265371	2.591079	3.238702	3.02233	3.993741	3.838823	3.456633	4.041554	4.061116	3.721386	3.079891	4.046557
Dek	0.182579	5.887948	0.85088	0.408642	0.578813	-6.882005	6.030504	6.120183	6.365045	6.649526	6.566548	7.040938	5.390917	5.454368	5.213859	5.476341	5.62557	5.642628	5.435298	5.447498
Cwc15	0.118887	5.551395	0.850297	0.408956	0.579124	-6.87945	6.058818	6.128726	6.100675	6.294865	6.536282	6.418977	4.962019	5.045558	5.142036	5.074991	5.088633	4.978233	4.942203	4.983491
Mbrl	0.138139	7.552006	0.850293	0.408958	0.579124	-6.884969	7.268853	7.452982	7.47298	7.12681	7.366137	7.052123	7.789312	7.726396	7.856923	7.919834	7.588232	7.693395	7.45024	7.972444
Bhlhb9	-0.141576	5.756413	-0.850145	0.409037	0.579168	-6.86869	5.611862	5.452583	5.611014	6.110122	5.854341	5.965451	6.023363	5.577792	5.970552	5.693914	5.618273	5.49113	5.744907	5.893796
Sgca	0.306688	3.366288	0.849615	0.409322	0.579503	-6.521833	3.529616	4.023929	2.853524	2.41457	3.352952	2.854719	3.428178	3.534475	3.480126	4.010503	3.441501	3.732929	2.964355	3.667444
RGD1559786	0.113809	5.215175	0.849228	0.409531	0.579731	-6.838588	5.245844	5.45088	5.286867	5.222759	5.407102	5.250499	5.164996	5.105197	5.299983	5.06243	5.369823	4.96361	5.185196	5.039604
Copa	-0.120952	7.875237	-0.849003	0.409652	0.579834	-6.873979	7.795254	7.480775	7.916526	8.052274	7.861217	7.954084	7.859649	7.933294	7.93373	7.775693	7.917083	7.823934	8.022182	7.934341
Mum1	0.183414	5.393597	0.848491	0.409928	0.580156	-6.819898	5.169999	5.637547	5.120598	4.748906	5.109198	4.702498	5.816264	5.516354	5.820657	5.762302	5.518329	5.454995	5.446106	5.72551
Gpr56	0.183496	7.755029	0.847783	0.410309	0.580627	-6.885682	7.777972	7.502302	7.41497	6.782172	7.560517	7.380784	7.827196	7.941888	7.777377	8.142038	7.929812	8.33046	8.03417	8.176368
Taf2	0.111597	5.878813	0.847153	0.410649	0.581039	-6.871459	5.602275	6.02042	5.990193	5.982944	5.769748	5.844248	5.989609	5.793417	6.03145	5.730896	5.912402	5.779719	6.013056	5.869771
Timp3	0.265972	5.93222	0.846842	0.410817	0.581173	-6.885442	7.047128	6.384641	7.010905	5.592754	6.243101	6.690084	5.31947	5.181064	5.336908	5.485779	5.781339	5.551045	5.788022	5.666301
Csde1	-0.139275	5.171562	-0.846765	0.410858	0.581173	-6.857419	5.384666	5.354021	5.355505	5.753545	5.529352	5.491028	5.191057	4.576933	4.986685	5.042103	5.067356	4.872617	4.731605	5.109662
Nmt1	-0.176522	7.480194	-0.846709	0.410888	0.581173	-6.879257	7.675415	7.107802	7.807371	7.913292	7.643421	7.916529	7.207884	7.560285	7.229416	7.156821	7.587859	7.379014	7.511549	7.034968
Dhrs1	0.190219	6.061434	0.845736	0.411413	0.581797	-6.877271	5.929345	6.535215	5.944466	5.894839	5.995546	5.937404	6.447584	5.982481	6.279223	6.277907	5.795641	5.828954	5.65616	6.379256
Tmem120b	-0.152752	3.63343	-0.845712	0.411427	0.581797	-6.554062	3.263791	2.943995	3.158259	3.233029	3.231692	3.038068	4.182238	3.770297	4.070936	4.237532	3.897149	4.014082	3.844626	4.116286
Tmed5	0.213575	4.224981	0.845032	0.411794	0.582248	-6.798611	4.559869	4.283127	5.289856	4.809659	4.810643	5.188389	3.537848	3.832115	3.584966	3.58107	3.706635	3.936334	4.337929	3.780579
Ddx58	0.278561	3.868605	0.844349	0.412163	0.582702	-6.71575	4.145858	3.785434	3.993619	3.847833	4.505252	4.777073	3.51327	4.101219	3.149895	3.879152	3.401255	4.848943	3.079891	3.246018
Arid1a	-0.118077	7.93882	-0.844106	0.412295	0.582819	-6.878545	7.707812	7.877552	7.960088	8.106793	7.553857	7.762229	7.994876	8.108669	7.984921	8.042903	7.969347	8.142825	7.932157	8.005896
Pcdhgc3	-0.186878	6.750002	-0.844005	0.412349	0.582828	-6.892651	7.342198	6.748396	6.949871	6.71388	6.74209	6.925638	6.400336	6.606995	6.278107	6.514849	6.573629	7.151176	6.97194	6.595705
Bsdc1	-0.12702	6.758048	-0.842996	0.412895	0.583518	-6.893532	7.025173	6.467796	7.059867	6.868662	6.881875	6.870119	6.469044	6.747302	6.584824	6.641856	6.884028	6.78252	6.702024	6.642146
Artn	0.527365	0.049847	0.842922	0.412935	0.583518	-5.66763	-1.642648	-0.572649	0.20995	0.22441	0.027932	-0.646023	1.258992	0.222004	0.571595	0.134646	0.924208	-0.070959	0.434663	1.355706
Stx19	-0.385905	0.590662	-0.84276	0.413023	0.583574	-5.855406	-0.046857	0.679782	-0.263851	0.705659	0.210178	-0.548781	1.888013	1.155984	1.255413	0.799637	1.03357	0.28321	1.218908	1.048408
Erich1	-0.192312	3.612263	-0.842578	0.413121	0.583644	-6.695116	3.917539	3.151065	3.784955	4.074684	4.309397	3.947882	3.445603	3.509467	3.464507	3.28495	3.525031	3.368666	3.576053	3.34228
Gpx4	0.15856	8.120107	0.84232	0.413261	0.583754	-6.867686	8.577917	8.712783	8.408011	7.994304	8.534353	8.126618	7.915426	7.653123	7.995828	8.006646	8.115659	7.797381	7.872982	7.976199
Samhd1	-0.160816	3.358197	-0.842255	0.413296	0.583754	-6.623481	3.439172	3.486315	3.203331	3.711838	3.579695	3.438669	3.270902	3.449372	3.244385	3.070874	3.228006	3.587916	3.515771	2.942457
Rab11b	-0.135725	8.239023	-0.841847	0.413517	0.583998	-6.861896	8.599762	8.092866	8.502167	8.582033	8.725522	8.505182	7.795696	8.159121	8.066076	8.111879	8.311148	8.049265	8.030285	7.820577
Golga3	-0.26337	2.635058	-0.8417	0.413597	0.584042	-6.469291	2.488111	1.953842	2.812554	3.254167	2.472441	3.207696	2.746221	2.665313	3.006262	2.202958	2.568656	2.568523	2.892424	2.310209
Mgat2	0.182304	4.910978	0.841392	0.413764	0.584208	-6.82025	4.938178	4.489567	5.157058	4.849557	5.223162	5.410191	4.645073	4.899205	4.743058	4.66726	4.714609	5.09241	5.057107	4.919916
Akap10	-0.17549	4.184016	-0.841265	0.413833	0.584237	-6.740784	4.132599	3.730367	4.323168	4.440107	4.151195	4.230676	4.455977	4.239446	4.029076	3.844356	4.272956	4.257294	4.553161	4.003043
Irf4	-0.365925	1.636362	-0.841	0.413977	0.584368	-6.056821	1.245833	0.854759	0.398943	1.097434	0.517419	1.541762	2.398025	2.186344	2.295923	2.586593	2.149958	2.2368	1.347449	2.635584
Srsf5	-0.158817	8.778519	-0.840915	0.414023	0.584368	-6.856799	8.665681	8.902206	8.357456	8.893606	8.49665	8.717522	9.2499	8.815901	9.006698	8.919871	8.559739	8.614475	8.624304	9.078892
Pold3	0.14905	3.769326	0.840431	0.414285	0.584604	-6.718826	3.92781	3.843675	4.269682	4.129095	4.312723	4.274516	3.241276	3.749088	3.641623	3.478908	3.695584	3.529544	3.633916	3.160583
Zfp347	-0.216532	3.797149	-0.840369	0.414318	0.584604	-6.657856	3.279917	3.874479	3.247038	4.246256	3.494398	3.693437	4.527267	3.825375	3.901486	3.808701	3.673224	3.78376	3.855477	4.065483
Wbp4	-0.124364	4.216108	-0.840263	0.414376	0.584604	-6.744508	4.110227	4.256089	4.293698	4.464495	4.109733	4.017012	4.502506	4.155962	4.308181	4.164187	4.172914	3.951235	4.246089	4.358053
Tmub2	-0.124806	6.660378	-0.840247	0.414385	0.584604	-6.890587	6.654283	6.382857	6.324819	6.135026	6.291944	6.077257	6.751543	6.856281	6.943529	6.991746	6.90936	6.980711	6.919263	7.042749
Dnai2	0.512143	0.296454	0.839895	0.414576	0.584733	-5.80626	1.071521	-0.273517	0.20995	-1.223791	0.320024	0.881356	0.357848	1.393205	-0.27702	0.20947	1.03357	0.760186	0.598632	0.499386
Ammecr1l	0.126984	5.902863	0.839877	0.414586	0.584733	-6.875649	5.739788	6.221501	5.862538	5.741122	5.652584	5.737448	6.069049	5.982481	6.01546	6.003481	6.081121	5.870046	5.817453	5.87246
Rorb	-0.208223	2.68779	-0.839811	0.414622	0.584733	-6.616045	3.827152	3.354134	3.295418	3.275	2.970456	3.708268	2.518182	2.207232	1.636128	2.202958	1.883989	2.375822	2.689088	1.951771
Spast	-0.178136	5.910166	-0.839512	0.414784	0.584845	-6.882788	5.626123	5.492845	5.670692	6.416264	5.954616	6.261147	6.014634	5.78302	5.867432	5.840894	5.975438	5.851633	5.993542	6.020439
St8sia4	0.20167	1.932897	0.839447	0.414819	0.584845	-6.264215	1.764345	1.725091	1.996308	1.892408	2.062471	2.31068	1.697535	1.934092	1.959523	1.942797	1.864385	2.315682	2.203293	1.80183
Gtf2h2	0.150085	3.934887	0.839396	0.414847	0.584845	-6.706041	3.646165	4.073446	4.185541	4.225001	4.043216	4.075375	4.228269	3.916976	4.01306	3.796617	3.755344	3.594258	3.695599	3.942904
Ech1	-0.144675	5.82228	-0.839091	0.415012	0.585009	-6.873762	6.044731	5.812117	5.514681	5.473152	5.791366	5.334148	5.799422	6.080544	5.819123	5.941725	5.925468	6.150622	5.843574	6.009486
Tspan8	0.331911	1.844379	0.838442	0.415365	0.585438	-6.251365	2.311111	1.747452	2.025285	1.188094	1.664612	2.695134	1.962529	2.16515	1.489496	2.40749	1.199219	1.691577	1.815874	1.910497
Ccdc115	0.18155	3.603242	0.838203	0.415495	0.585552	-6.65314	3.748215	3.719097	4.284739	3.486263	3.633881	3.502454	3.270902	3.484018	3.495578	3.424979	3.771221	3.686193	3.725479	3.34228
Baz2a	-0.168396	7.140219	-0.837967	0.415623	0.585606	-6.897784	6.780076	7.195296	6.998629	7.28152	6.436913	6.819225	7.533306	7.315959	7.582684	7.216429	7.031943	7.18781	7.134998	7.459738
Tlk2	-0.112944	6.009412	-0.837953	0.415631	0.585606	-6.889439	6.011315	5.905805	6.195348	6.423279	6.103179	6.215163	6.019005	5.909482	5.941229	5.881504	5.939588	5.856918	5.81884	5.935544
Ankrd6	-0.135416	6.494547	-0.837826	0.4157	0.585635	-6.893438	6.274363	6.444033	6.412428	6.622688	6.167047	6.232366	6.792973	6.363586	6.794554	6.638475	6.643584	6.435726	6.344902	6.774561
Sidt2	0.179015	7.880839	0.837586	0.41583	0.58575	-6.892825	7.559237	8.022409	7.500004	7.202617	7.633813	7.081372	8.243625	8.114555	8.193527	8.253704	8.031093	8.271823	7.849769	8.381207
Efemp1	0.295134	4.419108	0.837383	0.415941	0.585837	-6.756856	4.335237	4.192317	4.026238	4.14804	4.36492	5.545723	4.380979	4.853564	3.952765	4.690302	4.204441	4.975806	3.855477	4.418187
Zc3h15	-0.217824	4.905785	-0.836316	0.416521	0.586586	-6.846424	4.997891	4.557582	4.987046	5.59137	5.208953	5.560782	4.903326	4.958923	4.800172	4.369831	4.944375	4.376159	4.984828	4.493492
Adh5	0.137187	5.539224	0.835968	0.416711	0.586784	-6.861888	5.627699	5.300205	5.566119	5.192053	5.845183	5.457773	5.452543	5.634794	5.63778	5.42428	5.74562	5.613262	5.574785	5.511007
B4galt3	0.131369	6.180662	0.835713	0.41685	0.586845	-6.889274	6.241803	5.94261	6.376335	5.86995	6.219374	6.202998	6.066236	6.176342	6.212998	6.366405	6.129142	6.436609	5.99477	6.315465
Rhov	0.14592	3.285507	0.835709	0.416852	0.586845	-6.62915	3.402817	3.864284	3.515619	3.561676	3.761059	3.408717	2.814871	3.242343	2.524011	3.319142	3.352837	3.33876	3.03287	3.022122
Copg1	-0.120639	7.45807	-0.835514	0.416958	0.586926	-6.897689	7.281422	6.996102	7.304064	7.30703	7.278706	7.117004	7.537375	7.660241	7.743804	7.37619	7.755869	7.481365	7.909559	7.673359
Rnf39	-0.159935	4.718798	-0.835314	0.417067	0.586956	-6.810995	4.661839	5.110534	4.529394	5.007277	4.651639	4.446528	4.652595	4.568886	5.05867	4.824303	4.684183	4.451452	4.652936	4.823147
Magohb	0.266385	2.485163	0.835295	0.417078	0.586956	-6.461151	2.10932	2.411416	2.877559	3.16769	2.95351	3.426763	2.247205	1.830204	2.260183	2.866406	2.133674	2.315682	2.269694	2.211707
Nme7	-0.116477	4.16177	-0.834451	0.417538	0.587535	-6.753798	4.201945	4.397125	4.096187	4.430854	4.109733	4.227248	4.187425	4.078726	4.044916	3.968034	4.242906	4.023513	4.178567	4.16536
Gk	-0.24295	4.394842	-0.834039	0.417762	0.587758	-6.806196	4.3544	3.817493	4.959161	5.258066	4.57061	4.902733	4.425566	4.198308	4.409542	3.844356	4.341911	3.951235	4.477919	4.093415
Clk2	0.183603	6.392249	0.833847	0.417867	0.587758	-6.892879	6.086587	6.691147	6.21984	6.177138	6.296151	6.058089	6.788713	6.471118	6.597411	6.724703	6.28288	6.402682	5.973761	6.740373
Qrich1	-0.094255	6.503216	-0.833832	0.417875	0.587758	-6.898475	6.48203	6.473676	6.559585	6.624718	6.448298	6.356018	6.57675	6.580224	6.589332	6.507455	6.55617	6.412585	6.450653	6.444869
Mttp	0.353215	0.77733	0.83368	0.417958	0.587758	-5.959932	0.176305	1.425638	0.484899	0.860705	1.018831	0.65513	0.357848	1.49863	-0.080923	0.938085	0.998029	1.691577	0.649393	1.158276
Gpr149	-0.379259	1.240795	-0.833632	0.417984	0.587758	-6.048656	0.643243	0.04804	0.883381	1.593908	1.113405	1.014597	1.335758	1.908813	1.255413	1.704918	0.846404	2.2368	1.815874	1.684306
Npr1	0.193848	4.732724	0.833623	0.417989	0.587758	-6.769638	4.495729	5.007763	4.340564	4.188865	4.641095	4.164107	4.771359	5.121793	4.589521	4.984678	4.757751	5.327557	4.825648	5.102807
Synpo2	-0.321919	1.905726	-0.833265	0.418185	0.587964	-6.352233	2.196854	1.310208	1.996308	2.487785	1.578815	3.186669	1.258992	2.077116	1.743567	1.828755	1.903329	2.300247	1.922909	1.32462
Bcar1	0.182737	6.657082	0.833107	0.418271	0.588016	-6.901252	6.977767	6.948641	7.860345	6.976994	6.895801	6.939845	6.024813	6.481872	6.240771	6.284411	6.604237	6.440134	6.337182	6.202449
Plbd2	-0.144642	6.721754	-0.832684	0.418502	0.588272	-6.894408	6.505363	6.121254	6.191358	6.002855	6.325259	5.79647	6.968221	7.112332	7.136373	7.282904	7.164591	7.399983	6.887898	7.225818
Hip1r	-0.178491	6.97118	-0.832399	0.418657	0.588422	-6.901956	6.864036	7.202901	6.470209	6.742833	6.530581	6.299194	7.329016	7.081554	7.294356	7.329253	6.904569	7.266861	6.933414	7.360742
Bloc1s6	-0.143839	3.897569	-0.832075	0.418834	0.588551	-6.707425	3.997728	3.838476	4.126591	4.001571	3.579695	3.866483	3.957761	3.923301	3.860303	3.615871	4.136602	3.778199	4.061622	3.927469
Fam53a	0.134916	3.778848	0.832051	0.418848	0.588551	-6.662132	3.670834	3.909605	3.580509	3.622545	3.859855	3.789543	3.908348	4.020912	3.889839	3.994723	3.862983	3.755742	3.542874	3.610591
Rps6kc1	-0.141506	6.364006	-0.831808	0.41898	0.588669	-6.890605	6.19686	6.256029	5.879498	5.912674	5.812665	5.707007	6.840674	6.662876	6.765371	6.8322	6.563787	6.491156	6.438076	6.757204
Gorab	0.131175	4.329758	0.831407	0.419199	0.588885	-6.778941	4.350588	4.7275	4.79429	4.488478	4.325951	4.361521	4.27775	4.203515	4.231815	4.349296	4.272956	4.078836	4.13915	4.093415
Rnaseh1	0.157803	4.082011	0.831348	0.419232	0.588885	-6.737136	4.092076	4.640568	4.182328	4.098255	4.158607	4.052313	4.34429	4.015001	4.250151	4.000002	3.858035	3.900956	3.73726	3.911867
Ivd	-0.145072	5.964682	-0.829533	0.420225	0.590187	-6.885721	6.142777	5.978499	5.793682	5.556344	5.670842	5.370341	6.092744	6.137423	6.010091	6.272464	6.317534	6.155991	6.081661	5.950893
Cuedc1	0.199554	4.884575	0.829474	0.420257	0.590187	-6.808288	5.052887	5.433746	4.753743	4.253272	4.777328	4.428729	5.041519	4.856872	4.902249	5.016284	5.078034	4.961159	4.919116	4.96345
Phf14	-0.156893	5.631329	-0.829079	0.420473	0.590422	-6.894523	5.871991	5.688998	5.921529	6.372633	5.924498	6.329885	5.135774	5.573776	5.317478	5.213226	5.442685	5.428618	5.544933	5.105095
Stam	-0.160426	6.385188	-0.828746	0.420656	0.590609	-6.903637	6.536738	6.025003	6.800729	6.755859	6.439197	6.676951	6.199304	6.3425	6.245348	6.068994	6.406634	6.162408	6.569915	6.181999
Uqcrb	-0.159252	6.095073	-0.828511	0.420785	0.590721	-6.905791	6.812309	6.537623	6.610071	7.235761	6.973504	7.337321	5.478169	5.482485	5.579158	5.485779	5.524588	5.290932	5.541578	5.467202
Sync	0.178969	3.667952	0.828371	0.420861	0.59076	-6.69114	3.816137	4.240408	3.906747	3.747057	3.711525	3.99299	3.241276	3.180315	3.432752	3.456042	3.848089	3.496064	4.018826	3.388105
Cdk18	-0.275723	4.325826	-0.827901	0.421119	0.591052	-6.737581	4.300489	4.724685	3.447672	4.125276	4.3745	3.275651	4.502506	4.576933	4.457672	4.571225	4.086719	4.875224	4.526252	4.800732
Rassf8	-0.211638	3.336794	-0.827482	0.421349	0.59124	-6.700429	3.84352	3.466233	4.311452	4.070718	3.188895	3.984893	3.261094	2.901501	2.973531	2.375352	2.802949	2.931181	3.394539	3.372991
Sass6	0.320937	0.603256	0.827477	0.421351	0.59124	-6.072809	0.955837	1.151583	0.914692	1.000693	1.20216	1.910004	-0.399109	-0.380636	0.245823	0.055728	0.805871	0.969826	0.546019	0.554014
Slc7a6os	-0.140788	4.802673	-0.827272	0.421464	0.591329	-6.834635	4.835542	4.601207	5.202965	4.899589	5.05165	4.977075	4.70728	4.762349	4.690302	4.79964	4.496167	4.836642	4.468825
Slc39a9	-0.216902	4.436775	-0.827037	0.421593	0.591355	-6.769649	4.331374	3.889638	4.629506	4.523721	4.090486	4.213454	4.213088	4.761243	4.488889	4.031278	4.555623	4.805872	5.096513	4.558395
Adnp	-0.113346	7.576147	-0.827	0.421613	0.591355	-6.900277	7.335945	7.398828	7.568048	7.800771	7.278706	7.571965	7.635535	7.634931	7.66869	7.584684	7.669198	7.590082	7.611344	7.725607

Mrps33	-0.16195	4.908691	-0.82697	0.42163	0.591355	-6.838943	5.1416	4.719039	4.493553	5.00935	5.322193	5.121639	4.937596	4.647404	5.040349	4.844986	4.994832	4.665351	4.999576	4.836982
Cdc7	0.138725	3.797048	0.826742	0.421755	0.591376	-6.664927	3.549707	3.806886	3.633224	3.721989	3.680967	3.997021	4.134688	3.838823	4.018418	3.962637	3.70112	3.674268	3.766298	3.786295
Cyp2u1	0.17245	3.401565	0.826736	0.421758	0.591376	-6.574637	3.37306	3.279374	3.301354	2.968937	3.26641	3.479584	3.137884	3.683523	3.059216	3.602051	3.537463	3.946285	3.409342	3.728093
Pcnp	-0.195199	6.246071	-0.826673	0.421793	0.591376	-6.906927	6.763685	6.581069	6.799681	7.511414	6.8207	7.56621	5.664558	5.740666	5.723938	5.601432	5.715848	5.610136	5.843574	5.524749
Akr1a1	0.133665	8.290142	0.826431	0.421926	0.591493	-6.886696	8.338851	8.223731	8.303218	7.851064	8.39245	8.000759	8.333528	8.354336	8.385626	8.340276	8.659794	8.247131	8.409653	8.226629
Thsd1	0.25799	4.317533	0.826078	0.422119	0.591638	-6.737892	4.569741	4.425138	3.74196	3.527359	4.548211	4.461194	4.704179	4.015001	4.618038	4.479719	4.052479	4.539538	4.018826	4.825924
Myh3	0.394207	0.771303	0.826063	0.422128	0.591638	-6.308165	2.054131	3.073537	1.981598	1.760745	1.113405	3.069038	-0.399109	-0.992527	-0.080923	0.475109	-0.231835	-0.24119	0.313987	0.119416
Ankrd61	-0.355455	1.871167	-0.8258	0.422272	0.591772	-6.173821	1.071521	1.769471	1.386086	2.093442	0.517419	1.426914	3.159163	2.24812	2.611707	2.375352	1.844511	1.97042	2.168911	2.030937
Papolg	-0.133867	5.168619	-0.824408	0.423037	0.592664	-6.865875	5.101332	5.346717	5.303227	5.699348	5.114924	5.321343	5.361385	5.030987	5.218522	5.011064	4.87014	4.7671	5.014175	5.244487
P2rx7	0.182185	4.383361	0.824355	0.423066	0.592664	-6.732699	3.95819	4.432057	3.918379	4.07864	4.151195	4.329906	4.258161	5.042656	4.60385	4.581825	4.369987	4.89334	4.152409	4.673894
Phldb1	-0.219683	8.16662	-0.824262	0.423117	0.592664	-6.895498	8.020402	8.492402	7.465731	7.847591	7.620791	7.271859	8.381281	8.492356	8.432792	8.624367	8.096882	8.682652	8.240445	8.668935
Vsig10	0.17353	2.340869	0.824237	0.423131	0.592664	-6.401176	2.474162	2.724859	2.31067	2.241914	2.575715	2.515676	2.05625	1.983359	2.39812	2.165862	2.544222	2.433555	2.379013	2.278116
Akt1	0.12124	7.222093	0.824194	0.423155	0.592664	-6.906703	7.486843	7.25358	7.550713	6.984918	7.270605	7.357845	7.180178	7.088607	7.008249	7.233337	7.056446	7.204489	7.189077	7.254965
Acy3	0.213613	2.965707	0.822985	0.42382	0.593526	-6.577179	3.742407	3.532115	3.353699	2.57996	3.210452	3.295428	2.450751	2.488905	2.694377	3.090803	2.940364	3.118845	2.494578	2.733504
Adat1	0.158062	2.86364	0.822709	0.423972	0.59367	-6.48647	2.682606	2.991607	2.954902	2.797152	2.962008	2.695134	3.137884	3.000548	2.772565	3.110461	2.707156	2.880186	2.652228	2.962789
Fam101a	0.343592	0.873715	0.822505	0.424085	0.593758	-6.024783	1.514695	1.679303	1.089503	0.041976	0.917619	0.61372	1.17791	0.58326	0.628769	0.893396	1.290185	1.008331	0.698429	0.970221
Arap2	0.190403	7.059613	0.822387	0.424149	0.59378	-6.909227	6.993743	7.929204	6.950815	7.236204	7.091957	7.242869	7.424375	6.881677	6.927241	6.909977	6.666404	6.863517	6.809158	6.919436
Heatr6	0.137663	4.052459	0.821773	0.424488	0.594126	-6.679857	3.713016	4.073446	3.926082	3.68095	3.850732	3.49677	4.287446	4.187837	4.405458	4.369831	4.152854	4.123371	4.216936	4.346493
Arnt	0.163681	5.387173	0.821591	0.424588	0.594126	-6.855086	4.97826	5.653916	5.264256	5.26676	4.930226	5.314898	5.944361	5.3896	5.765958	5.374339	5.168735	5.387214	5.312711	5.707549
Med27	-0.129308	4.522866	-0.821516	0.424629	0.594126	-6.781412	4.506048	4.175923	4.534442	4.402734	4.463969	4.109292	4.678618	4.639742	4.765539	4.571225	4.741723	4.42323	4.539769	4.836982
Plekhg4	0.570887	1.237409	0.82149	0.424643	0.594126	-5.955222	-0.310971	1.79116	1.430088	1.128291	-0.718553	1.519512	2.247205	1.564863	1.891068	1.780488	1.068256	1.461155	1.377871	1.910497
Rragc	0.118301	6.271838	0.821408	0.424689	0.594126	-6.905149	6.157034	6.093156	6.535239	6.332618	6.411545	6.411444	6.179914	6.252422	6.118855	6.118373	6.432658	6.344615	6.352582	6.085628
Snx11	0.106496	5.164529	0.821159	0.424826	0.594126	-6.840831	5.132748	5.080024	5.050089	4.778387	5.143221	4.961683	5.293243	5.302504	5.151822	5.41047	5.336417	5.19195	5.278854	5.236159
Lman2l	-0.173687	2.237479	-0.821104	0.424856	0.594126	-6.322306	2.072763	1.702379	2.10888	2.169587	1.822268	2.146157	2.501617	2.571773	2.47808	2.184529	2.332254	2.330954	2.725031	2.516844
Dym	-0.118638	5.701631	-0.821061	0.42488	0.594126	-6.894594	5.904858	5.496151	5.74878	5.86995	6.0038	5.82283	5.67752	5.672833	5.559075	5.40451	5.789158	5.602291	5.682296	5.619042
Ak6	0.154219	3.215361	0.821056	0.424883	0.594126	-6.683648	3.633671	3.445867	4.026238	3.791112	3.882413	4.08677	2.955303	2.665313	3.120298	2.733273	2.843564	2.433555	2.902922	2.673087
Tmod1	-0.114039	5.007768	-0.821041	0.424891	0.594126	-6.850945	5.000326	4.827969	5.012595	5.295932	5.181928	5.119791	4.798736	5.107976	4.843082	5.016284	5.010524	5.231141	4.962418	4.748987
Srgap1	0.251991	3.447892	0.820697	0.425081	0.594179	-6.622438	3.045357	3.142653	4.369101	3.670505	3.452798	3.450478	3.769961	3.358932	3.464507	2.831299	3.525031	3.195935	3.760537	3.380568
Rab2b	-0.159381	4.709092	-0.820688	0.425086	0.594179	-6.825268	4.830077	4.46953	4.685039	4.888381	4.539721	5.09931	4.633716	4.771798	4.676828	4.4607	4.857801	4.677349	5.016594	4.380898
Ufm1	-0.155832	5.56998	-0.820628	0.425119	0.594179	-6.901349	6.127263	5.514194	6.040737	6.097508	6.050366	6.216028	5.509574	5.22269	5.268848	5.03183	5.409253	5.085673	5.463941	4.976008
Atraid	0.222144	4.589043	0.820537	0.425169	0.594179	-6.741673	4.485336	4.793461	4.305558	3.617117	4.316041	3.642872	4.8055	4.965068	4.864068	4.965917	4.784073	4.908688	4.792156	5.070381
Abtb1	0.187622	4.886874	0.820524	0.425176	0.594179	-6.787851	4.478365	5.192275	4.408127	4.324952	4.633136	4.146066	5.234309	5.175775	5.227804	5.347671	5.021628	5.105789	4.919116	5.25689
Pop4	0.111445	5.097848	0.819803	0.425574	0.594631	-6.875305	5.492389	5.552796	5.583271	5.334484	5.475469	5.482427	4.919005	4.7399	4.890608	4.916522	5.063063	4.665351	4.674541	4.626888
Kcnip1	-0.179503	4.59162	-0.819666	0.42565	0.594631	-6.807583	4.791228	4.779965	4.895848	4.575022	4.319352	4.013036	4.571588	4.360928	4.693673	4.760414	4.664481	4.581643	4.879797	4.461699
Map1lc3a	0.166445	7.034013	0.819519	0.425731	0.594631	-6.91143	7.011959	6.766985	6.733729	6.135975	6.938409	6.516992	7.47934	7.101216	7.409972	7.408309	7.416303	7.112483	7.209307	7.247733
Ints6	0.128214	5.500047	0.819481	0.425752	0.594631	-6.881266	5.172161	5.703367	5.567351	5.834133	5.611315	5.663279	5.686708	5.217552	5.446313	5.366186	5.276493	5.405362	5.493764	5.591559
Mir3102	0.375474	0.213501	0.819457	0.425765	0.594631	-5.745001	-0.046857	-0.273517	0.049949	-0.81875	0.027932	-0.291112	0.743216	0.449407	0.97585	0.055728	1.199219	0.151783	1.28461	0.929479
Tfcp2l1	-0.209746	3.623595	-0.8194	0.425797	0.594631	-6.702064	3.92781	4.196386	4.109779	3.915736	3.137307	3.420773	3.639767	3.221962	3.367065	3.790537	3.644778	3.680242	3.318158	3.489688
Zmynd19	-0.230117	3.67974	-0.819147	0.425937	0.594726	-6.739243	3.881004	3.184227	3.93756	4.449301	3.952316	4.659903	3.419386	3.431732	3.400282	3.335938	3.493474	3.516245	3.646466	3.334499
Gcs1	0.145194	5.032137	0.819064	0.425982	0.594726	-6.800766	4.579546	4.820078	4.71425	4.452353	4.762811	4.3458	5.381895	5.260653	5.458191	5.474446	5.168735	5.428618	5.071138	5.581733
Rxra	0.228797	3.507369	0.819009	0.426013	0.594726	-6.67119	3.881004	3.279374	4.182328	3.355447	3.930833	3.892687	2.930765	3.301815	2.894141	3.129854	3.487079	3.869926	3.713602	3.395603
Upk1b	1.194273	-2.8509	0.818859	0.426096	0.594772	-5.482596	-0.410969	-2.267963	0.914692	-2.731185	-2.73597	1.353343	-3.260591	-2.076466	-2.604521	-2.618976	-3.260591	-3.260591	-2.232431	-2.228037
Snx33	0.179061	4.679918	0.818498	0.426295	0.594981	-6.787621	4.586045	4.955898	4.162901	4.192519	4.725866	4.434686	4.57555	4.930944	4.646003	4.979343	4.678581	5.208581	4.579577	4.925106
Ivns1abp	0.137952	6.97186	0.818407	0.426346	0.594983	-6.913865	6.771547	7.365785	6.665192	7.076768	7.114524	7.100704	7.102666	6.823682	6.990636	6.944613	6.74957	6.980106	6.860556	7.07229
Heca	-0.129101	5.537404	-0.818112	0.426509	0.595055	-6.885396	5.497569	5.333844	5.483698	5.768314	5.407102	5.794155	5.738875	5.217552	5.483597	5.476341	5.431072	5.668406	5.663896	5.59319
Palm	-0.134987	8.508353	-0.818056	0.42654	0.595055	-6.878812	8.932133	8.634524	8.436606	8.442251	8.544997	8.597856	8.615084	8.237677	8.466998	8.643468	8.175049	8.408563	8.350001	8.636078
Cep95	0.233933	6.661533	0.818044	0.426546	0.595055	-6.911018	6.280387	7.347146	6.517502	6.554743	6.302019	6.443558	7.11429	6.801533	6.903546	7.041235	6.435982	6.449785	6.230782	6.854948
Slc35f2	-0.167409	2.463621	-0.817797	0.426683	0.595177	-6.479639	2.776524	2.884706	2.645305	2.806722	2.519256	2.481552	2.01015	2.345542	2.680923	2.359011	2.197736	2.330954	2.11575	2.622863
Ube2q1	-0.096743	7.591691	-0.817563	0.426812	0.595288	-6.900829	7.86272	7.619831	7.796644	7.945595	7.912535	7.901552	7.406135	7.406227	7.456114	7.424105	7.487712	7.278231	7.381526	7.412937
mrpl11	-0.119214	4.901424	-0.817376	0.426916	0.595363	-6.838205	5.159142	4.960691	4.749408	4.756333	4.93239	4.704965	5.151787	4.754164	5.171196	4.751052	4.942039	4.721475	4.967428	4.950782
Hdac3	0.110312	6.474146	0.817195	0.427016	0.595428	-6.910441	6.400486	6.582624	6.389628	6.304185	6.573496	6.304897	6.754168	6.444977	6.600093	6.580631	6.386159	6.351184	6.364974	6.618311
Hcrtr1	-0.362896	2.112375	-0.817087	0.427076	0.595428	-6.239223	2.569081	1.914859	1.705429	1.128291	0.691028	1.519512	3.190506	2.345542	2.583064	2.956054	2.133674	2.252925	2.285827	2.70964
St13	-0.168708	3.836329	-0.816857	0.427203	0.595428	-6.798652	4.395683	3.939046	4.440757	4.7588	4.770087	4.484354	3.436917	3.201289	3.307029	3.158464	3.61576	3.30822	3.754753	3.25429
Fip1l1	-0.208208	6.514755	-0.816788	0.427241	0.595428	-6.914729	6.731799	6.551986	7.021281	7.673178	6.809561	7.591428	6.066236	6.233457	6.1679	6.043656	6.111581	5.984319	6.183126	6.055178
Mthfs	0.143664	3.215885	0.81676	0.427256	0.595428	-6.642837	3.614724	3.63764	3.376367	3.335752	3.761059	3.68846	2.467906	3.272386	2.583064	2.998871	3.380703	3.33876	2.871195	2.868948
Tmem126b	-0.159443	3.785118	-0.816755	0.427259	0.595428	-6.695971	3.765497	3.479652	3.365078	3.946362	4.059146	3.905613	4.192594	3.550909	3.91304	3.602051	3.802462	3.732929	3.954679	3.836741
Lrrc9	-0.285094	0.693163	-0.815503	0.427953	0.596325	-6.015307	1.212615	0.725555	0.442561	0.577356	0.470544	0.809818	0.563361	1.393205	0.449965	0.348395	0.676952	1.252437	1.001683	0.753927
Gins3	0.221146	1.779148	0.814733	0.428379	0.59685	-6.319113	1.693879	1.894965	2.135702	2.227735	2.436306	2.28453	0.851758	1.830204	1.102347	1.704918	2.197736	1.890216	1.902128	1.227132
Sfxn3	0.128267	6.51932	0.81442	0.428553	0.597023	-6.915433	6.708904	6.993766	6.657693	6.406856	6.73467	6.357587	6.412571	6.267417	6.472184	6.411673	6.513543	6.311316	6.562485	6.477031
Polr2m	-0.130969	5.253986	-0.813858	0.428865	0.597342	-6.898076	5.922941	5.426835	5.796836	5.729848	5.702881	5.846484	4.812232	4.732714	4.771898	4.839106	5.111679	4.772703	5.235902	4.896325
Tom1	0.133688	6.073334	0.813755	0.428922	0.597342	-6.91081	6.388452	6.514177	6.334258	6.014257	6.540544	5.948896	5.715723	5.885385	5.877787	6.029528	5.96742	6.043456	5.958745	5.831584
[truncated: 6,737,261 more chars]
